# Direct carbonyl reductive functionalizations by diphenylphosphine oxide

**DOI:** 10.1126/sciadv.ads4626

**Published:** 2025-02-07

**Authors:** Feng Liu, Jianyu Dong, Ruofei Cheng, Shuang-Feng Yin, Lang Chen, Lebin Su, Renhua Qiu, Yongbo Zhou, Li-Biao Han, Chao-Jun Li

**Affiliations:** ^1^ Advanced Catalytic Engineering Research Center of the Ministry of Education, College of Chemistry and Chemical Engineering, Hunan University, Changsha 410082, China.; ^2^ School of Physics and Chemistry, Hunan First Normal University, Changsha 410205, China.; ^3^ Department of Chemistry, FQRNT Centre for Green Chemistry and Catalysis, McGill University, 801 Sherbrooke Street W., Montreal, Quebec H3A0B8, Canada.; ^4^ College of Science, Central South University of Forestry and Technology, Changsha 410004, China.

## Abstract

Reductive functionalization of aldehydes and ketones is one of the most challenging but ultimately rewarding areas in synthetic chemistry and related sciences. We report a simple and extremely versatile carbonyl reductive functionalization strategy achieving direct, highly selective, and efficient reductive amination, etherification, esterification, and phosphinylation reactions of (hetero)aryl aldehydes and ketones, which are extremely challenging or unattainable to achieve by traditional strategies, using only diphenylphosphine oxide and an inorganic base. It enables modular synthesis of functionally and structurally diverse tertiary amines, ethers, esters, phosphine oxides, etc., as well as related pesticides, drug intermediates, and pharmaceuticals. Compared to phosphorus-mediated name reactions, this strategy firstly transformed C═O bonds into C-element single bonds. Mechanistically, phosphinates are formed as intermediates, which undergo unconventional nucleophilic substitution at the C atom within their C─O─P unit. Thus, this work provides important strides in the field of reductive functionalization of aldehydes/ketones, phosphorus-mediated transformation, and various fundamental reactions.

## Teaser

Direct carbonyl amination, etherification, esterification, phosphinylation, etc. are realized by diphenylphosphine oxide.

## INTRODUCTION

**Fig. 1.**
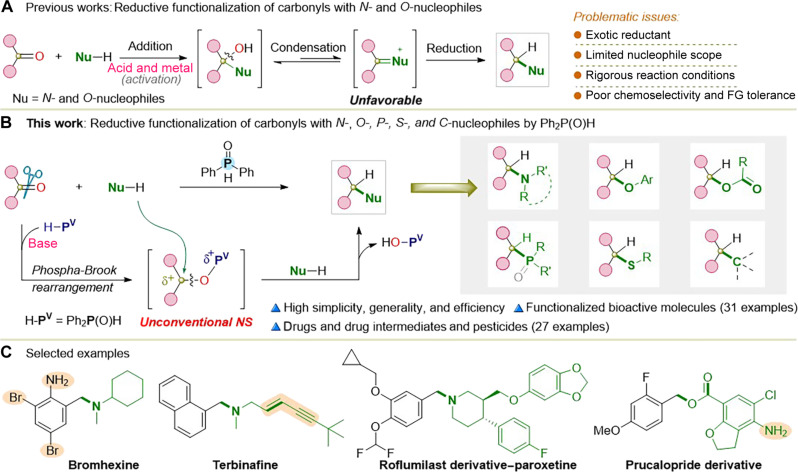
Strategies for carbonyl reductive functionalization. (**A**) Previous carbonyl reductive functionalization strategy. (**B**) Our strategy: direct and versatile reductive functionalizations by Ph_2_P(O)H. (**C**) Selected examples for drug and pesticide syntheses by our strategy.

The construction of functional molecules from readily available and abundant starting materials is a fundamental goal in chemistry and related sciences. In this context, carbonyl compounds, i.e., aldehydes and ketones, have become preferred feedstocks for numerous valuable transformations, owing to their high accessibility and chemical versatility (*1*–*5*). Among them, the reductive functionalization, using carbonyls as green electrophilic alkyl counterparts, stands out as a particularly lucrative avenue in synthetic chemistry (*3*–*13*). This strategy not only enables the skeletally and substitutionally diverse synthesis of higher-level chemicals but also avoids many undesirable side reactions, such as elimination and overalkylation of alkyl halides. As a result, it is becoming an ideal alternative to traditional halide chemistry; for example, at least a quarter of C─N bond formations are performed using carbonyl reductive amination in the pharmaceutical industry (*14*, *15*). However, reductive functionalization of carbonyl compounds encounters challenges in terms of cleavage and transformation of the strong C─O double bond. These difficult processes inevitably require the assistance of exotic reductants, transition-metal catalysts, and/or rigorous reaction conditions, and, in some cases, carbonyls even need to be preconverted into hydrazones to enable subsequent functionalization (*4*, *5*). Despite the forcing conditions, many types of carbonyl reductive functionalization reactions are intrinsically impeded or unrealized. Especially, the difficulty in forming iminium ion (C═N^+^) intermediates results in much less development of reductive amination with secondary amines, frequently encountering limitations in terms of substrate scope and compatibility with functional groups (*16*, *17*); the carbonyl etherification with phenols, esterification with carboxylic acids, and phosphinylation with P─H reagents have not yet been realized because of the challenge of forming unstable and highly reactive oxocarbenium ion (C═O^+^) and phosphonium ion (C═P^+^) intermediates, respectively (Fig. 1A) (*18*–*20*).

Herein, we present a simple and extremely versatile carbonyl reductive functionalization strategy achieving direct, highly selective, efficient, and general reductive amination, etherification, esterification, and phosphinylation reactions of (hetero)aryl aldehydes/ketones, which are extremely challenging or unattainable to achieve, using only diphenylphosphine oxide [Ph_2_P(O)H] and an inorganic base. These reductive functionalizations enable modular, structurally, and functionally diverse synthesis of (hetero)arylmethyl tertiary amines, ethers, esters, phosphine oxides, etc., as well as related pesticides, drug intermediates, pharmaceuticals, and their derivatives. Compared with classic carbonyl reductive functionalizations that involve addition, condensation, and subsequent reduction, our strategy proceeds via Pudovik addition, phospha-Brook rearrangement, and unconventional nucleophilic substitution, representing a previously unknown pathway for carbonyl reductive functionalization (Fig. 1B).

Phosphorus-mediated organic reactions, which involve phosphorus reagents but delivering phosphorus-free products, prove to undertake the precise C-element bond formation under metal-free conditions. Because of their excellent selectivity and practicability, phosphorus-mediated organic reactions like the Wittig, Appel, Mitsunobu, and Staudinger reactions have made great contributions in chemistry and the related fields for building complex chemicals, pharmaceuticals, natural products, and functional molecules (*21*–*24*). However, these reactions, as well as notable advancements in recent decades (*25*–*28*), commonly use nucleophilic P(III) reagents (e.g., triphenylphosphine) to generate phosphonium salts as intermediates and release tertiary phosphine oxides, such as triphenylphosphine oxide, as byproducts that are considered worthless and not easily separable. Our work is notably distinct from the known methods as it directly uses Ph_2_P(O)H, a typical P(V) reagent, produces phosphinates as intermediates, and releases easily isolatable and commercially valuable Ph_2_P(O)OH. Phosphorus-mediated reactions involving transformation of aldehydes and ketones, namely Wittig, Aza-Wittig, Corey-Fuchs, and Seyferth-Gilbert homologation reactions, have gained substantial recognition as famous name reactions. Rather than these name reactions that all transform C─O double bonds into other unsaturated bonds, our strategy realized the transformation of them into C-element (N, O, P, S, C, halogen) single bonds, representing a previously unknown phosphorus-mediated transformation of aldehydes and ketones.

Therefore, this Ph_2_P(O)H-mediated carbonyl reductive functionalization strategy represents an important advancement in the field of both carbonyl reductive functionalization and phosphorus-mediated transformation of aldehydes/ketones. Because of its extremely simple conditions, facile operation, a broad scope of substrates, remarkable functional group compatibility, and excellent selectivity, as well as avoidance of transition metals, external reductants, and alkyl halides, the reaction also provides an ideal alternative to the state-of-art fundamental reactions, enabling modular synthesis of high-order chemicals, particularly those with privileged structures, reductive and/or unprotected polar functional groups that are challenging to obtain using other methods.

## RESULTS AND DISCUSSION

By the direct treatment of benzaldehyde, *N*-methylpentylamine, and Ph_2_P(O)H with cesium carbonate (Cs_2_CO_3_) in acetonitrile (CH_3_CN) at 110°C for 10 min, we observed phosphinate **1B**, a [1,2]-phospha–Brook–type rearrangement product, in 90% gas chromatography (GC) yield (see the Supplementary Materials for details). The presence of *N*-nucleophile (*N*-methylpentylamine) did not affect the carbonyl transformation. Unexpectedly, by prolonging the reaction time, a nucleophilic substitution of **1B** occurred at the C atom within its C─O─P unit, and a carbonyl reductive amination product (**1**) was isolated in an excellent yield (92%), releasing the easily isolatable and commercially valuable diphenylphosphinic acid as the byproduct. Notably, phosphinates and in situ–formed ones are known to be used as electrophiles in transition-metal catalyzed cross-coupling reactions via the C─O bond cleavage within their C─O─P unit (*29*–*31*). An outstanding example is Yamaguchi’s work (*31*), which achieved palladium-catalyzed coupling using in situ–generated phosphinate intermediates from diaryl ketones and Ph_2_P(O)H. However, it is well known that phosphinates undergo nucleophilic substitution at their P atom within their C─O─P unit due to the higher electrophilicity of the P atom, as observed in all previous studies (>200 references found in SciFinder^n^). Recently, Zhang’s group reported a phospha-Brook rearrangement reaction, where the nucleophilic substitution of the phosphinate products also occurred at their P atom (*32*). To the best of our knowledge, nucleophilic substitution of phosphinates at the C atom within their C─O─P unit has not been reported yet. Thus, this finding represents an unconventional nucleophilic substitution.

**Fig. 2.**
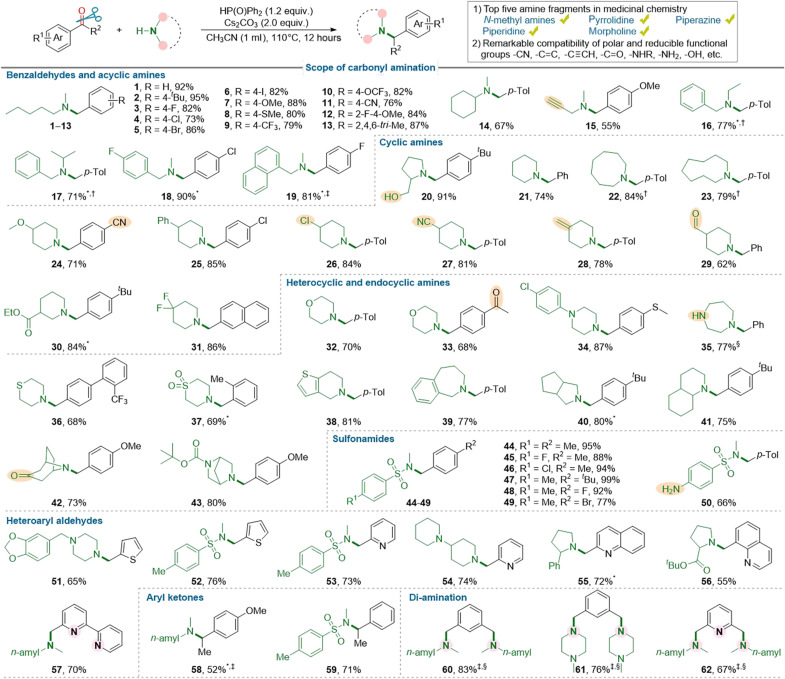
Substrate scope of reductive amination. Reaction conditions: carbonyl compound (0.24 mmol), amine (0.20 mmol), Ph_2_P(O)H (1.2 equiv.), Cs_2_CO_3_ (2.0 equiv.), and CH_3_CN (1.0 ml) under N_2_ at 110°C for 12 hours. Isolated yields are reported. *K_3_PO_4_ instead of Cs_2_CO_3_ as the base. †Reaction at 150°C. ‡Reaction at 130°C. §Carbonyl compound (0.2 mmol), amine (2.0 equiv.), Ph_2_P(O)H (2.0 equiv.).

As presented in Fig. 2, this extremely simple method allowed for the direct and selective synthesis of various tertiary amines in high to excellent yields from aryl aldehydes/ketones and secondary amines. Benzaldehydes bearing various electron-rich and electron-deficient substituents were found to be good substrates for the reaction (**1** to **13**). Then, we conducted the orthogonal reactions of aryl aldehydes with secondary amines, and it demonstrated a widespread substrate scope of secondary amines, as containing various types of acyclic (**14** to **19**), cyclic (**20** to **31**), heterocyclic (**32** to **39**), and endocyclic (**40** to **43**) skeletons and functional groups. The good compatibility of functional groups was highlighted by their presence in piperidine skeleton, one of the most prevalent frameworks in pharmaceuticals (*33*). In addition, secondary sulfonamides were also suitable substrates for the carbonyl amination reaction, producing complex tertiary sulfonamides (**44** to **50**) in high yields. Heteroaryl aldehydes also reacted smoothly with secondary amines to provide the desired heteroarylmethyl tertiary amines (**51** to **57**). Furthermore, the amination products of aryl ketones (**58** and **59**) were also obtained. Notably, direct diamination of (hetero)aryldialdehydes was also achieved (**60** to **62**), leading to the direct synthesis of potential tridentate pincer ligands (*34*). Unfortunately, aliphatic aldehydes and ketones are not suitable for this reductive amination reaction due to failure to form phosphinates as the key intermediates by [1,2]-phospha–Brook rearrangement (vide infra).

**Fig. 3.**
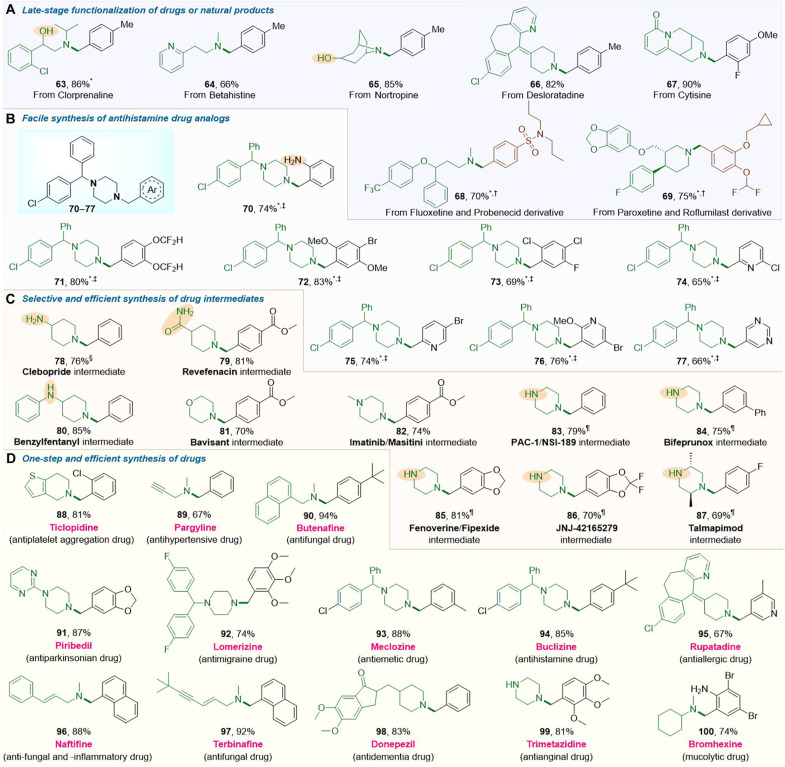
Applications in the synthesis of drug intermediates, drugs, and drug derivatives. (**A**) Late-stage functionalization of drugs and natural products. (**B**) The preparation of antihistamine drug analogs. (**C**) Selective and efficient synthesis of drug intermediates. (**D**) Direct and efficient synthesis of drugs. Reaction conditions: carbonyl compound (0.24 mmol), amine (0.20 mmol), Ph_2_P(O)H (1.2 equiv.), Cs_2_CO_3_ (2.0 equiv.), and CH_3_CN (1.0 ml) under N_2_ at 110°C for 12 hours. Isolated yields are reported. *K_3_PO_4_ instead of Cs_2_CO_3_ as the base. †Reaction at 140°C. ‡Reaction at 130°C. §Reaction of carbonyl compound and Ph_2_P(O)H for 20 min followed by the addition of amine. ¶Carbonyl compound (0.2 mmol), amine (2.0 equiv.), Ph_2_P(O)H (1.0 equiv.).

Tertiary amines containing arylmethyl and heteroarylmethyl units constitute an extremely important class of scaffolds in drugs, and more than 200 entries based on such fragments are found in the Drug Bank (*35*). Drugs like clorprenaline (**63**), betahistine (**64**), nortropine (**65**), desloratadine (**66**), and cytisine (**67**) were readily late-stage functionalized to form arylmethyl tertiary amine–based drug derivatives. Combined derivatives of two drugs were also obtained, such as compound **68** derived from fluoxetine and probenecid and compound **69** derived from paroxetine and roflumilast (Fig. 3A), potentially valuable for multi-targeting pharmaceuticals. Moreover, a series of highly functionalized drug analogs (**70** to **77**) based on the 1-diarylmethyl-4-(hetero)arylmethyl piperazine skeletons, which are found in many antihistamine drugs, were constructed modularly using various functionalized (hetero)aryl aldehydes (Fig. 3B). Furthermore, a variety of drug intermediates (**78** to **87**) could be selectively and efficiently synthesized, regardless of the presence of the reductive and reactive functional groups (Fig. 3C).

Commercially available feedstocks were successfully used to directly prepare dozens of drugs, such as ticlopidine (**88**, 81%), pargyline (**89**, 67%), butenafine (**90**, 94%), piribedil (**91**, 88%), lomerizine (**92**, 74%), meclizine (**93**, 88%), buclizine (**94**, 85%), rupatadine (**95**, 67%), naftifine (**96**, 88%), terbinafine (**97**, 92%), donepezil (**98**, 83%), trimetazidine (**99**, 81%), and bromhexine (**100**, 74%) (Fig. 3D). Notably, conventional synthesis of these drug and drug intermediates and their derivatives mainly rely on N-alkylation of alkyl halides and transition metal–catalyzed coupling reactions (*36*–*38*). Some cases need tedious multiple-step reactions. For instance, the drug bromhexine (**100**) was not directly synthesized by transition metal–catalyzed cross-coupling and N-alkylation because it contains susceptible bromo and primary aryl amino groups in its benzyl motif; instead, it is prepared through multistep processes in low yields (<45%) (*39*). In comparison with existing methods, this facile carbonyl amination not only enables the synthesis of these drugs and intermediates under transition metal- and organohalogen-free conditions but also achieves higher yields and selectivity directly from readily available feedstocks.

The construction of new and complex amine scaffolds, which encompass a wide range of structural units and polar functional groups, is a substantial challenge that often acts as a limiting factor in drug-discovery projects (*40*, *41*). Besides applicability to structurally diverse amine substrates, this carbonyl amination also exhibits decisive advantages in terms of functional group tolerance and selectivity. As shown in Figs. 1 and 2, the reducible groups such as terminal alkynyl, cyano, terminal alkenyl, *N*-Boc, aliphatic and aromatic esters, even aliphatic aldehydic, aliphatic, and aromatic keto carbonyls that are reactive in classic carbonyl reductive aminations, are tolerable. Furthermore, unprotected polar functional groups, such as primary and secondary alcohol, primary and secondary aryl amino, primary alkyl amino, and primary amide, were also well compatible. In addition, the primary amino group, which is more reactive than secondary alkyl amino group because of the easier formation of the C─N double bond (imines), was selectively accommodated. Consequently, this reductive functionalization is an excellent alternative to conventional amination reactions such as N-alkylation, transition metal–catalyzed cross-coupling, and classic reductive amination, particularly regarding substrate scope, polar functional group compatibility, and chemoselectivity. Therefore, it holds great potential to synthesize arylmethyl and heteroarylmethyl tertiary amines with diverse structural and functional properties that were previously challenging to access, thereby advancing drug discovery efforts. Gratifyingly, this reductive functionalization strategy enabled the direct formation of C─O single bond through the treatment of (hetero)aryl aldehydes/ketones with phenols and carboxylic acids, respectively, producing the corresponding unsymmetrical ethers and esters in high yields.

**Fig. 4.**
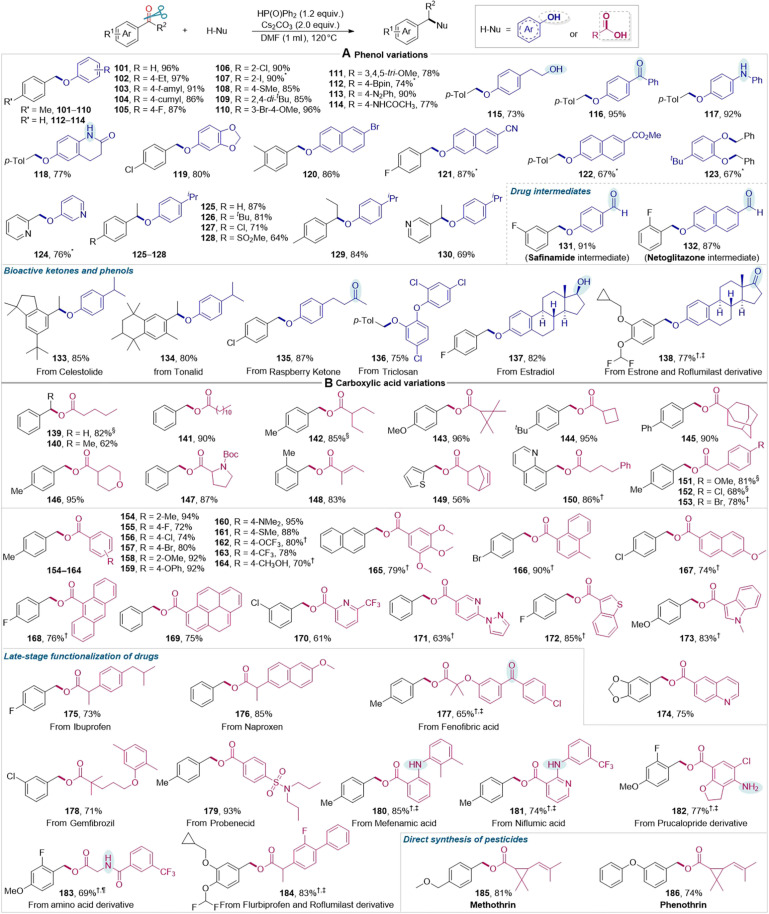
Substrate scope of carbonyl reductive etherification and esterification. (**A**) Carbonyl reductive etherification of phenols. (**B**) Carbonyl reductive etherification of carboxylic acids. Reaction conditions: carbonyl compound (0.24 mmol), *O*-nucleophile (0.20 mmol), Ph_2_P(O)H (1.2 equiv.), Cs_2_CO_3_ (2.0 equiv.), and *N*,*N*′-dimethylformamide (DMF, 1.0 ml) under N_2_ at 120°C for 12 hours. Isolated yields are reported. *CH_3_CN instead of DMF as the solvent. †K_3_PO_4_ instead of Cs_2_CO_3_ as the base. ‡Reaction at 140°C. §Cs_2_CO_3_ (1.0 equiv.). ¶Reaction at 130°C.

For the carbonyl reductive etherification (Fig. 4A), a wide range of phenol derivatives, including substituted phenols (**101** to **119**), naphthols (**120** to **122**), catechol (**123**), and heteroaryl phenol (**124**) all worked efficiently, which delivered structurally and functionally diverse unsymmetric (hetero)aryl ethers in 67 to 97% yields. For (hetero)aryl ketones (**125** to **130**), the reaction also proceeded smoothly without the elimination of their α-H. Phenol substrates containing reactive aromatic aldehydic carbonyls underwent selective and efficient etherification to produce the intermediate of safinamide (**131**) and intermediate of netoglitazone (**132**), respectively, while leaving the aldehydic carbonyls untouched. Even more complex feedstocks derived from both bioactive ketones (celestolide, **133**; tonalid, **134**) and phenol derivatives (raspberry ketone, **135**; triclosan, **136**; estradiol, **137**; estrone, **138**) proved to be viable substrates, providing the desired late-stage functionalized ethers in high yields. Notably, primary and secondary alcohols (**115** and **137**), which are reactive in reductive etherification, were also selectively tolerated.

Similarly, the reductive esterification of (hetero)aryl aldehydes and ketones with aliphatic carboxylic acids with various structural skeletons, such as linear (**139** to **141**) and branched (**142**) structures, strained three- and four-membered rings (**143** and **144**), bulky adamantine (**145**) and heterocycles (**146** and **147**), as well as α,β-unsaturated carboxylic acids (**148**), proceeded smoothly (Fig. 4B). The reaction was also effective with various benzyl (**151** to **153**), aromatic (**154** to **165**), and polyaromatic (**166** to **169**) carboxylic acids. In addition, heteroaromatic carboxyl acids were also viable substrates (**170** to **174**). Significantly, the late-stage functionalization of complex drug molecules and amino acid derivatives (**175** to **184**) was readily conducted, leaving unprotected aryl primary (**182**) and secondary (**180** and **181**) amino groups unaffected, which are typically intolerant in conventional esterification reactions. Moreover, this esterification method also enabled the direct synthesis of pesticides methothrin (**185**, 81% yield) and phenothrin (**186**, 74% yield), which feature an alkenyl group and/or strained three-membered ring.

Both ethers and esters are vital structural motifs in pharmaceuticals, natural products, agricultural chemicals, and functional materials (*42*–*46*). There are without doubt numerous methods available for ether and ester synthesis due to the fundamental nature of etherification and esterification reactions in chemistry, which have been evolving for almost two centuries. However, traditional etherification and esterification strategies, such as intramolecular dehydration etherification/esterification and O-alkylation with alkyl halides, encounter various problematic issues in terms of chemoselectivity, substrate scope, and functional group compatibility. This strategy used aldehydes and ketones as alkylating reagents to realize highly selective O-alkylation of phenols and carboxylic acids, respectively, under very simple conditions with broad substrate scope and remarkable functional group tolerance. Thus, this direct O-alkylation presents an alternative etherification and esterification strategy that enables a facile, green, and skeletally and substitutionally diverse synthesis of unsymmetrical ethers and esters.

**Fig. 5.**
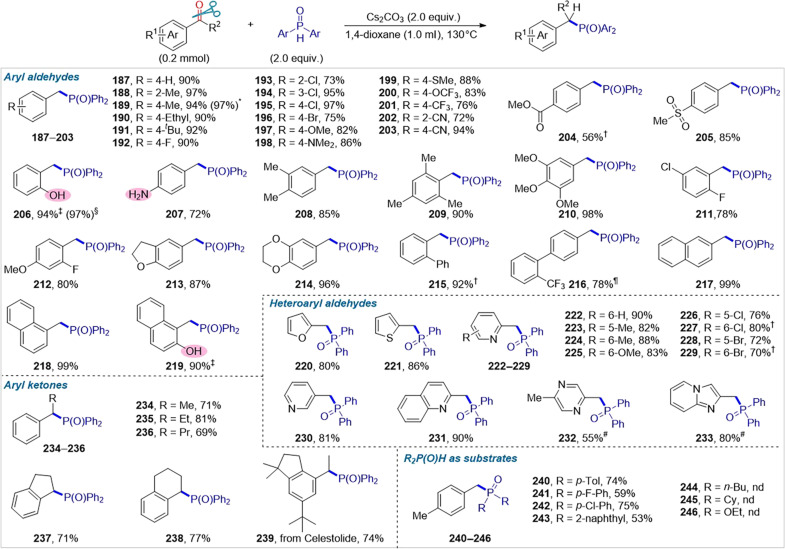
Substrate scope of carbonyl reductive phosphinylation. Reaction conditions: carbonyl compound (0.2 mmol), Ph_2_P(O)H (2.0 equiv.), Cs_2_CO_3_ (2.0 equiv.), and 1,4-dioxane (1.0 ml) under N_2_ at 130°C for 12 hours. Isolated yields are reported. *Yield of reaction is reported on a 6-mmol scale. †K_3_PO_4_ (2.0 equiv.) instead of Cs_2_CO_3_ as the base. ‡K_3_PO_4_ (2.0 equiv.) and dimethyl sulfoxide (1.0 ml) as the base and solvent, respectively. §Yield of reaction is reported on a 5-mmol scale. ¶DMF (1.0 ml) instead of 1,4-dioxane as the solvent. #K_3_PO_4_ (2.0 equiv.) and DMF (1.0 ml) as the base and solvent, respectively.

As shown in Fig. 5, this strategy also elicited the direct reductive phosphinylation of carbonyls with secondary phosphine oxides under extremely simple conditions. Aryl (**187** to **219**) and heteroaryl (**220** to **233**) aldehydes reacted smoothly with Ph_2_P(O)Hs to produce arylmethyl and heteroarylmethyl phosphine oxides in high to excellent yields with remarkable tolerance toward various functional groups. Aryl ketones also proved to be viable substrates, providing the corresponding α-substituted benzyl phosphine oxides (**234** to **239**) in 69 to 81% yields. Notably, aromatic aldehydes containing a phenolic hydroxyl (**206** and **219**), which was a reactive group in the aforementioned reductive etherification, performed well by slightly tuning the reaction conditions. Thus, 2-hydroxylbenzyl diphenyl phosphine oxide (**206**), which is the key catalyst in the work of Denton (*47*) and is typically prepared with only 68% yield in three steps under harsh reaction conditions, can now be directly produced with a better result (1.49 g, 97%) in a 5-mmol scale-up reaction. Notably, Yamaguchi’s group has pioneered a palladium/1,2-bis(dicyclohexylphosphino)ethane-catalyzed reductive coupling of (hetero)aryl esters with Ph_2_P(O)Hs for the synthesis of α-unsubstituted benzyl phosphine oxides (*48*).

The development of C─P bond–forming methods is of crucial importance in modern organophosphorus chemistry as it contributes to mitigating dependence on traditional synthetic methods, particularly the Michaelis-Arbuzov reaction and substitution of phosphorus halides with organometallic reagents. Therefore, this facile, general, and transition metal– and halogen-free strategy represents a notable advancement in C─P bond–forming reactions.

**Fig. 6.**
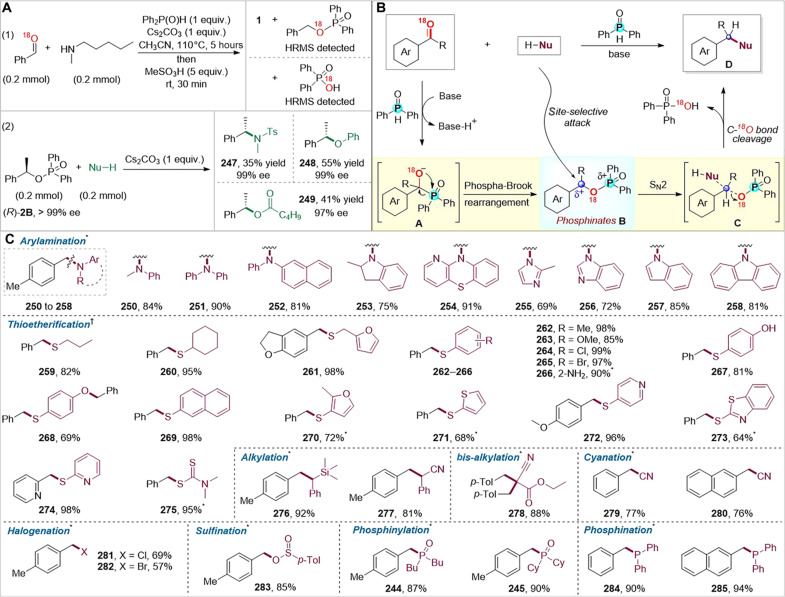
Proposed mechanism and reaction extension. (**A**) Control experiments. (**B**) Reaction pathway. (**C**) Other types of carbonyl reductive transformations. Reaction conditions (see the Supplementary Materials for details): *aldehyde (0.2 mmol), Ph_2_P(O)H (0.2 mmol), Cs_2_CO_3_ (1.0 equiv.), and solvent (1.0 ml) under N_2_ at 110°C for 20 min; then, nucleophile (1 to 1.5 equiv.) was added and allowed to continue for 12 hours. †Aldehyde (0.24 mmol), nucleophile (0.2 mmol), Ph_2_P(O)H (0.24 mmol), Cs_2_CO_3_ (2.0 equiv.), and solvent (1.0 ml) under N_2_ at 120°C for 12 hours.

The ^18^O isotope labeling experiments (see the Supplementary Materials for details) demonstrated that a [1,2]-phospha–Brook–type rearrangement product ^18^O-labeled phosphinate (^18^O-**1B**) was observed in 93% yield at the beginning of the reaction (20 min), which underwent selective nucleophilic substitution with *N*-methylpentylamine at the C atom within its C─O─P unit, releasing ^18^O-labeled Ph_2_P(O)^18^OH as the byproduct. Upon reacting arylmethylphosphinate (*R*)-**2B** [99% enantiomeric excess (ee)] with *N*-methyl-*p*-toluenesulfonamide, phenol, and *n*-valeric acid, respectively, the corresponding chiral compounds, namely chiral sulfonamide (**247**), ether (**248**), and ester (**249**) were yielded with ee values of 99, 99, and 97%, respectively. These results suggested that the key step of our strategy proceeded via S_N_2 nucleophilic substitution, and stereospecific nucleophilic substitution reactions might be developed by extension of this strategy. Thus, a plausible reaction mechanism was proposed (Fig. 6B). Initially, a Ph_2_P(O)H anion is formed in the presence of the base, which conducts the nucleophilic attack at the carbonyl to form anion intermediate **A**. Intermediate **A** rapidly undergoes a [1,2]-phospha–Brook–type rearrangement to form arylmethylphosphinate **B** (*31*, *49*), which is attacked by the nucleophiles via selective C─O cleavage to form the desired product (**D**), concomitantly releasing easily isolatable and commercially valuable diphenylphosphinic acid as the byproduct.

Enlightened by the mechanism pathway, this strategy was successfully extended to the direct and versatile formations of other chemical bonds, such as C─N (aryl), C─S, C─C(sp^3^), C─CN, C─X (Cl, Br), C─OS(O)R, and C─P(III) bonds (Fig. 6C). By using other nucleophiles, including secondary (hetero)aryl amines, *N*-heteroarenes, *S*-nucleophiles, *C*-nucleophiles, trimethylsilyl cyanide, ethyl cyanoacetate, butylzinc halides, and sodium benzenesulfinate, we achieved reductive arylamination (**250** to **258**), thioetherification (**259** to **275**), alkylation (**276** and **277**), bis-alkylation (**278**), cyanation (**279** and **280**), halogenation (**281** and **282**), and sulfination (**283**), respectively, obtaining the corresponding products in good to excellent yields. Dialkylphosphine oxides were also incorporated into corresponding products (**244** and **245**) in high yields using their ionic salts as the nucleophiles. To our delight, this protocol was also extended to diphenylphosphine, allowing for reductive phosphination of carbonyls to directly produce trivalent phosphorus compounds (**284** and **285**) in excellent yields, which not only avoids tedious multiple syntheses but also eliminates the need for additional isolation procedures.

In summary, the simple carbonyl reductive functionalization strategy by Ph_2_P(O)H allows for the direct construction of versatile chemical bonds, including C─NR_2_, C─OAr, C─OC(O)R, C─P(O)R_2_, C─N(*sp*^2^C), C─S, C─C(sp^3^), C─CN, C─X (Cl, Br), C─OS(O)R, and C─P(III) bonds. Successful demonstrations of its modular, efficient, and functionally and structurally diverse synthesis have been achieved for (hetero)arylmethyl tertiary amines, ethers, esters, phosphine oxides, as well as related pesticides, drug intermediates, pharmaceuticals, and their derivatives. This strategy overcomes the limitations of conventional carbonyl reductive functionalization, halide chemistry, and transition metal–catalyzed cross-coupling, particularly regarding substrate scope, polar functional compatibility, and selectivity, thus providing important strides in field of reductive functionalization and phosphorus-mediated transformation of aldehydes/ketones, as well as the fundamental reactions, especially amination, etherification, and esterification.

## MATERIALS AND METHODS

### General experimental procedures

The reactions were carried out in Schlenk tubes of 25 ml under N_2_ atmosphere. Reagents were used as received unless otherwise noted, and solvents were purified according to standard operation procedure. Column chromatography was performed using Silica Gel 60 (300 to 400 mesh). The reactions were monitored by GC and GC–mass spectrometry (GC-MS), GC-MS results were recorded on GC-MS QP2010, and GC analysis was performed on GC 2014 plus. With the exception of a few compounds that were analyzed using a Brucker-600 spectrometer [600 MHz for ^1^H nuclear magnetic resonance (NMR), 151 MHz for ^13^C NMR, and 565 MHz for ^19^F NMR], most of the samples were recorded on a Brucker-400 (400 MHz for ^1^H NMR, 101 MHz for ^13^C NMR, 376 MHz for ^19^F NMR, and 162 MHz for ^31^P NMR) spectrometer, and chemical shifts were reported in parts per million (ppm). Chemical shifts for ^1^H NMR are referred to internal Me_4_Si (0 ppm) and reported as follows: chemical shift (δ ppm), multiplicity, coupling constant (hertz), and integration. Data for ^31^P NMR were referred to H_3_PO_4_ (85% solution in D_2_O, 0 ppm). Electron impact ionization (EI) or atmospheric pressure chemical ionization (APCI) was used as the ionization method for the high-resolution MS measurement, and the mass analyzer type is time of flight for EI. The ee was determined by high-performance liquid chromatography analysis using the corresponding commercial chiral column as stated in the experimental procedures with ultraviolet detector at 220, 230 or 254 nm. Unless specified, all solvents and reagents were purchased from Energy Chemical, Alfa Aesar, Sigma-Aldrich, and Aladdin.

### General method for the synthesis of amines

Unless otherwise noted, all reactions are conducted in a glove box under a N_2_ atmosphere. An oven-dried Schlenk tube of 25 ml equipped with a magnetic stir bar was charged with Cs_2_CO_3_ (0.4 mmol, 2.0 equiv.), and Ph_2_P(O)H (0.24 mmol, 1.2 equiv.), carbonyl compound (0.24 mmol, 1.2 equiv.), amine (0.20 mmol), and CH_3_CN (1.0 ml) were added. The reaction mixture was heated at 110°C for 12 hours under N_2_. After completion of the reaction, the reaction mixture was cooled to room temperature and washed with saturated NH_4_Cl aqueous solution (5.0 ml). The reaction mixture was then extracted with dichloromethane (3 × 5 ml), and the organic layer was dried over anhydrous Na_2_SO_4_ and concentrated under vacuum. The desired aminated products (**1** to **100**) were isolated by column chromatography [0.1 to 10% (2.0 M aq. NH_3_ in MeOH) in dichloromethane] over silica gel (300 to 400 mesh).

### General method for the synthesis of ethers and carboxylic esters

Unless otherwise noted, all reactions are conducted in a glove box under a N_2_ atmosphere. An oven-dried Schlenk tube of 25 ml equipped with a magnetic stir bar was charged with Cs_2_CO_3_ (0.4 mmol, 2.0 equiv.), and Ph_2_P(O)H (0.24 mmol, 1.2 equiv.), *O*-nucleophile (0.20 mmol), carbonyl compound (0.24 mmol, 1.2 equiv.), and *N*,*N*′-dimethylformamide (DMF, 1.0 ml) were added. The reaction mixture was heated at 120°C for 12 hours under N_2_. After completion of the reaction, the reaction mixture was cooled to room temperature and washed with saturated NH_4_Cl aqueous solution (5.0 ml). The reaction mixture was then extracted with dichloromethane (3 × 5 ml). The organic layer was dried over anhydrous Na_2_SO_4_ and was concentrated under vacuum. The desired products (**101** to **186**) were isolated by column chromatography (eluent: ethyl acetate/petroleum ether = 1/30 to 1/5) over silica gel (300 to 400 mesh).

### General method for the synthesis of functionalized phosphine oxides

Unless otherwise noted, all reactions are conducted in a glove box under a N_2_ atmosphere. An oven-dried Schlenk tube of 25 ml equipped with a magnetic stir bar was charged with Cs_2_CO_3_ (0.2 mmol), and Ar_2_P(O)H (0.4 mmol, 2.0 equiv.), carbonyl compound (0.2 mmol), and 1,4-dioxane (1.0 ml) were added. The reaction mixture was heated at 130°C for 12 hours under N_2_. After completion of the reaction, the reaction mixture was cooled to room temperature and washed with saturated NH_4_Cl aqueous solution (5.0 ml). The reaction mixture was then extracted with dichloromethane (3 × 5 ml). The organic layer was dried over anhydrous Na_2_SO_4_ and concentrated under vacuum. The desired products (**187** to **243**) were isolated by column chromatography (eluent: ethyl acetate/petroleum ether = 1/5 to 3/1) over silica gel (300 to 400 mesh).

## Supplementary Materials

### This PDF file includes:

Supplementary Methods Supplementary Text Tables S1 to S4 Figs. S1 to S12 References
