# Supplementary material for: Direct carbonyl reductive functionalizations by diphenylphosphine oxide
Source: Sci Adv. 2025 Feb 7;11(6):eads4626. doi: 10.1126/sciadv.ads4626 (PMC11804924; doi:10.1126/sciadv.ads4626)
Supplement: Supplementary file 1 — Supplementary Methods Supplementary Text Tables S1 to S4 Figs. S1 to S12 References [file sciadv.ads4626_sm.pdf]

Science Advances
AAAS

Supplementary Materials for

Direct carbonyl reductive functionalizations by diphenylphosphine oxide

Feng Liu *et al.*

Corresponding author: Yongbo Zhou, zhouyb@hnu.edu.cn; Jianyu Dong, djyustc@hotmail.com; Chao-Jun Li, cj.li@mcgill.ca

Sci. Adv. **11**, eads4626 (2025)
DOI: 10.1126/sciadv.ads4626

This PDF file includes:

Supplementary Methods
Supplementary Text
Tables S1 to S4
Figs. S1 to S12
References

1. Supplementary Methods

The reactions were carried out in Schlenk tubes of 25 mL under N_2 atmosphere. Reagents were used as received unless otherwise noted, and solvents were purified according to standard operation procedure. Column chromatography was performed using Silica Gel 60 (300–400 mesh). The reactions were monitored by GC and GC-MS, GC-MS results were recorded on GC-MS QP2010, and GC analysis was performed on GC 2014 plus. With the exception of a few compounds that were analyzed using a Brucker-600 spectrometer (600 MHz for ^{1}H NMR, 151 MHz for ^{13}C NMR and 565 MHz for ^{19}F NMR), the majority of the samples were recorded on a Brucker-400 (400 MHz for ^{1}H NMR, 101 MHz for ^{13}C NMR, 376 MHz for ^{19}F NMR and 162 MHz for ^{31}P NMR) spectrometer, and chemical shifts were reported in parts per million (ppm). Chemical shifts for ^{1}H NMR are referred to internal Me_4Si (0 ppm) and reported as follows: chemical shift (δ ppm), multiplicity, coupling constant (Hz) and integration. Data for ^{31}P NMR were referred to H_3PO_4 (85% solution in D_2O, 0 ppm). The electron ionization (EI or APCI) method was used as the ionization method for the HRMS measurement, and the mass analyzer type is TOF for EI. Enantiomeric excess was determined by HPLC analysis using the corresponding commercial chiral column as stated in the experimental procedures with UV detector at 220, 230 or 254 nm. Unless specified, all solvents and reagents were purchased from Energy Chemical, Alfa Aesar, Sigma-Aldrich and Aladdin.

2. General Experimental Procedure

2.1 Table S1: Optimization of the Carbonyl Reductive Amination Reaction.

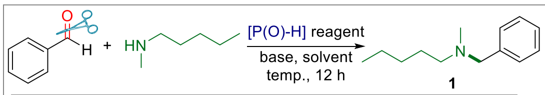

entry	[P(O)-H]	base	solvent	temp. (°C)	yield (%)
1	**P-1**	Cs_2CO_3	DMSO	120	44
2	**P-1**	Cs_2CO_3	1,4-dioxane	120	60
3	**P-1**	Cs_2CO_3	DMF	120	74
4	**P-1**	Cs_2CO_3	acetonitrile	120	81
5	**P-1**	Cs_2CO_3	EA	120	65
6	**P-1**	K_3PO_4	acetonitrile	120	77
7	**P-1**	Na_2CO_3	acetonitrile	120	25
8	**P-1**	K_2CO_3	acetonitrile	120	41
9	**P-1**	KOH	acetonitrile	120	51
10	**P-1**	NaOH	acetonitrile	120	33
11	**P-1**	*t*-BuOK	acetonitrile	120	11
12	**P-1**	*t*-BuONa	acetonitrile	120	trace
13	**P-1**	Cs_2CO_3	acetonitrile	50	N.D.
14	**P-1**	Cs_2CO_3	acetonitrile	80	49
15	**P-1**	Cs_2CO_3	acetonitrile	100	71
16	**P-1**	Cs_2CO_3	acetonitrile	110	84
17	**P-1**	Cs_2CO_3	acetonitrile	130	79
18	**P-2**	Cs_2CO_3	acetonitrile	110	80
19	**P-3**	Cs_2CO_3	acetonitrile	110	70
20	**P-4**	Cs_2CO_3	acetonitrile	110	85
21	**P-5**	Cs_2CO_3	acetonitrile	110	66
22	**P-6**	Cs_2CO_3	acetonitrile	110	trace
23	**P-7**	Cs_2CO_3	acetonitrile	110	72
24	**P-8**	Cs_2CO_3	acetonitrile	110	60
25	**P-9**	Cs_2CO_3	acetonitrile	110	85
26	**P-10**	Cs_2CO_3	acetonitrile	110	49
27	**P-11**	Cs_2CO_3	acetonitrile	110	N.D.
28	**P-12**	Cs_2CO_3	acetonitrile	110	N.D.
29	**P-13**	Cs_2CO_3	acetonitrile	110	N.D.
30*	**P-1**	Cs_2CO_3	acetonitrile	110	95 (92)

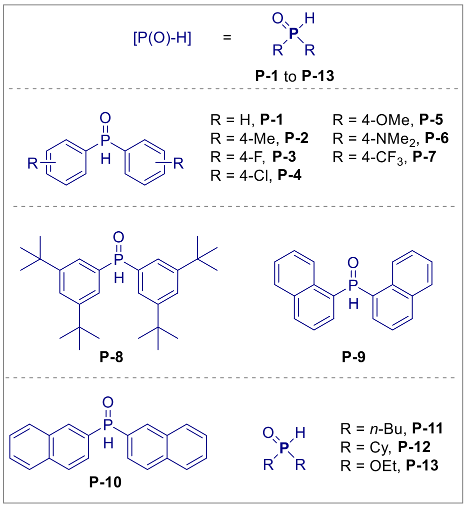

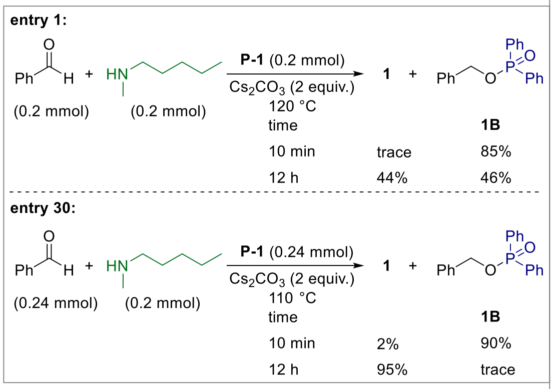

Reaction conditions: benzaldehyde (0.20 mmol), *N*-methylpentylamine (0.20 mmol), [P(O)-H] reagent (0.20 mmol), base (2.0 equiv.), solvent (1.0 mL), under N_2 for 12 h. GC yields using *n*-tridecane as internal standard. *Benzaldehyde (0.24 mmol), *N*-methylpentylamine (0.20 mmol), $Ph_2P(O)H$ (**P-1**, 1.2 equiv.).

Notes: Although [P(O)-H] reagents **P-4** (85%) and **P-9** (85%) showed slightly better performance than **P-1** (84%), for economic reason, the more readily available **P-1** was chosen for further investigation of the substrate scope of the carbonyl amination reaction.

Unless otherwise noted, all reactions were conducted in a glove box under a N_2 atmosphere. An oven-dried Schlenk tube of 25 mL equipped with a magnetic stir bar was charged with base (0.4 mmol, 2.0 equiv.) and [P(O)-H] reagent (0.2 mmol), benzaldehyde (0.2 mmol), *N*-

methylpentylamine (0.20 mmol), and solvent (1.0 mL) were added. The reaction mixture was heated for 12 h under N_2. After completion of the reaction, the reaction mixture was cooled to room temperature. *n*-Tridecane (30 uL) as internal standard and dichloromethane (5 mL) were then added to the mixture, and GC yield of the reaction was calculated. The desired aminated product **1** was isolated by column chromatography (1% (2.0 M aq. NH_3 in MeOH) in dichloromethane) over silica gel (300–400 mesh)

2.2 General Experimental Procedure of the Carbonyl Reductive Amination Reaction.

1) Fig. S1: Substrate Scope of the Carbonyl Reductive Amination Reaction.

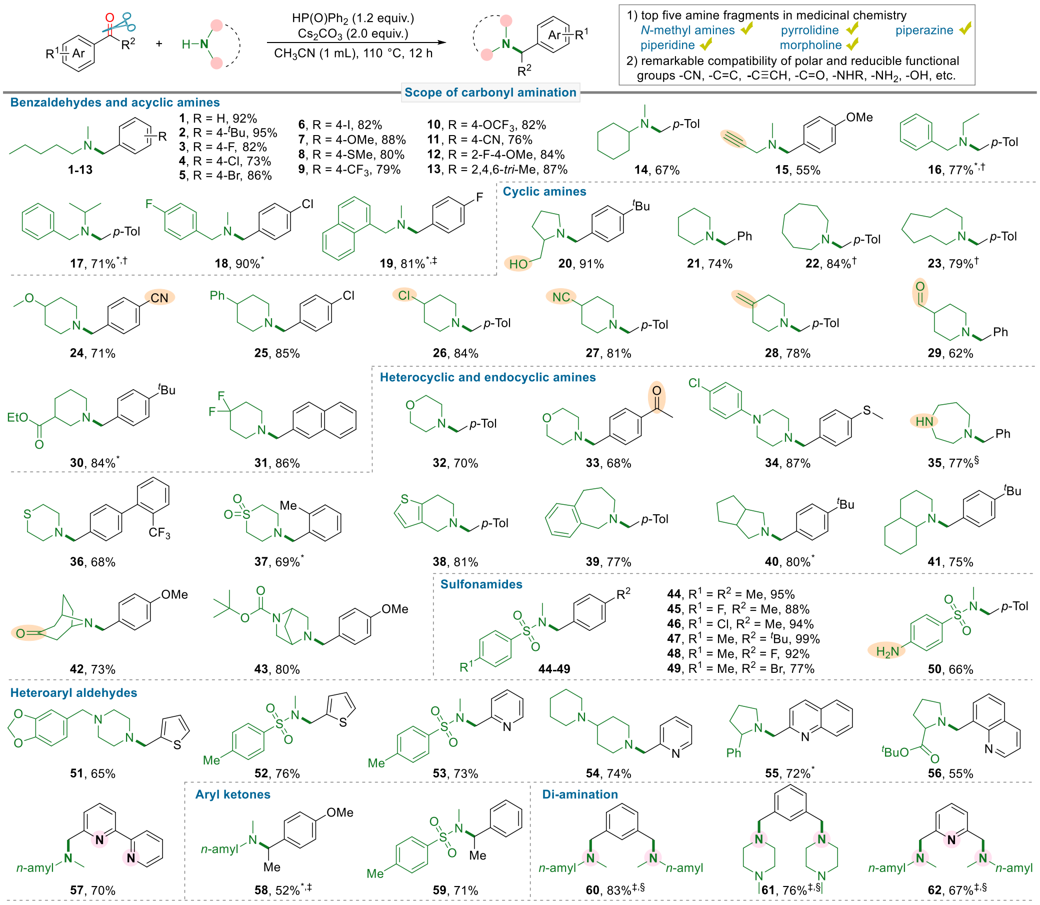

Reaction conditions: carbonyl compound (0.24 mmol), amine (0.20 mmol), $Ph_2P(O)H$ (1.2 equiv.), Cs_2CO_3 (2.0 equiv.), CH_3CN (1.0 mL), under N_2 at 110 °C for 12 h. Isolated yields are reported. *K_3PO_4 instead of Cs_2CO_3 as the base. †Reaction at 150 °C. ‡Reaction at 130 °C. §Carbonyl compound (0.2 mmol), amine (2.0 equiv.), $Ph_2P(O)H$ (2.0 equiv.).

2) Fig. S2: Applications in the Synthesis of Drug intermediates, Drugs, and Their Derivatives.

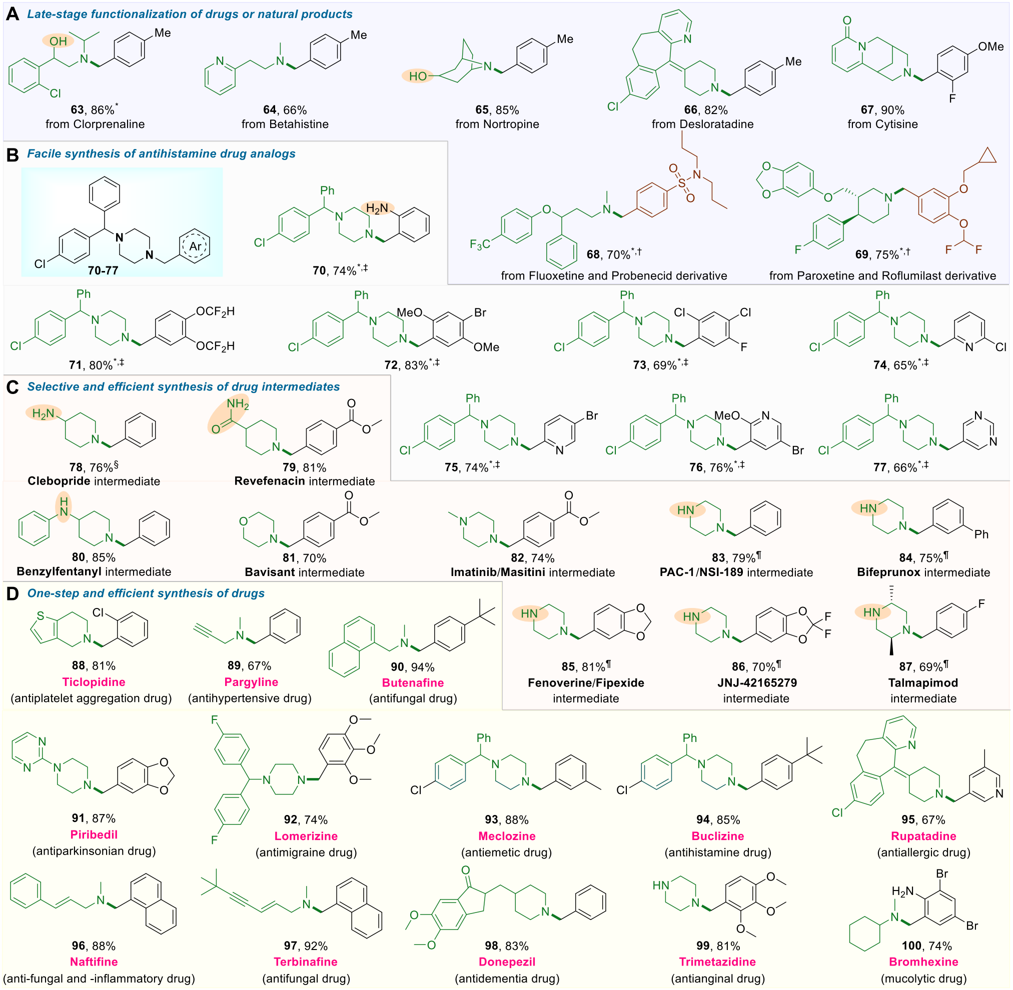

(**A**) Late-stage functionalization of drugs and natural products. (**B**) The preparation of antihistamine drug analogs. (**C**) Selective and efficient synthesis of drug intermediates. (**D**) Direct and efficient synthesis of drugs. Reaction conditions: carbonyl compound (0.24 mmol), amine (0.20 mmol), $Ph_2P(O)H$ (1.2 equiv.), Cs_2CO_3 (2.0 equiv.), CH_3CN (1.0 mL), under N_2 at 110 °C for 12 h. Isolated yields are reported. *K_3PO_4 instead of Cs_2CO_3 as the base. †Reaction at 140 °C; ‡Reaction at 130 °C. §Reaction of carbonyl compound and $Ph_2P(O)H$ for 20 min followed by the addition of amine. ¶Carbonyl compound (0.2 mmol), amine (2.0 equiv.), $Ph_2P(O)H$ (1.0 equiv.).

Unless otherwise noted, all reactions are conducted in a glove box under a N_2 atmosphere. An oven-dried Schlenk tube of 25 mL equipped with a magnetic stir bar was charged with Cs_2CO_3 (0.4 mmol, 2.0 equiv.) and $Ph_2P(O)H$ (0.24 mmol, 1.2 equiv.), carbonyl compound (0.24 mmol, 1.2 equiv.), amine (0.20 mmol), and CH_3CN (1.0 mL) were added. The reaction mixture was heated at 110 °C for 12 h under N_2. After completion of the reaction, the reaction mixture was cooled to room

temperature and washed with saturated NH_4Cl aqueous solution (5.0 mL). The reaction mixture was then extracted with dichloromethane (3 × 5 mL), and the organic layer was dried over anhydrous Na_2SO_4 and concentrated under vacuum. The desired aminated products (**1** to **100**) were isolated by column chromatography (0.1–10% (2.0 M aq. NH_3 in MeOH) in dichloromethane) over silica gel (300–400 mesh).

2.3 Table S2: Optimization of the Carbonyl Reductive Etherification Reaction.

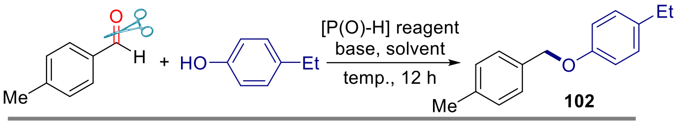

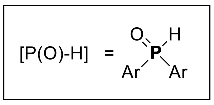

entry	[P(O)-H]	base	solvent	temp. (°C)	yield (%)
1	**P-1**	Cs_2CO_3	DMSO	120	65
2	**P-1**	Cs_2CO_3	1,4-dioxane	120	51
3	**P-1**	Cs_2CO_3	DMF	120	91
4	**P-1**	Cs_2CO_3	acetonitrile	120	79
5	**P-1**	Cs_2CO_3	EA	120	65
6	**P-1**	K_3PO_4	DMF	120	77
7	**P-1**	Na_2CO_3	DMF	120	44
8	**P-1**	K_2CO_3	DMF	120	67
9	**P-1**	KOH	DMF	120	59
10	**P-1**	NaOH	DMF	120	9
11	**P-1**	*t*-BuOK	DMF	120	trace
12	**P-1**	*t*-BuONa	DMF	120	N.D.
13	**P-1**	Cs_2CO_3	DMF	80	54
14	**P-1**	Cs_2CO_3	DMF	100	74
15	**P-1**	Cs_2CO_3	DMF	110	83
16	**P-1**	Cs_2CO_3	DMF	130	88
17	**P-2**	Cs_2CO_3	DMF	120	84
18	**P-3**	Cs_2CO_3	DMF	120	66
19	**P-4**	Cs_2CO_3	DMF	120	88
20	**P-5**	Cs_2CO_3	DMF	120	71
21	**P-9**	Cs_2CO_3	DMF	120	85
22*	**P-1**	Cs_2CO_3	DMF	120	99 (97)

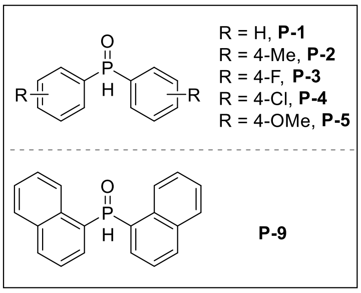

Reaction conditions: 4-tolualdehyde (0.20 mmol), 4-ethylphenol (0.20 mmol), [P(O)-H] reagent (1.0 equiv.), base (2.0 equiv.), solvent (1.0 mL), under N_2 for 12 h. GC yields using *n*-tridecane as internal standard. *4-Ethylphenol (0.20 mmol), 4-tolualdehyde (1.2 equiv.), $Ph_2P(O)H$ (1.2 equiv.).

Notes: Although the [P(O)-H] reagent **P-4** (88%) provided the same yield of etherification product **102** as the **P-1** (88%), for economic reason, the more readily available **P-1** was chosen for further investigation of the substrate scope of the carbonyl reductive etherification reaction.

Unless otherwise noted, all reactions are conducted in a glove box under a N_2 atmosphere. An oven-dried Schlenk tube of 25 mL equipped with a magnetic stir bar was charged with base (0.4 mmol, 2.0 equiv.) and [P(O)-H] reagent (0.2 mmol), 4-tolualdehyde (0.20 mmol), 4-ethylphenol (0.20 mmol), and solvent (1.0 mL) were added. The reaction mixture was heated for 12 h under N_2. After completion of the reaction, the reaction mixture was cooled to room temperature. *n*-Tridecane (30 uL) as internal standard and dichloromethane (5 mL) were then added to the mixture, and GC yield of the reaction was calculated. The desired etherification product **102** was isolated by column chromatography (eluent: ethyl acetate/petroleum ether = 1/10) over silica gel (300–400 mesh).

2.4 Table S3: Optimization of the Carbonyl Reductive Esterification Reaction.

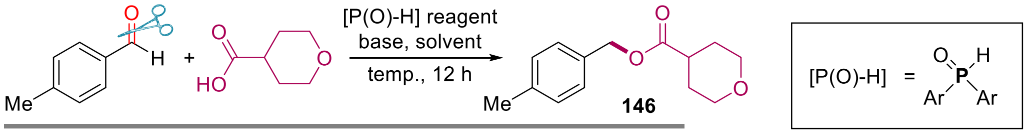

entry	[P(O)-H]	base	solvent	temp. (°C)	yield (%)
1	**P-1**	Cs_2CO_3	DMSO	120	67
2	**P-1**	Cs_2CO_3	1,4-dioxane	120	76
3	**P-1**	Cs_2CO_3	DMF	120	84
4	**P-1**	Cs_2CO_3	acetonitrile	120	71
5	**P-1**	K_3PO_4	DMF	120	77
6	**P-1**	Na_2CO_3	DMF	120	34
7	**P-1**	K_2CO_3	DMF	120	55
8	**P-1**	KOH	DMF	120	24
9	**P-1**	*t*-BuOK	DMF	120	trace
10	**P-1**	Cs_2CO_3	DMF	80	23
11	**P-1**	Cs_2CO_3	DMF	100	45
12	**P-1**	Cs_2CO_3	DMF	110	57
13	**P-1**	Cs_2CO_3	DMF	130	81
14	**P-2**	Cs_2CO_3	DMF	120	68
15	**P-3**	Cs_2CO_3	DMF	120	55
16	**P-4**	Cs_2CO_3	DMF	120	80
17	**P-5**	Cs_2CO_3	DMF	120	46
18	**P-9**	Cs_2CO_3	DMF	120	75
19*	**P-1**	Cs_2CO_3	DMF	120	97 (95)
20*	**P-1**	K_3PO_4	DMF	120	90

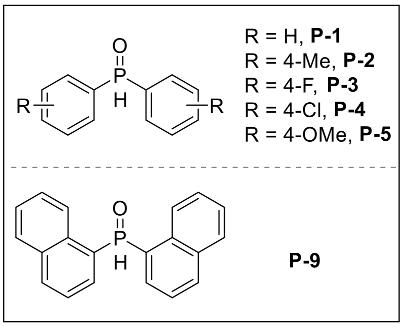

Reaction conditions: 4-tolualdehyde (0.20 mmol), tetrahydro-2*H*-pyran-4-carboxylic acid (0.20 mmol), [P(O)-H] reagent (1.0 equiv.), base (2.0 equiv.), solvent (1.0 mL), under N_2 for 12 h. GC yields using *n*-tridecane as internal standard. *4-Tolualdehyde (1.2 equiv.), $Ph_2P(O)H$ (1.2 equiv.).

Unless otherwise noted, all reactions are conducted in a glove box under a N_2 atmosphere. An oven-dried Schlenk tube of 25 mL equipped with a magnetic stir bar was charged with base (0.4 mmol, 2.0 equiv.) and [P(O)-H] reagent (0.2 mmol), 4-tolualdehyde (0.20 mmol), tetrahydro-2*H*-pyran-4-carboxylic acid (0.20 mmol), and solvent (1.0 mL) were added. The reaction mixture was heated for 12 h under N_2. After completion of the reaction, the reaction mixture was cooled to room temperature. *n*-Tridecane (30 uL) as internal standard and dichloromethane (5 mL) were then added to the mixture, and GC yield of the reaction was calculated. The desired esterification product **146** was isolated by column chromatography (eluent: ethyl acetate/petroleum ether = 1/10) over silica gel (300–400 mesh).

2.5 General Experimental Procedure of Carbonyl Reductive Etherification and Esterification Reactions.

Fig. S3: Substrate Scope of Carbonyl Etherification and Esterification Reactions.

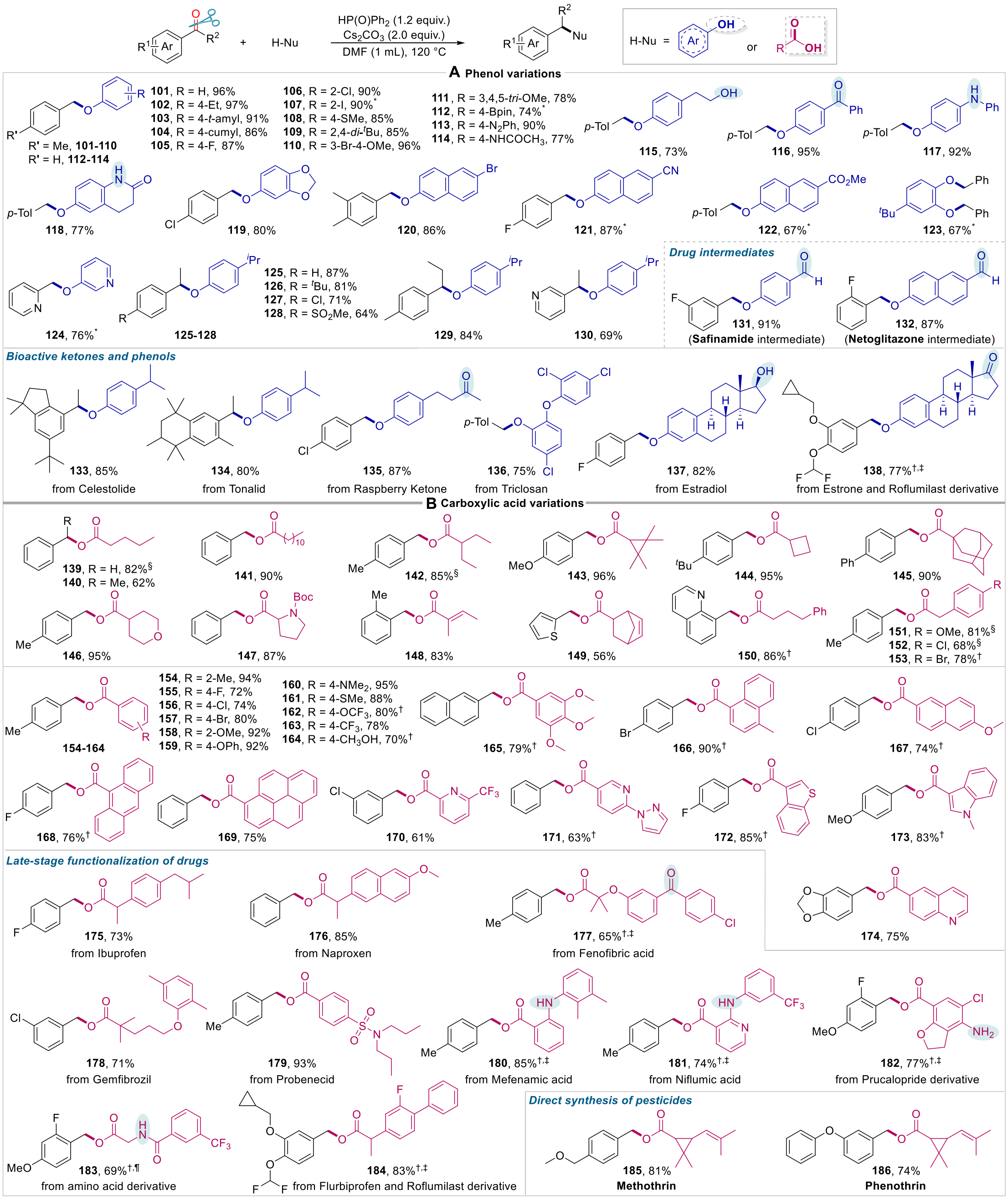

(**A**) Carbonyl etherification with phenols. (**B**) Carbonyl etherification with carboxylic acids. Reaction conditions: carbonyl compound (0.24 mmol), *O*-nucleophile (0.20 mmol), $Ph_2P(O)H$ (1.2 equiv.), Cs_2CO_3 (2.0 equiv.), DMF (1.0 mL), under N_2 at 120 °C for 12 h. Isolated yields are reported. *CH_3CN instead of DMF as the solvent. †K_3PO_4 instead of Cs_2CO_3 as the base. ‡Reaction at 140 °C. §Cs_2CO_3 (1.0 equiv.). ¶Reaction at 130 °C.

Unless otherwise noted, all reactions are conducted in a glove box under a N_2 atmosphere. An oven-dried Schlenk tube of 25 mL equipped with a magnetic stir bar was charged with Cs_2CO_3 (0.4 mmol, 2.0 equiv.) and $Ph_2P(O)H$ (0.24 mmol, 1.2 equiv.), *O*-nucleophile (0.20 mmol), carbonyl compound (0.24 mmol, 1.2 equiv.), and DMF (1.0 mL) were added. The reaction mixture was heated at 120 °C for 12 h under N_2. After completion of the reaction, the reaction mixture was cooled to room temperature and washed with saturated NH_4Cl aqueous solution (5.0 mL). The reaction mixture was then extracted with dichloromethane (3 × 5 mL). The organic layer was dried over anhydrous Na_2SO_4 and was concentrated under vacuum. The desired products (**101** to **186**) were isolated by column chromatography (eluent: ethyl acetate/petroleum ether = 1/30–1/5) over silica gel (300–400 mesh).

2.6 Table S4: Optimization of the Carbonyl Reductive Phosphinylation Reaction.

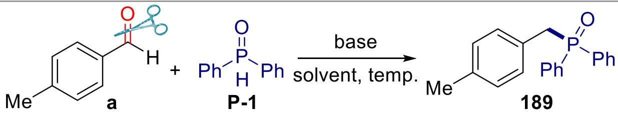

entry	a/P-1	base	solvent	temp. (°C)	yield (%)
1	1:1.5	Cs_2CO_3	1,4-dioxane	100	32
2	1:1	Cs_2CO_3	1,4-dioxane	100	N.D.
3	1:2	Cs_2CO_3	1,4-dioxane	100	70
4	1:2	Cs_2CO_3	1,4-dioxane	130	98 (94)
5	1:2	Cs_2CO_3	1,4-dioxane	140	95
6*	1:2	Cs_2CO_3	1,4-dioxane	130	74
7†	1:2	Cs_2CO_3	1,4-dioxane	130	95
8	1:2	Na_2CO_3	1,4-dioxane	130	trace
9	1:2	K_2CO_3	1,4-dioxane	130	44
10†	1:2	K_3PO_4	1,4-dioxane	130	93
11	1:2	KOH	1,4-dioxane	130	82
12	1:2	NaOH	1,4-dioxane	130	65
13	1:2	*t*-BuOK	1,4-dioxane	130	45
14	1:2	*t*-BuONa	1,4-dioxane	130	70
15	1:2	Cs_2CO_3	DMF	130	93
16	1:2	Cs_2CO_3	DMSO	130	80
17	1:2	Cs_2CO_3	acetonitrile	130	82
18	1:2	Cs_2CO_3	toluene	130	12
19	1:2	Cs_2CO_3	EtOH	130	N.D.
20	1:2	Cs_2CO_3	EA	130	25

Reaction conditions: 4-tolualdehyde (0.20 mmol), $Ph_2P(O)H$ (1-2 equiv.), base (1.0 equiv.), 1,4-dioxane, (1.0 mL), under N_2 for 12 h. GC yields using *n*-tridecane as internal standard. *Base (0.5 equiv.). †Base (2.0 equiv.).

An oven-dried Schlenk tube of 25 mL equipped with a magnetic stir bar was charged with base (0.2 mmol) and $Ph_2P(O)H$ (0.4 mmol), 4-tolualdehyde (0.20 mmol) and solvent (1.0 mL) were added. The reaction mixture was heated for 12 h under N_2. After completion of the reaction, the reaction mixture was cooled to room temperature. *n*-Tridecane (30 uL) as internal standard and dichloromethane (5 mL) were then added to the mixture, and GC yield of the reaction was calculated. The desired product **189** was isolated by column chromatography (eluent: ethyl acetate/petroleum ether = 1/1) over silica gel (300–400 mesh).

2.7 General Experimental Procedure of Carbonyl Reductive Phosphinylation Reaction.

Fig. S4: Substrate Scope of Carbonyl Reductive Phosphinylation Reaction.

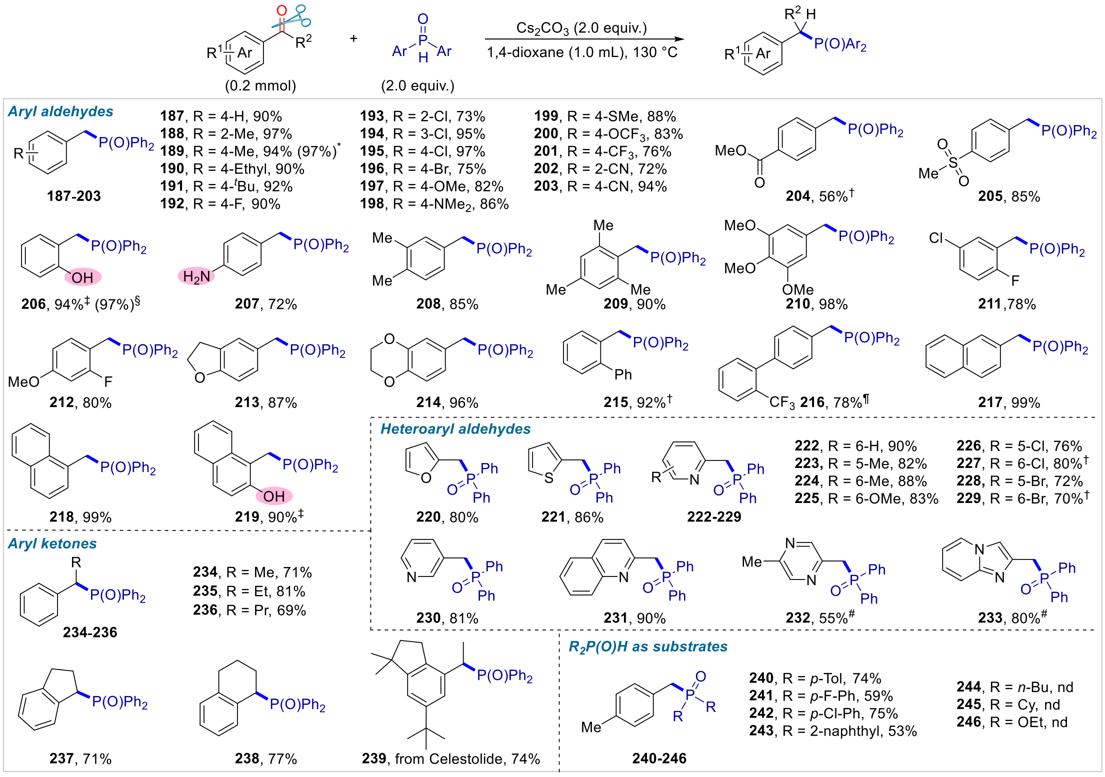

Reaction conditions: carbonyl compound (0.2 mmol), $Ph_2P(O)H$ (2.0 equiv.), Cs_2CO_3 (2.0 equiv.), 1,4-dioxane (1.0 mL), under N_2 at 130 °C for 12 h. Isolated yields are reported. *Yield of reaction is reported on a 6-mmol scale. †K_3PO_4 (2.0 equiv.) instead of Cs_2CO_3 as the base. ‡K_3PO_4 (2.0 equiv.) and DMSO (1.0 mL) as the base and solvent, respectively. §Yield of reaction is reported on a 5-mmol scale. ¶DMF (1.0 mL) instead of 1,4-dioxane as the solvent. #K_3PO_4 (2.0 equiv.) and DMF (1.0 mL) as the base and solvent, respectively.

Unless otherwise noted, all reactions are conducted in a glove box under a N_2 atmosphere. An

oven-dried Schlenk tube of 25 mL equipped with a magnetic stir bar was charged with Cs_2CO_3 (0.2 mmol) and $Ar_2P(O)H$ (0.4 mmol, 2.0 equiv.), carbonyl compound (0.2 mmol) and 1,4-dioxane (1.0 mL) were added. The reaction mixture was heated at 130 °C for 12 h under N_2. After completion of the reaction, the reaction mixture was cooled to room temperature and washed with saturated NH_4Cl aqueous solution (5.0 mL). The reaction mixture was then extracted with dichloromethane (3 × 5 mL). The organic layer was dried over anhydrous Na_2SO_4 and concentrated under vacuum. The desired products (**187** to **243**) were isolated by column chromatography (eluent: ethyl acetate/petroleum ether = 1/5–3/1) over silica gel (300–400 mesh).

2.8 General Experimental Procedure of Other Types of Carbonyl Transformations.

2.8.1: One-pot Reductive Arylamination, Alkylation, and Phosphinylation.

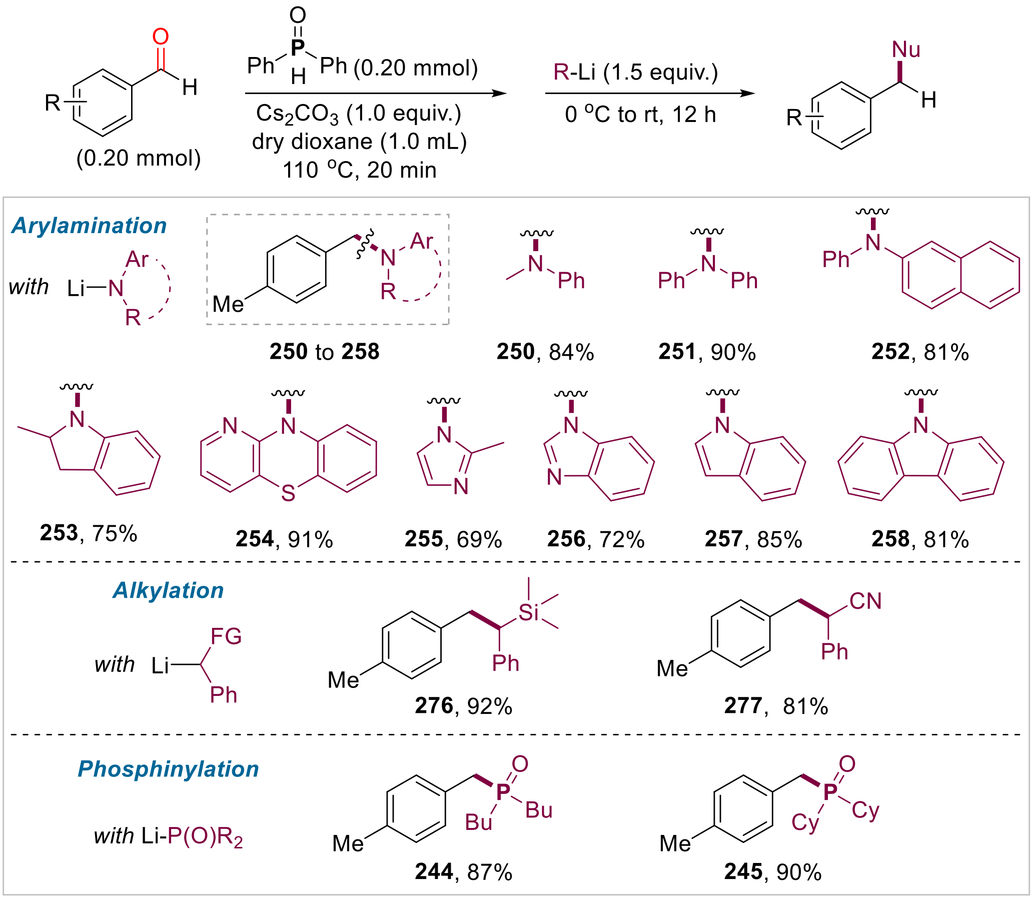

An oven-dried Schlenk tube of 25 mL equipped with a magnetic stir bar was charged with $Ph_2P(O)H$ (0.2 mmol) and Cs_2CO_3 (0.2 mmol), 4-tolualdehyde (0.2 mmol) and dry 1,4-dioxane (1.0 mL) were added. The mixture was heated at 110 °C under N_2 for 20 min. After cooling the mixture

to room temperature, the organolithium reagent (R-Li, 1M in THF, 0.3 mmol) was added under N_2. The mixture was allowed to continue to react at room temperature for 12 h. After completion of the reaction, the mixture was washed with saturated NH_4Cl aqueous solution (5.0 mL) and extracted with dichloromethane (3 × 5 mL). The organic layer was dried over anhydrous Na_2SO_4 and concentrated under vacuum. The desired products (**244**, **245**, **250** to **258**, **276**, and **277**) were isolated by column chromatography (eluent: ethyl acetate/petroleum ether = 1/40–1/1) over silica gel (300–400 mesh).

2.8.2: Direct Reductive Thioetherification of Aryl Carbonyls.

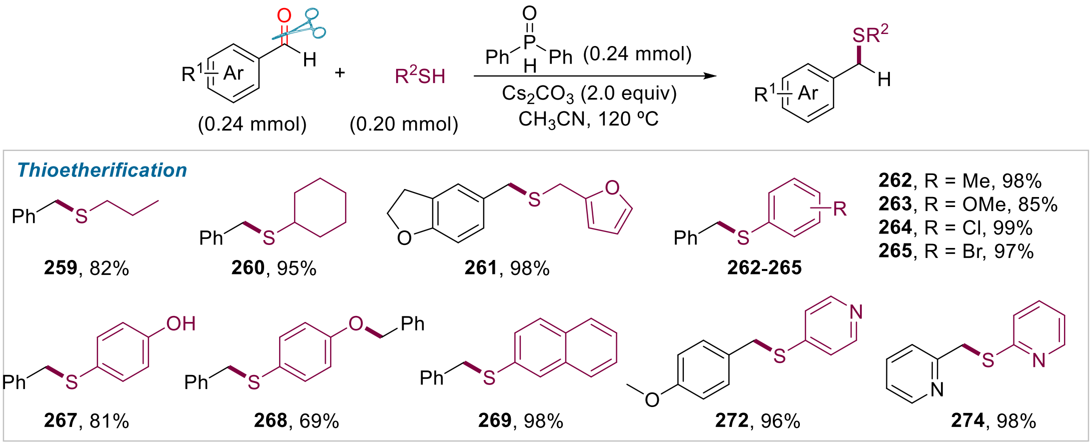

Unless otherwise noted, all reactions are conducted in a glove box under a N_2 atmosphere. An oven-dried Schlenk tube of 25 mL equipped with a magnetic stir bar was charged with Cs_2CO_3 (0.4 mmol, 2.0 equiv.) and $Ph_2P(O)H$ (0.24 mmol, 1.2 equiv.), *S*-nucleophile (0.20 mmol), carbonyl compound (0.24 mmol, 1.2 equiv.), and CH_3CN (1.0 mL) were added. The reaction mixture was heated at 120 °C for 12 h under N_2. After completion of the reaction, the reaction mixture was cooled to room temperature and washed with saturated NH_4Cl aqueous solution (5.0 mL). The reaction mixture was then extracted with dichloromethane (3 × 5 mL). The organic layer was dried over anhydrous Na_2SO_4 and was concentrated under vacuum. The desired products (**259** to **265**, **267** to **269**, **272**, and **274**) were isolated by column chromatography (eluent: ethyl acetate/petroleum ether = 1/20–1/10) over silica gel (300–400 mesh).

2.8.3: One-pot Reductive Thioetherification and Halogenation.

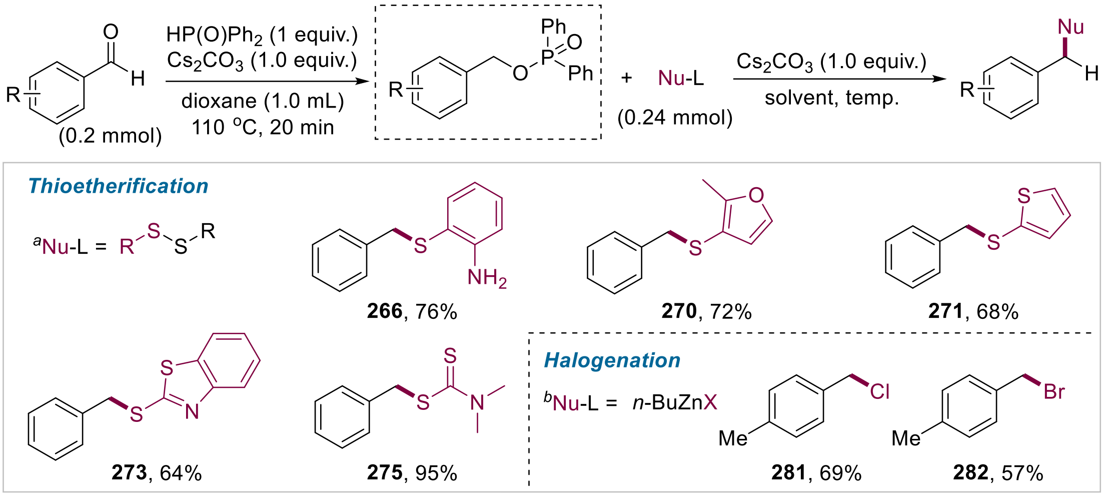

An oven-dried Schlenk tube of 25 mL equipped with a magnetic stir bar was charged with $Ph_2P(O)H$ (0.2 mmol) and Cs_2CO_3 (1 equiv.), aryl aldehyde (1 equiv.) and 1,4-dioxane (1.0 mL) were added. The mixture was heated at 110 °C under N_2 for 20 min. After cooling the mixture to room temperature, dichloromethane (5 mL) was added, and the supernatant was concentrated under vacuum. The resulting crude phosphinate intermediate was employed directly in the subsequent procedure. Following this, the crude phosphinate intermediate, Cs_2CO_3 (0.2 mmol), nucleophile (0.2 mmol), and solvent (1 mL) were added to an Schlenk tube and the reaction was continued for 12 h under N_2. Reaction conditions: (1) disulfide (0.2 mmol), Cs_2CO_3 (0.2 mmol), *N*-Methyl-2-pyrrolidone (NMP, 1 mL), at 100 °C for 12 h. (2) *n*-BuZnX (0.2 mmol), dry 1,4-dioxane (1mL), at 120 °C for 12 h. After completion of the reaction, the mixture was washed with saturated NH_4Cl aqueous solution (5.0 mL) and extracted with dichloromethane (3 × 5 mL). The organic layer was dried over anhydrous Na_2SO_4 and concentrated under vacuum. The desired products (**266**, **270**, **271**, **273**, **275**, **281**, and **282**) were isolated by column chromatography (eluent: ethyl acetate/petroleum ether = 1/20–1/10) over silica gel (300–400 mesh).

2.8.4: One-pot Reductive Bis-alkylation, Cyanation, and Sulfination.

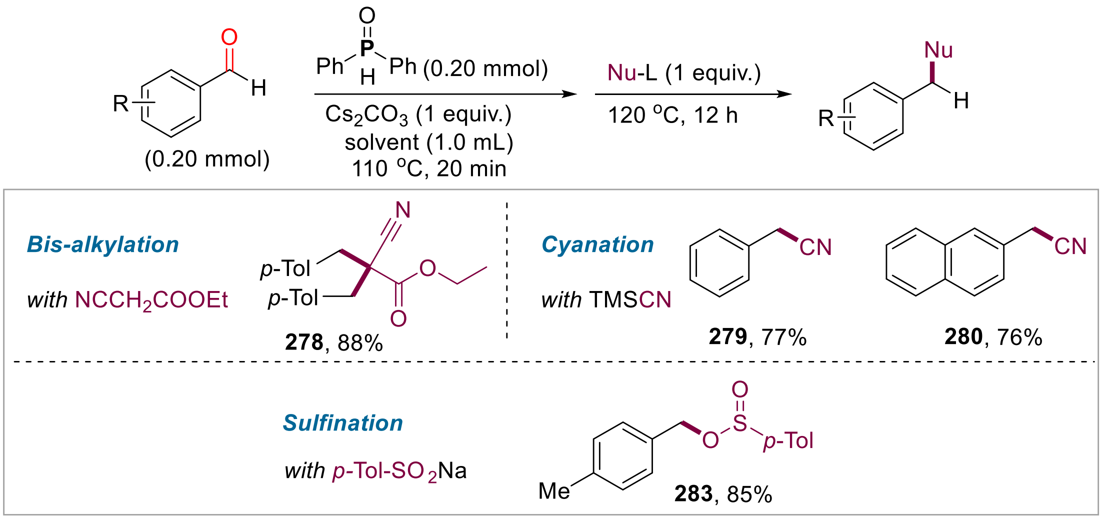

An oven-dried Schlenk tube of 25 mL equipped with a magnetic stir bar was charged with $Ph_2P(O)H$ (0.2 mmol) and Cs_2CO_3 (0.2 mmol), aryl aldehyde (0.2 mmol) and solvent (bis-alkylation and sulfination: DMF; cyanation: CH_3CN) (1.0 mL) were added. The mixture was heated at 110 °C under N_2 for 20 min. After cooling the mixture to room temperature, the nucleophile (0.2 mmol) was added under N_2. The mixture was allowed to continue to react at 120 °C for 12 h. After completion of the reaction, the mixture was washed with saturated NH_4Cl aqueous solution (5.0 mL) and extracted with dichloromethane (3 × 5 mL). The organic layer was dried over anhydrous Na_2SO_4 and concentrated under vacuum. The desired products (**278**, **279**, **280**, and **283**) were isolated by column chromatography (eluent: ethyl acetate/petroleum ether = 1/20–1/5) over silica gel (300–400 mesh).

2.8.5: One-pot Synthesis of Arylmethyl Diarylphosphine Oxides.

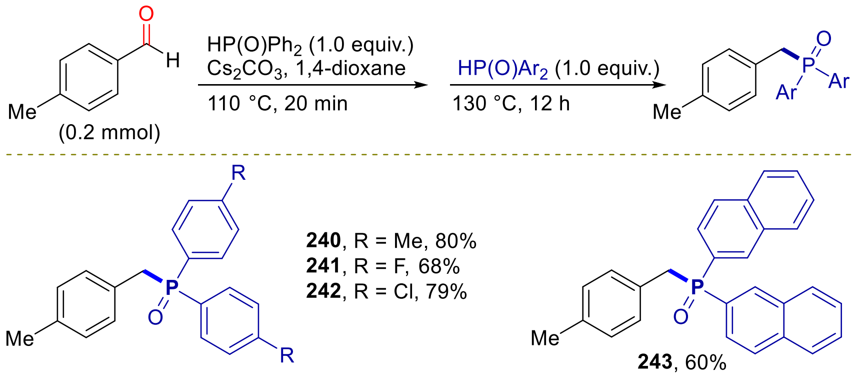

An oven-dried Schlenk tube of 25 mL equipped with a magnetic stir bar was charged with $Ph_2P(O)H$ (0.2 mmol) and Cs_2CO_3 (0.2 mmol), 4-tolualdehyde (0.2 mmol) and dry 1,4-dioxane (1.0 mL) were added. The mixture was heated at 110 °C under N_2 for 20 min. After cooling the mixture to room temperature, $Ar_2P(O)H$ (0.2 mmol, 1.0 equiv.) was added under N_2. The reaction mixture was allowed to continue at 130 °C for 12 h. After completion of the reaction, the reaction mixture was washed with saturated NH_4Cl aqueous solution (5.0 mL) and extracted with dichloromethane (3 × 5 mL). The organic layer was dried over anhydrous Na_2SO_4 and concentrated under vacuum. The desired products (**240** to **243**) were isolated by column chromatography (eluent: ethyl acetate/petroleum ether = 1/1–3/1) over silica gel (300–400 mesh) using petroleum ether-ethyl acetate as eluent.

2.8.6: One-pot Synthesis of Arylmethyl Diphenylphosphane.

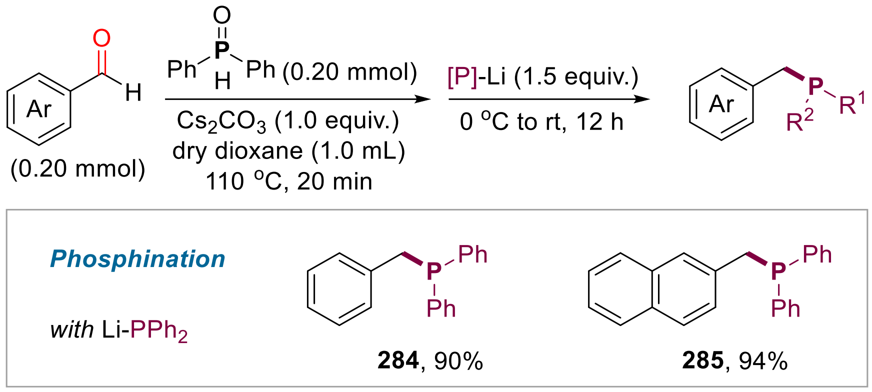

An oven-dried Schlenk tube of 25 mL equipped with a magnetic stir bar was charged with $Ph_2P(O)H$ (0.2 mmol) and Cs_2CO_3 (0.2 mmol), carbonyl compound (0.2 mmol) and dry 1,4-dioxane (1.0 mL) were added. The mixture was heated at 110 °C under N_2 for 20 min. After cooling the reaction mixture to 0 °C, the [P]-Li reagent (1M in THF, 0.2 mmol) was added under N_2. The reaction was allowed to proceed from 0 °C to room temperature for 12 h.

Purification procedure for compounds **284** and **285**: The entire process was conducted under N_2. After completion of the reaction, the degassed water (5 mL) and dichloromethane (5 mL) were added to the reaction mixture sequentially. The Schlenk tube was adequately shaken, and the upper aqueous solution was removed by a dropper. This process was repeated one more time. The organic layer was dried over anhydrous Na_2SO_4, filtered in a glove box, and ultimately concentrated under vacuum. Products **284** and **285** were obtained without additional purification.

2.9 Experimental Procedure of Gram-Scale Reactions.

Synthesis of (4-Methylbenzyl)diphenylphosphine Oxide (**189**).

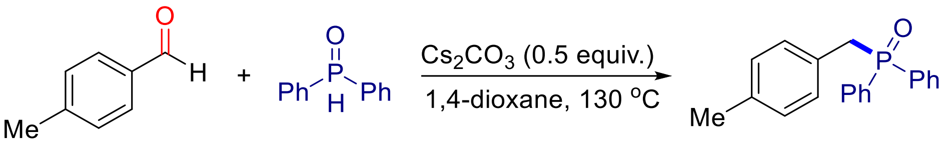

An oven-dried Schlenk tube of 100 mL equipped with a magnetic stir bar was charged with Cs_2CO_3 (3.0 mmol, 0.5 equiv.) and $Ph_2P(O)H$ (12.0 mmol, 2.0 equiv.), 4-tolualdehyde (6.0 mmol) and 1,4-dioxane (20 mL) were added. The reaction mixture was heated at 130 °C for 24 h. After completion of the reaction, the reaction mixture was cooled to room temperature and extracted with dichloromethane (3 × 25 mL). The organic layer was washed by water (3 × 25 mL), dried over anhydrous Na_2SO_4, and ultimately concentrated under vacuum. The white solid **189** was directly obtained without additional purification (97%, 1.78 g).

Fig. S5: Synthesis of (2-Hydroxylbenzyl)diphenylphosphine Oxide (**206**).

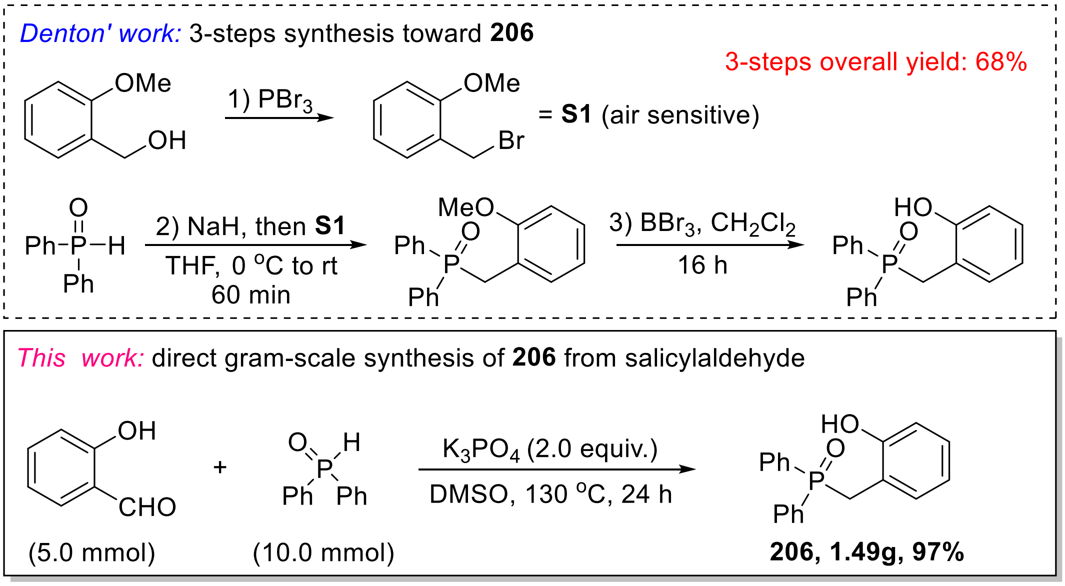

An oven-dried Schlenk tube of 100 mL equipped with a magnetic stir bar was charged with K_3PO_4 (10.0 mmol, 2.0 equiv.) and $Ph_2P(O)H$ (10.0 mmol, 2.0 equiv.), salicylaldehyde (5.0 mmol) and DMSO (15.0 mL) were added. The reaction mixture was heated at 130 °C for 24 h. After completion of the reaction, the reaction mixture was cooled to room temperature and quenched with 10% HCl aqueous solution (15.0 mL). The resulting mixture was then extracted with

dichloromethane (3 × 15 mL), and the organic layer was dried over anhydrous Na_2SO_4 and concentrated under vacuum. The residual DMSO was removed by vacuum distillation. The white solid **206** was directedly obtained (97%, 1.49 g) without additional purification.

Conclusion: The carbonyl reductive phosphinylation reaction is readily adapted to gram-scale experiments, which have proven to be more efficient than their 0.2 mmol counterparts. Additionally, these scale-up reactions offer great advantages in term of convenient operation and simplified purification.

3. Mechanism Investigation.

Fig. S6: Quenching Experiments of the Carbonyl Amination Reaction.

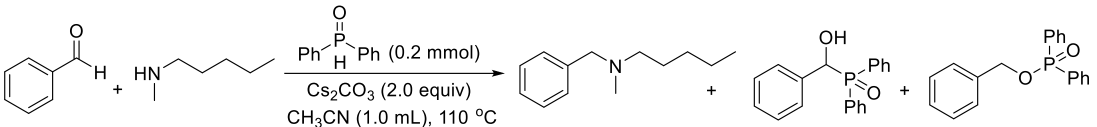

1a (0.2 mmol) **1b** (0.2 mmol) **1** **1c** **1B**

Reaction Time	yield of **1** (%)	yield of **1B** (%)	yield of **1c** (%)
10 min	2	90	0
20 min	5	91	0
40 min	17	76	0
1 h	24	67	0
2 h	34	55	0
5 h	63	23	0
12 h	81	3	0

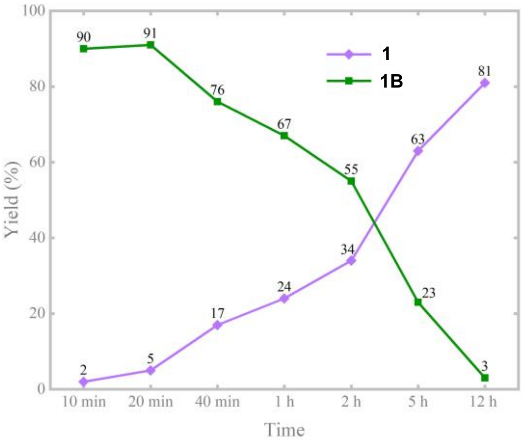

An oven-dried Schlenk tube of 25 mL equipped with a magnetic stir bar was charged with Cs_2CO_3 (0.4 mmol, 2.0 equiv.) and $Ph_2P(O)H$ (0.2 mmol), benzaldehyde (0.2 mmol), *N*-methylpentylamine (0.20 mmol), and CH_3CN (1.0 mL) were then added. The reaction mixtures were heated at 110 °C for varying durations of 10 min, 20 min, 40 min, 1 h. 2 h, 5 h, and 12 h under N_2, respectively. After completion of the reaction, the reaction mixtures were cooled to room temperature. *n*-Tridecane (30 μL) as internal standard and dichloromethane (5 mL) were then added, and GC yields of **1**, **1B**, and **1c** were calculated, respectively.

As demonstrated in Fig. S6, benzyl diphenylphosphinate (**1B**) was obtained in 91% yield after 20 min under standard conditions, owing to the highly rapid [1,2]-phospha–Book type arrangement process between benzaldehyde and diphenylphosphine oxide. As time progressed, the quantity of **1B** decreased, and yield of aminated product **1** gradually increased to 81% within 12 h. However, the potential Pudovik reaction adduct **1c** was never detected in any of these reactions.

Fig. S7: Control Experiments.

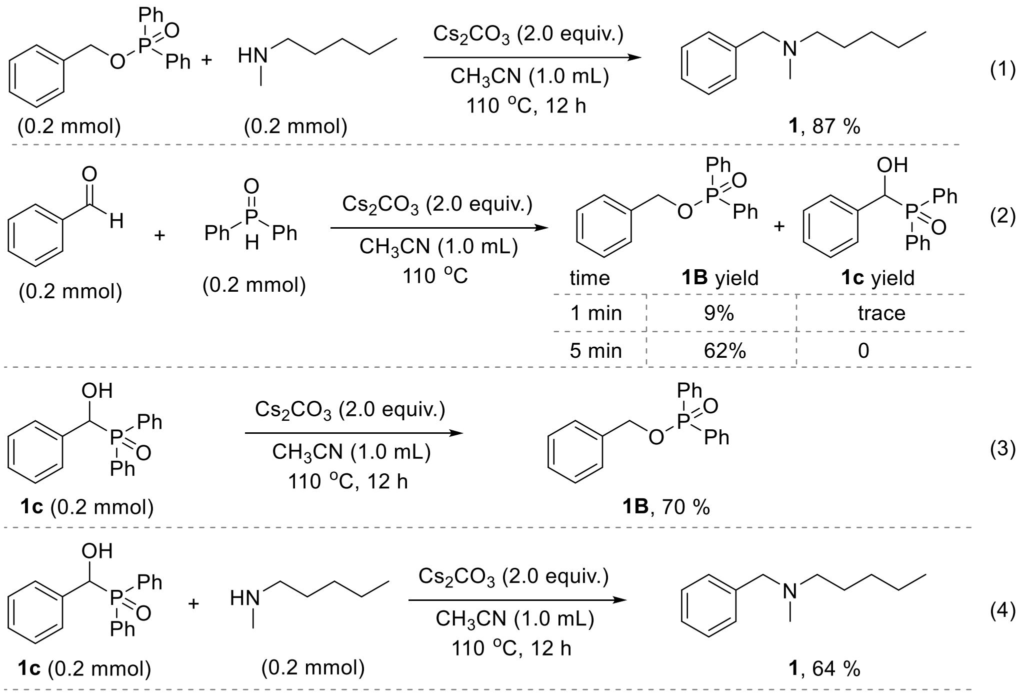

Benzyl diphenylphosphinate (**1B**) could be easily transformed into aminated product **1** with *N*-methylpentylamine under standard conditions (Fig. S7, eq 1). When benzaldehyde and diphenylphosphine oxide were reacted separately under standard conditions for 1 minute and 5 minutes, a rapid formation of benzyl diphenylphosphinate (**1B**) was observed, with the yield increasing from 9% to 62%. However, only trace amounts of compound **1c** were detected at the beginning of the reaction (Fig. S7, eq 2). Additionally, the pre-prepared Pudovik adduct **1c** was converted into **1B** with a yield of 70% under standard conditions (Fig. S7, eq 3). When 0.2 mmol *N*-methylpentylamine was added to the reaction, a 64% yield of aminated product **1** was obtained (Fig. S7, eq 4). These results indicate that **1c** could also be transformed into benzyl diphenylphosphinate (**1B**) under standard conditions.

Synthesis of ^{18}O-labelled Benzaldehyde

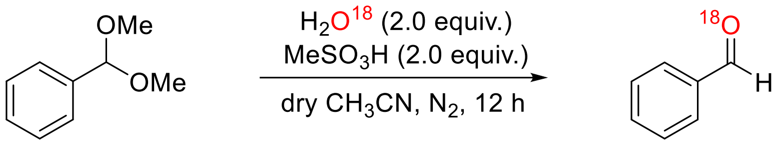

An oven-dried Schlenk tube of 50 mL equipped with a magnetic stir bar was charged with benzaldehyde dimethyl acetal (608 mg, 4 mmol) and dry CH_3CN (5 mL), H_2O^{18} (160 μL, 8 mmol) and $MeSO_3H$ (768 mg, 8 mmol) were then added under N_2 atmosphere. The reaction mixture was stirred overnight at room temperature for 12 h. After completion of the reaction, the solvent was removed, and the residue was purified by silica gel column chromatography to get the ^{18}O-labelled benzaldehyde, 397.5 mg (92%). GC-MS: M^+ m/z 108.05.

Fig. S8: Isotope Labeling Experiment.

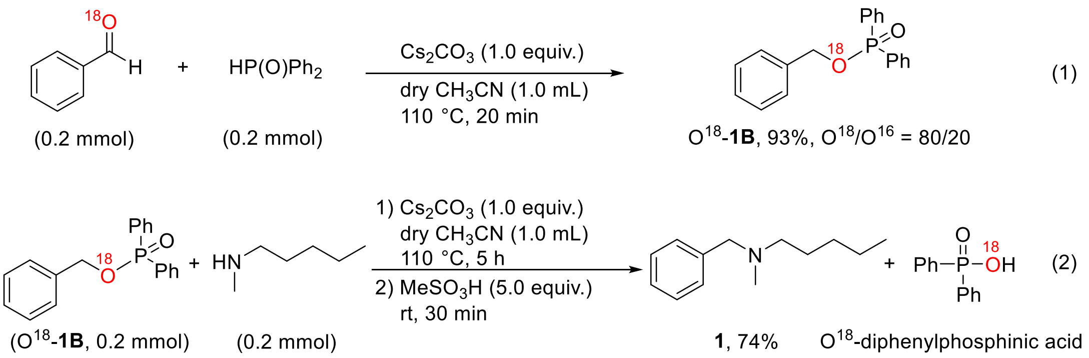

(1) An oven-dried Schlenk tube of 25 mL equipped with a magnetic stir bar was charged with Cs_2CO_3 (0.2 mmol), $Ph_2P(O)H$ (0.2 mmol), ^{18}O-labelled benzaldehyde (0.2 mmol) and dry CH_3CN (1.0 mL) were added. The reaction mixture was heated at 110 °C for 20 min under N_2. After completion of the reaction, the reaction mixture was cooled to room temperature. Dichloromethane (5 mL) was added and the organic layer was then concentrated under vacuum. The ^{18}O-labelled benzyl diphenylphosphinate (^{18}O-**1B**) was isolated by column chromatography (eluent: ethyl acetate/petroleum ether = 1/5) over silica gel (300–400 mesh).

(2) An oven-dried Schlenk tube of 25 mL equipped with a magnetic stir bar was charged with Cs_2CO_3 (0.2 mmol), *N*-methylpentylamine (0.20 mmol), ^{18}O-labelled benzyl diphenylphosphinate

(0.2 mmol) and dry CH_3CN (1.0 mL) were added. The reaction mixture was heated at 110 °C for 5 h under N_2. After completion of the reaction, the reaction mixture was cooled to room temperature. Afterward, methanesulfonic acid (5.0 equiv.) was added, and the reaction mixture was stirred at room temperature for 30 minutes. Following this, dry MeOH (1 mL) was added to the mixture, and the resulting solution was characterized using high-resolution mass spectrometry (HRMS).

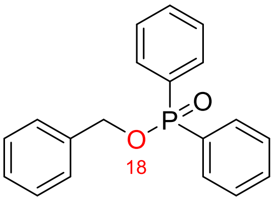

^{18}O-benzyl diphenylphosphinate: The title compound was purified by column chromatography on silica gel to afford a yellow oil in 93% yield (57.7 mg); ^{1}H NMR (400 MHz, $CDCl_3$) δ 7.92 – 7.69 (m, 4H), 7.49 (dd, J = 10.5, 4.2 Hz, 2H), 7.42 (td, J = 7.3, 3.5 Hz, 4H), 7.38 – 7.26 (m, 5H), 5.06 (d, J = 6.8 Hz, 2H). ^{13}C NMR (101 MHz, $CDCl_3$) δ 136.21 (d, J = 7.5 Hz), 132.08 (d, J = 2.8 Hz), 131.55 (d, J = 10.2 Hz), 131.21 (d, J = 136.6 Hz), 128.48, 128.37 (d, J = 4.9 Hz), 128.13, 127.70, 66.14 (d, J = 5.5 Hz). ^{31}P NMR (162 MHz, $CDCl_3$) δ 32.32. HRMS (ESI): [M+H$^+$] calcd for $C_{19}H_{18}O^{18}OP^+$: 311.1081; found, 311.1070.

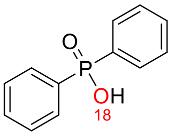

^{18}O-diphenylphosphinic acid: HRMS was used to verify the generation of ^{18}O-diphenylphosphinic acid. HRMS (ESI): [M+H$^+$] calcd for $C_{12}H_{12}O^{18}OP^+$: 221.0612; found, 221.0603.

Fig. S9: HRMS Spectrum of the Reaction Mixture (Fig. S4, eq 2)

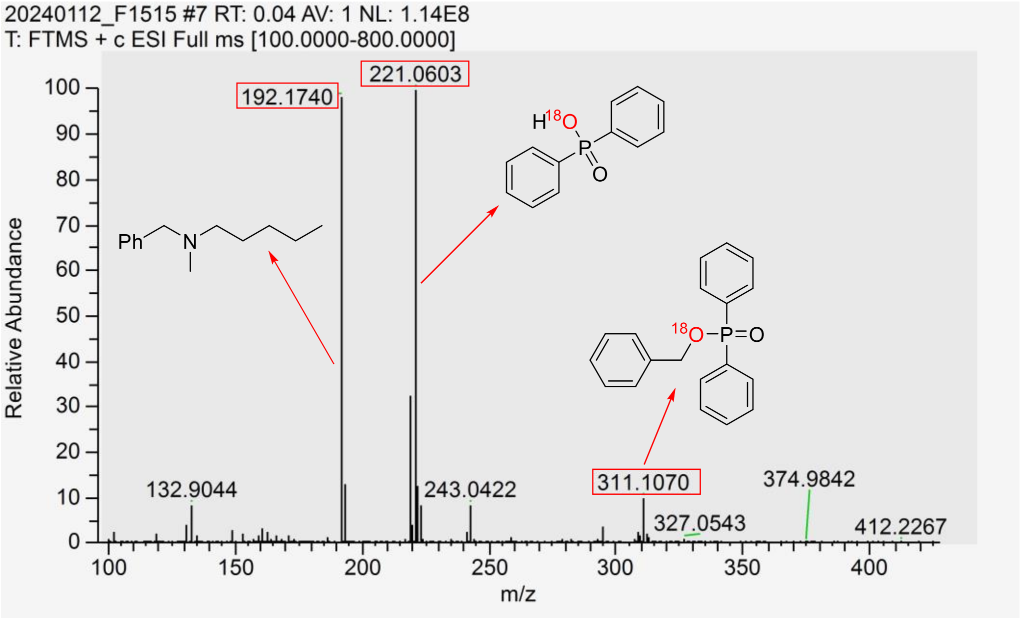

Figs. S10: HRMS Spectrum of ^{18}O-diphenylphosphinic Acid

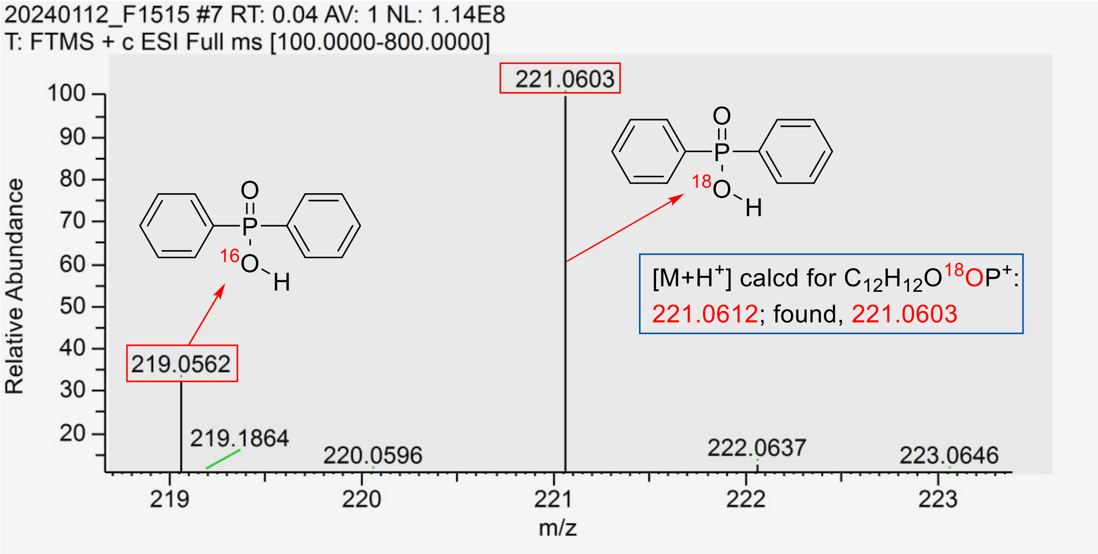

Figs. S11: HRMS Spectrum of ^{18}O-benzyl Diphenylphosphinate

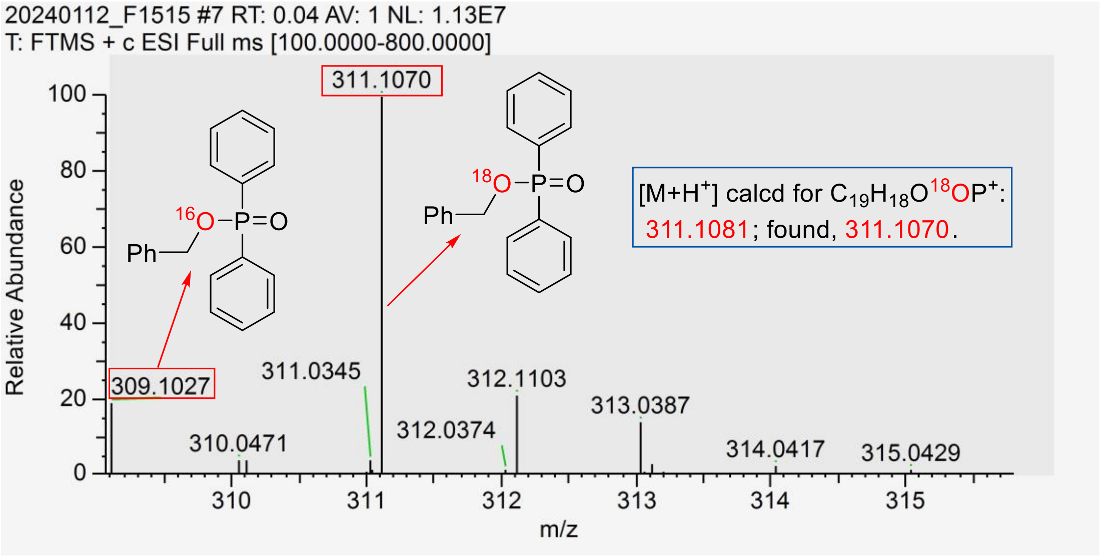

Conclusion: The aforementioned results revealed that the oxygen atom of carbonyls was integrated into diphenylphosphinic acid.

Synthesis of intermediate (*R*)-2B

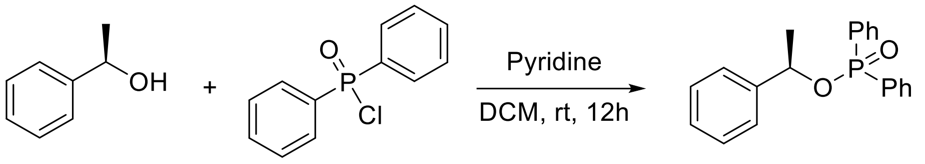

(*R*)-**2B**, 70%

A dichloromethane (10 ml) solution of diphenylphosphonic acid chloride (1.95 g, 8.2 mol) was added dropwise to a solution of (*R*)-1-phenylethanol (1 g, 8.2 mmol, >99% ee) in pyridine (1.4 mL, 16.4 mmol) and dichloromethane (5 mL). The mixture was stirred at ambient temperature for 12 hours, and then the dichloromethane was removed on the rotavapor. To the residue was added hydrochloric acid (5 ml 4N solution), and the product was extracted with ether. The solvent was removed under vacuum and the residue was purified by silica gel column chromatography with hexane/ethyl acetate (2:1) as eluent to give the phosphonate product (*R*)-**2B** (1.8 g, 70%) as a white solid.

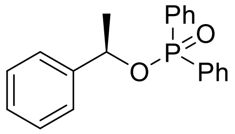

(*R*)-1-phenylethyl diphenylphosphinate ((*R*)-**2B**): ^{1}H NMR (400 MHz, $CDCl_3$) δ 7.90 – 7.80 (m, 2H), 7.70 – 7.61 (m, 2H), 7.56 – 7.49 (m, 1H), 7.49 – 7.40 (m, 3H), 7.36 – 7.26 (m, 7H), 5.56 – 5.47 (m, 1H), 1.67 (d, *J* = 6.5 Hz, 3H). The data is identical to this reported in the literature. (*50*)

The ee-determination of (*R*)-**2B** gave an ee >99%. HPLC condition: Chiralpak AD, n-hexane/isopropanol = 9:1, 0.5 mL/min, 220 nm UV detector, t_R = 20.2 min (major) and t_R = 22.6 min (minor).

Figs. S12: Mechanistic Investigation of Nucleophilic Substitution Procedure

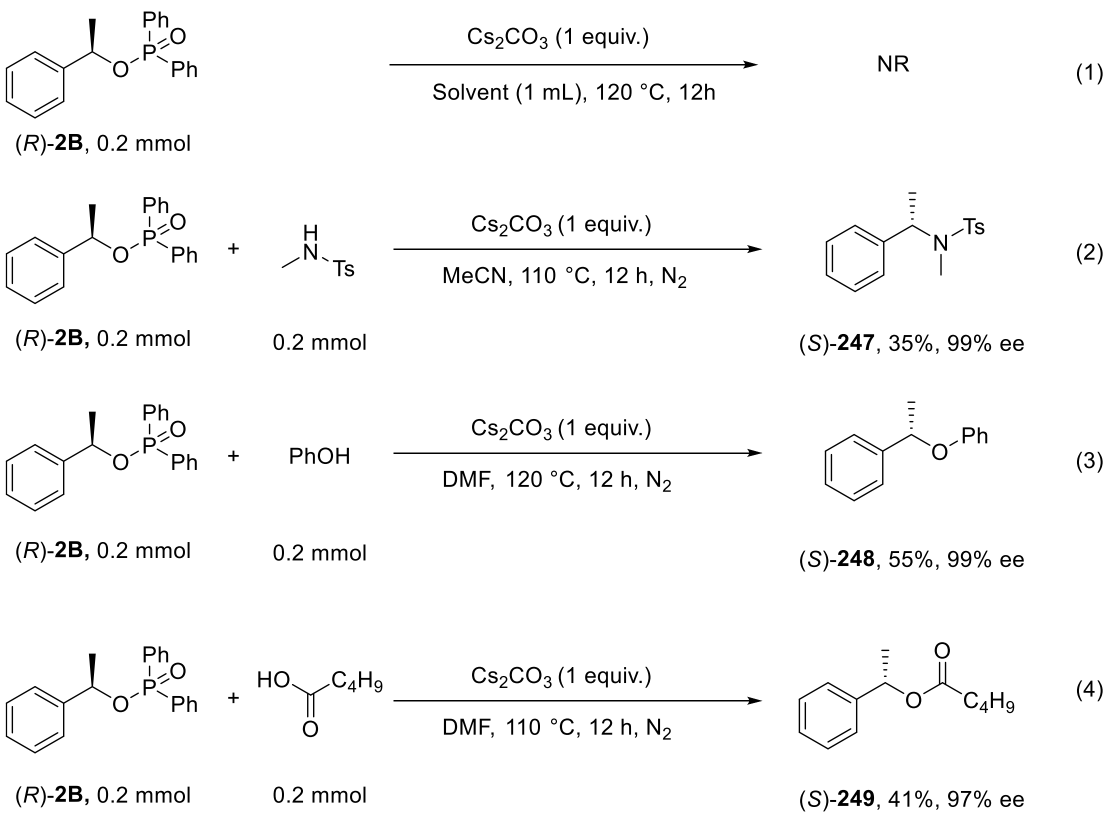

(1) A mixture of (*R*)-**2B** (64.4 mg, 0.2 mmol) and Cs_2CO_3 (65 mg, 0.2 mmol) in dry DMF or MeCN (1 mL) was stirred at 120°C under a nitrogen (N_2) atmosphere for 12 hours. Subsequently, the reaction mixture was filtered through a short celite pad and washed with ethyl acetate (10 mL × 3). The solvent was then removed under vacuum. Further analysis revealed that (*R*)-**2B** remained unaltered, with an ee value >99%.

(2) An oven-dried Schlenk tube of 25 mL equipped with a magnetic stir bar was charged with Cs_2CO_3 (0.2 mmol), *N*,4-dimethylbenzenesulfonamide (0.2 mmol), (*R*)-**2B** (0.2 mmol) and dry MeCN (1.0 mL) were added. The reaction mixture was heated at 110 °C for 12 h under N_2. After completion of the reaction, the reaction mixture was cooled to room temperature and washed with saturated NH_4Cl aqueous solution (5.0 mL). The reaction mixture was then extracted with dichloromethane (3 × 5 mL). The organic layer was dried over anhydrous Na_2SO_4 and was concentrated under vacuum. The desired products ((*S*)-**247**) were isolated by column chromatography (eluent: ethyl acetate/petroleum ether = 1/4) over silica gel (300–400 mesh) in 35% yield (20.3 mg) as a white solid.

(3) An oven-dried Schlenk tube of 25 mL equipped with a magnetic stir bar was charged with Cs_2CO_3 (0.2 mmol), phenol (0.2 mmol), (*R*)-**2B** (0.2 mmol) and dry DMF (1.0 mL) were added. The reaction mixture was heated at 120 °C for 12 h under N_2. After completion of the reaction, the reaction mixture was cooled to room temperature and washed with saturated NH_4Cl aqueous solution (5.0 mL). The reaction mixture was then extracted with dichloromethane (3 × 5 mL). The organic layer was dried over anhydrous Na_2SO_4 and was concentrated under vacuum. The desired products ((*S*)-**248**) were isolated by column chromatography (eluent: ethyl acetate/petroleum ether = 1/10) over silica gel (300–400 mesh) in 55% yield (21.8 mg) as a colorless oil.

(4) An oven-dried Schlenk tube of 25 mL equipped with a magnetic stir bar was charged with Cs_2CO_3 (0.2 mmol), valeric acid (0.2 mmol), (*R*)-**2B** (0.2 mmol) and dry DMF (1.0 mL) were added. The reaction mixture was heated at 110 °C for 12 h under N_2. After completion of the reaction, the reaction mixture was cooled to room temperature and washed with saturated NH_4Cl aqueous solution (5.0 mL). The reaction mixture was then extracted with dichloromethane (3 × 5 mL). The organic layer was dried over anhydrous Na_2SO_4 and was concentrated under vacuum. The desired products ((*S*)-**249**) were isolated by column chromatography (eluent: ethyl acetate/petroleum ether = 1/5) over silica gel (300–400 mesh) in 41% yield (16.9 mg) as a colorless oil.

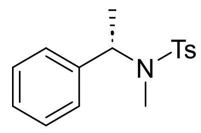

(*S*)-*N*,4-dimethyl-*N*-(1-phenylethyl)benzenesulfonamide (247): White solid. mp: 69-71 ºC. 99% ee, $[\alpha]_D^{26}$= -27.3 (*c* = 1.00, CH_2Cl_2). HPLC condition: Chiralpak IB (0.46 x 25 cm, 5 μm), hexane/isopropanol = 95:5, 1.0 mL/min, 230 nm UV detector, t_R = 8.13 min (major) and t_R = 9.24 min (minor);); ^1H NMR (400 MHz, $CDCl_3$) δ 7.74 (d, *J* = 6.5 Hz, 2H), 7.34 – 7.28 (m, 6H), 7.28 – 7.23 (m, 1H), 5.29 (q, *J* = 7.0 Hz, 1H), 2.57 (s, 3H), 2.44 (s, 3H), 1.29 (d, *J* = 7.0 Hz, 3H). ^{13}C NMR (126 MHz, $CDCl_3$) δ 143.2, 140.1, 137.4, 129.8, 128.5, 127.7, 127.4, 127.3, 54.9, 28.5, 21.7, 15.3. HRMS (APCI) m/z: [M+Na^+] calcd for $C_{16}H_{19}O_2NSNa$: 312.1029; found, 312.1028.

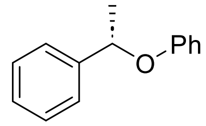

(*S*)-(1-phenoxyethyl)benzene (248): Colorless oil. >99% ee, $[\alpha]_D^{30}$= -39.5 (*c* = 1.00, CH_2Cl_2). HPLC condition: Chiralpak OD-H, hexane/isopropanol = 99.5:0.5, 0.7 mL/min, 230 nm UV detector, t_R = 6.03 min (major) and t_R = 6.67 min (minor); ^1H NMR (500 MHz, $CDCl_3$) δ 7.41 – 7.37 (m, 2H), 7.36 – 7.31 (m, 2H), 7.28 – 7.23 (m, 1H), 7.23 –

7.17 (m, 2H), 6.91 – 6.83 (m, 3H), 5.32 (q, *J* = 6.5 Hz, 1H), 1.64 (d, *J* = 6.5 Hz, 3H). ^{13}C NMR (126 MHz, $CDCl_3$) δ 158.1, 143.4, 129.4, 128.7, 127.5, 125.7, 120.8, 116.1, 76.0, 24.6. HRMS (APCI) m/z: [M+H$^+$] calcd for $C_{14}H_{13}O^+$: 197.0972; found, 197.0962.

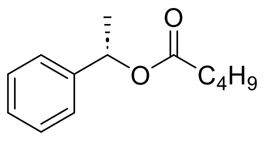

(*S*)-1-phenylethyl pentanoate (249): Colorless oil. 97% ee, $[\alpha]_D^{26}$= +25.4 (*c* = 1.00, CH_2Cl_2). HPLC condition: Chiralpak OJ-H, hexane/isopropanol = 95:5, 1.0 mL/min, 254 nm UV detector, t_R = 4.88 min (minor) and t_R = 5.60 min (major); ^{1}H NMR (400 MHz, $CDCl_3$) δ 7.38 – 7.33 (m, 4H), 7.33 – 7.27 (m, 1H), 5.90 (q, *J* = 6.6 Hz, 1H), 2.33 (td, *J* = 7.4, 1.1 Hz, 2H), 1.68 – 1.57 (m, 2H), 1.53 (d, *J* = 6.6 Hz, 3H), 1.40 – 1.27 (m, 2H), 0.91 (t, *J* = 7.3 Hz, 3H). ^{13}C NMR (126 MHz, $CDCl_3$) δ 173.2, 142.0, 128.6, 127.9, 126.2, 72.1, 34.5, 27.2, 22.4, 22.4, 13.9. HRMS (APCI) m/z: [M+Na$^+$] calcd for $C_{13}H_{18}O_2Na^+$: 229.1199; found, 229.1199.

4. Characterization Data of Products.

4.1 Characterization Data of Tertiary Amines

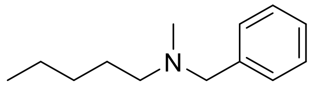

***N*-benzyl-*N*-methylpentan-1-amine (1):** The title compound was prepared according to the general procedure and purified by column chromatography on silica gel to afford a yellow oil in 92% yield (35.2 mg); ^{1}H NMR (400 MHz, $CDCl_3$) δ 7.30 (d, *J* = 4.4 Hz, 4H), 7.25 – 7.20 (m, 1H), 3.47 (s, 2H), 2.46 – 2.22 (m, 2H), 2.17 (s, 3H), 1.57 – 1.43 (m, 2H), 1.34 – 1.22 (m, 4H), 0.88 (t, *J* = 6.7 Hz, 3H). ^{13}C NMR (101 MHz, $CDCl_3$) δ 139.2, 129.0, 128.1, 126.8, 62.3, 57.6, 42.2, 29.7, 27.1, 22.6, 14.1. (*51*)

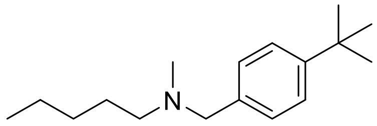

***N*-(4-(tert-butyl)benzyl)-*N*-methylpentan-1-amine (2):** The title compound was prepared according to the general procedure and purified by column chromatography on silica gel to afford a yellow oil in 95% yield (47.0 mg); ^{1}H NMR (400 MHz, $CDCl_3$) δ 7.33 (d, *J* = 8.2 Hz, 2H), 7.23 (d, *J* = 8.2 Hz, 2H), 3.45 (s, 2H), 2.41 – 2.30 (m, 2H), 2.18 (s, 3H), 1.56 – 1.46 (m, 2H), 1.31 (s, 9H), 1.30 – 1.24 (m, 4H), 0.89 (t, *J* = 6.7 Hz, 3H). ^{13}C NMR (101 MHz, $CDCl_3$) δ 149.7, 136.1, 128.7, 125.0, 61.9, 57.6, 42.2, 34.4, 31.4, 29.7, 27.1, 22.7, 14.1. HRMS (EI) m/z: [M]$^+$ calcd for

$C_{17}H_{29}N$: 247.2300; found, 247.2299.

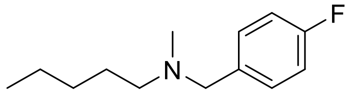

***N*-(4-fluorobenzyl)-*N*-methylpentan-1-amine (3):** The title compound was prepared according to the general procedure and purified by column chromatography on silica gel to afford a yellow oil in 82% yield (34.3 mg); ^{1}H NMR (400 MHz, $CDCl_3$) δ 7.29 – 7.22 (m, 2H), 6.98 (t, *J* = 8.6 Hz, 2H), 3.43 (s, 2H), 2.47 – 2.28 (m, 2H), 2.16 (s, 3H), 1.55 – 1.45 (m, 2H), 1.36 – 1.25 (m, 4H), 0.89 (t, *J* = 6.8 Hz, 3H). ^{13}C NMR (101 MHz, $CDCl_3$) δ 161.9 (d, *J* = 244.3 Hz), 135.0 (d, *J* = 3.2 Hz), 130.4 (d, *J* = 7.9 Hz), 114.9 (d, *J* = 21.1 Hz), 61.5, 57.5, 42.1, 29.6, 27.1, 22.6, 14.1. ^{19}F NMR (376 MHz, $CDCl_3$) δ -116.37. HRMS (EI) m/z: $[M]^+$ calcd for $C_{13}H_{20}FN$: 209.1580; found, 209.1581.

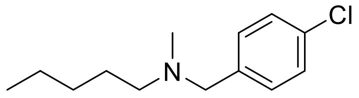

***N*-(4-chlorobenzyl)-*N*-methylpentan-1-amine (4):** The title compound was prepared according to the general procedure and purified by column chromatography on silica gel to afford a yellow oil in 73% yield (32.9 mg); ^{1}H NMR (400 MHz, $CDCl_3$) δ 7.29 (dd, *J* = 7.5, 6.4 Hz, 4H), 3.46 (s, 2H), 2.45 – 2.27 (m, 2H), 2.19 (s, 3H), 1.58 – 1.48 (m, 2H), 1.38 – 1.29 (m, 4H), 0.92 (t, *J* = 6.6 Hz, 3H). ^{13}C NMR (101 MHz, $CDCl_3$) δ 137.9, 132.5, 130.3, 128.3, 61.6, 57.5, 42.2, 29.6, 27.1, 22.6, 14.1. HRMS (EI) m/z: $[M]^+$ calcd for $C_{13}H_{20}ClN$: 225.1284; found, 225.1285.

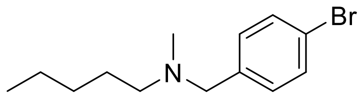

***N*-(4-bromobenzyl)-*N*-methylpentan-1-amine (5):** The title compound was prepared according to the general procedure and purified by column chromatography on silica gel to afford a brown oil in 86% yield (46.3 mg); ^{1}H NMR (400 MHz, $CDCl_3$) δ 7.42 (d, *J* = 8.3 Hz, 2H), 7.18 (d, *J* = 8.2 Hz, 2H), 3.41 (s, 2H), 2.39 – 2.27 (m, 2H), 2.15 (s, 3H), 1.49 (dt, *J* = 14.7, 7.4 Hz, 2H), 1.35 – 1.21 (m, 4H), 0.88 (t, *J* = 6.9 Hz, 3H). ^{13}C NMR (101 MHz, $CDCl_3$) δ 138.4, 131.2, 130.6, 120.6, 61.6, 57.5, 42.2, 29.6, 27.0, 22.6, 14.1. HRMS (EI) m/z: $[M]^+$ calcd for $C_{13}H_{20}BrN$: 269.0779; found, 269.0776.

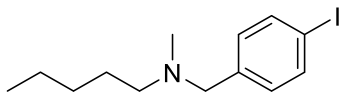

***N*-(4-iodobenzyl)-*N*-methylpentan-1-amine (6):** The title compound was prepared according to the general procedure and purified by column chromatography on silica gel to afford a brown oil in 82% yield (52.0 mg); ^{1}H NMR (400 MHz, $CDCl_3$) δ 7.62 (d, *J* = 8.2 Hz, 2H), 7.06 (d, *J* = 8.1 Hz, 2H), 3.40 (s, 2H), 2.39 – 2.27 (m, 2H), 2.15 (s, 3H), 1.49 (dt, *J* = 14.7, 7.4 Hz, 2H), 1.35 – 1.22 (m, 4H), 0.89 (t, *J* = 6.9 Hz,

3H). ^{13}C NMR (101 MHz, $CDCl_3$) δ 139.1, 137.2, 130.9, 92.1, 61.7, 57.5, 42.2, 29.6, 27.0, 22.6, 14.1. HRMS (EI) m/z: $[M]^+$ calcd for $C_{13}H_{20}IN$: 317.0640; found, 317.0636.

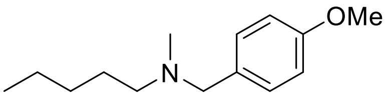

***N*-(4-methoxybenzyl)-*N*-methylpentan-1-amine (7):** The title compound was prepared according to the general procedure and purified by column chromatography on silica gel to afford a yellow oil in 88% yield (38.9 mg); ^{1}H NMR (400 MHz, $CDCl_3$) δ 7.21 (d, J = 8.4 Hz, 2H), 6.84 (d, J = 8.5 Hz, 2H), 3.79 (s, 3H), 3.41 (s, 2H), 2.45 – 2.24 (m, 2H), 2.16 (s, 3H), 1.55 – 1.46 (m, 2H), 1.34 – 1.26 (m, 4H), 0.89 (t, J = 6.8 Hz, 3H). ^{13}C NMR (101 MHz, $CDCl_3$) δ 158.6, 131.3, 130.2, 113.5, 61.6, 57.4, 55.2, 42.1, 29.7, 27.1, 22.6, 14.1. HRMS (EI) m/z: $[M]^+$ calcd for $C_{14}H_{23}NO$: 221.1780; found, 221.1781.

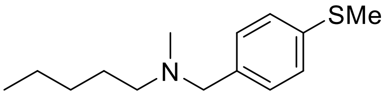

***N*-methyl-*N*-(4-(methylthio)benzyl)pentan-1-amine (8):** The title compound was prepared according to the general procedure and purified by column chromatography on silica gel to afford a yellow oil in 80% yield (37.9 mg); ^{1}H NMR (400 MHz, $CDCl_3$) δ 7.24 – 7.18 (m, 4H), 3.43 (s, 2H), 2.47 (s, 3H), 2.38 – 2.26 (m, 2H), 2.16 (s, 3H), 1.50 (dt, J = 14.6, 7.4 Hz, 2H), 1.35 – 1.25 (m, 4H), 0.89 (t, J = 6.9 Hz, 3H). ^{13}C NMR (101 MHz, $CDCl_3$) δ 136.5, 136.4, 129.6, 126.7, 61.83, 57.5, 42.2, 29.7, 27.1, 22.6, 16.1, 14.1. HRMS (EI) m/z: $[M]^+$ calcd for $C_{14}H_{23}NS$: 237.1551; found, 237.1549.

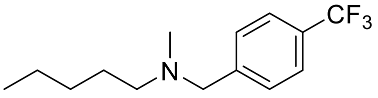

***N*-methyl-*N*-(4-(trifluoromethyl)benzyl)pentan-1-amine (9):** The title compound was prepared according to the general procedure and purified by column chromatography on silica gel to afford a yellow oil in 79% yield (40.9 mg); ^{1}H NMR (600 MHz, $CDCl_3$) δ 7.56 (d, J = 8.0 Hz, 2H), 7.43 (d, J = 7.9 Hz, 2H), 3.52 (s, 2H), 2.38 – 2.33 (m, 2H), 2.18 (s, 3H), 1.58 – 1.44 (m, 2H), 1.35 – 1.26 (m, 4H), 0.89 (t, J = 6.8 Hz, 3H). ^{13}C NMR (151 MHz, $CDCl_3$) δ 143.6, 129.1 (d, J = 31.7 Hz)), 129.1, 125.1 (q, J = 3.7 Hz), 123.4, 61.8, 57.6, 42.2, 29.6, 27.0, 22.6, 14.1. ^{19}F NMR (565 MHz, $CDCl_3$) δ -62.34. HRMS (EI) m/z: $[M]^+$ calcd for $C_{14}H_{20}F_3N$: 259.1548; found, 259.1546.

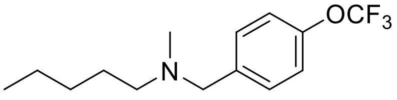

***N*-methyl-*N*-(4-(trifluoromethoxy)benzyl)pentan-1-amine (10):** The title compound was prepared according to the general procedure and purified by column chromatography on silica gel to afford a yellow oil in 82% yield (45.1 mg); ^{1}H NMR (400 MHz, $CDCl_3$) δ 7.33 (d, J = 8.4 Hz, 2H), 7.15 (d, J = 8.2 Hz, 2H),

3.46 (s, 2H), 2.46 – 2.27 (m, 2H), 2.17 (s, 3H), 1.56 – 1.42 (m, 2H), 1.31 (t, J = 8.5 Hz, 4H), 0.89 (t, J = 6.7 Hz, 3H). ^{13}C NMR (101 MHz, $CDCl_3$) δ 148.12 (d, J = 1.6 Hz), 138.3, 130.1, 120.7, 120.5 (q, J = 257.6 Hz), 61.6, 57.6, 42.2, 29.6, 27.1, 22.6, 14.0. ^{19}F NMR (376 MHz, $CDCl_3$) δ -57.90. HRMS (EI) m/z: $[M]^+$ calcd for $C_{14}H_{20}F_3NO$: 275.1497; found, 275.1499.

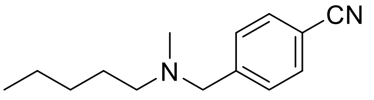

4-((methyl(pentyl)amino)methyl)benzonitrile (11): The title compound was prepared according to the general procedure and purified by column chromatography on silica gel to afford a brown oil in 76% yield (32.9 mg); ^{1}H NMR (400 MHz, $CDCl_3$) δ 7.59 (d, J = 8.1 Hz, 2H), 7.43 (d, J = 8.1 Hz, 2H), 3.51 (s, 2H), 2.44 – 2.29 (m, 2H), 2.17 (s, 3H), 1.56 – 1.43 (m, 2H), 1.37 – 1.24 (m, 4H), 0.88 (t, J = 6.8 Hz, 3H). ^{13}C NMR (101 MHz, $CDCl_3$) δ 145.3, 132.0, 129.4, 119.0, 110.6, 61.9, 57.6, 42.2, 29.5, 27.0, 22.6, 14.0. HRMS (EI) m/z: $[M]^+$ calcd for $C_{14}H_{20}N_2$: 216.1626; found, 216.1627.

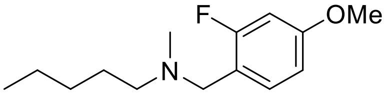

***N*-(2-fluoro-4-methoxybenzyl)-*N*-methylpentan-1-amine (12):** The title compound was prepared according to the general procedure and purified by column chromatography on silica gel to afford a yellow oil in 84% yield (40.2 mg); ^{1}H NMR (400 MHz, $CDCl_3$) δ 7.23 (t, J = 8.5 Hz, 1H), 6.65 (dd, J = 8.4, 2.3 Hz, 1H), 6.58 (dd, J = 11.7, 2.4 Hz, 1H), 3.78 (s, 3H), 3.47 (s, 2H), 2.42 – 2.26 (m, 2H), 2.18 (s, 3H), 1.51 (dt, J = 14.6, 7.4 Hz, 2H), 1.40 – 1.20 (m, 4H), 0.89 (t, J = 6.9 Hz, 3H). ^{13}C NMR (101 MHz, $CDCl_3$) δ 161.9 (d, J = 245.7 Hz), 159.9 (d, J = 11.0 Hz), 132.0 (d, J = 6.6 Hz), 117.5 (d, J = 15.3 Hz), 109.6 (d, J = 3.1 Hz), 101.3 (d, J = 26.2 Hz), 57.4, 55.5, 54.1, 42.0, 29.7, 27.1, 22.6, 14.1. ^{19}F NMR (376 MHz, $CDCl_3$) δ -115.94. HRMS (EI) m/z: $[M]^+$ calcd for $C_{14}H_{22}FNO$: 239.1685; found, 239.1685.

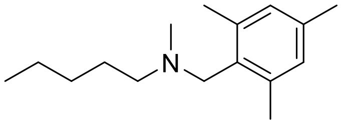

***N*-methyl-*N*-(2,4,6-trimethylbenzyl)pentan-1-amine (13):** The title compound was prepared according to the general procedure and purified by column chromatography on silica gel to afford a yellow oil in 87% yield (40.6 mg); ^{1}H NMR (600 MHz, $CDCl_3$) δ 6.83 (s, 2H), 3.41 (s, 2H), 2.36 (d, J = 9.7 Hz, 8H), 2.26 (s, 3H), 2.11 (s, 3H), 1.53 – 1.44 (m, 2H), 1.33 – 1.23 (m, 4H), 0.88 (t, J = 6.7 Hz, 3H). ^{13}C NMR (151 MHz, $CDCl_3$) δ 137.9, 136.1, 132.7, 128.8, 57.8, 55.7, 41.2, 29.6, 27.1, 22.6, 20.9, 20.1, 14.1. HRMS (EI) m/z: $[M]^+$ calcd for $C_{16}H_{27}N$: 233.2143; found, 233.2142.

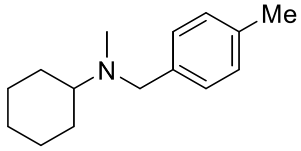

***N*-methyl-*N*-(4-methylbenzyl)cyclohexanamine (14):** The title compound was prepared according to the general procedure and purified by column chromatography on silica gel to afford a brown oil in 67% yield (29.1 mg); ^1H NMR (400 MHz, $CDCl_3$) δ 7.32 (dd, *J* = 15.9, 8.6 Hz, 2H), 7.24 – 7.17 (m, 2H), 3.72 – 3.52 (m, 2H), 2.53 (dd, *J* = 6.6, 3.4 Hz, 1H), 2.47 – 2.38 (m, 3H), 2.33 – 2.22 (m, 3H), 2.09 – 1.83 (m, 4H), 1.71 (s, 1H), 1.51 – 1.26 (m, 4H), 1.25 – 1.10 (m, 1H). ^{13}C NMR (101 MHz, $CDCl_3$) δ 137.2, 136.1, 128.8, 128.7, 62.3, 57.5, 37.6, 28.7, 26.4, 26.0, 21.0. HRMS (EI) m/z: $[M]^+$ calcd for $C_{15}H_{23}N$: 217.1830; found, 217.1830.

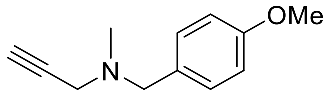

***N*-(4-methoxybenzyl)-*N*-methylprop-2-yn-1-amine (15):** The title compound was prepared according to the general procedure and purified by column chromatography on silica gel to afford a brown oil in 55% yield (20.7 mg); ^1H NMR (400 MHz, $CDCl_3$) δ 7.25 (d, *J* = 6.6 Hz, 2H), 6.85 (d, *J* = 6.6 Hz, 2H), 3.80 (s, 3H), 3.51 (s, 2H), 3.28 (s, 2H), 2.33 (s, 3H), 2.27 (d, *J* = 2.2 Hz, 1H). ^{13}C NMR (101 MHz, $CDCl_3$) δ 158.8, 130.3, 130.2, 113.7, 78.6, 73.3, 59.2, 55.2, 44.6, 41.6. (*52*)

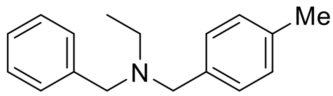

***N*-benzyl-*N*-(4-methylbenzyl)ethanamine (16):** The title compound was prepared according to the general procedure and purified by column chromatography on silica gel to afford a yellow oil in 77% yield (36.8 mg); ^1H NMR (400 MHz, $CDCl_3$) δ 7.42 (d, *J* = 7.5 Hz, 2H), 7.38 – 7.30 (m, 4H), 7.26 (dd, *J* = 9.3, 5.1 Hz, 1H), 7.17 (d, *J* = 7.7 Hz, 2H), 3.60 (d, *J* = 9.2 Hz, 4H), 2.55 (q, *J* = 7.1 Hz, 2H), 2.38 (s, 3H), 1.12 (t, *J* = 7.1 Hz, 3H). ^{13}C NMR (101 MHz, $CDCl_3$) δ 140.1, 136.8, 136.2, 128.8, 128.7, 128.6, 128.1, 126.6, 57.6, 57.4, 47.0, 21.1, 11.9. HRMS (EI) m/z: $[M]^+$ calcd for $C_{17}H_{21}N$: 239.1674; found, 239.1672.

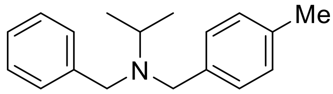

***N*-benzyl-*N*-(4-methylbenzyl)propan-2-amine (17):** The title compound was prepared according to the general procedure and purified by column chromatography on silica gel to afford a yellow oil in 71% yield (35.9 mg); ^1H NMR (400 MHz, $CDCl_3$) δ 7.46 (d, *J* = 7.5 Hz, 2H), 7.39 – 7.32 (m, 4H), 7.26 (dd, *J* = 9.4, 5.0 Hz, 1H), 7.17 (d, *J* = 7.8 Hz, 2H), 3.61 (d, *J* = 11.9 Hz, 4H), 3.10 – 2.88 (m, 1H), 2.39 (s, 3H), 1.13 (d, *J* = 6.7 Hz, 6H). ^{13}C NMR (101 MHz, $CDCl_3$) δ 141.1, 137.9, 136.0, 128.8, 128.4, 128.3 128.0 126.5

53.1, 52.9 48.0, 21.1 17.5. HRMS (EI) m/z: $[M]^+$ calcd for $C_{18}H_{23}N$: 253.1830; found, 253.1828.

***N*-(4-chlorobenzyl)-1-(4-fluorophenyl)-*N*-methylmethanamine (18):** The title compound was

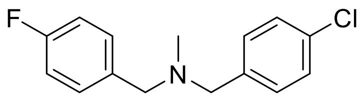

prepared according to the general procedure and purified by column chromatography on silica gel to afford a yellow oil in 90% yield (47.4 mg); ^{1}H NMR (400 MHz, $CDCl_3$) δ 7.33 – 7.27 (m, 6H), 7.01 (t, J = 8.7 Hz, 2H), 3.46 (s, 4H), 2.15 (s, 3H). ^{13}C NMR (101 MHz, $CDCl_3$) δ 162.0 (d, J = 244.7 Hz), 137.7, 134.8 (d, J = 3.1 Hz), 132.7, 130.3 (d, J = 7.9 Hz), 130.2, 128.4, 115.1 (d, J = 21.2 Hz), 61.0, 42.1. ^{19}F NMR (376 MHz, $CDCl_3$) δ -115.90. HRMS (EI) m/z: $[M]^+$ calcd for $C_{15}H_{15}ClFN$: 263.0877; found, 263.0876.

***N*-(4-fluorobenzyl)-*N*-methyl-1-(naphthalen-1-yl)methanamine (19):** The title compound was

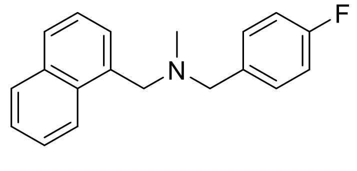

prepared according to the general procedure and purified by column chromatography on silica gel to afford a yellow oil in 81% yield (45.2 mg); ^{1}H NMR (400 MHz, $CDCl_3$) δ 8.29 (d, J = 7.9 Hz, 1H), 7.93 – 7.87 (m, 1H), 7.82 (d, J = 8.0 Hz, 1H), 7.61 – 7.48 (m, 3H), 7.45 (t, J = 7.5 Hz, 1H), 7.33 (dd, J = 8.1, 5.8 Hz, 2H), 7.04 (t, J = 8.7 Hz, 2H), 3.97 (s, 2H), 3.58 (s, 2H), 2.23 (s, 3H). ^{13}C NMR (101 MHz, $CDCl_3$) δ 161.9 (d, J = 244.5 Hz), 135.0 (d, J = 3.1 Hz), 134.8, 133.9, 132.5, 130.4 (d, J = 7.9 Hz), 128.4, 128.0, 127.4, 125.7, 125.6, 125.1, 124.8, 114.9 (d, J = 21.2 Hz), 61.4, 60.5, 42.2. ^{19}F NMR (376 MHz, $CDCl_3$) δ -115.97. HRMS (EI) m/z: $[M]^+$ calcd for $C_{19}H_{18}FN$: 279.1423; found, 279.1424.

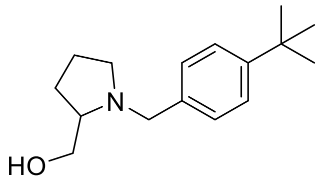

(1-(4-(tert-butyl)benzyl)pyrrolidin-2-yl)methanol (20): The title compound was prepared according to the general procedure and purified by column chromatography on silica gel to afford a yellow oil in 91% yield (45.0 mg); ^{1}H NMR (400 MHz, $CDCl_3$) δ 7.37 (d, J = 7.7 Hz, 2H), 7.26 (d, J = 7.8 Hz, 2H), 3.97 (d, J = 13.0 Hz, 1H), 3.69 (dd, J = 10.7, 2.5 Hz, 1H), 3.46 (d, J = 10.7 Hz, 1H), 3.37 (d, J = 13.0 Hz, 1H), 3.02 (dd, J = 8.7, 4.2 Hz, 1H), δ 2.80 (d, J = 34.6 Hz, 2H), 2.33 (q, J = 8.7 Hz, 1H), 1.95 (dd, J = 19.5, 10.6 Hz, 1H), 1.86 (td, J = 12.9, 6.3 Hz, 1H), 1.73 (dd, J = 13.6, 8.1 Hz, 2H), 1.35 (s, 9H). ^{13}C NMR (101 MHz, $CDCl_3$) δ 149.9, 136.2, 128.4, 125.2, 64.2, 61.8, 58.1, 54.4, 34.4, 31.4, 27.7, 23.4. IR (neat, ν/cm^{-1}) 3348, 3091, 3054, 2958, 2864, 2800, 1613, 1516, 1360, 1268, 1084, 1039, 1016, 832, 804, 552. HRMS (EI) m/z: $[M]^+$ calcd

for $C_{16}H_{25}NO$: 247.1936; found, 247.1933.

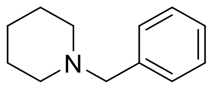

1-benzylpiperidine (21): The title compound was prepared according to the general procedure and purified by column chromatography on silica gel to afford a yellow oil in 74% yield (25.9 mg); ^{1}H NMR (400 MHz, $CDCl_3$) δ 7.35 (dd, J = 9.6, 3.7 Hz, 4H), 7.30 (dd, J = 6.0, 2.6 Hz, 1H), 3.53 (s, 2H), 2.37 (d, J = 45.0 Hz, 4H), 1.70 – 1.55 (m, 4H), 1.48 (d, J = 4.9 Hz, 2H). ^{13}C NMR (101 MHz, $CDCl_3$) δ 138.6, 129.2, 128.1, 126.8, 63.9, 54.5, 26.0, 24.4.

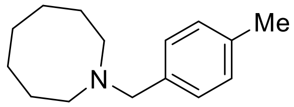

1-(4-methylbenzyl)azocane (22): The title compound was prepared according to the general procedure and purified by column chromatography on silica gel to afford a yellow oil in 84% yield (36.5 mg); ^{1}H NMR (400 MHz, $CDCl_3$) δ 7.30 (d, J = 8.5 Hz, 2H), 7.15 (d, J = 7.8 Hz, 2H), 3.63 (s, 2H), 2.62 (t, J = 5.1 Hz, 4H), 2.36 (s, 3H), 1.69 (s, 2H), 1.66 – 1.57 (m, 8H). ^{13}C NMR (101 MHz, $CDCl_3$) δ 136.4, 129.7, 129.0, 128.8, 62.9, 53.6, 27.6, 27.4, 26.2, 21.1. HRMS (EI) m/z: $[M]^+$ calcd for $C_{15}H_{23}N$: 217.1830; found, 217.1831.

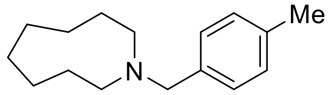

1-(4-methylbenzyl)azonane (23): The title compound was prepared according to the general procedure and purified by column chromatography on silica gel to afford a yellow oil in 79% yield (36.6 mg); ^{1}H NMR (400 MHz, $CDCl_3$) δ 7.23 (d, J = 7.8 Hz, 2H), 7.11 (d, J = 7.8 Hz, 2H), 3.53 (s, 2H), 2.52 – 2.39 (m, 4H), 2.33 (s, 3H), 1.56 (s, 8H), 1.50 – 1.40 (m, 4H). ^{13}C NMR (101 MHz, $CDCl_3$) δ 137.6, 136.1, 129.1, 128.7, 62.2, 52.9, 26.4, 25.6, 23.3, 21.1. HRMS (EI) m/z: $[M]^+$ calcd for $C_{16}H_{25}N$: 231.1987; found, 231.1984.

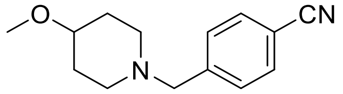

4-((4-methoxypiperidin-1-yl)methyl)benzonitrile (24): The title compound was prepared according to the general procedure and purified by column chromatography on silica gel to afford a yellow oil in 71% yield (32.7 mg); ^{1}H NMR (400 MHz, $CDCl_3$) δ 7.59 (d, J = 8.2 Hz, 2H), 7.44 (d, J = 8.1 Hz, 2H), 3.53 (s, 2H), 3.33 (s, 3H), 3.27 – 3.18 (m, 1H), 2.77 – 2.59 (m, 2H), 2.17 (t, J = 9.2 Hz, 2H), 1.88 (dd, J = 13.7, 3.3 Hz, 2H), 1.68 – 1.53 (m, 2H). ^{13}C NMR (101 MHz, $CDCl_3$) δ 144.6, 132.1, 129.4, 118.9, 110.8, 76.0,

62.4, 55.5, 51.0, 30.8. HRMS (EI) m/z: $[M]^+$ calcd for $C_{14}H_{18}N_2O$: 230.1419; found, 230.1421.

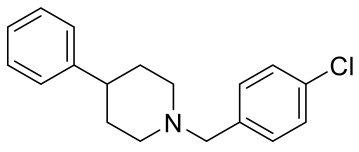

1-(4-chlorobenzyl)-4-phenylpiperidine (25): The title compound was prepared according to the general procedure and purified by column chromatography on silica gel to afford a yellow solid (mp: 62-63 ºC) in 85% yield (78.5 mg); ^1H NMR (400 MHz, $CDCl_3$) δ 7.37 – 7.32 (m, 6H), 7.26 (dd, *J* = 18.3, 7.4 Hz, 3H), 3.56 (s, 2H), 3.03 (d, *J* = 10.9 Hz, 2H), 2.61 – 2.47 (m, 1H), 2.13 (td, *J* = 10.9, 4.6 Hz, 2H), 1.93 – 1.76 (m, 4H). ^{13}C NMR (101 MHz, $CDCl_3$) δ 146.3, 137.0, 132.6, 130.4, 128.3, 128.3, 126.8, 126.1, 62.6, 54.2, 42.6, 33.4. IR (neat, ν/cm^{-1}) 3088, 3063, 3027, 294, 2933, 2787, 2752, 1597, 1487, 1336, 1256, 1120, 1080, 1016, 990, 844, 812, 778, 733, 693, 642, 567. HRMS (EI) m/z: $[M]^+$ calcd for $C_{18}H_{20}ClN$: 285.1284; found, 285.1285.

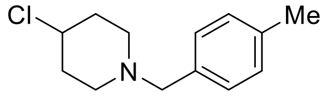

4-chloro-1-(4-methylbenzyl)piperidine (26): The title compound was prepared according to the general procedure and purified by column chromatography on silica gel to afford a yellow oil in 84% yield (39.2 mg); ^1H NMR (400 MHz, $CDCl_3$) δ 7.20 (d, *J* = 7.9 Hz, 2H), 7.13 (d, *J* = 7.8 Hz, 2H), 4.03 (s, 1H), 3.47 (s, 2H), 2.74 (d, *J* = 4.3 Hz, 2H), 2.35 (s, 3H), 2.23 (s, 2H), 2.13 – 2.01 (m, 2H), 1.95 – 1.83 (m, 2H). ^{13}C NMR (101 MHz, $CDCl_3$) δ 136.6, 135.1, 129.0, 128.9, 62.6, 57.6, 51.2, 35.6, 21.1. HRMS (EI) m/z: $[M]^+$ calcd for $C_{13}H_{18}ClN$: 223.1128; found, 223.1129.

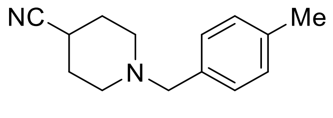

1-(4-methylbenzyl)piperidine-4-carbonitrile (27): The title compound was prepared according to the general procedure and purified by column chromatography on silica gel to afford a yellow oil in 81% yield (34.7 mg); ^1H NMR (400 MHz, $CDCl_3$) δ 7.18 (d, *J* = 8.0 Hz, 2H), 7.12 (d, *J* = 7.9 Hz, 2H), 3.47 (s, 2H), 2.78 – 2.54 (m, 3H), 2.32 (d, *J* = 12.5 Hz, 5H), 1.98 – 1.79 (m, 4H). ^{13}C NMR (101 MHz, $CDCl_3$) δ 136.8, 134., 129.0, 128.9, 121.8, 62.8, 51.2, 28.8, 26.2, 21.0. IR (neat, ν/cm^{-1}) 3056, 3028, 2958, 2933, 2817, 2767, 2234, 1516, 1441, 1259, 1143, 1096, 1040, 808, 770, 538. HRMS (EI) m/z: $[M]^+$ calcd for $C_{14}H_{18}N_2$: 214.1470; found, 214.1466.

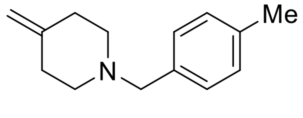

1-(4-methylbenzyl)-4-methylenepiperidine (28): The title compound was prepared according to the general procedure and

purified by column chromatography on silica gel to afford a yellow oil in 78% yield (31.4 mg); ^{1}H NMR (400 MHz, $CDCl_3$) δ 7.23 (d, *J* = 7.8 Hz, 2H), 7.14 (d, *J* = 7.8 Hz, 2H), 4.66 (s, 2H), 3.50 (s, 2H), 2.45 (t, *J* = 5.6 Hz, 4H), 2.35 (s, 3H), 2.26 (t, *J* = 5.6 Hz, 4H). ^{13}C NMR (101 MHz, $CDCl_3$) δ 146.7, 136.5, 135.3, 129.1, 128.8, 107.5, 62.6, 54.9, 34.6, 21.1. HRMS (EI) m/z: $[M]^+$ calcd for $C_{14}H_{19}N$: 201.1517; found, 201.1525.

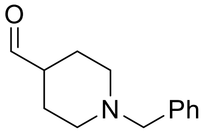

1-benzylpiperidine-4-carbaldehyde (29): The title compound was prepared according to the general procedure and purified by column chromatography on silica gel to afford a yellow oil in 62% yield (25.2 mg); ^{1}H NMR (400 MHz, $CDCl_3$) δ 9.66 (s, 1H), 7.33 (d, *J* = 4.2 Hz, 4H), 7.28 (dd, *J* = 9.0, 4.4 Hz, 1H), 3.52 (s, 2H), 2.84 (dd, *J* = 7.5, 4.1 Hz, 2H), 2.33 – 2.21 (m, 1H), 2.13 (t, *J* = 11.1 Hz, 2H), 1.90 (d, *J* = 13.2 Hz, 2H), 1.71 (ddd, *J* = 14.0, 10.7, 3.7 Hz, 2H). ^{13}C NMR (101 MHz, $CDCl_3$) δ 203.9, 138.1 129.0, 128.1, 127.0, 63.1, 52.4, 47.9, 25.3. (*53*)

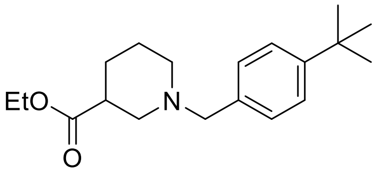

ethyl 1-(4-(tert-butyl)benzyl)piperidine-3-carboxylate (30): The title compound was prepared according to the general procedure and purified by column chromatography on silica gel to afford a yellow oil in 84% yield (50.9 mg); ^{1}H NMR (400 MHz, $CDCl_3$) δ 7.32 (d, *J* = 8.2 Hz, 2H), 7.22 (d, *J* = 8.2 Hz, 2H), 4.10 (q, *J* = 7.1 Hz, 2H), 3.49 (q, *J* = 13.1 Hz, 2H), 2.96 (d, *J* = 10.5 Hz, 1H), 2.74 (d, *J* = 11.1 Hz, 1H), 2.63 – 2.52 (m, 1H), 2.21 (t, *J* = 10.5 Hz, 1H), 2.02 (t, *J* = 9.6 Hz, 1H), 1.96 – 1.84 (m, 1H), 1.75 – 1.66 (m, 1H), 1.64 – 1.52 (m, 1H), 1.47 (t, *J* = 9.9 Hz, 1H), 1.31 (s, 9H), 1.22 (t, *J* = 7.1 Hz, 3H). ^{13}C NMR (101 MHz, $CDCl_3$) δ 174.3, 149.9, 135.0, 128.8, 125.0, 62.9, 60.2, 55.3, 53.5, 41.9, 34.4, 31.4, 27.0, 24.5, 14.2. HRMS (EI) m/z: $[M]^+$ calcd for $C_{19}H_{29}NO_2$: 303.2198; found, 303.2196.

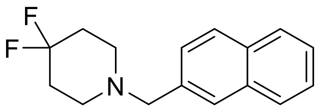

4,4-difluoro-1-(naphthalen-2-ylmethyl)piperidine (31): The title compound was prepared according to the general procedure and purified by column chromatography on silica gel to afford a yellow solid (mp: 82-83 ºC) in 86% yield (44.9 mg); ^{1}H NMR (400 MHz, $CDCl_3$) δ 7.82 (t, *J* = 7.0 Hz, 3H), 7.73 (s, 1H), 7.47 (dd, *J* = 16.6, 7.6 Hz, 3H), 3.71 (s, 2H), 2.60 (s, 4H), 2.11 – 1.90 (m, 4H), 1.68 (s, 1H). ^{13}C NMR (101 MHz, $CDCl_3$) δ 135.8, 133.3, 132.8, 128.0, 127.7, 127.6, 127.5, 127.1, 126.0, 125.7, 122.1 (t, *J* = 241.4

Hz), 62.3, 50.0 (t, $J = 5.3$ Hz), 34.1 (t, $J = 22.9$ Hz). ^{19}F NMR (376 MHz, $CDCl_3$) δ -98.19. IR (neat, ν/cm^{-1}) 3056, 3044, 3020, 2972, 2957, 2918, 2846, 2826, 1644, 1597, 1441, 1357, 1305, 1242, 1139, 1076, 1011, 933, 869, 827, 791, 754, 630. HRMS (EI) m/z: $[M]^+$ calcd for $C_{16}H_{17}F_2N$: 261.1329; found, 261.1332.

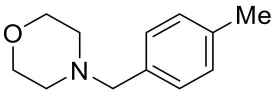

4-(4-methylbenzyl)morpholine (32): The title compound was prepared according to the general procedure and purified by column chromatography on silica gel to afford a yellow oil in 70% yield (26.8 mg); ^{1}H NMR (400 MHz, $CDCl_3$) δ 7.21 (d, $J = 7.9$ Hz, 2H), 7.13 (d, $J = 7.8$ Hz, 2H), 3.73 – 3.68 (m, 4H), 3.46 (s, 2H), 2.44 (d, $J = 4.0$ Hz, 4H), 2.34 (s, 3H). ^{13}C NMR (101 MHz, $CDCl_3$) δ 136.7, 134.5, 129.2, 128.9, 67.0, 63.2, 53.5, 21.1.

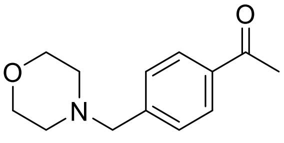

1-(4-(morpholinomethyl)phenyl)ethan-1-one (33): The title compound was prepared according to the general procedure and purified by column chromatography on silica gel to afford a yellow oil in 68% yield (29.8 mg); ^{1}H NMR (400 MHz, $CDCl_3$) δ 7.90 (d, $J = 8.2$ Hz, 2H), 7.42 (d, $J = 8.1$ Hz, 2H), 3.76 – 3.58 (m, 4H), 3.54 (s, 2H), 2.58 (s, 3H), 2.49 – 2.37 (m, 4H). ^{13}C NMR (101 MHz, $CDCl_3$) δ 197.7, 143.6, 136.2, 129.1, 128.4, 66.9, 62.9, 53.6, 26.5.

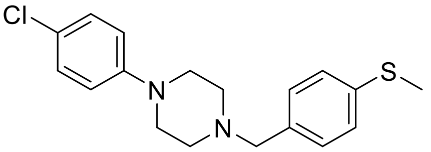

1-(4-chlorophenyl)-4-(4-(methylthio)benzyl)piperazine (34): The title compound was prepared according to the general procedure and purified by column chromatography on silica gel to afford a brown solid (mp 75-76 ºC) in 87% yield (57.8 mg); ^{1}H NMR (400 MHz, $CDCl_3$) δ 7.28 (dt, $J = 14.8$, 8.5 Hz, 6H), 6.87 (d, $J = 8.9$ Hz, 2H), 3.57 (s, 2H), 3.22 – 3.18 (m, 4H), 2.65 – 2.62 (m, 4H), 2.53 (s, 3H). ^{13}C NMR (101 MHz, $CDCl_3$) δ 149.9, 137.1, 134.8, 129.7, 128.9, 126.6, 124.4, 117.2, 62.5, 52.9, 49.1, 16.0. IR (neat, ν/cm^{-1}) 3086, 3041, 3016, 2921, 2818, 1598, 1498, 1231, 1135, 1006, 919, 814, 676. HRMS (EI) m/z: $[M]^+$ calcd for $C_{18}H_{21}ClN_2S$: 332.1114; found, 332.1115.

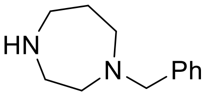

1-benzyl-1,4-diazepane (35): The title compound was prepared according to the general procedure and purified by column chromatography on silica gel to

afford a yellow oil in 77% yield (29.3 mg); ^{1}H NMR (400 MHz, $CDCl_3$) δ 7.30 (dt, *J* = 14.8, 7.3 Hz, 4H), 7.22 (t, *J* = 7.0 Hz, 1H), 3.64 (s, 2H), 2.93 (t, *J* = 6.1 Hz, 2H), 2.89 – 2.84 (m, 2H), 2.74 – 2.53 (m, 4H), 2.09 (s, 1H), 1.79 – 1.69 (m, 2H). ^{13}C NMR (101 MHz, $CDCl_3$) δ 139.5, 128.7, 128.1, 126.7, 62.8, 58.3, 54.5, 48.8, 47.3, 30.5.

4-((2'-(trifluoromethyl)-[1,1'-biphenyl]-4-yl)methyl)thiomorpholine (36): The title compound

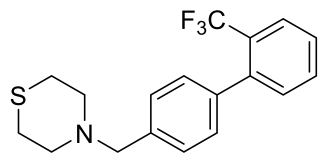

was prepared according to the general procedure and purified by column chromatography on silica gel to afford a brown oil in 68% yield (45.8 mg);^{1}H NMR (400 MHz, $CDCl_3$) δ 7.79 (d, *J* = 7.8 Hz, 1H), 7.60 (t, *J* = 7.5 Hz, 1H), 7.51 (t, *J* = 7.6 Hz, 1H), 7.40 (d, *J* = 2.7 Hz, 1H), 7.38 (s, 2H), 7.33 (d, *J* = 8.0 Hz, 2H), 3.62 (s, 2H), 2.78 (dd, *J* = 14.9, 6.2 Hz, 8H). ^{13}C NMR (101 MHz, $CDCl_3$) δ 141.2, 138.6, 137.5, 132.0, 131.2, 128.8, 128.3, 127.2, 126.0 (q, *J* = 5.3 Hz), 125.5, 122.8, 63.4, 55.0, 28.0. ^{19}F NMR (376 MHz, $CDCl_3$) δ -56.75. IR (neat, ν/cm^{-1}) 3067, 3027, 2917, 2806, 1598, 1488, 1447, 1309, 1165, 1103, 1071, 1033, 1002, 952, 767, 650. HRMS (EI) m/z: [M]$^+$ calcd for $C_{18}H_{18}F_3NS$: 337.1112; found, 337.1118.

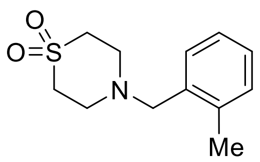

4-(2-methylbenzyl)thiomorpholine 1,1-dioxide (37): The title compound was prepared according to the general procedure and purified by column chromatography on silica gel to afford a yellow oil in 69% yield (33.0 mg); ^{1}H NMR (400 MHz, $CDCl_3$) δ 7.23 – 7.13 (m, 4H), 3.60 (s, 2H), 3.02 (d, *J* = 6.2 Hz, 4H), 2.98 (d, *J* = 6.2 Hz, 4H), 2.35 (s, 3H). ^{13}C NMR (101 MHz, $CDCl_3$) δ 137.5, 135.2, 130.6, 129.7, 127.7, 125.8, 59.4, 51.4, 50.5, 19.1. IR (neat, ν/cm^{-1}) 3067, 3027, 2972, 2952, 2921, 2820, 1597, 1484, 1266, 1185, 1117, 1044, 859, 767, 670. HRMS (EI) m/z: [M]$^+$ calcd for $C_{12}H_{17}NO_2S$: 239.0980; found, 239.0977.

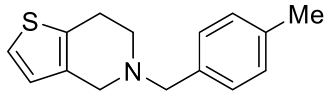

5-(4-methylbenzyl)-4,5,6,7-tetrahydrothieno[3,2-c]pyridine (38): The title compound was prepared according to the general procedure and purified by column chromatography on silica gel to afford a yellow oil in 81% yield (39.4 mg); ^{1}H NMR (400 MHz, $CDCl_3$) δ 7.32 (d, *J* = 7.9 Hz, 2H), 7.19 (d, *J* = 7.9 Hz, 2H), 7.09 (d, *J* = 5.1 Hz, 1H), 6.73 (d, *J* = 5.1 Hz, 1H), 3.72 (s, 2H), 3.59 (s, 2H), 2.92 (t, *J* = 5.5 Hz, 2H), 2.84 (t, *J* = 5.5 Hz, 2H), 2.40 (s, 3H). ^{13}C NMR (101 MHz, $CDCl_3$) δ 136.6, 135.2, 133.9, 133.4,

129.0, 128.9, 125.2, 122.5, 61.9, 53.0, 50.4, 25.4, 21.1. HRMS (EI) m/z: $[M]^+$ calcd for $C_{15}H_{17}NS$: 243.1082; found, 243.1082.

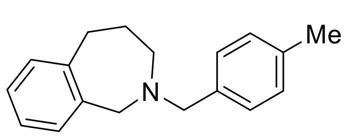

2-(4-methylbenzyl)-2,3,4,5-tetrahydro-1H-benzo[c]azepine (39): The title compound was prepared according to the general procedure and purified by column chromatography on silica gel to afford a yellow oil in 77% yield (38.6 mg); ^{1}H NMR (400 MHz, $CDCl_3$) δ 7.22 – 7.08 (m, 7H), 6.97 (d, J = 7.2 Hz, 1H), 3.89 (s, 2H), 3.51 (s, 2H), 3.21 – 3.02 (m, 2H), 3.01 – 2.80 (m, 2H), 2.36 (s, 3H), 1.77 (dd, J = 10.1, 5.2 Hz, 2H). ^{13}C NMR (101 MHz, $CDCl_3$) δ 143.1, 139.4, 136.4, 136.1, 129.9, 128.9, 128.8, 128.7, 127.1, 125.8, 59.3, 58.7, 57.6, 36.1, 25.2, 21.1. HRMS (EI) m/z: $[M]^+$ calcd for $C_{18}H_{21}N$: 251.1674; found, 251.1677.

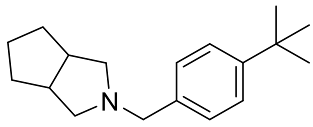

2-(4-(*tert*-butyl)benzyl)octahydrocyclopenta[*c*]pyrrole (40): The title compound was prepared according to the general procedure and purified by column chromatography on silica gel to afford a yellow oil in 80% yield (41.2 mg); ^{1}H NMR (400 MHz, $CDCl_3$) δ 7.33 (d, J = 8.2 Hz, 2H), 7.27 (d, J = 4.1 Hz, 2H), 3.55 (s, 2H), 2.89 – 2.77 (m, 2H), 2.62 (s, 2H), 2.06 (dd, J = 9.0, 5.0 Hz, 2H), 1.68 – 1.58 (m, 3H), 1.56 – 1.47 (m, 1H), 1.43 (d, J = 3.0 Hz, 2H), 1.32 (s, 9H). ^{13}C NMR (101 MHz, $CDCl_3$) δ 149.8, 135.8, 128.5, 125.1, 61.5, 59.7, 42.4, 34.4, 32.5, 31.4, 25.8. HRMS (EI) m/z: $[M]^+$ calcd for $C_{18}H_{27}N$: 257.2143; found, 257.2145.

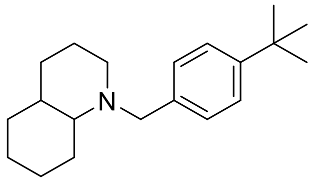

1-(4-(tert-butyl)benzyl)decahydroquinoline (41): The title compound was prepared according to the general procedure and purified by column chromatography on silica gel to afford a yellow oil in 75% yield (42.8 mg); ^{1}H NMR (400 MHz, $CDCl_3$) δ 7.33 (dd, J = 4.9, 3.0 Hz, 2H), 7.24 – 7.20 (m, 2H), 4.05 (dd, J = 13.4, 2.9 Hz, 1H), 3.20 (dd, J = 13.5, 3.3 Hz, 1H), 2.85 (d, J = 10.8 Hz, 1H), 2.30 (d, J = 11.9 Hz, 1H), 1.93 (t, J = 11.1 Hz, 1H), 1.82 (d, J = 8.2 Hz, 1H), 1.76 (t, J = 9.9 Hz, 1H), 1.70 – 1.48 (m, 5H), 1.32 (d, J = 3.6 Hz, 9H), 1.29 – 1.14 (m, 4H), 1.11 – 0.90 (m, 2H). ^{13}C NMR (101 MHz, $CDCl_3$) δ 149.4, 136.3, 128.9, 124.9, 66.8, 56.7, 53.7, 42.1, 34.4, 33.3, 32.7, 31.4, 30.7, 26.1, 25.8, 25.6. HRMS (EI) m/z: $[M]^+$ calcd for $C_{20}H_{31}N$: 285.2457; found, 285.2456.

(1S)-8-(4-methoxybenzyl)-8-azabicyclo[3.2.1]octan-3-one (42): The title compound was prepared

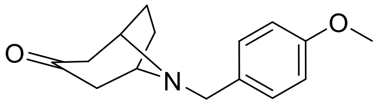

according to the general procedure and purified by column chromatography on silica gel to afford a yellow oil in 73% yield (35.8 mg); ^{1}H NMR (400 MHz, $CDCl_3$) δ 7.32 (d, *J* = 8.5 Hz, 2H), 6.87 (d, *J* = 8.5 Hz, 2H), 3.80 (s, 3H), 3.67 (s, 2H), 3.48 (s, 2H), 2.67 (dd, *J* = 16.0, 4.0 Hz, 2H), 2.19 (d, *J* = 15.6 Hz, 2H), 2.09 (dd, *J* = 7.4, 3.2 Hz, 2H), 1.61 (d, *J* = 7.9 Hz, 2H). ^{13}C NMR (101 MHz, $CDCl_3$) δ 210.4, 158.8, 131.4, 129.6, 113.8, 58.4, 55.3, 54.5, 48.3, 27.8. HRMS (EI) m/z: $[M]^+$ calcd for $C_{15}H_{19}NO_2$: 245.1416; found, 245.1419.

***tert*-butyl 5-(4-methoxybenzyl)-2,5-diazabicyclo[2.2.1]heptane-2-carboxylate (43):** The title

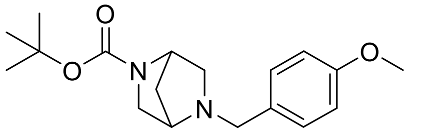

compound was prepared according to the general procedure and purified by column chromatography on silica gel to afford a yellow solid (mp: 62-63 ºC) in 80% yield (50.9 mg); ^{1}H NMR (400 MHz, $CDCl_3$) δ 7.22 (d, *J* = 8.5 Hz, 2H), 6.82 (d, *J* = 8.6 Hz, 2H), 4.20 (s, 1H), 3.76 (s, 3H), 3.64 (s, 2H), 3.58 (d, *J* = 10.2 Hz, 1H), 3.41 (d, *J* = 8.8 Hz, 1H), 3.12 (t, *J* = 9.0 Hz, 1H), 2.86 (d, *J* = 9.5 Hz, 1H), 2.51 (d, *J* = 9.5 Hz, 1H), 1.81 (s, 1H), 1.67 (d, *J* = 9.4 Hz, 1H), 1.44 (s, 9H). ^{13}C NMR (101 MHz, $CDCl_3$) δ 158.5, 154.3, 131.4, 129.4, 113.6, 79.2, 60.1, 59.8, 57.9, 57.2, 55.1, 49.1, 35.8, 28.4. IR (neat, ν/cm^{-1}) 3067, 3044, 3004, 2976, 2928, 2902, 2857, 2801, 1677, 1608, 1507, 1411, 1245, 1181, 1144, 1103, 1034, 809, 749, 621. HRMS (EI) m/z: $[M]^+$ calcd for $C_{18}H_{26}N_2O_3$: 318.1943; found, 318.1940.

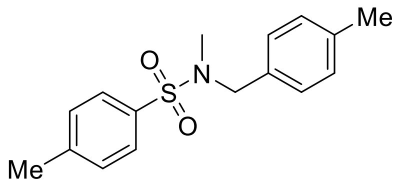

***N*,4-dimethyl-*N*-(4-methylbenzyl)benzenesulfonamide (44):** The title compound was prepared according to the general procedure and purified by column chromatography on silica gel to afford a yellow solid (mp: 76-77 ºC) in 95% yield (54.9 mg); ^{1}H NMR (400 MHz, $CDCl_3$) δ 7.72 (d, *J* = 8.0 Hz, 2H), 7.35 (d, *J* = 7.9 Hz, 2H), 7.19 (d, *J* = 7.8 Hz, 2H), 7.13 (d, *J* = 7.8 Hz, 2H), 4.08 (s, 2H), 2.56 (s, 3H), 2.44 (s, 3H), 2.32 (s, 3H). ^{13}C NMR (101 MHz, $CDCl_3$) δ 143.2, 137.3, 134.1, 132.4, 129.5, 129.1, 128.1, 127.2, 53.6, 34.0, 21.3, 20.9.

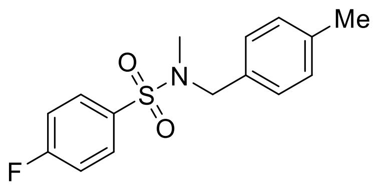

4-fluoro-*N*-methyl-*N*-(4-methylbenzyl)benzenesulfonamide (45): The title compound was prepared according to the general procedure and purified by column chromatography on silica gel to afford a yellow solid (mp: 70-71 ºC) in 88% yield (51.6 mg); ^{1}H NMR (400 MHz, $CDCl_3$) δ 7.86 (dd, *J* = 8.1, 5.2 Hz, 2H), 7.23 (t, *J* = 8.4 Hz, 2H), 7.18 (d, *J* = 7.9 Hz, 2H), 7.14 (d, *J* = 7.9 Hz, 2H), 4.11 (s, 2H), 2.60 (s, 3H), 2.34 (s, 3H). ^{13}C NMR (101 MHz, $CDCl_3$) δ 165.1 (d, *J* = 254.6 Hz), 137.8, 133.6 (d, *J* = 3.2 Hz), 132.3, 130.1 (d, *J* = 9.2 Hz), 129.4, 128.4, 116.4 (d, *J* = 22.5 Hz), 53.9, 34.2, 21.1. ^{19}F NMR (376 MHz, $CDCl_3$) δ -105.39. HRMS (EI) m/z: $[M]^+$ calcd for $C_{15}H_{16}FNO_2S$: 293.0886; found, 293.0884.

4-chloro-*N*-methyl-*N*-(4-methylbenzyl)benzenesulfonamide (46): The title compound was

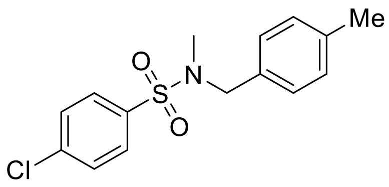

prepared according to the general procedure and purified by column chromatography on silica gel to afford a yellow solid (mp: 78-79 ºC) in 94% yield (58.1 mg); ^{1}H NMR (400 MHz, $CDCl_3$) δ 7.77 (d, *J* = 8.7 Hz, 2H), 7.52 (d, *J* = 8.7 Hz, 2H), 7.18 (d, *J* = 8.2 Hz, 2H), 7.14 (d, *J* = 8.1 Hz, 2H), 4.11 (s, 2H), 2.59 (s, 3H), 2.34 (s, 3H). ^{13}C NMR (101 MHz, $CDCl_3$) δ 139.2, 137.8, 136.1, 132.2, 129.5, 129.4, 128.9, 128.4, 53.9, 34.2, 21.1. HRMS (EI) m/z: $[M]^+$ calcd for $C_{15}H_{16}ClNO_2S$: 309.0590; found, 309.0594.

***N*-(4-(tert-butyl)benzyl)-*N*,4-dimethylbenzenesulfonamide (47):** The title compound was

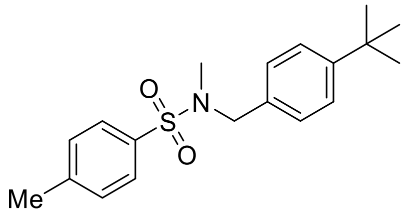

prepared according to the general procedure and purified by column chromatography on silica gel to afford a yellow solid (mp: 84-85 ºC) in 99% yield (65.5 mg); ^{1}H NMR (400 MHz, $CDCl_3$) δ 7.73 (d, *J* = 8.1 Hz, 2H), 7.38 – 7.32 (m, 4H), 7.24 (d, *J* = 8.2 Hz, 2H), 4.11 (s, 2H), 2.59 (s, 3H), 2.44 (s, 3H), 1.32 (s, 9H). ^{13}C NMR (101 MHz, $CDCl_3$) δ 150.7, 143.3, 134.3, 132.5, 129.6, 128.0, 127.4, 125.4, 53.7, 34.4, 34.2, 31.2, 21.4. HRMS (EI) m/z: $[M]^+$ calcd for $C_{19}H_{25}NO_2S$: 331.1606; found, 331.1608.

N-(4-fluorobenzyl)-N,4-dimethylbenzenesulfonamide (48): The title compound was prepared

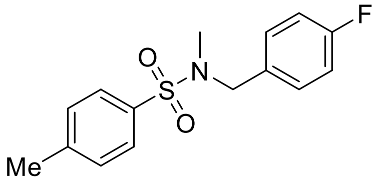

according to the general procedure and purified by column chromatography on silica gel to afford a yellow solid (mp: 76-77 ºC) in 92% yield (53.9 mg); ^{1}H NMR (400 MHz, $CDCl_3$) δ 7.73 (d, J = 8.3 Hz, 2H), 7.37 (d, J = 8.0 Hz, 2H), 7.34 – 7.26 (m, 2H), 7.06 – 6.97 (m, 2H), 4.11 (s, 2H), 2.59 (s, 3H), 2.46 (s, 3H). ^{13}C NMR (101 MHz, $CDCl_3$) δ 162.4 (d, J = 246.2 Hz), 143.5, 134.2, 131.4 (d, J = 3.2 Hz), 130.0 (d, J = 8.2 Hz), 129.7, 127.4, 115.4 (d, J = 21.5 Hz). 53.4, 34.2, 21.4. ^{19}F NMR (376 MHz, $CDCl_3$) δ -114.44.

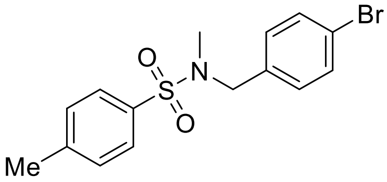

N-(4-bromobenzyl)-N,4-dimethylbenzenesulfonamide (49): The title compound was prepared according to the general procedure and purified by column chromatography on silica gel to afford a yellow solid (mp: 94-95 ºC) in 77% yield (54.4 mg); ^{1}H NMR (400 MHz, $CDCl_3$) δ 7.70 (d, J = 8.2 Hz, 2H), 7.44 (d, J = 8.3 Hz, 2H), 7.34 (d, J = 8.1 Hz, 2H), 7.17 (d, J = 8.3 Hz, 2H), 4.06 (s, 2H), 2.57 (s, 3H), 2.44 (s, 3H). ^{13}C NMR (101 MHz, $CDCl_3$) δ 143.7, 134.9, 134.2, 131.9, 130.0, 129.8, 127.5, 121.8, 53.6, 34.5, 21.6. HRMS (EI) m/z: $[M]^+$ calcd for $C_{15}H_{16}BrNO_2S$: 353.0085; found, 353.0084.

4-amino-N-methyl-N-(4-methylbenzyl)benzenesulfonamide (50): The title compound was

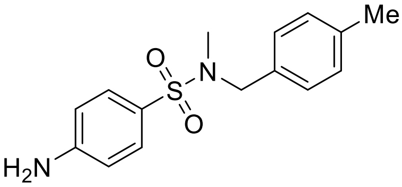

prepared according to the general procedure and purified by column chromatography on silica gel to afford a yellow oil in 66% yield (38.3 mg); ^{1}H NMR (400 MHz, $CDCl_3$) δ 7.60 (d, J = 8.6 Hz, 2H), 7.18 (d, J = 7.9 Hz, 2H), 7.12 (d, J = 7.9 Hz, 2H), 6.71 (d, J = 8.6 Hz, 2H), 4.22 – 4.13 (m, 2H), 4.04 (s, 2H), 2.53 (s, 3H), 2.33 (s, 3H).^{13}C NMR (101 MHz, $CDCl_3$) δ 150.6, 137.5, 132.9, 129.6, 129.3, 128.4, 125.3, 114.1, 53.9, 34.3, 21.1. HRMS (EI) m/z: $[M]^+$ calcd for $C_{15}H_{18}N_2O_2S$: 290.1089; found, 290.1090.

1-(benzo[d][1,3]dioxol-5-ylmethyl)-4-(thiophen-2-ylmethyl)piperazine (51): The title compound was prepared according to the general procedure and purified by column

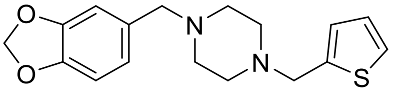

chromatography on silica gel to afford a brown solid (mp 78-79 ºC) in 65% yield (41.1 mg); ^{1}H NMR (400 MHz, $CDCl_3$) δ 7.21 (d, J = 5.0 Hz, 1H), 6.95 – 6.92 (m, 1H), 6.90 (d, J = 2.9 Hz, 1H), 6.84 (s, 1H), 6.73 (s, 2H), 5.92 (s, 2H), 3.72 (s, 2H), 3.41 (s, 2H), 2.48 (s, 8H). ^{13}C NMR (101 MHz, $CDCl_3$) δ 147.5, 146.4, 141.4, 132.0, 126.3, 125.9, 124.8, 122.1, 109.4, 107.7, 100.7, 63.0, 56.9, 52.8, 52.7. HRMS (EI) m/z: $[M]^+$ calcd for $C_{17}H_{20}N_2O_2S$: 316.1245; found, 316.1243.

***N*,4-dimethyl-*N*-(thiophen-2-ylmethyl)benzenesulfonamide (52):** The title compound was

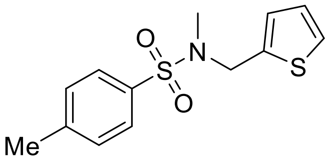

prepared according to the general procedure and purified by column chromatography on silica gel to afford a brown oil in 76% yield (42.7 mg); ^{1}H NMR (400 MHz, $CDCl_3$) δ 7.70 (d, J = 8.3 Hz, 2H), 7.33 (d, J = 8.0 Hz, 2H), 7.24 (dd, J = 3.9, 2.5 Hz, 1H), 6.92 (dd, J = 4.4, 3.0 Hz, 2H), 4.36 (s, 2H), 2.66 (s, 3H), 2.44 (s, 3H). ^{13}C NMR (101 MHz, $CDCl_3$) δ 143.6, 138.6, 134.4, 129.8, 127.5, 127.2, 126.7, 126.1, 48.9, 34.2, 21.6. HRMS (EI) m/z: $[M]^+$ calcd for $C_{13}H_{15}NO_2S_2$: 281.0544; found, 281.0547.

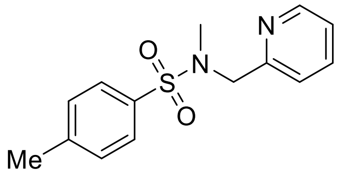

***N*,4-dimethyl-*N*-(pyridin-2-ylmethyl)benzenesulfonamide (53):** The title compound was prepared according to the general procedure and purified by column chromatography on silica gel to afford a brown oil in 73% yield (40.3 mg); ^{1}H NMR (400 MHz, $CDCl_3$) δ 8.48 (d, J = 4.7 Hz, 1H), 7.71 (t, J = 6.4 Hz, 3H), 7.56 (d, J = 7.9 Hz, 1H), 7.33 (d, J = 8.1 Hz, 2H), 7.22 – 7.14 (m, 1H), 4.29 (s, 2H), 2.68 (s, 3H), 2.44 (s, 3H). ^{13}C NMR (101 MHz, $CDCl_3$) δ 156.5, 149.1, 143.6, 137.1, 134.2, 129.8, 127.5, 122.7, 122.4, 56.1, 35.3, 21.5. HRMS (EI) m/z: $[M]^+$ calcd for $C_{14}H_{16}N_2O_2S$: 276.0932; found, 276.0936.

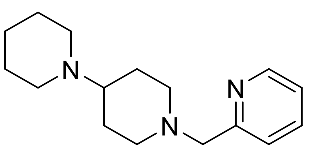

1'-(pyridin-2-ylmethyl)-1,4'-bipiperidine (54): The title compound was prepared according to the general procedure and purified by column chromatography on silica gel to afford a brown oil in 74% yield (38.4 mg); ^{1}H NMR (400 MHz, $CDCl_3$) δ 8.44 (d, J = 3.1 Hz, 1H), 7.54 (t, J = 7.6 Hz, 1H),

7.30 (d, J = 7.7 Hz, 1H), 7.04 (t, J = 6.0 Hz, 1H), 3.53 (s, 2H), 2.85 (d, J = 10.8 Hz, 2H), 2.40 (s, 4H), 2.18 (t, J = 11.5 Hz, 1H), 1.96 (t, J = 11.6 Hz, 2H), 1.66 (d, J = 11.6 Hz, 2H), 1.56 (t, J = 12.0 Hz, 2H), 1.47 (s, 4H), 1.32 (d, J = 4.4 Hz, 2H). ^{13}C NMR (101 MHz, $CDCl_3$) δ 158.7, 148.8, 136.0, 122.8, 121.6, 64.3, 62.4, 53.5, 49.9, 27.5, 26.1, 24.5. (*54*)

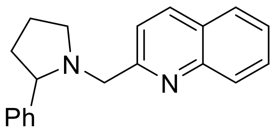

2-((2-phenylpyrrolidin-1-yl)methyl)quinoline (55): The title compound was prepared according to the general procedure and purified by column chromatography on silica gel to afford a yellow oil in 72% yield (41.5 mg); ^{1}H NMR (400 MHz, $CDCl_3$) δ 8.09 (dd, J = 20.3, 8.5 Hz, 2H), 7.81 (d, J = 8.0 Hz, 1H), 7.75 – 7.61 (m, 2H), 7.52 (t, J = 7.7 Hz, 3H), 7.37 (t, J = 7.5 Hz, 2H), 7.28 (d, J = 6.6 Hz, 1H), 4.13 (d, J = 13.9 Hz, 1H), 3.55 (dd, J = 16.7, 11.0 Hz, 2H), 3.14 (dd, J = 12.6, 4.8 Hz, 1H), 2.43 (q, J = 8.7 Hz, 1H), 2.31 – 2.22 (m, 1H), 2.01 – 1.92 (m, 1H), 1.91 – 1.77 (m, 2H). ^{13}C NMR (101 MHz, $CDCl_3$) δ 160.9, 147.4, 143.6, 136.2, 129.2, 128.9, 128.4, 127.6, 127.4, 127.3, 127.1, 125.9, 121.1, 70.0, 60.7, 53.8, 35.1, 22.7. IR (neat, ν/cm^{-1}) 3059, 3024, 2967, 2921, 2848, 1598, 1503, 1424, 1374, 1264, 1084, 1048, 878, 759, 699, 667. HRMS (EI) m/z: [M]$^+$ calcd for $C_{20}H_{20}N_2$: 288.1626; found, 288.1630.

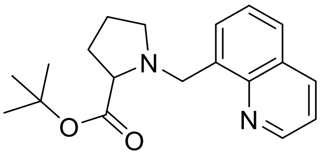

***tert*-butyl (quinolin-8-ylmethyl)prolinate (56):** The title compound was prepared according to the general procedure and purified by column chromatography on silica gel to afford a yellow oil in 55% yield (34.3 mg); ^{1}H NMR (400 MHz, $CDCl_3$) δ 8.95 – 8.83 (m, 1H), 8.13 (d, J = 8.2 Hz, 1H), 7.95 (d, J = 7.0 Hz, 1H), 7.70 (d, J = 8.1 Hz, 1H), 7.53 (t, J = 7.6 Hz, 1H), 7.37 (dd, J = 8.2, 4.2 Hz, 1H), 4.52 (s, 2H), 3.34 (dd, J = 8.4, 6.3 Hz, 1H), 3.18 (dd, J = 11.0, 5.5 Hz, 1H), 2.46 (dd, J = 16.5, 8.1 Hz, 1H), 2.20 – 2.07 (m, 1H), 1.94 (dt, J = 20.7, 7.4 Hz, 2H), 1.82 – 1.68 (m, 1H), 1.41 (s, 9H). ^{13}C NMR (101 MHz, $CDCl_3$) δ 173.6, 149.2, 146.7, 137.2, 136.2, 129.6, 128.1, 126.6, 126.4, 120.7, 80.3, 66.3, 53.7, 53.1, 29.3, 28.1, 23.2. IR (neat, ν/cm^{-1}) 3081, 3064, 2976, 2928, 2876, 1721, 1654, 1597, 1495, 1364, 1251, 1144, 1084, 1044, 879, 830, 778, 668. HRMS (EI) m/z: [M]$^+$ calcd for $C_{19}H_{24}N_2O_2$: 312.1838; found, 312.1836.

***N*-([2,2'-bipyridin]-6-ylmethyl)-*N*-methylpentan-1-amine (57):** The title compound was

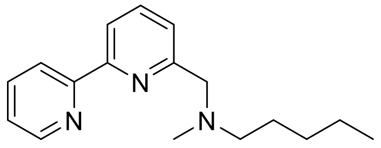

prepared according to the general procedure and purified by column chromatography on silica gel to afford a yellow oil in 70% yield (37.7 mg); ^{1}H NMR (600 MHz, $CDCl_3$) δ 8.66 (d, *J* = 4.6 Hz, 1H), 8.42 (d, *J* = 8.0 Hz, 1H), 8.24 (d, *J* = 7.8 Hz, 1H), 7.79 (dt, *J* = 14.1, 4.8 Hz, 2H), 7.47 (d, *J* = 7.6 Hz, 1H), 7.28 (ddd, *J* = 7.4, 4.8, 0.9 Hz, 1H), 3.76 (s, 2H), 2.58 – 2.40 (m, 2H), 2.31 (s, 3H), 1.60 – 1.50 (m, 2H), 1.39 – 1.28 (m, 4H), 0.98 – 0.86 (m, 3H). ^{13}C NMR (151 MHz, $CDCl_3$) δ 156.4, 155.3, 149.1, 137.2, 136.8, 129.9, 123.5, 123.0, 121.2, 119.2, 63.8, 57.7, 42.6, 29.6, 27.0, 22.6, 14.1 HRMS (EI) m/z: $[M]^+$ calcd for $C_{17}H_{23}N_3$: 269.1892; found, 269.1893.

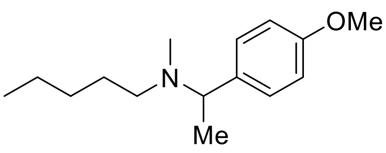

***N*-(1-(4-methoxyphenyl)ethyl)-*N*-methylpentan-1-amine (58):** The title compound was prepared according to the general procedure and purified by column chromatography on silica gel to afford a brown oil in 52% yield (24.5 mg); ^{1}H NMR (600 MHz, $CDCl_3$) δ 7.22 (d, *J* = 8.6 Hz, 2H), 6.90 – 6.72 (m, 2H), 3.80 (s, 3H), 3.54 (d, *J* = 6.5 Hz, 1H), 2.45 – 2.34 (m, 1H), 2.27 – 2.18 (m, 1H), 2.16 (s, 3H), 1.51 – 1.40 (m, 2H), 1.34 (d, *J* = 6.7 Hz, 3H), 1.28 – 1.24 (m, 2H), 1.21 (dt, *J* = 19.7, 6.5 Hz, 2H), 0.86 (t, *J* = 7.2 Hz, 3H). ^{13}C NMR (151 MHz, $CDCl_3$) δ 158.3, 135.8, 128.7, 113.3, 62.5, 55.2, 54.3, 38.4, 29.7, 26.8, 22.6, 18.5, 14.1. HRMS (EI) m/z: $[M]^+$ calcd for $C_{15}H_{25}NO$: 235.1936; found, 235.1938.

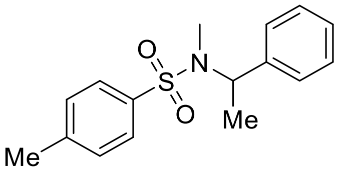

***N*,4-dimethyl-*N*-(1-phenylethyl)benzenesulfonamide (59):** The title compound was prepared according to the general procedure and purified by column chromatography on silica gel to afford a yellow solid (mp 69-70 ºC) in 71% yield (41.1 mg); ^{1}H NMR (400 MHz, $CDCl_3$) δ 7.76 (d, *J* = 8.3 Hz, 2H), 7.35 – 7.30 (m, 6H), 7.29 – 7.26 (m, 1H), 5.31 (q, *J* = 7.0 Hz, 1H), 2.59 (s, 3H), 2.45 (s, 3H), 1.31 (d, *J* = 7.0 Hz, 3H). ^{13}C NMR (101 MHz, $CDCl_3$) δ 143.1, 139.9, 137.2, 129.6, 128.3, 127.5, 127.2, 127.1, 54.7, 28.3, 21.5, 15.2. (*55*)

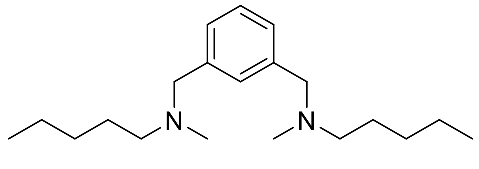

***N*,*N*'-(1,3-phenylenebis(methylene))bis(*N*-methylpentan-1-amine) (60):** The title compound was prepared according to the general procedure and

purified by column chromatography on silica gel to afford a yellow oil in 83% yield (50.5 mg); ^{1}H NMR (600 MHz, $CDCl_3$) δ 7.24 (s, 2H), 7.19 (d, *J* = 7.5 Hz, 2H), 3.47 (s, 4H), 2.42 – 2.29 (m, 4H), 2.18 (s, 6H), 1.58 – 1.46 (m, 4H), 1.28 (ddd, *J* = 15.6, 10.8, 5.3 Hz, 8H), 0.88 (t, *J* = 7.0 Hz, 6H). ^{13}C NMR (151 MHz, $CDCl_3$) δ 138.9, 129.9, 128.0, 127.7, 62.2, 57.6, 42.3, 29.7, 27.0, 22.6, 14.1. HRMS (EI) m/z: $[M]^+$ calcd for $C_{20}H_{36}N_2$: 304.2878; found, 304.2879.

1,3-bis((4-methylpiperazin-1-yl)methyl)benzene (61): The title compound was prepared

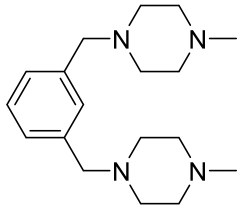

according to the general procedure and purified by column chromatography on silica gel to afford a yellow oil in 76% yield (45.9 mg); ^{1}H NMR (600 MHz, $CDCl_3$) δ 7.22 (d, *J* = 7.3 Hz, 2H), 7.18 (d, *J* = 7.5 Hz, 2H), 3.48 (s, 4H), 2.46 (t, *J* = 58.2 Hz, 16H), 2.26 (s, 6H). ^{13}C NMR (151 MHz, $CDCl_3$) δ 137.9, 130.0, 128.0, 127.9, 62.9, 55.1, 53.0, 46.0. HRMS (EI) m/z: $[M]^+$ calcd for $C_{18}H_{30}N_4$: 302.2470; found, 302.2471.

***N*,*N'*-(pyridine-2,6-diylbis(methylene))bis(*N*-methylpentan-1-amine) (62):** The title compound

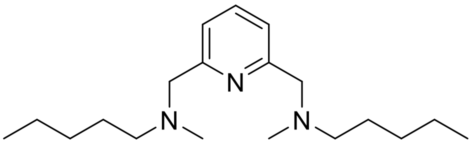

was prepared according to the general procedure and purified by column chromatography on silica gel to afford a yellow oil in 67% yield (40.9 mg); ^{1}H NMR (600 MHz, $CDCl_3$) δ 7.59 (s, 1H), 7.28 (d, *J* = 7.6 Hz, 2H), 3.61 (s, 4H), 2.38 (t, *J* = 7.4 Hz, 4H), 2.22 (s, 6H), 1.64 – 1.40 (m, 4H), 1.36 – 1.16 (m, 8H), 0.86 (t, *J* = 6.6 Hz, 6H). ^{13}C NMR (151 MHz, $CDCl_3$) δ 158.9, 136.6, 120.9, 63.9, 57.9, 42.5, 29.6, 27.0, 22.6, 14.0. HRMS (EI) m/z: $[M]^+$ calcd for $C_{19}H_{35}N_3$: 305.2831; found, 305.2835.

1-(2-chlorophenyl)-2-(isopropyl(4-methylbenzyl)amino)ethan-1-ol (63): The title compound

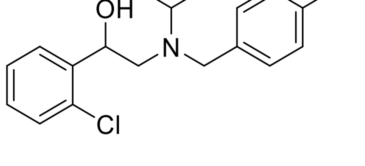

was prepared according to the general procedure and purified by column chromatography on silica gel to afford a yellow oil in 85% yield (54.1 mg); ^{1}H NMR (400 MHz, $CDCl_3$) δ 7.68 (d, *J* = 7.7 Hz, 1H), 7.39 – 7.29 (m, 4H), 7.24 (t, *J* = 7.7 Hz, 3H), 5.03 (dd, *J* = 10.1, 2.6 Hz, 1H), 3.90 (d, *J* = 13.6 Hz, 1H), 3.62 (d, *J* = 13.6 Hz, 1H), 3.12 (dt, *J* = 13.2, 6.6 Hz, 1H), 2.91 (dd, *J* = 12.9, 3.0 Hz, 1H), 2.42 (d, *J* = 6.1 Hz, 4H), 1.18 (d, *J* = 6.7 Hz, 3H), 1.08 (d, *J* = 6.5 Hz, 3H). ^{13}C NMR (101 MHz, $CDCl_3$) δ 140.0, 136.6, 136.5, 131.6, 129.1, 129.0, 128.6, 128.1, 127.2, 127.0, 66.3, 55.3,

54.4, 50.0, 21.1, 20.6, 15.4. IR (neat, ν/cm^{-1}) 3071, 3048, 3017, 2967, 2923, 2843, 1717, 1516, 1439, 1361, 1270, 1161, 1079, 1034, 886, 808, 754, 693, 620. HRMS (EI) m/z: $[M]^+$ calcd for $C_{19}H_{24}ClNO$: 317.1546; found, 317.1536.

***N*-methyl-*N*-(4-methylbenzyl)-2-(pyridin-2-yl)ethan-1-amine (64):** The title compound was

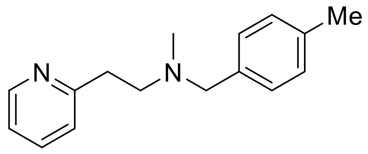

prepared according to the general procedure and purified by column chromatography on silica gel to afford a brown oil in 66% yield (31.7 mg); ^{1}H NMR (400 MHz, $CDCl_3$) δ 8.49 (d, *J* = 4.0 Hz, 1H), 7.55 (t, *J* = 7.6 Hz, 1H), 7.14 (d, *J* = 4.2 Hz, 3H), 7.08 (d, *J* = 7.2 Hz, 3H), 3.52 (s, 2H), 3.03 – 2.96 (m, 2H), 2.80 (t, *J* = 7.6 Hz, 2H), 2.30 (s, 3H), 2.26 (s, 3H). ^{13}C NMR (101 MHz, $CDCl_3$) δ 160.4, 149.0, 136.4, 136.1, 135.5, 128.9, 128.8, 123.1, 121.0, 61.7, 57.0, 41.8, 35.9, 21.0. IR (neat, ν/cm^{-1}) 3050, 3012, 2921, 2792, 1594, 1511, 1429, 1181, 1116, 1048, 984, 796, 749, 689. HRMS (EI) m/z: $[M]^+$ calcd for $C_{16}H_{20}N_2$: 240.1626; found, 240.1629.

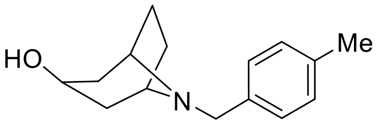

8-(4-methylbenzyl)-8-azabicyclo[3.2.1]octan-3-ol (65): The title compound was prepared according to the general procedure and purified by column chromatography on silica gel to afford a colorless oil in 85% yield (39.3 mg); ^{1}H NMR (400 MHz, $CDCl_3$) δ 7.29 (d, *J* = 8.1 Hz, 2H), 7.16 (d, *J* = 7.8 Hz, 2H), 4.08 (t, *J* = 5.1 Hz, 1H), 3.52 (s, 2H), 3.17 (s, 2H), 2.37 (s, 3H), 2.23 – 1.96 (m, 6H), 1.67 (d, *J* = 14.2 Hz, 2H). ^{13}C NMR (101 MHz, $CDCl_3$) δ 136.9, 136.2, 128.8, 128.5, 65.1, 57.8, 56.3, 39.7, 26.2, 21.0. IR (neat, ν/cm^{-1}) 3265, 3093, 3071, 3044, 2976, 2921, 2853, 1677, 1610, 1511, 1406, 1324, 1246, 1175, 1145, 1094, 1044, 830, 773, 665. HRMS (EI) m/z: $[M]^+$ calcd for $C_{15}H_{21}NO$: 231.1623; found, 231.1622.

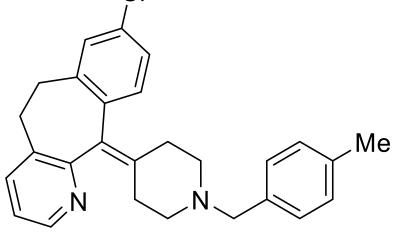

8-chloro-11-(1-(4-methylbenzyl)piperidin-4-ylidene)-6,11-dihydro-5H-benzo[5,6]cyclohepta[1,2-*b*]pyridine (66): The title compound was prepared according to the general procedure and purified by column chromatography on silica gel to afford a red oil in 82% yield (67.9 mg); ^{1}H NMR (400 MHz, $CDCl_3$) δ 8.38 (d, *J* = 4.5 Hz, 1H), 7.39 (d, *J* = 7.6 Hz, 1H), 7.19 (d, *J* = 7.7 Hz, 2H), 7.15 – 7.08 (m, 5H), 7.04 (dd, *J* = 7.3, 5.0 Hz, 1H), 3.48 (s, 2H), 3.44 – 3.26 (m, 2H), 2.86 – 2.66 (m, 4H),

2.58 – 2.49 (m, 1H), 2.48 – 2.39 (m, 1H), 2.34 (d, J = 22.2 Hz, 5H), 2.22 – 2.10 (m, 2H). ^{13}C NMR (101 MHz, $CDCl_3$) δ 157.5, 146.4, 139.3, 138.9, 137.7, 137.1, 136.4, 134.7, 133.2, 132.5, 132.3, 130.7, 129.1, 128.8, 128.7, 125.8, 121.9, 62.4, 54.5, 54.4, 31.7, 31.3, 30.7, 30.5, 21.0. IR (neat, ν/cm^{-1}) 3047, 3015, 2968, 2925, 2850, 2800, 1650, 1587, 1515, 1476, 1438, 1364, 1293, 1175, 1088, 1053, 987, 879, 827, 785, 644. HRMS (EI) m/z: [M]$^+$ calcd for $C_{27}H_{27}ClN_2$: 414.1863; found, 414.1867.

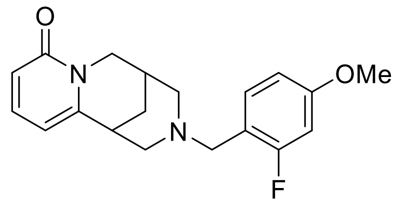

3-(2-fluoro-4-methoxybenzyl)-1,2,3,4,5,6-hexahydro-8H-1,5-methanopyrido[1,2-*a*][1,5]diazocin-8-one (67): The title compound was prepared according to the general procedure and purified by column chromatography on silica gel to afford a brown oil in 90% yield (59.1 mg); ^{1}H NMR (400 MHz, $CDCl_3$) δ 7.31 – 7.20 (m, 1H), 6.83 (t, J = 8.6 Hz, 1H), 6.54 – 6.43 (m, 3H), 5.94 (d, J = 6.8 Hz, 1H), 4.05 (d, J = 15.3 Hz, 1H), 3.87 (dd, J = 15.2, 6.5 Hz, 1H), 3.74 (s, 3H), 3.42 (s, 2H), 2.91 (d, J = 13.0 Hz, 2H), 2.84 (d, J = 10.6 Hz, 1H), 2.37 (dd, J = 20.9, 10.4 Hz, 3H), 1.87 (d, J = 12.5 Hz, 1H), 1.75 (d, J = 12.8 Hz, 1H). ^{13}C NMR (101 MHz, $CDCl_3$) δ 162.6, 160.2, 159.6 (d, J = 11.1 Hz), 151.4, 138.5, 130.9 (d, J = 6.4 Hz), 116.3, 116.0 (d, J = 15.0 Hz), 109.5, 104.6, 101.0 (d, J = 26.1 Hz), 59.7, 59.3, 55.3, 53.6, 49.9, 35.4, 28.0, 25.7. ^{19}F NMR (376 MHz, $CDCl_3$) δ -64.51. IR (neat, ν/cm^{-1}) 3076, 3010, 2930, 2789, 1645, 1539, 1503, 1443, 1282, 1144, 1089, 1025, 942, 800, 731. HRMS (EI) m/z: [M]$^+$ calcd for $C_{19}H_{21}FN_2O_2$: 328.1587; found, 328.1586.

4-((methyl(3-phenyl-3-(4-(trifluoromethyl)phenoxy)propyl)amino)methyl)-*N*,*N*-

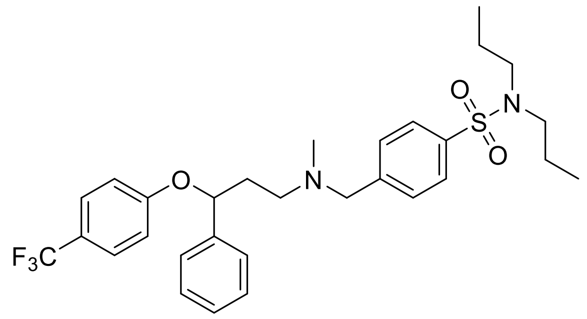

dipropylbenzenesulfonamide (68): The title compound was prepared according to the general procedure and purified by column chromatography on silica gel to afford a yellow oil in 70% yield (78.7 mg); ^{1}H NMR (600 MHz, $CDCl_3$) δ 7.66 (d, J = 8.3 Hz, 2H), 7.44 (d, J = 8.8 Hz, 2H), 7.36 (d, J = 8.2 Hz, 2H), 7.34 – 7.30 (m, 4H), 7.28 – 7.26 (m, 1H), 6.88 (d, J = 8.7 Hz, 2H), 5.33 (dd, J = 8.4, 4.6 Hz, 1H), 3.53 (q, J = 13.7 Hz, 2H), 3.05 (dd, J = 8.5, 6.8 Hz,

4H), 2.63 (dt, J = 12.7, 7.3 Hz, 1H), 2.48 (ddd, J = 12.6, 7.4, 5.3 Hz, 1H), 2.27 – 2.13 (m, 4H), 2.08 – 1.97 (m, 1H), 1.58 – 1.50 (m, 4H), 0.86 (t, J = 7.4 Hz, 6H). ^{13}C NMR (151 MHz, $CDCl_3$) δ 160.6, 144.0, 141.1, 138.7, 129.1, 128.8, 127.8, 127.0, 126.7 (q, J = 3.7 Hz), 125.7, 123.4, 122.7 (q, J = 32.8 Hz), 115.7, 78.1, 61.9, 53.5, 50.0, 42.1, 36.5, 22.0, 11.1. ^{19}F NMR (565 MHz, $CDCl_3$) δ -61.48. HRMS (EI) m/z: $[M]^+$ calcd for $C_{30}H_{37}F_3N_2O_3S$: 562.2477; found, 562.2475.

(3*R*,4*S*)-3-((benzo[d][1,3]dioxol-5-yloxy)methyl)-1-(3-(cyclopropylmethoxy)-4-(difluoromethoxy)benzyl)-4-(4-fluorophenyl)piperidine (69): The title compound was prepared

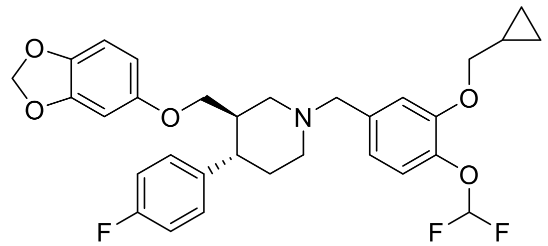

according to the general procedure and purified by column chromatography on silica gel to afford a yellow oil in 75% yield (83.3 mg); ^{1}H NMR (600 MHz, $CDCl_3$) δ 7.18 (dd, J = 8.5, 5.4 Hz, 2H), 7.12 (d, J = 8.1 Hz, 1H), 7.03 (s, 1H), 6.98 (t, J = 8.7 Hz, 2H), 6.90 (dd, J = 8.1, 1.7 Hz, 1H), 6.65 (t, J = 75.8 Hz, 1H), 6.62 (d, J = 8.5 Hz, 1H), 6.33 (d, J = 2.5 Hz, 1H), 6.12 (dd, J = 8.5, 2.5 Hz, 1H), 5.87 (s, 2H), 3.91 (d, J = 6.9 Hz, 2H), 3.63 – 3.54 (m, 2H), 3.52 – 3.41 (m, 2H), 3.24 (dd, J = 11.1, 2.0 Hz, 1H), 2.98 (d, J = 11.2 Hz, 1H), 2.50 (td, J = 11.7, 4.2 Hz, 1H), 2.24 (ddd, J = 18.0, 9.1, 5.5 Hz, 1H), 2.14 – 2.02 (m, 2H), 1.94 – 1.74 (m, 2H), 1.41 – 1.25 (m, 1H), 0.65 (q, J = 5.9 Hz, 2H), 0.38 (q, J = 4.8 Hz, 2H). ^{13}C NMR (151 MHz, $CDCl_3$) δ 162.2, 160.6, 154.2, 150.3, 148.0, 141.4, 139.5 (d, J = 55.9 Hz), 137.2, 128.7 (d, J = 7.6 Hz), 121.8 (d, J = 107.9 Hz), 118.0, 116.3, 115.3 (d, J = 21.0 Hz), 114.7 (d, J = 39.9 Hz), 107.7, 105.4, 101.0, 97.8, 73.7, 69.4, 62.8, 57.5, 53.8, 43.9, 42.0, 34.2, 10.1, 3.1, 3.0. ^{19}F NMR (565 MHz, $CDCl_3$) δ -81.34 (d, J = 75.9 Hz), -116.44. IR (neat, ν/cm^{-1}) 3085, 3062, 3010, 2921, 2803, 2761, 1603, 1509, 1498, 1382, 1270, 1183, 1108, 1021, 930, 829, 780, 610. HRMS (EI) m/z: $[M]^+$ calcd for $C_{31}H_{32}F_3NO_5$: 555.2233; found, 555.2230.

2-((4-((4-chlorophenyl)(phenyl)methyl)piperazin-1-yl)methyl)aniline (70): The title compound

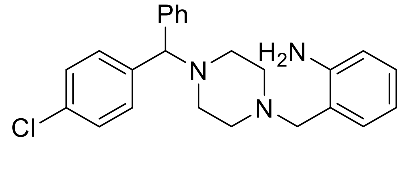

was prepared according to the general procedure and purified by column chromatography on silica gel to afford a yellow solid (mp: 150-151 °C) in 74% yield (57.9 mg); ^{1}H NMR (400 MHz, $CDCl_3$) δ 7.38 (dd, J = 7.6, 5.3 Hz, 4H), 7.33 – 7.25 (m, 4H), 7.21 (t, J = 7.2 Hz, 1H), 7.13 –

7.06 (m, 1H), 7.00 (d, J = 7.3 Hz, 1H), 6.77 – 6.57 (m, 2H), 4.26 (d, J = 20.9 Hz, 3H), 3.53 (s, 2H), 2.67 – 2.16 (m, 8H). ^{13}C NMR (101 MHz, $CDCl_3$) δ 146.8, 142.0, 141.2, 132.4, 130.3, 129.1, 128.6, 128.5, 128.2, 127.8, 127.1, 122.2, 117.5, 115.4, 75.3, 61.9, 52.9, 51.9. IR (neat, ν/cm^{-1}) 3290, 3024, 2963, 2942, 2810, 1610, 1493, 1455, 1276, 1105, 1091, 1001, 934, 844, 750, 696, 670, 616. HRMS (EI) m/z: [M]$^+$ calcd for $C_{24}H_{26}ClN_3$: 391.1815; found, 391.1816.

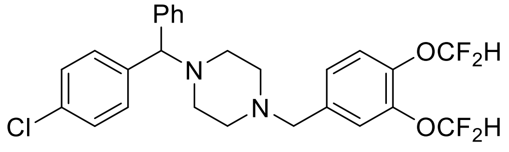

1-(3,4-bis(difluoromethoxy)benzyl)-4-((4-chlorophenyl)(phenyl)methyl)piperazine (71): The title compound was prepared according to the general procedure and purified by column chromatography on silica gel to afford a yellow oil in 81% yield (82.3 mg); ^{1}H NMR (400 MHz, $CDCl_3$) δ 7.40 (dd, J = 7.7, 4.2 Hz, 4H), 7.29 (dd, J = 16.1, 7.8 Hz, 5H), 7.24 – 7.18 (m, 3H), 6.54 (td, J = 73.8, 8.9 Hz, 2H), 4.26 (s, 1H), 3.51 (s, 2H), 2.47 (d, J = 16.7 Hz, 8H). ^{13}C NMR (101 MHz, $CDCl_3$) δ 142.2 (t, J = 2.8 Hz), 142.1, 141.3, 141.0 (t, J = 2.8 Hz), 137.7, 132.5, 129.2, 128.6 (d, J = 6.1 Hz), 127.8, 127.1, 126.8, 122.5, 122.0, 118.5, 115.9, 113.3. 75.4, 61.8, 53.2, 51.8. ^{19}F NMR (376 MHz, $CDCl_3$) δ -81.14 (d, J = 19.5 Hz), -81.33 (d, J = 19.6 Hz). IR (neat, ν/cm^{-1}) 3086, 3064, 3027, 2961, 2933, 2815, 2766, 1669, 1597, 1507, 1482, 1378, 1267, 1126, 1039, 1004, 844, 800, 755, 699, 621. HRMS (EI) m/z: [M]$^+$ calcd for $C_{26}H_{25}ClF_4N_2O_2$: 508.1541; found, 508.1539.

1-(4-bromo-2,5-dimethoxybenzyl)-4-((4-chlorophenyl)(phenyl)methyl)piperazine (72): The

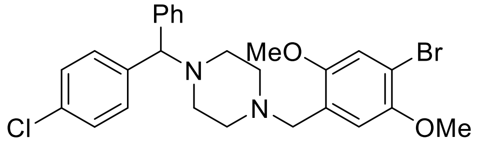

title compound was prepared according to the general procedure and purified by column chromatography on silica gel to afford a yellow oil in 83% yield (85.3 mg); ^{1}H NMR (400 MHz, $CDCl_3$) δ 7.37 (dd, J = 7.6, 4.7 Hz, 4H), 7.30 – 7.22 (m, 4H), 7.18 (t, J = 7.2 Hz, 1H), 7.04 (s, 1H), 6.99 (s, 1H), 4.21 (s, 1H), 3.84 (s, 3H), 3.75 (s, 3H), 3.53 (s, 2H), 2.47 (d, J = 39.5 Hz, 8H). ^{13}C NMR (101 MHz, $CDCl_3$) δ 152.1, 149.9, 142.2, 141.4, 132.4, 129.1, 128.6, 128.5, 127.8, 127.0, 126.4, 115.9, 114.5, 109.5, 75.5, 56.8, 56.2, 55.5, 53.1, 51.8. IR (neat, ν/cm^{-1}) 3055, 3017, 2964, 2825, 2806, 2766, 1597, 1493, 1317, 1205, 1131, 1006, 902, 853, 797, 759, 719, 663. HRMS (EI) m/z: [M]$^+$ calcd for $C_{26}H_{28}BrClN_2O_2$: 514.1023; found, 514.1022.

1-((4-chlorophenyl)(phenyl)methyl)-4-(2,4-dichloro-5-fluorobenzyl)piperazine (73): The title

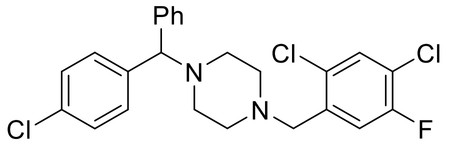

compound was prepared according to the general procedure and purified by column chromatography on silica gel to afford a yellow oil in 69% yield (63.8 mg); ^{1}H NMR (400 MHz, $CDCl_3$) δ 7.40 (dd, *J* = 11.3, 5.3 Hz, 6H), 7.30 (dd, *J* = 17.0, 7.9 Hz, 4H), 7.23 (t, *J* = 7.2 Hz, 1H), 4.27 (s, 1H), 3.59 (s, 2H), 2.57 (s, 4H), 2.47 (s, 4H). ^{13}C NMR (101 MHz, $CDCl_3$) δ 156.9 (d, *J* = 248.4 Hz), 142.1, 141.3, 137.1 (d, *J* = 6.3 Hz), 132.5, 130.6, 129.2, 128.8 (d, *J* = 3.5 Hz), 128.6, 128.6, 127.8, 127.1, 119.7 (d, *J* = 19.3 Hz), 117.7 (d, *J* = 23.2 Hz). 75.4, 58.4, 53.3, 51.8. ^{19}F NMR (376 MHz, $CDCl_3$) δ -117.76. HRMS (EI) m/z: $[M]^+$ calcd for $C_{24}H_{22}Cl_3FN_2$: 462.0833; found, 462.0835.

1-((4-chlorophenyl)(phenyl)methyl)-4-((6-chloropyridin-2-yl)methyl)piperazine (74): The title

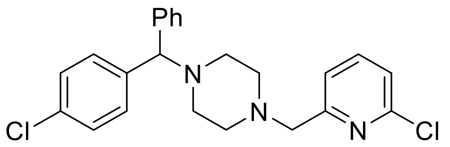

compound was prepared according to the general procedure and purified by column chromatography on silica gel to afford a brown oil in 65% yield (53.4 mg); ^{1}H NMR (400 MHz, $CDCl_3$) δ 7.59 (t, *J* = 7.7 Hz, 1H), 7.42 – 7.35 (m, 5H), 7.28 (dd, *J* = 17.4, 7.9 Hz, 4H), 7.20 (t, *J* = 7.5 Hz, 2H), 4.25 (s, 1H), 3.67 (s, 2H), 2.51 (d, *J* = 46.5 Hz, 8H). ^{13}C NMR (101 MHz, $CDCl_3$) δ 159.8, 150.5, 142.0, 141.2, 138.9, 132.4, 129.1, 128.5, 128.5, 127.7, 127.0, 122.3, 121.3, 75.3, 63.6, 53.3, 51.7. IR (neat, ν/cm^{-1}) 3086, 3062, 3024, 2964, 2925, 2878, 2808, 1585, 1559, 1487, 1440, 1408, 1331, 1295, 1160, 1139, 1084, 1006, 851, 800, 757, 696, 623. HRMS (EI) m/z: $[M]^+$ calcd for $C_{23}H_{23}Cl_2N_3$: 411.1269; found, 411.1270.

1-((5-bromopyridin-2-yl)methyl)-4-((4-chlorophenyl)(phenyl)methyl)piperazine (75): The title

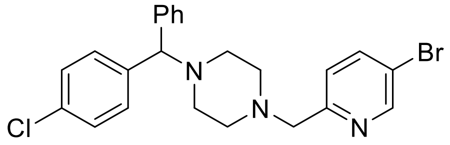

compound was prepared according to the general procedure and purified by column chromatography on silica gel to afford a brown oil in 74% yield (67.4 mg); ^{1}H NMR (400 MHz, $CDCl_3$) δ 8.63 (d, *J* = 1.9 Hz, 1H), 7.76 (dd, *J* = 8.3, 2.0 Hz, 1H), 7.38 (dd, *J* = 7.6, 4.4 Hz, 4H), 7.28 (dt, *J* = 17.1, 8.7 Hz, 5H), 7.20 (t, *J* = 7.2 Hz, 1H), 4.25 (s, 1H), 3.65 (s, 2H), 2.50 (d, *J* = 40.1 Hz, 8H). ^{13}C NMR (101 MHz, $CDCl_3$) δ 157.0, 150.1, 142.0, 141.2, 138.9, 132.4, 129.1, 128.5, 128.5, 127.7, 127.0, 124.4, 118.9, 75.3, 63.5, 53.3, 51.5. HRMS (EI) m/z: $[M]^+$

calcd for $C_{23}H_{23}BrClN_3$: 455.0764; found, 455.0763.

1-((5-bromo-2-methoxypyridin-3-yl)methyl)-4-((4-chlorophenyl)(phenyl)methyl)piperazine

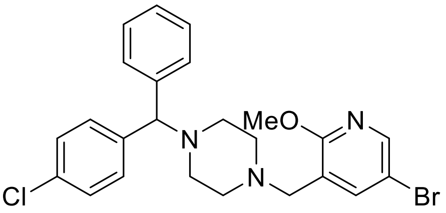

(76): The title compound was prepared according to the general procedure and purified by column chromatography on silica gel to afford a yellow oil in 76% yield (73.7 mg); ^{1}H NMR (400 MHz, $CDCl_3$) δ 8.07 (d, *J* = 2.3 Hz, 1H), 7.73 (d, *J* = 2.1 Hz, 1H), 7.36 (dd, *J* = 7.8, 3.9 Hz, 4H), 7.29 – 7.22 (m, 4H), 7.18 (t, *J* = 7.2 Hz, 1H), 4.22 (s, 1H), 3.91 (s, 3H), 3.48 (s, 2H), 2.48 (d, *J* = 35.0 Hz, 8H). ^{13}C NMR (101 MHz, $CDCl_3$) δ 160.9, 145.4, 142.1, 141.3, 140.2, 132.5, 129.1, 128.6, 128.5, 127.8, 127.1, 122.8, 111.8, 75.4, 55.1, 53.7, 53.3, 51.8. IR (neat, ν/cm^{-1}) 3085, 3059, 3064, 2953, 2810, 1570, 1488, 1458, 1416, 1382, 1347, 1302, 1242, 1135, 1084, 1009, 883, 855, 799, 754, 696, 649. HRMS (EI) m/z: $[M]^+$ calcd for $C_{24}H_{25}BrClN_3O$: 485.0870; found, 485.0869.

5-((4-((4-chlorophenyl)(phenyl)methyl)piperazin-1-yl)methyl)pyrimidine (77): The title compound was prepared according to the general procedure and purified by column chromatography on silica gel to afford a yellow oil in 66% yield (49.9 mg); ^{1}H NMR (400 MHz, $CDCl_3$) δ 9.10 (s, 1H), 8.65 (s, 2H), 7.33 (dd, *J* = 7.8, 4.0 Hz, 4H), 7.28 – 7.13 (m, 5H), 4.20 (s, 1H), 3.49 (s, 2H), 2.42 (d, *J* = 28.0 Hz, 8H). ^{13}C NMR (101 MHz, $CDCl_3$) δ 157.7, 157.3, 141.9, 141.1, 132.5, 131.3, 129.0, 128.6, 128.5, 127.7, 127.1, 75.2, 57.5, 53.1, 51.5. IR (neat, ν/cm^{-1}) 3085, 3059, 3024, 2972, 2928, 2820, 1658, 1564, 1483, 1448, 1404, 1294, 1131, 1084, 1046, 1001, 847, 802, 719, 701, 632. HRMS (EI) m/z: $[M]^+$ calcd for $C_{22}H_{23}ClN_4$: 378.1611; found, 378.1613.

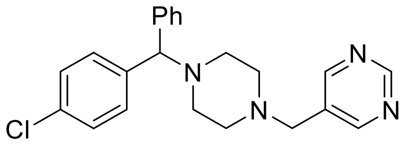

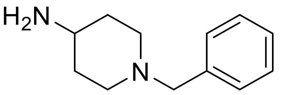

1-benzylpiperidin-4-amine (78): The title compound was prepared according to the general procedure and purified by column chromatography on silica gel to afford a yellow oil in 76% yield (28.9 mg); ^{1}H NMR (400 MHz, $CDCl_3$) δ 7.28 (d, *J* = 4.3 Hz, 4H), 7.22 (dd, *J* = 8.3, 3.9 Hz, 1H), 3.46 (s, 2H), 2.80 (d, *J* = 11.2 Hz, 2H), 2.69 – 2.55 (m, 1H), 1.99 (t, *J* = 11.5 Hz, 2H), 1.75 (d, *J* = 12.4 Hz, 2H), 1.53 (s, 2H), 1.42 – 1.29 (m, 2H). ^{13}C NMR (101 MHz, $CDCl_3$) δ 138.4, 129.0, 128.0, 126.8, 63.0, 52.3, 48.7, 35.9. (*13*)

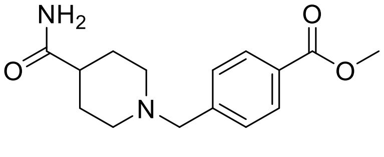

methyl 4-((4-carbamoylpiperidin-1-yl)methyl)benzoate (79): The title compound was prepared according to the general procedure and purified by column chromatography on silica gel to afford a yellow solid (mp: 131-132 ºC) in 81% yield (44.7 mg); ^{1}H NMR (400 MHz, $CDCl_3$) δ 7.95 (d, J = 8.1 Hz, 2H), 7.37 (d, J = 8.1 Hz, 2H), 6.01 (s, 1H), 5.73 (s, 1H), 3.88 (s, 3H), 3.53 (s, 2H), 2.89 (d, J = 11.5 Hz, 2H), 2.19 – 2.09 (m, 1H), 2.01 (dd, J = 16.2, 6.5 Hz, 2H), 1.88 – 1.67 (m, 4H). ^{13}C NMR (101 MHz, $CDCl_3$) δ 177.7, 167.0, 143.5 129.5, 128.9, 128.8, 62.5, 52.9, 52.0, 42.4, 28.7. HRMS (EI) m/z: $[M]^+$ calcd for $C_{15}H_{20}N_2O_3$: 276.1474; found, 276.1475.

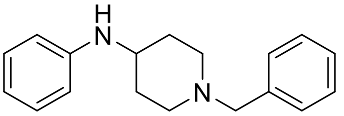

1-benzyl-N-phenylpiperidin-4-amine (80): The title compound was prepared according to the general procedure and purified by column chromatography on silica gel to afford a brown oil in 85% yield (45.2 mg); ^{1}H NMR (400 MHz, $CDCl_3$) δ 7.36 (d, J = 4.3 Hz, 4H), 7.32 – 7.26 (m, 1H), 7.19 (t, J = 7.8 Hz, 2H), 6.70 (t, J = 7.3 Hz, 1H), 6.62 (d, J = 8.1 Hz, 2H), 3.56 (s, 2H), 3.33 (ddd, J = 13.9, 9.8, 3.9 Hz, 1H), 2.88 (d, J = 11.7 Hz, 2H), 2.18 (t, J = 11.4 Hz, 2H), 2.06 (d, J = 12.2 Hz, 2H), 1.51 (td, J = 13.6, 3.5 Hz, 2H). ^{13}C NMR (101 MHz, $CDCl_3$) δ 147.1, 138.2, 129.2, 129.1, 128.2, 127.0, 117.1, 113.2, 63.1, 52.3, 49.9, 32.5. IR (neat, ν/cm^{-1}) 3375, 3079, 3050, 3027, 2930, 2846, 1597, 1505, 1371, 1314, 1260, 1145, 1120, 1098, 1070, 1023, 860, 748, 691. HRMS (EI) m/z: $[M]^+$ calcd for $C_{18}H_{22}N_2$: 266.1783; found, 266.1785.

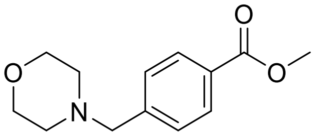

methyl 4-(morpholinomethyl)benzoate (81): The title compound was prepared according to the general procedure and purified by column chromatography on silica gel to afford a yellow oil in 70% yield (32.9 mg); ^{1}H NMR (400 MHz, $CDCl_3$) δ 7.98 (d, J = 8.0 Hz, 2H), 7.40 (d, J = 8.0 Hz, 2H), 3.90 (s, 3H), 3.74 – 3.66 (m, 4H), 3.54 (s, 2H), 2.53 – 2.33 (m, 4H). ^{13}C NMR (101 MHz, $CDCl_3$) δ 167.0, 143.3, 129.6, 129.1, 128.9, 67.0, 63.0, 53.7, 52.0.

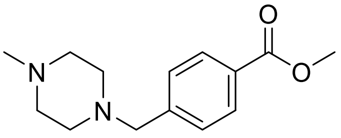

methyl 4-((4-methylpiperazin-1-yl)methyl)benzoate (82): The title compound was prepared according to the general procedure and purified by column chromatography on silica gel to afford a yellow oil in 74% yield (36.7 mg); ^{1}H NMR (400 MHz, $CDCl_3$) δ 7.98 (t, J = 9.1 Hz, 2H), 7.40 (t,

J = 7.7 Hz, 2H), 3.90 (s, 3H), 3.54 (s, 2H), 2.47 (s, 8H), 2.29 (s, 3H). ^{13}C NMR (101 MHz, $CDCl_3$) δ 167.0, 143.8, 129.5, 129.0, 128.9, 62.5, 55.0, 53.0, 52.0, 45.9.

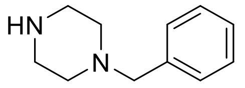

1-benzylpiperazine (83): The title compound was prepared according to the general procedure and purified by column chromatography on silica gel to afford a yellow oil in 79% yield (27.8 mg); ^{1}H NMR (400 MHz, $CDCl_3$) δ 7.30 – 7.27 (m, 4H), 7.21 (ddd, J = 10.9, 5.2, 2.8 Hz, 1H), 3.46 (s, 2H), 2.85 (t, J = 4.8 Hz, 4H), 2.38 (s, 4H), 2.10 (s, 1H). ^{13}C NMR (101 MHz, $CDCl_3$) δ 137.9, 129.1, 128.0, 126.9, 63.5, 54.3, 45.9. HRMS (EI) m/z: $[M]^+$ calcd for $C_{11}H_{16}N_2$: 176.1313; found, 176.1312.

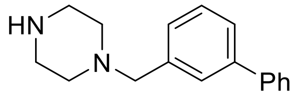

1-([1,1'-biphenyl]-3-ylmethyl)piperazine (84): The title compound was prepared according to the general procedure and purified by column chromatography on silica gel to afford a yellow oil in 75% yield (37.8 mg); ^{1}H NMR (400 MHz, $CDCl_3$) δ 7.64 – 7.55 (m, 3H), 7.49 (d, J = 7.6 Hz, 1H), 7.46 – 7.29 (m, 5H), 3.54 (s, 2H), 2.88 (t, J = 4.6 Hz, 4H), 2.55 (s, 1H), 2.44 (s, 4H). ^{13}C NMR (101 MHz, $CDCl_3$) δ 140.8, 140.8, 138.3, 128.4, 128.4, 127.9, 127.6, 127.0, 126.9, 125.6, 63.4, 54.0, 45.6. IR (neat, ν/cm^{-1}) 3394, 3060, 3029, 2945, 2802, 1649, 1595, 1482, 1455, 1343, 1279, 1135, 1046, 1001, 898, 804, 755, 696, 612. HRMS (EI) m/z: $[M]^+$ calcd for $C_{17}H_{20}N_2$: 252.1626; found, 252.1626.

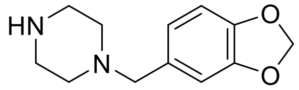

1-(benzo[*d*][1,3]dioxol-5-ylmethyl)piperazine (85): The title compound was prepared according to the general procedure and purified by column chromatography on silica gel to afford a yellow oil in 81% yield (35.6 mg); ^{1}H NMR (400 MHz, $CDCl_3$) δ 6.78 (s, 1H), 6.74 – 6.64 (m, 2H), 5.90 (s, 2H), 5.81 – 5.63 (m, 1H), 3.40 (s, 2H), 3.23 – 2.93 (m, 4H), 2.80 – 2.53 (m, 4H). ^{13}C NMR (101 MHz, $CDCl_3$) δ 147.6, 146.7, 131.0, 122.1, 109.2, 107.8, 100.9, 62.4, 50.9, 44.3. (*6*)

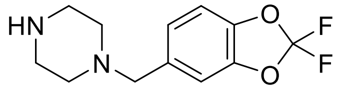

1-((2,2-difluorobenzo[*d*][1,3]dioxol-5-yl)methyl)piperazine (86): The title compound was prepared according to the general procedure and purified by column chromatography on silica gel to afford a yellow oil in 70% yield (35.9 mg); ^{1}H NMR (400 MHz, $CDCl_3$) δ 7.04 (s, 1H), 6.91 (q, J = 8.2 Hz, 2H), 3.38 (s, 2H), 2.90 (s, 1H), 2.82 (t, J = 4.6 Hz, 4H), 2.34 (s, 4H). ^{13}C NMR (101 MHz, $CDCl_3$) δ 143.1 (d, J = 113.9

Hz), 134.4, 134.0, 131.5, 128.9, 123.8, 109.3 (d, J = 128.7 Hz), 62.8, 53.8, 45.6. ^{19}F NMR (376 MHz, $CDCl_3$) δ -50.05. HRMS (EI) m/z: $[M]^+$ calcd for $C_{12}H_{14}F_2N_2O_2$: 256.1023; found, 256.1023.

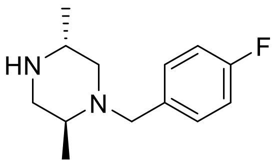

(2*S*,5*R*)-1-(4-fluorobenzyl)-2,5-dimethylpiperazine (87): The title compound was prepared according to the general procedure and purified by column chromatography on silica gel to afford a yellow solid (mp: 96-97 ^{o}C) in 69% yield (30.6 mg); ^{1}H NMR (600 MHz, $CDCl_3$) δ 7.27 (d, J = 7.6 Hz, 2H), 6.99 (t, J = 8.2 Hz, 2H), 4.05 (d, J = 13.4 Hz, 1H), 3.04 (d, J = 13.4 Hz, 1H), 2.93 (d, J = 12.1 Hz, 1H), 2.80 (d, J = 1.9 Hz, 1H), 2.63 (t, J = 10.6 Hz, 2H), 2.55 (s, 1H), 2.26 (s, 1H), 1.65 (t, J = 10.8 Hz, 1H), 1.13 (d, J = 5.8 Hz, 3H), 0.96 (d, J = 6.1 Hz, 3H). ^{13}C NMR (151 MHz, $CDCl_3$) δ 161.8 (d, J = 244.5 Hz), 134.3 (d, J = 2.9 Hz), 130.4 (d, J = 7.9 Hz), 114.9 (d, J = 21.1 Hz), 59.7, 57.2, 55.9, 53.5, 50.8, 19.5, 17.7. ^{19}F NMR (565 MHz, $CDCl_3$) δ -116.23. HRMS (EI) m/z: $[M]^+$ calcd for $C_{13}H_{19}FN_2$: 222.1532; found, 222.1532.

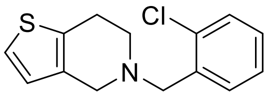

Ticlopidine (88): The title compound was prepared according to the general procedure and purified by column chromatography on silica gel to afford a yellow oil in 81% yield (42.6 mg); ^{1}H NMR (400 MHz, $CDCl_3$) δ 7.54 (d, J = 7.2 Hz, 1H), 7.36 (d, J = 7.7 Hz, 1H), 7.25 – 7.16 (m, 2H), 7.06 (d, J = 5.1 Hz, 1H), 6.70 (d, J = 5.1 Hz, 1H), 3.82 (s, 2H), 3.63 (s, 2H), 2.87 (dt, J = 7.9, 3.8 Hz, 4H). ^{13}C NMR (101 MHz, $CDCl_3$) δ 136.1, 134.2, 133.9, 133.4, 130.6, 129.4, 128.2, 126.7, 125.2, 122.6, 58.4, 53.1, 50.7, 25.5. IR (neat, ν/cm^{-1}) 3091, 3064, 2918, 2846, 1645, 1594, 1482, 1444, 1338, 1195, 1100, 1048, 962, 757, 703, 646. HRMS (EI) m/z: $[M]^+$ calcd for $C_{14}H_{14}ClNS$: 263.0535; found, 263.0533.

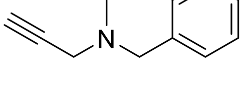

Pargyline (89): The title compound was prepared according to the general procedure and purified by column chromatography on silica gel to afford a yellow oil in 67% yield (21.3 mg); ^{1}H NMR (400 MHz, $CDCl_3$) δ 7.37 – 7.29 (m, 4H), 7.29 – 7.26 (m, 1H), 3.58 (s, 2H), 3.31 (d, J = 2.3 Hz, 2H), 2.35 (s, 3H), 2.27 (t, J = 2.4 Hz, 1H). ^{13}C NMR (101 MHz, $CDCl_3$) δ 138.3, 129.2, 128.3, 127.2, 78.5, 73.3, 60.0, 44.8, 41.7. HRMS (EI) m/z: $[M]^+$ calcd for $C_{11}H_{13}N$:159.1048; found, 159.1047.

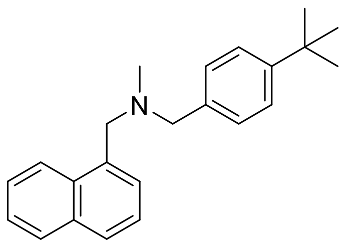

Butenafine (90): The title compound was prepared according to the general procedure and purified by column chromatography on silica gel to afford a yellow oil in 94% yield (59.6 mg); ^{1}H NMR (400 MHz, $CDCl_3$) δ 8.33 (d, *J* = 8.7 Hz, 1H), 7.94 – 7.87 (m, 1H), 7.83 (d, *J* = 8.1 Hz, 1H), 7.60 – 7.51 (m, 3H), 7.47 (t, *J* = 7.6 Hz, 1H), 7.42 (d, *J* = 8.3 Hz, 2H), 7.37 (d, *J* = 8.0 Hz, 2H), 4.01 (s, 2H), 3.66 (s, 2H), 2.29 (s, 3H), 1.40 (s, 9H). ^{13}C NMR (101 MHz, $CDCl_3$) δ 149.8, 136.3, 135.1, 133.9, 132.6, 129.6, 128.8, 128.3, 127.8, 127.3, 125.6, 125.5, 125.0, 124.9, 62.0, 60.4, 42.4, 34.4, 31.4. HRMS (EI) m/z: $[M]^+$ calcd for $C_{23}H_{27}N$: 317.2143; found, 317.2142.

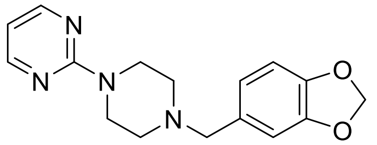

Piribedil (91): The title compound was prepared according to the general procedure and purified by column chromatography on silica gel to afford a brown oil in 86% yield (51.3 mg); ^{1}H NMR (400 MHz, $CDCl_3$) δ 8.26 (d, *J* = 4.7 Hz, 2H), 6.86 (s, 1H), 6.73 (s, 2H), 6.43 (t, *J* = 4.7 Hz, 1H), 5.91 (s, 2H), 3.85 – 3.73 (m, 4H), 3.42 (s, 2H), 2.47 – 2.43 (m, 4H). ^{13}C NMR (101 MHz, $CDCl_3$) δ 161.5, 157.5, 147.6, 146.6, 131.7, 122.1, 109.6, 109.4, 107.8, 100.8, 62.7, 52.7, 43.6. HRMS (EI) m/z: $[M]^+$ calcd for $C_{16}H_{18}N_4O_2$: 298.1430; found, 298.1426.

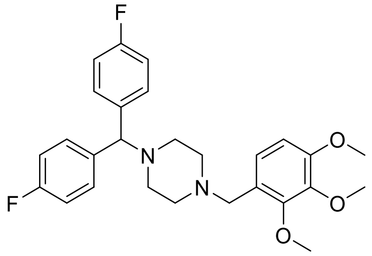

Lomerizine (92): The title compound was prepared according to the general procedure and purified by column chromatography on silica gel to afford a yellow oil in 73% yield (68.4 mg); ^{1}H NMR (400 MHz, $CDCl_3$) δ 7.37 – 7.29 (m, 4H), 6.94 (t, *J* = 7.6 Hz, 5H), 6.61 (d, *J* = 8.4 Hz, 1H), 4.20 (s, 1H), 3.89 – 3.80 (m, 9H), 3.49 (s, 2H), 2.43 (d, *J* = 47.7 Hz, 8H). ^{13}C NMR (101 MHz, $CDCl_3$) δ 162.9, 160.4, 152.7 (d, *J* = 23.5 Hz), 142.2, 138.3 (d, *J* = 3.0 Hz), 129.2 (d, *J* = 7.8 Hz), 125.1, 123.8, 115.2 (d, *J* = 21.2 Hz), 106.9, 74.5, 61.1, 60.7, 56.3, 55.9, 53.0, 51.8. ^{19}F NMR (376 MHz, $CDCl_3$) δ -115.73. HRMS (EI) m/z: $[M]^+$ calcd for $C_{27}H_{30}F_2N_2O_3$: 468.2224; found, 468.2227.

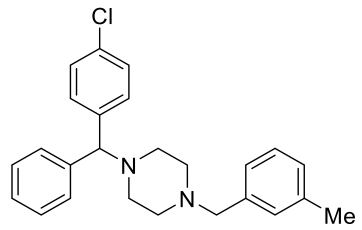

Meclozine (93): The title compound was prepared according to the general procedure and purified by column chromatography on silica gel to afford a yellow oil in 88% yield (68.6 mg); ^{1}H NMR (400 MHz, $CDCl_3$) δ 7.44 (t, *J* = 6.5 Hz, 4H), 7.33 (dd, *J* = 16.2, 7.8 Hz, 4H), 7.28 – 7.24 (m, 2H), 7.22 – 7.09 (m, 3H), 4.30 (s, 1H), 3.57 (s, 2H), 2.54 (d, *J* = 22.4 Hz, 8H), 2.41 (s, 3H). ^{13}C NMR (101 MHz, $CDCl_3$) δ 142.1, 141.3, 137.7, 137.7, 132.4, 129.9, 129.1, 128.5, 128.4, 128.0, 127.8, 127.7, 127.0, 126.3, 75.4, 63.0, 53.2, 51.7, 21.3. IR (neat, ν/cm^{-1}) 3069, 3036, 2967, 2930, 2825, 1589, 1501, 1463, 1418, 1279, 1222, 1150, 1094, 1044, 1004, 825, 788, 693. HRMS (EI) m/z: [M]$^+$ calcd for $C_{25}H_{27}ClN_2$: 390.1863; found, 390.1862.

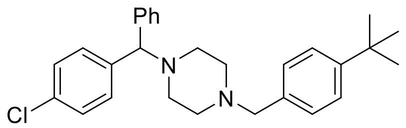

Buclizine (94): The title compound was prepared according to the general procedure and purified by column chromatography on silica gel to afford a yellow oil in 85% yield (73.5 mg); ^{1}H NMR (400 MHz, $CDCl_3$) δ 7.43 – 7.34 (m, 6H), 7.33 – 7.25 (m, 6H), 7.22 (t, *J* = 7.3 Hz, 1H), 4.26 (s, 1H), 3.53 (s, 2H), 2.49 (d, *J* = 26.6 Hz, 8H), 1.35 (s, 9H). ^{13}C NMR (101 MHz, $CDCl_3$) δ 149.8, 142.2, 141.4, 134.9, 132.4, 129.2, 128.9, 128.5, 128.5, 127.9, 127.0, 125.0, 75.4, 62.6, 53.3, 51.8, 34.4, 31.4. IR (neat, ν/cm^{-1}) 3083, 3057, 3022, 2925, 2806, 2763, 1667, 1604, 1488, 1451, 1289, 1144, 1084, 1006, 855, 801, 756, 696, 623. HRMS (EI) m/z: [M]$^+$ calcd for $C_{28}H_{33}ClN_2$: 432.2332; found, 432.2332.

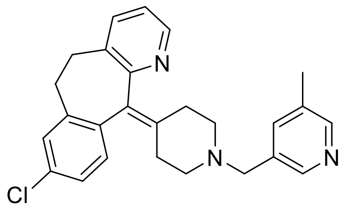

Rupatadine (95): The title compound was prepared according to the general procedure and purified by column chromatography on silica gel to afford a yellow solid (mp: 59-60 °C) in 67% yield (55.6 mg); ^{1}H NMR (600 MHz, $CDCl_3$) δ 8.36 (dd, *J* = 4.7, 1.4 Hz, 1H), 8.29 (s, 2H), 7.45 (s, 1H), 7.39 (dd, *J* = 7.6, 1.2 Hz, 1H), 7.12 – 7.06 (m, 3H), 7.04 (dd, *J* = 7.6, 4.8 Hz, 1H), 3.43 (s, 2H), 3.40 – 3.29 (m, 2H), 2.84 – 2.73 (m, 2H), 2.72 – 2.64 (m, 2H), 2.49 (ddd, *J* = 14.2, 10.1, 4.3 Hz, 1H), 2.40 (ddd, *J* = 14.0, 9.9, 4.3 Hz, 1H), 2.35 – 2.30 (m, 2H), 2.29 (s, 3H), 2.12 (qd, *J* = 10.9, 3.2 Hz, 2H). ^{13}C NMR (151 MHz, $CDCl_3$) δ 157.5, 149.0, 147.5, 146.5, 139.4, 138.6, 137.7, 137.1, 133.3, 133.0, 132.6, 132.5, 130.7, 128.8, 125.9, 122.0. 59.9, 54.7, 54.6, 31.7, 31.3, 30.8, 30.6, 18.2. HRMS (EI) m/z: [M]$^+$ calcd for $C_{26}H_{26}ClN_3$: 415.1815; found, 415.1814.

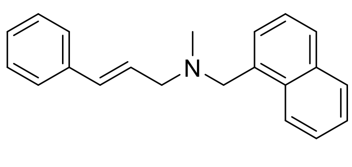

Naftifine (96): The title compound was prepared according to the general procedure and purified by column chromatography on silica gel to afford a yellow oil in 87% yield (50.1 mg); ^{1}H NMR (400 MHz, $CDCl_3$) δ 8.69 (d, *J* = 8.3 Hz, 1H), 8.21 (d, *J* = 7.7 Hz, 1H), 8.14 (d, *J* = 8.0 Hz, 1H), 7.94 – 7.82 (m, 3H), 7.79 – 7.74 (m, 3H), 7.68 (t, *J* = 7.5 Hz, 2H), 7.58 (dd, *J* = 12.9, 5.7 Hz, 1H), 6.95 (d, *J* = 15.9 Hz, 1H), 6.75 (dt, *J* = 15.9, 6.6 Hz, 1H), 4.32 (s, 2H), 3.65 (d, *J* = 6.6 Hz, 2H), 2.65 (s, 3H). ^{13}C NMR (101 MHz, $CDCl_3$) δ 137.1, 134.8, 133.9, 132.6, 132.5, 128.5, 128.4, 127.9, 127.5, 127.4, 127.3, 126.3, 125.8, 125.5, 125.1, 124.6, 60.3, 60.0, 42.4. IR (neat, ν/cm^{-1}) 3059, 3027, 2940, 2831, 2783, 1600, 1507, 1446, 1361, 1230, 1121, 1011, 966, 772, 738, 686. HRMS (EI) m/z: $[M]^+$ calcd for $C_{21}H_{21}N$: 287.1674; found, 287.1680.

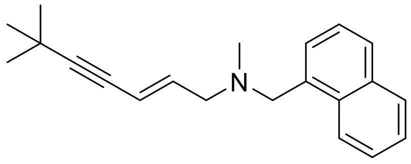

Terbinafine (97): The title compound was prepared according to the general procedure and purified by column chromatography on silica gel to afford a yellow oil in 92% yield (53.6 mg); ^{1}H NMR (400 MHz, $CDCl_3$) δ 8.29 (d, *J* = 8.2 Hz, 1H), 7.87 (d, *J* = 7.7 Hz, 1H), 7.79 (d, *J* = 7.5 Hz, 1H), 7.57 – 7.47 (m, 2H), 7.47 – 7.38 (m, 2H), 6.38 – 6.05 (m, 1H), 5.71 (d, *J* = 15.9 Hz, 1H), 3.92 (s, 2H), 3.16 (d, *J* = 6.6 Hz, 2H), 2.25 (s, 3H), 1.27 (s, 9H). ^{13}C NMR (101 MHz, $CDCl_3$) δ 139.2, 134.7, 133.8, 132.4, 128.4, 127.9, 127.2, 125.8, 125.5, 125.1, 124.6, 112.8, 98.4, 77.2, 60.0, 59.6, 42.3, 31.0, 27.9. IR (neat, ν/cm^{-1}) 3069, 3048, 2960, 2865, 2838, 2782, 2218, 1594, 1507, 1453, 1359, 1262, 1196, 1124, 1013, 957, 776, 644. HRMS (EI) m/z: $[M]^+$ calcd for $C_{21}H_{25}N$: 291.1987; found, 291.1990.

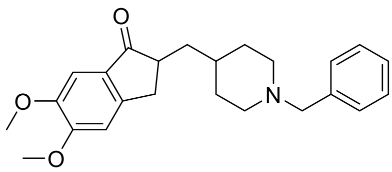

Donepezil (98): The title compound was prepared according to the general procedure and purified by column chromatography on silica gel to afford a yellow oil in 83% yield (62.9 mg); ^{1}H NMR (400 MHz, $CDCl_3$) δ 7.31 – 7.27 (m, 4H), 7.24 – 7.19 (m, 1H), 7.14 (s, 1H), 6.83 (s, 1H), 3.93 (s, 3H), 3.87 (s, 3H), 3.48 (s, 2H), 3.20 (dd, *J* = 17.6, 8.1 Hz, 1H), 2.94 – 2.80 (m, 2H), 2.71 – 2.63 (m, 2H), 2.03 – 1.82 (m, 3H), 1.74 – 1.60 (m, 2H), 1.53 – 1.42 (m, 1H), 1.37 – 1.21 (m, 3H). ^{13}C NMR (101 MHz, $CDCl_3$) δ 207.7, 155.3, 149.3, 148.7, 138.2, 129.2, 128.4, 128.0, 126.8, 107.2, 104.3, 63.3, 56.1, 56.0, 53.7, 53.6, 45.3, 38.6,

34.3, 33.2, 32.8, 31.6. (*37*)

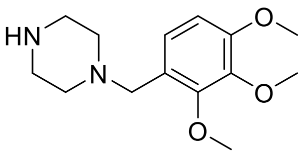

Trimetazidine (99): The title compound was prepared according to the general procedure and purified by column chromatography on silica gel to afford a yellow oil in 81% yield (43.1 mg); ^{1}H NMR (400 MHz, $CDCl_3$) δ 6.94 (d, *J* = 8.5 Hz, 1H), 6.58 (d, *J* = 8.5 Hz, 1H), 3.93 – 3.71 (m, 9H), 3.41 (d, *J* = 7.6 Hz, 2H), 2.82 (dd, *J* = 10.3, 5.7 Hz, 4H), 2.38 (s, 4H), 2.30 (s, 1H). ^{13}C NMR (101 MHz, $CDCl_3$) δ 152.7, 152.5, 142.1, 125.0, 123.7, 106.8, 61.0, 60.6, 56.9, 55.8, 54.1, 52.8, 51.5, 45.9. (*6*)

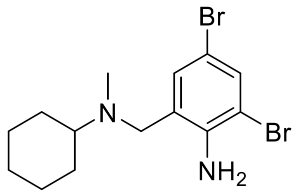

Bromhexine (100): The title compound was prepared according to the general procedure and purified by column chromatography on silica gel to afford a yellow oil in 74% yield (55.4 mg); ^{1}H NMR (400 MHz, $CDCl_3$) δ 7.45 (d, *J* = 2.0 Hz, 1H), 7.04 (d, *J* = 1.9 Hz, 1H), 5.46 (s, 2H), 3.58 (s, 2H), 2.40 (t, *J* = 10.7 Hz, 1H), 2.11 (s, 3H), 1.80 (d, *J* = 10.1 Hz, 4H), 1.63 (d, *J* = 12.7 Hz, 1H), 1.38 – 1.17 (m, 4H), 1.09 (dd, *J* = 24.0, 12.0 Hz, 1H). ^{13}C NMR (101 MHz, $CDCl_3$) δ 144.1, 132.9, 131.6, 126.1, 110.0, 108.0, 61.9, 57.7, 36.3, 28.2, 26.2, 25.9. HRMS (EI) m/z: $[M]^+$ calcd for $C_{14}H_{20}Br_2N_2$: 373.9993; found, 373.9996.

4.2 Characterization Data of Ethers

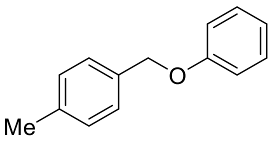

1-methyl-4-(phenoxymethyl)benzene (101): The title compound was prepared according to the general procedure and purified by column chromatography on silica gel to afford a yellow solid (mp: 79-80 ºC) in 96% yield (38.0 mg); ^{1}H NMR (400 MHz, $CDCl_3$) δ 7.40 (dd, *J* = 19.6, 7.6 Hz, 4H), 7.29 (d, *J* = 7.6 Hz, 2H), 7.07 (dd, *J* = 13.4, 7.6 Hz, 3H), 5.10 (s, 2H), 2.46 (s, 3H). ^{13}C NMR (101 MHz, $CDCl_3$) δ 158.8, 137.6, 134.0, 129.4, 129.2, 127.5, 120.8, 114.8, 69.8, 21.1.

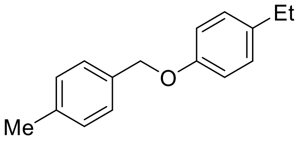

1-Ethyl-4-((4-methylbenzyl)oxy)benzene (102): The title compound was prepared according to the general procedure and purified by column chromatography on silica gel to afford a grey solid (mp: 70-71 ºC) in 97% yield (43.9 mg); ^{1}H NMR (400 MHz, $CDCl_3$) δ 7.44 (d, *J* = 7.2 Hz, 2H), 7.30 (d, *J* = 7.9

Hz, 2H), 7.23 (d, J = 7.5 Hz, 2H), 7.02 (d, J = 7.5 Hz, 2H), 5.10 (s, 2H), 2.71 (dd, J = 14.4, 7.0 Hz, 2H), 2.47 (s, 3H), 1.34 (t, J = 7.2 Hz, 3H). ^{13}C NMR (101 MHz, $CDCl_3$) δ 156.9, 137.5, 136.5, 134.2, 129.2, 128.6, 127.5, 114.7, 69.9, 27.9, 21.1, 15.8. HRMS (EI) m/z: $[M]^+$ calcd for $C_{16}H_{18}O$: 226.1358; found, 226.1356.

1-Methyl-4-((4-(*tert*-pentyl)phenoxy)methyl)benzene (103): The title compound was prepared

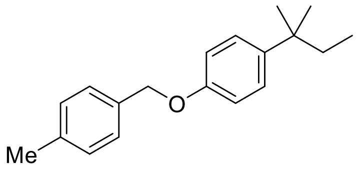

according to the general procedure and purified by column chromatography on silica gel to afford a yellow solid (mp: 48-49 ºC) in 91% yield (48.8 mg); ^{1}H NMR (400 MHz, $CDCl_3$) δ 7.44 (d, J = 7.3 Hz, 2H), 7.36 (d, J = 7.8 Hz, 2H), 7.30 (d, J = 7.5 Hz, 2H), 7.04 (d, J = 7.9 Hz, 2H), 5.11 (s, 2H), 2.47 (s, 3H), 1.74 (q, J = 7.1 Hz, 2H), 1.38 (s, 6H), 0.81 (t, J = 7.2 Hz, 3H). IR (neat, ν/cm^{-1}) 3093, 3044, 3025, 3010, 2959, 2921, 2874, 1608, 1557, 1510, 1458, 1373, 1293, 1242, 1178, 1013, 873, 799, 669. HRMS (EI) m/z: $[M]^+$ calcd for $C_{19}H_{24}O$: 268.1827; found, 268.1827.

1-methyl-4-((4-(2-phenylpropan-2-yl)phenoxy)methyl)benzene (104): The title compound was

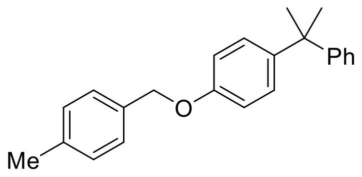

prepared according to the general procedure and purified by column chromatography on silica gel to afford a yellow oil in 86% yield (54.4 mg); ^{1}H NMR (400 MHz, $CDCl_3$) δ 7.41 (d, J = 7.4 Hz, 2H), 7.35 (d, J = 6.5 Hz, 4H), 7.26 (dd, J = 12.8, 8.2 Hz, 5H), 6.98 (d, J = 8.1 Hz, 2H), 5.08 (s, 2H), 2.45 (s, 3H), 1.76 (s, 6H). ^{13}C NMR (101 MHz, $CDCl_3$) δ 156.8, 150.9, 143.0, 137.6, 134.1, 129.2, 127.9, 127.7, 127.6, 126.7, 125.5, 114.1, 69.9, 42.3, 30.9, 21.2. HRMS (EI) m/z: $[M]^+$ calcd for $C_{23}H_{24}O$: 316.1827; found, 316.1829.

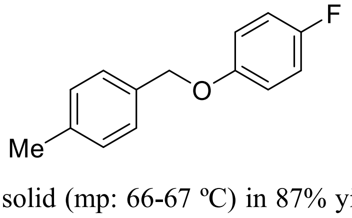

1-Fluoro-4-((4-methylbenzyl)oxy)benzene (105): The title compound was prepared according to the general procedure and purified by column chromatography on silica gel to afford a yellow solid (mp: 66-67 ºC) in 87% yield (37.6 mg); ^{1}H NMR (400 MHz, $CDCl_3$) δ 7.38 (d, J = 7.4 Hz, 2H), 7.26 (d, J = 7.5 Hz, 2H), 7.03 (t, J = 8.0 Hz, 2H), 7.00 – 6.94 (m, 2H), 5.04 (s, 2H), 2.43 (s, 3H). ^{13}C NMR (101 MHz, $CDCl_3$) δ 157.3 (d, J = 238.3 Hz), 154.9 (d, J = 1.9 Hz), 137.8, 133.8, 129.2, 127.6, 115.9 (d, J = 3.9 Hz), 115.7 (d, J = 19.0 Hz), 70.5, 21.1. ^{19}F NMR (377 MHz, $CDCl_3$) δ -123.81.

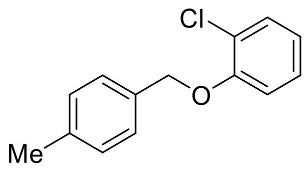

1-Chloro-2-((4-methylbenzyl)oxy)benzene (106): The title compound was prepared according to the general procedure and purified by column chromatography on silica gel to afford a yellow solid (mp 70-71 ºC) in 90% yield (41.8 mg); ^{1}H NMR (400 MHz, $CDCl_3$) δ 7.43 (t, *J* = 8.6 Hz, 3H), 7.32 – 7.18 (m, 3H), 7.02 (d, *J* = 8.2 Hz, 1H), 6.95 (t, *J* = 7.6 Hz, 1H), 5.17 (s, 2H), 2.42 (s, 3H). ^{13}C NMR (101 MHz, $CDCl_3$) δ 154.2, 137.6, 133.5, 130.3, 129.2, 127.6, 127.2, 123.2, 121.5, 114.1, 70.7, 21.1. HRMS (EI) m/z: $[M]^+$ calcd for $C_{14}H_{13}ClO$: 232.0655; found, 232.0657.

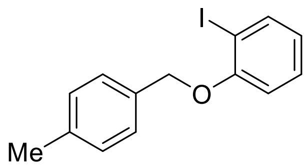

1-Iodo-2-((4-methylbenzyl)oxy)benzene (107): The title compound was prepared according to the general procedure and purified by column chromatography on silica gel to afford a yellow oil in 88% yield (57.1 mg); ^{1}H NMR (400 MHz, $CDCl_3$) δ 7.86 (d, *J* = 7.7 Hz, 1H), 7.45 (d, *J* = 7.4 Hz, 2H), 7.32 (t, *J* = 7.7 Hz, 1H), 7.26 (d, *J* = 7.5 Hz, 2H), 6.91 (d, *J* = 8.2 Hz, 1H), 6.77 (t, *J* = 7.5 Hz, 1H), 5.16 (s, 2H), 2.43 (s, 3H). ^{13}C NMR (101 MHz, $CDCl_3$) δ 157.2, 139.4, 137.5, 133.4, 129.3, 129.2, 127.0, 122.7, 112.7, 86.8, 70.7, 21.1.

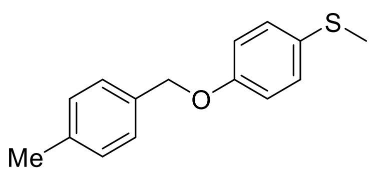

Methyl(4-((4-methylbenzyl)oxy)phenyl)sulfane (108): The title compound was prepared according to the general procedure and purified by column chromatography on silica gel to afford a yellow solid (mp: 93-94 ºC) in 85% yield (41.5 mg); ^{1}H NMR (400 MHz, $CDCl_3$) δ 7.33 (d, *J* = 7.5 Hz, 2H), 7.28 (d, *J* = 8.1 Hz, 2H), 7.21 (d, *J* = 7.5 Hz, 2H), 6.94 (d, *J* = 8.1 Hz, 2H), 5.02 (s, 2H), 2.46 (s, 3H), 2.38 (s, 3H). ^{13}C NMR (101 MHz, $CDCl_3$) δ 157.5, 137.8, 133.9, 130.1, 129.3, 129.1, 127.6, 115.6, 70.1, 21.2, 18.0. HRMS (EI) m/z: $[M]^+$ calcd for $C_{15}H_{16}OS$: 244.0922; found, 244.0923.

2,4-Di-*tert*-butyl-1-((4-methylbenzyl)oxy)benzene (109): The title compound was prepared

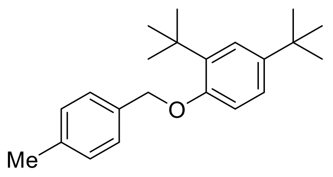

according to the general procedure and purified by column chromatography on silica gel to afford a yellow solid (mp: 119-120 ºC) in 85% yield (52.7 mg); ^{1}H NMR (400 MHz, $CDCl_3$) δ 7.47 (d, *J* = 8.6 Hz, 3H), 7.35 – 7.20 (m, 3H), 6.99 (d, *J* = 8.3 Hz, 1H), 5.16 (s, 2H), 2.48 (s, 3H), 1.55 (s, 9H), 1.44 (s, 9H). ^{13}C NMR (101 MHz, $CDCl_3$) δ 155.4, 142.6, 137.4, 137.3, 134.6, 129.2, 127.4,

123.9, 123.3, 111.7, 70.0, 35.1, 34.3, 31.6, 29.9, 21.2. IR (neat, ν/cm^{-1}) 3055, 3022, 3001, 2949, 2921, 2865, 1554, 1495, 1458, 1380, 1230, 1088, 1006, 867, 797, 766, 669, 639. HRMS (EI) m/z: $[M]^+$ calcd for $C_{22}H_{30}O$: 310.2297; found, 310.2299.

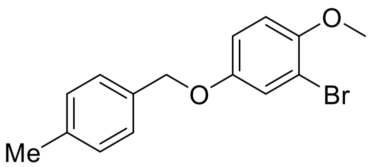

1-Bromo-1-methoxy-4-((4-methylbenzyl)oxy)benzene (110): The title compound was prepared according to the general procedure and purified by column chromatography on silica gel to afford a yellow solid (mp: 60-61 ºC) in 96% yield (58.8 mg); ^{1}H NMR (400 MHz, $CDCl_3$) δ 7.35 (d, *J* = 7.3 Hz, 2H), 7.30 – 7.21 (m, 3H), 6.93 (d, *J* = 8.9 Hz, 1H), 6.86 (d, *J* = 8.9 Hz, 1H), 4.99 (s, 2H), 3.88 (s, 3H), 2.42 (s, 3H). ^{13}C NMR (101 MHz, $CDCl_3$) δ 153.1, 150.3, 137.7, 133.6, 129.2, 127.5, 120.1, 114.6, 112.7, 111.8, 70.7, 56.7, 21.1. IR (neat, ν/cm^{-1}) 3095, 3076, 3052, 3012, 2972, 2916, 2850, 1650, 1558, 1493, 1458, 1220, 1208, 1039, 1013, 893, 839, 804, 766, 741, 674. HRMS (EI) m/z: $[M]^+$ calcd for $C_{15}H_{15}BrO_2$: 306.0255; found, 306.0252.

1,2,3-Trimethoxy-5-((4-methylbenzyl)oxy)benzene (111): The title compound was prepared

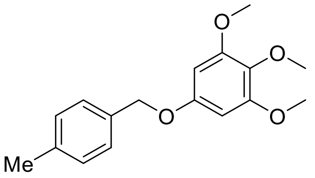

according to the general procedure and purified by column chromatography on silica gel to afford a yellow solid (mp: 56-57 ºC) in 78% yield (44.9 mg); ^{1}H NMR (400 MHz, $CDCl_3$) δ 7.33 (d, *J* = 7.4 Hz, 2H), 7.20 (d, *J* = 7.4 Hz, 2H), 6.24 (s, 2H), 4.98 (s, 2H), 3.83 (s, 6H), 3.80 (s, 3H), 2.37 (s, 3H). ^{13}C NMR (101 MHz, $CDCl_3$) δ 155.4, 153.5, 137.7, 133.7, 132.3, 129.1, 127.6, 92.6, 70.3, 60.9, 55.9, 21.0. IR (neat, ν/cm^{-1}) 3080, 3064, 3047, 3010, 2960, 2918, 2871, 2835, 1589, 1506, 1449, 1418, 1380, 1340, 1226, 1191, 1176, 1150, 1122, 1043, 1020, 1005, 962, 816, 780, 667, 637, 623. HRMS (EI) m/z: $[M]^+$ calcd for $C_{17}H_{20}O_4$: 288.1362; found, 288.1369.

2-(4-(Benzyloxy)phenyl)-4,4,5,5-tetramethyl-1,3,2-dioxaborolane (112): The title compound

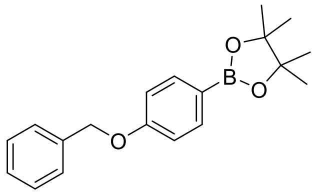

was prepared according to the general procedure and purified by column chromatography on silica gel to afford a yellow solid (mp: 82-83 ºC) in 74% yield (45.9 mg); ^{1}H NMR (400 MHz, $CDCl_3$) δ 7.77 (d, *J* = 7.8 Hz, 2H), 7.51 – 7.29 (m, 5H), 6.98 (d, *J* = 7.7 Hz, 2H), 5.10 (s, 2H), 1.34 (s, 12H). ^{13}C NMR (101 MHz, $CDCl_3$) δ 161.3, 136.8, 136.5, 128.5, 128.0, 127.9, 127.4, 114.2, 83.5, 69.7, 24.8. (*56*)

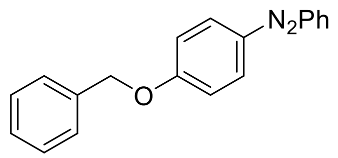

1-(4-(Benzyloxy)phenyl)-2-phenyldiazene (113): The title compound was prepared according to the general procedure and purified by column chromatography on silica gel to afford a yellow solid (mp: 110-111 ºC) in 90% yield (51.9 mg); ^{1}H NMR (400 MHz, $CDCl_3$) δ 7.99 (dd, *J* = 13.7, 8.2 Hz, 4H), 7.65 – 7.27 (m, 8H), 7.14 (d, *J* = 7.7 Hz, 2H), 5.16 (s, 2H). ^{13}C NMR (101 MHz, $CDCl_3$) δ 161.1, 152.7, 147.1, 136.4, 130.3, 129.0, 128.6, 128.1, 127.4, 124.7, 122.5, 115.0, 70.1. HRMS (EI) m/z: $[M]^+$ calcd for $C_{19}H_{16}N_2O$: 288.1263; found, 288.1262.

***N*-(4-(benzyloxy)phenyl)acetamide (114):** The title compound was prepared according to the

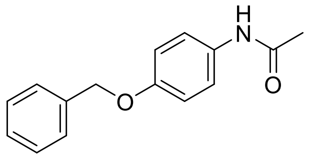

general procedure and purified by column chromatography on silica gel to afford a brown solid (mp: 142-143 ºC) in 75% yield (36.2 mg); ^{1}H NMR (400 MHz, $CDCl_3$) δ 7.52 (s, 1H), 7.45 – 7.29 (m, 7H), 6.91 (d, *J* = 8.0 Hz, 2H), 5.02 (s, 2H), 2.12 (s, 3H). ^{13}C NMR (101 MHz, $CDCl_3$) δ 168.4, 155.5, 136.9, 131.2, 128.5, 127.9, 127.4, 121.9, 115.1, 70.2, 24.2.

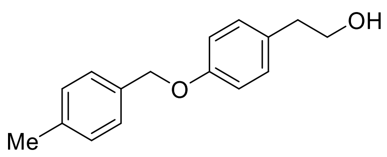

2-(4-((4-methylbenzyl)oxy)phenyl)ethan-1-ol (115): The title compound was prepared according to the general procedure and purified by column chromatography on silica gel to afford a yellow solid (mp: 87-88 ºC) in 73% yield (35.3 mg); ^{1}H NMR (400 MHz, $CDCl_3$) δ 7.34 (d, *J* = 7.9 Hz, 2H), 7.21 (d, *J* = 7.8 Hz, 2H), 7.15 (d, *J* = 8.5 Hz, 2H), 6.94 (d, *J* = 8.5 Hz, 2H), 5.02 (s, 2H), 3.82 (t, *J* = 6.6 Hz, 2H), 2.81 (t, *J* = 6.6 Hz, 2H), 2.38 (s, 3H), 1.67 (s, 1H). ^{13}C NMR (101 MHz, $CDCl_3$) δ 157.6, 137.7, 134.1, 130.7, 130.0, 129.3, 127.6, 115.0, 70.0, 63.8, 38.3, 21.2. IR (neat, ν/cm^{-1}) 3238, 3066, 3030, 3009, 2905, 2864, 1616, 1510, 1237, 1173, 1110, 1049, 1006, 867, 811, 764, 667. HRMS (EI) m/z: $[M]^+$ calcd for $C_{16}H_{18}O_2$: 242.1307; found, 242.1306.

(4-((4-methylbenzyl)oxy)phenyl)(phenyl)methanone (116): The title compound was prepared

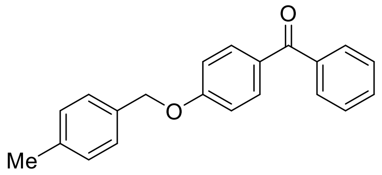

according to the general procedure and purified by column chromatography on silica gel to afford a yellow solid (mp 98-99 ºC) in 95% yield (57.4 mg); ^{1}H NMR (400 MHz, $CDCl_3$) δ 7.87 (d, *J* = 8.3 Hz, 2H), 7.80 (d, *J* = 7.2 Hz, 2H), 7.60 (t, *J* = 7.2 Hz, 1H), 7.51 (t, *J* = 7.3 Hz, 2H), 7.37 (d, *J* = 7.5 Hz, 2H), 7.25 (d, *J* = 7.4 Hz, 2H), 7.07 (d, *J* = 8.3 Hz, 2H), 5.14 (s, 2H), 2.41 (s,

3H). ^{13}C NMR (101 MHz, $CDCl_3$) δ 195.4, 162.4, 138.2, 138.0 133.1, 132.4, 131.8, 130.2, 129.6, 129.3, 128.1, 127.5, 114.3, 70.0, 21.1. (*57*)

4-((4-methylbenzyl)oxy)-*N*-phenylaniline (117): The title compound was prepared according to

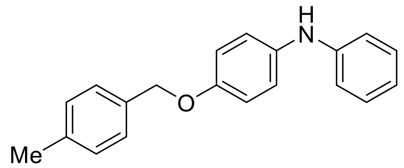

the general procedure and purified by column chromatography on silica gel to afford a grey solid (mp: 80-81 ºC) in 92% yield (53.2 mg); ^{1}H NMR (400 MHz, $CDCl_3$) δ 7.41 (d, *J* = 7.9 Hz, 2H), 7.29 (t, *J* = 8.0 Hz, 4H), 7.16 – 7.09 (m, 2H), 7.00 (dt, *J* = 7.6, 4.2 Hz, 4H), 6.91 (t, *J* = 7.3 Hz, 1H). 5.54 (s, 1H), 5.07 (s, 2H), 2.44 (s, 3H). ^{13}C NMR (101 MHz, $CDCl_3$) δ 154.4, 145, 137.6, 135.9, 134.1, 129.2, 129.2, 127.6, 121.9, 119.6, 115.7, 115.7, 70.3, 21.2. HRMS (EI) m/z: [M]$^+$ calcd for $C_{20}H_{19}NO$: 289.1467; found, 289.1465.

6-((4-methylbenzyl)oxy)-3,4-dihydroquinolin-2(1*H*)-one (118): The title compound was

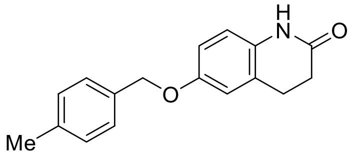

prepared according to the general procedure and purified by column chromatography on silica gel to afford a yellow solid (mp: 162-163 ºC) in 77% yield (41.1 mg); ^{1}H NMR (400 MHz, $CDCl_3$) δ 9.25 (d, *J* = 17.9 Hz, 1H), 7.31 (d, *J* = 7.9 Hz, 2H), 7.19 (d, *J* = 7.8 Hz, 2H), 6.78 (d, *J* = 8.4 Hz, 3H), 4.98 (s, 2H), 2.92 (t, *J* = 7.5 Hz, 2H), 2.71 – 2.50 (m, 2H), 2.36 (s, 3H). ^{13}C NMR (101 MHz, $CDCl_3$) δ 171.84, 154.7, 137.7, 133.9, 131.0, 129.2, 127.5, 124.9, 116.3, 114.8, 113.4, 70.3, 30.5, 25.6, 21.2. (*58*)

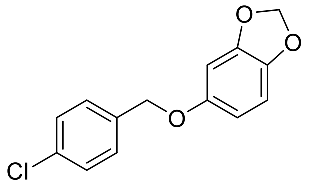

5-((4-chlorobenzyl)oxy)benzo[d][1,3]dioxole (119): The title compound was prepared according to the general procedure and purified by column chromatography on silica gel to afford a black solid (mp 71-72 ºC) in 80% yield (41.9 mg); ^{1}H NMR (400 MHz, $CDCl_3$) δ 7.35 (s, 4H), 6.72 (d, *J* = 8.5 Hz, 1H), 6.56 (d, *J* = 2.5 Hz, 1H), 6.38 (dd, *J* = 8.5, 2.5 Hz, 1H), 5.92 (s, 2H), 4.95 (s, 2H). ^{13}C NMR (101 MHz, $CDCl_3$) δ 153.9, 148.2, 141.9, 135.5, 133.6, 128.7, 128.6, 107.9, 106.0, 101.1, 98.3, 70.0. IR (neat, ν/cm^{-1}) 3069, 3051, 3035, 3012, 2991, 2918, 2871, 1626, 1507, 1484, 1374, 1236, 1181, 1089, 1016, 919, 860, 832, 806, 785, 771, 705, 665, 636. HRMS (EI) m/z: [M]$^+$ calcd for $C_{14}H_{11}ClO_3$: 262.0397; found, 262.0400.

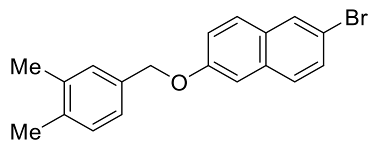

1-bromo-6-((3,4-dimethylbenzyl)oxy)naphthalene (120): The title compound was prepared according to the general procedure and purified by column chromatography on silica gel to afford a yellow solid (mp: 116-117 ºC) in 86% yield (58.5 mg); ^{1}H NMR (400 MHz, $CDCl_3$) δ 7.96 (s, 1H), 7.69 (d, *J* = 8.9 Hz, 1H), 7.63 (d, *J* = 8.6 Hz, 1H), 7.55 (d, *J* = 8.7 Hz, 1H), 7.35 – 7.17 (m, 5H), 5.14 (s, 2H), 2.36 (s, 6H). ^{13}C NMR (101 MHz, $CDCl_3$) δ 157.1, 136.8, 136.5, 133.9, 133.0, 130.0, 129.8, 129.6, 129.5, 129.1, 128.4, 128.4, 125.3, 120.1, 117.0, 107.0, 70.0, 19.7, 19.5. IR (neat, ν/cm^{-1}) 3119, 3044, 3017, 2980, 2937, 2912, 2855, 1625, 1580, 1498, 1448, 1349, 1267, 1206, 1150, 1044, 883, 863, 825, 799, 707, 649, 639, 609. HRMS (EI) m/z: [M]$^+$ calcd for $C_{19}H_{17}BrO$: 340.0463; found, 340.0466.

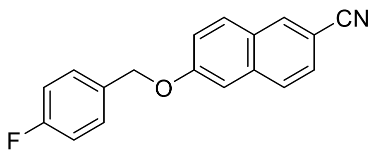

6-((4-fluorobenzyl)oxy)-2-naphthonitrile (121): The title compound was prepared according to the general procedure and purified by column chromatography on silica gel to afford a yellow solid (mp:133-134 ºC) in 87% yield (48.2 mg); ^{1}H NMR (400 MHz, $CDCl_3$) δ 8.13 (s, 1H), 7.86 – 7.71 (m, 2H), 7.56 (d, *J* = 8.5 Hz, 1H), 7.50 – 7.41 (m, 2H), 7.31 (d, *J* = 8.9 Hz, 1H), 7.22 (s, 1H), 7.10 (t, *J* = 8.2 Hz, 2H), 5.16 (s, 2H). ^{13}C NMR (101 MHz, $CDCl_3$) δ 162.7 (d, *J* = 246.9 Hz), 158.9, 136.3, 133.7, 131.9 (d, *J* = 3.3 Hz), 130.1, 129.4 (d, *J* = 8.2 Hz), 127.9, 127.8, 127.1, 120.8, 119.5, 115.7 (d, *J* = 21.6 Hz), 107.2, 107.0. 69.6. ^{19}F NMR (377 MHz, $CDCl_3$) δ -113.57. IR (neat, ν/cm^{-1}) 3071, 3050, 3012, 2921, 2848, 2218, 1627, 1604, 1511, 1476, 1392, 1260, 1225, 1175, 1159, 1001, 947, 881, 847, 827, 799, 744, 658, 603. HRMS (EI) m/z: [M]$^+$ calcd for $C_{18}H_{12}FNO$: 277.0903; found, 277.0905.

methyl 6-((4-methylbenzyl)oxy)-2-naphthoate (122): The title compound was prepared according

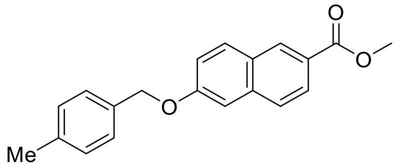

to the general procedure and purified by column chromatography on silica gel to afford a yellow solid (mp: 134-135 ºC) in 67% yield (41.0 mg); ^{1}H NMR (400 MHz, $CDCl_3$) δ 8.51 (s, 1H), 8.00 (d, *J* = 8.5 Hz, 1H), 7.83 (d, *J* = 8.8 Hz, 1H), 7.72 (d, *J* = 8.5 Hz, 1H), 7.36 (d, *J* = 7.3 Hz, 2H), 7.22 (dd, *J* = 16.1, 8.3 Hz, 4H), 5.13 (s, 2H), 3.95 (s, 3H), 2.36 (s, 3H). ^{13}C NMR (101 MHz, $CDCl_3$) δ 167.3, 158.7, 138.0, 137.1, 133.4, 130.9, 130.8, 129.3, 128.0, 127.7,

126.9, 125.9, 125.3, 120.0, 106.9, 70.1, 52.1, 21.2. IR (neat, ν/cm^{-1}) 3067, 3025, 3006, 2950, 2918, 2870, 1716, 1623, 1598, 1479, 1457, 1436, 1380, 1337, 1287, 1196, 1093, 1009, 913, 866, 819, 804, 768, 752, 669, 585. HRMS (EI) m/z: [M]$^+$ calcd for $C_{20}H_{18}O_3$: 306.1256; found, 306.1255.

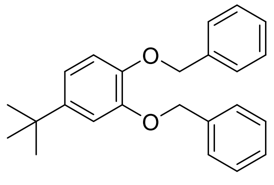

(((4-(*tert*-butyl)-1,2-phenylene)bis(oxy))bis(methylene))dibenzene (123): The title compound was prepared according to the general procedure and purified by column chromatography on silica gel to afford a yellow oil in 67% yield (46.4 mg); ^{1}H NMR (400 MHz, $CDCl_3$) δ 7.52 (s, 4H), 7.42 (t, *J* = 7.1 Hz, 4H), 7.39 – 7.31 (m, 2H), 7.07 (s, 1H), 7.00 – 6.89 (m, 2H), 5.23 (s, 2H), 5.20 (s, 2H), 1.32 (s, 9H). ^{13}C NMR (101 MHz, $CDCl_3$) δ 148.3, 147.0, 144.7, 137.6, 128.4, 128.3, 127.7, 127.6, 127.5, 127.2, 125.4, 118.2, 114.5, 113.9, 71.8, 71.3, 34.3, 31.4. HRMS (EI) m/z: [M]$^+$ calcd for $C_{24}H_{26}O_2$: 346.1933; found, 346.1930.

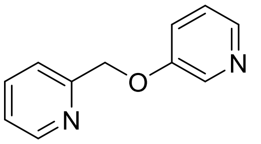

2-((pyridin-3-yloxy)methyl)pyridine (124): The title compound was prepared according to the general procedure and purified by column chromatography on silica gel to afford a brown oil in 76% yield (28.3 mg); ^{1}H NMR (400 MHz, $CDCl_3$) δ 8.54 (d, *J* = 3.6 Hz, 1H), 8.35 (s, 1H), 8.17 (d, *J* = 3.3 Hz, 1H), 7.65 (t, *J* = 7.6 Hz, 1H), 7.44 (d, *J* = 7.8 Hz, 1H), 7.23 – 7.08 (m, 3H), 5.17 (s, 2H). ^{13}C NMR (101 MHz, $CDCl_3$) δ 156.1, 154.5, 149.2, 142.3, 138.3, 136.8, 123.7, 122.8, 121.2, 121.1, 70.7. IR (neat, ν/cm^{-1}) 3093, 3064, 2930, 1594, 1576, 1477, 1428, 1378, 1272, 1227, 1100, 1053, 1001, 797, 757, 705, 654, 614.

1-isopropyl-4-(1-phenylethoxy)benzene (125): The title compound was prepared according to the

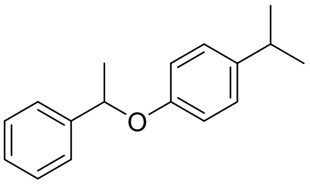

general procedure and purified by column chromatography on silica gel to afford a yellow oil in 87% yield (41.8 mg); ^{1}H NMR (400 MHz, $CDCl_3$) δ 7.41 (d, *J* = 7.5 Hz, 2H), 7.36 (t, *J* = 7.5 Hz, 2H), 7.30 – 7.26 (m, 1H), 7.08 (d, *J* = 8.5 Hz, 2H), 6.82 (d, *J* = 8.5 Hz, 2H), 5.29 (q, *J* = 6.5 Hz, 1H), 2.89 – 2.77 (m, 1H), 1.65 (d, *J* = 6.5 Hz, 3H), 1.21 (d, *J* = 7.0 Hz, 6H). ^{13}C NMR (101 MHz, $CDCl_3$) δ 156.1, 143.5, 140.9, 128.5, 127.3, 127.1, 125.5, 115.6, 76.0, 33.2, 24.5, 24.1. HRMS (EI) m/z: [M]$^+$ calcd for $C_{17}H_{20}O$: 240.1514; found, 240.1515.

1-(*tert*-butyl)-4-(1-(4-isopropylphenoxy)ethyl)benzene (126):

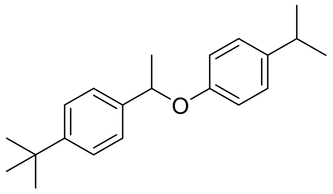

The title compound was prepared according to the general procedure and purified by column chromatography on silica gel to afford a yellow oil in 81% yield (48.0 mg); ^{1}H NMR (400 MHz, $CDCl_3$) δ 7.34 (q, J = 8.4 Hz, 4H), 7.07 (d, J = 8.5 Hz, 2H), 6.82 (d, J = 8.7 Hz, 2H), 5.26 (q, J = 6.4 Hz, 1H), 2.89 – 2.76 (m, 1H), 1.61 (d, J = 6.4 Hz, 3H), 1.31 (s, 9H), 1.20 (d, J = 6.9 Hz, 6H). ^{13}C NMR (101 MHz, $CDCl_3$) δ 156.2, 150.1, 140.8, 140.4, 127.1, 125.4, 125.2, 115.6, 75.8, 34.5, 33.2, 31.4, 24.3, 24.2. HRMS (EI) m/z: $[M]^+$ calcd for $C_{21}H_{28}O$: 296.2140; found, 296.2144.

1-chloro-4-(1-(4-isopropylphenoxy)ethyl)benzene (127): The title compound was prepared

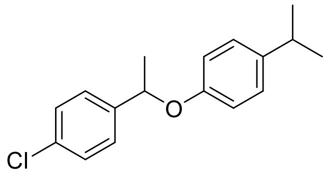

according to the general procedure and purified by column chromatography on silica gel to afford a yellow oil in 71% yield (38.9 mg); ^{1}H NMR (400 MHz, $CDCl_3$) δ 7.31 (s, 4H), 7.06 (d, J = 8.7 Hz, 2H), 6.77 (d, J = 8.7 Hz, 2H), 5.24 (q, J = 6.4 Hz, 1H), 2.88 – 2.75 (m, 1H)., 1.60 (d, J = 6.4 Hz, 3H), 1.19 (d, J = 7.0 Hz, 6H). ^{13}C NMR (101 MHz, $CDCl_3$) δ 155.8, 142.0, 141.2, 133.0, 128.8, 127.2, 127.0, 115.6, 75.3, 33.2, 24.4, 24.1. IR (neat, ν/cm^{-1}) 3097, 3032, 2959, 2928, 2870, 1608, 1507, 1491, 1232, 1178, 1081, 1011, 933, 827, 719, 662. HRMS (EI) m/z: $[M]^+$ calcd for $C_{17}H_{19}ClO$: 274.1124; found, 274.1126.

1-isopropyl-4-(1-(4-(methylsulfonyl)phenyl)ethoxy)benzene (128): The title compound was

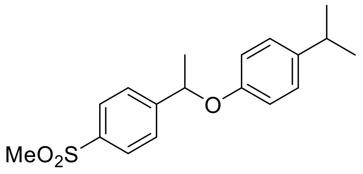

prepared according to the general procedure and purified by column chromatography on silica gel to afford a yellow oil in 64% yield (40.7 mg); ^{1}H NMR (400 MHz, $CDCl_3$) δ 7.91 (d, J = 8.1 Hz, 2H), 7.58 (d, J = 8.1 Hz, 2H), 7.06 (d, J = 8.5 Hz, 2H), 6.75 (d, J = 8.6 Hz, 2H), 5.34 (q, J = 6.5 Hz, 1H), 3.04 (s, 3H), 2.87 – 2.74 (m, 1H), 1.62 (d, J = 6.4 Hz, 3H), 1.18 (d, J = 6.9 Hz, 6H). ^{13}C NMR (101 MHz, $CDCl_3$) δ 155.4, 149.9, 141.6, 139.5, 127.8, 127.2, 126.5, 115.5, 75.1, 44.4, 33.1, 24.2, 24.1. IR (neat, ν/cm^{-1}) 3064, 3029, 2959, 2923, 2871, 1606, 1510, 1404, 1305, 1234, 1178, 1147, 1075, 1011, 954, 827, 757, 724, 658. HRMS (EI) m/z: $[M]^+$ calcd for $C_{18}H_{22}O_3S$: 318.1290; found, 318.1288.

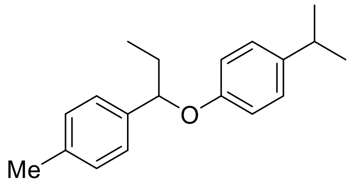

1-isopropyl-4-(1-(p-tolyl)propoxy)benzene (129): The title compound was prepared according to the general procedure and purified by column chromatography on silica gel to afford a yellow oil in 84% yield (45.0 mg); ^{1}H NMR (400 MHz, $CDCl_3$) δ 7.29 (d, *J* = 8.0 Hz, 2H), 7.18 (d, *J* = 8.0 Hz, 2H), 7.08 (d, *J* = 8.7 Hz, 2H), 6.83 (d, *J* = 8.6 Hz, 2H), 4.99 (dd, *J* = 7.3, 5.6 Hz, 1H), 2.97 – 2.75 (m, 1H), 2.37 (s, 3H), 2.12 – 1.96 (m, 1H), 1.95 – 1.81 (m, 1H), 1.22 (d, *J* = 6.9 Hz, 6H), 1.04 (t, *J* = 7.4 Hz, 3H). ^{13}C NMR (101 MHz, $CDCl_3$) δ 156.6, 140.7, 139.3, 136.8, 129.1, 127.0, 126.0, 115.6, 81.4, 33.2, 31.7, 24.1, 21.1, 10.3. HRMS (EI) m/z: $[M]^+$ calcd for $C_{19}H_{24}O$: 268.1827; found, 268.1828.

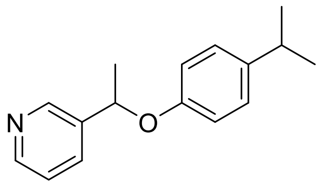

3-(1-(4-isopropylphenoxy)ethyl)pyridine (130): The title compound was prepared according to the general procedure and purified by column chromatography on silica gel to afford a brown oil in 69% yield (33.3 mg); ^{1}H NMR (400 MHz, $CDCl_3$) δ 8.66 (s, 1H), 8.53 (d, *J* = 4.7 Hz, 1H), 7.74 (d, *J* = 7.9 Hz, 1H), 7.27 (dd, *J* = 7.9, 4.8 Hz, 1H), 7.09 (d, *J* = 8.6 Hz, 2H), 6.81 (d, *J* = 8.6 Hz, 2H), 5.35 (q, *J* = 6.4 Hz, 1H), 2.90 – 2.76 (m, 1H), 1.66 (d, *J* = 6.5 Hz, 3H), 1.20 (d, *J* = 6.9 Hz, 6H). ^{13}C NMR (101 MHz, $CDCl_3$) δ 155.5, 148.8, 147.6, 141.5, 138.7, 133.3, 127.2, 123.6, 115.7, 73.7, 33.1, 24.1, 24.0. IR (neat, ν/cm^{-1}) 3088, 3059, 3032, 2959, 2925, 2870, 1606, 1507, 1423, 1232, 1175, 1076, 1011, 934, 827, 806, 712, 632. HRMS (EI) m/z: $[M]^+$ calcd for $C_{16}H_{19}NO$: 241.1467; found, 241.1465.

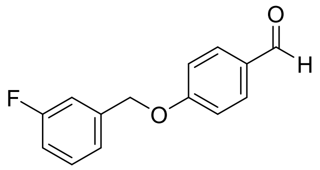

4-((3-fluorobenzyl)oxy)benzaldehyde (131): The title compound was prepared according to the general procedure and purified by column chromatography on silica gel to afford a white solid (mp: 46-47 ^{o}C) in 91% yield (41.9 mg); ^{1}H NMR (400 MHz, $CDCl_3$) δ 9.87 (s, 1H), 7.83 (d, *J* = 8.7 Hz, 2H), 7.35 (dd, *J* = 13.8, 7.9 Hz, 1H), 7.16 (dd, *J* = 19.2, 8.6 Hz, 2H), 7.09 – 6.98 (m, 3H), 5.12 (s, 2H). ^{13}C NMR (101 MHz, $CDCl_3$) δ 190.6, 163.3, 162.9 (d, *J* = 246.6 Hz), 138.4 (d, *J* = 7.4 Hz), 131.9, 130.3, 130.2 (d, *J* = 2.1 Hz), 122.7 (d, *J* = 3.0 Hz), 115.1 (d, *J* = 21.1 Hz), 115.0, 114.1 (d, *J* = 22.2 Hz), 69.2 (d, *J* = 1.9 Hz). ^{19}F NMR (376 MHz, $CDCl_3$) δ -118.43. HRMS (EI) m/z: $[M]^+$ calcd for $C_{14}H_{11}FO_2$: 230.0743; found, 230.0744.

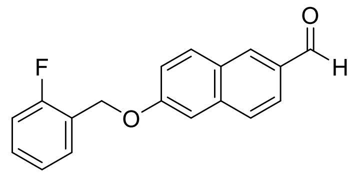

6-((2-fluorobenzyl)oxy)-2-naphthaldehyde (132): The title compound was prepared according to the general procedure and purified by column chromatography on silica gel to afford a white solid (mp: 83-84 °C) in 87% yield (48.7 mg); ^{1}H NMR (400 MHz, $CDCl_3$) δ 10.08 (s, 1H), 8.23 (s, 1H), 7.90 (t, *J* = 9.1 Hz, 2H), 7.79 (d, *J* = 8.5 Hz, 1H), 7.62 – 7.47 (m, 1H), 7.39 – 7.24 (m, 3H), 7.16 (dt, *J* = 18.4, 8.4 Hz, 2H), 5.26 (s, 2H). ^{13}C NMR (101 MHz, $CDCl_3$) δ 192.0, 160.6 (d, *J* = 247.2 Hz), 159.1, 138.2, 134.2, 132.5, 131.3, 130.1 (d, *J* = 8.2 Hz), 129.8 (d, *J* = 3.8 Hz), 128.1, 127.9, 124.4 (d, *J* = 3.6 Hz), 123.7, 123.5 (d, *J* = 14.2 Hz), 120.1, 115.5 (d, *J* = 21.1 Hz), 107.4, 63.9 (d, *J* = 4.5 Hz). ^{19}F NMR (376 MHz, $CDCl_3$) δ -112.35. HRMS (EI) m/z: $[M]^+$ calcd for $C_{18}H_{13}FO_2$: 280.0900; found, 280.0903.

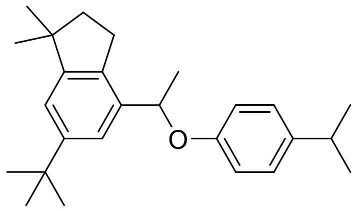

6-(*tert*-butyl)-4-(1-(4-isopropylphenoxy)ethyl)-1,1-dimethyl-2,3-dihydro-1H-indene (133): The title compound was prepared according to the general procedure and purified by column chromatography on silica gel to afford a yellow oil in 85% yield (61.9 mg); ^{1}H NMR (400 MHz, $CDCl_3$) δ 7.29 (s, 1H), 7.06 (d, *J* = 7.9 Hz, 3H), 6.79 (d, *J* = 8.5 Hz, 2H), 5.32 (q, *J* = 6.4 Hz, 1H), 2.99 – 2.77 (m, 3H), 1.94 (t, *J* = 7.2 Hz, 2H), 1.61 (d, *J* = 6.4 Hz, 3H), 1.30 (s, 9H), 1.26 (d, *J* = 12.9 Hz, 6H), 1.19 (d, *J* = 6.9 Hz, 6H). ^{13}C NMR (101 MHz, $CDCl_3$) δ 156.3, 152.7, 150.1, 140.8, 138.0, 136.4, 127.1, 120.4, 117.8, 115.7, 74.8, 43.7, 41.5, 34.8, 33.2, 31.6, 28.7, 27.9, 24.2, 22.4. IR (neat, ν/cm^{-1}) 3067, 3039, 3004, 2952, 2923, 2860, 1731, 1672, 1578, 1460, 1359, 1255, 1234, 1143, 1072, 1017, 891, 799, 743, 698, 639. HRMS (EI) m/z: $[M]^+$ calcd for $C_{26}H_{36}O$: 364.2766; found, 364.2763.

6-(1-(4-isopropylphenoxy)ethyl)-1,1,2,4,4,7-hexamethyl-1,2,3,4-tetrahydronaphthalene (134):

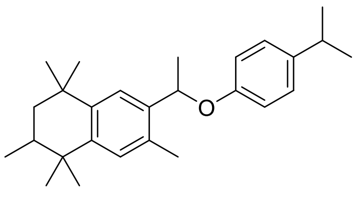

The title compound was prepared according to the general procedure and purified by column chromatography on silica gel to afford a yellow oil in 80% yield (60.5 mg); ^{1}H NMR (400 MHz, $CDCl_3$) δ 7.38 (d, *J* = 3.0 Hz, 1H), 7.12 (s, 1H), 7.08 (d, *J* = 8.6 Hz, 2H), 6.81 (dd, *J* = 8.5, 2.2 Hz, 2H), 5.46 – 5.36 (m, 1H), 2.84 (dt, *J* = 13.8, 6.9 Hz, 1H), 2.36 (s, 3H), 1.94 – 1.78 (m, 1H), 1.62 (t, *J* = 6.1 Hz, 3H), 1.32 (dd, *J* = 16.4, 9.0 Hz, 8H), 1.22 (s, 6H), 1.15

(s, 3H), 1.07 (s, 3H), 0.99 (s, 3H). ^{13}C NMR (101 MHz, $CDCl_3$) δ 156.2, 144.7, 142.6, 140.8, 137.9, 131.1, 128.8, 127.0, 123.5, 115.7, 73.5, 43.8, 37.4, 34.6, 34.1, 33.2, 32.2, 31.9, 28.6, 24.9, 24.2, 22.3, 18.8, 16.8. HRMS (EI) m/z: $[M]^+$ calcd for $C_{27}H_{38}O$: 378.2923; found, 378.2920.

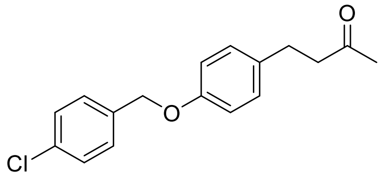

4-(4-((4-chlorobenzyl)oxy)phenyl)butan-2-one (135): The title compound was prepared according to the general procedure and purified by column chromatography on silica gel to afford a yellow solid (mp: 74-75 ºC) in 87% yield (50.1 mg); ^{1}H NMR (400 MHz, $CDCl_3$) δ 7.34 (s, 4H), 7.10 (d, *J* = 7.7 Hz, 2H), 6.87 (d, *J* = 7.8 Hz, 2H), 4.99 (s, 2H), 2.84 (t, *J* = 7.2 Hz, 2H), 2.72 (t, *J* = 7.3 Hz, 2H), 2.13 (s, 3H). ^{13}C NMR (101 MHz, $CDCl_3$) δ 207.8, 156.8, 135.6, 133.5, 133.5, 129.2, 128.6, 128.6, 114.8, 69.1, 45.2, 30.0, 28.8. IR (neat, ν/cm^{-1}) 3049, 3028, 2994, 2930, 1708, 1510, 1232, 1162, 1013, 872, 809, 772, 670. HRMS (EI) m/z: $[M]^+$ calcd for $C_{17}H_{17}ClO_2$: 288.0917; found, 288.0919.

2,4-dichloro-1-(4-chloro-2-((4-methylbenzyl)oxy)phenoxy)benzene (136): The title compound

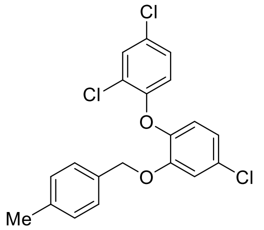

was prepared according to the general procedure and purified by column chromatography on silica gel to afford a yellow solid (mp: 79-80 ºC) in 75% yield (58.8 mg); ^{1}H NMR (400 MHz, $CDCl_3$) δ 7.41 (d, *J* = 2.5 Hz, 1H), 7.15 – 7.02 (m, 6H), 6.95 (dd, *J* = 7.2, 5.2 Hz, 2H), 6.66 (d, *J* = 8.8 Hz, 1H), 5.00 (s, 2H), 2.34 (s, 3H). ^{13}C NMR (101 MHz, $CDCl_3$) δ 152.4, 150.5, 143.4, 137.8, 132.7, 130.4, 130.1, 129.1, 127.9, 127.5, 127.1, 124.5, 122.0, 121.4, 118.0, 115.6, 70.9, 21.1. HRMS (EI) m/z: $[M]^+$ calcd for $C_{20}H_{15}Cl_3O_2$: 392.0138; found, 392.0140.

(8*R*,9*S*,13*S*,14*S*,17*S*)-3-((4-fluorobenzyl)oxy)-13-methyl-7,8,9,11,12,13,14,15,16,17-decahydro-

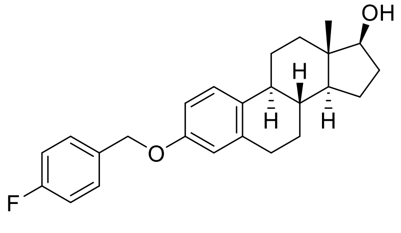

6H-cyclopenta[*a*]phenanthren-17-ol (137): The title compound was prepared according to the general procedure and purified by column chromatography on silica gel to afford a yellow solid (mp: 94-95 ºC) in 82% yield (62.4 mg); ^{1}H NMR (400 MHz, $CDCl_3$) δ 7.49 – 7.36 (m, 2H), 7.33 – 7.21 (m, 1H), 7.10 (dd, *J* = 14.6, 6.4 Hz, 2H), 6.87 – 6.69 (m, 2H), 5.03 (d, *J* = 13.2 Hz, 2H), 4.69 (d, *J* = 13.2 Hz, 1H), 3.76 (dd, *J* = 14.6, 6.7 Hz, 1H), 2.91 (s, 2H), 2.36 (s, 1H), 2.29 – 2.07 (m, 2H), 1.98 (dd, *J* = 26.4, 12.8 Hz, 2H), 1.75

(s, 2H), 1.59 – 1.07 (m, 6H), 0.83 (d, *J* = 13.1 Hz, 3H). ^{13}C NMR (101 MHz, $CDCl_3$) δ 162.4 (d, *J* = 246.0 Hz), 156.5, 138.0, 133.0, 129.0 (t, *J* = 32.5 Hz), 126.3, 115.5, 115.2, 114.8, 112.2, 81.8, 69.2, 50.0, 43.9, 43.2, 38.8, 36.7, 30.5, 29.7, 27.2, 26.3, 23.1, 11.0. ^{19}F NMR (565 MHz, $CDCl_3$) δ -114.20. IR (neat, ν/cm^{-1}) 3332, 3069, 3059, 3015, 2923, 2862, 1603, 1509, 1220, 1156, 1051, 1010, 858, 825, 669. HRMS (EI) m/z: $[M]^+$ calcd for $C_{25}H_{29}FO_2$: 380.2152; found, 380.2153.

(8*R*,9*S*,13*S*,14*S*)-3-((3-(cyclopropylmethoxy)-4-(difluoromethoxy)benzyl)oxy)-13-methyl-6,7,8,9,11,12,13,14,15,16-decahydro-17H-cyclopenta[a]phenanthren-17-one (138): The title

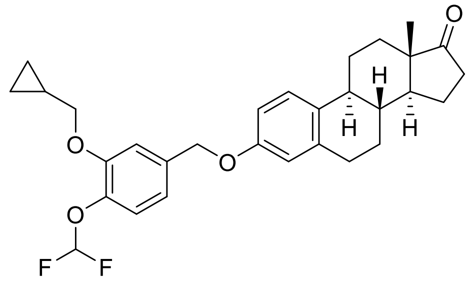

compound was prepared according to the general procedure and purified by column chromatography on silica gel to afford a yellow oil in 77% yield (76.4 mg); ^1H NMR (400 MHz, $CDCl_3$) δ 7.21 (d, *J* = 8.6 Hz, 1H), 7.15 (d, *J* = 8.1 Hz, 1H), 7.05 (s, 1H), 6.97 (d, *J* = 8.1 Hz, 1H), 6.78 (d, *J* = 8.5 Hz, 1H), 6.73 (s, 1H), 6.63 (t, *J* = 75.7 Hz, 1H), 4.98 (s, 2H), 3.89 (d, *J* = 6.8 Hz, 2H), 2.90 (dd, *J* = 10.2, 4.6 Hz, 2H), 2.51 (dd, *J* = 18.7, 8.7 Hz, 1H), 2.39 (d, *J* = 10.0 Hz, 1H), 2.25 (dd, *J* = 13.1, 6.8 Hz, 1H), 2.21 – 1.93 (m, 4H), 1.72 – 1.38 (m, 6H), 1.30 (dd, *J* = 12.6, 6.1 Hz, 1H), 0.92 (s, 3H), 0.65 (q, *J* = 5.0 Hz, 2H), 0.37 (t, *J* = 4.7 Hz, 2H). ^{13}C NMR (101 MHz, $CDCl_3$) δ 156.5, 150.5, 139.9 (t, *J* = 3.1 Hz), 137.8, 135.9, 132.5, 126.3, 122.5, 119.9, 118.8, 116.2, 114.8, 113.4, 112.3, 73.7, 69.3, 50.3, 47.9, 43.9, 38.3, 35.7, 31.5, 29.5, 26.4, 25.8, 21.5, 13.7, 10.1, 3.1. ^{19}F NMR (376 MHz, $CDCl_3$) δ -81.48 (d, *J* = 75.7 Hz). IR (neat, ν/cm^{-1}) 3076, 3039, 2957, 2925, 2865, 1733, 1611, 1512, 1400, 1371, 1239, 1115, 1030, 1004, 856, 812, 788, 668, 648. HRMS (EI) m/z: $[M]^+$ calcd for $C_{30}H_{34}F_2O_4$: 496.2425; found, 496.2424.

4.3 Characterization Data of Arylmethyl Esters

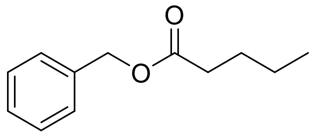

benzyl pentanoate (139): The title compound was prepared according to the general procedure and purified by column chromatography on silica gel to afford a yellow oil in 82% yield (31.5 mg); ^1H NMR (400 MHz, $CDCl_3$) δ 7.36 (s, 5H), 5.12 (s, 2H), 2.36 (d, *J* = 5.4 Hz, 2H), 1.64 (s, 2H), 1.36 (d, *J* = 5.5 Hz, 2H), 0.92 (s, 3H). ^{13}C NMR (101 MHz, $CDCl_3$) δ 173.6, 136.1, 129.6, 128.5, 128.1, 66.0, 34.0, 27.0, 22.2, 13.6.

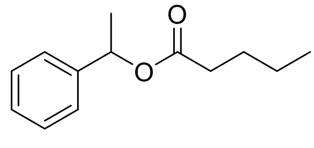

1-phenylethyl pentanoate (140): The title compound was prepared according to the general procedure and purified by column chromatography on silica gel to afford a yellow oil in 62% yield (25.6 mg); ^{1}H NMR (400 MHz, $CDCl_3$) δ 7.35 (d, *J* = 4.3 Hz, 4H), 7.30 – 7.26 (m, 1H), 5.89 (d, *J* = 6.6 Hz, 1H), 2.33 (t, *J* = 7.7 Hz, 2H), 1.61 (dt, *J* = 15.3, 7.6 Hz, 2H), 1.53 (d, *J* = 6.6 Hz, 3H), 1.33 (dq, *J* = 14.7, 7.3 Hz, 2H), 0.90 (t, *J* = 7.4 Hz, 3H). ^{13}C NMR (101 MHz, $CDCl_3$) δ 173.1, 141.8, 128.4, 127.7, 126.0, 72.0, 34.3, 27.0, 22.3, 22.2, 13.7. HRMS (EI) m/z: $[M]^+$ calcd for $C_{13}H_{18}O_2$: 206.1307; found, 206.1308.

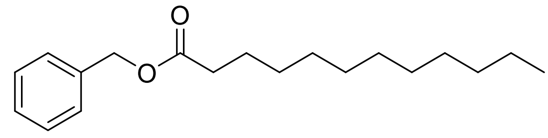

benzyl dodecanoate (141): The title compound was prepared according to the general procedure and purified by column chromatography on silica gel to afford a yellow oil in 90% yield (52.2 mg); ^{1}H NMR (400 MHz, $CDCl_3$) δ 7.35 (s, 5H), 5.11 (s, 2H), 2.35 (t, *J* = 7.2 Hz, 2H), 1.63 (d, *J* = 6.0 Hz, 2H), 1.25 (s, 16H), 0.88 (t, *J* = 5.9 Hz, 3H). ^{13}C NMR (101 MHz, $CDCl_3$) δ 173.7, 136.1, 128.5, 128.1, 127.8, 66.0, 34.3, 31.9, 29.6, 29.4, 29.3, 29.2, 29.1, 25.0, 24.7, 22.7, 14.1.

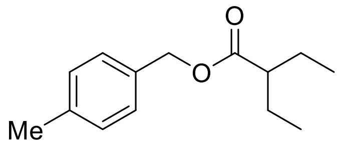

4-methylbenzyl 2-ethylbutanoate (142): The title compound was prepared according to the general procedure and purified by column chromatography on silica gel to afford a yellow oil in 85% yield (37.4 mg); ^{1}H NMR (400 MHz, $CDCl_3$) δ 7.28 (d, *J* = 7.1 Hz, 2H), 7.20 (d, *J* = 7.5 Hz, 2H), 5.13 (s, 2H), 2.38 (s, 3H), 2.33 – 2.24 (m, 1H), 1.68 (tt, *J* = 15.3, 7.6 Hz, 2H), 1.56 (tt, *J* = 13.2, 6.7 Hz, 2H), 0.92 (t, *J* = 7.3 Hz, 6H). ^{13}C NMR (101 MHz, $CDCl_3$) δ 176.1, 137.8, 133.3, 129.1, 128.2, 65.8, 48.9, 25.0, 21.1, 11.8. HRMS (EI) m/z: $[M]^+$ calcd for $C_{14}H_{20}O_2$: 220.1463; found, 220.1465.

4-methoxybenzyl 2,2,3,3-tetramethylcyclopropane-1-carboxylate (143): The title compound

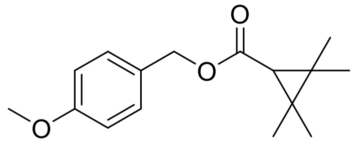

was prepared according to the general procedure and purified by column chromatography on silica gel to afford a yellow oil in 96% yield (50.3 mg); ^{1}H NMR (400 MHz, $CDCl_3$) δ 7.30 (d, *J* = 7.7 Hz, 2H), 6.89 (d, *J* = 7.6 Hz, 2H), 5.02 (s, 2H), 3.80 (s, 3H), 1.26 (s, 6H), 1.23 (s, 1H), 1.18 (s, 6H). ^{13}C NMR (101 MHz, $CDCl_3$) δ 171.9, 159.4, 129.9, 128.6, 113.8, 65.2, 55.2, 35.7, 30.0, 23.4, 16.5.

HRMS (EI) m/z: $[M]^+$ calcd for $C_{16}H_{22}O_3$: 262.1569; found, 262.1567.

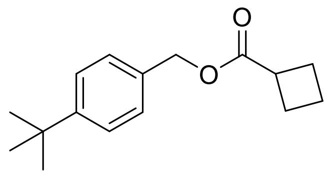

4-(*tert*-butyl)benzyl cyclobutanecarboxylate (144): The title compound was prepared according to the general procedure and purified by column chromatography on silica gel to afford a yellow oil in 95% yield (46.8 mg); ^{1}H NMR (400 MHz, $CDCl_3$) δ 7.42 (d, *J* = 7.2 Hz, 2H), 7.32 (d, *J* = 7.3 Hz, 2H), 5.12 (s, 2H), 3.31 – 3.05 (m, 1H), 2.42 – 2.29 (m, 2H), 2.23 (d, *J* = 7.9 Hz, 2H), 2.08 – 1.85 (m, 2H), 1.35 (s, 9H). ^{13}C NMR (101 MHz, $CDCl_3$) δ 175.4, 151.2, 133.2, 127.9, 125.5, 65.9, 38.1, 34.6, 31.3, 25.3, 18.4. HRMS (EI) m/z: $[M]^+$ calcd for $C_{16}H_{22}O_2$: 246.1620; found, 246.1618.

[1,1'-biphenyl]-4-ylmethyl (1*s*,3*s*)-adamantane-1-carboxylate (145): The title compound was prepared according to the general procedure and purified by column chromatography on silica gel to afford a yellow oil in 90% yield (62.3 mg); ^{1}H NMR (400 MHz, $CDCl_3$) δ 7.59 (d, *J* = 7.6 Hz, 4H), 7.43 (dd, *J* = 16.6, 7.5 Hz, 4H), 7.38 – 7.31 (m, 1H), 5.14 (s, 2H), 2.03 (s, 3H), 1.95 (s, 6H), 1.72 (s, 5H), 1.58 (s, 1H). ^{13}C NMR (101 MHz, $CDCl_3$) δ 177.5, 140.9, 140.7, 135.6, 128.8, 128.1, 127.4, 127.2, 127.1, 65.5, 40.8, 38.9, 36.5, 28.0. IR (neat, ν/cm^{-1}) 3081, 3055, 3032, 2900, 2848, 1722, 1488, 1448, 1222, 1180, 1069, 820, 759, 693, 674. HRMS (EI) m/z: $[M]^+$ calcd for $C_{24}H_{26}O_2$: 346.1933; found, 346.1929.

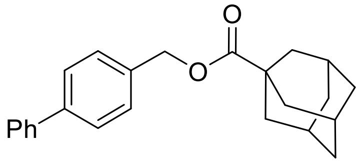

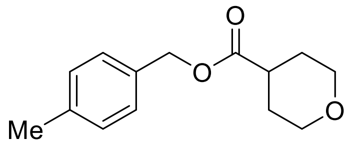

4-methylbenzyl tetrahydro-2*H*-pyran-4-carboxylate (146): The title compound was prepared according to the general procedure and purified by column chromatography on silica gel to afford a yellow oil in 95% yield (44.5 mg); ^{1}H NMR (400 MHz, $CDCl_3$) δ 7.23 (d, *J* = 7.5 Hz, 2H), 7.17 (d, *J* = 7.6 Hz, 2H), 5.09 (s, 2H), 3.95 (d, *J* = 11.5 Hz, 2H), 3.41 (t, *J* = 11.0 Hz, 2H), 2.56 (dt, *J* = 9.5, 5.0 Hz, 1H), 2.35 (s, 3H), 1.81 (dt, *J* = 19.8, 10.7 Hz, 4H). ^{13}C NMR (101 MHz, $CDCl_3$) δ 174.2, 138.0, 132.9, 129.2, 128.1, 67.0, 66.2, 40.1, 28.6, 21.1. HRMS (EI) m/z: $[M]^+$ calcd for $C_{14}H_{18}O_3$: 234.1256; found, 234.1255.

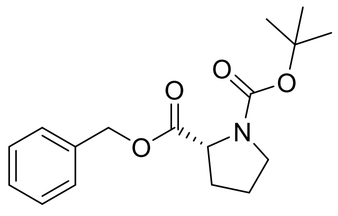

2-benzyl 1-(*tert*-butyl) pyrrolidine-1,2-dicarboxylate (147): The title compound was prepared according to the general procedure and purified by column chromatography on silica gel to afford a colorless oil in 87% yield (53.1 mg); ^{1}H NMR (400 MHz, $CDCl_3$) δ 7.32 (s, 5H), 5.28 – 5.01 (m, 2H), 4.43 – 4.11 (m, 1H), 3.62 – 3.17 (m, 2H), 2.29 – 2.07 (m, 1H), 2.02 – 1.77 (m, 3H), 1.44 (s, 3H), 1.32 (s, 6H). ^{13}C NMR (101 MHz, $CDCl_3$) δ 172.9, 153.7, 135.6, 128.5, 128.2, 127.9, 79.8, 66.5, 59.1, 46.2, 30.8, 28.1, 23.5.

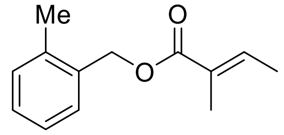

2-methylbenzyl (*E*)-2-methylbut-2-enoate (148): The title compound was prepared according to the general procedure and purified by column chromatography on silica gel to afford a colorless oil in 83% yield (33.9 mg); ^{1}H NMR (400 MHz, $CDCl_3$) δ 7.38 (d, J = 6.7 Hz, 1H), 7.31 – 7.18 (m, 3H), 6.94 (q, J = 6.5 Hz, 1H), 5.23 (s, 2H), 2.40 (s, 3H), 1.90 (s, 3H), 1.82 (d, J = 6.9 Hz, 3H). ^{13}C NMR (101 MHz, $CDCl_3$) δ 167.8, 137.4, 136.8, 134.3, 130.2, 128.9, 128.5, 128.3, 125.9, 64.6, 18.9, 14.3, 12.0. IR (neat, ν/cm^{-1}) 3069, 3027, 2975, 2928, 1708, 1651, 1455, 1380, 1251, 1131, 1072, 740, 606. HRMS (EI) m/z: [M]$^+$ calcd for $C_{13}H_{16}O_2$: 204.1150; found, 204.1145.

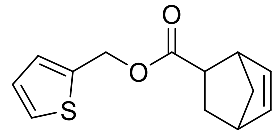

thiophen-2-ylmethyl bicyclo[2.2.1]hept-5-ene-2-carboxylate (149): The title compound was prepared according to the general procedure and purified by column chromatography on silica gel to afford a yellow oil in 56% yield (26.2 mg); ^{1}H NMR (400 MHz, $CDCl_3$) δ 7.30 (d, J = 4.8 Hz, 1H), 7.06 (s, 1H), 6.97 (d, J = 3.6 Hz, 1H), 6.17 (s, 1H), 5.89 – 5.81 (m, 1H), 5.22 (dd, J = 31.6, 13.0 Hz, 2H), 3.20 (s, 1H), 3.00 – 2.94 (m, 1H), 2.90 (s, 1H), 1.89 (dd, J = 11.3, 9.5 Hz, 1H), 1.43 (dd, J = 16.5, 5.3 Hz, 2H), 1.26 (d, J = 6.7 Hz, 1H). ^{13}C NMR (101 MHz, $CDCl_3$) δ 174.4, 138.4, 137.8, 132.3, 127.8, 126.7, 126.6, 60.3, 49.6, 45.8, 43.3, 42.6, 29.3. HRMS (EI) m/z: [M]$^+$ calcd for $C_{13}H_{14}O_2S$: 234.0715; found, 234.0718.

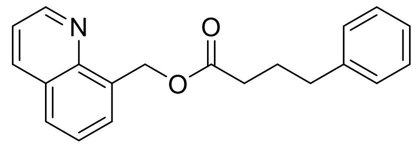

quinolin-8-ylmethyl 4-phenylbutanoate (150): The title compound was prepared according to the general procedure and purified by column chromatography on silica gel to afford a yellow oil in 86% yield (52.5 mg); ^{1}H NMR (400 MHz, $CDCl_3$) δ 9.02 – 8.93 (m, 1H), 8.18

(d, J = 8.2 Hz, 1H), 7.87 – 7.73 (m, 2H), 7.56 (t, J = 7.6 Hz, 1H), 7.45 (dd, J = 7.8, 4.0 Hz, 1H), 7.29 (t, J = 7.1 Hz, 2H), 7.20 (dd, J = 14.6, 7.2 Hz, 3H), 5.91 (s, 2H), 2.69 (t, J = 7.5 Hz, 2H), 2.47 (t, J = 7.4 Hz, 2H), 2.15 – 2.01 (m, 2H). ^{13}C NMR (101 MHz, $CDCl_3$) δ 173.3, 149.8, 146.1, 141.4, 136.1, 134.3, 128.6, 128.4, 128.3, 128.1, 128.0, 126.1, 125.8, 121.2, 62.6, 35.1, 33.7, 26.5. HRMS (EI) m/z: $[M]^+$ calcd for $C_{20}H_{19}NO_2$: 305.1416; found, 305.1414.

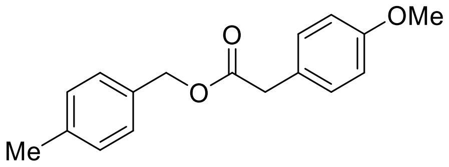

4-methylbenzyl 2-(4-methoxyphenyl)acetate (151): The title compound was prepared according to the general procedure and purified by column chromatography on silica gel to afford a colorless oil in 81% yield (43.7 mg); ^{1}H NMR (400 MHz, $CDCl_3$) δ 7.30 – 7.18 (m, 6H), 6.90 (d, J = 7.6 Hz, 2H), 5.13 (s, 2H), 3.83 (s, 3H), 3.63 (s, 2H), 2.39 (s, 3H). ^{13}C NMR (101 MHz, $CDCl_3$) δ 171.7, 158.7, 138.0, 132.9, 130.3, 129.2, 128.3, 126.0, 114.0, 66.5, 55.2, 40.4, 21.1.

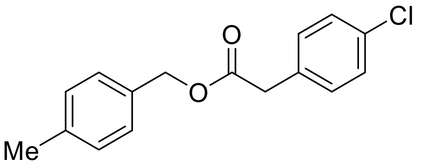

4-methylbenzyl 2-(4-chlorophenyl)acetate (152): The title compound was prepared according to the general procedure and purified by column chromatography on silica gel to afford a yellow solid (mp: 54-55 ºC) in 68% yield (37.3 mg); ^{1}H NMR (400 MHz, $CDCl_3$) δ 7.33 (d, J = 7.5 Hz, 2H), 7.25 (d, J = 6.8 Hz, 4H), 7.20 (d, J = 7.5 Hz, 2H), 5.13 (s, 2H), 3.66 (s, 2H), 2.39 (s, 3H). ^{13}C NMR (101 MHz, $CDCl_3$) δ 170.9, 138.1, 133.0, 132.6, 132.3, 130.6, 129.2, 128.6, 128.3, 66.7, 40.6, 21.1.

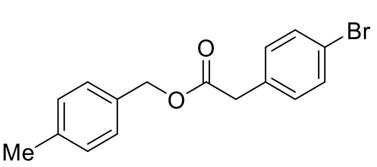

4-methylbenzyl 2-(4-bromophenyl)acetate (153): The title compound was prepared according to the general procedure and purified by column chromatography on silica gel to afford a yellow solid (mp: 54-55 ºC) in 78% yield (49.6mg); ^{1}H NMR (400 MHz, $CDCl_3$) δ 7.45 (d, J = 7.7 Hz, 2H), 7.28 – 7.21 (m, 2H), 7.20 – 7.13 (m, 4H), 5.11 (s, 2H), 3.62 (s, 2H), 2.37 (s, 3H). ^{13}C NMR (101 MHz, $CDCl_3$) δ 170.8, 138.1, 132.8, 132.6, 131.6, 131.0, 129.2, 128.3, 121.1, 66.7, 40.6, 21.1. IR (neat, ν/cm^{-1}) 3055, 3029, 3008, 2972, 2914, 2853, 1719, 1486, 1410, 1377, 1335, 1211, 1159, 1137, 1069, 1010, 966, 848, 799, 766, 681. HRMS (EI) m/z: $[M]^+$ calcd for $C_{16}H_{15}BrO_2$: 318.0255; found, 318.0253.

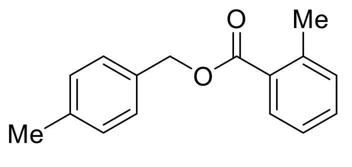

4-methylbenzyl 2-methylbenzoate (154): The title compound was prepared according to the general procedure and purified by column chromatography on silica gel to afford a yellow oil in 94% yield (45.1 mg); ^{1}H NMR (400 MHz, CDCl$_3$) δ 8.00 (d, *J* = 7.7 Hz, 1H), 7.42 (dd, *J* = 17.0, 7.7 Hz, 3H), 7.26 (dd, *J* = 14.1, 7.4 Hz, 4H), 5.36 (s, 2H), 2.66 (s, 3H), 2.41 (s, 3H). ^{13}C NMR (101 MHz, CDCl$_3$) δ 167.4, 140.2, 138.0, 133.1, 131.9, 131.6, 130.6, 129.6, 129.2, 128.3, 125.6, 66.4, 21.7, 21.2. HRMS (EI) m/z: [M]$^+$ calcd for $C_{16}H_{16}O_2$: 240.1150; found, 240.1153.

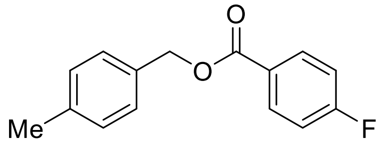

4-methylbenzyl 4-fluorobenzoate (155): The title compound was prepared according to the general procedure and purified by column chromatography on silica gel to afford a yellow oil in 72% yield (35.1 mg); ^{1}H NMR (400 MHz, CDCl$_3$) δ 8.15 – 8.03 (m, 2H), 7.35 (d, *J* = 7.4 Hz, 2H), 7.21 (d, *J* = 7.5 Hz, 2H), 7.10 (t, *J* = 8.1 Hz, 2H), 5.33 (s, 2H), 2.38 (s, 3H). ^{13}C NMR (101 MHz, CDCl$_3$) δ 167.0, 165.0 (d, *J* = 97.6 Hz), 138.1, 132.9, 132.2 (d, *J* = 9.3 Hz), 129.3, 128.4, 126.5 (d, *J* = 2.9 Hz), 115.4 (d, *J* = 22.0 Hz). ^{19}F NMR (377 MHz, CDCl$_3$) δ -105.62. IR (neat, ν/cm^{-1}) 3055, 3027, 3008, 2952, 2925, 1716, 1604, 1507, 1376, 1265, 1239, 1155, 1105, 1088, 851, 804, 764, 686, 604. HRMS (EI) m/z: [M]$^+$ calcd for $C_{15}H_{13}FO_2$: 244.0900; found, 244.0903.

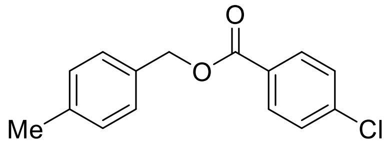

4-methylbenzyl 4-chlorobenzoate (156): The title compound was prepared according to the general procedure and purified by column chromatography on silica gel to afford a yellow solid (mp: 47-48 ºC) in 74% yield (38.5 mg); ^{1}H NMR (400 MHz, CDCl$_3$) δ 8.00 (d, *J* = 7.9 Hz, 2H), 7.40 (d, *J* = 8.0 Hz, 2H), 7.34 (d, *J* = 7.4 Hz, 2H), 7.21 (d, *J* = 7.5 Hz, 2H), 5.33 (s, 2H), 2.37 (s, 3H). ^{13}C NMR (101 MHz, CDCl$_3$) δ 165.5, 139.4, 138.2, 132.7, 131.0, 129.3, 128.6, 128.5, 128.4, 66.9, 21.2.

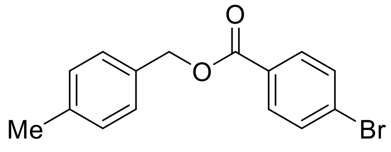

4-methylbenzyl 4-bromobenzoate (157): The title compound was prepared according to the general procedure and purified by column chromatography on silica gel to afford a yellow solid (mp: 54-55 ºC) in 80% yield (48.6 mg); ^{1}H NMR (400 MHz, CDCl$_3$) δ 7.93 (d, *J* = 7.4 Hz, 2H), 7.57 (d, *J* = 7.4 Hz, 2H), 7.34 (d, *J* = 7.2 Hz, 2H), 7.21 (d, *J* = 7.4 Hz, 2H), 5.32 (s, 2H), 2.37

(s, 3H). ^{13}C NMR (101 MHz, $CDCl_3$) δ 165.6, 138.2, 132.7, 131.6, 131.2, 129.3, 129.1, 128.4, 128.0, 66.9, 21.2.

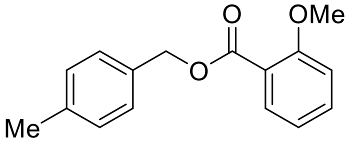

4-methylbenzyl 2-methoxybenzoate (158): The title compound was prepared according to the general procedure and purified by column chromatography on silica gel to afford a yellow oil in 92% yield (47.1mg); ^{1}H NMR (400 MHz, $CDCl_3$) δ 7.83 (d, *J* = 7.7 Hz, 1H), 7.46 (t, *J* = 7.8 Hz, 1H), 7.36 (d, *J* = 7.5 Hz, 2H), 7.19 (d, *J* = 7.5 Hz, 2H), 6.96 (t, *J* = 7.3 Hz, 2H), 5.33 (s, 2H), 3.90 (s, 3H), 2.36 (s, 3H). ^{13}C NMR (101 MHz, $CDCl_3$) δ 165.8, 159.2, 137.7, 133.4, 133.1, 131.6, 129.1, 128.1, 120.0, 111.9, 66.3, 55.8, 21.1. HRMS (EI) m/z: $[M]^+$ calcd for $C_{16}H_{16}O_3$: 256.1099; found, 256.1096.

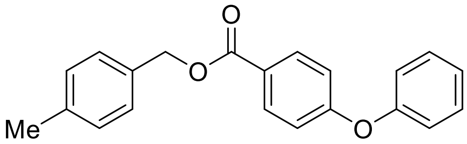

4-methylbenzyl 4-phenoxybenzoate (159): The title compound was prepared according to the general procedure and purified by column chromatography on silica gel to afford a yellow oil in 92% yield (58.5 mg); ^{1}H NMR (400 MHz, $CDCl_3$) δ 8.06 (d, *J* = 7.8 Hz, 2H), 7.38 (dd, *J* = 19.0, 7.5 Hz, 4H), 7.21 (d, *J* = 7.2 Hz, 3H), 7.07 (d, *J* = 7.4 Hz, 2H), 6.99 (d, *J* = 7.8 Hz, 2H), 5.33 (s, 2H), 2.38 (s, 3H). ^{13}C NMR (101 MHz, $CDCl_3$) δ 165.90, 161.79, 155.61, 137.97, 133.11, 131.75, 129.95, 129.20, 128.25, 124.52, 124.40, 119.98, 117.27, 66.48, 21.15. IR (neat, ν/cm^{-1}) 3055, 3027, 3010, 2954, 2921, 1721, 1585, 1488, 1267, 1232, 1156, 1091, 1006, 872, 804, 766, 691. HRMS (EI) m/z: $[M]^+$ calcd for $C_{21}H_{18}O_3$: 318.1256; found, 318.1256.

4-methylbenzyl 4-(dimethylamino)benzoate (160): The title compound was prepared according

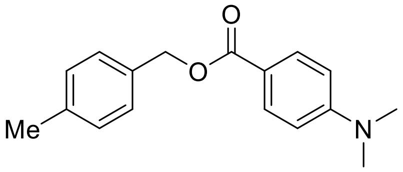

to the general procedure and purified by column chromatography on silica gel to afford a yellow solid (mp: 57-58 ºC) in 95% yield (51.1 mg); ^{1}H NMR (400 MHz, $CDCl_3$) δ 8.00 (d, *J* = 7.5 Hz, 2H), 7.38 (d, *J* = 6.8 Hz, 2H), 7.23 (t, *J* = 9.3 Hz, 2H), 6.65 (d, *J* = 7.6 Hz, 2H), 5.33 (s, 2H), 3.01 (s, 6H), 2.39 (s, 3H). ^{13}C NMR (101 MHz, $CDCl_3$) δ 166.6, 153.2, 137.5, 133.6, 131.2, 129.0, 128.0, 116.7, 110.5, 65.6, 39.8, 21.0. HRMS (EI) m/z: $[M]^+$ calcd for $C_{17}H_{19}NO_2$: 269.1416; found, 269.1422.

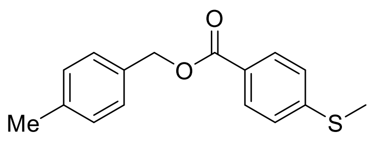

4-methylbenzyl 4-(methylthio)benzoate (161): The title compound was prepared according to the general procedure and purified by column chromatography on silica gel to afford a yellow solid (mp: 58-59 ºC) in 87% yield (47.4 mg); ^{1}H NMR (400 MHz, $CDCl_3$) δ 8.00 (d, *J* = 7.6 Hz, 2H), 7.37 (d, *J* = 7.1 Hz, 2H), 7.25 (dd, *J* = 15.3, 7.8 Hz, 4H), 5.35 (s, 2H), 2.53 (s, 3H), 2.40 (s, 3H). ^{13}C NMR (101 MHz, $CDCl_3$) δ 166.1, 145.5, 138.0, 133.1, 129.9, 129.2, 128.2, 126.3, 124.9, 66.5, 21.1, 14.7. IR (neat, ν/cm^{-1}) 3052, 3015, 2921, 2850, 1703, 1590, 1491, 1404, 1371, 1262, 1178, 1103, 1011, 931, 806, 755, 686. HRMS (EI) m/z: $[M]^+$ calcd for $C_{16}H_{16}O_2S$: 272.0871; found, 272.0875.

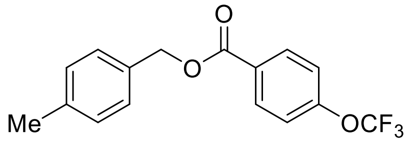

4-methylbenzyl 4-(trifluoromethoxy)benzoate (162): The title compound was prepared according to the general procedure and purified by column chromatography on silica gel to afford a yellow oil in 80% yield (49.6 mg); ^{1}H NMR (400 MHz, $CDCl_3$) δ 8.15 (d, *J* = 8.0 Hz, 2H), 7.37 (d, *J* = 7.4 Hz, 2H), 7.29 (d, *J* = 7.9 Hz, 2H), 7.24 (d, *J* = 7.5 Hz, 2H), 5.37 (s, 2H), 2.40 (s, 3H). ^{13}C NMR (101 MHz, $CDCl_3$) δ 165.2, 152.6 (d, *J* = 1.6 Hz), 138.3, 132.7, 131.7, 129.3, 128.6, 128.4, 120.3 (q, *J* = 258.6 Hz), 120.2, 67.0, 21.2. ^{19}F NMR (376 MHz, $CDCl_3$) δ -57.66. IR (neat, ν/cm^{-1}) 3057, 3034, 3010, 2954, 2925, 1721, 1609, 1505, 1249, 1209, 1162, 1096, 1021, 926, 867, 807, 768, 708, 658. HRMS (EI) m/z: $[M]^+$ calcd for $C_{16}H_{13}F_3O_3$: 310.0817; found, 310.0820.

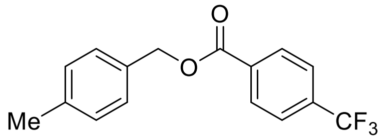

4-methylbenzyl 4-(trifluoromethyl)benzoate (163): The title compound was prepared according to the general procedure and purified by column chromatography on silica gel to afford a yellow oil in 78% yield (45.9 mg); ^{1}H NMR (400 MHz, $CDCl_3$) δ 8.18 (d, *J* = 7.8 Hz, 2H), 8.01 – 7.62 (m, 2H), 7.36 (d, *J* = 7.3 Hz, 2H), 7.23 (t, *J* = 9.4 Hz, 2H), 5.36 (s, 2H), 2.38 (s, 3H). ^{13}C NMR (101 MHz, $CDCl_3$) δ 165.2, 138.3, 134.4 (q, *J* = 32.6 Hz), 133.5, 132.6, 130.0, 129.3, 128.5, 125.3 (q, *J* = 3.7 Hz), 122.3, 67.2, 21.2. ^{19}F NMR (376 MHz, $CDCl_3$) δ -63.12. IR (neat, ν/cm^{-1}) 3048, 3024, 2921, 2846, 1716, 1582, 1406, 1321, 1274, 1163, 1131, 1100, 1064, 1016, 860, 796, 773, 702. HRMS (EI) m/z: $[M]^+$ calcd for $C_{16}H_{13}F_3O_2$: 294.0868; found, 294.0866.

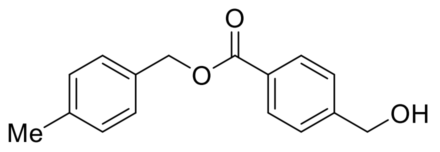

4-methylbenzyl 4-(hydroxymethyl)benzoate (164): The title compound was prepared according to the general procedure and purified by column chromatography on silica gel to afford a yellow solid (mp: 58-59 ºC) in 70% yield (31.2 mg); ^{1}H NMR (400 MHz, $CDCl_3$) δ 8.01 (d, J = 7.8 Hz, 2H), 7.40 – 7.32 (m, 4H), 7.22 (t, J = 12.0 Hz, 2H), 5.30 (s, 2H), 4.69 (s, 2H), 3.15 (s, 1H), 2.37 (s, 3H). ^{13}C NMR (101 MHz, $CDCl_3$) δ 166.4, 146.2, 137.9, 132.8, 129.7, 129.1, 129.0, 128.2, 126.2, 66.6, 64.2, 21.0. IR (neat, ν/cm^{-1}) 3285, 3055, 3022, 2957, 2918, 2855, 1712, 1610, 1444, 1412, 1361, 1265, 1175, 1091, 1044, 1013, 933, 808, 749, 697. HRMS (EI) m/z: [M]$^+$ calcd for $C_{16}H_{16}O_3$: 256.1099; found, 256.1096.

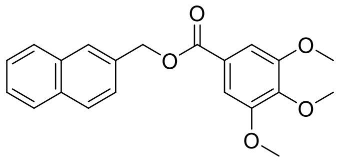

naphthalen-2-ylmethyl 3,4,5-trimethoxybenzoate (165): The title compound was prepared according to the general procedure and purified by column chromatography on silica gel to afford a yellow solid (mp: 94-96 ºC) in 79% yield (55.6 mg); ^{1}H NMR (400 MHz, $CDCl_3$) δ 7.89 (s, 1H), 7.84 (s, 3H), 7.55 (d, J = 8.2 Hz, 1H), 7.48 (s, 2H), 7.38 (d, J = 1.0 Hz, 2H), 5.52 (s, 2H), 3.92 (s, 3H), 3.87 (s, 6H). ^{13}C NMR (101 MHz, $CDCl_3$) δ 165.8, 152.8, 142.2, 133.3, 133.0, 132.9, 128.2, 127.8, 127.5, 127.2, 126.1, 126.0, 125.7, 124.9, 106.8, 66.8, 60.6, 56.0. HRMS (EI) m/z: [M]$^+$ calcd for $C_{21}H_{20}O_5$: 352.1311; found, 352.1309.

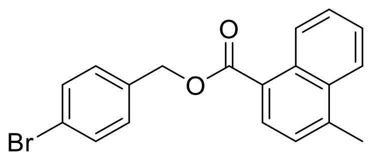

4-bromobenzyl 4-methyl-1-naphthoate (166): The title compound was prepared according to the general procedure and purified by column chromatography on silica gel to afford a yellow solid (mp 58-59 ºC) in 90% yield (60.7 mg); ^{1}H NMR (400 MHz, $CDCl_3$) δ 9.05 (d, J = 8.5 Hz, 1H), 8.14 (d, J = 7.4 Hz, 1H), 8.05 (d, J = 8.3 Hz, 1H), 7.67 – 7.57 (m, 2H), 7.53 (d, J = 7.9 Hz, 2H), 7.35 (dd, J = 14.9, 7.7 Hz, 3H), 5.39 (s, 2H), 2.74 (s, 3H). ^{13}C NMR (101 MHz, $CDCl_3$) δ 167.0, 140.6, 135.2, 132.8, 131.7, 131.4, 130.2, 129.8, 127.4, 126.2, 126.0, 125.4, 124.8, 124.4, 122.1, 65.7, 20.0. IR (neat, ν/cm^{-1}) 3074, 3017, 2961, 2921, 1710, 1590, 1453, 1279, 1242, 1227, 1185, 1009, 797, 785, 764, 642. HRMS (EI) m/z: [M]$^+$ calcd for $C_{19}H_{15}BrO_2$: 354.0255; found, 337.0242.

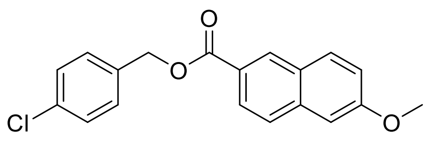

4-chlorobenzyl 6-methoxy-2-naphthoate (167): The title compound was prepared according to the general procedure and purified by column chromatography on silica gel to afford a yellow solid (mp: 87-88 ºC) in 74% yield (48.2 mg); ^{1}H NMR (400 MHz, $CDCl_3$) δ 8.54 (s, 1H), 8.05 (d, *J* = 8.5 Hz, 1H), 7.82 (d, *J* = 8.9 Hz, 1H), 7.74 (d, *J* = 8.5 Hz, 1H), 7.39 (dd, *J* = 19.2, 7.9 Hz, 4H), 7.19 (d, *J* = 8.9 Hz, 1H), 7.13 (s, 1H), 5.36 (s, 2H), 3.92 (s, 3H). ^{13}C NMR (101 MHz, $CDCl_3$) δ 166.5, 159.6, 137.2, 134.7, 134.0, 130.9, 130.8, 129.5, 128.7, 127.8 126.8, 125.8, 124.8, 119.6, 105.6, 65.7, 55.3. IR (neat, ν/cm^{-1}) 3076, 3052, 3020, 2945, 2921, 2850, 1709, 1625, 1483, 1392, 1278, 1255, 1199, 1095, 1029, 997, 916, 860, 822, 806, 747. HRMS (EI) m/z: [M]$^+$ calcd for $C_{19}H_{15}ClO_3$: 326.0710; found, 326.0711.

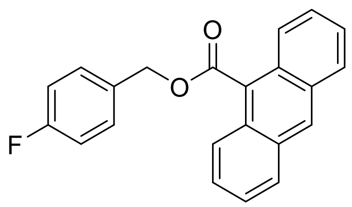

4-fluorobenzyl anthracene-9-carboxylate (168): The title compound was prepared according to the general procedure and purified by column chromatography on silica gel to afford a yellow solid (mp: 113-114 ºC) in 76% yield (50.2 mg); ^{1}H NMR (400 MHz, $CDCl_3$) δ 8.48 (s, 1H), 8.01 (dd, *J* = 18.0, 8.4 Hz, 4H), 7.63 – 7.41 (m, 6H), 7.13 (t, *J* = 8.2 Hz, 2H), 5.64 (s, 2H). ^{13}C NMR (101 MHz, $CDCl_3$) δ 169.3, 162.7 (d, *J* = 247.2 Hz), 131.4 (d, *J* = 3.2 Hz), 130.8, 130.6 (d, *J* = 8.3 Hz), 129.4, 128.6, 128.4, 127.3, 126.9, 125.3, 124.7, 115.5 (d, *J* = 21.6 Hz), 66.5. ^{19}F NMR (376 MHz, $CDCl_3$) δ -112.94. IR (neat, ν/cm^{-1}) 3054, 3041, 2961, 2921, 1714, 1519, 1515, 1220, 1197, 1151, 1006, 832, 790, 729, 632. HRMS (EI) m/z: [M]$^+$ calcd for $C_{22}H_{15}FO_2$: 330.1056; found, 330.1058.

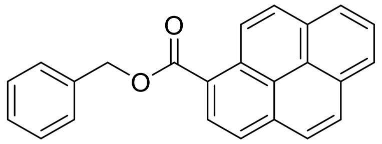

benzyl pyrene-1-carboxylate (169): The title compound was prepared according to the general procedure and purified by column chromatography on silica gel to afford a yellow solid (mp: 96-97 ºC) in 75% yield (50.4 mg); ^{1}H NMR (400 MHz, $CDCl_3$) δ 9.30 (d, *J* = 9.4 Hz, 1H), 8.65 (d, *J* = 8.1 Hz, 1H), 8.20 (t, *J* = 8.9 Hz, 3H), 8.09 (t, *J* = 8.5 Hz, 2H), 8.01 (dd, *J* = 11.9, 7.4 Hz, 2H), 7.60 (d, *J* = 7.4 Hz, 2H), 7.47 (t, *J* = 7.3 Hz, 2H), 7.44 – 7.36 (m, 1H), 5.58 (s, 2H). ^{13}C NMR (101 MHz, $CDCl_3$) δ 167.6, 136.2, 134.3, 131.2, 130.9, 130.2, 129.5, 129.4, 128.6, 128.4, 128.3, 128.2, 127.0, 126.3, 126.2, 126.1, 124.8, 124.7, 124.0, 124.0, 123.1, 66.9. (*59*)

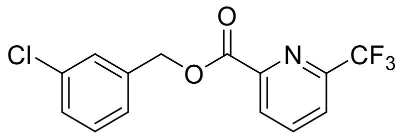

3-chlorobenzyl 6-(trifluoromethyl)picolinate (170): The title compound was prepared according to the general procedure and purified by column chromatography on silica gel to afford a yellow solid (mp: 53-54 ºC) in 61% yield (38.4 mg); ^{1}H NMR (400 MHz, $CDCl_3$) δ 8.28 (d, J = 7.7 Hz, 1H), 8.04 (t, J = 7.7 Hz, 1H), 7.86 (d, J = 7.7 Hz, 1H), 7.46 (s, 1H), 7.32 (d, J = 14.4 Hz, 3H), 5.42 (s, 2H). ^{13}C NMR (101 MHz, $CDCl_3$) δ 163.7, 148.6 (q, J = 35.6 Hz), 148.3, 138.8, 137.3, 134.5, 129.9, 128.6, 128.4, 127.6, 126.4, 123.6 (d, J = 2.5 Hz), 121.0 (d, J = 274.7 Hz), 66.8. ^{19}F NMR (376 MHz, $CDCl_3$) δ -67.7. HRMS (EI) m/z: $[M]^+$ calcd for $C_{14}H_9ClF_3NO_2$: 315.0274; found, 315.0275.

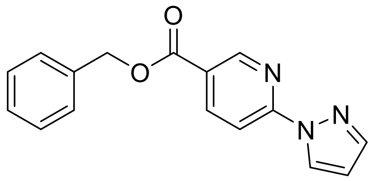

benzyl 6-(1*H*-pyrazol-1-yl)nicotinate (171): The title compound was prepared according to the general procedure and purified by column chromatography on silica gel to afford a yellow solid (mp: 94-95 ºC) in 63% yield (35.2 mg); ^{1}H NMR (400 MHz, $CDCl_3$) δ 9.06 (s, 1H), 8.61 (s, 1H), 8.41 (d, J = 8.6 Hz, 1H), 8.04 (d, J = 8.6 Hz, 1H), 7.77 (s, 1H), 7.46 (d, J = 6.9 Hz, 2H), 7.43 – 7.32 (m, 3H), 6.49 (s, 1H), 5.40 (s, 2H). ^{13}C NMR (101 MHz, $CDCl_3$) δ 164.6, 153.9, 150.3, 143.1, 140.0, 135.5, 128.7, 128.5, 128.3, 127.7, 123.6, 111.7, 108.6, 67.0. HRMS (EI) m/z: $[M]^+$ calcd for $C_{16}H_{13}N_3O_2$: 279.1008; found, 279.1008.

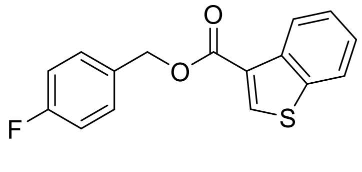

4-fluorobenzyl benzo[b]thiophene-3-carboxylate (172): The title compound was prepared according to the general procedure and purified by column chromatography on silica gel to afford a yellow solid (mp: 63-64 ºC) in 85% yield (48.6 mg); ^{1}H NMR (400 MHz, $CDCl_3$) δ 8.61 (d, J = 8.1 Hz, 1H), 8.40 (s, 1H), 7.86 (d, J = 7.9 Hz, 1H), 7.55 – 7.37 (m, 4H), 7.09 (t, J = 7.9 Hz, 2H), 5.37 (s, 2H). ^{13}C NMR (101 MHz, $CDCl_3$) δ 163.1 (d, J = 152.2 Hz), 161.4, 139.9, 136.9, 136.6, 131.9 (d, J = 3.2 Hz), 130.20 (d, J = 8.3 Hz), 126.8, 125.4, 125.0, 124.6, 122.4, 115.5 (d, J = 21.6 Hz), 65.6. ^{19}F NMR (376 MHz, $CDCl_3$) δ -113.43. IR (neat, ν/cm^{-1}) 3121, 3064, 3041, 2959, 2918, 2848, 1712, 1604, 1510, 1463, 1359, 1218, 1147, 1063, 1028, 856, 827, 762, 736, 721, 668. HRMS (EI) m/z: $[M]^+$ calcd for $C_{16}H_{11}FO_2S$: 286.0464; found, 286.0464.

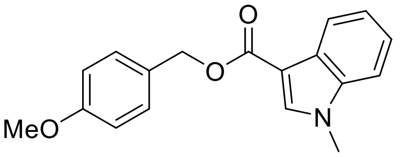

4-methoxybenzyl 1-methyl-1*H*-indole-3-carboxylate (173): The title compound was prepared according to the general procedure and purified by column chromatography on silica gel to afford a yellow solid (mp: 71-72 ºC) in 83% yield (49.0 mg); ^{1}H NMR (400 MHz, $CDCl_3$) δ 8.24 (d, *J* = 2.4 Hz, 1H), 7.73 (s, 1H), 7.46 (d, *J* = 7.9 Hz, 2H), 7.29 (s, 3H), 6.95 (d, *J* = 7.9 Hz, 2H), 5.36 (s, 2H), 3.81 (s, 3H), 3.69 (s, 3H). ^{13}C NMR (101 MHz, $CDCl_3$) δ 164.6, 159.3, 137.0, 135.1, 129.7, 128.8, 126.4, 122.5, 121.7, 121.4, 113.7, 109.6, 106.5, 65.0, 55.0, 33.0. (*60*)

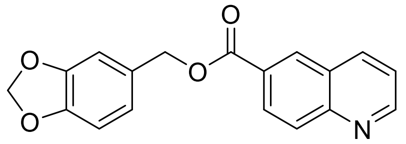

benzo[*d*][1,3]dioxol-5-ylmethyl quinoline-6-carboxylate (174): The title compound was prepared according to the general procedure and purified by column chromatography on silica gel to afford a brown solid (mp: 124-125 ºC) in 75% yield (46.1 mg); ^{1}H NMR (400 MHz, $CDCl_3$) δ 8.97 (d, *J* = 1.4 Hz, 1H), 8.56 (s, 1H), 8.29 (d, *J* = 8.8 Hz, 1H), 8.22 (d, *J* = 8.3 Hz, 1H), 8.11 (d, *J* = 8.8 Hz, 1H), 7.43 (dd, *J* = 7.3, 3.4 Hz, 1H), 6.95 (d, *J* = 13.2 Hz, 2H), 6.80 (d, *J* = 7.8 Hz, 1H), 5.95 (s, 2H), 5.30 (s, 2H). ^{13}C NMR (101 MHz, $CDCl_3$) δ 165.8, 152.4, 150.0, 147.8, 147.7, 137.3, 131.0, 129.7, 129.5, 128.9, 128.0, 127.3, 122.3, 121.8 109.0, 108.2, 101.1, 67.0. IR (neat, ν/cm^{-1}) 3071, 3039, 2963, 2900, 1709, 1620, 1498, 1446, 1359, 1260, 1230, 1185, 1093, 1037, 959, 938, 785, 646. HRMS (EI) m/z: $[M]^+$ calcd for $C_{18}H_{13}NO_4$: 307.0845; found, 307.0845.

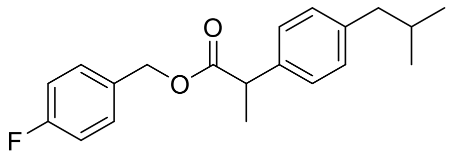

4-fluorobenzyl 2-(4-isobutylphenyl)propanoate (175): The title compound was prepared according to the general procedure and purified by column chromatography on silica gel to afford a yellow oil in 73% yield (45.9 mg); ^{1}H NMR (400 MHz, $CDCl_3$) δ 7.18 (d, *J* = 6.6 Hz, 4H), 7.09 (d, *J* = 7.2 Hz, 2H), 6.97 (t, *J* = 8.0 Hz, 2H), 5.07 (s, 2H), 3.74 (d, *J* = 7.0 Hz, 1H), 2.46 (d, *J* = 6.9 Hz, 2H), 1.96 – 1.75 (m, 1H), 1.50 (d, *J* = 6.9 Hz, 3H), 0.91 (d, *J* = 6.2 Hz, 6H). ^{13}C NMR (101 MHz, $CDCl_3$) δ 174.4, 162.5 (d, *J* = 246.5 Hz), 140.6, 137.5, 131.9 (d, *J* = 3.3 Hz), 129.7 (d, *J* = 8.2 Hz), 129.3, 127.2, 115.3 (d, *J* = 21.6 Hz), 65.5, 45.1, 45.0, 30.2, 22.3, 18.3. ^{19}F NMR (377 MHz, $CDCl_3$) δ -114.00. IR (neat, ν/cm^{-1}) 3050, 3012, 2949, 2928, 2870, 1733, 1606, 1511, 1455, 1380, 1222, 1151, 1091, 1013, 823, 764, 721. HRMS (EI) m/z: $[M]^+$ calcd for $C_{20}H_{23}FO_2$: 314.1682; found, 314.1681.

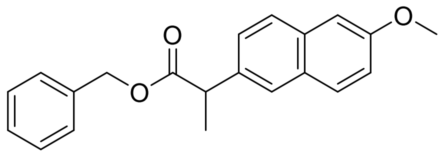

benzyl 2-(6-methoxynaphthalen-2-yl)propanoate (176): The title compound was prepared according to the general procedure and purified by column chromatography on silica gel to afford a yellow solid (mp: 54-55 ºC) in 85% yield (54.4 mg); ^{1}H NMR (400 MHz, CDCl$_3$) δ 7.77 – 7.67 (m, 3H), 7.46 (d, J = 8.4 Hz, 1H), 7.35 – 7.24 (m, 5H), 7.22 – 7.12 (m, 2H), 5.17 (q, J = 12.5 Hz, 2H), 3.99 – 3.94 (m, 1H), 3.93 (s, 3H), 1.65 (d, J = 7.1 Hz, 3H). ^{13}C NMR (101 MHz, CDCl$_3$) δ 174.3, 157.6, 135.9, 135.5, 133.6, 129.2, 128.9, 128.4, 128.0, 127.8, 127.1, 126.2, 125.9, 118.9, 105.5, 66.4, 55.2, 45.4, 18.5. IR (neat, ν/cm^{-1}) 3086, 3029, 2982, 2933, 1743, 1604, 1266, 1227, 1152, 1093, 1025, 856, 820, 729, 693. HRMS (EI) m/z: [M]$^+$ calcd for $C_{21}H_{20}O_3$: 320.1412; found, 320.1414.

4-methylbenzyl 2-(3-(4-chlorobenzoyl)phenoxy)-2-methylpropanoate (177): The title compound was prepared according to the general procedure and purified by column chromatography on silica gel to afford a white solid (mp: 69-70 ºC) in 65% yield (54.9 mg); ^{1}H NMR (400 MHz, CDCl$_3$) δ 7.71 – 7.66 (m, 2H), 7.66 – 7.61 (m,2H), 7.49 – 7.36 (m, 2H), 7.14 (d, J = 8.1 Hz, 2H), 7.09 (d, J = 8.0 Hz, 2H), 6.84 – 6.66 (m, 2H), 5.16 (s, 2H), 2.29 (s, 3H), 1.67 (s, 6H). ^{13}C NMR (101 MHz, CDCl$_3$) δ 194.0, 173.3, 159.4, 138.2, 138.1, 136.3, 132.0, 131.8, 131.0, 130.1, 129.1, 128.5, 128.4, 117.1, 79.3, 67.2, 25.3, 21.0. IR (neat, ν/cm^{-1}) 3052, 3012, 2972, 2949, 2890, 1731, 1646, 1594, 1505, 1388, 1277, 1242, 1168, 1140, 1088, 929, 847, 802, 762, 649. HRMS (EI) m/z: [M]$^+$ calcd for $C_{25}H_{23}ClO_4$: 422.1285; found, 422.1286.

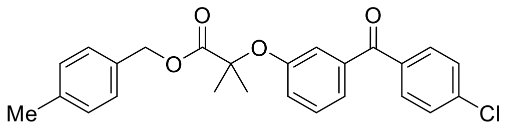

3-chlorobenzyl-5-(2,5-dimethylphenoxy)-2,2-dimethylpentanoate (178): The title compound was prepared according to the general procedure and purified by column chromatography on silica gel to afford a yellow oil in 71% yield (53.1 mg); ^{1}H NMR (400 MHz, CDCl$_3$) δ 7.35 (s, 1H), 7.29 (d, J = 4.0 Hz, 2H), 7.24 (s, 1H), 7.01 (d, J = 7.4 Hz, 1H), 6.68 (d, J = 7.4 Hz, 1H), 6.62 (s, 1H), 5.09 (s, 2H), 3.92 (d, J = 5.6 Hz, 2H), 2.32 (s, 3H), 2.18 (s, 3H), 1.76 (s, 4H), 1.28 (s, 6H). ^{13}C NMR (101 MHz, CDCl$_3$) δ 177.3, 156.9, 138.3, 136.3, 134.3, 130.2, 129.7, 128.1, 127.8, 125.8, 123.5, 120.7, 111.9, 67.8, 65.2, 42.1, 37.1, 25.1, 25.0, 21.3 15.7. HRMS

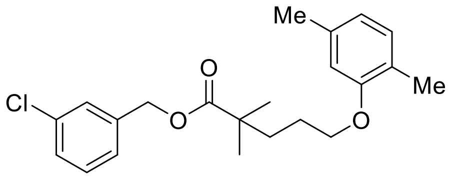

(EI) m/z: $[M]^+$ calcd for $C_{22}H_{27}ClO_3$: 374.1649; found, 374.1650.

4-methylbenzyl 4-(*N,N*-dipropylsulfamoyl)benzoate (179): The title compound was prepared according to the general procedure and purified by column chromatography on silica gel to afford a green oil in 93% yield (72.4 mg); ^{1}H NMR (400 MHz, $CDCl_3$) δ 8.16 (d, *J* = 7.9 Hz, 2H), 7.85 (d, *J* = 7.9 Hz, 2H), 7.34 (d, *J* = 7.4 Hz, 2H), 7.19 (d, *J* = 7.5 Hz, 2H), 5.34 (s, 2H), 3.09 (t, *J* = 7.5 Hz, 4H), 2.35 (s, 3H), 1.68 – 1.40 (m, 4H), 0.86 (t, *J* = 7.3 Hz, 6H). ^{13}C NMR (101 MHz, $CDCl_3$) δ 164.9, 144.2, 138.2, 133.4, 132.4, 130.2, 129.2, 128.4, 126.8, 67.1, 49.8, 21.8, 21.1, 11.0. IR (neat, ν/cm^{-1}) 3074, 3041, 2963, 2925, 2871, 1710, 1599, 1336, 1267, 1157, 1103, 1084, 983, 855, 811, 760, 736, 689. HRMS (EI) m/z: $[M]^+$ calcd for $C_{21}H_{27}NO_4S$: 389.1661; found, 389.1664.

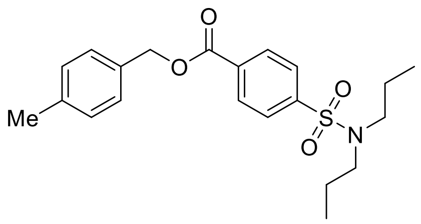

4-methylbenzyl 2-((2,3-dimethylphenyl)amino)benzoate (180): The title compound was prepared according to the general procedure and purified by column chromatography on silica gel to afford a yellow solid (mp: 63-64 ºC) in 85% yield (58.7 mg); ^{1}H NMR (400 MHz, $CDCl_3$) δ 9.39 (s, 1H), 8.12 (dd, *J* = 8.0, 1.4 Hz, 1H), 7.46 (d, *J* = 7.9 Hz, 2H), 7.36 – 7.25 (m, 4H), 7.20 (t, *J* = 7.6 Hz, 1H), 7.12 (d, *J* = 7.3 Hz, 1H), 6.87 (d, *J* = 8.5 Hz, 1H), 6.74 (t, *J* = 7.5 Hz, 1H), 5.42 (s, 2H), 2.47 (s, 3H), 2.43 (s, 3H), 2.29 (s, 3H). ^{13}C NMR (101 MHz, $CDCl_3$) δ 168.4, 149.5, 138.7, 138.1, 137.9, 134.1, 133.1, 132.4, 131.5, 129.2, 128.2, 126.7, 125.9, 123.0, 116.0, 113.6, 110.7, 66.1, 21.2, 20.6, 14.0. IR (neat, ν/cm^{-1}) 3297, 3055, 3021, 3006, 2961, 2919, 2853, 1674, 1574, 1503, 1448, 1378, 1317, 1249, 1230, 1138, 1080, 959, 812, 785, 745, 722, 698. HRMS (EI) m/z: $[M]^+$ calcd for $C_{23}H_{23}NO_2$: 345.1729; found, 345.1733.

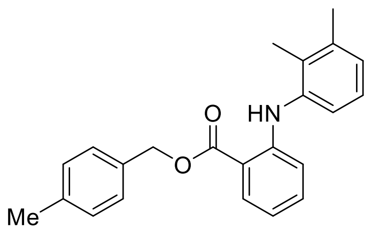

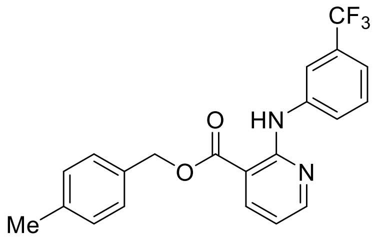

4-methylbenzyl-2-((3-(trifluoromethyl)phenyl)amino)nicotinate (181): The title compound was prepared according to the general procedure and purified by column chromatography on silica gel to afford a white solid (mp: 98-99 ºC) in 74% yield (57.1 mg); ^{1}H NMR (400 MHz, $CDCl_3$) δ 10.35 (s, 1H), 8.40 (dd, *J* = 4.7, 2.0 Hz, 1H), 8.29 (dd,

J = 7.8, 2.0 Hz, 1H), 8.07 (s, 1H), 7.88 (d, J = 8.1 Hz, 1H), 7.42 (t, J = 7.9 Hz, 1H), 7.35 (d, J = 8.0 Hz, 2H), 7.28 (d, J = 7.7 Hz, 1H), 7.22 (d, J = 7.9 Hz, 2H), 6.76 (dd, J = 7.8, 4.8 Hz, 1H), 5.34 (s, 2H), 2.38 (s, 3H). ^{13}C NMR (101 MHz, $CDCl_3$) δ 167.3, 155.8, 153.1, 140.4, 138.5, 132.4, 131.1 (d, J = 32.1 Hz), 129.4, 129.2, 128.5, 125.5, 123.5, 122.8, 119.0 (q, J = 3.9 Hz), 117.1 (q, J = 3.9 Hz), 114.0, 107.5, 67.1, 21.2. ^{19}F NMR (376 MHz, $CDCl_3$) δ -62.58. IR (neat, ν/cm^{-1}) 3269, 3034, 2923, 1684, 1620, 1582, 1465, 1331, 1286, 1254, 1140, 1108, 942, 863, 792, 766, 696. HRMS (EI) m/z: $[M]^+$ calcd for $C_{21}H_{17}F_3N_2O_2$: 386.1242; found, 386.1241.

2-fluoro-4-methoxybenzyl 4-amino-5-chloro-2,3-dihydrobenzofuran-7-carboxylate (182): The

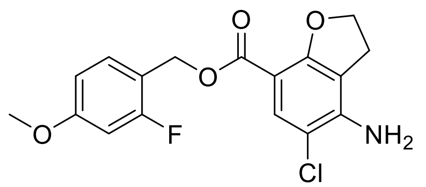

title compound was prepared according to the general procedure and purified by column chromatography on silica gel to afford a brown oil in 77% yield (54.1 mg); ^{1}H NMR (400 MHz, $CDCl_3$) δ 7.65 (s, 1H), 7.38 (t, J = 8.5 Hz, 1H), 6.64 (ddd, J = 13.8, 10.1, 2.1 Hz, 2H), 5.27 (s, 2H), 4.74 (t, J = 8.8 Hz, 2H), 4.35 (s, 2H), 3.77 (s, 3H), 2.97 (t, J = 8.8 Hz, 2H). ^{13}C NMR (101 MHz, $CDCl_3$) δ 164.0, 163.0, 160.9 (d, J = 2.9 Hz), 160.8 (d, J = 49.3 Hz), 143.6, 131.7 (d, J = 5.8 Hz), 130.4, 115.4 (d, J = 15.2 Hz), 111.7, 110.6, 109.7 (d, J = 3.1 Hz), 103.7, 101.5 (d, J = 25.0 Hz), 72.7, 59.7, 55.5, 26.8. ^{19}F NMR (376 MHz, $CDCl_3$) δ -115.70. IR (neat, ν/cm^{-1}) 3375, 3083, 3059, 2923, 2853, 1695, 1627, 1509, 1432, 1250, 1121, 1029, 997, 945, 776, 724, 693, 667. HRMS (EI) m/z: $[M]^+$ calcd for $C_{17}H_{15}ClFNO_4$: 351.0674; found, 351.0675.

2-fluoro-4-methoxybenzyl (3-(trifluoromethyl)benzoyl)glycinate (183): The title compound was

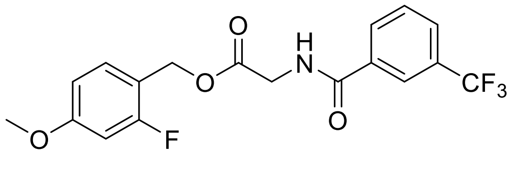

prepared according to the general procedure and purified by column chromatography on silica gel to afford a yellow solid (mp: 103-104 ºC) in 69% yield (53.1 mg); ^{1}H NMR (400 MHz, $CDCl_3$) δ 8.05 (s, 1H), 7.95 (d, J = 7.8 Hz, 1H), 7.73 (d, J = 7.7 Hz, 1H), 7.52 (t, J = 7.8 Hz, 1H), 7.28 (t, J = 8.8 Hz, 1H), 6.99 (s, 1H), 6.64 (ddd, J = 14.0, 10.1, 2.3 Hz, 2H), 5.20 (s, 2H), 4.23 (d, J = 5.2 Hz, 2H), 3.78 (s, 3H). ^{13}C NMR (101 MHz, $CDCl_3$) δ 169.8, 166.1, 162.4 (d, J = 154.9 Hz), 161.1 (d, J = 82.8 Hz), 134.4, 131.9 (d, J = 5.5 Hz), 131.1 (q, J = 32.9 Hz), 130.3, 129.2, 128.3 (q, J = 3.6 Hz), 124.1 (q, J = 3.8 Hz), 123.6 (d, J = 272.5 Hz), 114.1

(d, J = 15.2 Hz), 109.9 (d, J = 3.1 Hz), 101.7 (d, J = 24.9 Hz), 61.2, 55.6, 41.8. ^{19}F NMR (376 MHz, $CDCl_3$) δ -62.80, -115.47 (dd, J = 11.3, 8.9 Hz). IR (neat, ν/cm^{-1}) 3337, 3086, 3012, 2918, 2848, 1740, 1643, 1554, 1514, 1325, 1283, 1156, 1116, 1064, 1032, 940, 831, 759, 693, 649. HRMS (EI) m/z: [M]$^+$ calcd for $C_{18}H_{15}F_4NO_4$: 385.0937; found, 385.0936.

3-(cyclopropylmethoxy)-4-(difluoromethoxy)benzyl 2-(2-fluoro-[1,1'-biphenyl]-4-yl)propanoate (184): The title compound was prepared according to the general procedure and purified by column chromatography on silica gel to afford a yellow oil in 83% yield (78.1 mg); ^{1}H NMR (400 MHz, $CDCl_3$) δ 7.54 (d, J = 7.8 Hz, 2H), 7.44 (dd, J = 13.9, 6.2 Hz, 2H), 7.40 – 7.34 (m, 2H), 7.13 (dd, J = 11.4, 8.0 Hz, 3H), 6.85 (d, J = 7.2 Hz, 2H), 6.61 (t, J = 75.6 Hz, 1H), 5.36 – 4.72 (m, 2H), 3.81 (dd, J = 15.9, 7.0 Hz, 3H), 1.57 (t, J = 7.6 Hz, 3H), 1.27 – 1.18 (m, 1H), 0.60 (q, J = 5.4 Hz, 2H), 0.31 (q, J = 5.0 Hz, 2H). ^{13}C NMR (101 MHz, $CDCl_3$) δ 173.6, 159.6 (d, J = 248.5 Hz), 150.5, 141.5 (d, J = 7.6 Hz), 140.2 (t, J = 3.1 Hz), 135.3, 134.4, 130.8 (d, J = 4.0 Hz), 128.9 (d, J = 2.9 Hz), 128.4, 127.7, 123.5 (d, J = 3.4 Hz), 122.6, 120.6, 118.7, 116.1, 115.3 (d, J = 23.7 Hz), 114.0, 73.8, 66.0, 45.0, 18.2, 10.1, 3.1. ^{19}F NMR (376 MHz, $CDCl_3$) δ -81.62 (d, J = 75.6 Hz), -117.44 (d, J = 8.8 Hz). HRMS (EI) m/z: [M]$^+$ calcd for $C_{27}H_{25}F_3O_4$: 470.1705; found, 470.1705.

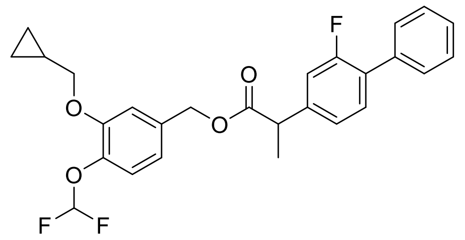

4-(methoxymethyl)benzyl (1*S*,3*S*)-2,2-dimethyl-3-(2-methylprop-1-en-1-yl)cyclopropane-1-carboxylate (185): The title compound was prepared according to the general procedure and purified by column chromatography on silica gel to afford a yellow oil in 81% yield (49.0 mg); ^{1}H NMR (400 MHz, $CDCl_3$) δ 7.34 (t, J = 5.5 Hz, 4H), 5.12 (t, J = 8.8 Hz, 2H), 4.88 (d, J = 7.8 Hz, 1H), 4.45 (s, 2H), 3.38 (s, 3H), 2.10 – 2.04 (m, 1H), 1.70 (d, J = 4.9 Hz, 6H), 1.44 (d, J = 5.4 Hz, 1H), 1.26 (s, 3H), 1.12 (s, 3H). ^{13}C NMR (101 MHz, $CDCl_3$) δ 172.4, 138.1, 135.8, 135.6, 128.3, 127.8, 121.1, 74.4, 65.9, 58.1, 34.8, 32.9, 28.8, 25.5, 22.2, 20.5, 18.5. IR (neat, ν/cm^{-1}) 3067, 2977, 2952, 2925, 2871, 1719, 1436, 1377, 1277, 1154, 1109, 1017, 959, 843, 756, 729, 702. HRMS (EI) m/z: [M]$^+$ calcd for $C_{19}H_{26}O_3$: 302.1882; found, 302.1885.

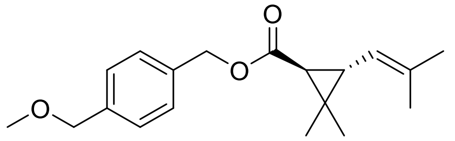

3-phenoxybenzyl (1*S*,3*S*)-2,2-dimethyl-3-(2-methylprop-1-en-1-yl)cyclopropane-1-

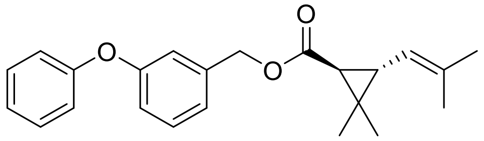

carboxylate (186): The title compound was prepared according to the general procedure and purified by column chromatography on silica gel to afford a yellow oil in 74% yield (51.8 mg); ^{1}H NMR (400 MHz, $CDCl_3$) δ 7.39 – 7.30 (m, 3H), 7.15 – 7.07 (m, 2H), 7.02 (d, *J* = 7.7 Hz, 3H), 6.95 (dd, *J* = 8.1, 2.4 Hz, 1H), 5.09 (s, 2H), 4.90 (d, *J* = 7.8 Hz, 1H), 2.08 (dd, *J* = 7.5, 5.7 Hz, 1H), 1.70 (t, *J* = 6.8 Hz, 6H), 1.45 (d, *J* = 5.4 Hz, 1H), 1.26 (s, 3H), 1.13 (s, 3H). ^{13}C NMR (101 MHz, $CDCl_3$) δ 172.3, 157.5, 157.0, 138.4, 135.6, 129.8, 123.4, 122.6, 121.0, 119.0, 118.9, 118.3, 118.2, 65.6, 34.7, 32.9, 28.8, 25.5, 22.1, 20.4, 18.5. HRMS (EI) m/z: $[M]^+$ calcd for $C_{23}H_{26}O_3$: 350.1882; found, 350.1882.

4.4 Characterization Data of Arylmethyl Phosphine Oxides.

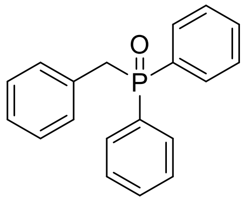

benzyldiphenylphosphine oxide (187): The title compound was prepared according to the general procedure and purified by column chromatography on silica gel to afford a white solid (mp: 187-189 ºC) in 90% yield (52.6 mg); ^{1}H NMR (400 MHz, $CDCl_3$) δ 7.69 (dd, *J* = 11.2, 8.0 Hz, 4H), 7.50 (t, *J* = 7.3 Hz, 2H), 7.43 (dt, *J* = 7.0, 3.7 Hz, 4H), 7.17 (d, *J* = 5.3 Hz, 3H), 7.10 (s, 2H), 3.65 (d, *J* = 13.7 Hz, 2H). ^{13}C NMR (101 MHz, $CDCl_3$) δ 132.8, 131.7 (d, *J* = 2.8 Hz), 131.2 (d, *J* = 9.0 Hz), 131.1 (d, *J* = 8.0 Hz), 130.1 (d, *J* = 5.3 Hz), 128.5 (d, *J* = 11.7 Hz), 128.3 (d, *J* = 2.5 Hz), 126.7 (d, *J* = 3.0 Hz), 38.1 (d, *J* = 66.5 Hz). ^{31}P NMR (162 MHz, $CDCl_3$) δ 29.53. (*61*)

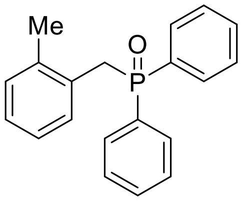

(2-methylbenzyl)diphenylphosphine oxide (188): The title compound was prepared according to the general procedure and purified by column chromatography on silica gel to afford a white solid (mp: 144-145 ºC) in 97% yield (59.4 mg); ^{1}H NMR (400 MHz, $CDCl_3$) δ 7.66 (dd, *J* = 11.1, 7.6 Hz, 4H), 7.48 (d, *J* = 6.5 Hz, 2H), 7.42 (d, *J* = 7.4 Hz, 4H), 7.06 (s, 2H), 6.95 (s, 2H), 3.66 (d, *J* = 14.0 Hz, 2H), 2.13 (s, 3H). ^{13}C NMR (101 MHz, $CDCl_3$) δ 137.3 (d, *J* = 5.4 Hz), 132.3 (d, *J* = 98.0 Hz), 131.7 (d, *J* = 2.7 Hz), 131.2 (d, *J* = 9.1 Hz), 130.6 (d, *J* = 4.6 Hz), 130.3 (d, *J* = 2.6 Hz), 129.5 (d, *J* = 8.1 Hz), 128.3 (d, *J* = 11.6 Hz), 126.6 (d, *J* = 3.1 Hz), 125.6 (d, *J* = 2.9 Hz), 35.1 (d, *J* = 66.6

Hz), 19.9. ^{31}P NMR (162 MHz, $CDCl_3$) δ 29.67. (*61*)

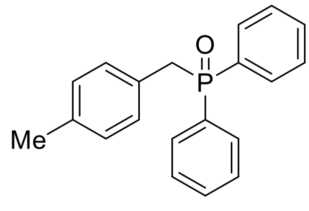

(4-methylbenzyl)diphenylphosphine oxide (189): The title compound was prepared according to the general procedure and purified by column chromatography on silica gel to afford a white solid (mp: 202-203 ºC) in 94% yield (57.5 mg); ^{1}H NMR (400 MHz, $CDCl_3$) δ 7.73 – 7.65 (m, 4H), 7.47 (dd, *J* = 10.6, 3.9 Hz, 2H), 7.44 – 7.36 (m, 4H), 7.01 – 6.92 (m, 4H), 3.60 (d, *J* = 13.6 Hz, 2H), 2.23 (s, 3H). ^{13}C NMR (101 MHz, $CDCl_3$) δ 136.3 (d, *J* = 3.1 Hz), 132.4 (d, *J* = 98.0 Hz), 131.6 (d, *J* = 2.7 Hz), 131.1 (d, *J* = 9.1 Hz), 129.9 (d, *J* = 5.3 Hz), 129.0 (d, *J* = 2.5 Hz), 128.4 (d, *J* = 11.6 Hz), 127.8 (d, *J* = 8.0 Hz), 37.6 (d, *J* = 67.0 Hz), 21.0. ^{31}P NMR (162 MHz, $CDCl_3$) δ 29.37. (*61*)

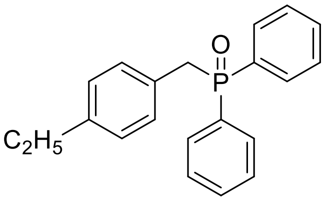

(4-ethylbenzyl)diphenylphosphine oxide (190): The title compound was prepared according to the general procedure and purified by column chromatography on silica gel to afford a white solid (mp: 182-183ºC) in 90% yield (57.6 mg); ^{1}H NMR (400 MHz, $CDCl_3$) δ 7.73 – 7.64 (m, 4H), 7.50 – 7.44 (m, 2H), 7.44 – 7.37 (m, 4H), 7.04 – 6.97 (m, 4H), 3.61 (d, *J* = 13.6 Hz, 2H), 2.54 (q, *J* = 7.6 Hz, 2H), 1.15 (t, *J* = 7.6 Hz, 3H). ^{13}C NMR (101 MHz, $CDCl_3$) δ 142.6 (d, *J* = 3.1 Hz), 132.3 (d, *J* = 98.0 Hz), 131.6 (d, *J* = 2.7 Hz), 131.0 (d, *J* = 9.1 Hz), 129.9 (d, *J* = 5.3 Hz), 128.3 (d, *J* = 11.7 Hz), 128.0 (d, *J* = 8.0 Hz), 127.8 (d, *J* = 2.5 Hz), 37.5 (d, *J* = 67.0 Hz), 28.3, 15.3. ^{31}P NMR (162 MHz, $CDCl_3$) δ 29.40. HRMS (EI) m/z: $[M]^+$ calcd for $C_{21}H_{21}OP$: 320.1330; found, 320.1328.

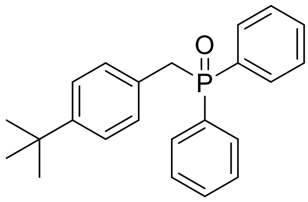

(4-(*tert*-butyl)benzyl)diphenylphosphine oxide (191): The title compound was prepared according to the general procedure and purified by column chromatography on silica gel to afford a white solid (mp: 245-246 ºC) in 92% yield (64.1 mg); ^{1}H NMR (400 MHz, $CDCl_3$) δ 7.69 (dd, *J* = 11.3, 7.2 Hz, 4H), 7.49 (t, *J* = 7.3 Hz, 2H), 7.45 – 7.38 (m, 4H), 7.20 (d, *J* = 8.1 Hz, 2H), 7.03 (dd, *J* = 8.2, 1.8 Hz, 2H), 3.62 (d, *J* = 13.5 Hz, 2H), 1.24 (s, 9H). ^{13}C NMR (101 MHz, $CDCl_3$) δ 149.5 (d, *J* = 3.2 Hz), 132.4 (d, *J* = 98.0 Hz), 131.6 (d, *J* = 2.7 Hz), 131.1 (d, *J* = 9.1 Hz), 129.7 (d, *J* = 5.2 Hz), 128.4 (d, *J* = 11.7 Hz), 127.8 (d, *J* = 8.0 Hz), 125.3 (d, *J* = 2.5 Hz),

37.4 (d, J = 67.0 Hz), 34.3, 31.2. ^{31}P NMR (162 MHz, $CDCl_3$) δ 29.66. HRMS (EI) m/z: $[M]^+$ calcd for $C_{23}H_{25}OP$: 348.1643; found, 348.1636.

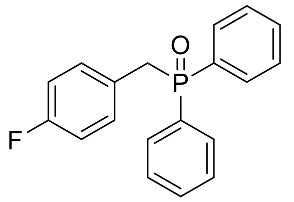

(4-fluorobenzyl)diphenylphosphine oxide (192): The title compound was prepared according to the general procedure and purified by column chromatography on silica gel to afford a white solid (mp: 206-207 ºC) in 90% yield (55.8 mg); ^{1}H NMR (400 MHz, $CDCl_3$) δ 7.72 – 7.60 (m, 4H), 7.49 (dd, J = 7.3, 6.3 Hz, 2H), 7.45 – 7.37 (m, 4H), 7.04 (dd, J = 7.1, 4.7 Hz, 2H), 6.84 (t, J = 8.6 Hz, 2H), 3.59 (d, J = 13.4 Hz, 2H). ^{13}C NMR (101 MHz, $CDCl_3$) δ 161.8 (dd, J = 245.4, 3.1 Hz), 132.4, 131.8 (d, J = 2.7 Hz), 131.5 (dd, J = 8.0, 5.2 Hz), 131.0 (d, J = 9.2 Hz), 128.5 (d, J = 11.7 Hz), 126.7 (dd, J = 7.9, 3.3 Hz), 115.2 (dd, J = 21.4, 2.5 Hz), 37.1 (d, J = 66.8 Hz). ^{31}P NMR (162 MHz, $CDCl_3$) δ 29.49. ^{19}F NMR (377 MHz, $CDCl_3$) δ -115.87. IR (neat, ν/cm^{-1}) 3079, 3059, 2940, 2890, 1597, 1505, 1436, 1218, 1180, 1119, 855, 827, 771, 740, 693. (*62*)

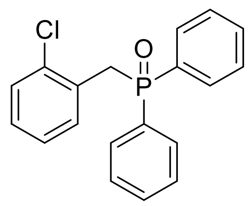

(2-chlorobenzyl)diphenylphosphine oxide (193): The title compound was prepared according to the general procedure and purified by column chromatography on silica gel to afford a white solid (mp: 153-154 ºC) in 72% yield (47.1 mg); ^{1}H NMR (400 MHz, $CDCl_3$) δ 7.71 – 7.59 (m, 4H), 7.51 – 7.42 (m, 3H), 7.37 (d, J = 5.7 Hz, 4H), 7.16 (d, J = 7.4 Hz, 1H), 7.08 (dd, J = 17.5, 8.2 Hz, 2H), 3.82 (d, J = 13.9 Hz, 2H). ^{13}C NMR (101 MHz, $CDCl_3$) δ 134.2 (d, J = 7.0 Hz), 131.9, 131.8 (d, J = 2.9 Hz), 131.8 (d, J = 99 Hz), 131.0 (d, J = 9.4 Hz), 129.5 (d, J = 7.5 Hz), 129.2 (d, J = 2.2 Hz), 128.4 (d, J = 11.8 Hz), 128.2 (d, J = 2.7 Hz), 126.7 (d, J = 2.5 Hz), 34.4 (d, J = 66.7 Hz). ^{31}P NMR (162 MHz, $CDCl_3$) δ 29.87. HRMS (EI) m/z: $[M]^+$ calcd for $C_{19}H_{16}ClOP$: 326.0627; found, 326.0620.

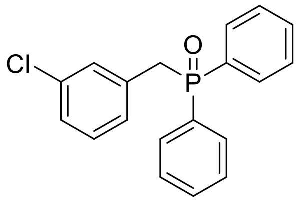

(3-chlorobenzyl)diphenylphosphine oxide (194): The title compound was prepared according to the general procedure and purified by column chromatography on silica gel to afford a white solid (mp: 106-107 ºC) in 95% yield (62.0 mg); ^{1}H NMR (400 MHz, $CDCl_3$) δ 7.67 (t, J = 8.0, 10.4 Hz, 4H), 7.48 (d, J = 6.8 Hz, 2H), 7.42 (d, J = 6.0 Hz, 4H), 7.15 – 6.96 (m, 4H), 3.58 (d, J = 13.6 Hz, 2H). ^{13}C NMR (101 MHz, $CDCl_3$) δ 133.9 (d, J = 2.8 Hz), 133.1 (d, J =

7.9 Hz), 131.9 (d, J = 2.7 Hz), 131.7 (d, J = 100.0 Hz), 131.0 (d, J = 9.2 Hz), 130.0 (d, J = 5.3 Hz), 129.4 (d, J = 2.5 Hz), 128.5 (d, J = 11.8 Hz), 128.2 (d, J = 5.1 Hz), 126.9 (d, J = 2.9 Hz), 37.7 (d, J = 65.8 Hz). ^{31}P NMR (162 MHz, $CDCl_3$) δ 29.32. HRMS (EI) m/z: $[M]^+$ calcd for $C_{19}H_{16}ClOP$: 326.0627; found, 326.0622.

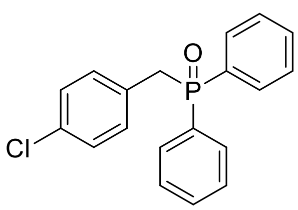

(4-chlorobenzyl)diphenylphosphine oxide (195): The title compound was prepared according to the general procedure and purified by column chromatography on silica gel to afford a white solid (mp: 241-242 ºC) in 97% yield (63.3 mg); ^{1}H NMR (400 MHz, $CDCl_3$) δ 7.68 (dd, J = 11.2, 7.5 Hz, 4H), 7.55 – 7.41 (m, 6H), 7.15 (d, J = 8.2 Hz, 2H), 7.03 (d, J = 6.8 Hz, 2H), 3.60 (d, J = 13.5 Hz, 2H). ^{13}C NMR (101 MHz, $CDCl_3$) δ 132.8 (d, J = 3.5 Hz), 132.0 (d, J = 99.0 Hz), 131.9 (d, J = 2.7 Hz), 131.3 (d, J = 5.3 Hz), 131.1 (d, J = 9.2 Hz), 129.7 (d, J = 8.1 Hz), 128.6, 128.5 (d, J = 1.7 Hz), 37.4 (d, J = 66.2 Hz). ^{31}P NMR (162 MHz, $CDCl_3$) δ 29.09. (*61*)

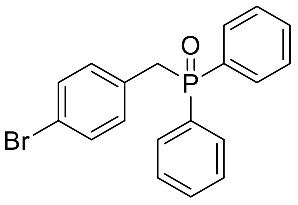

(4-bromobenzyl)diphenylphosphine oxide (196): The title compound was prepared according to the general procedure and purified by column chromatography on silica gel to afford a white solid (mp: 247-248 ºC) in 75% yield (55.5 mg); ^{1}H NMR (400 MHz, $CDCl_3$) δ 7.68 (dd, J = 11.3, 7.3 Hz, 4H), 7.51 (t, J = 6.9 Hz, 2H), 7.45 (dd, J = 9.7, 4.7 Hz, 4H), 7.30 (d, J = 8.2 Hz, 2H), 6.97 (d, J = 6.7 Hz, 2H), 3.58 (d, J = 13.6 Hz, 2H). ^{13}C NMR (101 MHz, $CDCl_3$) δ 132.4, 131.9 (d, J = 2.6 Hz), 131.7 (d, J = 5.2 Hz), 131.4 (d, J = 2.3 Hz), 131.1 (d, J = 9.2 Hz), 130.2 (d, J = 8.0 Hz), 128.6 (d, J = 11.8 Hz), 120.9 (d, J = 3.7 Hz), 37.5 (d, J = 66.2 Hz). ^{31}P NMR (162 MHz, $CDCl_3$) δ 29.04. (*63*)

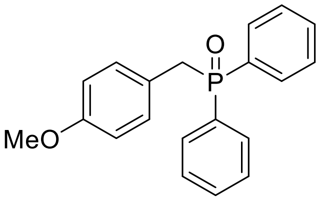

(4-methoxybenzyl)diphenylphosphine oxide (197): The title compound was prepared according to the general procedure and purified by column chromatography on silica gel to afford a white solid (mp: 227-228 ºC) in 82% yield (52.8 mg); ^{1}H NMR (400 MHz, $CDCl_3$) δ 7.68 (dd, J = 11.0, 7.6 Hz, 4H), 7.52 – 7.47 (m, 2H), 7.43 (dd, J = 9.8, 4.4 Hz, 4H), 7.00 (d, J = 6.9 Hz, 2H), 6.72 (d, J = 8.4 Hz, 2H), 3.72 (s, 3H), 3.58 (d, J = 13.3 Hz, 2H). ^{13}C NMR (101 MHz, $CDCl_3$) δ 158.5 (d, J = 2.7 Hz), 132.4 (d, J = 98.0 Hz), 131.7 (d, J = 2.7 Hz), 131.1 (d, J =

9.0 Hz), 131.1, 128.4 (d, *J* = 11.6 Hz), 122.9 (d, *J* = 8.1 Hz), 113.9 (d, *J* = 2.4 Hz), 55.2, 37.1 (d, *J* = 67.5 Hz). ^{31}P NMR (162 MHz, $CDCl_3$) δ 29.56. (*64*)

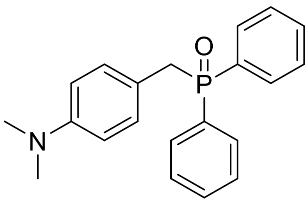

(4-(dimethylamino)benzyl)diphenylphosphine oxide (198): The title compound was prepared according to the general procedure and purified by column chromatography on silica gel to afford a yellow solid (mp: 212-213 ºC) in 86% yield (57.6 mg); ^{1}H NMR (400 MHz, $CDCl_3$) δ 7.72 – 7.65 (m, 4H), 7.53 – 7.46 (m, 3H), 7.42 (dd, *J* = 7.1, 4.9 Hz, 3H), 6.95 (d, *J* = 7.0 Hz, 2H), 6.56 (d, *J* = 8.5 Hz, 2H), 3.56 (d, *J* = 13.3 Hz, 2H), 2.86 (s, 6H). ^{13}C NMR (101 MHz, $CDCl_3$) δ 149.5 (d, *J* = 2.3 Hz), 132.6 (d, *J* = 97.0 Hz), 131.6 (d, *J* = 2.6 Hz), 131.2 (d, *J* = 9.0 Hz), 130.7 (d, *J* = 5.1 Hz), 128.4 (d, *J* = 11.5 Hz), 118.3 (d, *J* = 8.1 Hz), 112.7 (d, *J* = 2.1 Hz), 40.6 (s), 36.9 (d, *J* = 67.9 Hz). ^{31}P NMR (162 MHz, $CDCl_3$) δ 29.85. (*65*)

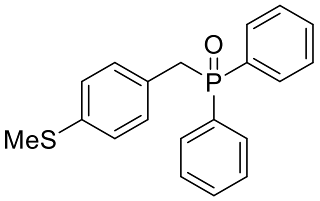

(4-(methylthio)benzyl)diphenylphosphine oxide (199): The title compound was prepared according to the general procedure and purified by column chromatography on silica gel to afford a white solid (mp: 245-246 ºC) in 88% yield (59.5 mg); ^{1}H NMR (400 MHz, $CDCl_3$) δ 7.67 (dd, *J* = 11.0, 7.6 Hz, 4H), 7.52 – 7.45 (m, 2H), 7.42 (dd, *J* = 9.9, 4.5 Hz, 4H), 7.06 (d, *J* = 8.2 Hz, 2H), 7.01 (d, *J* = 8.0 Hz, 2H), 3.59 (d, *J* = 13.6 Hz, 2H), 2.39 (s, 3H). ^{13}C NMR (101 MHz, $CDCl_3$) δ 136.8(d, *J* = 3.3 Hz), 132.1 (d, *J* = 98.0Hz), 131.8 (d, *J* = 2.7 Hz), 131.0 (d, *J* = 9.1 Hz), 130.4 (d, *J* = 5.3 Hz), 128.4 (d, *J* = 11.7 Hz), 127.8 (d, *J* = 8.2 Hz), 126.5 (d, *J* = 2.5 Hz), 37.4 (d, *J* = 66.6 Hz), 15.7. ^{31}P NMR (162 MHz, $CDCl_3$) δ 29.42. (*61*)

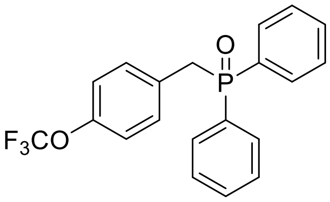

(4-(trifluoromethoxy)benzyl)diphenyl phosphine oxide (200): The title compound was prepared according to the general procedure and purified by column chromatography on silica gel to afford a white solid (mp: 203-204 ºC) in 83% yield (62.4 mg); ^{1}H NMR (400 MHz, $CDCl_3$) δ 7.67 (dd, *J* = 11.3, 7.4 Hz, 4H), 7.51 (t, *J* = 7.3 Hz, 2H), 7.43 (td, *J* = 7.8, 2.7 Hz, 4H), 7.11 (dd, *J* = 8.5, 1.8 Hz, 2H), 7.01 (d, *J* = 8.3 Hz, 2H), 3.63 (d, *J* = 13.4 Hz, 2H). ^{13}C NMR (101 MHz, $CDCl_3$) δ 148.1 (dd, *J* = 3.3, 1.7 Hz), 132.4, 131.9 (d, *J* = 2.7 Hz), 131.3 (d, *J* = 5.2 Hz), 131.0 (d, *J* = 9.2 Hz), 130.0 (d, *J* = 8.0 Hz), 128.5 (d, *J* = 11.8 Hz), 120.8 (d, *J* = 1.3 Hz),

120.4 (d, J = 255.0 Hz), 37.3 (d, J = 66.2 Hz). ^{31}P NMR (162 MHz, $CDCl_3$) δ 29.28 (t, J = 11.5 Hz). ^{19}F NMR (377 MHz, $CDCl_3$) δ -57.89. IR (neat, ν/cm^{-1}) 3081, 3059, 2952, 2900, 2842, 1592, 1505, 1435, 1265, 1220, 1180, 1152, 865, 790, 743, 720, 691, 665. HRMS (EI) m/z: $[M]^+$ calcd for $C_{20}H_{16}F_3O_2P$: 376.0840; found, 376.0835.

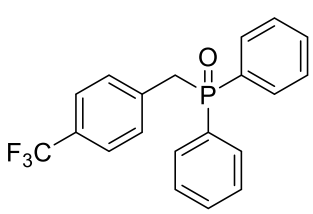

diphenyl(4-(trifluoromethyl)benzyl)phosphine oxide (201): The title compound was prepared according to the general procedure and purified by column chromatography on silica gel to afford a white solid (mp: 217-218 ºC) in 76% yield (54.7 mg); ^{1}H NMR (400 MHz, $CDCl_3$) δ 7.68 (dd, J = 11.4, 7.4 Hz, 4H), 7.50 (t, J = 7.3 Hz, 2H), 7.43 (dd, J = 12.9, 5.1 Hz, 6H), 7.21 (d, J = 7.5 Hz, 2H), 3.68 (d, J = 13.7 Hz, 2H). ^{13}C NMR (101 MHz, $CDCl_3$) δ 135.5 (d, J = 7.8 Hz), 132.0 (d, J = 2.7 Hz), 131.8 (d, J = 99 Hz), 131.0 (d, J = 9.3 Hz), 130.4 (d, J = 5.1 Hz), 129.0 (d, J = 29.1 Hz), 128.6 (d, J = 11.8 Hz), 125.2 (d, J = 3.2 Hz), 124.1 (d, J = 217.0 Hz), 38.0 (d, J = 65.4 Hz). ^{31}P NMR (162 MHz, $CDCl_3$) δ 29.06. ^{19}F NMR (376 MHz, $CDCl_3$) δ -62.54. IR (neat, ν/cm^{-1}) 3079, 3052, 2951, 2921, 2897, 1618, 1437, 1329, 1239, 1183, 1117, 1068, 1021, 842, 743, 720, 691, 630. (*62*)

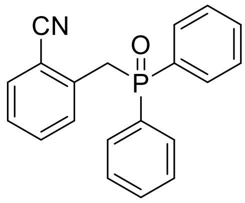

2-((diphenylphosphoryl)methyl)benzonitrile (202): The title compound was prepared according to the general procedure and purified by column chromatography on silica gel to afford a yellow solid (mp: 168-169 ºC) in 72% yield (45.6 mg); ^{1}H NMR (400 MHz, $CDCl_3$) δ 7.85 – 7.67 (m, 5H), 7.57 – 7.39 (m, 8H), 7.28 (d, J = 5.2 Hz, 1H), 3.92 (d, J = 13.7 Hz, 2H). ^{13}C NMR (101 MHz, $CDCl_3$) δ 135.5 (d, J = 7.6 Hz), 132.6 (d, J = 2.6 Hz), 132.4 (d, J = 2.0 Hz), 132.1 (d, J = 2.7 Hz), 131.2 (d, J = 4.3 Hz), 131.1 (d, J = 100.0 Hz), 131.0 (d, J = 9.4 Hz), 128.6 (d, J = 12.0 Hz), 127.3 (d, J = 2.6 Hz), 117.7 (d, J = 1.5 Hz), 113.1 (d, J = 6.3 Hz), 36.3 (d, J = 64.5 Hz). ^{31}P NMR (162 MHz, $CDCl_3$) δ 29.20.

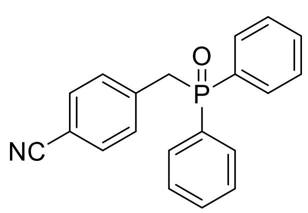

4-((diphenylphosphoryl)methyl)benzonitrile (203): The title compound was prepared according to the general procedure and purified by column chromatography on silica gel to afford a white solid (mp: 225-226 ºC) in 94% yield (59.6 mg); ^{1}H NMR (400 MHz,

$CDCl_3$) δ 7.71 – 7.64 (m, 4H), 7.57 – 7.50 (m, 2H), 7.50 – 7.41 (m, 6H), 7.21 (dd, *J* = 8.3, 1.9 Hz, 2H), 3.68 (d, *J* = 13.7 Hz, 2H). ^{13}C NMR (101 MHz, $CDCl_3$) δ 137.1 (d, *J* = 8.0 Hz), 132.2 (d, *J* = 2.8 Hz), 132.0 (d, *J* = 2.5 Hz), 131.5 (d, *J* = 100.0 Hz), 131.0 (d, *J* = 9.3 Hz), 130.8 (d, *J* = 5.1 Hz), 128.7 (d, *J* = 11.9 Hz), 118.7 (d, *J* = 1.9 Hz), 110.7 (d, *J* = 3.2 Hz), 38.4 (d, *J* = 64.3 Hz). ^{31}P NMR (162 MHz, $CDCl_3$) δ 28.87. (*62*)

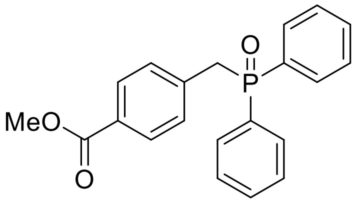

methyl 4-((diphenylphosphoryl)methyl)benzoate (204): The title compound was prepared according to the general procedure and purified by column chromatography on silica gel to afford a white solid (mp: 251-252 ºC) in 55% yield (38.6 mg); ^{1}H NMR (400 MHz, $CDCl_3$) δ 7.83 (d, *J* = 7.8 Hz, 2H), 7.71 – 7.61 (m, 4H), 7.55 – 7.35 (m, 6H), 7.16 (d, *J* = 7.6 Hz, 2H), 3.84 (s, 3H), 3.68 (d, *J* = 13.9 Hz, 2H). ^{13}C NMR (101 MHz, $CDCl_3$) δ 166.82, 136.66 (d, *J* = 8.0 Hz), 131.94 (d, *J* = 2.7 Hz), 131.78 (d, *J* = 100.0 Hz), 131.01 (d, *J* = 9.2 Hz), 130.07 (d, *J* = 5.2 Hz), 129.50 (d, *J* = 2.5 Hz), 128.60, 128.48, 51.96, 38.29 (d, *J* = 65.1 Hz). ^{31}P NMR (162 MHz, $CDCl_3$) δ 29.20. (*48*)

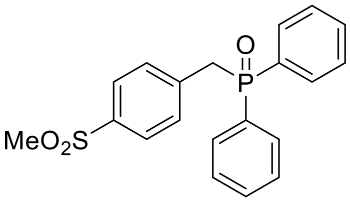

(4-(methylsulfonyl)benzyl)diphenylphosphine oxide (205): The title compound was prepared according to the general procedure and purified by column chromatography on silica gel to afford a white solid (mp: 233-234 ºC) in 84% yield (62.2 mg); ^{1}H NMR (400 MHz, $CDCl_3$) δ 7.73 – 7.65 (m, 6H), 7.52 (t, *J* = 7.3 Hz, 2H), 7.44 (td, *J* = 7.3, 2.6 Hz, 4H), 7.29 (d, *J* = 6.7 Hz, 2H), 3.73 (d, *J* = 13.7 Hz, 2H), 2.97 (s, 3H). ^{13}C NMR (101 MHz, $CDCl_3$) δ 138.9 (d, *J* = 3.2 Hz), 138.0 (d, *J* = 8.0 Hz), 132.2 (d, *J* = 2.7 Hz), 131.6 (d, *J* = 71.0 Hz), 130.9 (d, *J* = 9.0 Hz), 130.9, 128.7 (d, *J* = 11.9 Hz), 127.3 (d, *J* = 2.4 Hz), 44.4, 38.0 (d, *J* = 64.3 Hz). ^{31}P NMR (162 MHz, $CDCl_3$) δ 29.15. HRMS (EI) m/z: $[M]^+$ calcd for $C_{20}H_{19}O_3PS$: 370.0793; found, 370.0790.

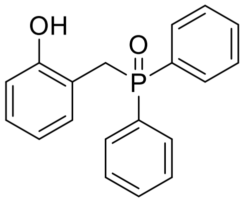

(2-hydroxybenzyl)diphenylphosphine oxide (206): The title compound was prepared according to the general procedure and purified by column chromatography on silica gel to afford a white solid (mp: 155-156 ºC) in 94% yield (57.9 mg); ^{1}H NMR (400 MHz, $CDCl_3$) δ 9.83 (s, 1H), 7.72 (dd,

J = 11.2, 7.9 Hz, 4H), 7.60 – 7.38 (m, 6H), 7.08 (t, J = 7.5 Hz, 1H), 6.98 (d, J = 8.0 Hz, 1H), 6.79 (d, J = 7.2 Hz, 1H), 6.68 (t, J = 7.2 Hz, 1H), 3.71 (d, J = 12.9 Hz, 2H). ^{13}C NMR (101 MHz, $CDCl_3$) δ 156.4 (d, J = 4.1 Hz), 132.3 (d, J = 2.8 Hz), 131.6 (d, J = 6.3 Hz), 130.9 (d, J = 9.5 Hz), 130.5 (d, J = 99.2 Hz), 128.8 (d, J = 2.7 Hz), 128.7 (d, J = 12.0 Hz), 120.4 (d, J = 1.8 Hz), 119.2 (d, J = 2.1 Hz), 119.1, 35.0 (d, J = 67.4 Hz). ^{31}P NMR (162 MHz, $CDCl_3$) δ 38.10. (*47*)

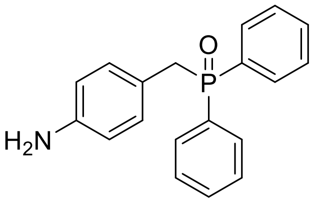

(4-aminobenzyl)diphenylphosphine oxide (207): The title compound was prepared according to the general procedure and purified by column chromatography on silica gel to afford a yellow solid (mp: 110-111 ºC) in 72% yield (44.2 mg); ^{1}H NMR (400 MHz, $CDCl_3$) δ 7.66 (dd, J = 10.6, 7.9 Hz, 4H), 7.44 (dt, J = 12.9, 7.2 Hz, 6H), 6.85 (d, J = 7.3 Hz, 2H), 6.48 (d, J = 8.0 Hz, 3H), 3.53 (d, J = 13.2 Hz, 2H), 3.18 (s, 2H). ^{13}C NMR (101 MHz, $CDCl_3$) δ 145.17 (d, J = 2.4 Hz), 132.31 (d, J = 98.6 Hz), 131.59 (d, J = 2.4 Hz), 131.09 (d, J = 9.0 Hz), 130.85 (d, J = 5.1 Hz), 128.34 (d, J = 11.5 Hz), 120.19 (d, J = 8.0 Hz), 115.10 (d, J = 2.0 Hz), 37.01 (d, J = 68.0 Hz). ^{31}P NMR (162 MHz, $CDCl_3$) δ 29.95. HRMS (EI) m/z: $[M]^+$ calcd for $C_{19}H_{18}NOP$: 307.1126; found, 307.1126.

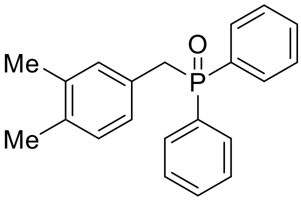

(3,4-dimethylbenzyl)diphenylphosphine oxide (208): The title compound was prepared according to the general procedure and purified by column chromatography on silica gel to afford a white solid (mp: 152-153 ºC) in 85% yield (54.4 mg); ^{1}H NMR (400 MHz, $CDCl_3$) δ 7.74 – 7.66 (m, 4H), 7.48 (dd, J = 7.1, 1.1 Hz, 2H), 7.44 – 7.38 (m, 4H), 6.92 (d, J = 7.7 Hz, 1H), 6.88 (s, 1H), 6.78 (d, J = 7.6 Hz, 1H), 3.58 (d, J = 13.6 Hz, 2H), 2.15 (s, 3H), 2.11 (s, 3H). ^{13}C NMR (101 MHz, $CDCl_3$) δ 136.4 (d, J = 2.6 Hz), 134.9 (d, J = 3.2 Hz), 132.5 (d, J = 98.0 Hz), 131.6 (d, J = 2.7 Hz), 131.4 (d, J = 5.2 Hz), 131.1 (d, J = 9.1 Hz), 129.5 (d, J = 2.6 Hz), 128.3 (d, J = 11.6 Hz), 128.1 (d, J = 8.0 Hz), 127.3 (d, J = 5.4 Hz), 37.5 (d, J = 67.1 Hz), 19.5, 19.2. ^{31}P NMR (162 MHz, $CDCl_3$) δ 29.42. HRMS (EI) m/z: $[M]^+$ calcd for $C_{21}H_{21}OP$: 320.1330; found, 320.1317.

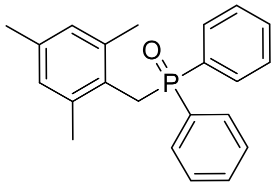

diphenyl(2,4,6-trimethylbenzyl)phosphine oxide (209): The title compound was prepared according to the general procedure and purified by column chromatography on silica gel to afford a white solid (mp: 184-185 ºC) in 90% yield (60.1 mg); ^{1}H NMR (400 MHz, $CDCl_3$) δ 7.69 – 7.62 (m, 4H), 7.52 (dd, *J* = 7.8, 6.9 Hz, 2H), 7.42 (td, *J* = 7.6, 2.8 Hz, 4H), 6.77 (s, 2H), 3.71 (d, *J* = 13.9 Hz, 2H), 2.23 (d, *J* = 1.5 Hz, 3H), 1.99 (s, 6H). ^{13}C NMR (101 MHz, $CDCl_3$) δ 137.7 (d, *J* = 4.7 Hz), 136.1 (d, *J* = 3.5 Hz), 133.1 (d, *J* = 96.0 Hz), 131.7 (d, *J* = 2.7 Hz), 131.2 (d, *J* = 9.0 Hz), 129.0 (d, *J* = 3.0 Hz), 128.4 (d, *J* = 11.5 Hz), 125.4 (d, *J* = 8.8 Hz), 32.8 (d, *J* = 67.0 Hz), 20.8 (d, *J* = 0.9 Hz), 20.6 (d, *J* = 1.3 Hz). ^{31}P NMR (162 MHz, $CDCl_3$) δ 28.69. (*63*)

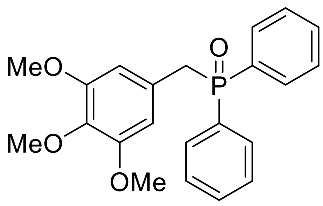

diphenyl(3,4,5-trimethoxybenzyl)phosphine oxide (210): The title compound was prepared according to the general procedure and purified by column chromatography on silica gel to afford a white solid (mp: 177-178 ºC) in 98% yield (74.9 mg); ^{1}H NMR (400 MHz, $CDCl_3$) δ 7.72 – 7.57 (m, 4H), 7.52 – 7.30 (m, 6H), 6.19 (s, 2H), 3.73 (s, 3H), 3.58 (s, 6H), 3.54 (d, *J* = 14.3 Hz, 2H). ^{13}C NMR (101 MHz, $CDCl_3$) δ 152.7 (d, *J* = 2.7 Hz), 136.7 (d, *J* = 3.4 Hz), 132.0 (d, *J* = 98.0 Hz), 131.7 (d, *J* = 2.6 Hz), 131.1 (d, *J* = 9.1 Hz), 128.3 (d, *J* = 11.7 Hz), 126.4 (d, *J* = 8.1 Hz), 107.1 (d, *J* = 5.2 Hz), 60.6, 55.7, 38.2 (d, *J* = 66.6 Hz). ^{31}P NMR (162 MHz, $CDCl_3$) δ 29.52. (*61*)

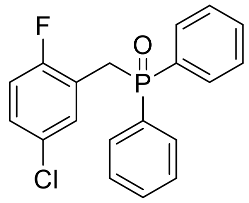

(5-chloro-2-fluorobenzyl)diphenylphosphine oxide (211): The title compound was prepared according to the general procedure and purified by column chromatography on silica gel to afford a white solid (mp: 128-129 ºC) in 78% yield (53.7 mg); ^{1}H NMR (400 MHz, $CDCl_3$) δ 7.71 (dd, *J* = 11.6, 7.2 Hz, 4H), 7.54 – 7.40 (m, 6H), 7.38 – 7.30 (m, 1H), 7.13 – 7.02 (m, 1H), 6.81 (t, *J* = 9.0 Hz, 1H), 3.63 (d, *J* = 13.6 Hz, 2H). ^{13}C NMR (101 MHz, $CDCl_3$) δ 159.1 (dd, *J* = 245.8, 6.1 Hz), 132.0 (d, *J* = 2.8 Hz), 131.7 (t, *J* = 4.0 Hz), 131.7 (d, *J* = 100.0 Hz), 130.9 (d, *J* = 9.4 Hz), 129.0 (t, *J* = 3.3 Hz), 128.5 (d, *J* = 11.9 Hz), 128.5, 120.6 (dd, *J* = 17.1, 7.8 Hz), 116.3 (dd, *J* = 24.4, 2.4 Hz), 30.3 (d, *J* = 66.5 Hz). ^{31}P NMR (162 MHz, $CDCl_3$) δ 28.93. ^{19}F NMR (377 MHz, $CDCl_3$) δ -119.51. IR (neat, ν/cm^{-1}) 3081, 3052, 2959, 2918, 2850, 1481, 1436, 1241, 1185, 1119, 1097, 879, 743, 823,

773, 742, 709, 693, 644. HRMS (EI) m/z: $[M]^+$ calcd for $C_{19}H_{15}ClFOP$: 344.0533; found, 344.0529.

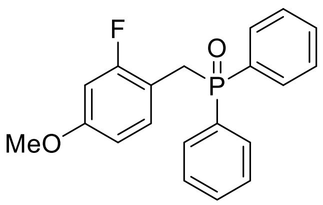

(2-fluoro-4-methoxybenzyl)diphenylphosphine oxide (212): The title compound was prepared according to the general procedure and purified by column chromatography on silica gel to afford a white solid (mp: 175-176 ºC) in 80% yield (54.4 mg); ^{1}H NMR (400 MHz, $CDCl_3$) δ 7.70 (dd, *J* = 11.0, 7.8 Hz, 4H), 7.47 (t, *J* = 6.4 Hz, 2H), 7.41 (dd, *J* = 9.9, 4.0 Hz, 4H), 7.29 (t, *J* = 8.6 Hz, 1H), 6.57 (d, *J* = 8.5 Hz, 1H), 6.44 (d, *J* = 11.7 Hz, 1H), 3.69 (s, 3H), 3.60 (d, *J* = 13.2 Hz, 2H). ^{13}C NMR (101 MHz, $CDCl_3$) δ 162.2 (d, *J* = 6.2 Hz), 160.0 – 159.6 (m), 132.1 (d, *J* = 98.0 Hz), 132.2 (t, *J* = 5.0 Hz), 131.7 (d, *J* = 2.7 Hz), 130.9 (d, *J* = 9.3 Hz), 128.4 (d, *J* = 11.7 Hz), 110.0 (dd, *J* = 15.9, 7.8 Hz), 109.9 (t, *J* = 2.8 Hz), 101.3 (dd, *J* = 26.1, 2.2 Hz), 55.4, 29.4 (d, *J* = 68.5 Hz). ^{31}P NMR (162 MHz, $CDCl_3$) δ 29.53 (t, *J* = 11.34 Hz). ^{19}F NMR (377 MHz, $CDCl_3$) δ -114.91. IR (neat, ν/cm^{-1}) 3076, 3059, 3008, 2963, 2945, 2897, 1620, 1587, 1505, 1436, 1277, 1185, 1145, 1096, 1034, 934, 851, 808, 788, 748, 726, 691, 625. HRMS (EI) m/z: $[M]^+$ calcd for $C_{20}H_{18}FO_2P$: 340.1028; found, 340.1021.

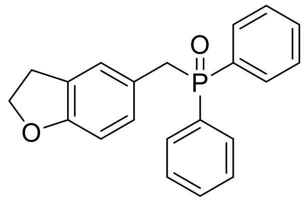

((2,3-dihydrobenzofuran-5-yl)methyl)diphenylphosphine oxide (213): The title compound was prepared according to the general procedure and purified by column chromatography on silica gel to afford a white solid (mp: 190-191 ºC) in 87% yield (58.1 mg); ^{1}H NMR (400 MHz, $CDCl_3$) δ 7.68 (dd, *J* = 10.7, 7.7 Hz, 4H), 7.48 (t, *J* = 7.3, 7.2 Hz 2H), 7.42 (td, *J* = 7.3, 2.5 Hz, 4H), 7.03 (s, 1H), 6.70 (d, *J* = 8.1 Hz, 1H), 6.55 (d, *J* = 8.1 Hz, 1H), 4.47 (t, *J* = 8.7 Hz, 2H), 3.56 (d, *J* = 13.2 Hz, 2H), 3.08 (t, *J* = 8.6 Hz, 2H). ^{13}C NMR (101 MHz, $CDCl_3$) δ 159.0 (d, *J* = 2.8 Hz), 132.4 (d, *J* = 97.0 Hz), 131.7 (d, *J* = 2.7 Hz), 131.1 (d, *J* = 9.1 Hz), 129.5 (d, *J* = 5.8 Hz), 128.4 (d, *J* = 11.6 Hz), 127.2 (d, *J* = 2.5 Hz), 126.7 (d, *J* = 4.9 Hz), 122.5 (d, *J* = 8.2 Hz), 108.9 (d, *J* = 2.4 Hz), 71.2, 37.3 (d, *J* = 67.6 Hz), 29.6. ^{31}P NMR (162 MHz, $CDCl_3$) δ 29.58. HRMS (EI) m/z: $[M]^+$ calcd for $C_{21}H_{19}O_2P$: 334.1123; found, 334.1123.

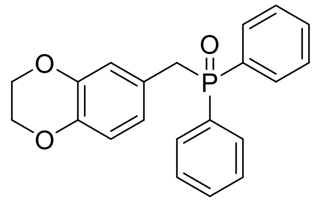

((2,3-dihydrobenzo[*b*][1,4]dioxin-6-yl)methyl)diphenylphosphine oxide (214): The title compound was prepared according to the general procedure and purified by column chromatography on silica gel to afford a white solid (mp: 177-178 ºC) in 96% yield (67.2 mg); ^{1}H NMR (400 MHz, $CDCl_3$) δ 7.69 (dd, *J* = 11.1, 7.6 Hz, 4H), 7.50 (t, *J* = 7.2 Hz, 2H), 7.46 – 7.38 (m, 4H), 6.64 (t, *J* = 8.0 Hz, 2H), 6.54 (d, *J* = 8.2 Hz, 1H), 4.16 (s, 4H), 3.54 (d, *J* = 13.4 Hz, 2H). ^{13}C NMR (101 MHz, $CDCl_3$) δ 143.2 (d, *J* = 2.5 Hz), 142.5 (d, *J* = 2.9 Hz), 132.3 (d, *J* = 100.0 Hz), 131.7 (d, *J* = 2.7 Hz), 131.1 (d, *J* = 9.1 Hz), 128.4 (d, *J* = 11.7 Hz), 123.9 (d, *J* = 8.1 Hz), 123.1 (d, *J* = 5.2 Hz), 118.9 (d, *J* = 5.4 Hz), 117.0 (d, *J* = 2.5 Hz), 64.2, 64.12, 37.1 (d, *J* = 67.5 Hz). ^{31}P NMR (162 MHz, $CDCl_3$) δ 29.51. HRMS (EI) m/z: $[M]^+$ calcd for $C_{22}H_{19}O_3P$: 350.1072; found, 350.1062.

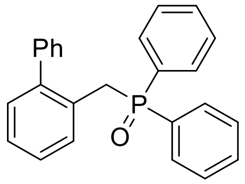

([1,1'-biphenyl]-2-ylmethyl)diphenylphosphine oxide (215): The title compound was prepared according to the general procedure and purified by column chromatography on silica gel to afford a yellow oil in 91% yield (67.1 mg); ^{1}H NMR (400 MHz, $CDCl_3$) δ 7.78 – 7.66 (m, 1H), 7.59 (d, *J* = 5.9 Hz, 1H), 7.50 – 7.28 (m, 14H), 7.11 (d, *J* = 6.3 Hz, 1H), 6.97 (s, 2H), 3.72 (d, *J* = 13.9 Hz, 2H). ^{13}C NMR (101 MHz, $CDCl_3$) δ 142.6 (d, *J* = 6.4 Hz), 140.9, 131.3 (d, *J* = 99.0 Hz), 131.6 (d, *J* = 2.6 Hz), 131.1 (d, *J* = 9.2 Hz), 130.8 (d, *J* = 4.3 Hz), 130.5 (d, *J* = 9.8 Hz), 130.2 (d, *J* = 2.1 Hz), 129.4, 128.4 (d, *J* = 11.7 Hz), 128.1, 127.4 (d, *J* = 2.5 Hz), 126.9, 126.8 (d, *J* = 2.6 Hz), 34.1 (d, *J* = 66.7 Hz). ^{31}P NMR (162 MHz, $CDCl_3$) δ 30.12. (*48*)

diphenyl((2'-(trifluoromethyl)-[1,1'-biphenyl]-4-yl)methyl)phosphine oxide (216): The title

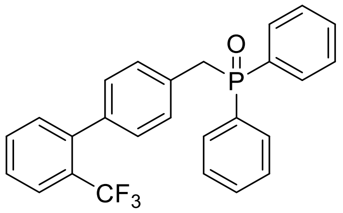

compound was prepared according to the general procedure and purified by column chromatography on silica gel to afford a yellow solid (mp: 182-183 ºC) in 78% yield (68.0 mg); ^{1}H NMR (400 MHz, $CDCl_3$) δ 7.78 – 7.60 (m, 5H), 7.51 (t, *J* = 7.2 Hz, 3H), 7.44 (t, *J* = 6.6 Hz, 5H), 7.27 (s, 1H), 7.12 (q, *J* = 8.0 Hz, 4H), 3.69 (d, *J* = 13.8 Hz, 2H). ^{13}C NMR (101 MHz, $CDCl_3$) δ 140.9, 138.3 (d, *J* = 3.2 Hz), 132.0 (d, *J* = 99.0 Hz), 131.9, 131.8 (d, *J* = 2.7 Hz), 131.2 (d, *J* = 9.1 Hz), 130.6 (d, *J* = 8.0 Hz), 129.5 (d, *J* = 5.2 Hz), 128.9, 128.4 (d, *J* = 11.7 Hz), 128.2, 127.2, 125.9 (q, *J* = 5.4 Hz), 125.4, 122.7, 38.0 (d, *J* = 66.1 Hz). ^{31}P NMR (162 MHz,

$CDCl_3$) δ 29.90. ^{19}F NMR (376 MHz, $CDCl_3$) δ -56.73. HRMS (EI) m/z: $[M]^+$ calcd for $C_{26}H_{20}F_3OP$: 436.1204; found, 436.1205.

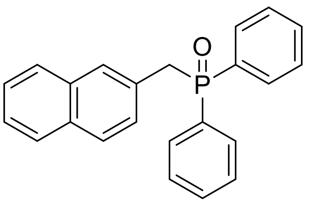

(naphthalen-2-ylmethyl)diphenylphosphine oxide (217): The title compound was prepared according to the general procedure and purified by column chromatography on silica gel to afford a white solid (mp: 210-211 ºC) in 99% yield (67.7 mg); ^{1}H NMR (400 MHz, $CDCl_3$) δ 7.76 – 7.63 (m, 7H), 7.56 (s, 1H), 7.49 (td, *J* = 7.4, 1.1 Hz, 2H), 7.44 – 7.37 (m, 6H), 7.23 (d, *J* = 8.4 Hz, 1H), 3.80 (d, *J* = 13.8 Hz, 2H). ^{13}C NMR (101 MHz, $CDCl_3$) δ 133.3 (d, *J* = 2.6 Hz), 132.7, 132.2 (d, *J* = 2.1 Hz), 131.8 (d, *J* = 2.5 Hz), 131.1 (d, *J* = 9.1 Hz), 129.0 (d, *J* = 6.7 Hz), 128.7 (d, *J* = 8.2 Hz), 128.5 (d, *J* = 11.7 Hz), 128.1 (d, *J* = 4.3 Hz), 127.9 (d, *J* = 1.9 Hz), 127.6 (d, *J* = 0.9 Hz), 127.5 (d, *J* = 1.1 Hz), 125.9, 125.6 (d, *J* = 0.9 Hz), 38.3 (d, *J* = 66.3 Hz). ^{31}P NMR (162 MHz, $CDCl_3$) δ 29.52. (*61*)

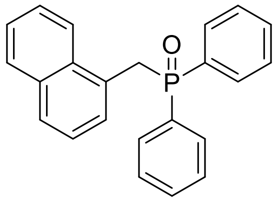

(naphthalen-1-ylmethyl)diphenylphosphine oxide (218): The title compound was prepared according to the general procedure and purified by column chromatography on silica gel to afford a white solid (mp: 162-163 ºC) in 99% yield (67.8 mg); ^{1}H NMR (400 MHz, $CDCl_3$) δ 7.90 (d, *J* = 8.0 Hz, 1H), 7.75 (d, *J* = 8.3 Hz, 1H), 7.66 (dd, *J* = 11.3, 7.3 Hz, 5H), 7.44 (t, *J* = 7.3 Hz, 2H), 7.35 (t, *J* = 7.4 Hz, 6H), 7.22 (t, *J* = 8.5 Hz, 2H), 4.09 (d, *J* = 13.9 Hz, 2H). ^{13}C NMR (101 MHz, $CDCl_3$) δ 133.7 (d, *J* = 1.9 Hz), 132.4 (d, *J* = 4.3 Hz), 132.3 (d, *J* = 98.0 Hz), 131.7 (d, *J* = 2.7 Hz), 131.1 (d, *J* = 9.1 Hz), 128.6 (d, *J* = 6.2 Hz), 128.4, 128.4 (d, *J* = 12.0 Hz), 127.7 (d, *J* = 8.4 Hz), 127.6 (d, *J* = 3.3 Hz), 125.9, 125.5, 125.0 (d, *J* = 3.2 Hz), 124.2, 34.8 (d, *J* = 66.8 Hz). ^{31}P NMR (162 MHz, $CDCl_3$) δ 29.63. (*62*)

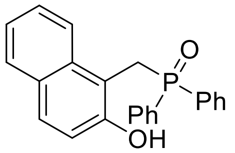

((2-hydroxynaphthalen-1-yl)methyl)diphenylphosphine oxide (219): The title compound was prepared according to the general procedure and purified by column chromatography on silica gel to afford a white solid (mp: 220-221 ºC) in 88% yield (63.1 mg); ^{1}H NMR (400 MHz, $CDCl_3$) δ 10.22 (s, 1H), 7.79 – 7.62 (m, 6H), 7.47 (d, *J* = 7.0 Hz, 2H), 7.36 (d, *J* = 22.8 Hz, 5H), 7.29 (d, *J* = 8.9 Hz, 1H), 7.20 (s, 2H), 4.14 (d, *J* = 13.2 Hz, 2H). ^{13}C NMR (101 MHz, $CDCl_3$) δ 155.1 (d, *J* = 4.9 Hz), 132.9 (d, *J* = 4.6 Hz),

132.5 (d, J = 2.7 Hz), 131.1 (d, J = 9.7 Hz), 129.4 (d, J = 98.0 Hz), 129.3, 128.7 (d, J = 11.8 Hz), 126.0, 123.2, 122.7, 121.6, 121.1 (d, J = 2.7 Hz), 118.3, 111.0 (d, J = 8.9 Hz), 29.1 (d, J = 68.6 Hz). ^{31}P NMR (162 MHz, $CDCl_3$) δ 39.11. IR (neat, ν/cm^{-1}) 3057, 3004, 2947, 2902, 2846, 1629, 1585, 1514, 1434, 1359, 1281, 1149, 961, 860, 813, 742, 717, 686, 608. HRMS (EI) m/z: $[M]^+$ calcd for $C_{23}H_{19}O_2P$: 358.1123; found, 358.1127.

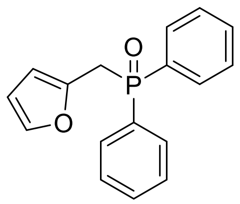

(furan-2-ylmethyl)diphenylphosphine oxide (220): The title compound was prepared according to the general procedure and purified by column chromatography on silica gel to afford a brown solid (mp: 122-123 ºC) in 80% yield (45.1 mg); ^{1}H NMR (400 MHz, $CDCl_3$) δ 7.68 (dd, J = 11.6, 7.3 Hz, 4H), 7.49 (t, J = 6.8 Hz, 2H), 7.44 – 7.39 (m, 4H), 7.20 (s, 1H), 6.19 (s, 1H), 6.05 (t, J = 2.8 Hz, 1H), 3.73 (d, J = 13.9 Hz, 2H). ^{13}C NMR (101 MHz, $CDCl_3$) δ 145.1 (d, J = 7.9 Hz), 141.8 (d, J = 2.9 Hz), 131.9 (d, J = 2.7 Hz), 131.8 (d, J = 100.0 Hz), 131.0 (d, J = 9.4 Hz), 128.4 (d, J = 11.9 Hz), 110.7 (d, J = 2.6 Hz), 109.0 (d, J = 5.9 Hz), 31.2 (d, J = 69.4 Hz). ^{31}P NMR (162 MHz, $CDCl_3$) δ 28.36. (*63*)

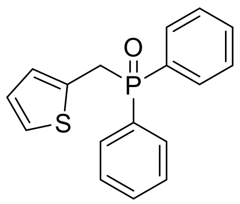

diphenyl(thiophen-2-ylmethyl)phosphine oxide (221): The title compound was prepared according to the general procedure and purified by column chromatography on silica gel to afford a yellow solid (mp: 143-144 ºC) in 85% yield (50.8 mg); ^{1}H NMR (400 MHz, $CDCl_3$) δ 7.71 (dd, J = 11.1, 7.8 Hz, 4H), 7.50 (t, J = 7.2, 2H), 7.44 (dd, J = 9.8, 4.3 Hz, 4H), 7.06 (d, J = 4.4 Hz, 1H), 6.83 (d, J = 4.6 Hz, 2H), 3.85 (d, J = 13.1 Hz, 2H). ^{13}C NMR (101 MHz, $CDCl_3$) δ131.9 (d, J = 2.7 Hz), 131.8 (d, J = 8.7 Hz), 131.7 (d, J = 99.0 Hz), 131.1 (d, J = 9.2 Hz), 128.5 (d, J = 11.8 Hz), 127.7 (d, J = 6.6 Hz), 126.9 (d, J = 2.8 Hz), 124.8 (d, J = 3.1 Hz), 32.5 (d, J = 68.9 Hz). ^{31}P NMR (162 MHz, $CDCl_3$) δ 28.43. ^{31}P NMR (162 MHz, $CDCl_3$) δ 28.61. IR (neat, ν/cm^{-1}) 3097, 3067, 3041, 2937, 2914, 2808, 2773, 1488, 1436, 1366, 1336, 1297, 1244, 1150, 1126, 1037, 1009, 924, 834, 808, 721, 642. (*64*)

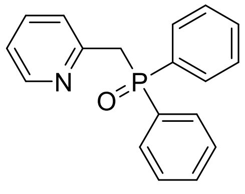

diphenyl(pyridin-2-ylmethyl)phosphine oxide (222): The title compound was prepared according to the general procedure and purified by column chromatography on silica gel to afford a brown solid (mp: 127-128 ºC) in 90% yield (52.8 mg); ^{1}H NMR (400 MHz, $CDCl_3$) δ 8.28 (d, *J* = 4.1 Hz, 1H), 7.67 (dd, *J* = 11.2, 7.7 Hz, 4H), 7.47 – 7.27 (m, 8H), 6.95 (t, *J* = 5.6 Hz, 1H), 3.85 (d, *J* = 14.2 Hz, 2H). ^{13}C NMR (101 MHz, $CDCl_3$) δ 152.2 (d, *J* = 7.1 Hz), 148.9 (d, *J* = 1.6 Hz), 136.1 (d, *J* = 2.2 Hz), 132.5, 131.5 (d, *J* = 2.8 Hz), 130.8 (d, *J* = 9.5 Hz), 128.2 (d, *J* = 11.9 Hz), 124.7 (d, J = 3.5 Hz), 121.5 (d, *J* = 2.5 Hz), 40.6 (d, *J* = 64.5 Hz). ^{31}P NMR (162 MHz, $CDCl_3$) δ 29.79. (*66*)

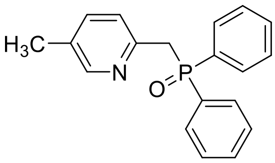

((5-methylpyridin-2-yl)methyl)diphenylphosphine oxide (223): The title compound was prepared according to the general procedure and purified by column chromatography on silica gel to afford a yellow solid (mp: 251-252 ºC) in 82% yield (50.4 mg); ^{1}H NMR (400 MHz, $CDCl_3$) δ 8.18 (s, 1H), 7.81 (dd, *J* = 22.1, 9.2 Hz, 6H), 7.37 (d, *J* = 8.3 Hz, 6H), 4.30 (d, *J* = 13.8 Hz, 2H), 2.27 (s, 3H). ^{13}C NMR (101 MHz, $CDCl_3$) δ 146.8 (d, *J* = 6.2 Hz), 143.3, 141.9, 134.5 (d, *J* = 1.5 Hz), 132.1 (d, *J* = 2.7 Hz), 130.8 (d, *J* = 9.8 Hz), 130.5 (d, *J* = 101.0 Hz), 128.6 (d, *J* = 12.3 Hz), 127.0 (d, *J* = 3.1 Hz), 35.8 (d, *J* = 61.6 Hz), 17.9. ^{31}P NMR (162 MHz, $CDCl_3$) δ 28.69. (*66*)

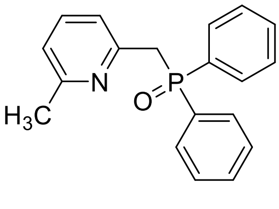

((6-methylpyridin-3-yl)methyl)diphenylphosphine oxide (224): The title compound was prepared according to the general procedure and purified by column chromatography on silica gel to afford a brown oil in 88% yield (54.1 mg). ^{1}H NMR (400 MHz, $CDCl_3$) δ 7.73 (dd, *J* = 11.2, 7.8 Hz, 4H), 7.49 – 7.33 (m, 7H), 7.23 (t, *J* = 9.1 Hz, 1H), 6.89 (d, *J* = 7.5 Hz, 1H), 3.87 (d, *J* = 14.2 Hz, 2H), 2.34 (s, 3H). ^{13}C NMR (101 MHz, $CDCl_3$) δ 157.7 (d, *J* = 1.5 Hz), 151.5 (d, *J* = 7.1 Hz), 136.6 (d, *J* = 2.1 Hz), 132.3 (d, J = 100.0 Hz), 131.6 (d, *J* = 2.8 Hz), 131.1 (d, *J* = 9.5 Hz), 128.3 (d, *J* = 11.9 Hz), 121.7 (d, *J* = 3.7 Hz), 121.2 (d, *J* = 2.5 Hz), 40.73 (d, *J* = 64.7 Hz), 24.08. ^{31}P NMR (162 MHz, $CDCl_3$) δ 30.10. (*66*)

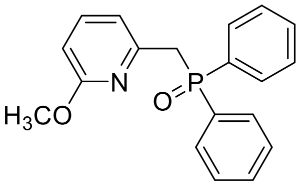

((6-methoxypyridin-3-yl)methyl)diphenylphosphine oxide (225): The title compound was prepared according to the general procedure and purified by column chromatography on silica gel to afford a yellow oil in 83% yield (53.6 mg). ^{1}H NMR (400 MHz, $CDCl_3$) δ 7.76 (dd, J = 11.4, 7.7 Hz, 4H), 7.50 – 7.37 (m, 7H), 6.87 (d, J = 7.0 Hz, 1H), 6.48 (d, J = 8.3 Hz, 1H), 3.81 (d, J = 14.5 Hz, 2H), 3.55 (s, 3H). ^{13}C NMR (101 MHz, $CDCl_3$) δ 163.2 (d, J = 1.9 Hz), 149.6 (d, J = 7.9 Hz), 138.8 (d, J = 2.4 Hz), 132.5 (d, J = 100.0 Hz), 131.6 (d, J = 2.7 Hz), 131.2 (d, J = 9.5 Hz), 128.3 (d, J = 11.9 Hz), 117.7 (d, J = 4.9 Hz), 108.7 (d, J = 2.9 Hz), 53.0, 40.3 (d, J = 65.4 Hz). ^{31}P NMR (162 MHz, $CDCl_3$) δ 30.56. HRMS (EI) m/z: $[M]^+$ calcd for $C_{19}H_{18}NO_2P$: 323.1075; found, 323.1077.

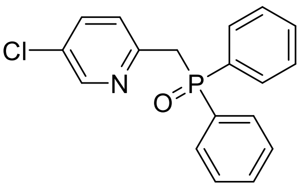

((5-chloropyridin-2-yl)methyl)diphenylphosphine oxide (226): The title compound was prepared according to the general procedure and purified by column chromatography on silica gel to afford a yellow solid (mp: 164-165 ºC) in 76% yield (49.7 mg); ^{1}H NMR (400 MHz, $CDCl_3$) δ 8.33 (s, 1H), 7.73 (dd, J = 11.0, 7.9 Hz, 4H), 7.51 (dd, J = 16.1, 7.9 Hz, 3H), 7.44 (d, J = 4.6 Hz, 5H), 3.90 (d, J = 13.9 Hz, 2H). ^{13}C NMR (101 MHz, $CDCl_3$) δ 150.7 (d, J = 6.5 Hz), 148.0, 136.2, 132.0 (d, J = 100.0 Hz), 131.9 (d, J = 2.4 Hz), 131.0 (d, J = 9.4 Hz), 130.5 (d, J = 2.7 Hz), 128.6 (d, J = 11.9 Hz), 125.7, 40.2 (d, J = 63.8 Hz). ^{31}P NMR (162 MHz, $CDCl_3$) δ 29.56. HRMS (EI) m/z: $[M]^+$ calcd for $C_{18}H_{15}ClNOP$: 327.0580; found, 327.0581.

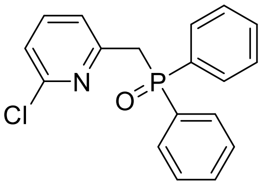

((6-chloropyridin-2-yl)methyl)diphenylphosphine oxide (227): The title compound was prepared according to the general procedure and purified by column chromatography on silica gel to afford a brown oil in 80% yield (52.3 mg). ^{1}H NMR (400 MHz, $CDCl_3$) δ 7.71 (dd, J = 11.2, 7.8 Hz, 4H), 7.50 – 7.36 (m, 8H), 7.06 (d, J = 7.6 Hz, 1H), 3.85 (d, J = 14.1 Hz, 2H). ^{13}C NMR (101 MHz, $CDCl_3$) δ 153.2 (d, J = 6.9 Hz), 150.3 (d, J = 1.7 Hz), 138.9 (d, J = 2.2 Hz), 131.9 (d, J = 2.8 Hz), 131.8 (d, J = 101.0 Hz), 130.9 (d, J = 9.5 Hz), 128.4 (d, J = 12.0 Hz), 123.3 (d, J = 3.6 Hz), 122.2 (d, J = 2.5 Hz), 40.3 (d, J = 63.9 Hz). ^{31}P NMR (162 MHz, $CDCl_3$) δ 29.55. HRMS (EI) m/z: $[M]^+$ calcd for $C_{18}H_{15}ClNOP$: 327.0580; found, 327.0579.

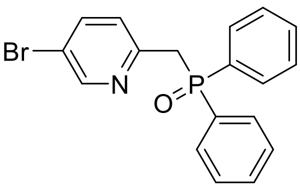

((5-bromopyridin-2-yl)methyl)diphenylphosphine oxide (228): The title compound was prepared according to the general procedure and purified by column chromatography on silica gel to afford a yellow solid (mp: 178-179 ºC) in 72% yield (53.4 mg); ^{1}H NMR (400 MHz, $CDCl_3$) δ 8.40 (s, 1H), 7.71 (dd, *J* = 11.0, 7.9 Hz, 4H), 7.64 (d, *J* = 8.0 Hz, 1H), 7.53 – 7.31 (m, 7H), 3.86 (d, *J* = 14.0 Hz, 1H). ^{13}C NMR (101 MHz, $CDCl_3$) δ 151.0 (d, *J* = 7.2 Hz), 150.1, 138.9 (d, *J* = 2.1 Hz), 131.9 (d, *J* = 2.7 Hz), 131.8 (d, *J* = 101.0 Hz), 130.9 (d, *J* = 9.5 Hz), 128.5 (d, *J* = 12.0 Hz), 126.1 (d, *J* = 3.3 Hz), 119.0 (d, *J* = 3.0 Hz), 40.1 (d, *J* = 64.1 Hz). ^{31}P NMR (162 MHz, $CDCl_3$) δ 29.39. IR (neat, ν/cm^{-1}) 3052, 2947, 2890, 1462, 1434, 1361, 1241, 1178, 1119, 1005, 853, 823, 747, 731, 689, 642. HRMS (EI) m/z: [M]$^+$ calcd for $C_{18}H_{15}BrNOP$: 371.0075; found, 371.0072.

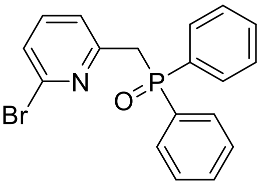

((6-bromopyridin-2-yl)methyl)diphenylphosphine oxide (229): The title compound was prepared according to the general procedure and purified by column chromatography on silica gel to afford a brown oil in 70% yield (51.9 mg). ^{1}H NMR (400 MHz, $CDCl_3$) δ 7.70 (dd, *J* = 11.3, 7.7 Hz, 4H), 7.48 – 7.32 (m, 8H), 7.19 (d, *J* = 7.7 Hz, 1H), 3.85 (d, *J* = 14.1 Hz, 2H). ^{13}C NMR (101 MHz, $CDCl_3$) δ 153.6 (d, *J* = 7.0 Hz), 140.9 (d, *J* = 1.8 Hz), 138.6 (d, *J* = 2.2 Hz), 131.9 (d, *J* = 2.8 Hz), 131.7 (d, *J* = 100.0 Hz), 130.9 (d, *J* = 9.6 Hz), 128.4 (d, *J* = 12.0 Hz), 126.0 (d, *J* = 2.5 Hz), 123.6 (d, *J* = 3.6 Hz), 40.3 (d, *J* = 63.7 Hz). ^{31}P NMR (162 MHz, $CDCl_3$) δ 29.77. IR (neat, ν/cm^{-1}) 3052, 2952, 2905, 1578, 1552, 1435, 1190, 1103, 870, 832, 802, 749, 731, 719, 693, 672, 622. HRMS (EI) m/z: [M]$^+$ calcd for $C_{18}H_{15}BrNOP$: 371.0075; found, 371.0065.

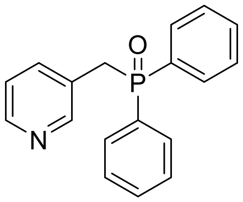

diphenyl(pyridin-3-ylmethyl)phosphine oxide (230): The title compound was prepared according to the general procedure and purified by column chromatography on silica gel to afford a brown solid (mp: 203-204 ºC) in 81% yield (47.5 mg); ^{1}H NMR (400 MHz, $CDCl_3$) δ 7.70 – 7.62 (m, 4H), 7.48 (d, *J* = 6.8 Hz, 2H), 7.42 (d, *J* = 6.0 Hz, 4H), 7.13 – 6.95 (m, 4H), 3.58 (d, *J* = 13.6 Hz, 2H). ^{13}C NMR (101 MHz, $CDCl_3$) δ 150.4 (d, *J* = 5.8 Hz), 147.9 (d, *J* = 2.1 Hz), 137.6 (d, *J* = 4.4 Hz), 132.0 (d, *J* = 2.8 Hz), 131.7 (d, *J* = 9.9 Hz), 131.5 (d, *J* = 99.0 Hz), 130.9 (d, *J* = 9.3 Hz), 128.6 (d, *J* = 11.8 Hz), 123.2, 35.0 (d, *J* = 65.9 Hz). ^{31}P NMR (162 MHz, $CDCl_3$) δ 29.39.

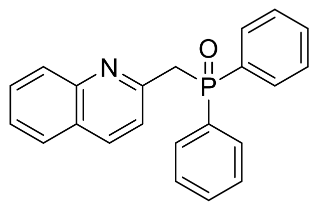

diphenyl(quinolin-2-ylmethyl)phosphine oxide (231): The title compound was prepared according to the general procedure and purified by column chromatography on silica gel to afford a white solid (mp: 150-151 ºC) in 90% yield (61.8 mg); ^{1}H NMR (400 MHz, $CDCl_3$) δ 7.98 (d, *J* = 8.4 1Hz, 1H), 7.87 (d, *J* = 8.4 Hz, 1H), 7.77 (dd, *J* = 11.1, 7.8 Hz, 4H), 7.69 (d, *J* = 8.0 Hz, 1H), 7.58 (dd, *J* = 14.4, 7.7 Hz, 2H), 7.44 – 7.34 (m, 7H), 4.10 (d, *J* = 14.3 Hz, 2H). ^{13}C NMR (101 MHz, $CDCl_3$) δ 153.2 (d, *J* = 6.9 Hz), 147.7 (d, *J* = 1.5 Hz), 136.2, 132.7, 131.7 (d, *J* = 2.8 Hz), 131.0 (d, *J* = 9.5 Hz), 129.3, 128.5, 128.4 (d, *J* = 12.0 Hz), 127.4, 126.7 (d, *J* = 1.5 Hz), 126.1, 122.6 (d, *J* = 2.4 Hz), 41.7 (d, *J* = 63.6 Hz). ^{31}P NMR (162 MHz, $CDCl_3$) δ 29.93. (*66*)

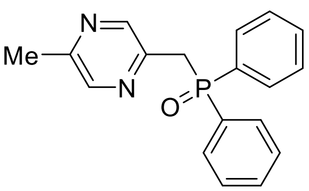

((5-methylpyrazin-2-yl)methyl)diphenylphosphine oxide (232): The title compound was prepared according to the general procedure and purified by column chromatography on silica gel to afford a yellow oil in 55% yield (33.9 mg); ^{1}H NMR (400 MHz, $CDCl_3$) δ 8.38 (s, 1H), 8.17 (s, 1H), 7.77 – 7.54 (m, 4H), 7.41 (dd, *J* = 18.2, 6.4 Hz, 6H), 3.83 (d, *J* = 13.8 Hz, 1H), 2.38 (s, 1H). ^{13}C NMR (101 MHz, $CDCl_3$) δ 151.5, 145.1 (d, *J* = 7.7 Hz), 144.5 (d, *J* = 3.4 Hz), 143.5, 131.9 (d, *J* = 2.2 Hz), 131.7 (d, *J* = 100.0 Hz), 130.9 (d, *J* = 9.4 Hz), 128.4 (d, *J* = 11.9 Hz), 37.8 (d, *J* = 64.3 Hz), 20.9. ^{31}P NMR (162 MHz, $CDCl_3$) δ 29.31. IR (neat, ν/cm^{-1}) 3076, 3052, 2949, 2902, 1488, 1434, 1342, 1262, 1195, 1126, 1039, 905, 844, 752, 731, 714, 689. HRMS (EI) m/z: $[M]^+$ calcd for $C_{18}H_{17}N_2OP$: 308.1078; found, 308.1081.

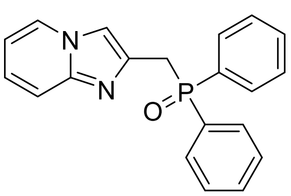

(imidazo[1,2-*a*]pyridin-2-ylmethyl)diphenylphosphine oxide (233): The title compound was prepared according to the general procedure and purified by column chromatography on silica gel to afford a yellow oil in 79% yield (52.5 mg); ^{1}H NMR (400 MHz, $CDCl_3$) δ 9.70 (s, 1H), 8.34 (d, *J* = 6.2 Hz, 1H), 8.05 (s, 1H), 7.80 (dd, *J* = 11.0, 7.6 Hz, 4H), 7.73 (d, *J* = 8.7 Hz, 1H), 7.50 – 7.30 (m, 6H), 7.03 (t, *J* = 6.6 Hz, 1H), 4.17 (d, *J* = 12.9 Hz, 2H). ^{13}C NMR (101 MHz, $CDCl_3$) δ 139.6, 132.2 (d, *J* = 2.6 Hz), 131.3, 130.7 (d, *J* = 9.8 Hz), 130.4 (d, *J* = 14.7 Hz), 128.8 (d, *J* = 12.2 Hz), 127.1, 123.4 (d, *J* = 216.1 Hz), 115.9, 113.4, 112.9 (d, *J* = 4.5 Hz), 28.0 (d, *J* = 66.5 Hz). ^{31}P NMR (162 MHz, $CDCl_3$) δ 29.26. IR (neat, ν/cm^{-1}) 3097, 3050, 2913, 2850, 1655, 1528, 1436, 1361, 1199,

1102, 992, 816, 726, 693, 634. HRMS (EI) m/z: $[M]^+$ calcd for $C_{20}H_{17}N_2OP$: 332.1078; found, 332.1080.

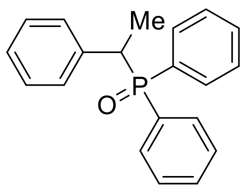

diphenyl(1-phenylethyl)phosphine oxide (234): The title compound was prepared according to the general procedure and purified by column chromatography on silica gel to afford a white solid (mp: 157-158 ºC) in 71% yield (43.5 mg); ^{1}H NMR (400 MHz, $CDCl_3$) δ 7.98 (t, *J* =9.2 Hz, 2H), 7.65 – 7.49 (m, 5H), 7.41 (t, *J* = 7.2 Hz, 1H), 7.35 – 7.21 (m, 7H), 3.68 (p, *J* = 7.2 Hz, 1H), 1.65 (dd, *J* = 16.1, 7.3 Hz, 3H). ^{13}C NMR (101 MHz, $CDCl_3$) δ 137.8 (d, *J* = 5.5 Hz), 131.6 (d, *J* = 2.7 Hz), 131.3 (d, *J* = 8.4 Hz), 131.2 (d, *J* = 2.8 Hz), 131.0 (d, *J* = 8.7 Hz), 129.1 (d, *J* = 5.5 Hz), 128.6 (d, *J* = 11.2 Hz), 128.2 (d, *J* = 2.0 Hz), 127.9 (d, *J* = 11.5 Hz), 126.8 (d, *J* = 2.5 Hz), 40.9 (d, *J* = 67.2 Hz), 15.4 (d, *J* = 2.8 Hz). ^{31}P NMR (162 MHz, $CDCl_3$) δ 33.56. (*67*)

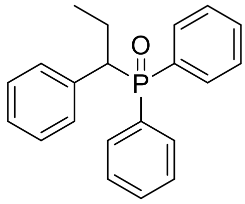

diphenyl(1-phenylpropyl)phosphine oxide (235): The title compound was prepared according to the general procedure and purified by column chromatography on silica gel to afford a white solid (mp: 181-182 ºC) in 81% yield (51.9 mg); ^{1}H NMR (400 MHz, $CDCl_3$) δ 7.90 (dd, *J* = 12.9, 4.8 Hz, 2H), 7.51 (d, *J* = 6.6 Hz, 3H), 7.42 (dd, *J* = 10.0, 8.8 Hz, 2H), 7.29 (t, *J* = 7.3 Hz, 1H), 7.24 – 7.11 (m, 7H), 3.37 – 3.24 (m, 1H), 2.23 – 2.03 (m, 1H), 2.04 – 1.86 (m, 1H), 0.78 (t, *J* = 7.3 Hz, 3H). ^{13}C NMR (101 MHz, $CDCl_3$) δ 135.6 (d, *J* = 5.4 Hz), 131.6 (d, *J* = 2.7 Hz), 131.2 (d, *J* = 8.4 Hz), 131.1 (d, *J* = 2.7 Hz), 130.9 (d, *J* = 8.7 Hz), 129.8 (d, *J* = 5.9 Hz), 128.6 (d, *J* = 11.2 Hz), 128.1 (d, *J* = 1.8 Hz), 127.9 (d, *J* = 11.6 Hz), 126.8 (d, *J* = 2.5 Hz), 48.8 (d, *J* = 67.6 Hz), 22.5, 12.6 (d, *J* = 14.0 Hz). ^{31}P NMR (162 MHz, $CDCl_3$) δ 32.77.

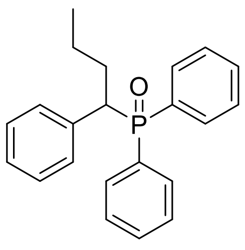

diphenyl(1-phenylbutyl)phosphine oxide (236): The title compound was prepared according to the general procedure and purified by column chromatography on silica gel to afford a white solid (mp: 212-213 ºC) in 69% yield (46.1 mg); ^{1}H NMR (400 MHz, $CDCl_3$) δ 7.90 (t, *J* = 8.8 Hz, 2H), 7.53 (s, 3H), 7.41 (t, *J* = 6.8 Hz, 2H), 7.30 (t, *J* = 7.0 Hz, 1H), 7.18 (dd, *J* = 15.6, 8.2 Hz, 7H), 3.42 (t, *J* = 9.7 Hz, 1H), 2.13 (dd, *J* = 20.7, 13.4 Hz, 1H), 1.84 (d, *J* = 7.6 Hz, 1H), 1.25 (s, 1H), 1.11 (td, *J* = 14.5, 7.4 Hz, 1H), 0.78 (t, *J* = 7.1 Hz, 3H). ^{13}C NMR (101 MHz,

$CDCl_3$) δ 135.9 (d, *J* = 5.6 Hz), 131.6 (d, *J* = 2.7 Hz), 131.3 (d, *J* = 8.3 Hz), 131.0 (d, *J* = 2.7 Hz), 130.9 (d, *J* = 8.7 Hz), 129.8 (d, *J* = 5.9 Hz), 128.6 (d, *J* = 11.2 Hz), 128.2 (d, *J* = 2.0 Hz), 127.9 (d, *J* = 11.6 Hz), 126.8 (d, *J* = 2.6 Hz), 46.6 (d, *J* = 67.5 Hz), 31.0 (d, *J* = 1.5 Hz), 20.8 (d, *J* = 13.3 Hz), 13.5 (s). ^{31}P NMR (162 MHz, $CDCl_3$) δ 32.76. (*67*)

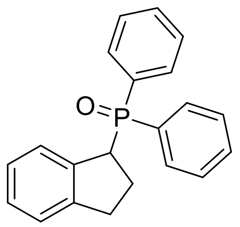

(2,3-dihydro-1*H*-inden-1-yl)diphenylphosphine oxide (237): The title compound was prepared according to the general procedure and purified by column chromatography on silica gel to afford a white solid (mp: 151-152 ºC) in 71% yield (45.2 mg); ^{1}H NMR (400 MHz, $CDCl_3$) δ 7.78 – 7.70 (m, 2H), 7.65 – 7.56 (m, 2H), 7.52 (t, *J* = 7.3 Hz, 2H), 7.47 – 7.37 (m, 4H), 7.13 (d, *J* = 3.8 Hz, 2H), 6.99 – 6.89 (m, 1H), 6.61 (d, *J* = 7.6 Hz, 1H), 4.18 (td, *J* = 9.7, 3.9 Hz, 1H), 2.86 – 2.74 (m, 1H), 2.72 – 2.59 (m, 1H), 2.56 – 2.39 (m, 2H). ^{13}C NMR (101 MHz, $CDCl_3$) δ 145.5 (d, *J* = 5.9 Hz), 137.8 (d, *J* = 5.2 Hz), 131.8 (d, *J* = 8.6 Hz), 131.3 (d, *J* = 8.9 Hz), 128.5 (d, *J* = 11.3 Hz), 128.3 (d, *J* = 11.3 Hz), 127.4 (d, *J* = 2.9 Hz), 126.0 (d, *J* = 2.7 Hz), 125.4 (d, *J* = 3.4 Hz), 124.7 (d, *J* = 2.3 Hz), 45.5 (d, *J* = 69.5 Hz), 31.6, 26.6. HRMS (EI) m/z: $[M]^+$ calcd for $C_{21}H_{19}OP$: 318.1174; found, 318.1177.

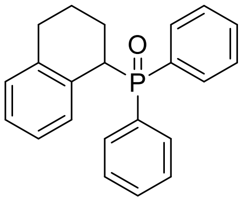

diphenyl(1,2,3,4-tetrahydronaphthalen-1-yl)phosphine oxide (238): The title compound was prepared according to the general procedure and purified by column chromatography on silica gel to afford a white solid (mp: 146-147 ºC) in 77% yield (51.1 mg); ^{1}H NMR (400 MHz, $CDCl_3$) δ 7.81 (dd, *J* = 19.2, 10.7 Hz, 2H), 7.50 (dt, *J* = 17.2, 8.1 Hz, 6H), 7.36 (t, *J* = 6.2 Hz, 2H), 7.08 (t, *J* = 7.6 Hz, 2H), 6.79 (t, *J* = 6.7 Hz, 1H), 6.46 (d, *J* = 7.6 Hz, 1H), 3.89 (dt, *J* = 11.0, 5.2 Hz, 1H), 2.65 (s, 2H), 2.41 – 2.27 (m, 1H), 2.09 – 1.87 (m, 2H), 1.66 – 1.50 (m, 1H). ^{13}C NMR (101 MHz, $CDCl_3$) δ 139.4 (d, *J* = 5.3 Hz), 131.9 (d, *J* = 8.3 Hz), 131.6 (d, *J* = 8.0 Hz), 131.5 (d, *J* = 8.0 Hz), 129.8 (d, *J* = 4.2 Hz), 129.4 (d, *J* = 2.5 Hz), 128.5 (d, *J* = 11.2 Hz), 128.1 (d, *J* = 11.3 Hz), 126.6 (d, *J* = 3.1 Hz), 125.0 (d, *J* = 2.9 Hz), 40.4 (d, *J* = 66.7 Hz), 29.0, 23.7 (d, *J* = 2.4 Hz), 20.5 (d, *J* = 4.0 Hz). ^{31}P NMR (162 MHz, $CDCl_3$) δ 34.60. (*67*)

(6-(*tert*-butyl)-1,1-dimethyl-2,3-dihydro-1*H*-inden-4-yl)ethyl)diphenylphosphine oxide (239):

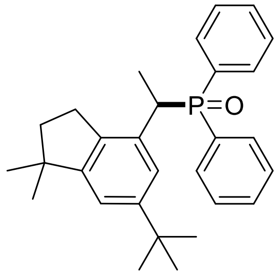

The title compound was prepared according to the general procedure and purified by column chromatography on silica gel to afford a yellow oil in 74% yield (63.7 mg); ^{1}H NMR (400 MHz, $CDCl_3$) δ 7.97 – 7.79 (m, 2H), 7.64 – 7.51 (m, 3H), 7.40 (t, *J* = 1.8 Hz, 1H), 7.36 – 7.25 (m, 3H), 7.22 – 7.15 (m, 2H), 7.02 (s, 1H), 3.76 – 3.61 (m, 1H), 2.67 – 2.53 (m, 1H), 1.98 (ddd, *J* = 15.3, 8.3, 1.4 Hz, 1H), 1.78 – 1.70 (m, 1H), 1.64 (dd, *J* = 16.4, 7.3 Hz, 3H), 1.53 (ddd, *J* = 12.5, 8.2, 4.7 Hz, 1H), 1.34 (s, 9H), 1.23 (s, 3H), 1.00 (s, 3H). ^{13}C NMR (101 MHz, $CDCl_3$) δ 151.9 (d, *J* = 2.0 Hz), 150.1 (d, *J* = 2.6 Hz), 138.8 (d, *J* = 6.3 Hz), 132.5 (d, *J* = 5.6 Hz), 131.7 (d, *J* = 8.1 Hz), 131.1 (d, *J* = 9.1 Hz), 128.6 (d, *J* = 11.0 Hz), 127.7 (d, *J* = 11.5 Hz), 123.7 (d, *J* = 4.2 Hz), 117.3 (d, *J* = 2.7 Hz), 44.1, 40.9, 38.1 (d, *J* = 67.2 Hz), 34.7, 31.5, 28.5 (d, *J* = 37.0 Hz), 28.0, 14.7 (d, *J* = 3.1 Hz). ^{31}P NMR (162 MHz, $CDCl_3$) δ 33.86. IR (neat, ν/cm^{-1}) 3052, 3039, 3022, 2949, 2858, 1436, 1359, 1183, 1116, 905, 875, 719, 691. HRMS (EI) m/z: $[M]^+$ calcd for $C_{29}H_{35}OP$: 430.2426; found, 430.2424.

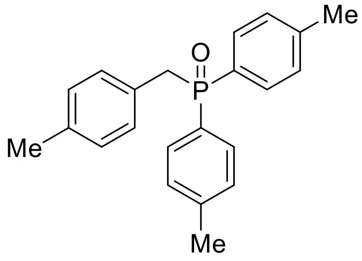

(4-methylbenzyl)di-*p*-tolylphosphine oxide (240): The title compound was prepared according to the general procedure and purified by column chromatography on silica gel to afford a white solid (mp: 185-186 ºC) in 80% yield (53.5 mg); ^{1}H NMR (400 MHz, $CDCl_3$) δ 7.55 (dd, *J* = 10.9, 8.1 Hz, 4H), 7.21 (d, *J* = 6.8 Hz, 4H), 6.98 (s, 4H), 3.57 (d, *J* = 13.6 Hz, 2H), 2.35 (s, 6H), 2.24 (s, 3H). ^{13}C NMR (101 MHz, $CDCl_3$) δ 141.9 (d, *J* = 2.2 Hz), 136.1 (d, *J* = 2.9 Hz), 131.1 (d, *J* = 9.4 Hz), 129.9 (d, *J* = 5.1 Hz), 129.2 (d, *J* = 101.0 Hz), 129.1 (d, *J* = 12.1 Hz), 129.0, 128.1 (d, *J* = 7.7 Hz), 37.7 (d, *J* = 66.8 Hz), 21.4, 20.9. ^{31}P NMR (162 MHz, $CDCl_3$) δ 29.92. HRMS (EI) m/z: $[M]^+$ calcd for $C_{22}H_{23}OP$: 334.1487; found, 334.1490.

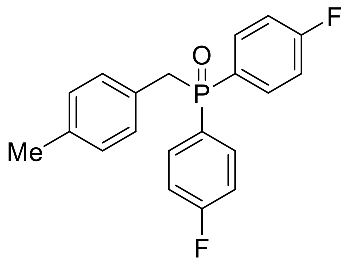

bis(4-fluorophenyl)(4-methylbenzyl)phosphine oxide (241): The title compound was prepared according to the general procedure and purified by column chromatography on silica gel to afford a white solid (mp: 191-192 ºC) in 68% yield (45.6 mg); ^{1}H NMR (400 MHz, $CDCl_3$) δ 7.65 (s, 4H), 7.12 (t, *J* = 7.6 Hz, 4H), 7.03 – 6.90 (m, 4H), 3.57 (d, *J* = 13.2 Hz, 2H), 2.25 (s, 3H). ^{13}C NMR (101 MHz, $CDCl_3$) δ 164.9 (d, *J* = 254.0 Hz), 136.6, 133.6 (t, *J* = 9.5 Hz), 129.9 (d, *J* = 4.8 Hz), 129.2, 128.0 (d, *J* = 102.2 Hz), 127.3 (d, *J* = 7.9 Hz), 115.9 (dd, *J* = 21.4, 12.7 Hz), 37.9 (d, *J* = 67.8 Hz), 21.0. ^{31}P NMR (162 MHz, $CDCl_3$) δ 28.60. ^{19}F NMR (376 MHz, $CDCl_3$) δ -106.64. HRMS (EI) m/z: $[M]^+$ calcd for $C_{20}H_{17}F_2ON$: 342.0985; found, 342.0983.

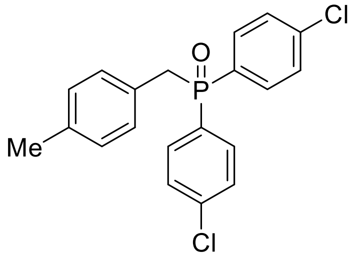

bis(4-chlorophenyl)(4-methylbenzyl)phosphine oxide (242): The title compound was prepared according to the general procedure and purified by column chromatography on silica gel to afford a white solid (mp: 247-248 ºC) in 79% yield (59.1 mg); ^{1}H NMR (400 MHz, $CDCl_3$) δ 7.58 (dd, *J* = 10.9, 8.4 Hz, 4H), 7.41 (dd, *J* = 8.4, 2.1 Hz, 4H), 7.05 – 6.92 (m, 4H), 3.58 (d, *J* = 13.7 Hz, 2H), 2.26 (s, 3H). ^{13}C NMR (101 MHz, $CDCl_3$) δ 138.6 (d, *J* = 3.3 Hz), 136.7 (d, *J* = 3.2 Hz), 132.5 (d, *J* = 9.9 Hz), 130.5 (d, *J* = 100.0 Hz), 129.9 (d, *J* = 5.4 Hz), 129.2 (d, *J* = 2.6 Hz), 128.9 (d, *J* = 12.3 Hz), 127.1 (d, *J* = 8.2 Hz), 37.5 (d, *J* = 68.0 Hz), 21.0. ^{31}P NMR (162 MHz, $CDCl_3$) δ 28.43. HRMS (EI) m/z: $[M]^+$ calcd for $C_{20}H_{17}Cl_2OP$: 374.0394; found, 374.0392.

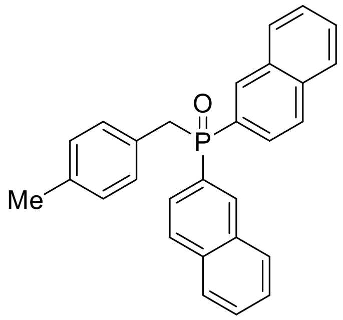

(4-methylbenzyl)di(naphthalen-2-yl)phosphine oxide (243): The title compound was prepared according to the general procedure and purified by column chromatography on silica gel to afford a yellow solid (mp: 206-207 ºC) in 60% yield (48.7 mg); ^{1}H NMR (400 MHz, $CDCl_3$) δ 8.36 (d, *J* = 13.2 Hz, 2H), 7.88 (dd, *J* = 15.1, 8.0 Hz, 6H), 7.71 (t, *J* = 8.9 Hz, 2H), 7.55 (dt, *J* = 14.9, 6.9 Hz, 4H), 7.05 (d, *J* = 7.4 Hz, 2H), 6.97 (d, *J* = 7.5 Hz, 2H), 3.82 (d, *J* = 13.6 Hz, 2H), 2.23 (s, 3H). ^{13}C NMR (101 MHz, $CDCl_3$) δ 136.4 (d, *J* = 3.1 Hz), 134.6 (d, *J* = 2.2 Hz), 133.3 (d, *J* = 8.2 Hz),

132.5 (d, *J* = 12.8 Hz), 130.0 (d, *J* = 5.3 Hz), 129.2, 129.0, 128.9, 128.3, 128.2, 128.1, 127.8, 126.9, 125.9 (d, *J* = 10.4 Hz), 37.5 (d, *J* = 67.5 Hz), 21.0. ^{31}P NMR (162 MHz, $CDCl_3$) δ 29.97. IR (neat, ν/cm^{-1}) 3052, 2923, 2853, 1515, 1185, 1173, 1136, 1088, 858, 823, 748, 696, 649. HRMS (EI) m/z: $[M]^+$ calcd for $C_{28}H_{23}OP$: 406.1487; found, 406.1484.

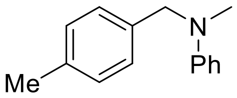

***N*-methyl-*N*-(4-methylbenzyl)aniline (250):** The title compound was prepared according to the general procedure and purified by column chromatography on silica gel to afford a yellow oil in 84% yield (35.4 mg); ^{1}H NMR (400 MHz, $CDCl_3$) δ 7.27 – 7.19 (m, 2H), 7.14 (s, 4H), 6.78 (d, *J* = 8.2 Hz, 2H), 6.73 (t, *J* = 7.2 Hz, 1H), 4.51 (s, 2H), 3.01 (s, 3H), 2.35 (s, 3H). ^{13}C NMR (101 MHz, $CDCl_3$) δ 149.8, 136.4, 135.8, 129.2, 129.1, 126.7, 116.5, 112.4, 56.4, 38.4, 21.0.

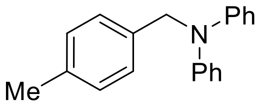

***N*-(4-methylbenzyl)-*N*-phenylaniline (251):** The title compound was prepared according to the general procedure and purified by column chromatography on silica gel to afford a yellow oil in 90% yield (49.2 mg); ^{1}H NMR (400 MHz, $CDCl_3$) δ 7.34 (dd, *J* = 10.9, 4.4 Hz, 6H), 7.20 (t, *J* = 9.1 Hz, 6H), 7.03 (t, *J* = 7.1 Hz, 2H), 5.07 (s, 2H), 2.42 (s, 3H). ^{13}C NMR (101 MHz, $CDCl_3$) δ 148.0, 136.2, 136.0, 129.4, 129.2, 126.4, 121.2, 120.6, 56.0, 21.0. IR (neat, ν/cm^{-1}) 3080, 3050, 3033, 3020, 2918, 2857, 1587, 1491, 1366, 1250, 1213, 1093, 1009, 989, 863, 797, 752, 691. HRMS (EI) m/z: $[M]^+$ calcd for $C_{20}H_{19}N$: 273.1517; found, 273.1517.

***N*-(4-methylbenzyl)-*N*-phenylnaphthalen-2-amine (252):** The title compound was prepared

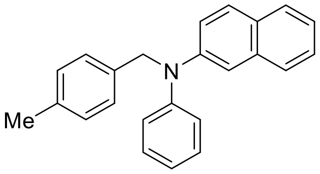

according to the general procedure and purified by column chromatography on silica gel to afford a brown oil in 81% yield (52.4 mg); ^{1}H NMR (400 MHz, $CDCl_3$) δ 7.85 (dd, *J* = 14.4, 8.5 Hz, 2H), 7.76 (d, *J* = 8.2 Hz, 1H), 7.56 (d, *J* = 1.7 Hz, 1H), 7.54 – 7.38 (m, 7H), 7.29 (dd, *J* = 16.4, 7.9 Hz, 4H), 7.13 (t, *J* = 7.3 Hz, 1H), 5.23 (s, 2H), 2.46 (s, 3H). ^{13}C NMR (101 MHz, $CDCl_3$) δ 148.0, 145.6, 136.3, 135.9, 134.6, 129.2, 129.2, 129.2, 128.7, 127.4, 126.7, 126.5, 126.1, 123.7, 121.9, 121.7, 121.1, 115.5, 56.2, 21.0. IR (neat, ν/cm^{-1}) 3050, 3022, 2965, 2912, 2850, 1627, 1587, 1491, 1469, 1376, 1272, 1220, 1020, 902, 835, 801, 767, 749, 707, 691, 656. HRMS (EI) m/z: $[M]^+$ calcd for $C_{24}H_{21}N$: 323.1674; found, 323.1673.

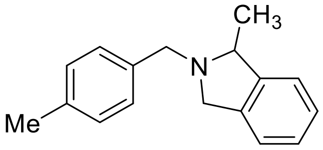

1-methyl-2-(4-methylbenzyl)isoindoline (253): The title compound was prepared according to the general procedure and purified by column chromatography on silica gel to afford a brown oil in 75% yield (35.6 mg); ^{1}H NMR (400 MHz, $CDCl_3$) δ 7.29 (d, *J* = 7.8 Hz, 2H), 7.16 (d, *J* = 7.8 Hz, 2H), 7.08 (d, *J* = 7.2 Hz, 1H), 7.03 (t, *J* = 7.6 Hz, 1H), 6.66 (t, *J* = 7.3 Hz, 1H), 6.37 (d, *J* = 7.8 Hz, 1H), 4.38 (d, *J* = 15.9 Hz, 1H), 4.19 (d, *J* = 15.9 Hz, 1H), 3.76 (tq, *J* = 9.0, 6.1 Hz, 1H), 3.20 (dd, *J* = 15.4, 8.6 Hz, 1H), 2.71 (dd, *J* = 15.4, 9.5 Hz, 1H), 2.38 (s, 3H), 1.33 (d, *J* = 6.1 Hz, 3H). ^{13}C NMR (101 MHz, $CDCl_3$) δ 152.7, 136.4, 136.1, 129.1, 128.7, 127.3, 127.3, 124.1, 117.2, 106.8, 60.4, 50.7, 37.3, 21.1, 19.6. IR (neat, ν/cm^{-1}) 3048, 3020, 2961, 2921, 2838, 1604, 1482, 1458, 1352, 1267, 1021, 797, 745, 713. HRMS (EI) m/z: $[M]^+$ calcd for $C_{17}H_{19}N$: 237.1517; found, 237.1518.

10-(4-methylbenzyl)-10*H*-benzo[*b*]pyrido[2,3-*e*][1,4]thiazine (254): The title compound was

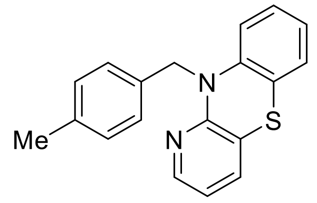

prepared according to the general procedure and purified by column chromatography on silica gel to afford a brown oil in 91% yield (55.3 mg); ^{1}H NMR (400 MHz, $CDCl_3$) δ 8.01 (d, *J* = 4.7 Hz, 1H), 7.28 (t, *J* = 10.4 Hz, 3H), 7.18 (d, *J* = 7.8 Hz, 2H), 7.08 (d, *J* = 7.5 Hz, 1H), 6.99 (t, *J* = 7.6 Hz, 1H), 6.89 (t, *J* = 7.4 Hz, 1H), 6.80 – 6.71 (m, 2H), 5.40 (s, 2H), 2.38 (s, 3H). ^{13}C NMR (101 MHz, $CDCl_3$) δ 154.4, 145.0, 142.4, 135.9, 134.4, 133.8, 129.0, 127.2, 126.4, 126.4, 122.7, 120.6, 117.8, 116.6, 116.1, 48.7, 20.9. IR (neat, ν/cm^{-1}) 3095, 3052, 3015, 2925, 2850, 1590, 1479, 1444, 1406, 1216, 1124, 1108, 1044, 877, 776, 738, 651. HRMS (EI) m/z: $[M]^+$ calcd for $C_{19}H_{16}N_2S$: 304.1034; found, 304.1035.

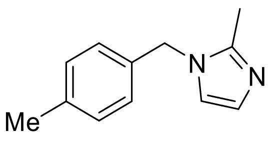

2-methyl-1-(4-methylbenzyl)-1*H*-imidazole (255): The title compound was prepared according to the general procedure and purified by column chromatography on silica gel to afford a brown oil in 69% yield (25.7 mg); ^{1}H NMR (400 MHz, $CDCl_3$) δ 7.13 (d, *J* = 7.6 Hz, 2H), 6.94 (d, *J* = 8.4 Hz, 3H), 6.81 (s, 1H), 4.99 (s, 2H), 2.33 (s, 6H). ^{13}C NMR (101 MHz, $CDCl_3$) δ 144.9, 137.7, 133.3, 129.6, 127.1, 126.7, 119.8, 49.5, 21.0, 13.1.

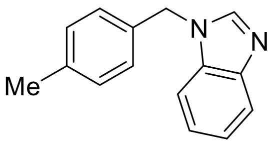

1-(4-methylbenzyl)-1*H*-benzo[d]imidazole (256): The title compound was prepared according to the general procedure and purified by column chromatography on silica gel to afford a yellow solid (mp: 51-52 ºC) in 72% yield (32.0 mg); ^{1}H NMR (400 MHz, $CDCl_3$) δ 8.14 (s, 1H), 7.84 (d, *J* = 7.3 Hz, 1H), 7.26 (dd, *J* = 16.5, 9.0 Hz, 3H), 7.10 (dd, *J* = 16.5, 7.5 Hz, 4H), 5.27 (s, 2H), 2.31 (s, 3H). ^{13}C NMR (101 MHz, $CDCl_3$) δ 142.8, 142.5, 137.9, 133.4, 131.9, 129.4, 127.0, 123.1, 122.4, 119.6, 110.1, 48.5, 20.8.

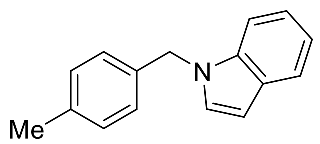

1-(4-methylbenzyl)-1*H*-indole (257): The title compound was prepared according to the general procedure and purified by column chromatography on silica gel to afford a yellow oil in 85% yield (37.6 mg); ^{1}H NMR (400 MHz, $CDCl_3$) δ 7.76 (d, *J* = 7.7 Hz, 1H), 7.39 (d, *J* = 8.1 Hz, 1H), 7.28 (d, *J* = 6.4 Hz, 1H), 7.21 (t, *J* = 9.6 Hz, 4H), 7.11 (d, *J* = 7.7 Hz, 2H), 6.65 (s, 1H), 5.35 (s, 2H), 2.41 (s, 3H). ^{13}C NMR (101 MHz, $CDCl_3$) δ 137.2, 136.3, 134.4, 129.4, 128.7, 128.1, 126.8, 121.6, 120.9, 119.4, 109.7, 101.5, 49.8, 21.0.

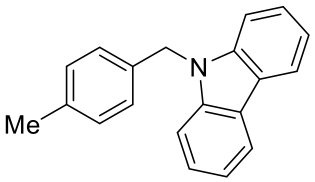

9-(4-methylbenzyl)-9*H*-carbazole (258): The title compound was prepared according to the general procedure and purified by column chromatography on silica gel to afford a yellow solid (mp 116-117 ºC) in 81% yield (43.9 mg); ^{1}H NMR (400 MHz, $CDCl_3$) δ 8.20 (d, *J* = 7.7 Hz, 2H), 7.49 (t, *J* = 7.6 Hz, 2H), 7.42 (d, *J* = 8.1 Hz, 2H), 7.31 (t, *J* = 7.3 Hz, 2H), 7.15 – 7.06 (m, 4H), 5.50 (s, 2H), 2.34 (s, 3H). ^{13}C NMR (101 MHz, $CDCl_3$) δ 140.6, 137.0, 134.1, 129.4, 126.3, 125.8, 123.0, 120.3, 119.1, 108.9, 46.3, 21.0.

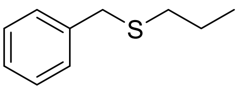

benzyl(propyl)sulfane (259): The title compound was prepared according to the general procedure and purified by column chromatography on silica gel to afford a yellow oil in 82% yield (27.3 mg); ^{1}H NMR (400 MHz, $CDCl_3$) δ 7.31 (d, *J* = 4.4 Hz, 4H), 7.23 (dd, *J* = 8.6, 4.6 Hz, 1H), 3.70 (s, 2H), 2.49 – 2.33 (m, 2H), 1.58 (dd, *J* = 14.1, 6.7 Hz, 2H), 0.95 (t, *J* = 7.4 Hz, 3H). ^{13}C NMR (101 MHz, $CDCl_3$) δ 138.7, 128.8, 128.4, 126.8, 36.2, 33.4, 22.5, 13.5.

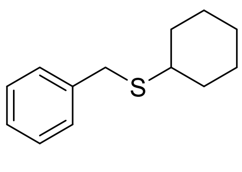

benzyl(cyclohexyl)sulfane (260): The title compound was prepared according to the general procedure and purified by column chromatography on silica gel to afford a yellow oil in 95% yield (39.2 mg); ^{1}H NMR (400 MHz, $CDCl_3$) δ 7.28 (d, *J* = 6.1 Hz, 4H), 7.23 – 7.19 (m, 1H), 3.72 (s, 2H), 2.54 (t, *J* = 9.0 Hz, 1H), 1.92 (d, *J* = 12.6 Hz, 2H), 1.72 (s, 2H), 1.57 (s, 1H), 1.38 – 1.16 (m, 5H). ^{13}C NMR (101 MHz, $CDCl_3$) δ 138.9, 128.7, 128.4, 126.7, 42.9, 34.6, 33.4, 26.0, 25.8.

5-(((furan-2-ylmethyl)thio)methyl)-2,3-dihydrobenzofuran (261): The title compound was

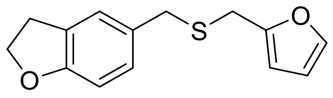

prepared according to the general procedure and purified by column chromatography on silica gel to afford a brown oil in 98% yield (48.3 mg); ^{1}H NMR (400 MHz, $CDCl_3$) δ 7.38 (s, 1H), 7.17 (s, 1H), 7.02 (d, *J* = 8.1 Hz, 1H), 6.72 (d, *J* = 8.0 Hz, 1H), 6.32 (s, 1H), 6.17 (s, 1H), 4.56 (t, *J* = 8.6 Hz, 2H), 3.65 (s, 2H), 3.60 (s, 2H), 3.19 (t, *J* = 8.6 Hz, 2H). ^{13}C NMR (101 MHz, $CDCl_3$) δ 159.2, 151.8, 141.9, 129.6, 128.7, 127.3, 125.5, 110.3, 108.8, 107.3, 71.2, 35.5, 29.6, 27.3. IR (neat, ν/cm^{-1}) 3116, 3048, 3012, 2957, 2912, 2855, 1613, 1488, 1238, 1149, 1100, 1008, 982, 933, 818, 733. HRMS (EI) m/z: $[M]^+$ calcd for $C_{14}H_{14}O_2S$: 246.0715; found, 246.0719.

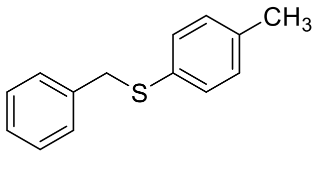

benzyl(*p*-tolyl)sulfane (262): The title compound was prepared according to the general procedure and purified by column chromatography on silica gel to afford a yellow solid in 98% yield (42.0 mg), mp 45-46 ºC; ^{1}H NMR (400 MHz, $CDCl_3$) δ 7.31 (s, 4H), 7.29 – 7.23 (m, 3H), 7.11 (d, *J* = 7.5 Hz, 2H), 4.11 (s, 2H), 2.35 (s, 3H). ^{13}C NMR (101 MHz, $CDCl_3$) δ 137.8, 136.5, 132.5, 130.7, 129.6, 128.8, 128.4, 127.0, 39.8, 21.0.

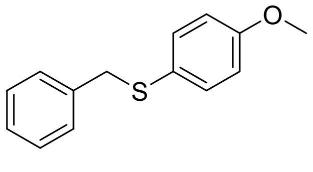

benzyl(4-methoxyphenyl)sulfane (263): The title compound was prepared according to the general procedure and purified by column chromatography on silica gel to afford a yellow solid in 85% yield (39.2 mg), mp 51-52 ºC; ^{1}H NMR (400 MHz, $CDCl_3$) δ 7.27 (dd, *J* = 23.7, 7.1 Hz, 7H), 6.84 (d, *J* = 7.7 Hz, 2H), 4.03 (s, 2H), 3.81 (s, 3H). ^{13}C NMR (101 MHz, $CDCl_3$) δ 159.2, 138.1, 134.0, 128.8, 128.3, 126.9, 126.0, 114.4, 55.2, 41.2. IR (neat, ν/cm^{-1}) 3081, 3059, 3040, 3024, 3012, 2918, 2838,

1597, 1491, 1451, 1282, 1239, 1175, 1021, 806, 710, 693, 637.

benzyl(4-chlorophenyl)sulfane (264): The title compound was prepared according to the general

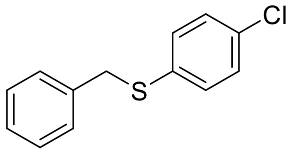

procedure and purified by column chromatography on silica gel to afford a yellow solid in 99% yield (46.5 mg), mp 52-53 ºC; ^{1}H NMR (400 MHz, $CDCl_3$) δ 7.30 (t, *J* = 5.0 Hz, 5H), 7.24 (s, 4H), 4.11 (s, 2H). ^{13}C NMR (101 MHz, $CDCl_3$) δ 137.1, 134.6, 132.5, 131.4, 128.9, 128.8, 128.5, 127.3, 39.3. IR (neat, ν/cm^{-1}) 3079, 3059, 3044, 3022, 2918, 2850, 1476, 1451, 1387, 1091, 1069, 1009, 813, 709, 693. (*68*)

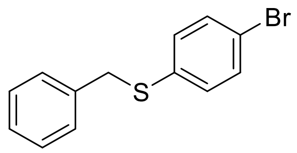

benzyl(4-bromophenyl)sulfane (265): The title compound was prepared according to the general procedure and purified by column chromatography on silica gel to afford a yellow solid in 97% yield (54.3 mg), mp 52-53 ºC; ^{1}H NMR (400 MHz, $CDCl_3$) δ 7.39 (d, *J* = 7.9 Hz, 2H), 7.29 (d, *J* = 7.0 Hz, 5H), 7.17 (d, *J* = 7.8 Hz, 2H), 4.11 (s, 2H). ^{13}C NMR (101 MHz, $CDCl_3$) δ 137.0, 135.4, 131.9, 131.5, 128.8, 128.6, 127.3, 120.3, 39.1. (*68*)

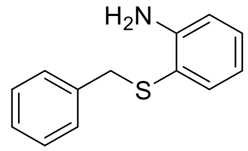

2-(benzylthio)aniline (266): The title compound was prepared according to the general procedure and purified by column chromatography on silica gel to afford a yellow solid in 90% yield (38.8 mg), mp 48-49 ºC; ^{1}H NMR (400 MHz, $CDCl_3$) δ 7.32 – 7.22 (m, 4H), 7.21 – 7.10 (m, 3H), 6.73 (d, *J* = 6.9 Hz, 1H), 6.66 (t, *J* = 7.5 Hz, 1H), 4.29 (s, 2H), 3.93 (s, 2H). ^{13}C NMR (101 MHz, $CDCl_3$) δ 148.5, 138.3, 136.4, 130.0, 128.8, 128.3, 127.0, 118.4, 117.4, 114.8, 39.6. (*69*)

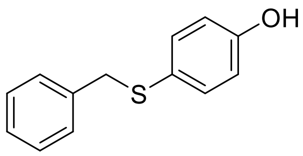

4-(benzylthio)phenol (267): The title compound was prepared according to the general procedure and purified by column chromatography on silica gel to afford a yellow solid in 81% yield (35.1 mg), mp 90-91 ºC; ^{1}H NMR (400 MHz, $CDCl_3$) δ 7.26 (dd, *J* = 19.5, 6.1 Hz, 7H), 6.74 (d, *J* = 7.5 Hz, 2H), 5.26 (s, 1H), 4.01 (s, 2H). ^{13}C NMR (101 MHz, $CDCl_3$) δ 155.2, 138.0, 134.3, 128.9, 128.3, 127.0, 126.1, 115.9, 41.2.

benzyl(4-(benzyloxy)phenyl)sulfane (268): The title compound was prepared according to the general procedure and purified by column chromatography on silica gel to afford a yellow solid in 69% yield (42.3 mg), mp 97-98 ºC; ^{1}H NMR (400 MHz, $CDCl_3$) δ 7.47 – 7.39 (m, 4H), 7.37 (d, *J* = 6.1 Hz, 1H), 7.28 (d, *J* = 6.9 Hz, 5H), 7.22 (d, *J* = 7.2 Hz, 2H), 6.89 (d, *J* = 8.0 Hz, 2H), 5.06 (s, 2H), 4.02 (s, 2H). ^{13}C NMR (101 MHz, $CDCl_3$) δ 158.3, 138.0, 136.7, 133.9, 128.9, 128.6, 128.3, 128.0, 127.4, 127.0, 126.5, 115.4, 70.1, 41.1. HRMS (EI) m/z: $[M]^+$ calcd for $C_{20}H_{18}OS$: 306.1078; found, 306.1079.

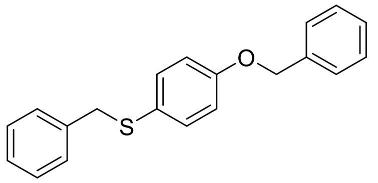

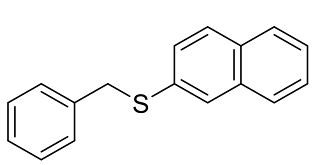

benzyl(naphthalen-2-yl)sulfane (269): The title compound was prepared according to the general procedure and purified by column chromatography on silica gel to afford a yellow solid in 98% yield (49.1 mg), mp 92-93 ºC; ^{1}H NMR (400 MHz, $CDCl_3$) δ 7.83 (d, *J* = 7.4 Hz, 1H), 7.80 – 7.68 (m, 3H), 7.49 (dt, *J* = 16.2, 7.7 Hz, 3H), 7.42 – 7.21 (m, 5H), 4.27 (s, 2H). ^{13}C NMR (101 MHz, $CDCl_3$) δ 137.31, 133.90, 133.67, 131.84, 128.81, 128.49, 128.27, 127.65, 127.61, 127.19, 127.11, 126.43, 125.69, 38.88. IR (neat, ν/cm^{-1}) 3050, 3029, 2961, 2918, 1585, 1491, 1453, 1200, 1133, 1065, 940, 844, 811, 740, 717, 691.

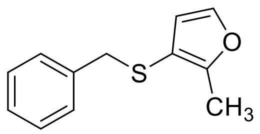

3-(benzylthio)-2-methylfuran (270): The title compound was prepared according to the general procedure and purified by column chromatography on silica gel to afford a yellow oil in 72% yield (29.4 mg); ^{1}H NMR (400 MHz, $CDCl_3$) δ 7.30 – 7.23 (m, 4H), 7.15 – 7.10 (m, 2H), 6.25 (d, *J* = 2.0 Hz, 1H), 3.77 (s, 2H), 1.99 (s, 3H). ^{13}C NMR (101 MHz, $CDCl_3$) δ 156.2, 140.4, 138.4, 128.9, 128.2, 126.9, 115.2, 109.2, 40.7, 11.2. (*70*)

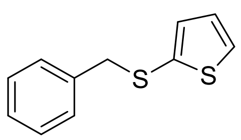

2-(benzylthio)thiophene (271): The title compound was prepared according to the general procedure and purified by column chromatography on silica gel to afford a yellow oil in 68% yield (28.1 mg); ^{1}H NMR (400 MHz, $CDCl_3$) δ 7.34 (dd, *J* = 5.1, 1.4 Hz, 1H), 7.30 – 7.26 (m, 3H), 7.22 – 7.15 (m, 2H), 6.99 – 6.90 (m, 2H), 3.98 (s, 2H). ^{13}C NMR (101 MHz, $CDCl_3$) δ 137.6, 134.3, 133.5, 129.7, 128.9, 128.3, 127.4,

127.2, 43.8. (*71*)

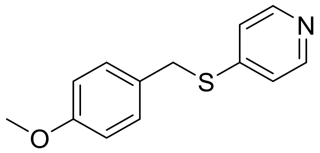

4-((4-methoxybenzyl)thio)pyridine (272): The title compound was prepared according to the general procedure and purified by column chromatography on silica gel to afford a yellow solid in 96% yield (44.5 mg), mp 94-95 ºC; ^{1}H NMR (400 MHz, $CDCl_3$) δ 8.36 (d, *J* = 3.7 Hz, 2H), 7.29 (d, *J* = 7.6 Hz, 2H), 7.09 (d, *J* = 4.3 Hz, 2H), 6.85 (d, *J* = 7.8 Hz, 2H), 4.14 (s, 2H), 3.76 (s, 3H). ^{13}C NMR (101 MHz, $CDCl_3$) δ 158.9, 149.0, 148.9, 129.7, 127.1, 120.6, 114.0, 55.1, 34.9. IR (neat, ν/cm^{-1}) 3069, 3036, 3015, 2963, 2916, 2841, 1606, 1573, 1510, 1406, 1244, 1173, 1098, 1025, 835, 813, 797, 755, 705. (*72*)

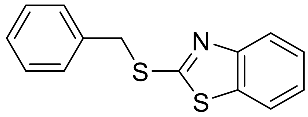

2-(benzylthio)benzo[*d*]thiazole (273): The title compound was prepared according to the general procedure and purified by column chromatography on silica gel to afford a yellow oil in 64% yield (33.0 mg); ^{1}H NMR (400 MHz, $CDCl_3$) δ 7.93 (d, *J* = 8.1 Hz, 1H), 7.75 (d, *J* = 7.9 Hz, 1H), 7.50 – 7.40 (m, 3H), 7.38 – 7.26 (m, 4H), 4.62 (s, 2H). ^{13}C NMR (101 MHz, $CDCl_3$) δ 166.3, 153.1, 136.1, 135.3, 129.1, 128.6, 127.7, 126.0, 124.2, 121.5, 120.9, 37.7. HRMS (EI) m/z: $[M]^+$ calcd for $C_{14}H_{11}NS_2$: 257.0333; found, 257.0334.

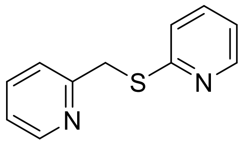

2-((pyridin-2-ylmethyl)thio)pyridine (274): The title compound was prepared according to the general procedure and purified by column chromatography on silica gel to afford a black oil in 98% yield (39.7 mg); ^{1}H NMR (400 MHz, $CDCl_3$) δ 8.51 (s, 1H), 8.30 (d, *J* = 3.8 Hz, 2H), 7.60 (t, *J* = 7.6 Hz, 1H), 7.39 (d, *J* = 7.7 Hz, 1H), 7.14 (s, 3H), 4.30 (s, 2H). ^{13}C NMR (101 MHz, $CDCl_3$) δ 156.2, 149.3, 149.1, 148.4, 136.9, 122.7, 122.4, 120.7, 37.2. (*73*)

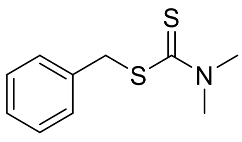

benzyl dimethylcarbamodithioate (275): The title compound was prepared according to the general procedure and purified by column chromatography on silica gel to afford a yellow solid in 95% yield (40.2 mg), mp 40-41 ºC; ^{1}H NMR (400 MHz, $CDCl_3$) δ 7.36 – 7.31 (m, 2H), 7.29 – 7.20 (m, 3H), 4.50 (s,

2H), 3.49 (s, 3H), 3.28 (s, 3H). ^{13}C NMR (101 MHz, $CDCl_3$) δ 196.6, 136.0, 129.1, 128.4, 127.3, 45.2, 42.4, 41.3. (*74*)

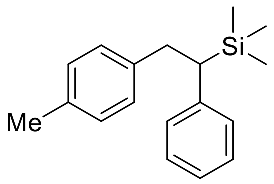

trimethyl(1-phenyl-2-(*p*-tolyl)ethyl)silane (276): The title compound was prepared according to the general procedure and purified by column chromatography on silica gel to afford a yellow oil in 92% yield (49.4 mg); ^{1}H NMR (400 MHz, $CDCl_3$) δ 7.21 (t, J = 7.6 Hz, 2H), 7.10 – 7.03 (m, 3H), 6.99 (s, 4H), 3.11 – 3.06 (m, 2H), 2.42 (dd, J = 8.8, 6.8 Hz, 1H), 2.26 (s, 3H), -0.00 (s, 9H). ^{13}C NMR (101 MHz, $CDCl_3$) δ 143.3, 139.2, 134.9, 128.7, 128.3, 128.0, 127.9, 124.3, 38.8, 35.3, 20.9, -2.8. HRMS (EI) m/z: $[M]^+$ calcd for $C_{18}H_{24}Si$: 268.1647; found, 268.1646.

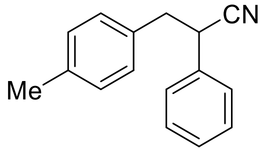

2-phenyl-3-(*p*-tolyl)propanenitrile (277): The title compound was prepared according to the general procedure and purified by column chromatography on silica gel to afford a yellow oil in 81% yield (35.9 mg); ^{1}H NMR (400 MHz, $CDCl_3$) δ 7.40 – 7.32 (m, 3H), 7.27 (dd, J = 9.4, 2.7 Hz, 2H), 7.11 (d, J = 7.8 Hz, 2H), 7.04 (d, J = 7.9 Hz, 2H), 4.05 – 3.82 (m, 1H), 3.13 (qd, J = 13.6, 7.4 Hz, 2H), 2.33 (s, 3H). ^{13}C NMR (101 MHz, $CDCl_3$) δ 137.0, 135.3, 133.2, 129.3, 129.0, 129.0, 128.1, 127.4, 120.4, 41.8, 39.9, 21.1. IR (neat, ν/cm^{-1}) 3067, 3024, 2957, 2921, 2855, 2239, 1599, 1512, 830, 792, 764, 738, 696. (*75*)

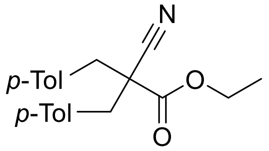

ethyl 2-cyano-2-(4-methylbenzyl)-3-(*p*-tolyl)propanoate (278): The title compound was prepared according to the general procedure and purified by column chromatography on silica gel to afford a yellow oil in 88% yield (56.6 mg); ^{1}H NMR (400 MHz, $CDCl_3$) δ 7.21 (d, J = 7.7 Hz, 4H), 7.14 (d, J = 7.7 Hz, 4H), 4.05 (q, J = 7.1 Hz, 2H), 3.30 (d, J = 13.5 Hz, 2H), 3.08 (d, J = 13.5 Hz, 2H), 2.35 (s, 6H), 1.04 (t, J = 7.1 Hz, 3H). ^{13}C NMR (101 MHz, $CDCl_3$) δ 168.2, 137.4, 131.0, 129.8, 129.1, 118.6, 62.4, 53.4, 42.8, 21.0, 13.6. HRMS (EI) m/z: $[M]^+$ calcd for $C_{21}H_{23}NO_2$: 321.1729; found, 321.1732.

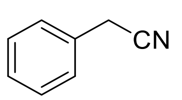

2-phenylacetonitrile (279): The title compound was prepared according to the general procedure and purified by column chromatography on silica gel to afford a yellow oil in 77% yield (18.1 mg); ^{1}H NMR (400 MHz, $CDCl_3$) δ 7.35 (dt, *J* = 12.9, 7.5 Hz, 1H), 3.71 (s, 1H). ^{13}C NMR (101 MHz, $CDCl_3$) δ 129.8, 128.9, 127.8, 127.7, 117.8, 23.3.

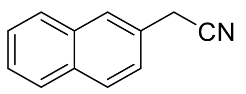

2-(naphthalen-2-yl)acetonitrile (280): The title compound was prepared according to the general procedure and purified by column chromatography on silica gel to afford a white solid in 76% yield (25.4 mg), mp 82-83 ºC; ^{1}H NMR (400 MHz, $CDCl_3$) δ 7.83 (dd, *J* = 15.1, 6.2 Hz, 4H), 7.58 – 7.47 (m, 2H), 7.37 (d, *J* = 8.4 Hz, 1H), 3.88 (s, 2H). ^{13}C NMR (101 MHz, $CDCl_3$) δ 133.2, 132.6, 128.9, 127.7, 127.6, 127.1, 126.7, 126.7, 126.4, 125.4, 117.8, 23.7.

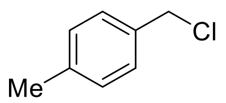

1-(chloromethyl)-4-methylbenzene (281): The title compound was prepared according to the general procedure and purified by column chromatography on silica gel to afford a yellow oil in 69% yield (19.4 mg); ^{1}H NMR (400 MHz, $CDCl_3$) δ 7.33 (t, *J* = 5.6 Hz, 2H), 7.23 (d, *J* = 4.9 Hz, 2H), 4.61 (s, 2H), 2.41 (s, 3H). ^{13}C NMR (101 MHz, $CDCl_3$) δ 138.2, 134.5, 129.4, 128.5, 46.2, 21.1.

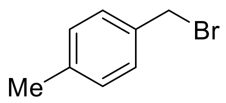

1-(bromomethyl)-4-methylbenzene (282): The title compound was prepared according to the general procedure and purified by column chromatography on silica gel to afford a yellow solid in 57% yield (21.1 mg), mp 35-36 ºC; ^{1}H NMR (400 MHz, $CDCl_3$) δ 7.31 (d, *J* = 8.0 Hz, 2H), 7.18 (d, *J* = 8.0 Hz, 2H), 4.51 (s, 2H), 2.38 (s, 3H). ^{13}C NMR (101 MHz, $CDCl_3$) δ 138.3, 134.8, 129.4, 128.9, 33.7, 21.2.

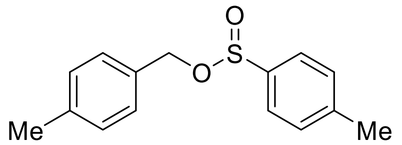

4-methylbenzyl 4-methylbenzenesulfinate (283): The title compound was prepared according to the general procedure and purified by column chromatography on silica gel to afford a yellow oil in 85% yield (44.3 mg); ^{1}H NMR (400 MHz, $CDCl_3$) δ 7.51 (d, *J* = 7.8 Hz, 2H), 7.24 (t, *J* = 7.1 Hz, 2H), 7.06 (d, *J* = 7.6 Hz, 2H), 6.96 (d, *J* = 7.4 Hz, 2H), 4.24 (s, 2H), 2.41 (s, 3H), 2.31 (s, 3H). ^{13}C NMR (101 MHz, $CDCl_3$) δ 144.5, 138.5, 135.1, 130.6, 129.4, 129.2, 128.6, 125.1,

62.6, 21.6, 21.2. (*76*)

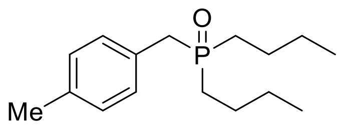

dibutyl(4-methylbenzyl)phosphine oxide (244): The title compound was prepared according to the general procedure and purified by column chromatography on silica gel to afford a yellow oil in 87% yield (46.3 mg); ^{1}H NMR (400 MHz, $CDCl_3$) δ 7.12 (s, 4H), 3.10 (d, *J* = 14.3 Hz, 2H), 2.32 (s, 3H), 1.70 – 1.48 (m, 8H), 1.45 – 1.33 (m, 4H), 0.91 (t, *J* = 7.3 Hz, 6H). ^{13}C NMR (101 MHz, $CDCl_3$) δ 136.4, 129.4, 129.2 (d, *J* = 4.2 Hz), 129.1 (d, *J* = 6.4 Hz), 35.7 (d, *J* = 59.1 Hz), 26.9 (d, *J* = 66.3 Hz), 24.1 (d, *J* = 14.1 Hz), 23.6 (d, *J* = 3.1 Hz), 20.9, 13.5. ^{31}P NMR (162 MHz, $CDCl_3$) δ 47.58. HRMS (EI) m/z: $[M]^+$ calcd for $C_{16}H_{27}OP$: 266.1800; found, 266.1803.

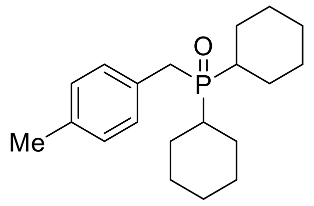

dicyclohexyl(4-methylbenzyl)phosphine oxide (245): The title compound was prepared according to the general procedure and purified by column chromatography on silica gel to afford a white solid (mp: 134-135 ºC) in 90% yield (57.3 mg); ^{1}H NMR (400 MHz, $CDCl_3$) δ 7.17 (d, *J* = 7.5 Hz, 2H), 7.08 (d, *J* = 7.7 Hz, 2H), 3.01 (d, *J* = 12.7 Hz, 2H), 2.29 (s, 3H), 1.80 (dd, *J* = 45.9, 14.2 Hz, 9H), 1.68 (d, *J* = 12.2 Hz, 3H), 1.44 – 1.12 (m, 10H). ^{13}C NMR (101 MHz, $CDCl_3$) δ 136.1 (d, *J* = 2.4 Hz), 129.6 (d, *J* = 4.8 Hz), 129.5, 129.2 (d, *J* = 1.7 Hz), 36.1 (d, *J* = 63.6 Hz), 31.5 (d, *J* = 56.1 Hz), 26.6 (d, *J* = 12.1 Hz), 25.9, 25.7 (d, *J* = 8.9 Hz), 21.0. ^{31}P NMR (162 MHz, $CDCl_3$) δ 48.57. IR (neat, ν/cm^{-1}) 3091, 3051, 3029, 2924, 2848, 1515, 1446, 1211, 1166, 1136, 1004, 895, 853, 832, 816, 768, 719. HRMS (EI) m/z: $[M]^+$ calcd for $C_{20}H_{31}ON$: 318.2113; found, 318.2114.

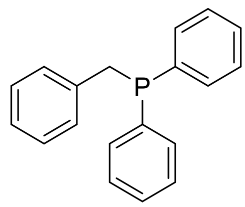

benzyldiphenylphosphane (284): The title compound was prepared according to the general procedure and purified by column chromatography on silica gel to afford a yellow solid in 90% yield (49.7 mg); ^{1}H NMR (400 MHz, $CDCl_3$) δ 7.43 (s, 4H), 7.35 (s, 6H), 7.24 – 7.14 (m, 3H), 7.09 (d, *J* = 6.9 Hz, 2H), 3.45 (s, 2H). ^{13}C NMR (101 MHz, $CDCl_3$) δ 138.2 (d, *J* = 15.1 Hz), 137.4 (d, *J* = 8.1 Hz), 132.9 (d, *J* = 18.5 Hz), 129.3 (d, *J* = 6.7 Hz), 128.6, 128.3 (d, *J* = 6.5 Hz), 128.2 (d, *J* = 1.3 Hz), 125.8 (d, *J* = 2.7 Hz), 36.0 (d, *J* = 15.6 Hz). ^{31}P NMR (162 MHz, $CDCl_3$) δ -9.76. (*77*)

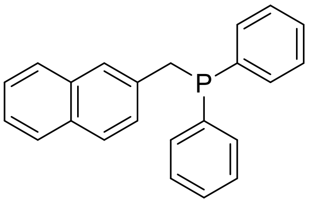

(naphthalen-2-ylmethyl)diphenylphosphane (285): The title compound was prepared according to the general procedure and purified by column chromatography on silica gel to afford a yellow solid in 94% yield (61.3 mg); ^{1}H NMR (400 MHz, $CDCl_3$) δ 7.83 (s, 1H), 7.76 (d, *J* = 8.2 Hz, 2H), 7.50 (s, 7H), 7.39 (s, 5H), 7.34 – 7.26 (m, 2H), 3.65 (s, 2H). ^{13}C NMR (101 MHz, $CDCl_3$) δ 138.1 (d, *J* = 15.1 Hz), 134.9 (d, *J* = 7.9 Hz), 133.9 (d, *J* = 16.8 Hz), 133.4, 132.9 (d, *J* = 18.5 Hz), 131.9, 128.7, 128.3 (d, *J* = 6.5 Hz), 128.0 (d, *J* = 6.3 Hz), 127.7, 127.6, 127.5, 127.4, 125.5 (d, *J* = 63.4 Hz), 36.3 (d, *J* = 15.7 Hz). ^{31}P NMR (162 MHz, $CDCl_3$) δ -10.20. (*78*)

5. Copies of ^{1}H, ^{13}C, ^{19}F, and ^{31}P NMR Spectra of the Products

^{1}H NMR Spectrum of ***O***

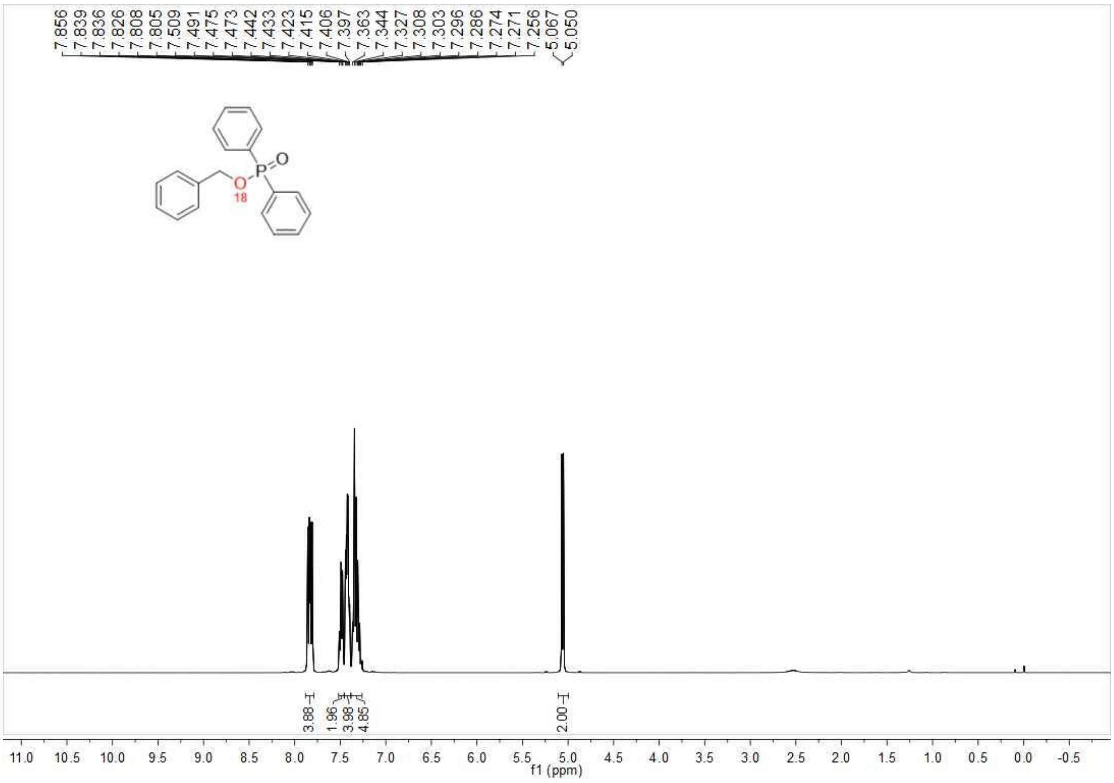

^{13}C NMR Spectrum of ***O*^{18}-benzyl diphenylphosphinate**

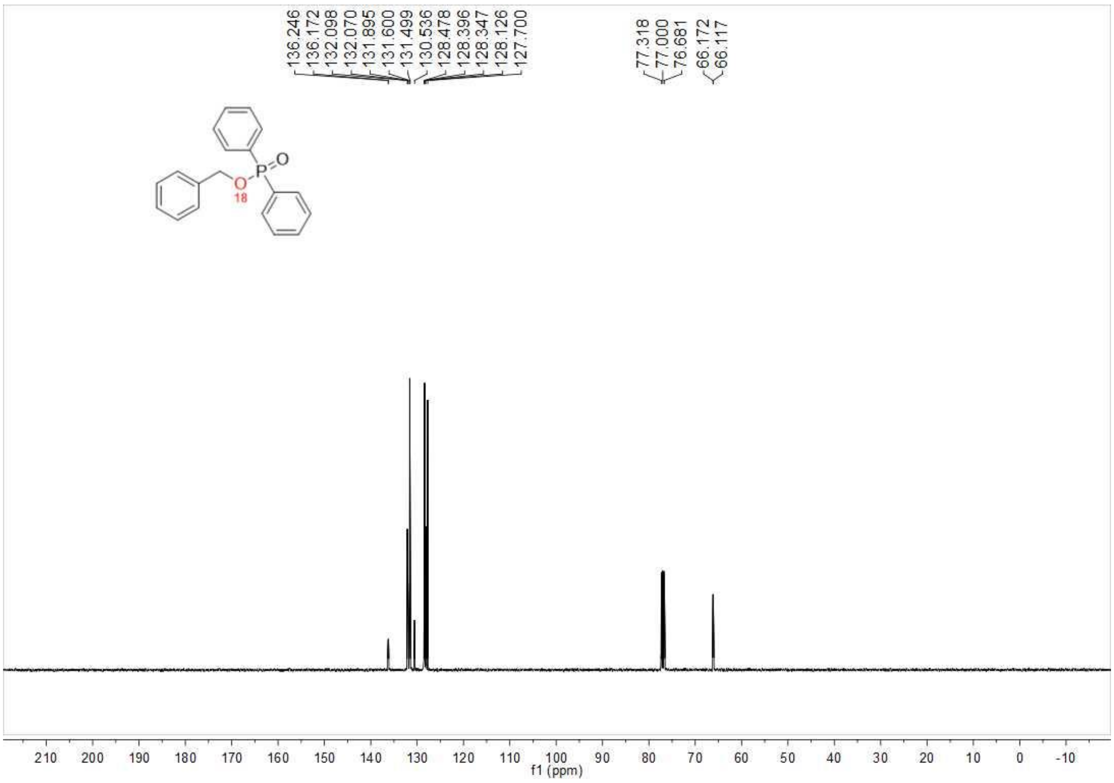

^{31}P NMR Spectrum of ***O*^{18}-benzyl diphenylphosphinate**

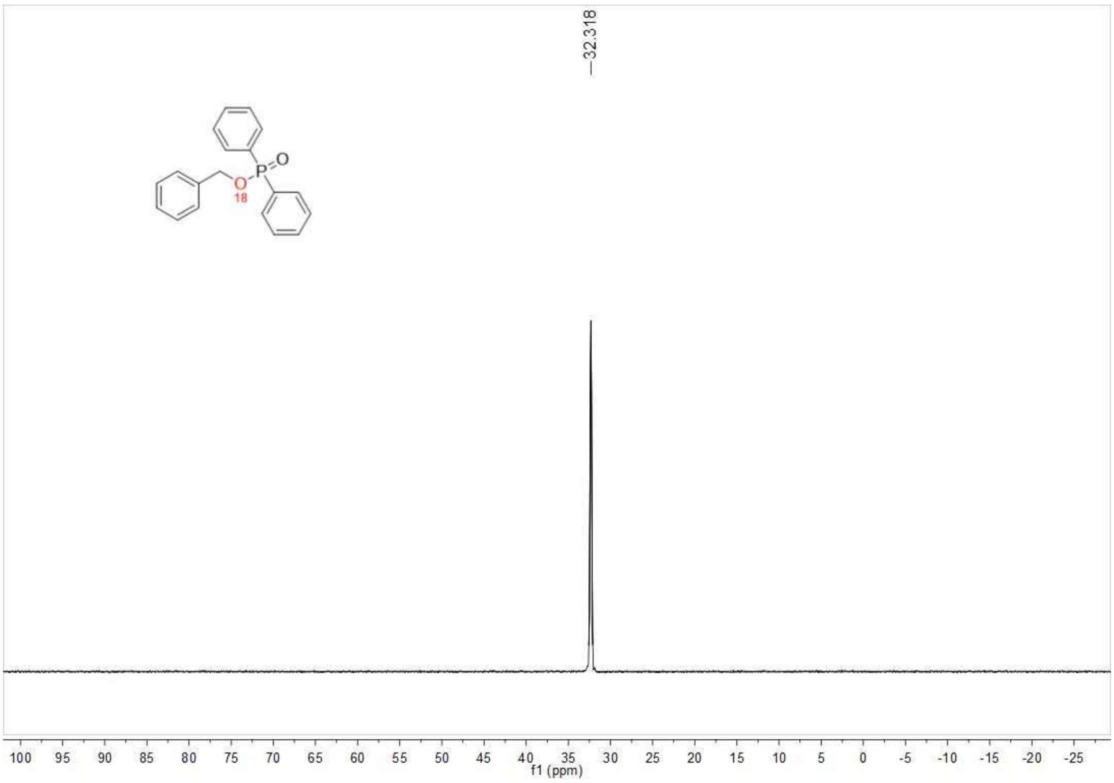

^{1}H NMR Spectrum of (*R*)-**2B**

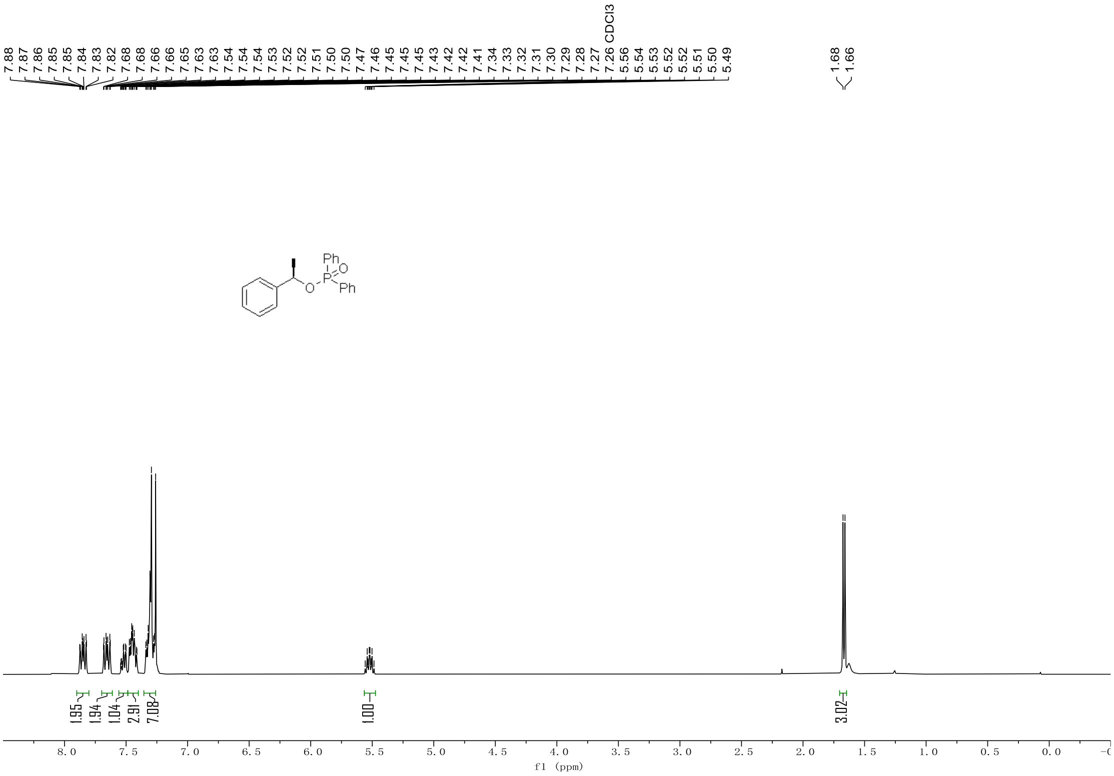

^{1}H NMR Spectrum of **1**

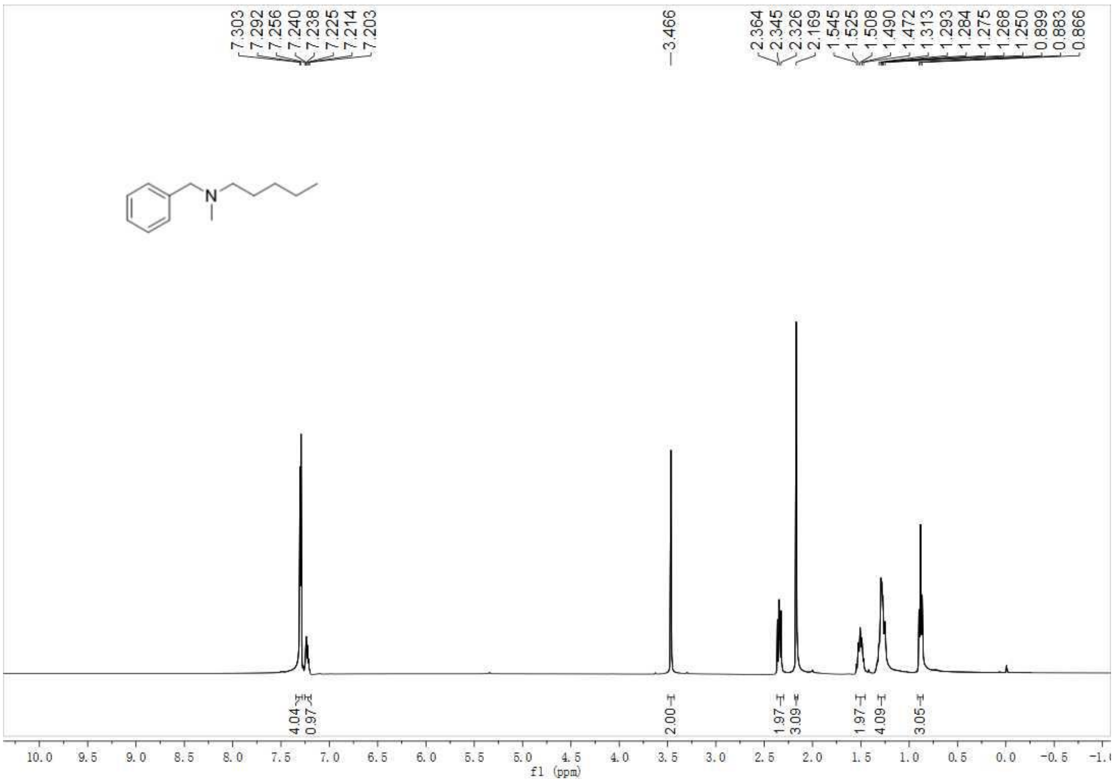

^{13}C NMR Spectrum of **1**

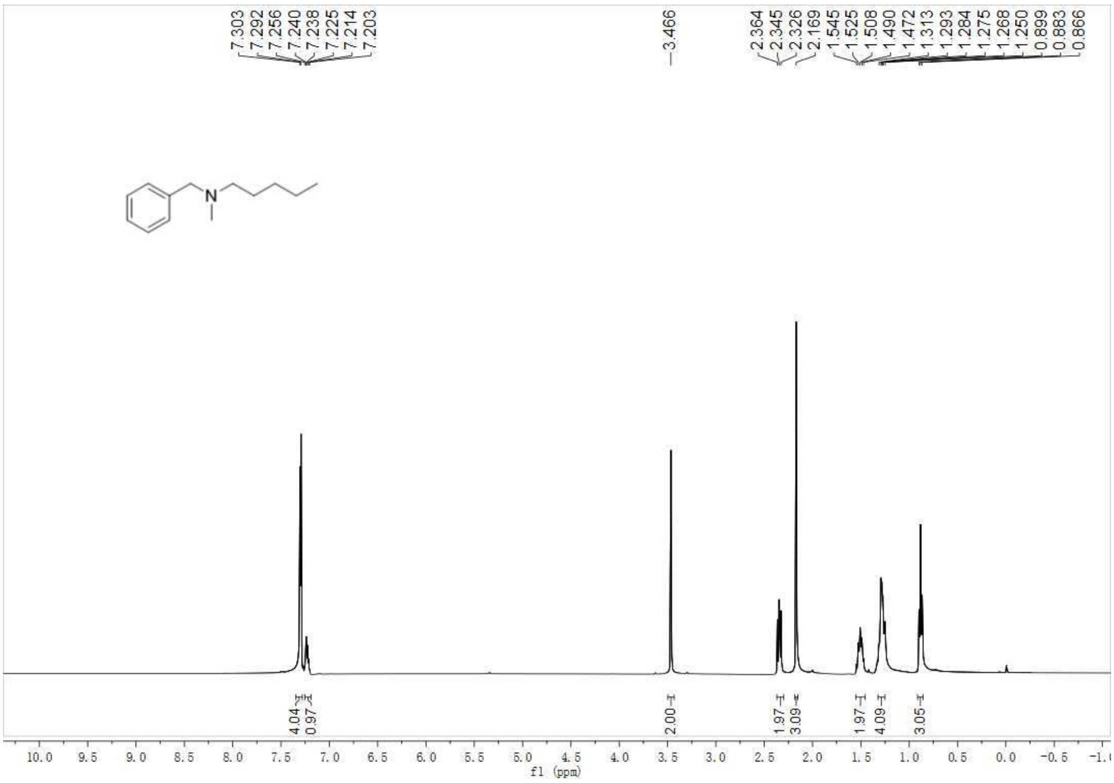

^{1}H NMR Spectrum of **2**

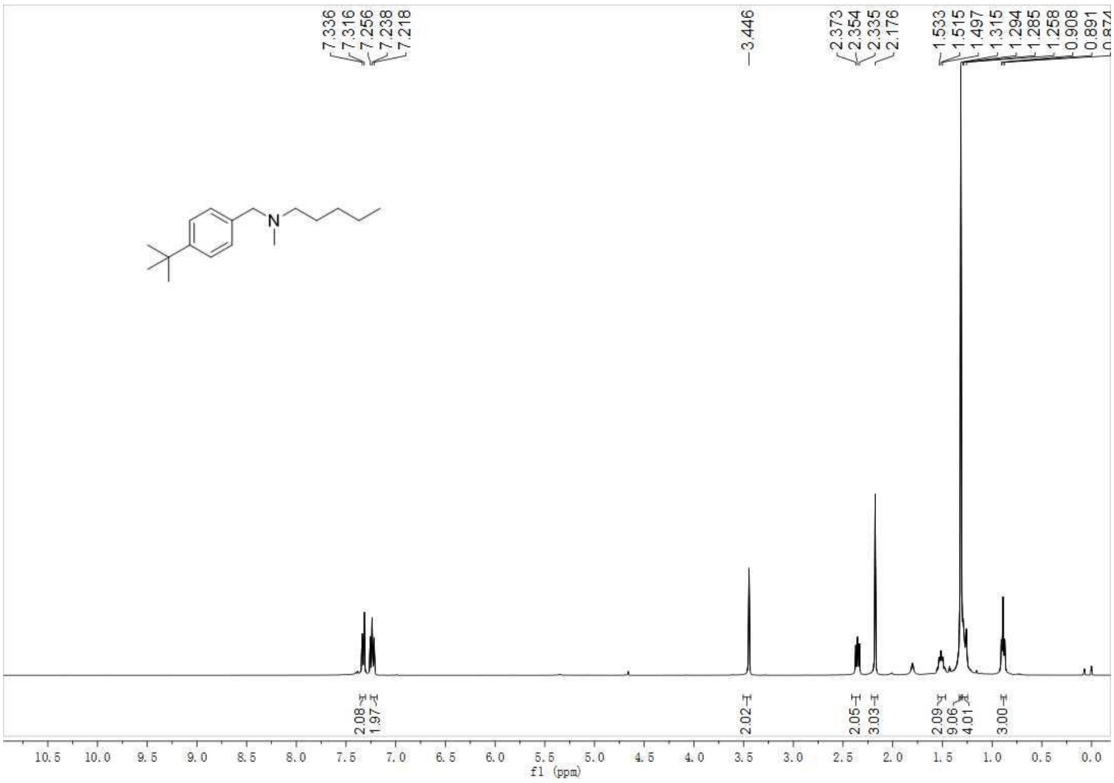

^{13}C NMR Spectrum of **2**

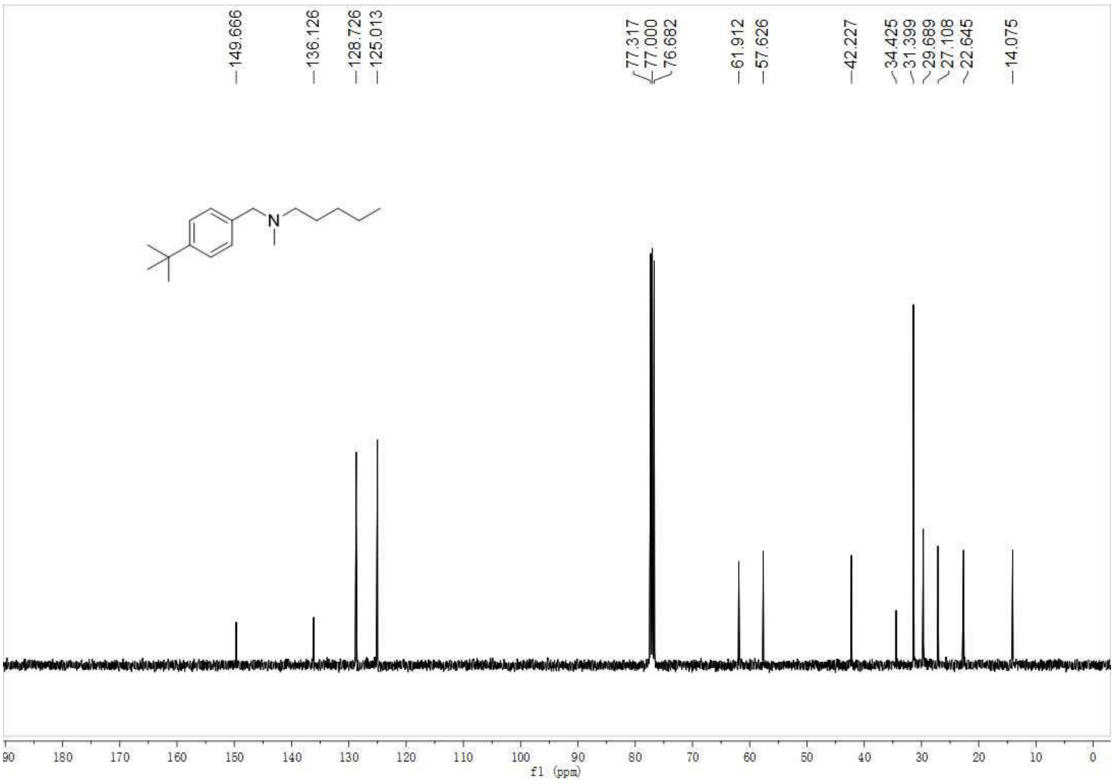

^{1}H NMR Spectrum of **3**

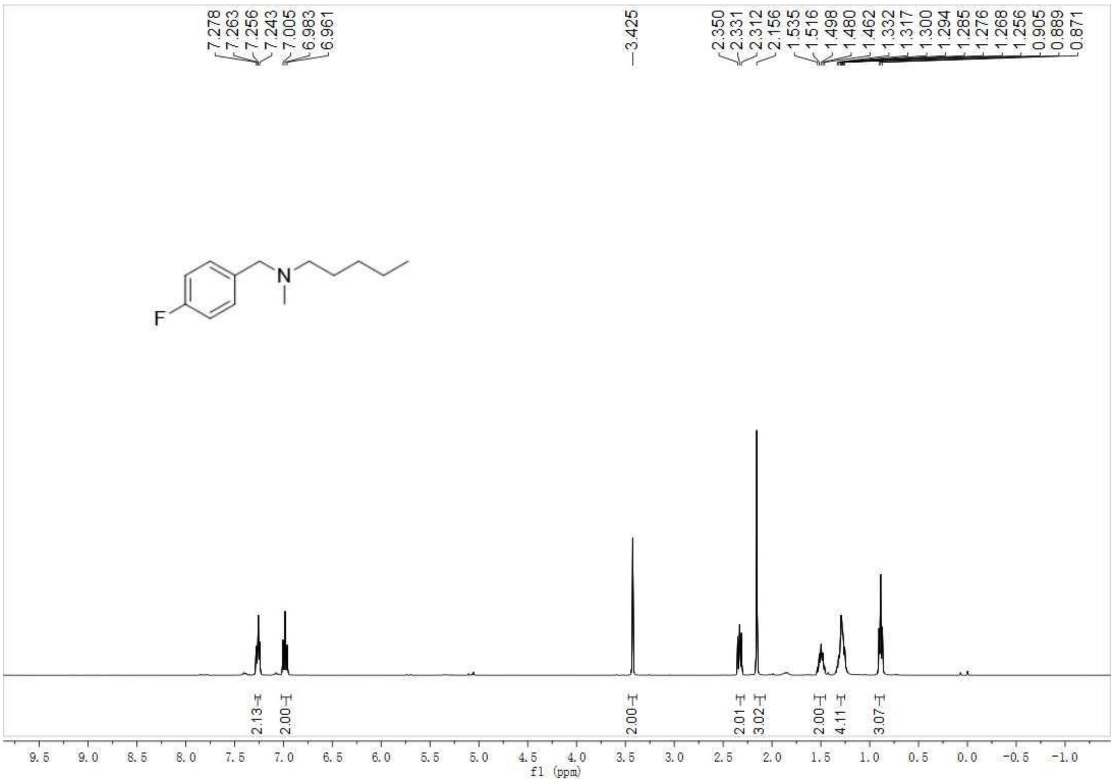

^{13}C NMR Spectrum of **3**

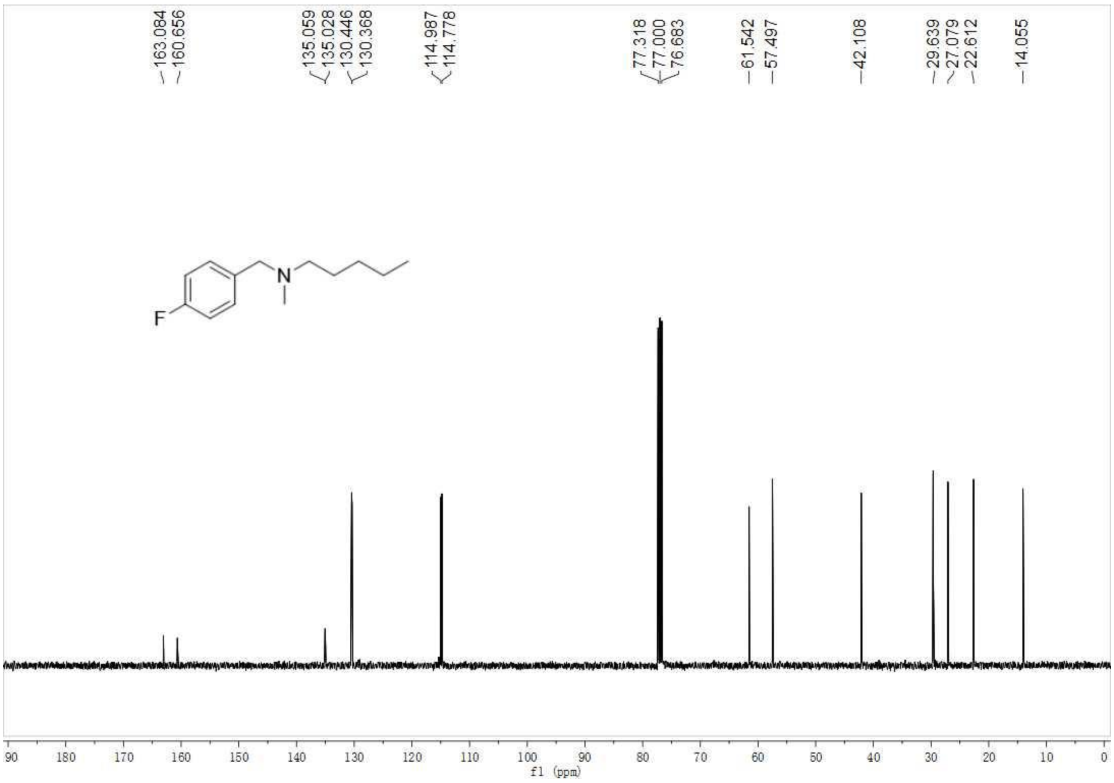

^{19}F NMR Spectrum of **3**

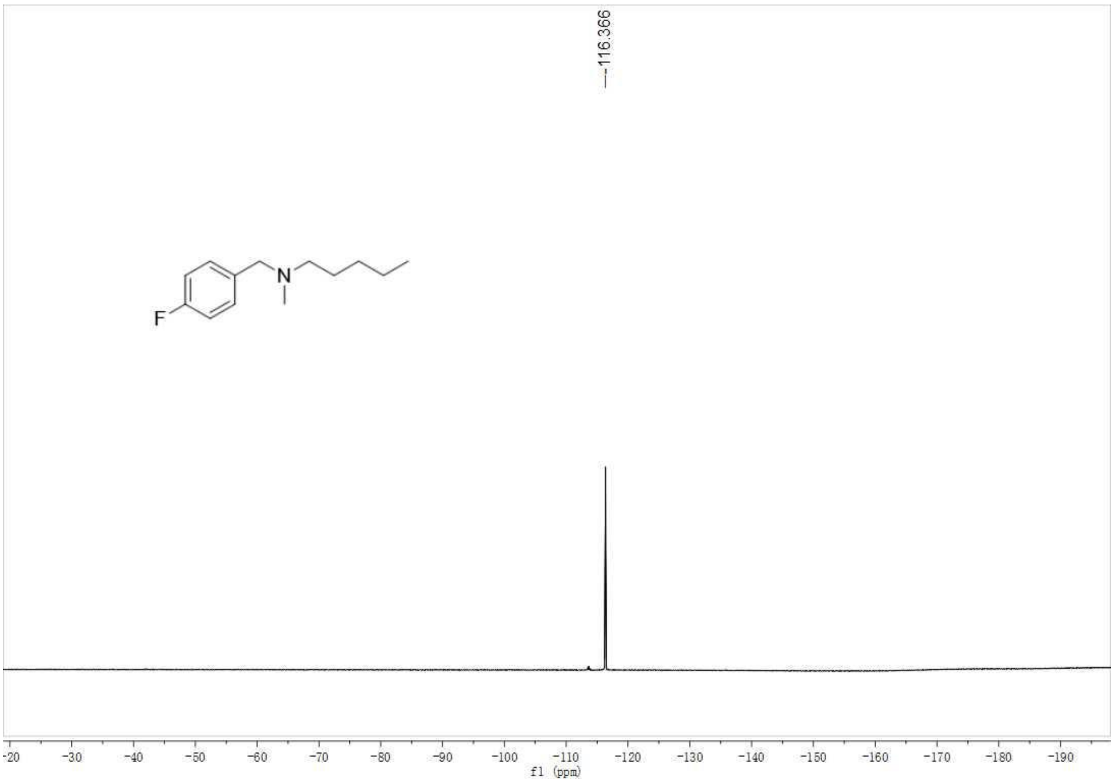

^{1}H NMR Spectrum of **4**

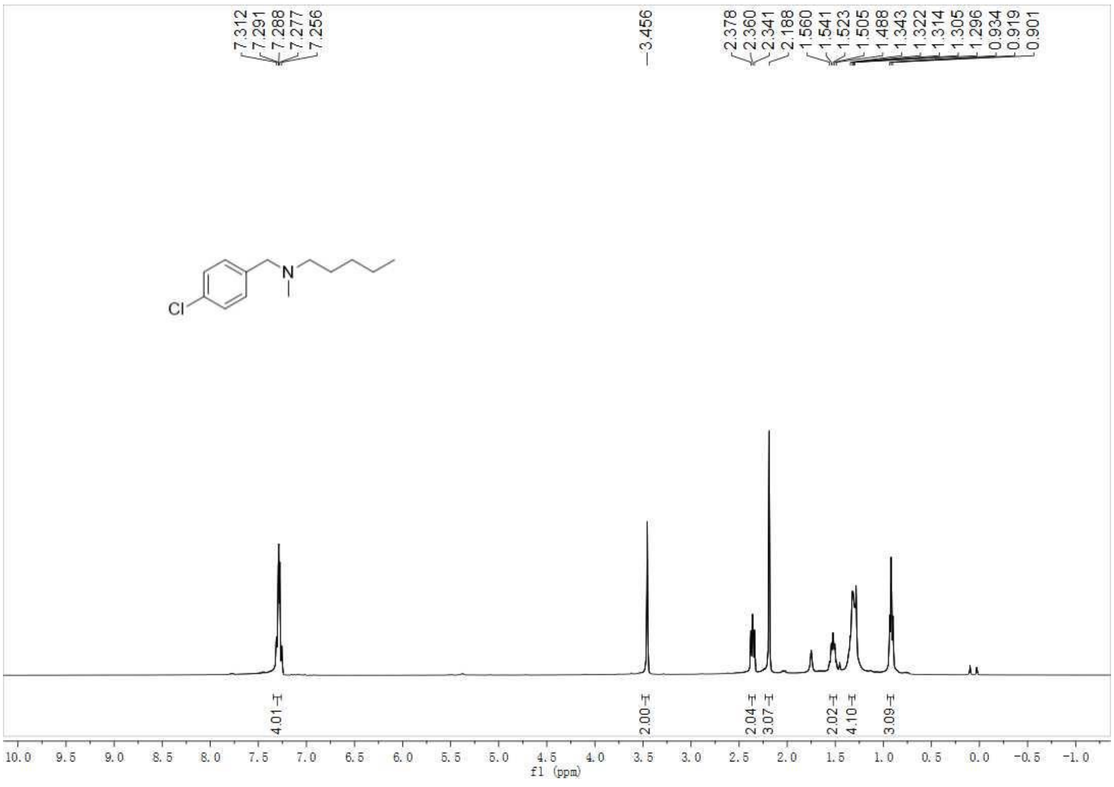

^{13}C NMR Spectrum of **4**

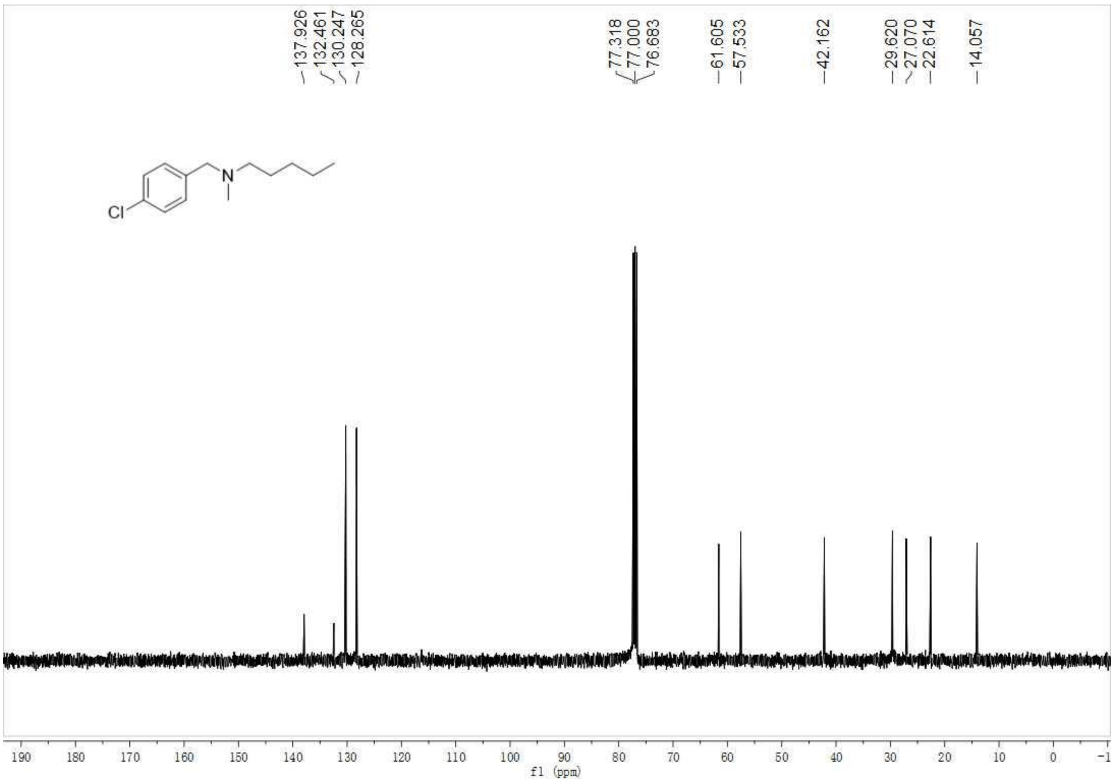

^{1}H NMR Spectrum of **5**

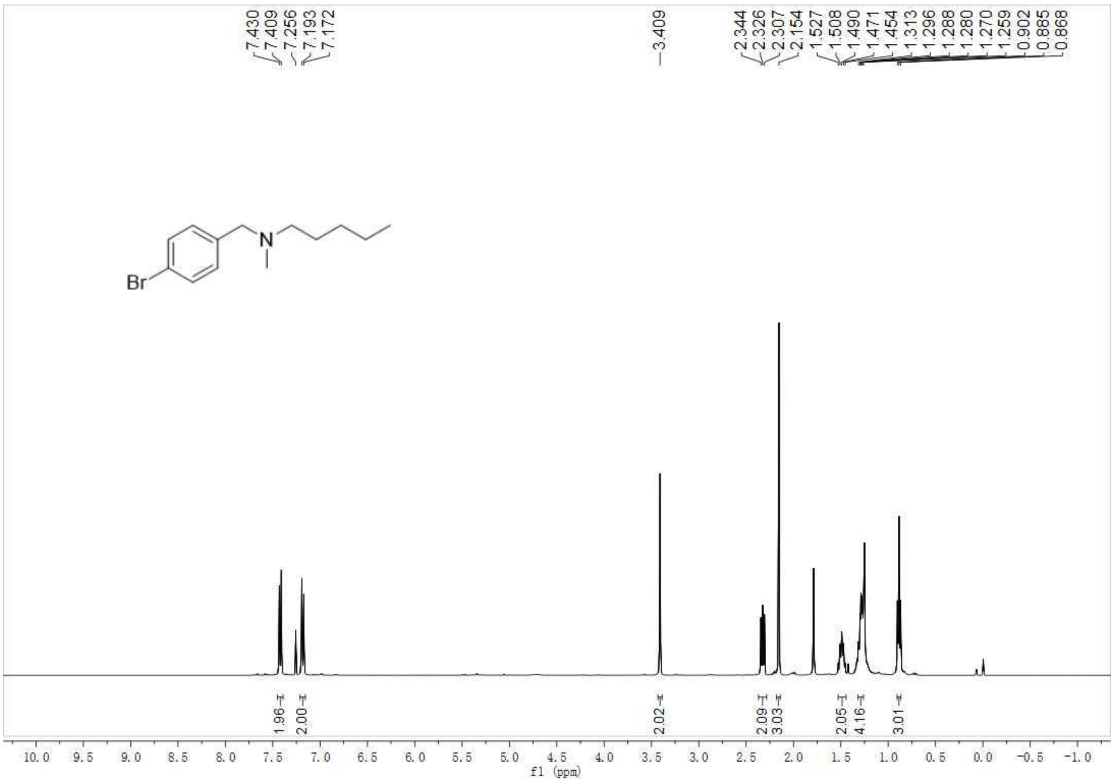

^{13}C NMR Spectrum of **5**

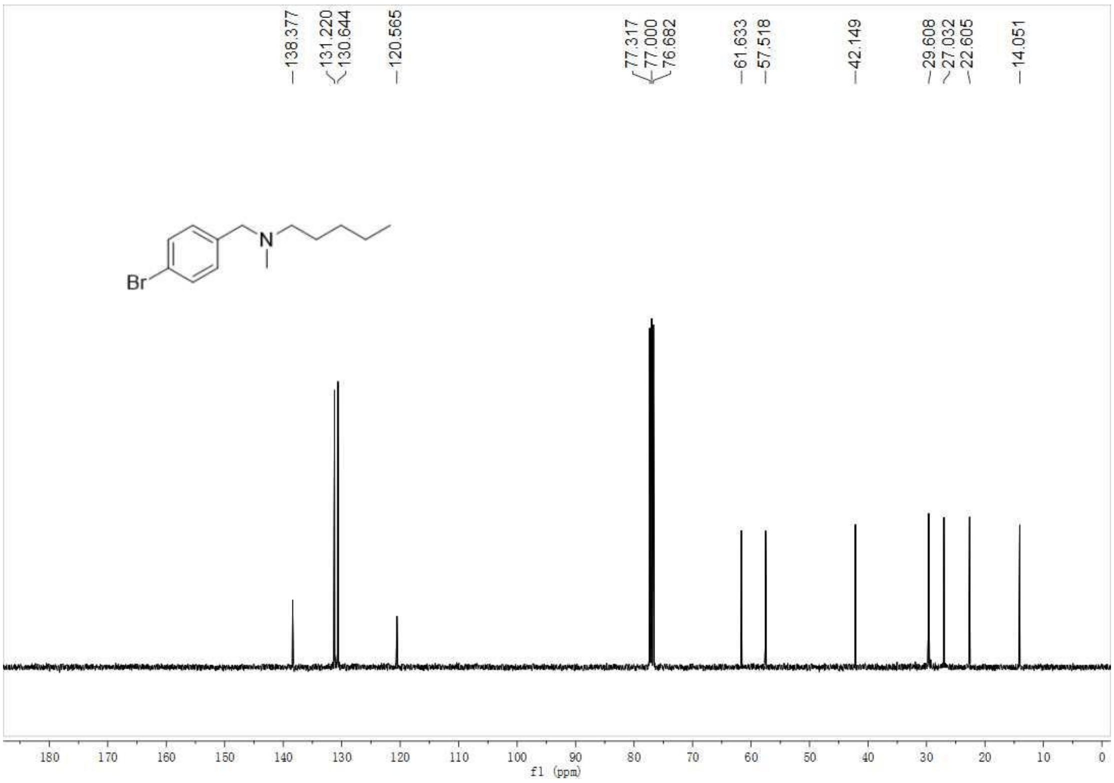

^{1}H NMR Spectrum of **6**

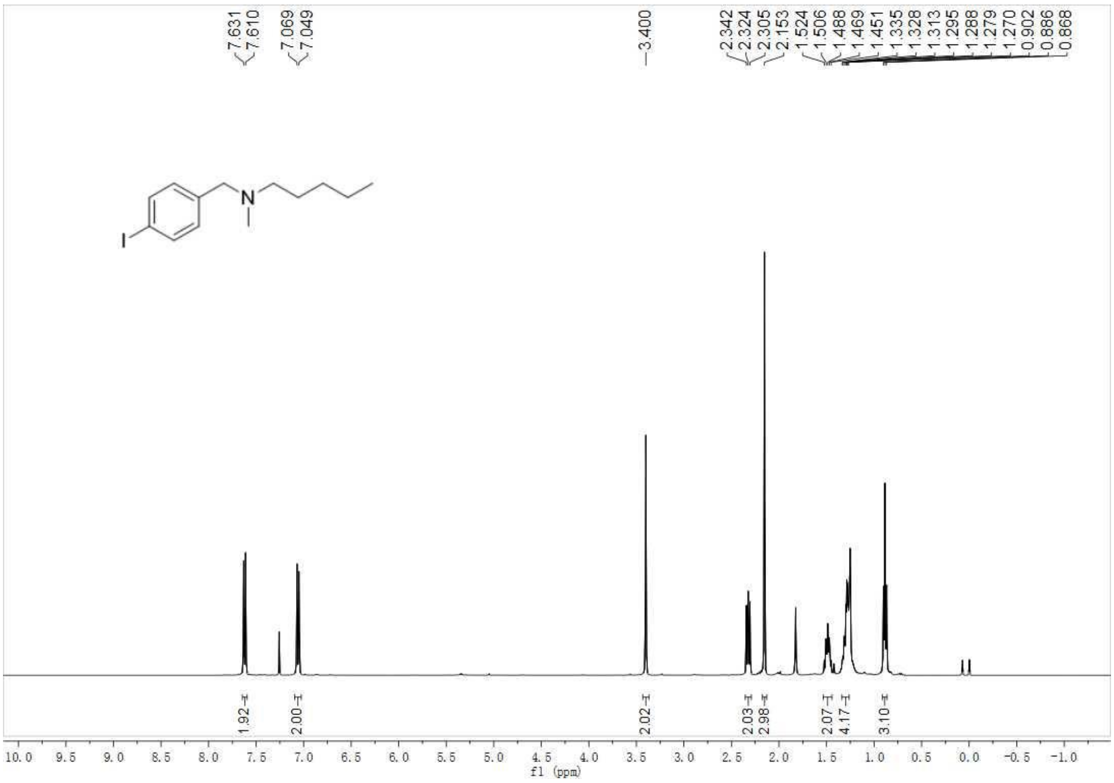

^{13}C NMR Spectrum of **6**

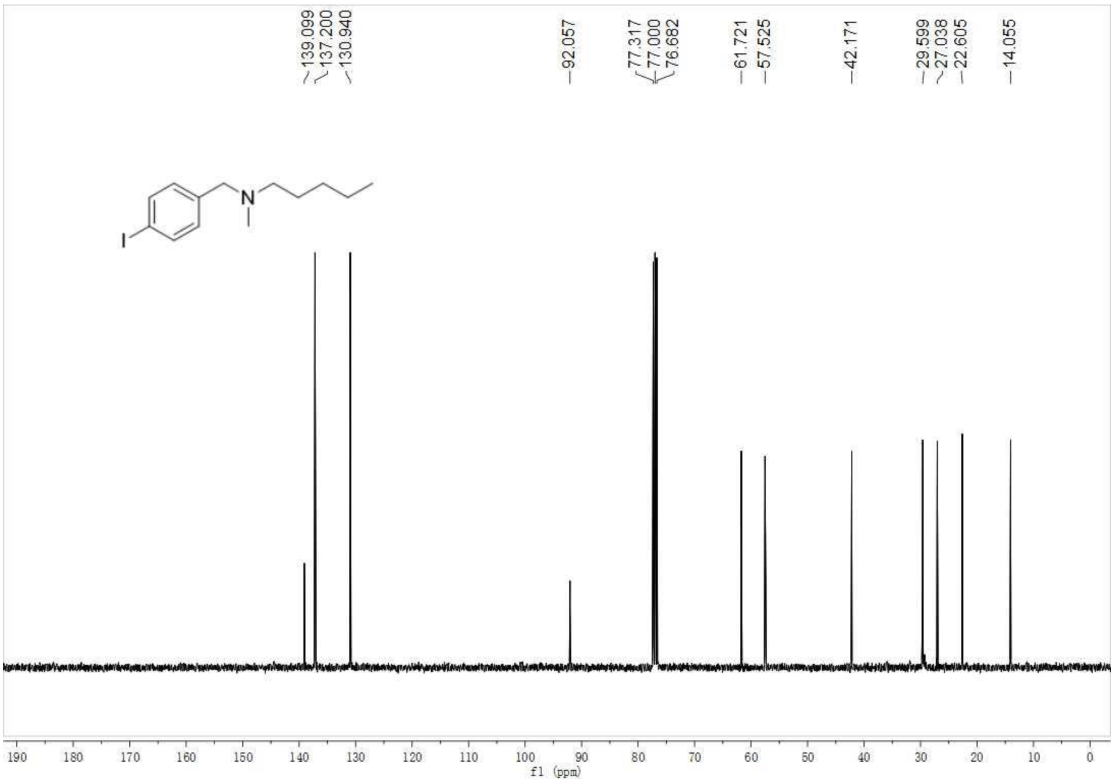

^{1}H NMR Spectrum of **7**

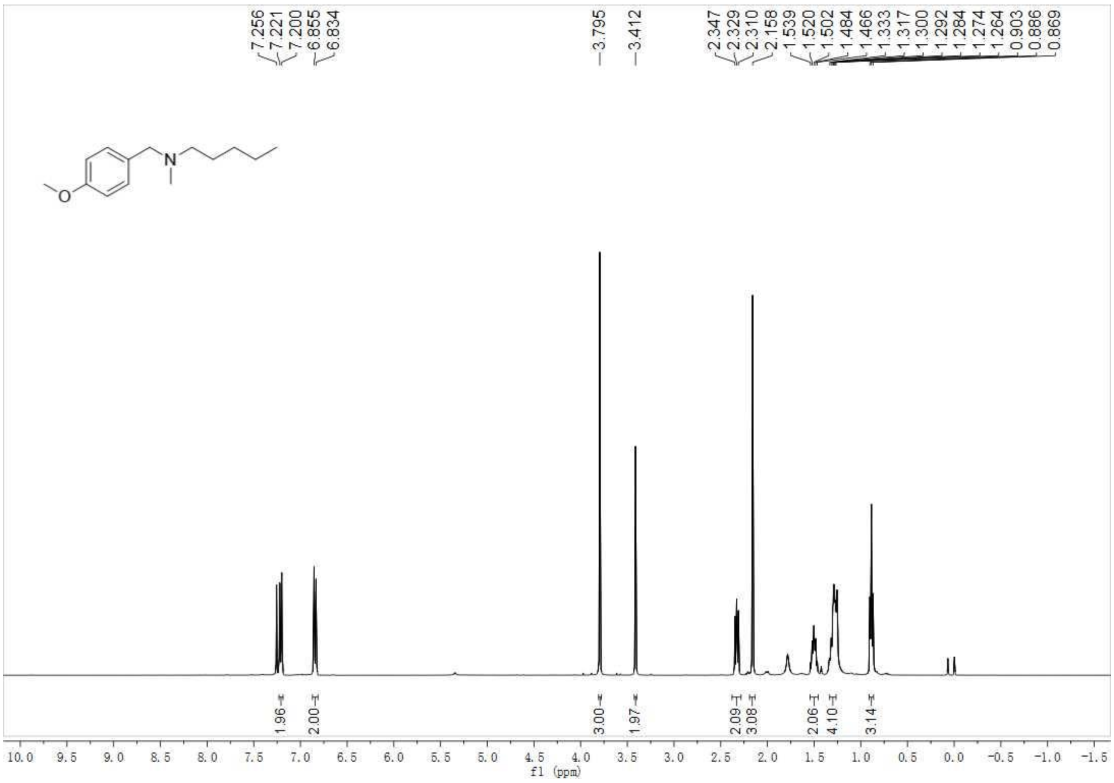

^{13}C NMR Spectrum of **7**

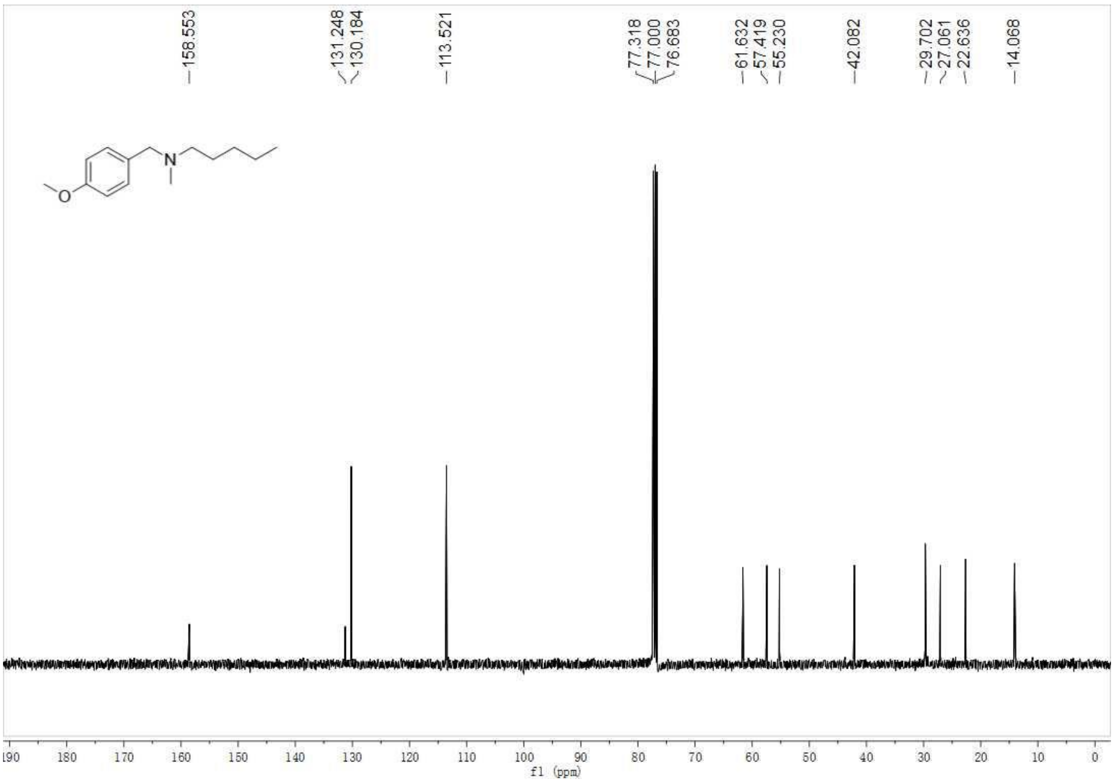

^{1}H NMR Spectrum of **8**

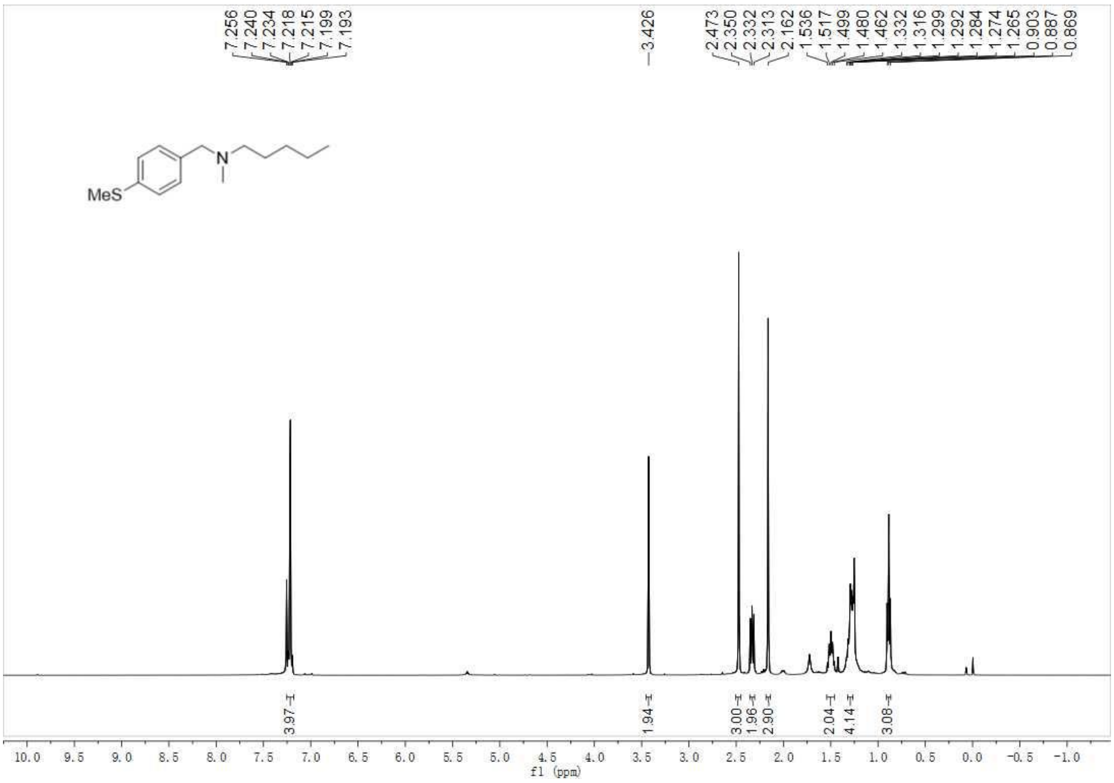

^{13}C NMR Spectrum of **8**

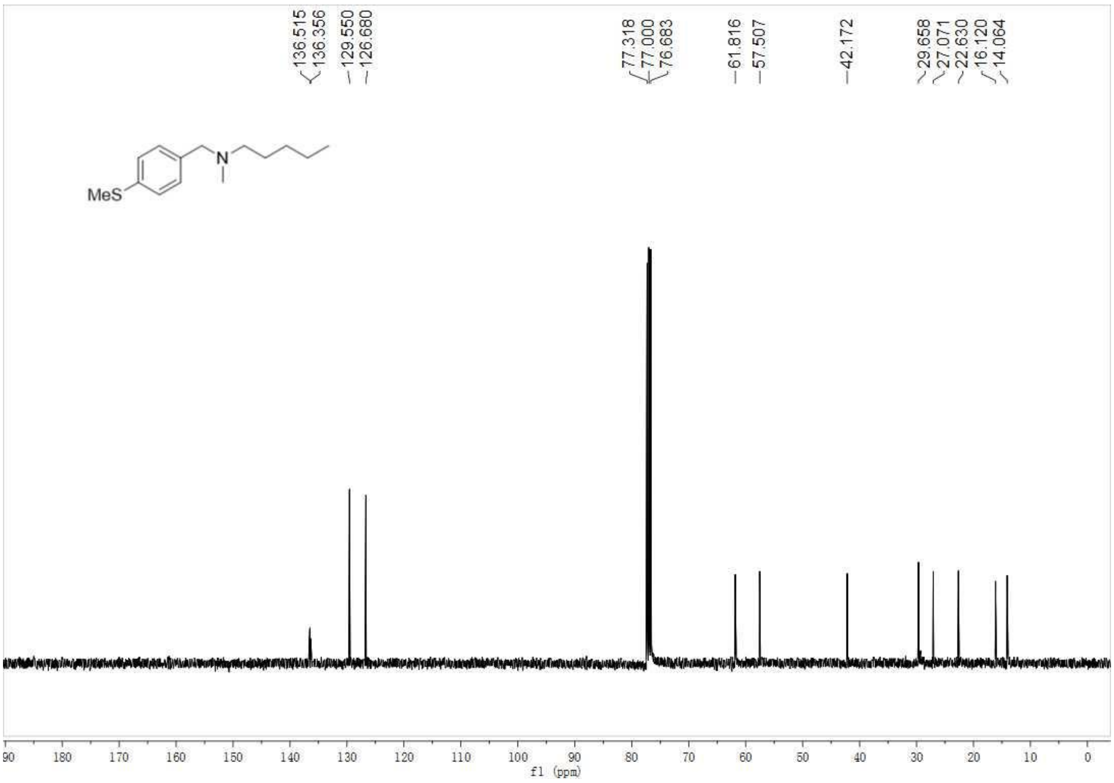

^{1}H NMR Spectrum of **9**

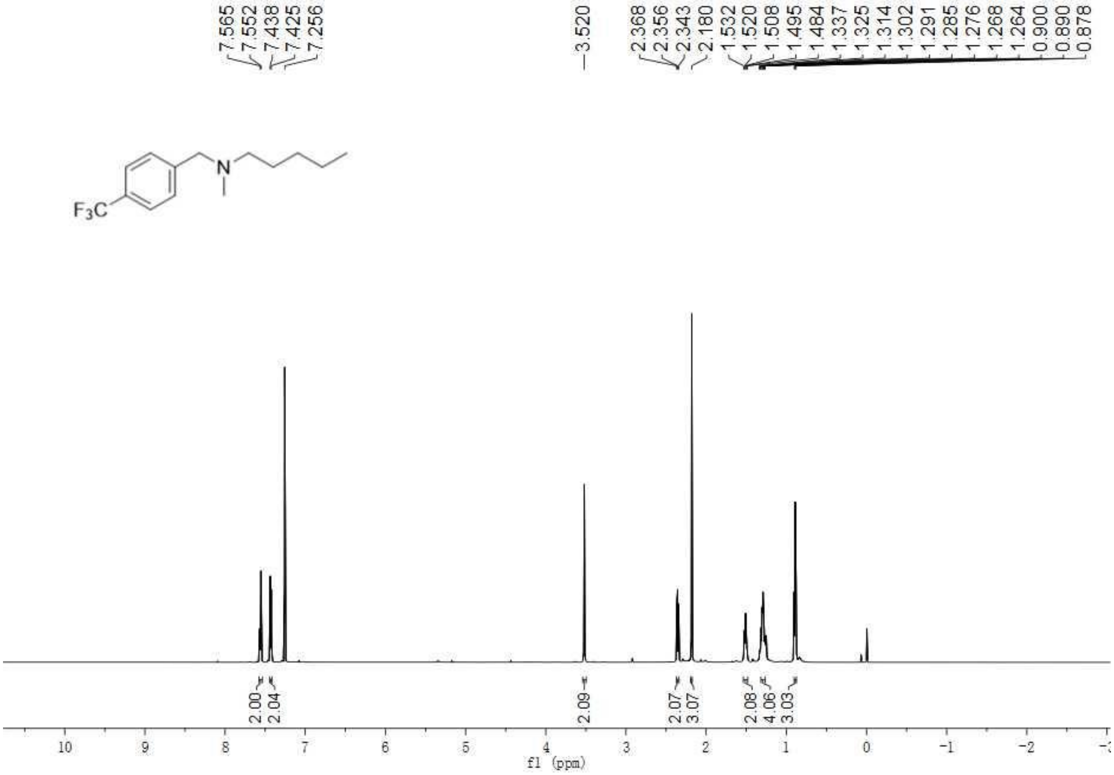

^{13}C NMR Spectrum of **9**

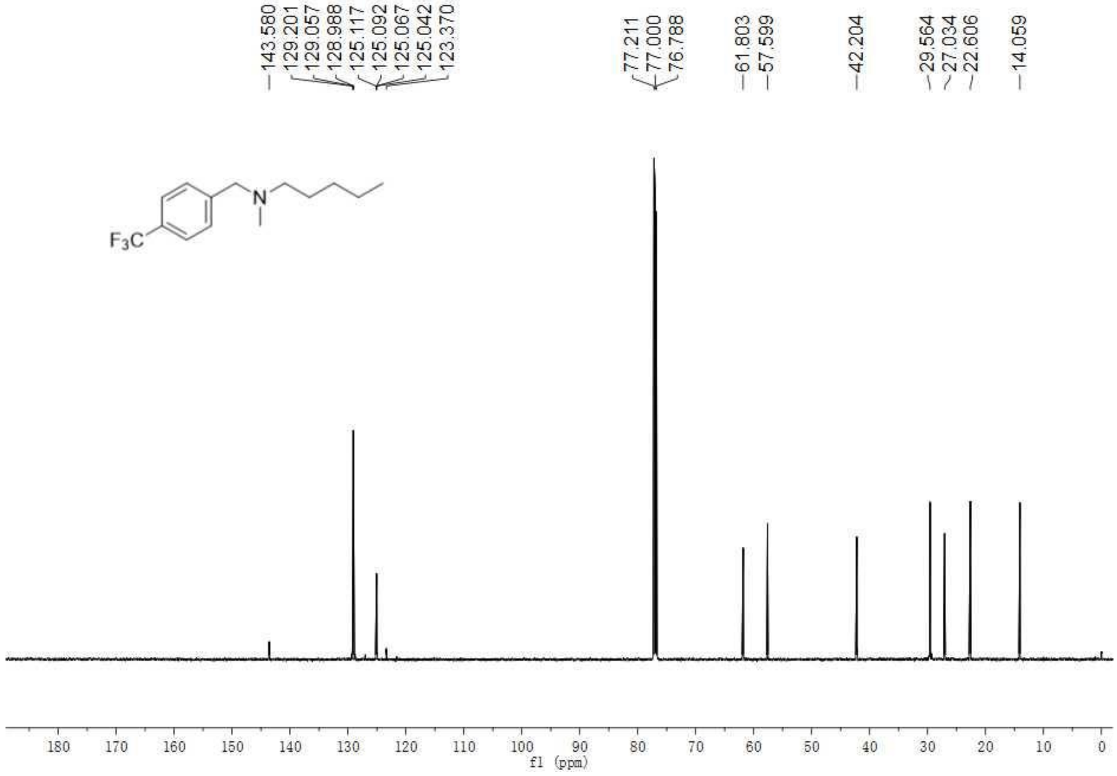

^{19}F NMR Spectrum of **9**

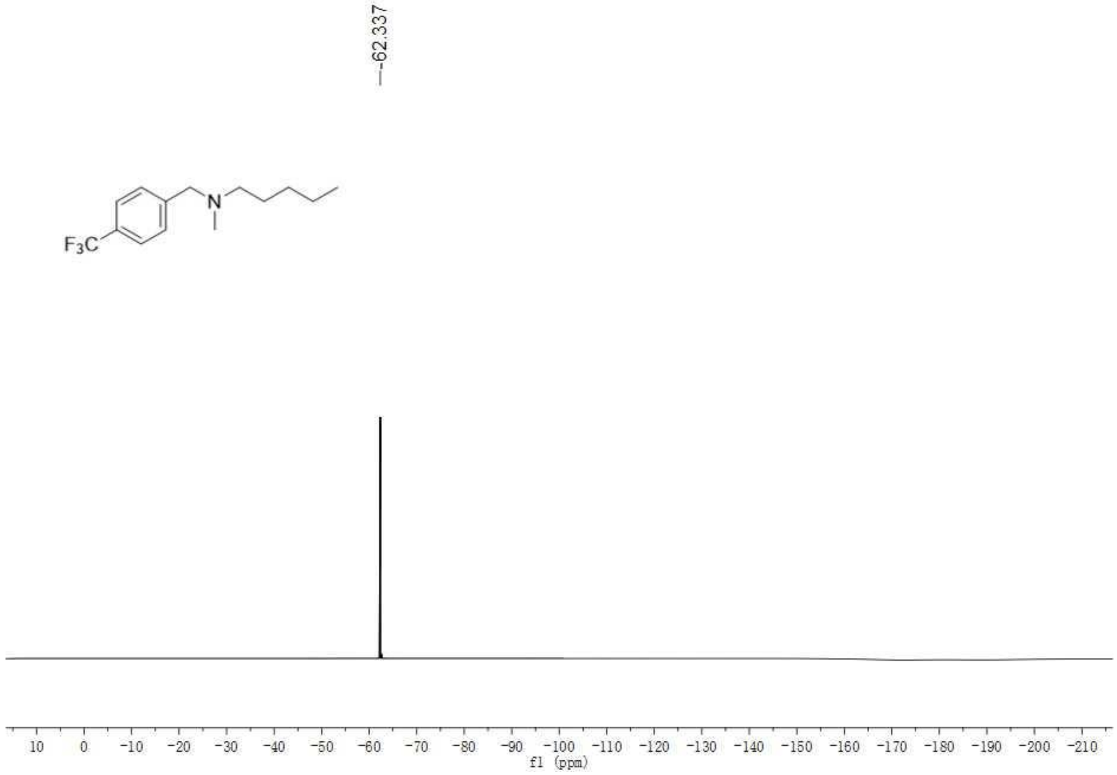

^{1}H NMR Spectrum of **10**

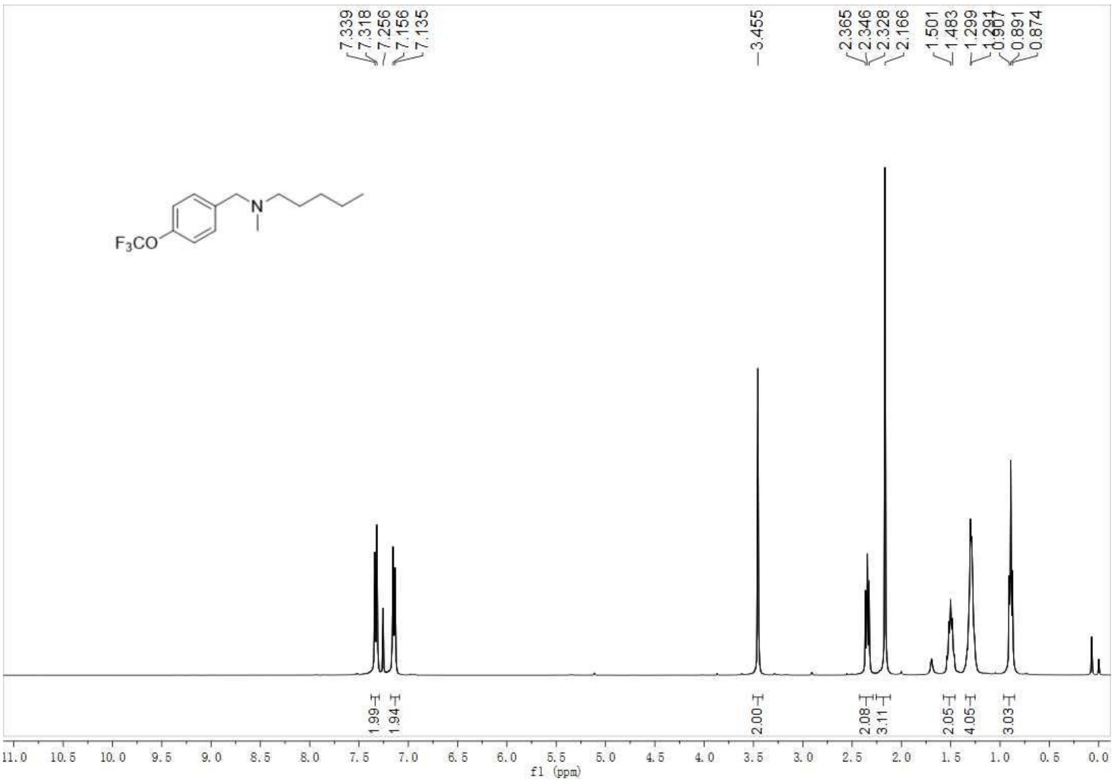

^{13}C NMR Spectrum of **10**

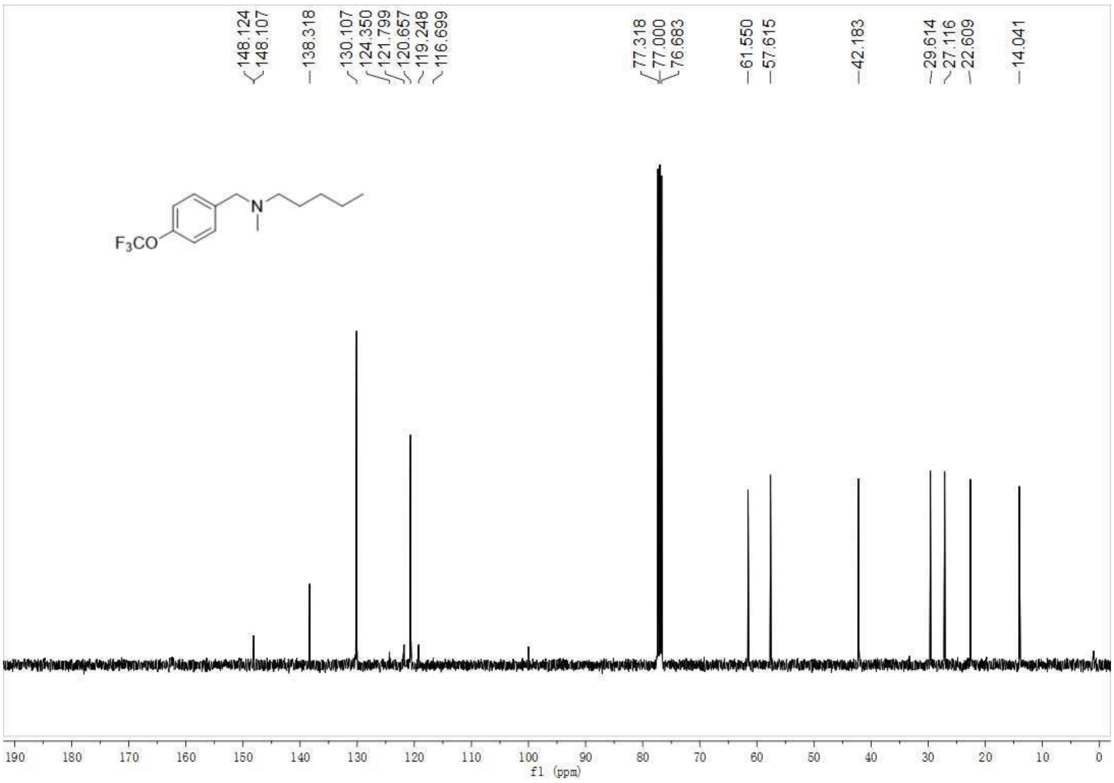

^{19}F NMR Spectrum of **10**

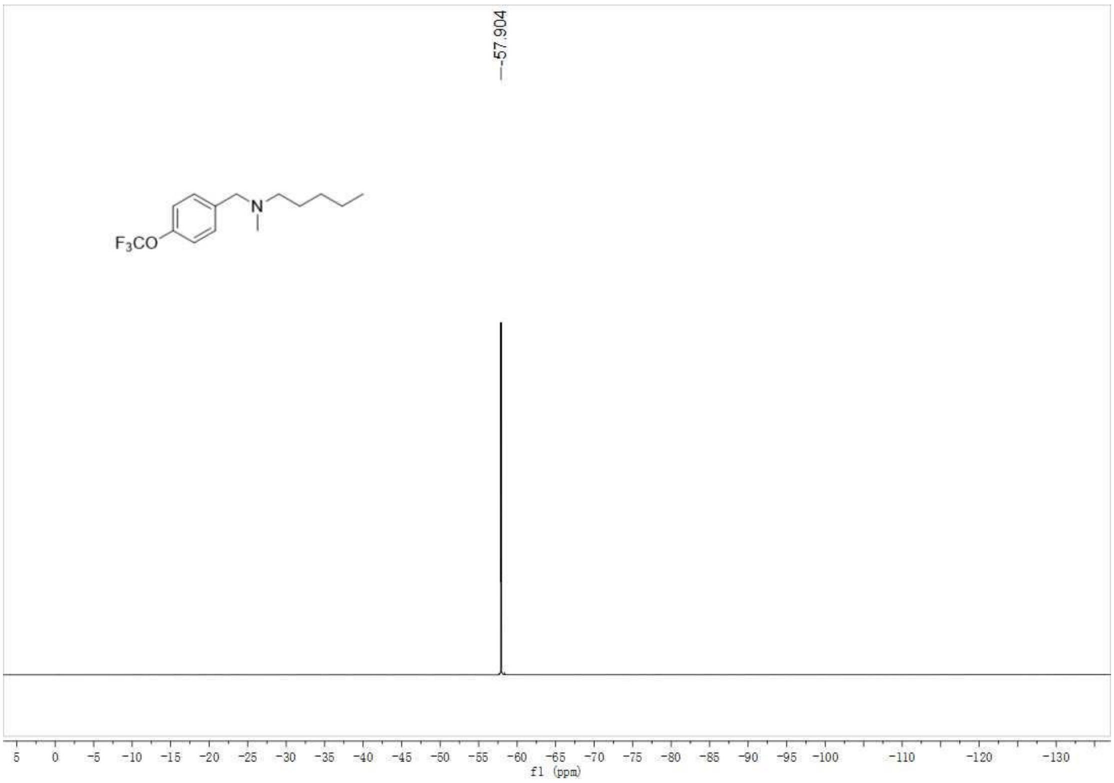

^{1}H NMR Spectrum of **11**

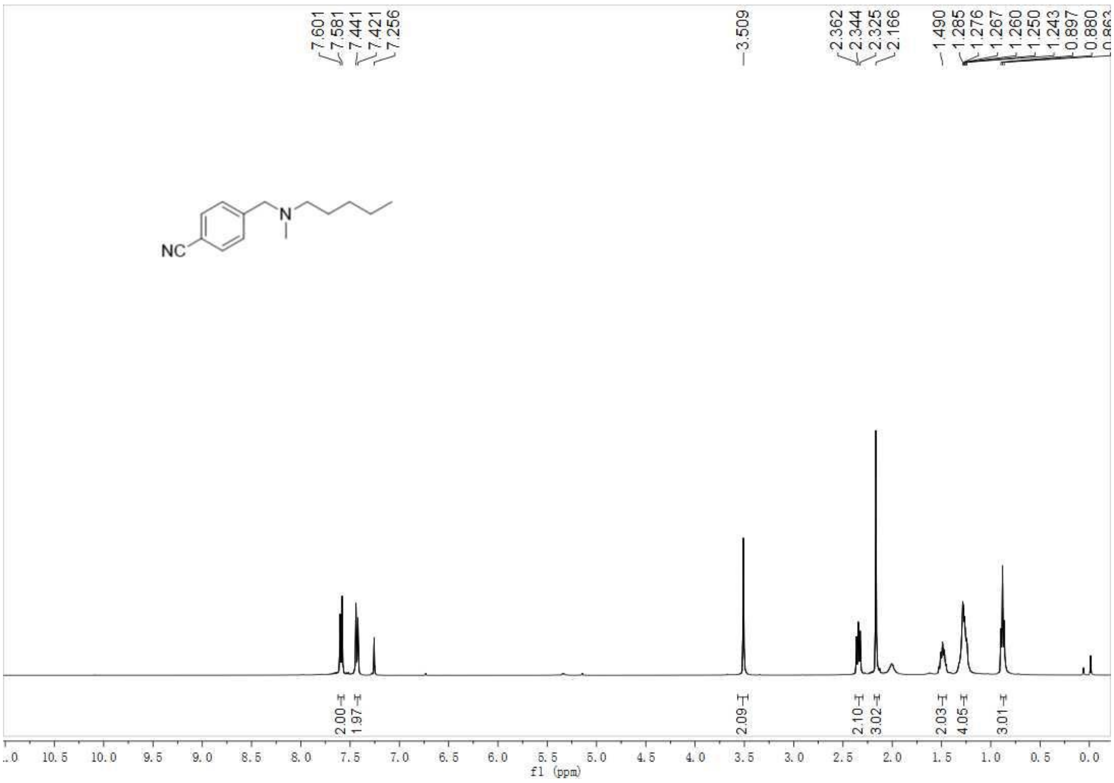

^{13}C NMR Spectrum of **11**

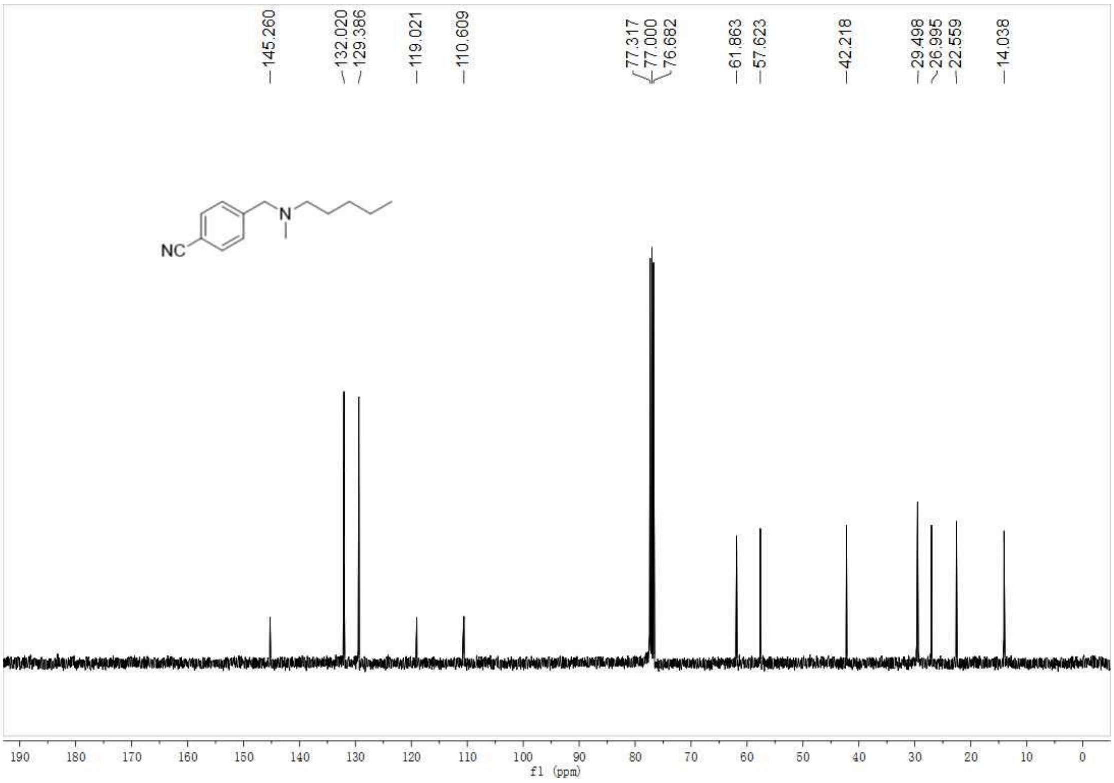

^{1}H NMR Spectrum of **12**

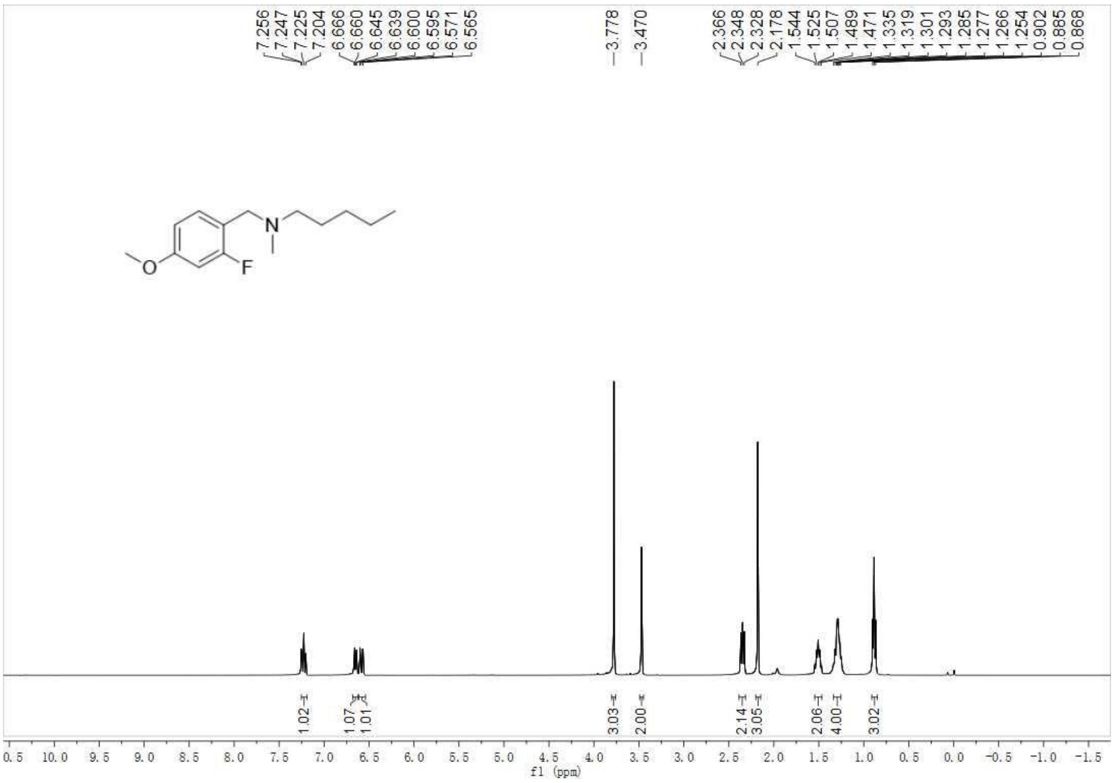

^{13}C NMR Spectrum of **12**

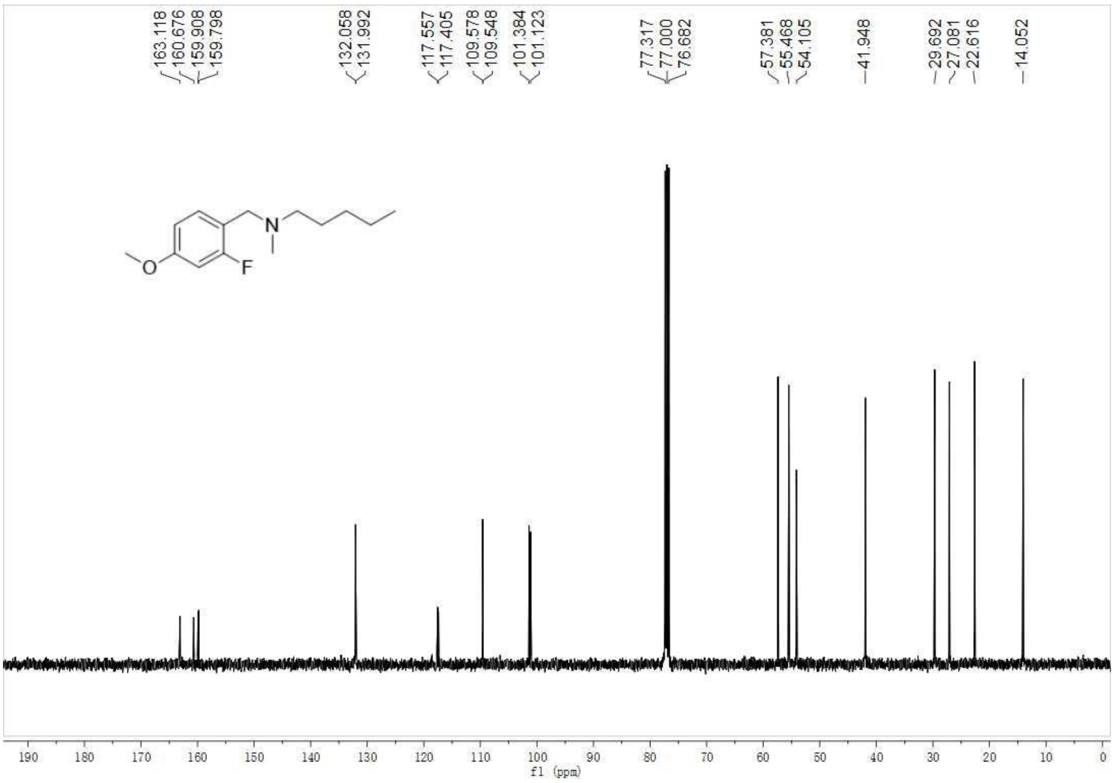

^{19}F NMR Spectrum of **12**

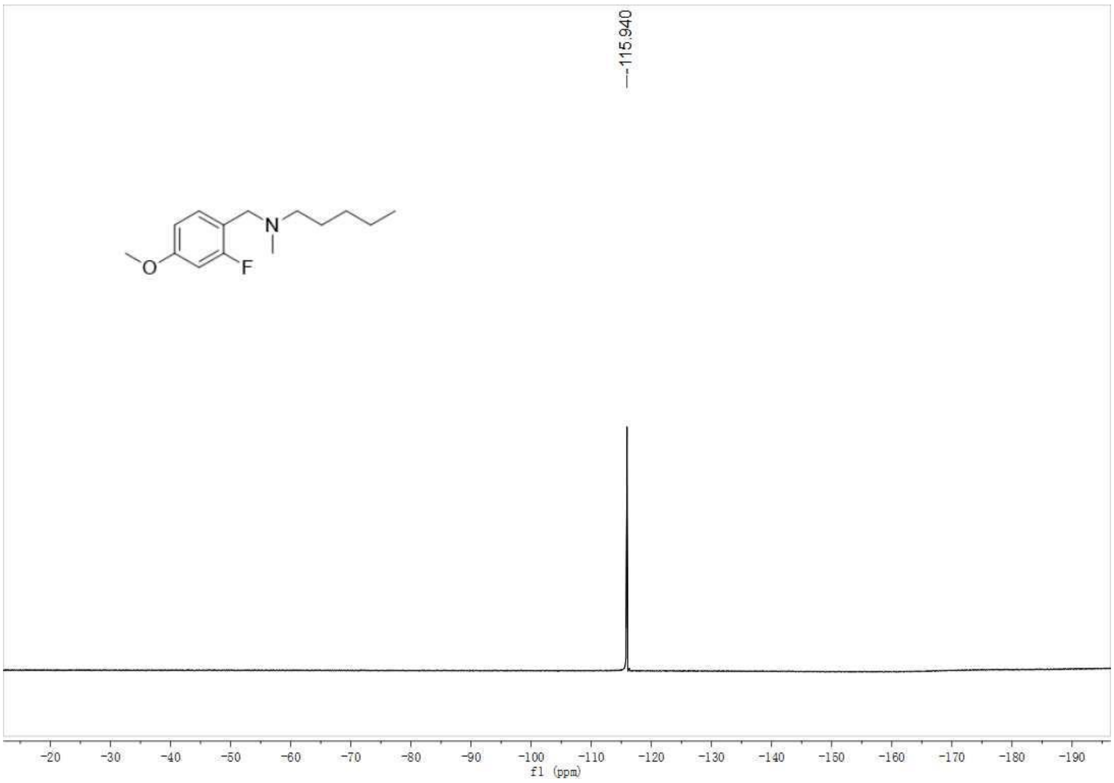

^{1}H NMR Spectrum of **13**

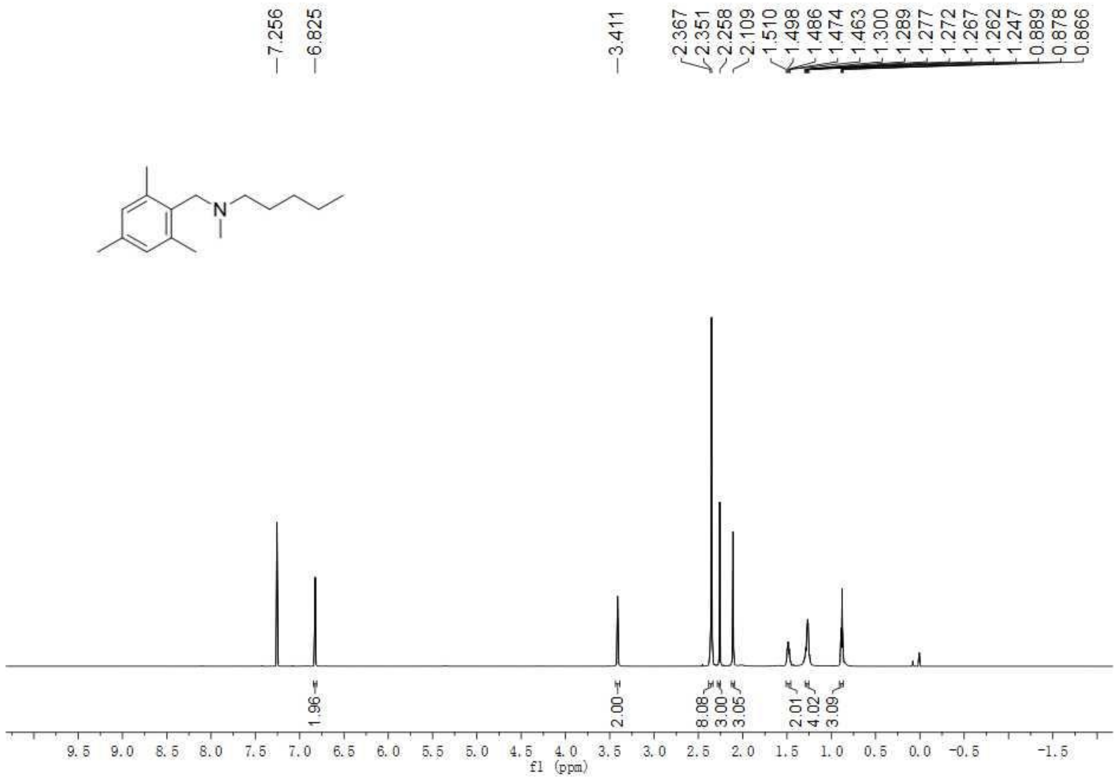

^{13}C NMR Spectrum of **13**

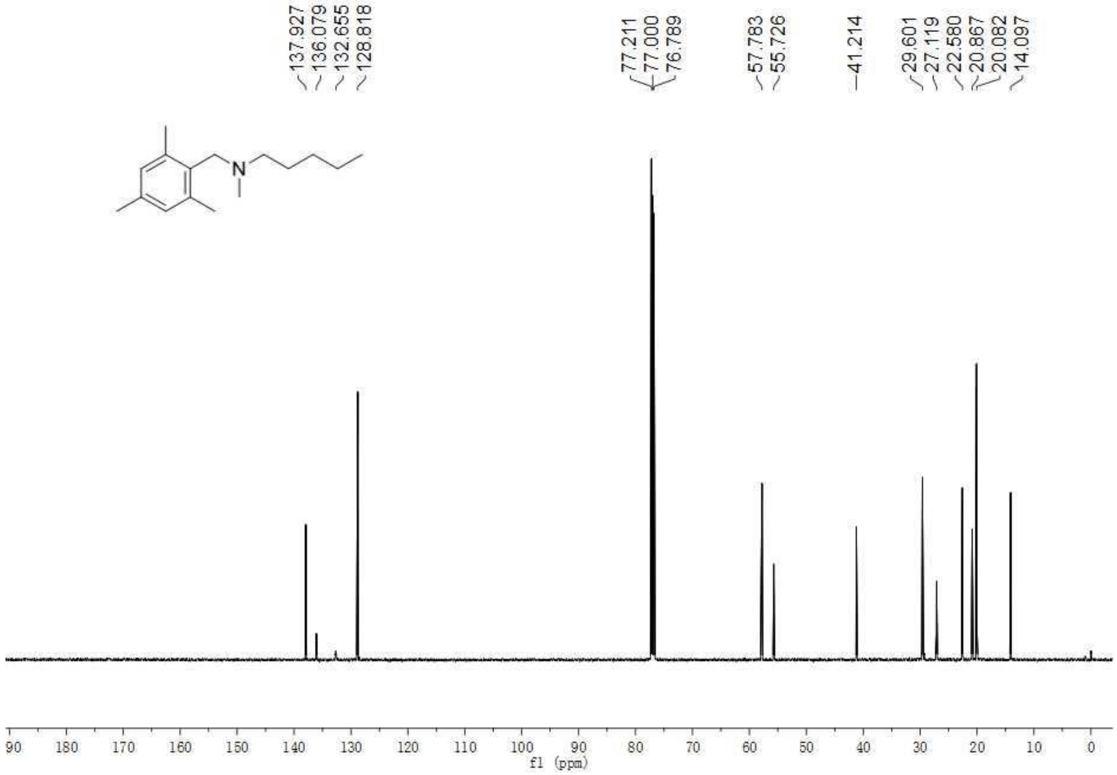

^{1}H NMR Spectrum of **14**

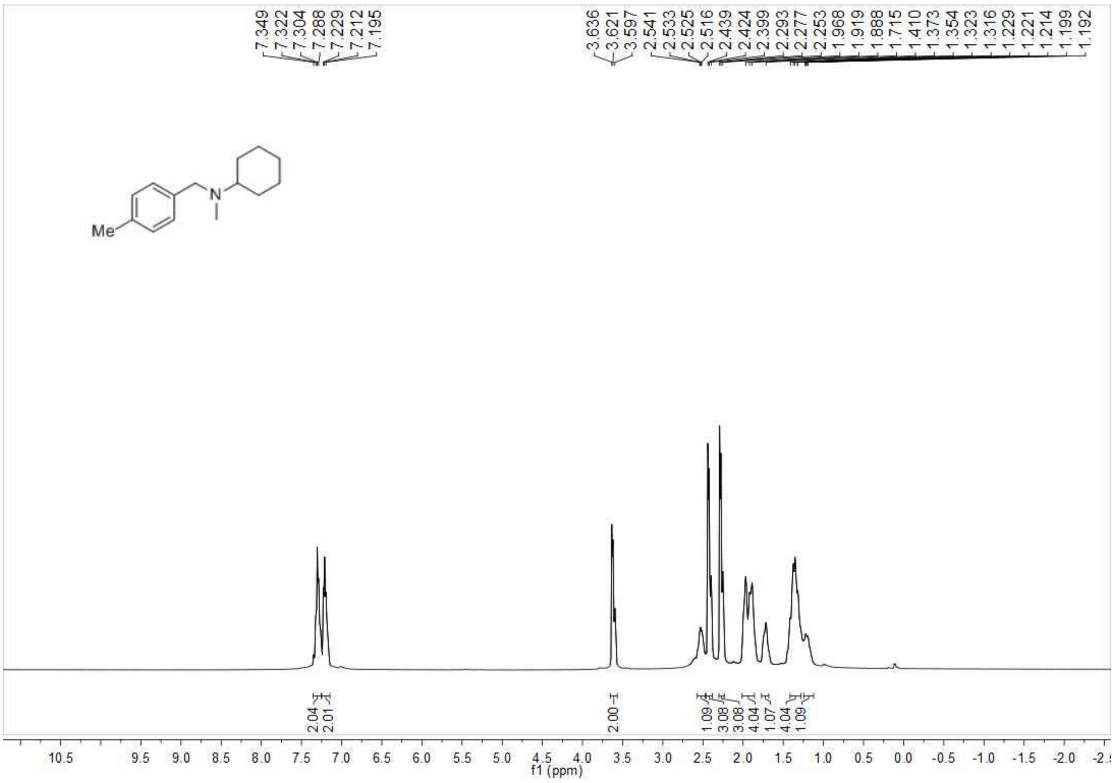

^{13}C NMR Spectrum of **14**

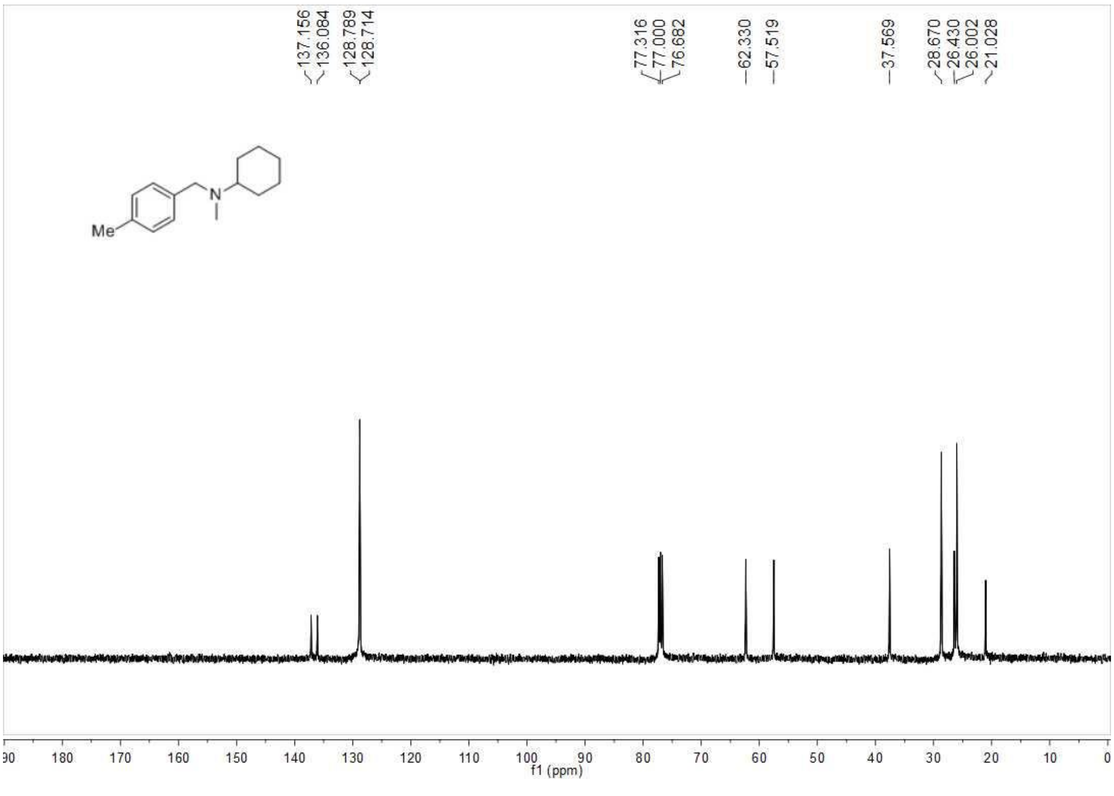

^{1}H NMR Spectrum of **15**

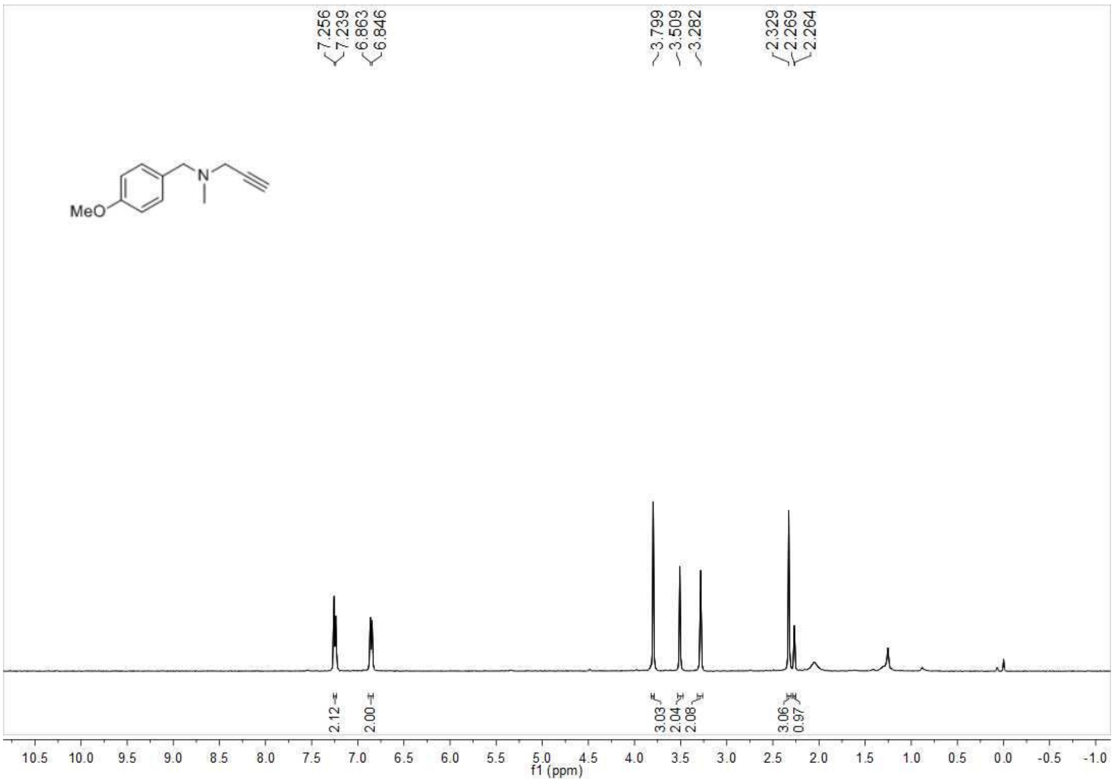

^{13}C NMR Spectrum of **15**

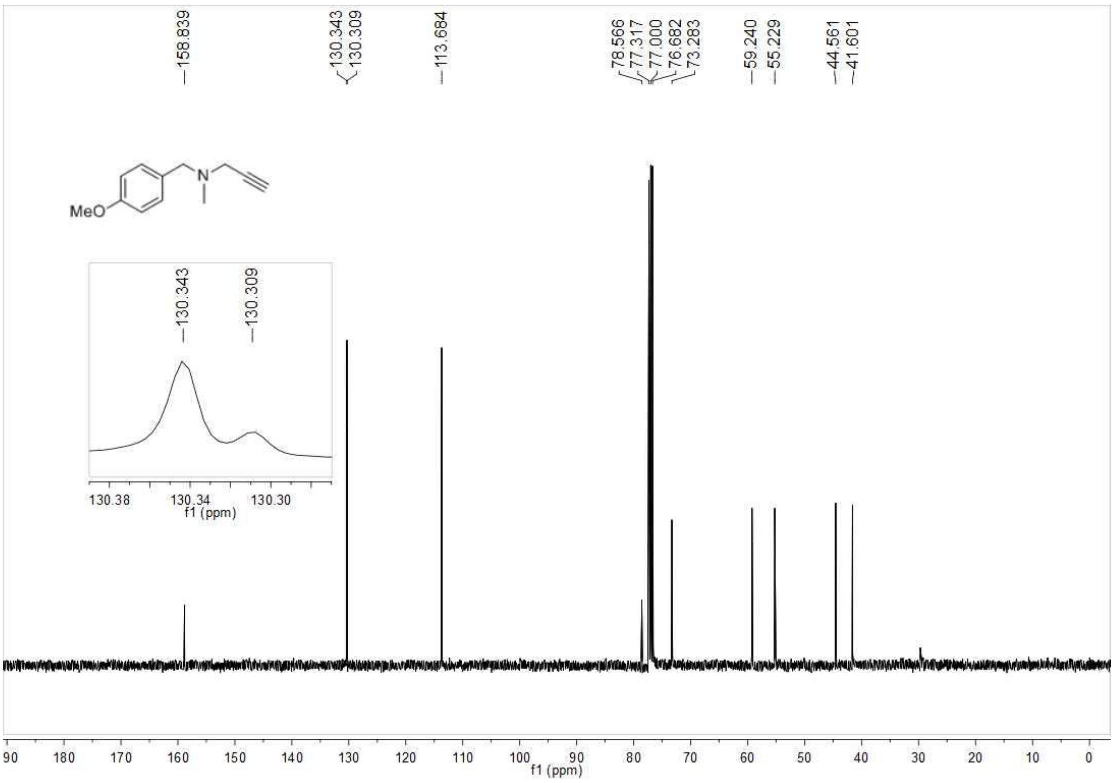

^{1}H NMR Spectrum of **16**

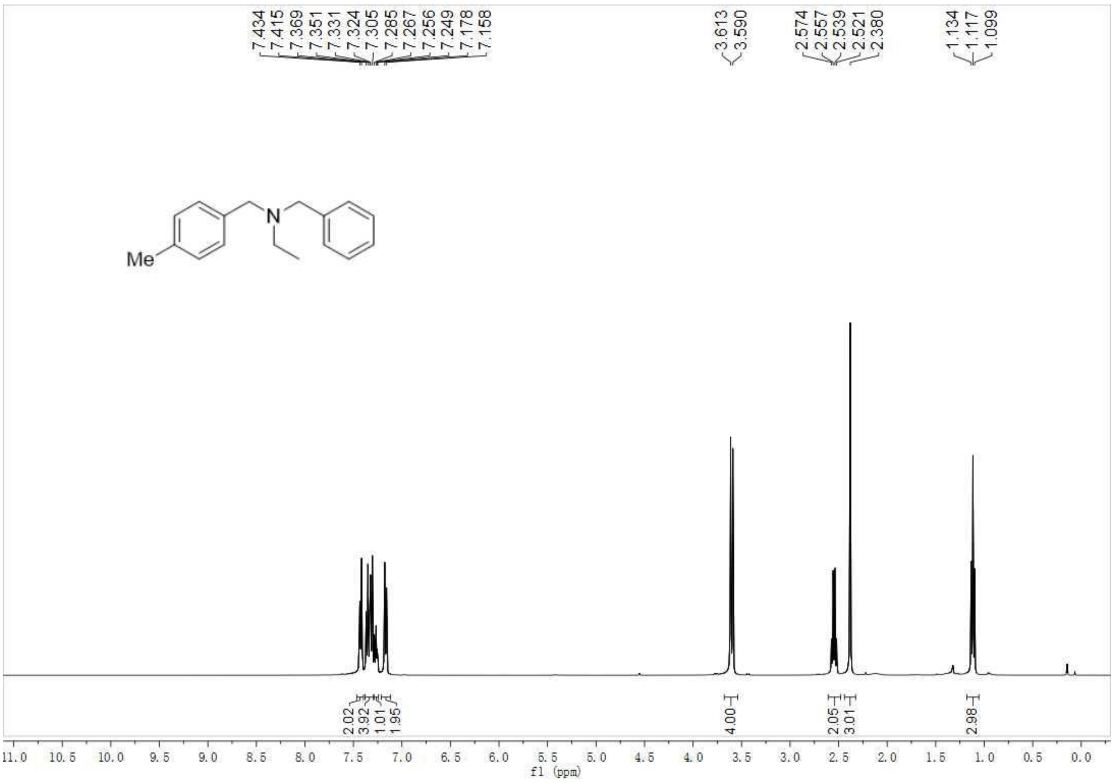

^{13}C NMR Spectrum of **16**

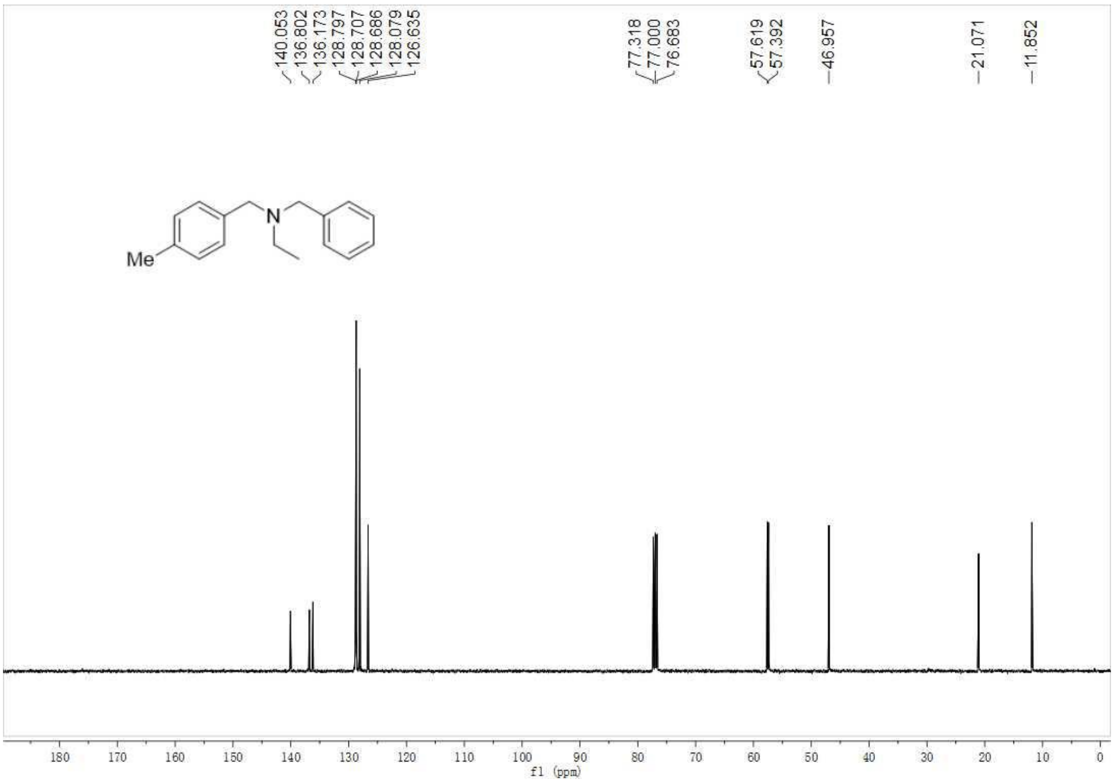

^{1}H NMR Spectrum of **17**

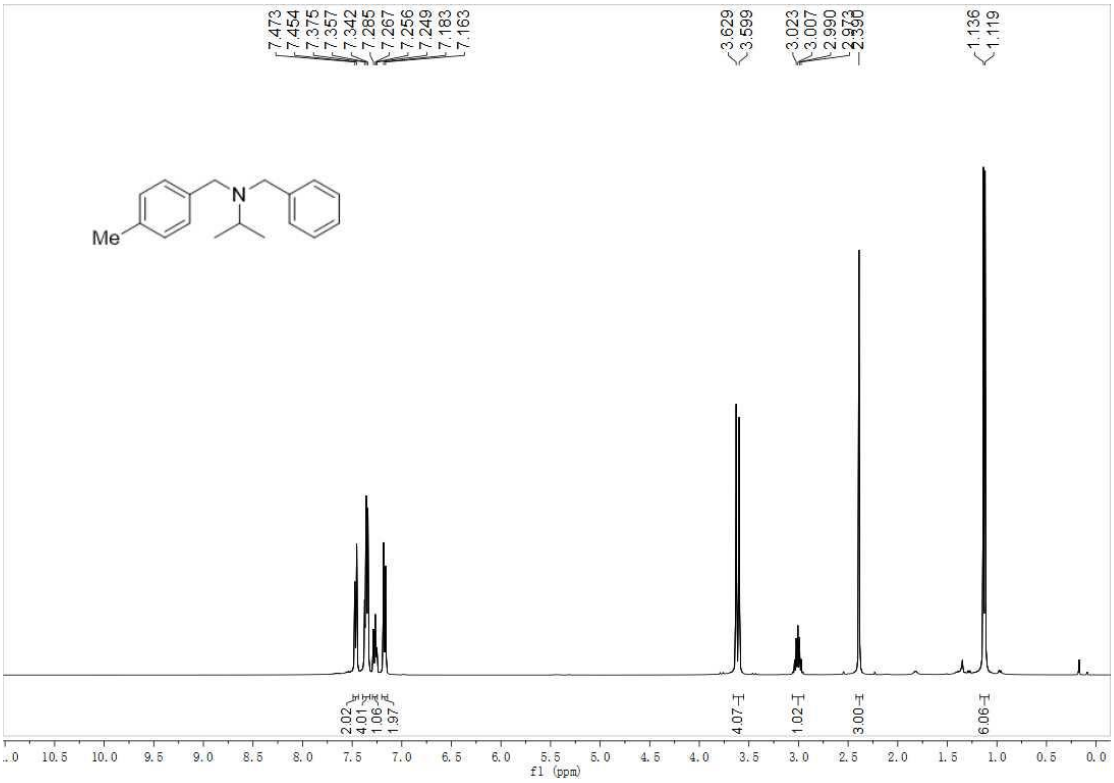

^{13}C NMR Spectrum of **17**

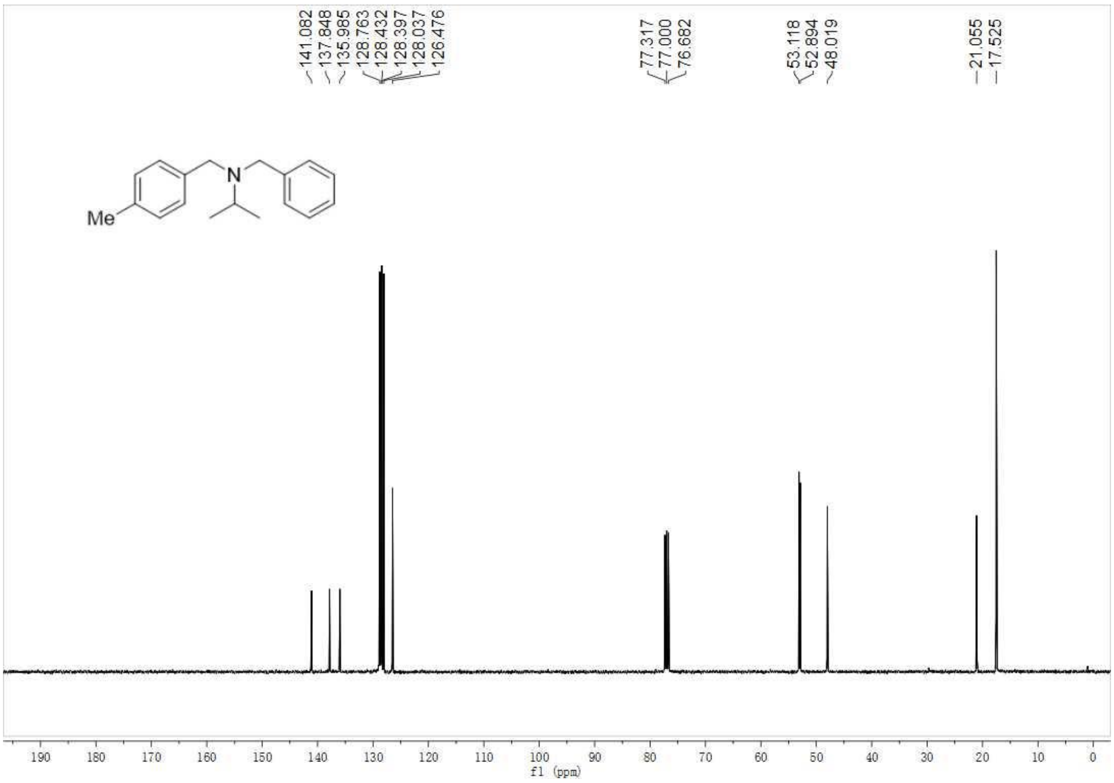

^{1}H NMR Spectrum of **18**

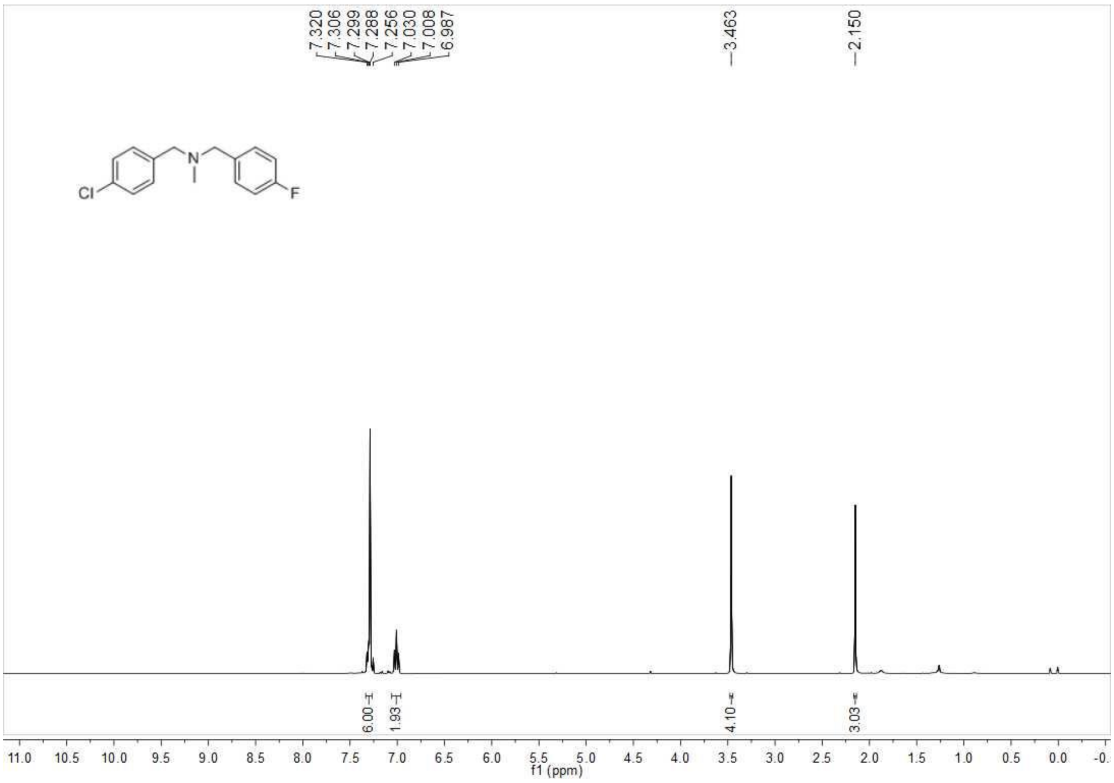

^{13}C NMR Spectrum of **18**

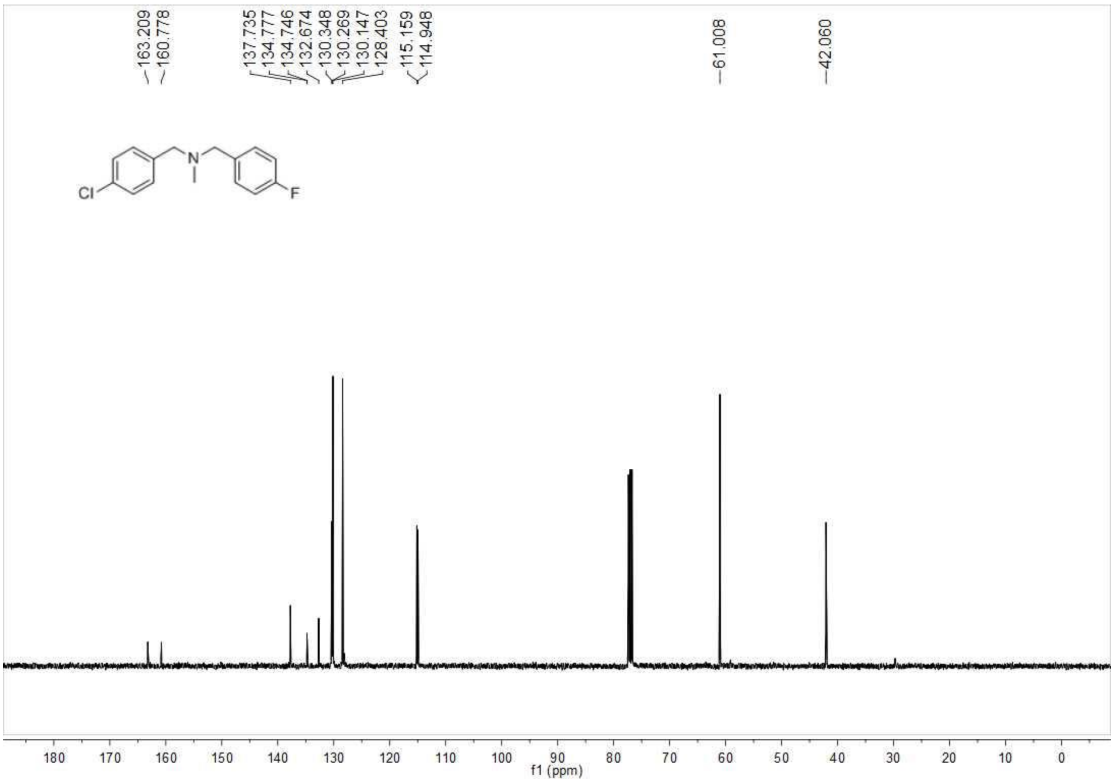

^{19}F NMR Spectrum of **18**

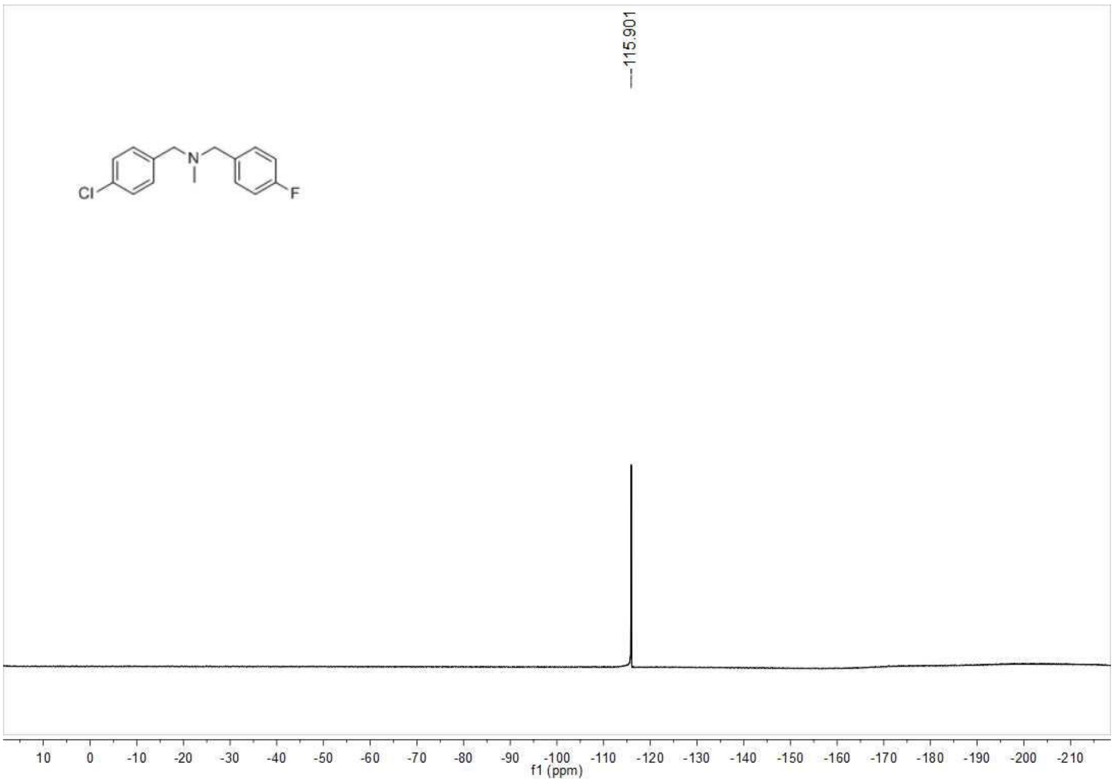

^{1}H NMR Spectrum of **19**

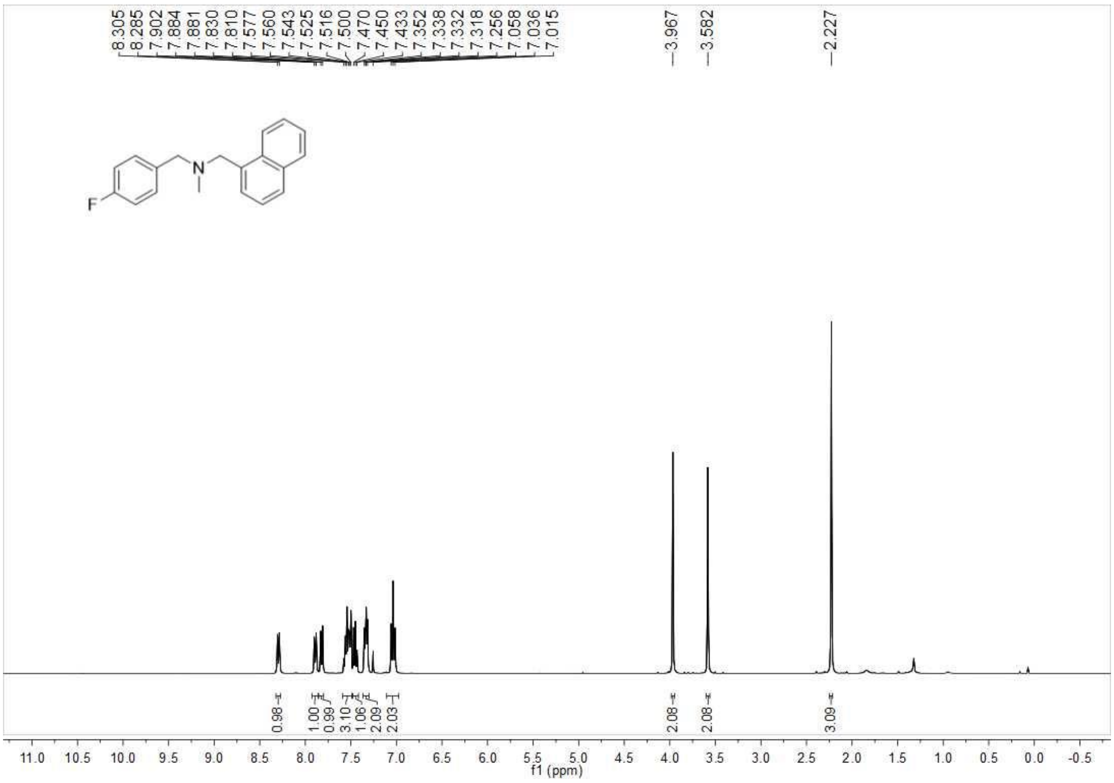

^{13}C NMR Spectrum of **19**

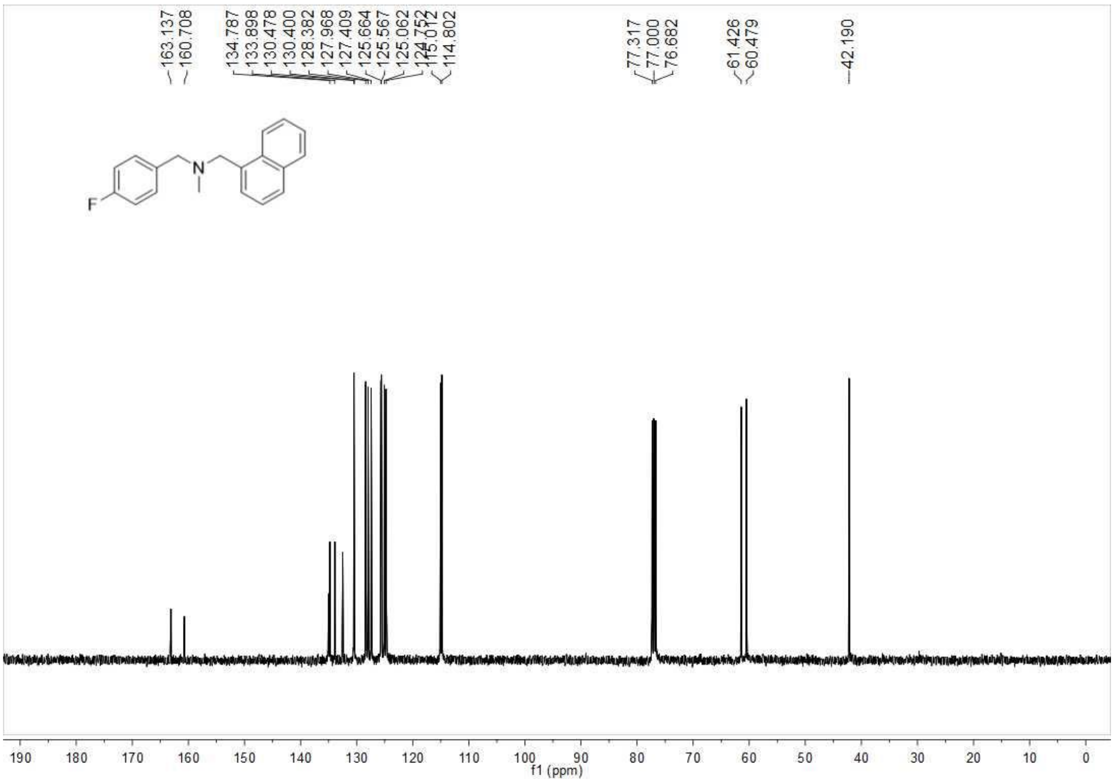

^{19}F NMR Spectrum of **19**

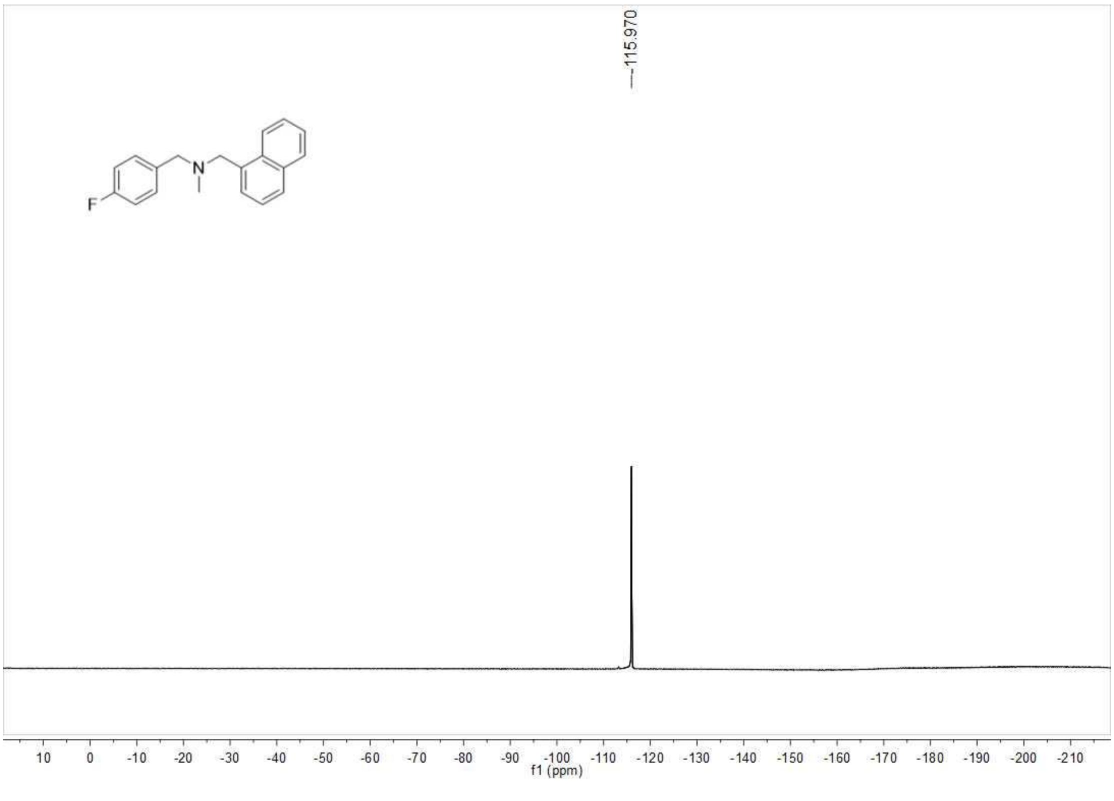

^{1}H NMR Spectrum of **20**

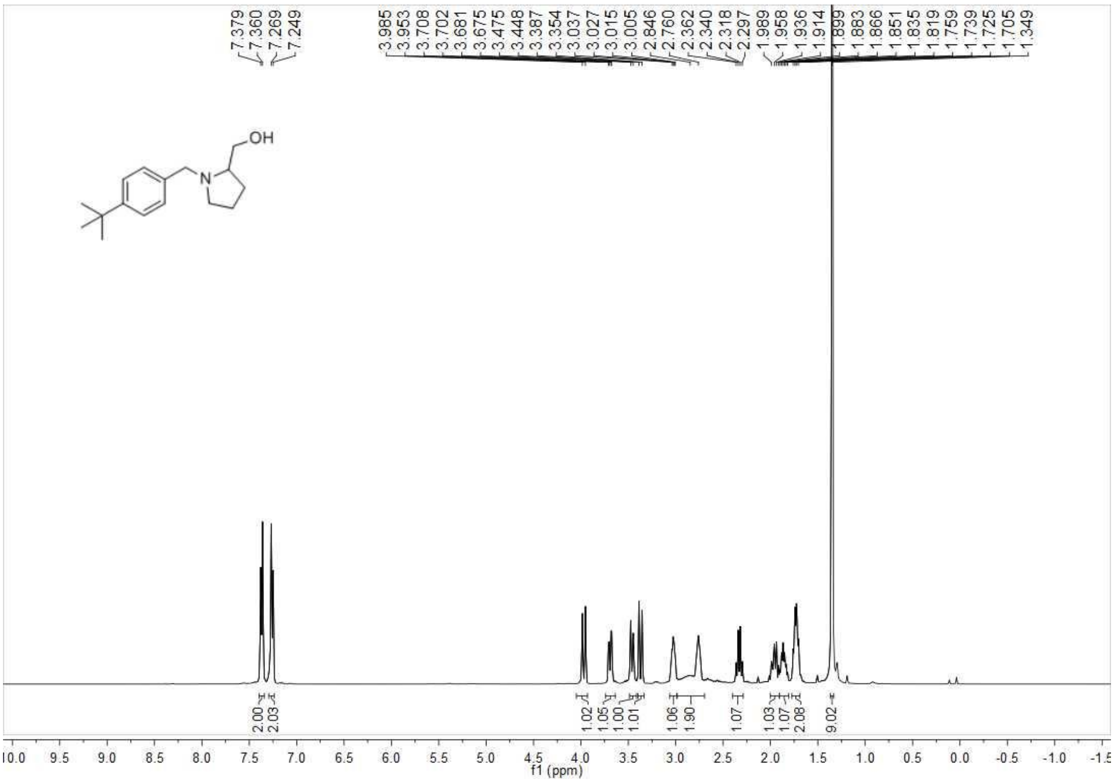

^{13}C NMR Spectrum of **20**

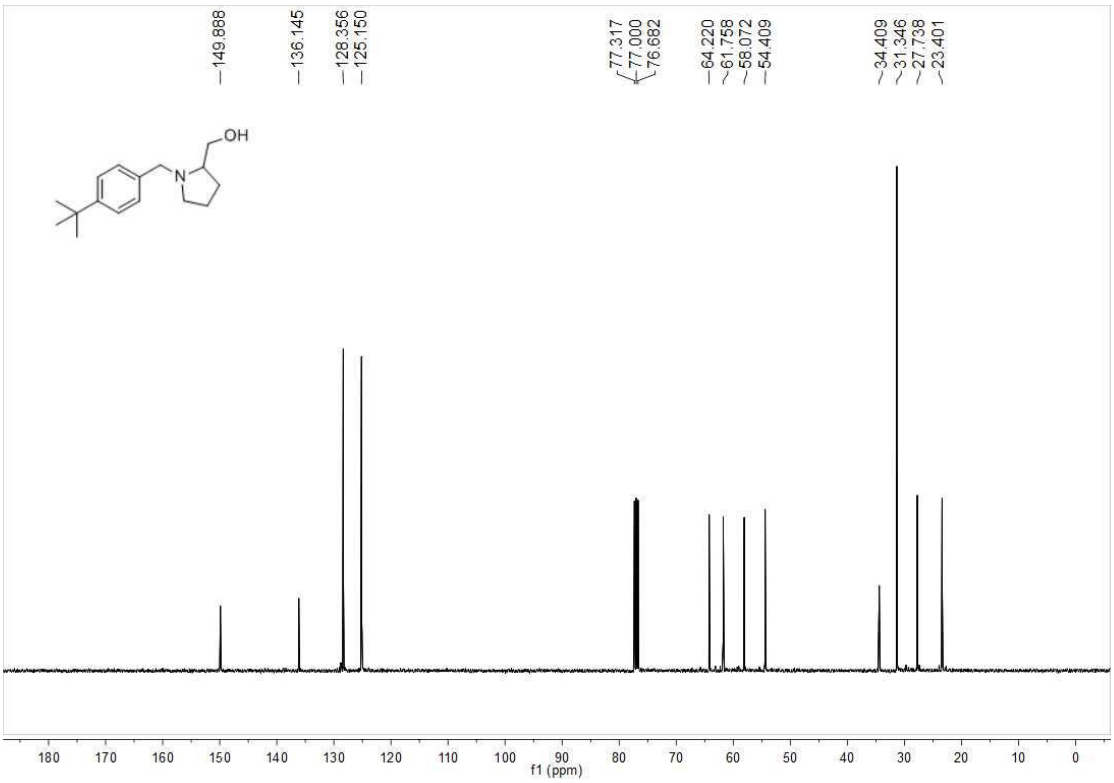

^{1}H NMR Spectrum of **21**

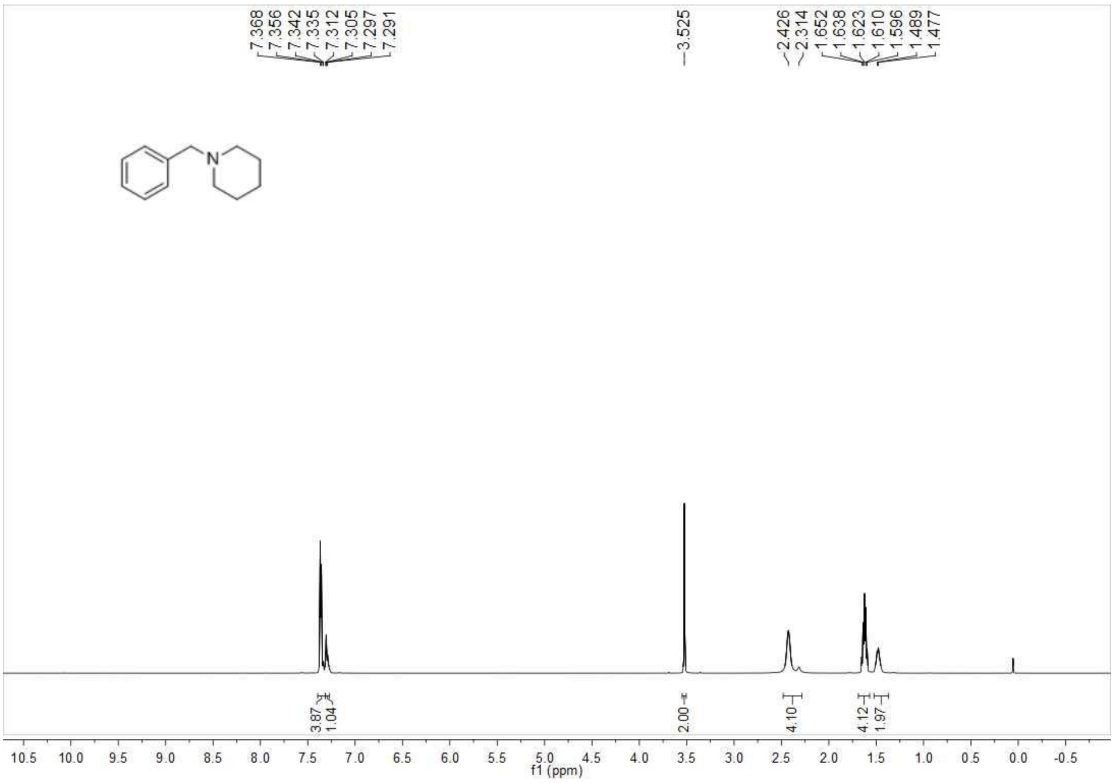

^{13}C NMR Spectrum of **21**

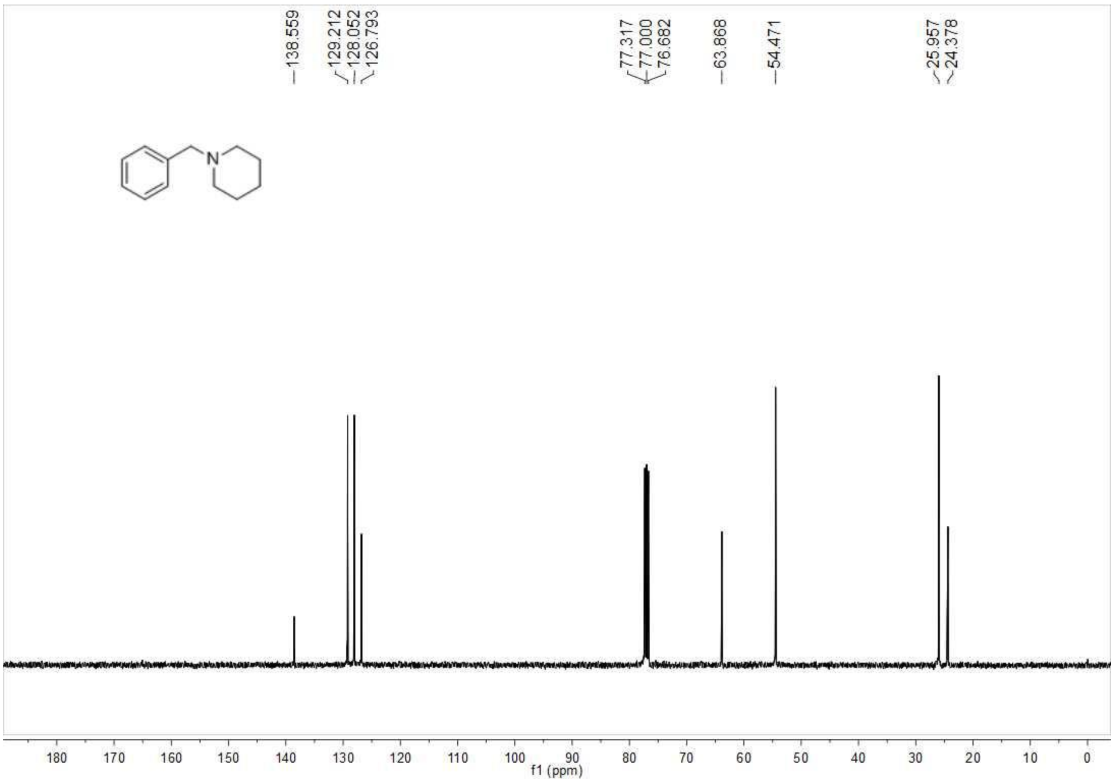

^{1}H NMR Spectrum of **22**

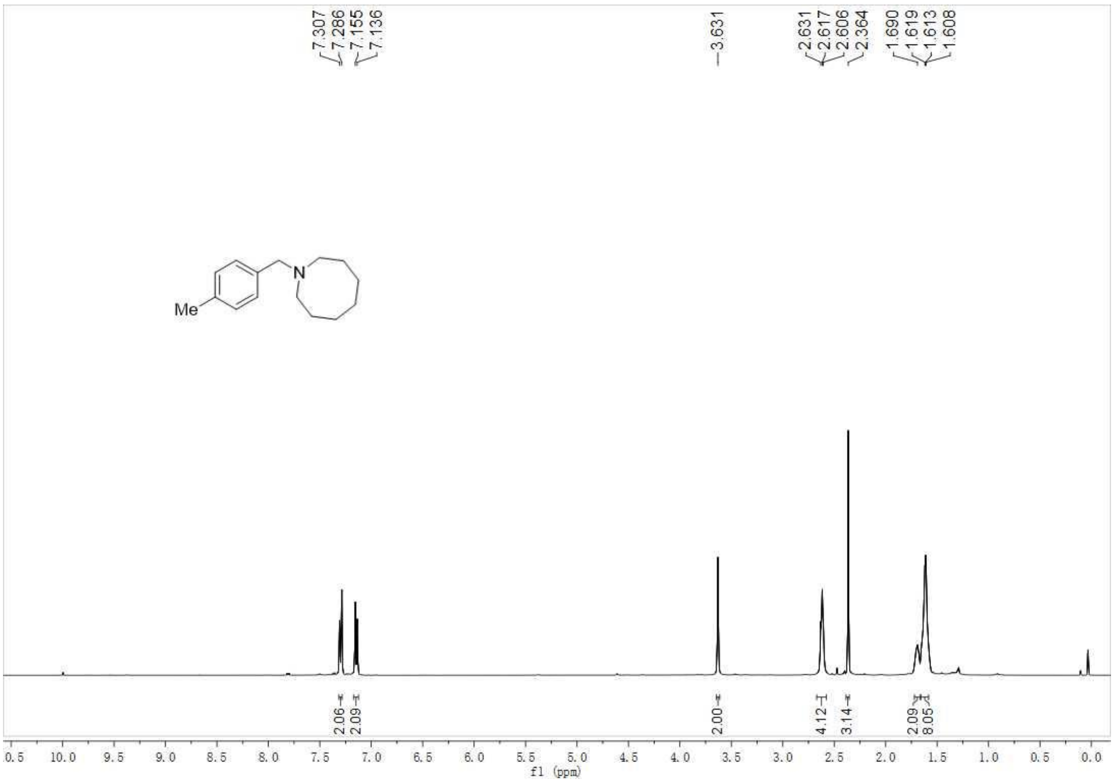

^{13}C NMR Spectrum of **22**

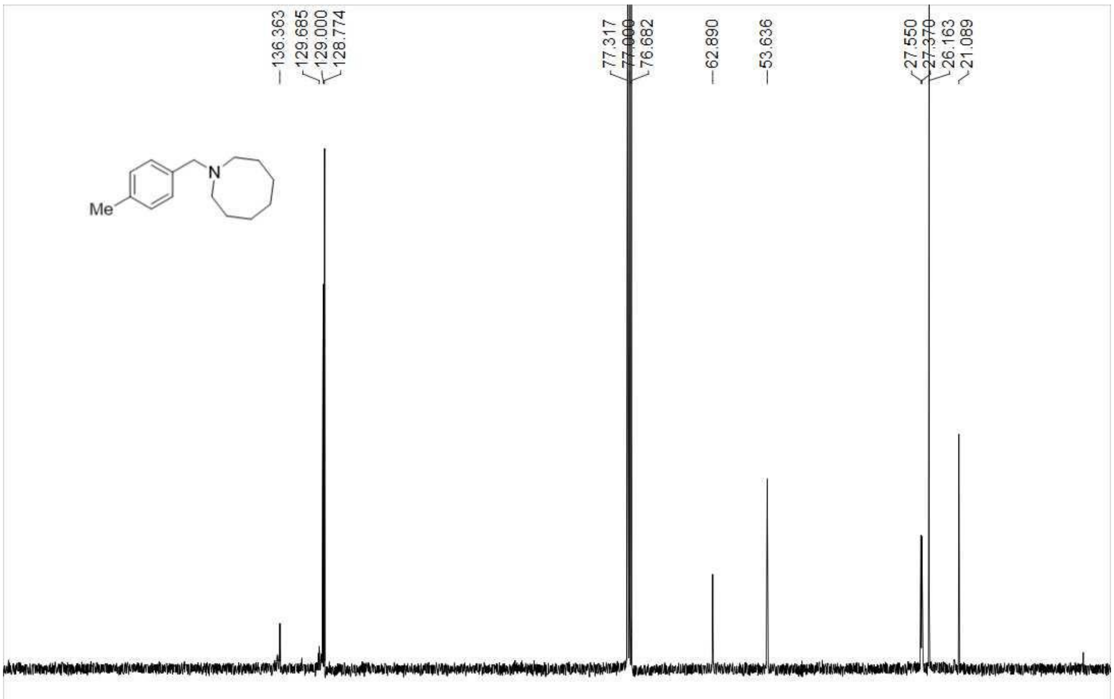

^{1}H NMR Spectrum of **23**

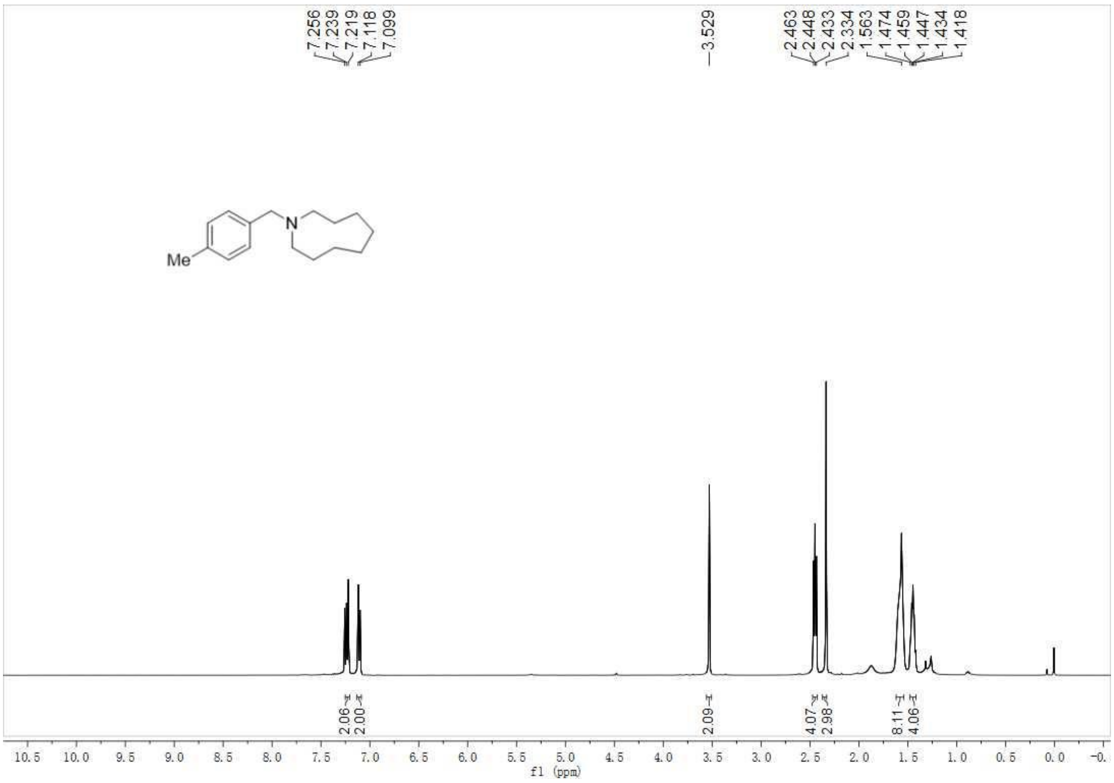

^{13}C NMR Spectrum of **23**

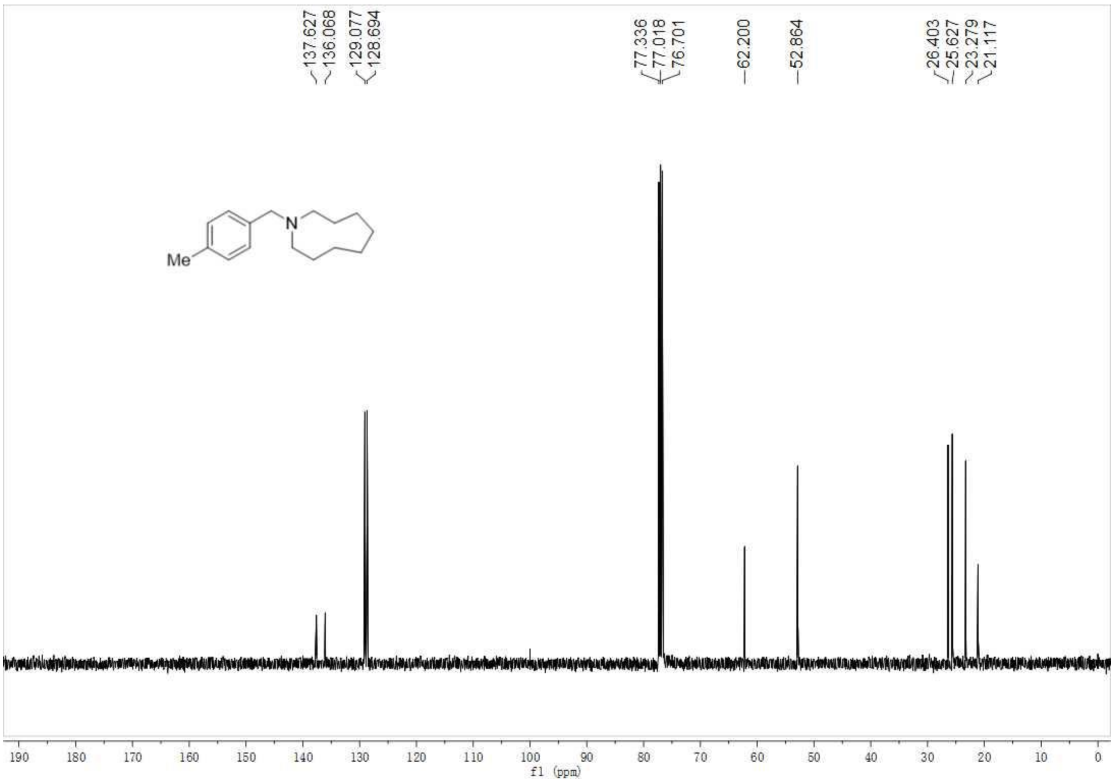

^{1}H NMR Spectrum of **24**

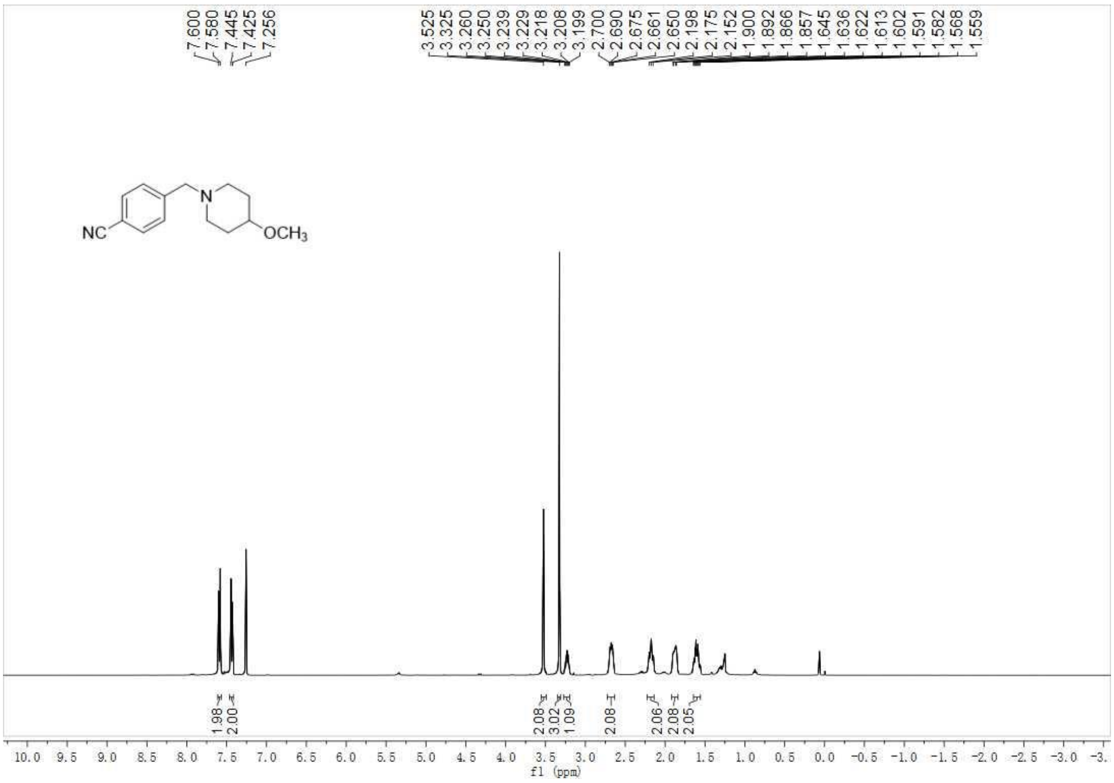

^{13}C NMR Spectrum of **24**

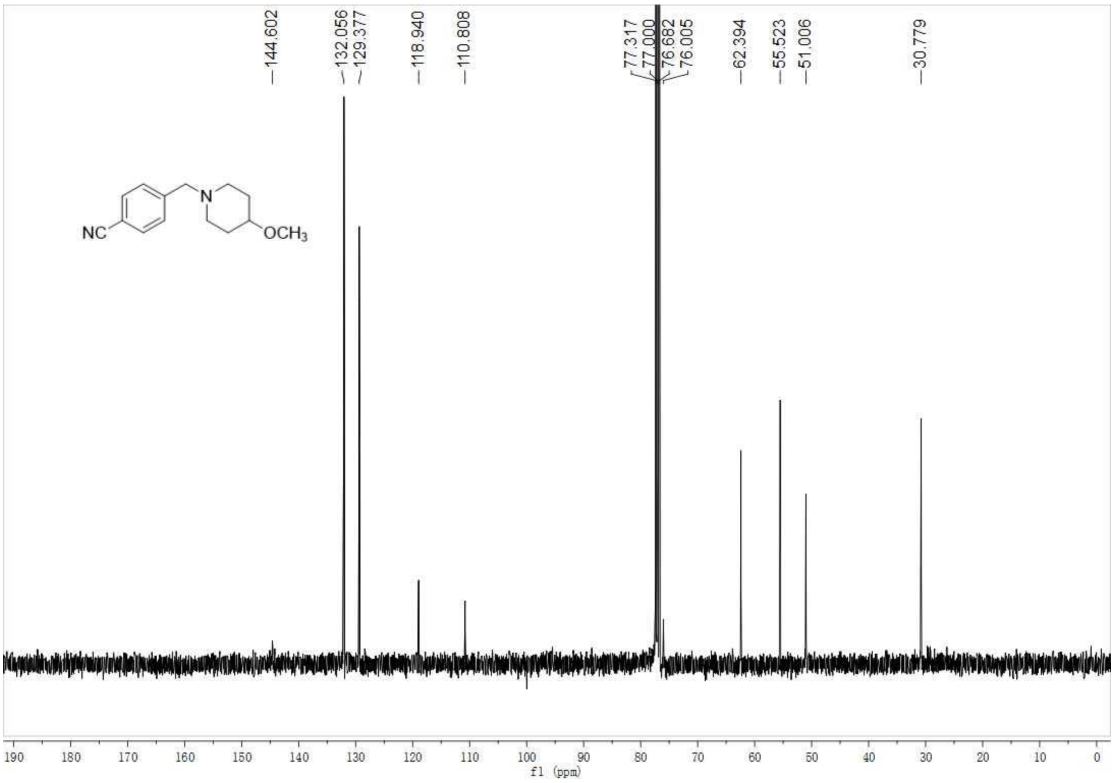

^{1}H NMR Spectrum of **25**

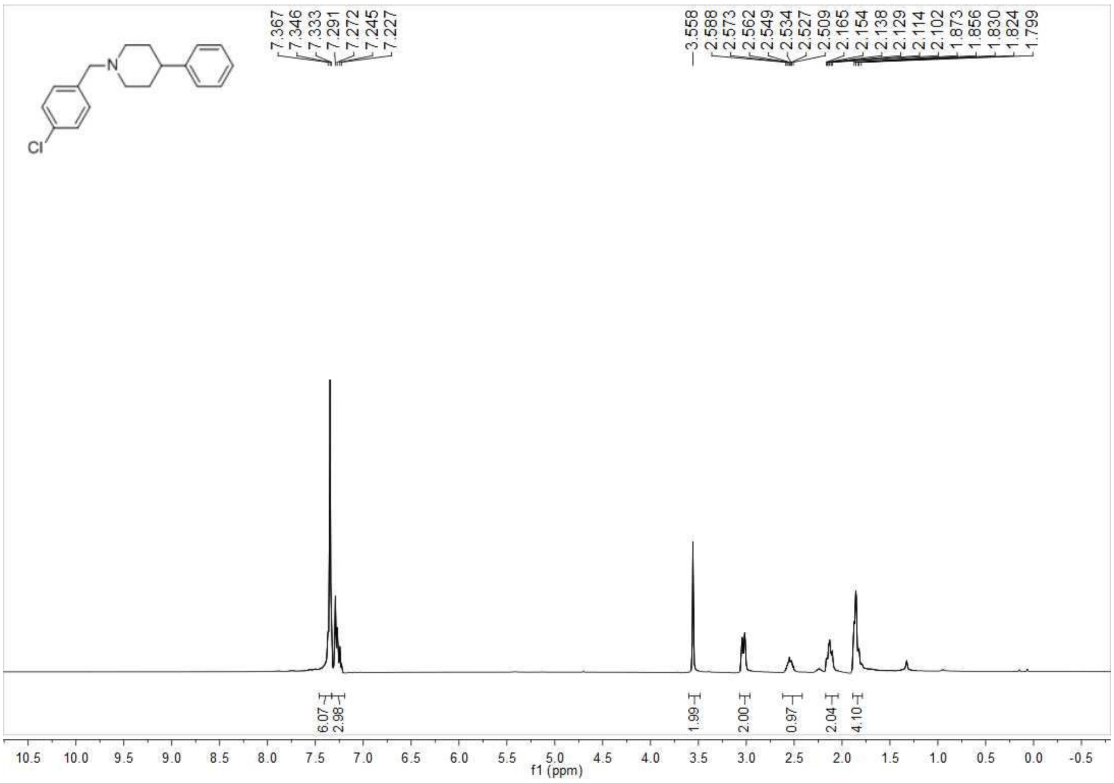

^{13}C NMR Spectrum of **25**

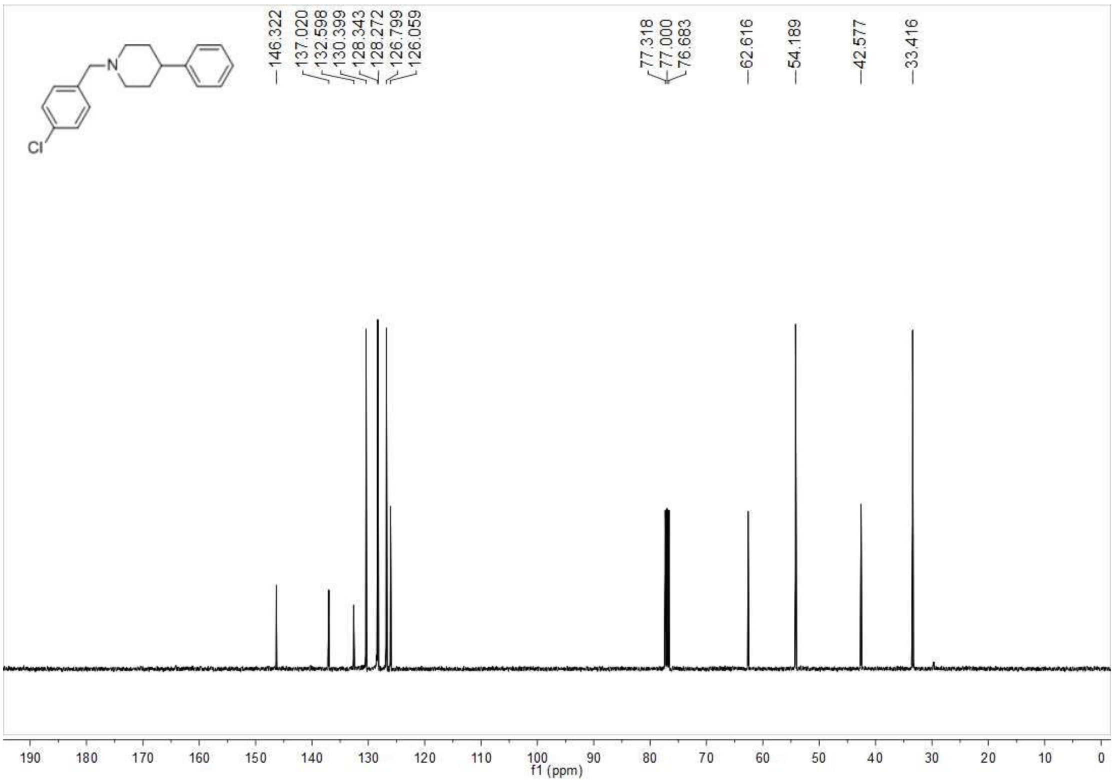

^{1}H NMR Spectrum of **26**

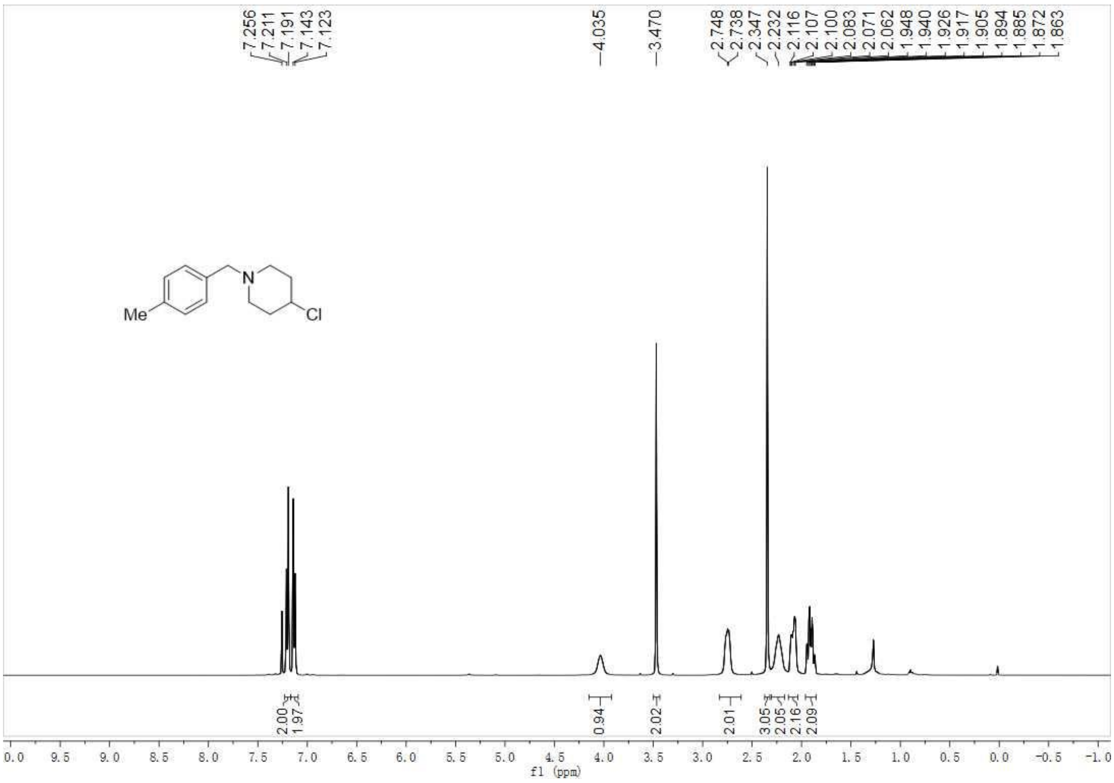

^{13}C NMR Spectrum of **26**

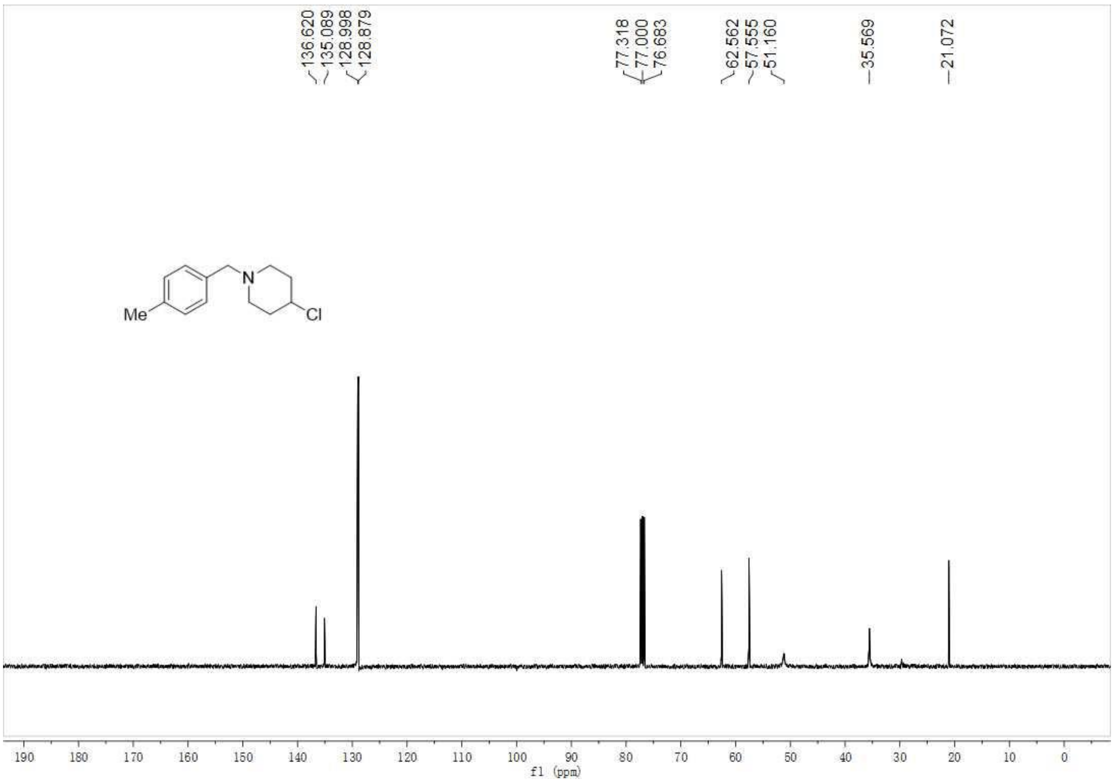

^{1}H NMR Spectrum of **27**

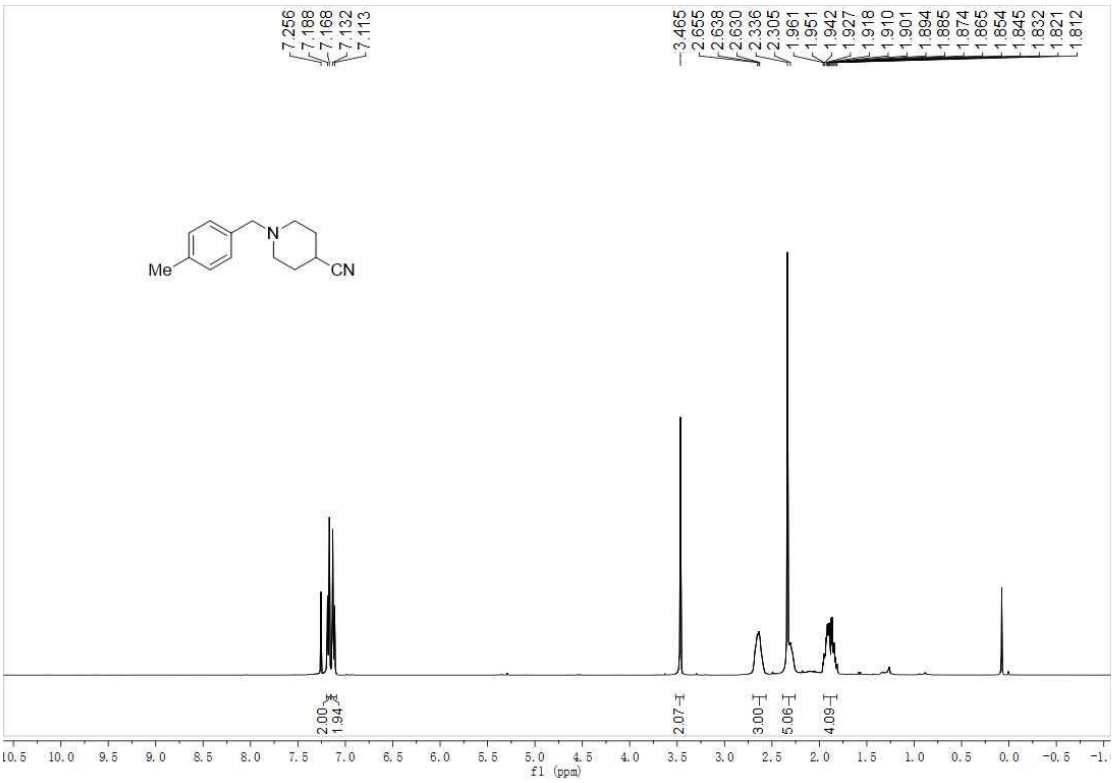

^{13}C NMR Spectrum of **27**

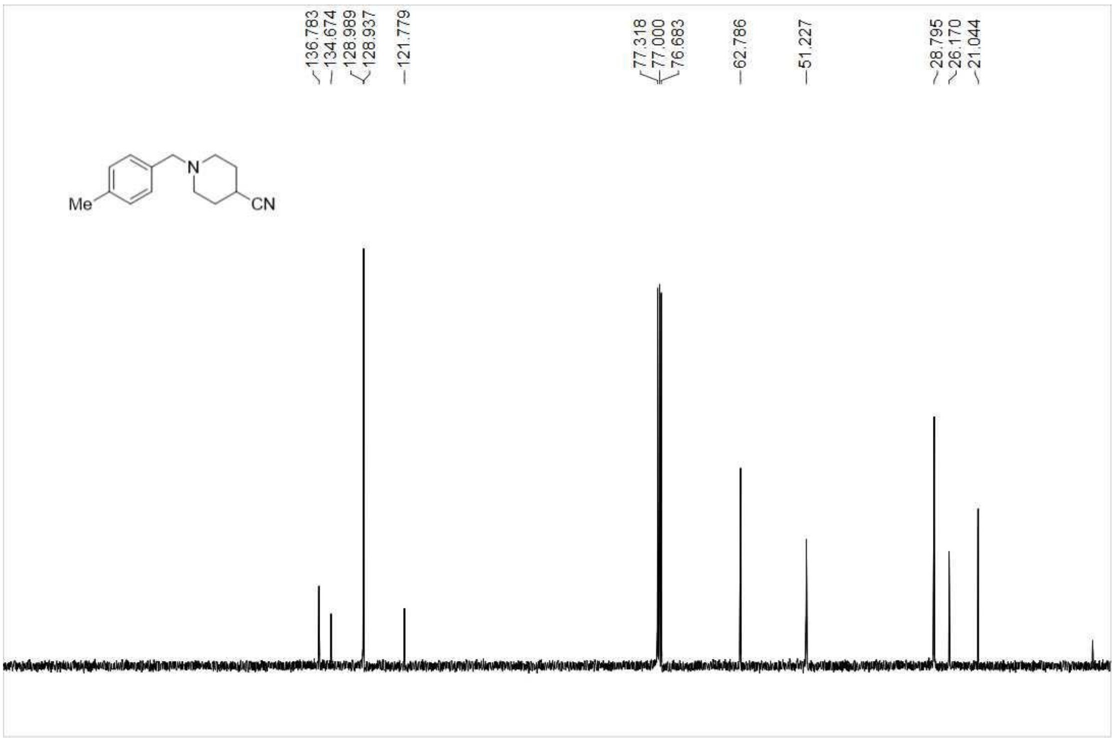

^{1}H NMR Spectrum of **28**

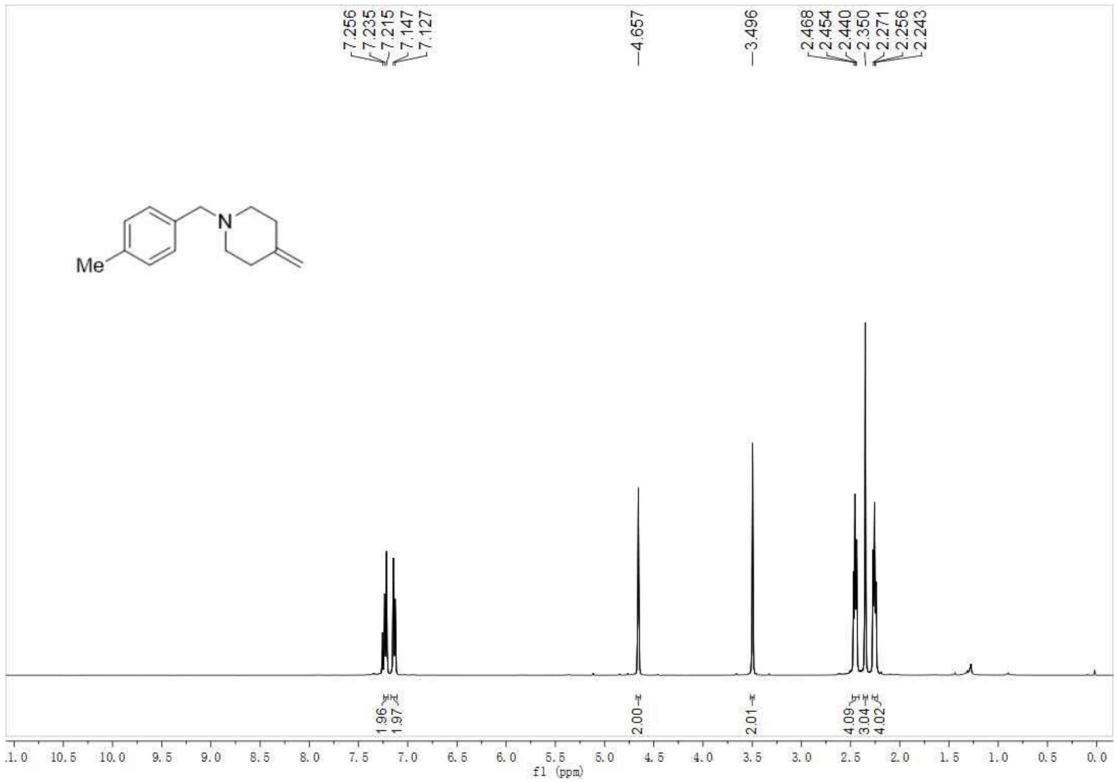

^{13}C NMR Spectrum of **28**

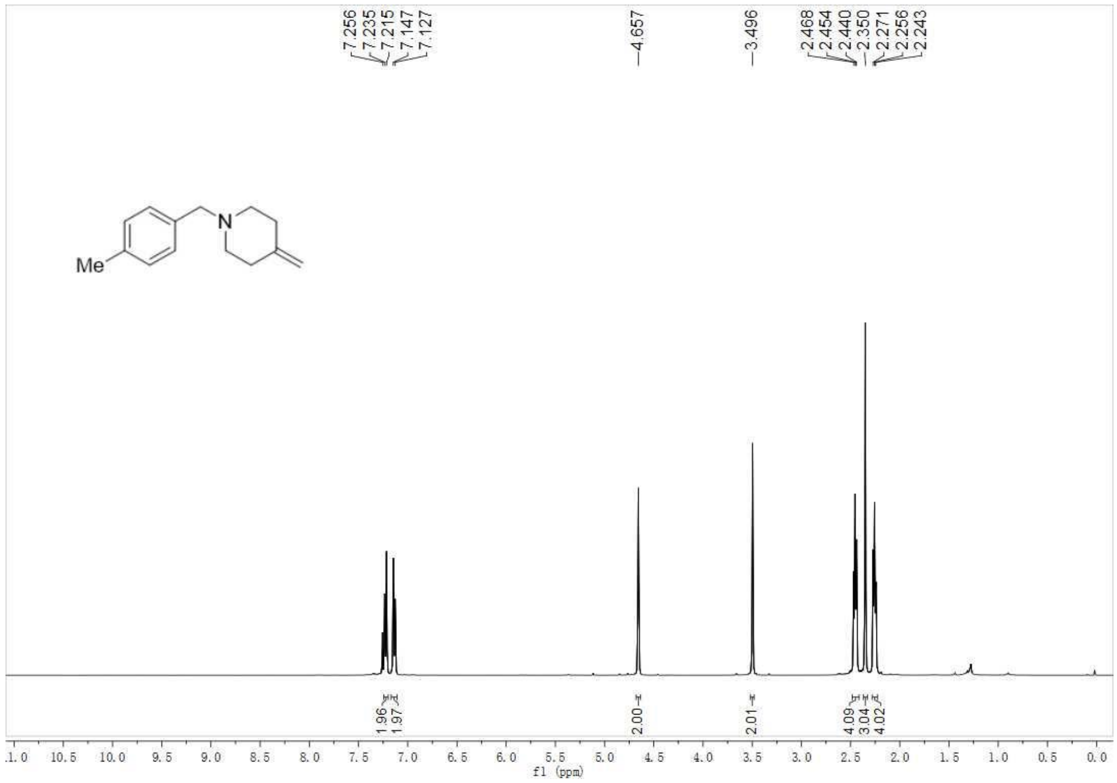

^{1}H NMR Spectrum of **29**

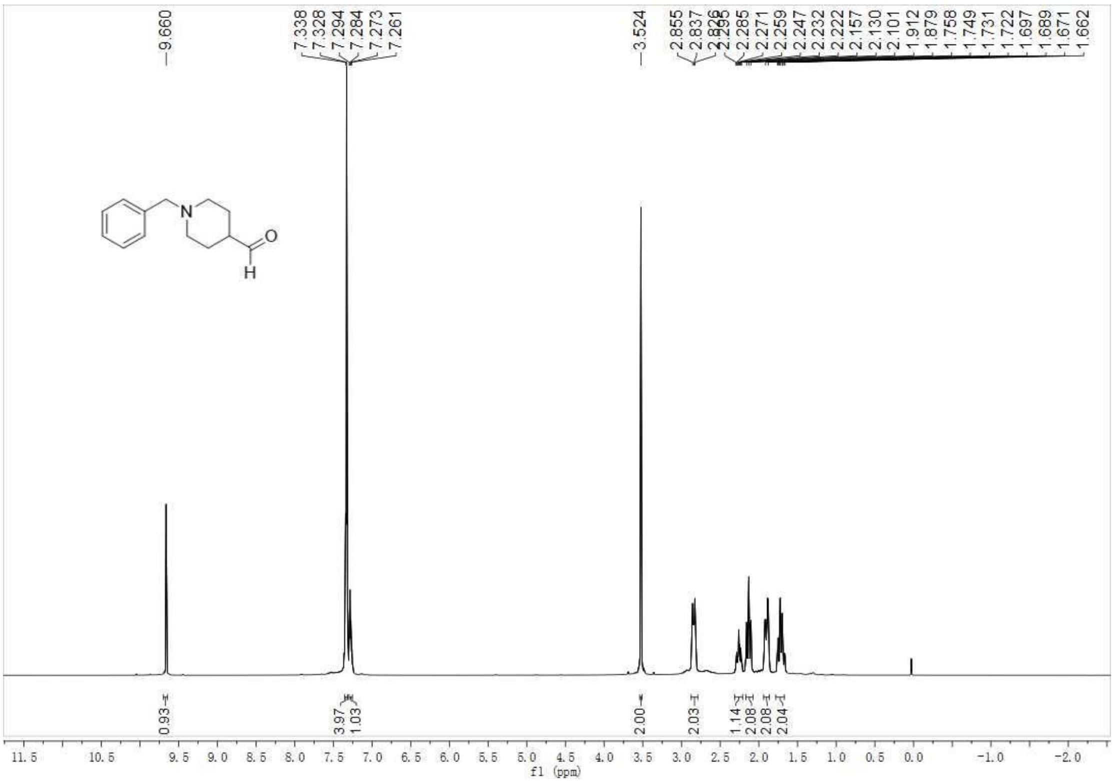

^{13}C NMR Spectrum of **29**

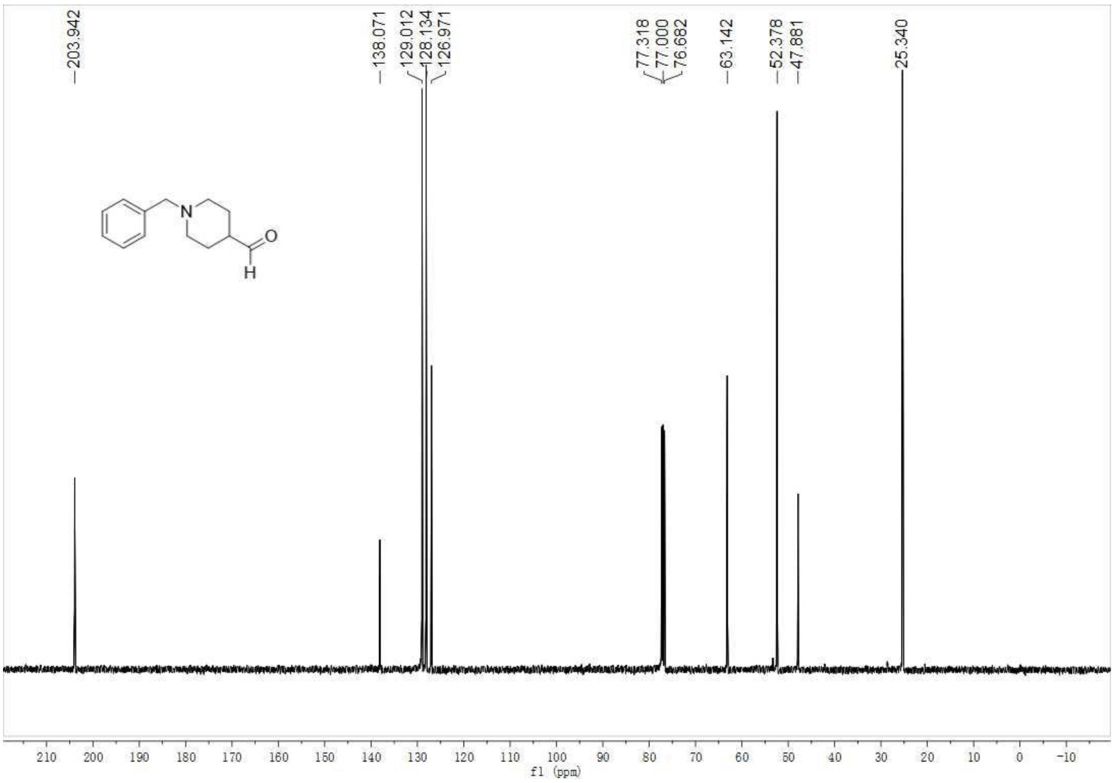

^{1}H NMR Spectrum of **30**

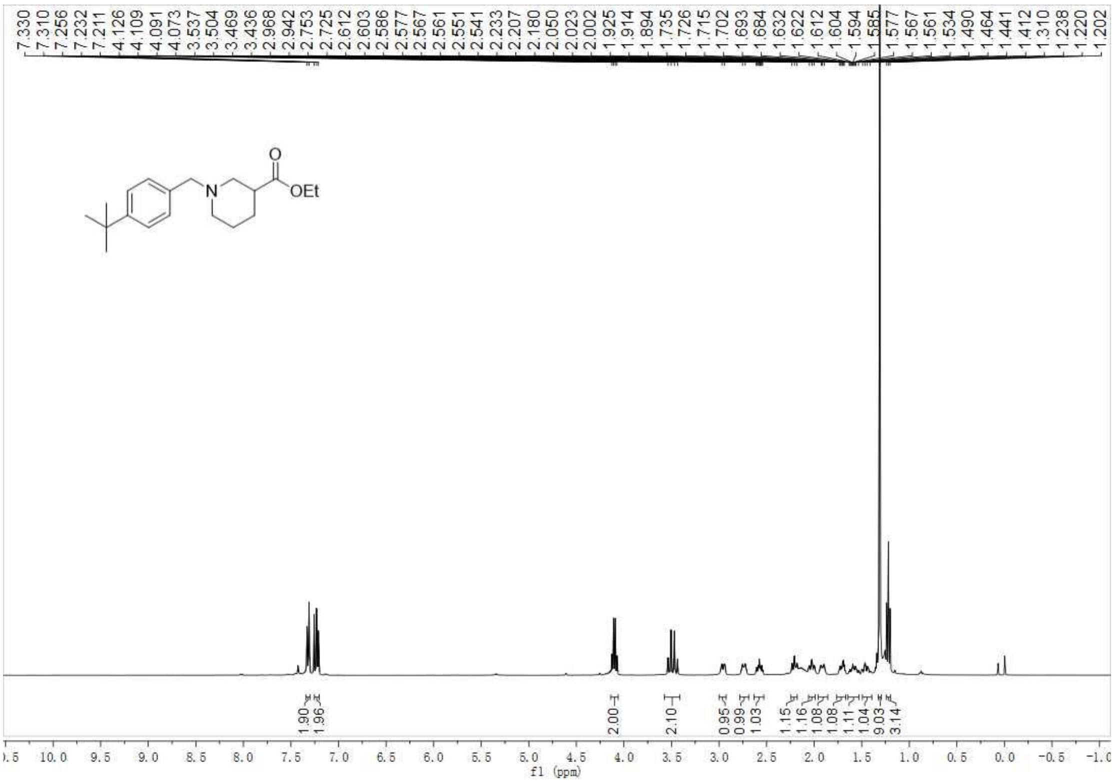

^{13}C NMR Spectrum of **30**

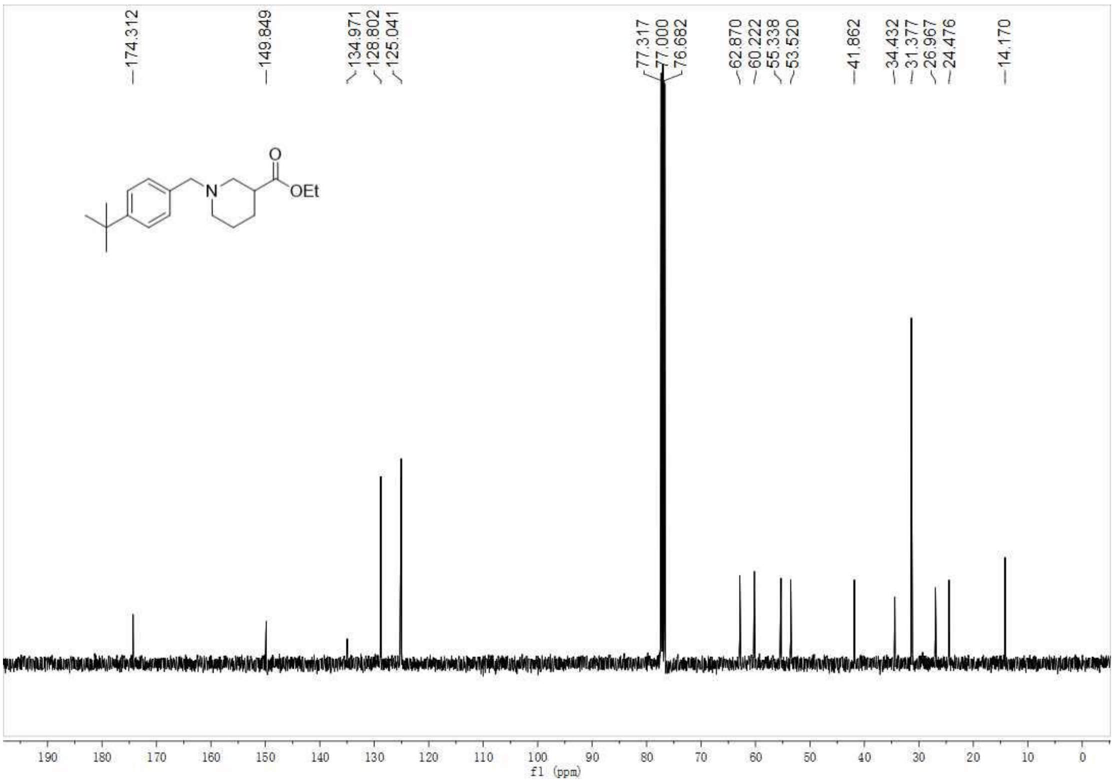

^{1}H NMR Spectrum of **31**

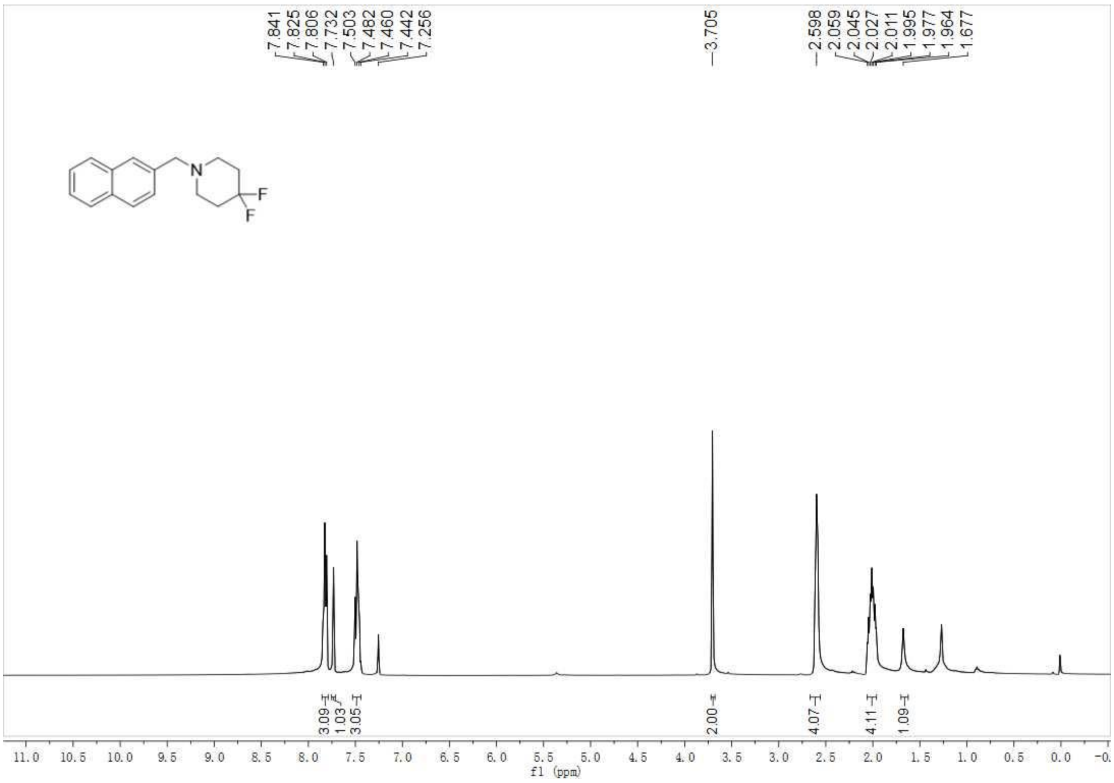

^{13}C NMR Spectrum of **31**

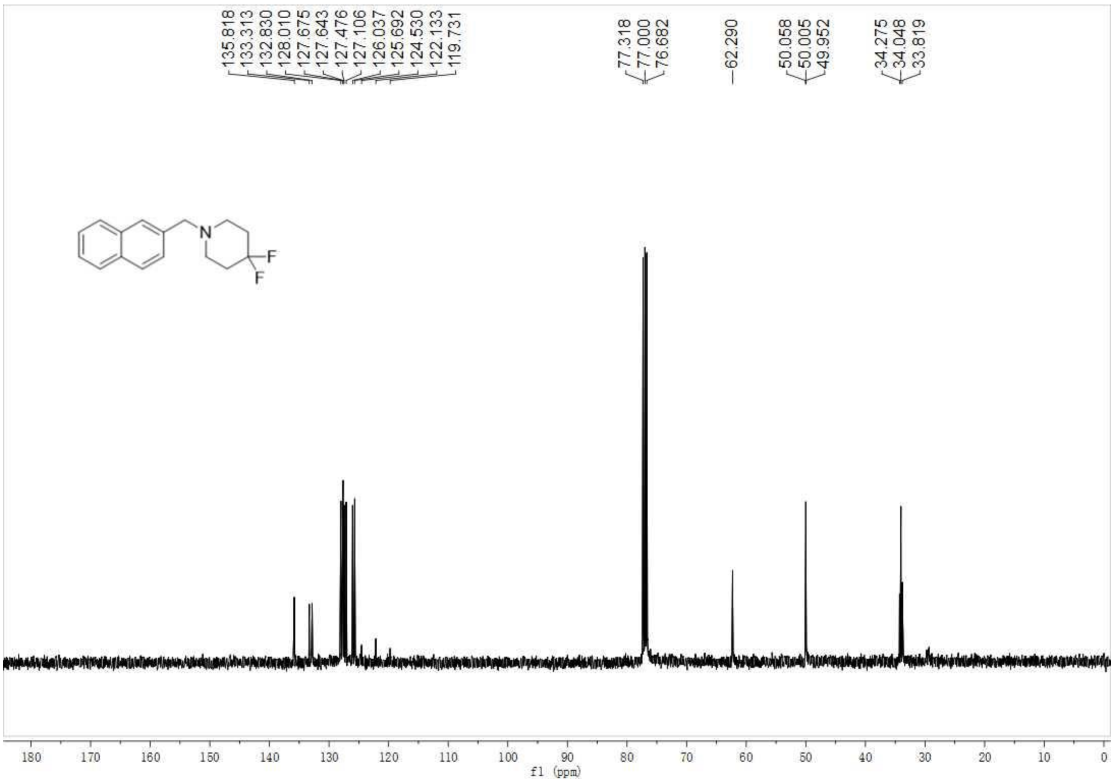

^{19}F NMR Spectrum of 3**1**

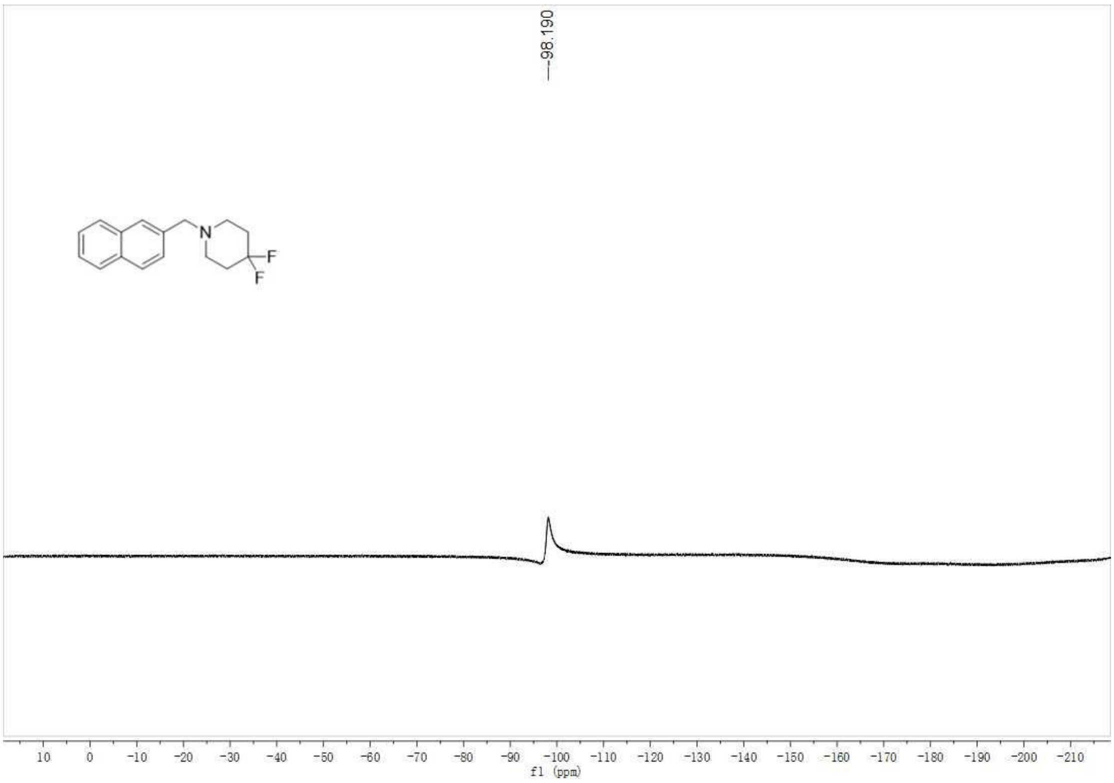

^{1}H NMR Spectrum of **32**

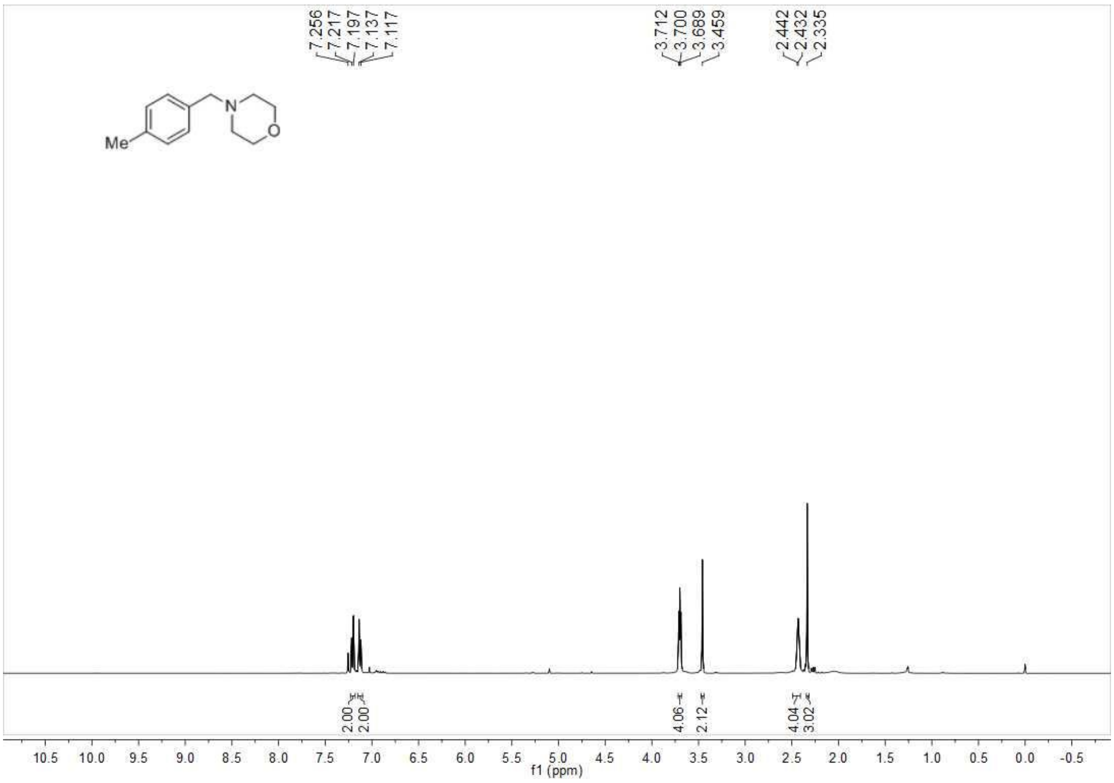

^{13}C NMR Spectrum of **32**

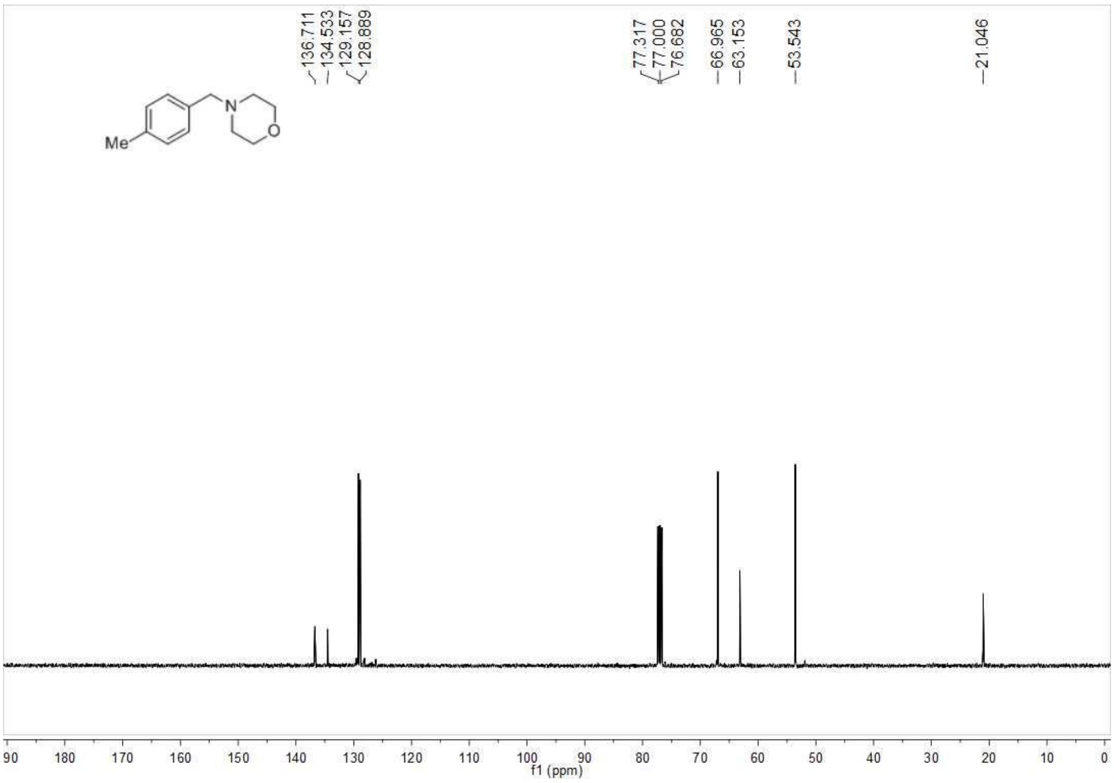

^{1}H NMR Spectrum of **33**

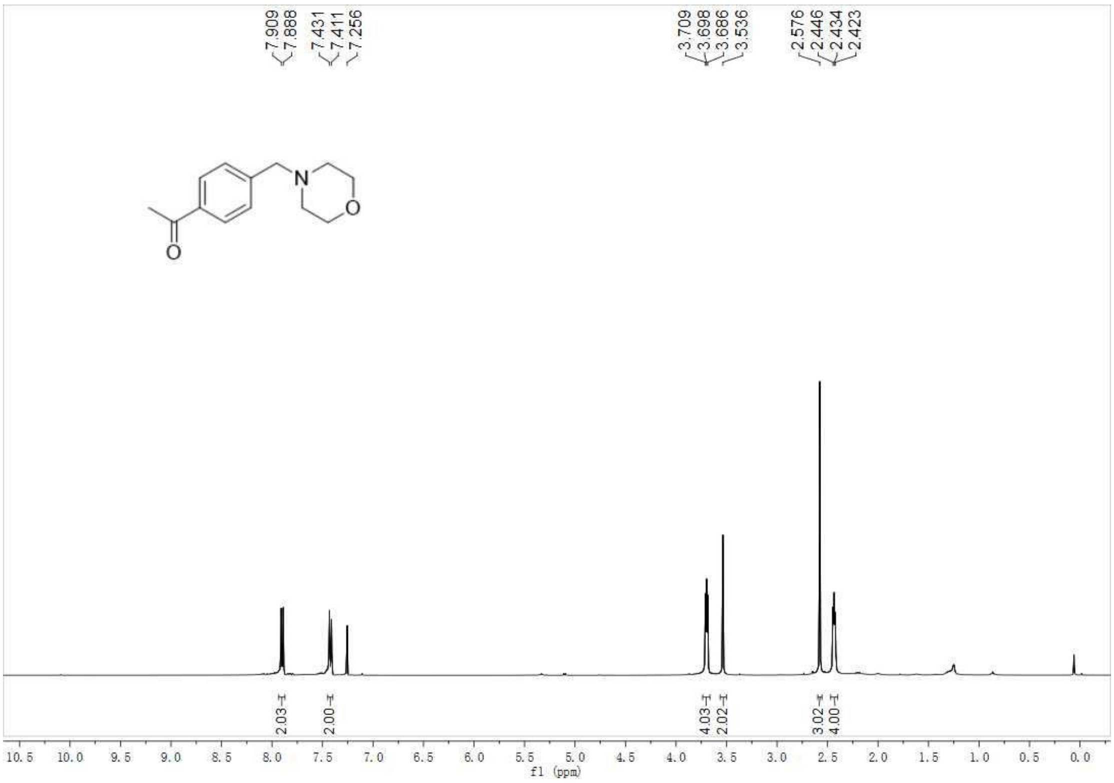

^{13}C NMR Spectrum of **33**

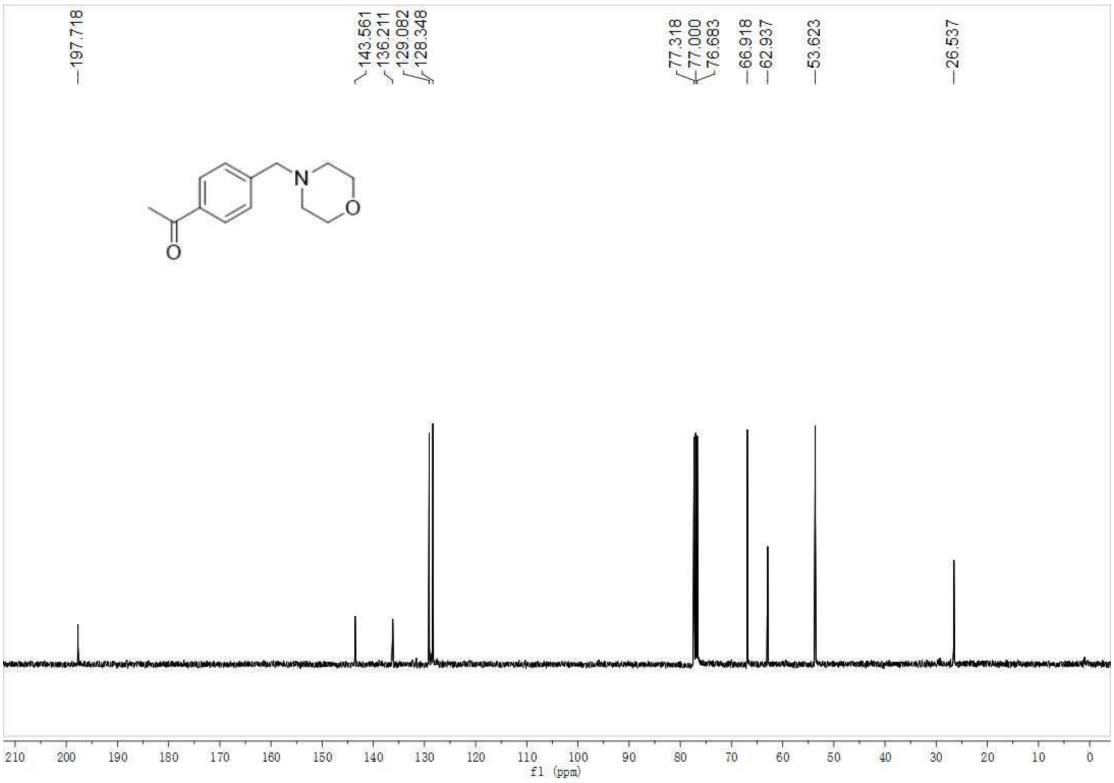

^{1}H NMR Spectrum of **34**

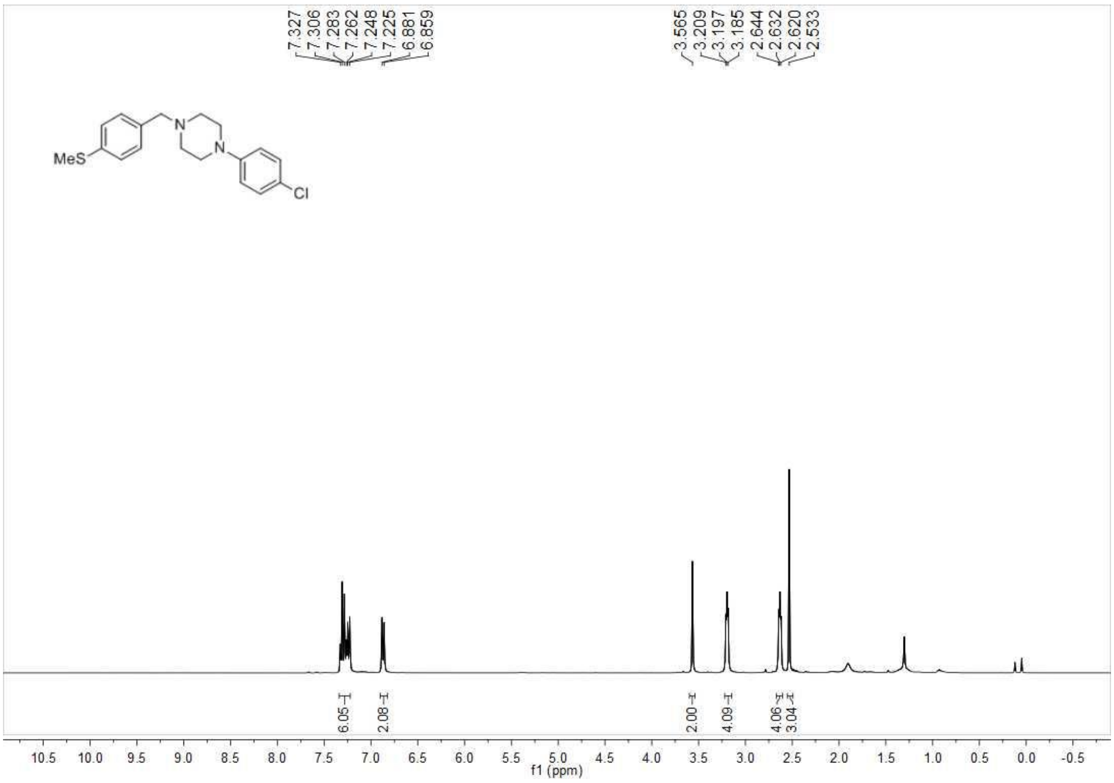

^{13}C NMR Spectrum of **34**

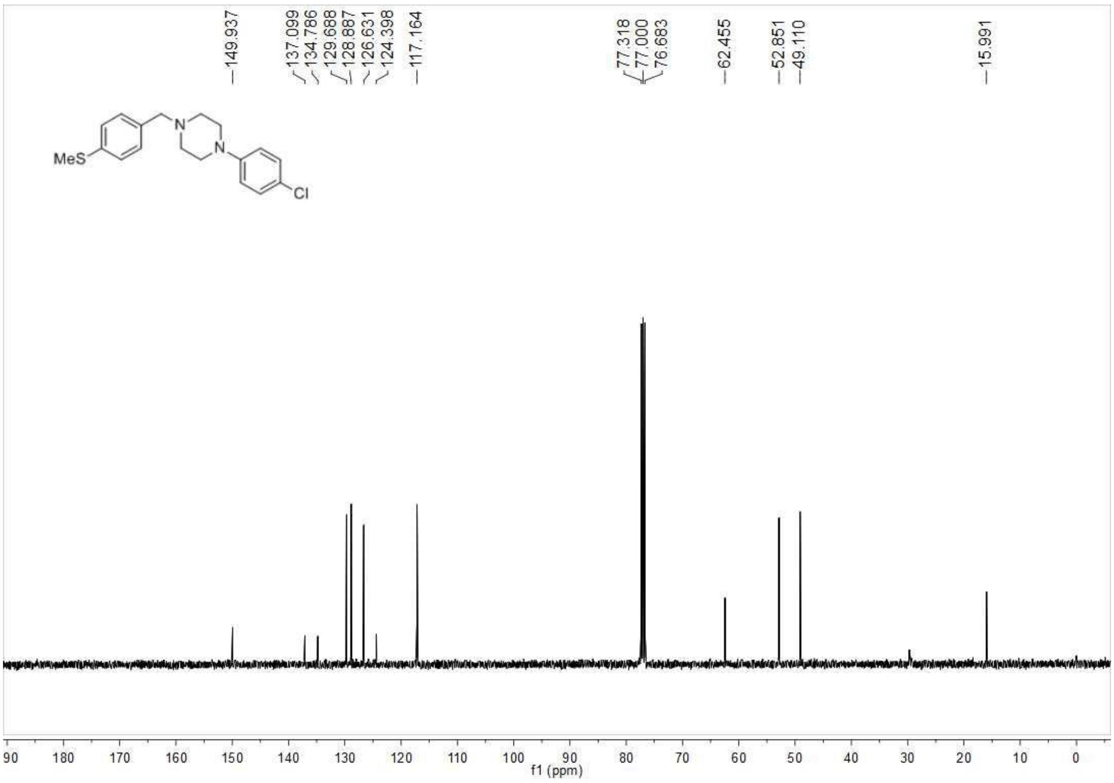

^{1}H NMR Spectrum of **35**

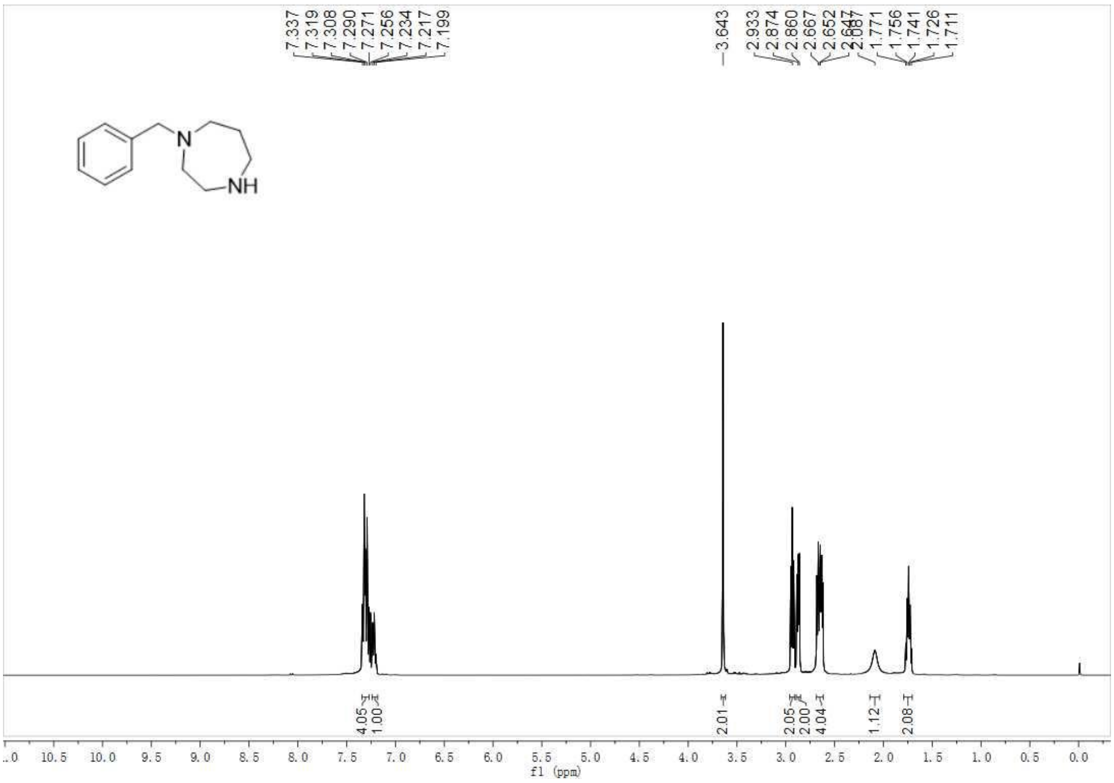

^{13}C NMR Spectrum of **35**

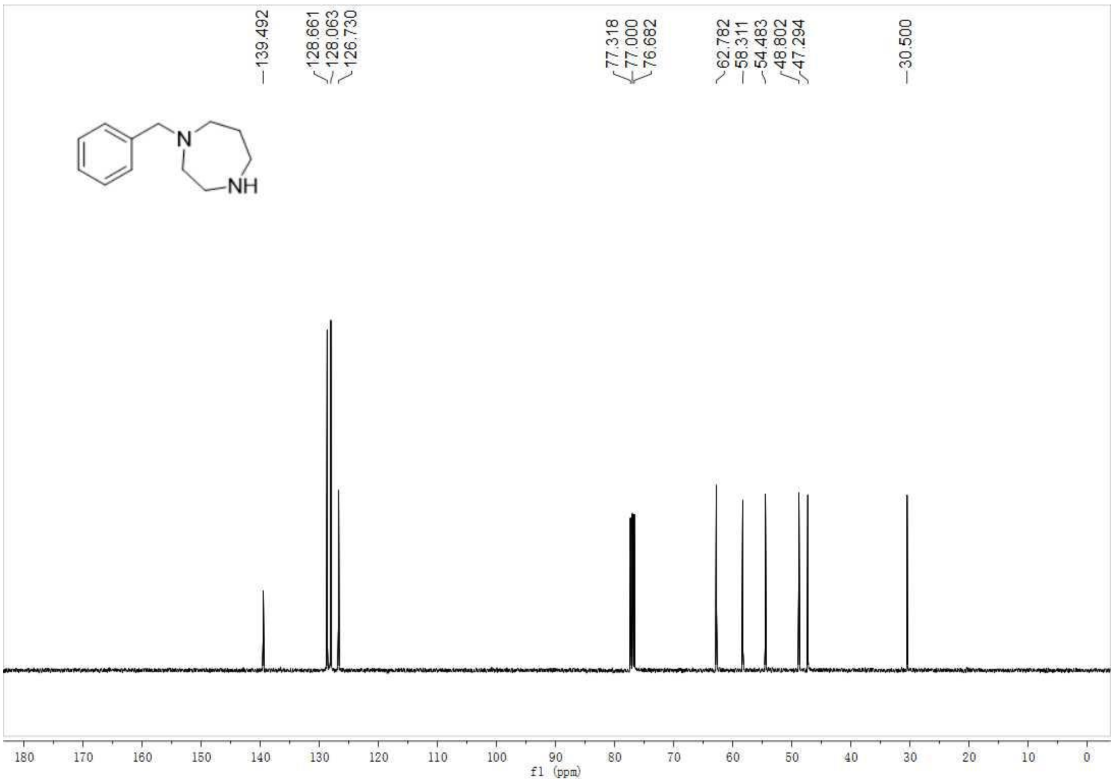

^{1}H NMR Spectrum of **36**

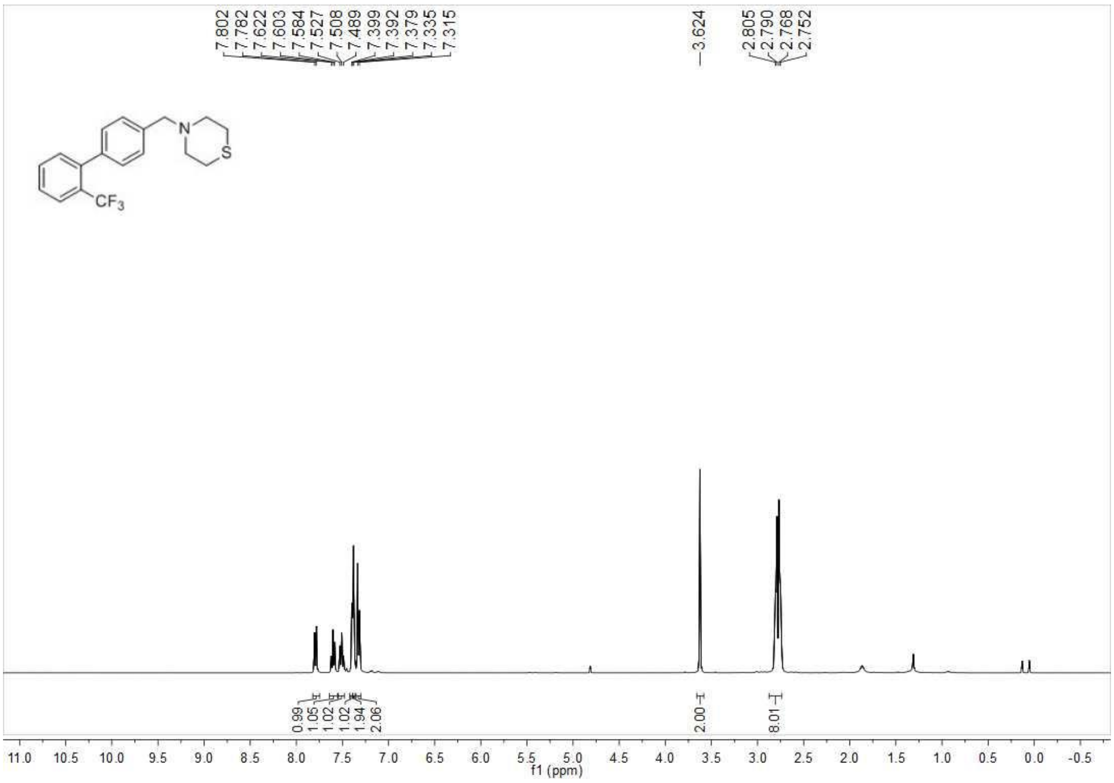

^{13}C NMR Spectrum of **36**

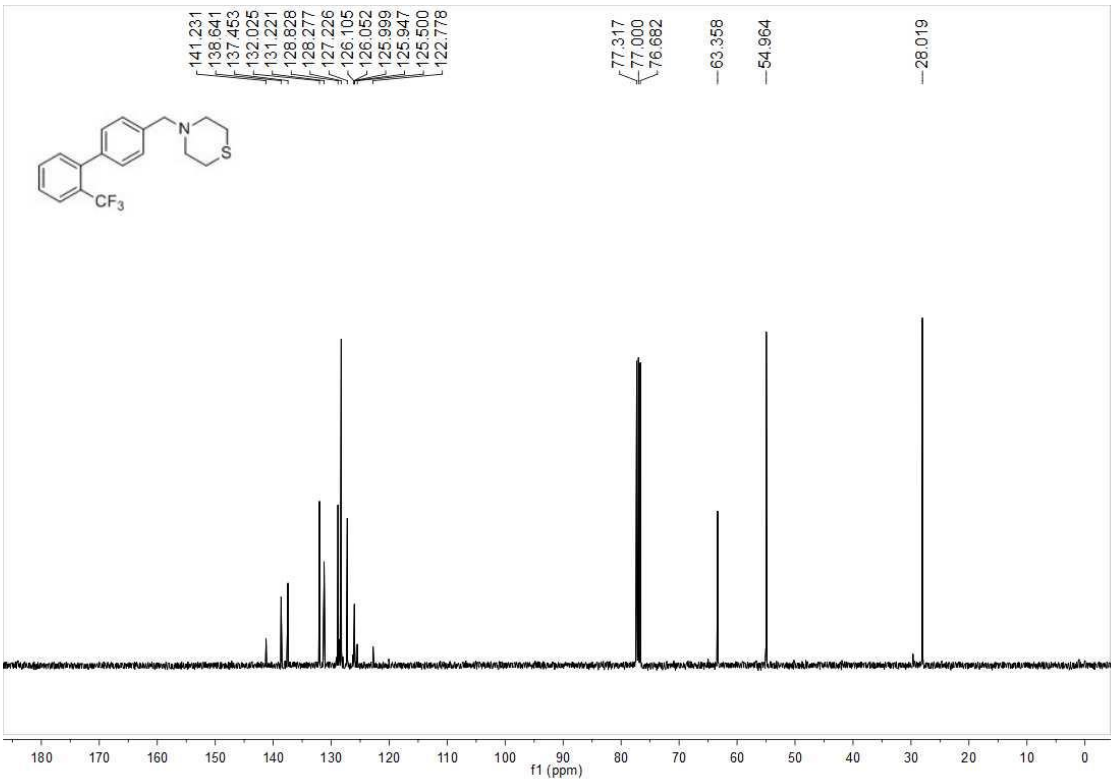

^{19}F NMR Spectrum of **36**

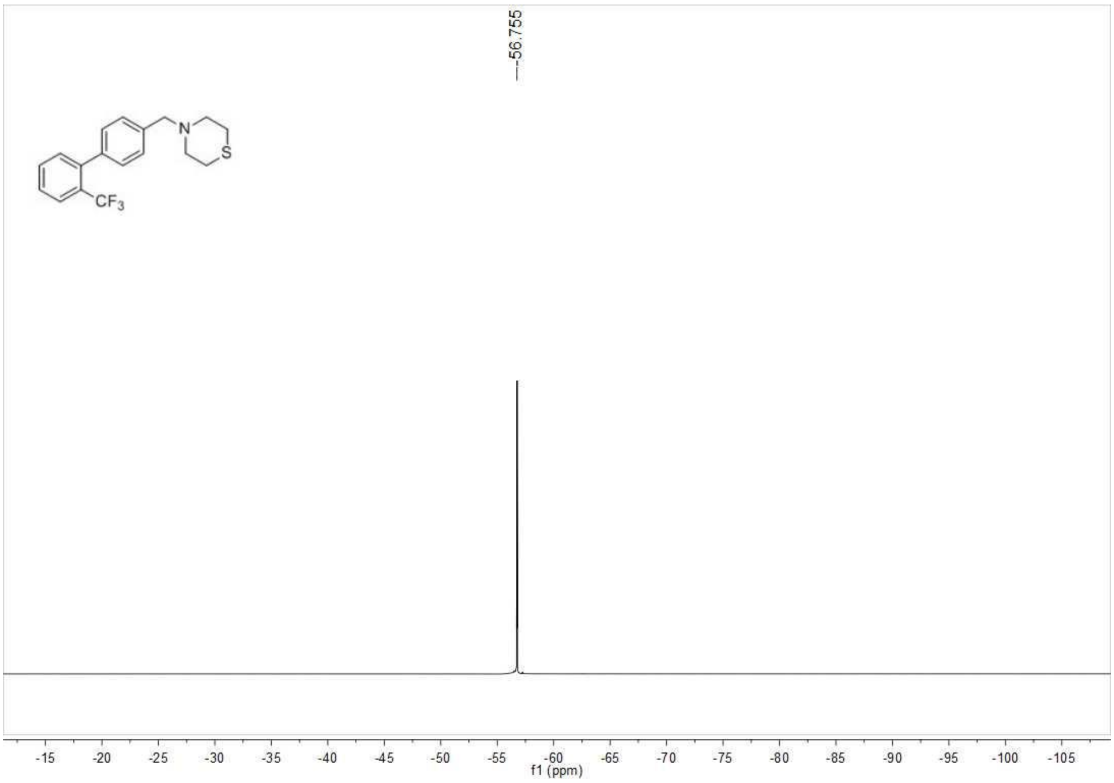

^{1}H NMR Spectrum of **37**

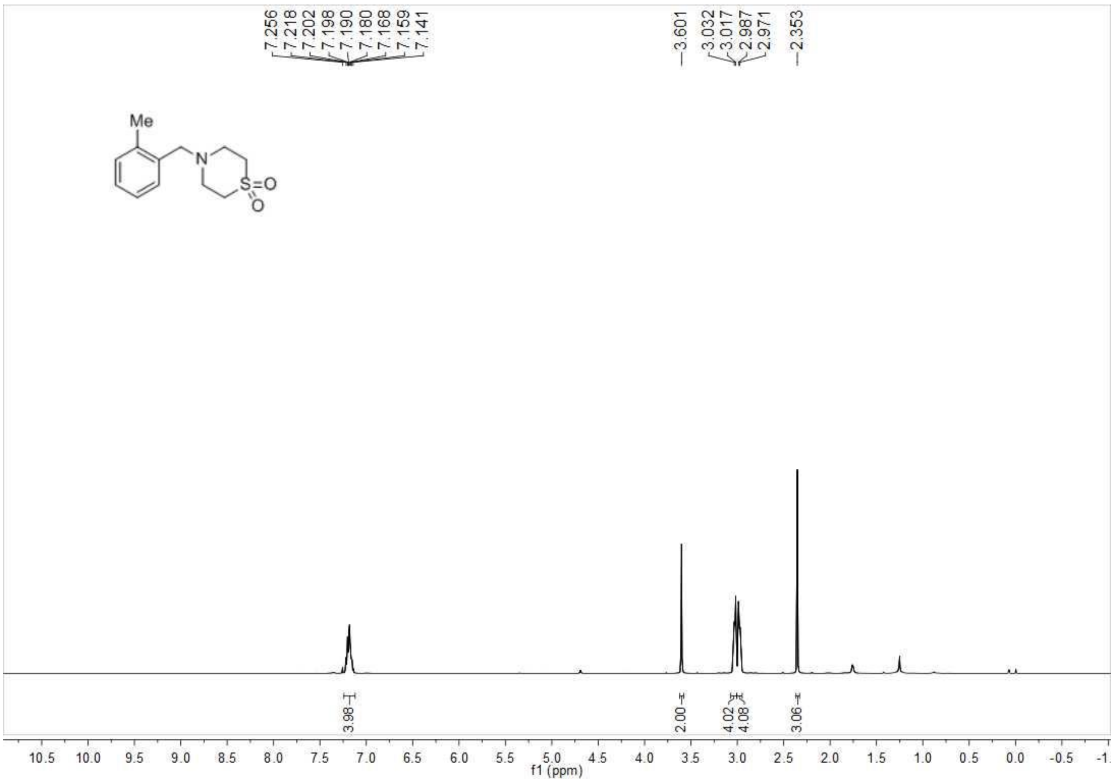

^{13}C NMR Spectrum of **37**

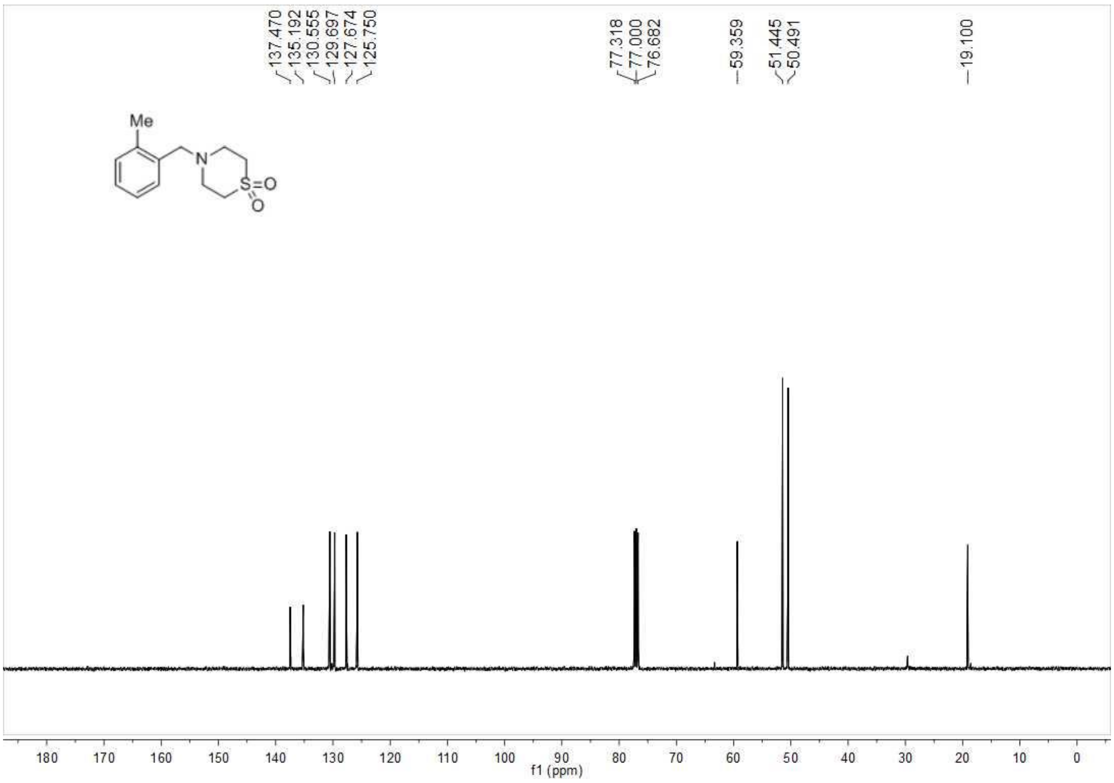

^{1}H NMR Spectrum of **38**

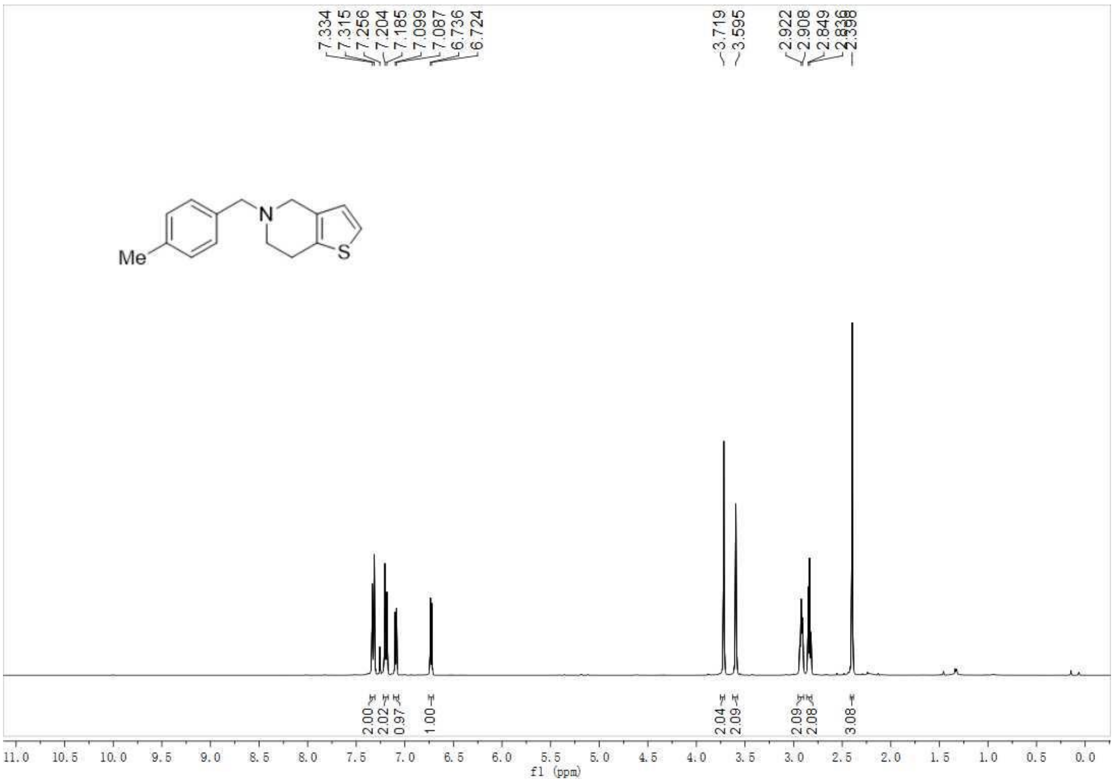

^{13}C NMR Spectrum of **38**

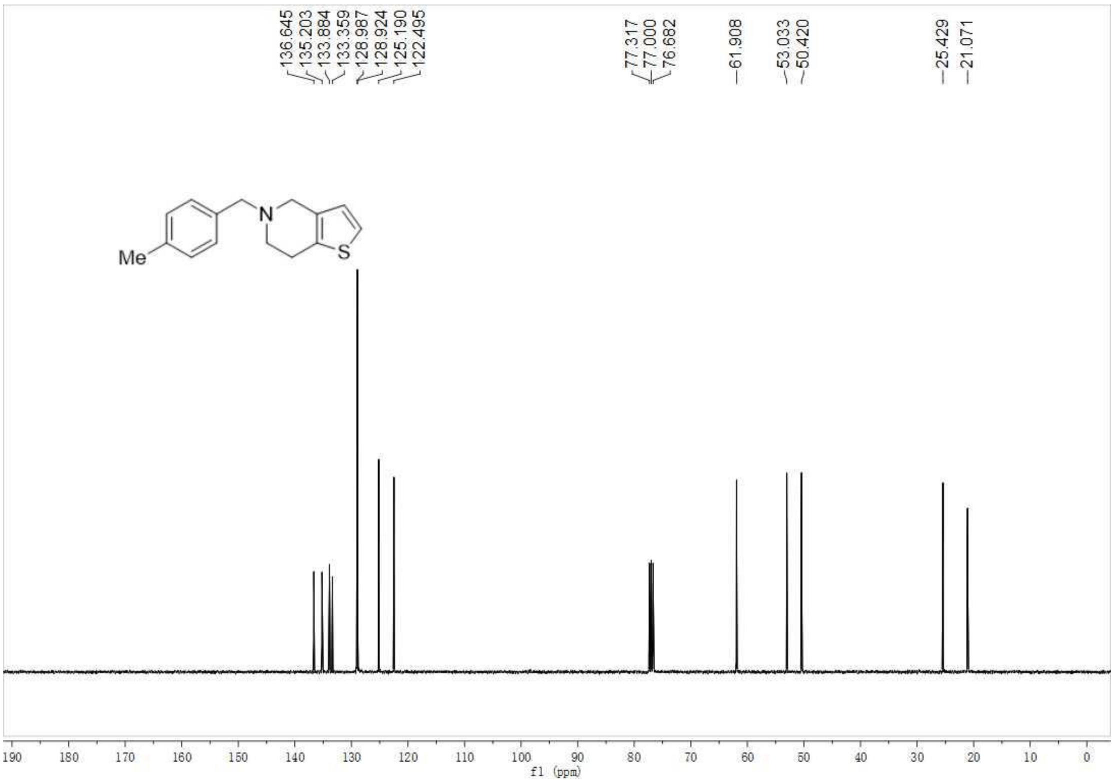

^{1}H NMR Spectrum of **39**

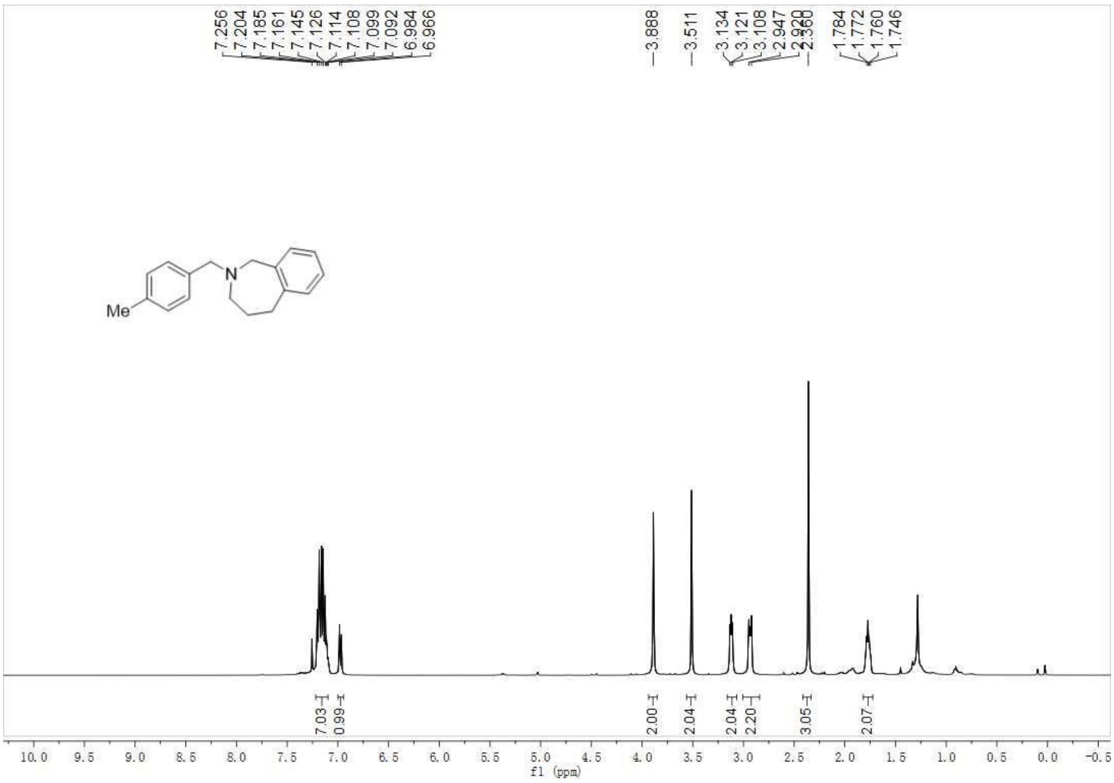

^{13}C NMR Spectrum of **39**

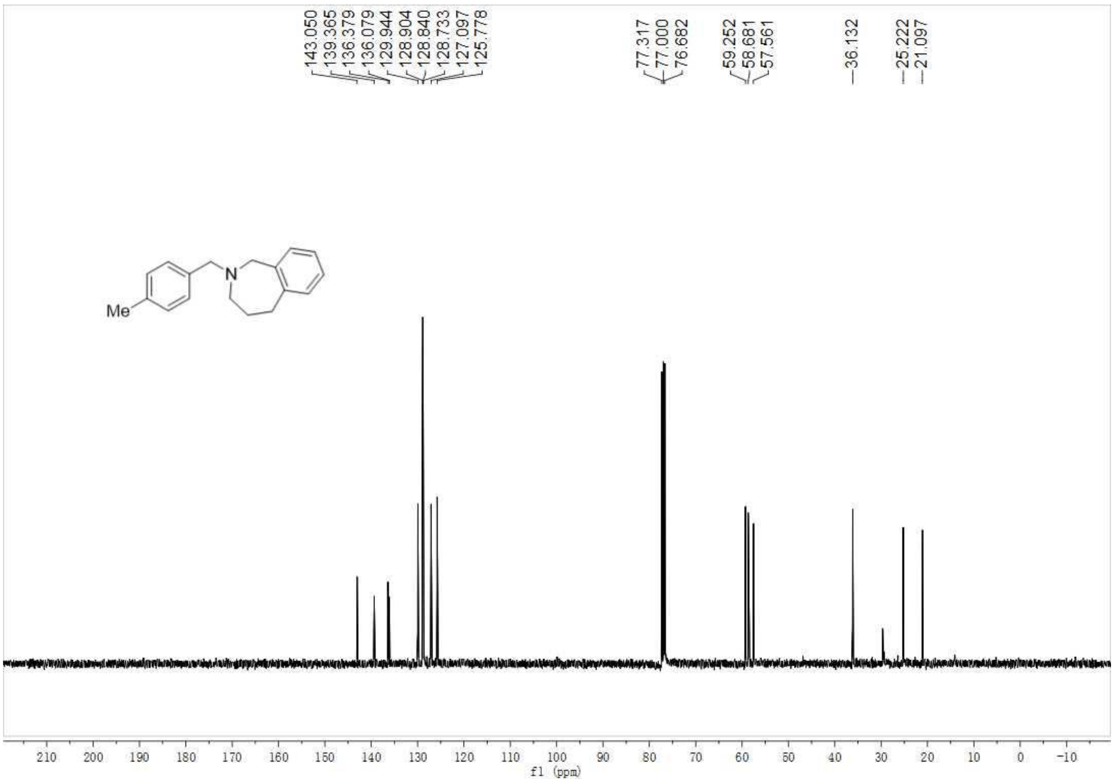

^{1}H NMR Spectrum of **40**

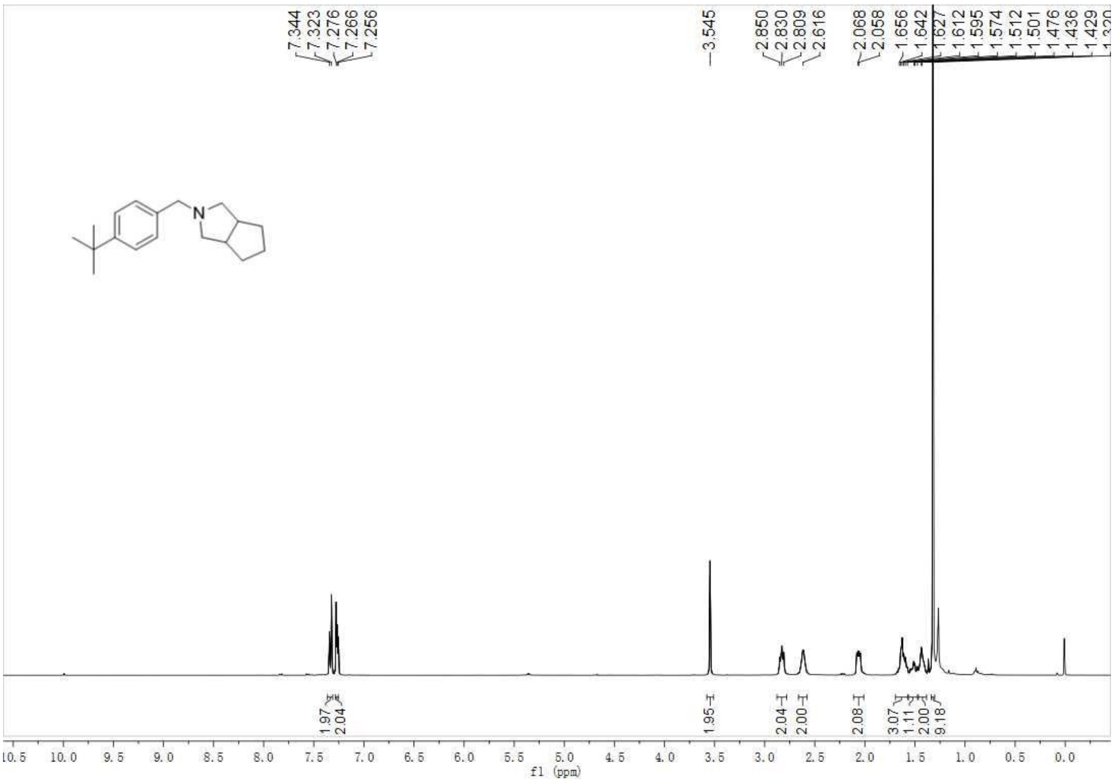

^{13}C NMR Spectrum of **40**

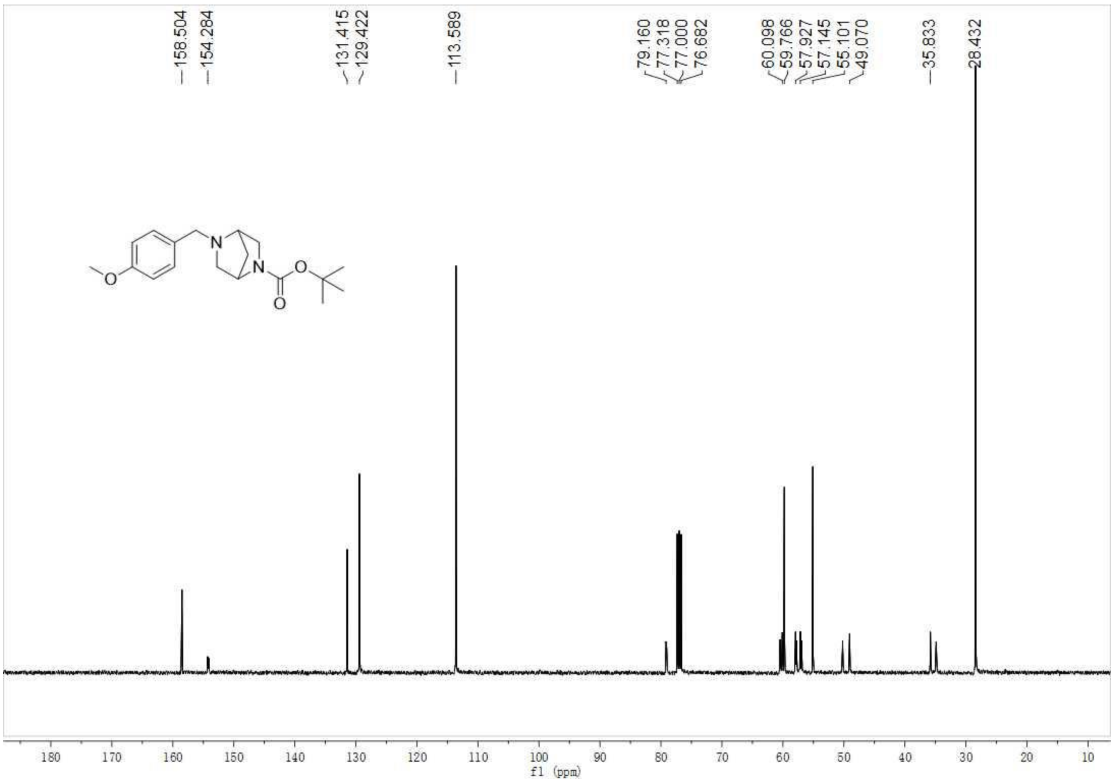

^{1}H NMR Spectrum of 4**1**

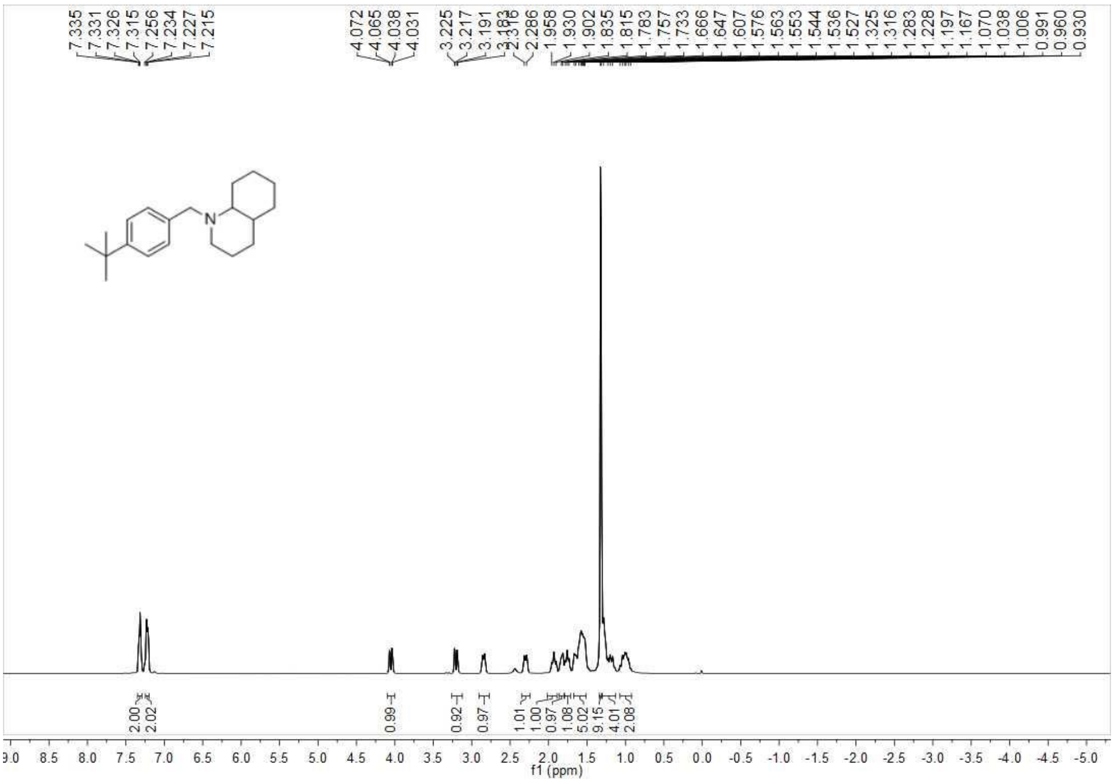

^{13}C NMR Spectrum of 4**1**

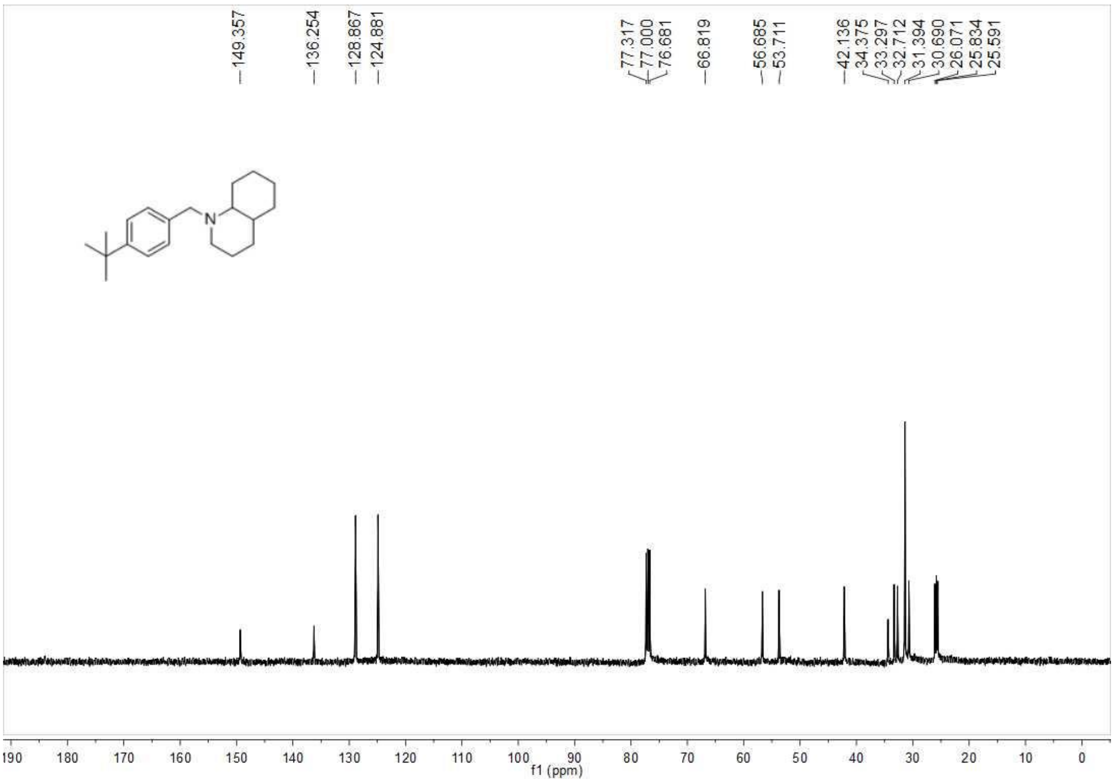

^{1}H NMR Spectrum of **42**

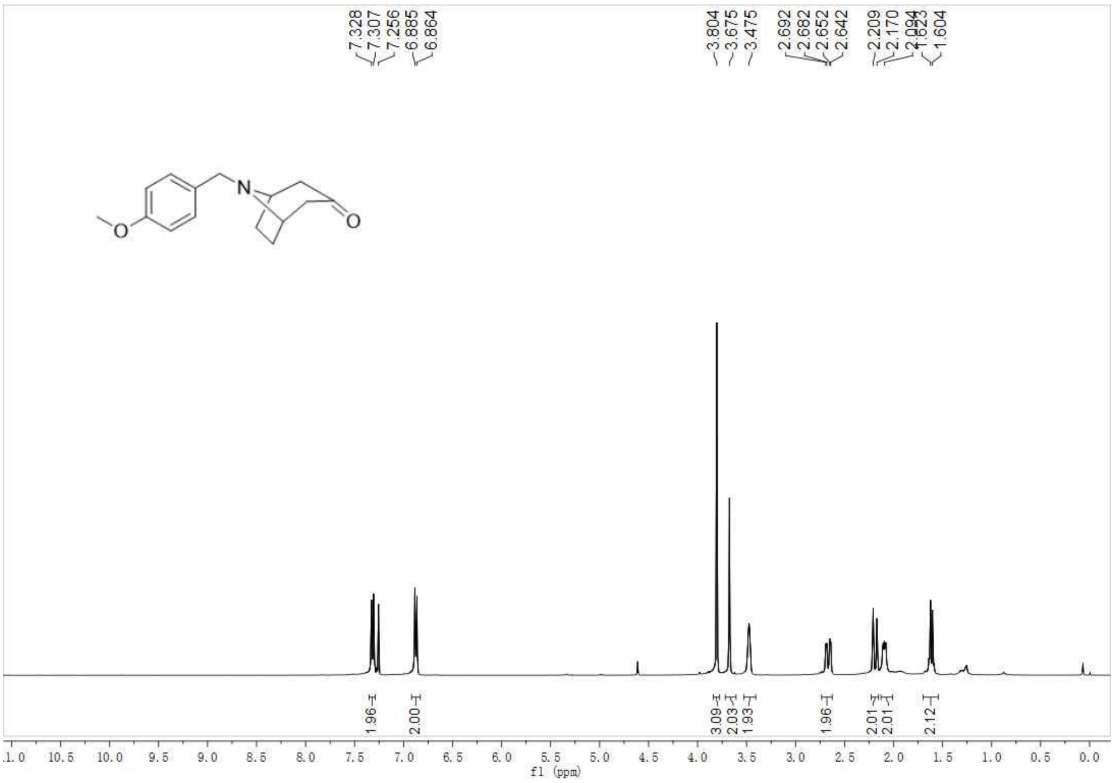

^{13}C NMR Spectrum of **42**

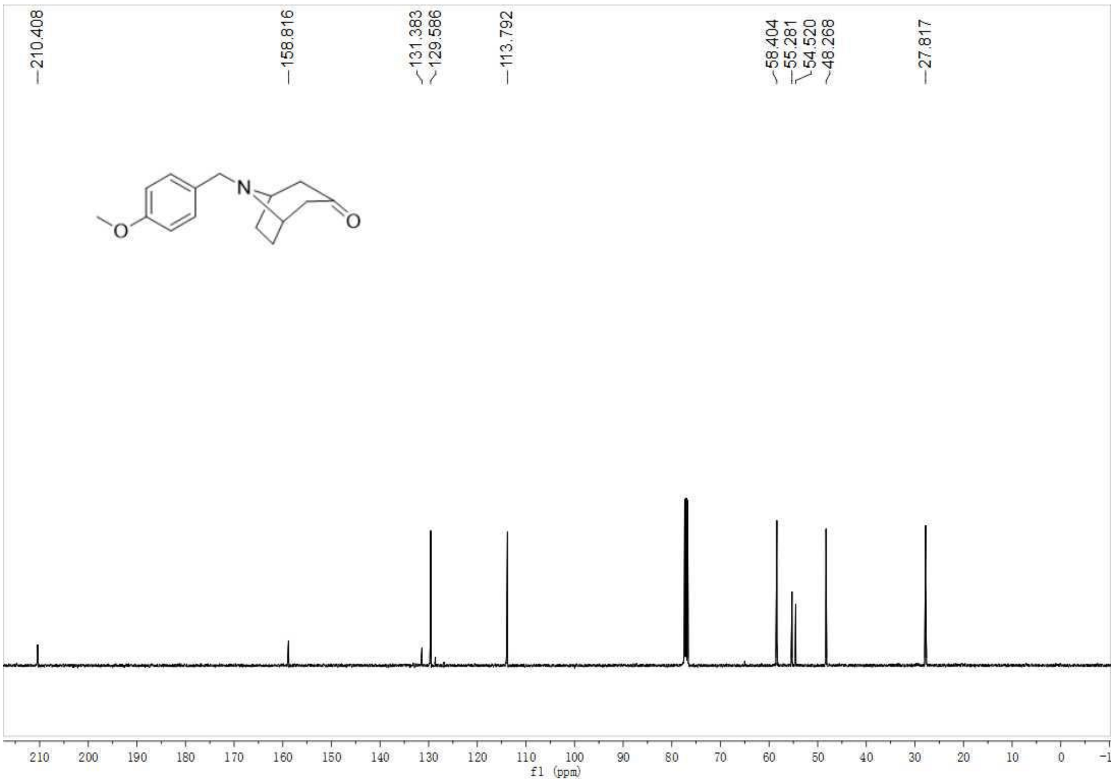

^{1}H NMR Spectrum of **43**

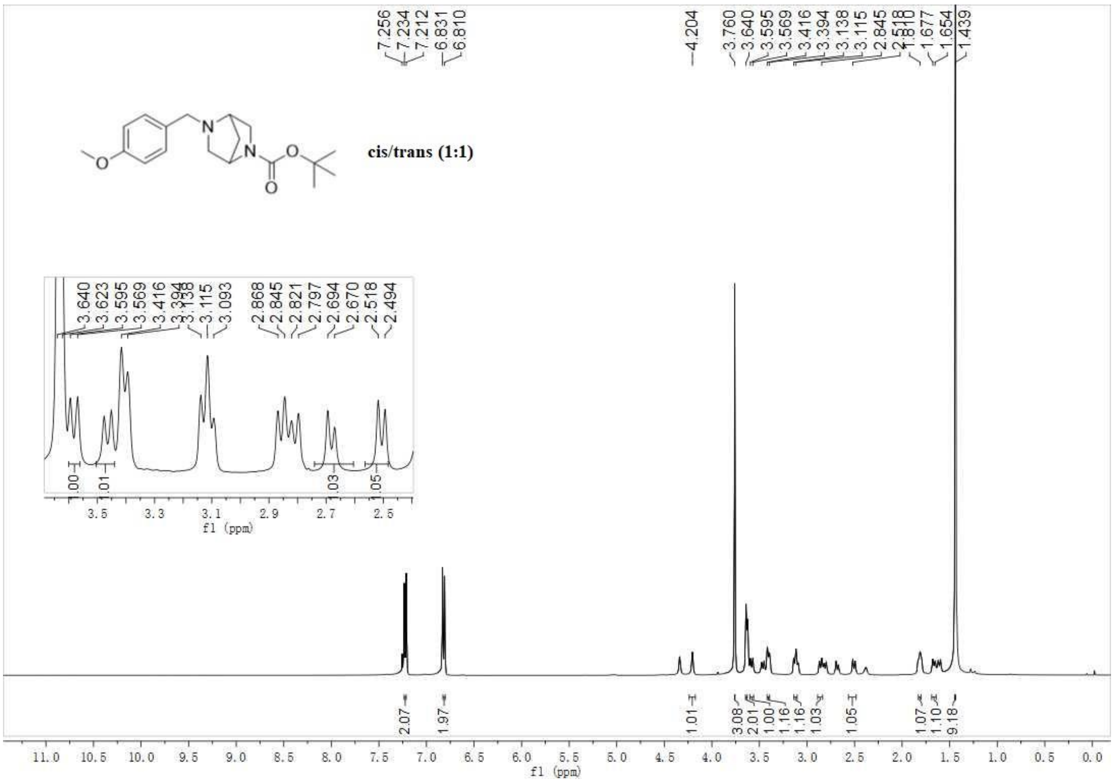

^{13}C NMR Spectrum of **43**

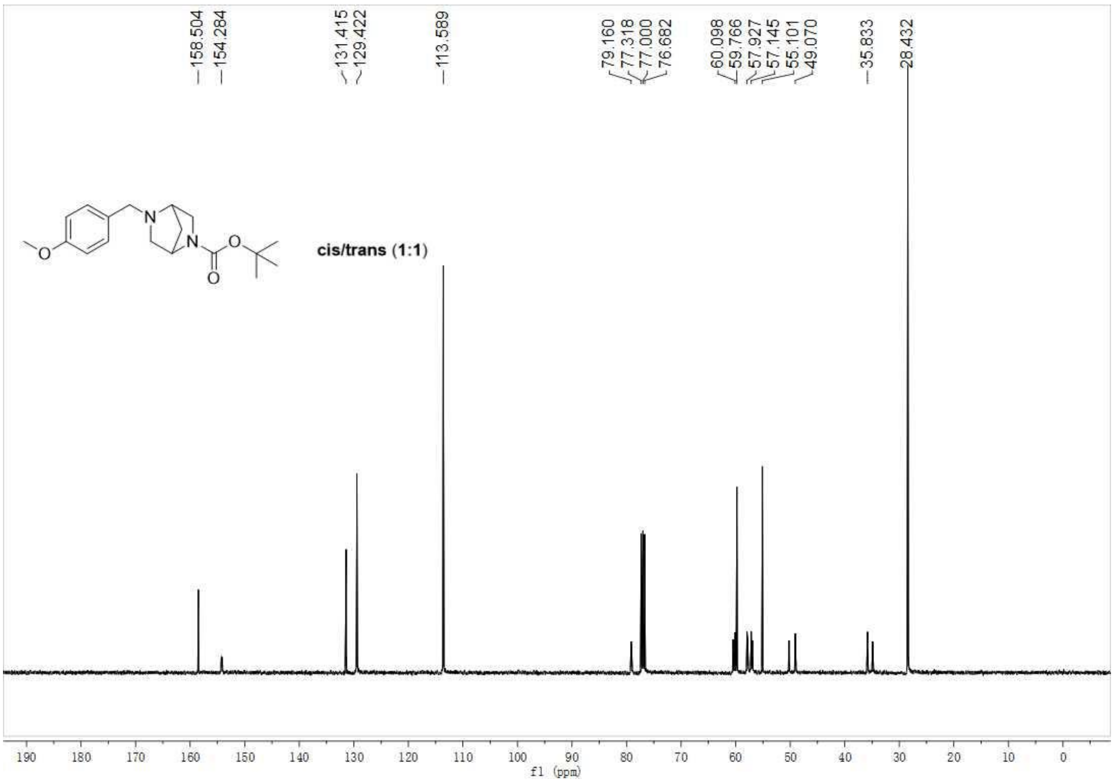

^{1}H NMR Spectrum of **44**

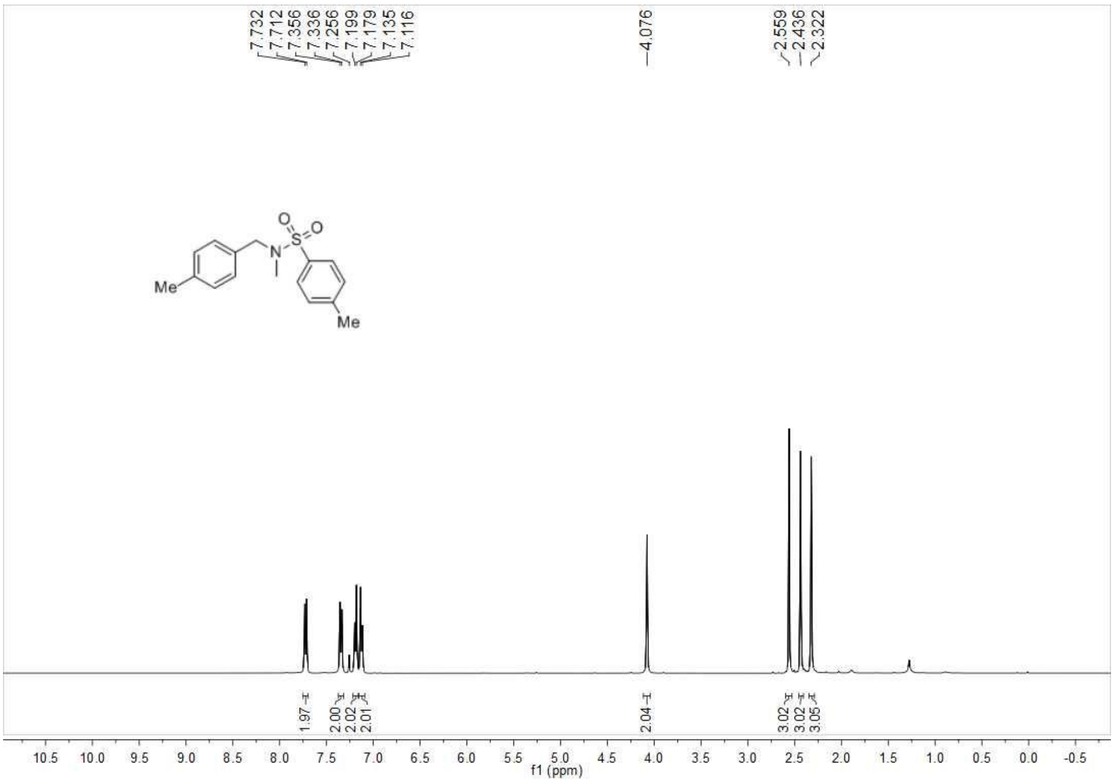

^{13}C NMR Spectrum of **44**

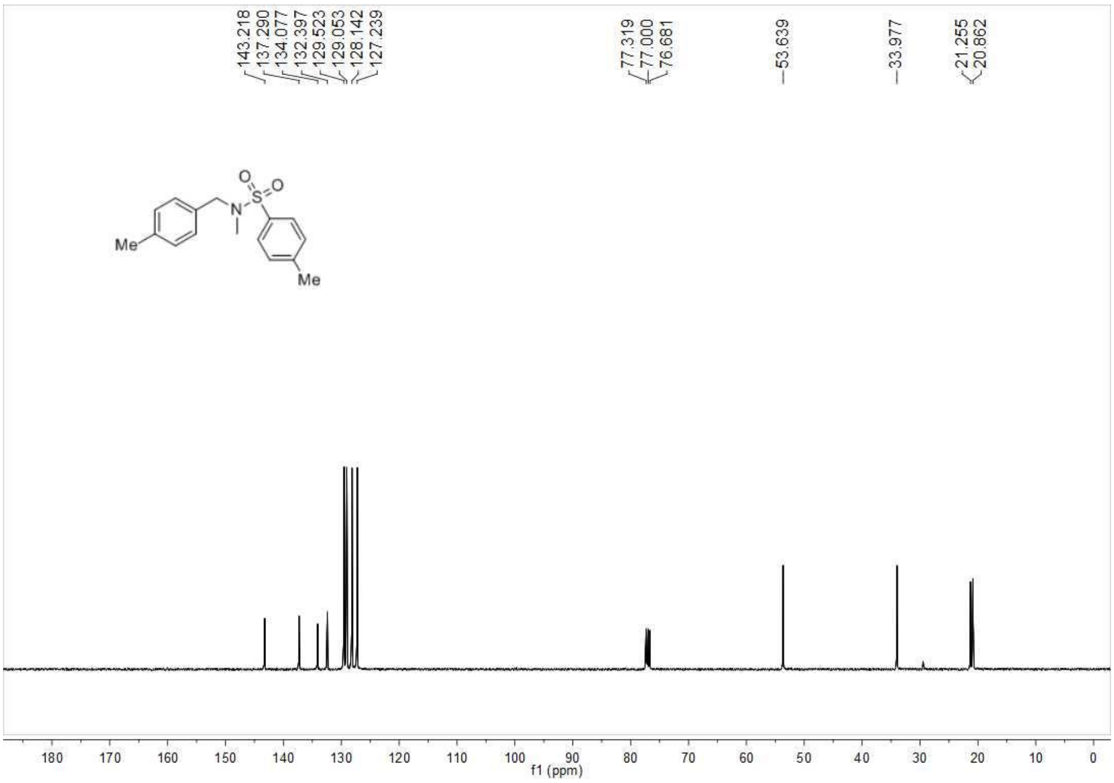

^{1}H NMR Spectrum of **45**

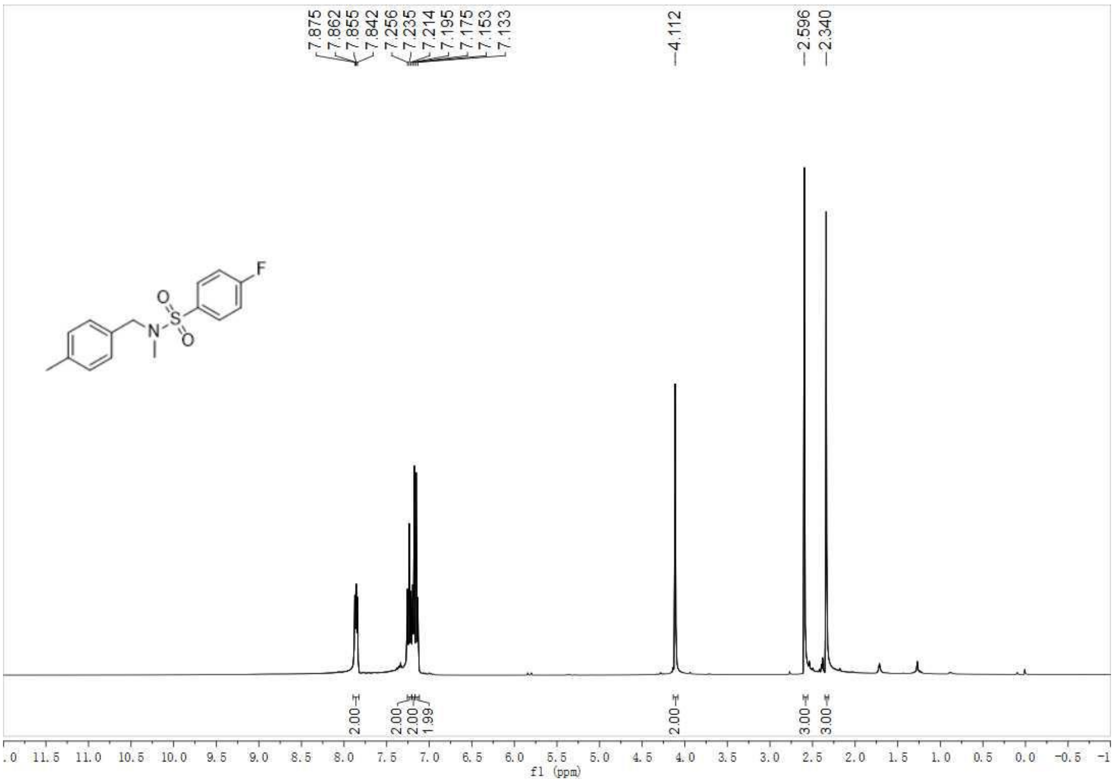

^{13}C NMR Spectrum of **45**

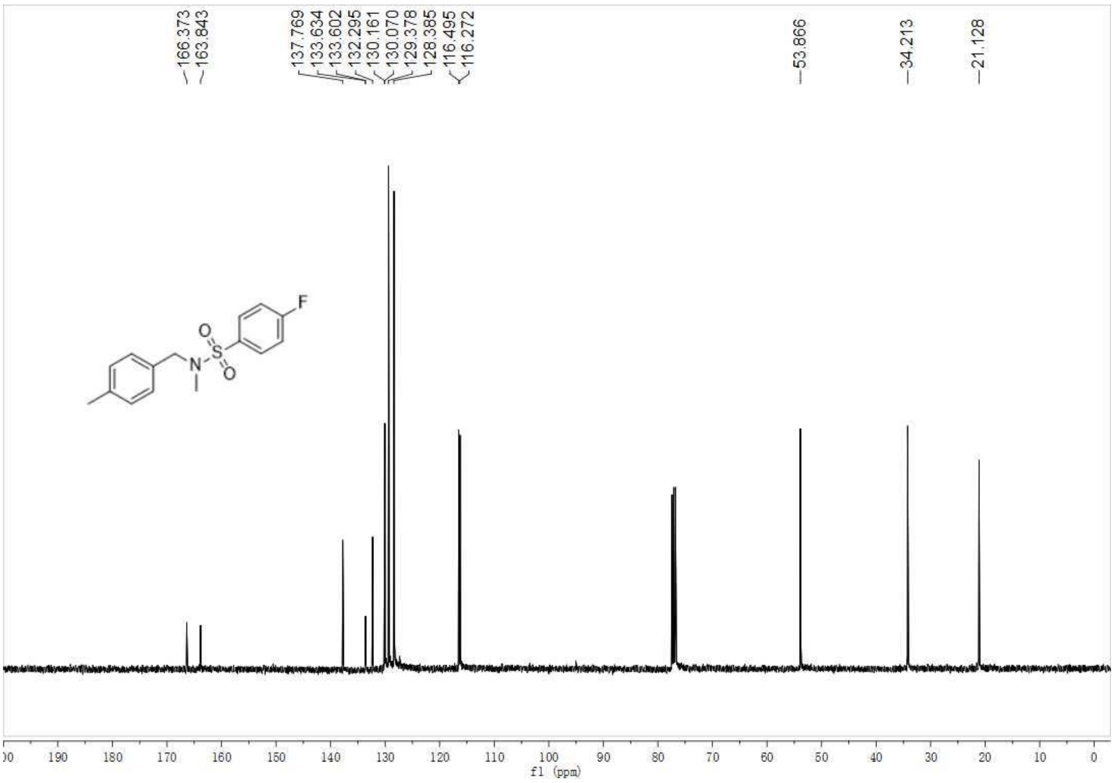

^{19}F NMR Spectrum of **45**

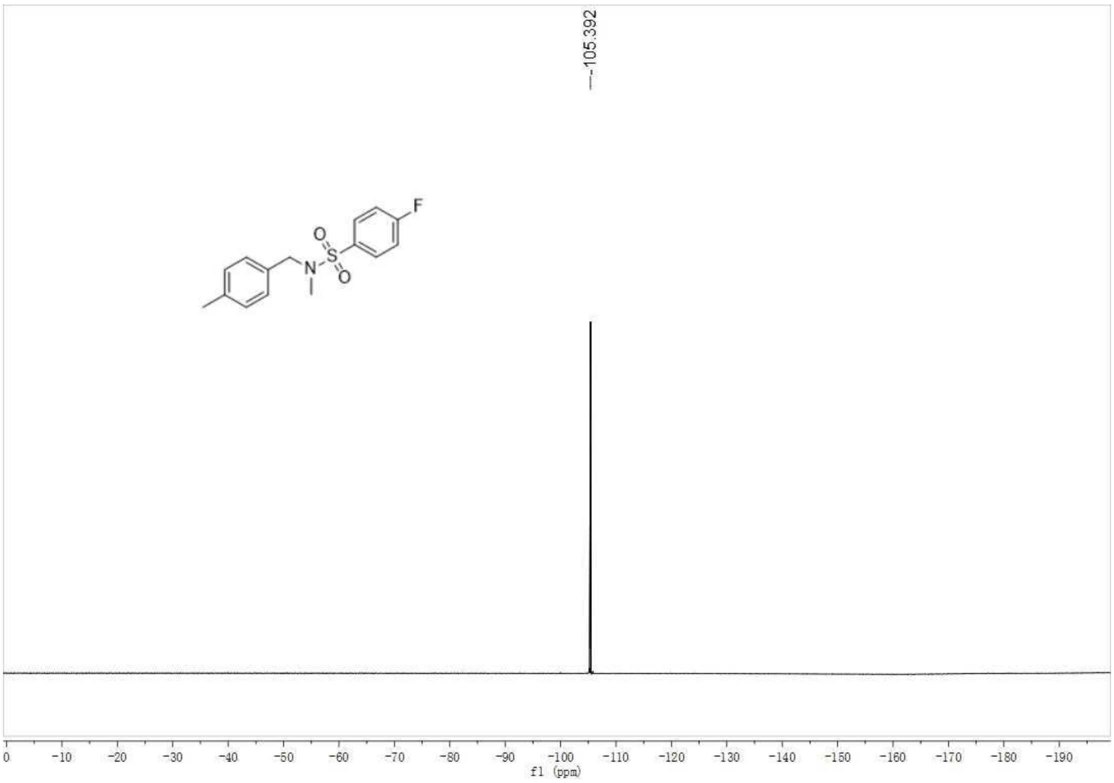

^{1}H NMR Spectrum of **46**

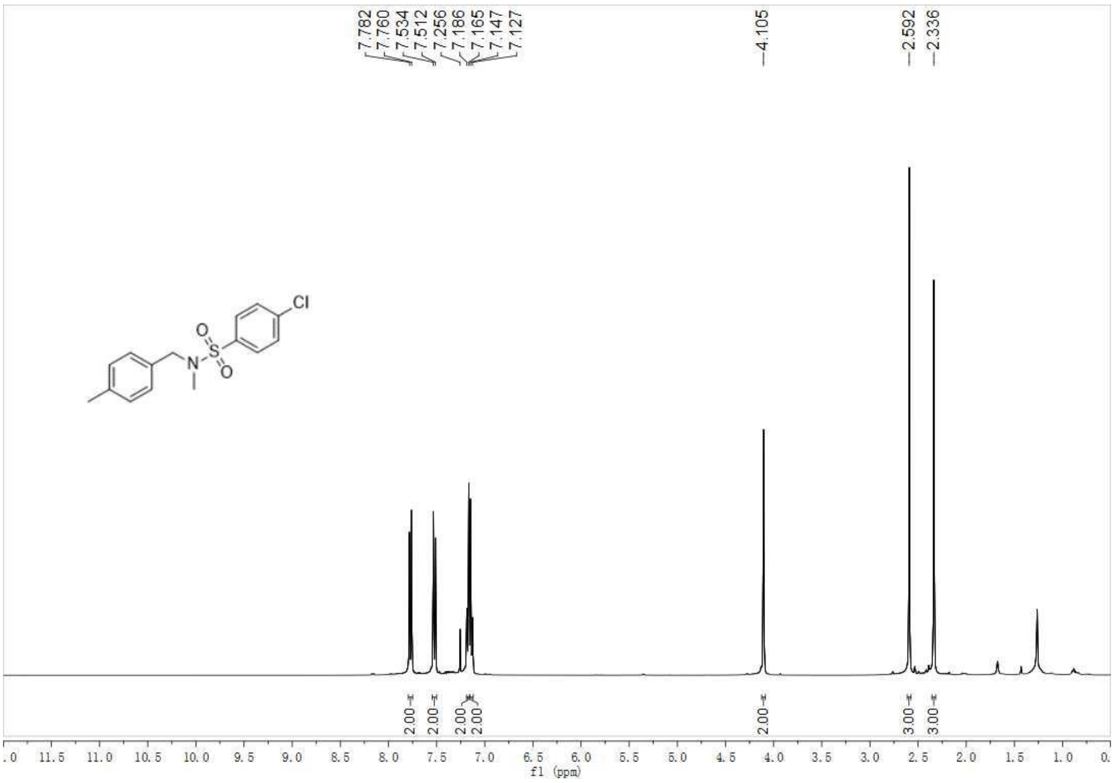

^{13}C NMR Spectrum of **46**

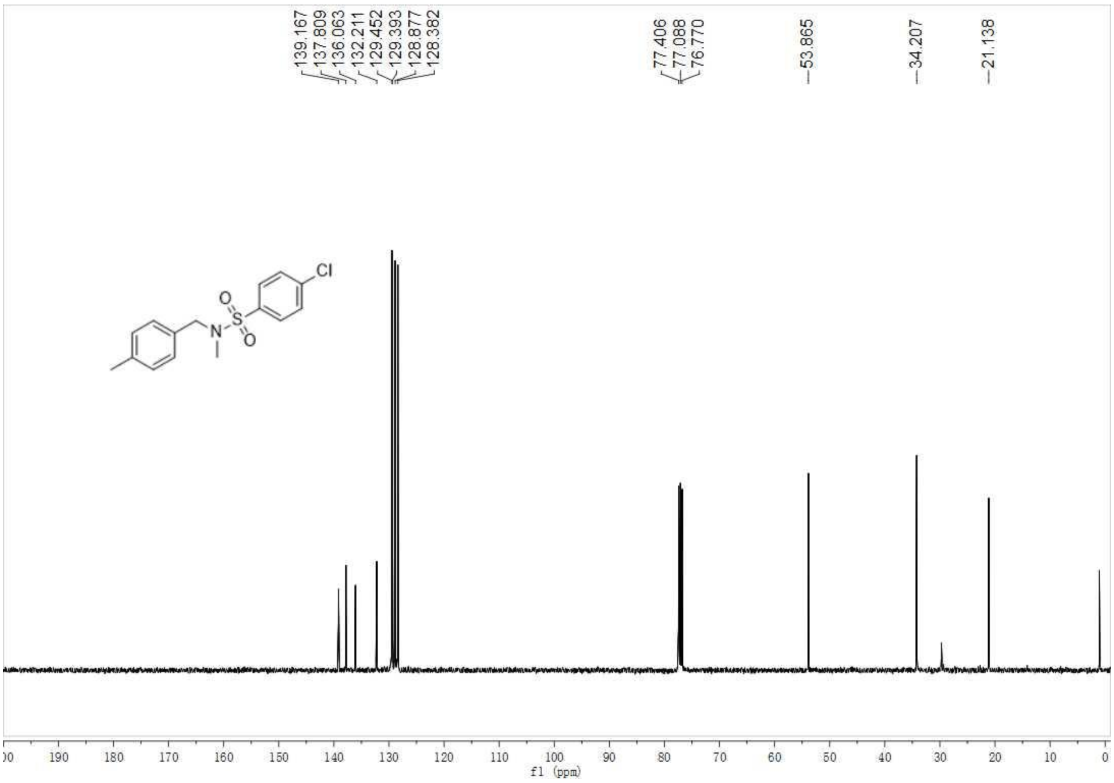

^{1}H NMR Spectrum of **47**

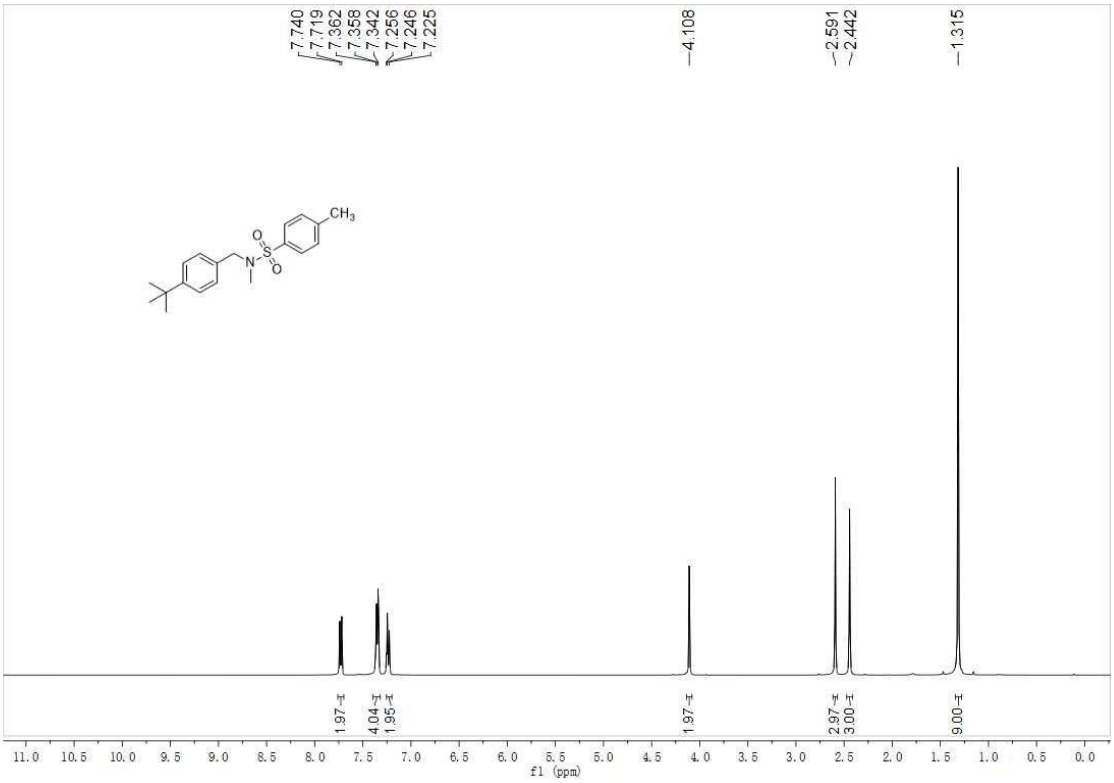

^{13}C NMR Spectrum of **47**

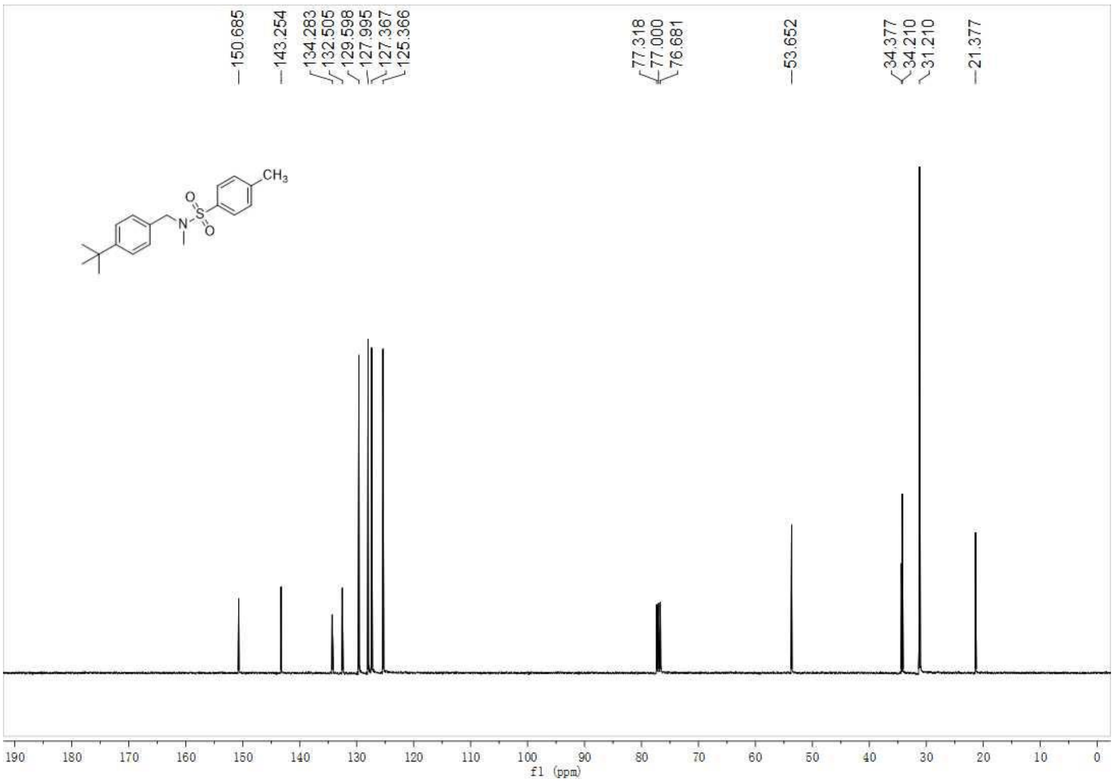

^{1}H NMR Spectrum of **48**

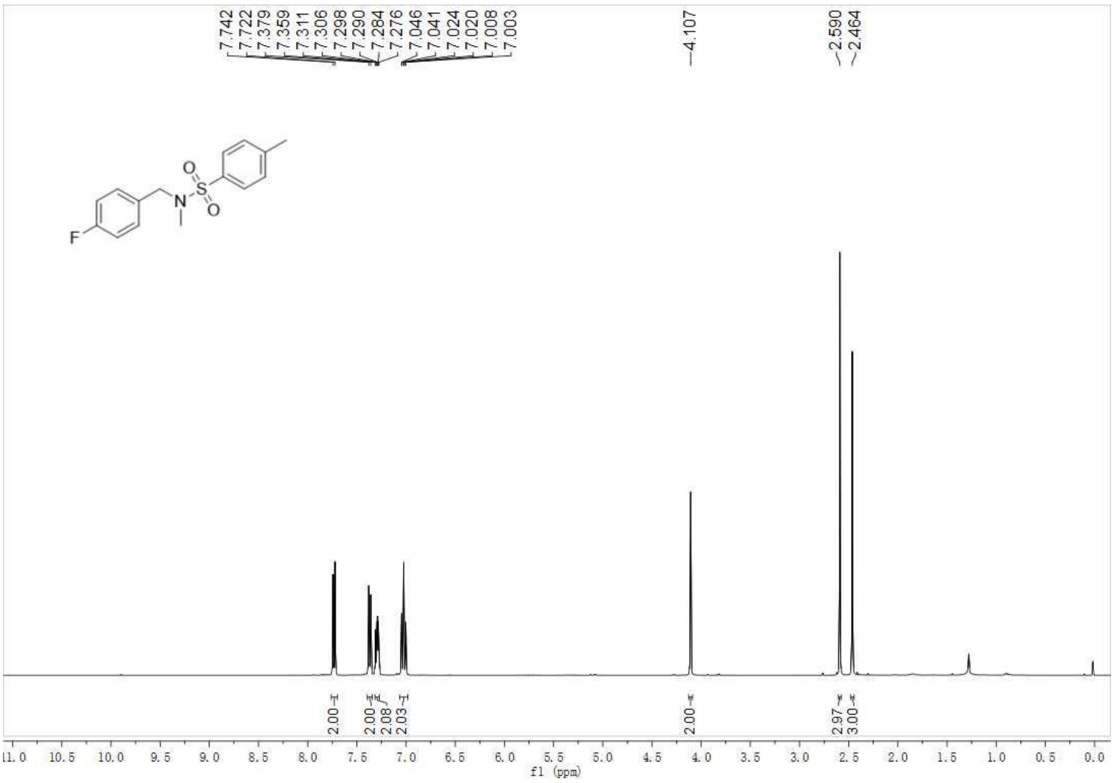

^{13}C NMR Spectrum of **48**

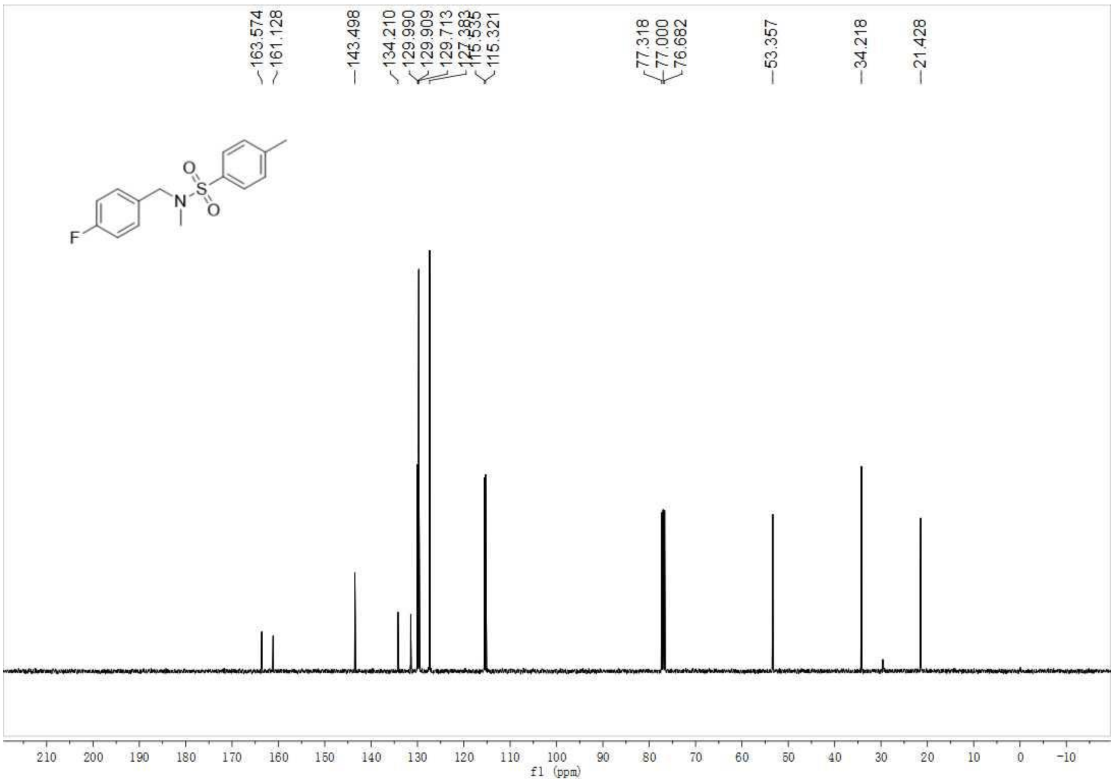

^{19}F NMR Spectrum of **48**

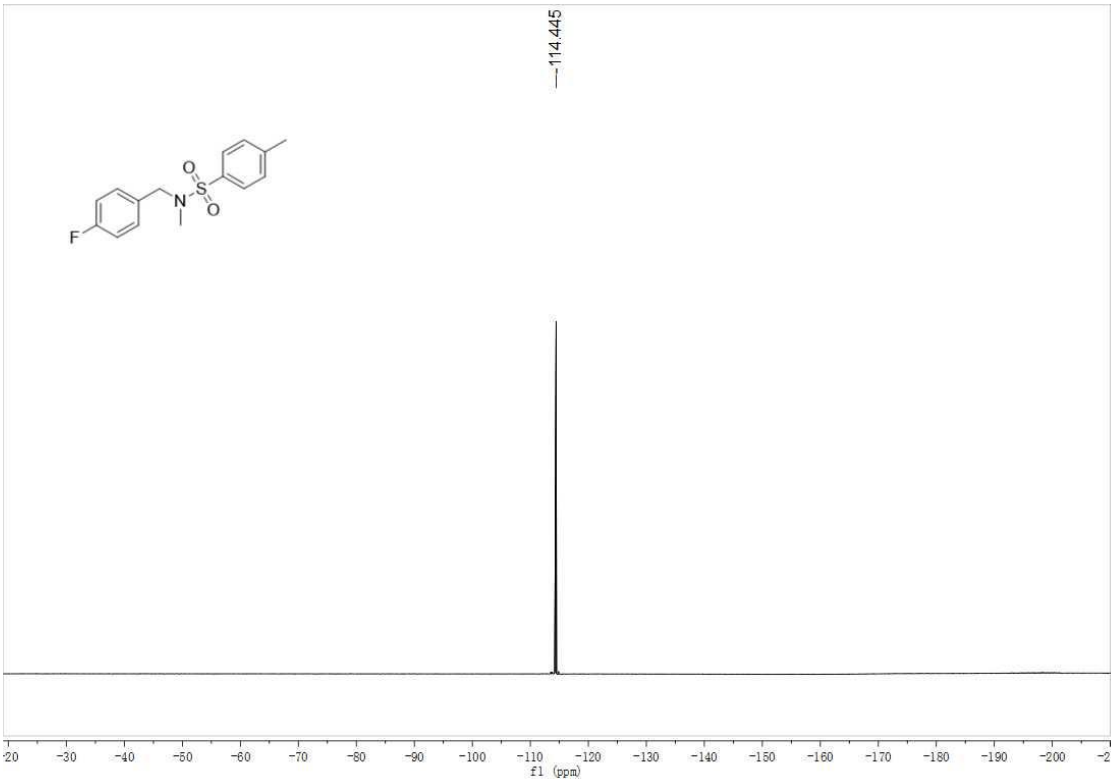

^{1}H NMR Spectrum of **49**

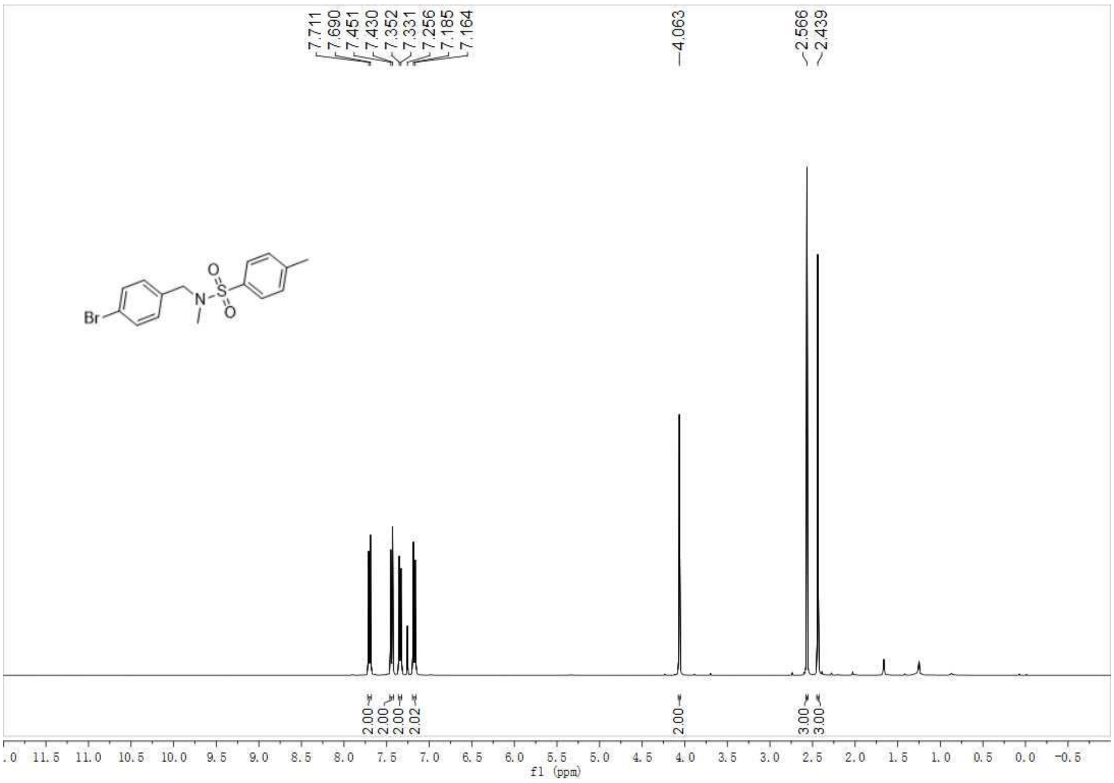

^{13}C NMR Spectrum of **49**

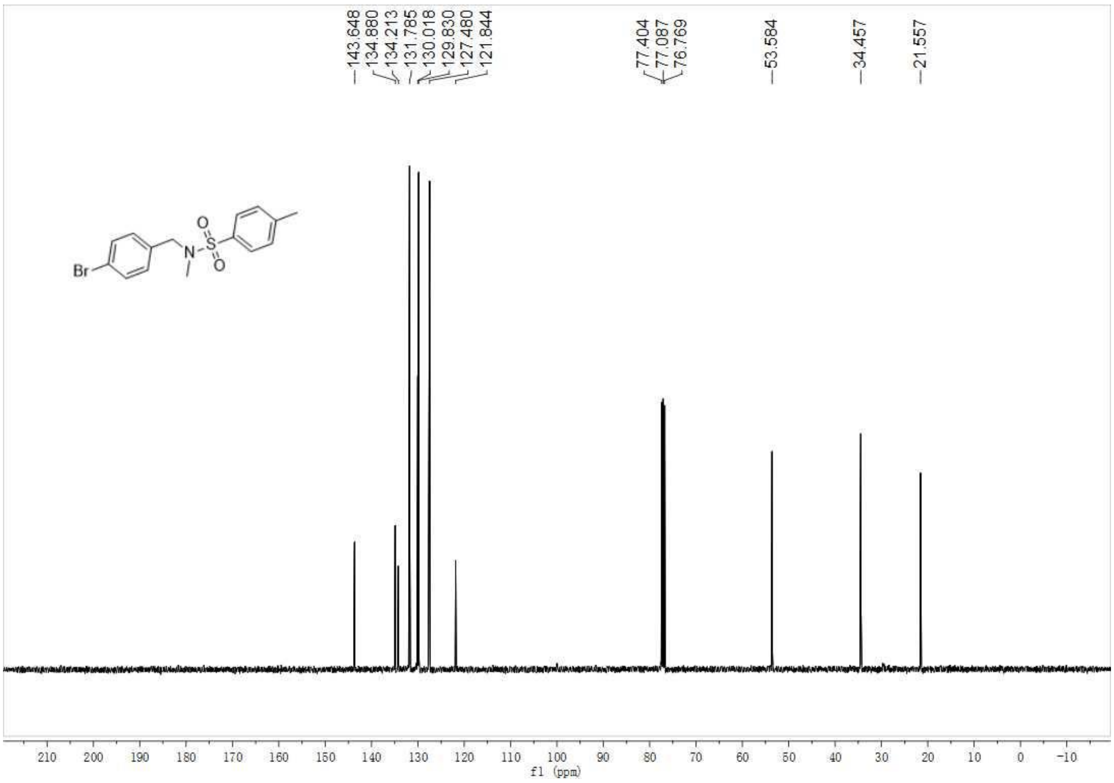

^{1}H NMR Spectrum of **50**

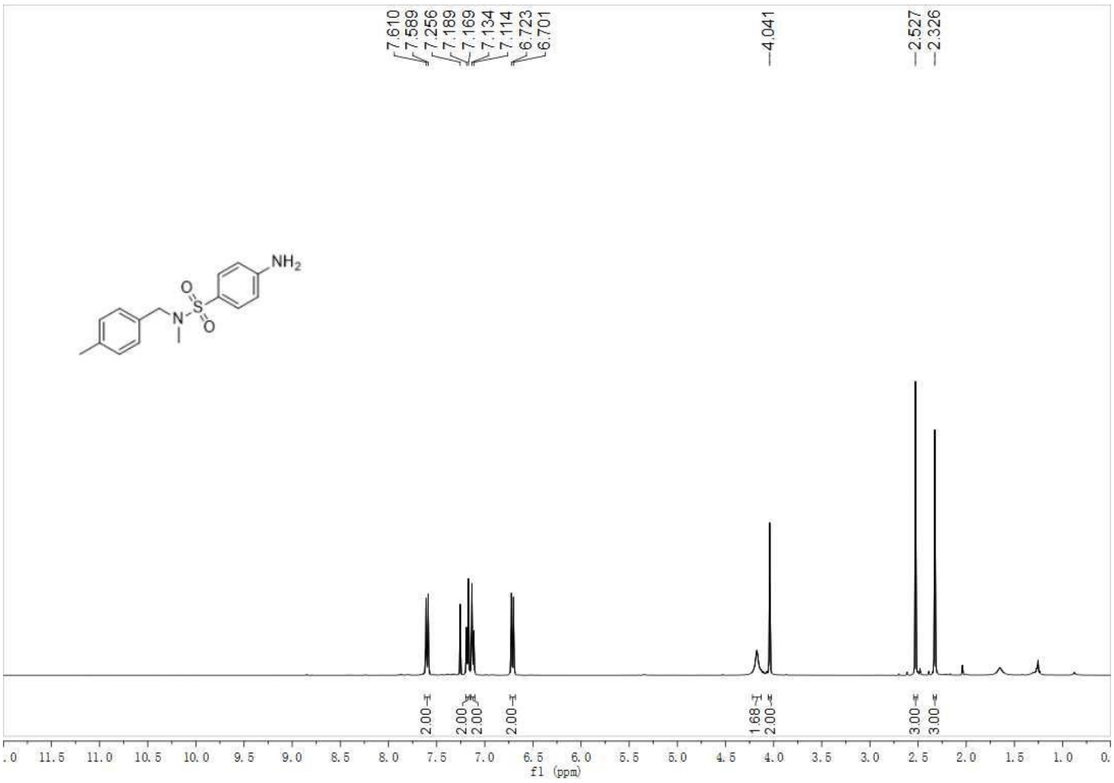

^{13}C NMR Spectrum of **50**

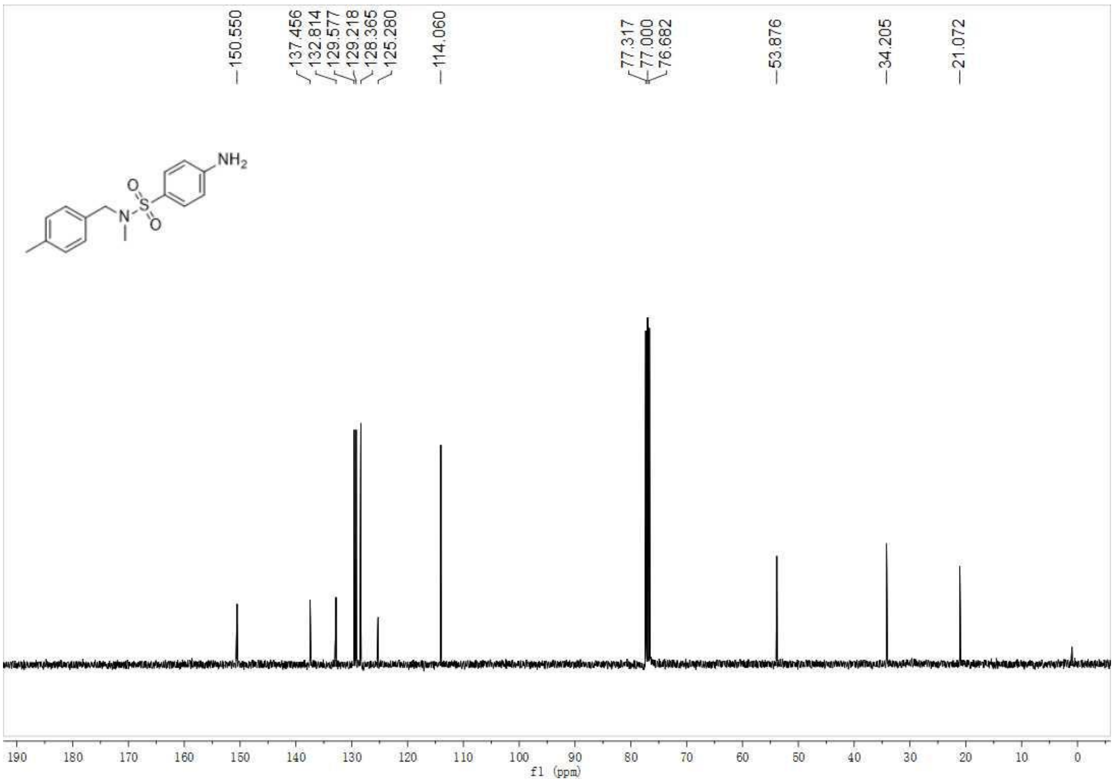

^{1}H NMR Spectrum of **51**

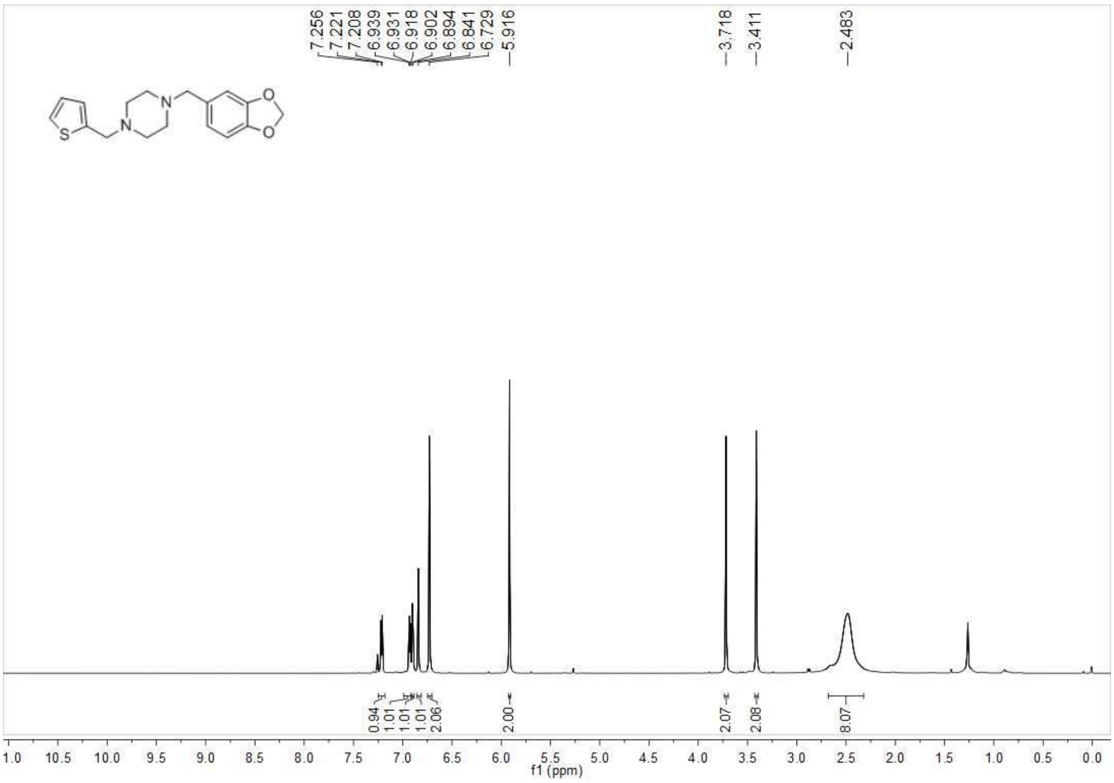

^{13}C NMR Spectrum of **51**

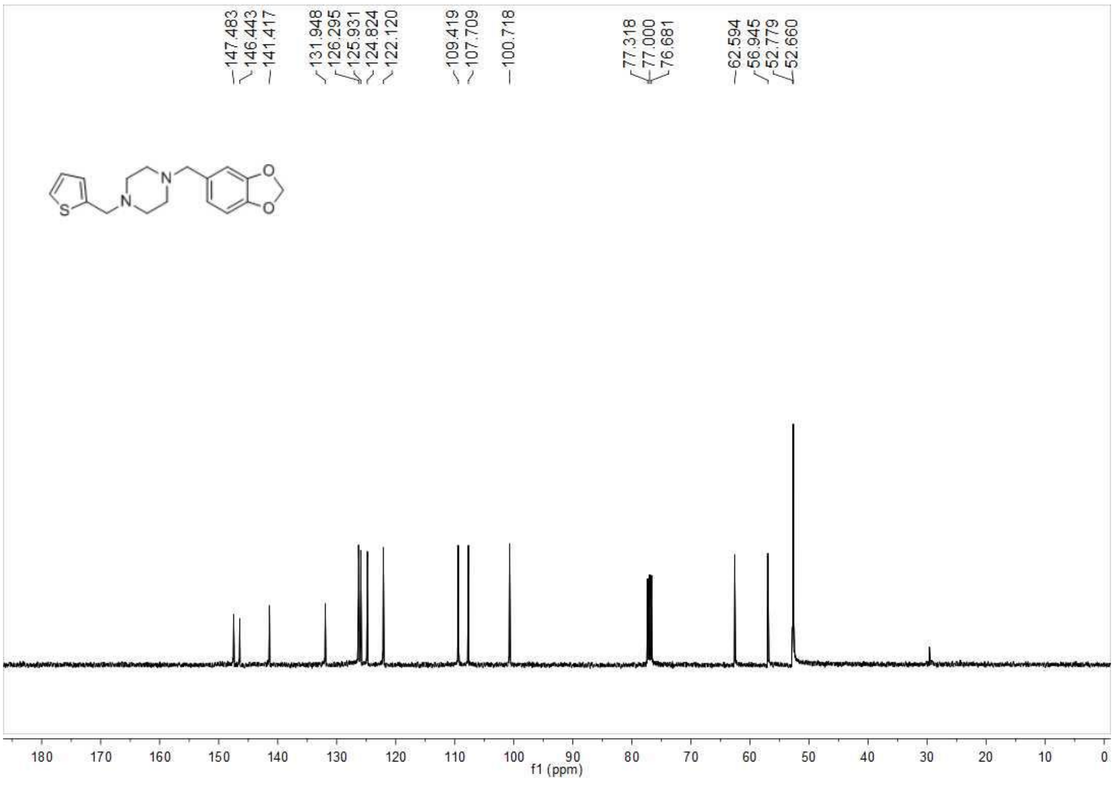

^{1}H NMR Spectrum of **52**

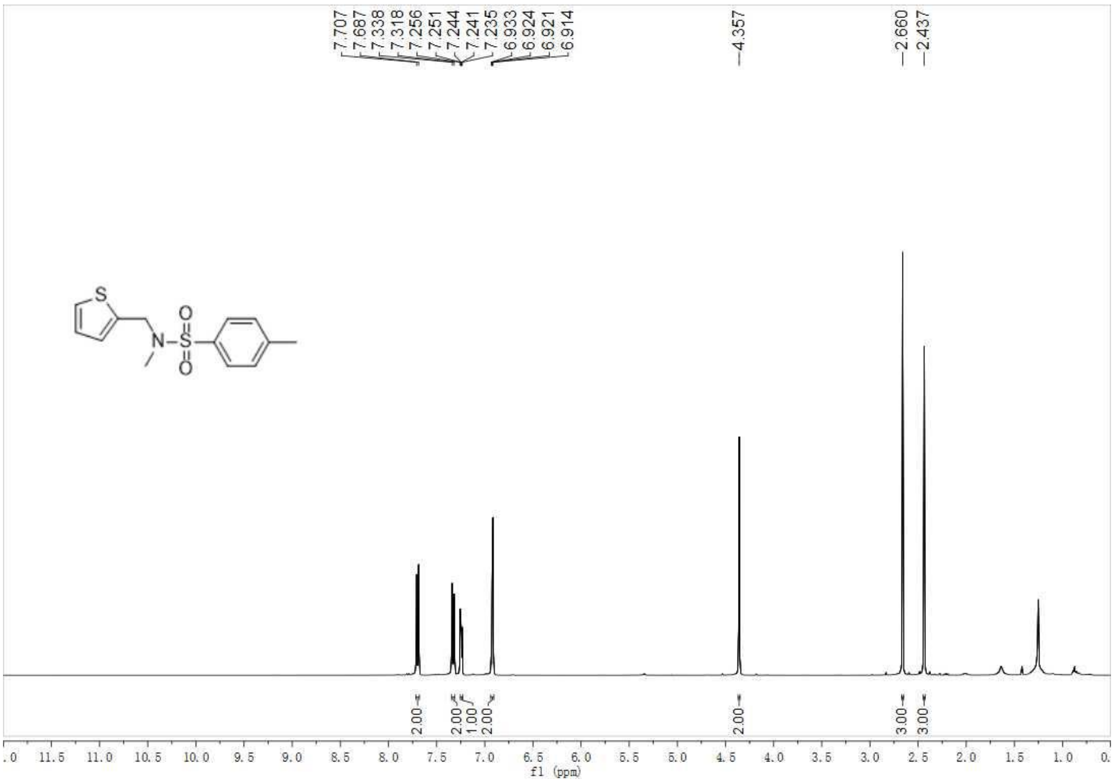

^{13}C NMR Spectrum of **52**

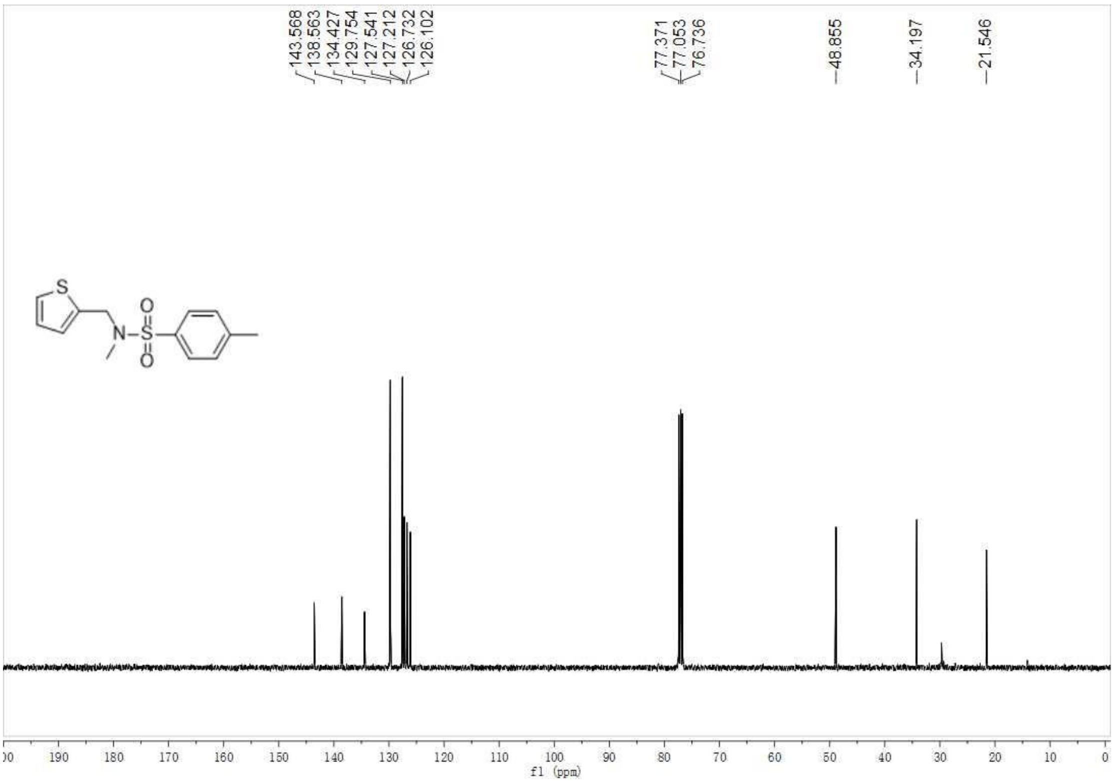

^{1}H NMR Spectrum of **53**

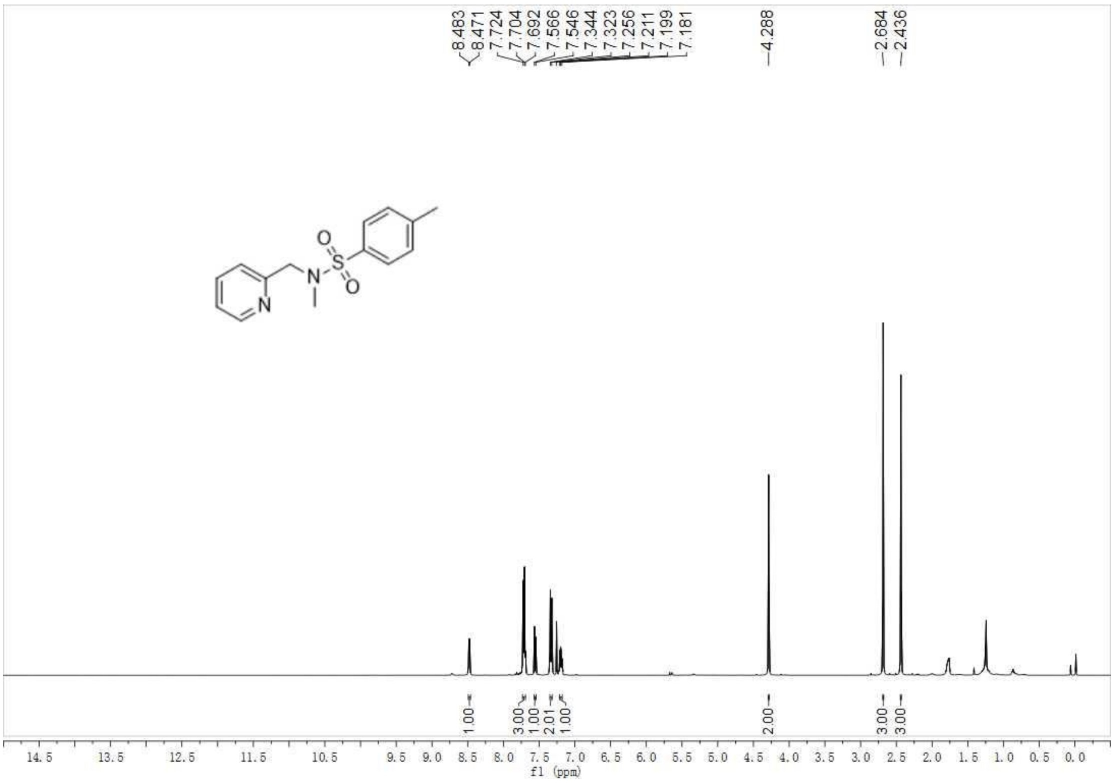

^{13}C NMR Spectrum of **53**

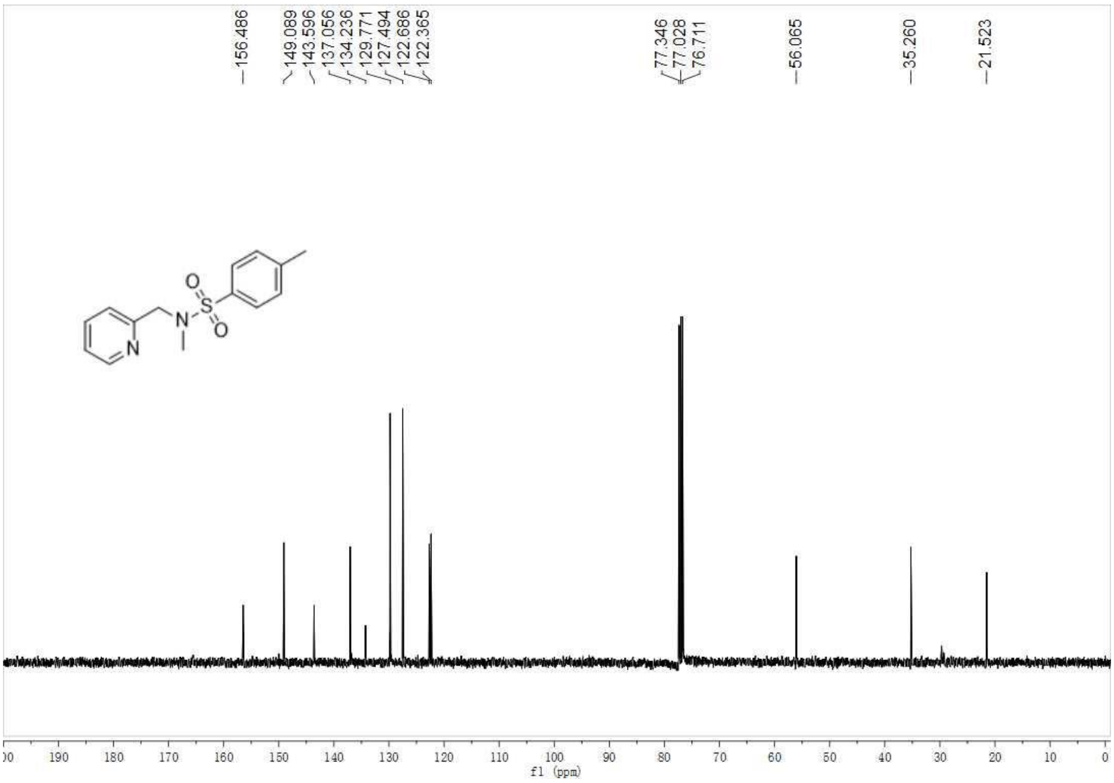

^{1}H NMR Spectrum of **54**

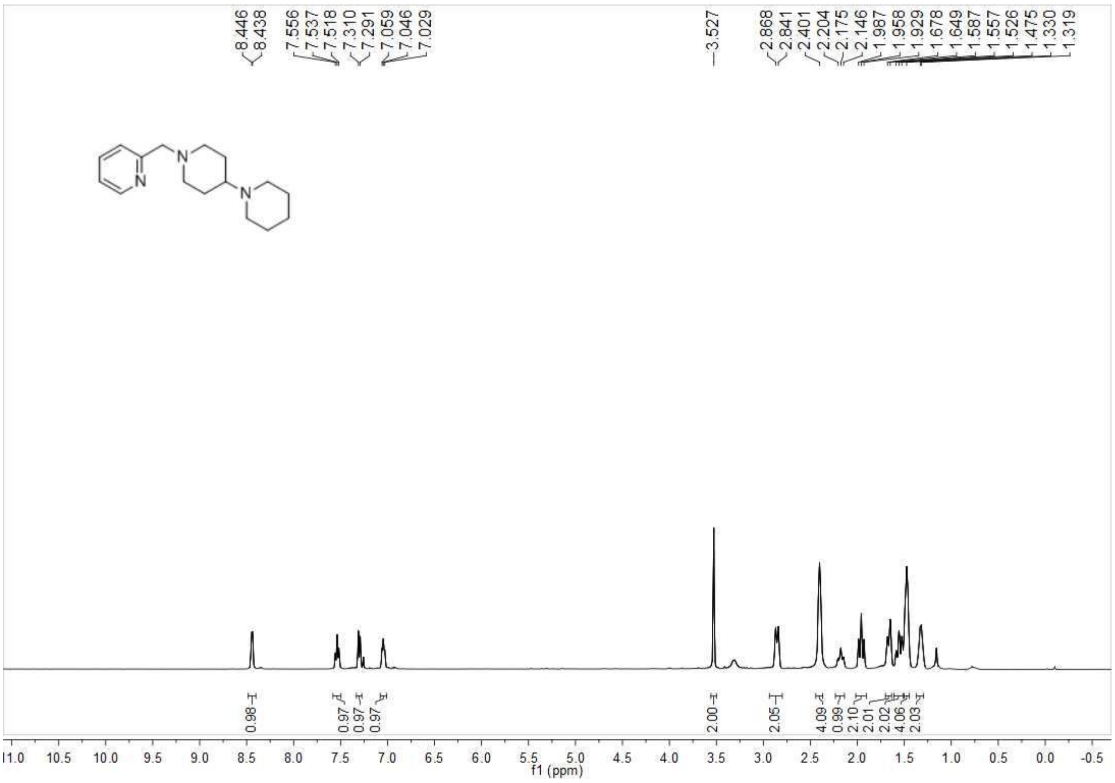

^{13}C NMR Spectrum of **54**

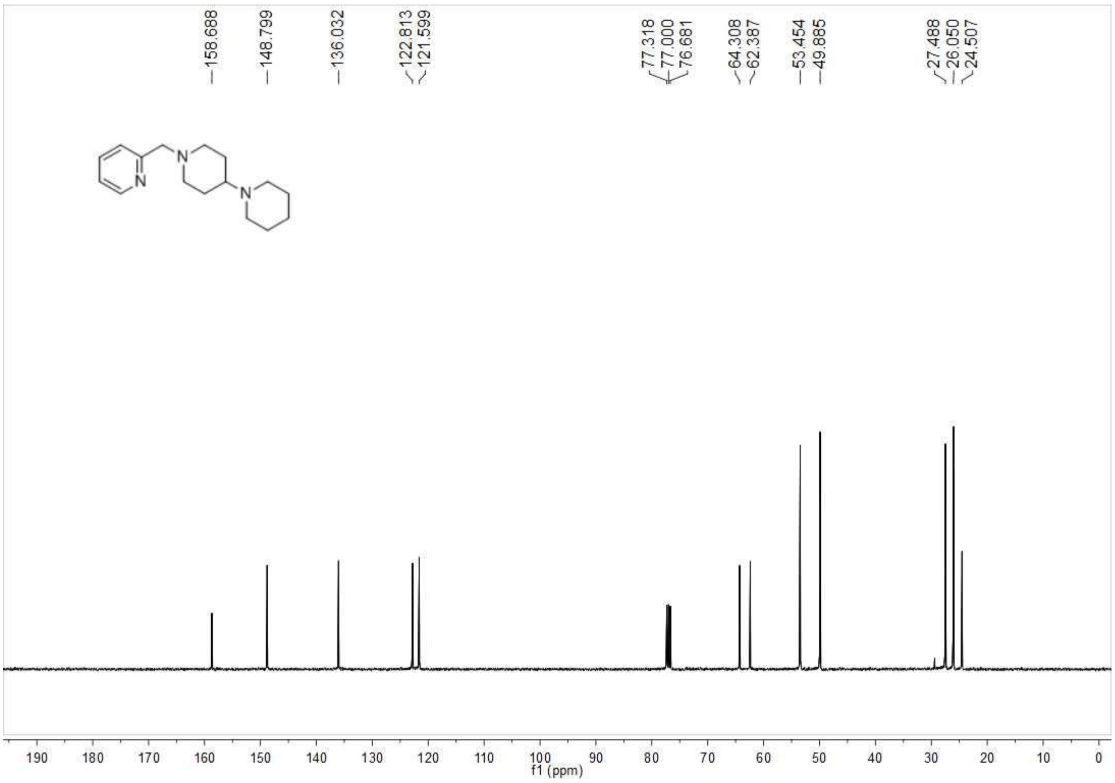

^{1}H NMR Spectrum of **55**

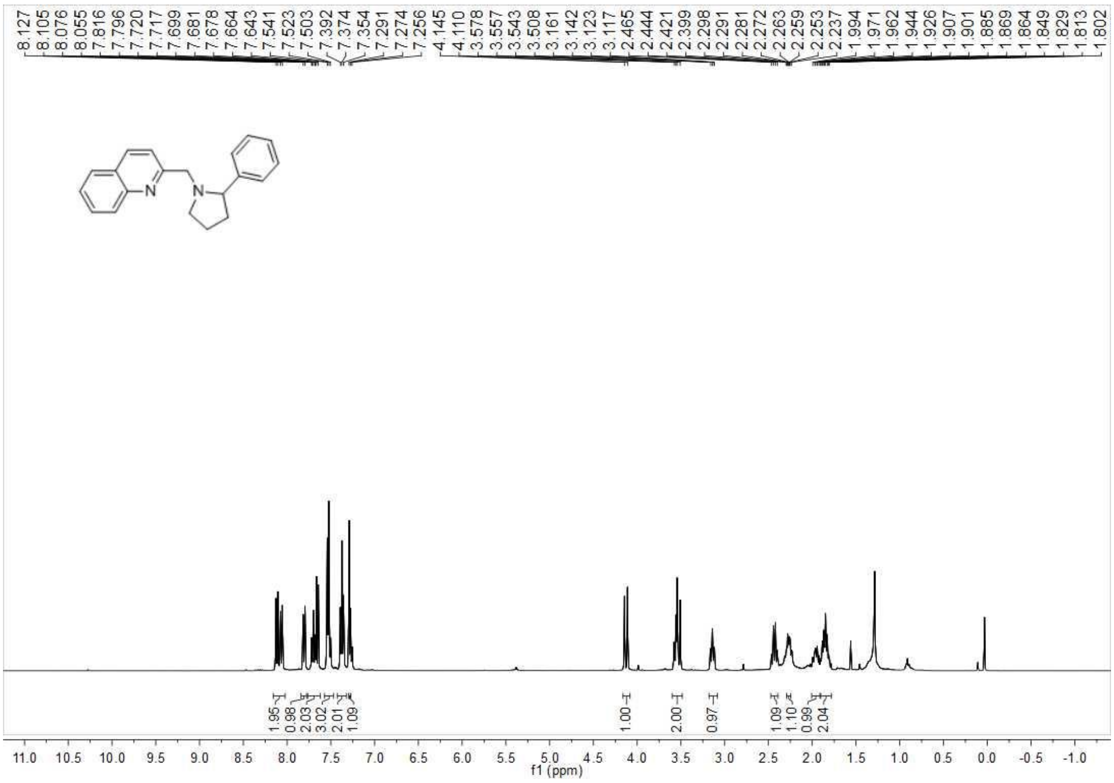

^{13}C NMR Spectrum of **55**

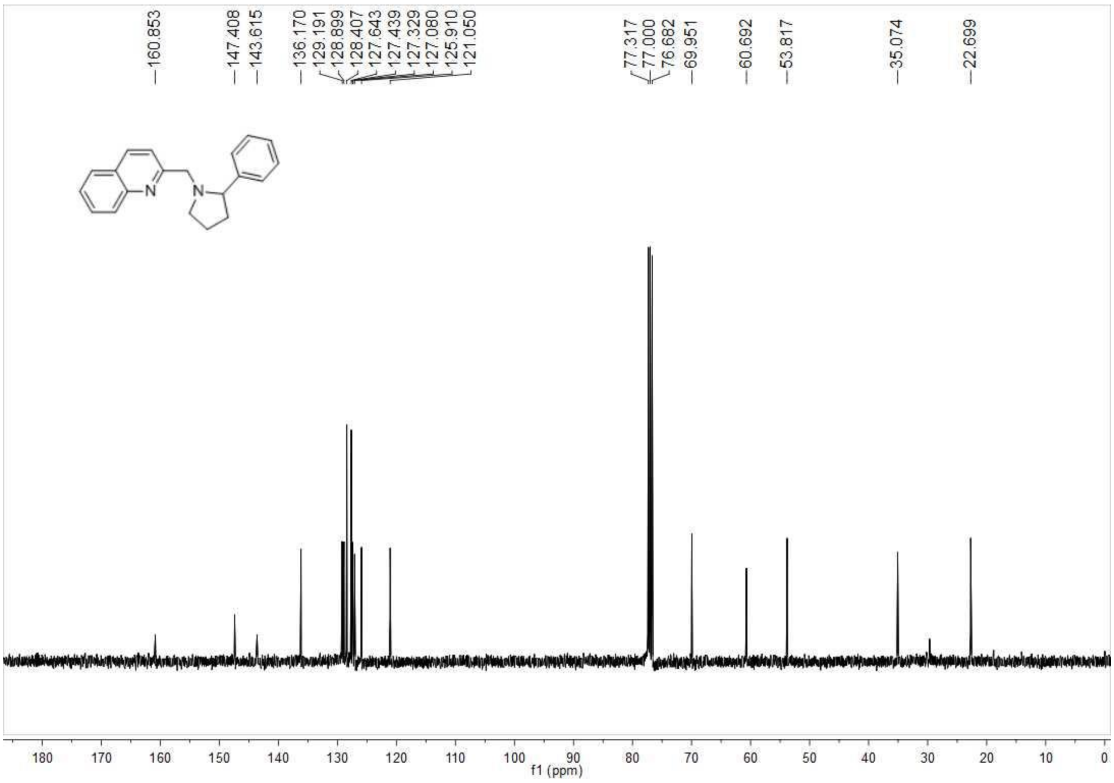

^{1}H NMR Spectrum of **56**

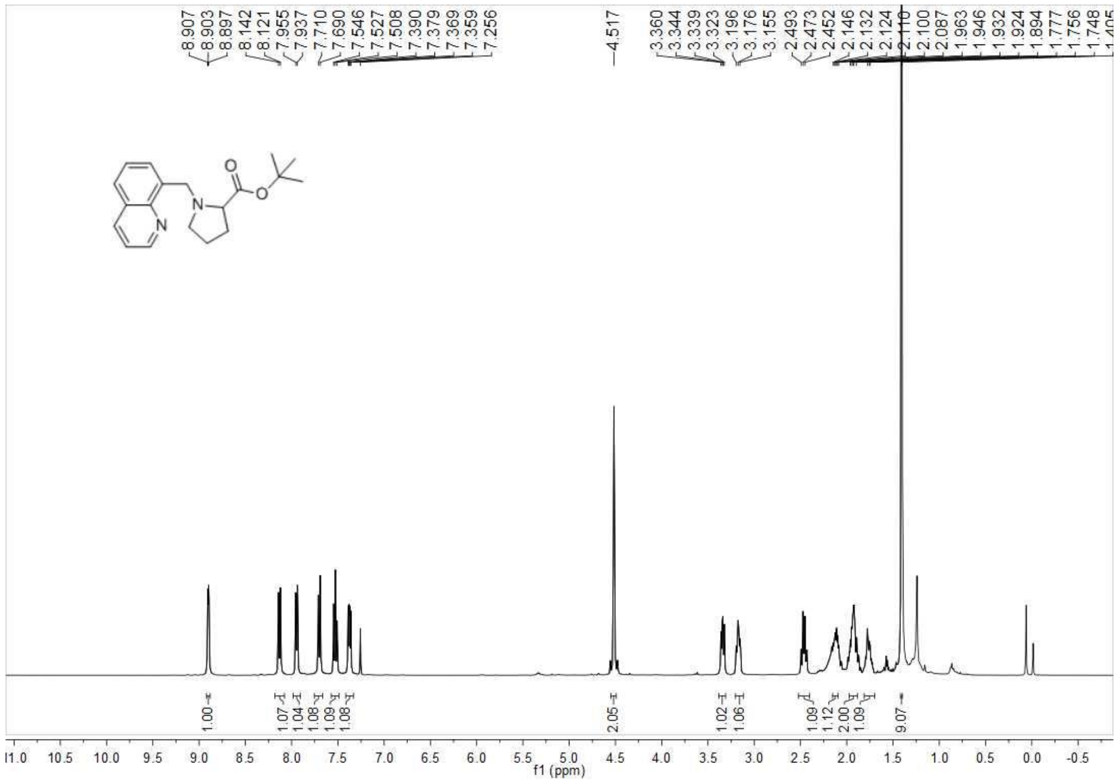

^{13}C NMR Spectrum of **56**

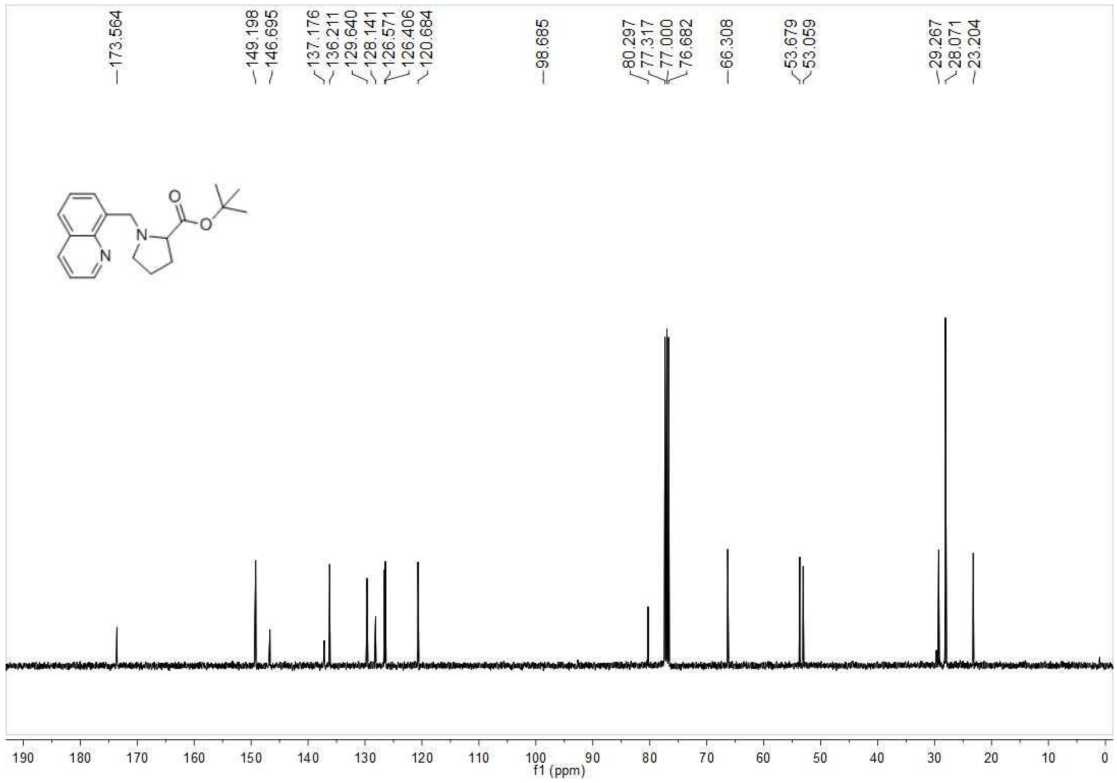

^{1}H NMR Spectrum of **57**

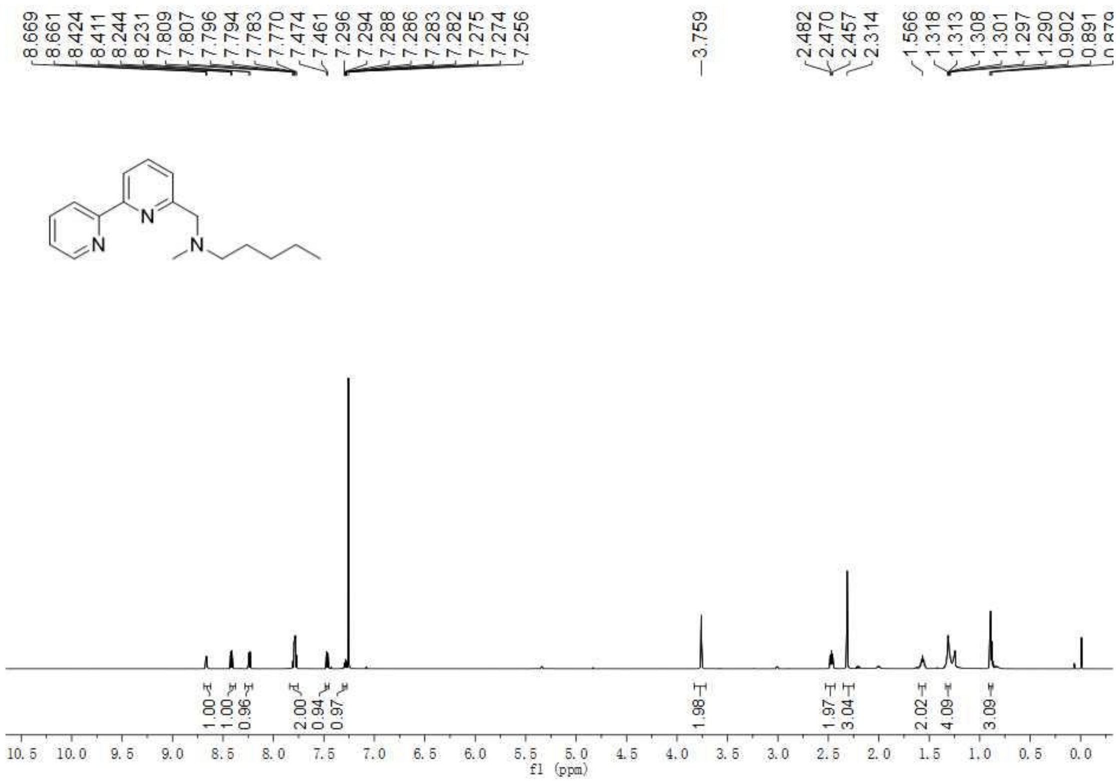

^{13}C NMR Spectrum of **57**

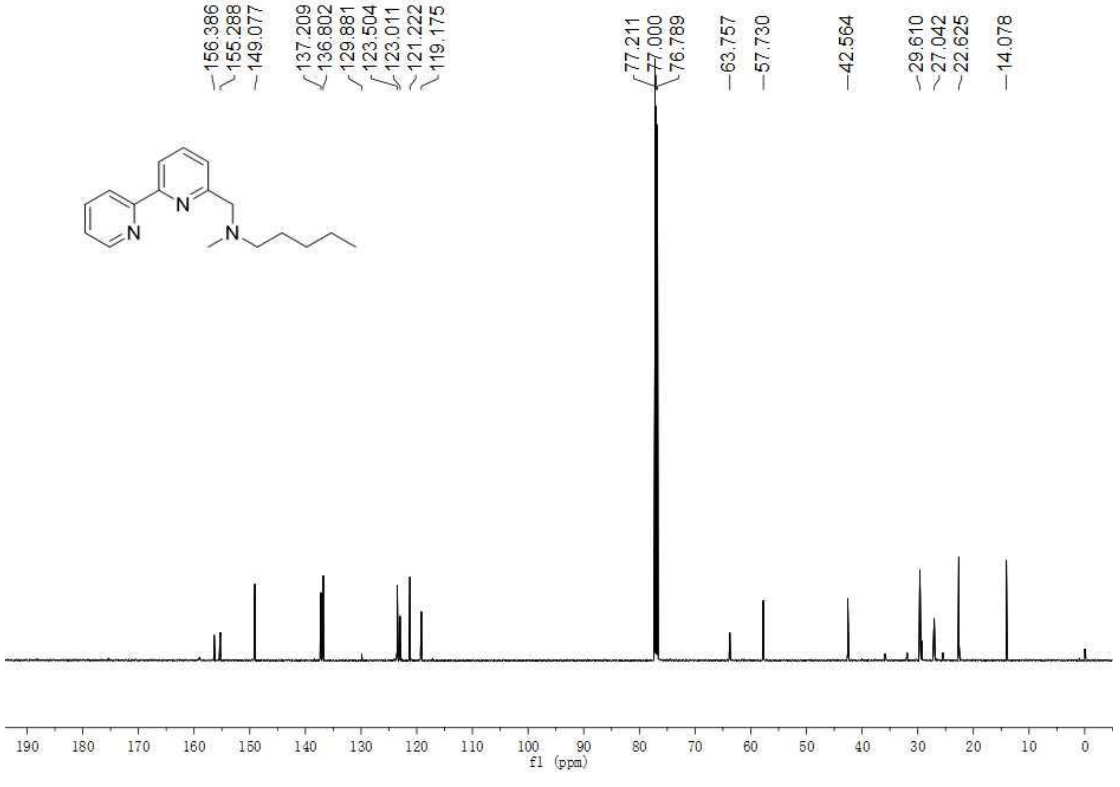

^{1}H NMR Spectrum of **58**

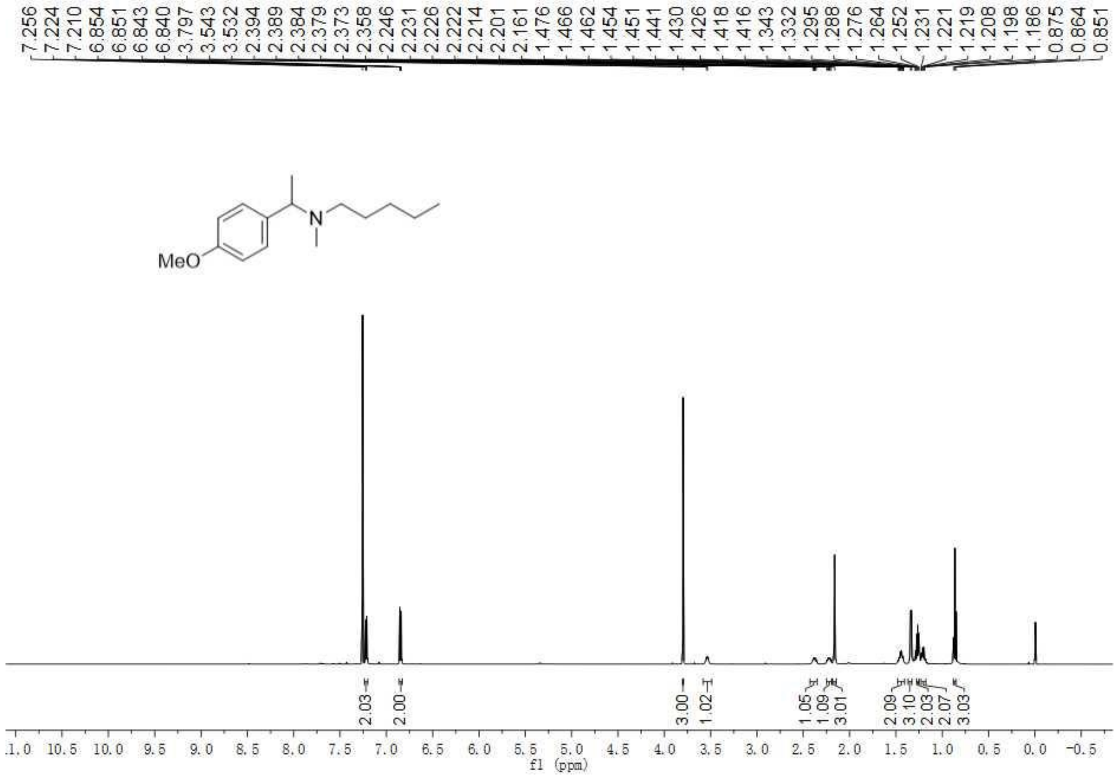

^{13}C NMR Spectrum of **58**

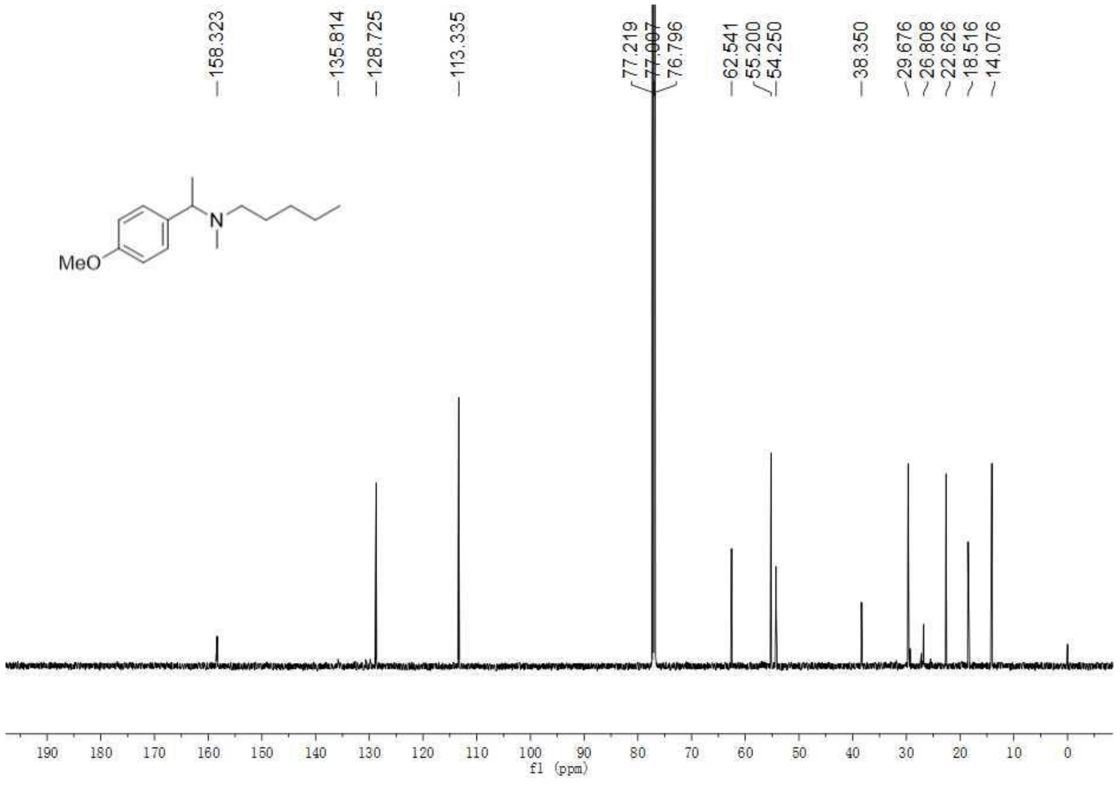

^{1}H NMR Spectrum of **59**

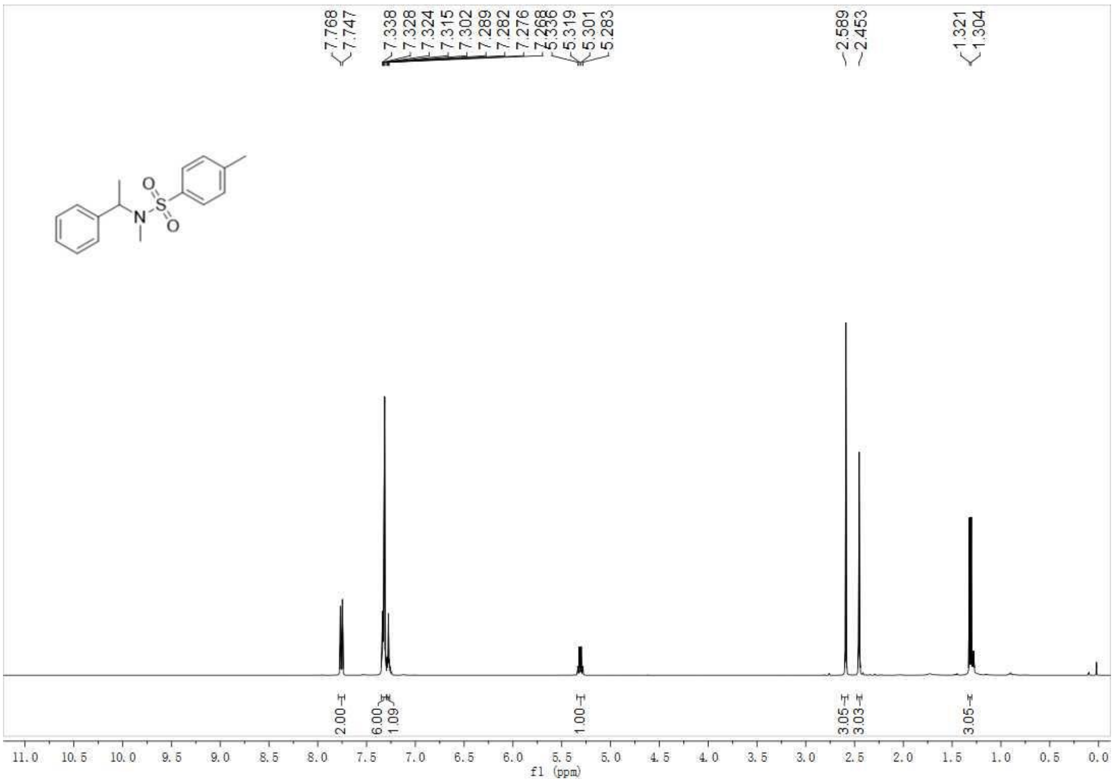

^{13}C NMR Spectrum of **59**

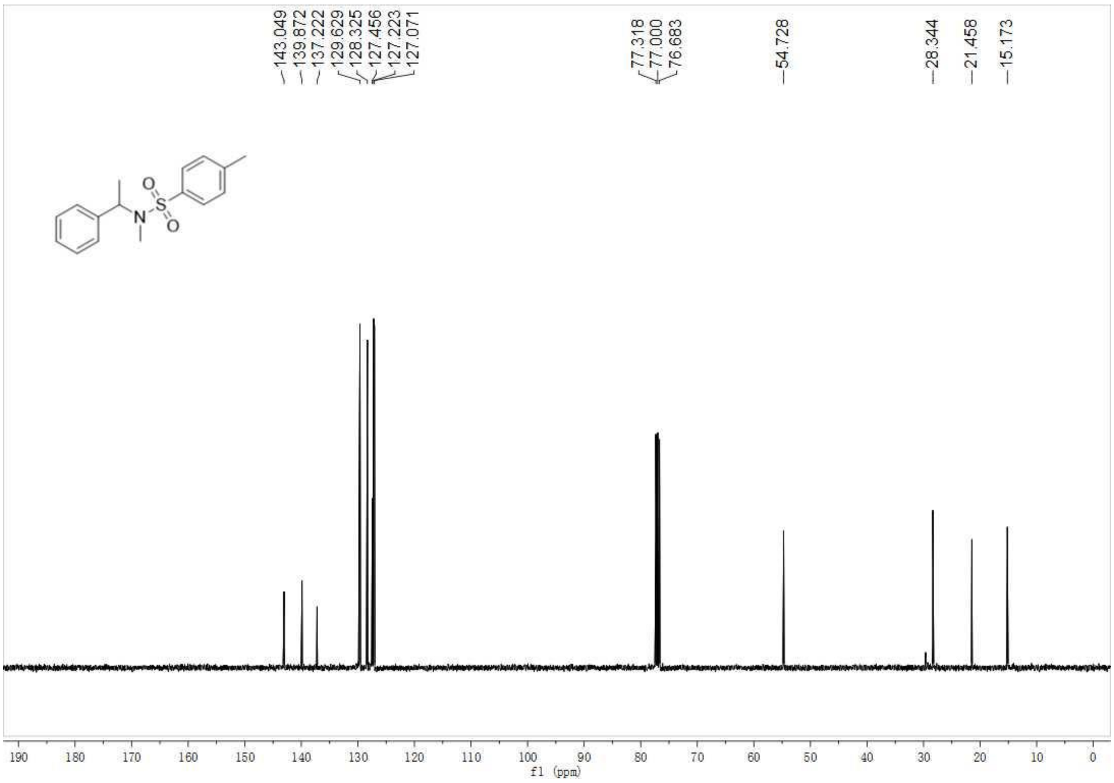

^{1}H NMR Spectrum of **60**

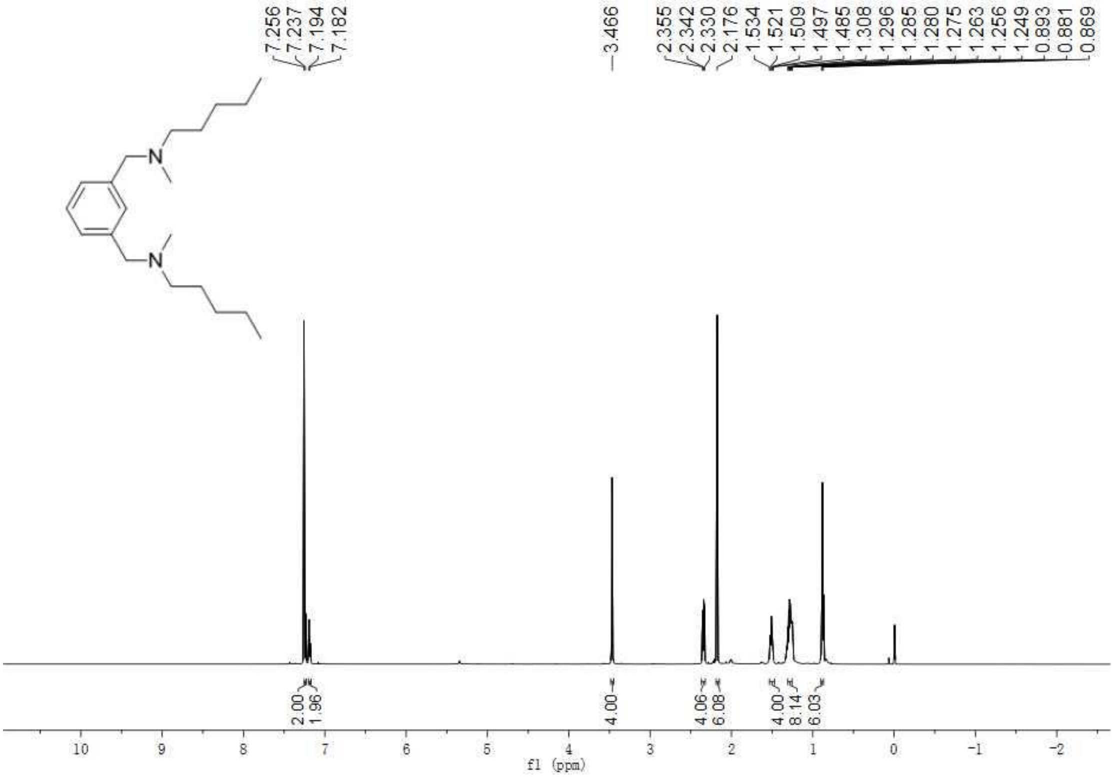

^{13}C NMR Spectrum of **60**

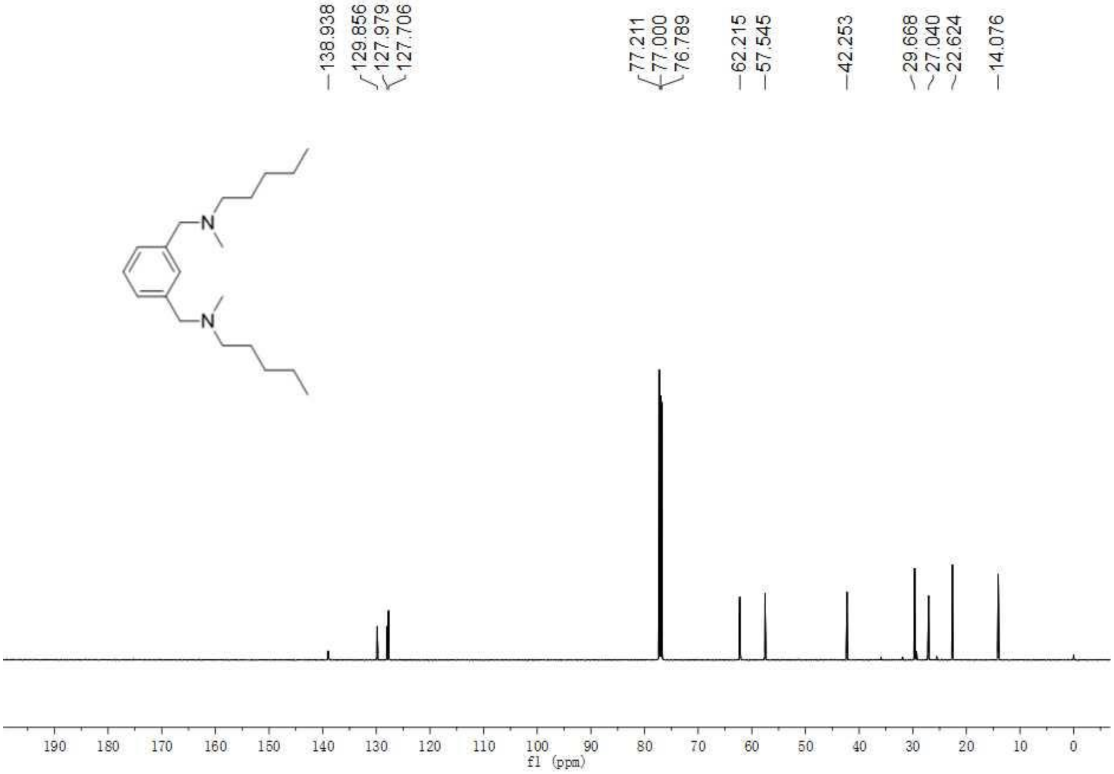

^{1}H NMR Spectrum of **61**

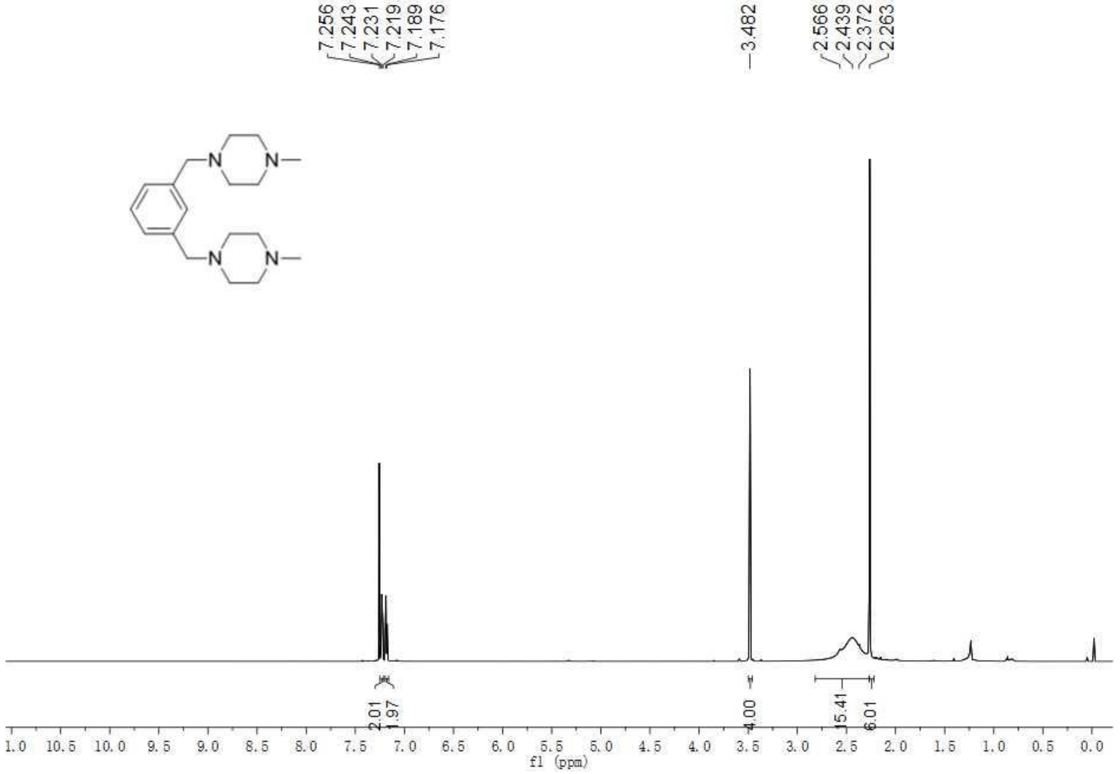

^{13}C NMR Spectrum of **61**

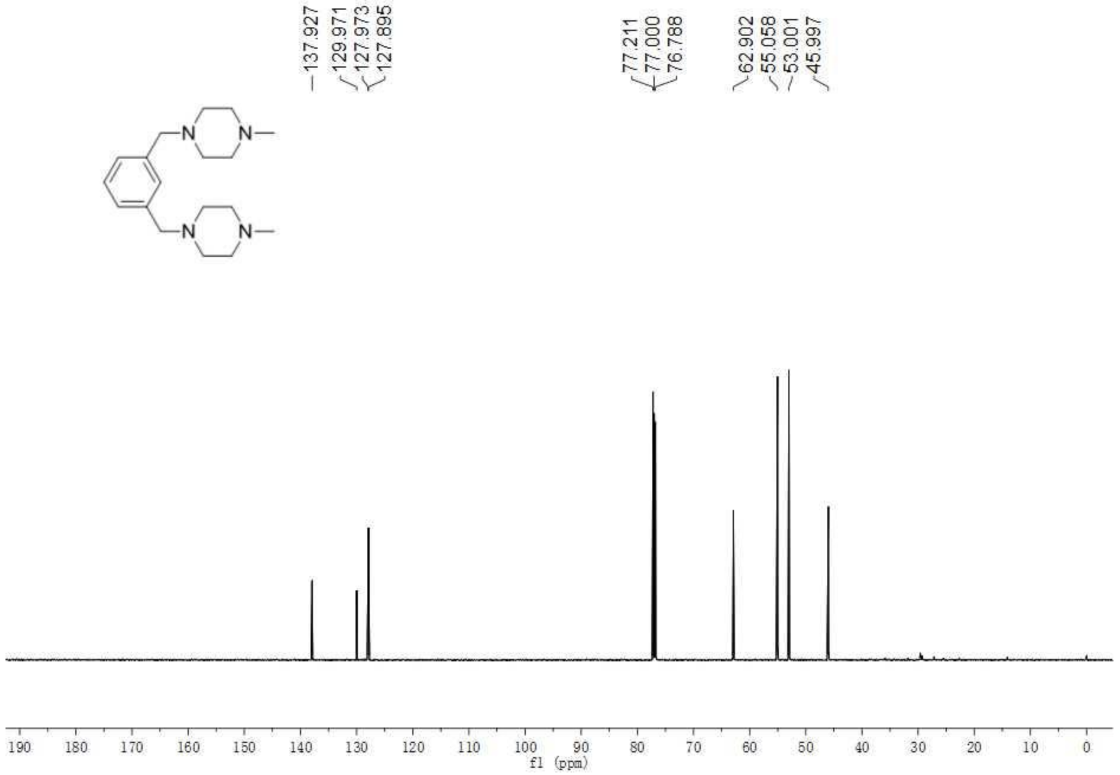

^{1}H NMR Spectrum of **63**

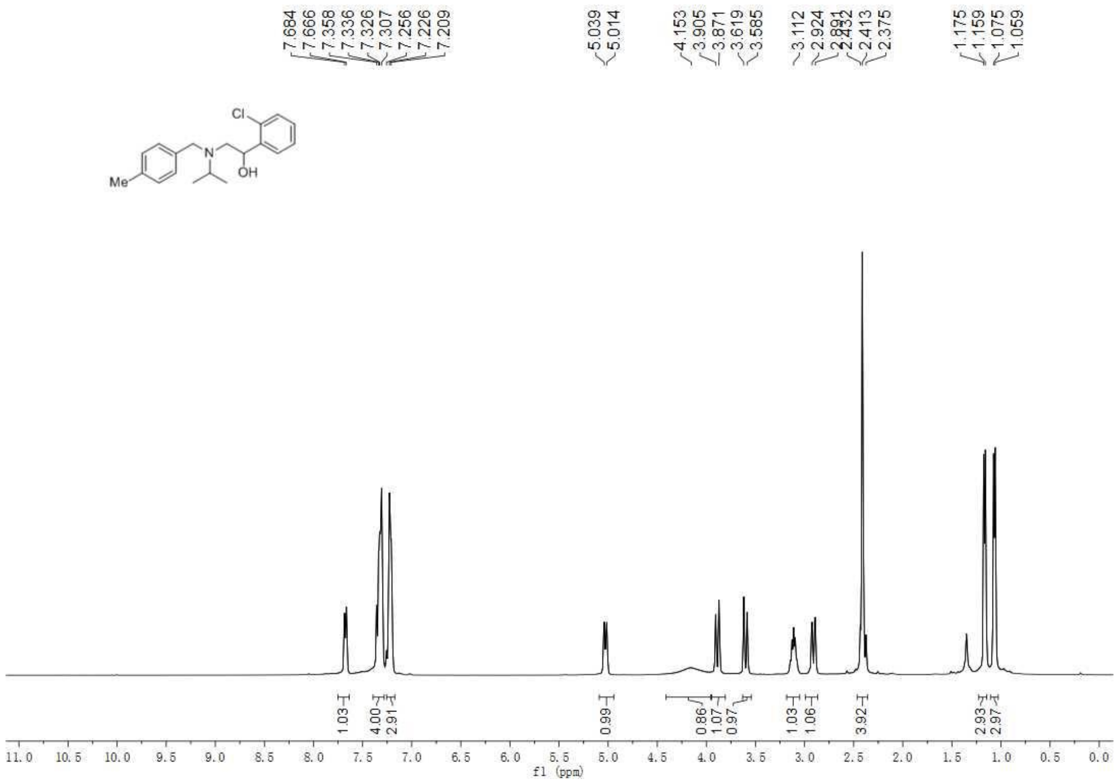

^{13}C NMR Spectrum of **63**

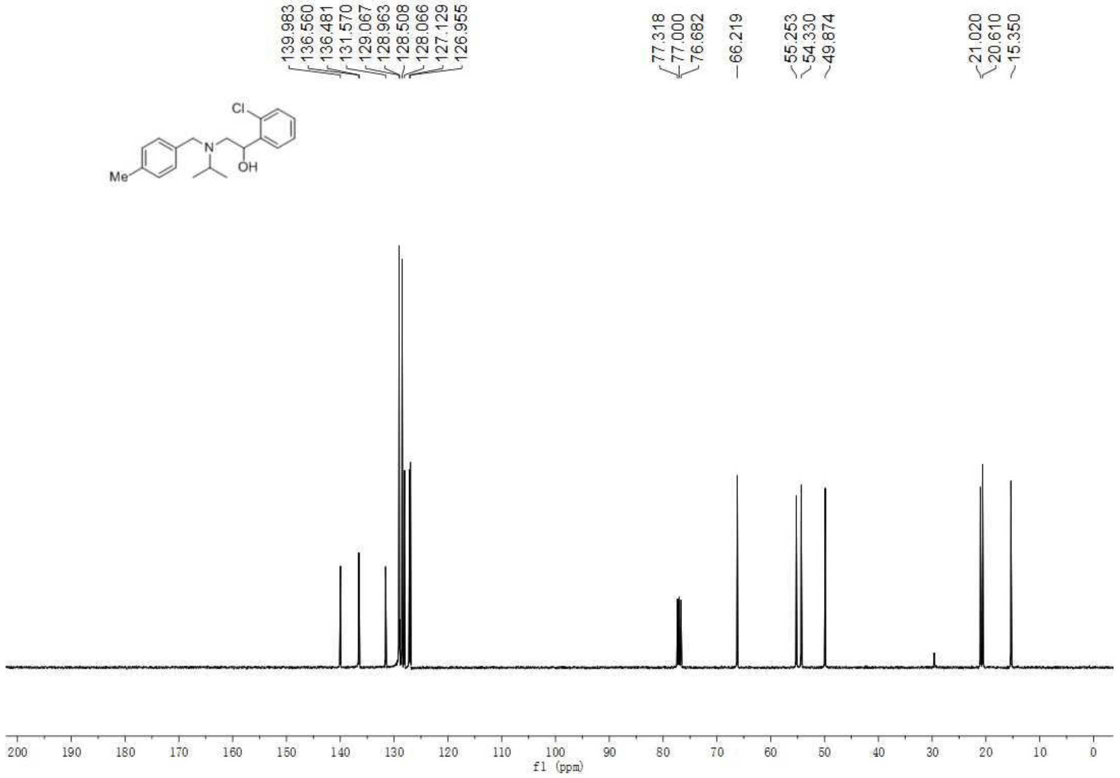

^{1}H NMR Spectrum of **64**

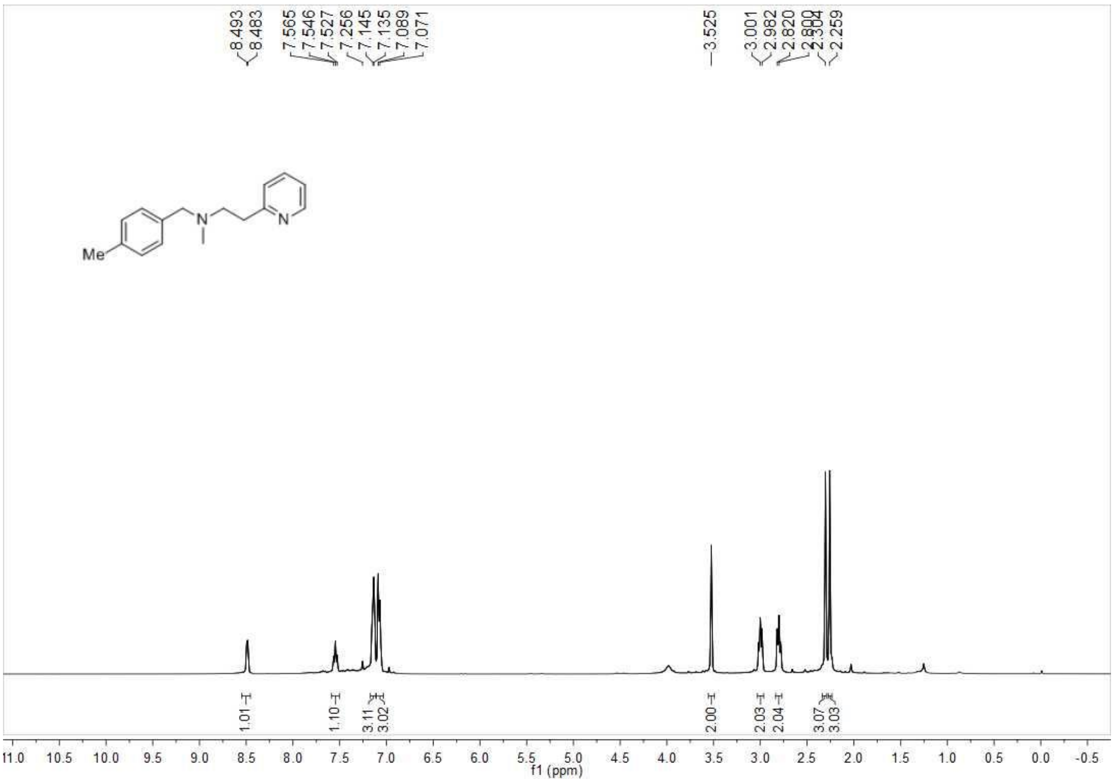

^{13}C NMR Spectrum of **64**

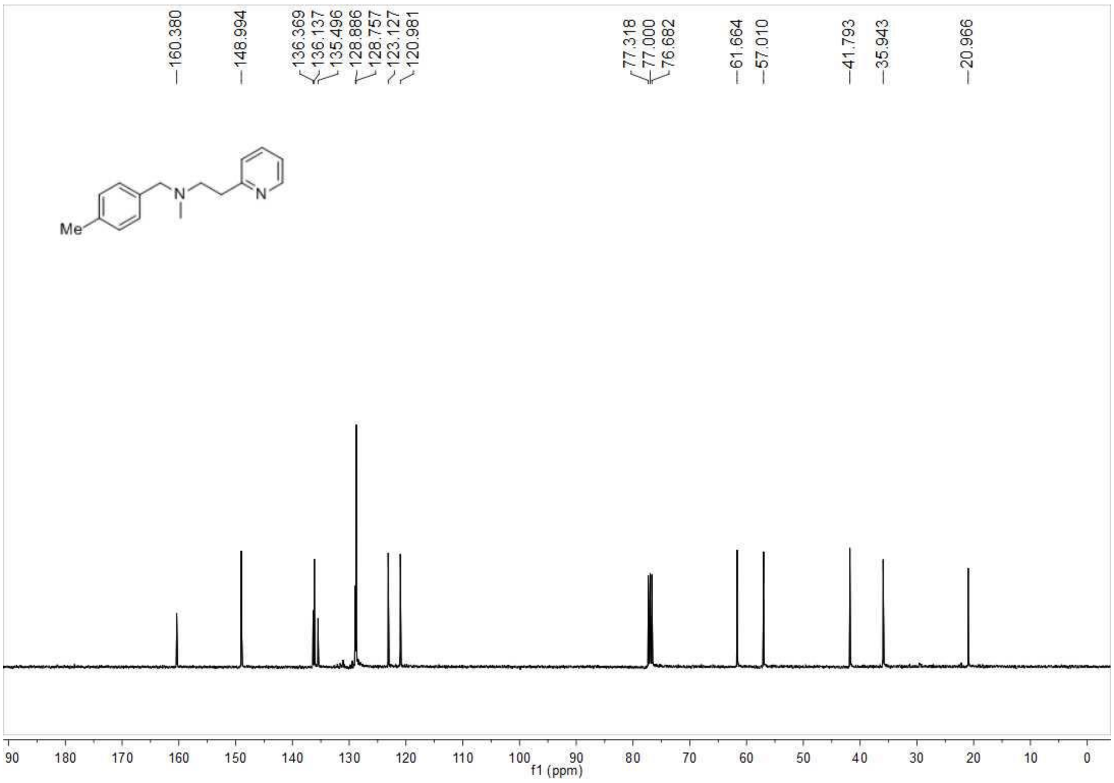

^{1}H NMR Spectrum of **65**

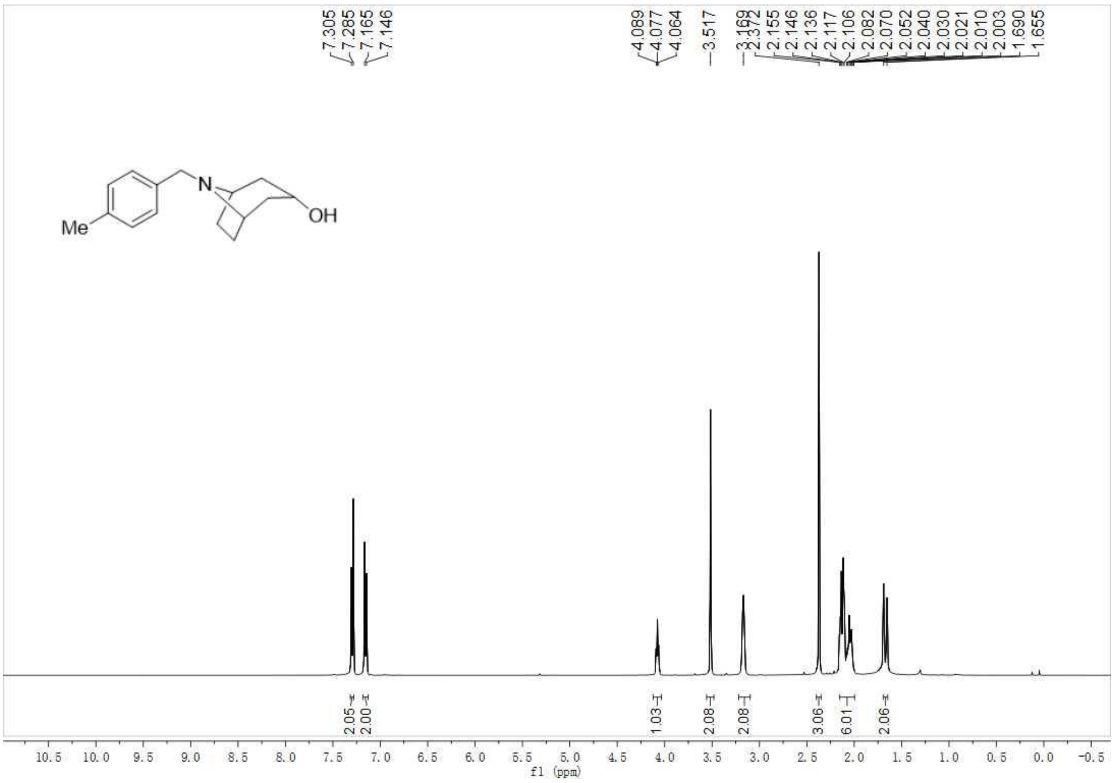

^{13}C NMR Spectrum of **65**

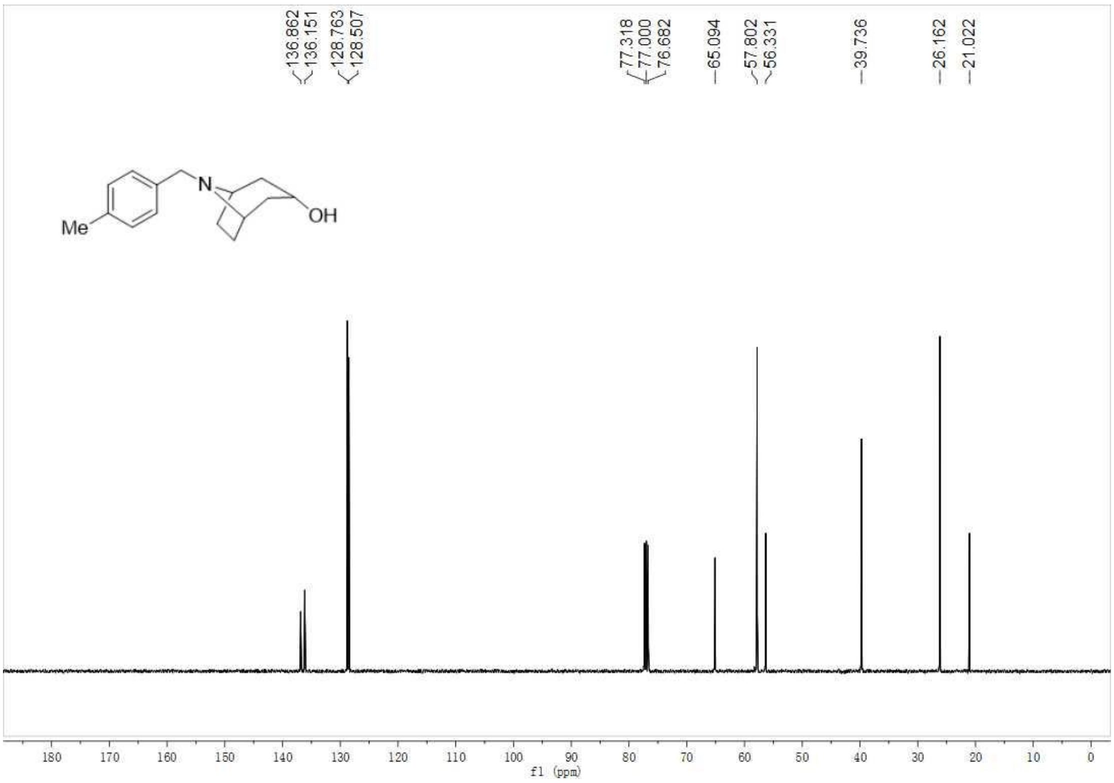

^{1}H NMR Spectrum of **66**

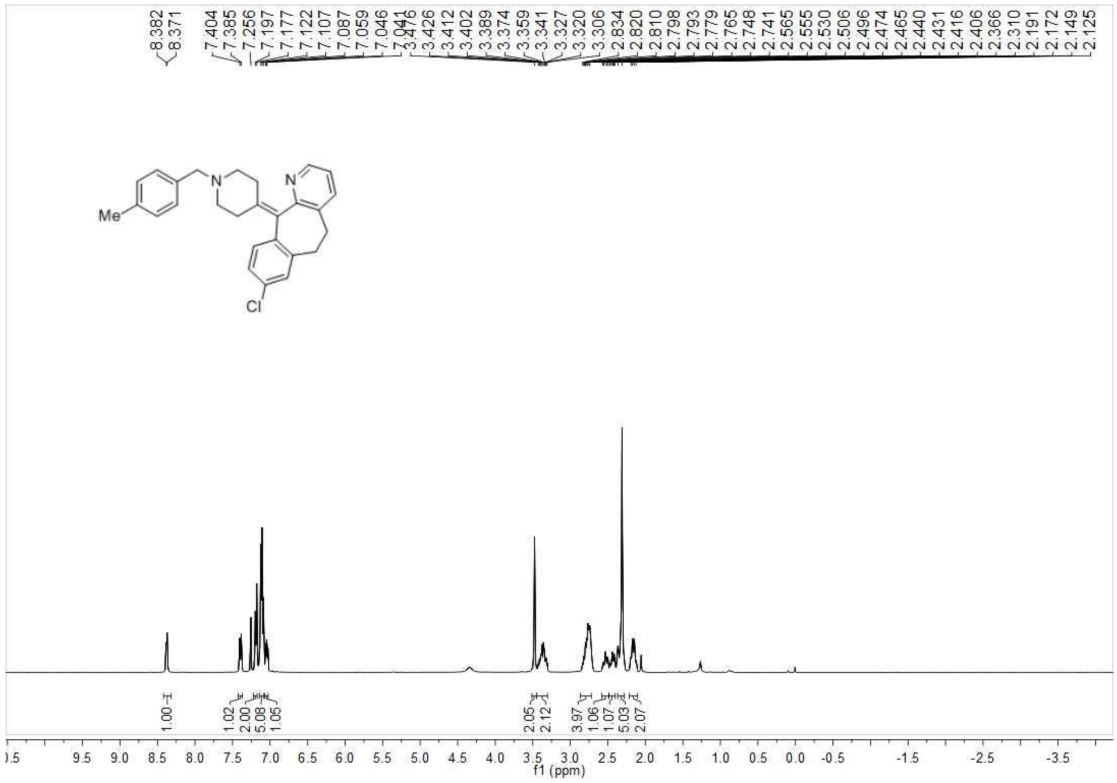

^{13}C NMR Spectrum of **66**

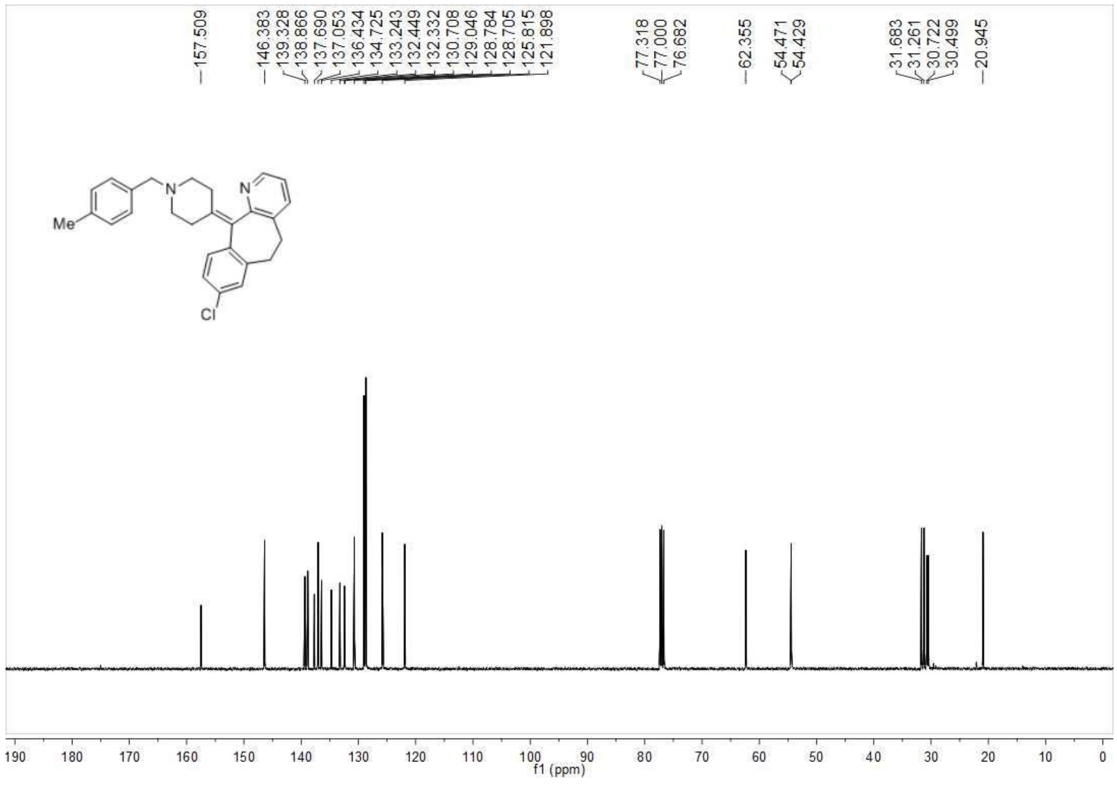

^{1}H NMR Spectrum of **67**

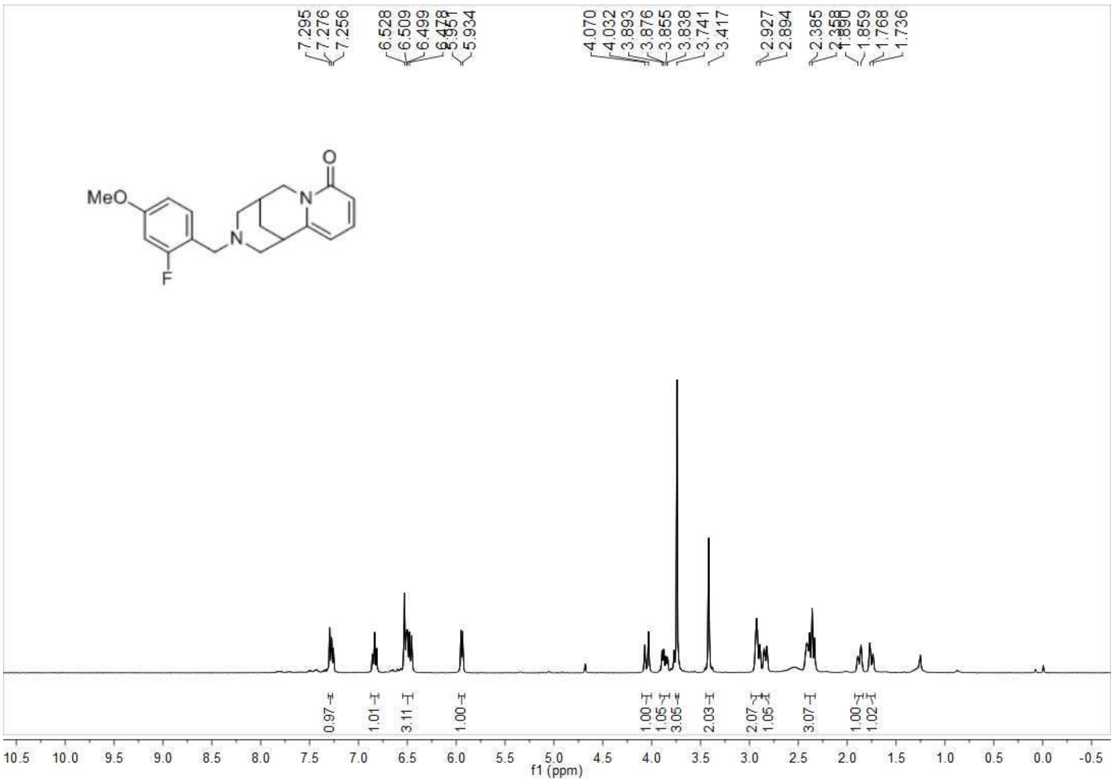

^{13}C NMR Spectrum of **67**

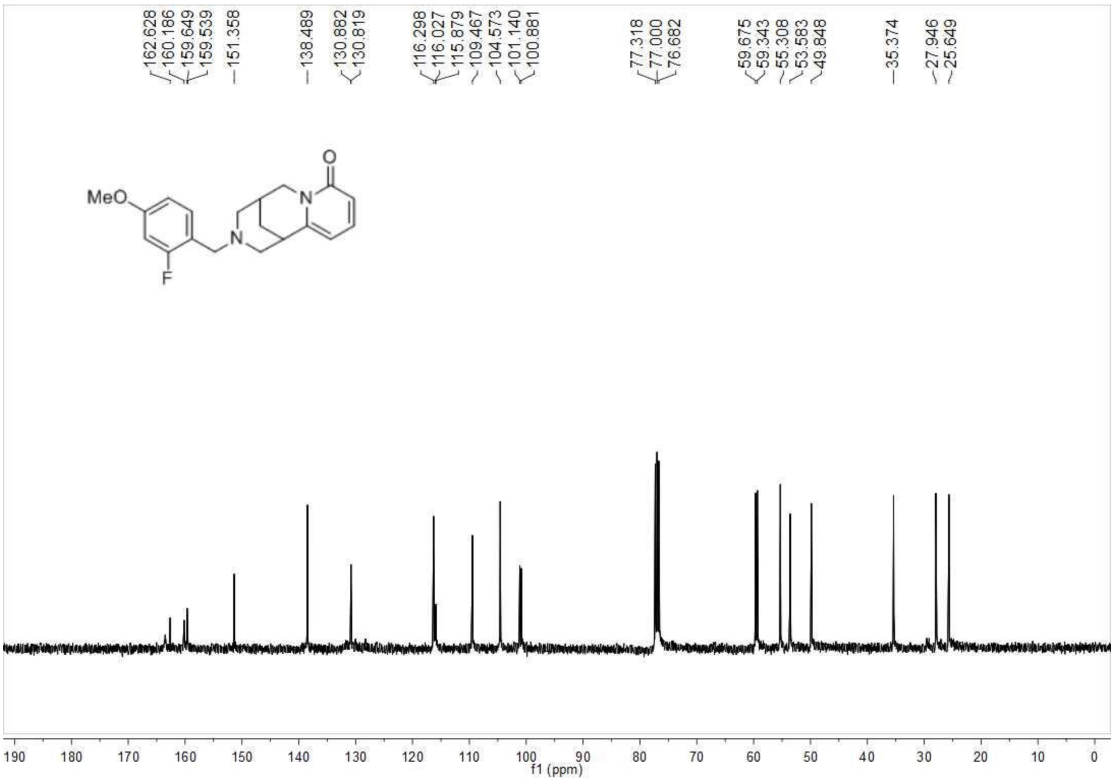

^{19}F NMR Spectrum of **67**

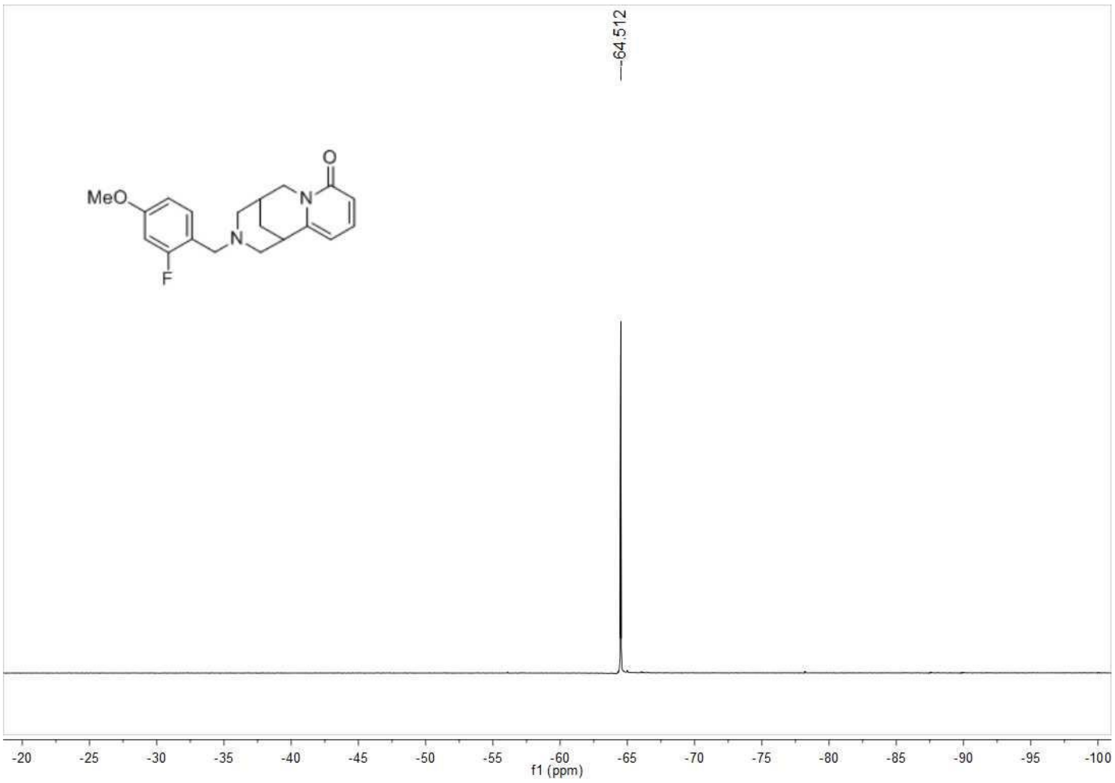

^{1}H NMR Spectrum of **68**

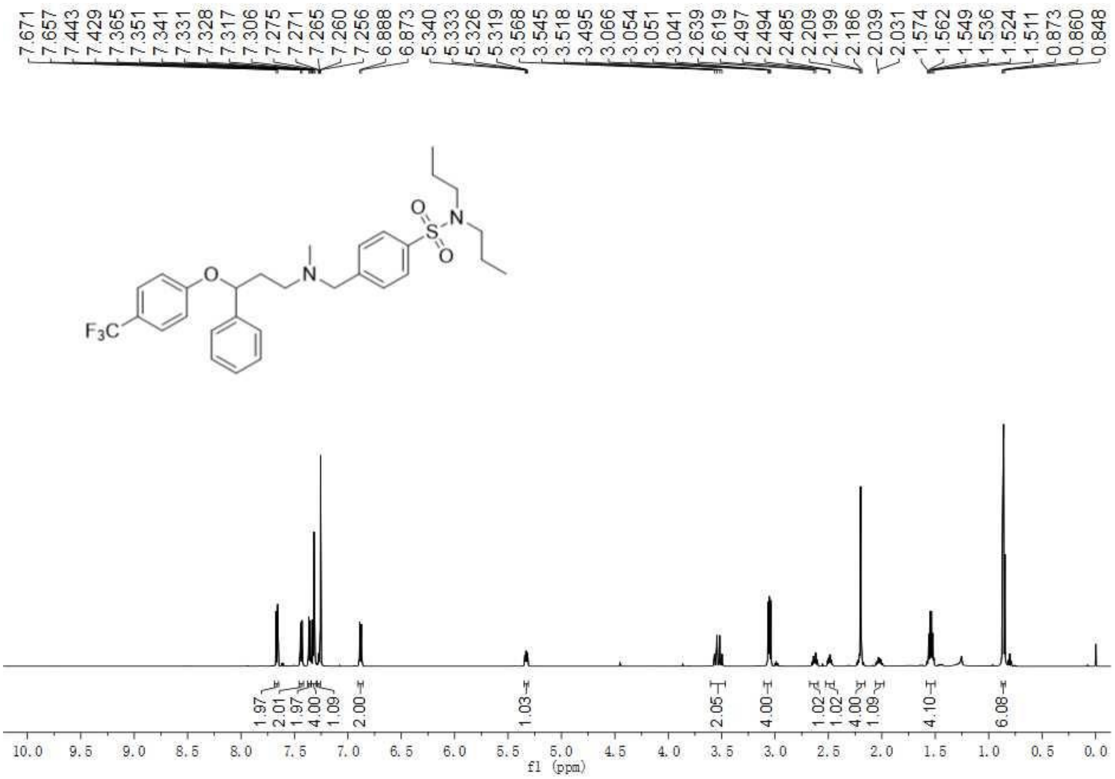

^{13}C NMR Spectrum of **68**

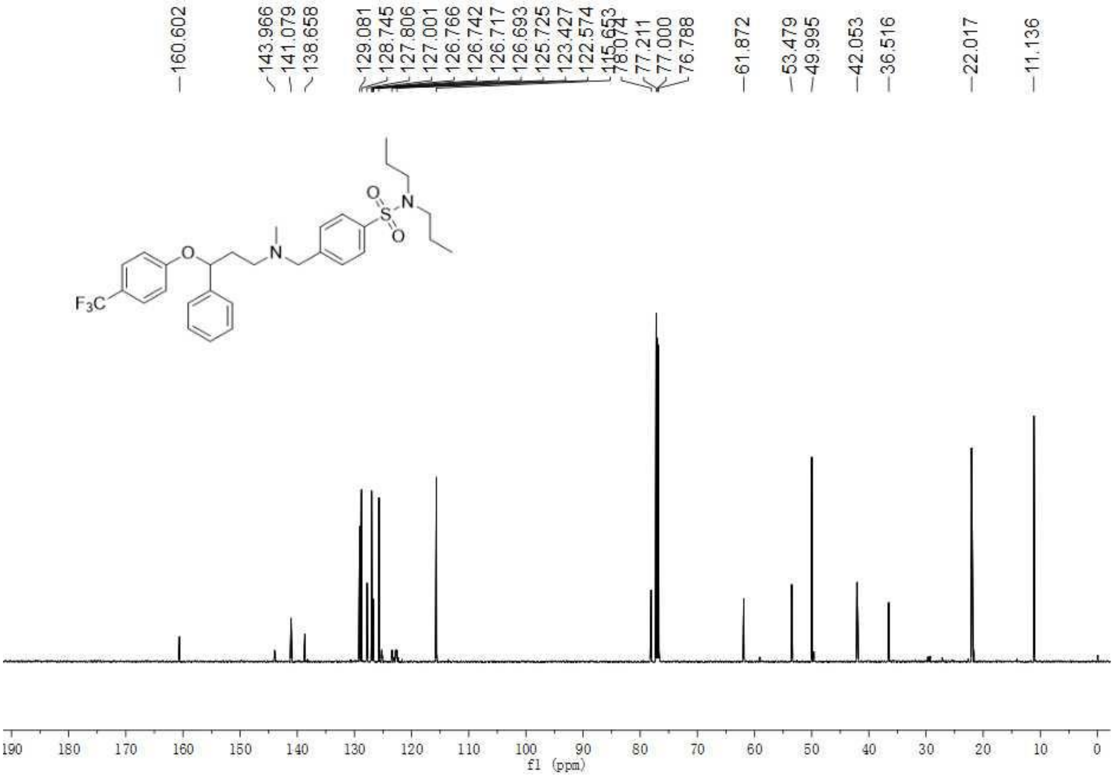

^{19}F NMR Spectrum of **68**

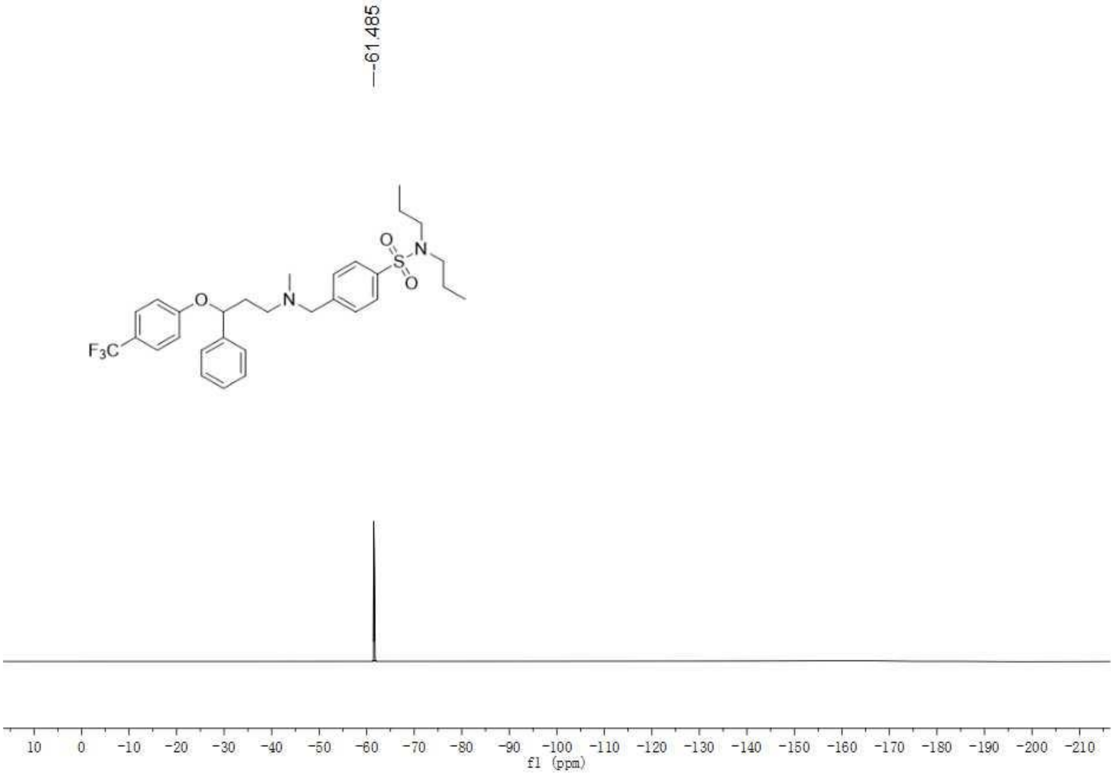

^{1}H NMR Spectrum of **69**

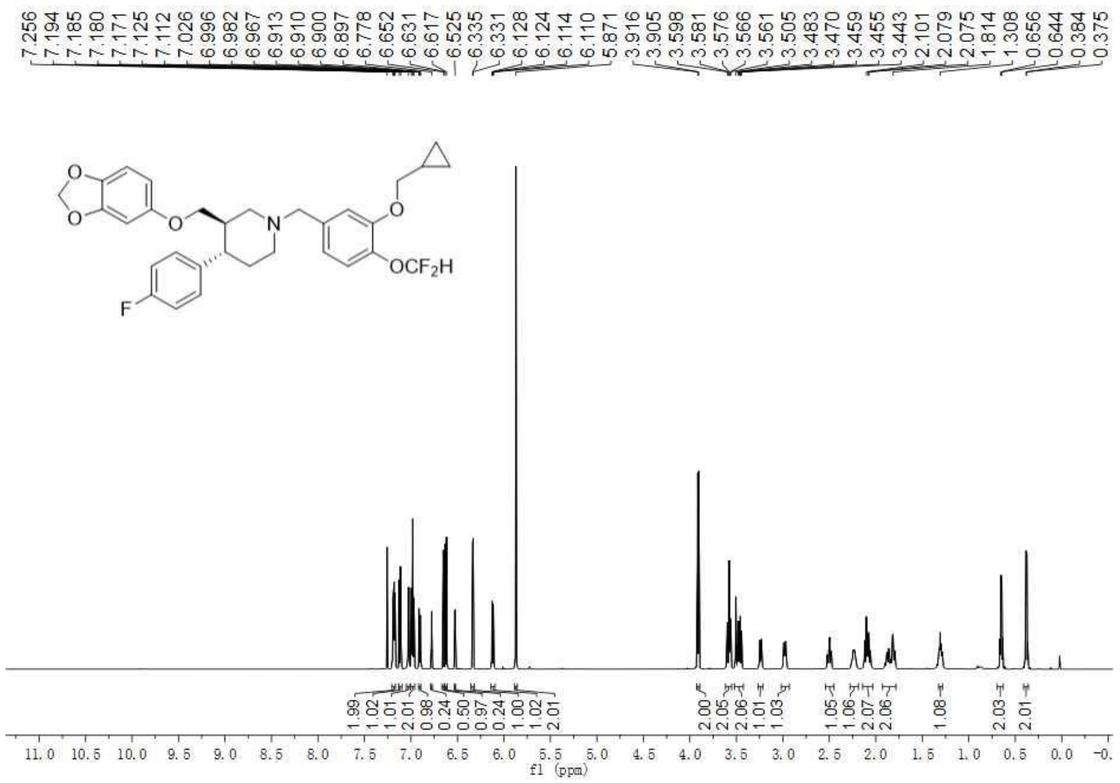

^{13}C NMR Spectrum of **69**

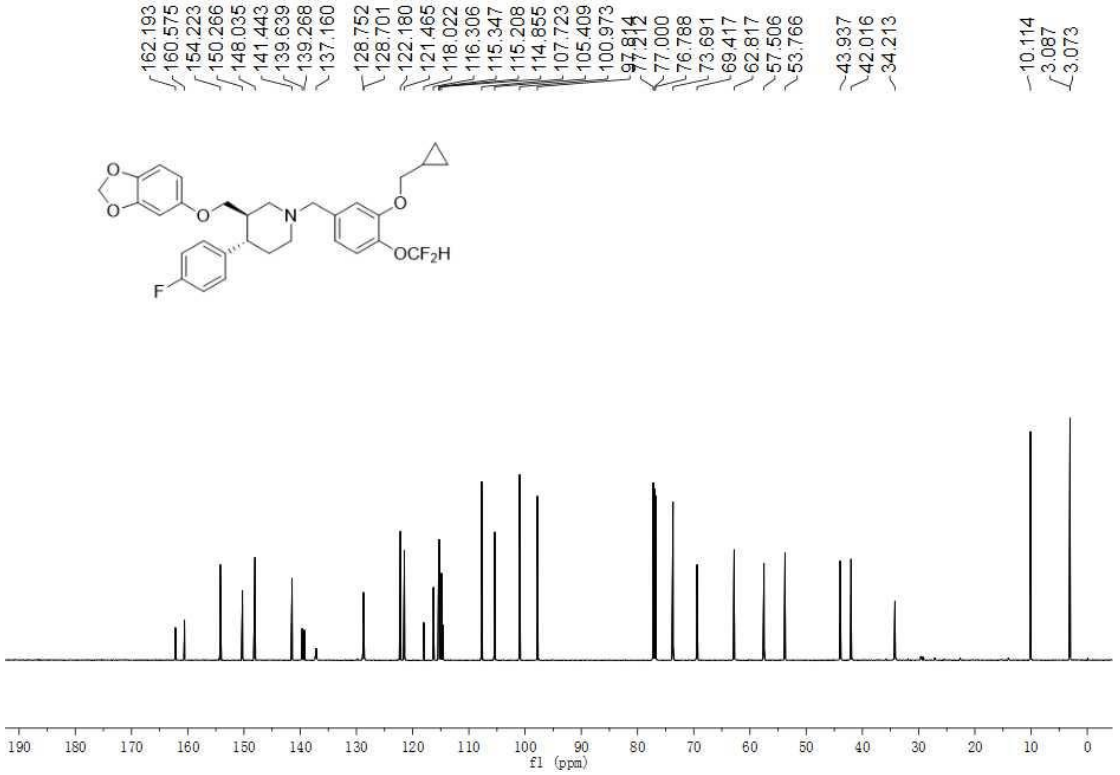

^{19}F NMR Spectrum of **69**

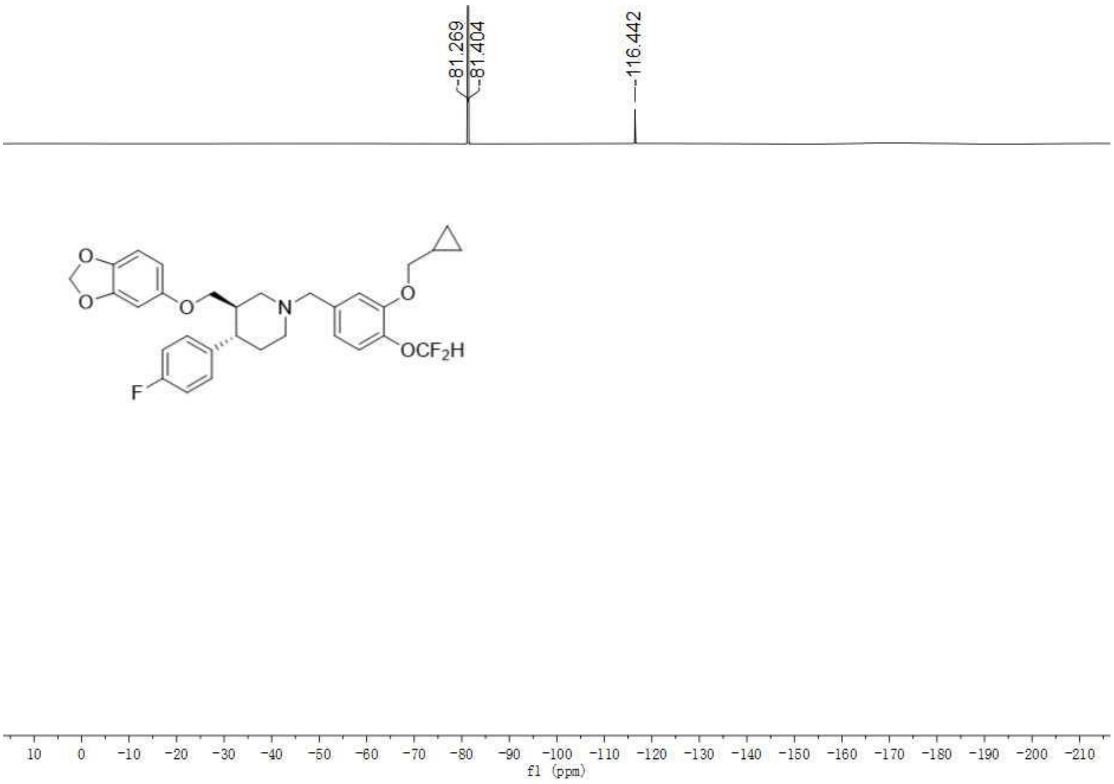

^{1}H NMR Spectrum of **70**

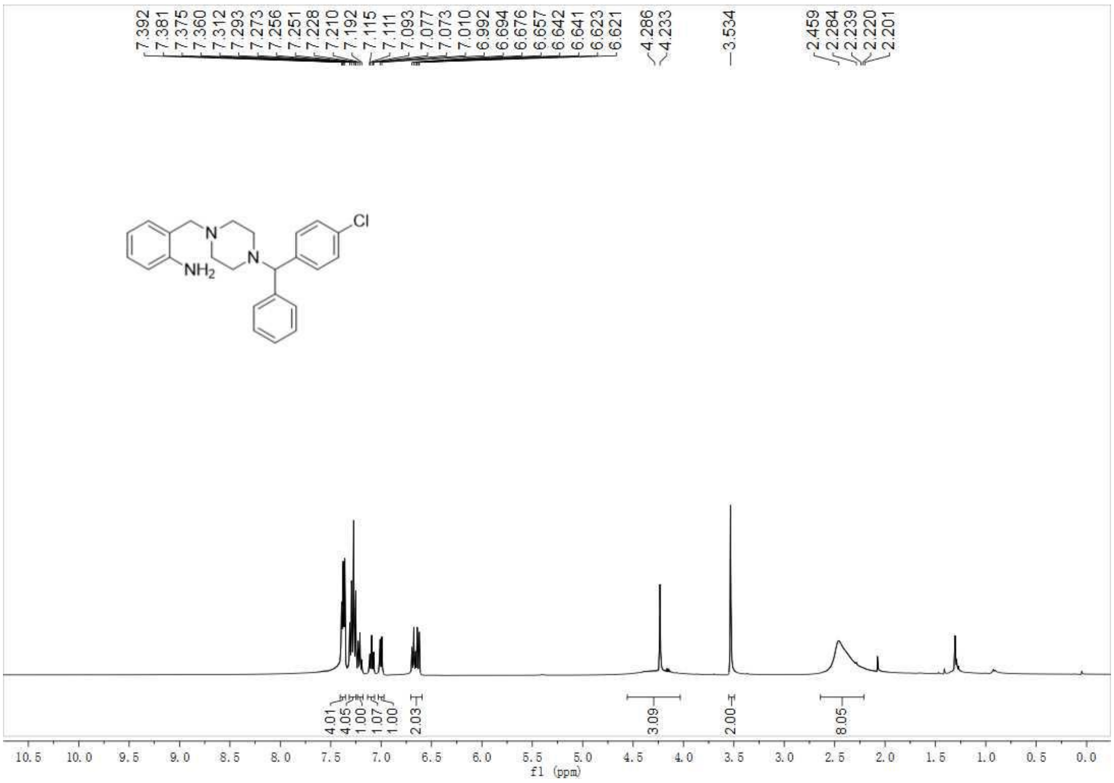

^{13}C NMR Spectrum of **70**

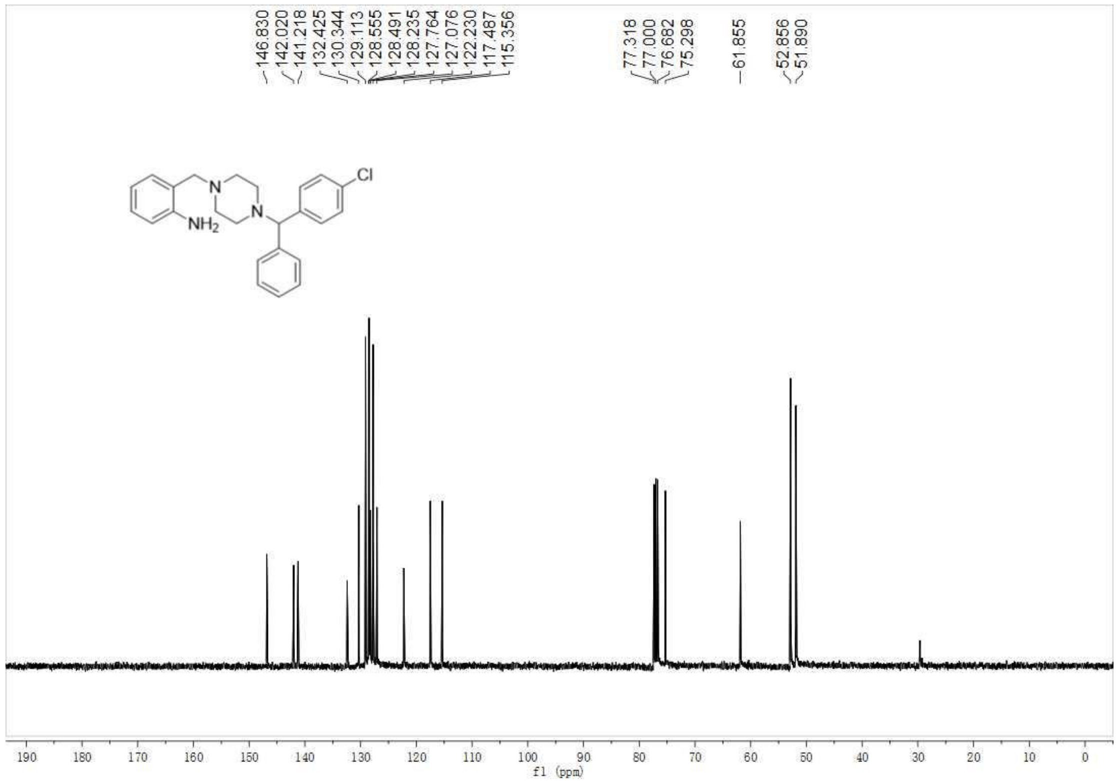

^{1}H NMR Spectrum of **71**

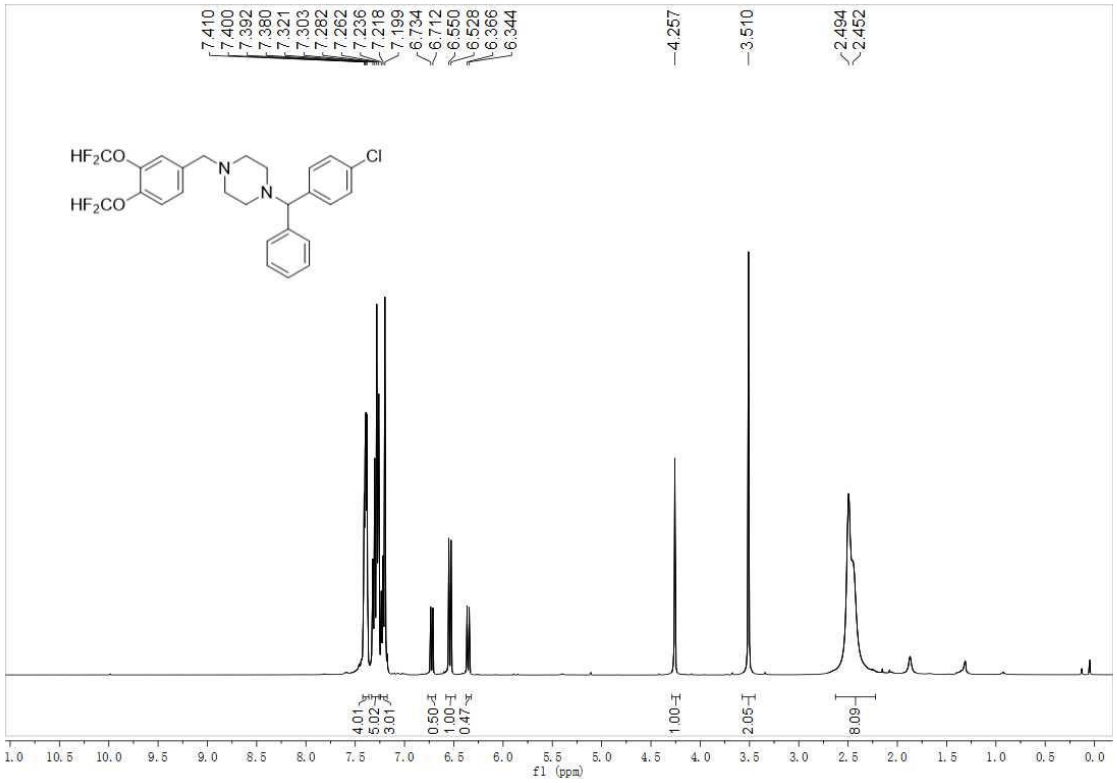

^{13}C NMR Spectrum of **71**

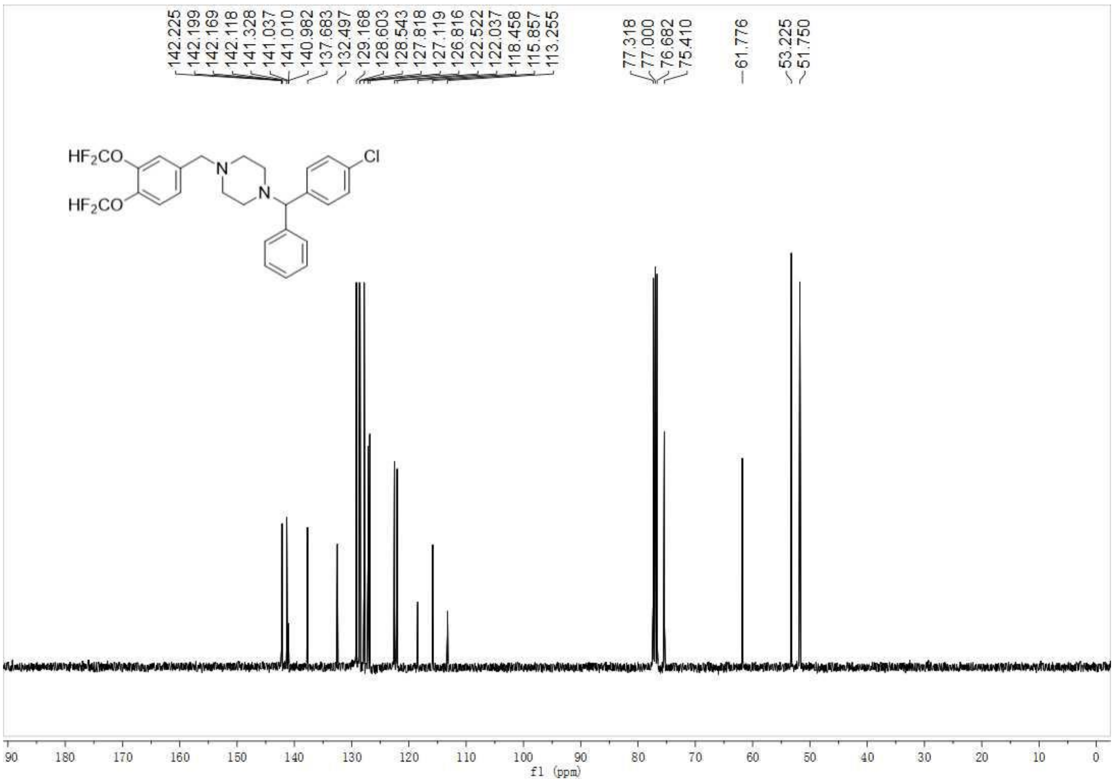

^{19}F NMR Spectrum of **71**

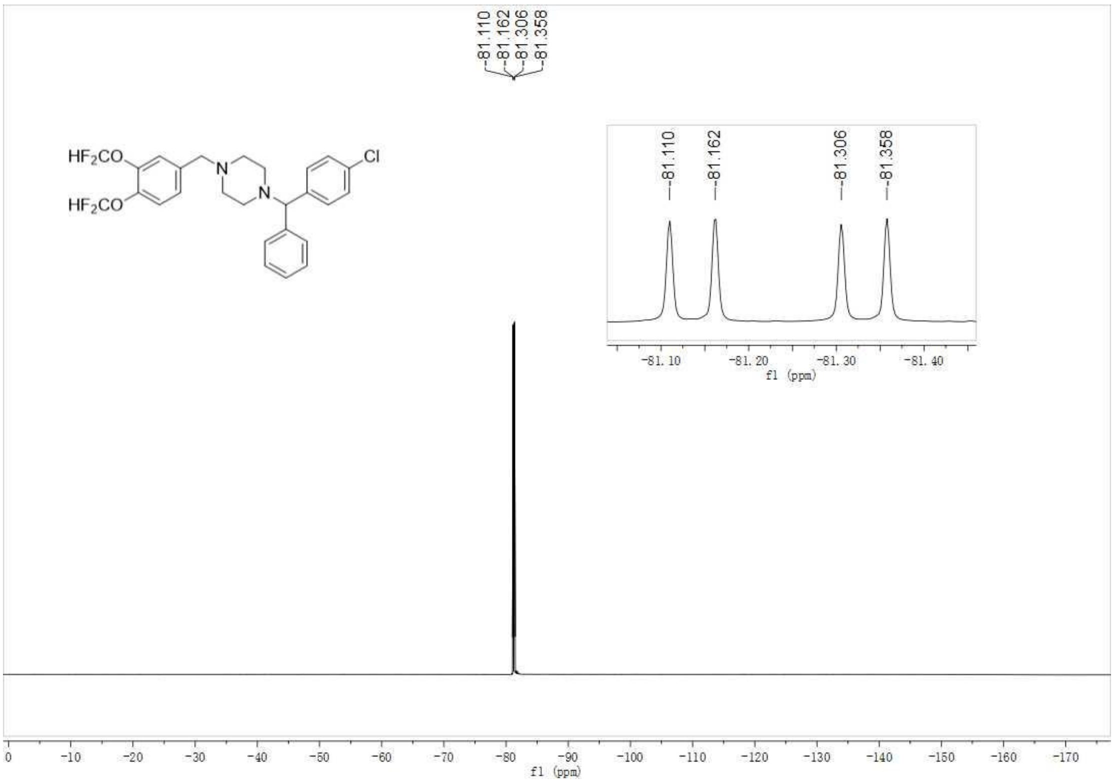

^{1}H NMR Spectrum of **72**

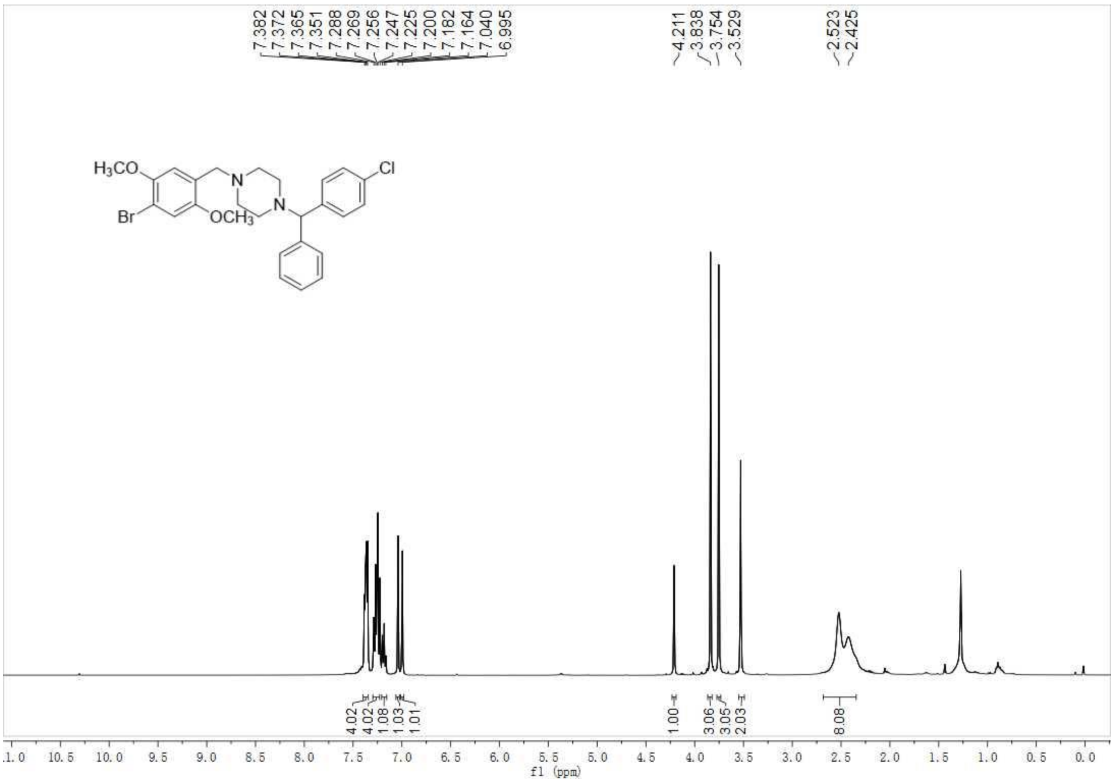

^{13}C NMR Spectrum of **72**

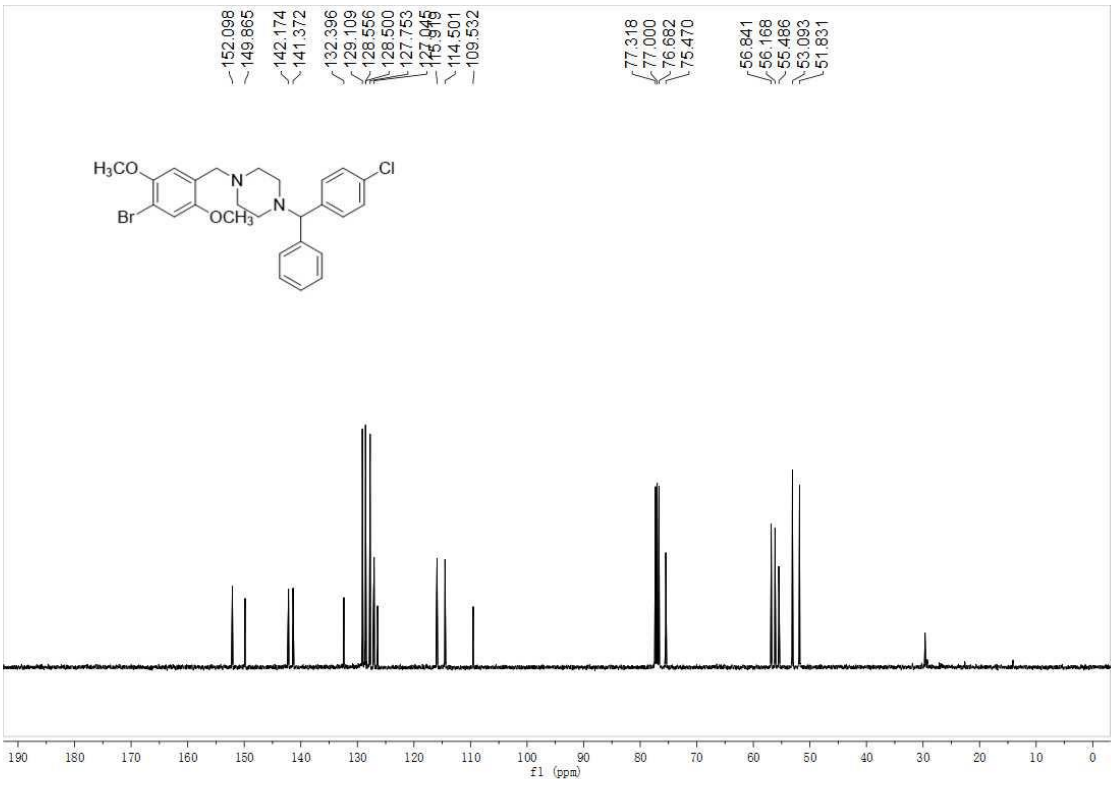

^{1}H NMR Spectrum of **73**

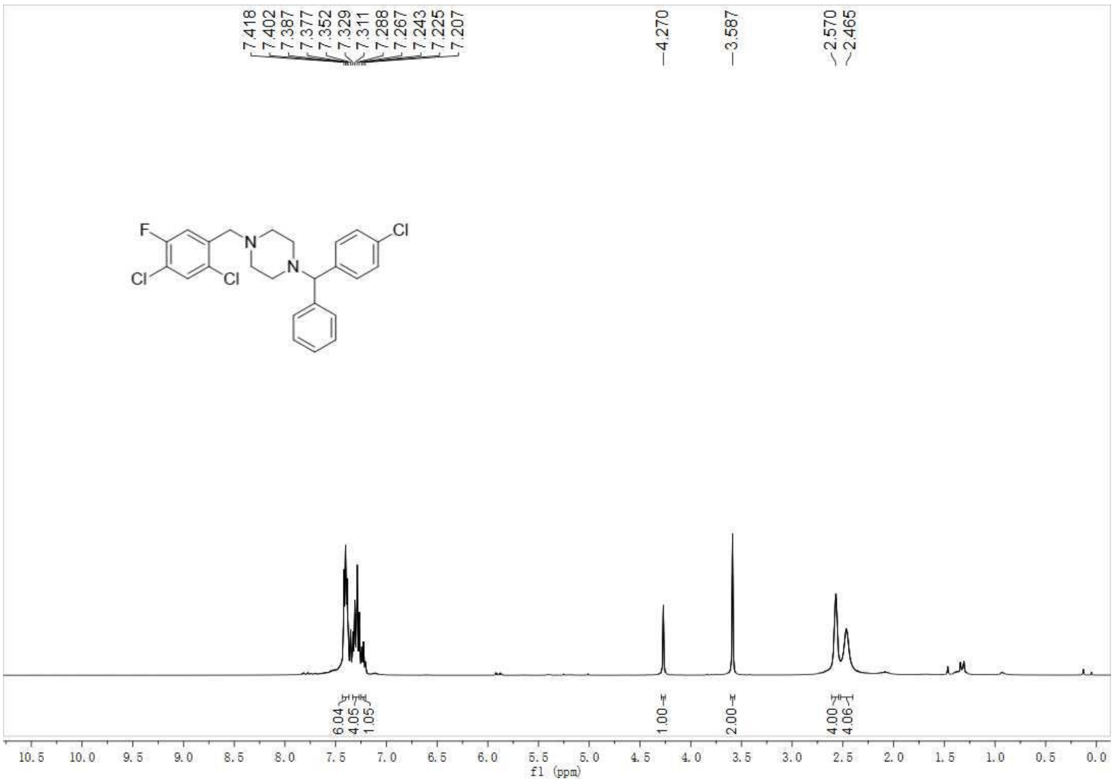

^{13}C NMR Spectrum of **73**

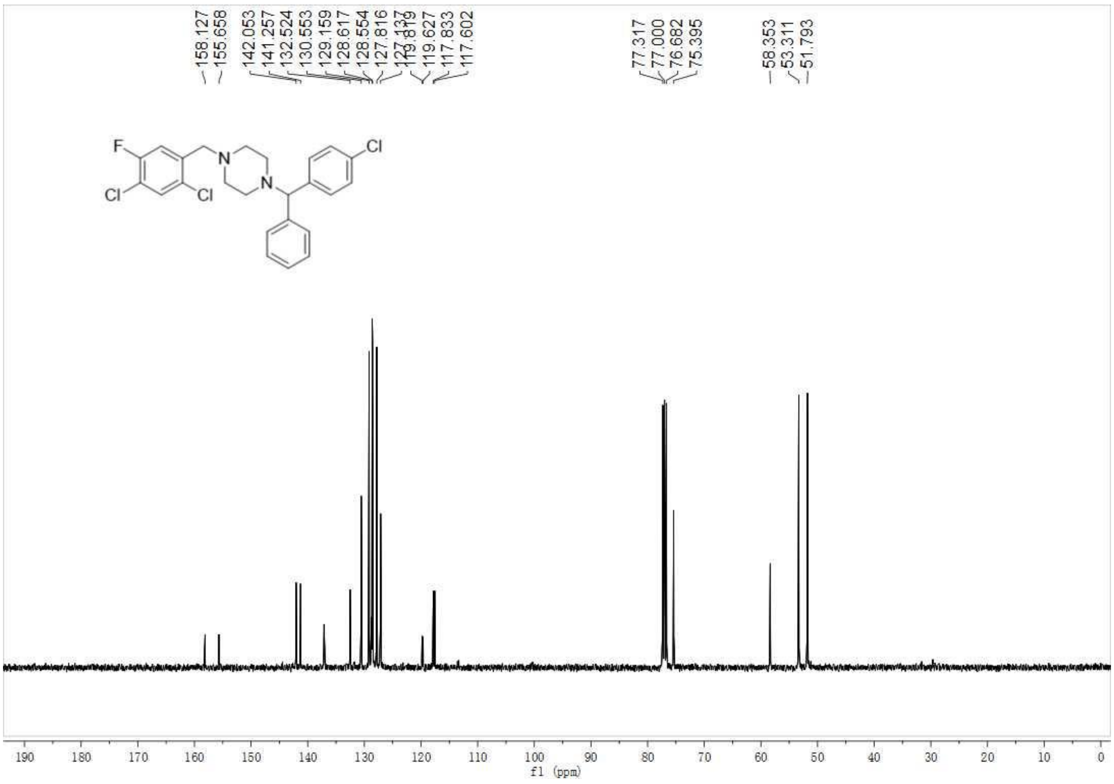

^{19}F NMR Spectrum of **73**

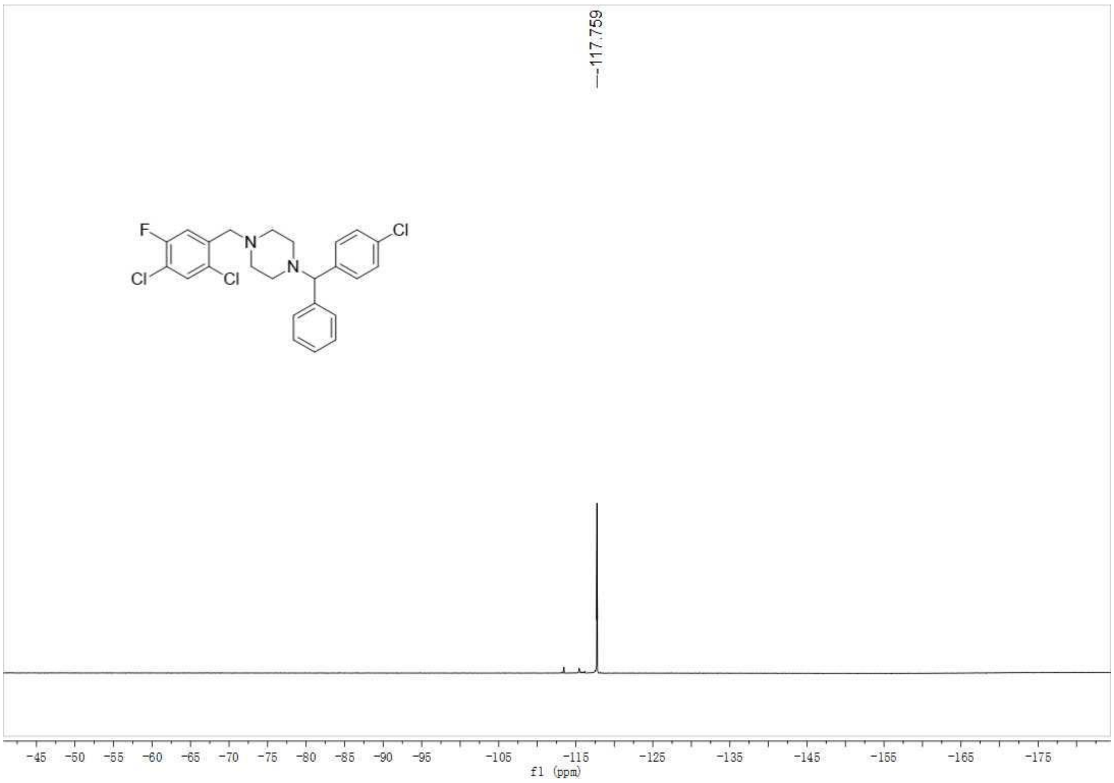

^{1}H NMR Spectrum of **74**

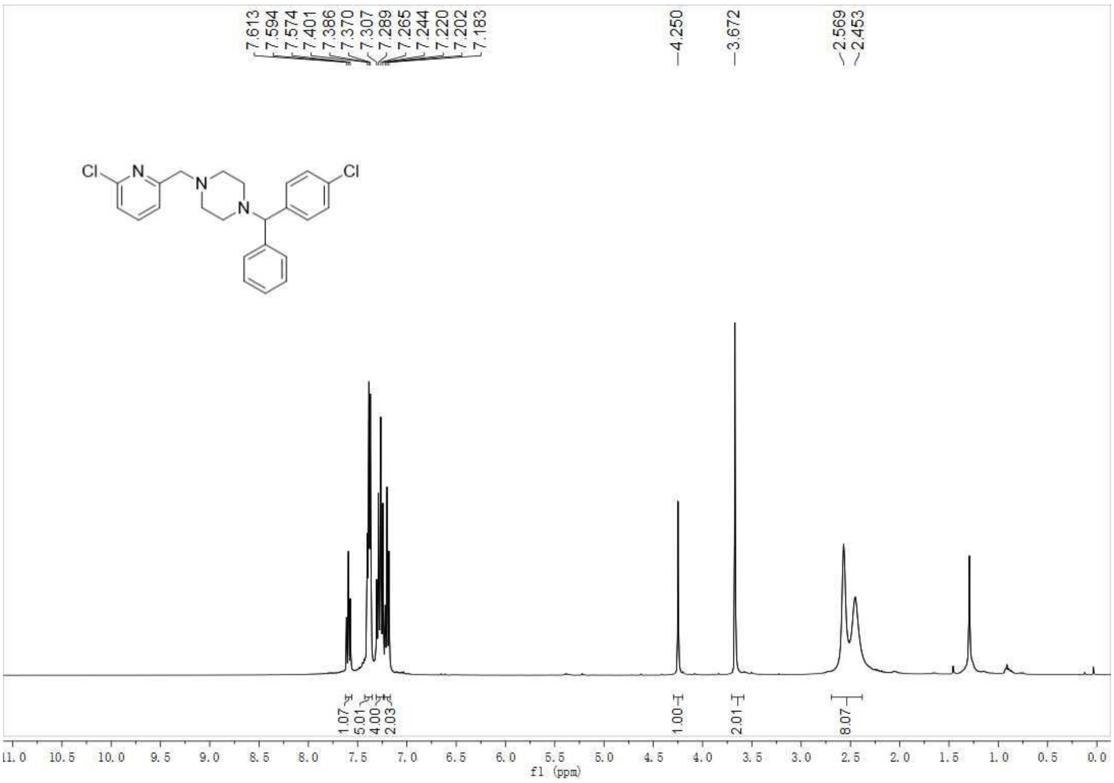

^{13}C NMR Spectrum of **74**

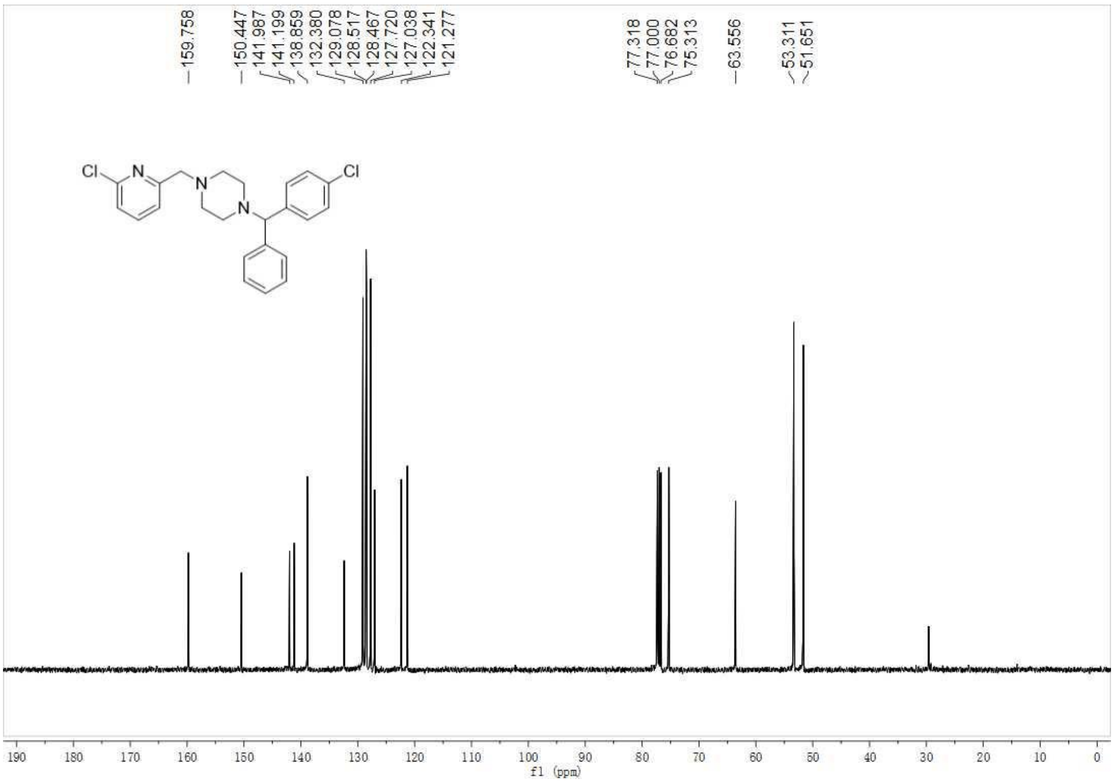

^{1}H NMR Spectrum of **75**

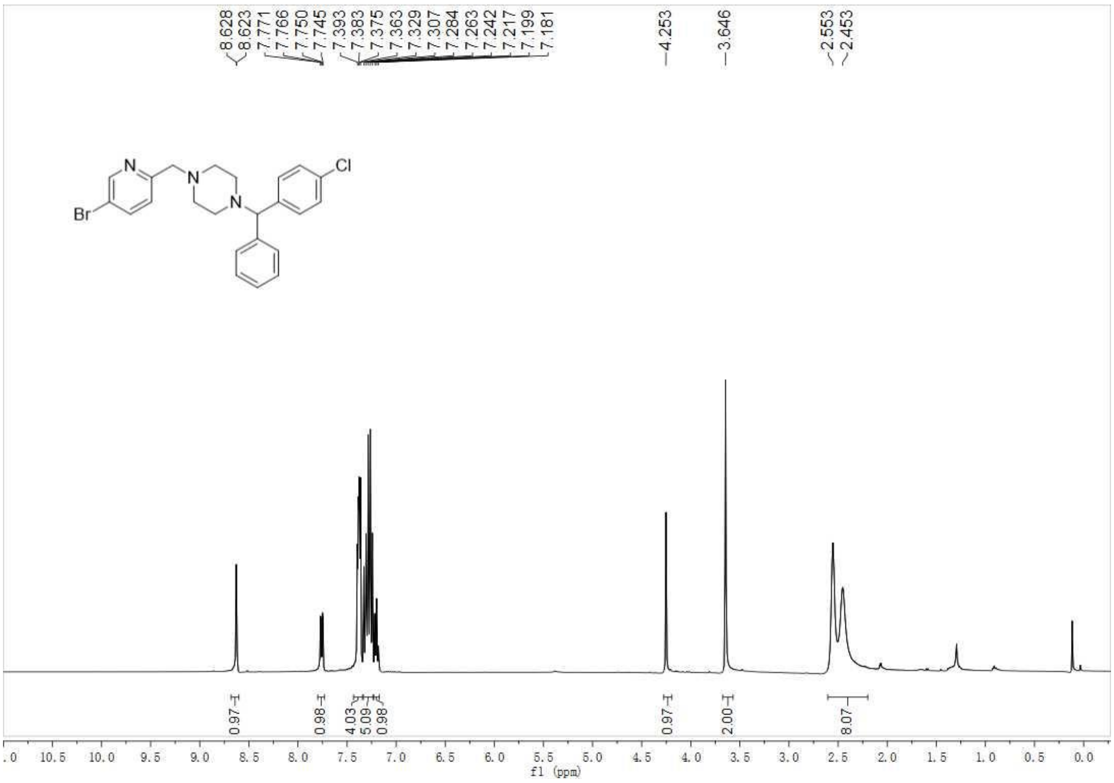

^{13}C NMR Spectrum of **75**

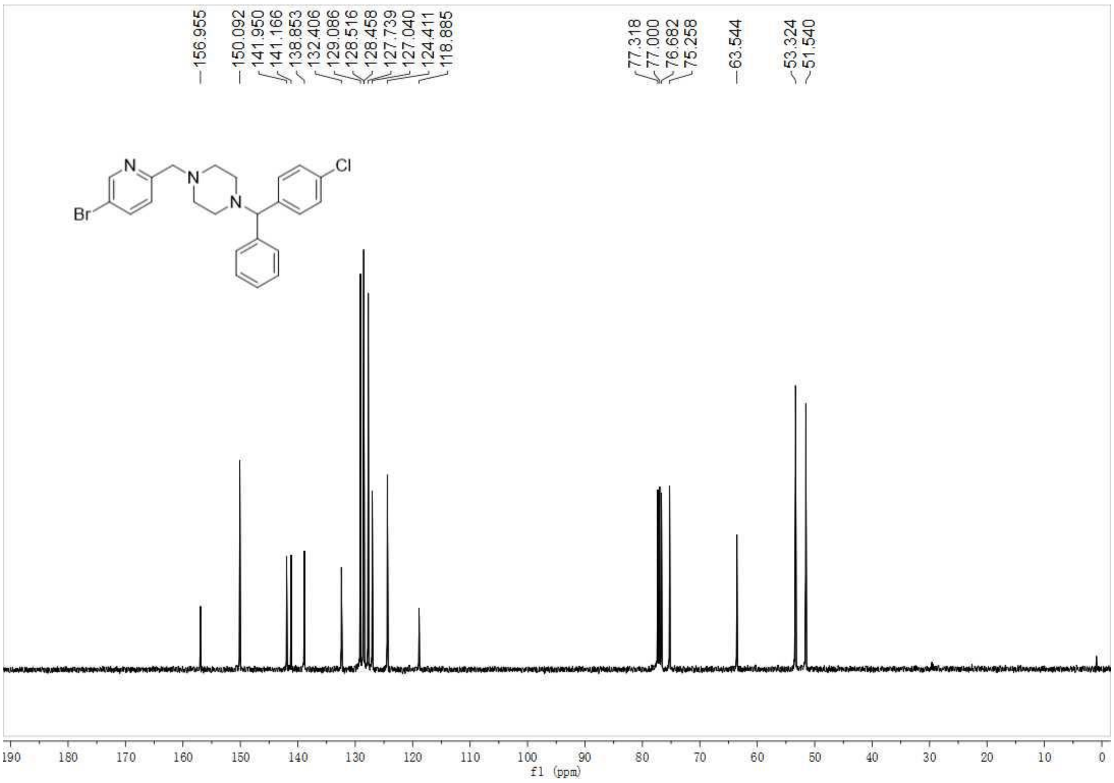

^{1}H NMR Spectrum of **76**

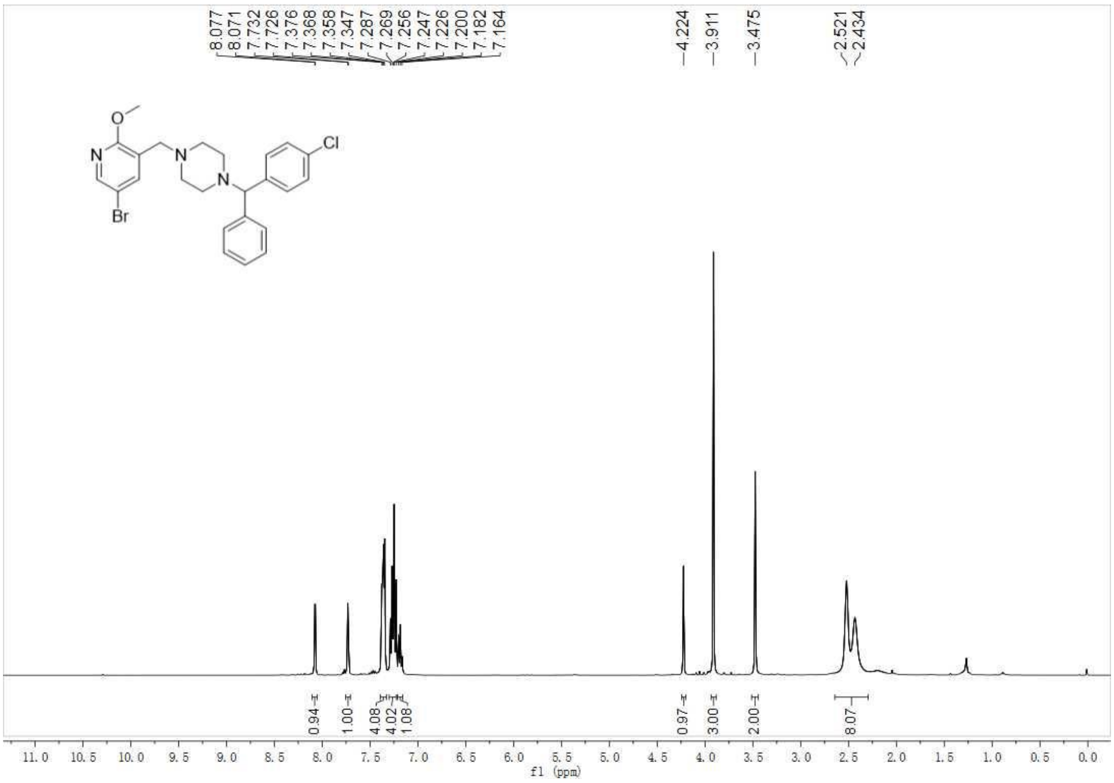

^{13}C NMR Spectrum of **76**

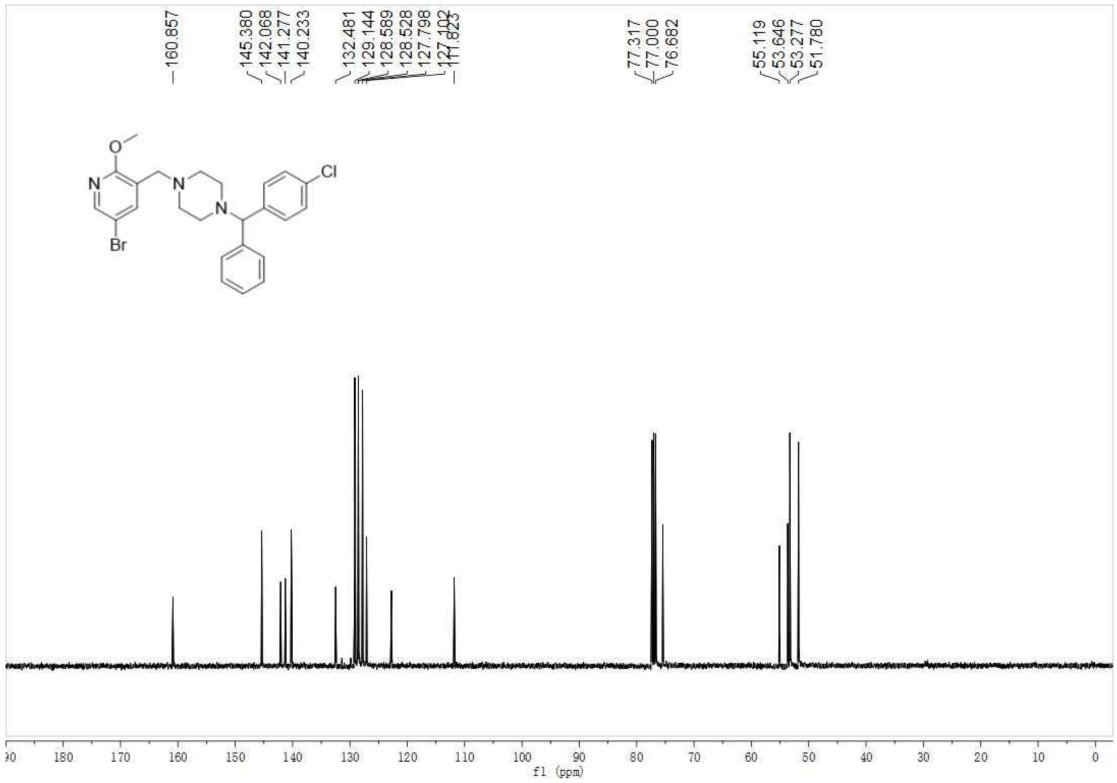

^{1}H NMR Spectrum of **77**

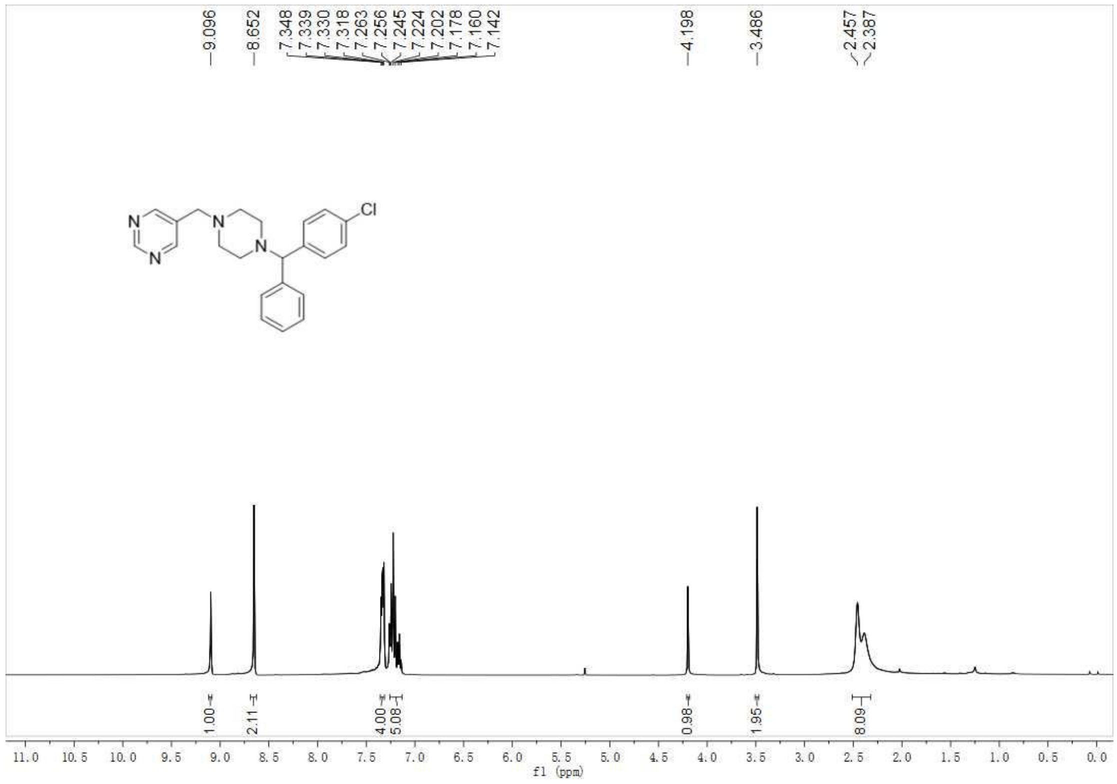

^{13}C NMR Spectrum of **77**

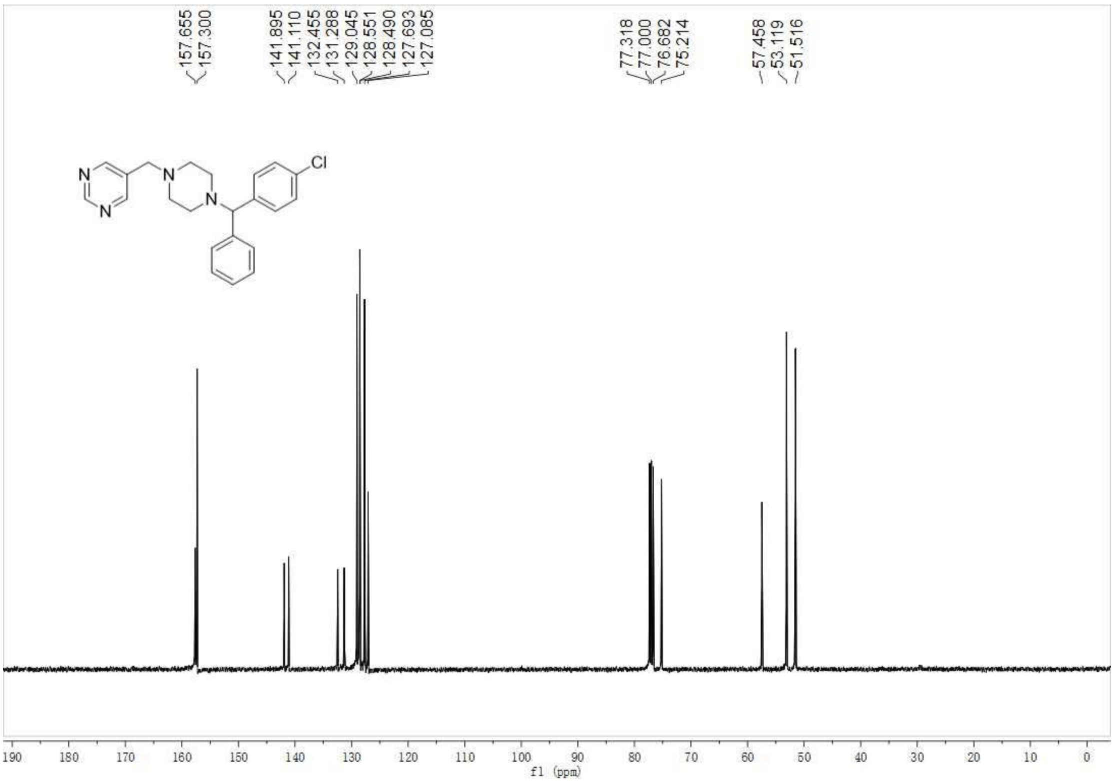

^{1}H NMR Spectrum of **78**

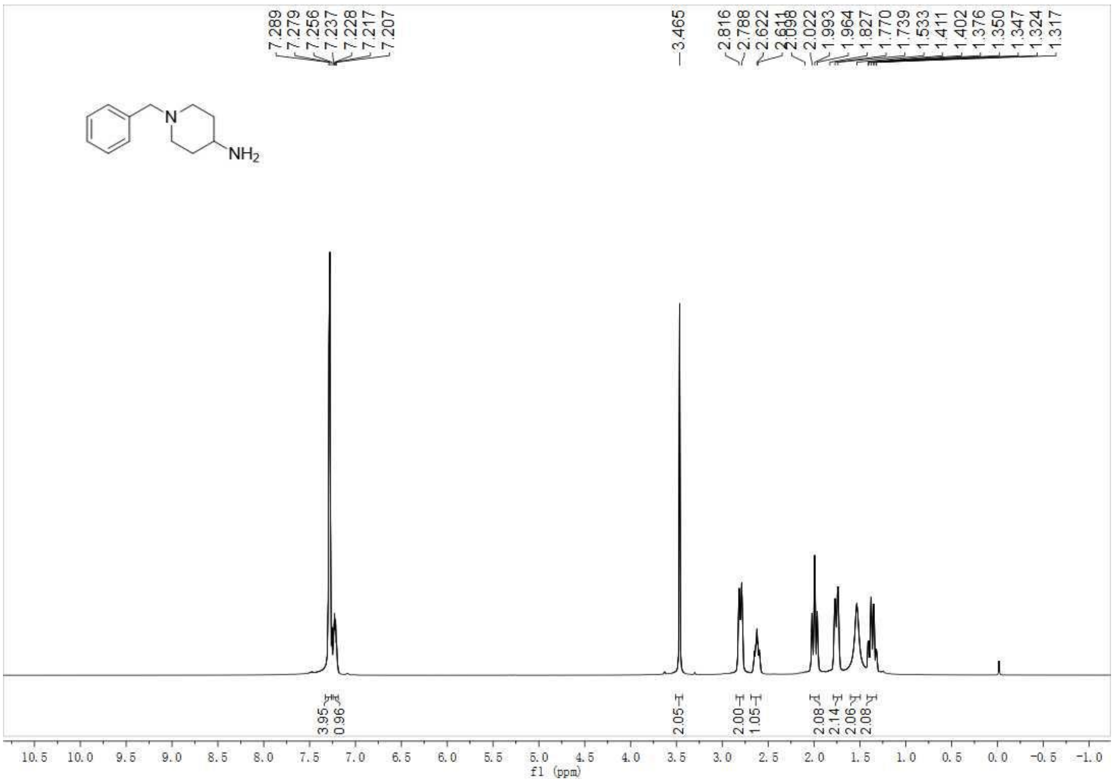

^{13}C NMR Spectrum of **78**

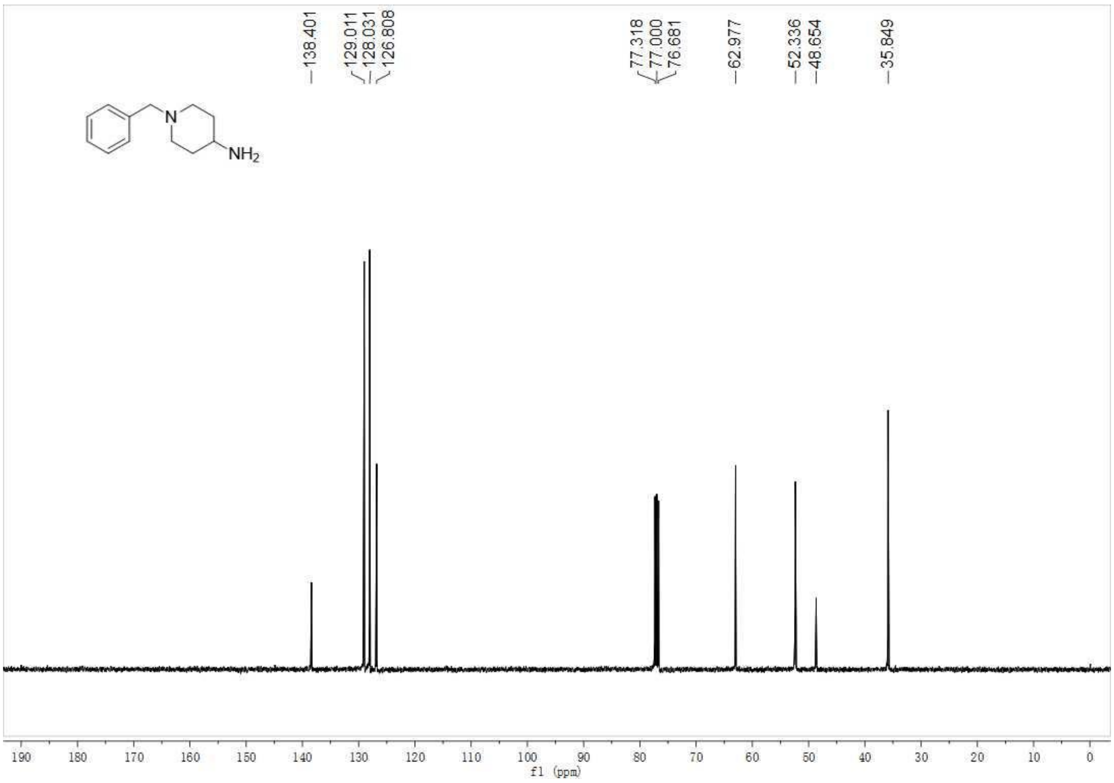

^{1}H NMR Spectrum of **79**

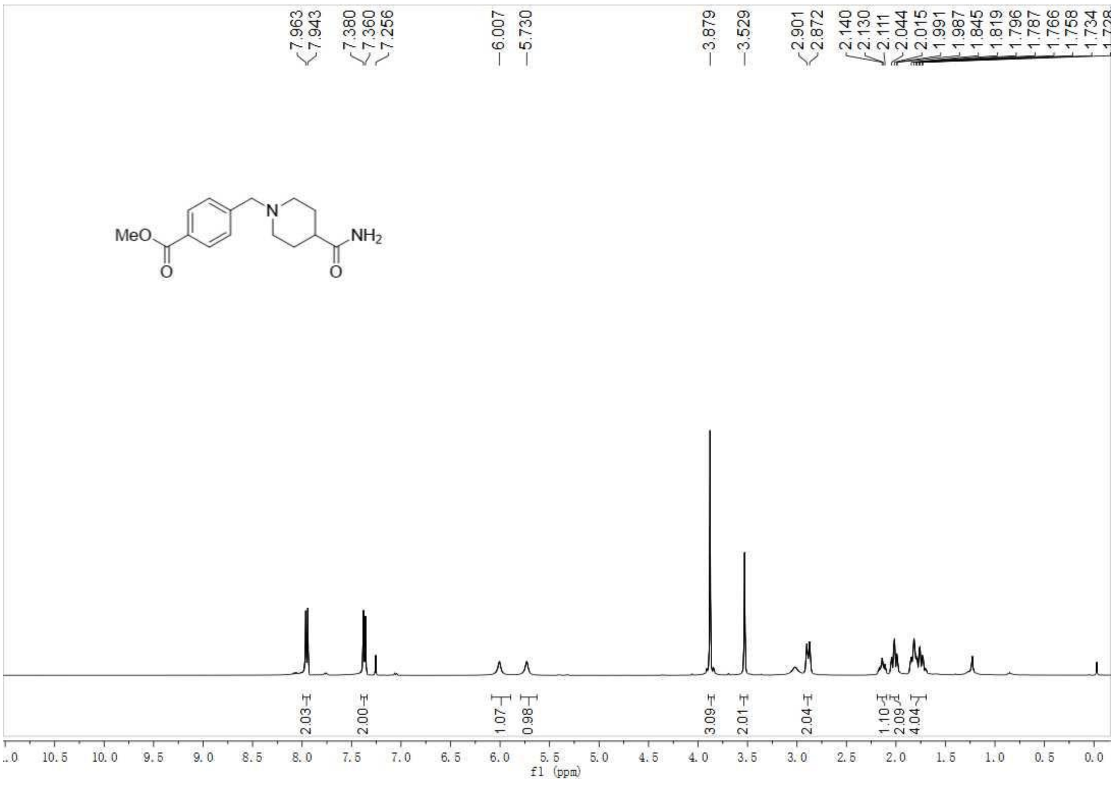

^{13}C NMR Spectrum of **79**

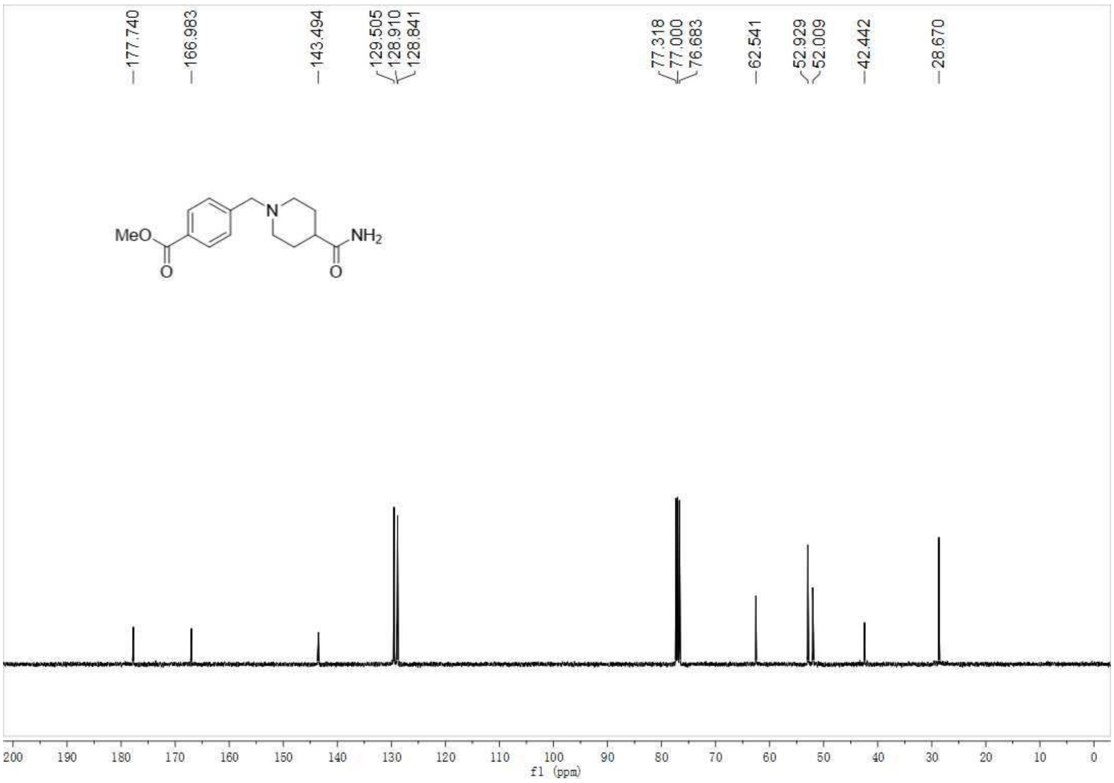

^{1}H NMR Spectrum of **80**

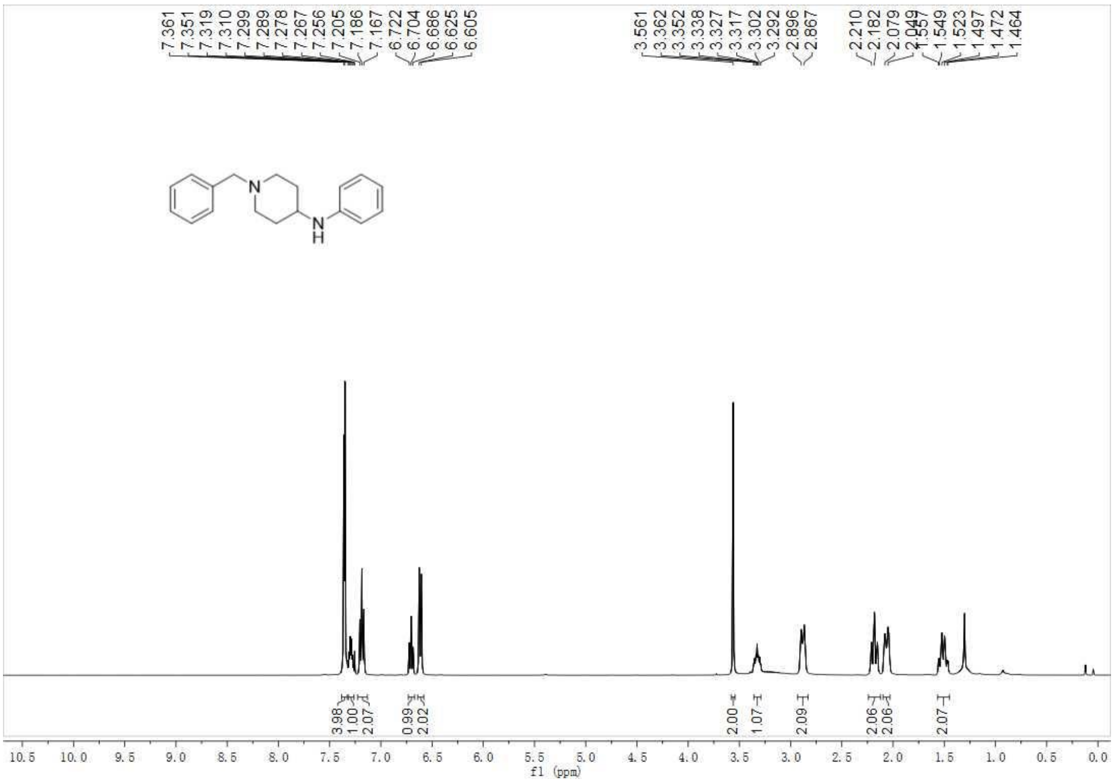

^{13}C NMR Spectrum of **80**

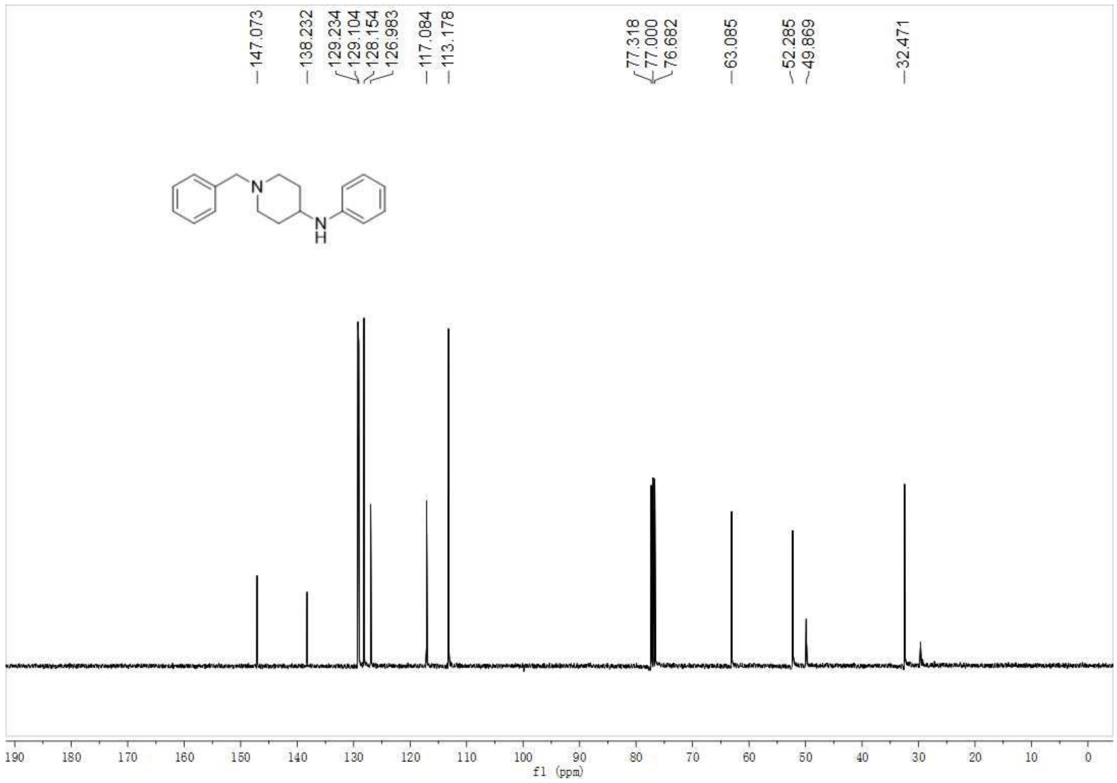

^{1}H NMR Spectrum of **81**

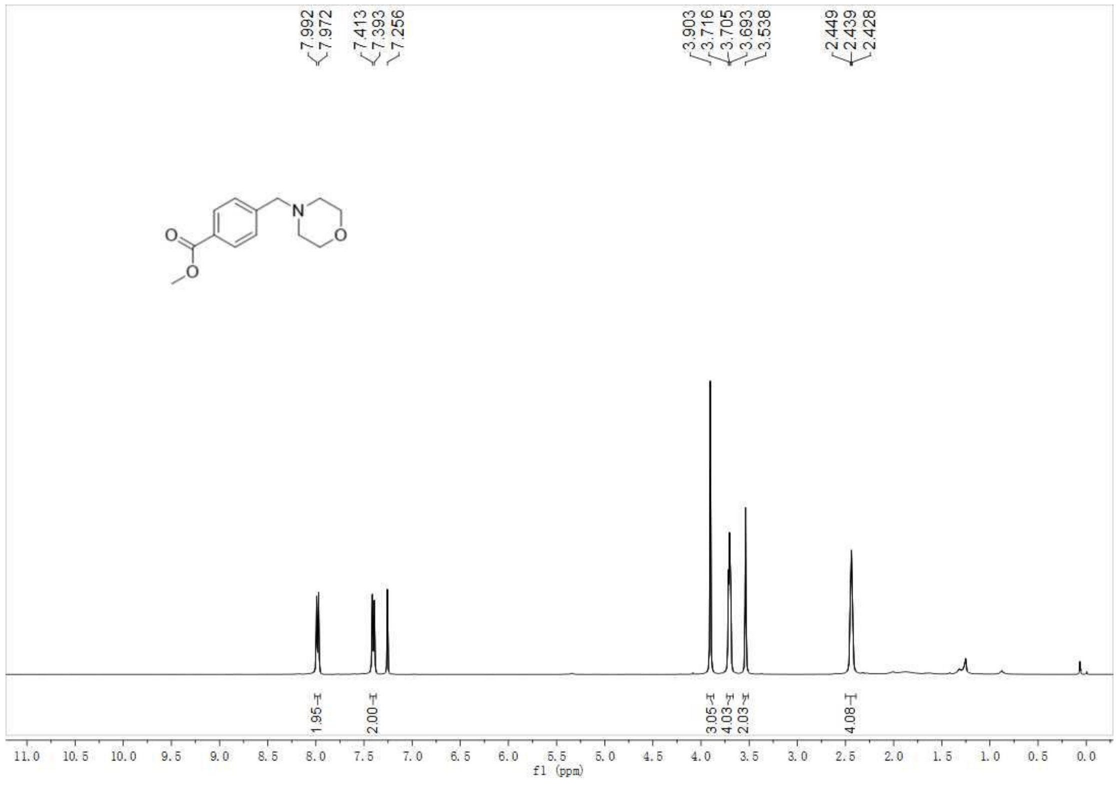

^{13}C NMR Spectrum of **81**

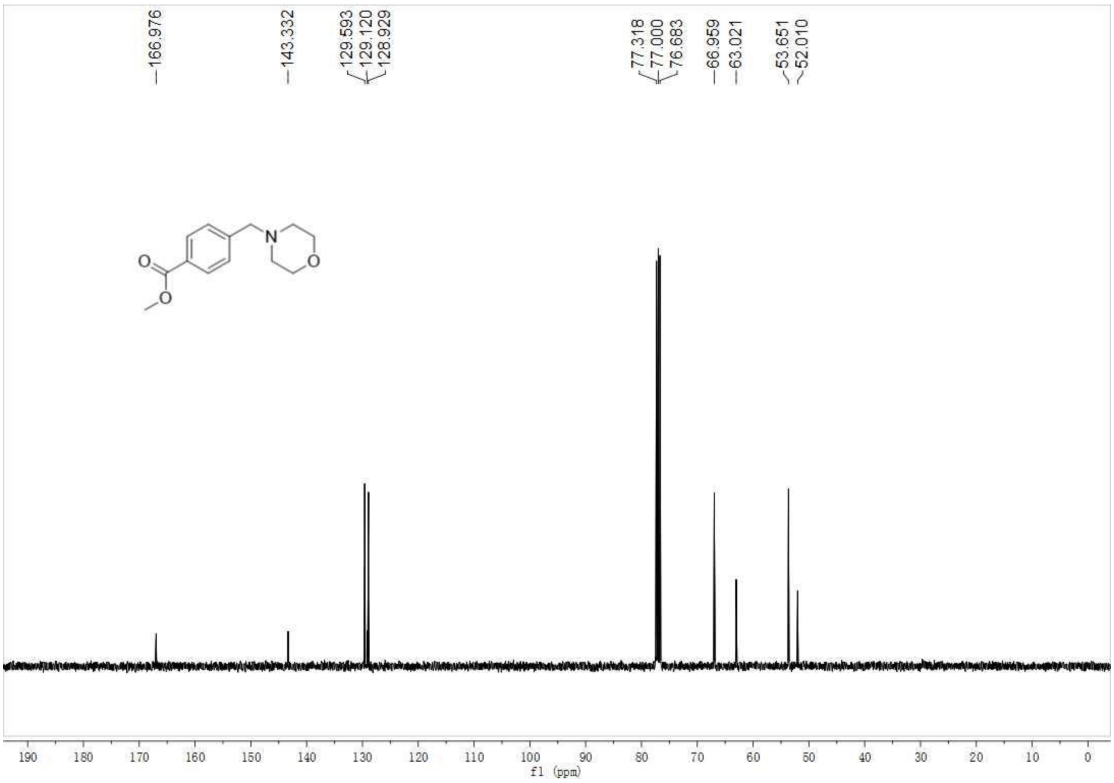

^{1}H NMR Spectrum of **82**

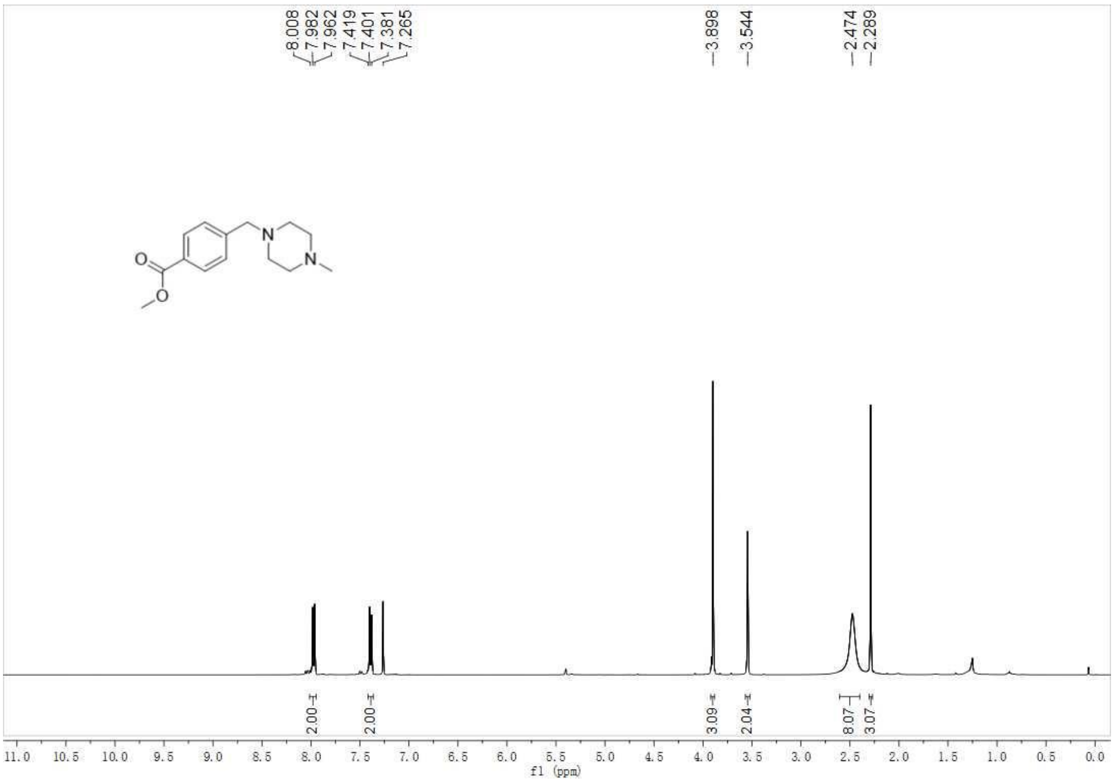

^{13}C NMR Spectrum of **82**

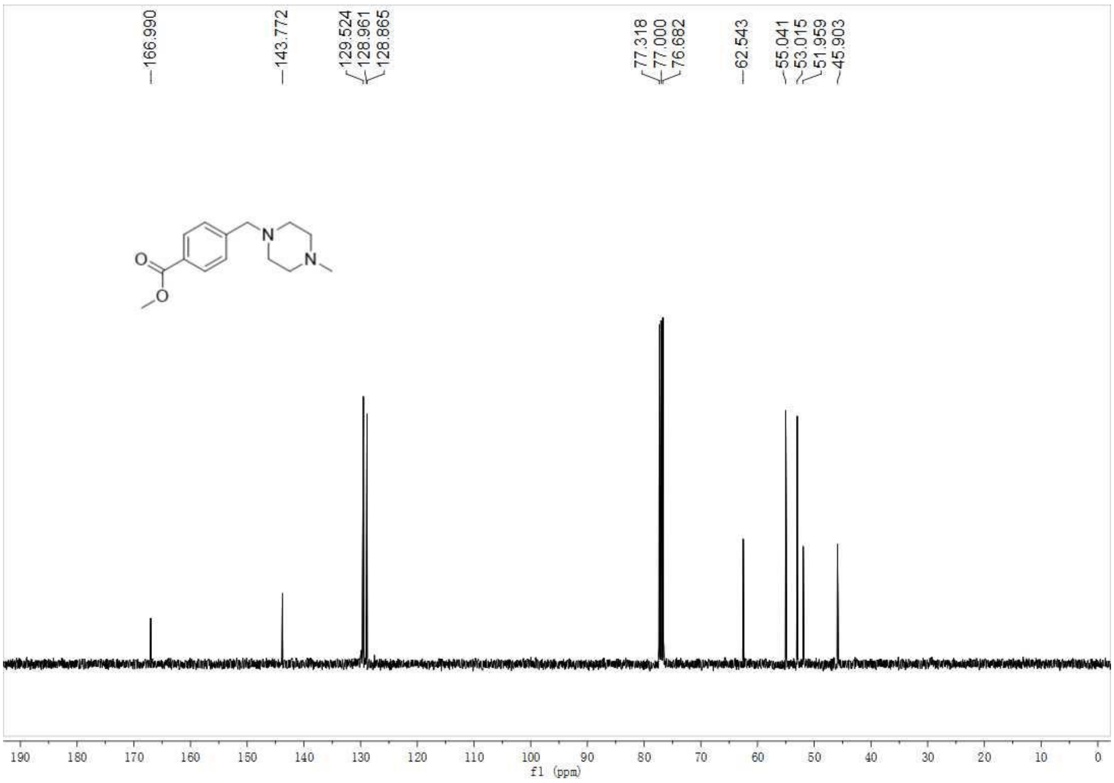

^{1}H NMR Spectrum of **83**

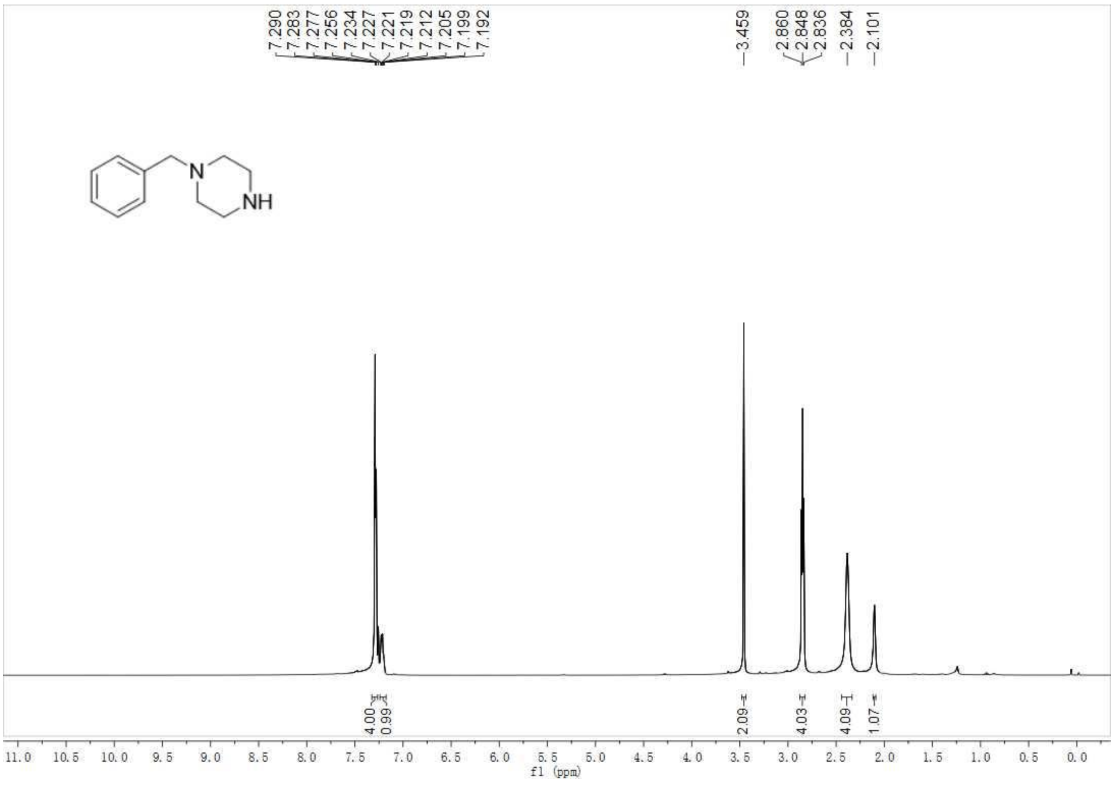

^{13}C NMR Spectrum of **83**

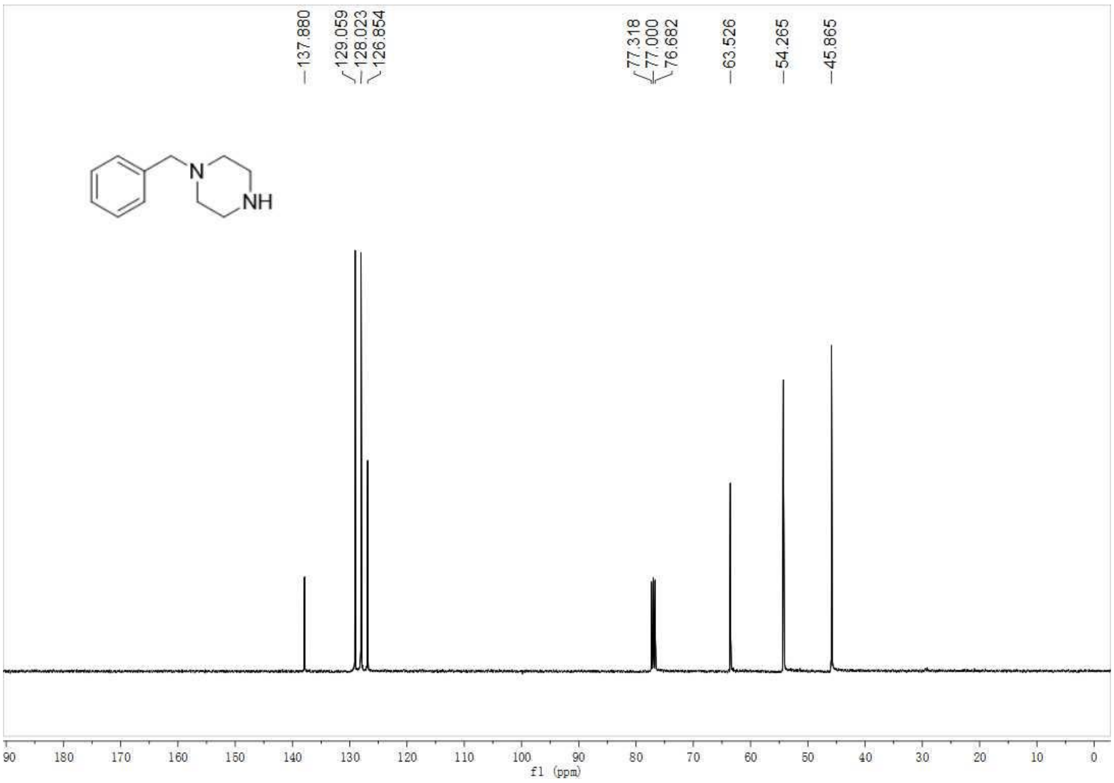

^{1}H NMR Spectrum of **84**

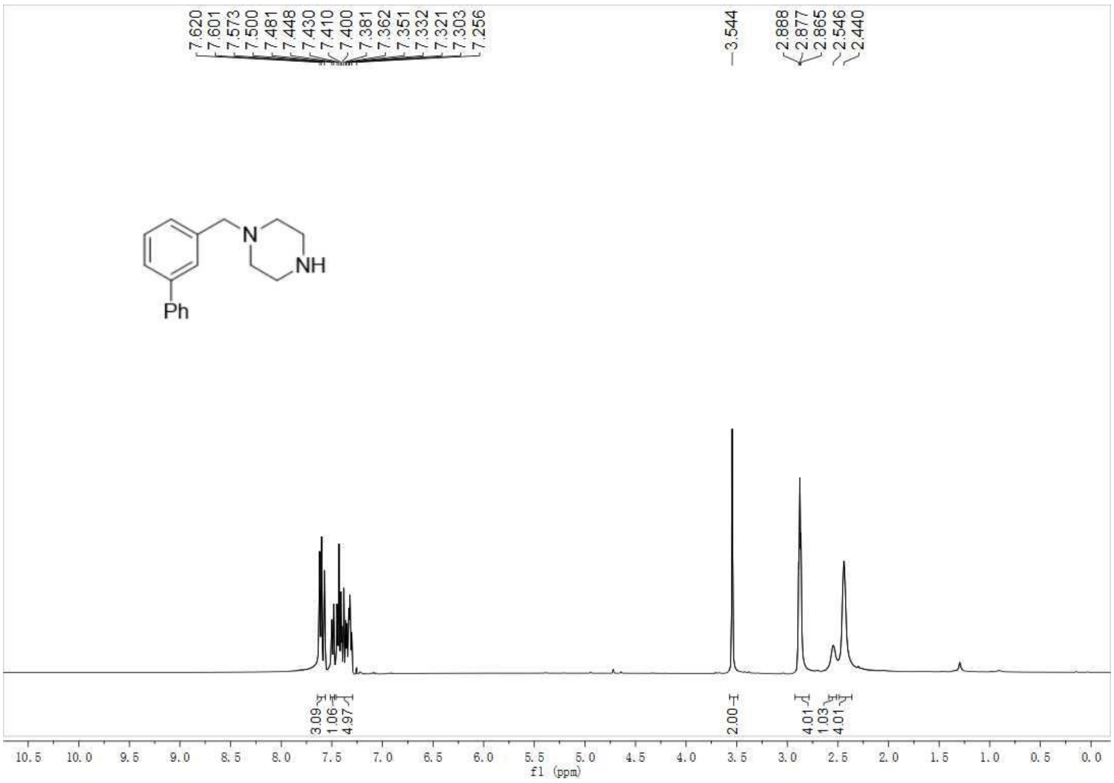

^{13}C NMR Spectrum of **84**

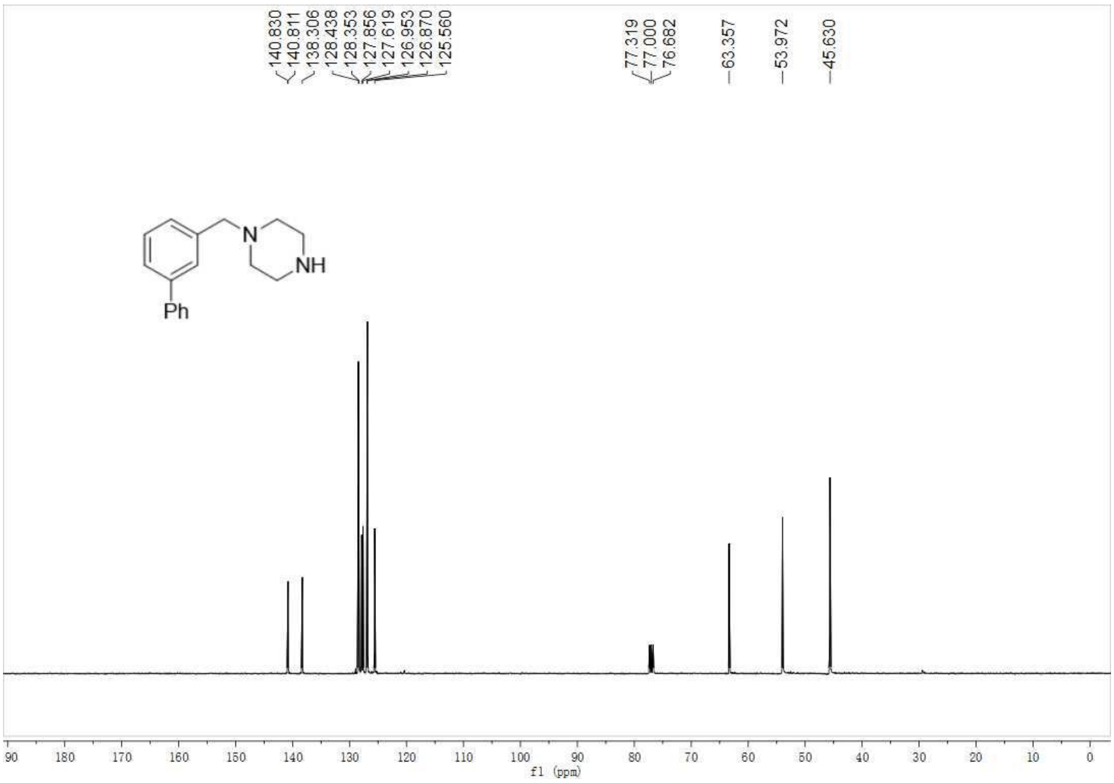

^{1}H NMR Spectrum of **85**

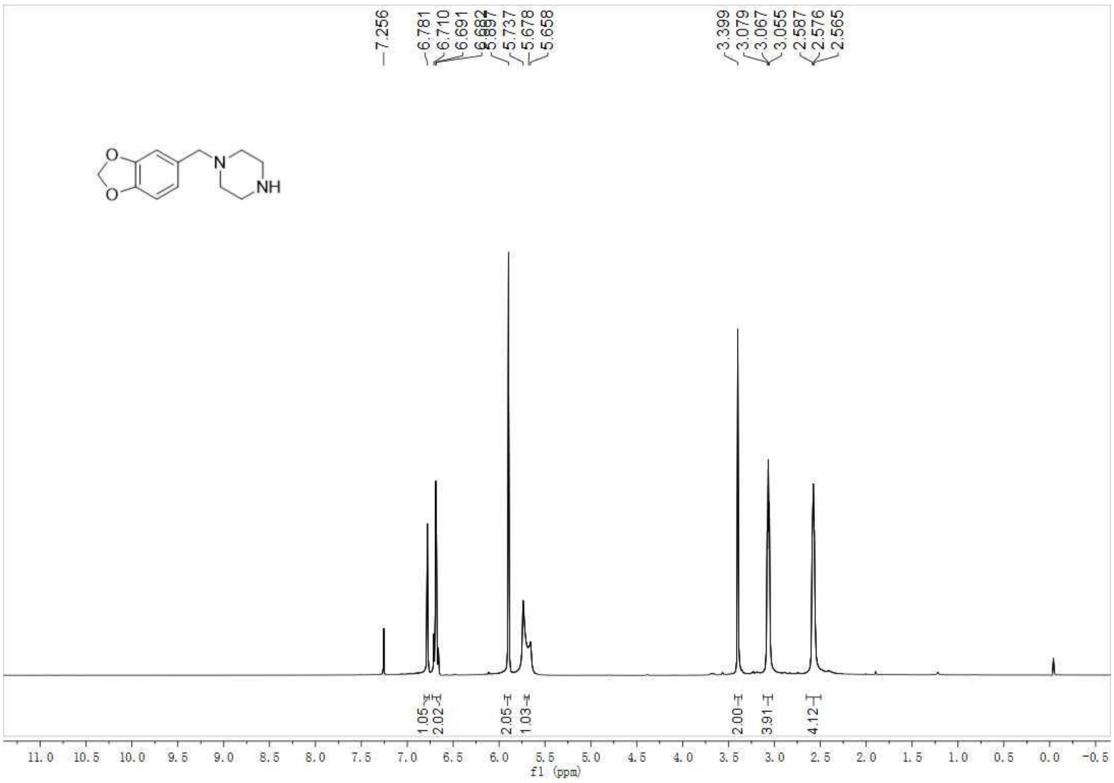

^{13}C NMR Spectrum of **85**

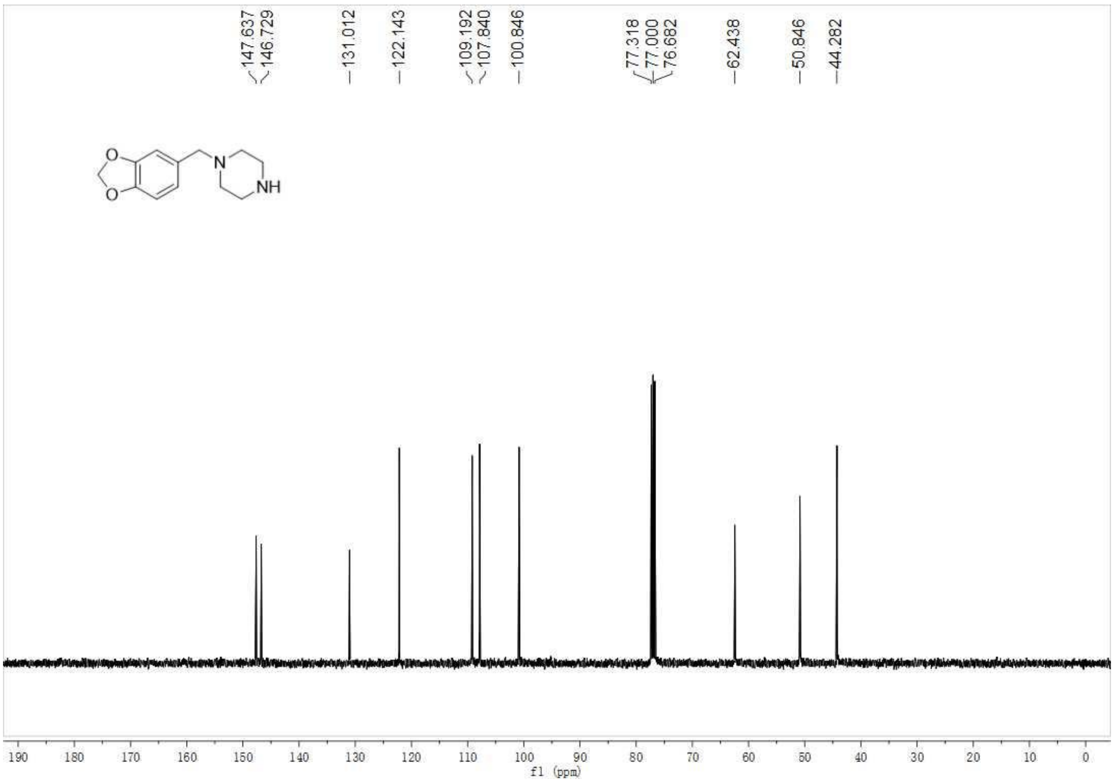

^{1}H NMR Spectrum of **86**

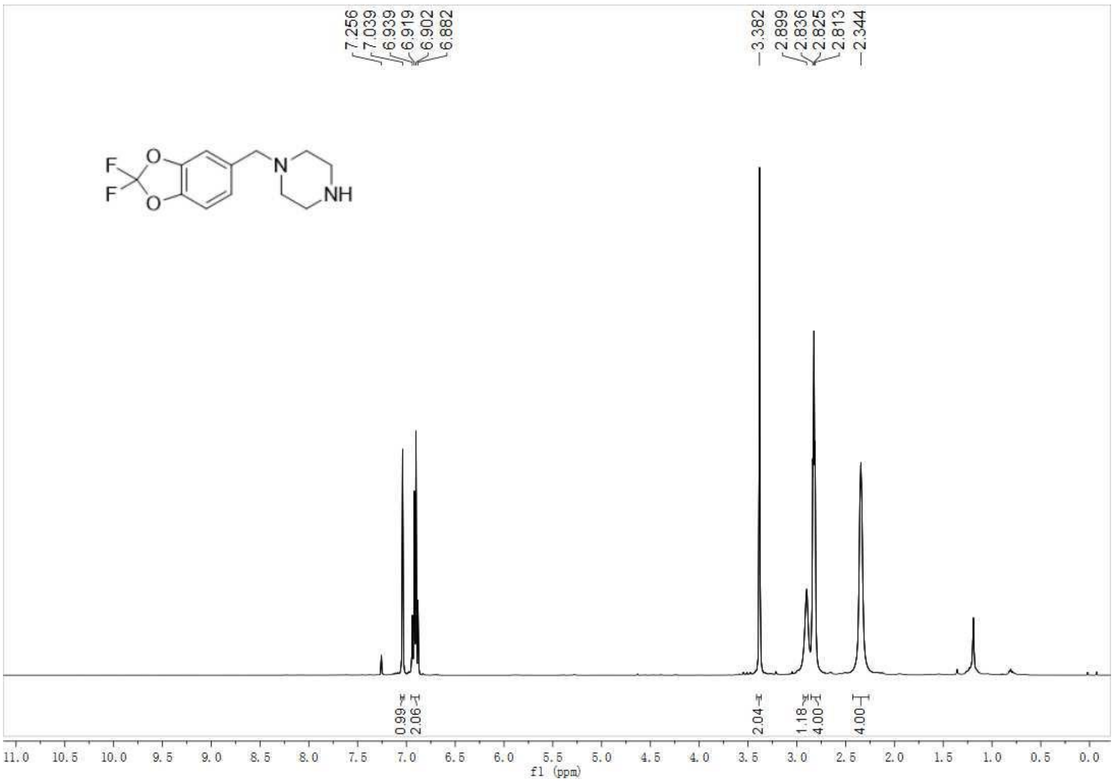

^{13}C NMR Spectrum of **86**

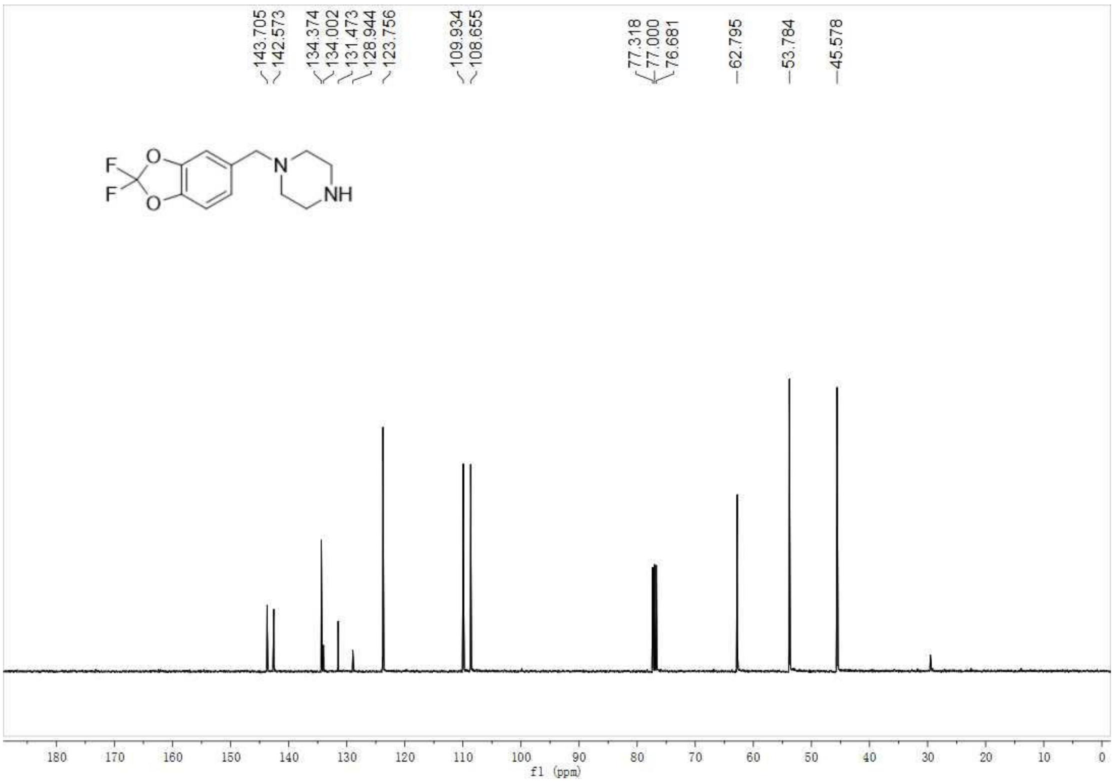

^{19}F NMR Spectrum of **86**

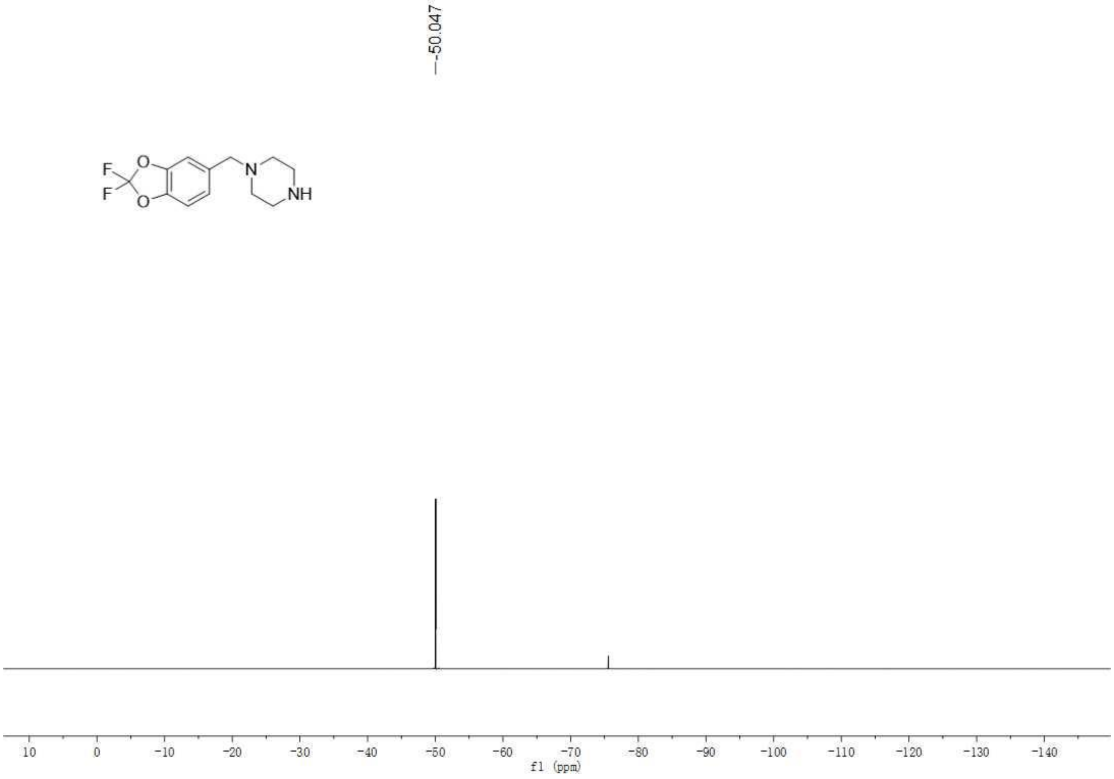

^{1}H NMR Spectrum of **87**

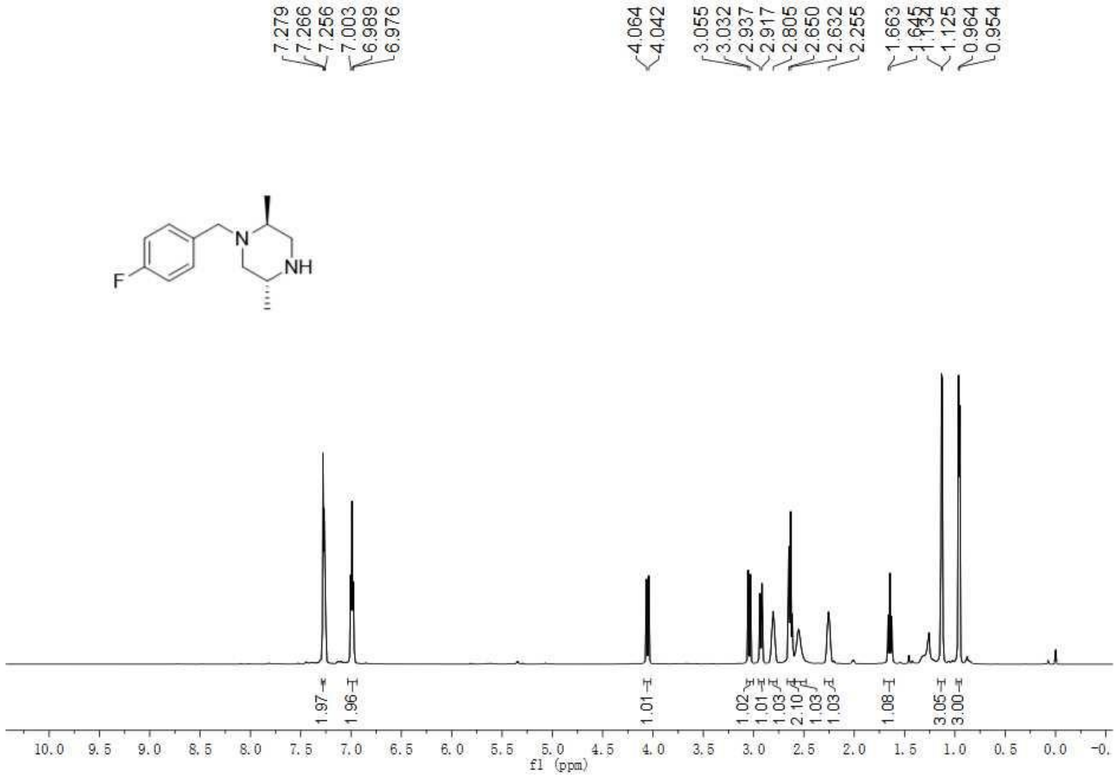

^{13}C NMR Spectrum of **87**

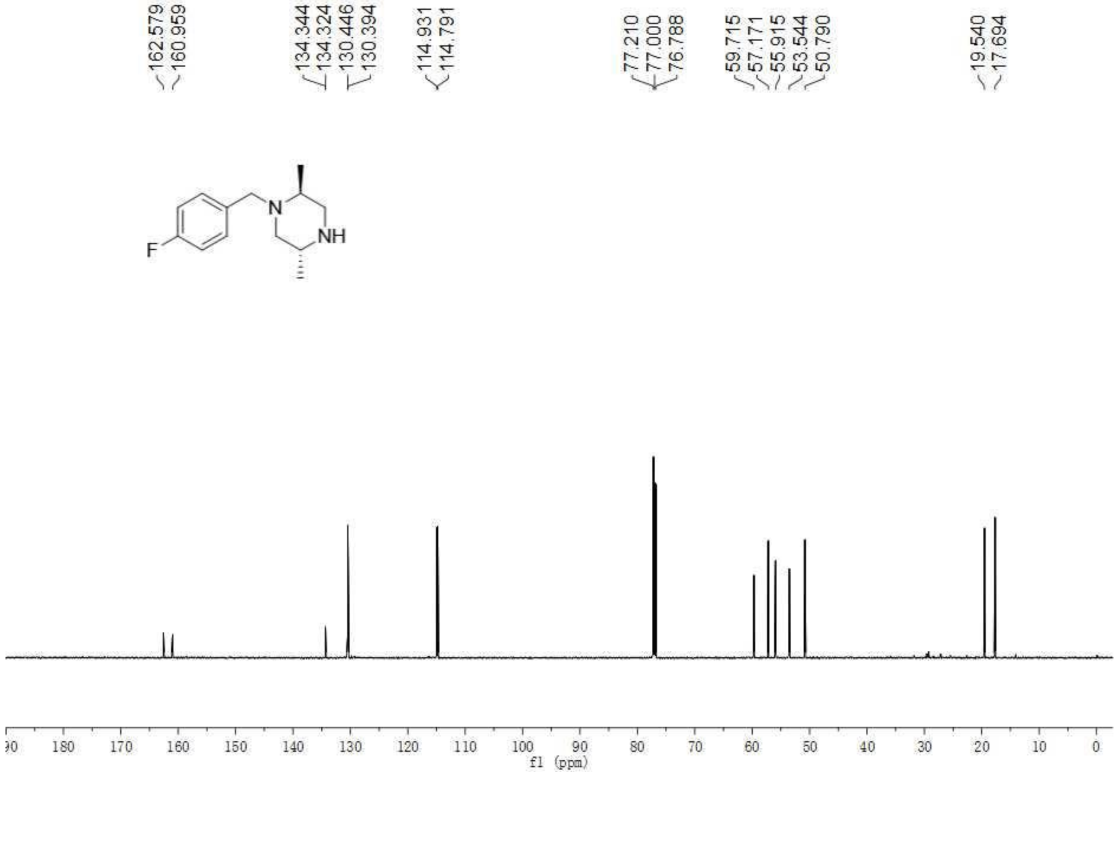

^{19}F NMR Spectrum of **87**

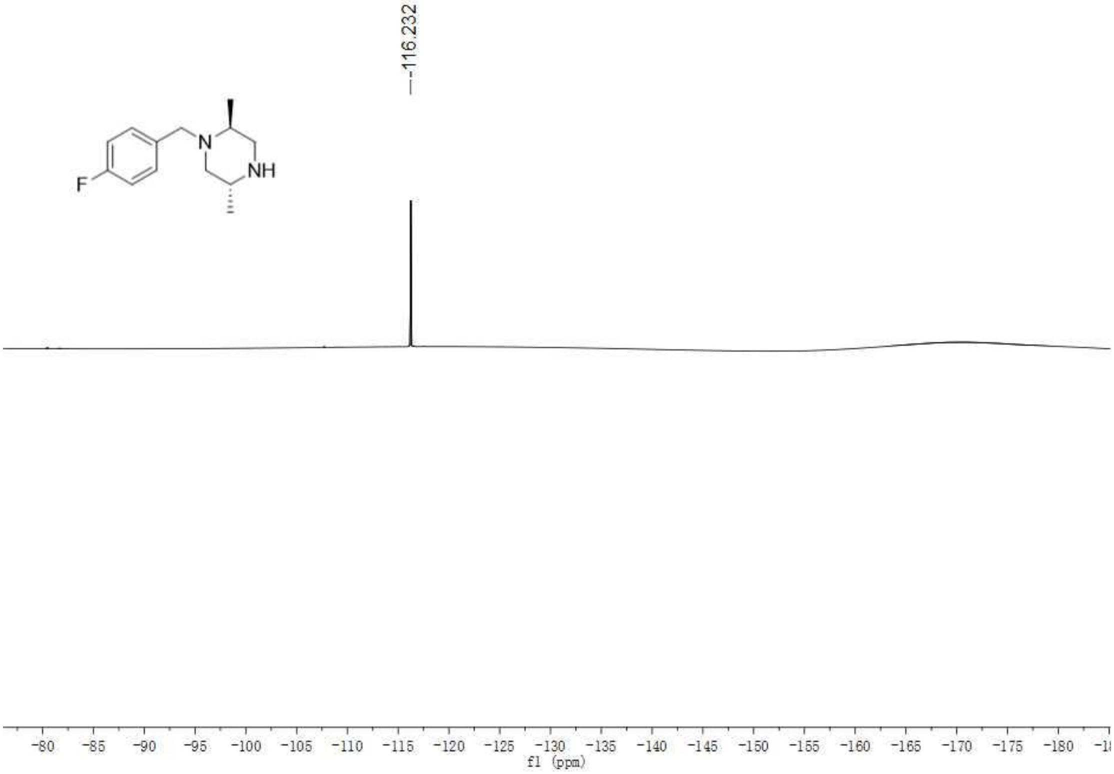

^{1}H NMR Spectrum of **88**

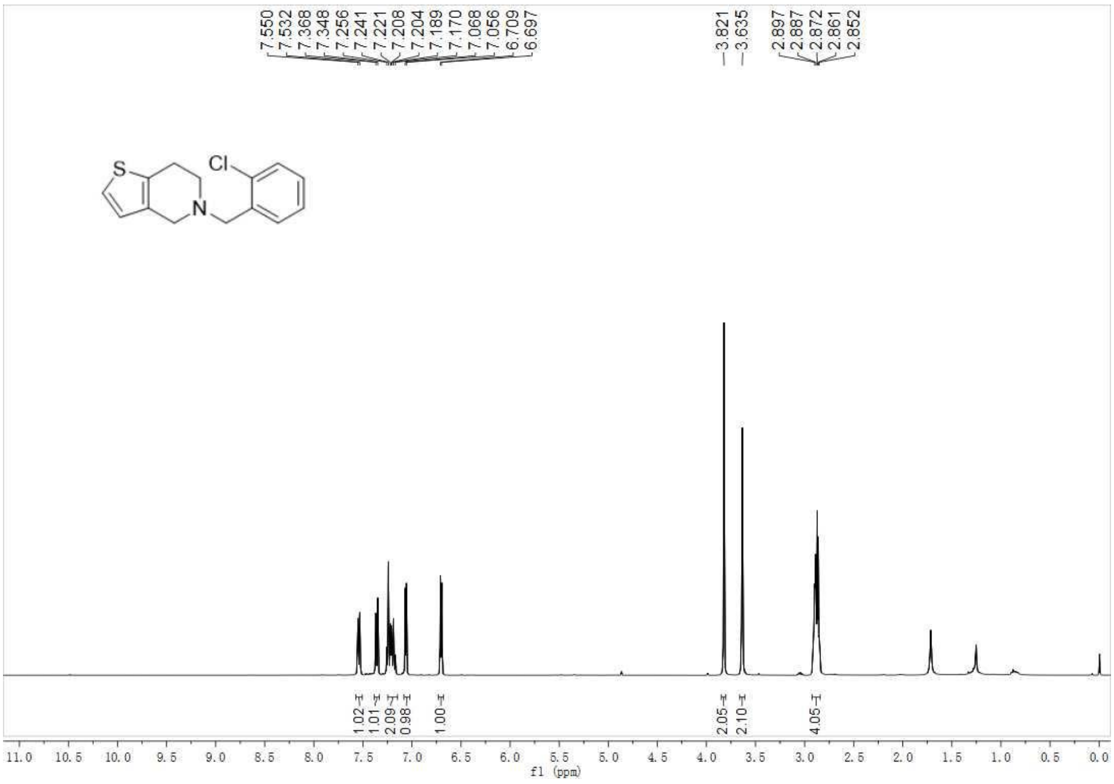

^{13}C NMR Spectrum of **88**

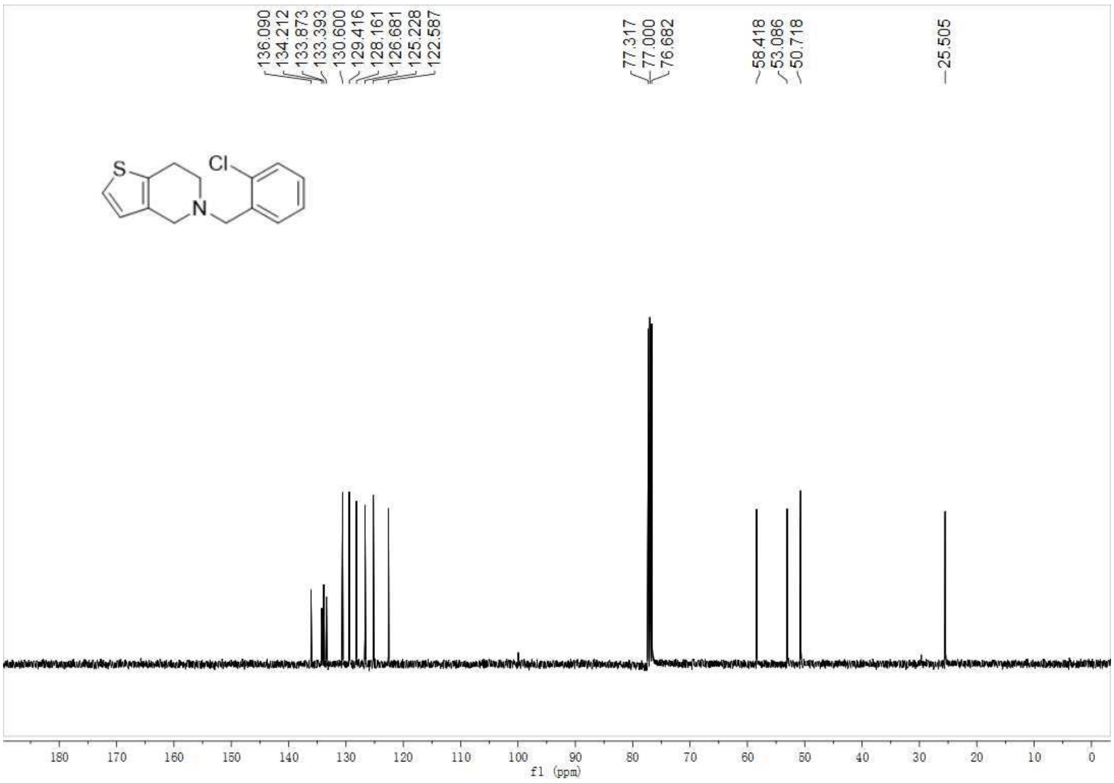

^{1}H NMR Spectrum of **89**

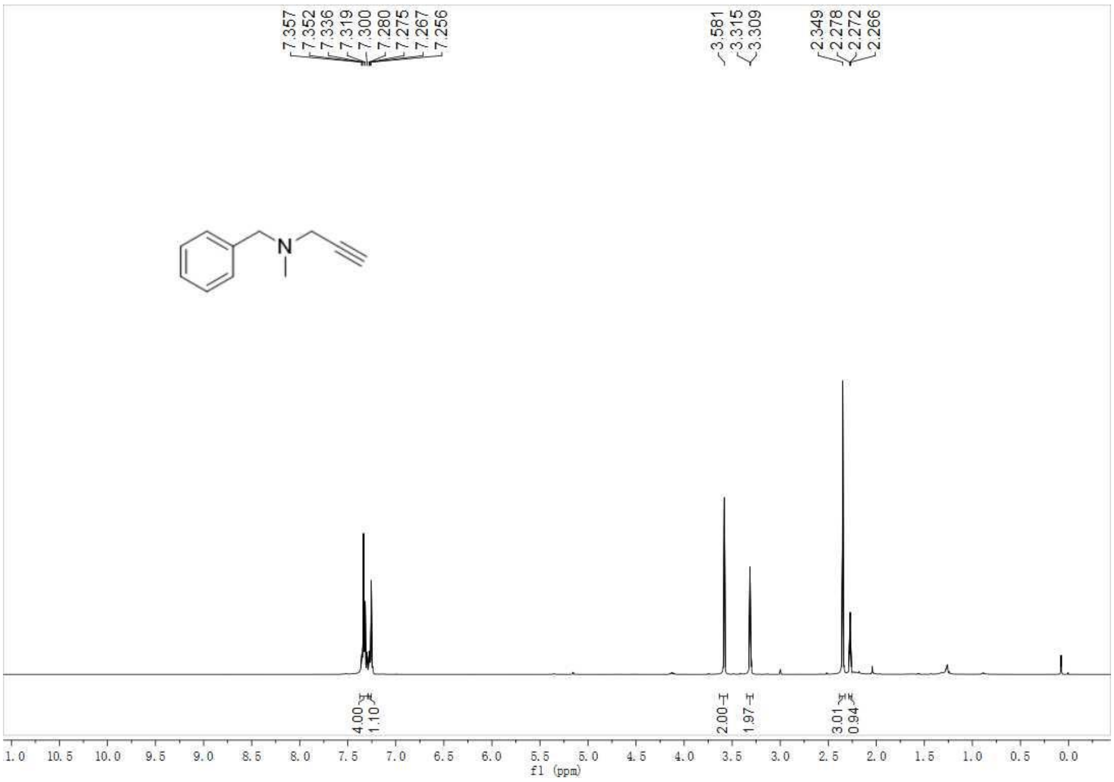

^{13}C NMR Spectrum of **89**

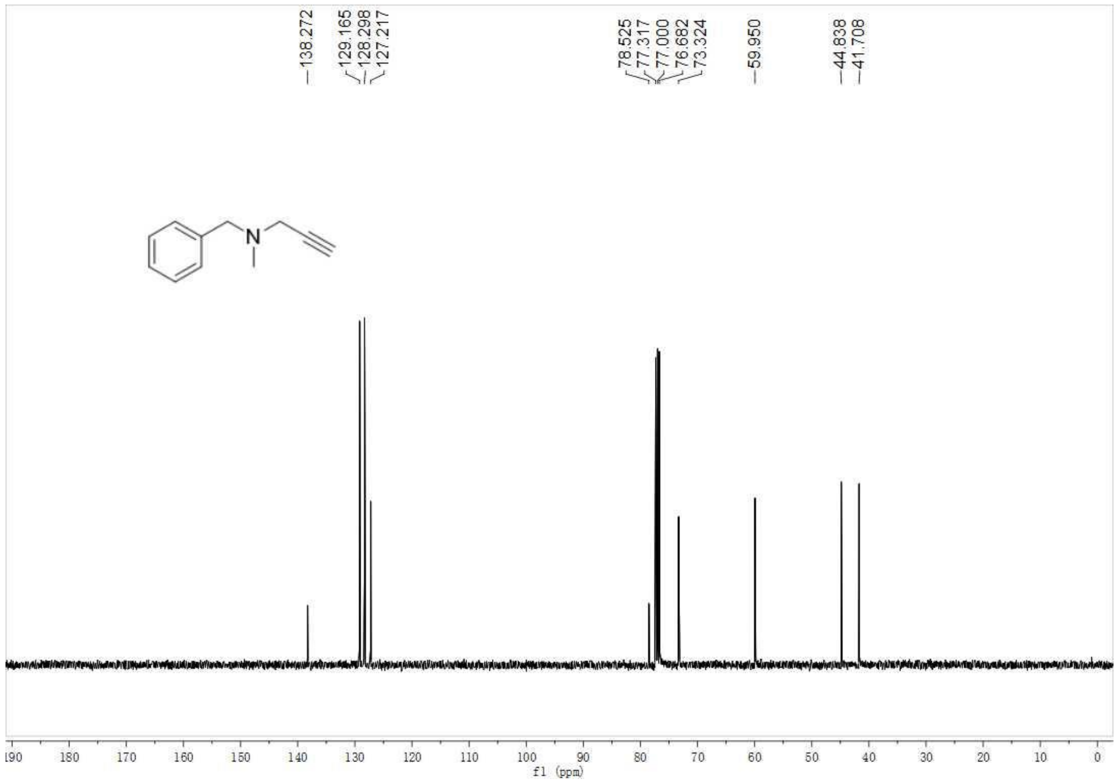

^{1}H NMR Spectrum of **90**

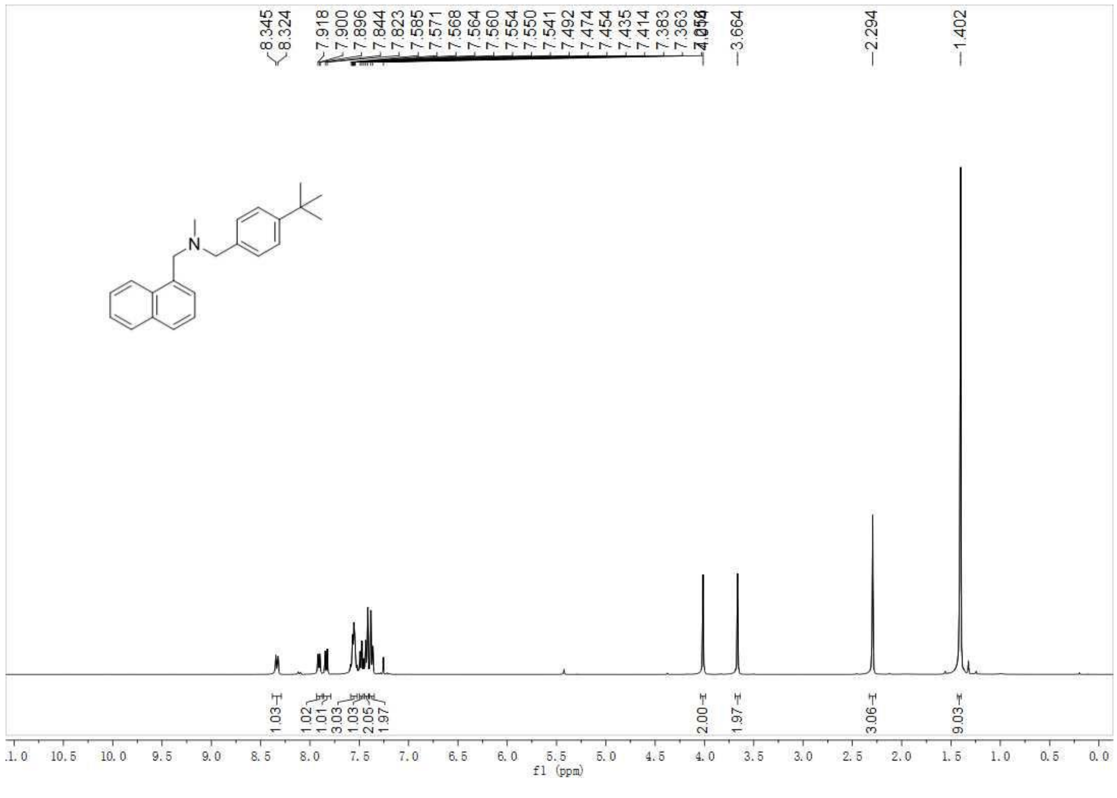

^{13}C NMR Spectrum of **90**

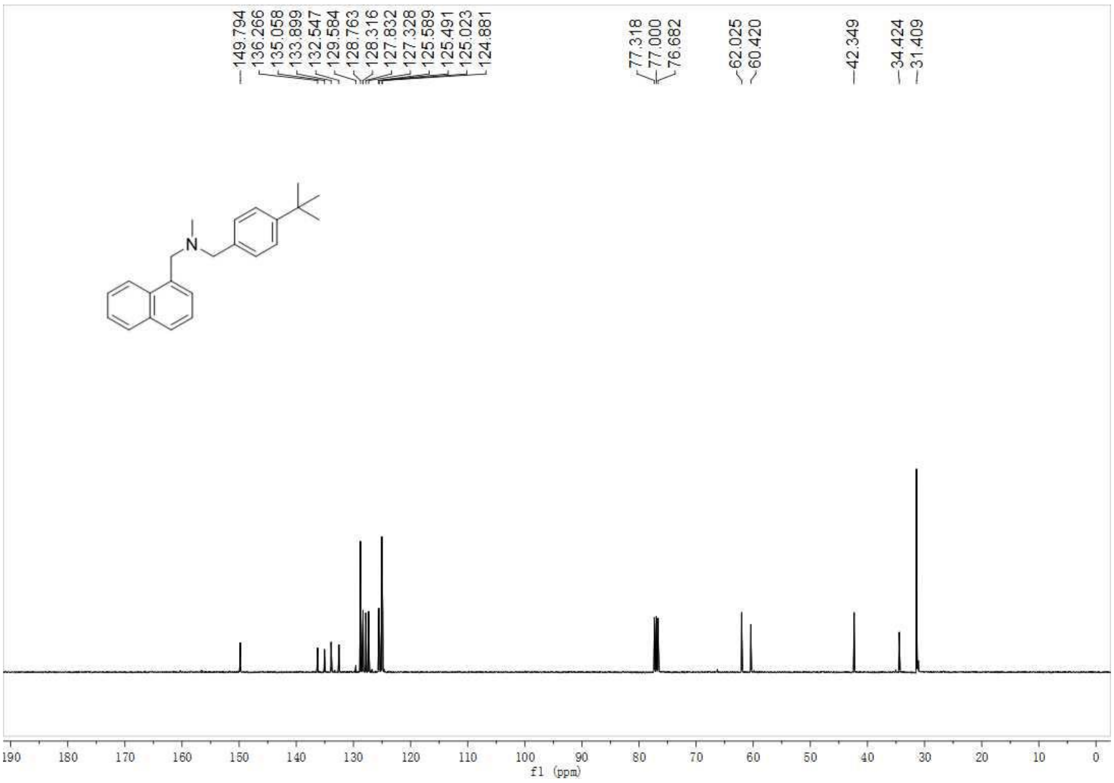

^{1}H NMR Spectrum of **91**

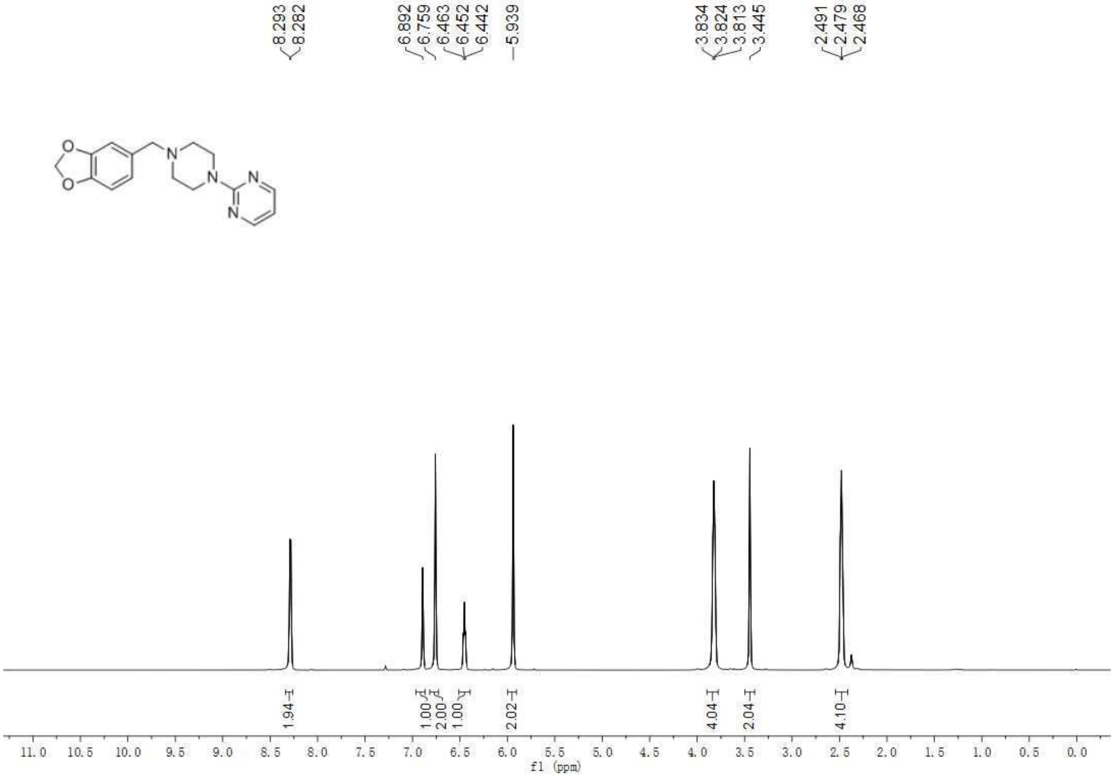

^{13}C NMR Spectrum of **91**

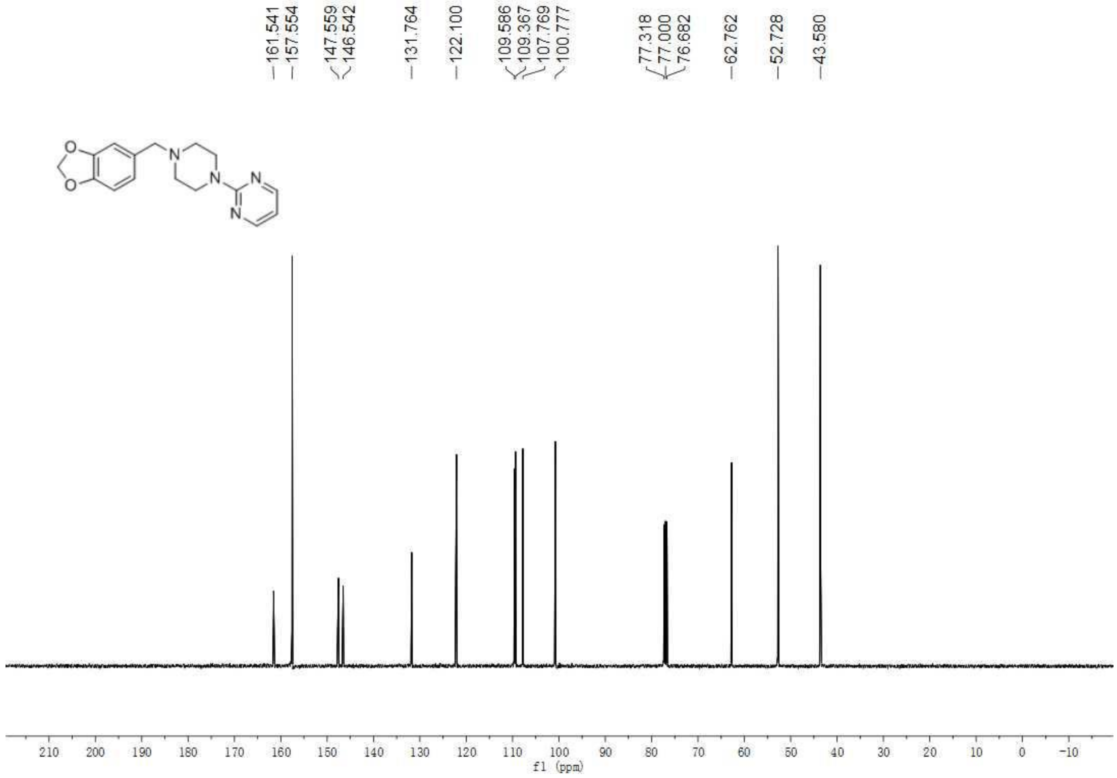

^{1}H NMR Spectrum of **92**

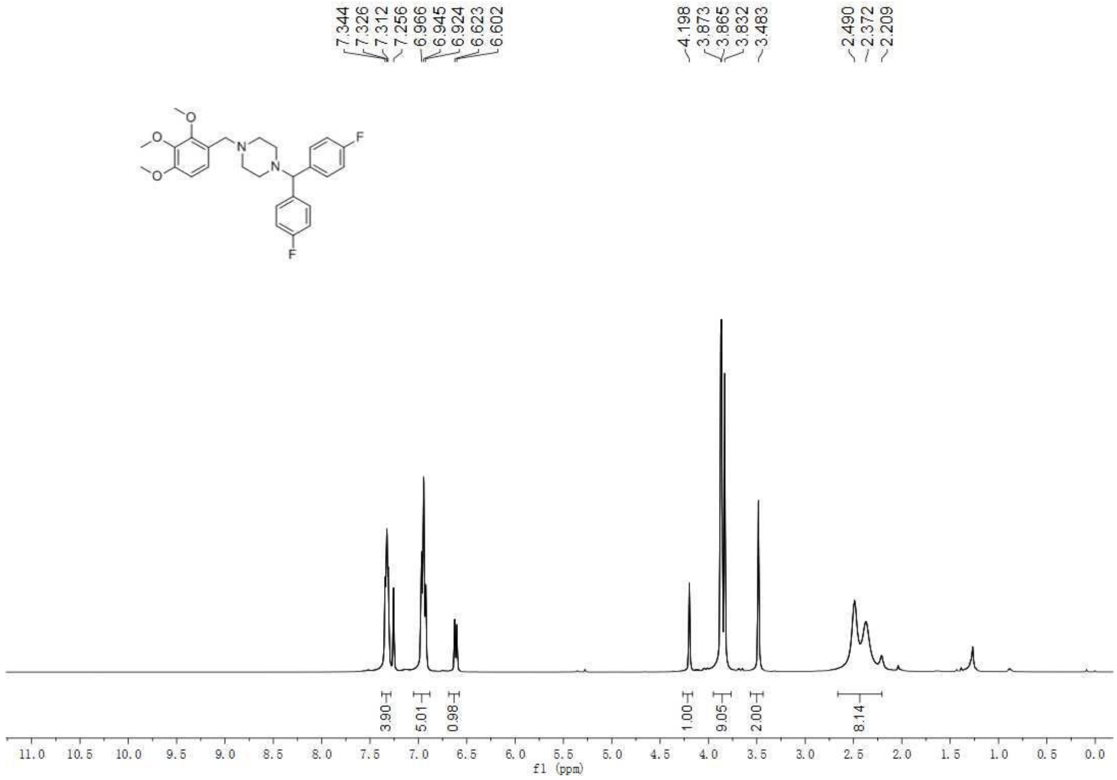

^{13}C NMR Spectrum of **92**

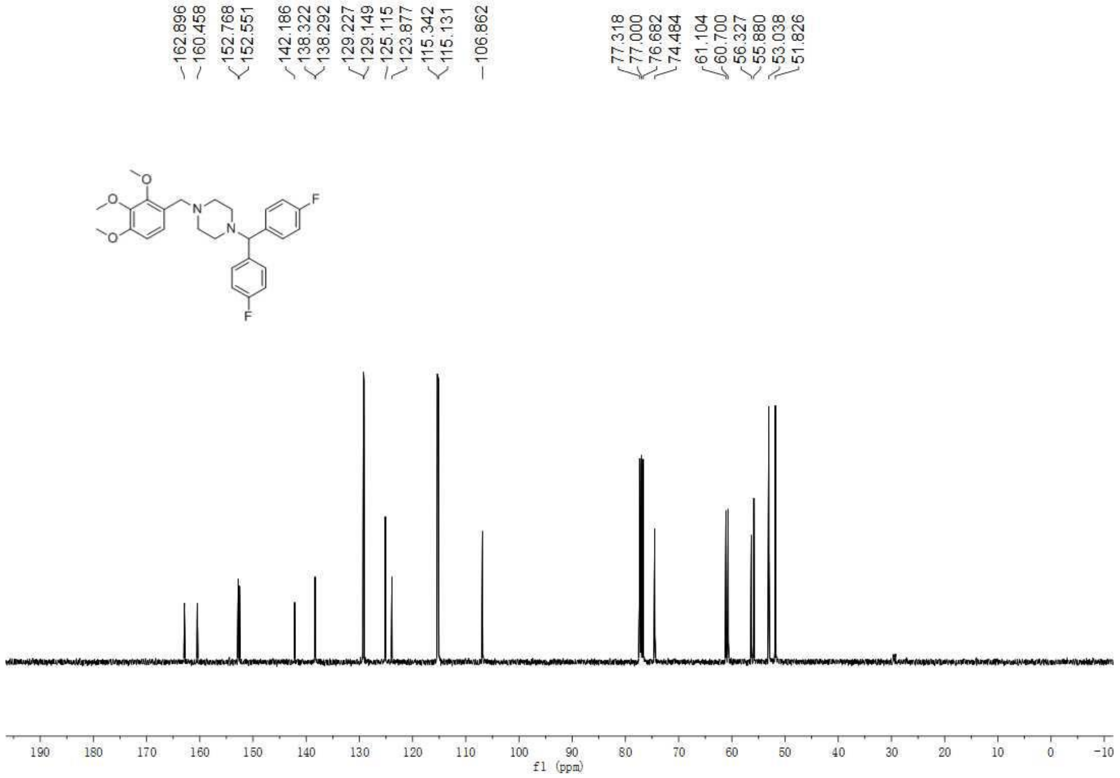

^{19}F NMR Spectrum of **92**

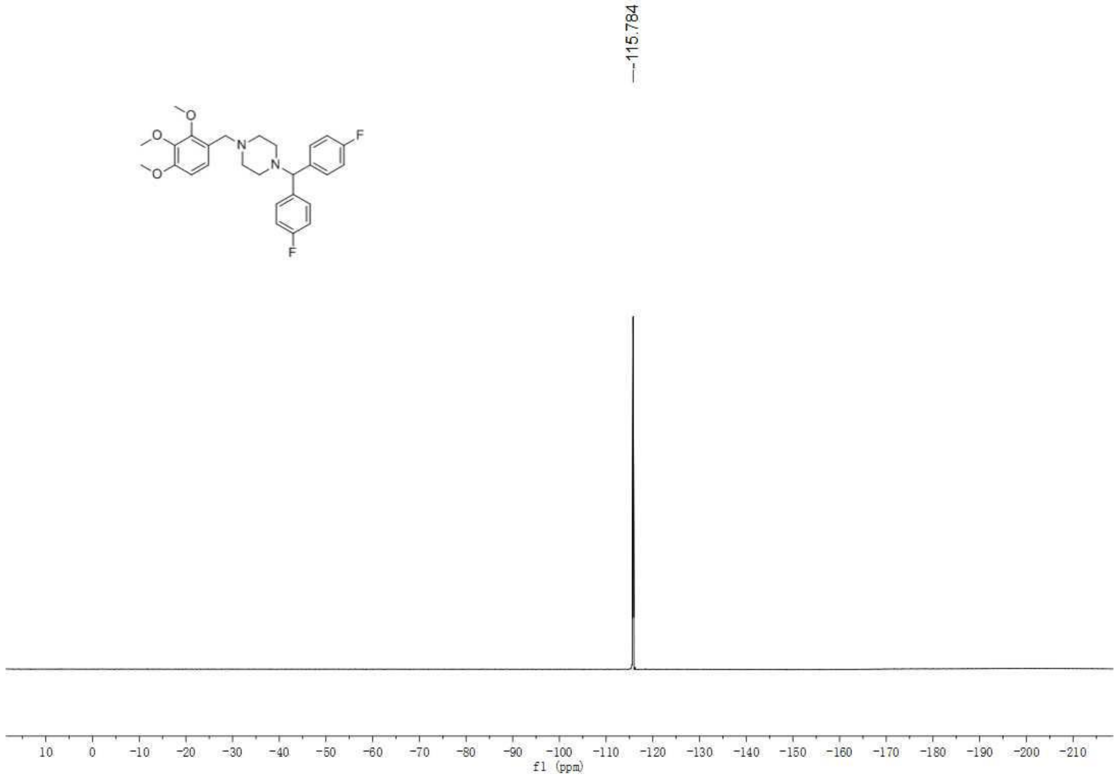

^{1}H NMR Spectrum of **93**

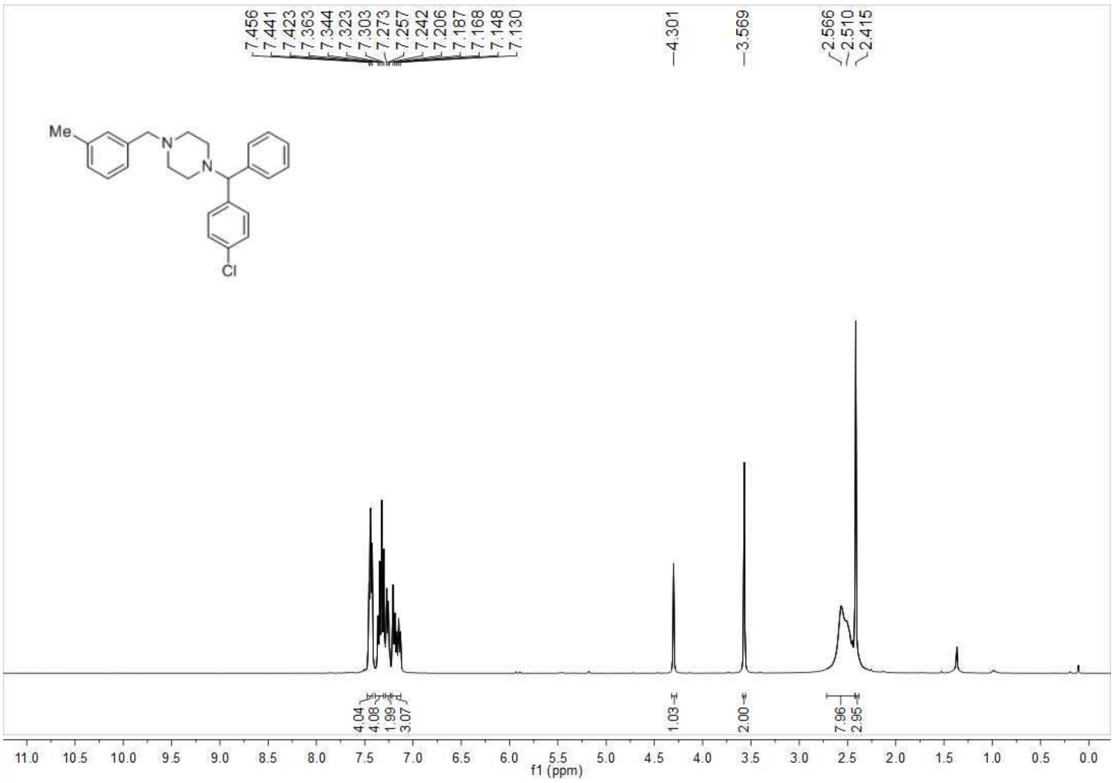

^{13}C NMR Spectrum of **93**

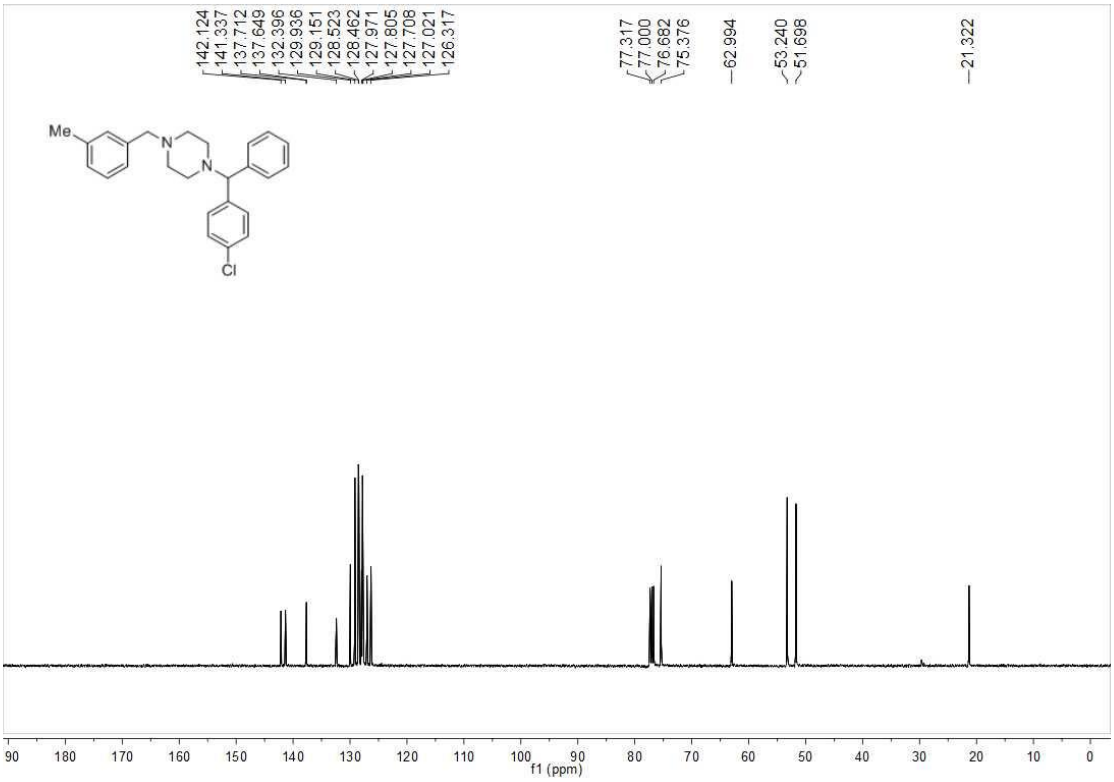

^{1}H NMR Spectrum of **94**

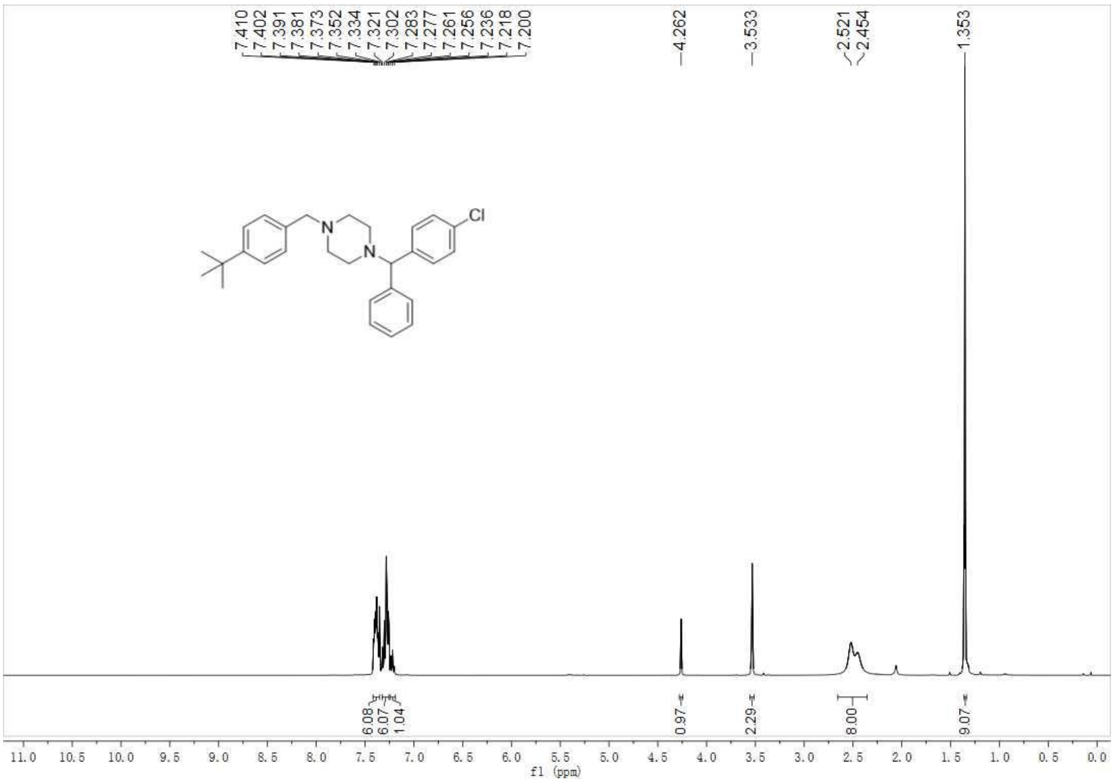

^{13}C NMR Spectrum of **94**

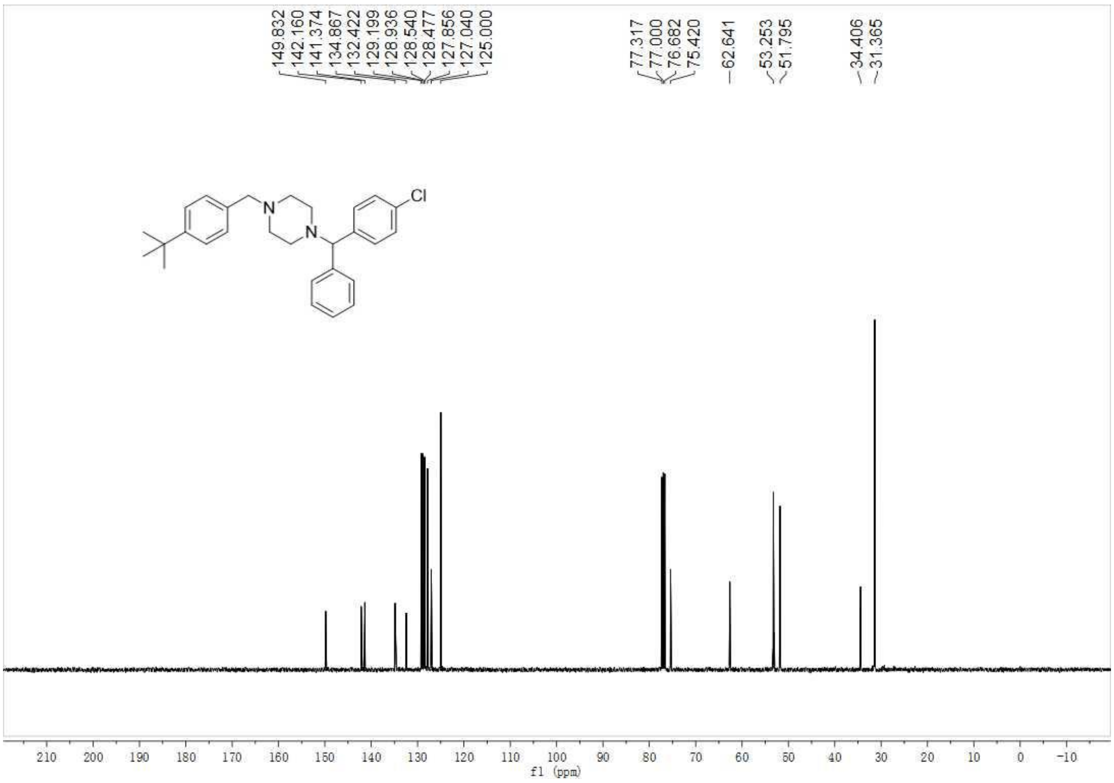

^{1}H NMR Spectrum of **95**

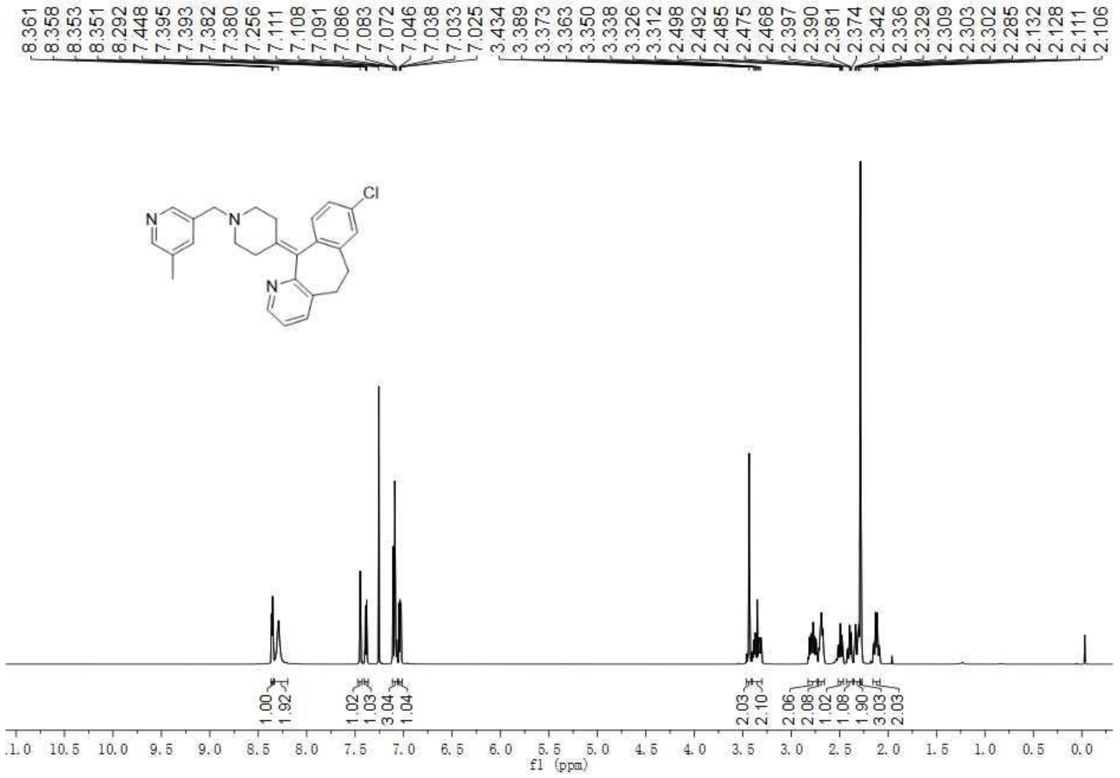

^{13}C NMR Spectrum of **95**

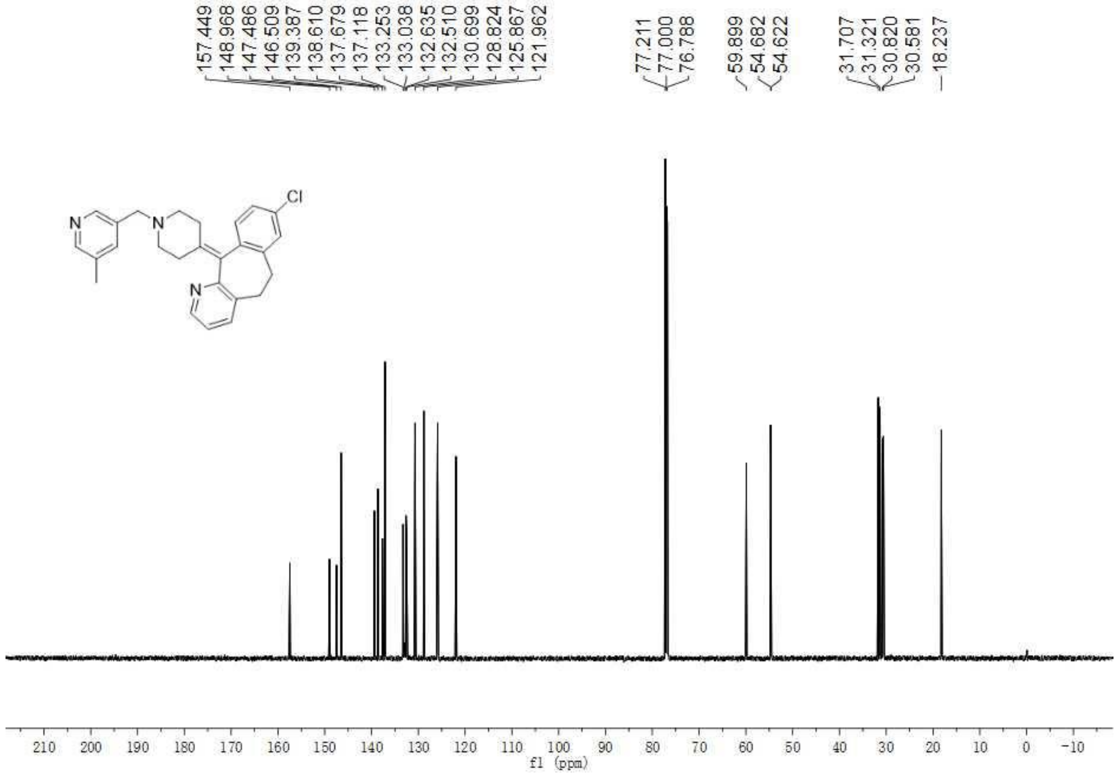

^{1}H NMR Spectrum of **96**

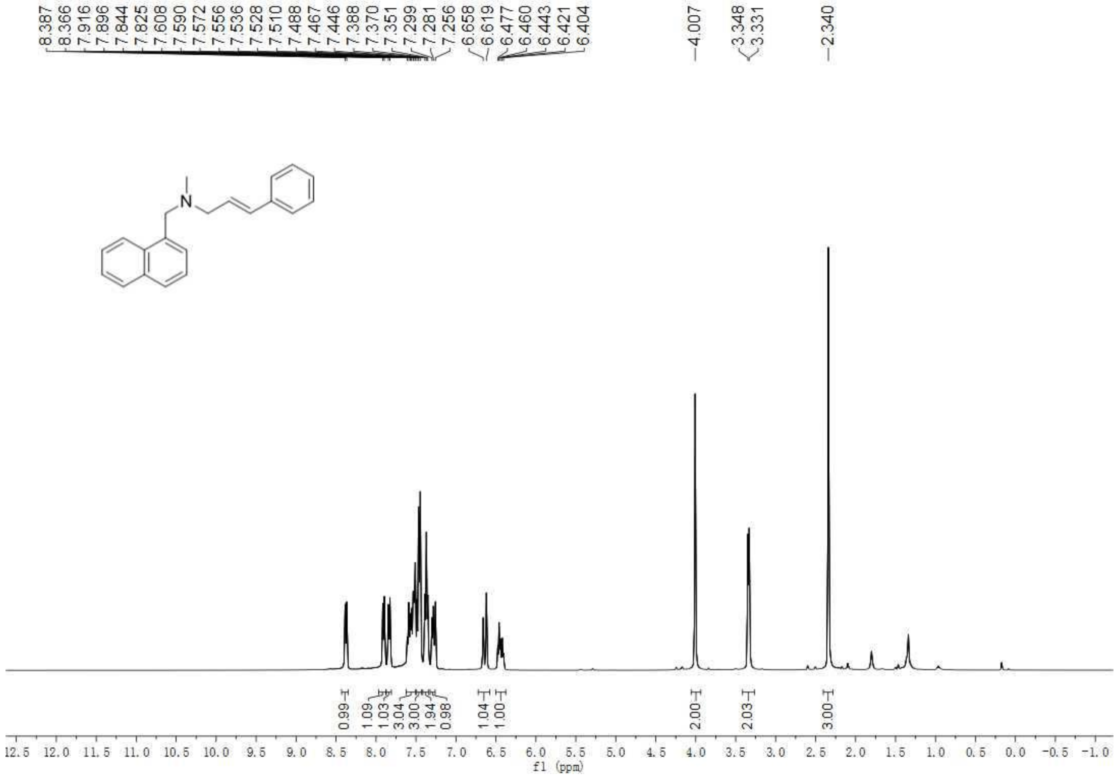

^{13}C NMR Spectrum of **96**

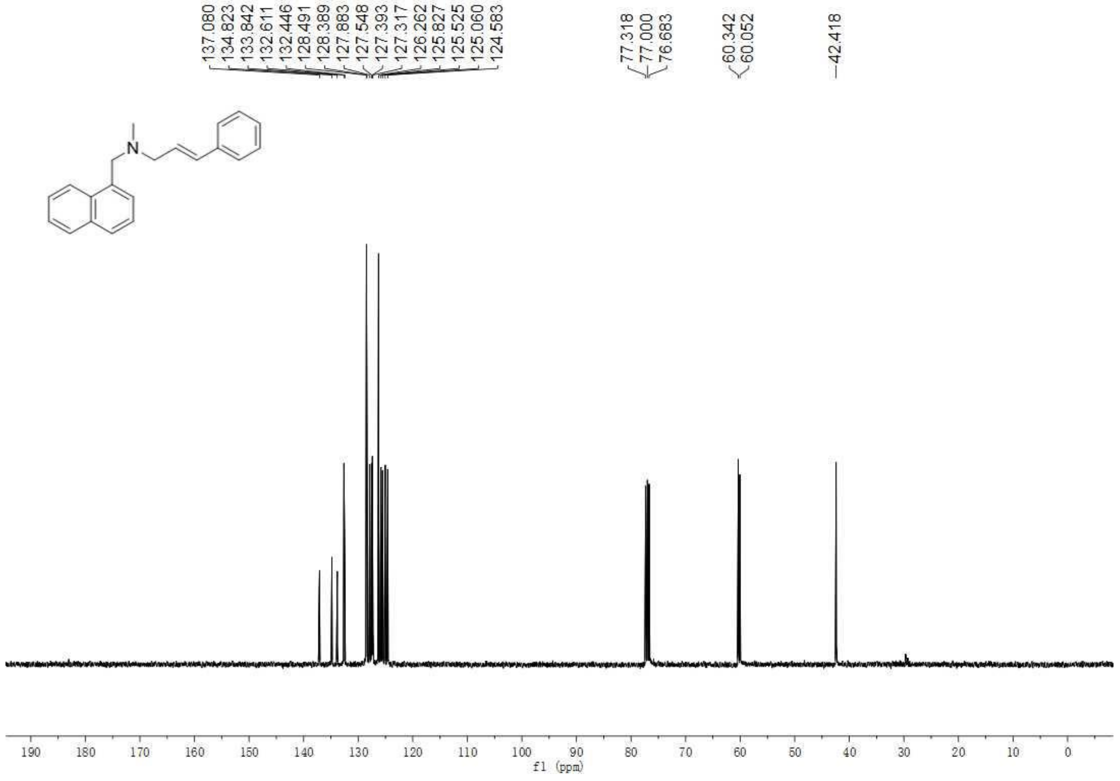

^{1}H NMR Spectrum of **97**

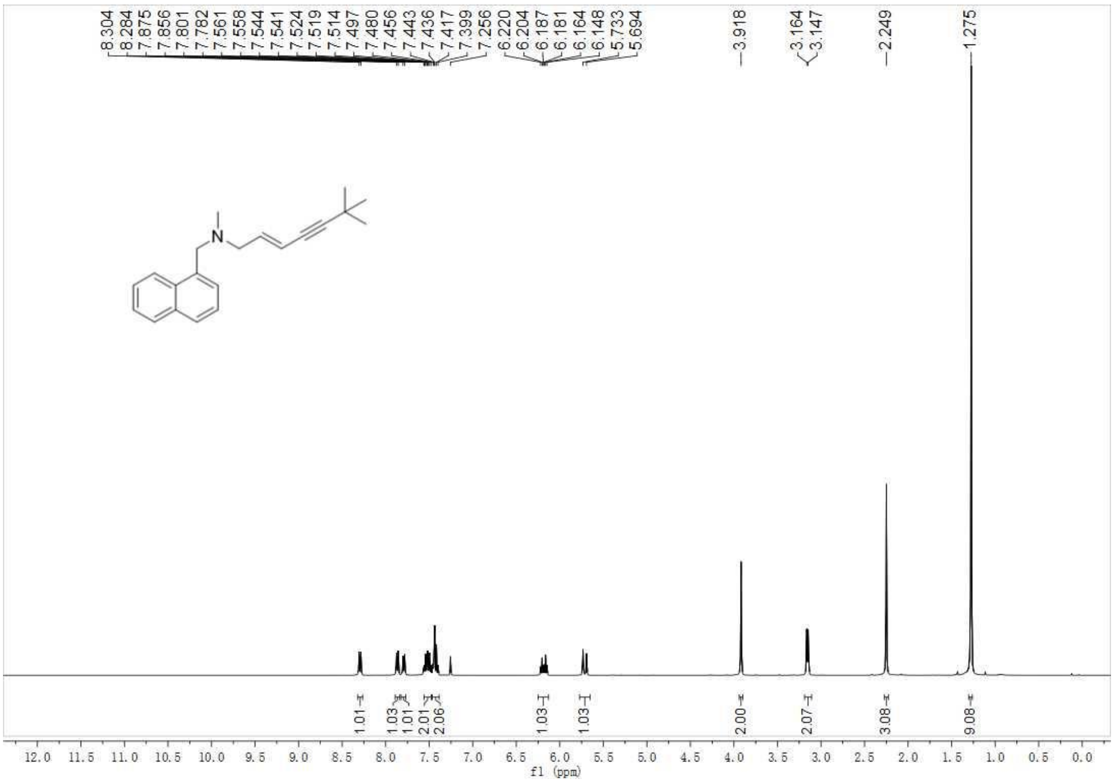

^{13}C NMR Spectrum of **97**

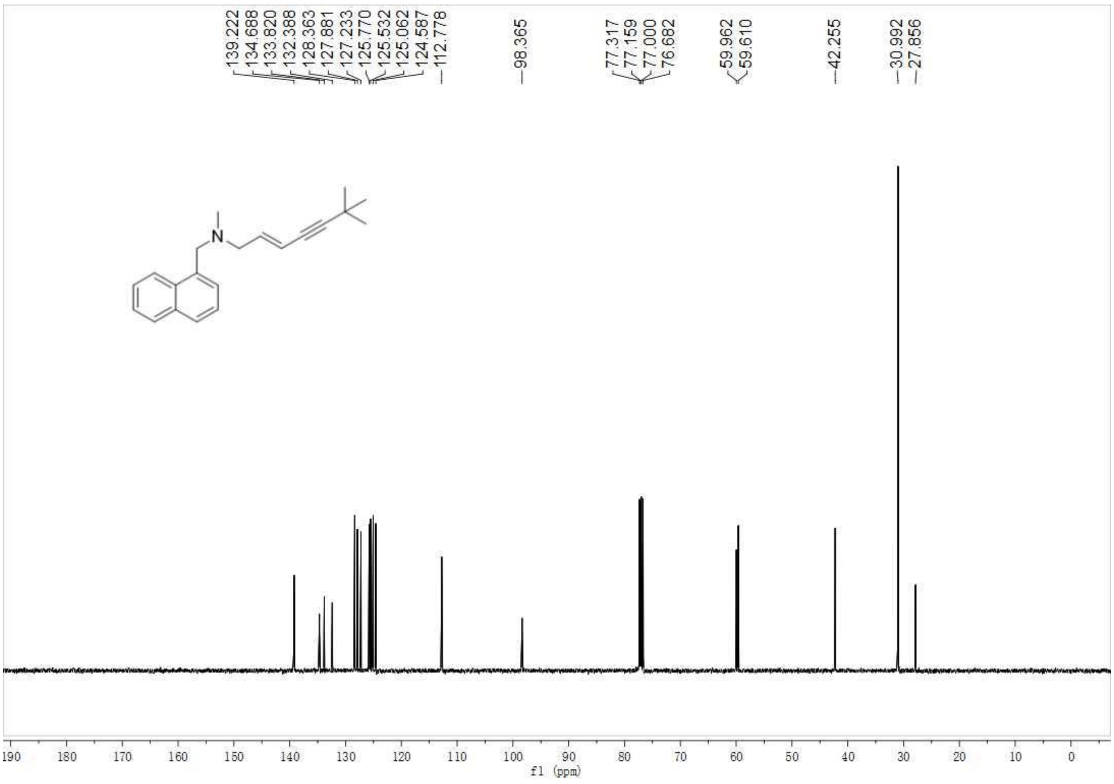

^{1}H NMR Spectrum of **98**

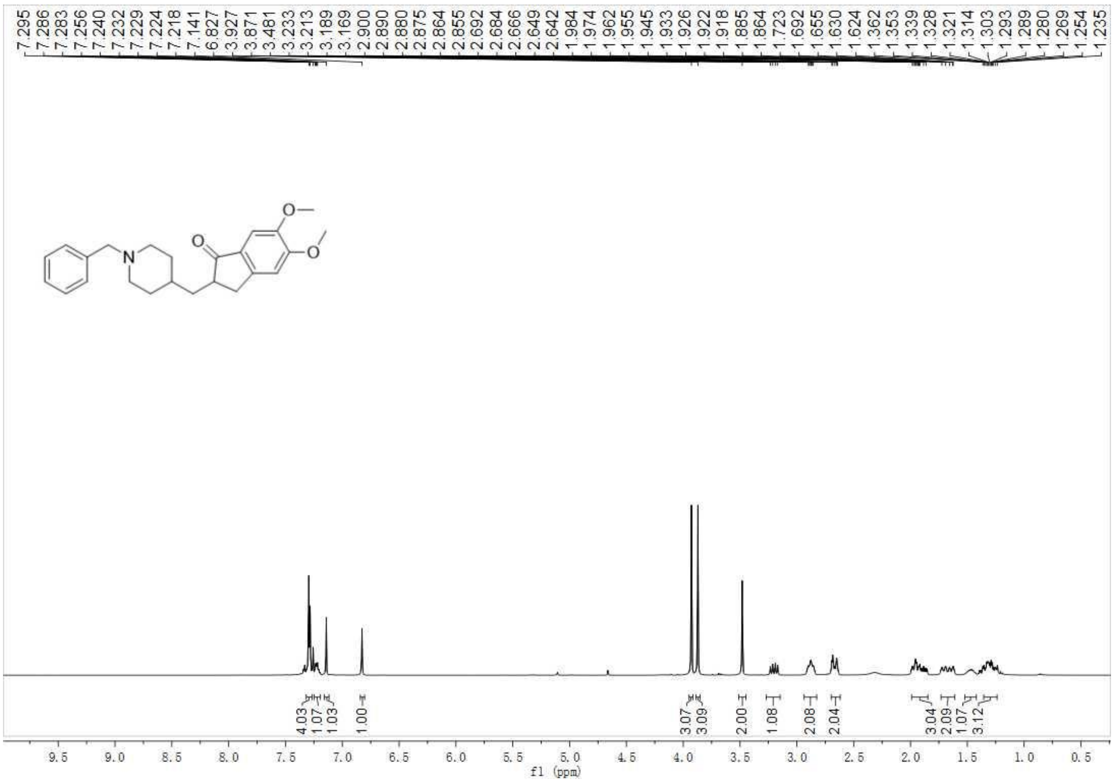

^{13}C NMR Spectrum of **98**

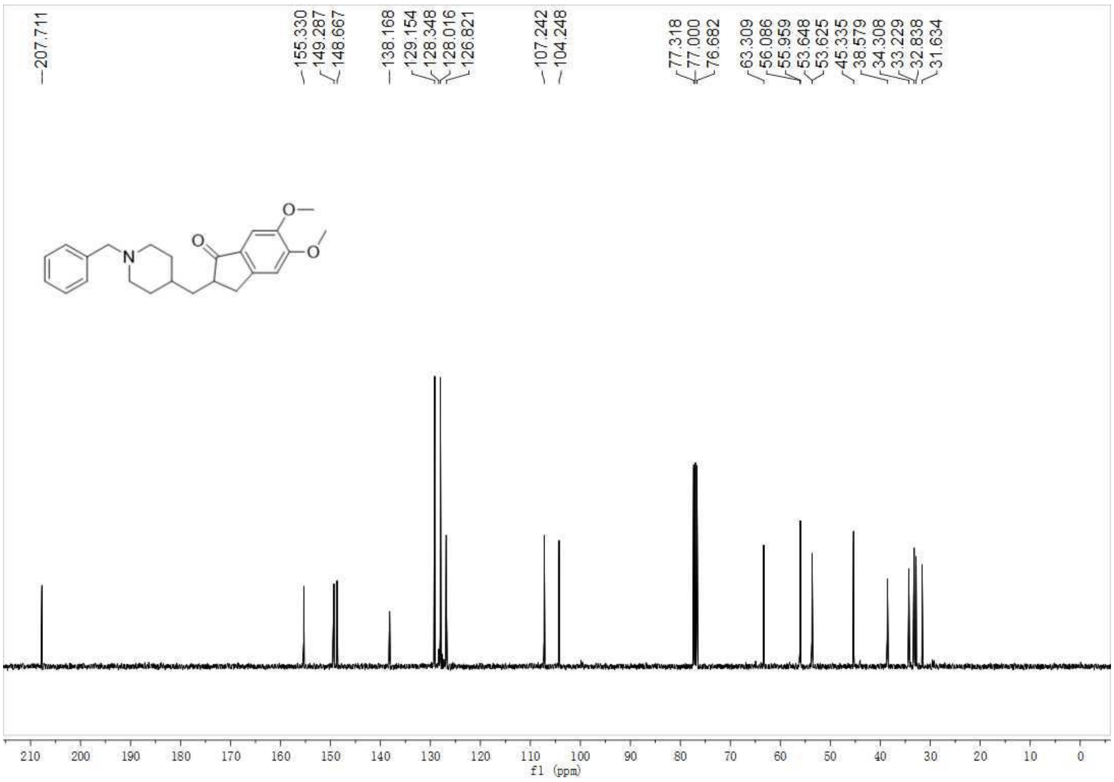

^{1}H NMR Spectrum of **99**

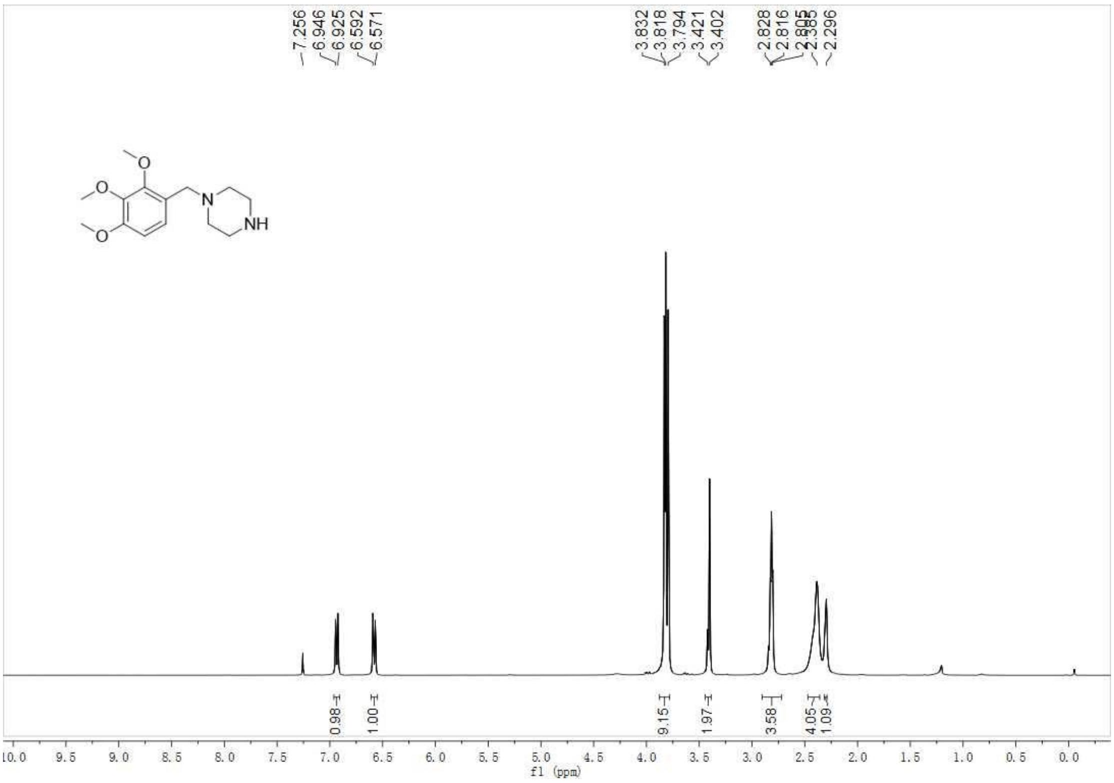

^{13}C NMR Spectrum of **99**

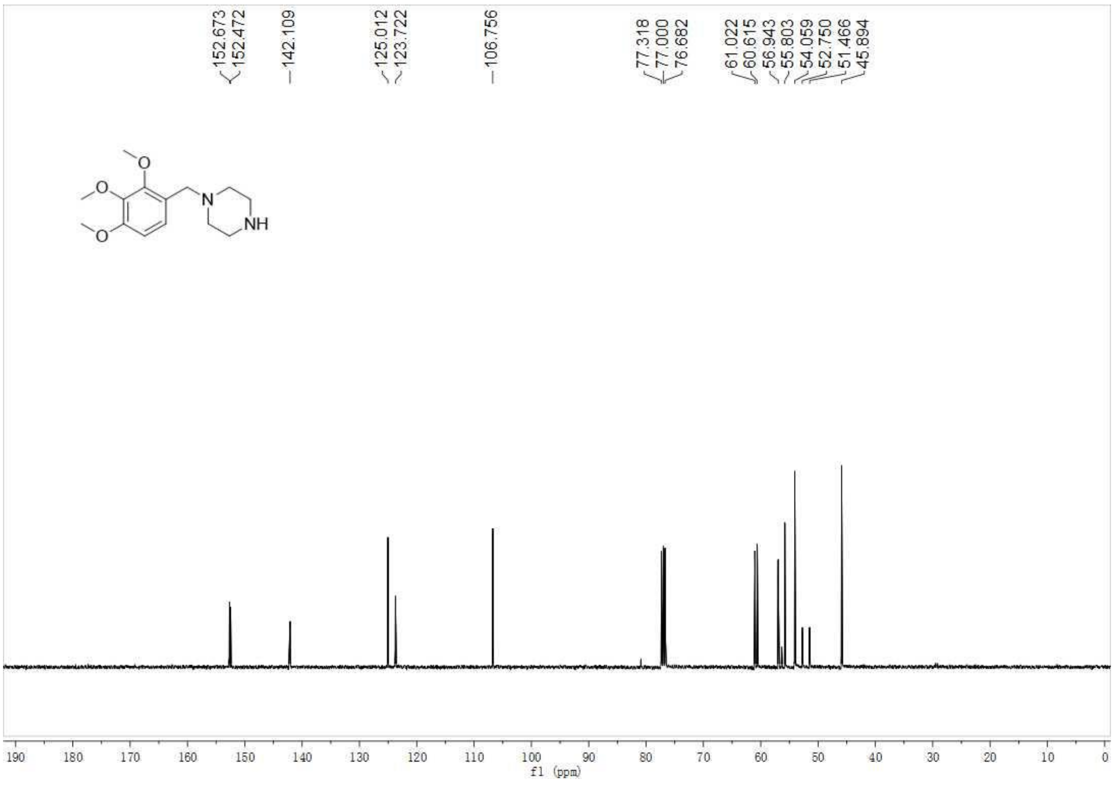

^{1}H NMR Spectrum of **100**

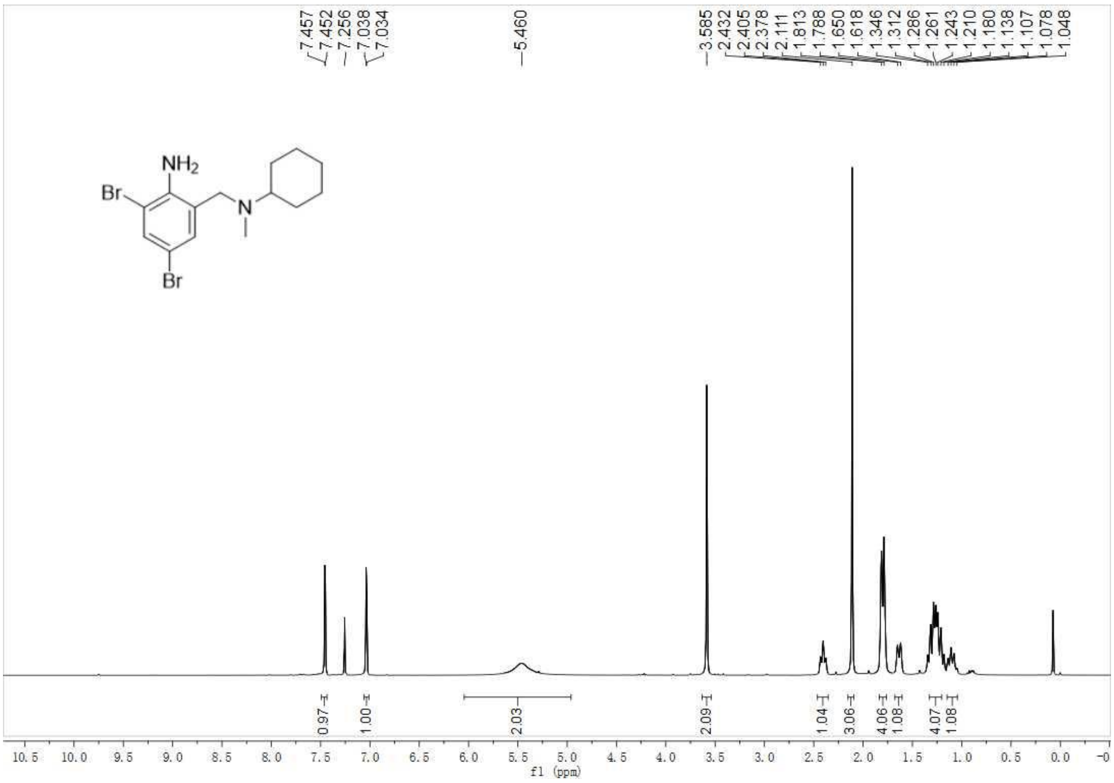

^{13}C NMR Spectrum of **100**

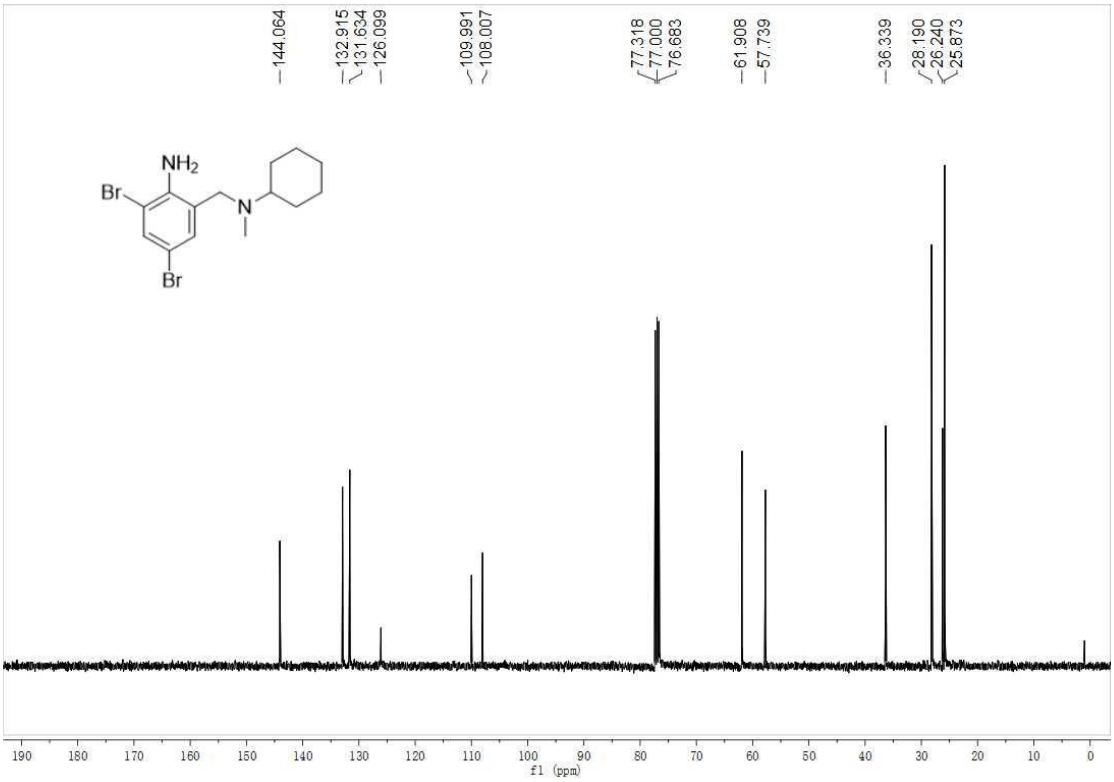

^{1}H NMR Spectrum of **101**

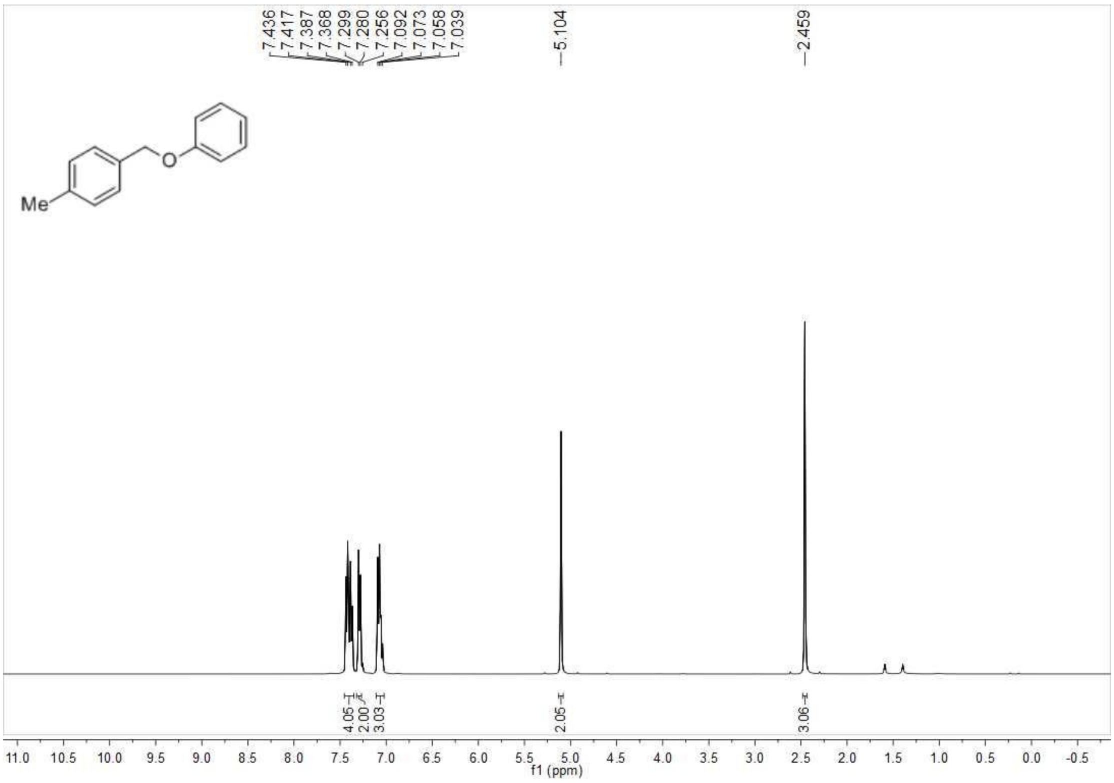

^{13}C NMR Spectrum of **101**

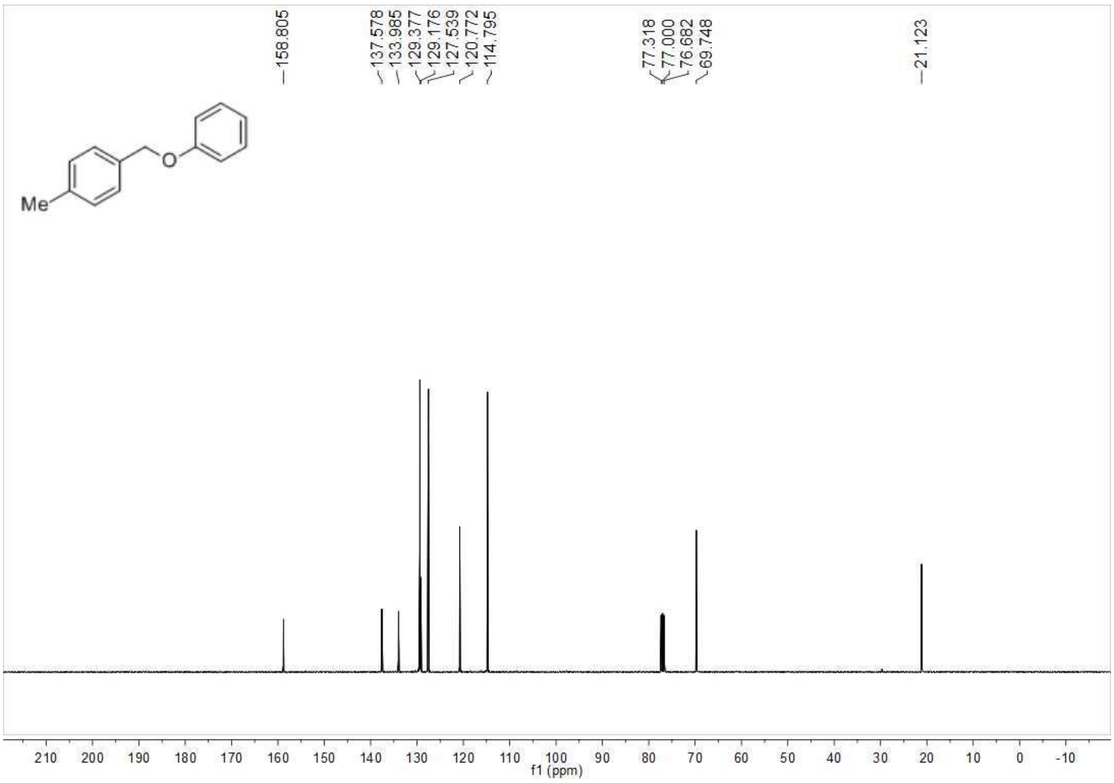

^{1}H NMR Spectrum of **102**

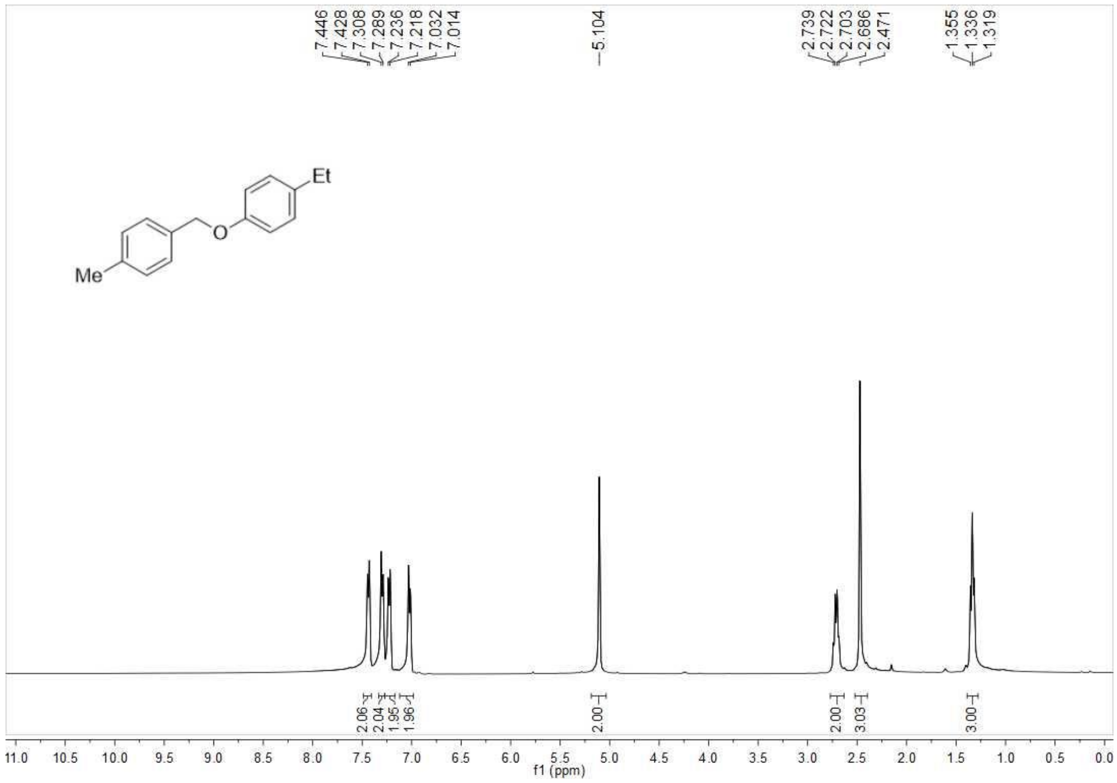

^{13}C NMR Spectrum of **102**

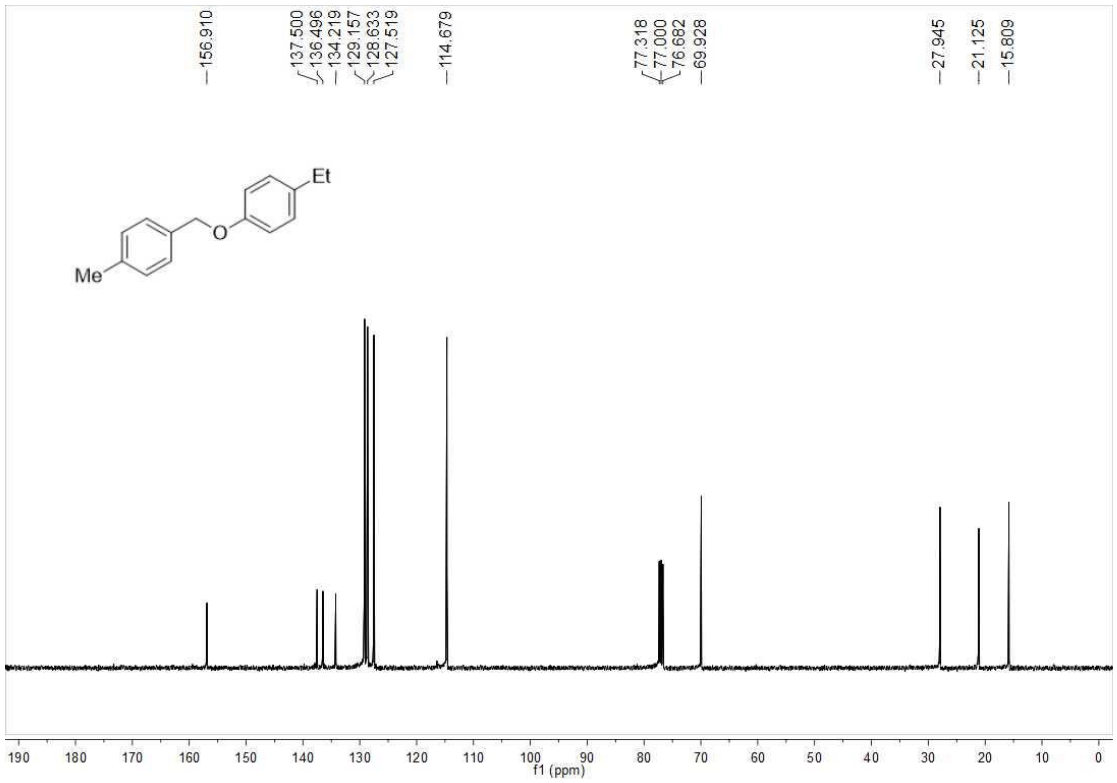

^{1}H NMR Spectrum of **103**

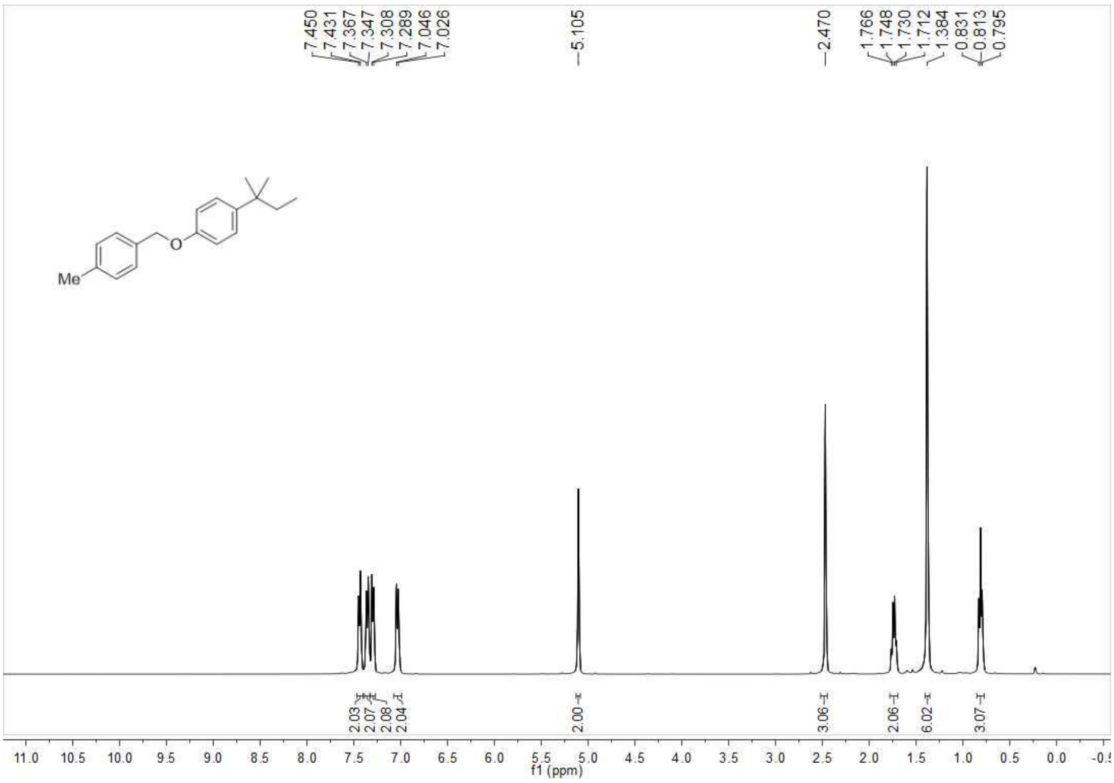

^{13}C NMR Spectrum of **103**

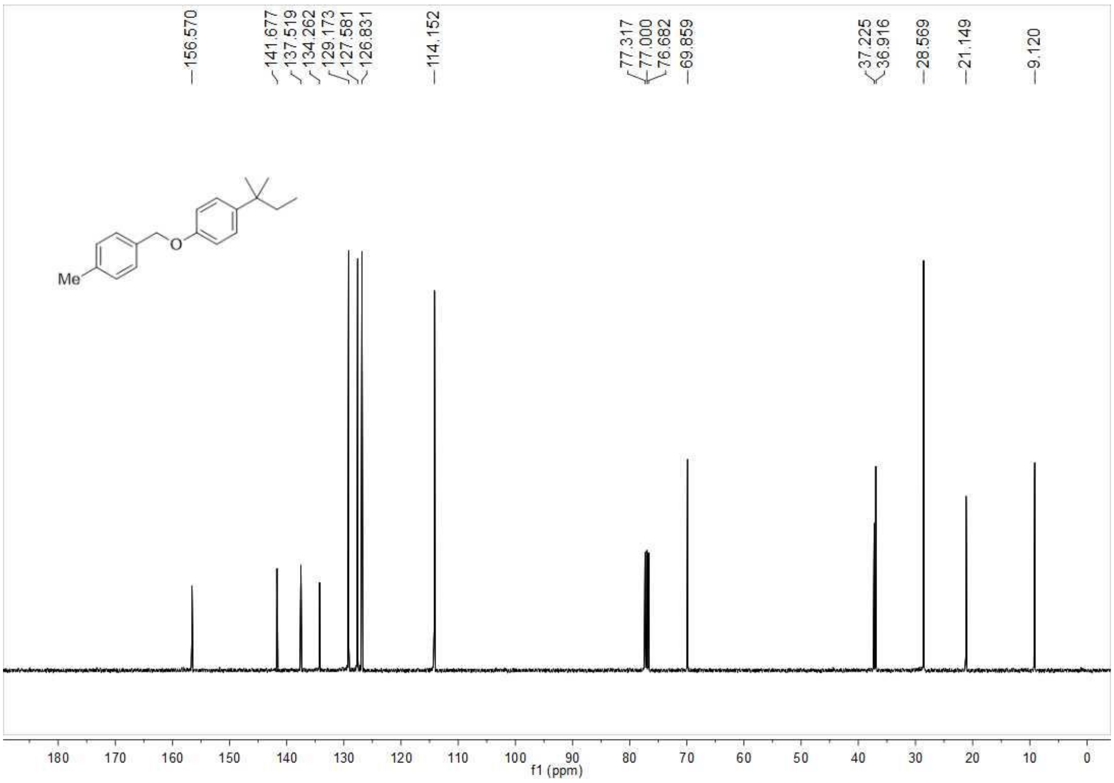

^{1}H NMR Spectrum of **104**

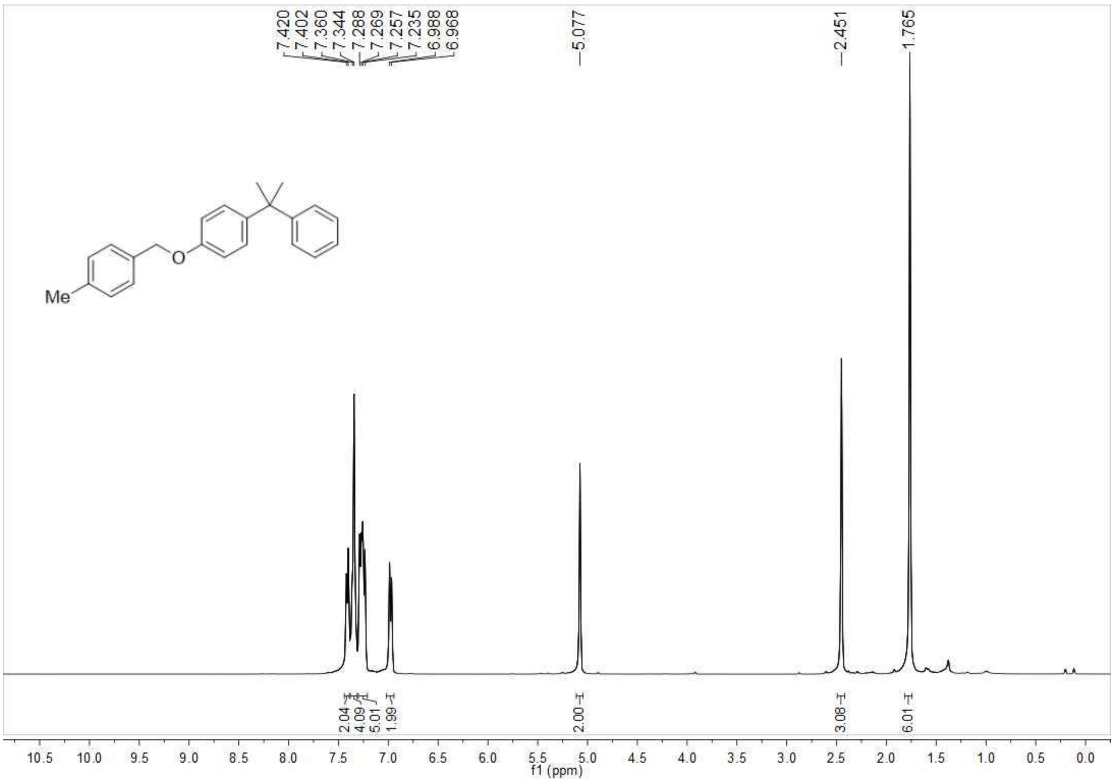

^{13}C NMR Spectrum of **104**

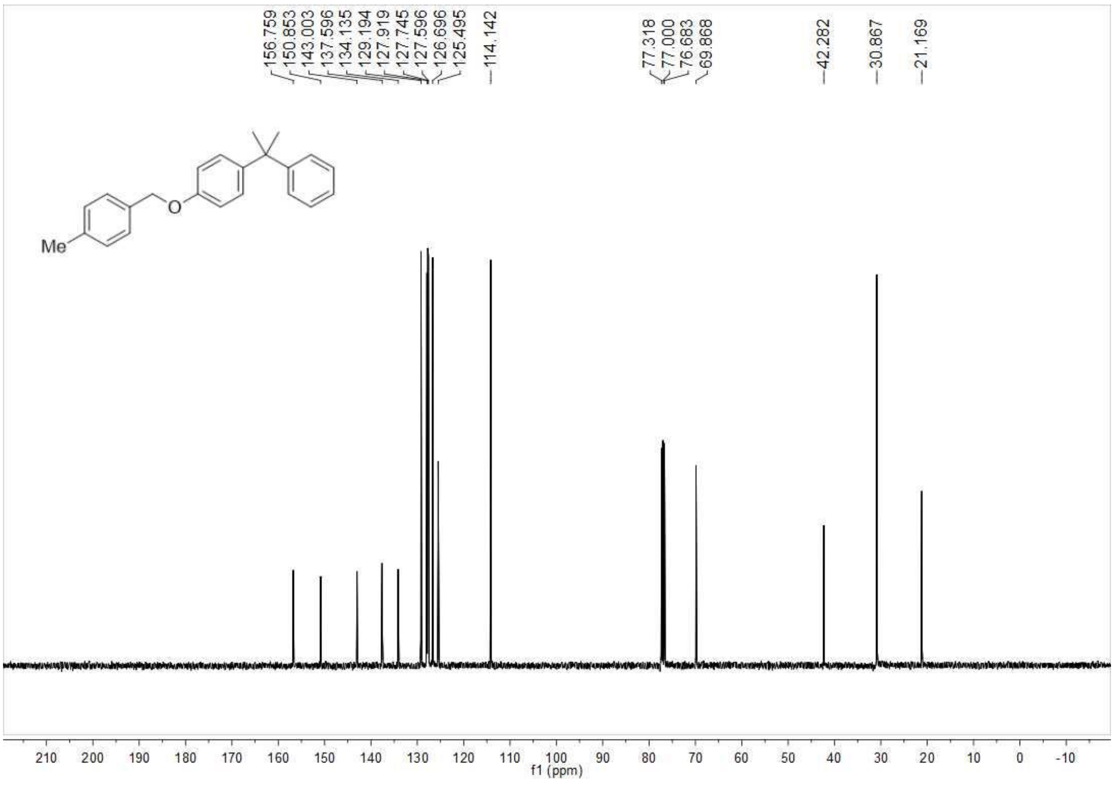

^{1}H NMR Spectrum of **105**

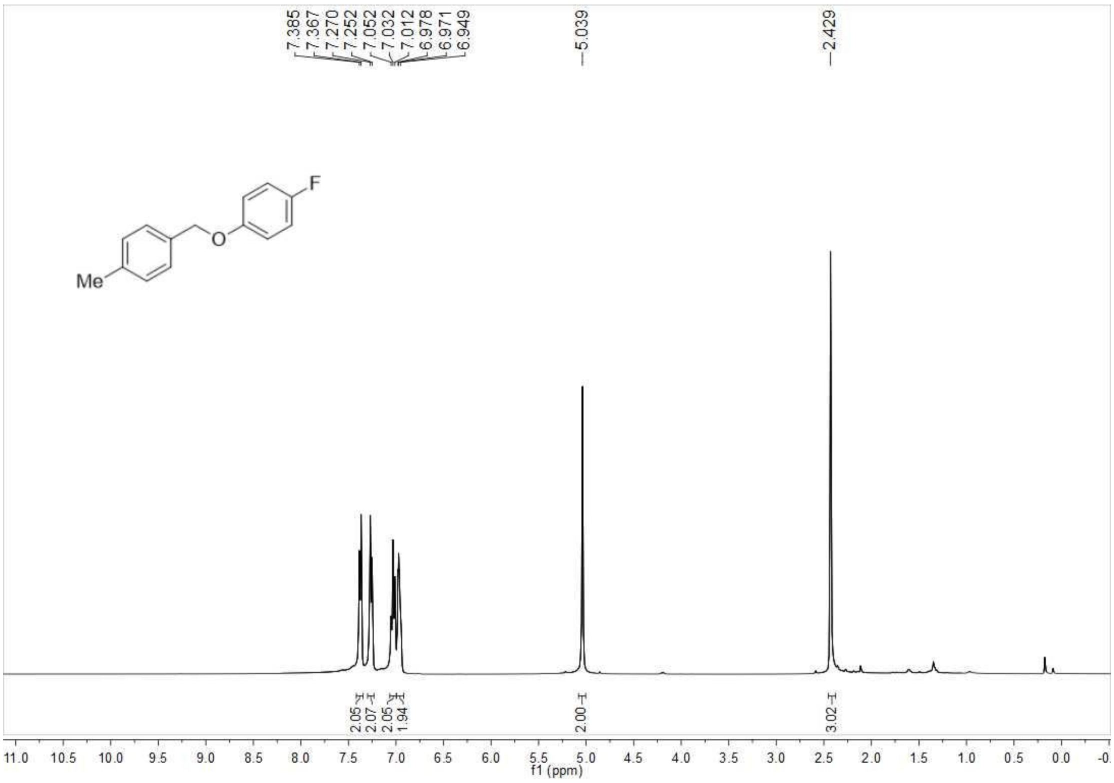

^{13}C NMR Spectrum of **105**

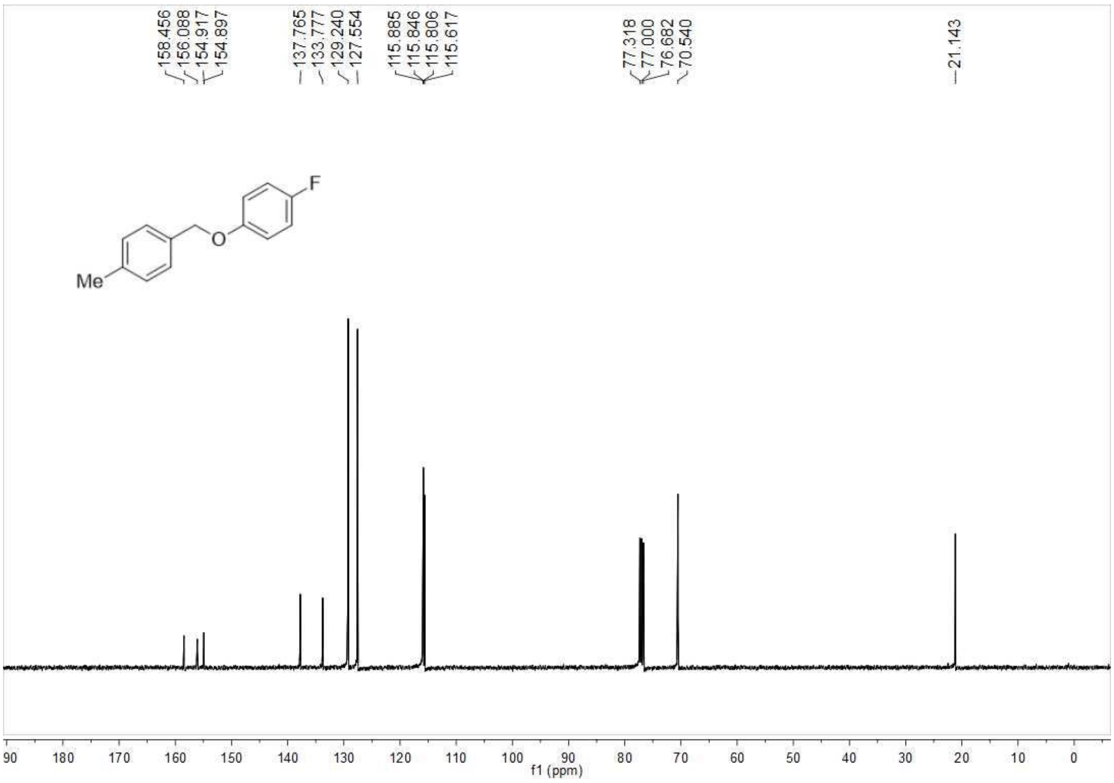

^{19}F NMR Spectrum of **105**

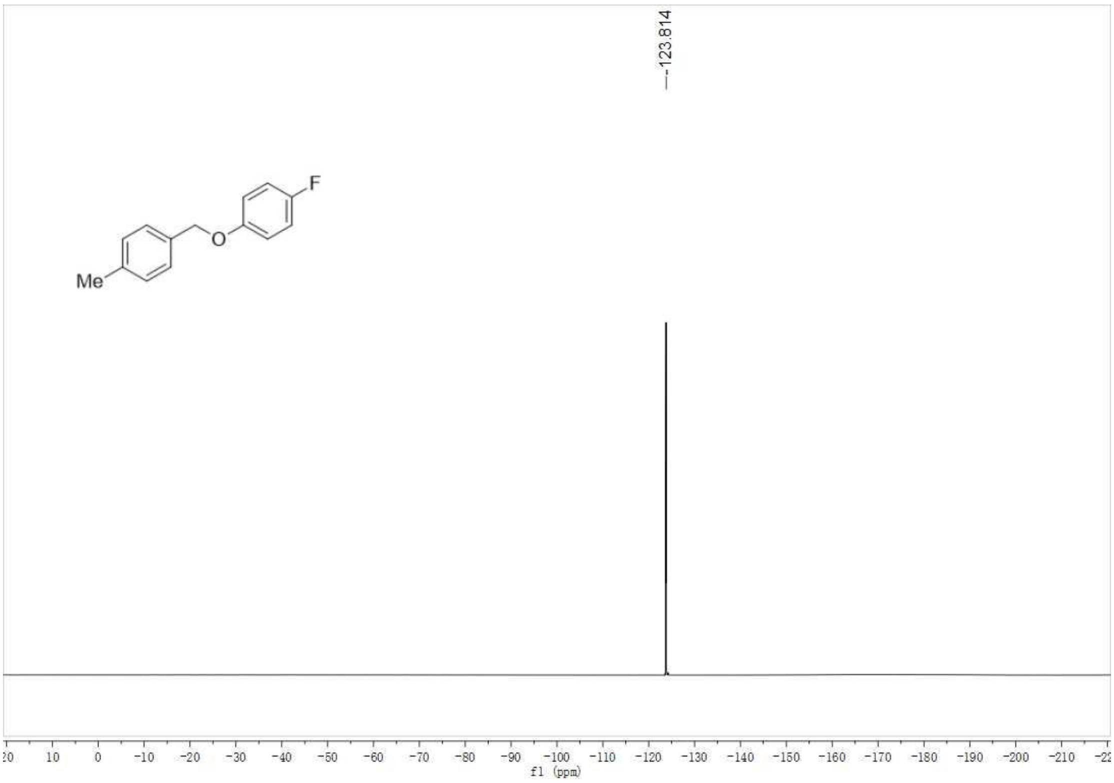

^{1}H NMR Spectrum of **106**

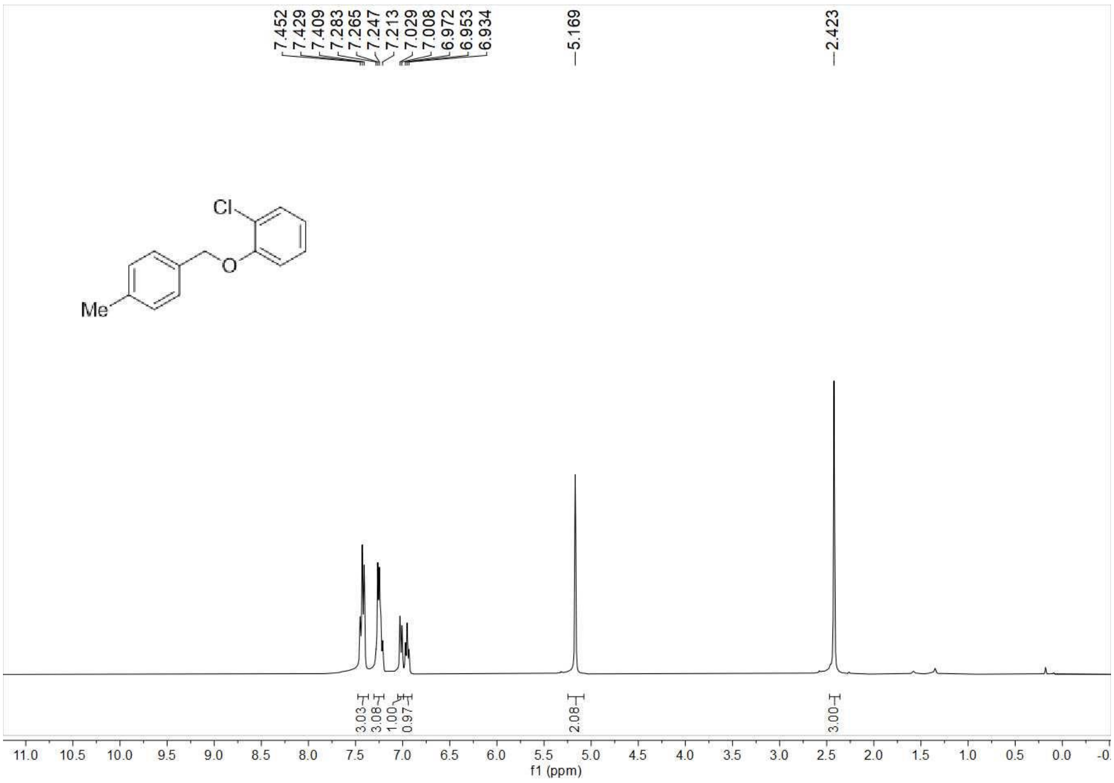

^{13}C NMR Spectrum of **106**

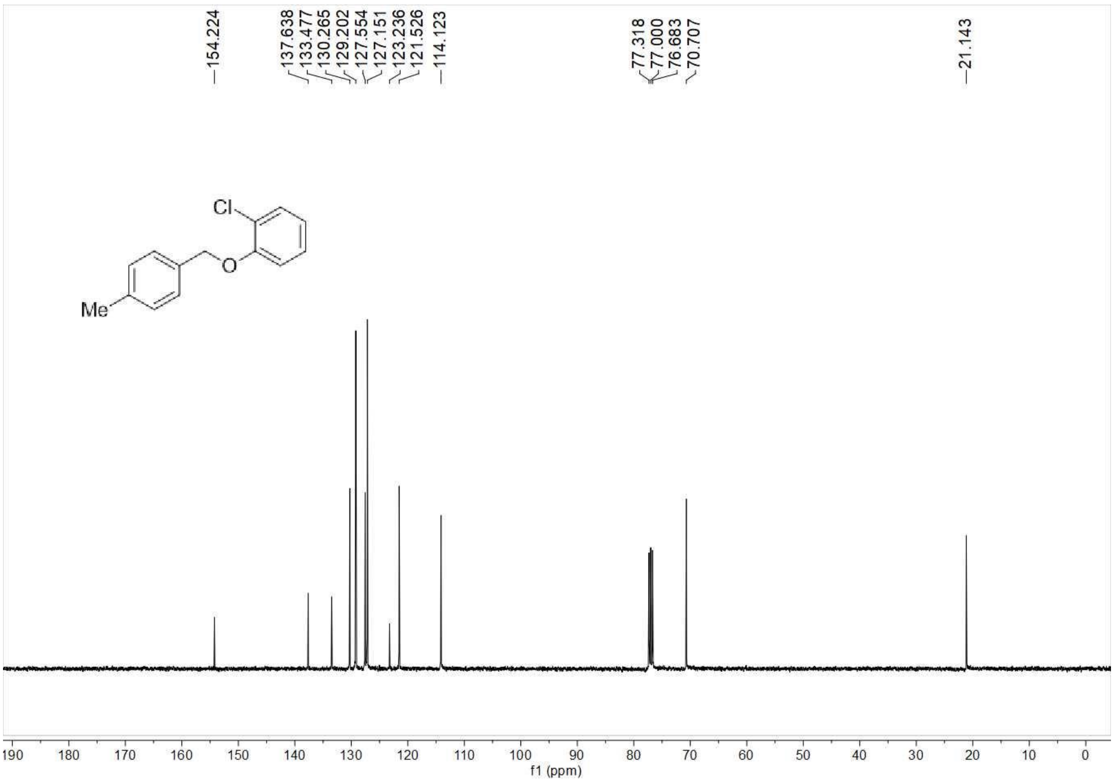

^{1}H NMR Spectrum of **107**

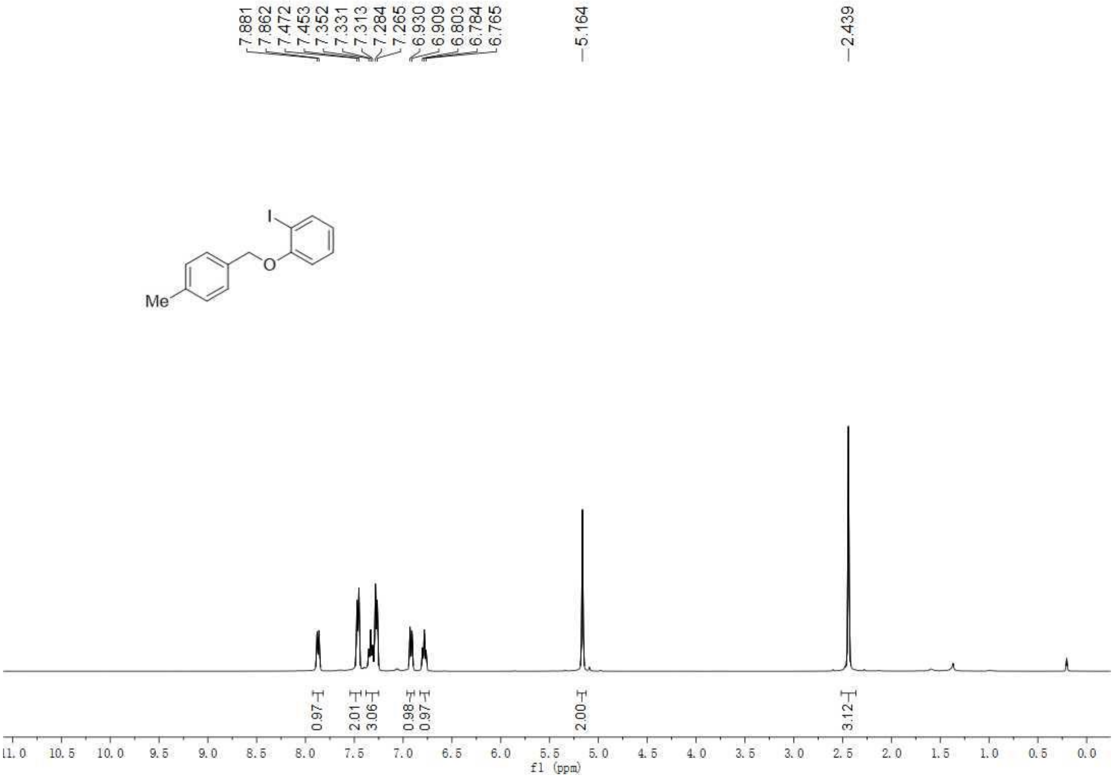

^{13}C NMR Spectrum of **107**

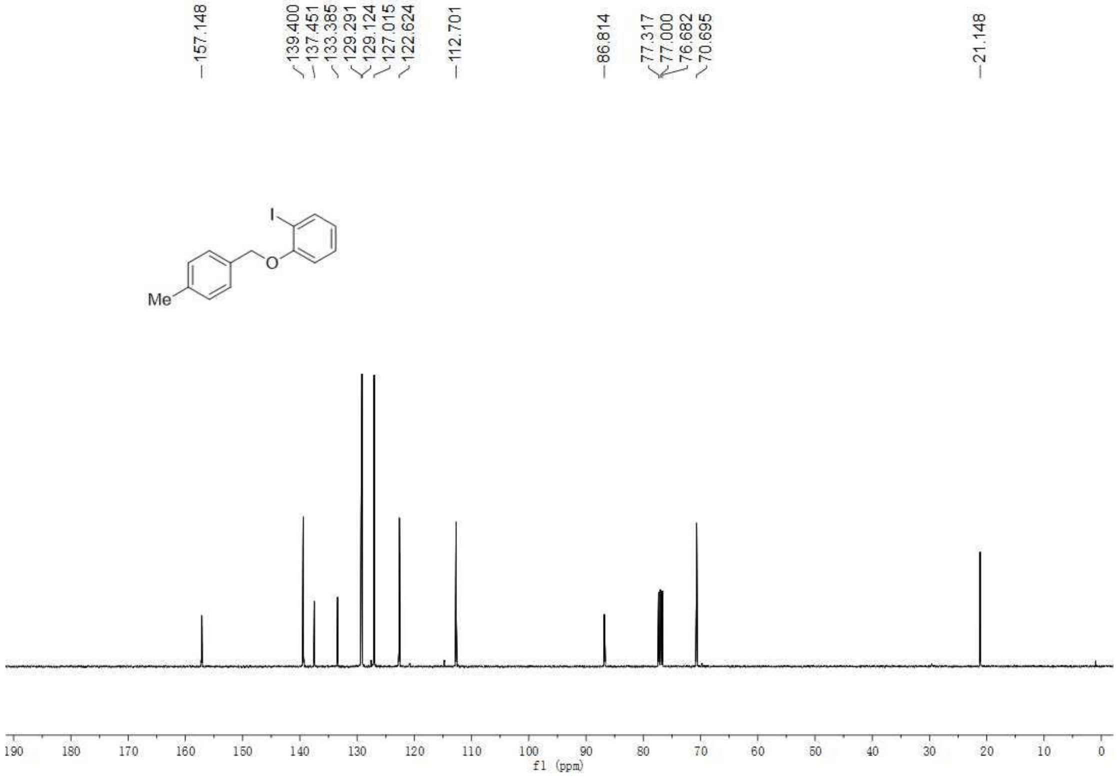

^{1}H NMR Spectrum of **108**

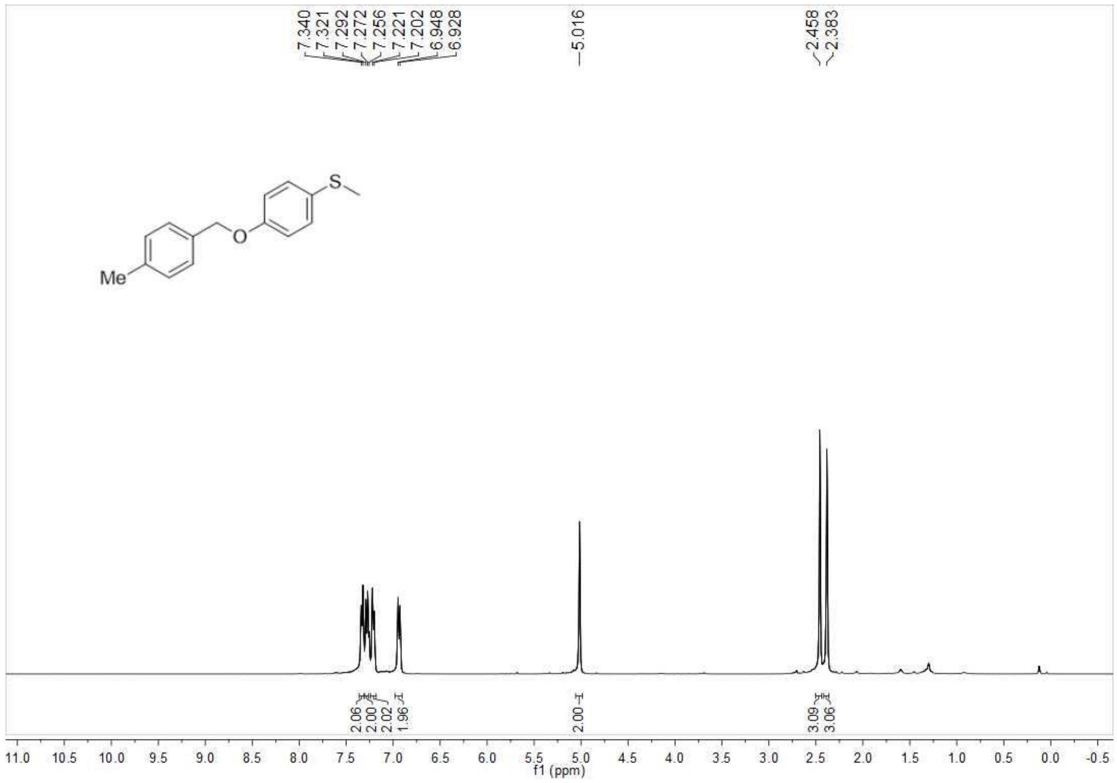

^{13}C NMR Spectrum of **108**

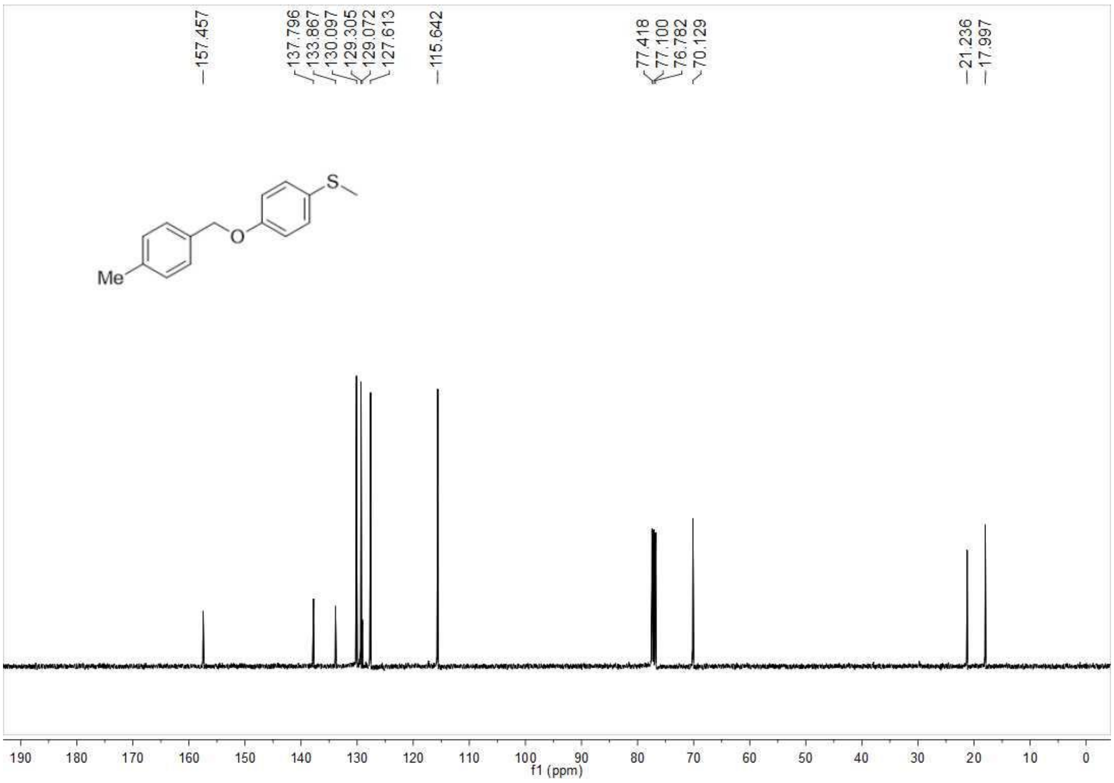

^{1}H NMR Spectrum of **109**

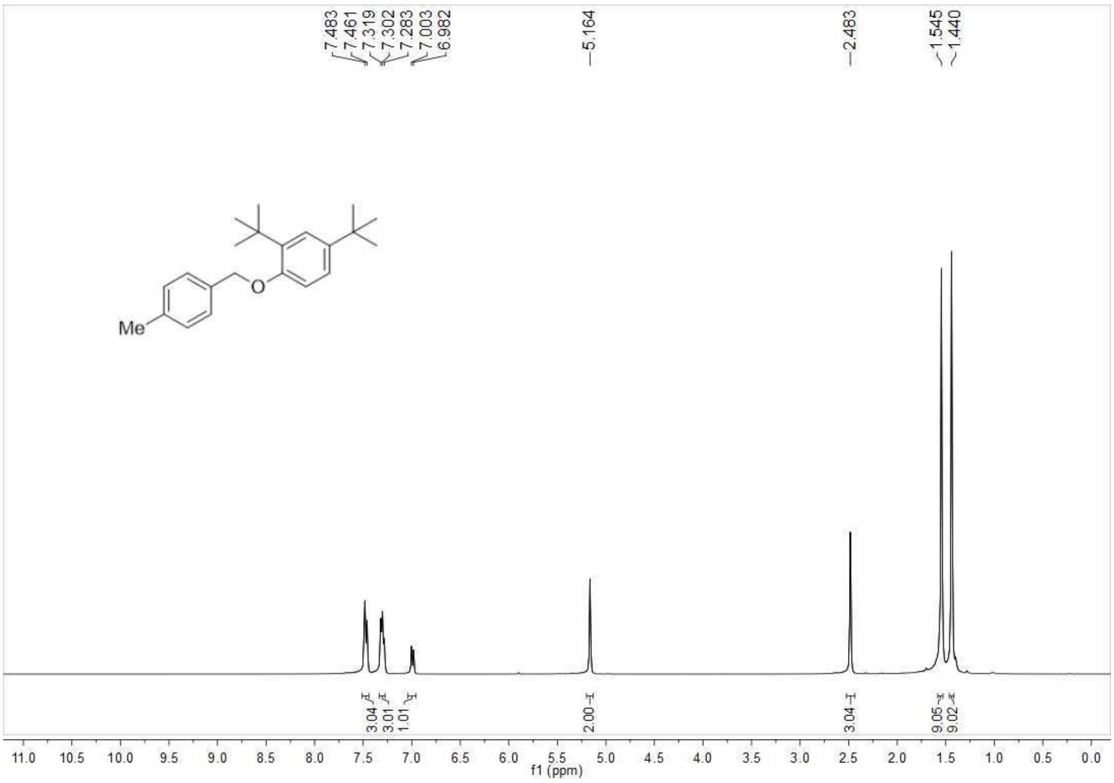

^{13}C NMR Spectrum of **109**

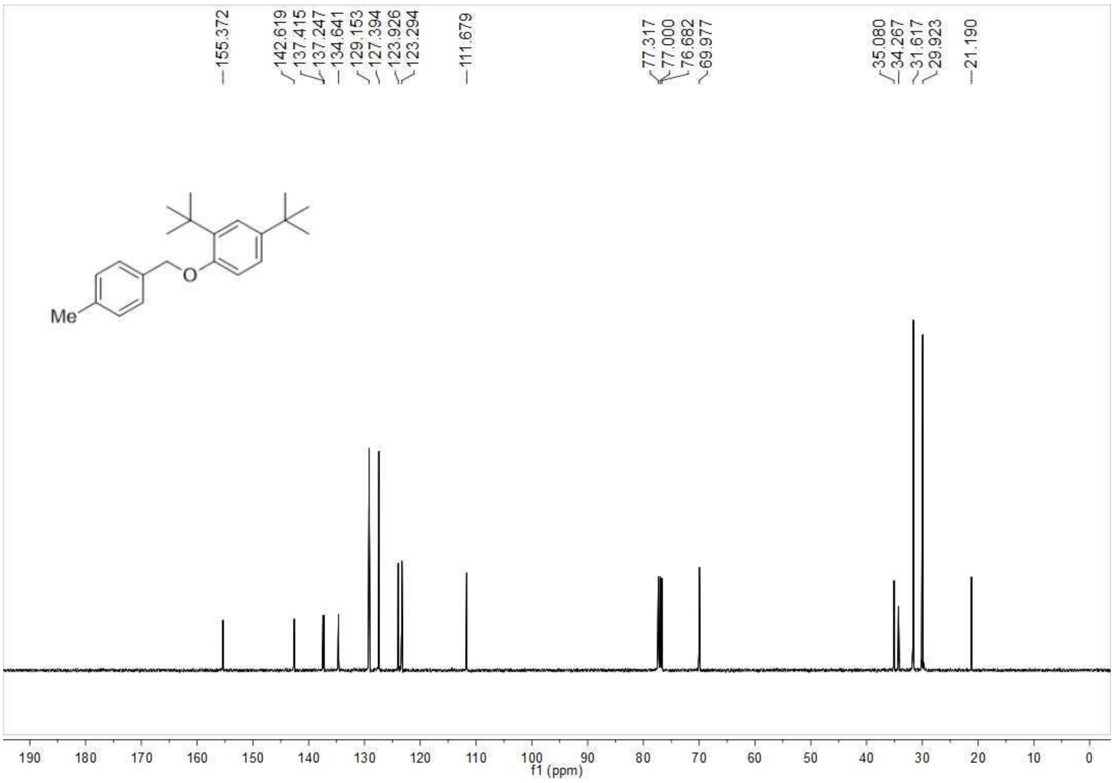

^{1}H NMR Spectrum of **110**

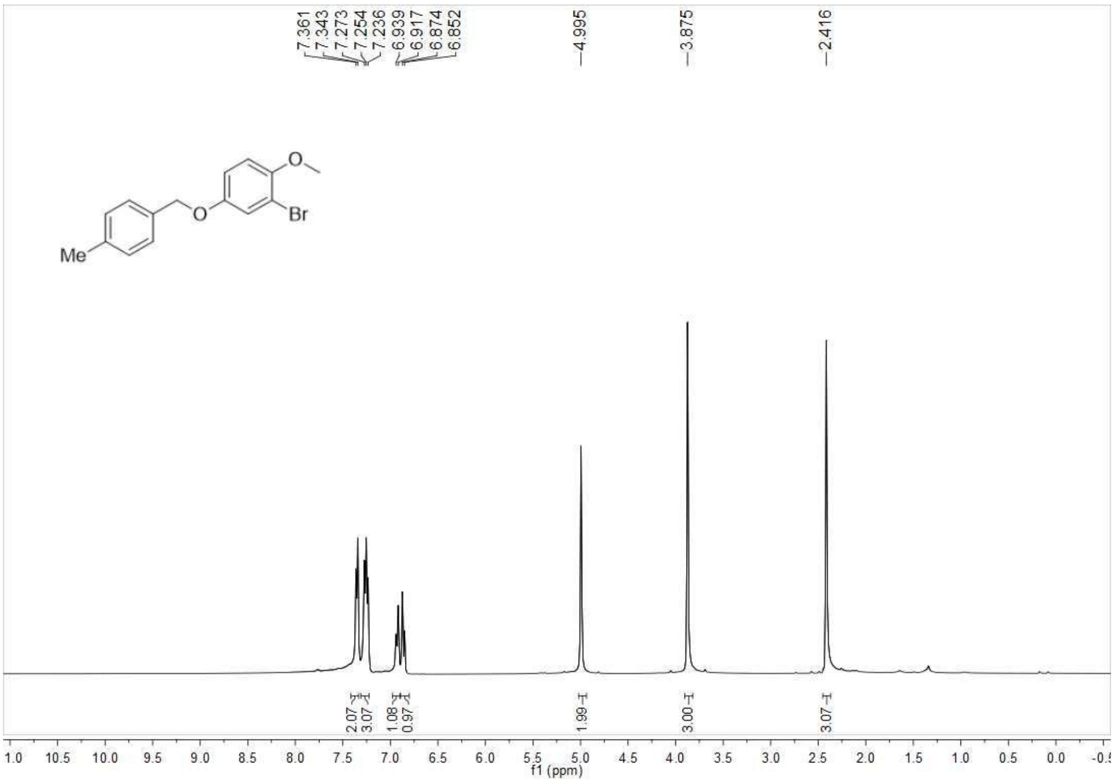

^{13}C NMR Spectrum of **110**

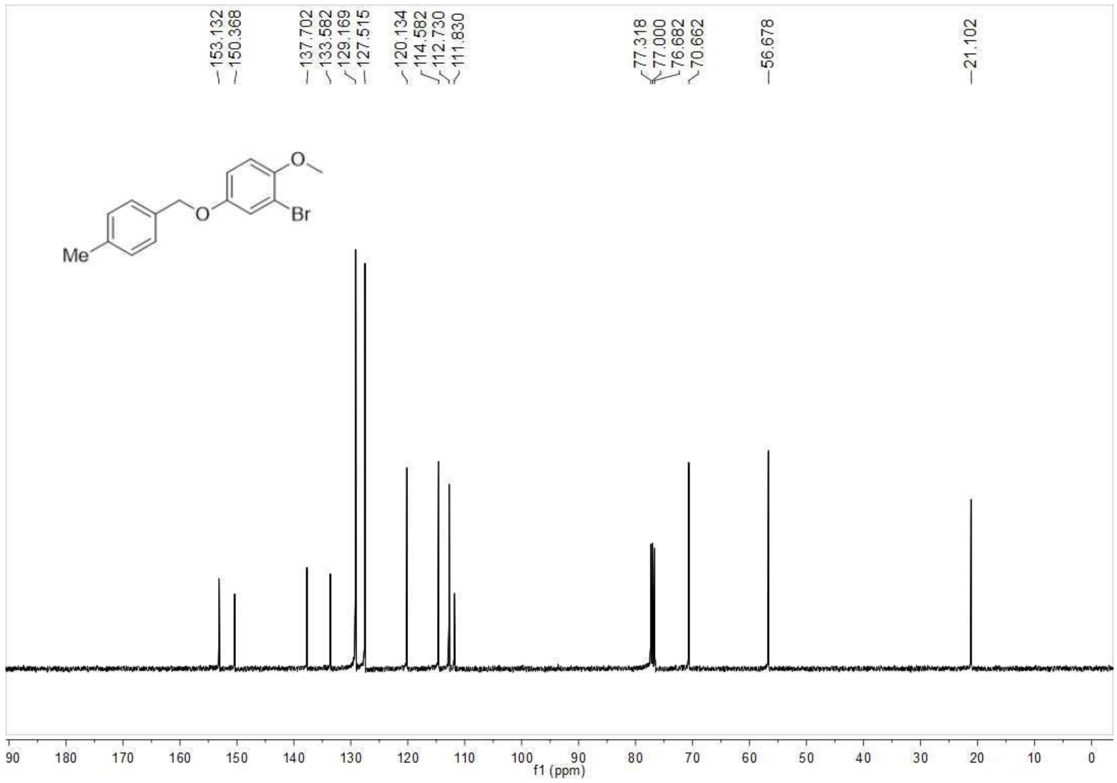

^{1}H NMR Spectrum of **111**

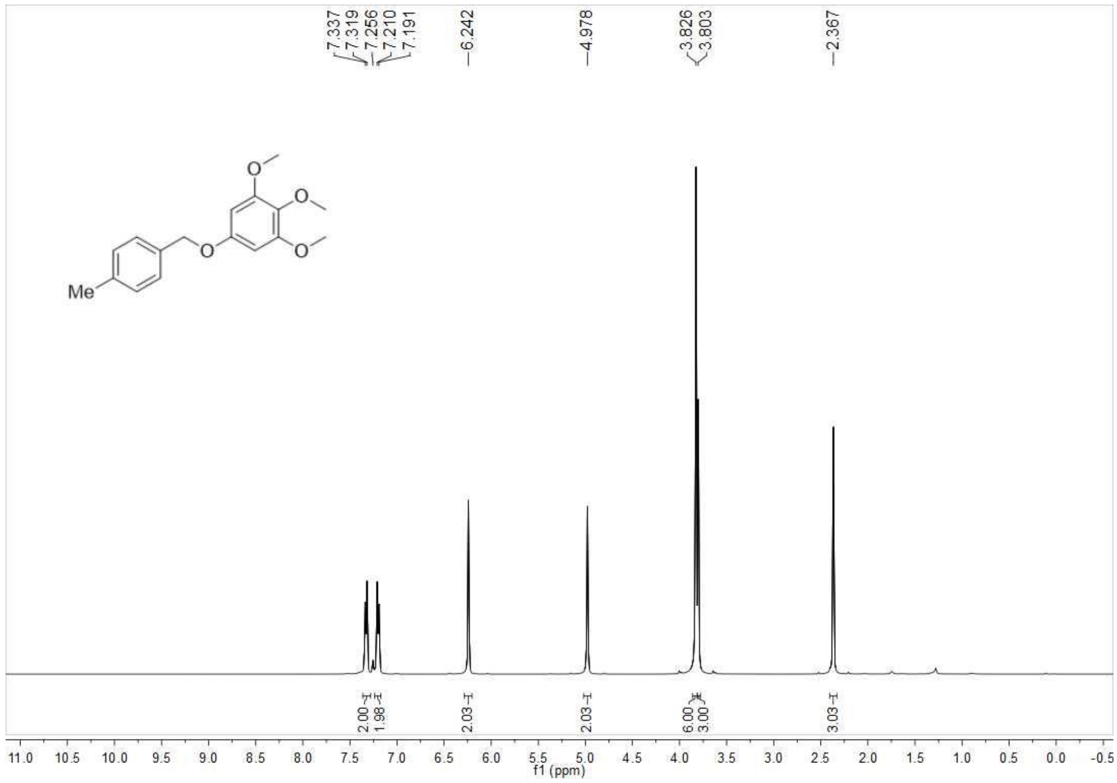

^{13}C NMR Spectrum of **111**

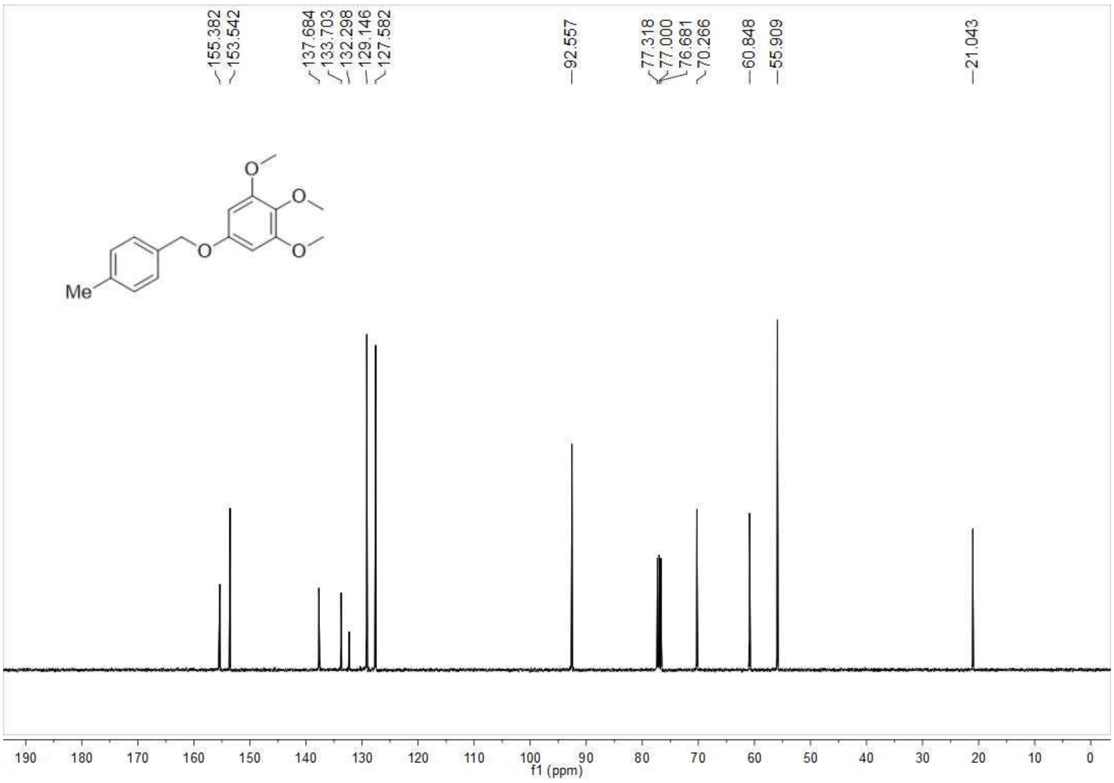

^{1}H NMR Spectrum of **112**

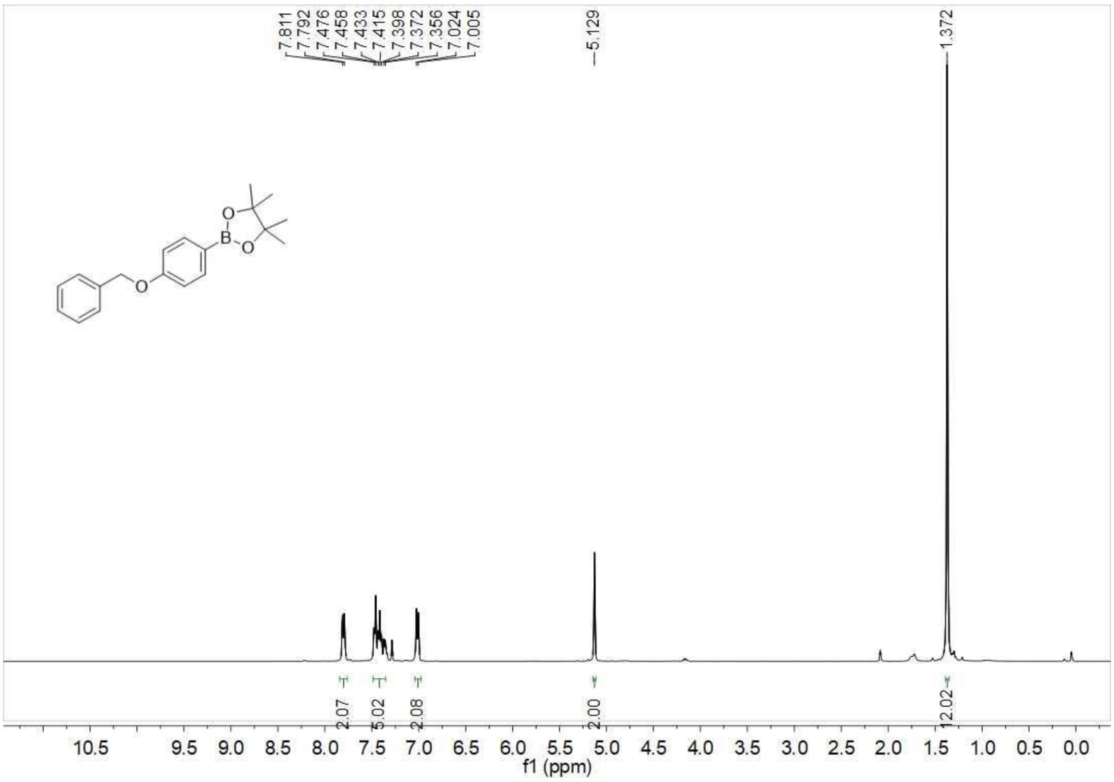

^{13}C NMR Spectrum of **112**

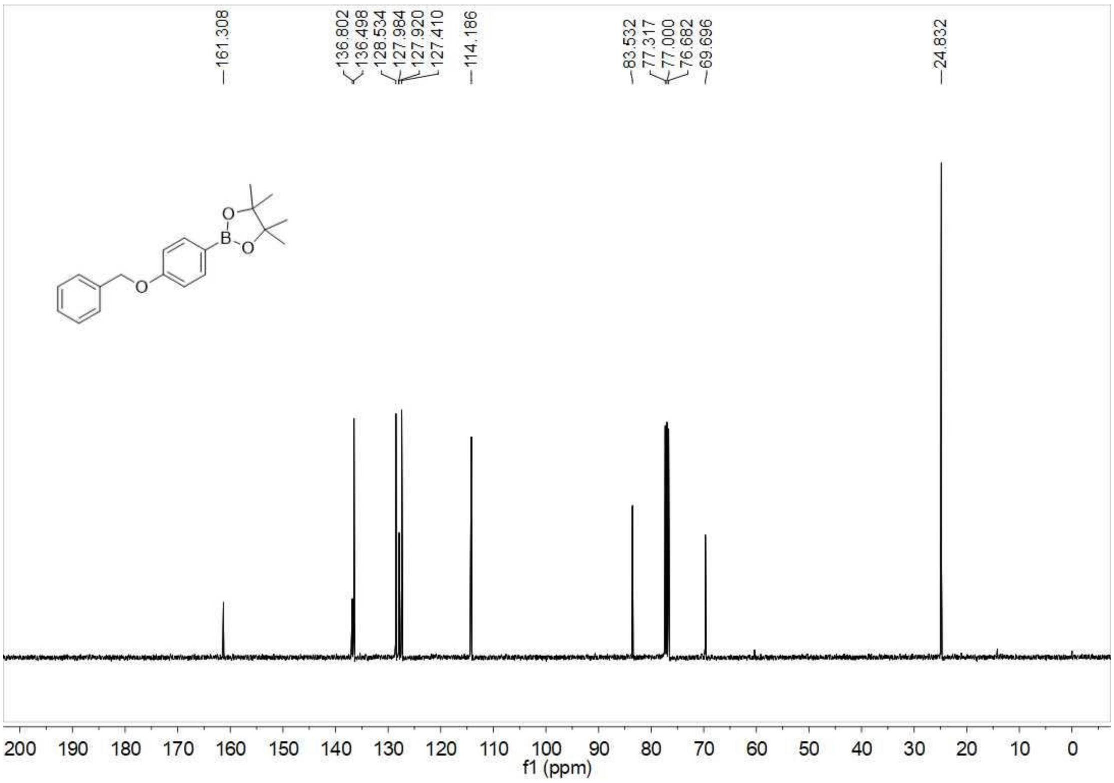

^{1}H NMR Spectrum of **113**

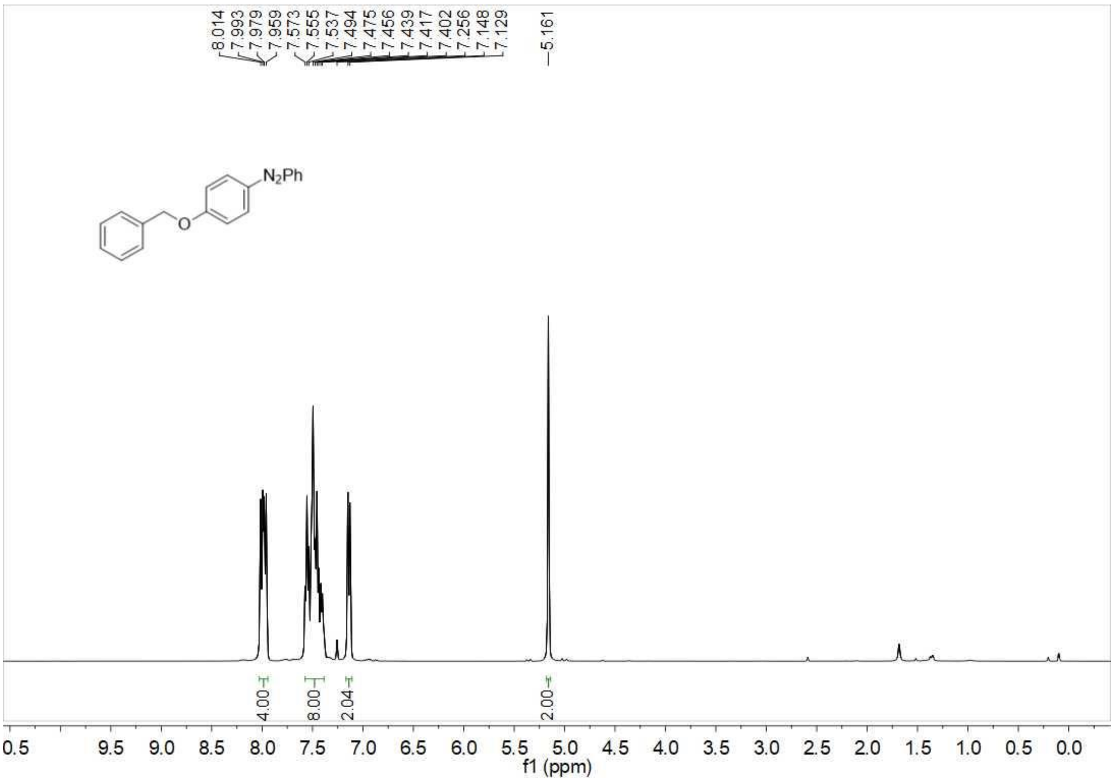

^{13}C NMR Spectrum of **113**

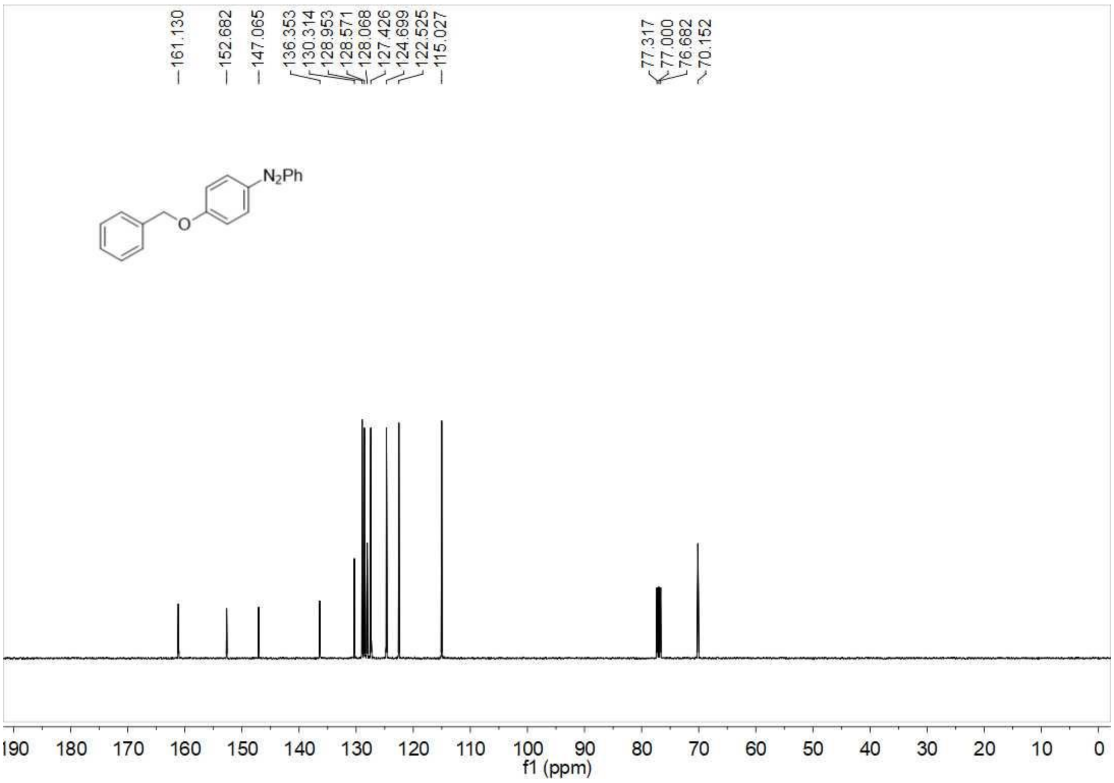

^{1}H NMR Spectrum of **114**

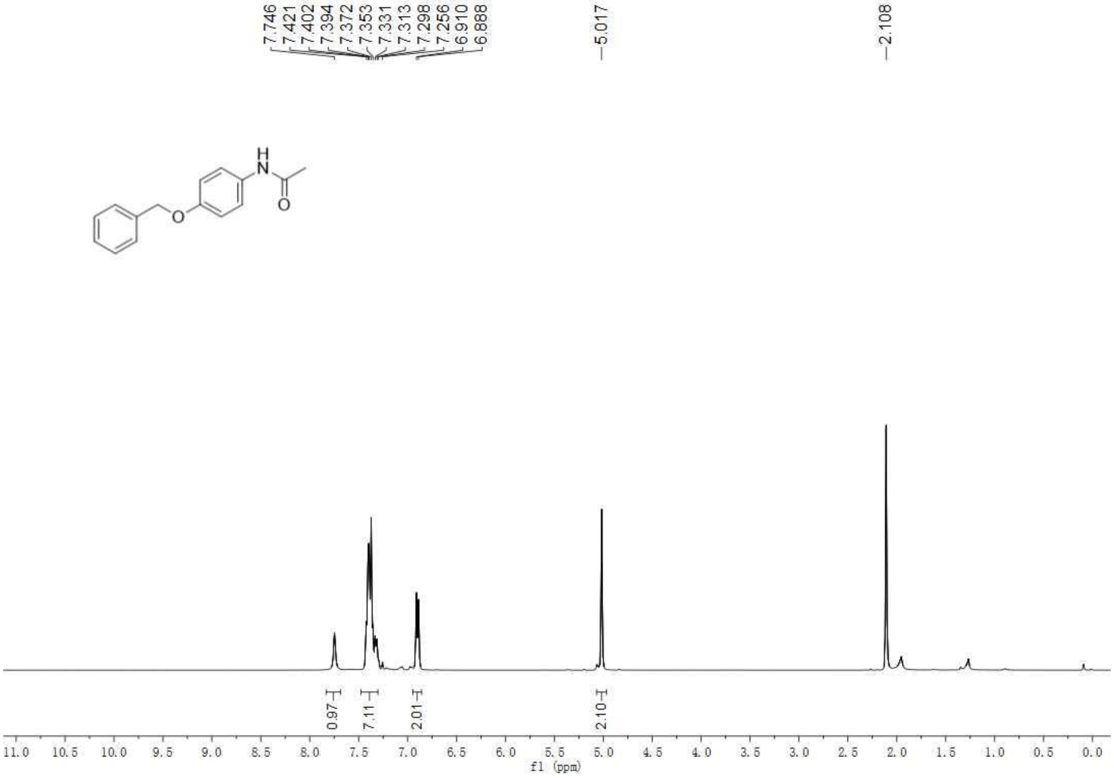

^{13}C NMR Spectrum of **114**

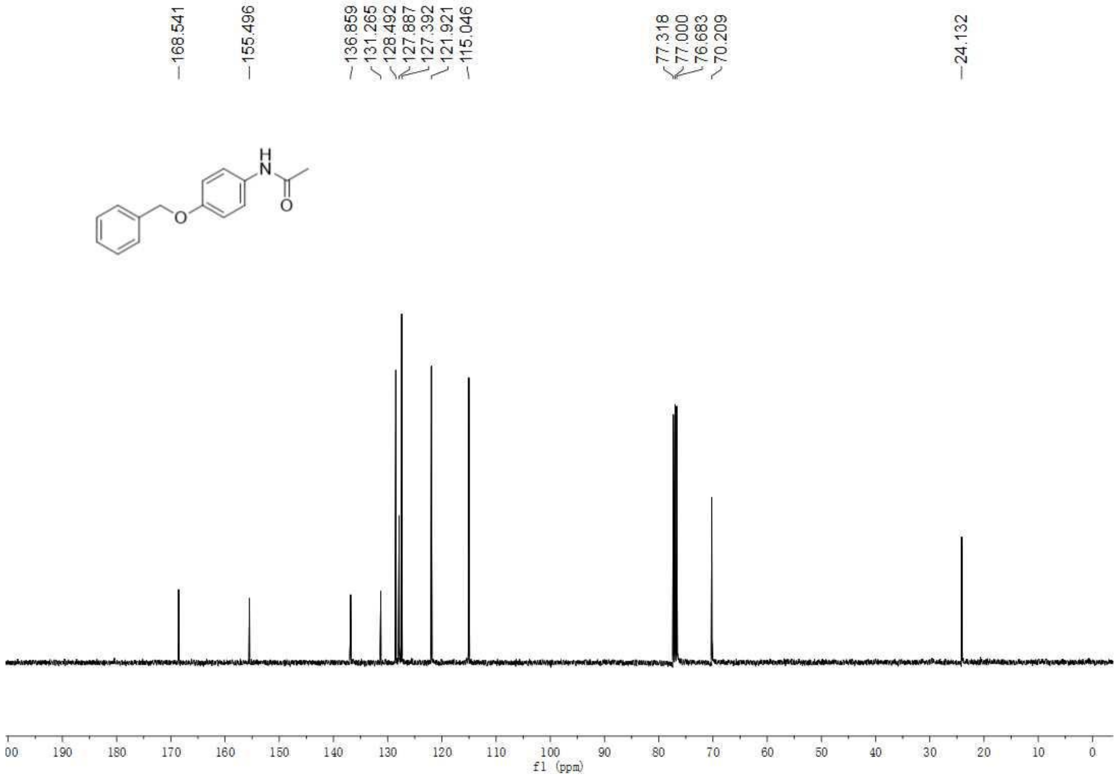

^{1}H NMR Spectrum of **115**

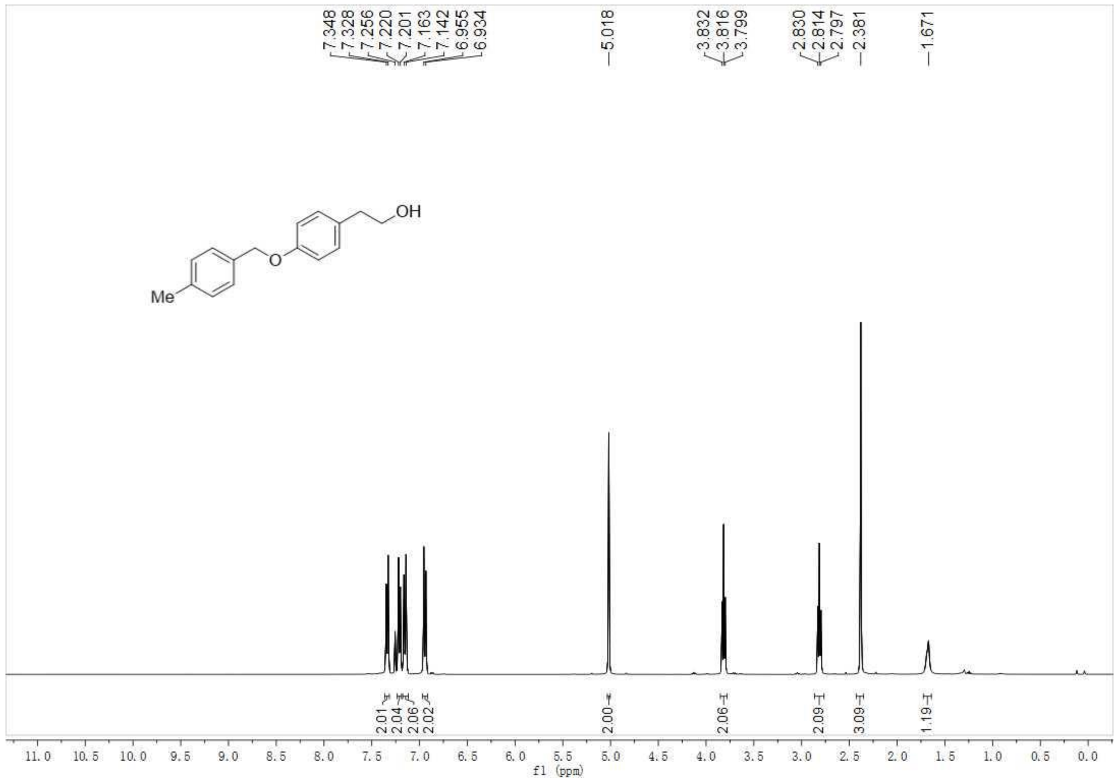

^{13}C NMR Spectrum of **115**

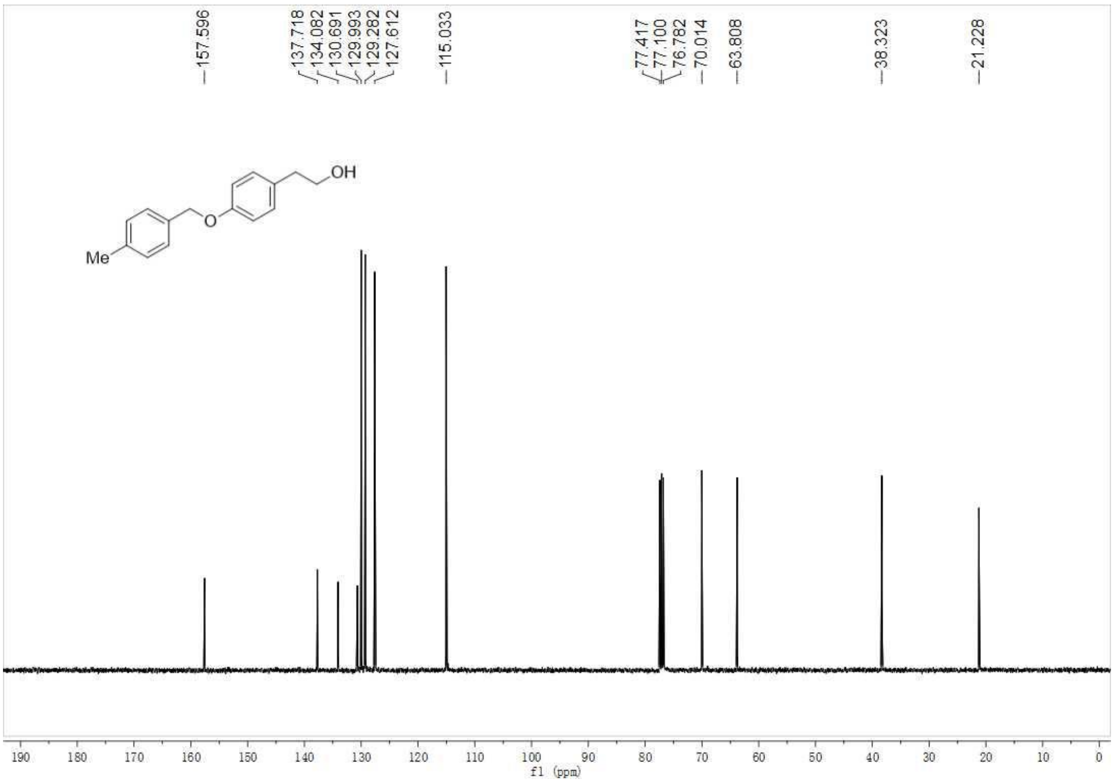

^{1}H NMR Spectrum of **116**

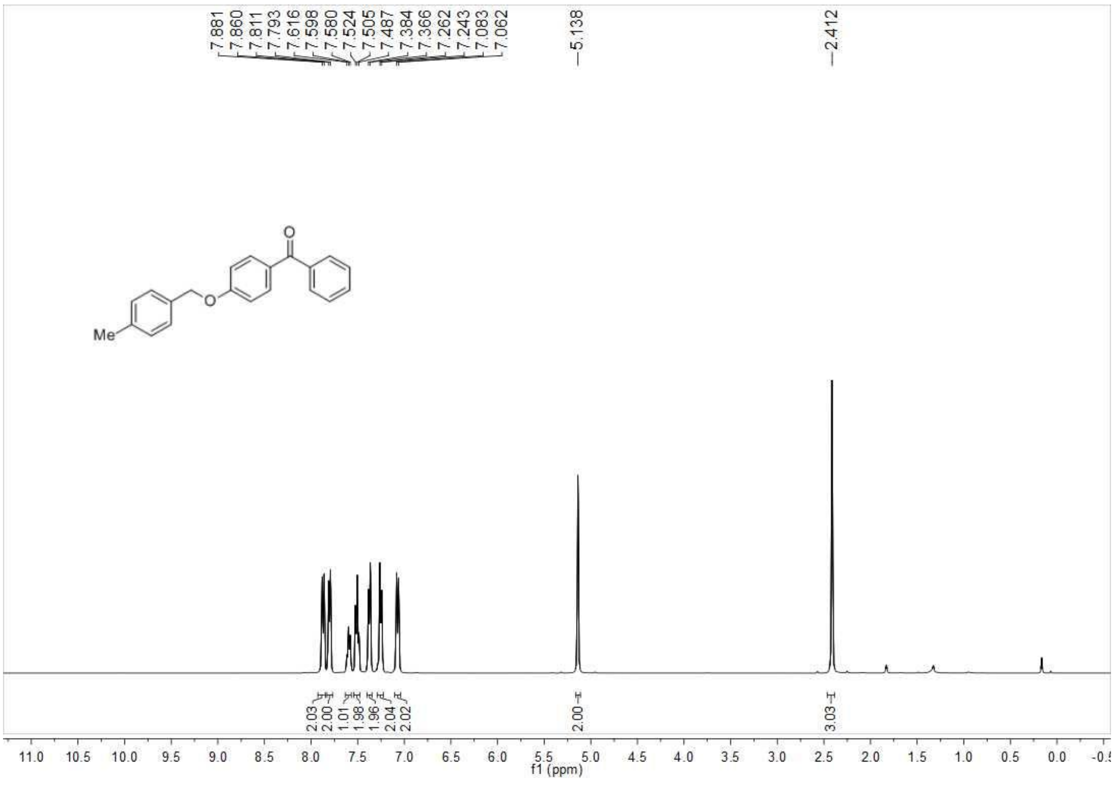

^{13}C NMR Spectrum of **116**

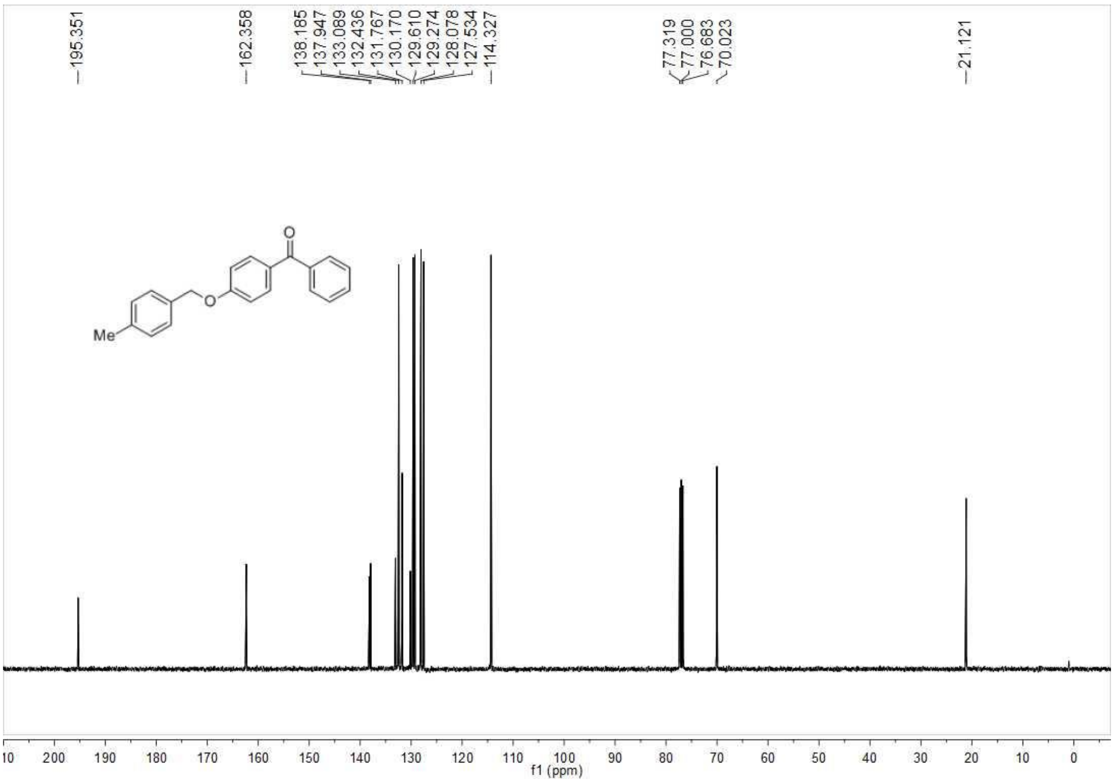

^{1}H NMR Spectrum of **117**

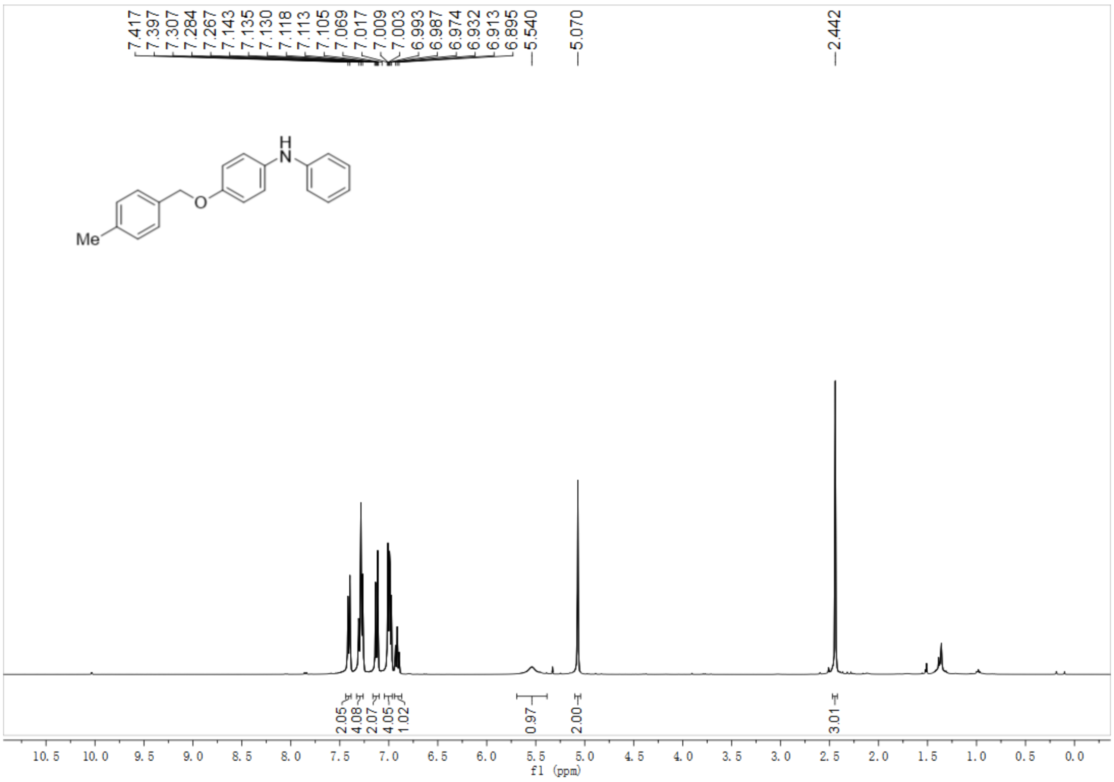

^{13}C NMR Spectrum of **117**

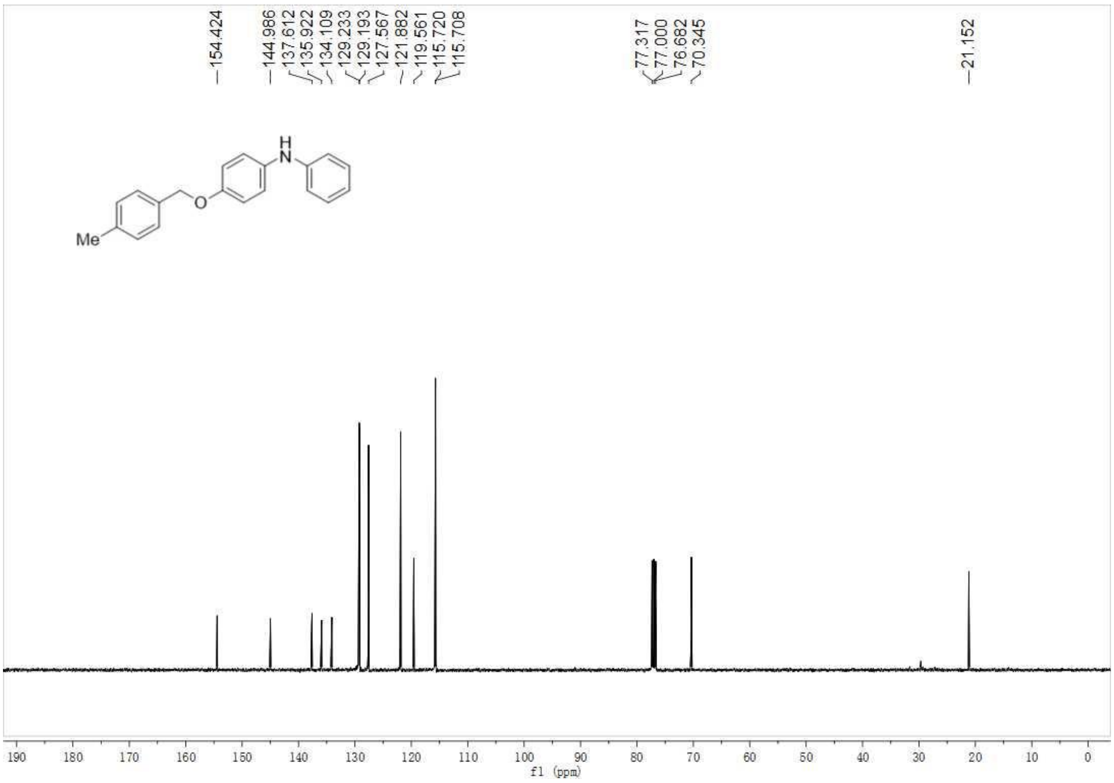

^{1}H NMR Spectrum of **118**

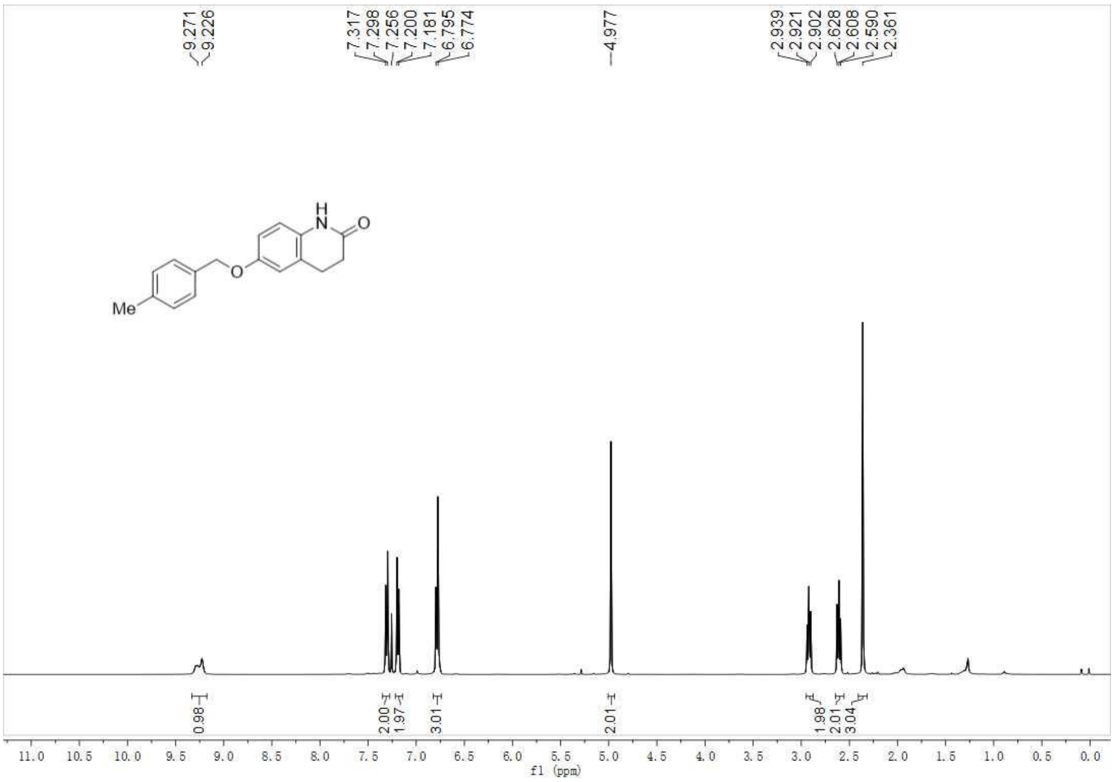

^{13}C NMR Spectrum of **118**

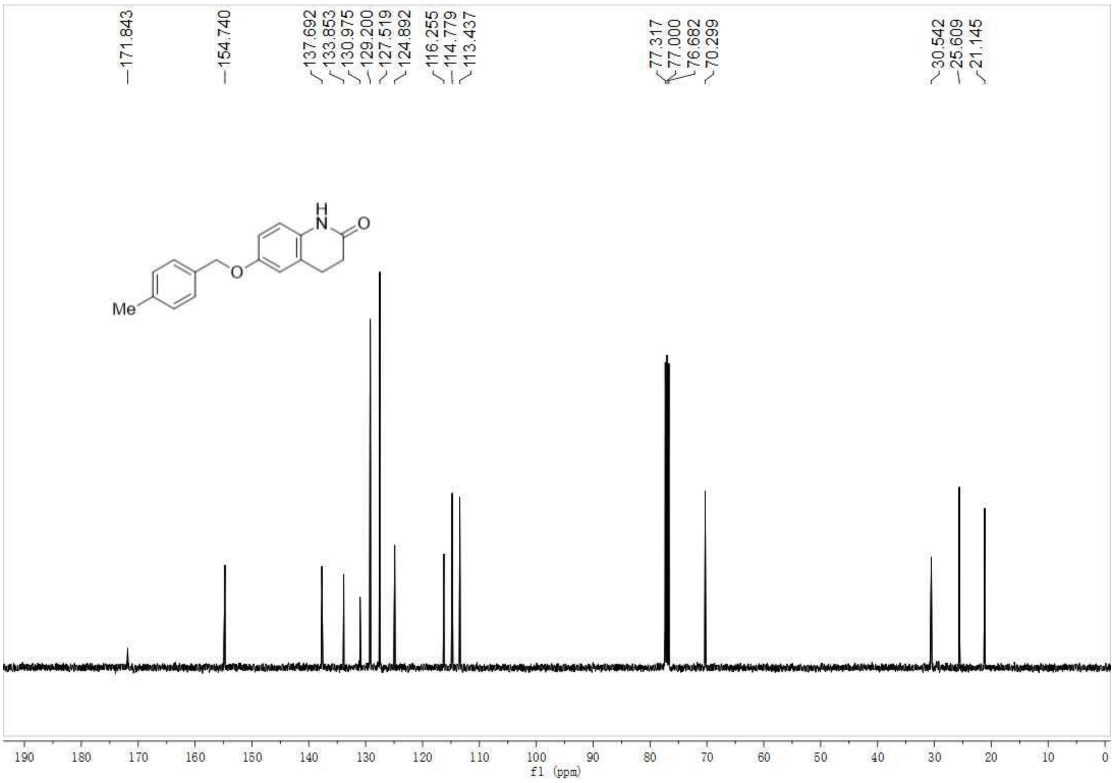

^{1}H NMR Spectrum of **119**

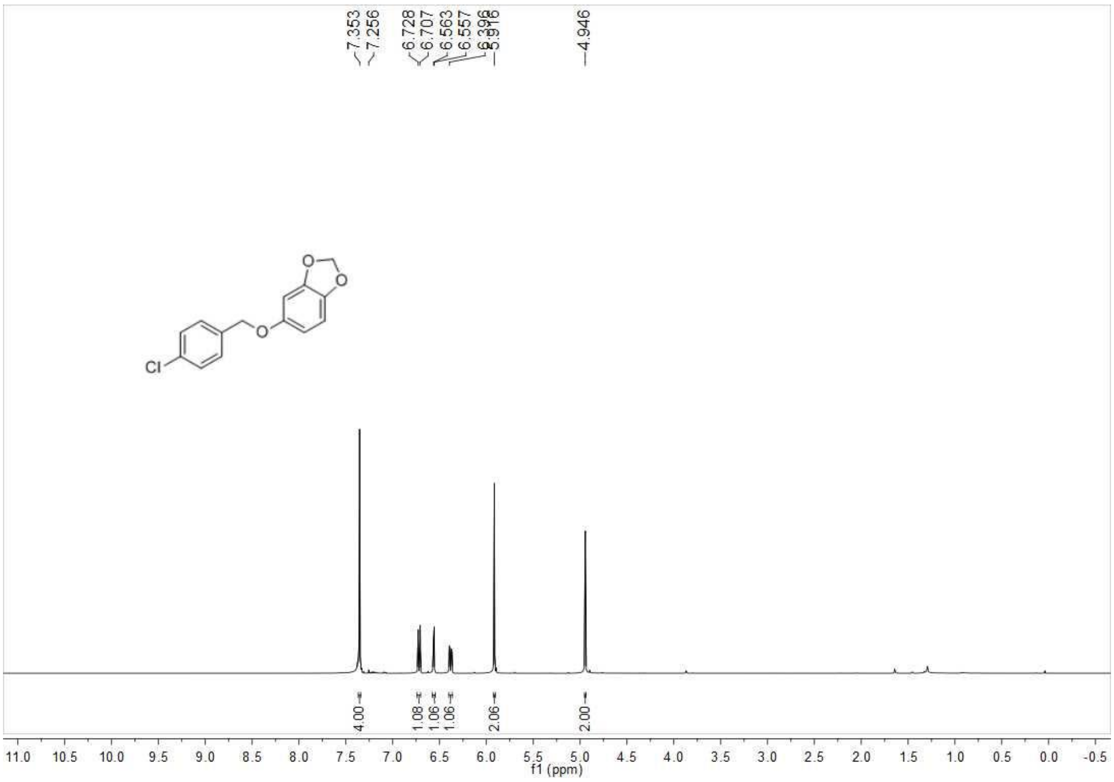

^{13}C NMR Spectrum of **119**

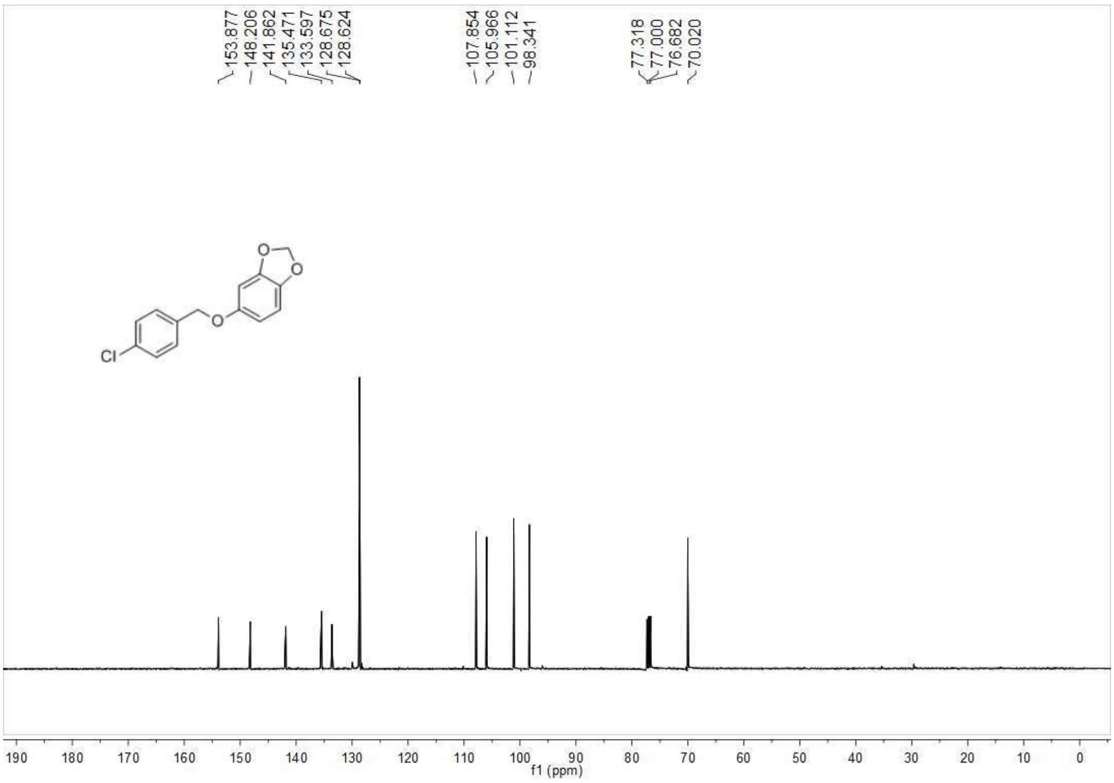

^{1}H NMR Spectrum of **120**

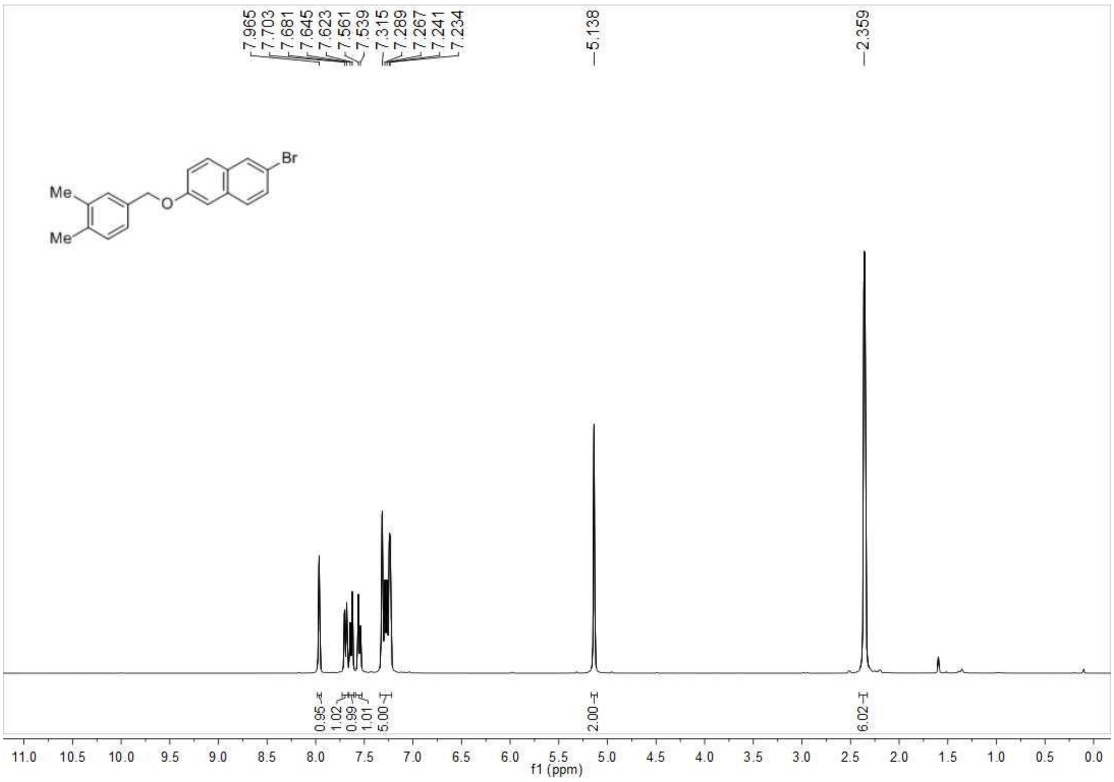

^{13}C NMR Spectrum of **120**

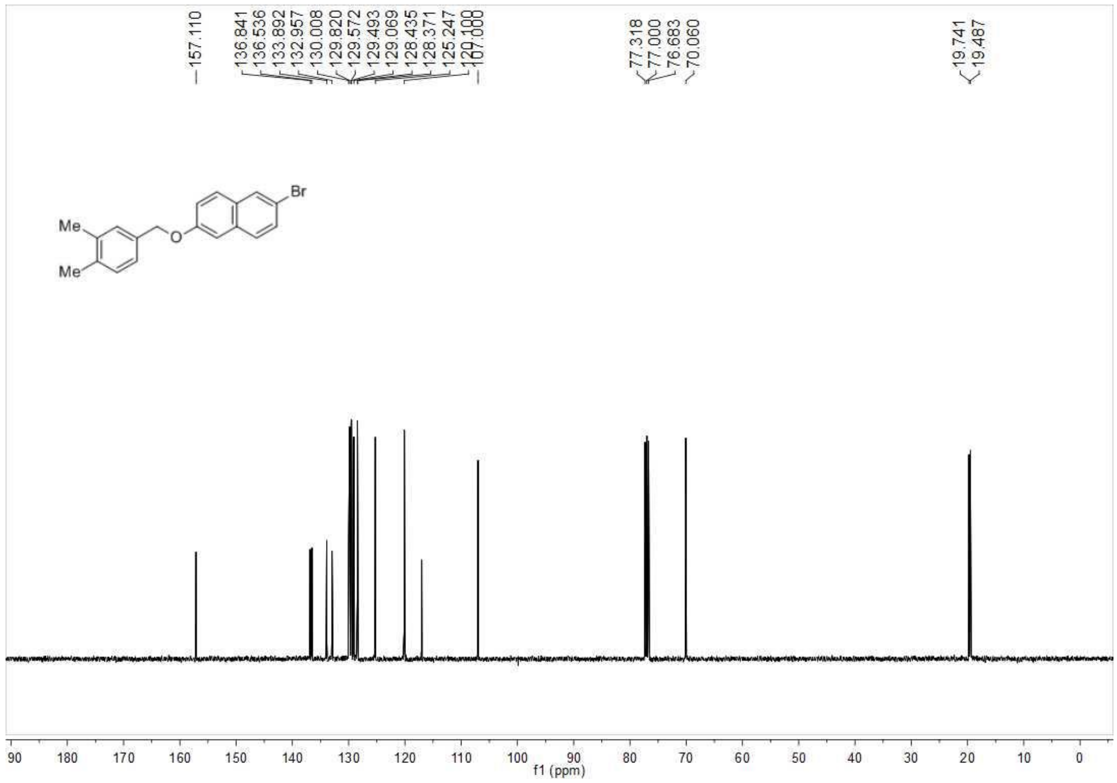

^{1}H NMR Spectrum of **121**

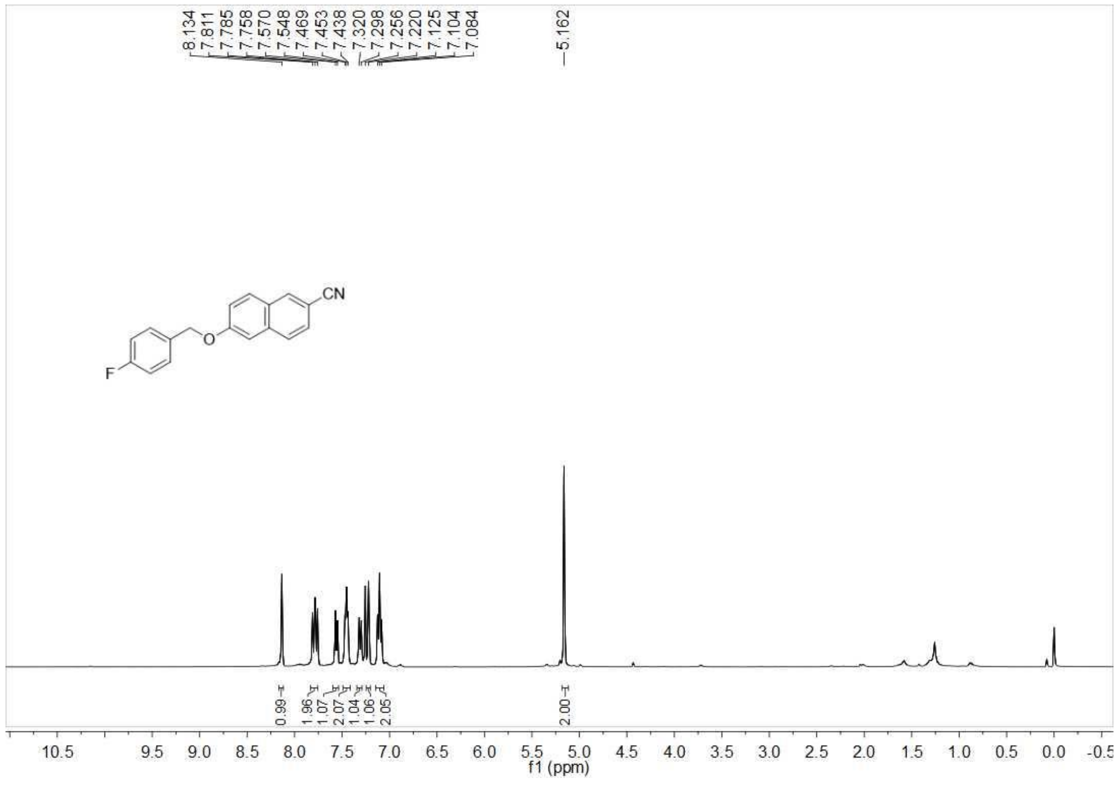

^{13}C NMR Spectrum of **121**

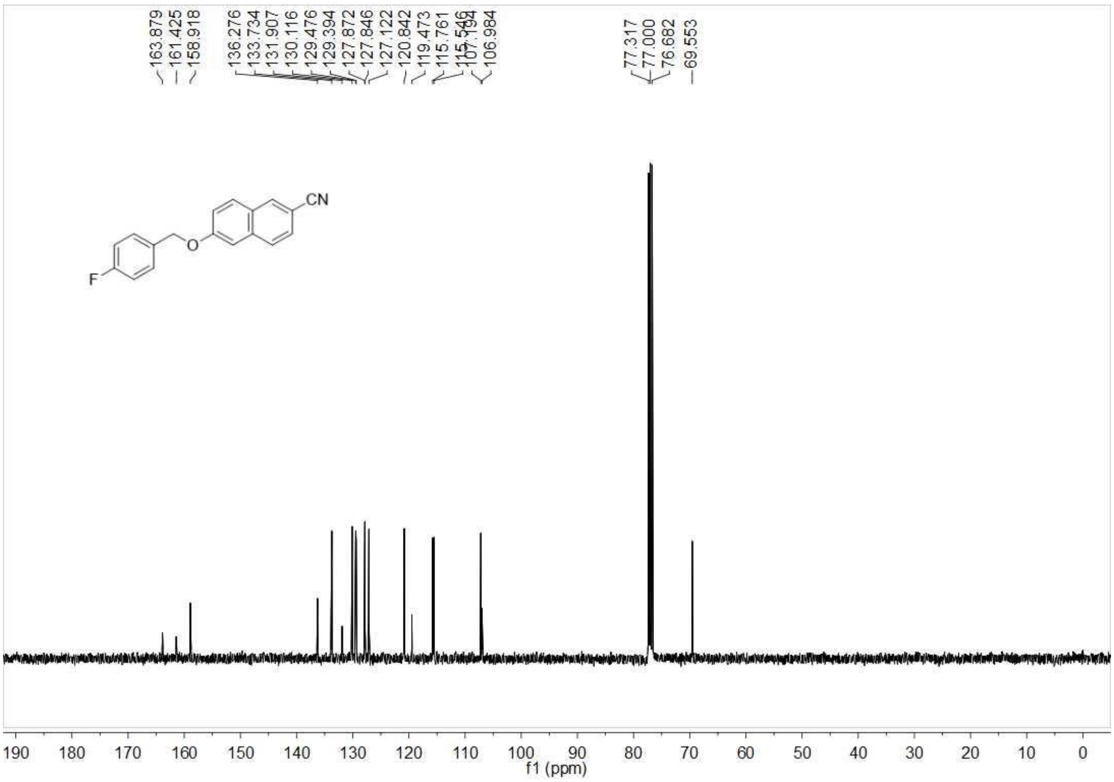

^{19}F NMR Spectrum of **121**

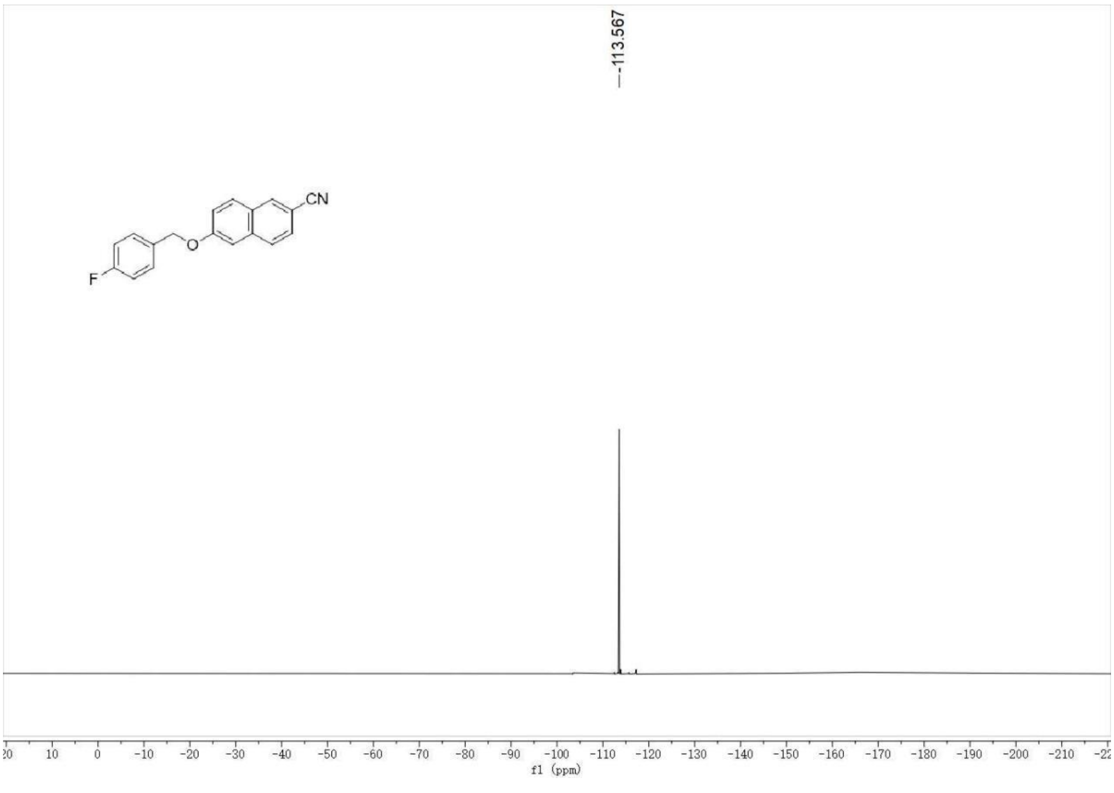

^{1}H NMR Spectrum of **122**

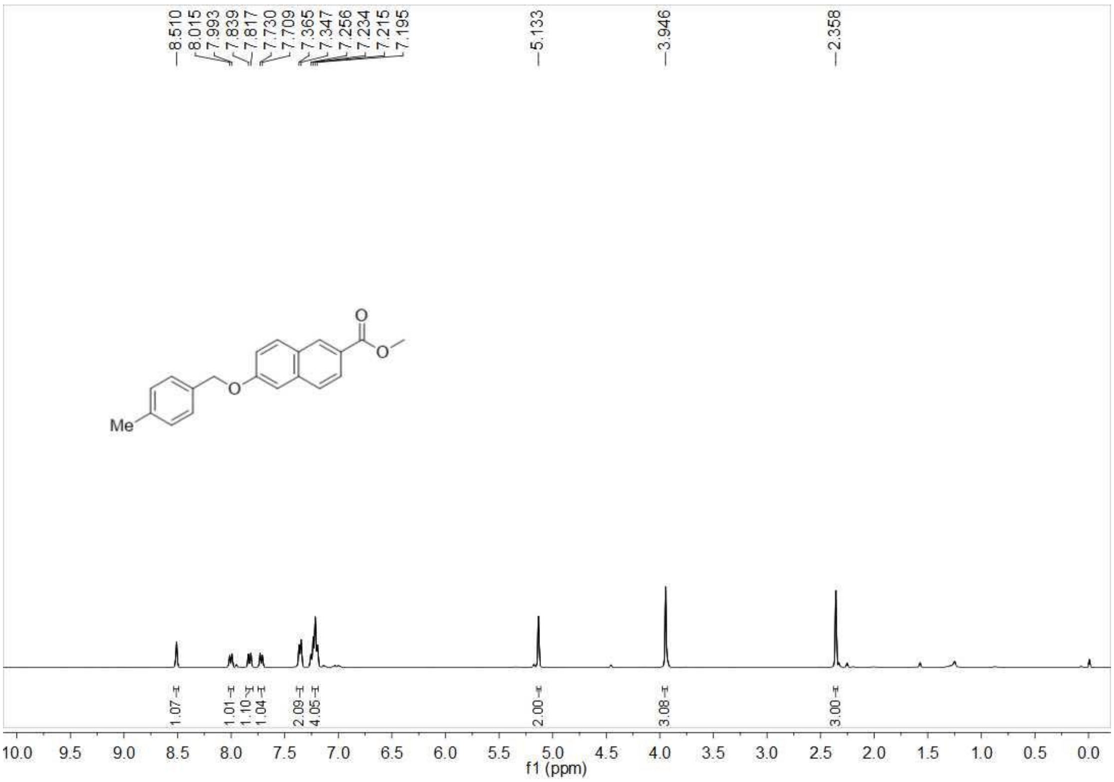

^{13}C NMR Spectrum of **122**

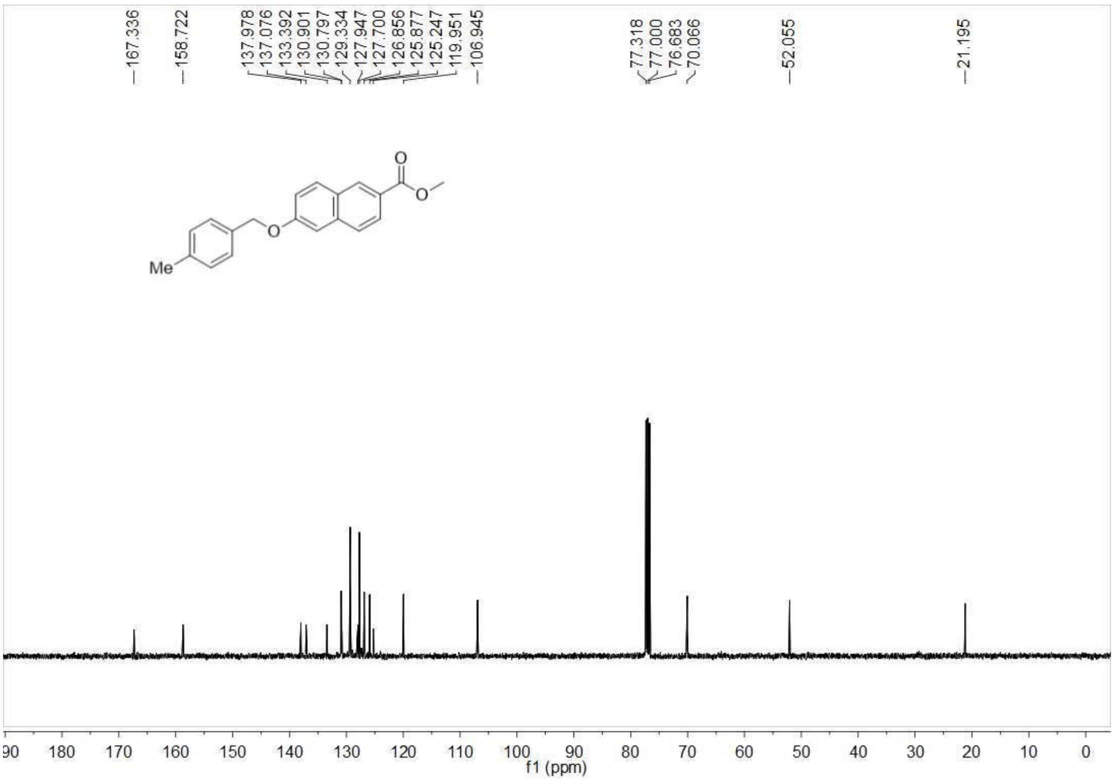

^{1}H NMR Spectrum of **122**

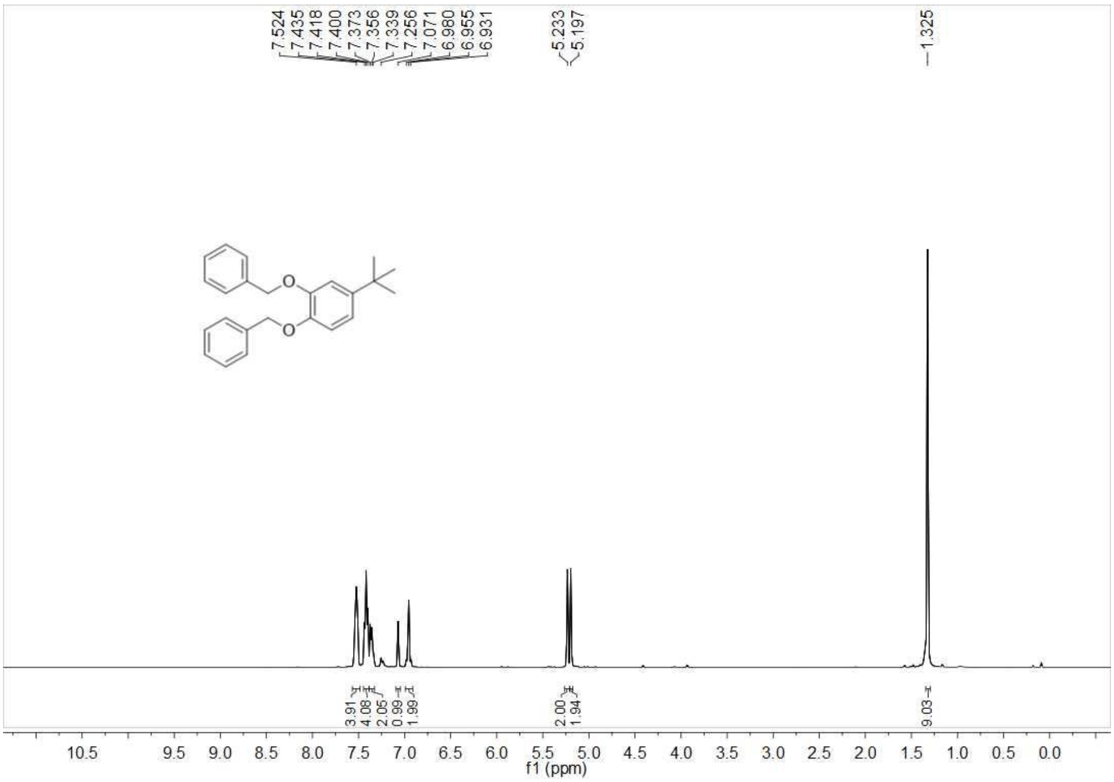

^{13}C NMR Spectrum of **123**

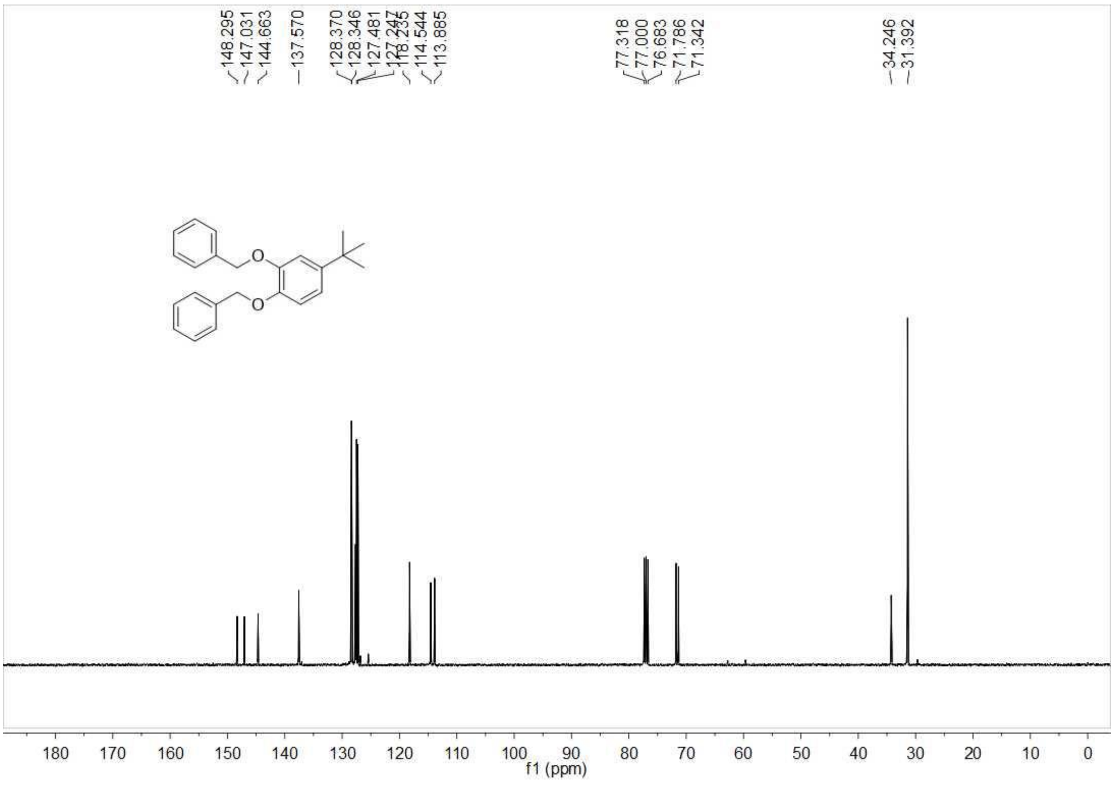

^{1}H NMR Spectrum of **124**

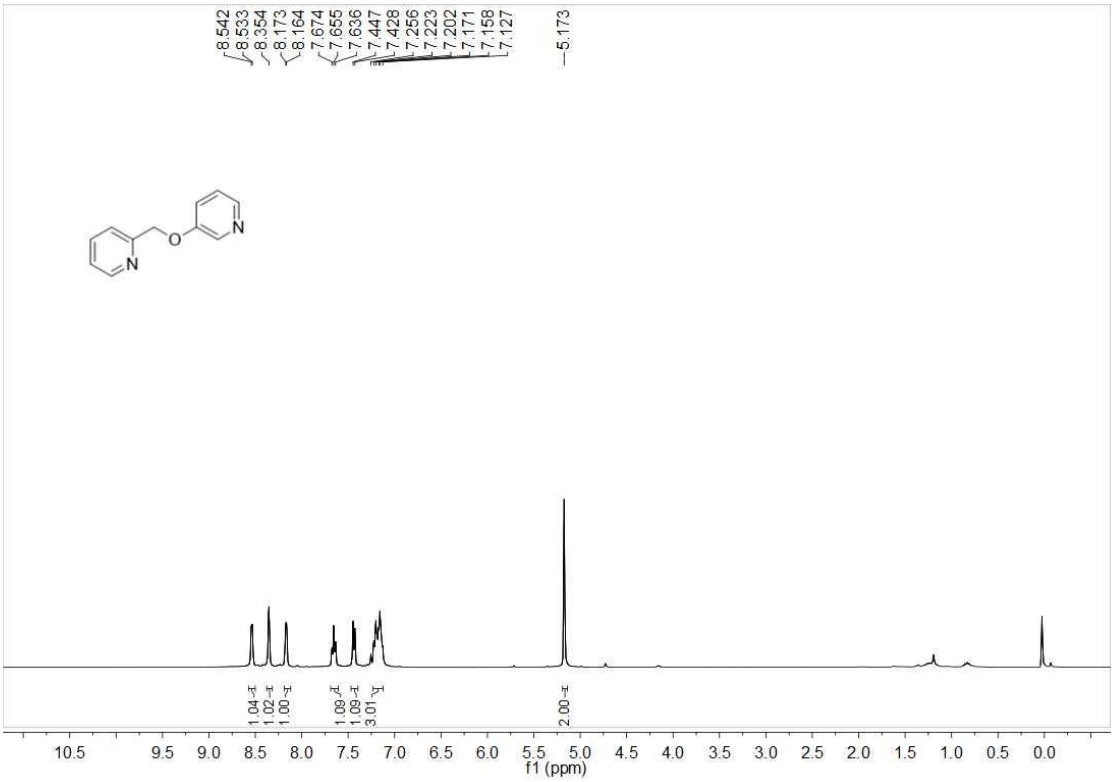

^{13}C NMR Spectrum of **124**

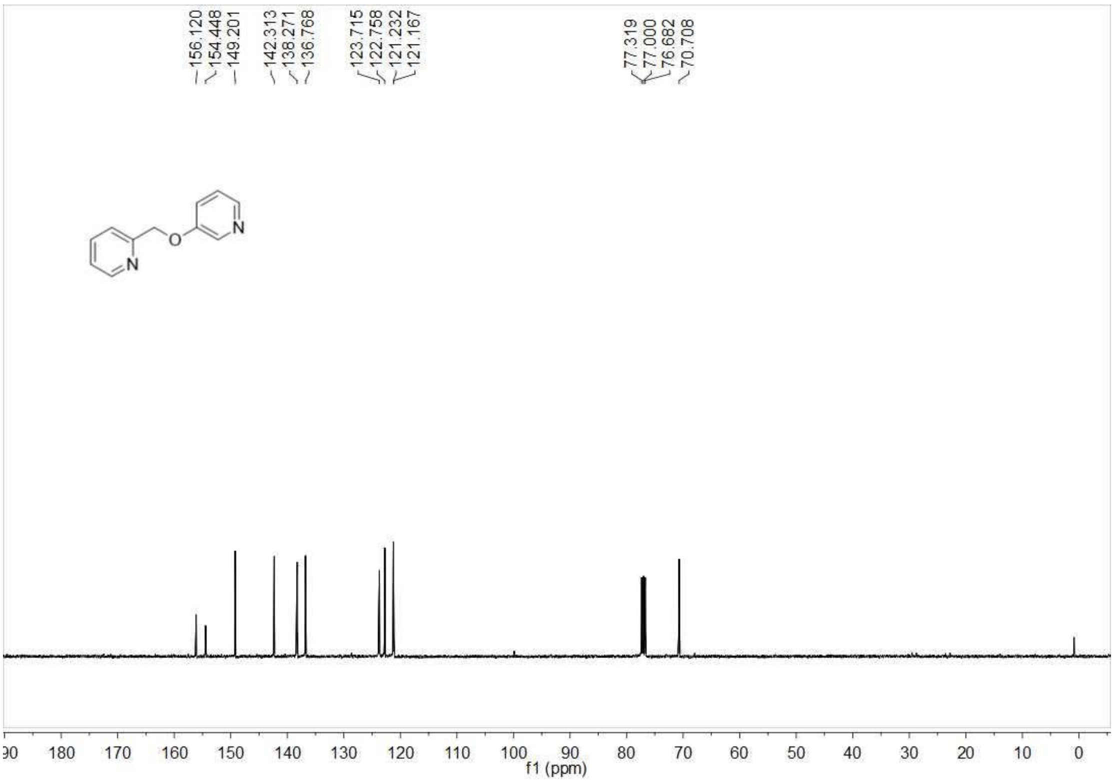

^{1}H NMR Spectrum of **125**

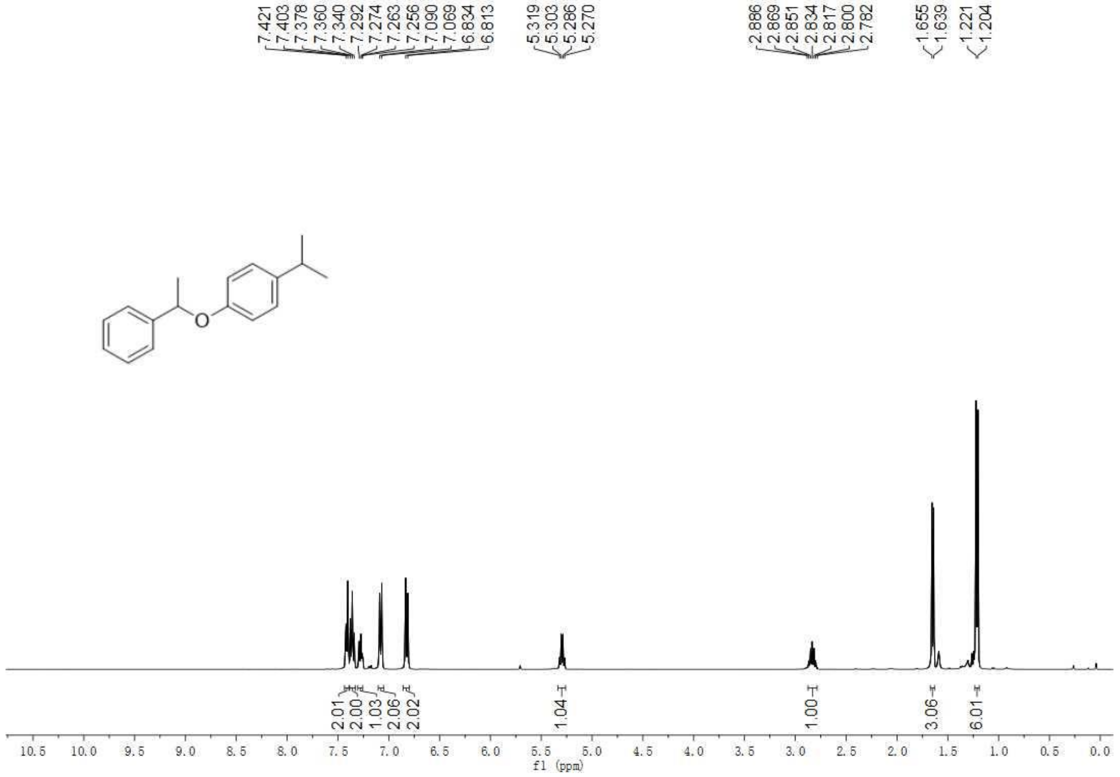

^{13}C NMR Spectrum of **125**

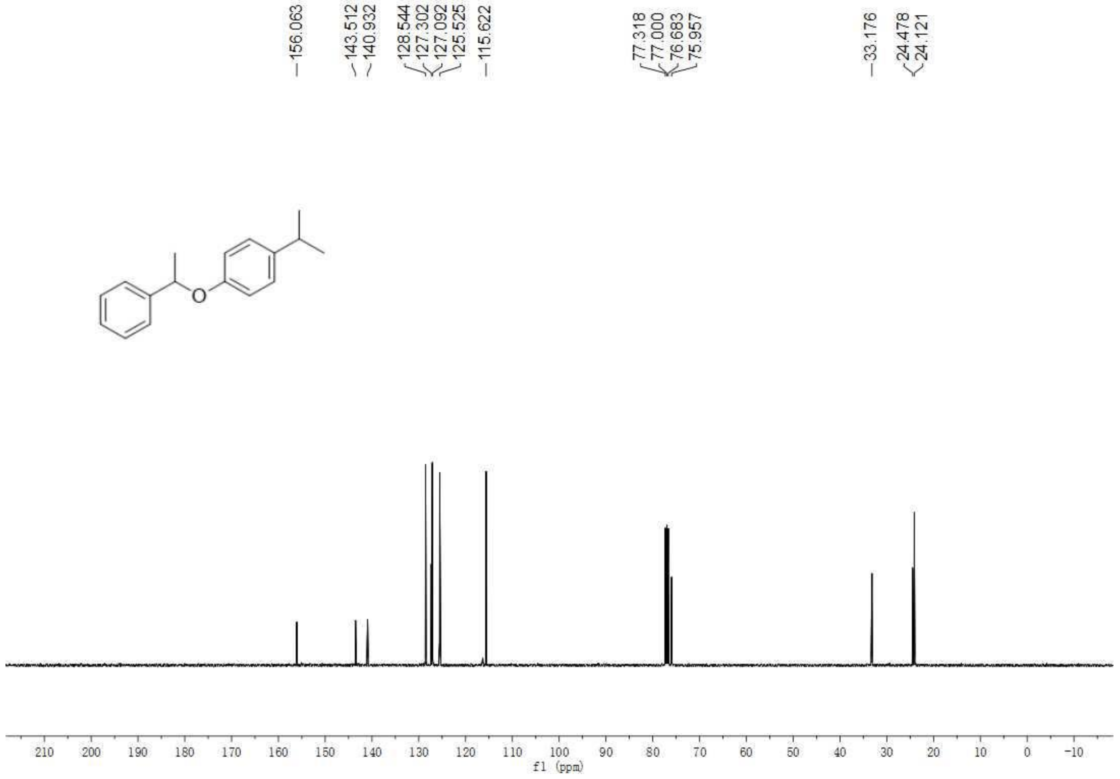

^{1}H NMR Spectrum of **126**

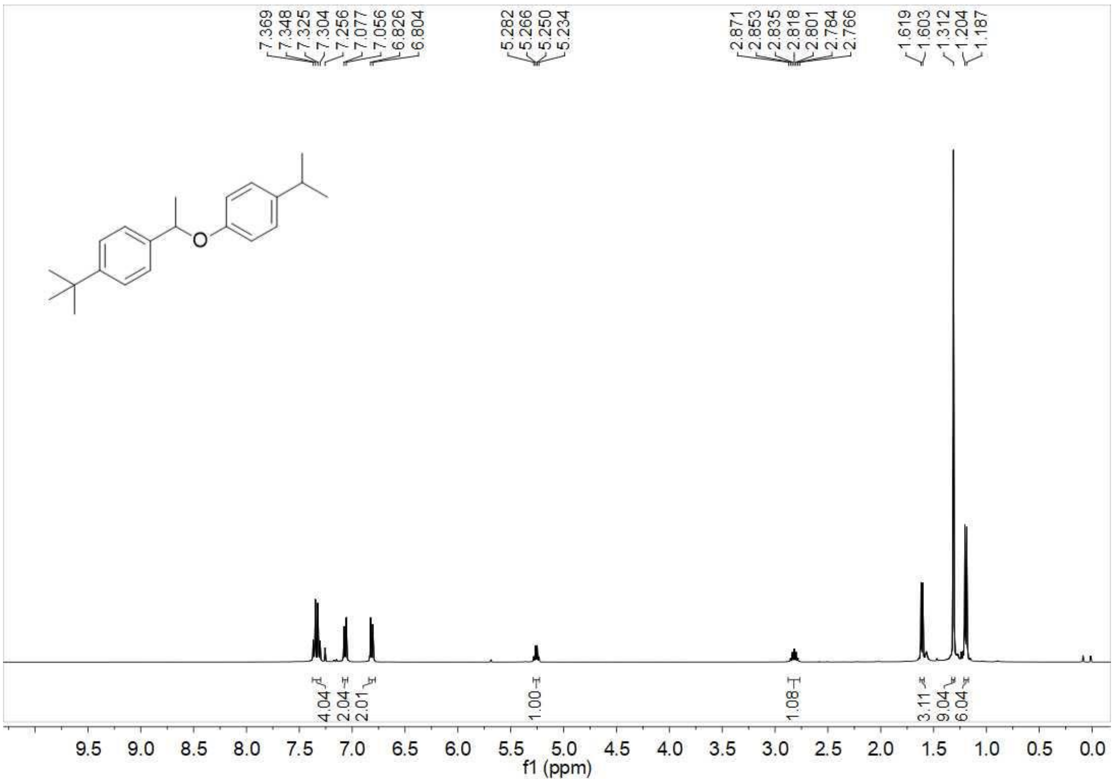

^{13}C NMR Spectrum of **126**

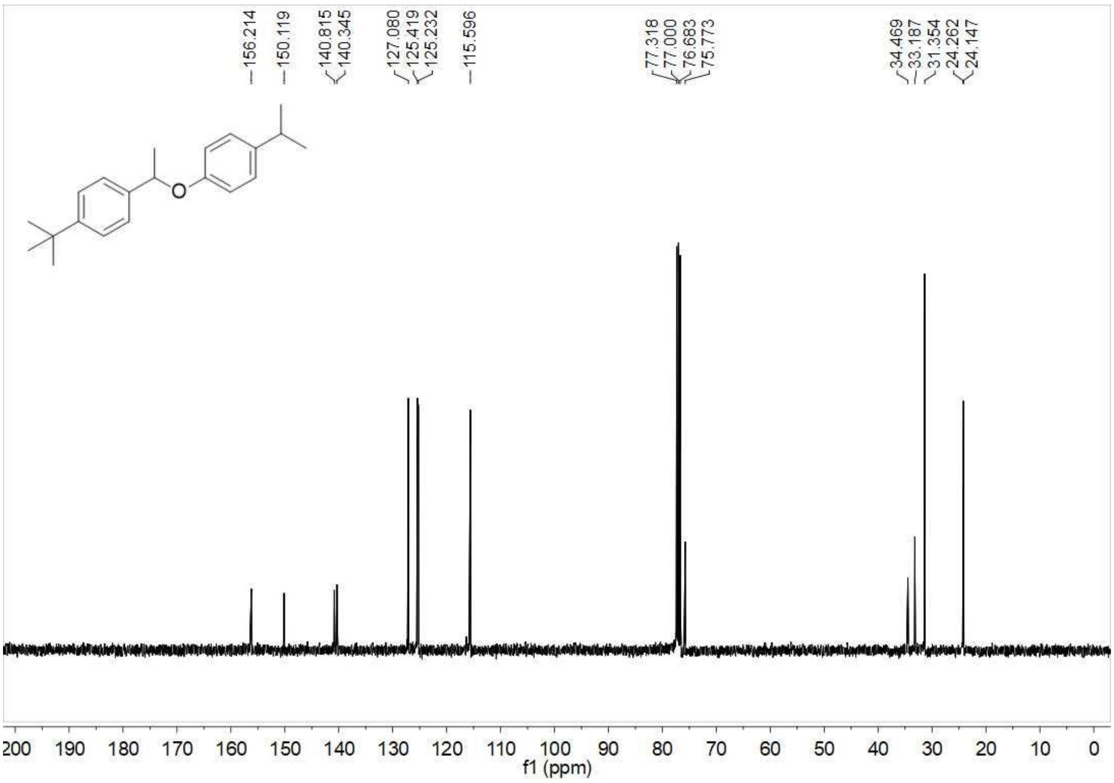

^{1}H NMR Spectrum of **127**

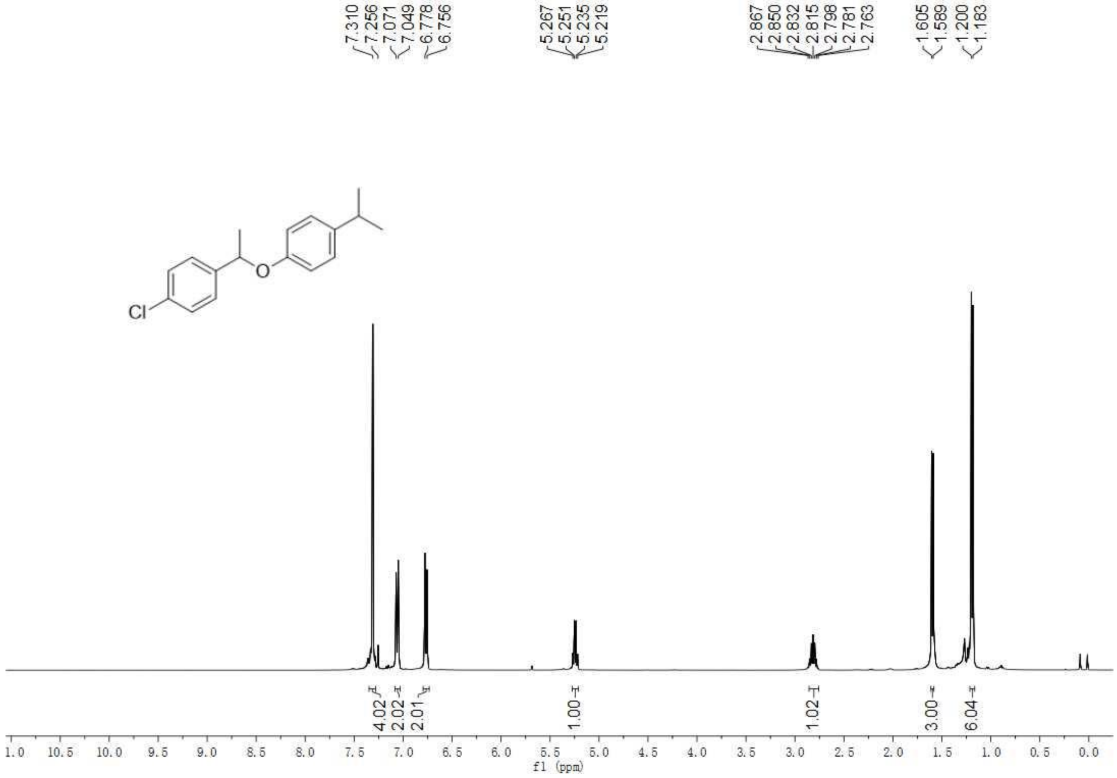

^{13}C NMR Spectrum of **127**

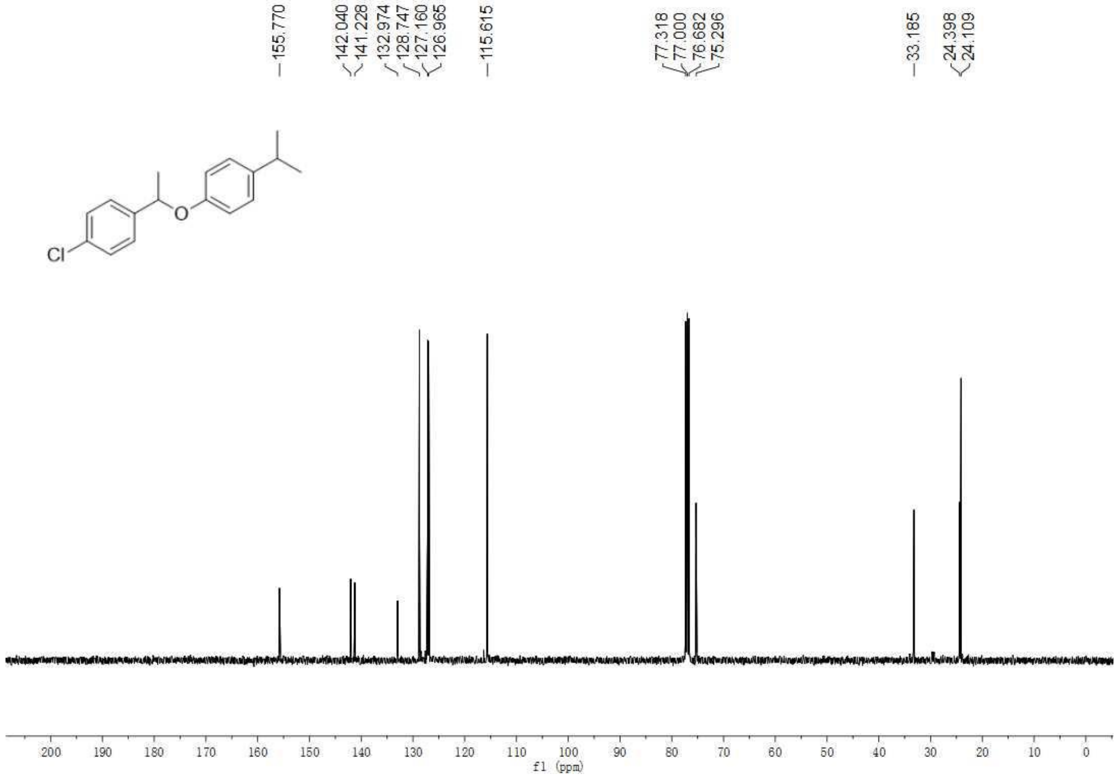

^{1}H NMR Spectrum of **128**

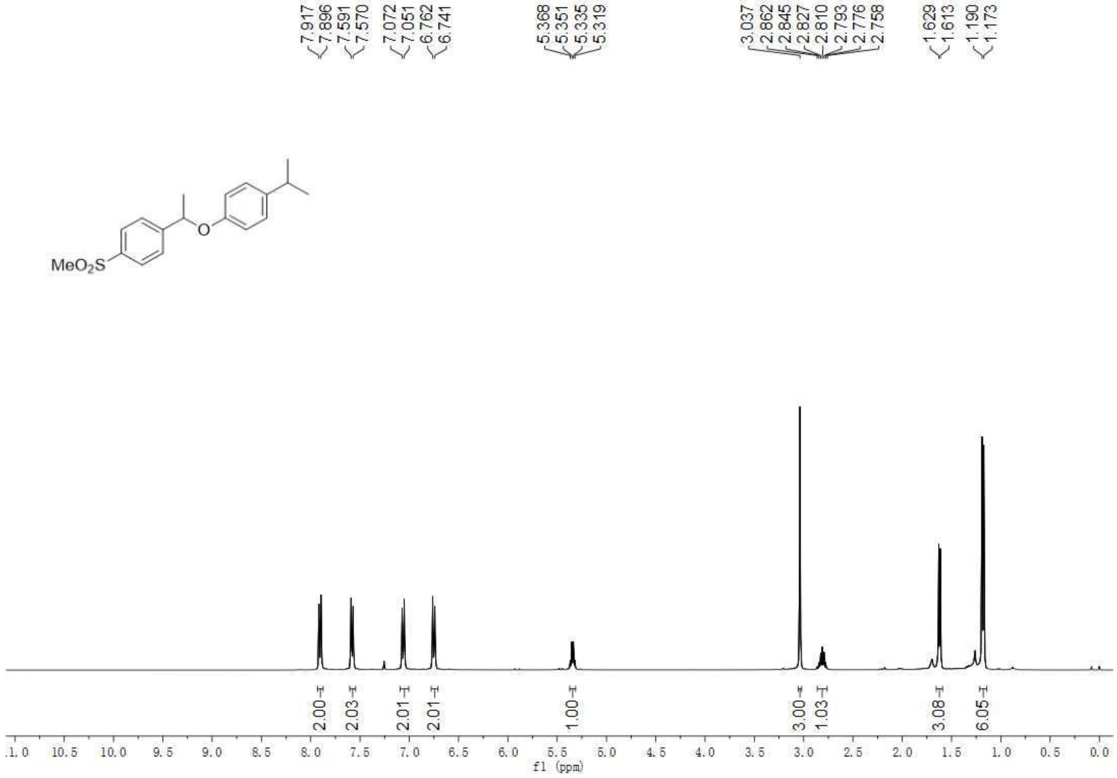

^{13}C NMR Spectrum of **128**

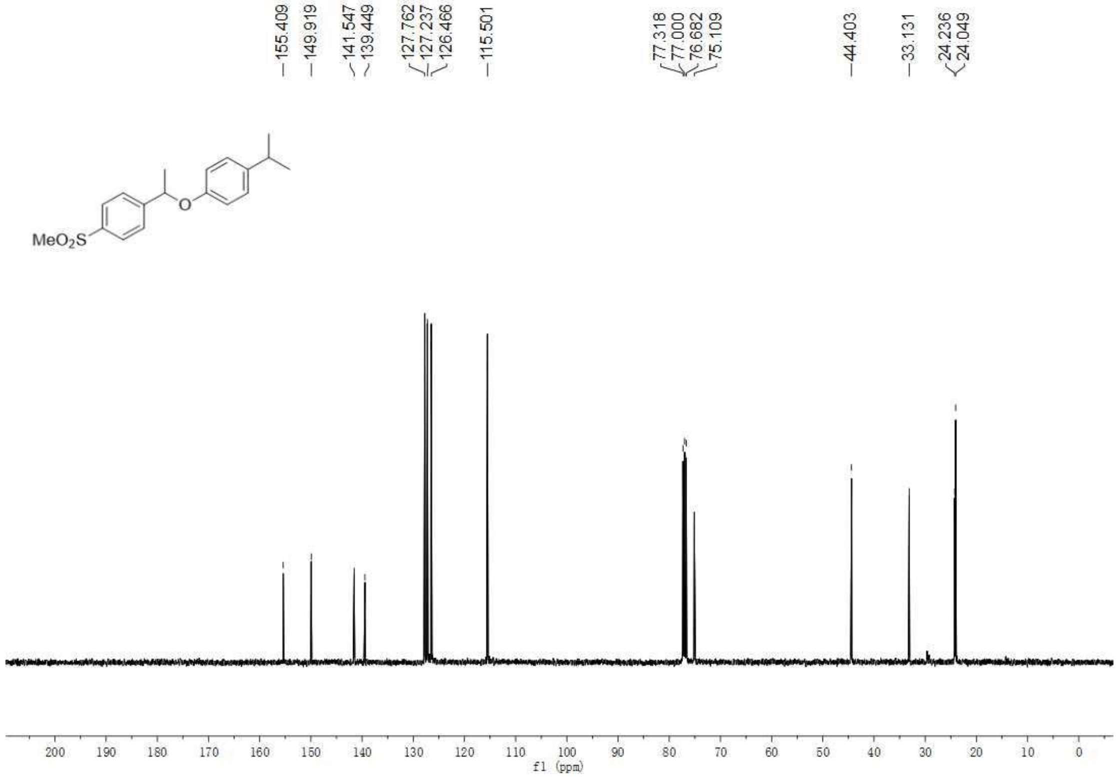

^{1}H NMR Spectrum of **129**

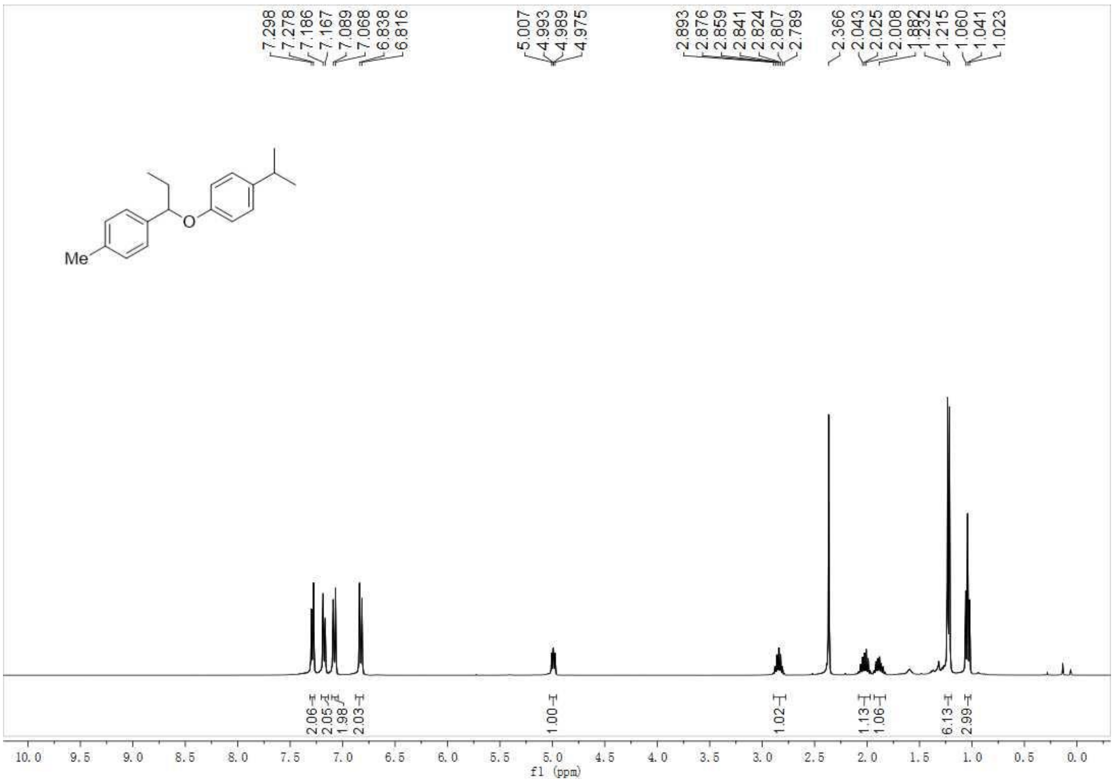

^{13}C NMR Spectrum of **129**

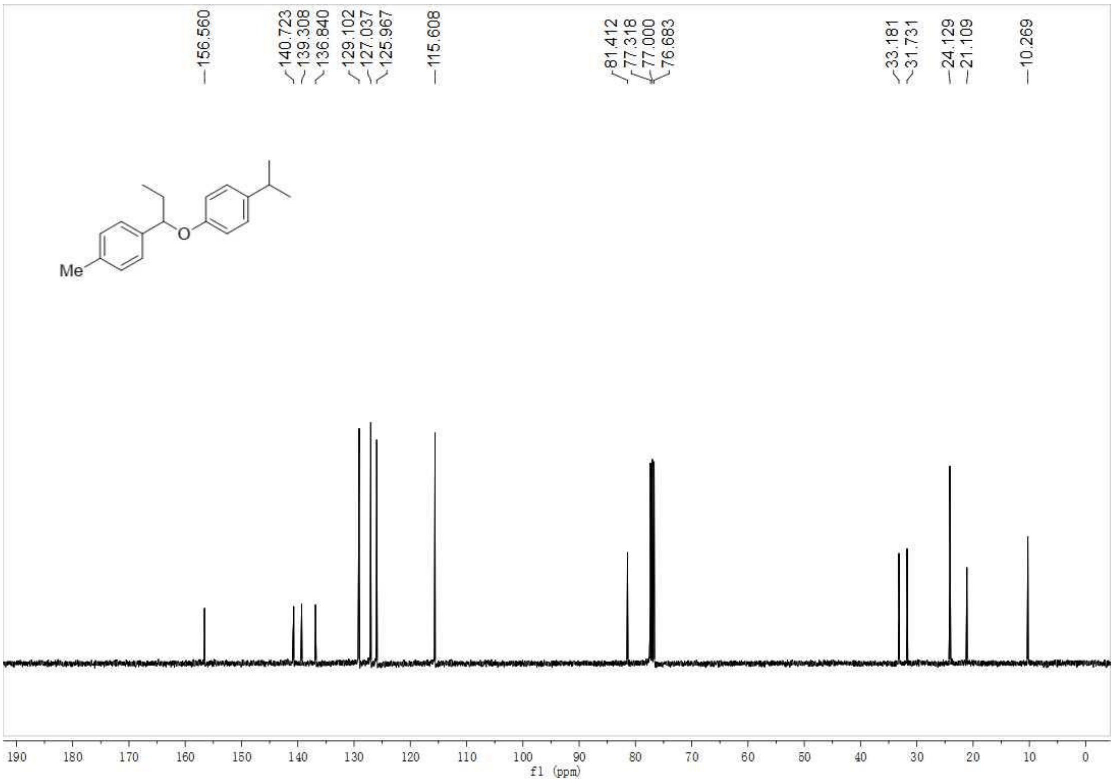

^{1}H NMR Spectrum of **130**

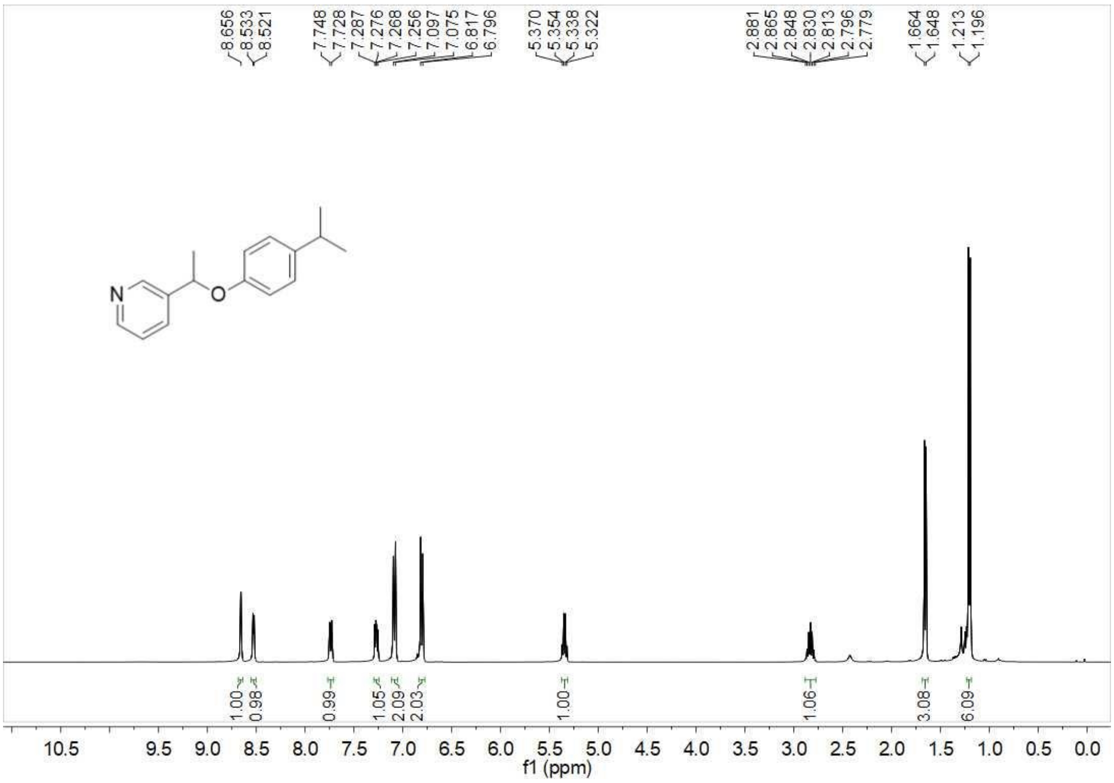

^{13}C NMR Spectrum of **130**

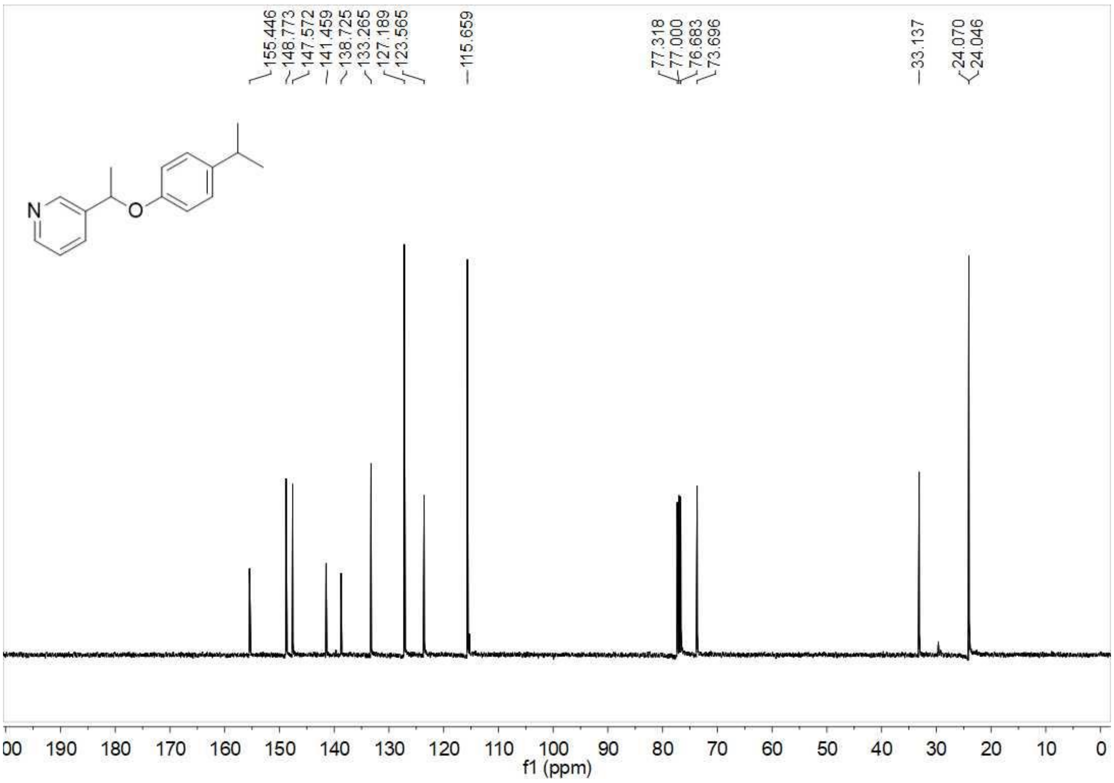

^{1}H NMR Spectrum of **131**

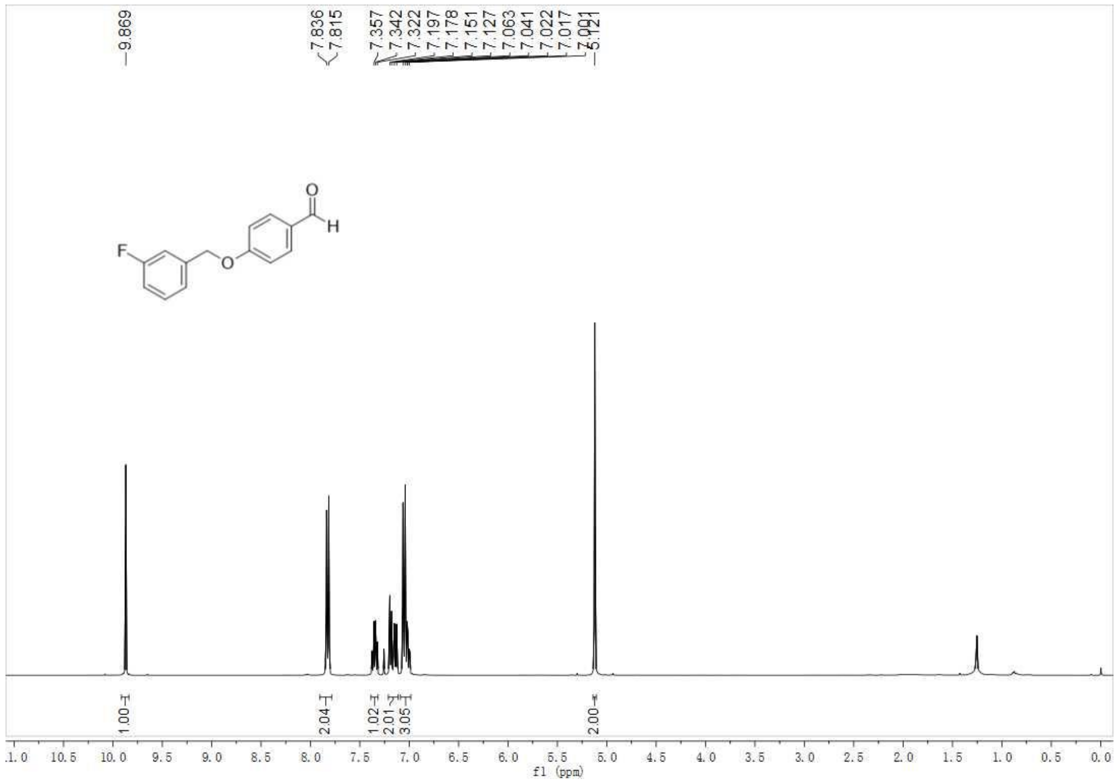

^{13}C NMR Spectrum of **131**

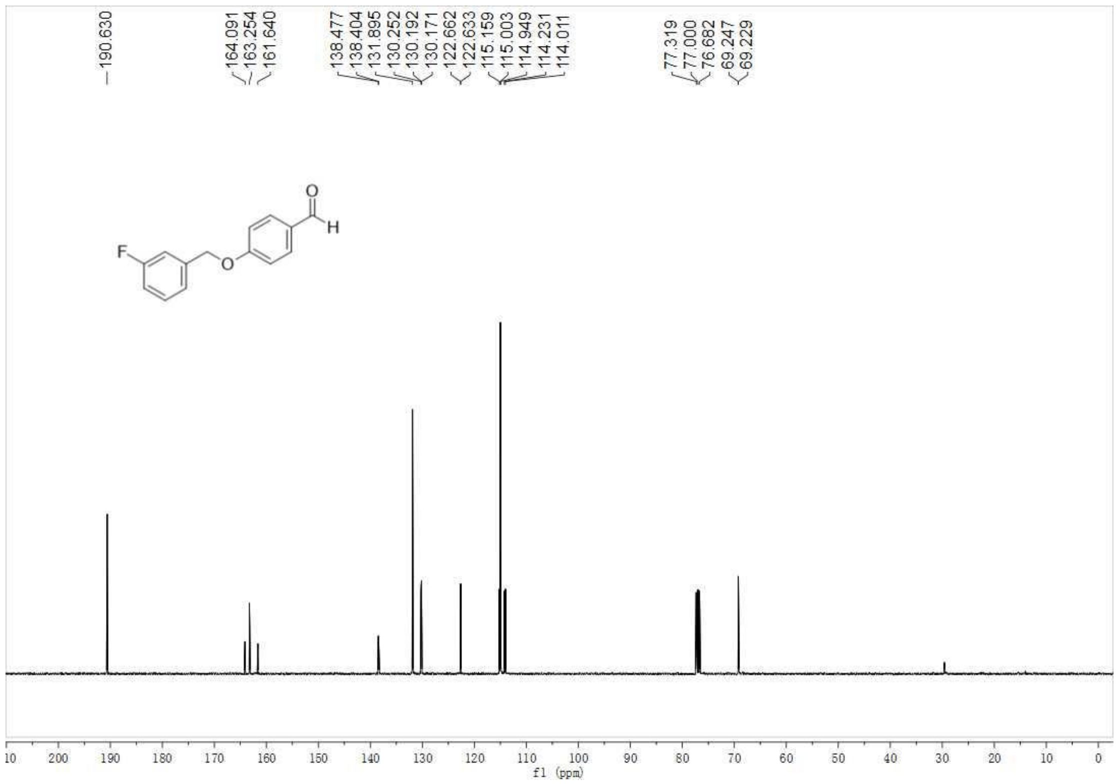

^{19}F NMR Spectrum of **131**

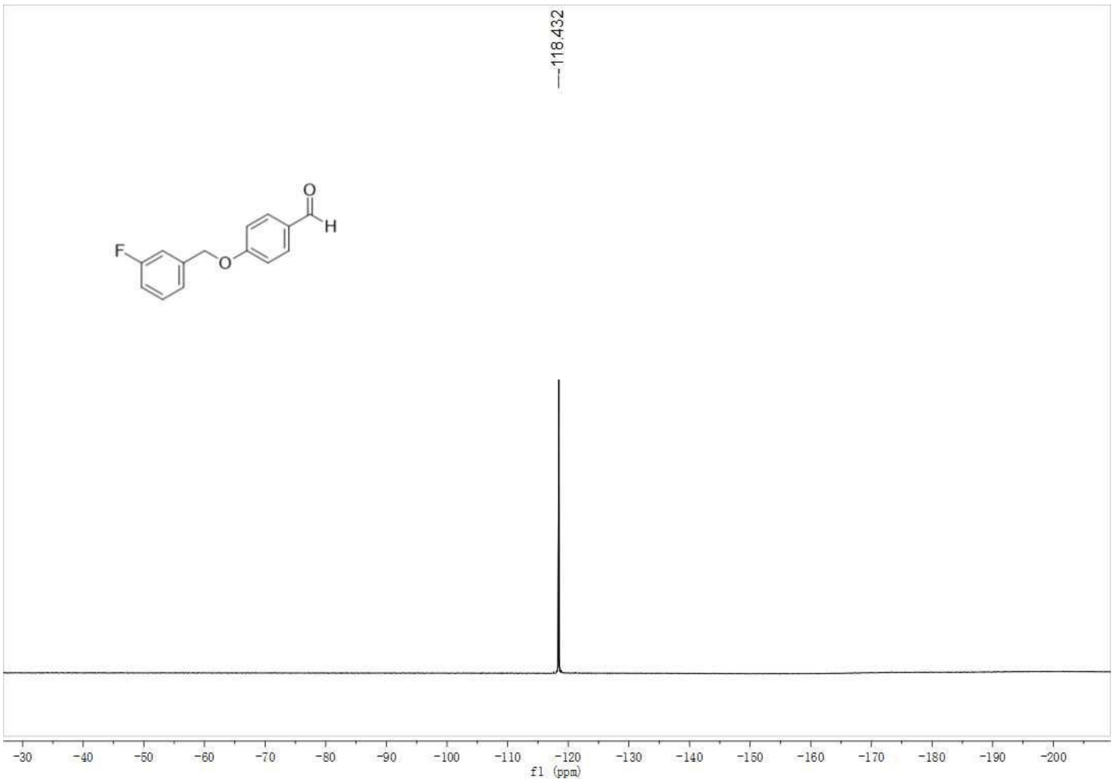

^{1}H NMR Spectrum of **132**

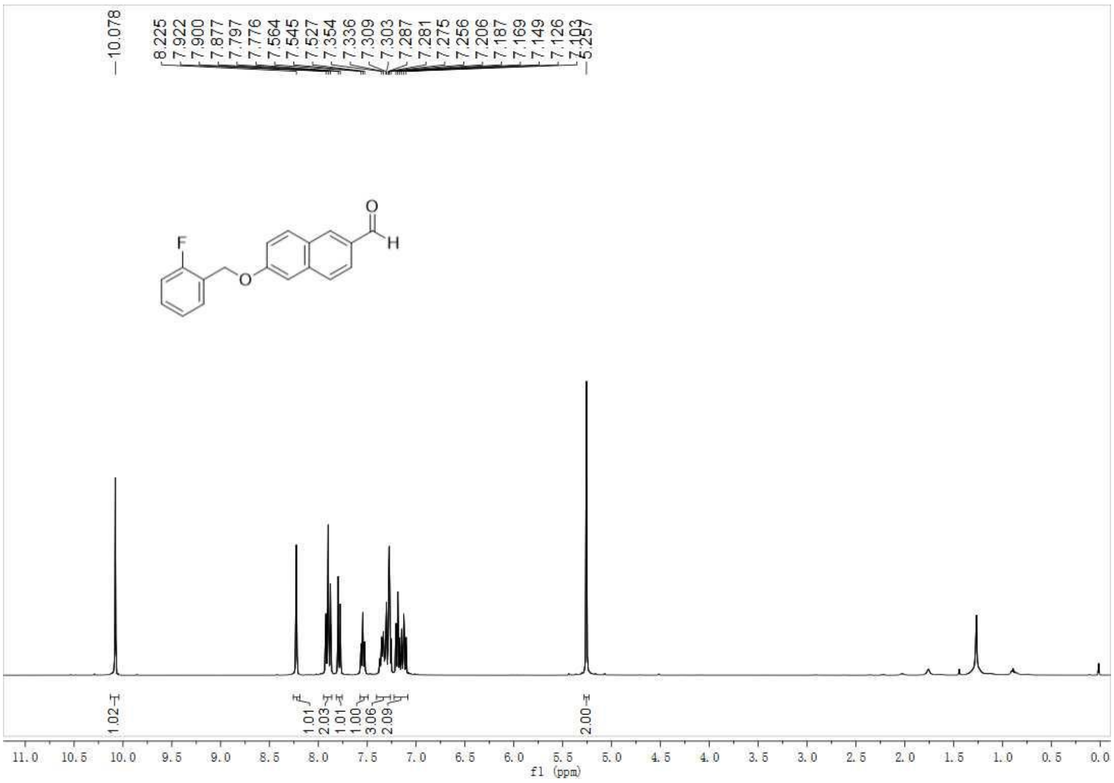

^{13}C NMR Spectrum of **132**

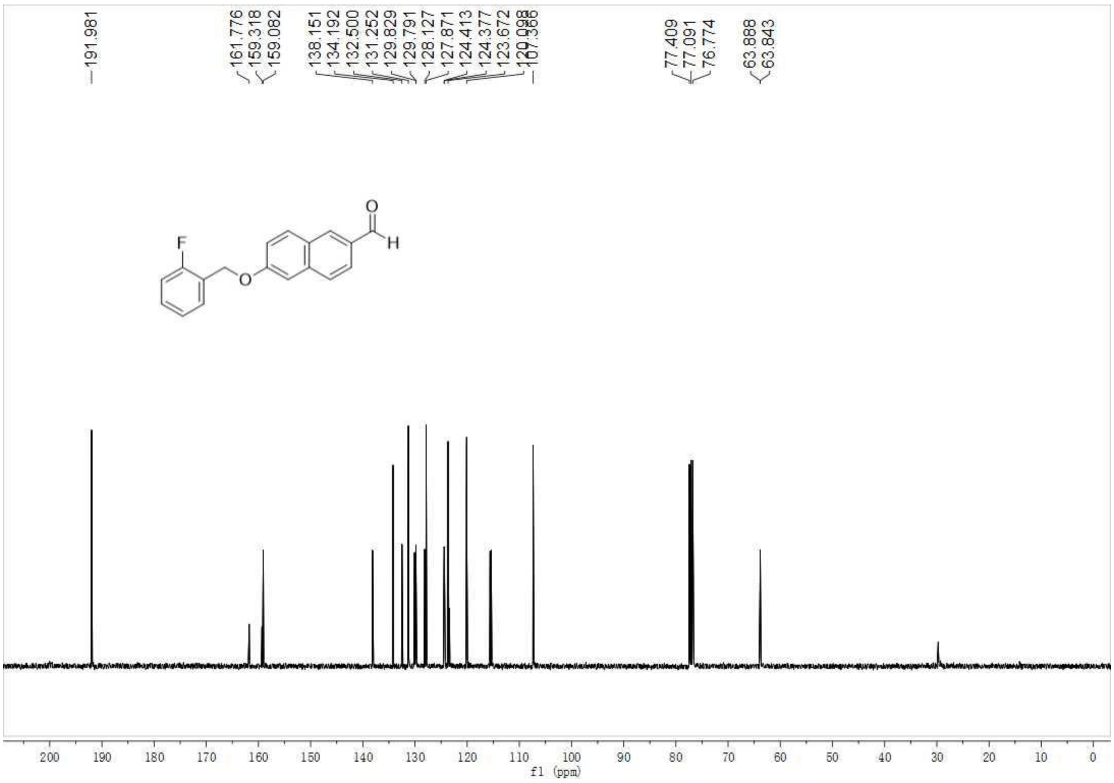

^{19}F NMR Spectrum of **132**

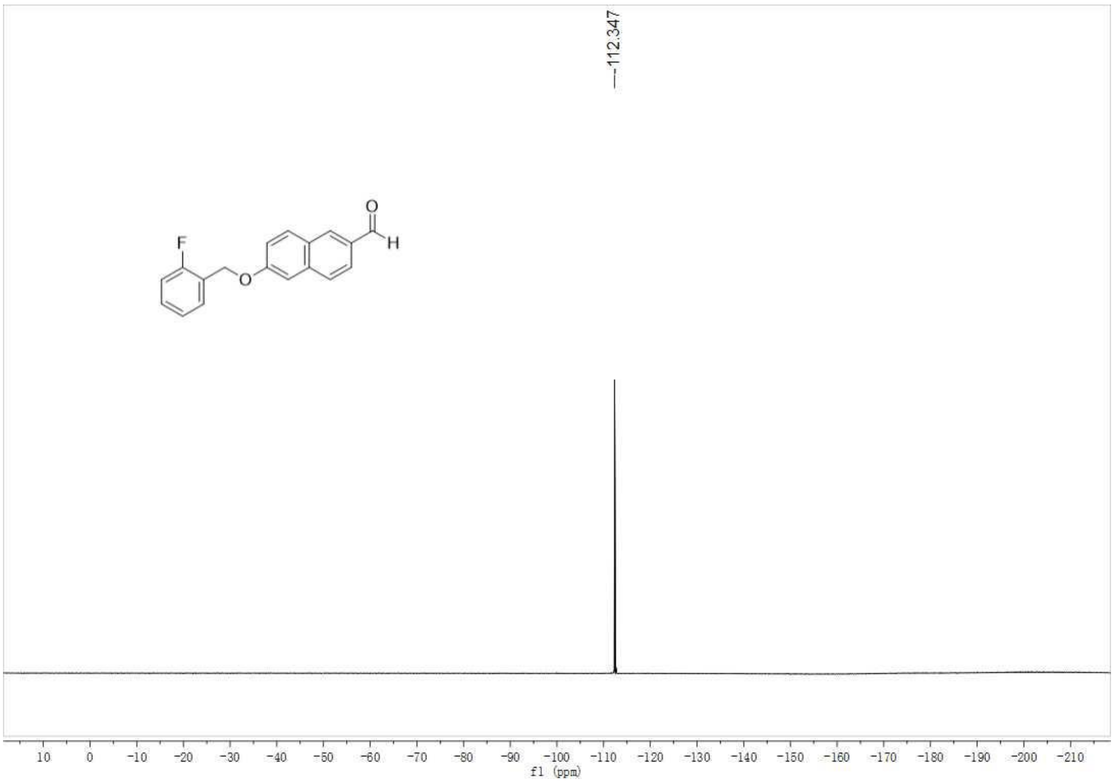

^{1}H NMR Spectrum of **133**

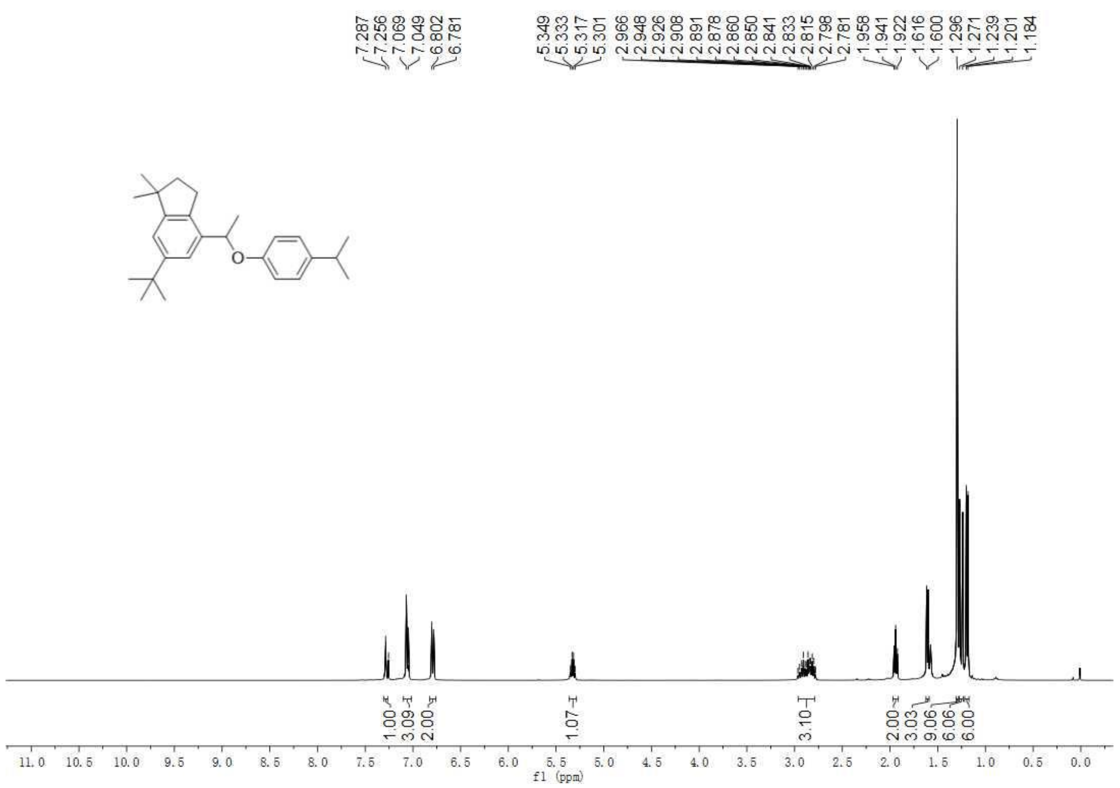

^{13}C NMR Spectrum of **133**

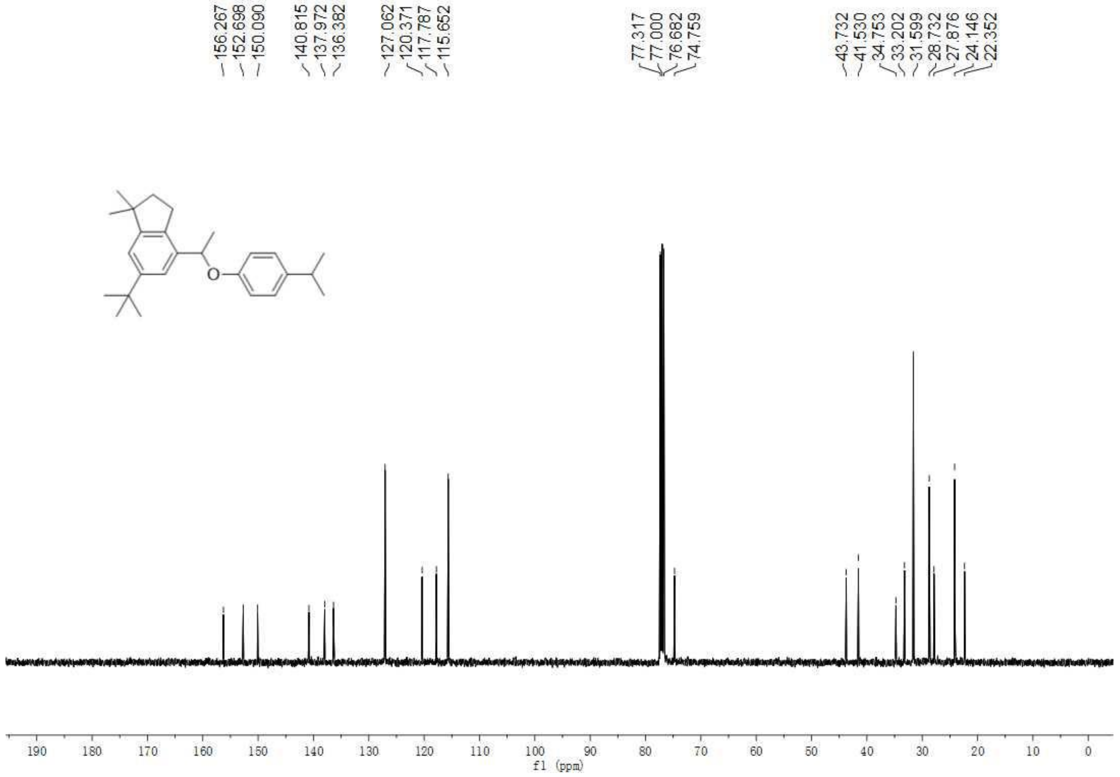

^{1}H NMR Spectrum of **134**

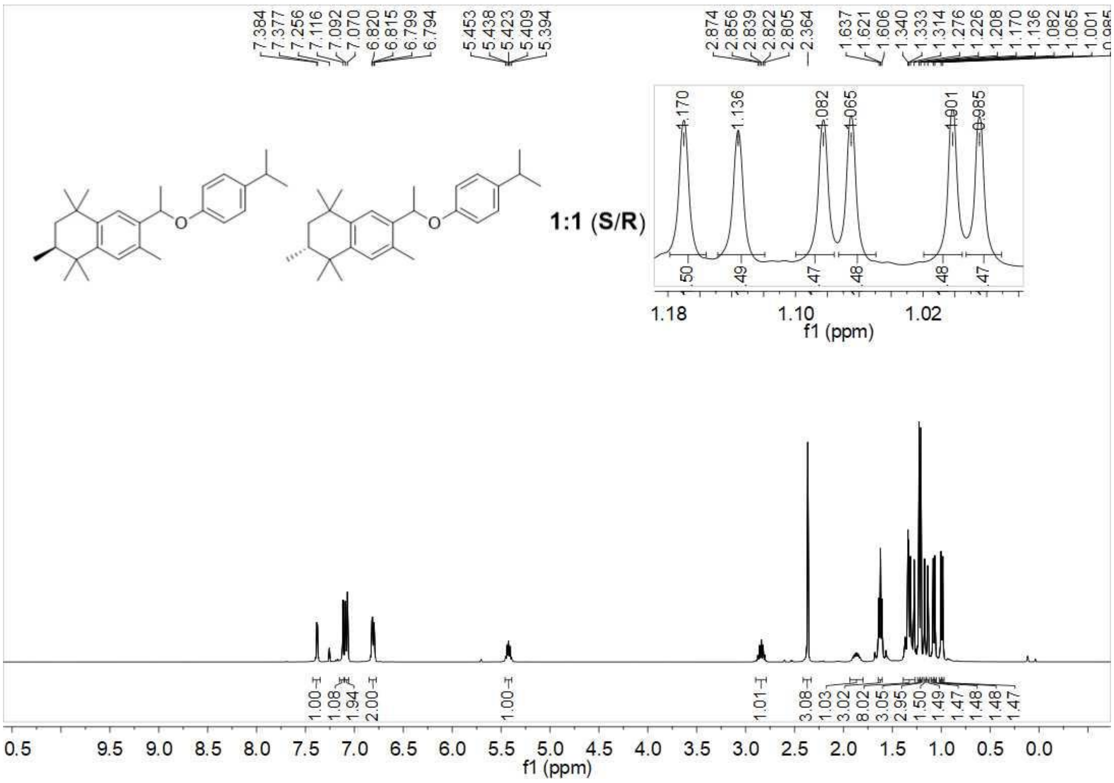

^{13}C NMR Spectrum of **134**

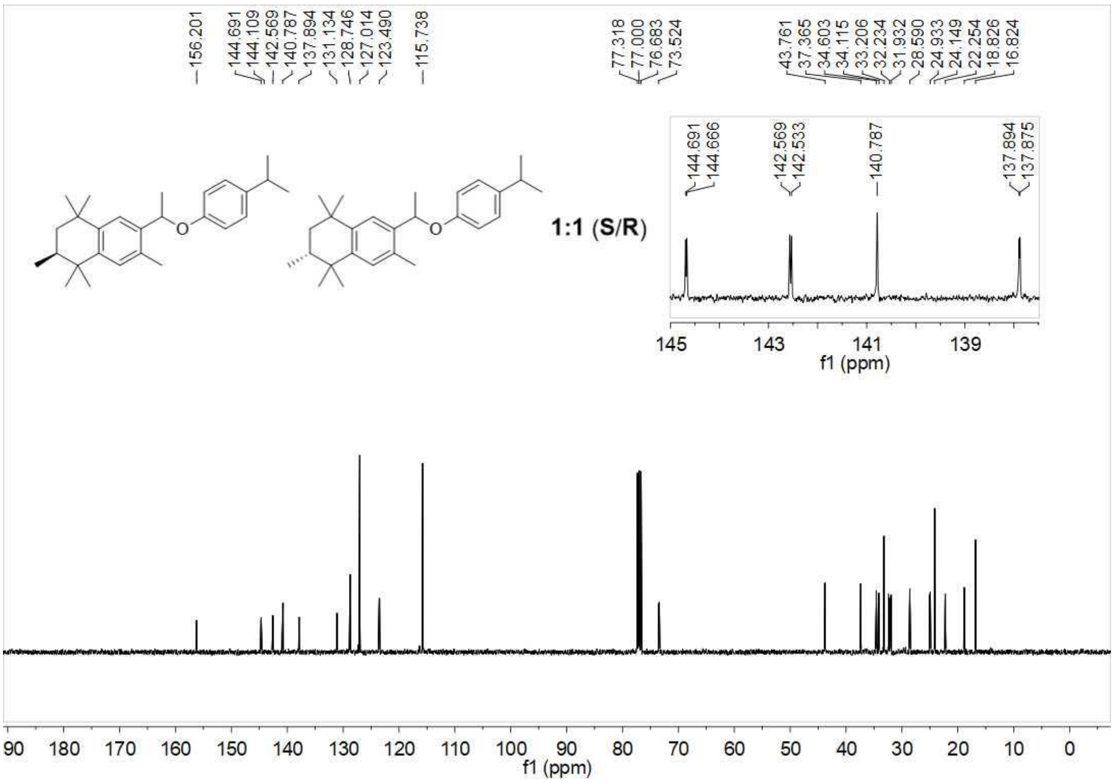

^{1}H NMR Spectrum of **135**

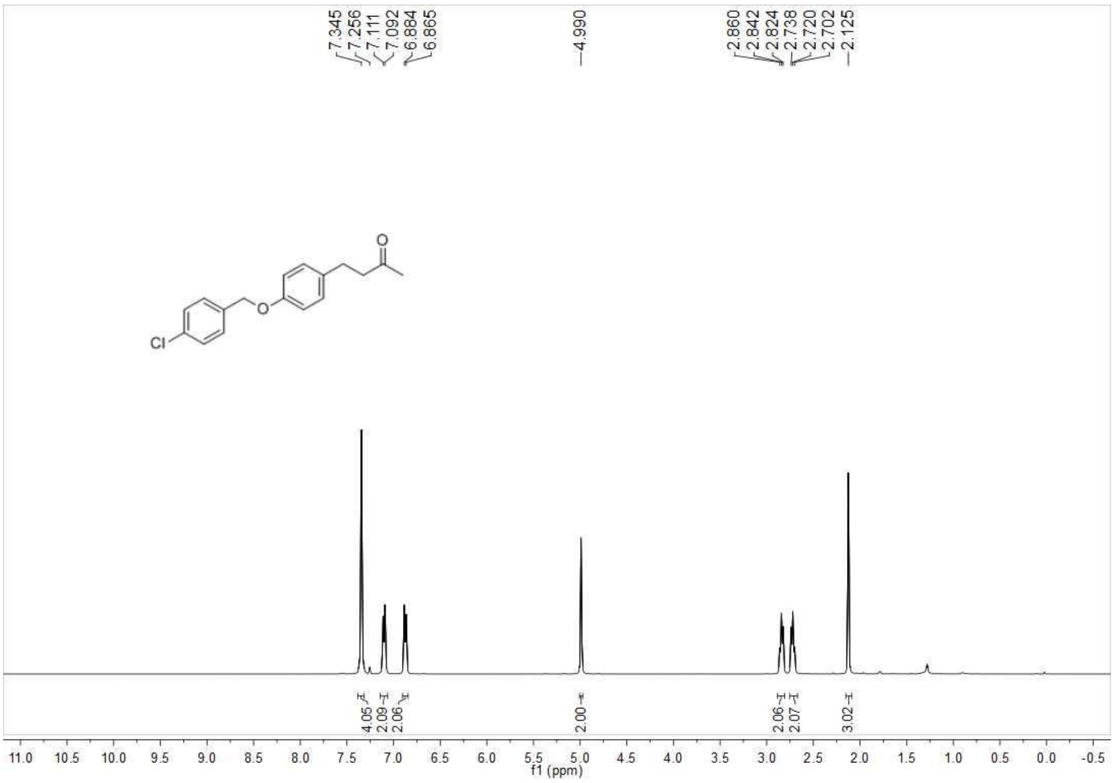

^{13}C NMR Spectrum of **135**

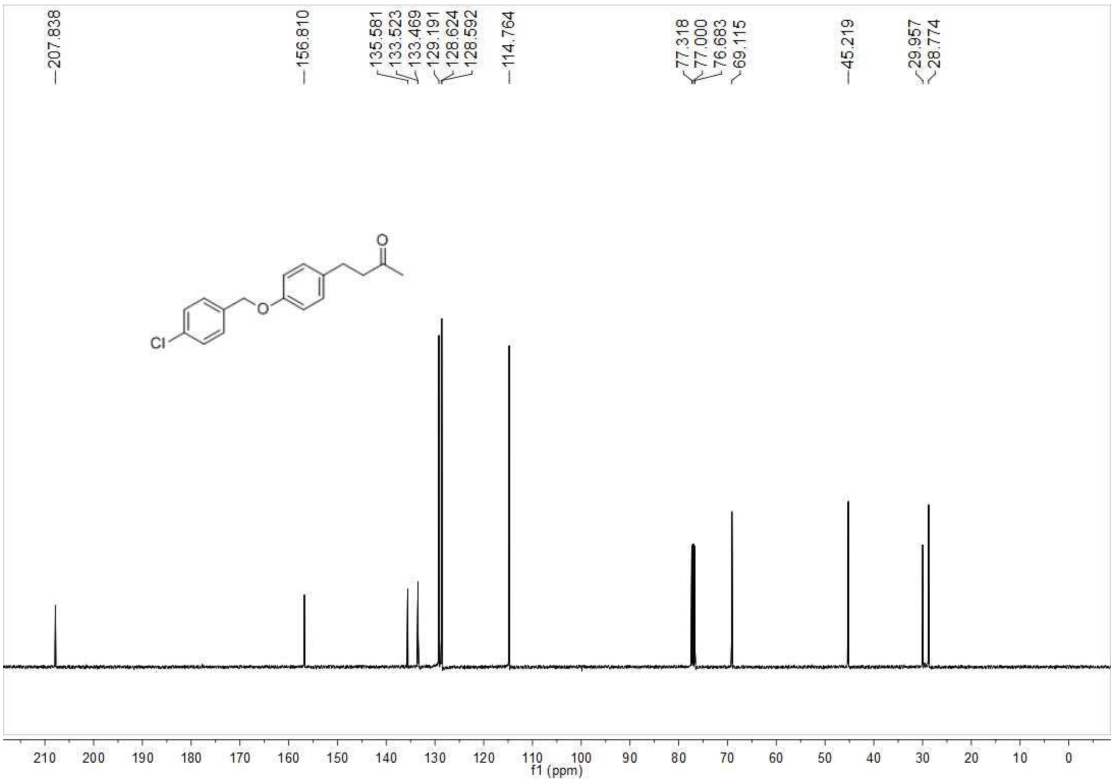

^{1}H NMR Spectrum of **136**

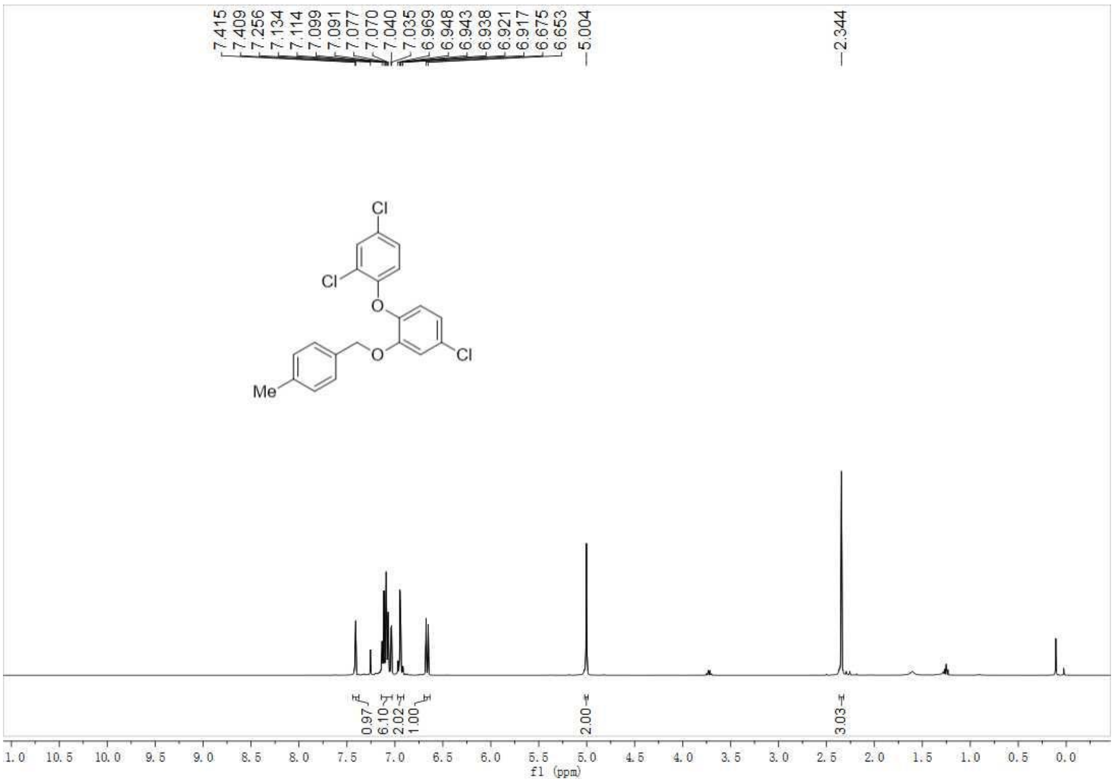

^{13}C NMR Spectrum of **136**

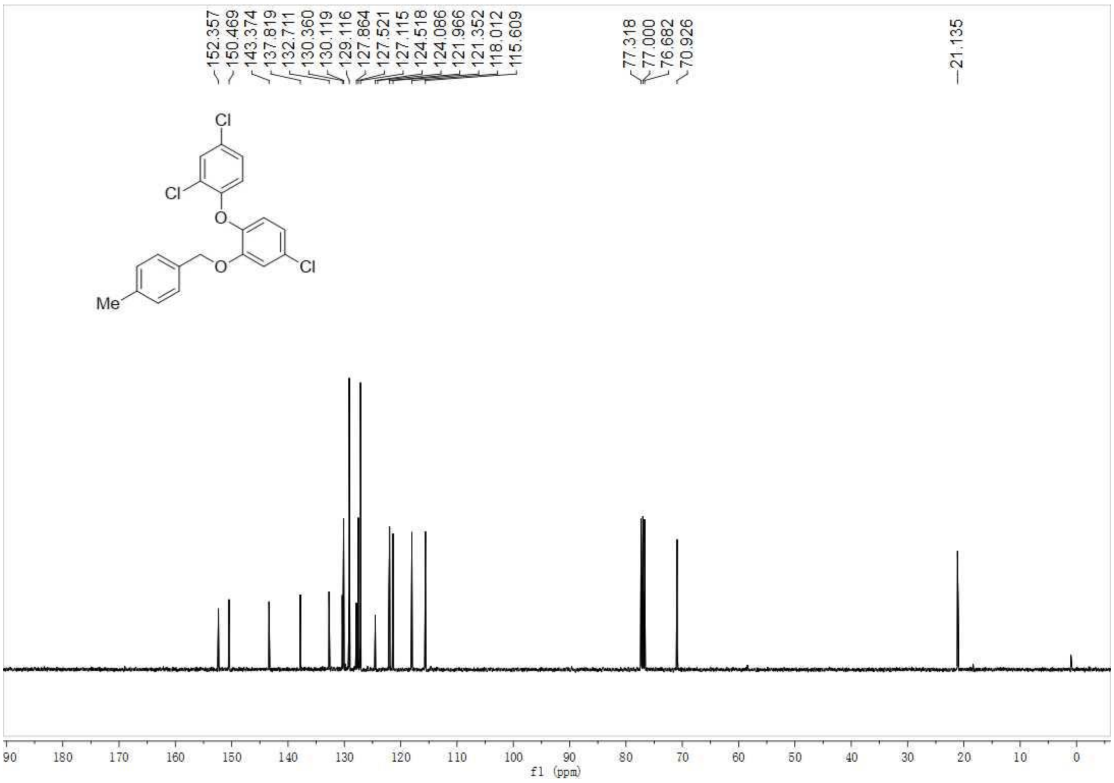

^{1}H NMR Spectrum of **137**

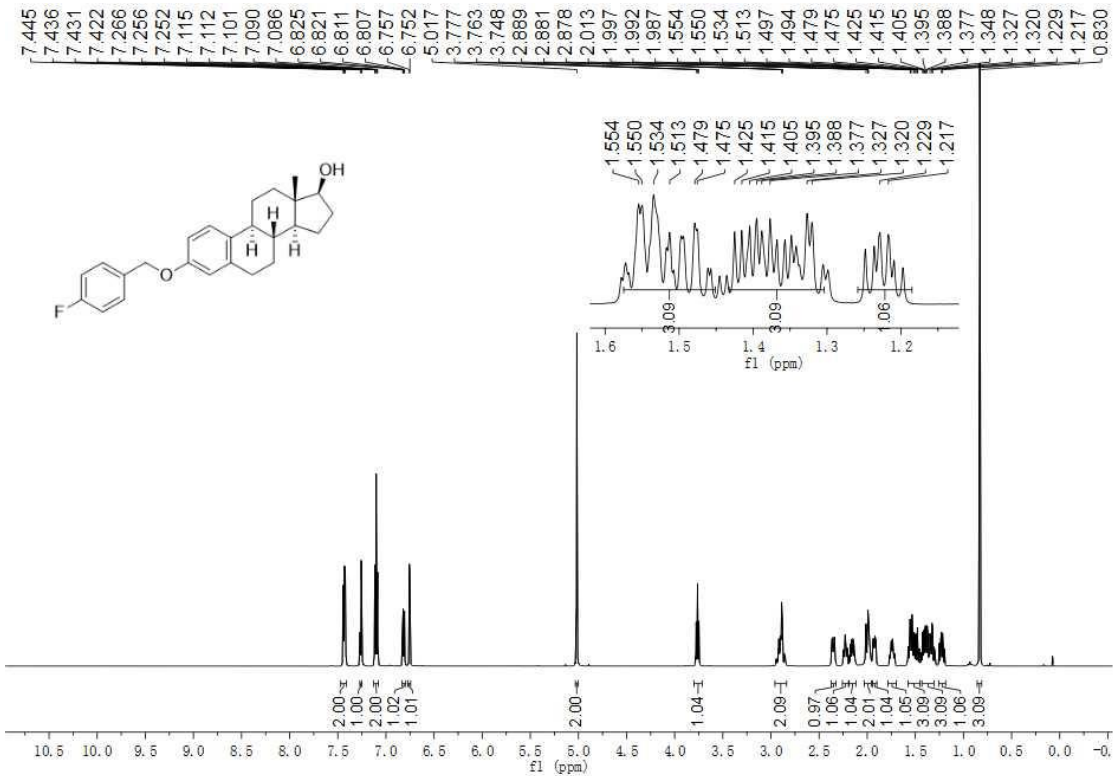

^{13}C NMR Spectrum of **137**

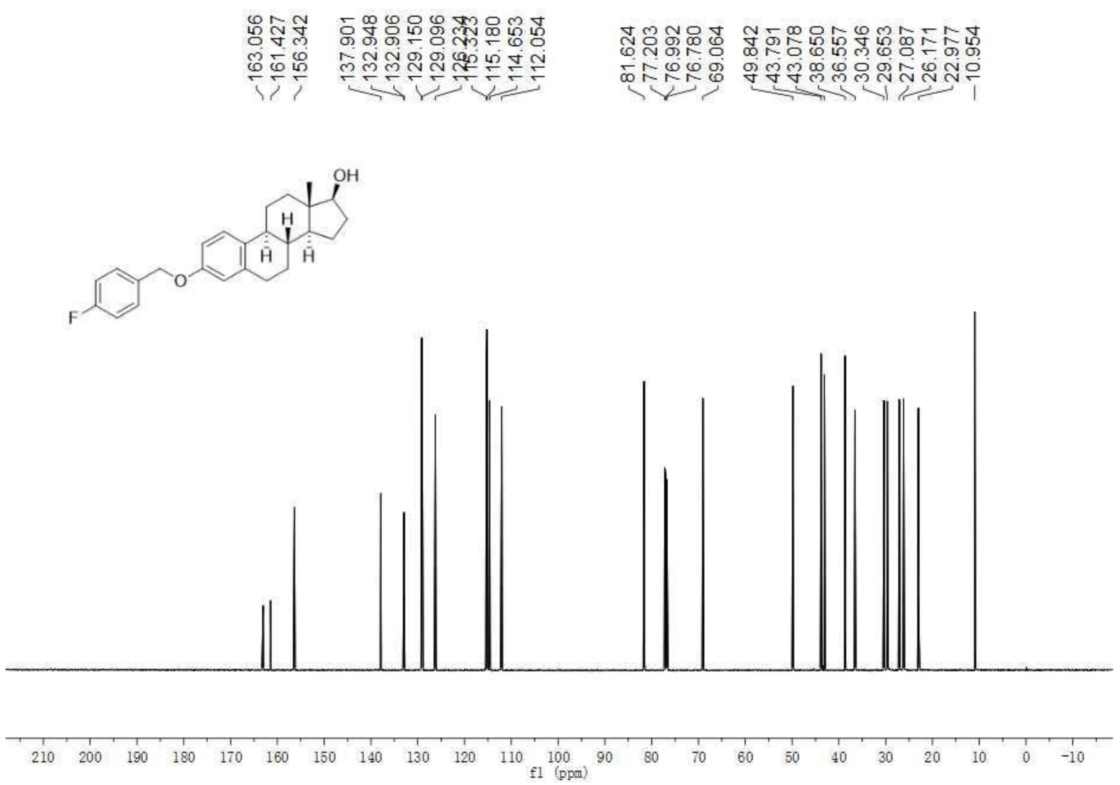

^{19}F NMR Spectrum of **137**

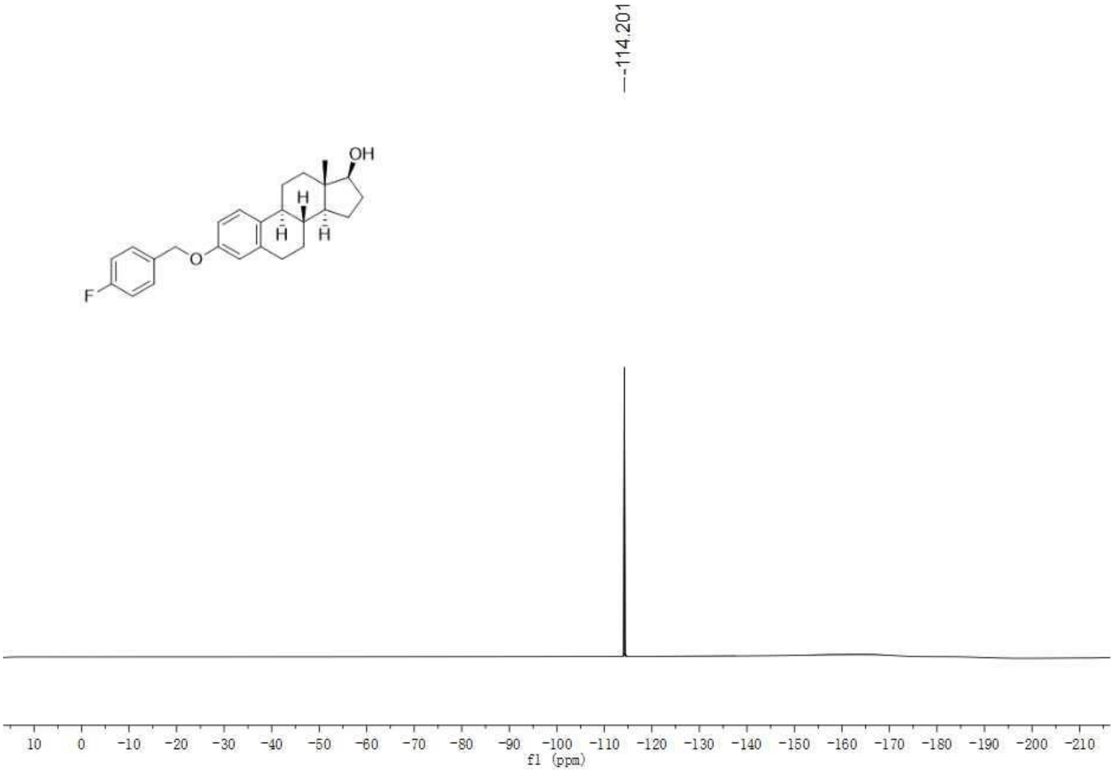

^{1}H NMR Spectrum of **138**

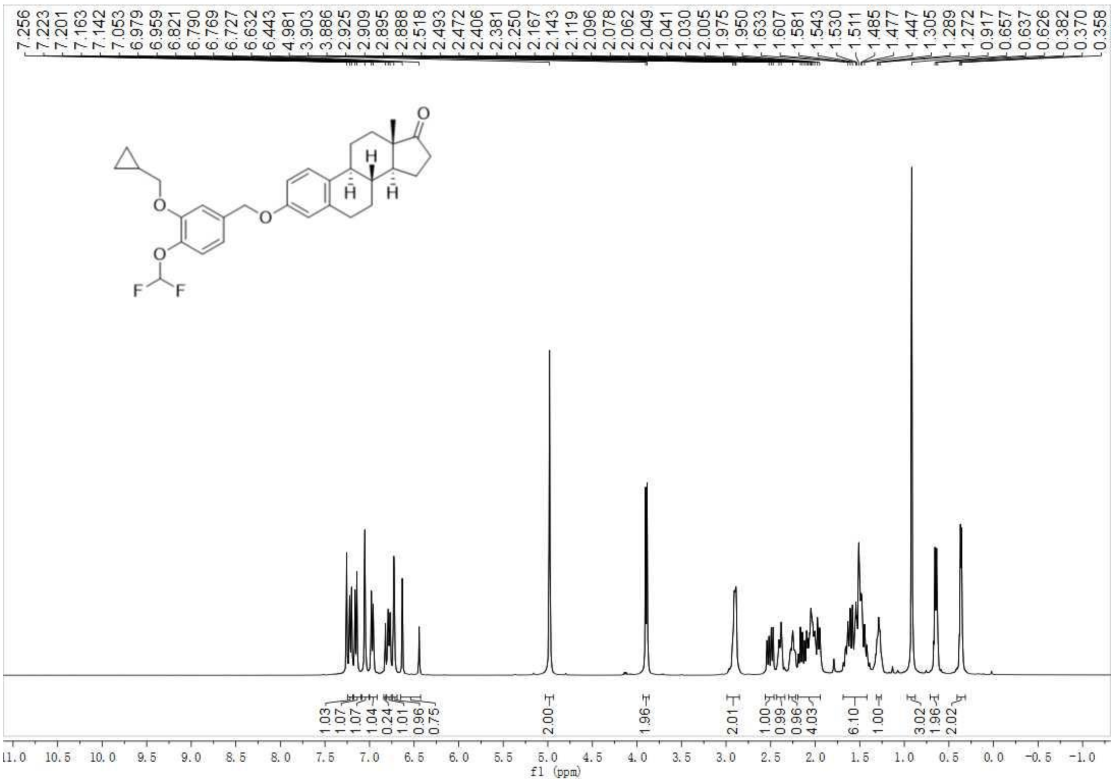

^{13}C NMR Spectrum of **138**

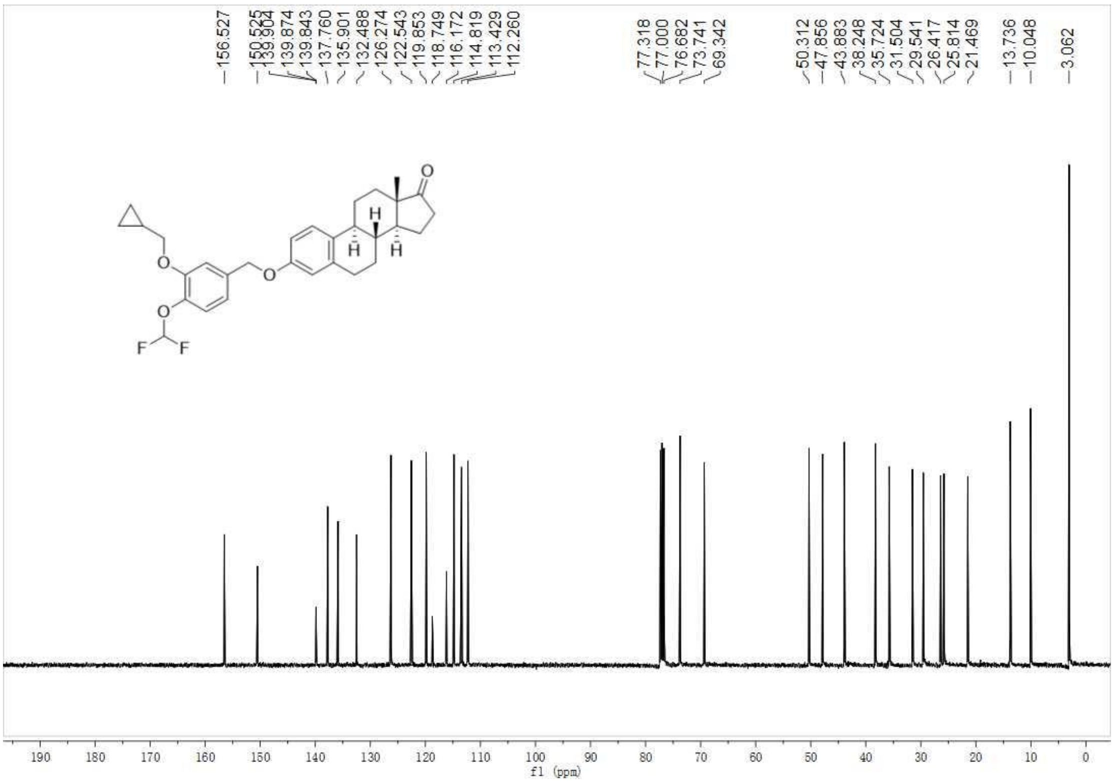

^{19}F NMR Spectrum of **138**

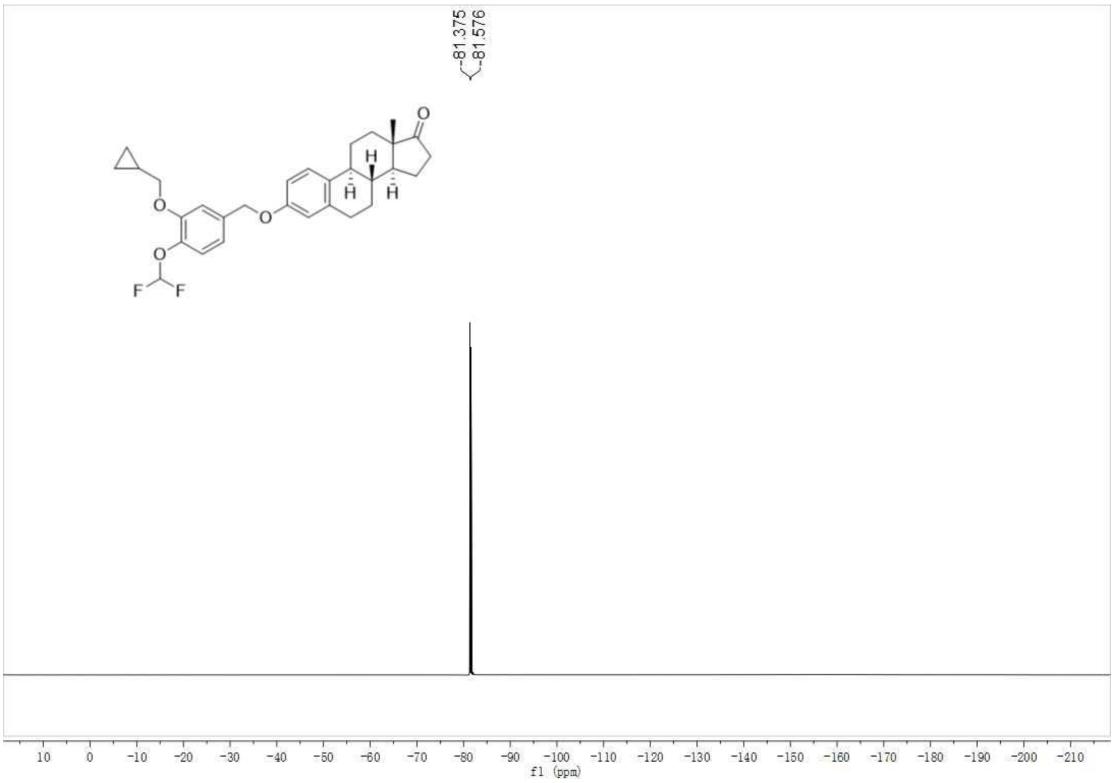

^{1}H NMR Spectrum of **139**

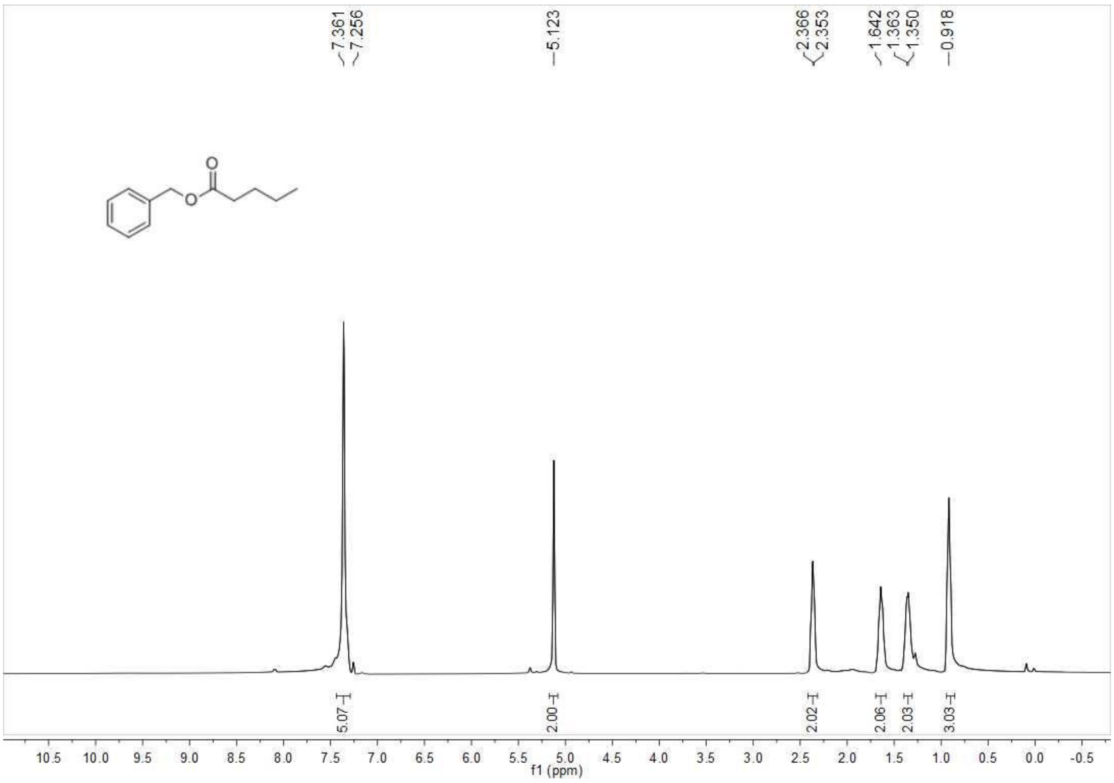

^{13}C NMR Spectrum of **139**

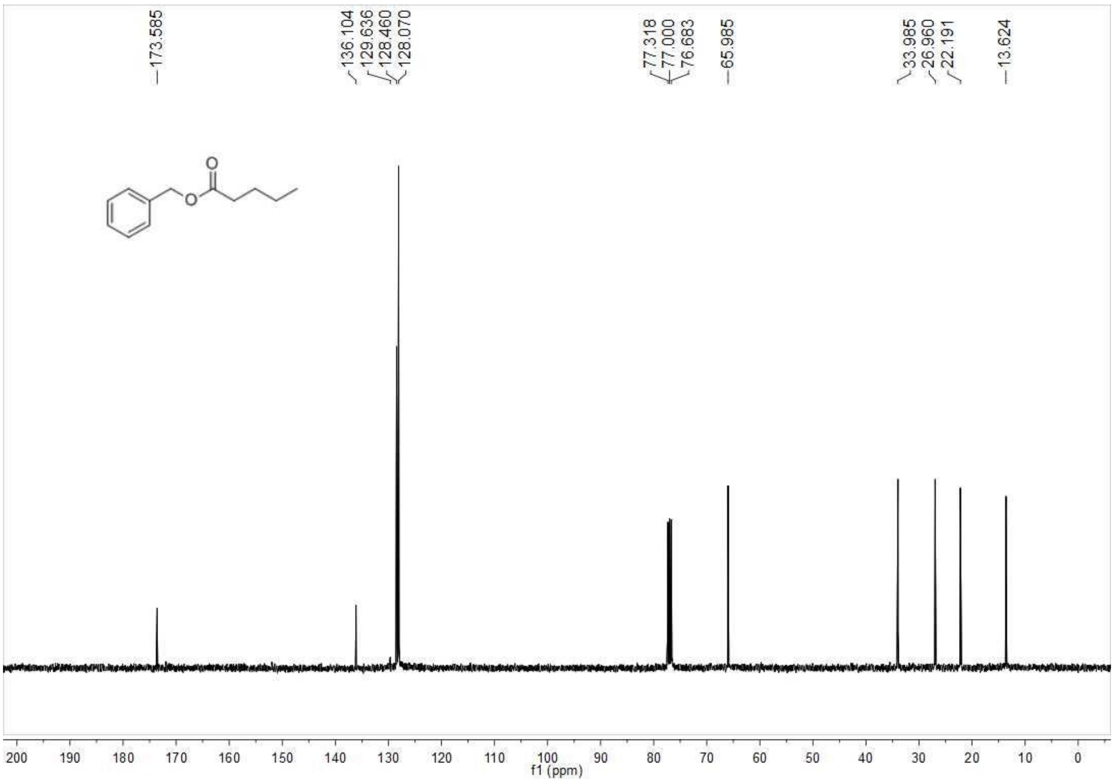

^{1}H NMR Spectrum of **140**

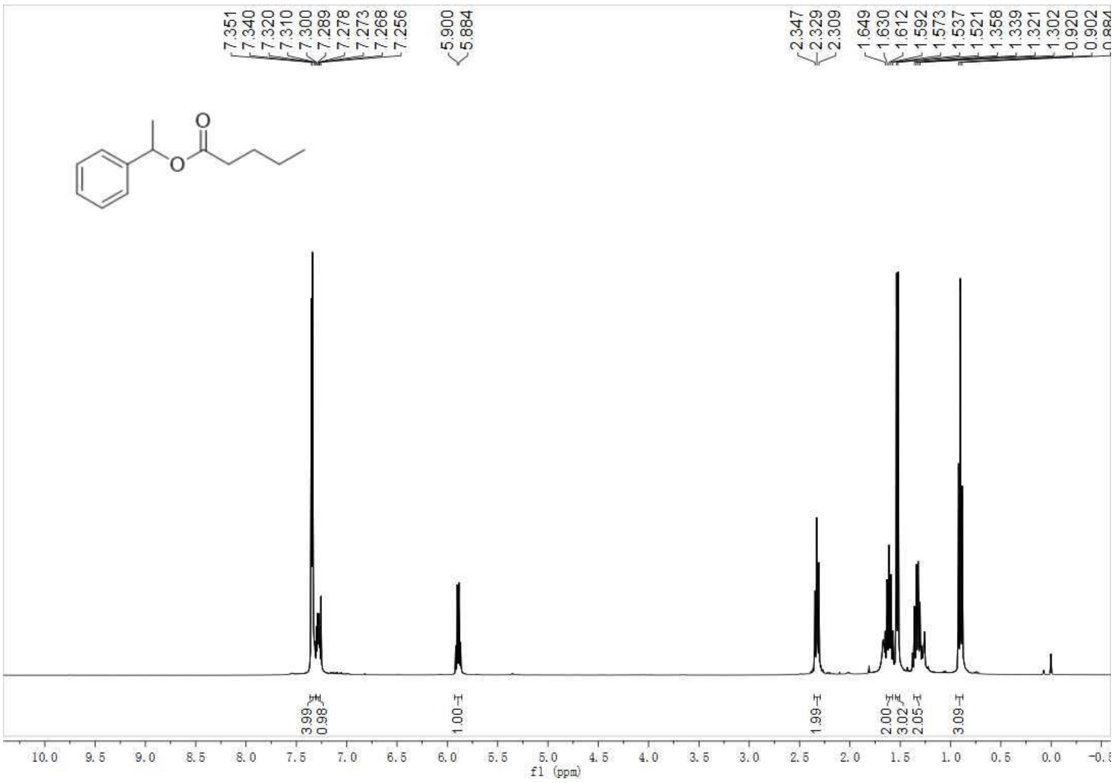

^{13}C NMR Spectrum of **140**

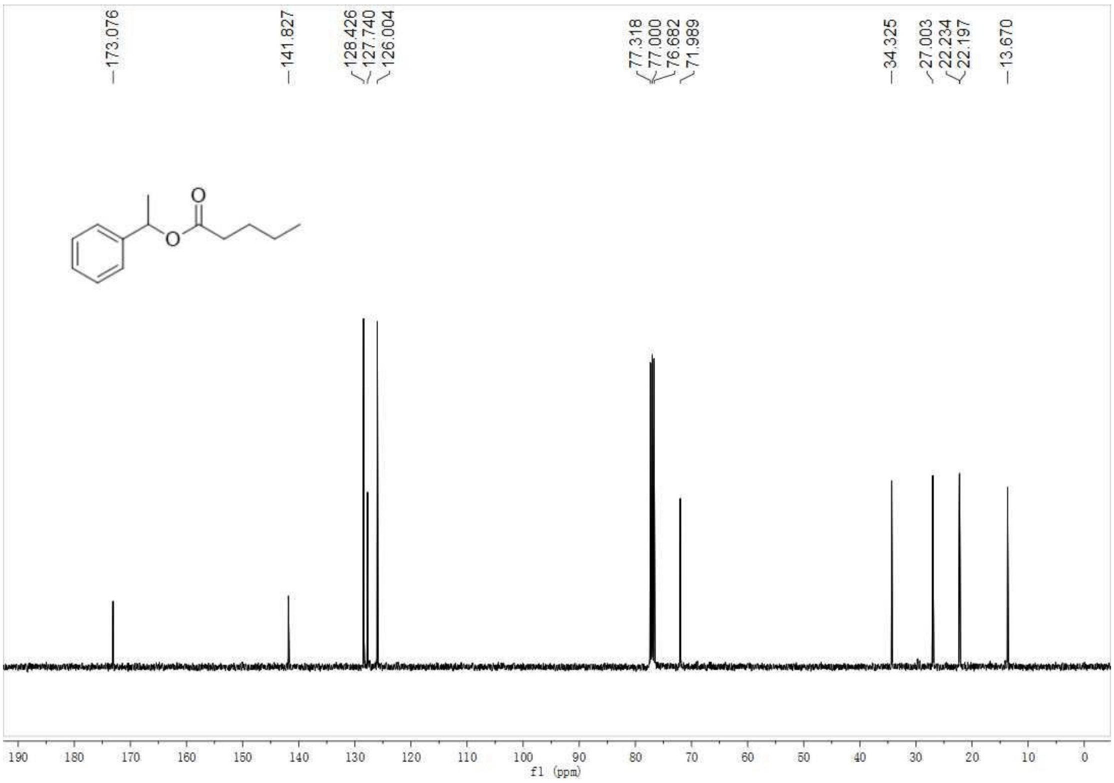

^{1}H NMR Spectrum of **141**

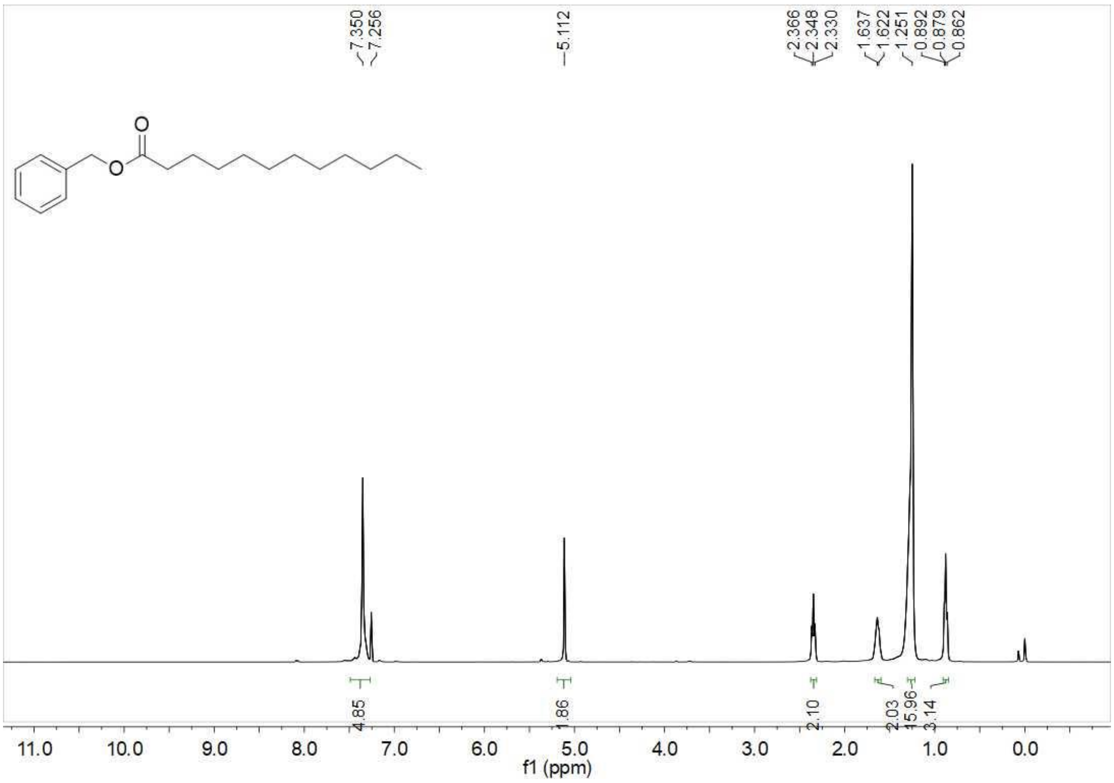

^{13}C NMR Spectrum of **141**

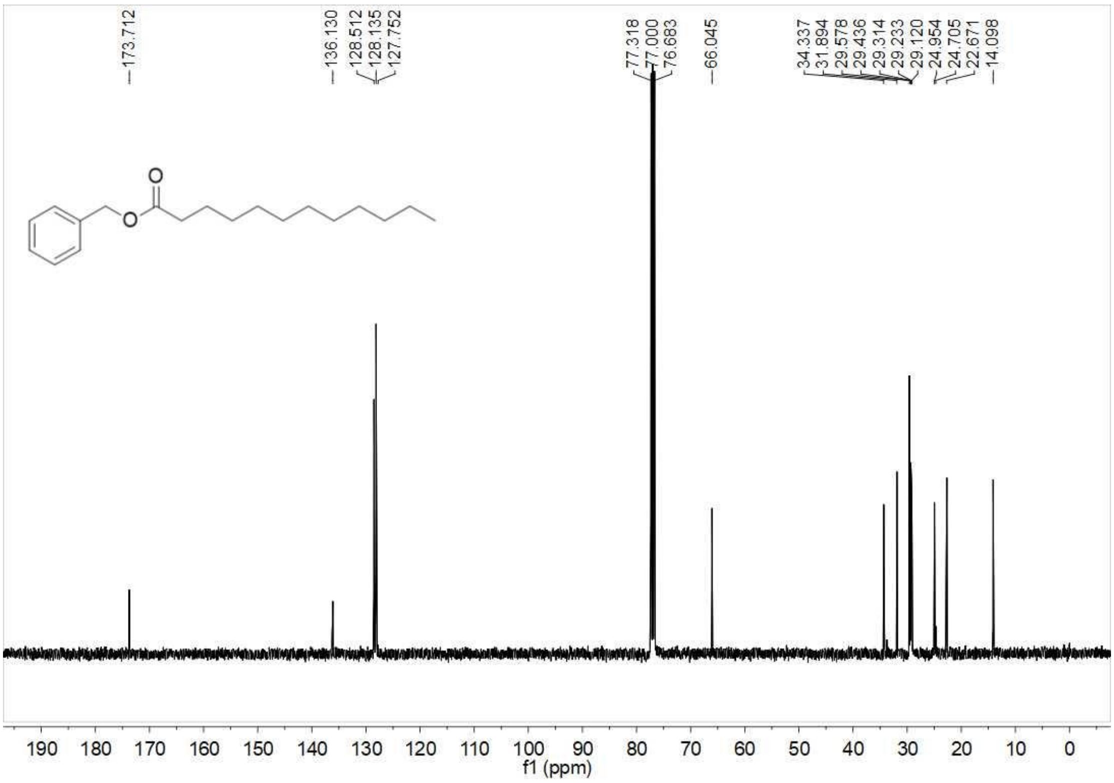

^{1}H NMR Spectrum of **142**

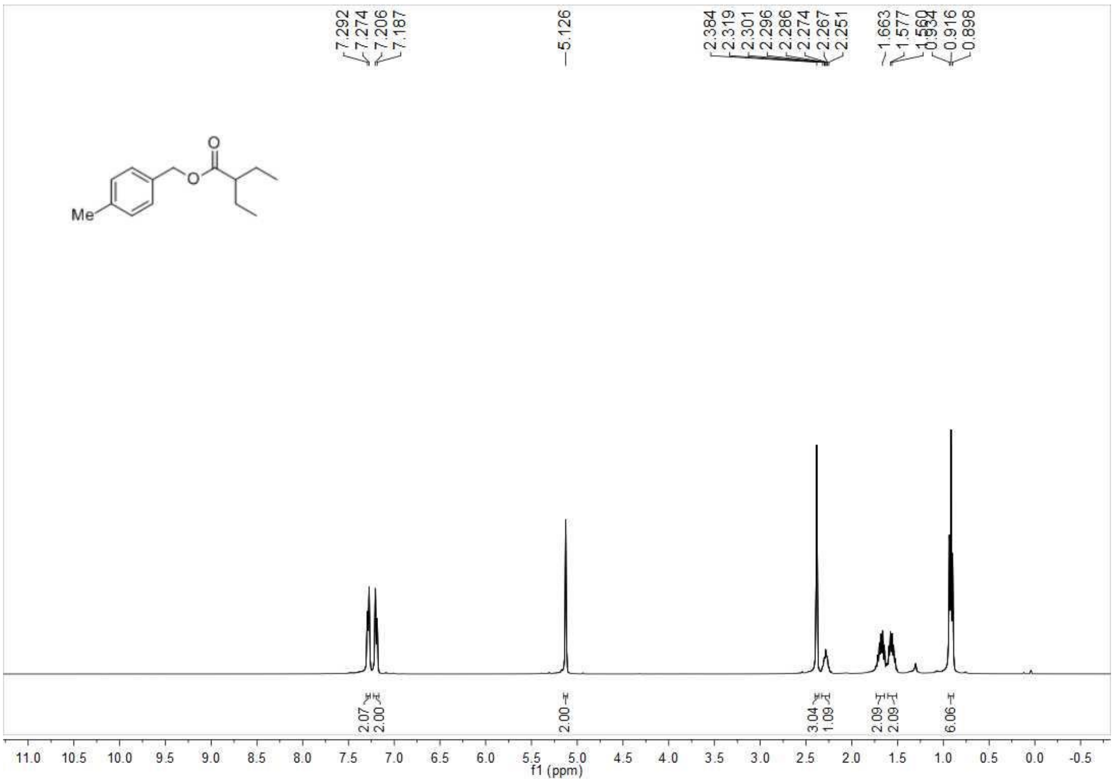

^{13}C NMR Spectrum of **142**

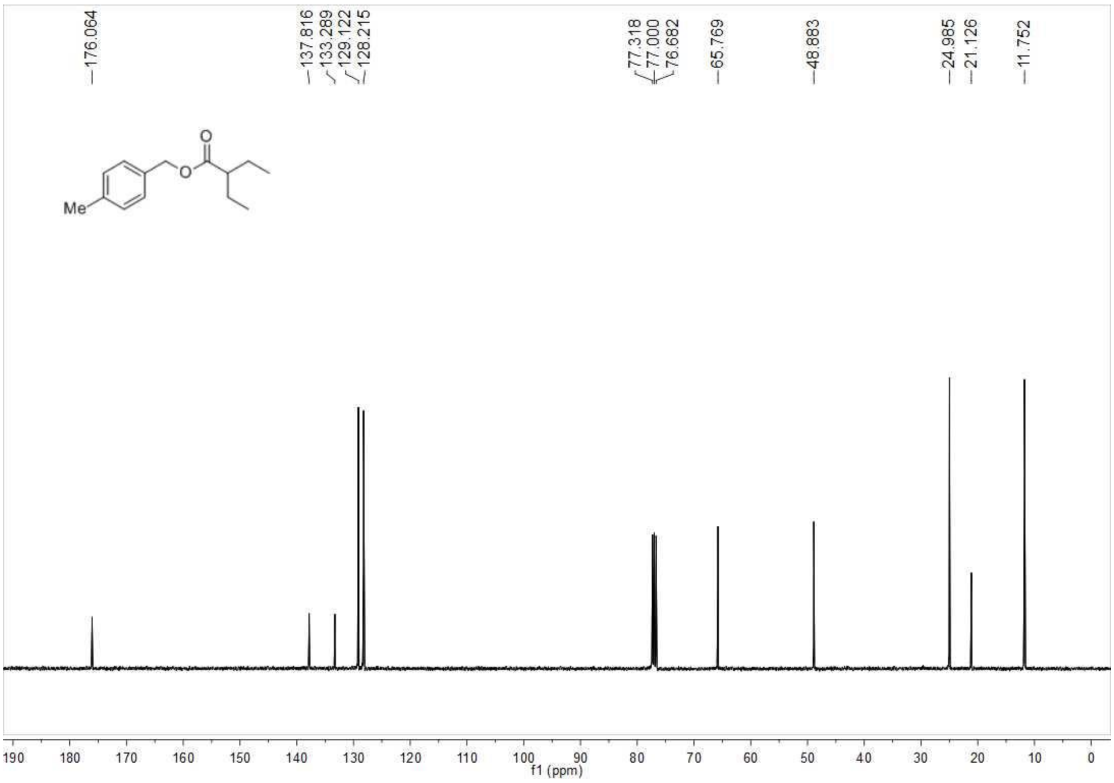

^{1}H NMR Spectrum of **143**

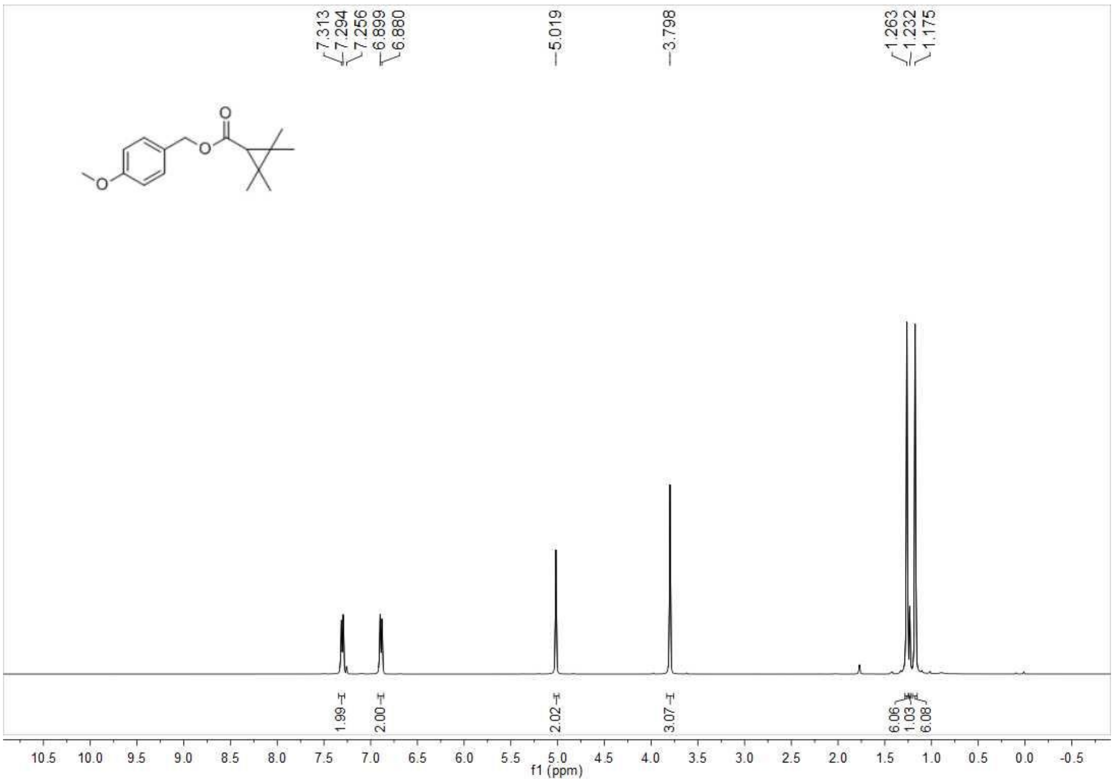

^{13}C NMR Spectrum of **143**

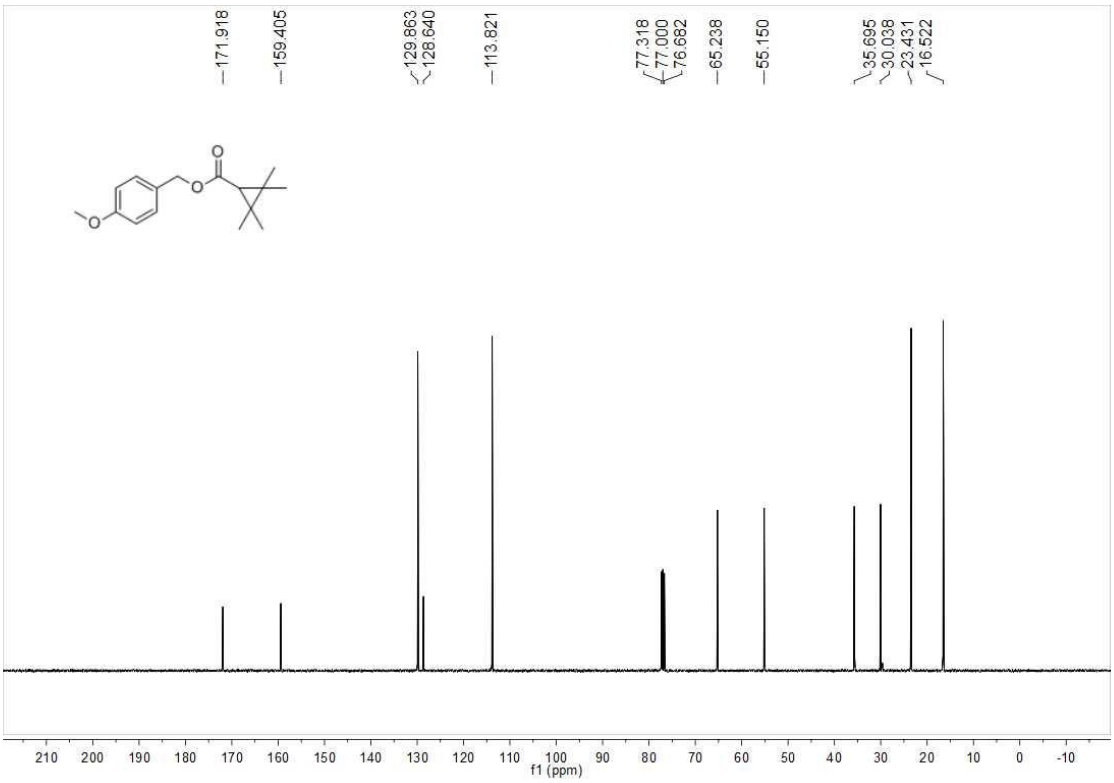

^{1}H NMR Spectrum of **144**

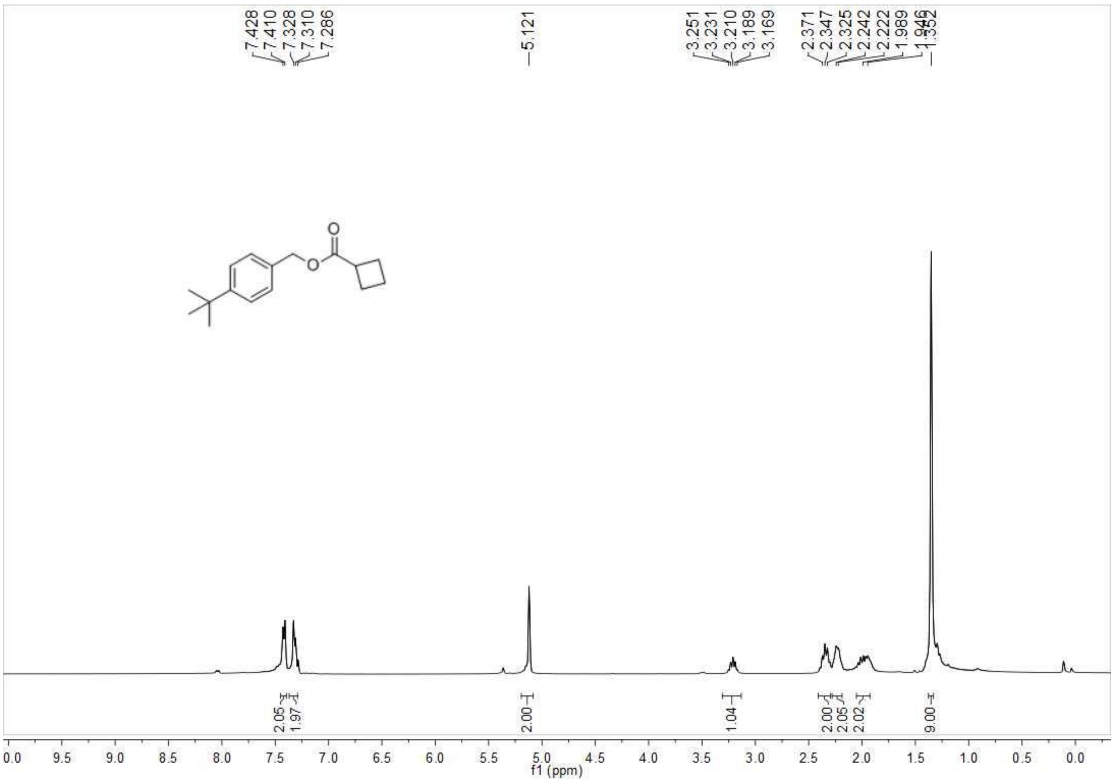

^{13}C NMR Spectrum of **144**

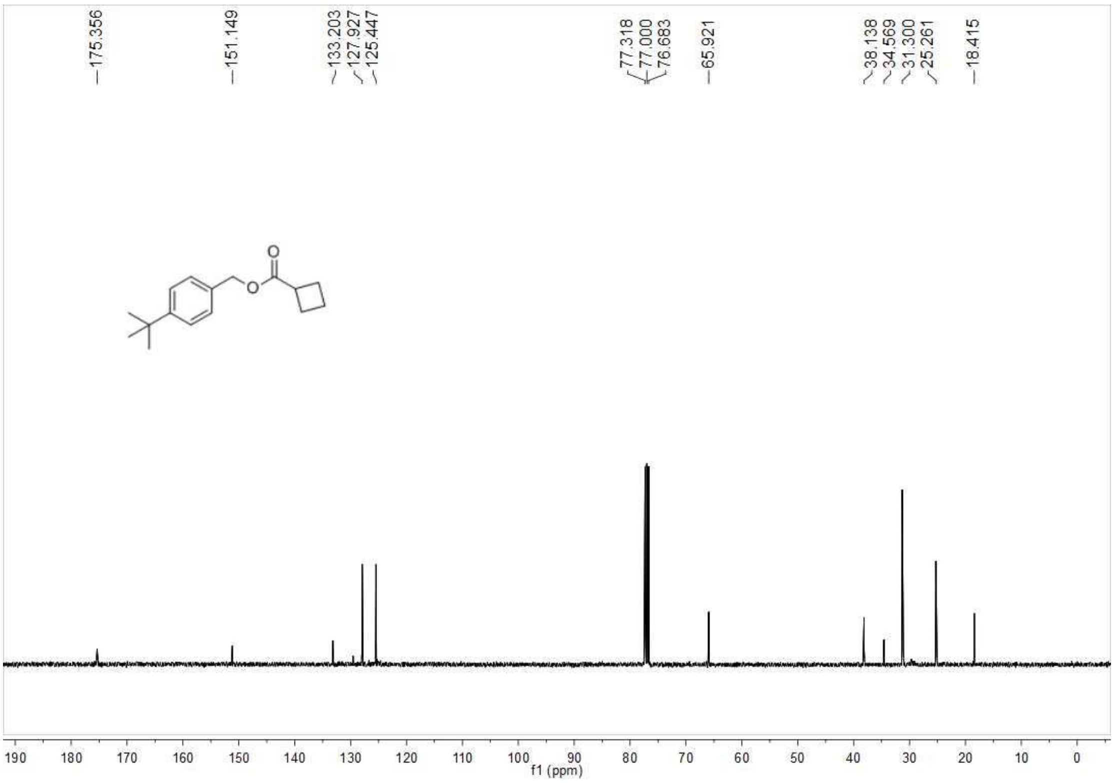

^{1}H NMR Spectrum of **145**

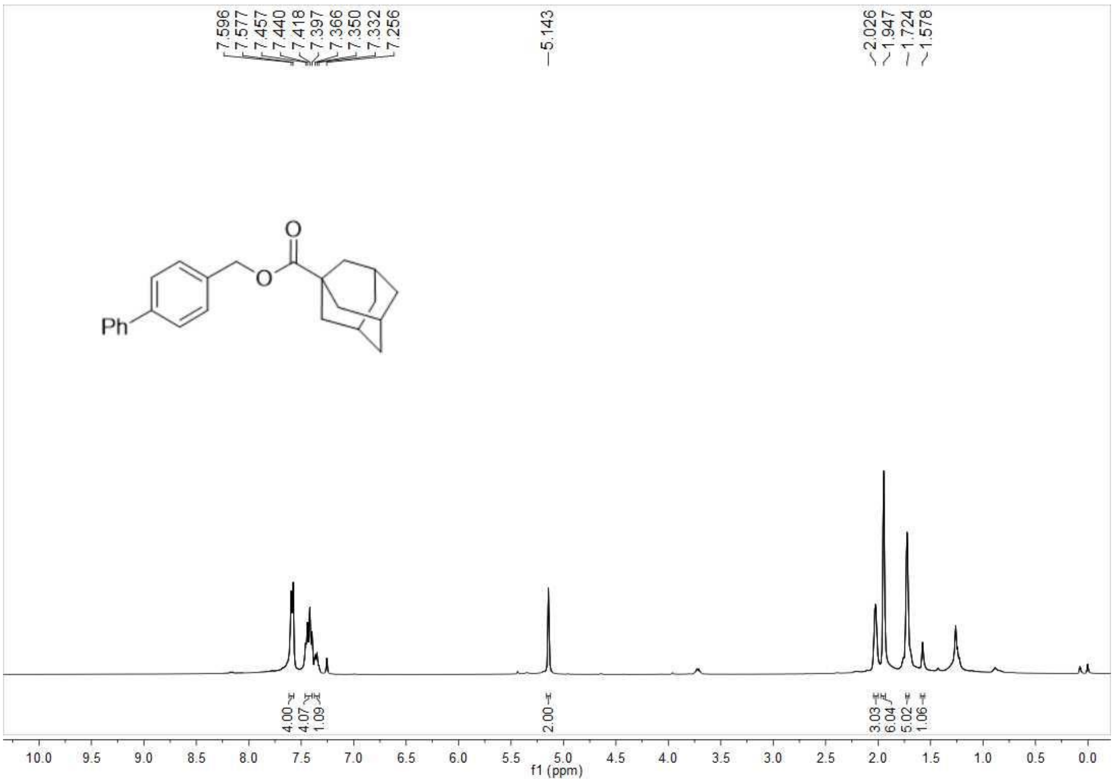

^{13}C NMR Spectrum of **145**

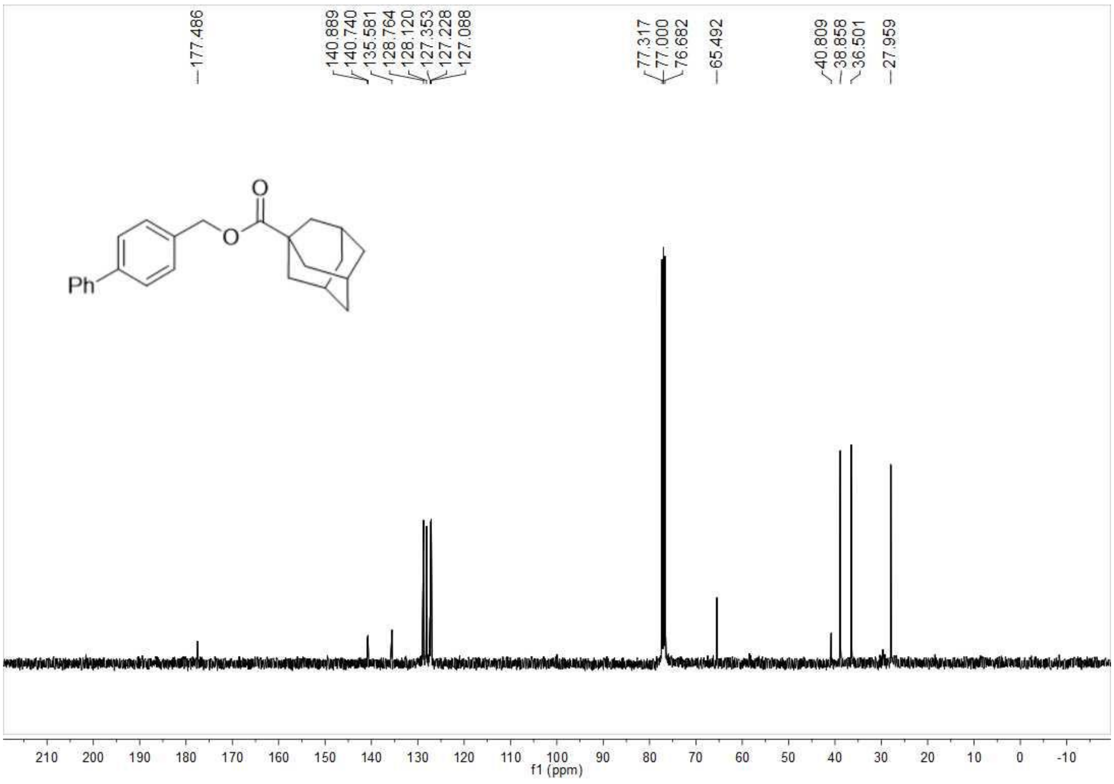

^{1}H NMR Spectrum of **146**

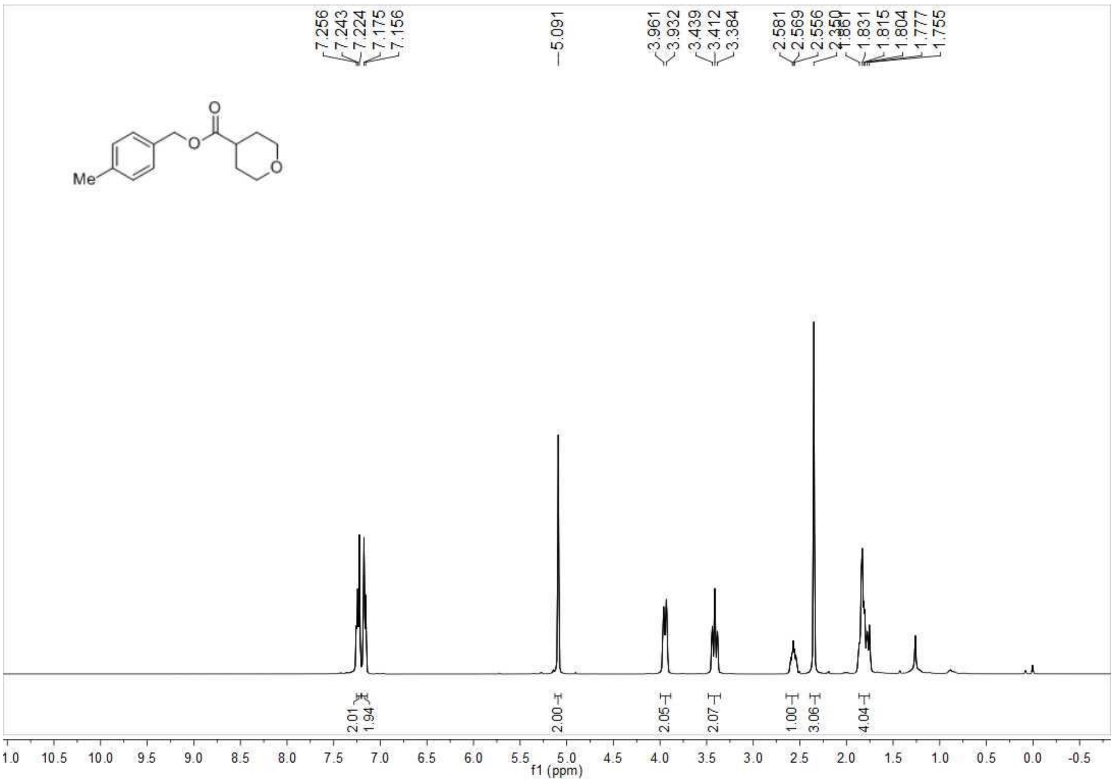

^{13}C NMR Spectrum of **146**

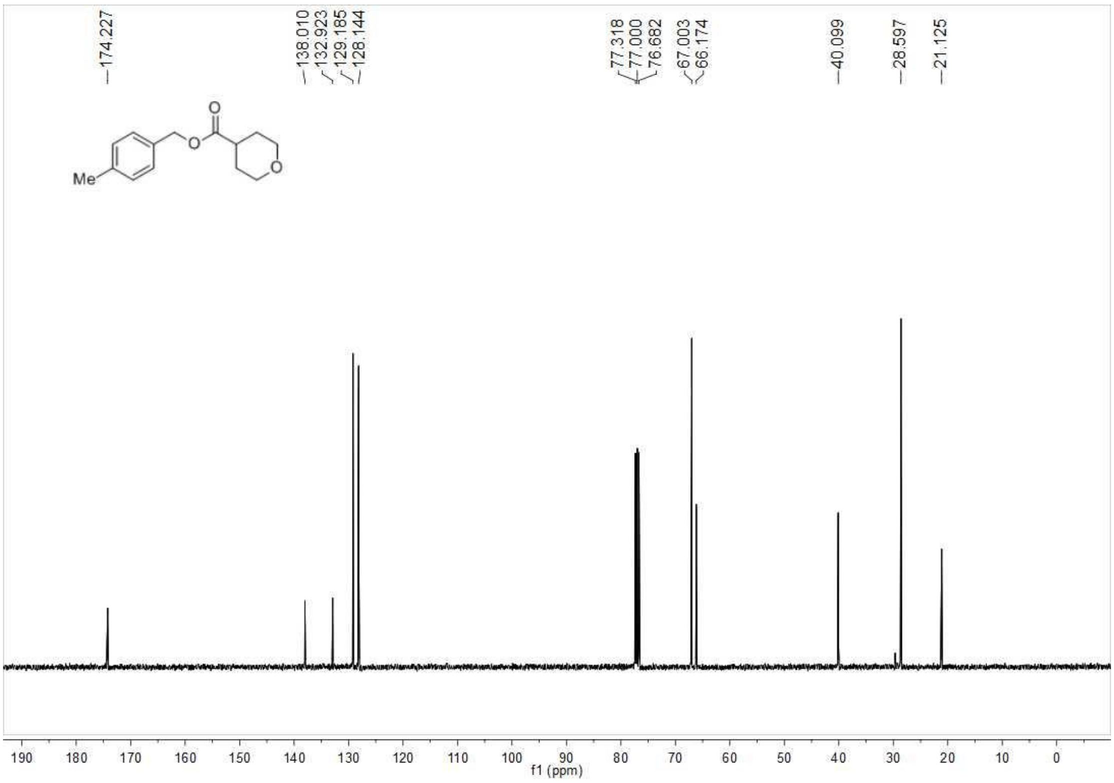

^{1}H NMR Spectrum of **147**

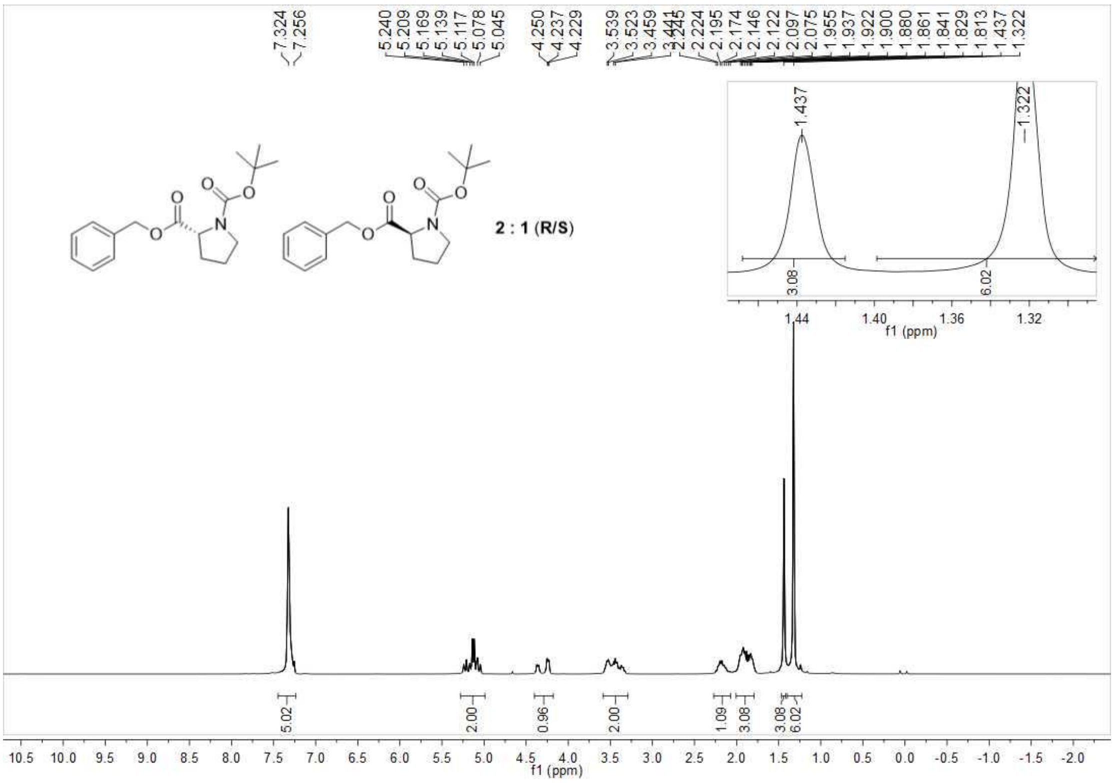

^{13}C NMR Spectrum of **147**

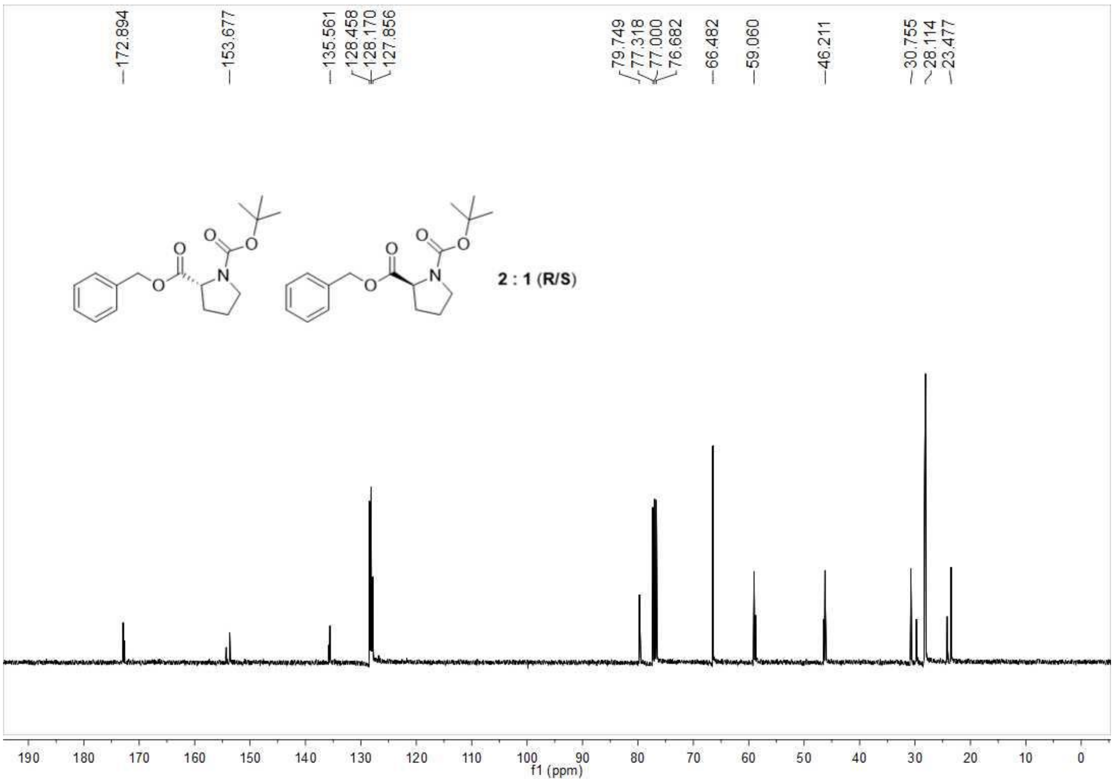

^{1}H NMR Spectrum of **148**

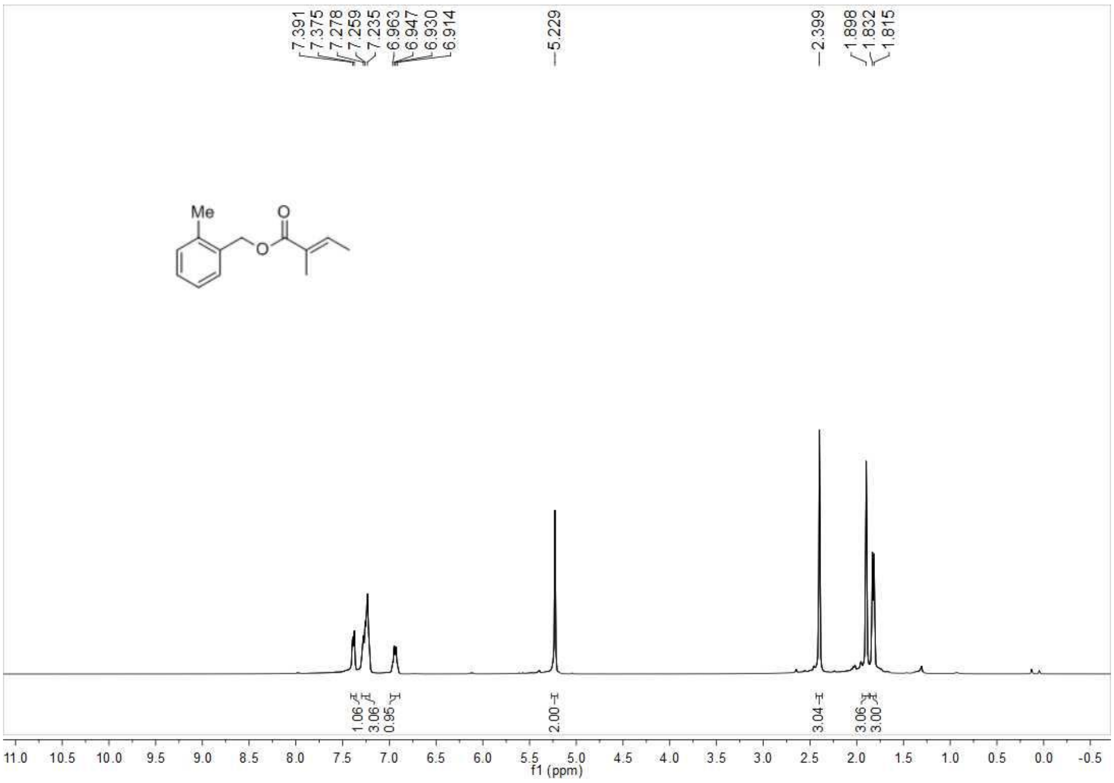

^{13}C NMR Spectrum of **148**

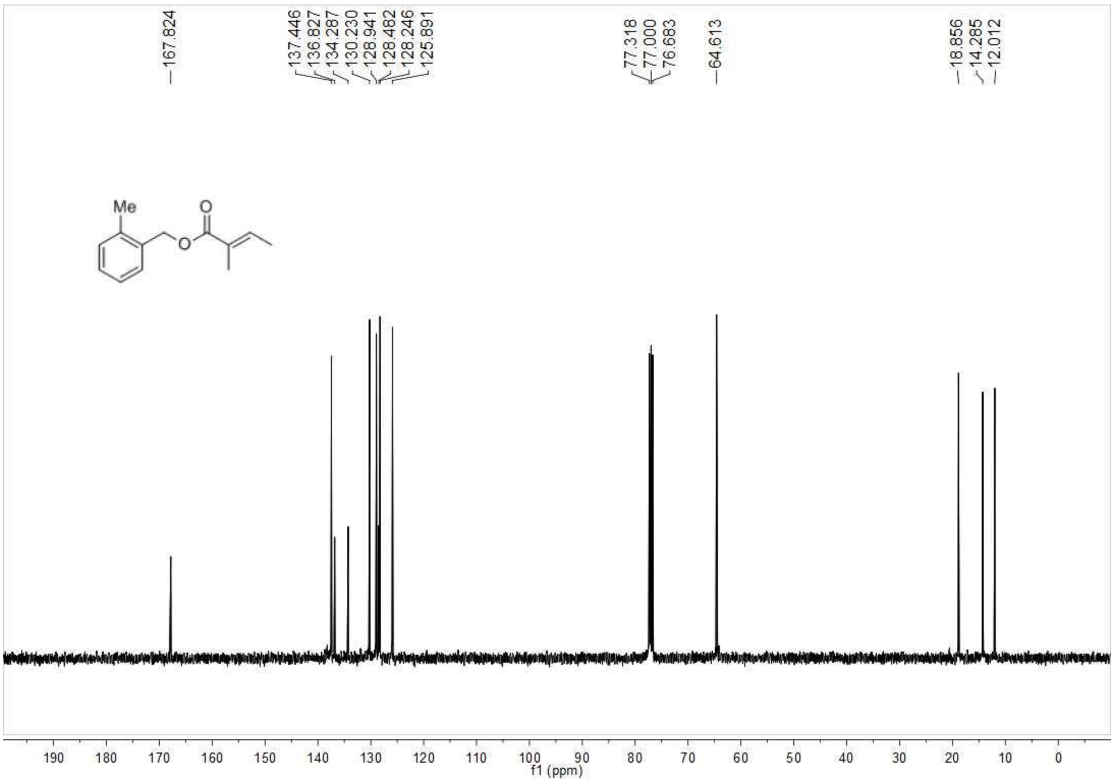

^{1}H NMR Spectrum of **149**

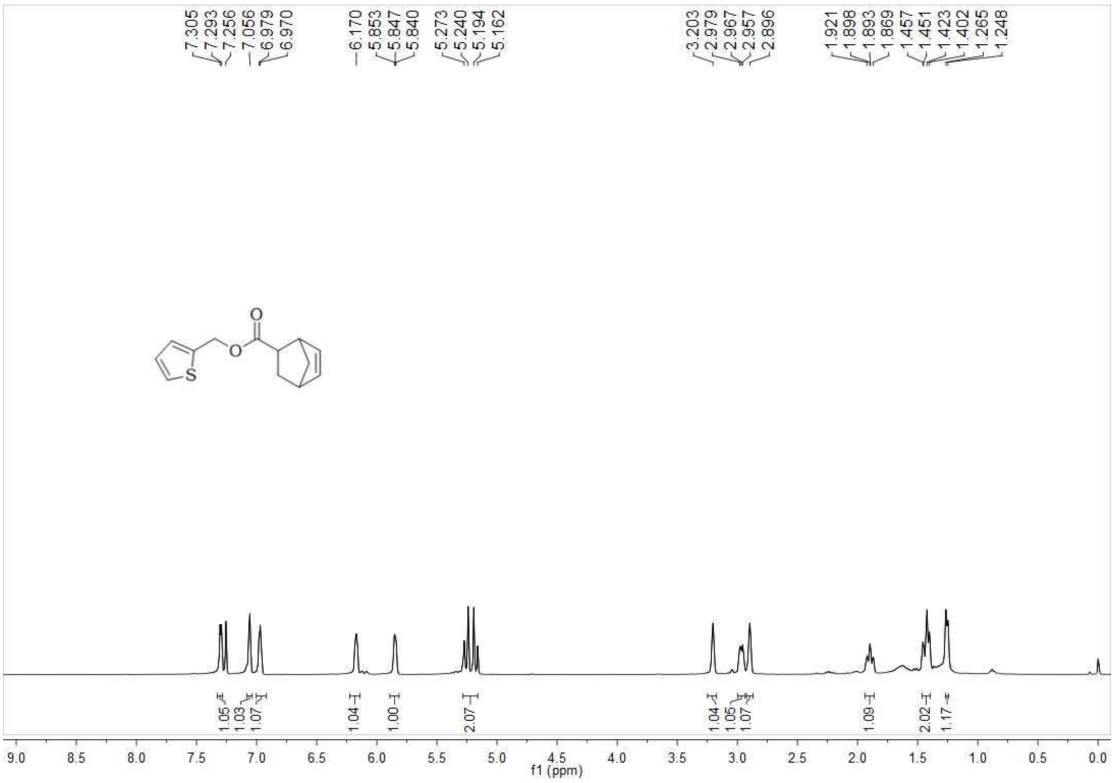

^{13}C NMR Spectrum of **149**

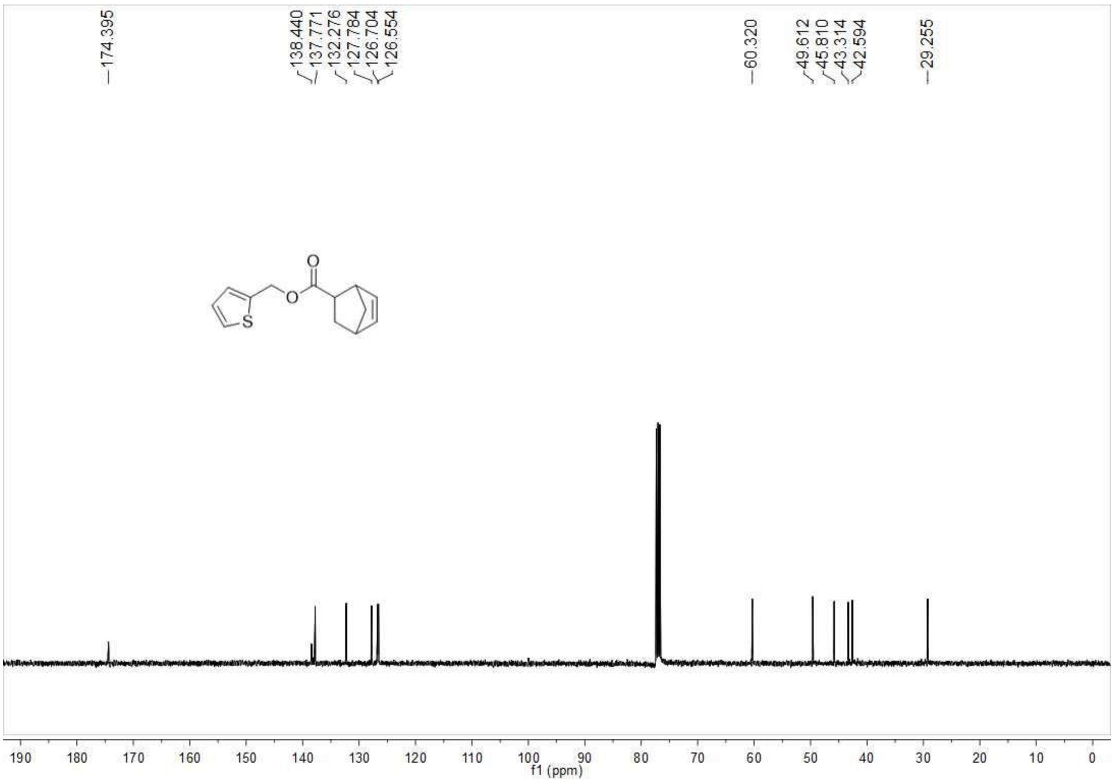

^{1}H NMR Spectrum of **150**

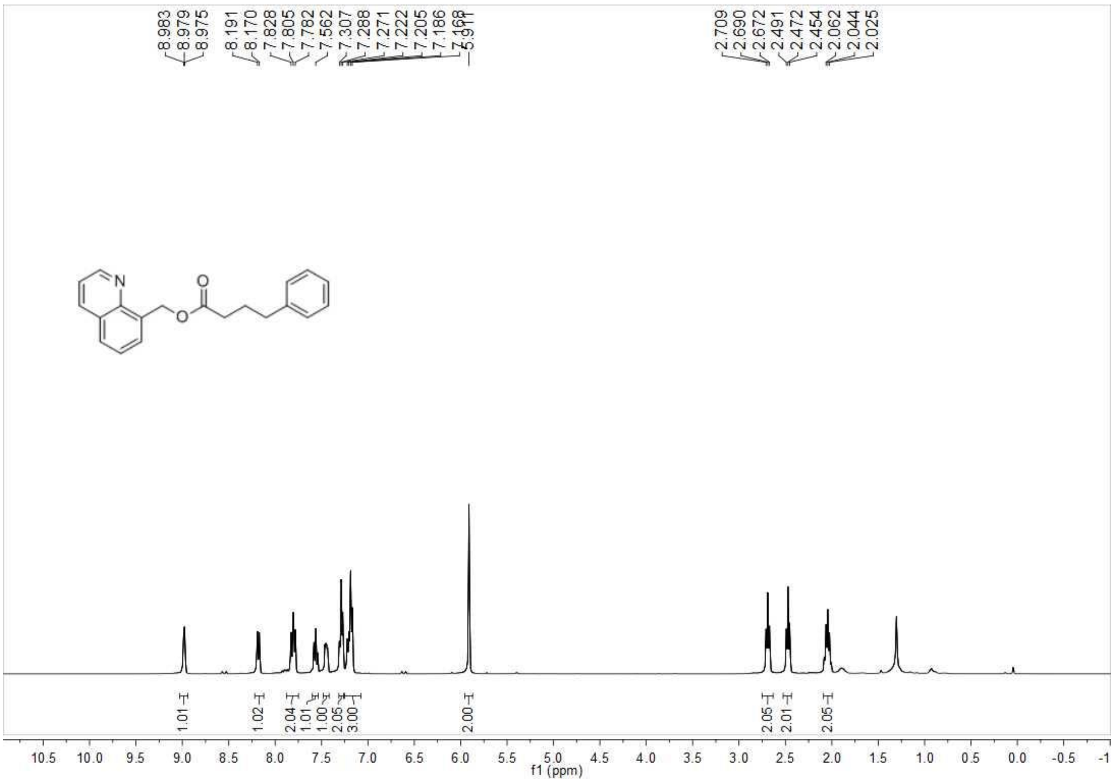

^{13}C NMR Spectrum of **150**

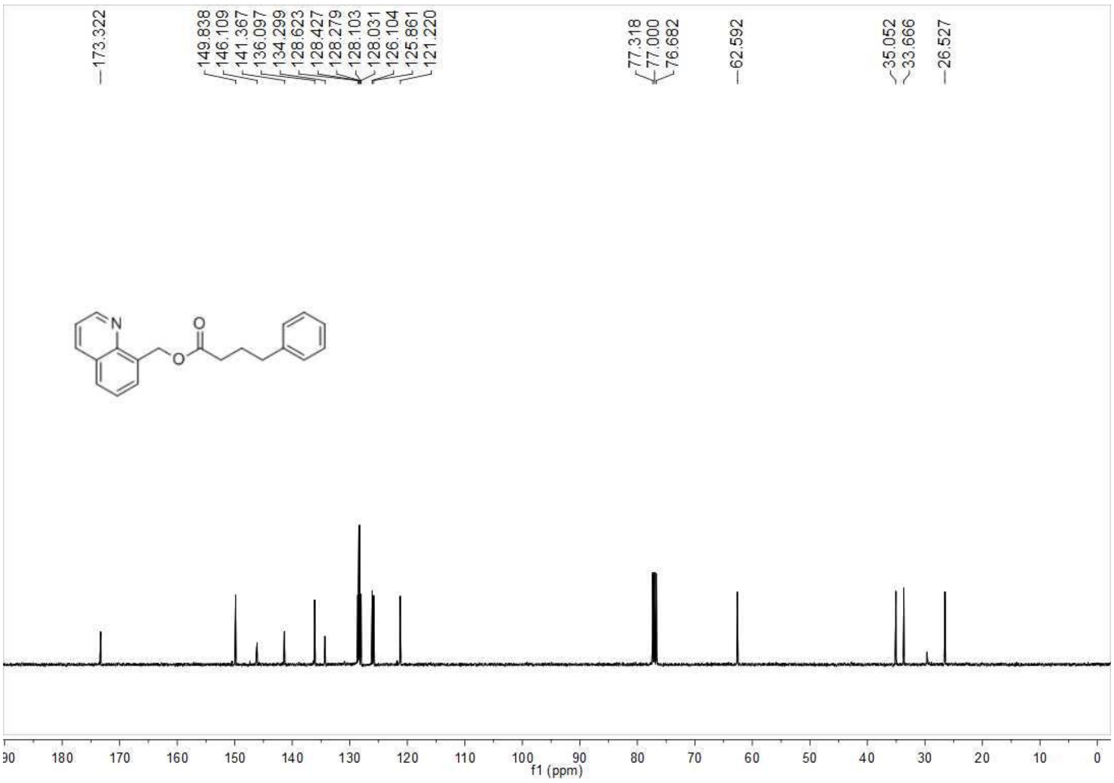

^{1}H NMR Spectrum of **151**

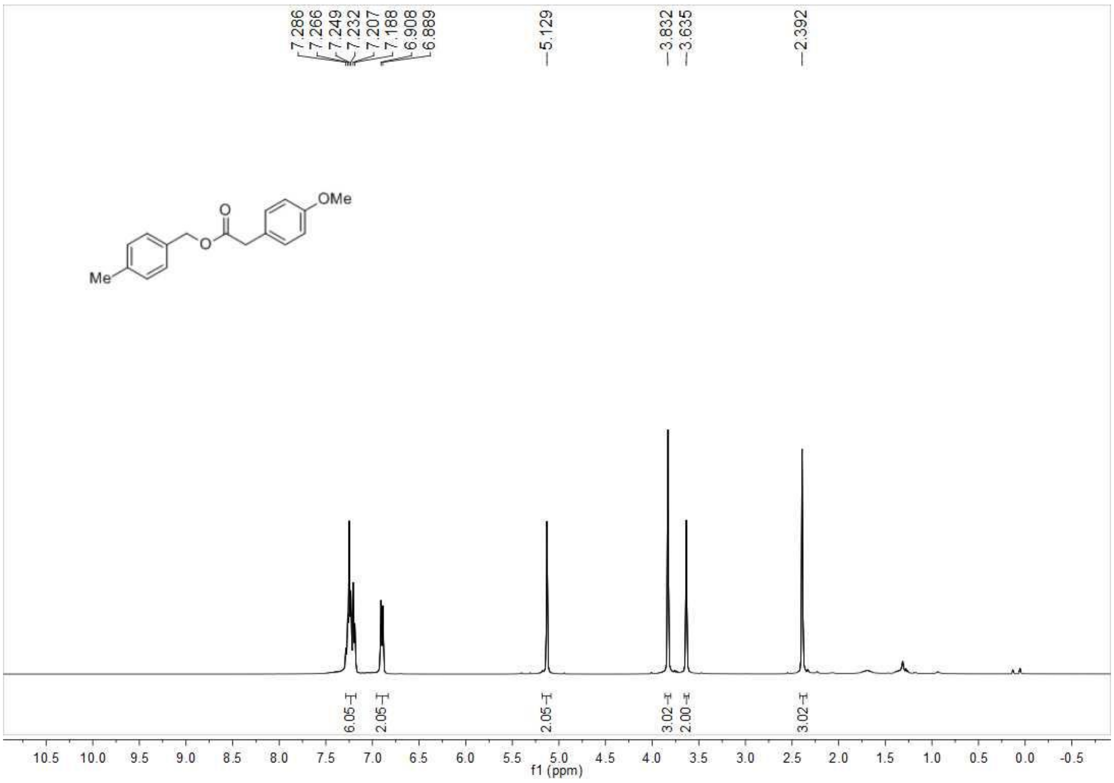

^{13}C NMR Spectrum of **151**

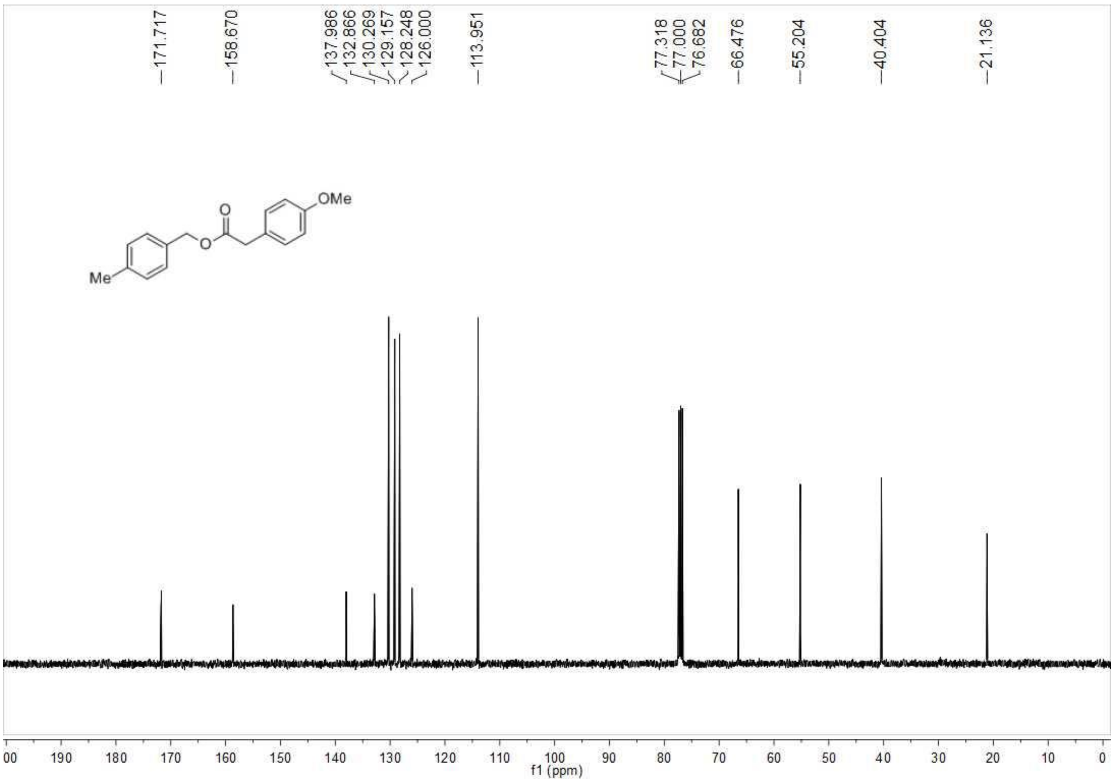

^{1}H NMR Spectrum of **152**

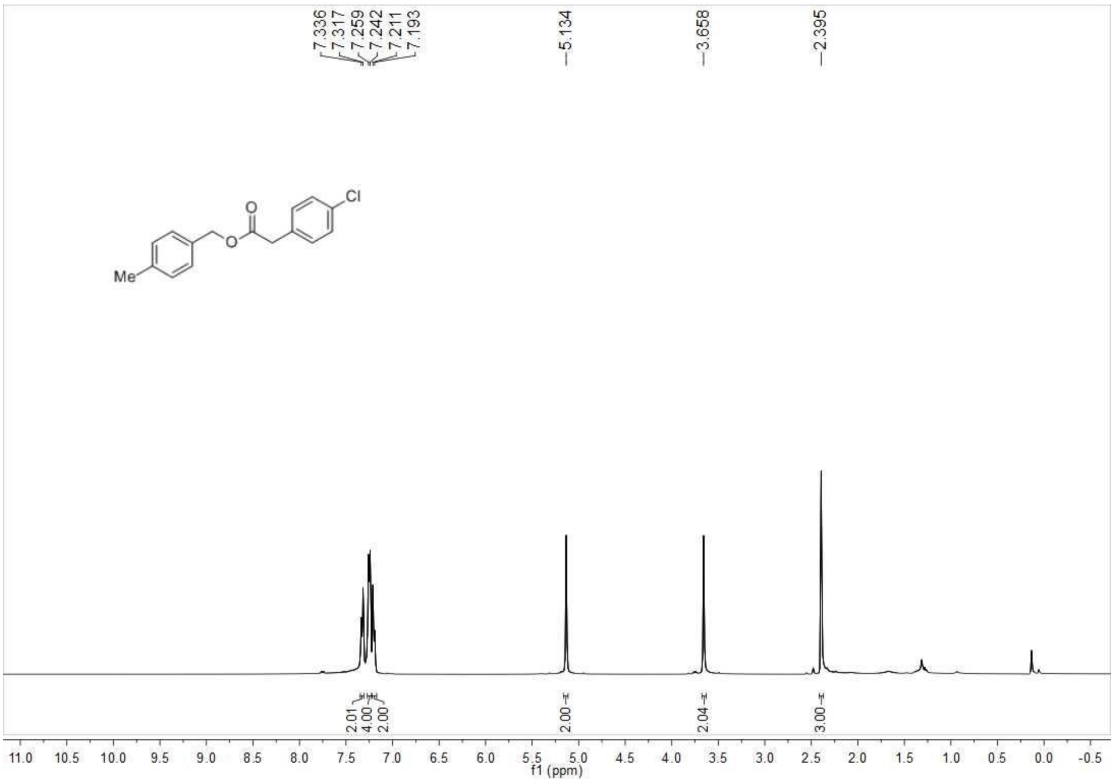

^{13}C NMR Spectrum of **152**

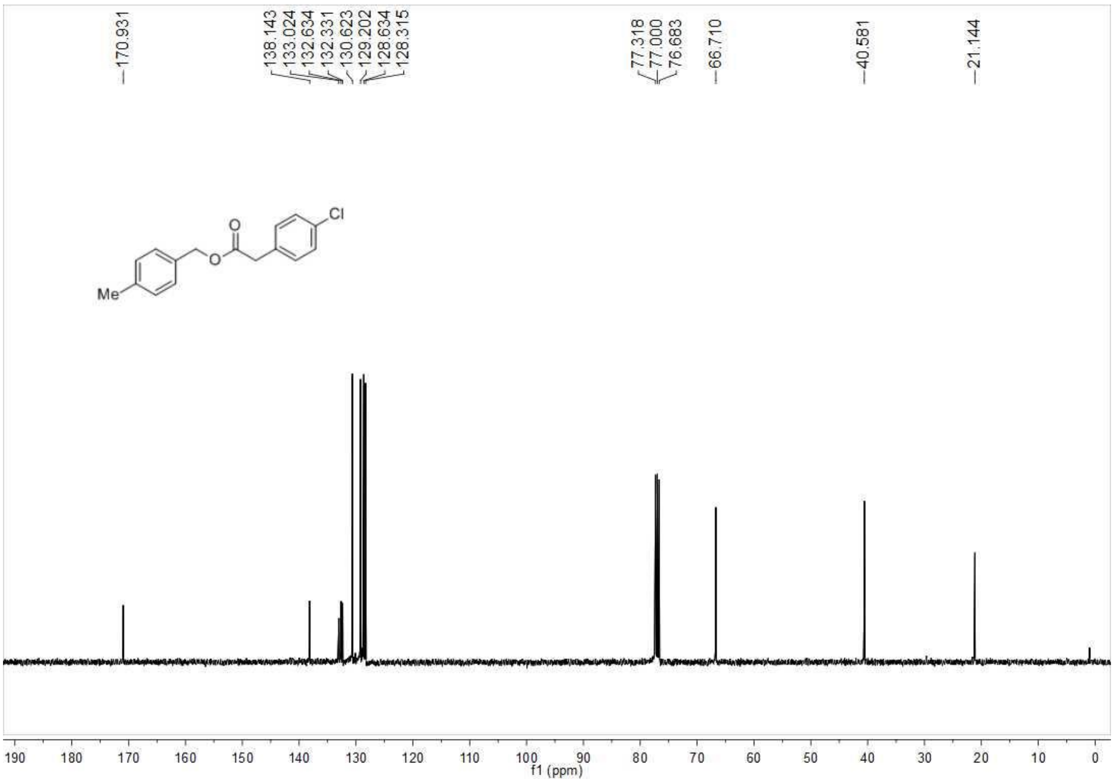

^{1}H NMR Spectrum of **153**

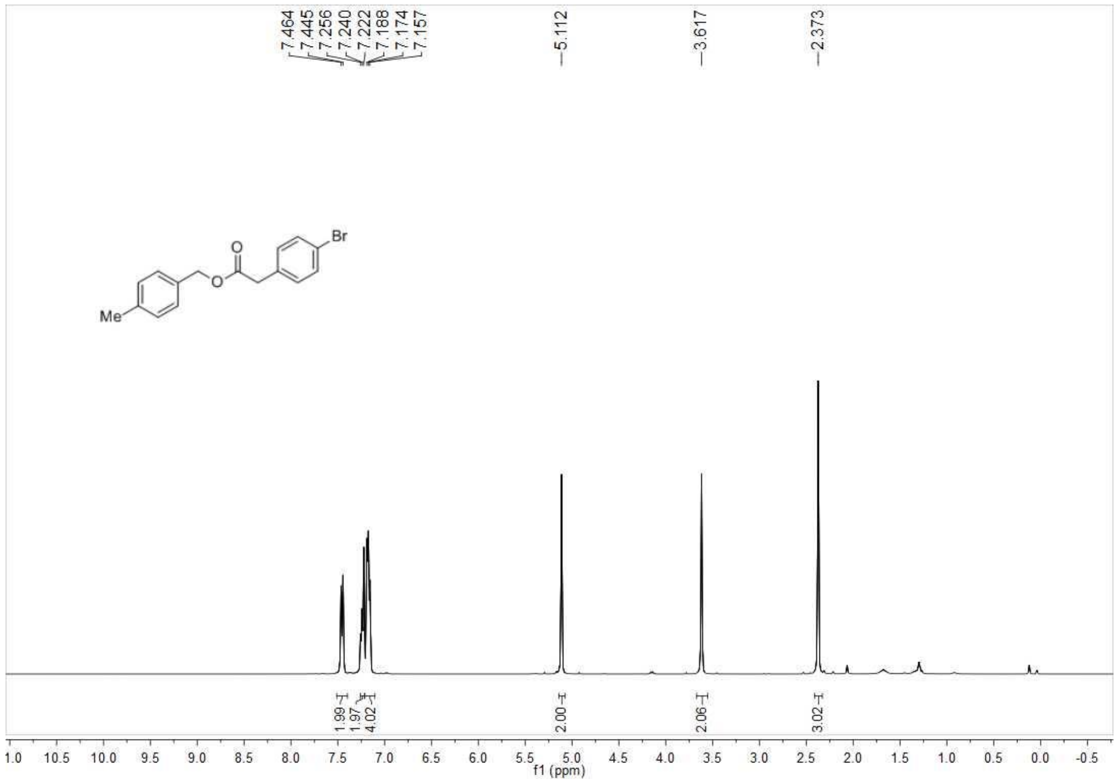

^{13}C NMR Spectrum of **153**

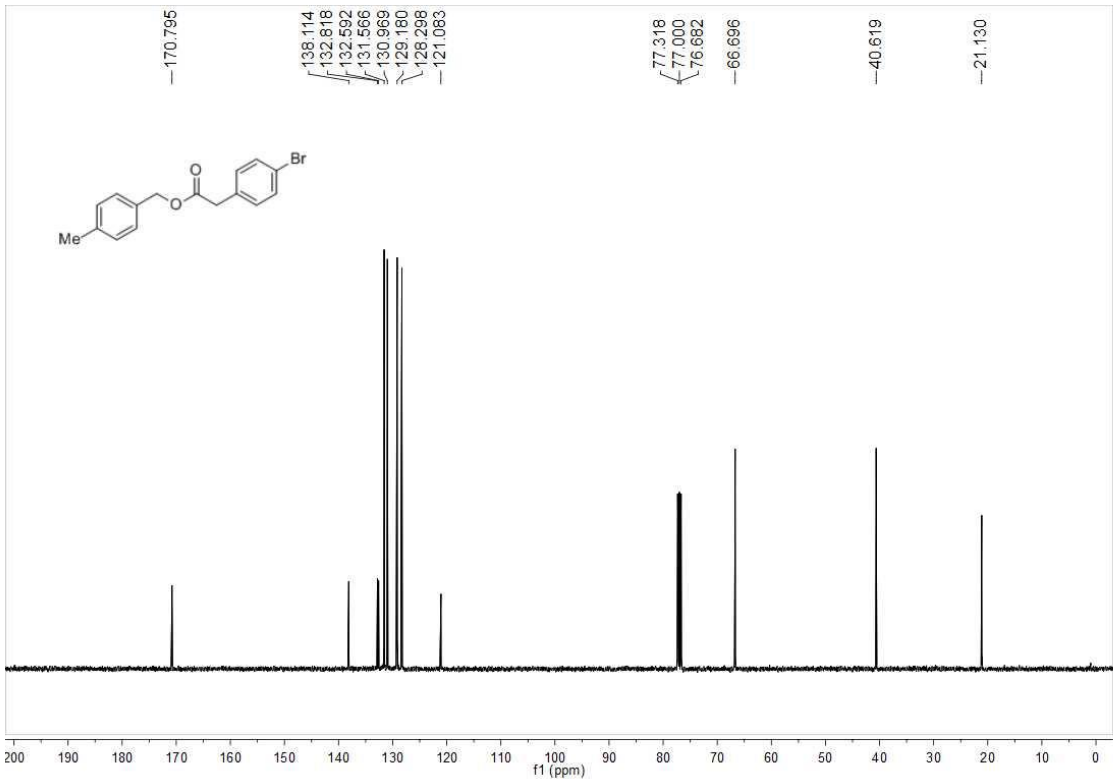

^{1}H NMR Spectrum of **154**

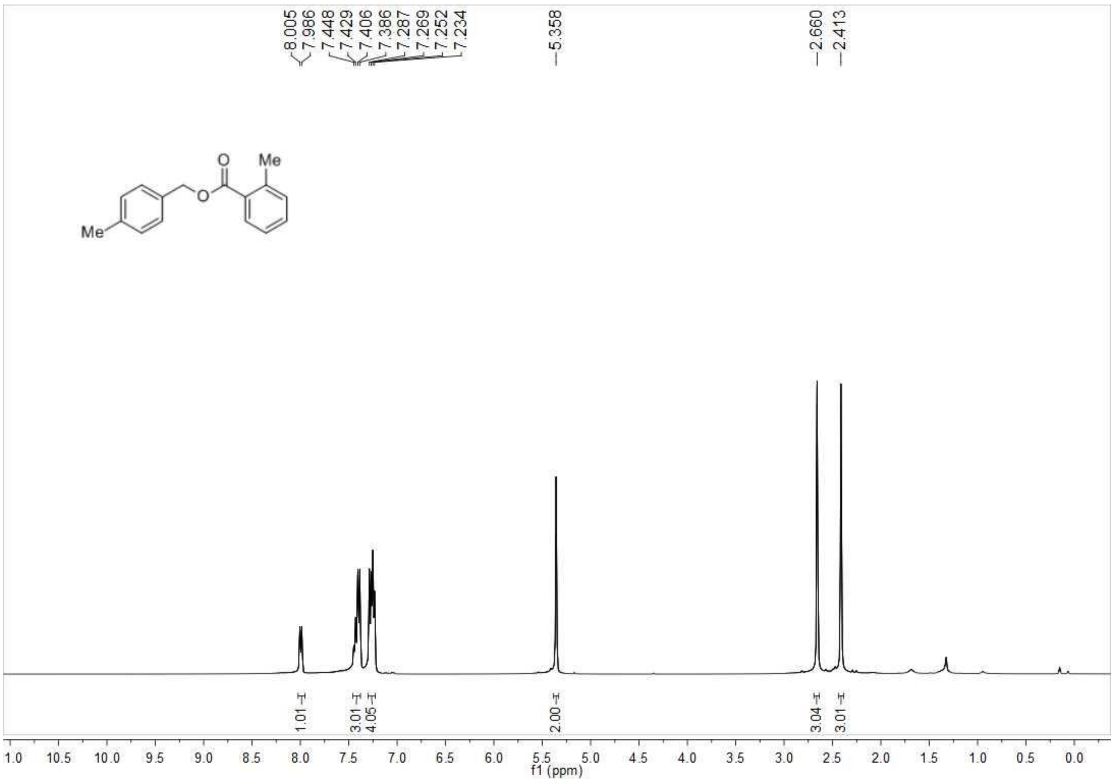

^{13}C NMR Spectrum of **154**

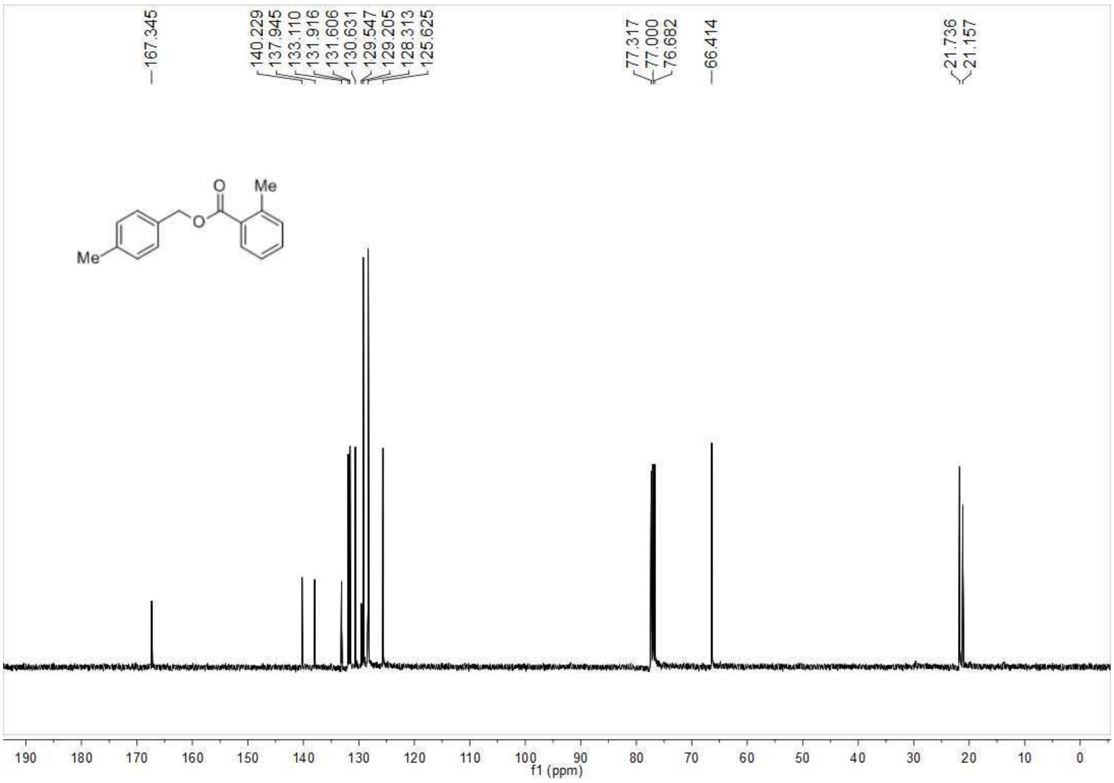

^{1}H NMR Spectrum of **155**

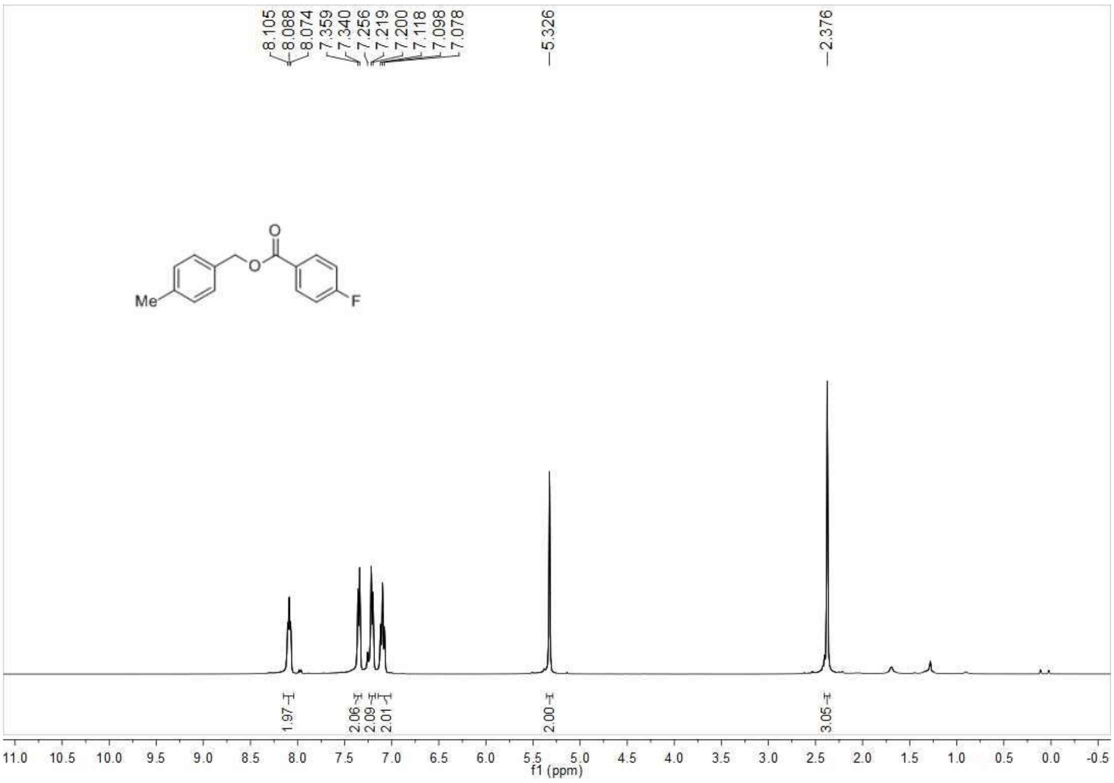

^{13}C NMR Spectrum of **155**

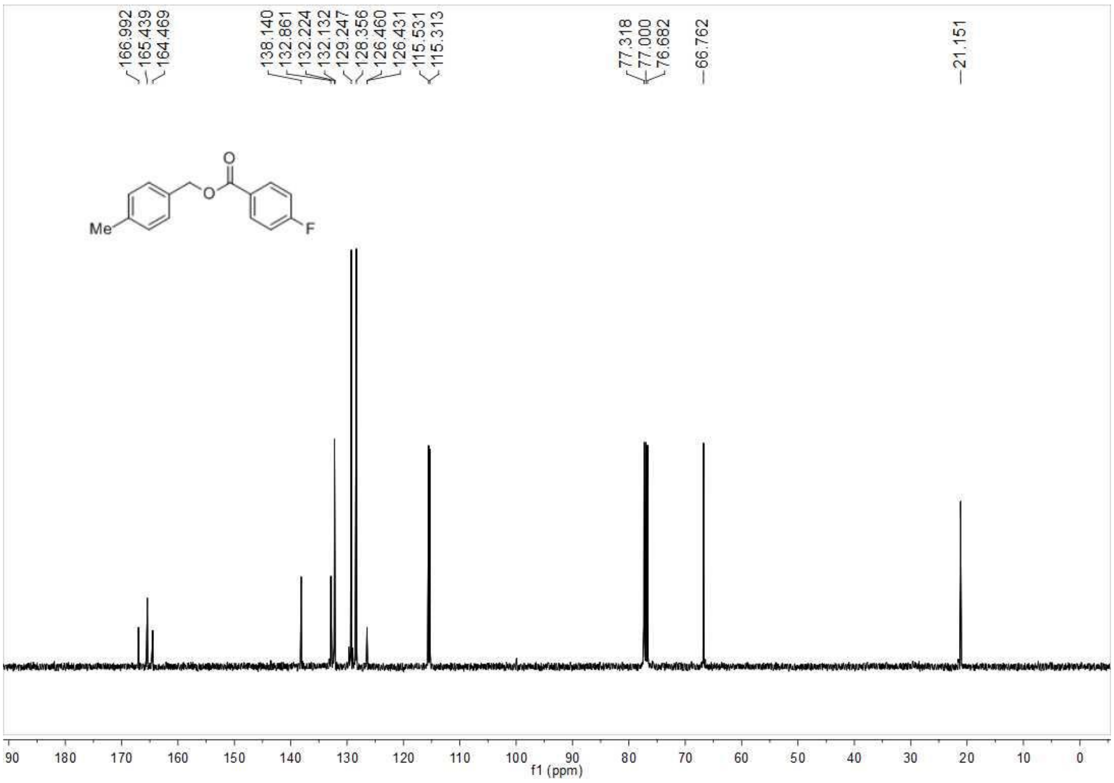

^{19}F NMR Spectrum of **155**

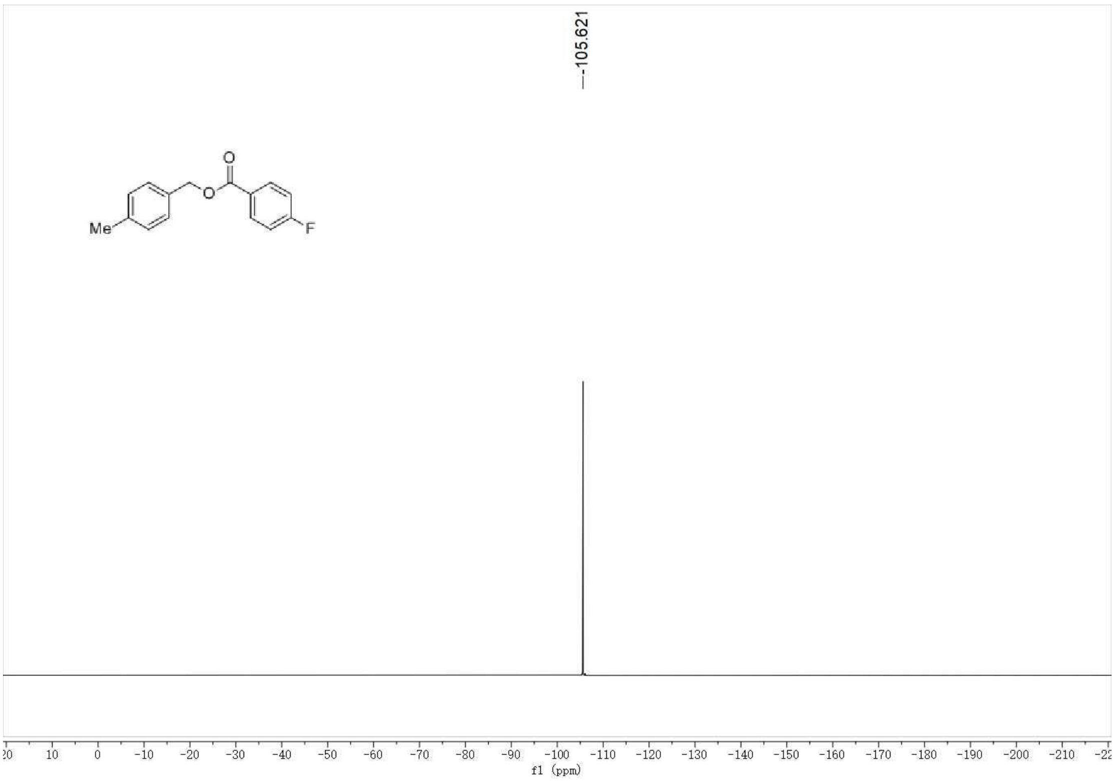

^{1}H NMR Spectrum of **156**

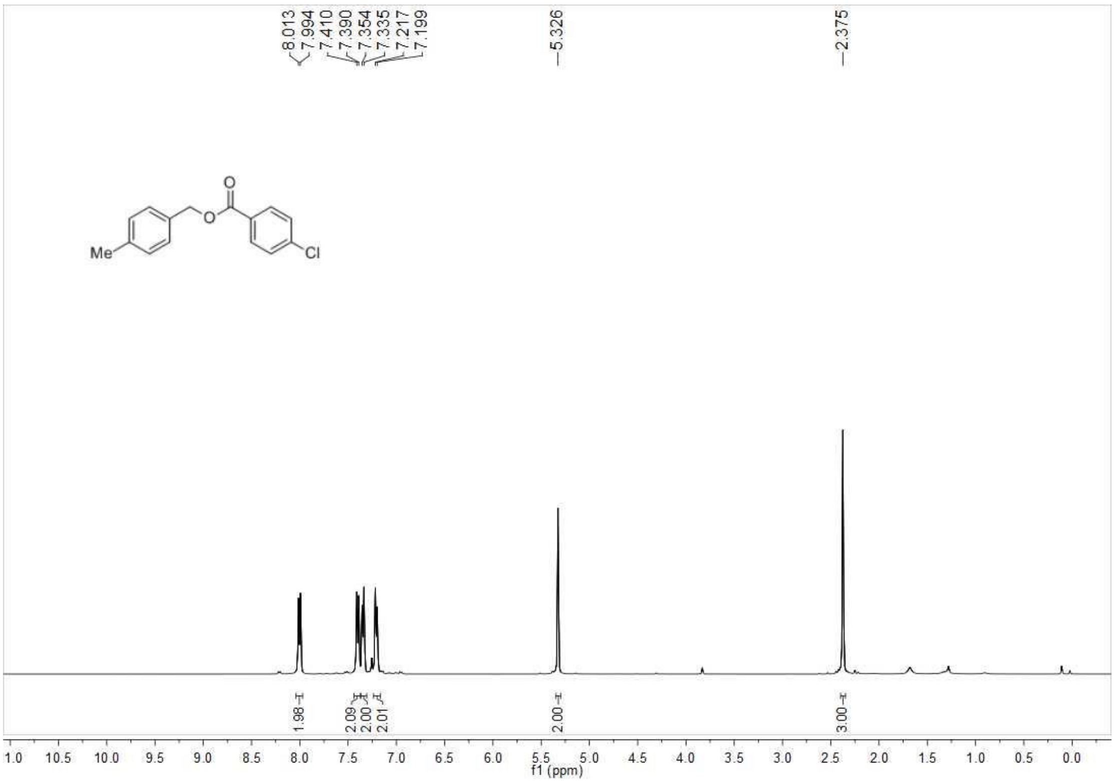

^{13}C NMR Spectrum of **156**

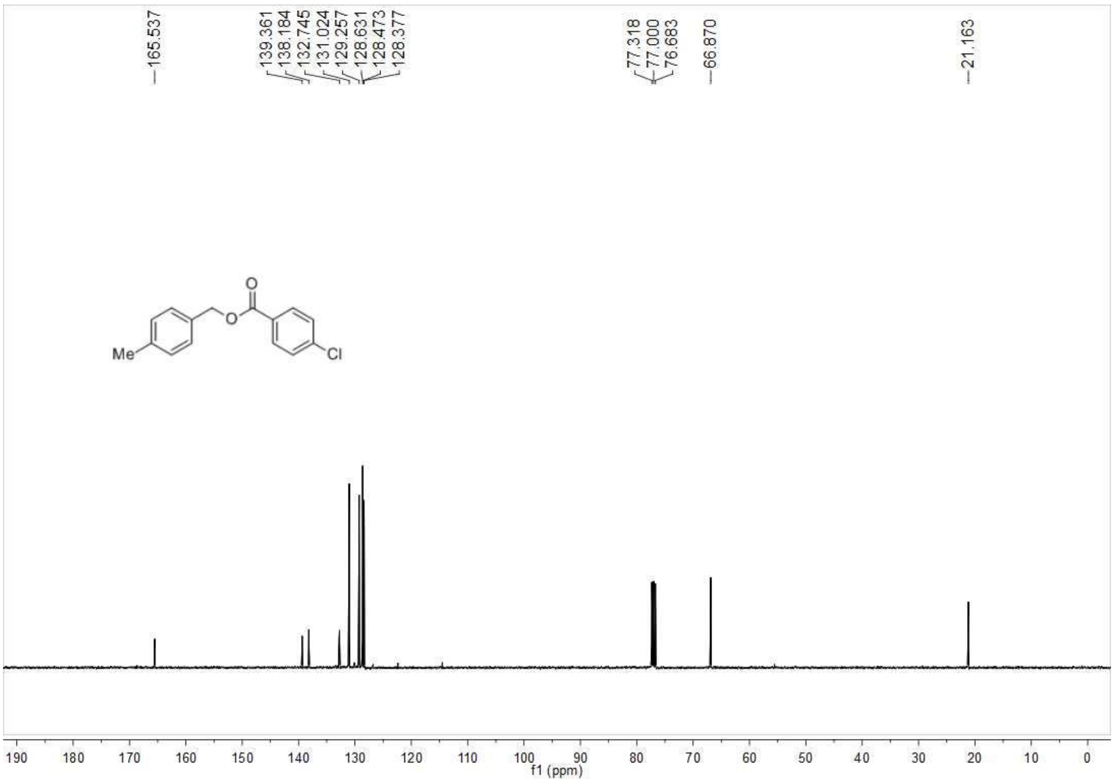

^{1}H NMR Spectrum of **157**

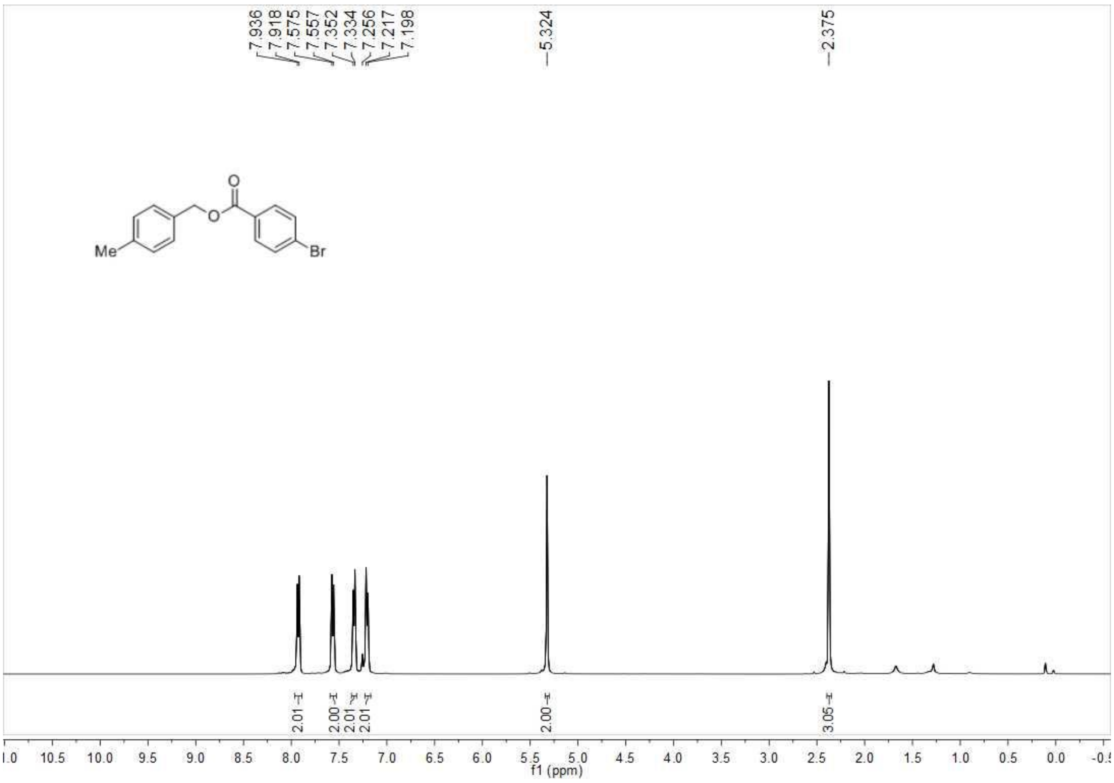

^{13}C NMR Spectrum of **157**

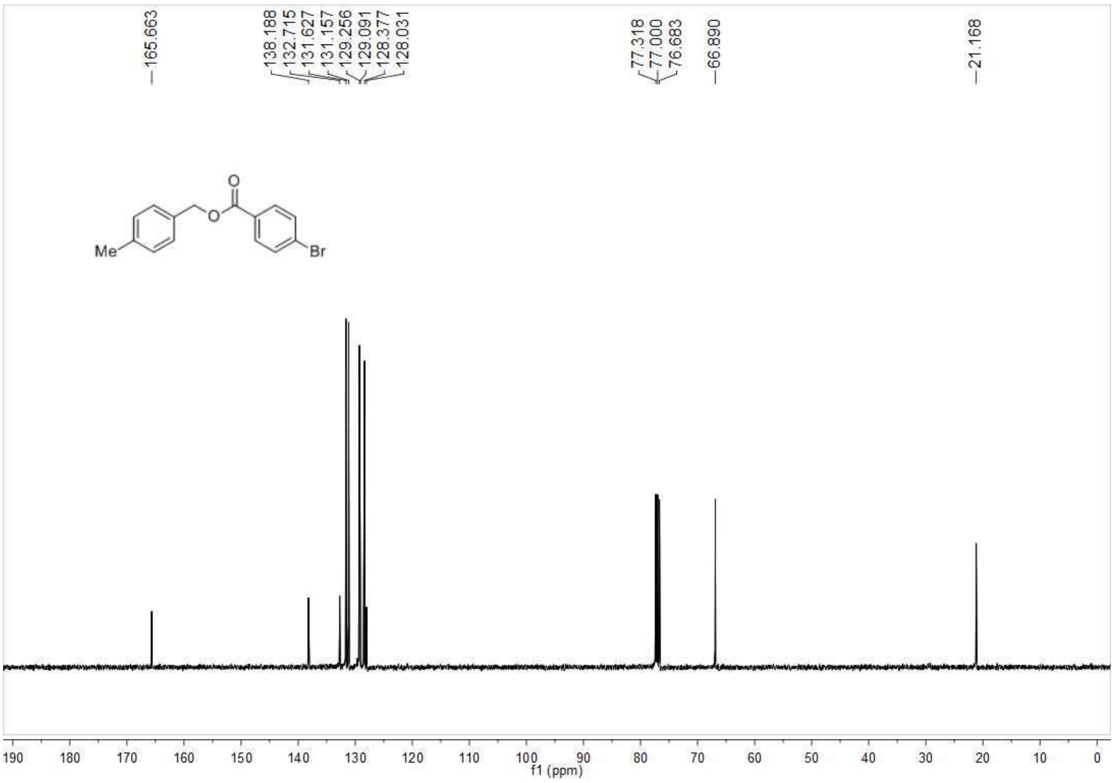

^{1}H NMR Spectrum of **158**

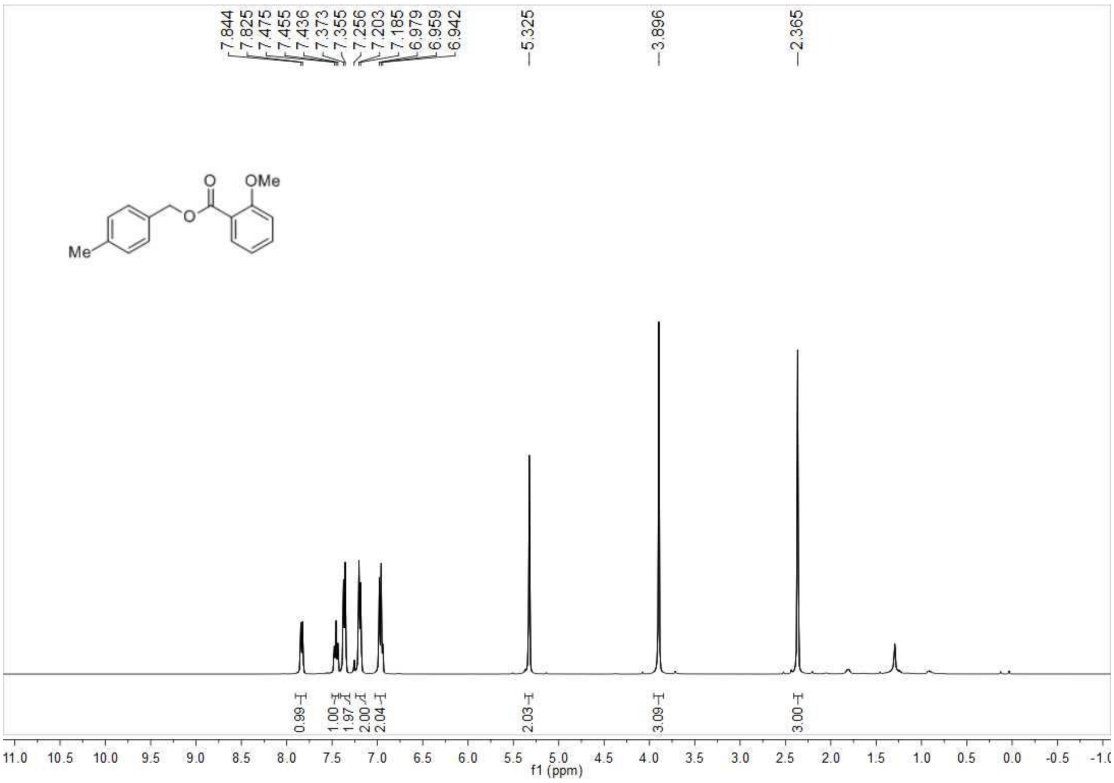

^{13}C NMR Spectrum of **158**

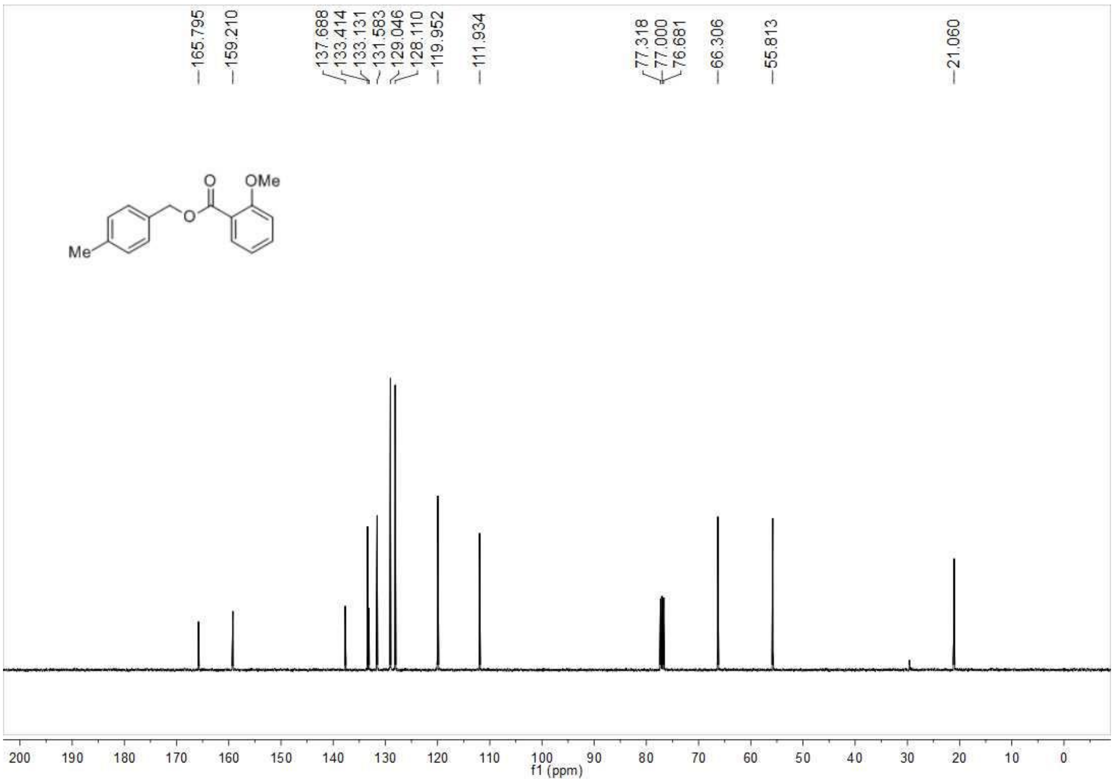

^{1}H NMR Spectrum of **159**

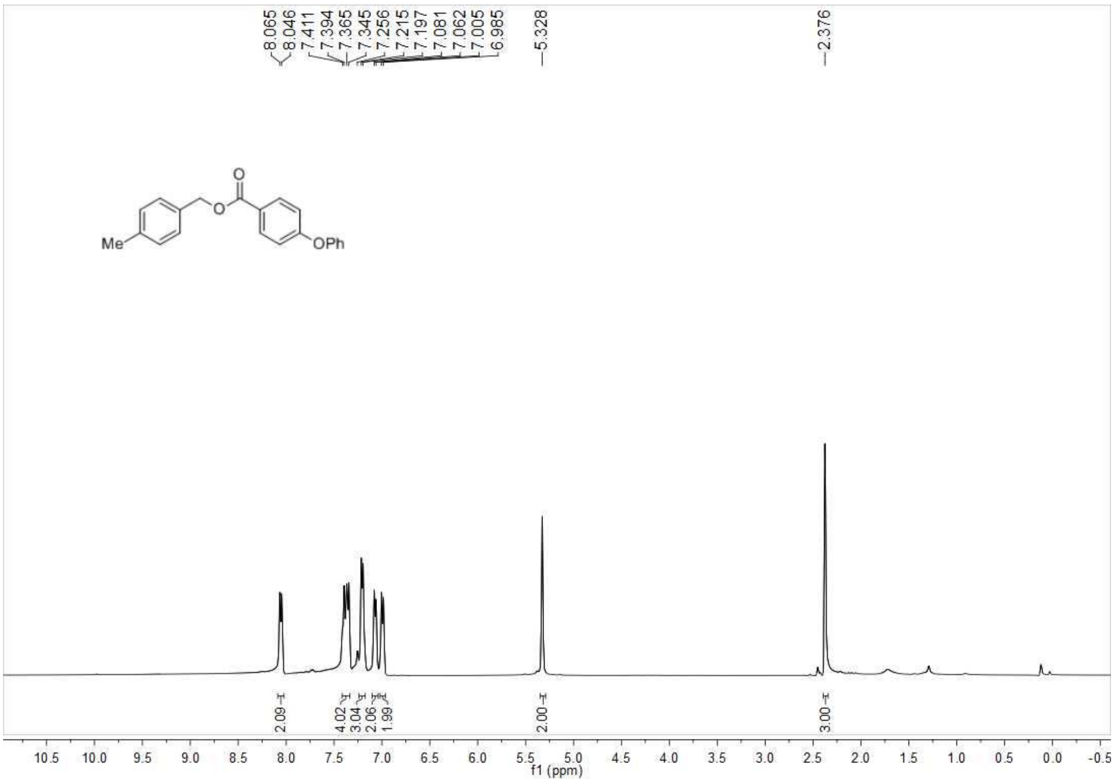

^{13}C NMR Spectrum of **159**

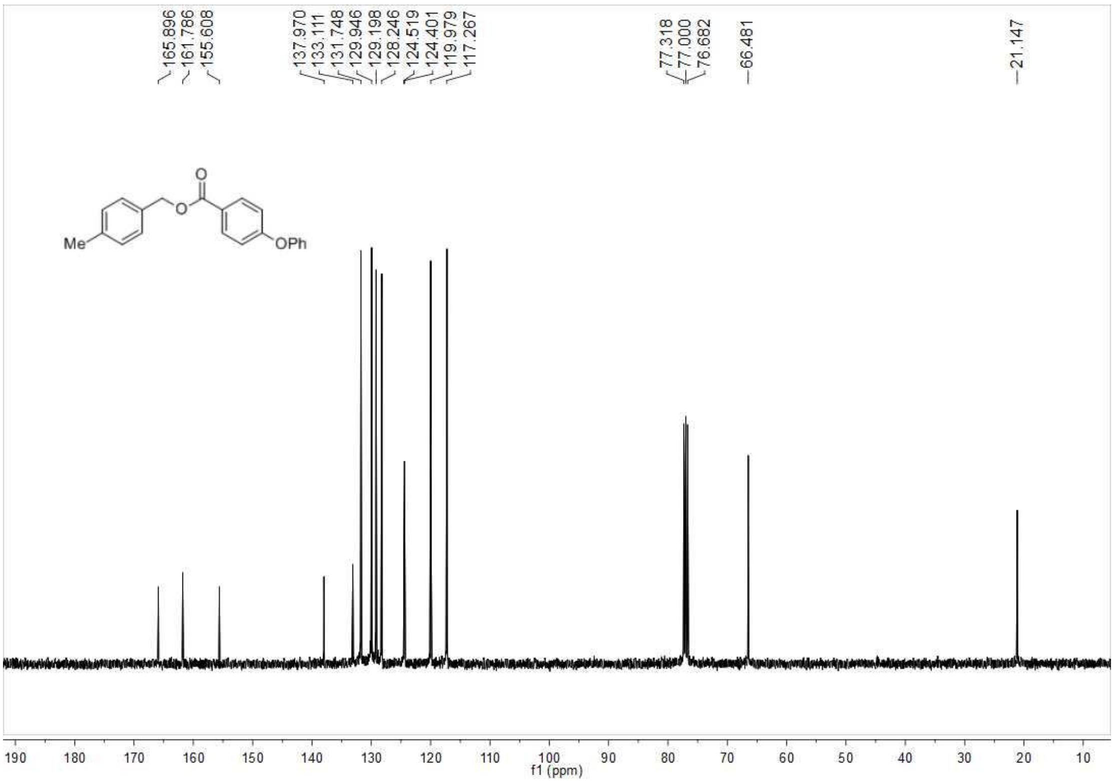

^{1}H NMR Spectrum of **160**

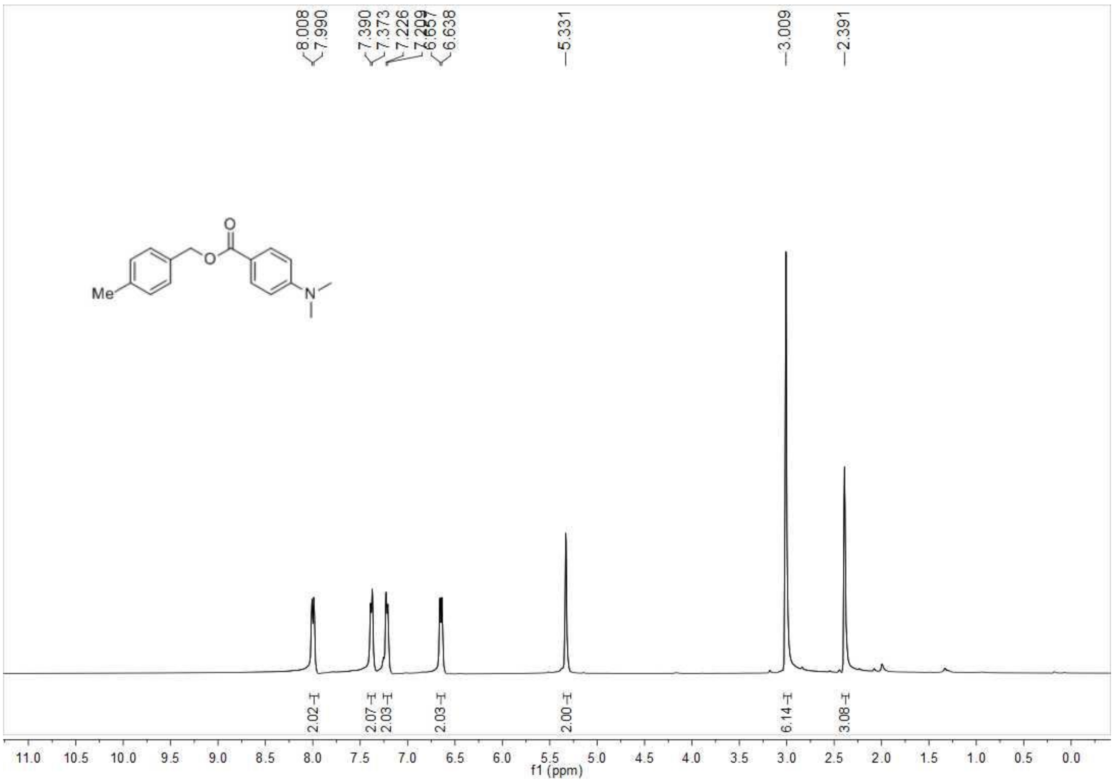

^{13}C NMR Spectrum of **160**

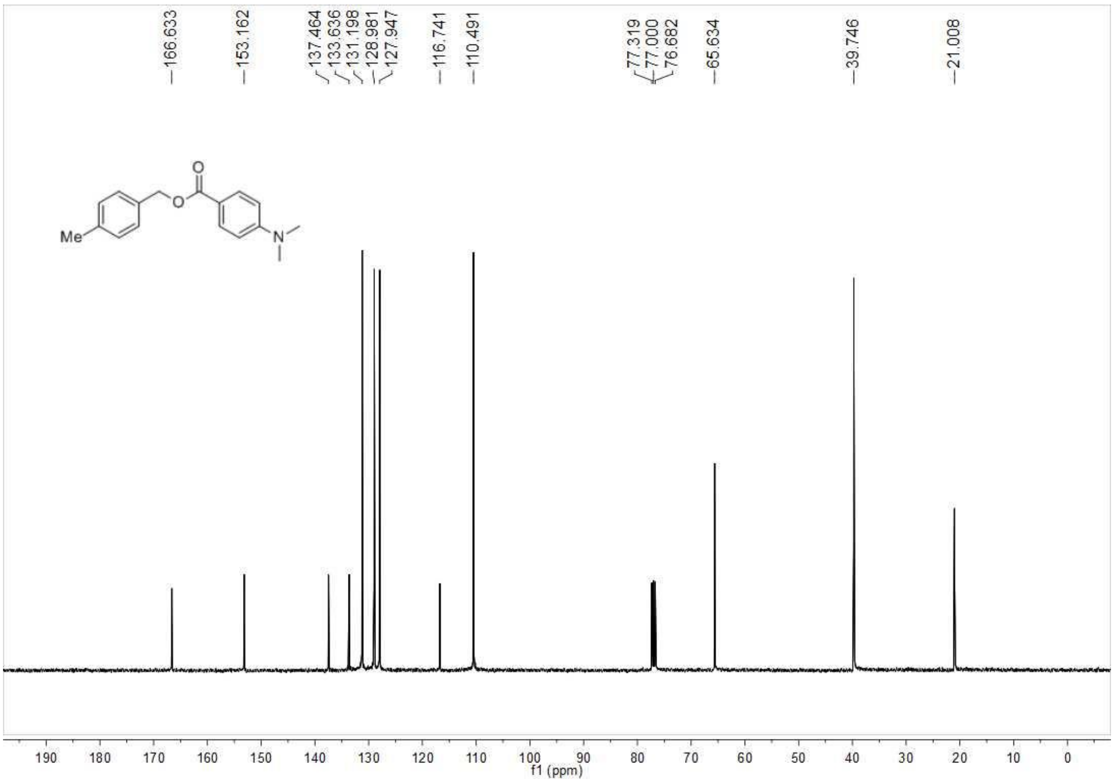

^{1}H NMR Spectrum of **161**

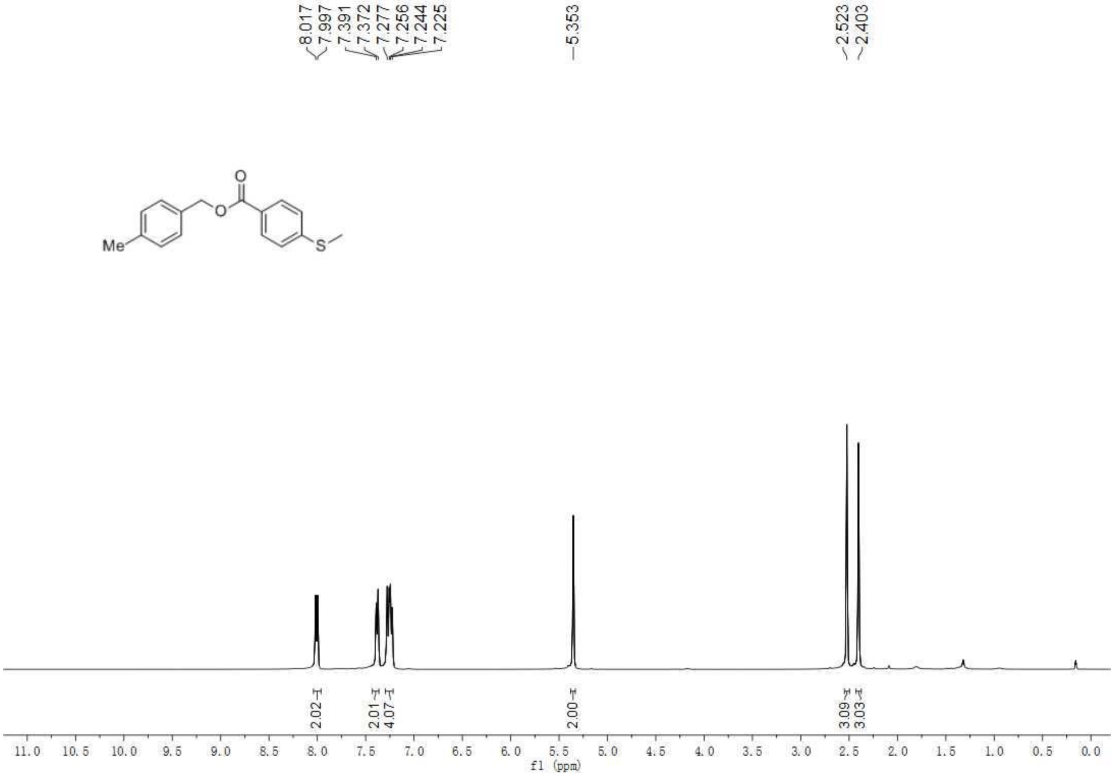

^{13}C NMR Spectrum of **161**

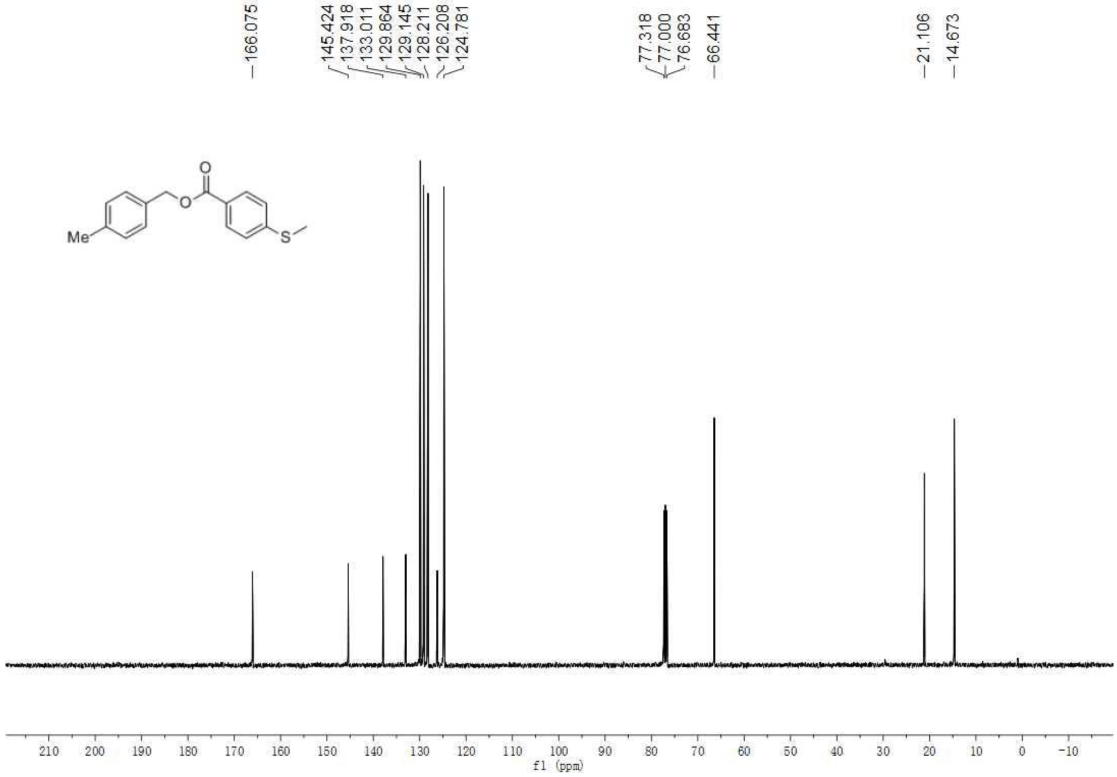

^{1}H NMR Spectrum of **162**

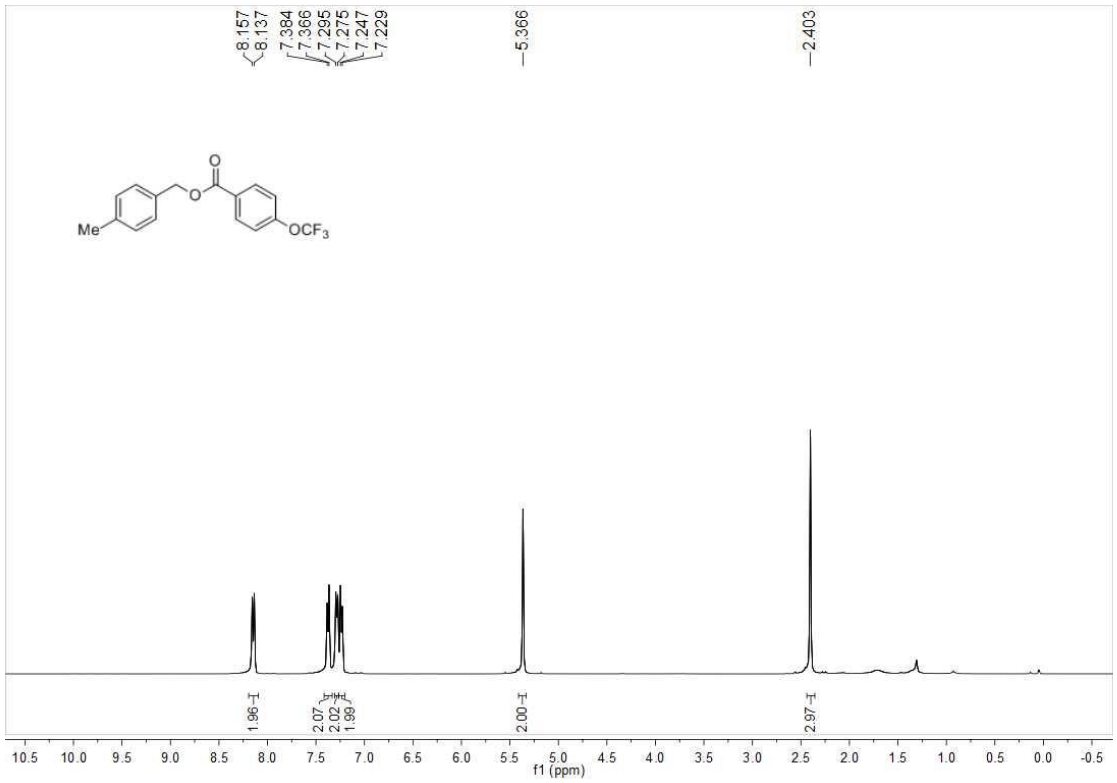

^{13}C NMR Spectrum of **162**

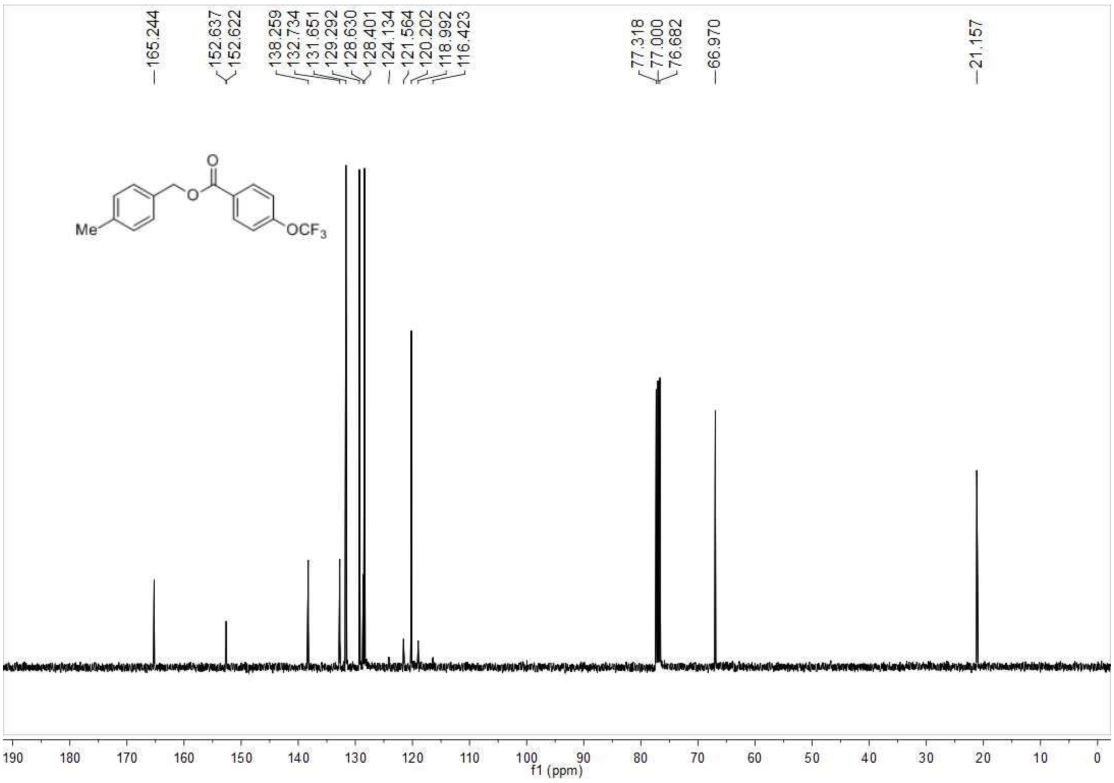

^{19}F NMR Spectrum of **162**

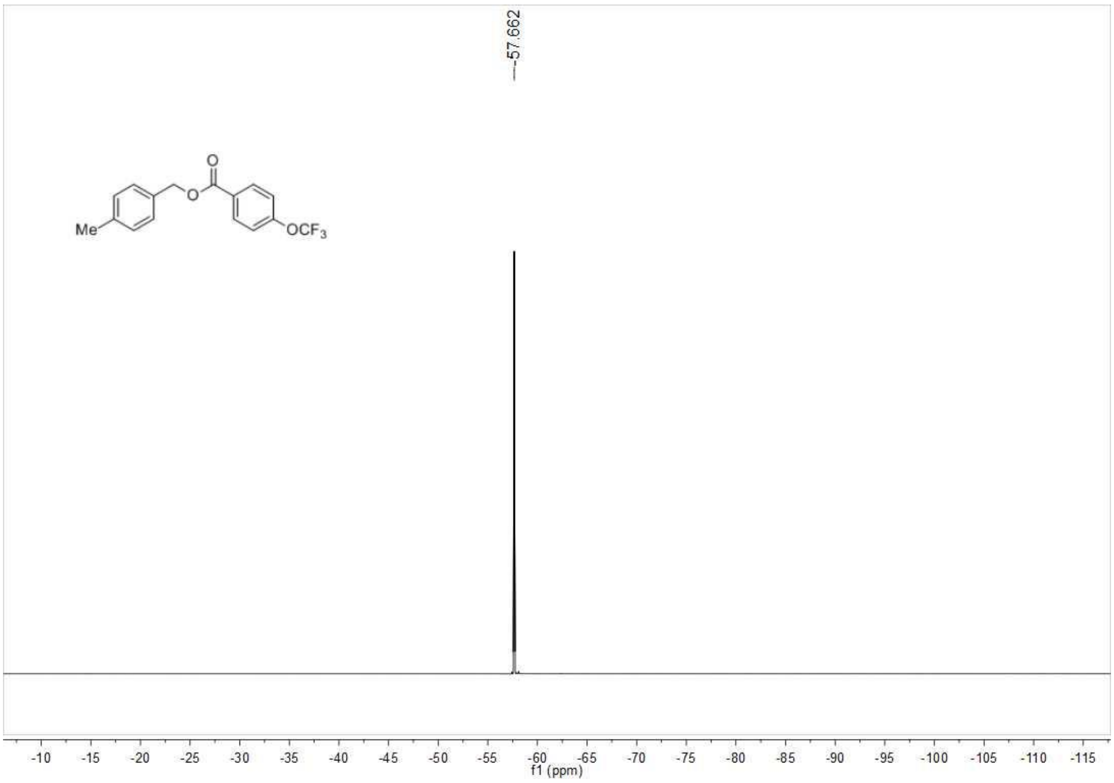

^{1}H NMR Spectrum of **163**

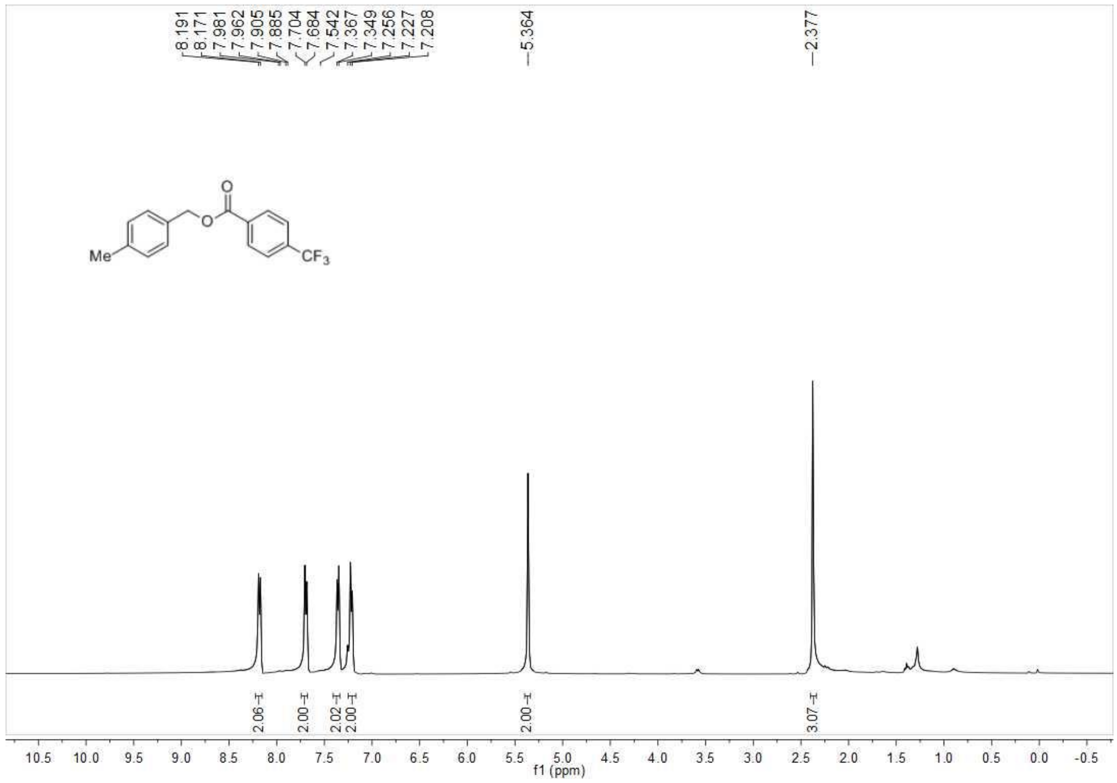

^{13}C NMR Spectrum of **163**

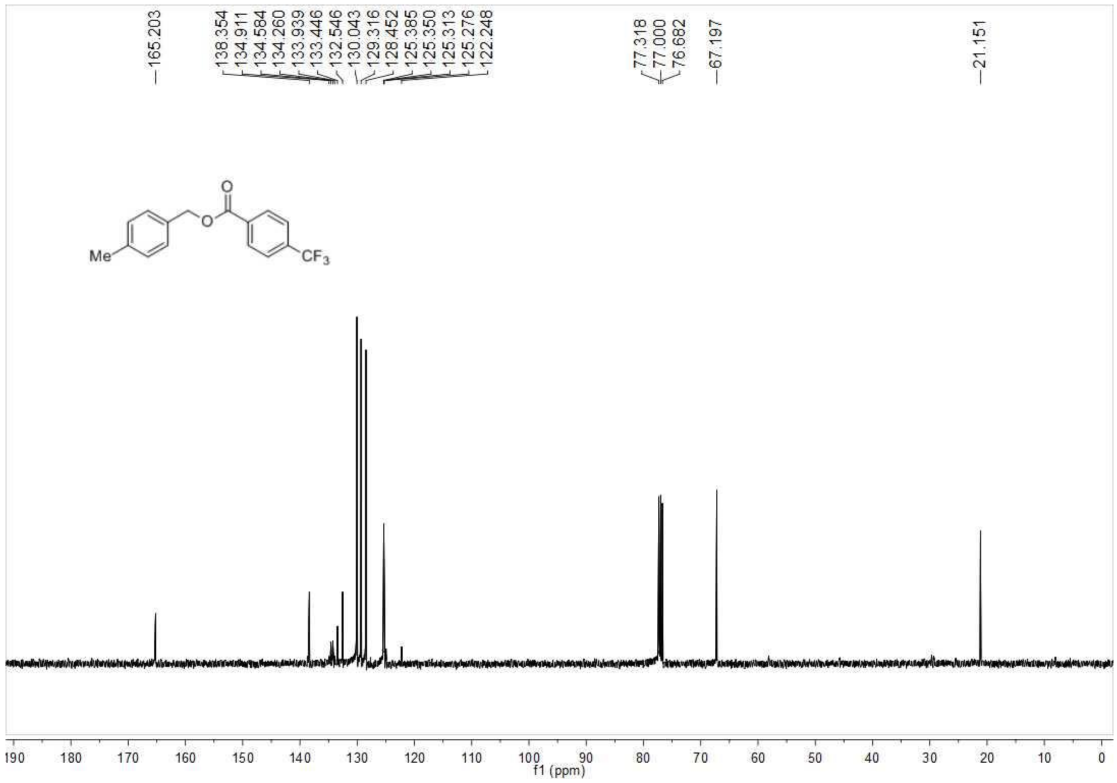

^{19}F NMR Spectrum of **163**

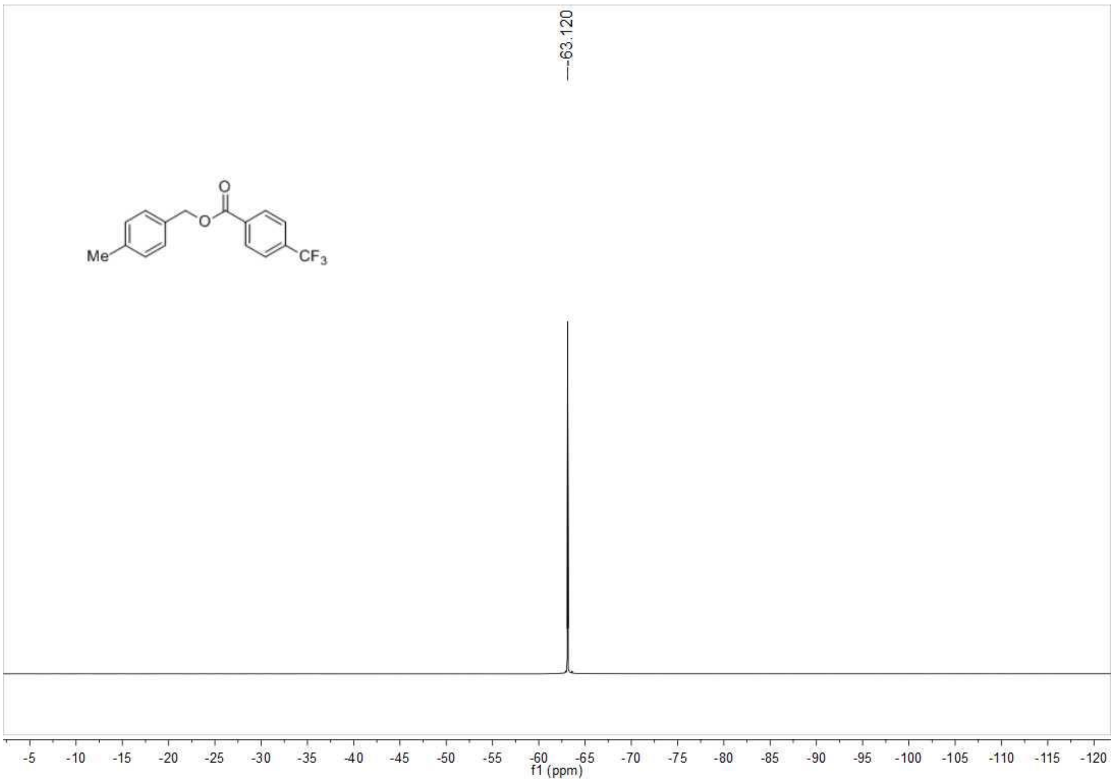

^{1}H NMR Spectrum of **164**

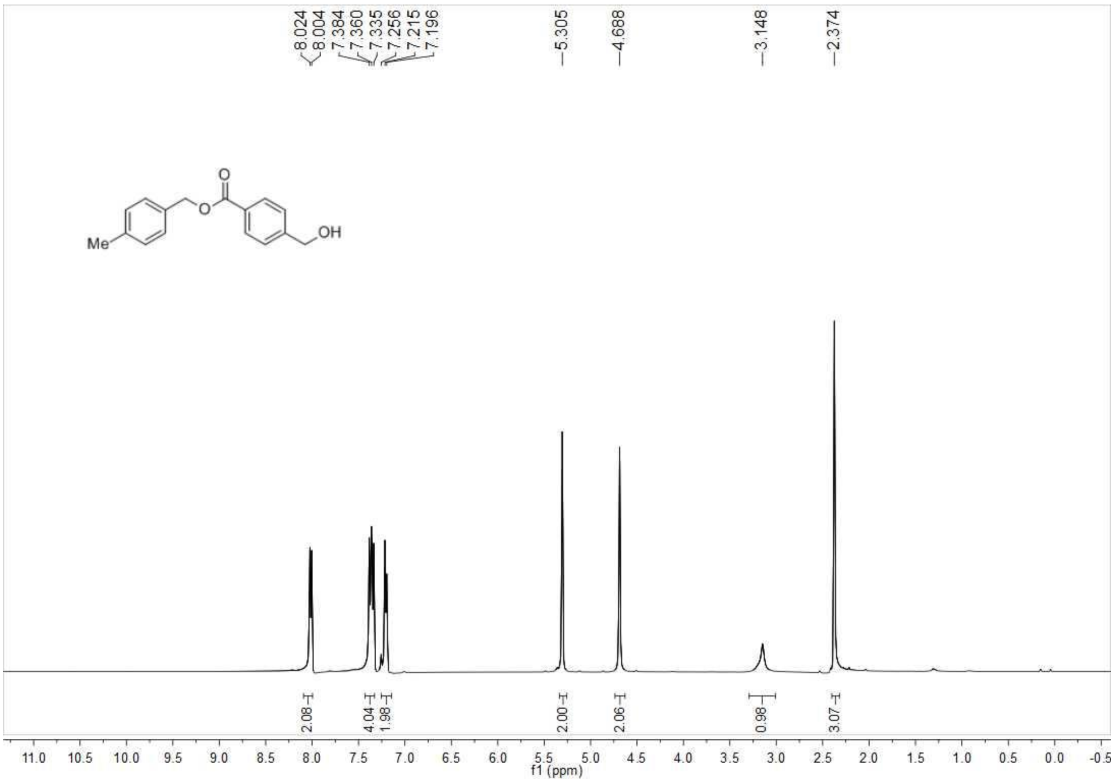

^{13}C NMR Spectrum of **164**

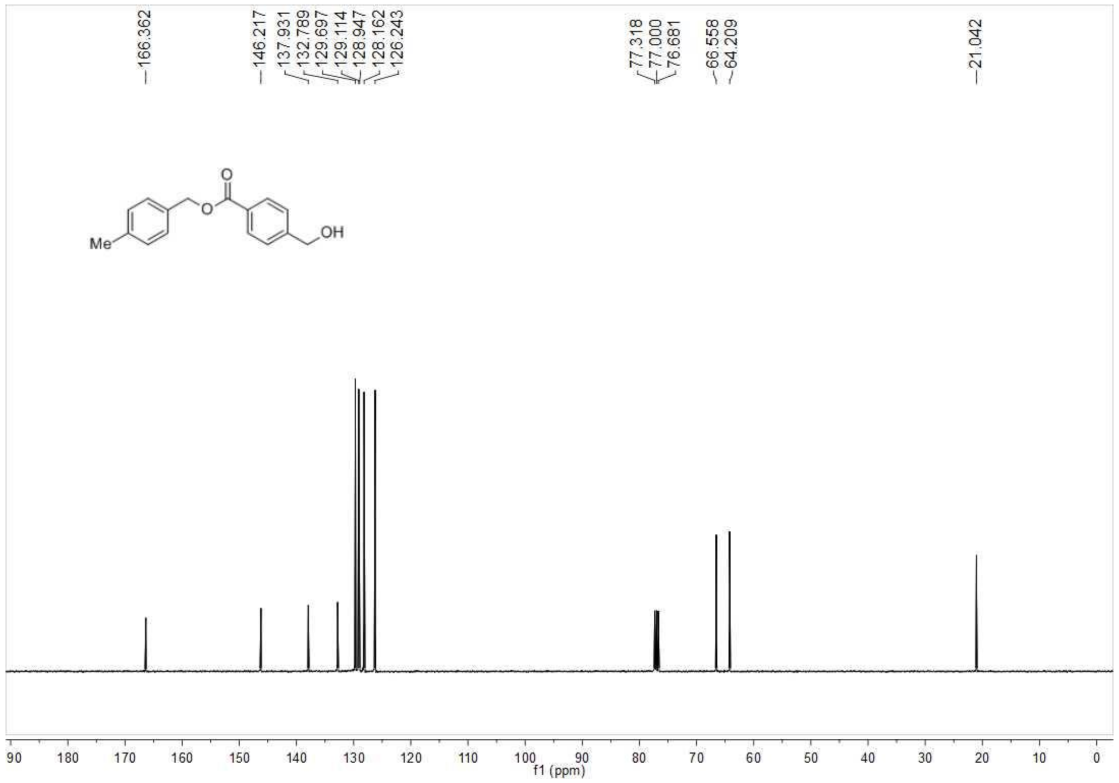

^{1}H NMR Spectrum of **165**

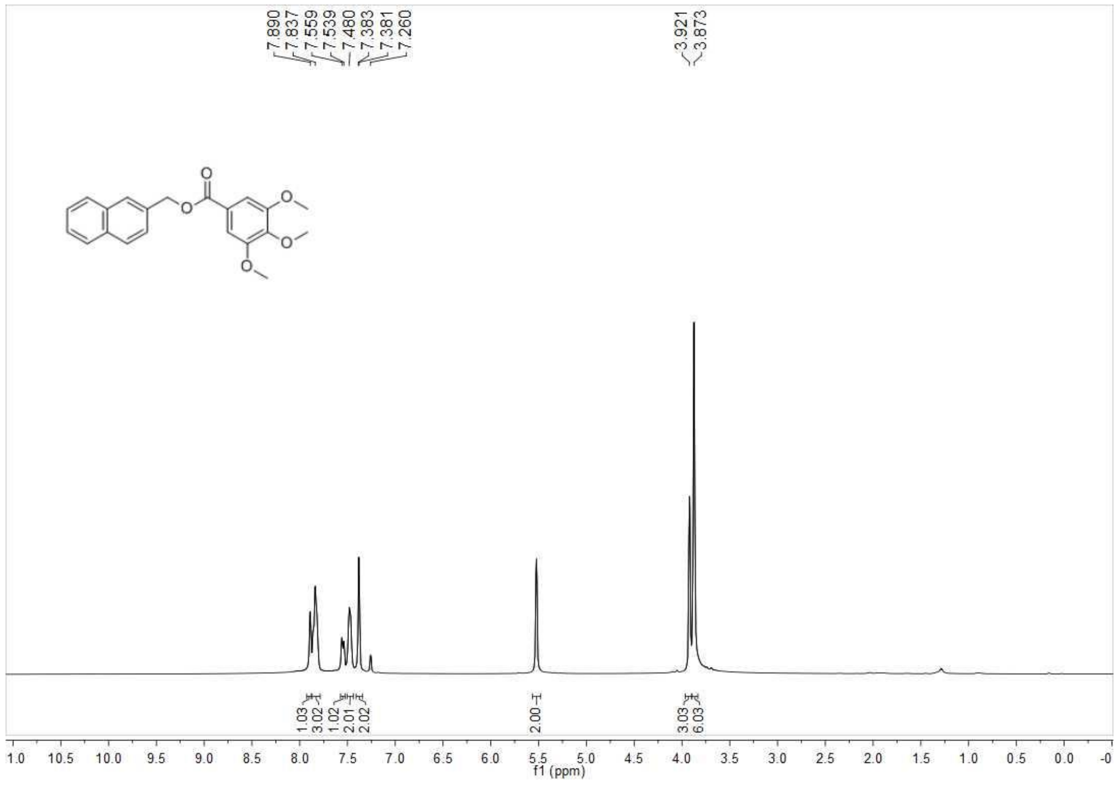

^{13}C NMR Spectrum of **165**

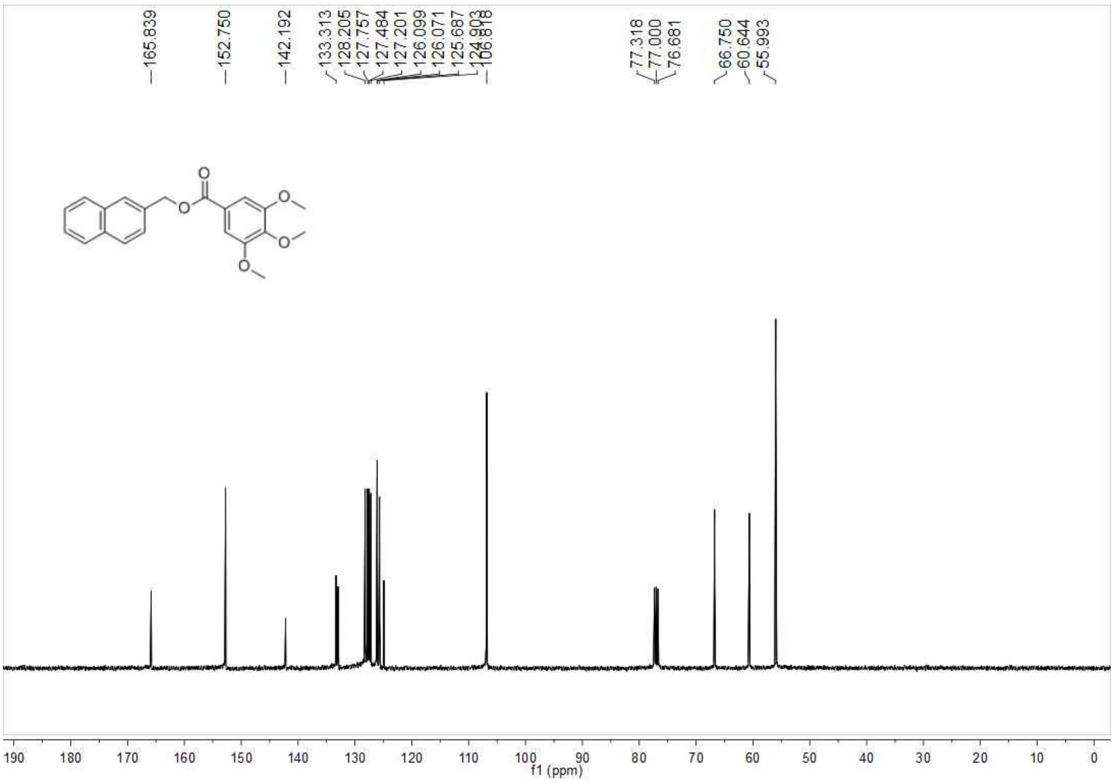

^{1}H NMR Spectrum of **166**

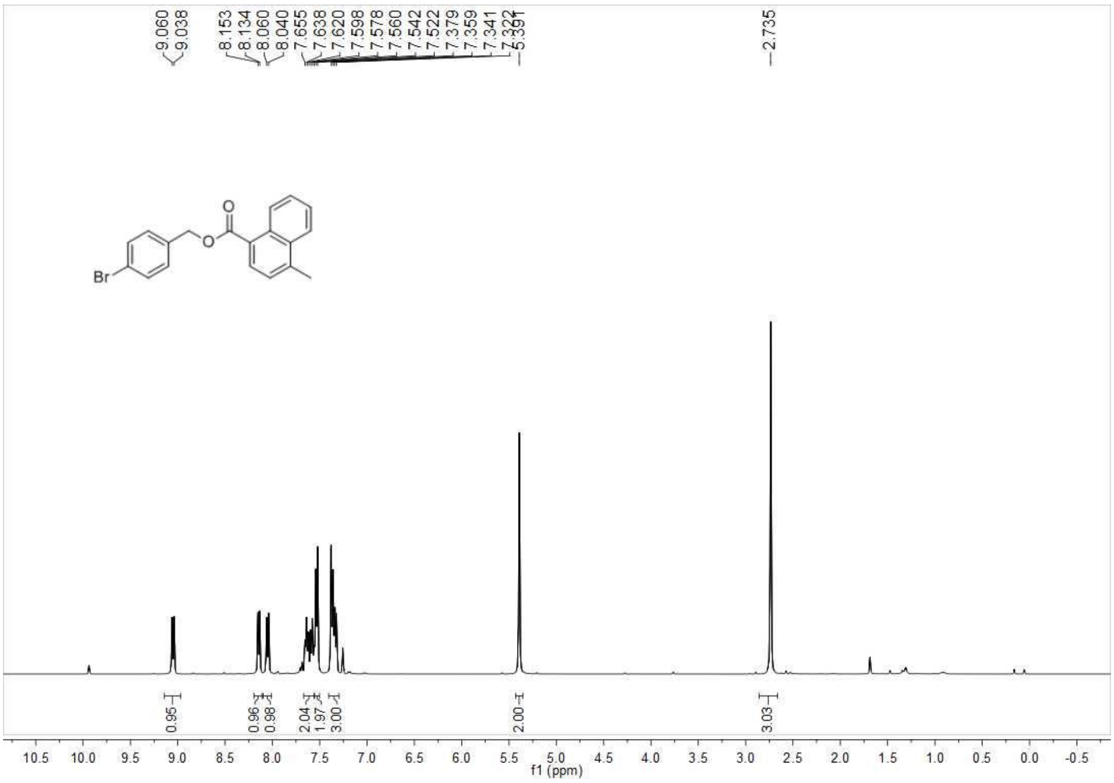

^{13}C NMR Spectrum of **166**

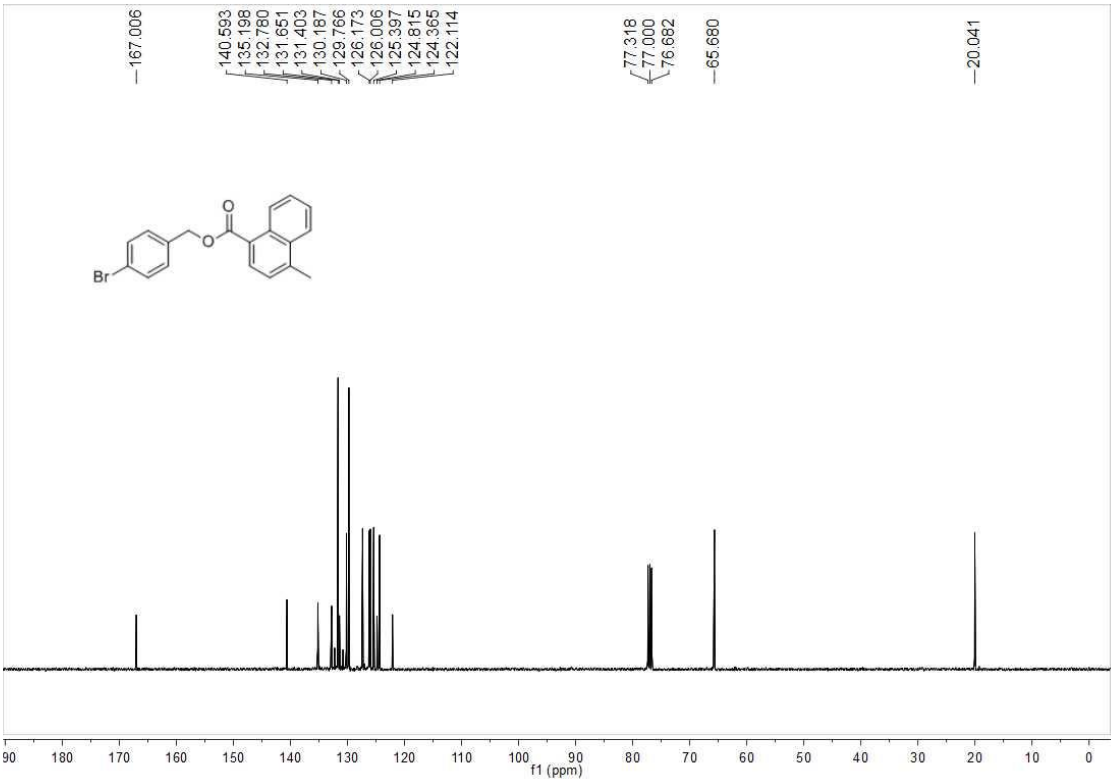

^{1}H NMR Spectrum of **167**

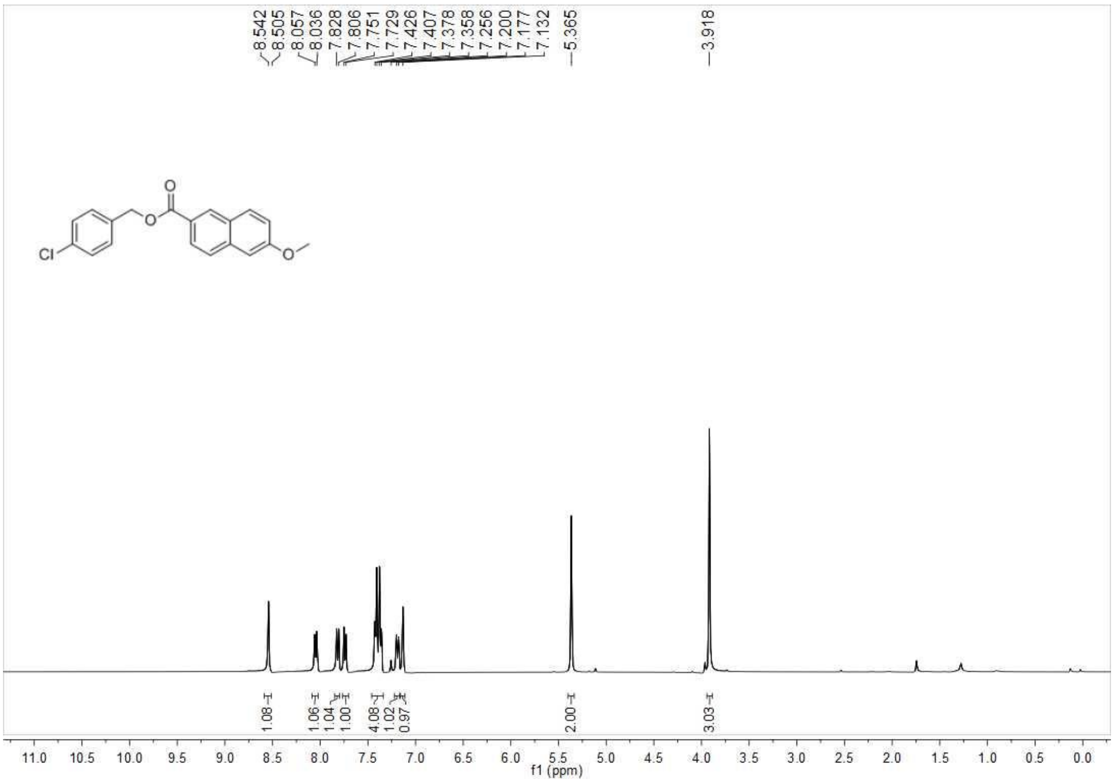

^{13}C NMR Spectrum of **167**

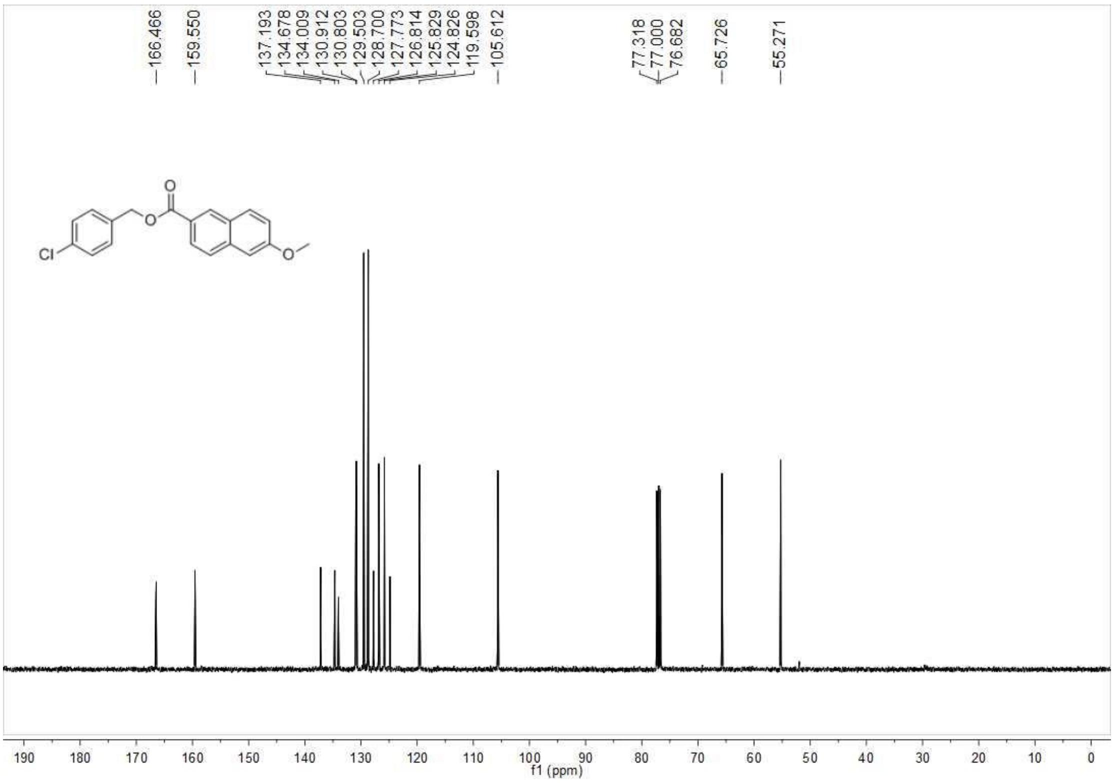

^{1}H NMR Spectrum of **168**

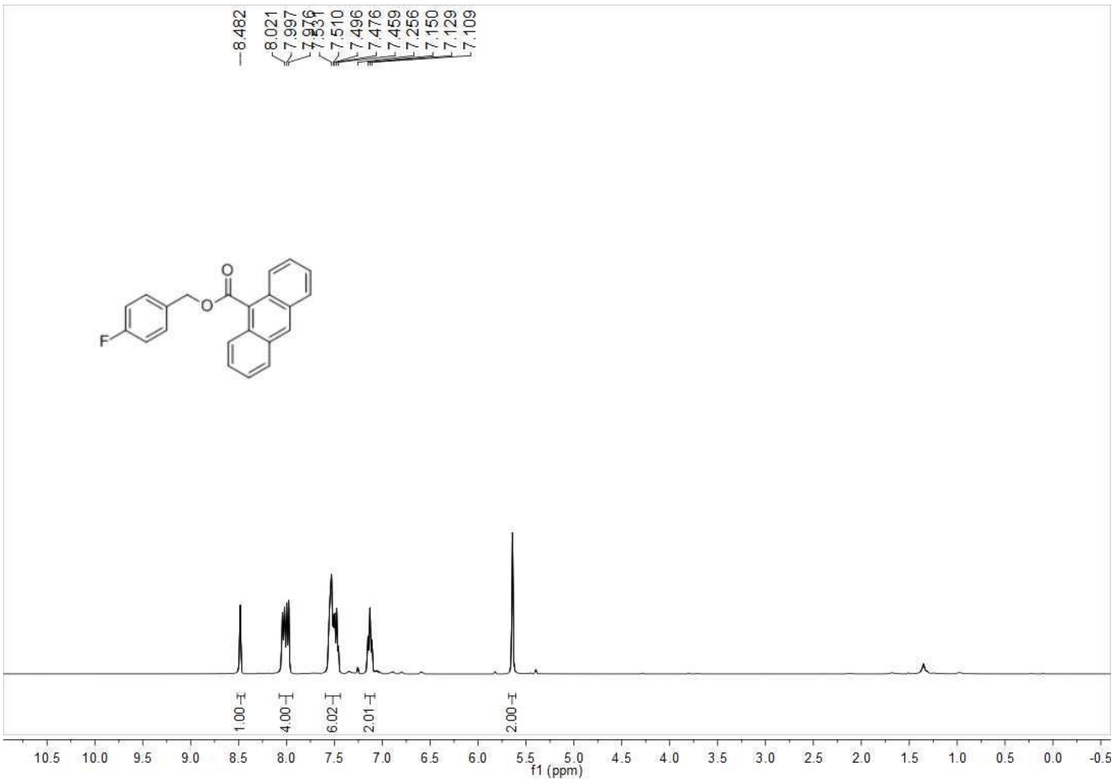

^{13}C NMR Spectrum of **168**

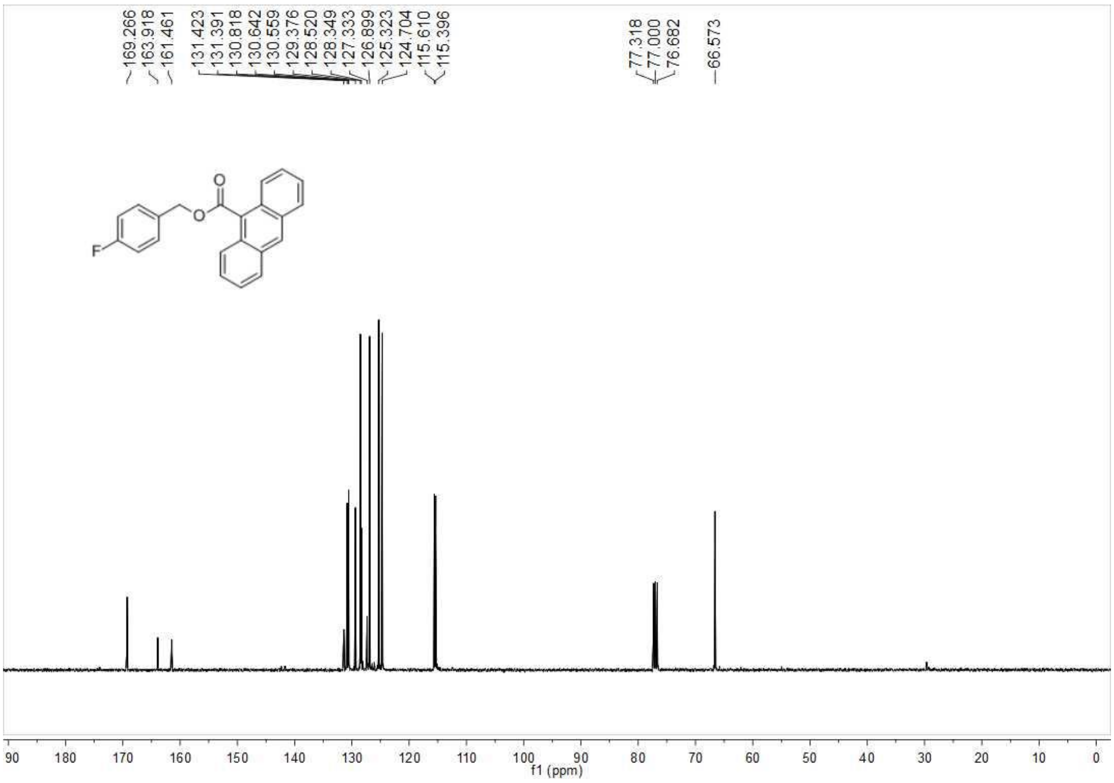

^{19}F NMR Spectrum of **168**

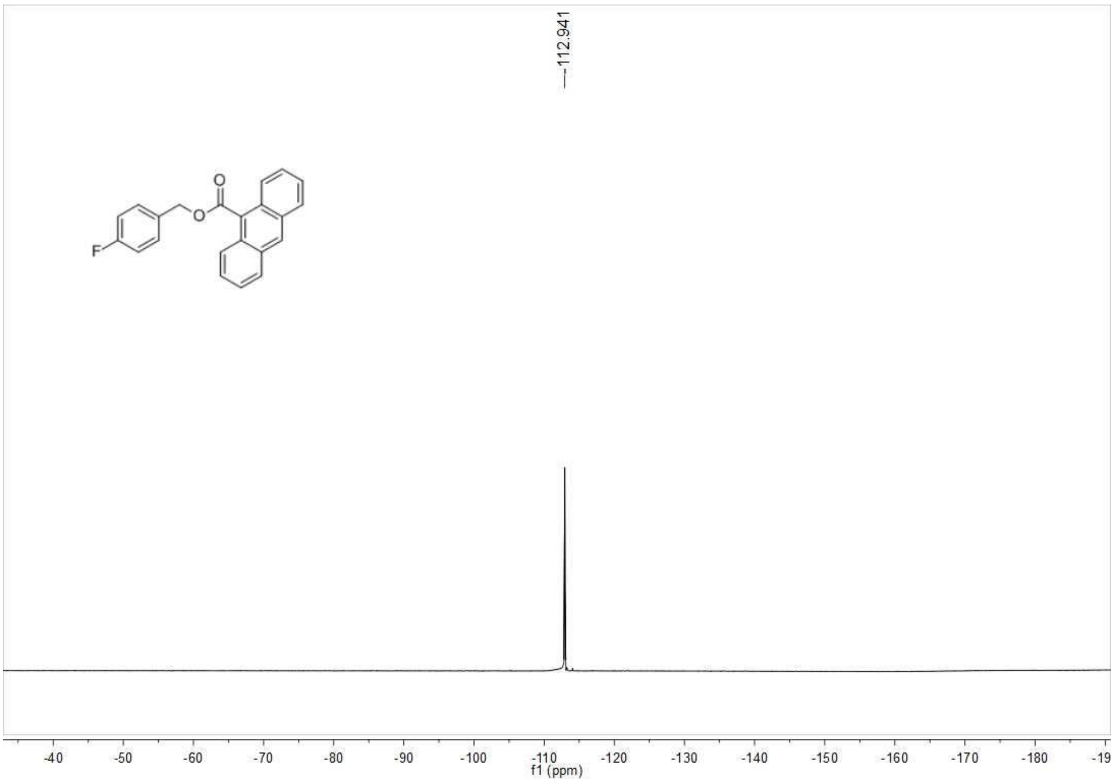

^{1}H NMR Spectrum of **169**

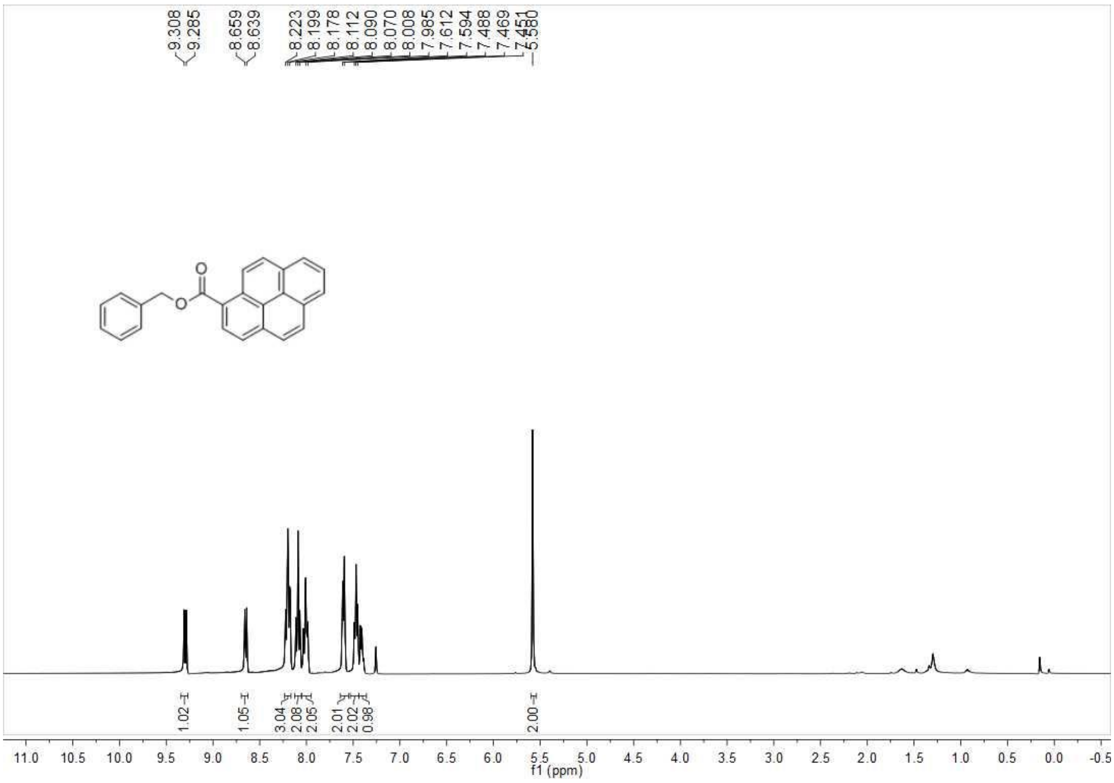

^{13}C NMR Spectrum of **169**

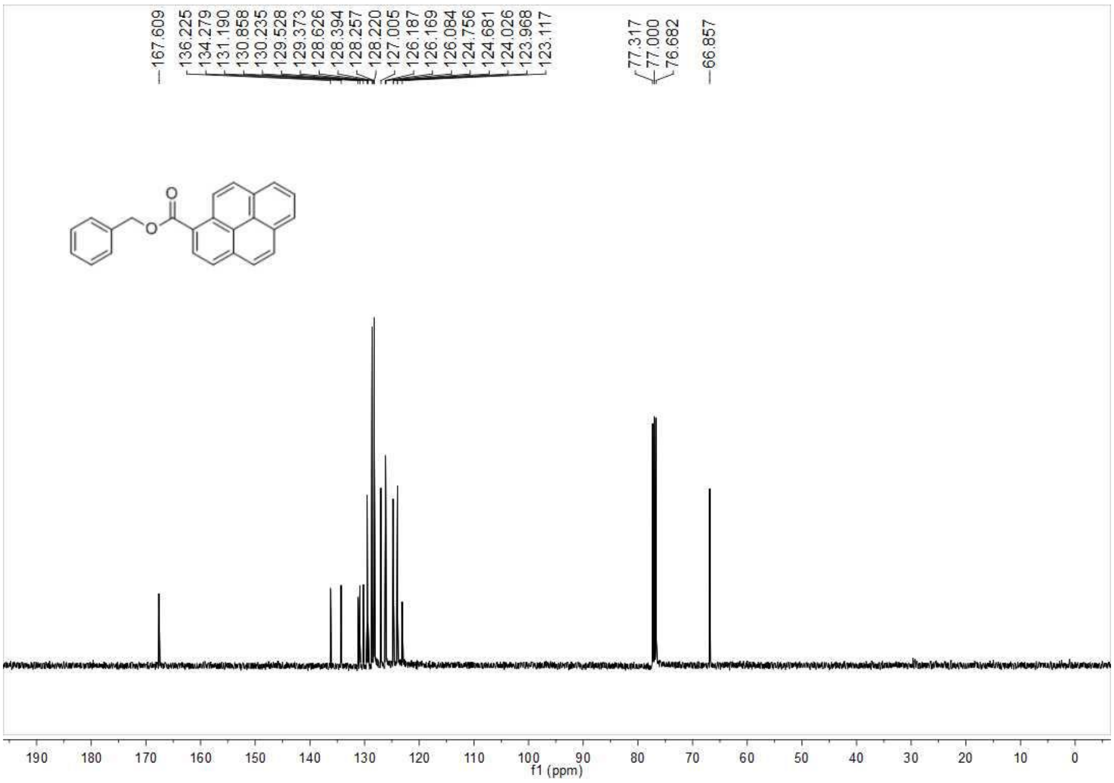

^{1}H NMR Spectrum of **170**

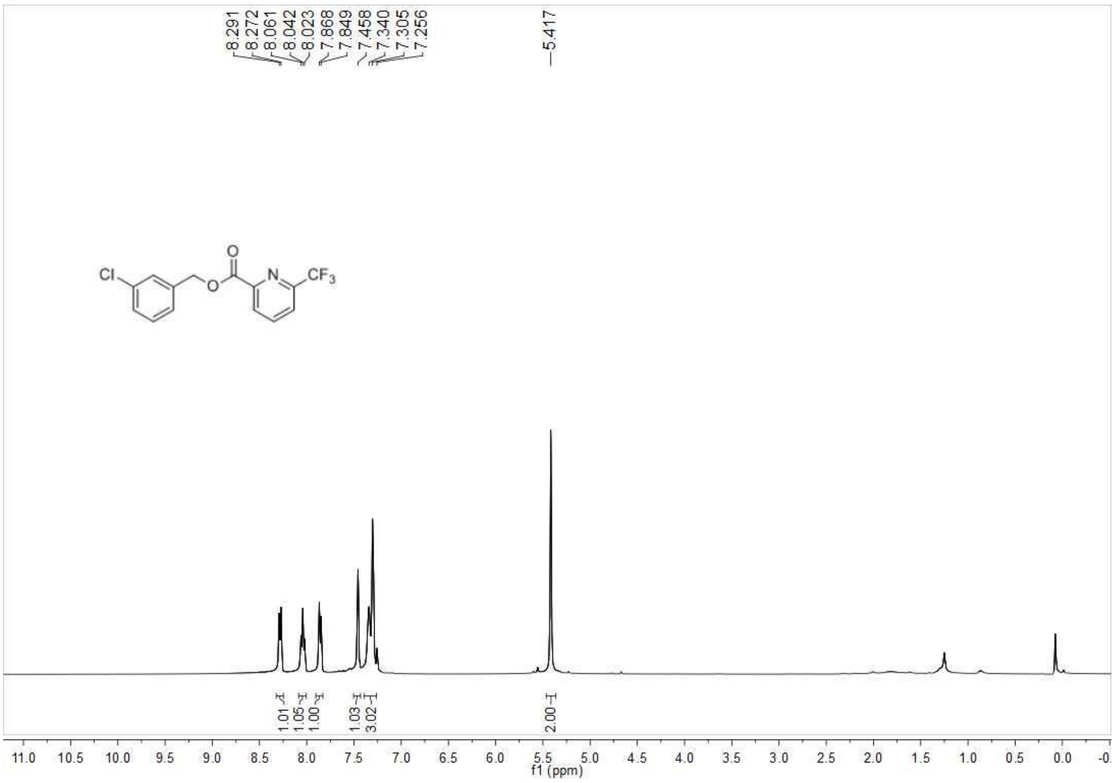

^{13}C NMR Spectrum of **170**

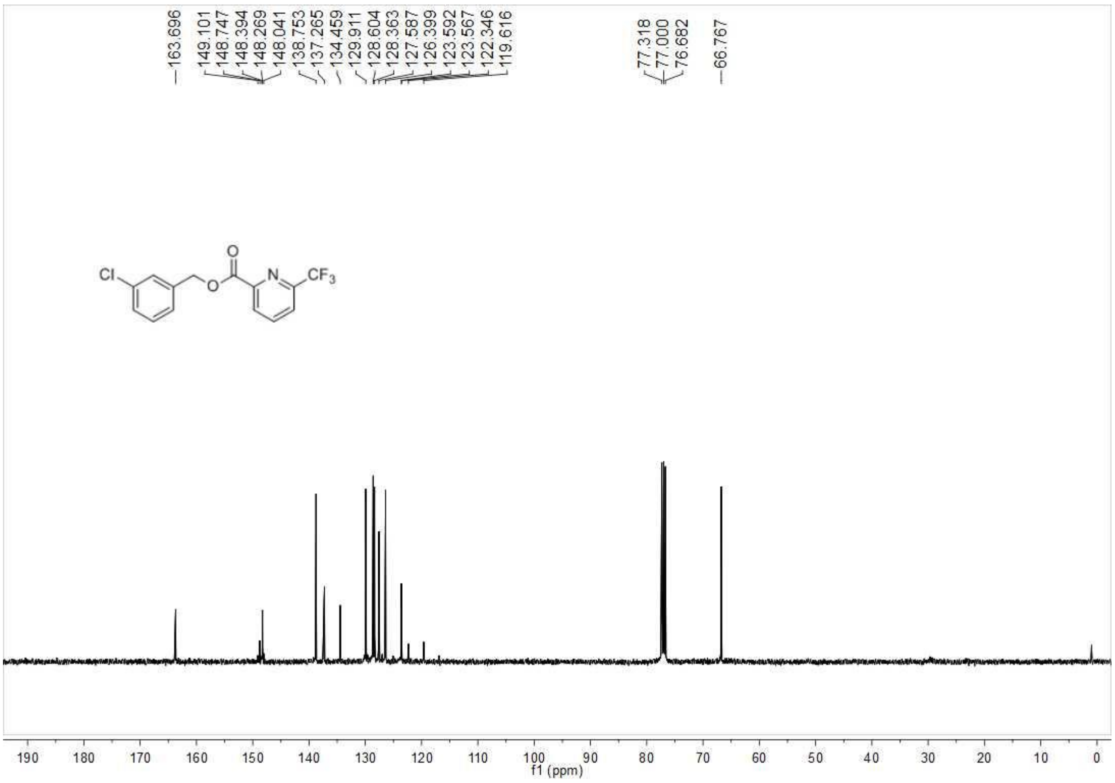

^{19}F NMR Spectrum of **170**

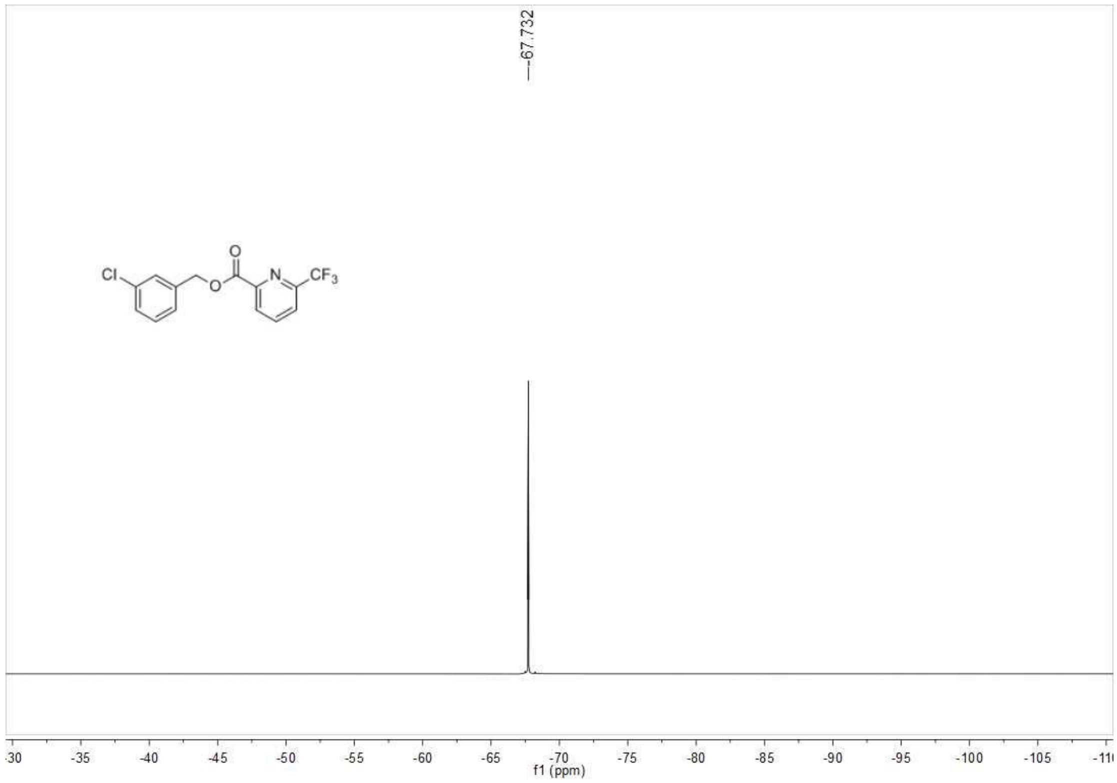

^{1}H NMR Spectrum of **171**

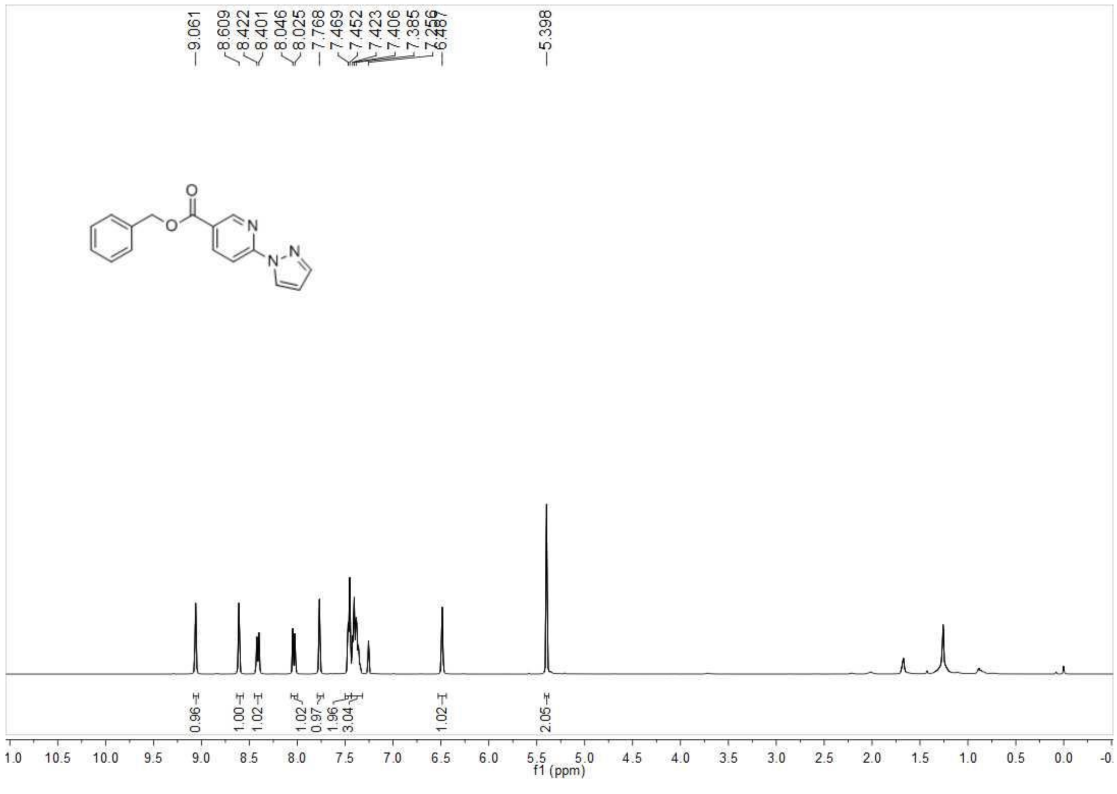

^{13}C NMR Spectrum of **171**

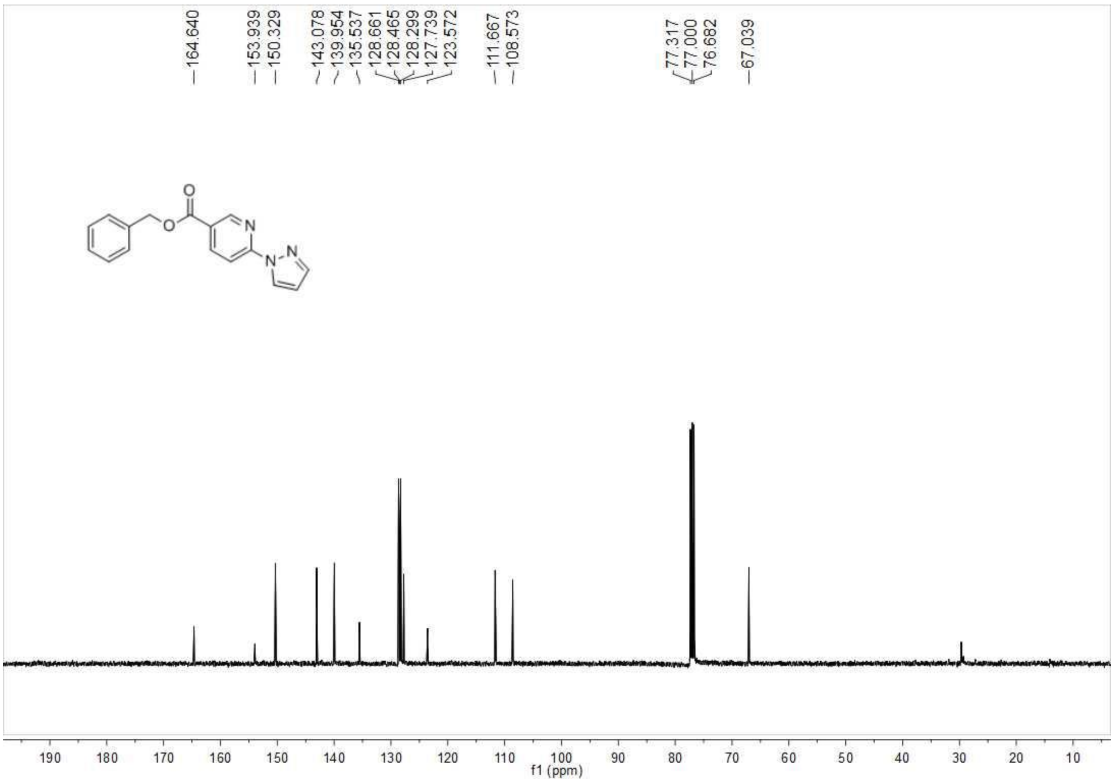

^{1}H NMR Spectrum of **172**

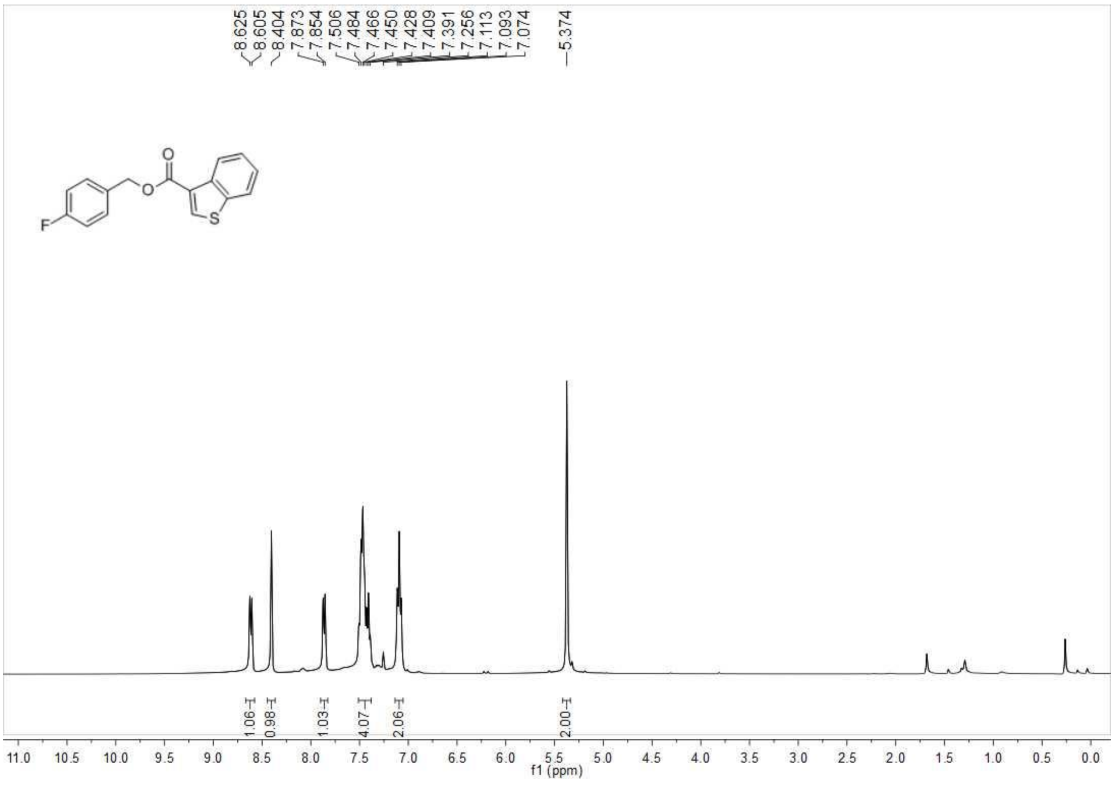

^{13}C NMR Spectrum of **172**

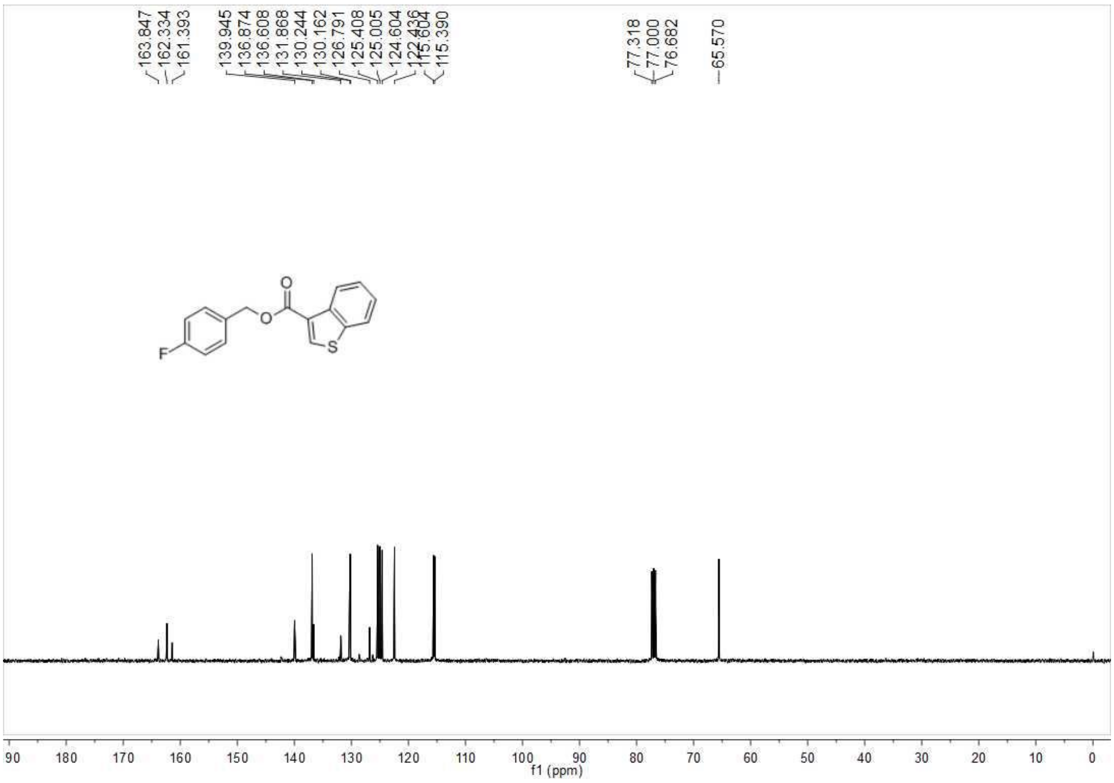

^{19}F NMR Spectrum of **172**

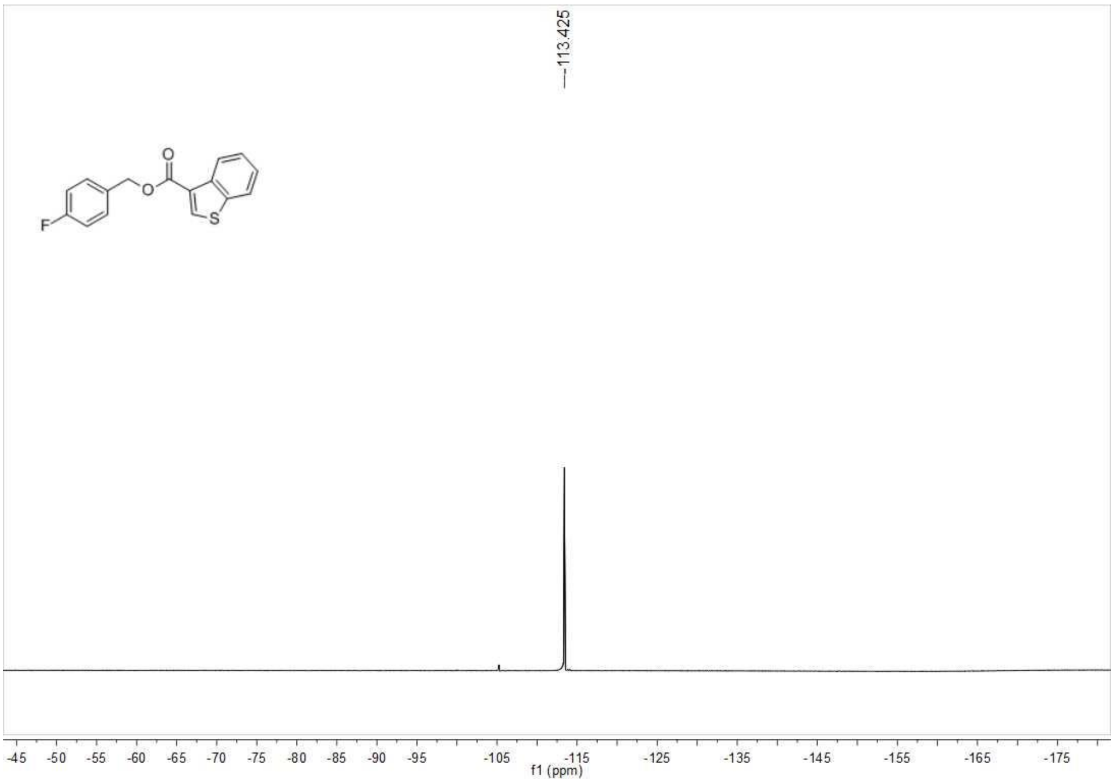

^{1}H NMR Spectrum of **173**

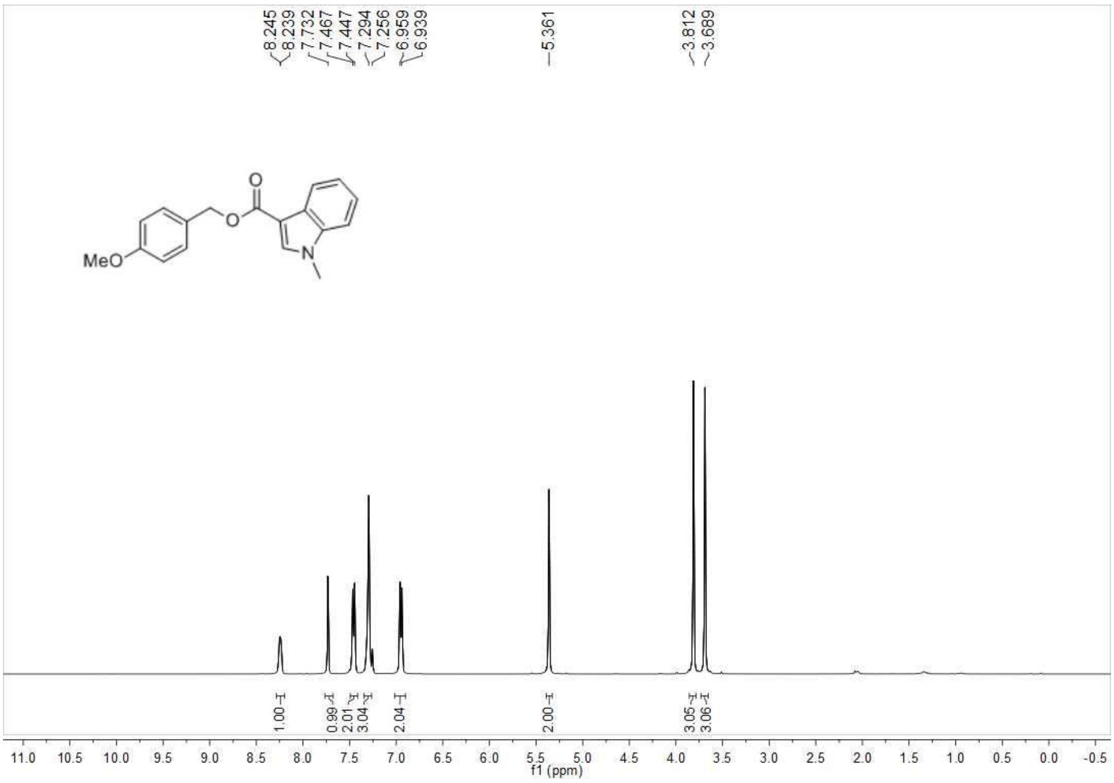

^{13}C NMR Spectrum of **173**

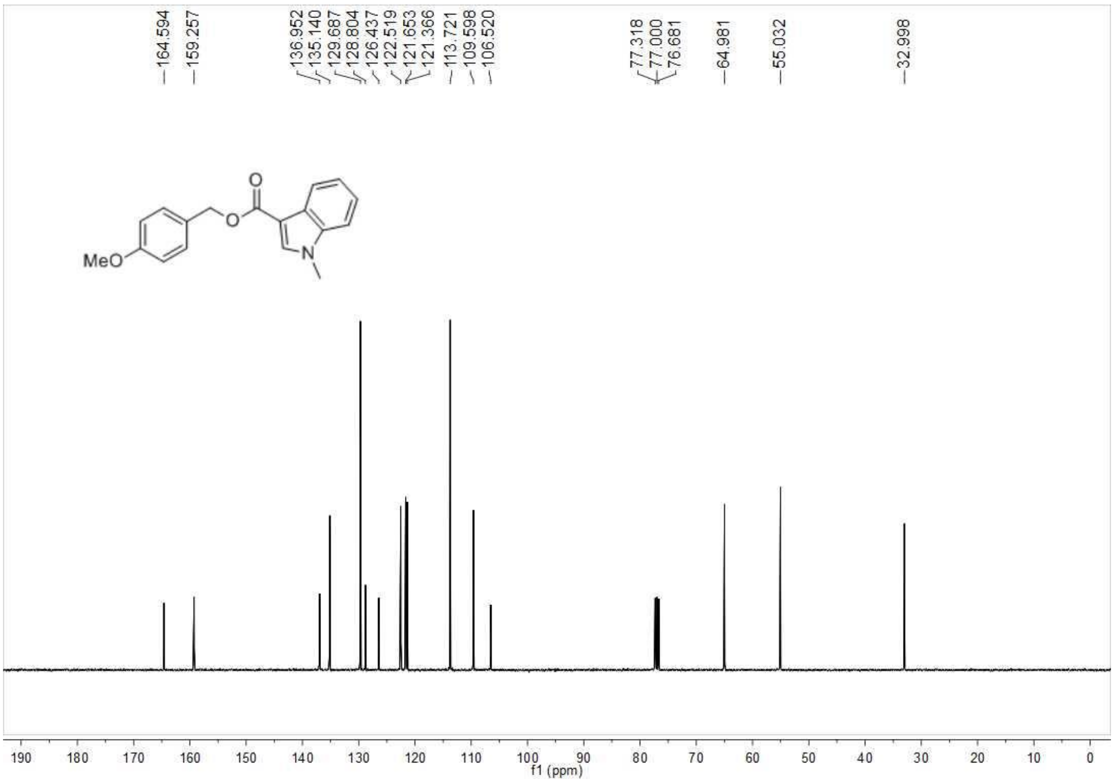

^{1}H NMR Spectrum of **174**

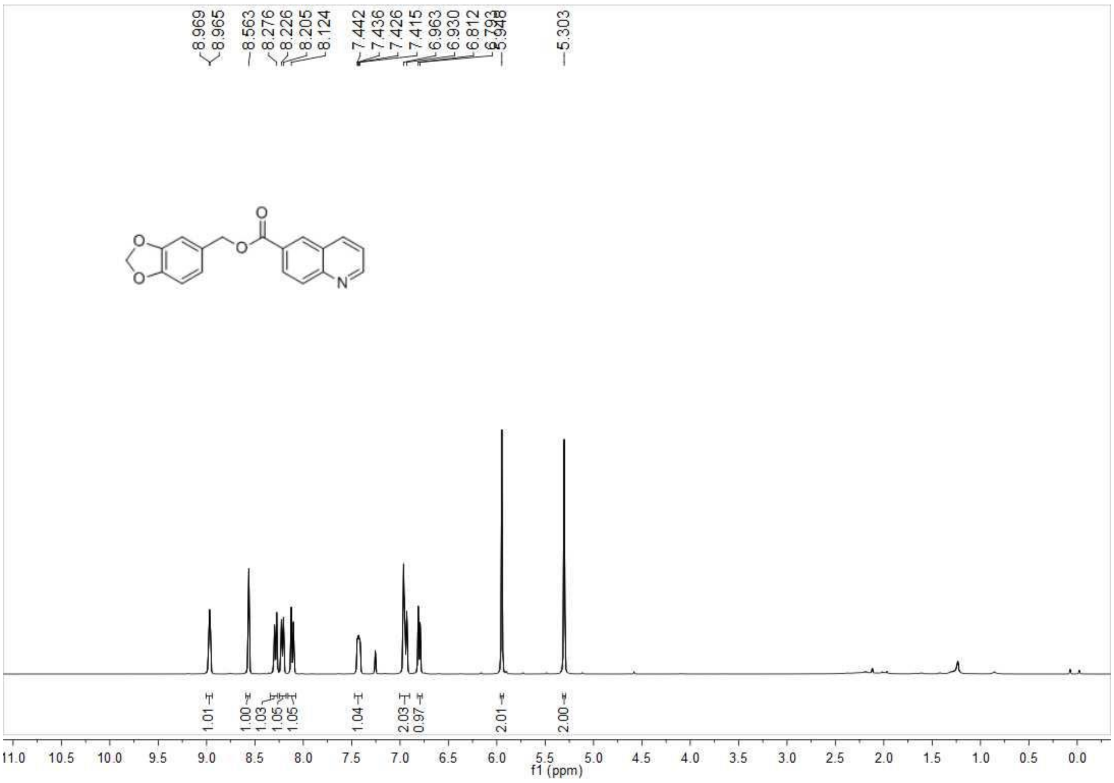

^{13}C NMR Spectrum of **174**

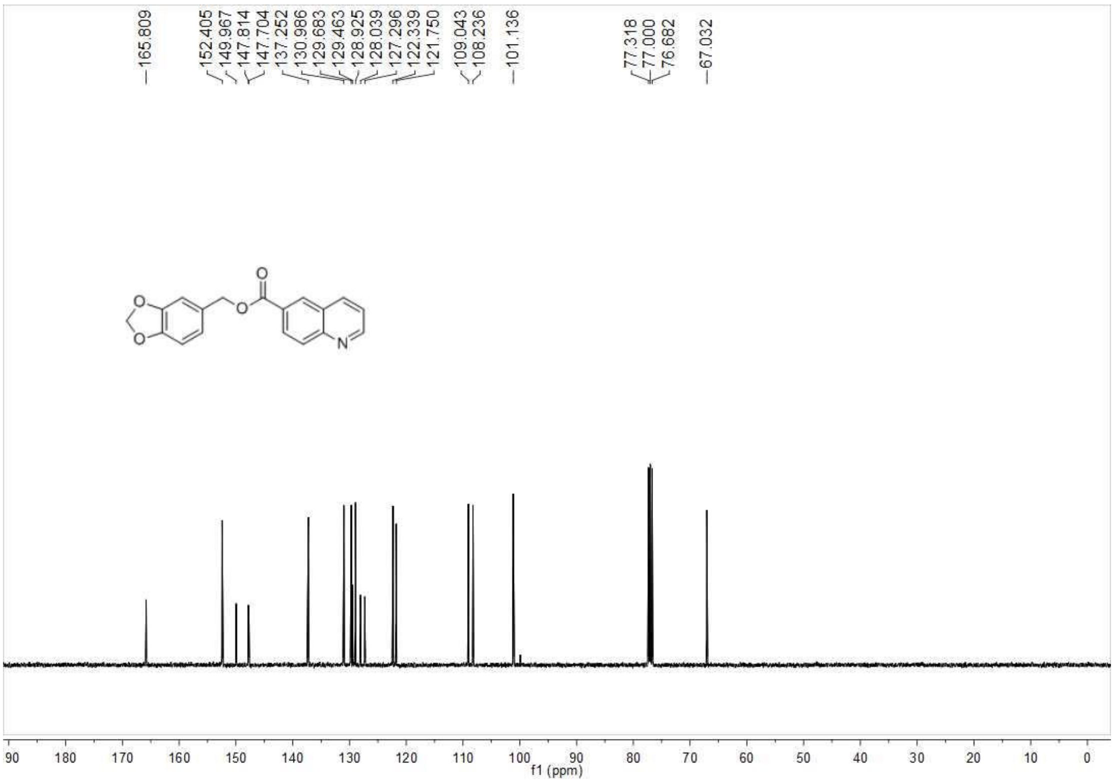

^{1}H NMR Spectrum of **175**

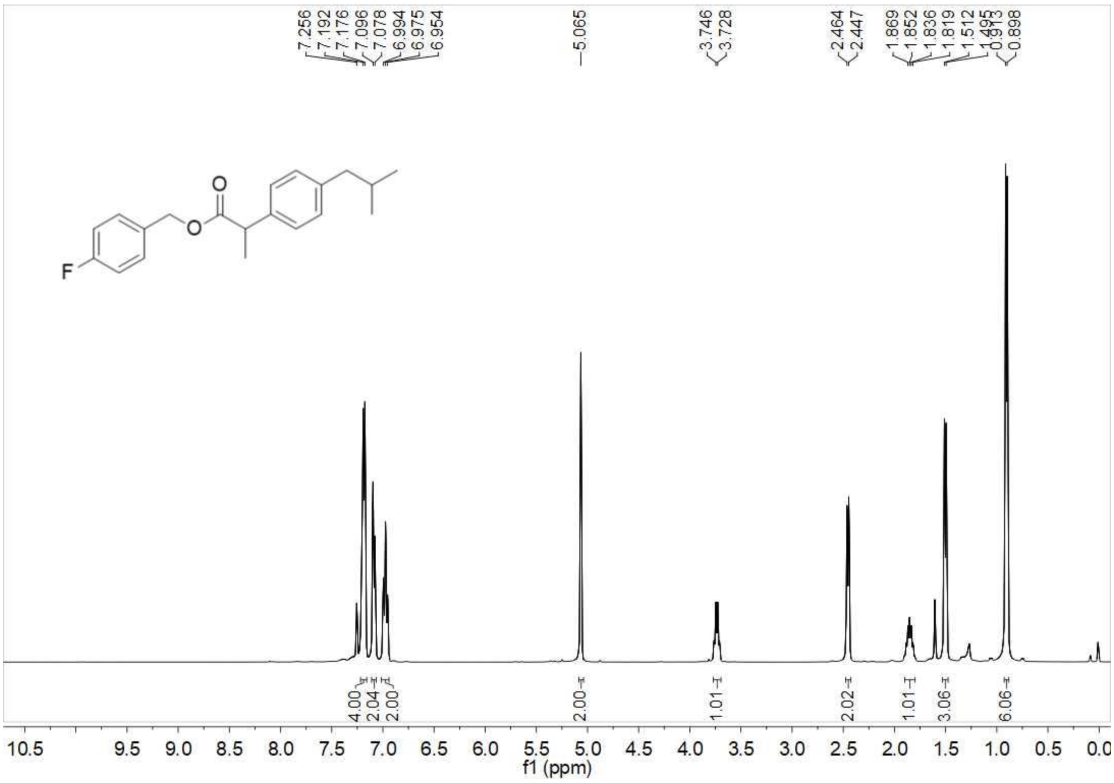

^{13}C NMR Spectrum of **175**

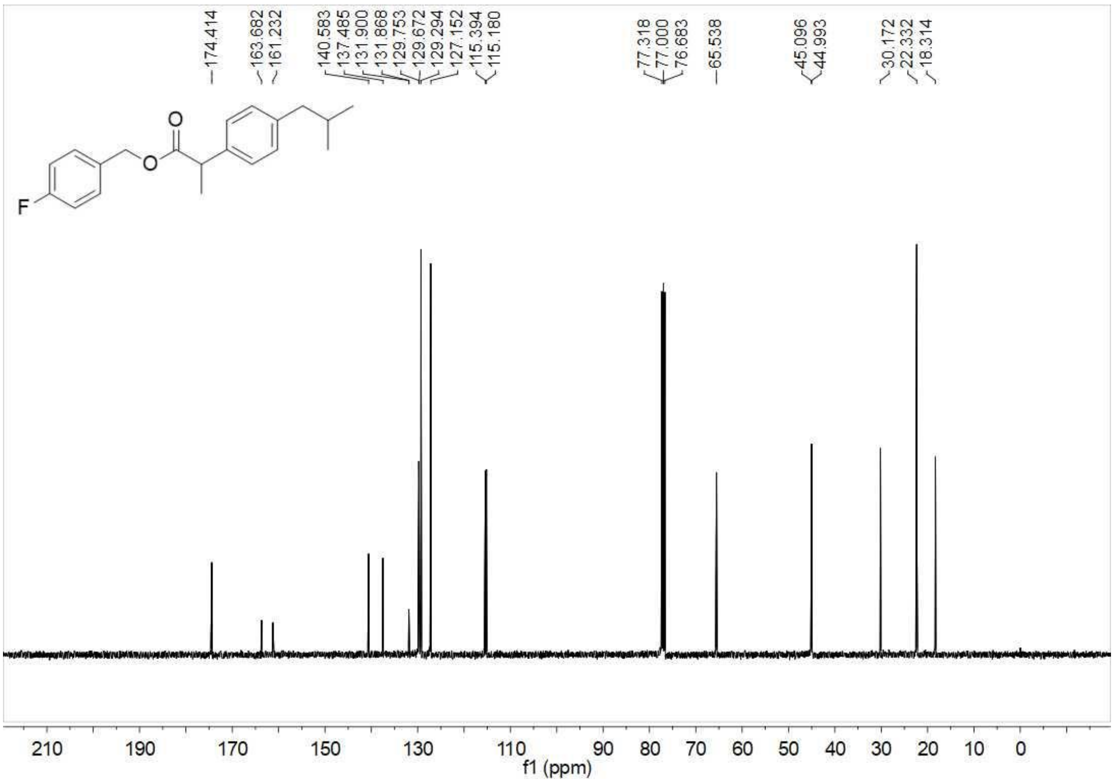

^{19}F NMR Spectrum of **175**

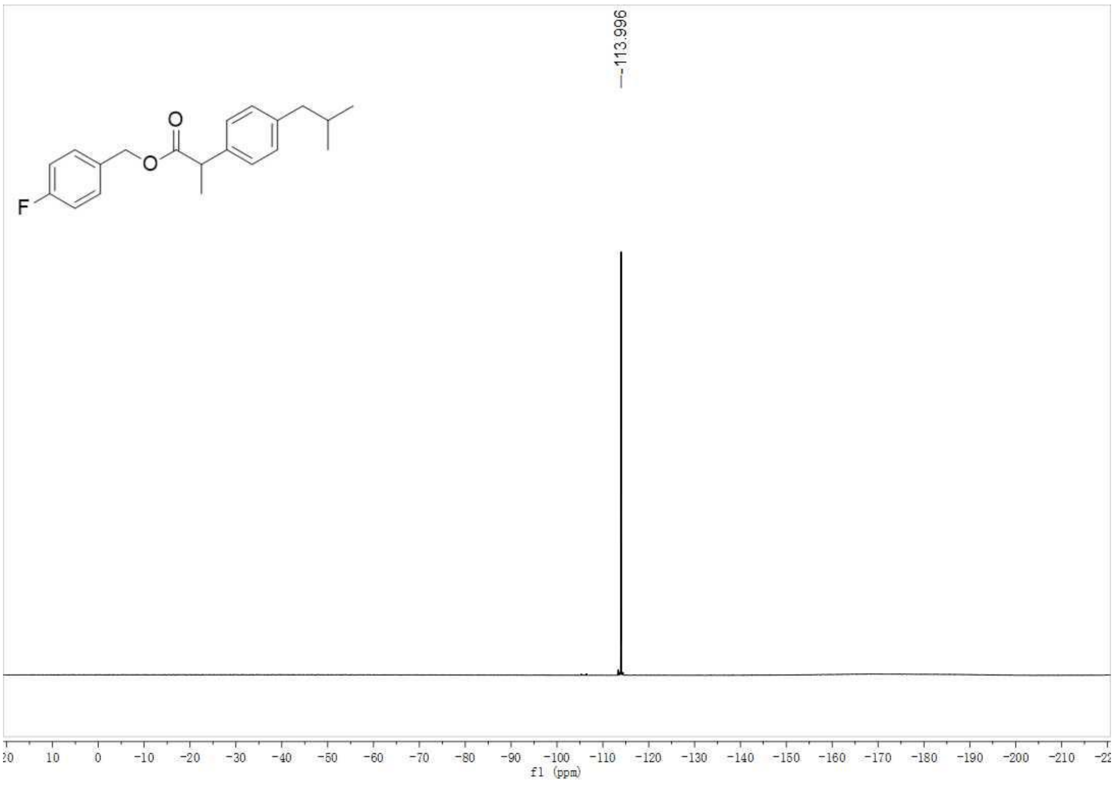

^{1}H NMR Spectrum of **176**

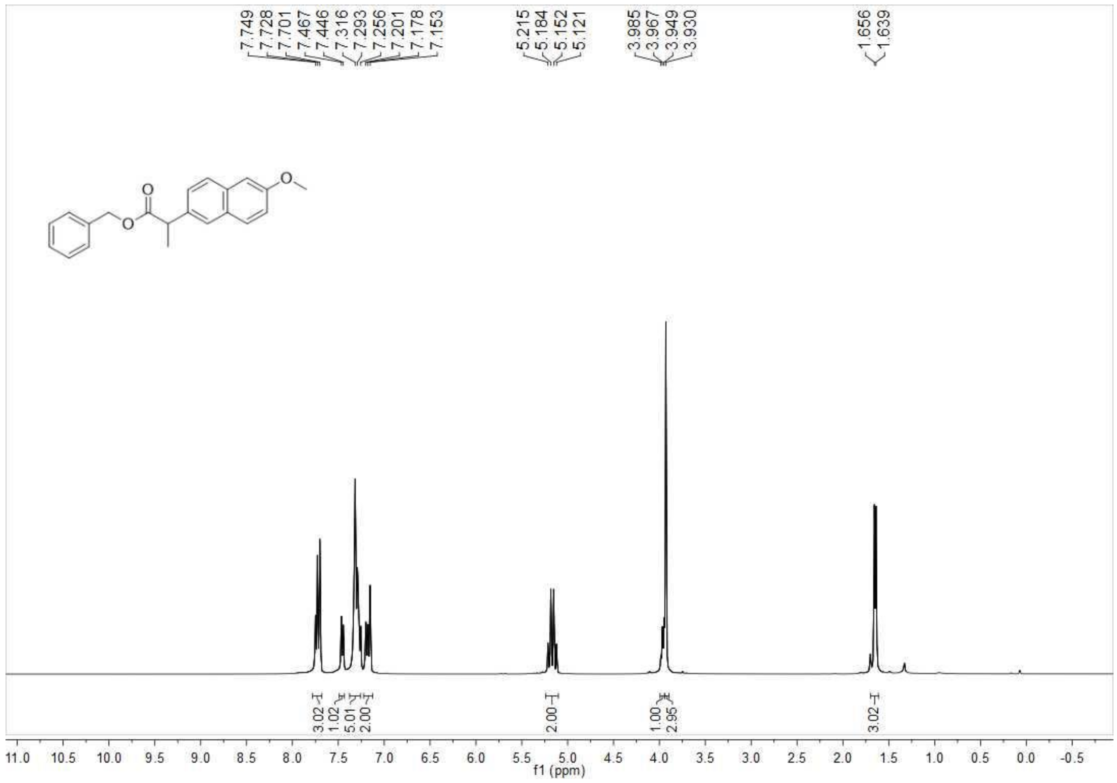

^{13}C NMR Spectrum of **176**

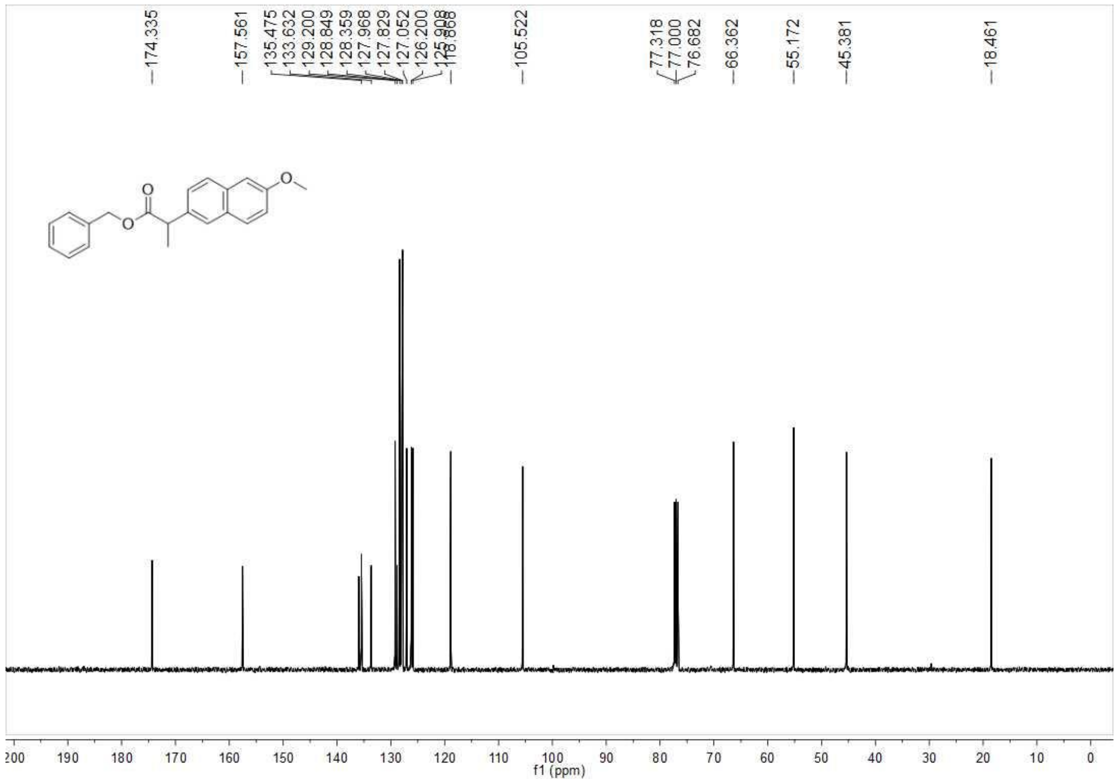

^{1}H NMR Spectrum of **177**

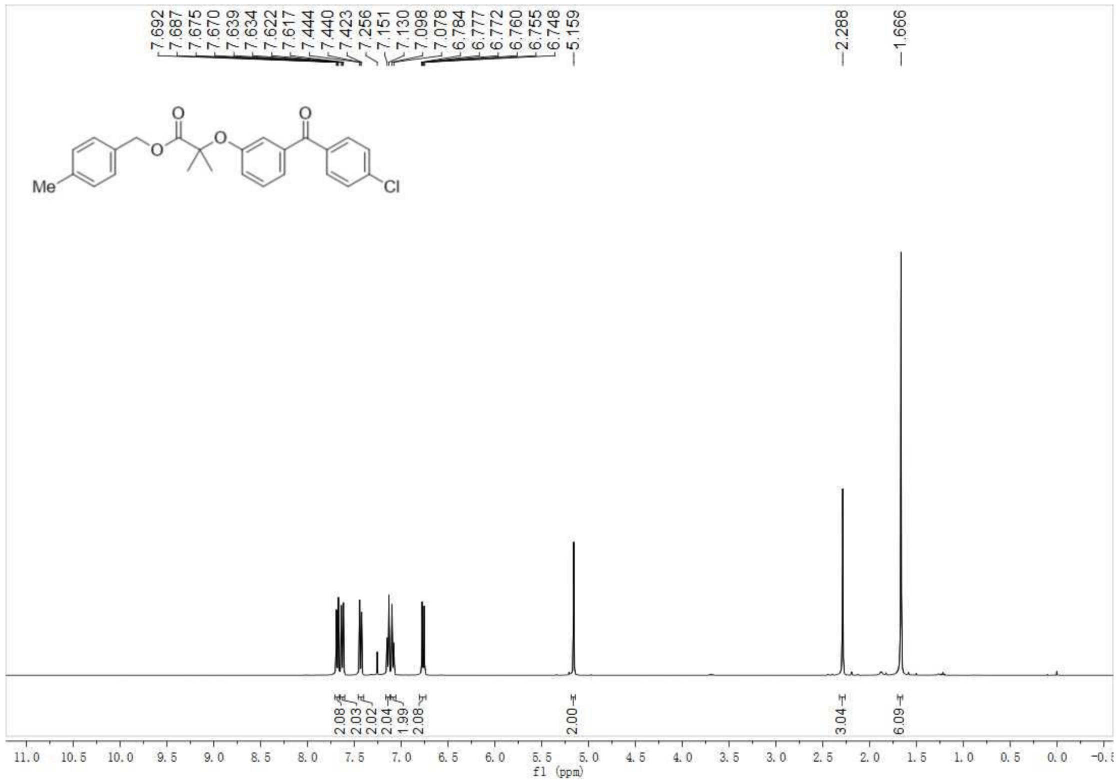

^{13}C NMR Spectrum of **177**

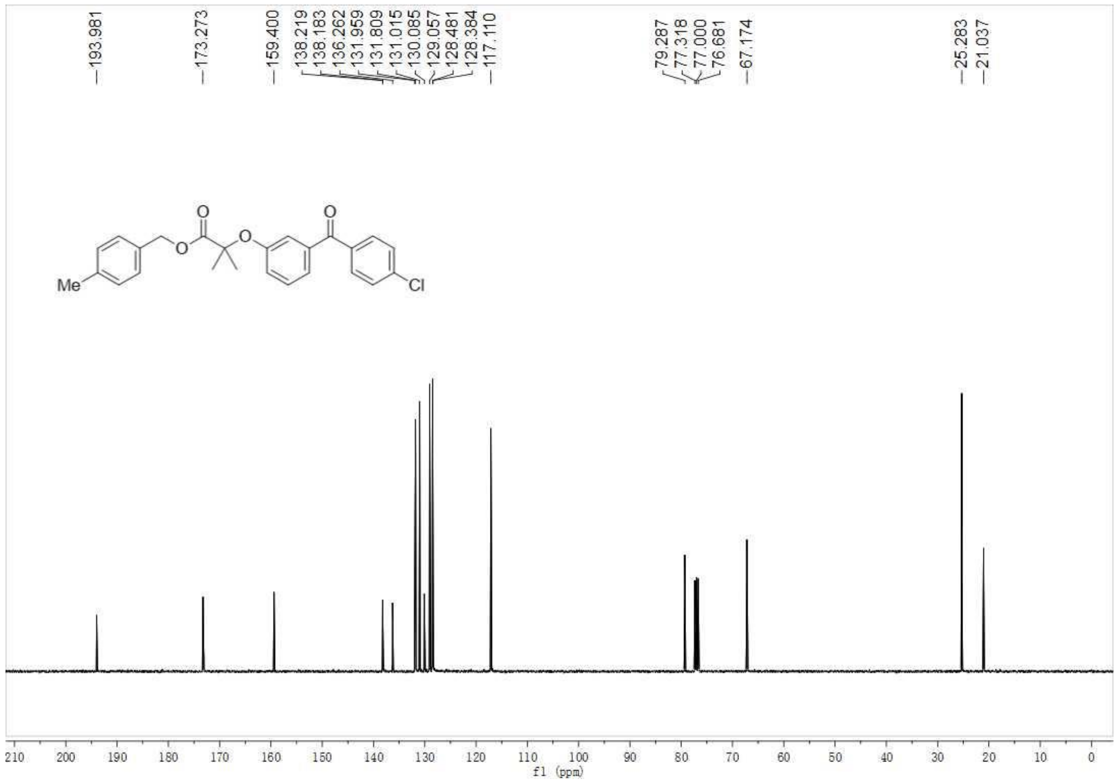

^{1}H NMR Spectrum of **178**

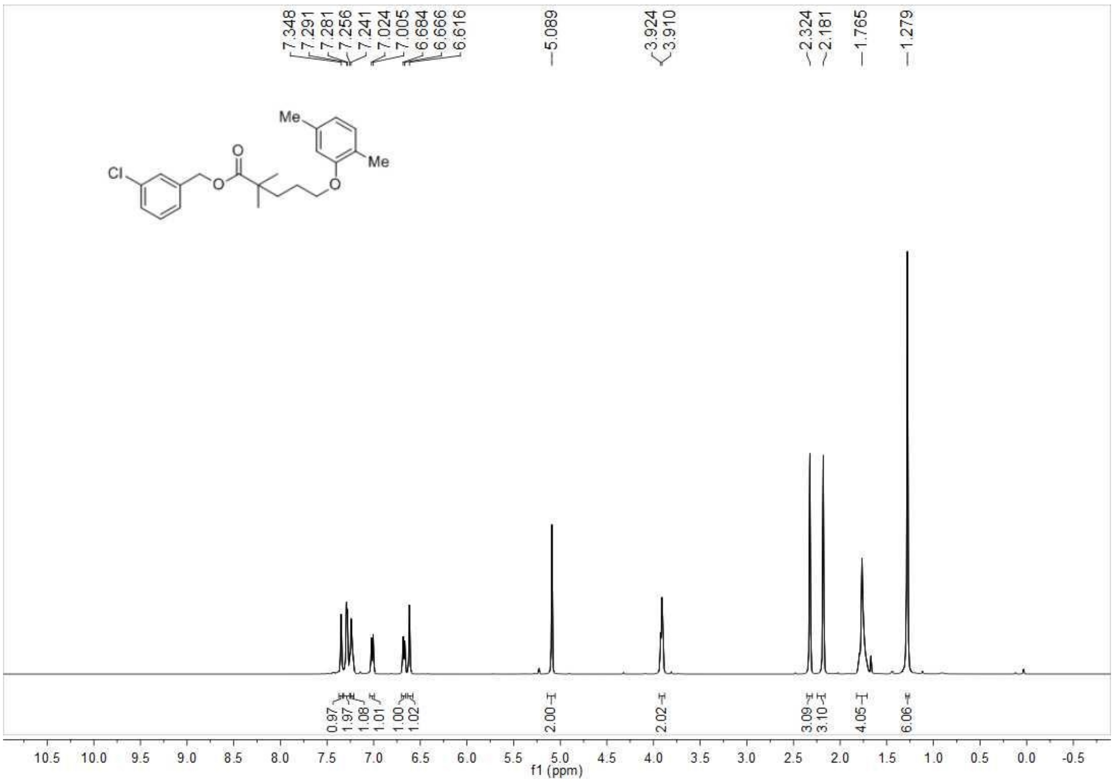

^{13}C NMR Spectrum of **178**

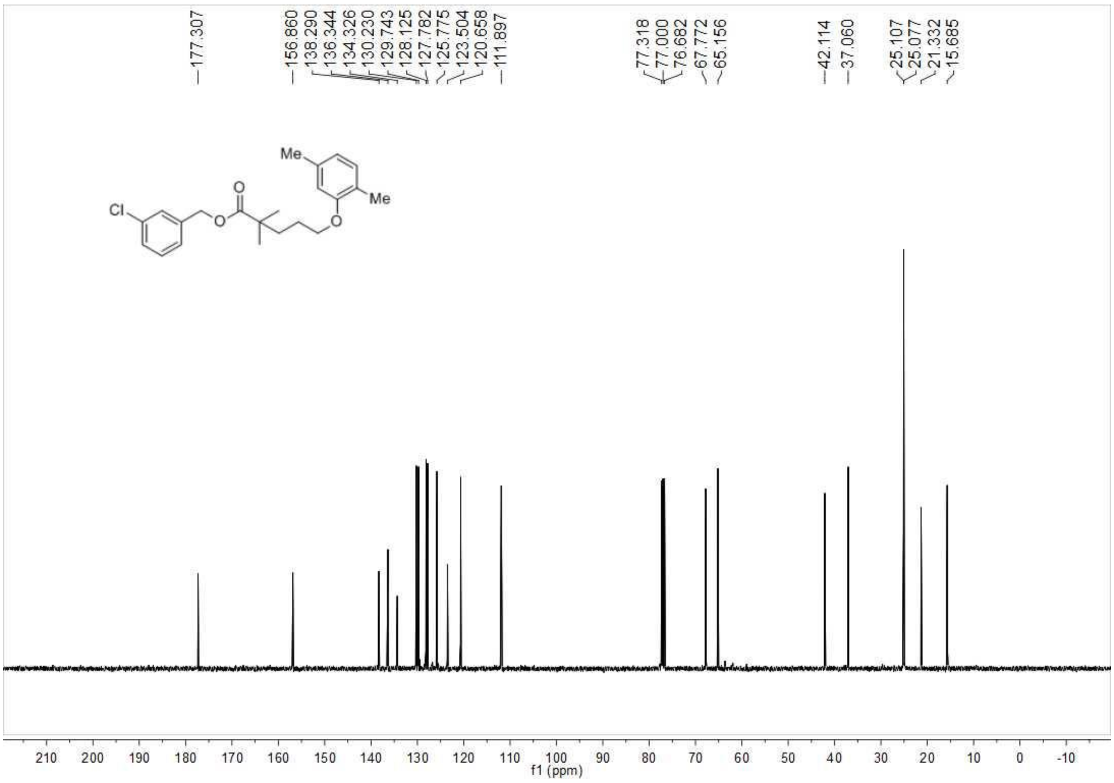

^{1}H NMR Spectrum of **179**

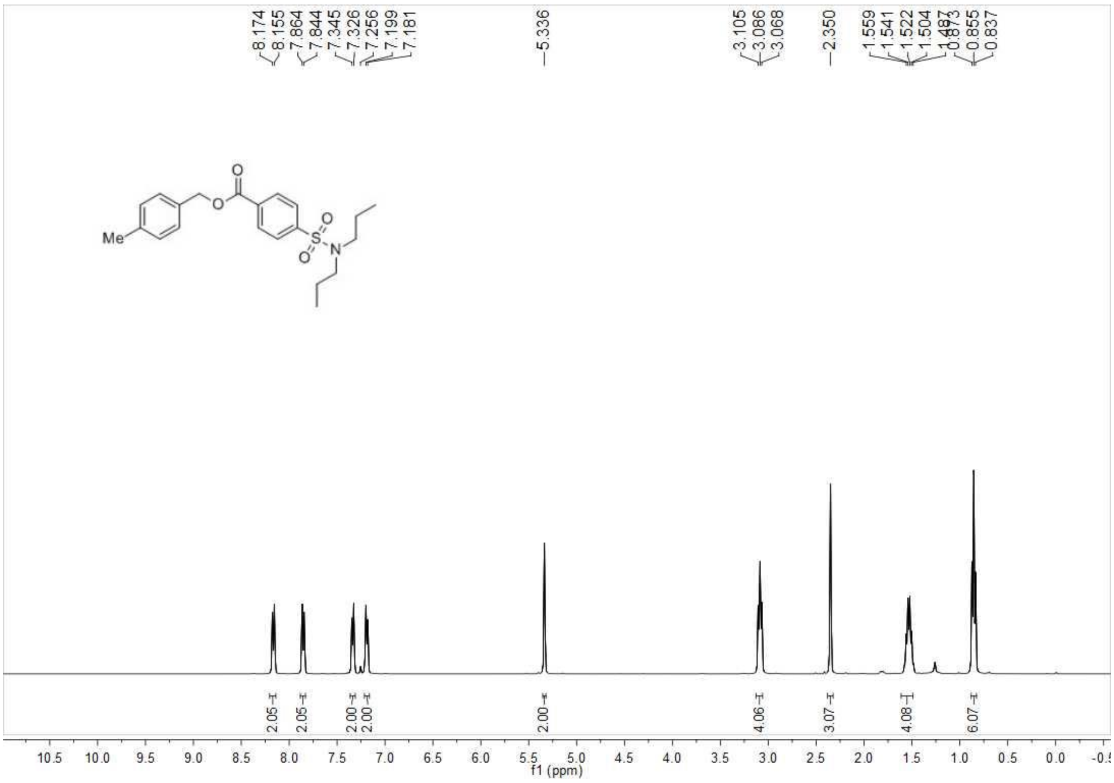

^{13}C NMR Spectrum of **179**

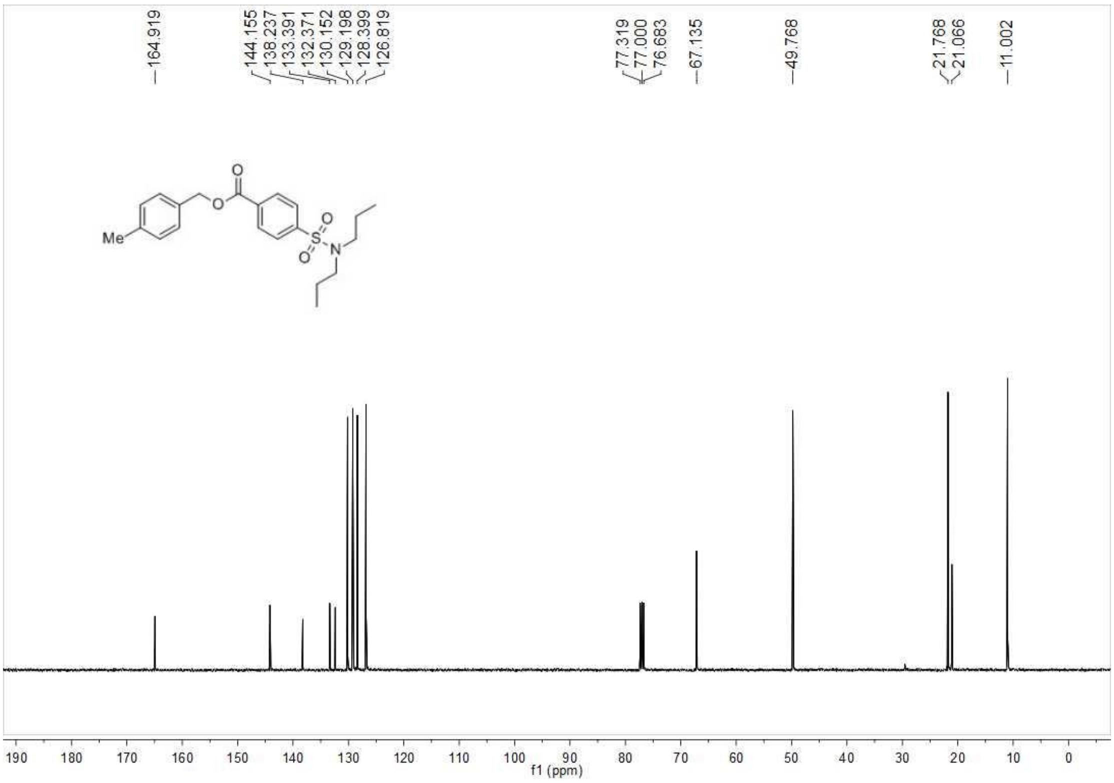

^{1}H NMR Spectrum of **180**

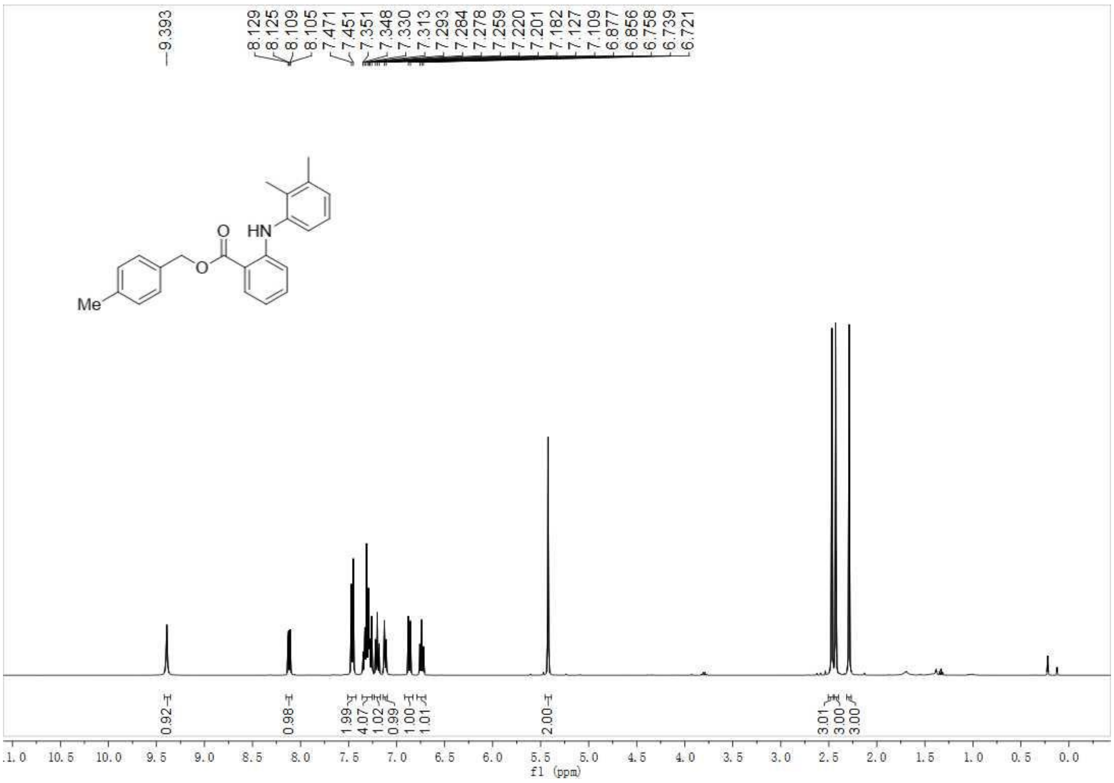

^{13}C NMR Spectrum of **180**

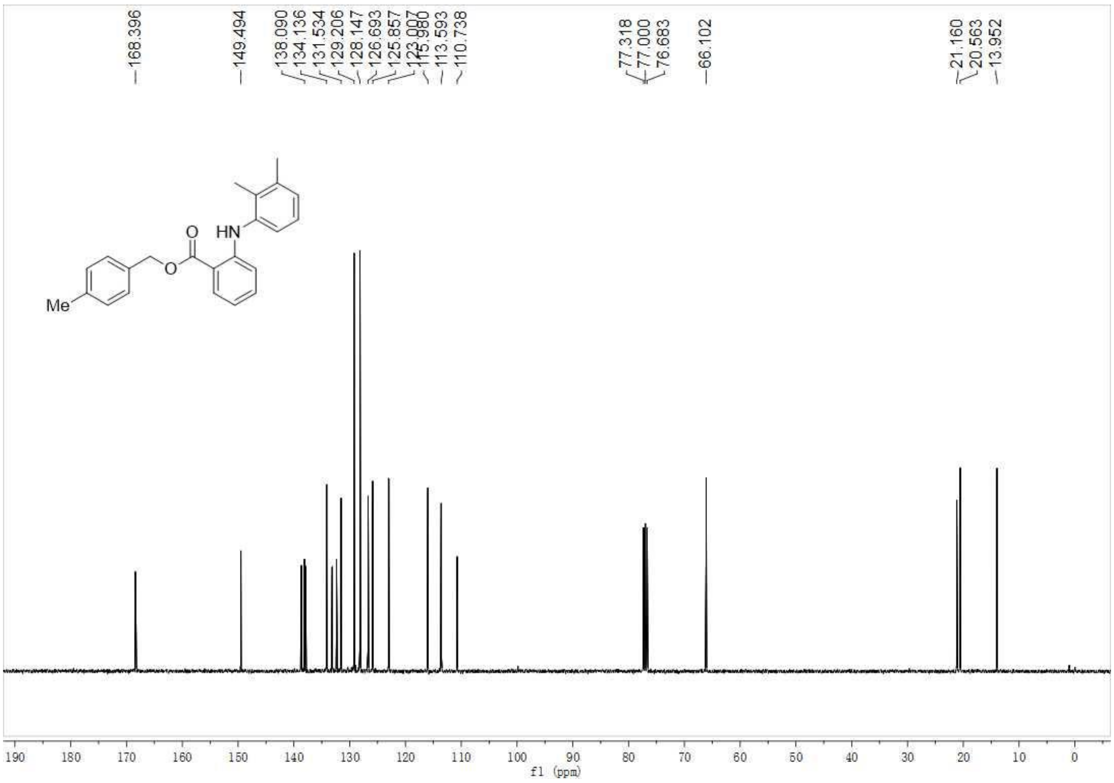

^{1}H NMR Spectrum of **181**

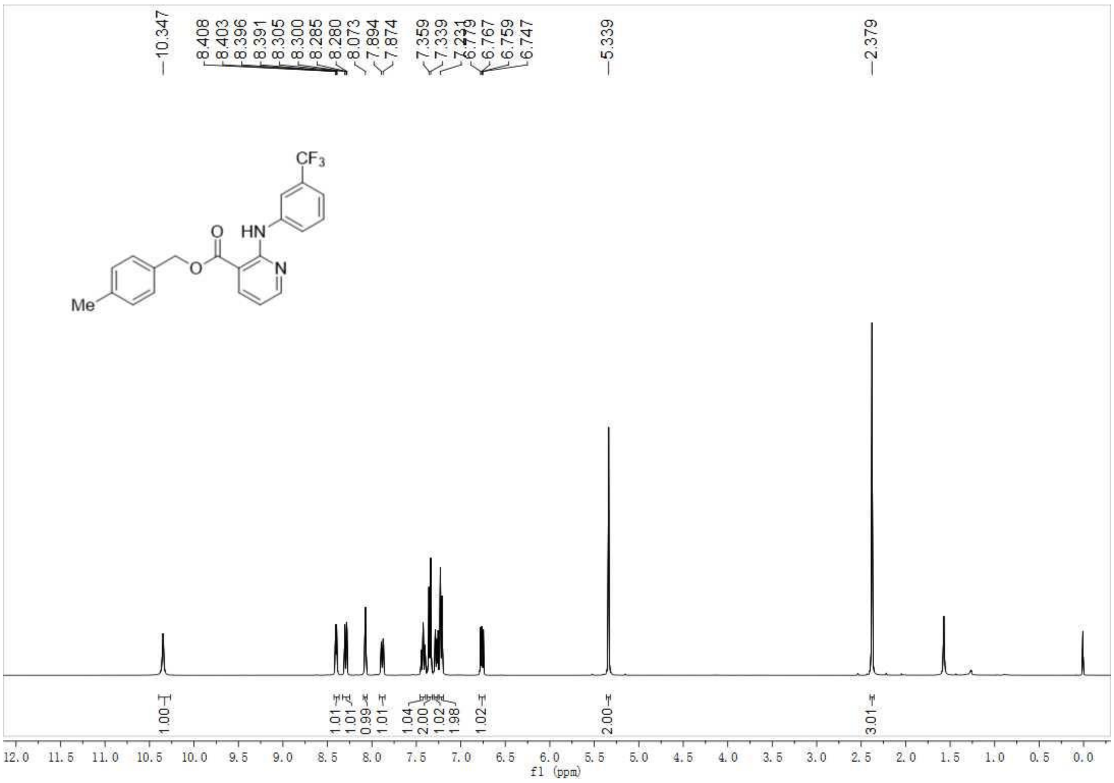

^{13}C NMR Spectrum of **181**

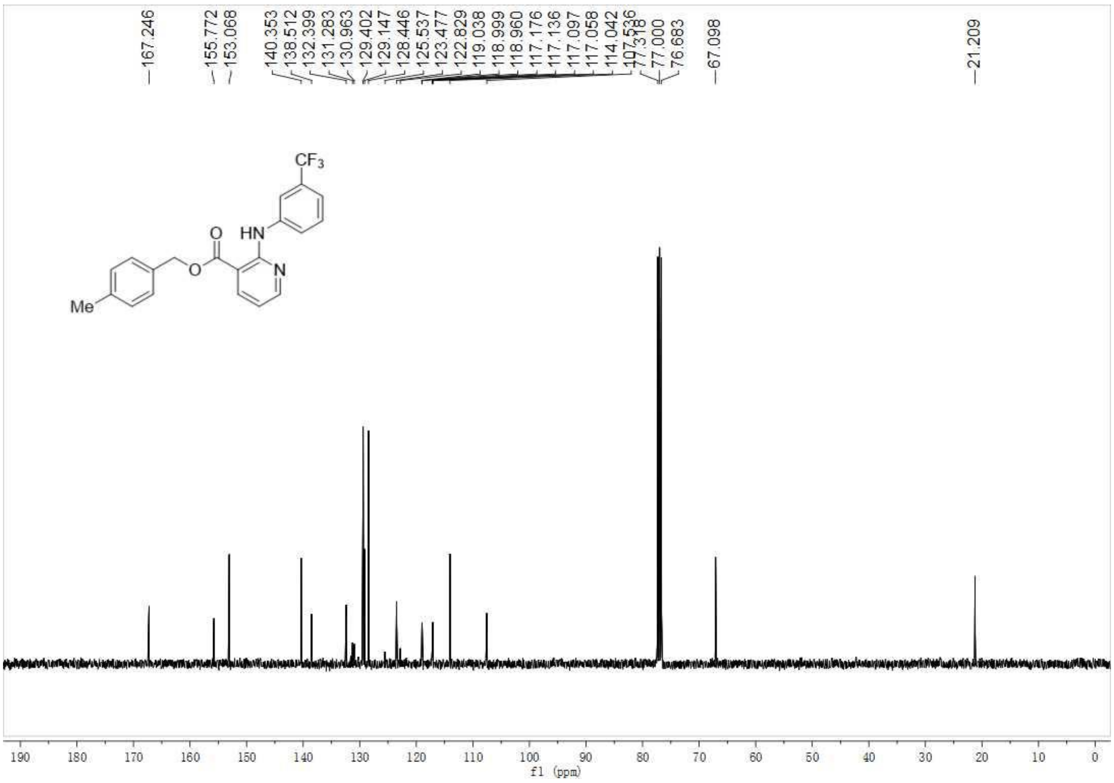

^{19}F NMR Spectrum of **181**

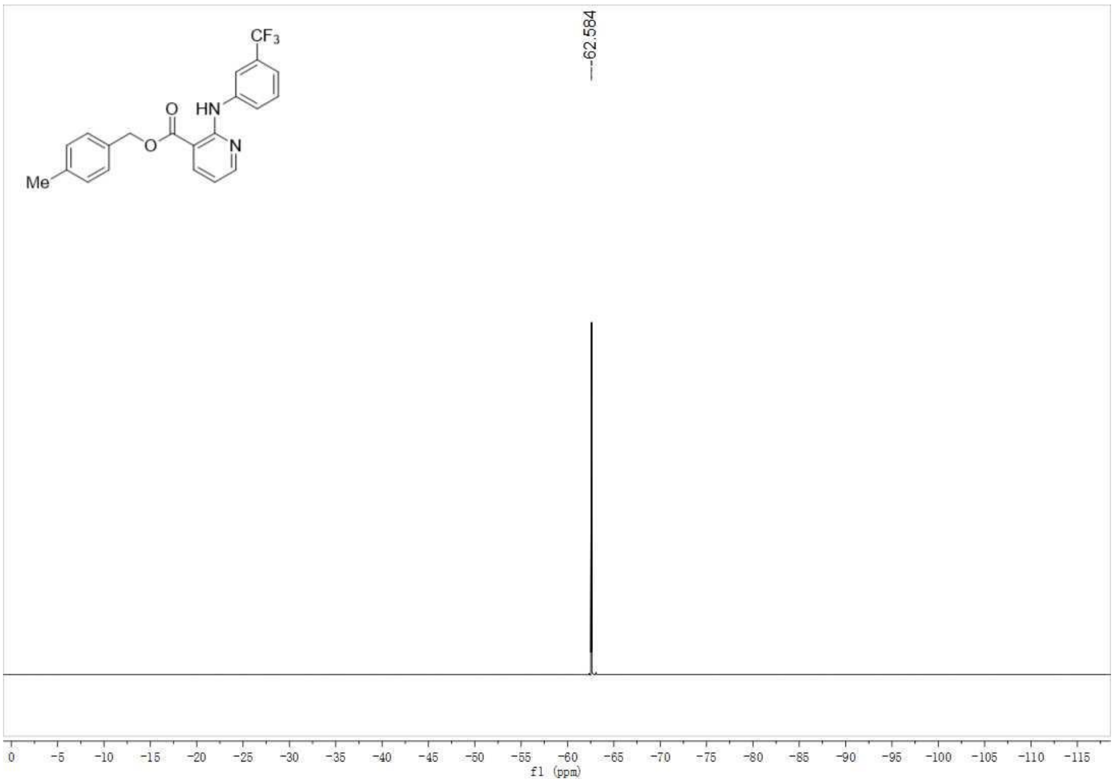

^{1}H NMR Spectrum of **182**

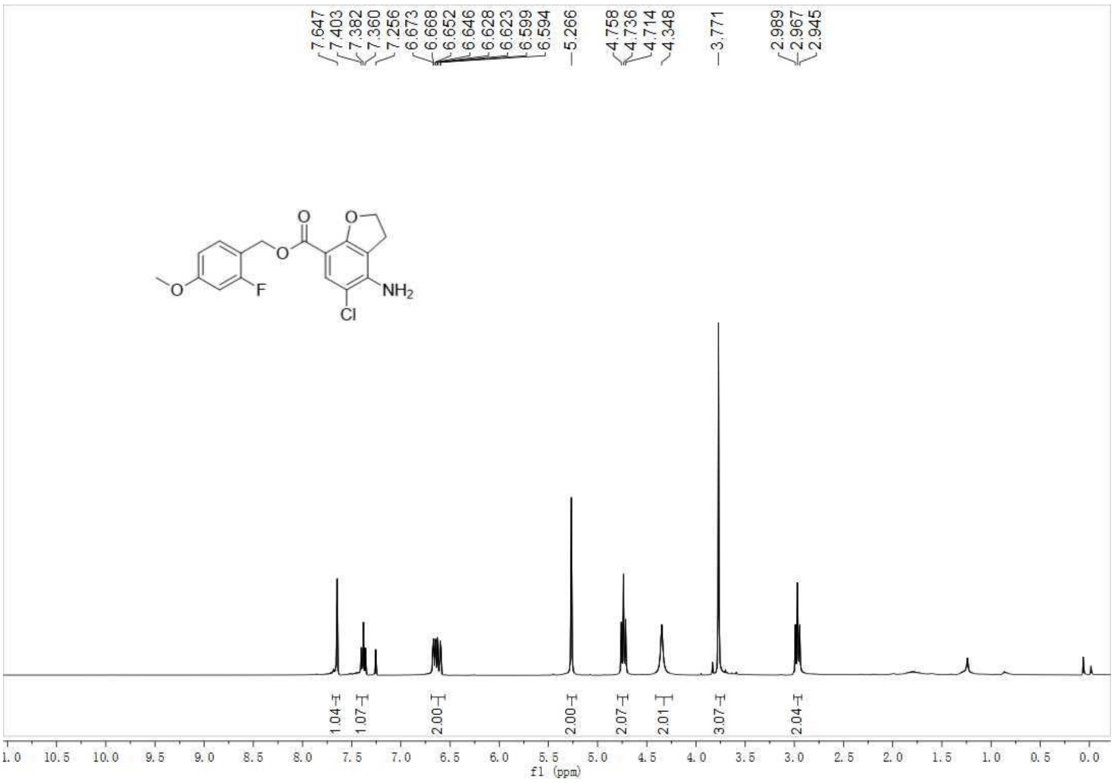

^{13}C NMR Spectrum of **182**

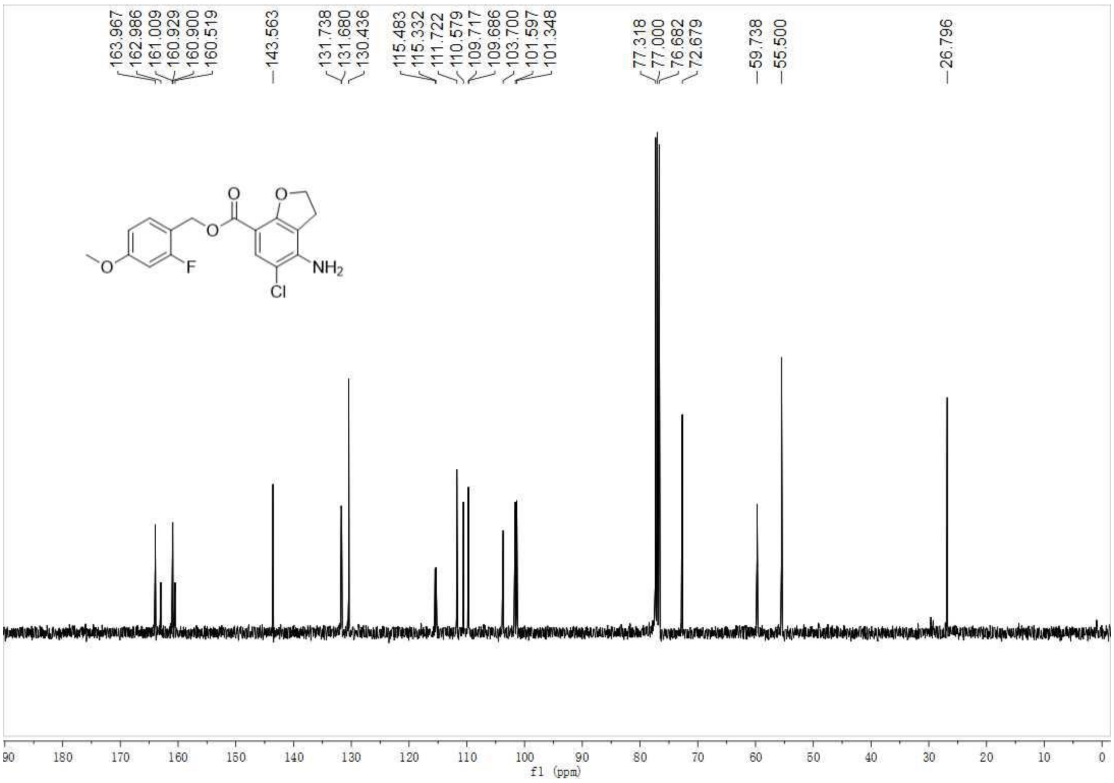

^{19}F NMR Spectrum of **182**

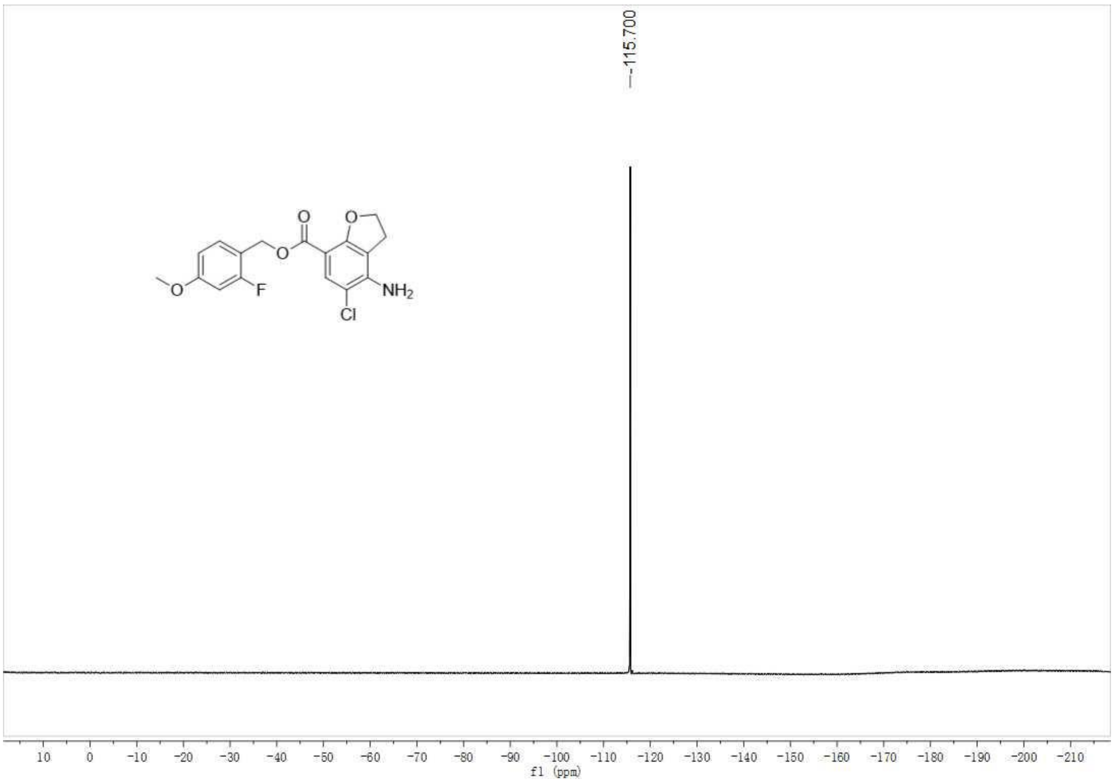

^{1}H NMR Spectrum of **183**

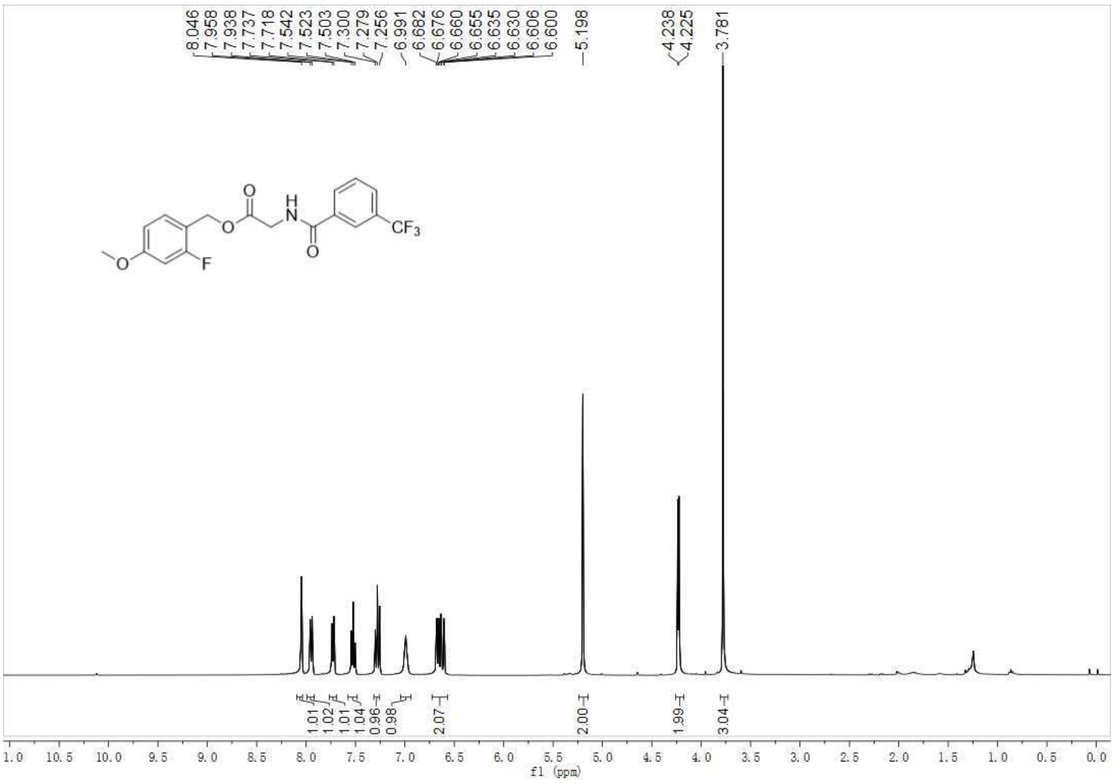

^{13}C NMR Spectrum of **183**

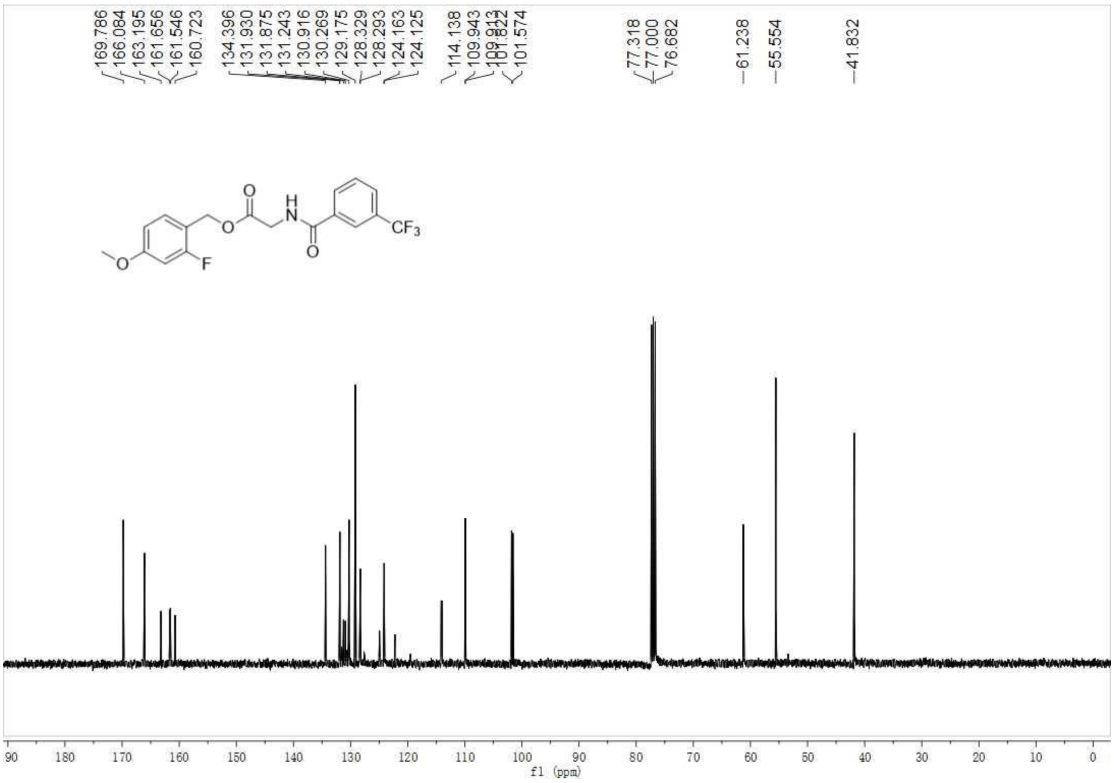

^{19}F NMR Spectrum of **183**

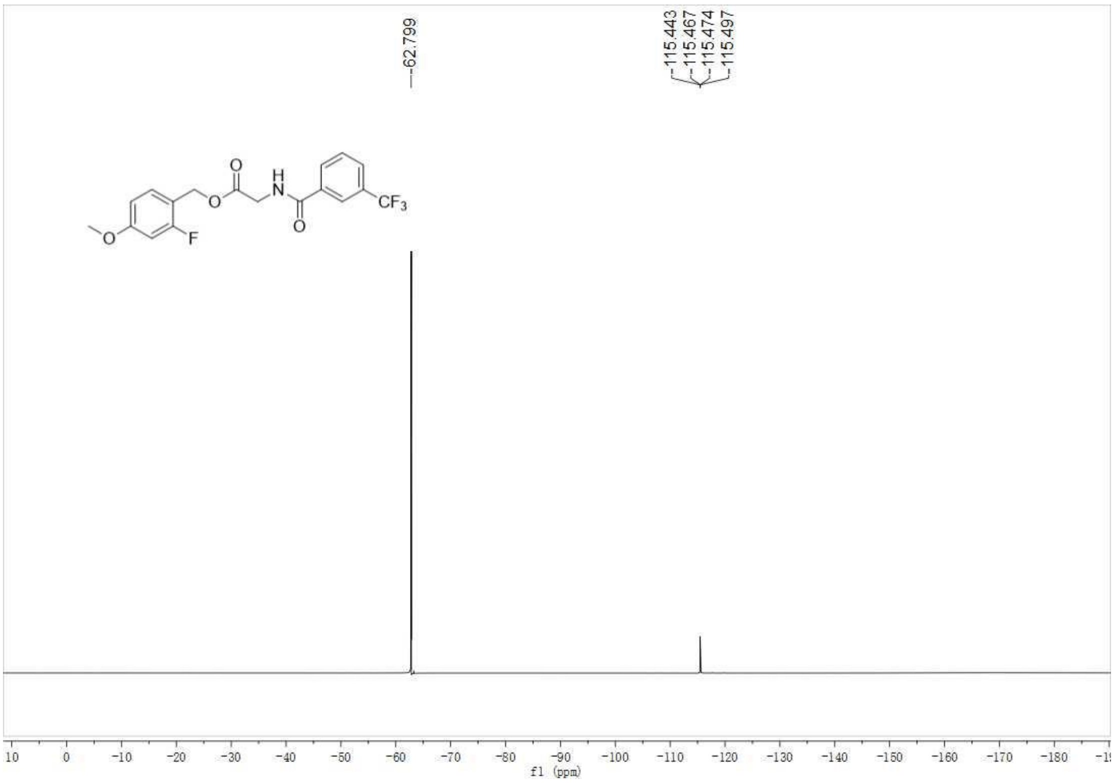

^{1}H NMR Spectrum of **184**

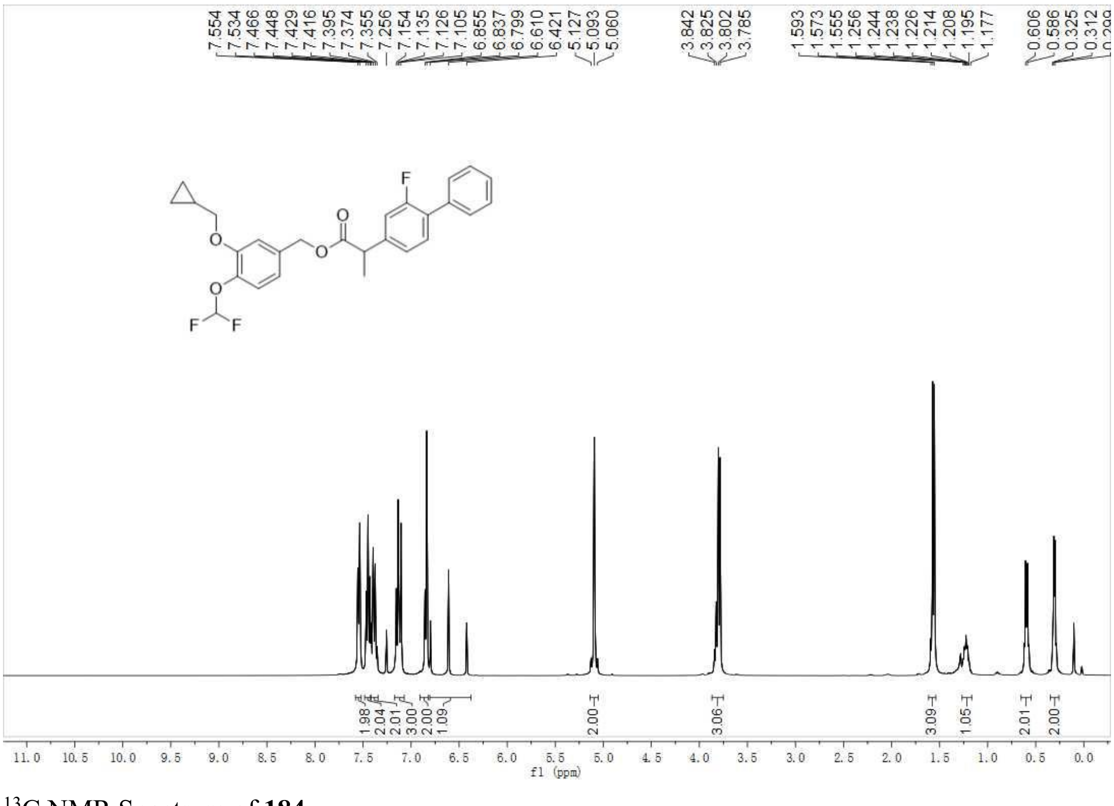

^{13}C NMR Spectrum of **184**

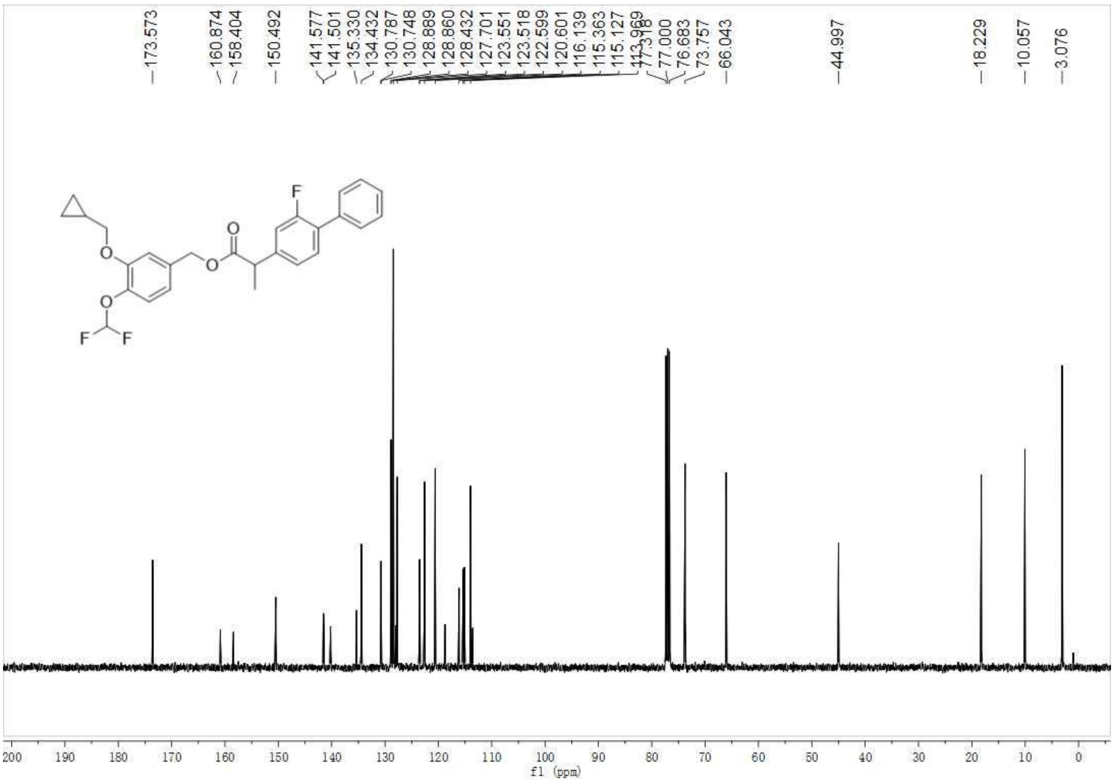

^{19}F NMR Spectrum of **184**

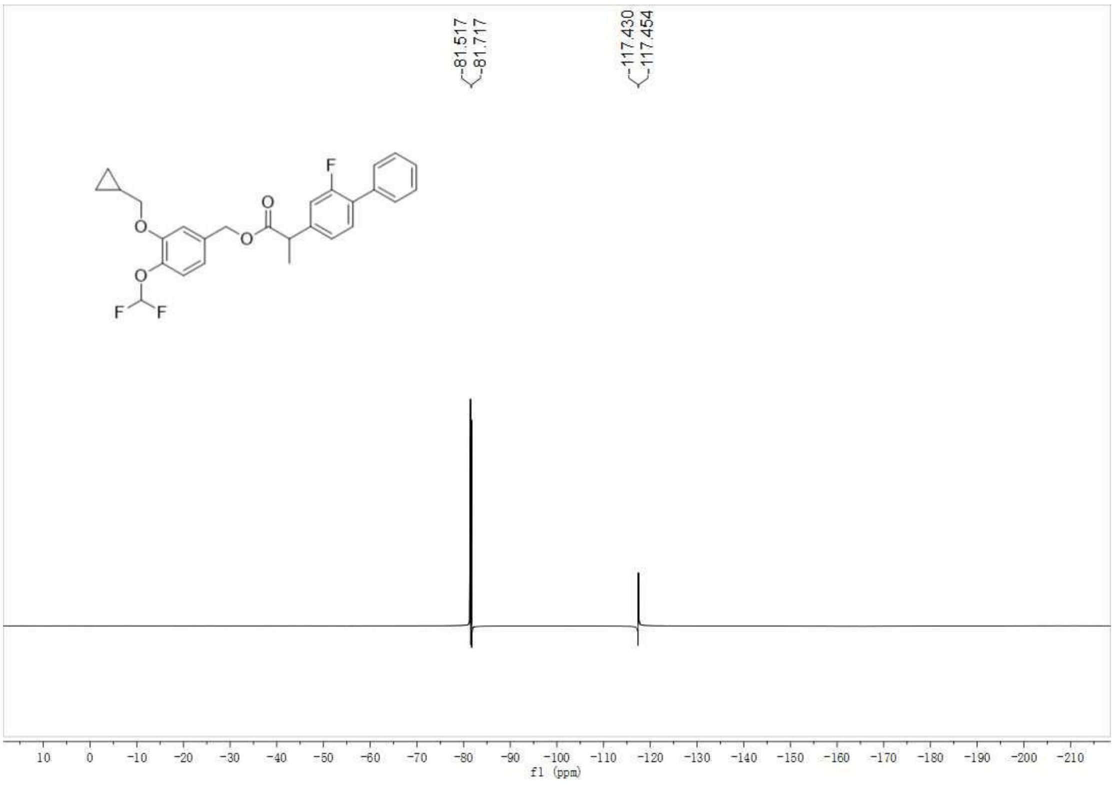

^{1}H NMR Spectrum of **185**

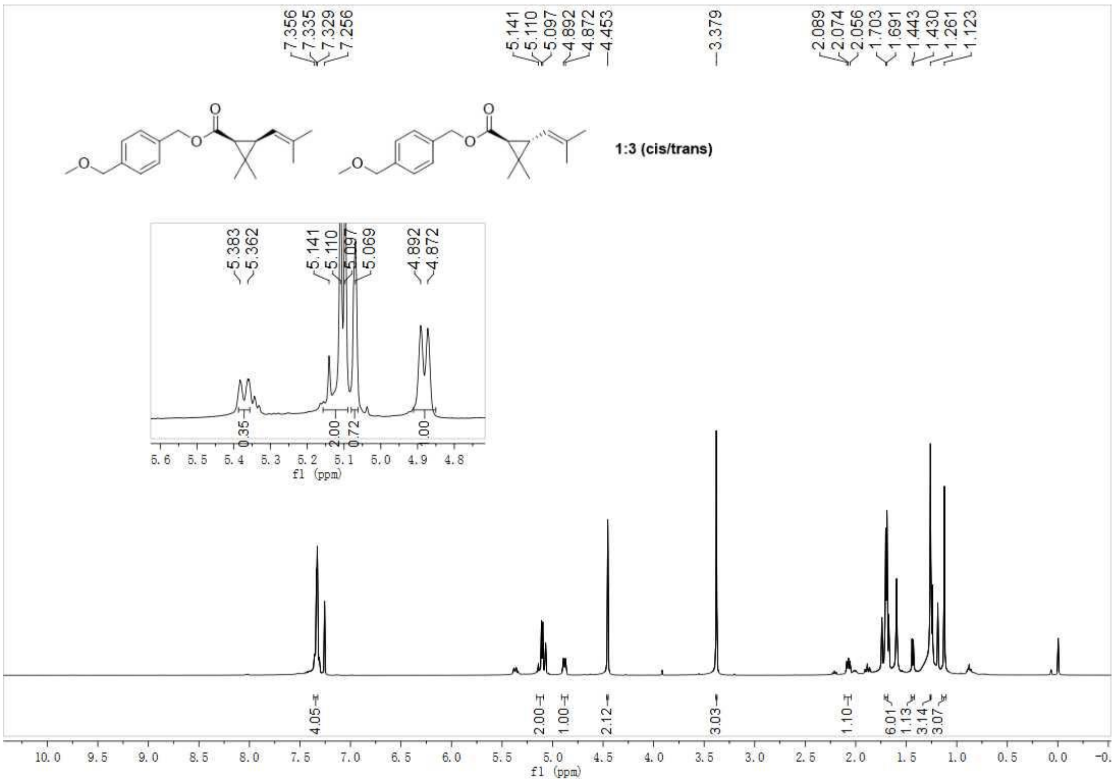

^{13}C NMR Spectrum of **185**

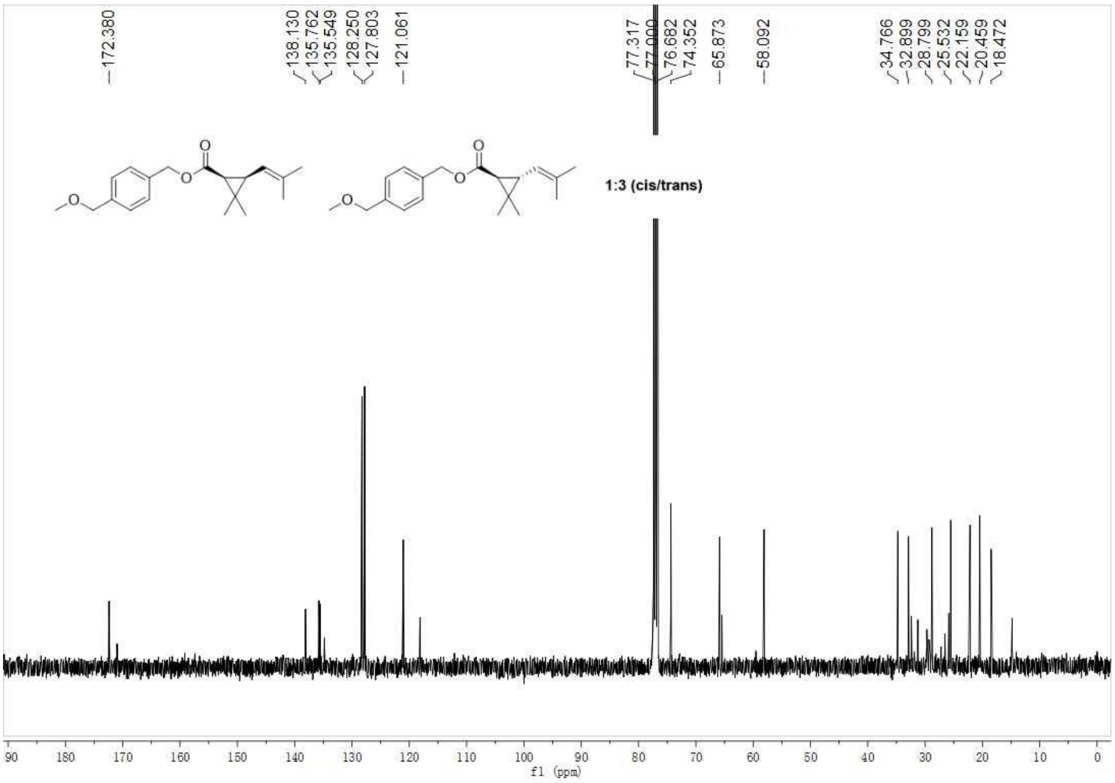

^{1}H NMR Spectrum of **186**

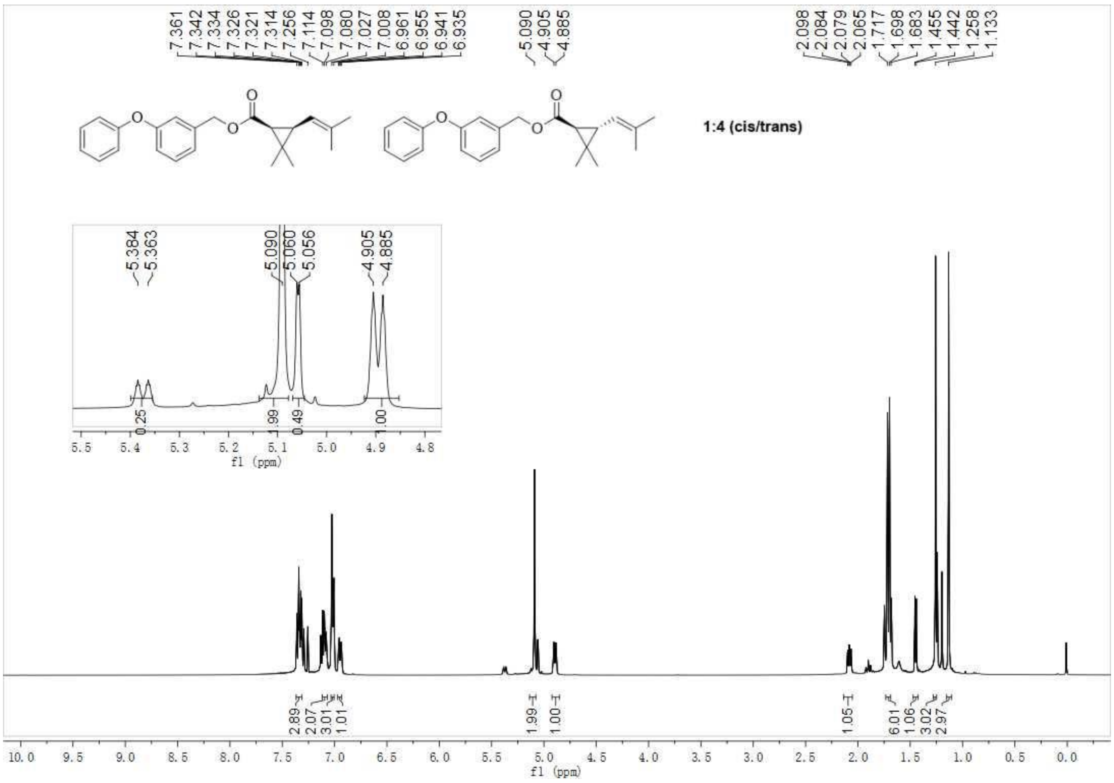

^{13}C NMR Spectrum of **186**

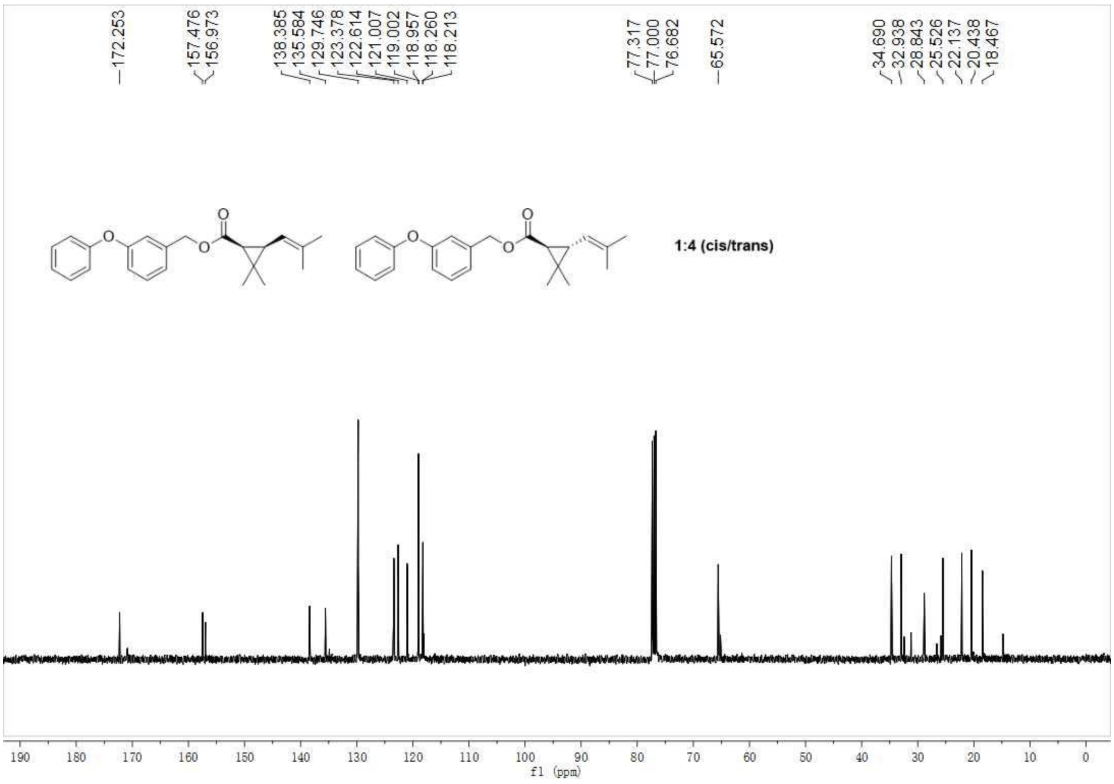

^{1}H NMR Spectrum of **186**

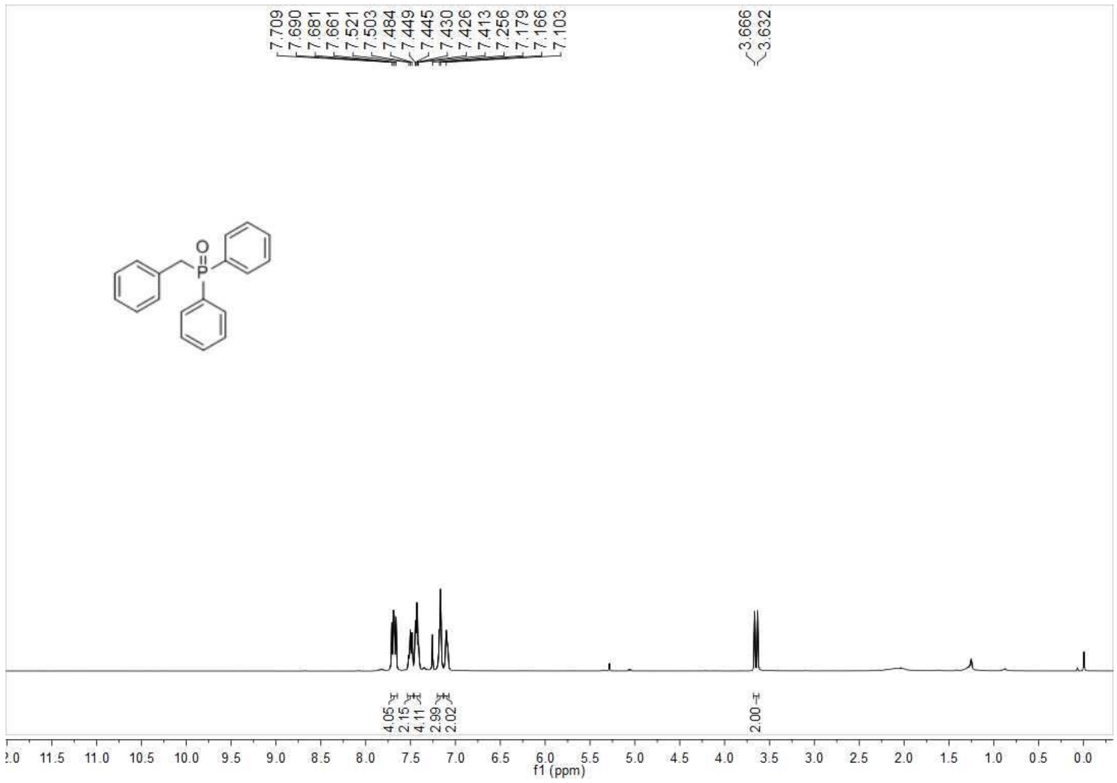

^{13}C NMR Spectrum of **187**

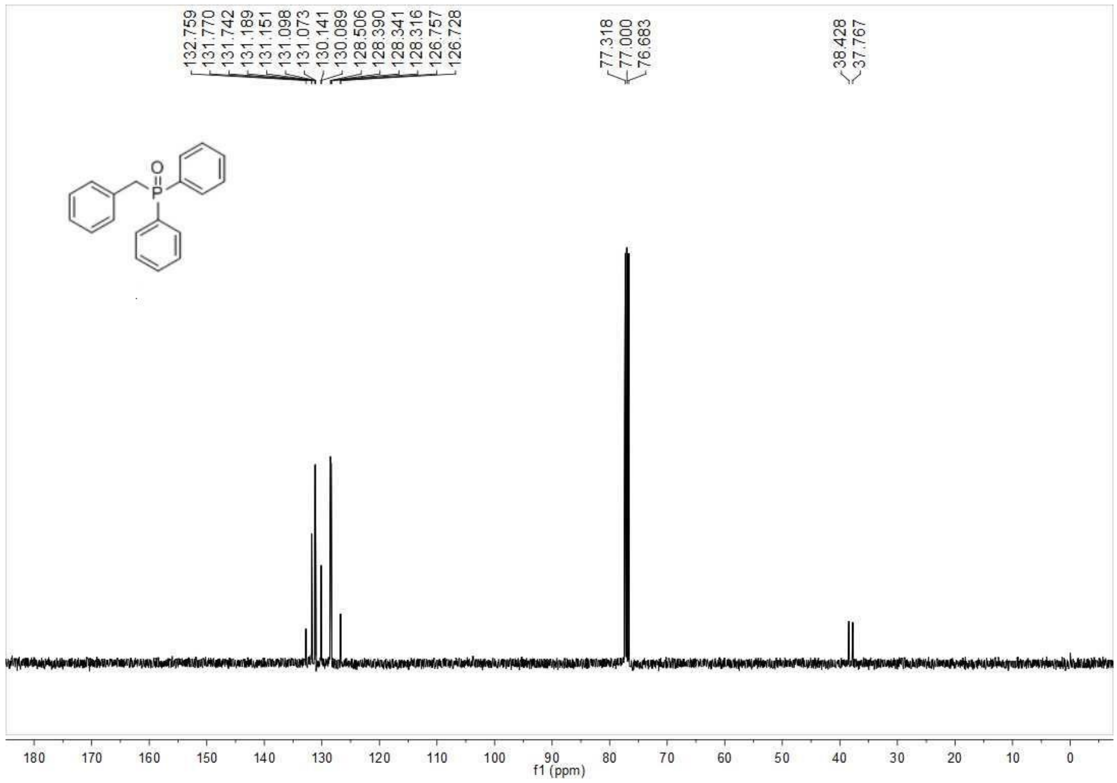

^{31}P NMR Spectrum of **187**

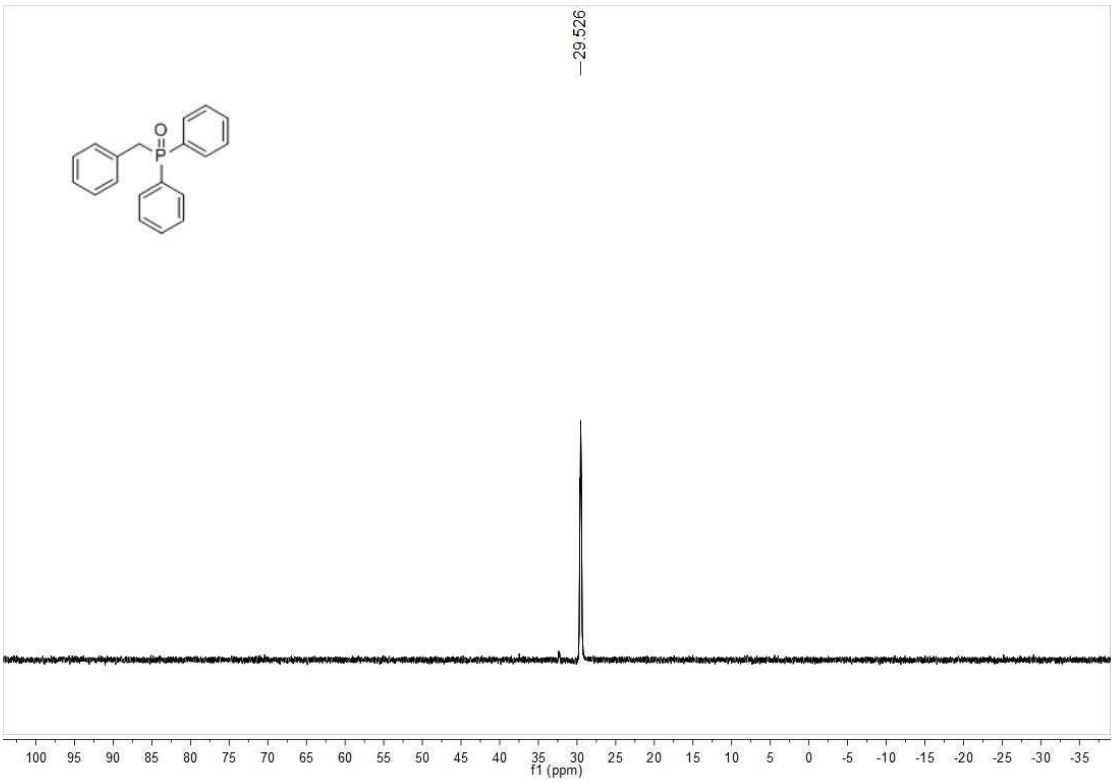

^{1}H NMR Spectrum of **188**

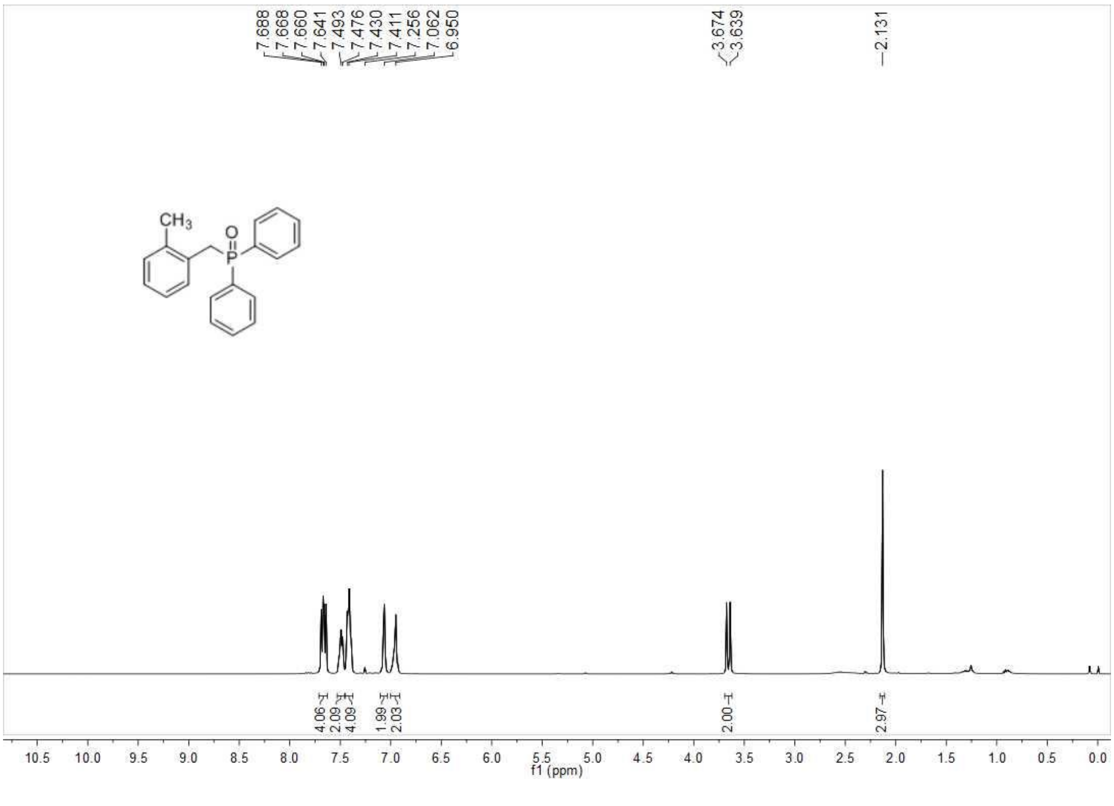

^{13}C NMR Spectrum of **188**

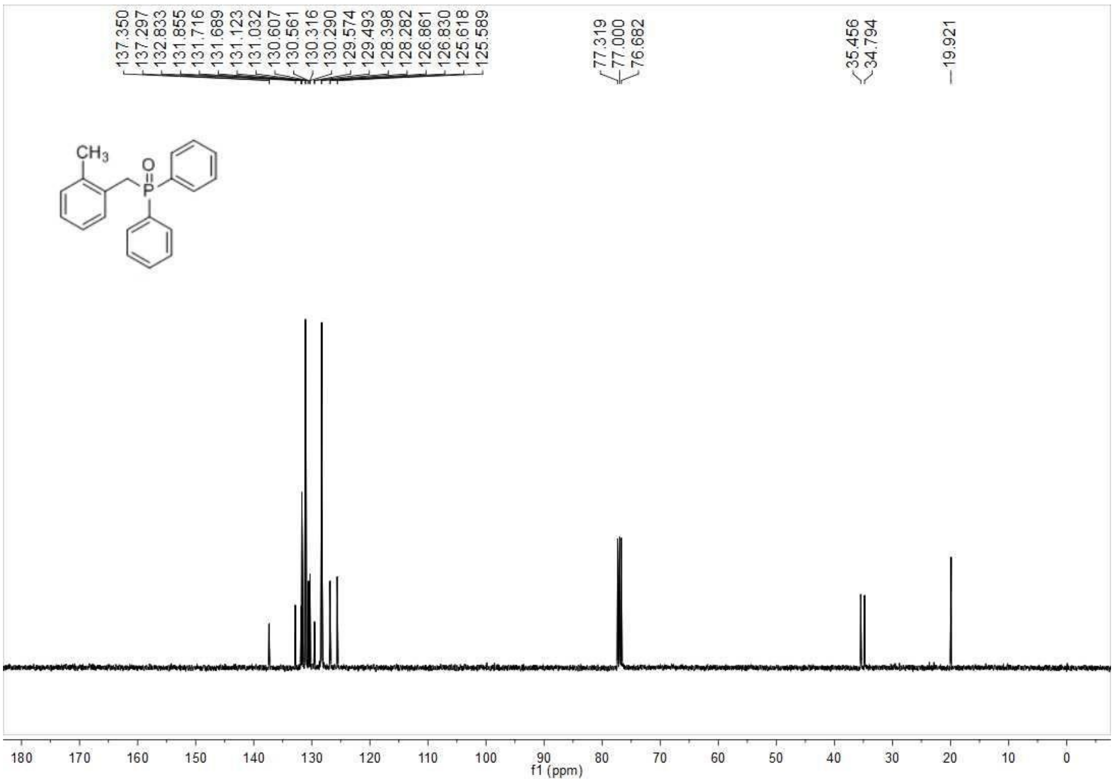

^{31}P NMR Spectrum of **188**

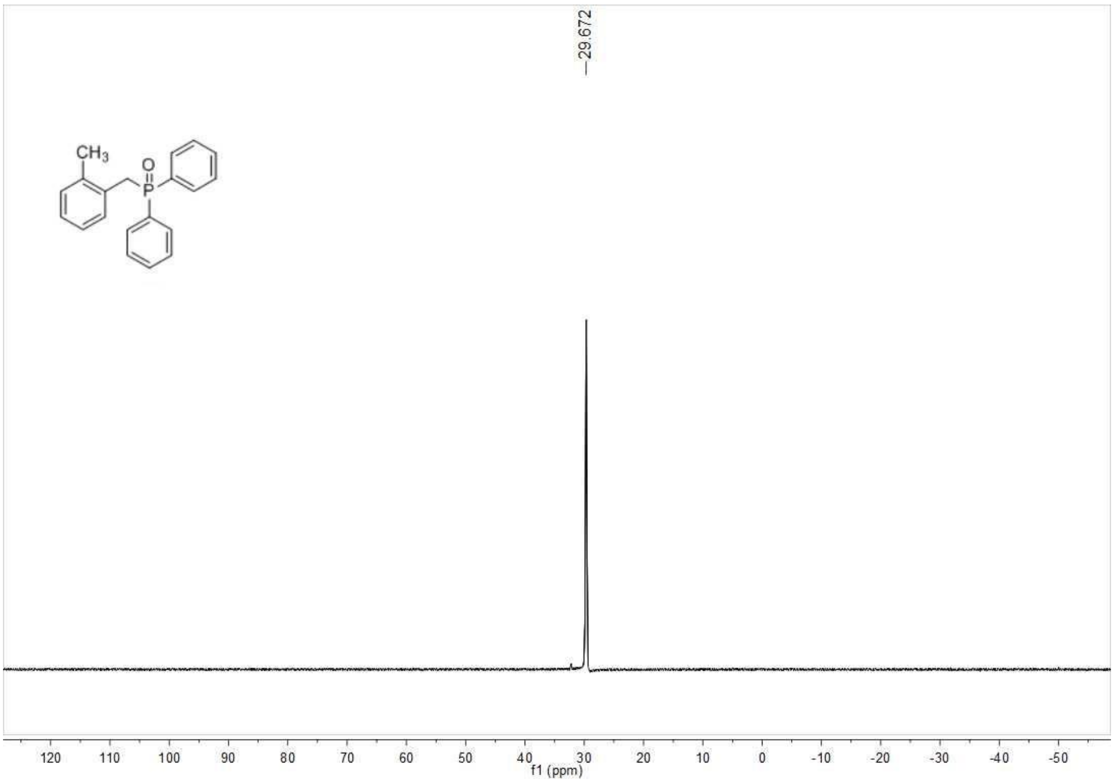

^{1}H NMR Spectrum of **189**

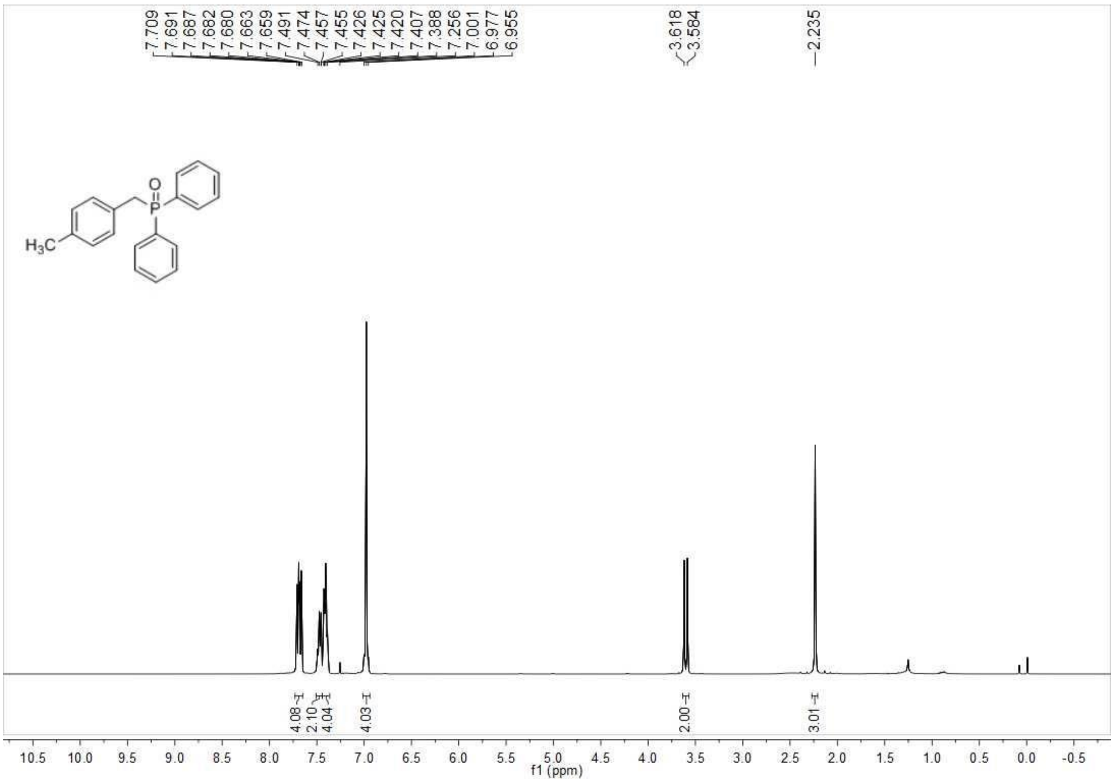

^{13}C NMR Spectrum of **189**

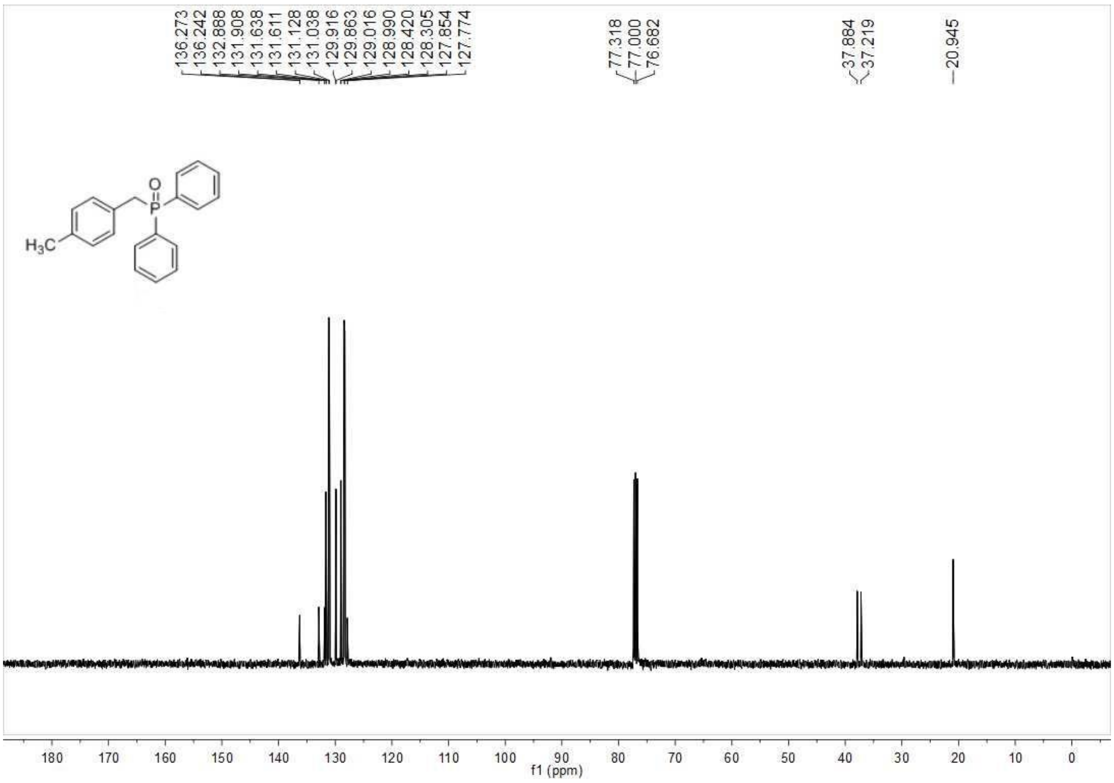

^{31}P NMR Spectrum of **189**

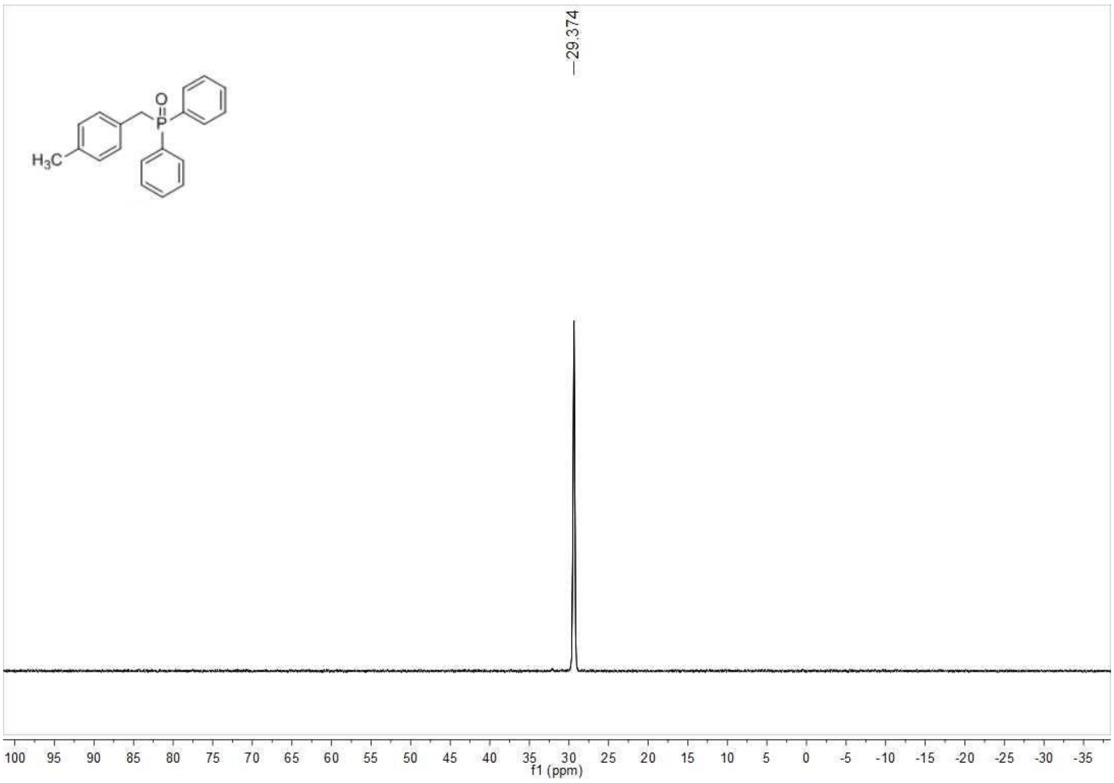

^{1}H NMR Spectrum of **190**

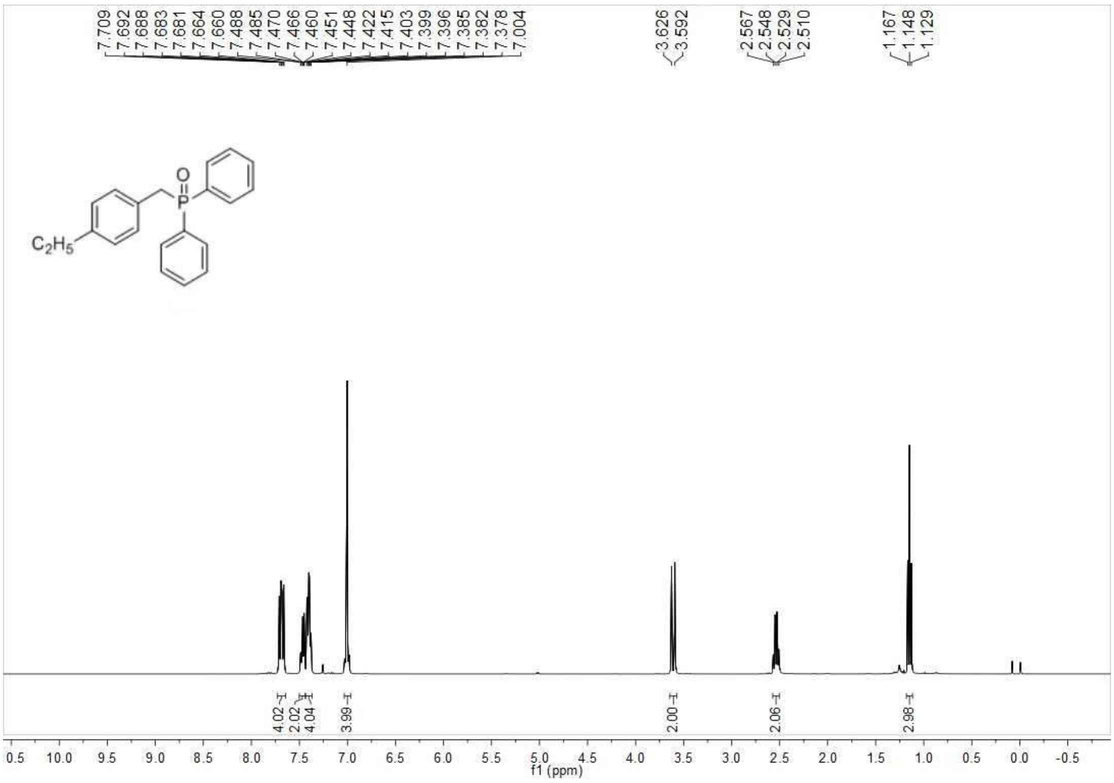

^{13}C NMR Spectrum of **190**

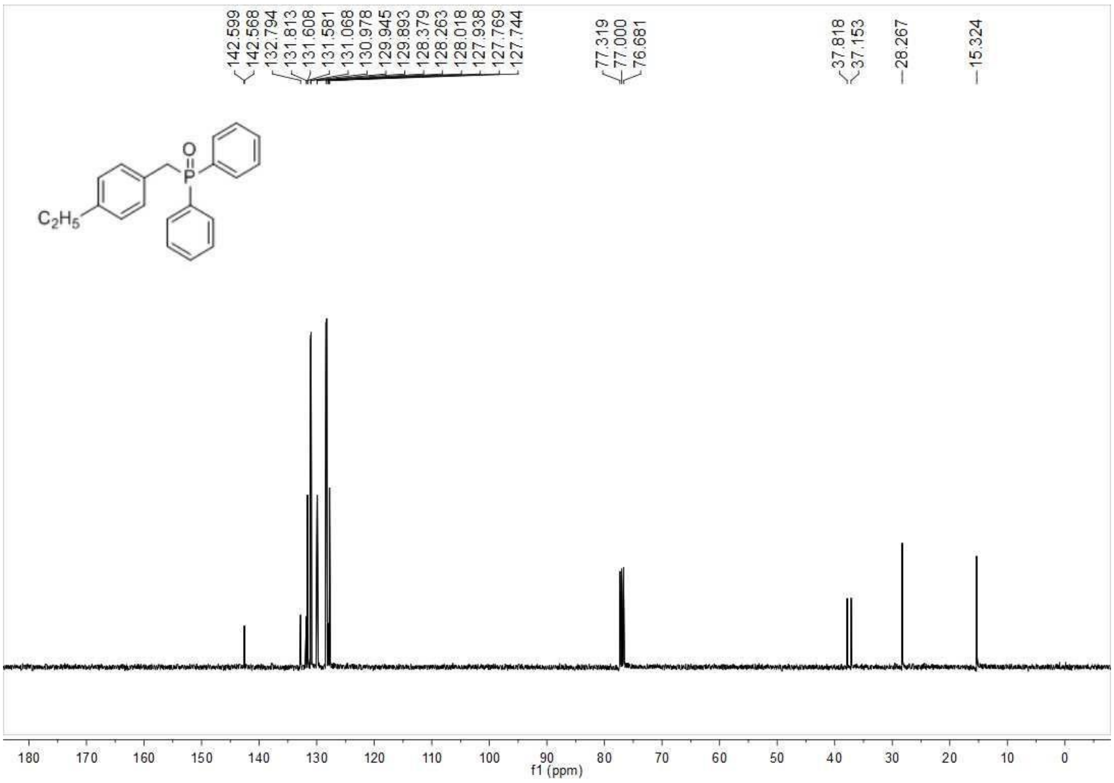

^{31}P NMR Spectrum of **190**

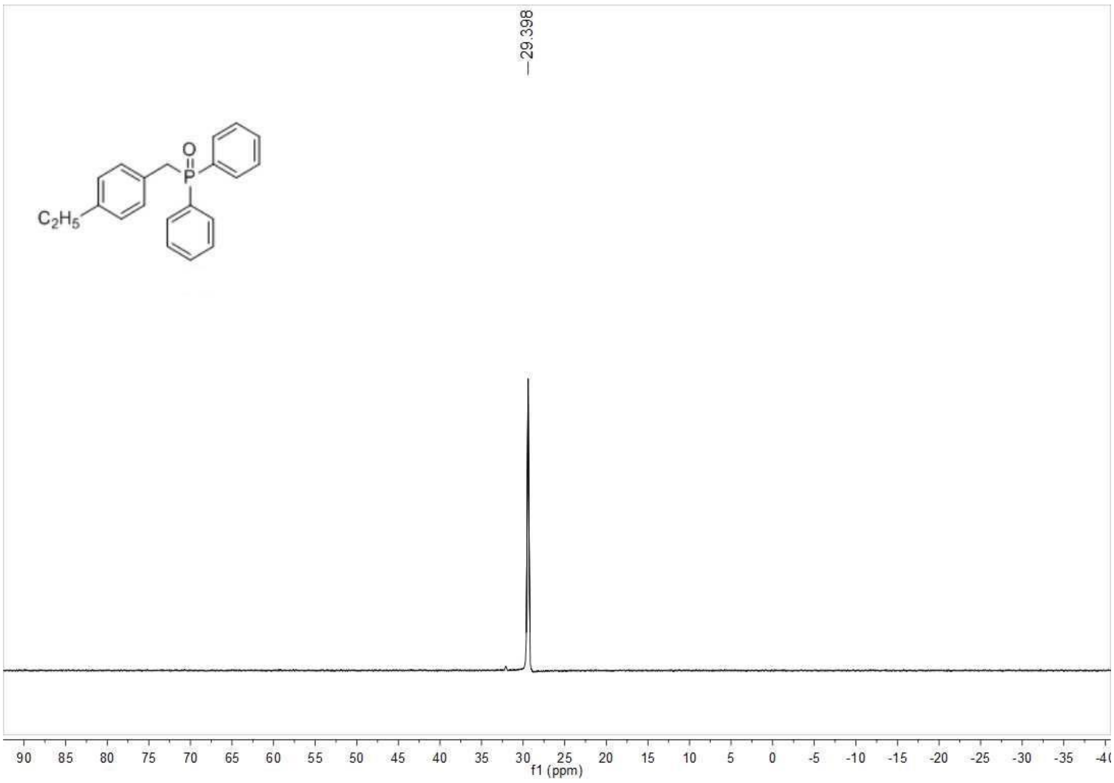

^{1}H NMR Spectrum of **191**

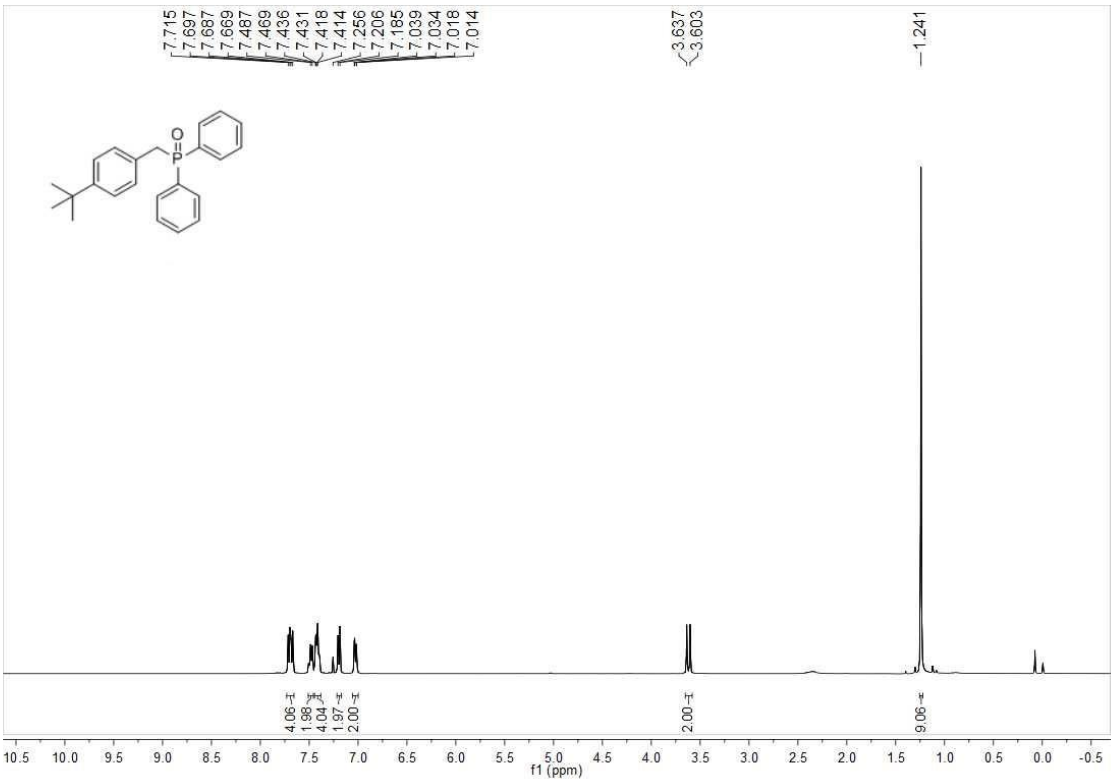

^{13}C NMR Spectrum of **191**

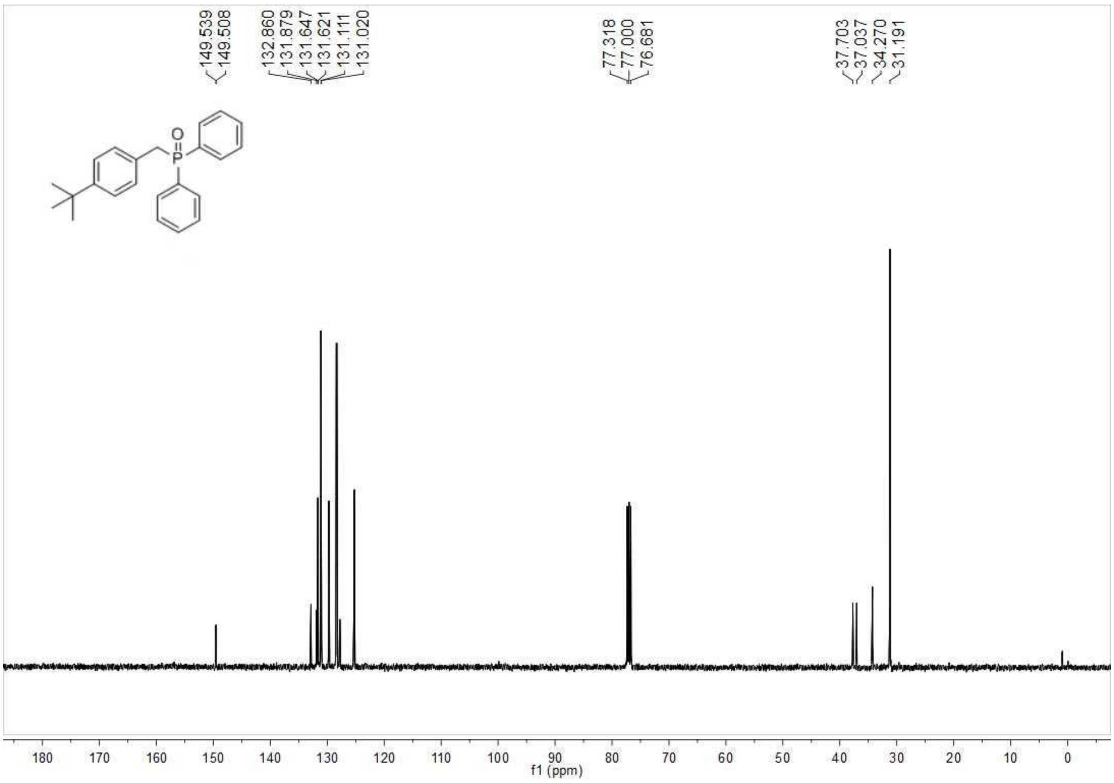

^{31}P NMR Spectrum of **191**

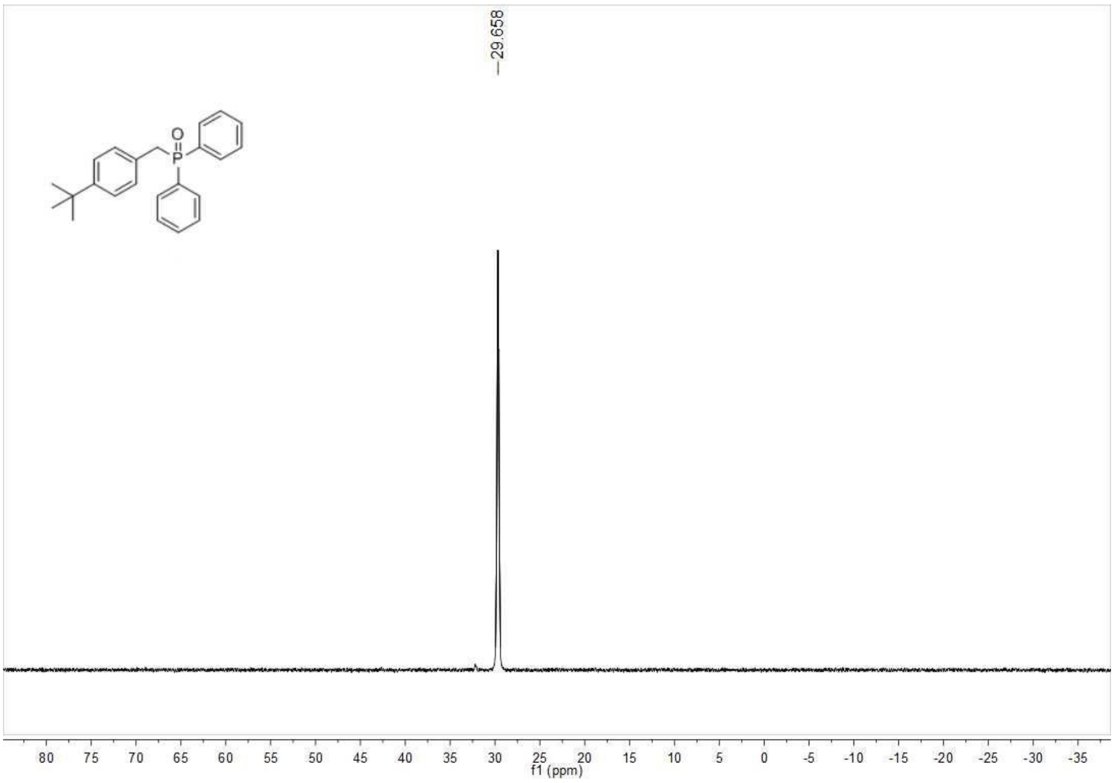

^{1}H NMR Spectrum of **192**

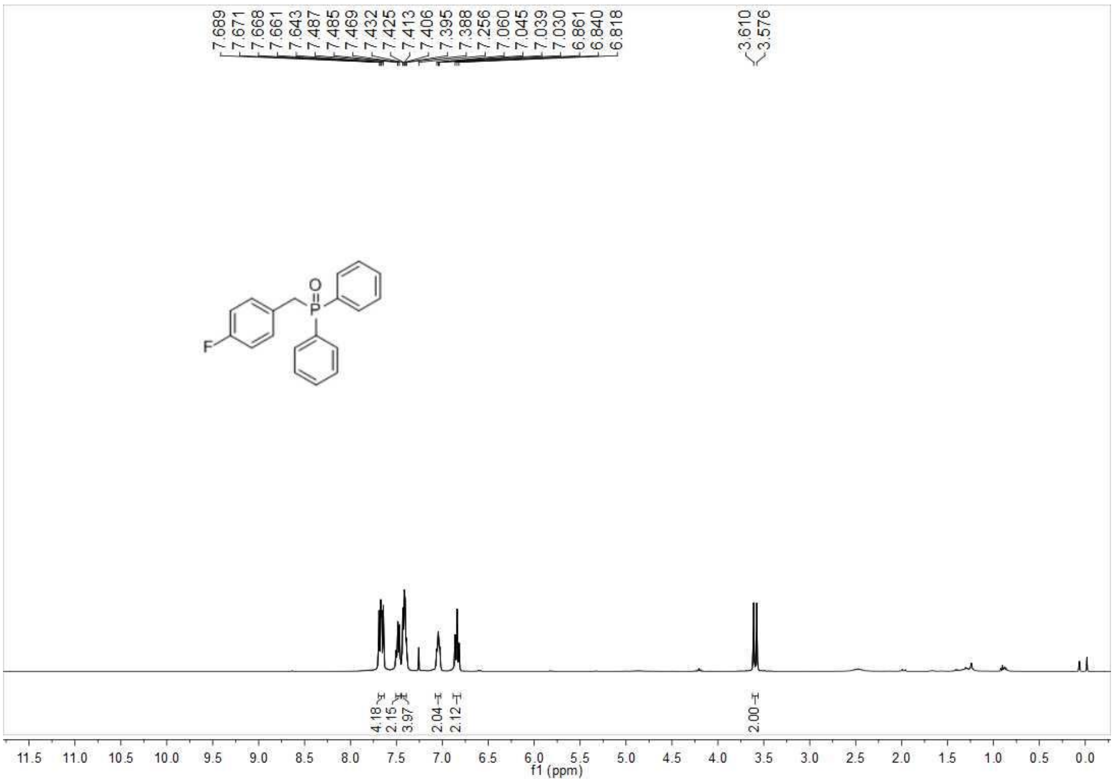

^{13}C NMR Spectrum of **192**

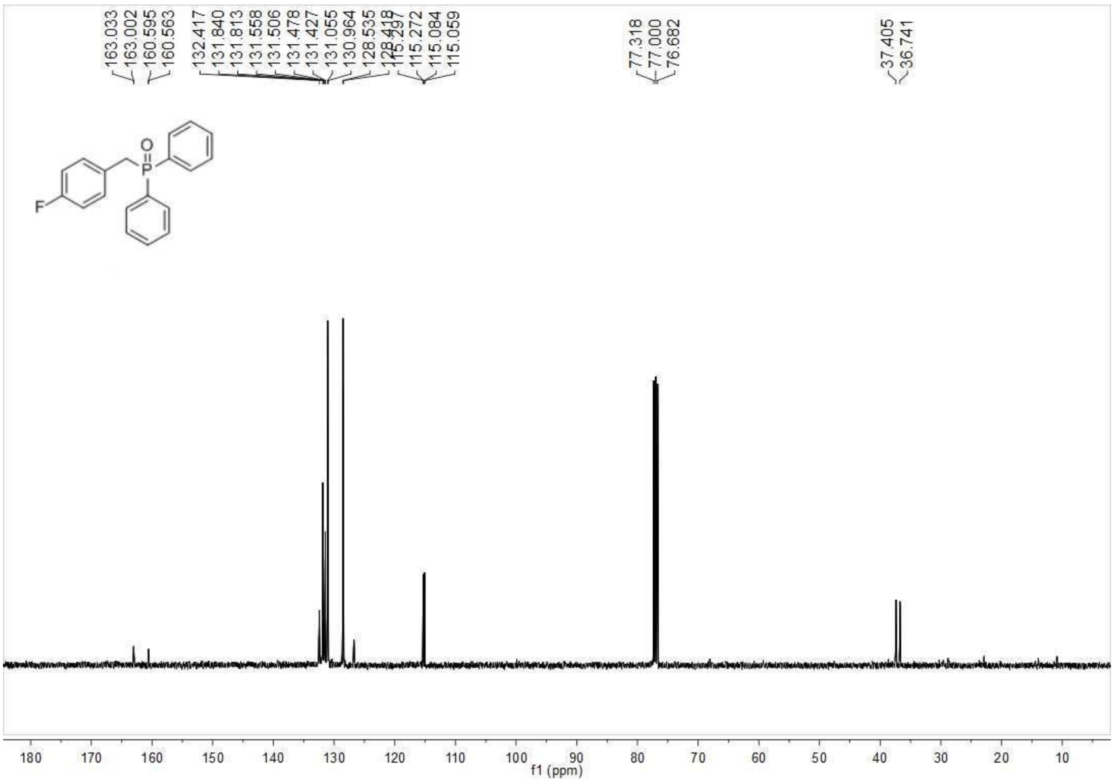

^{31}P NMR Spectrum of **192**

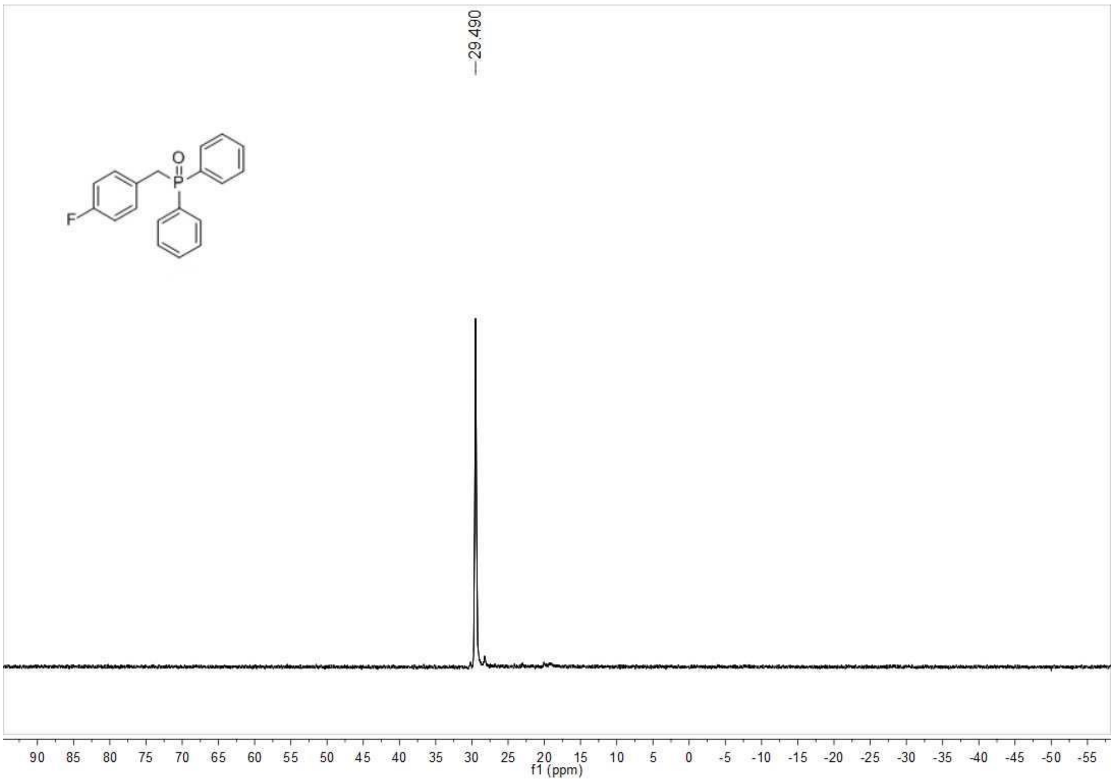

^{19}F NMR Spectrum of **192**

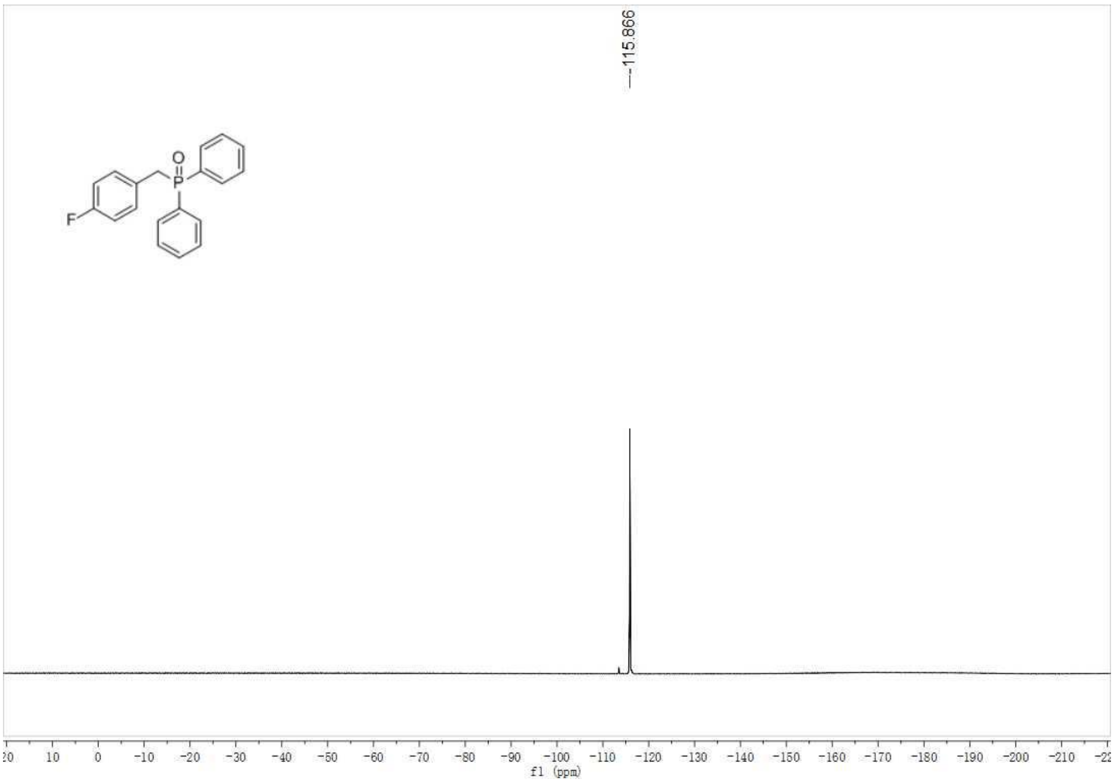

^{1}H NMR Spectrum of **193**

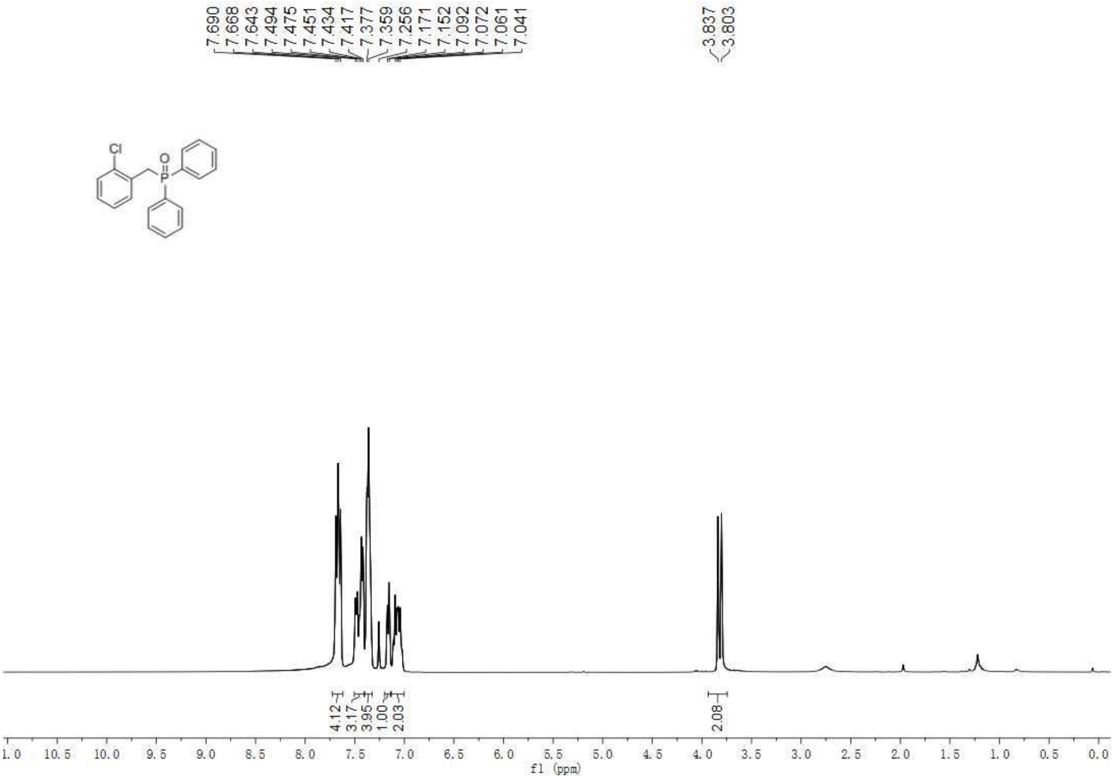

^{13}C NMR Spectrum of **193**

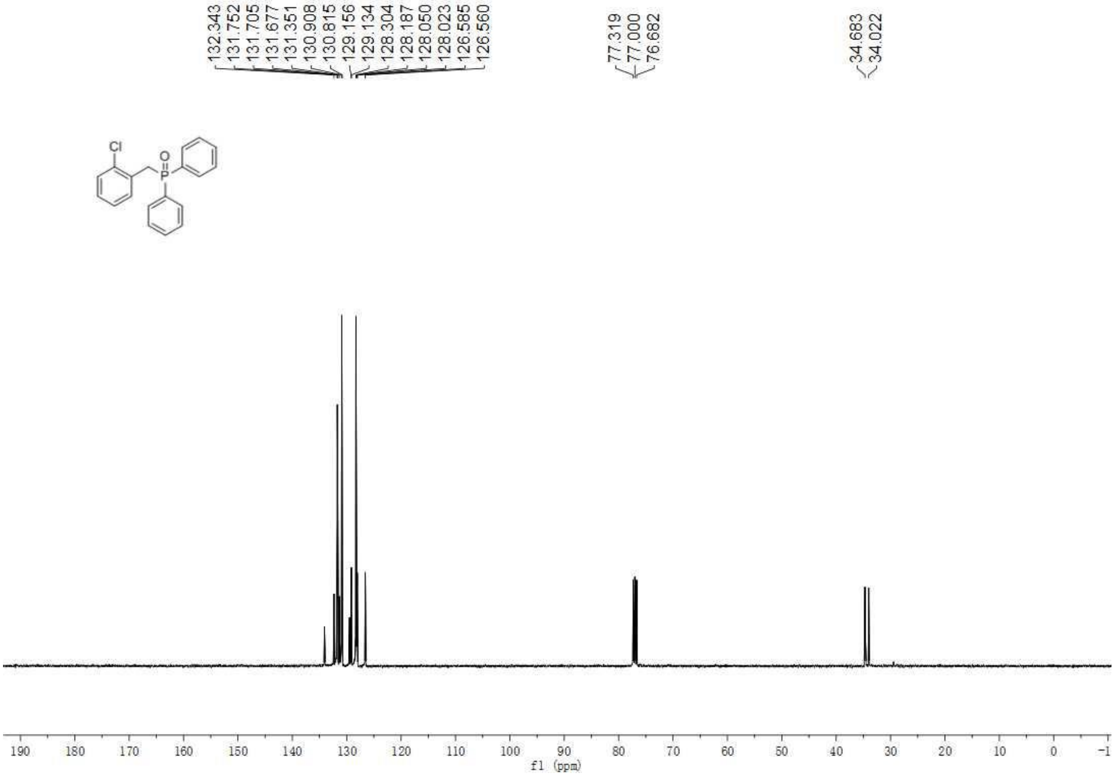

^{31}P NMR Spectrum of **193**

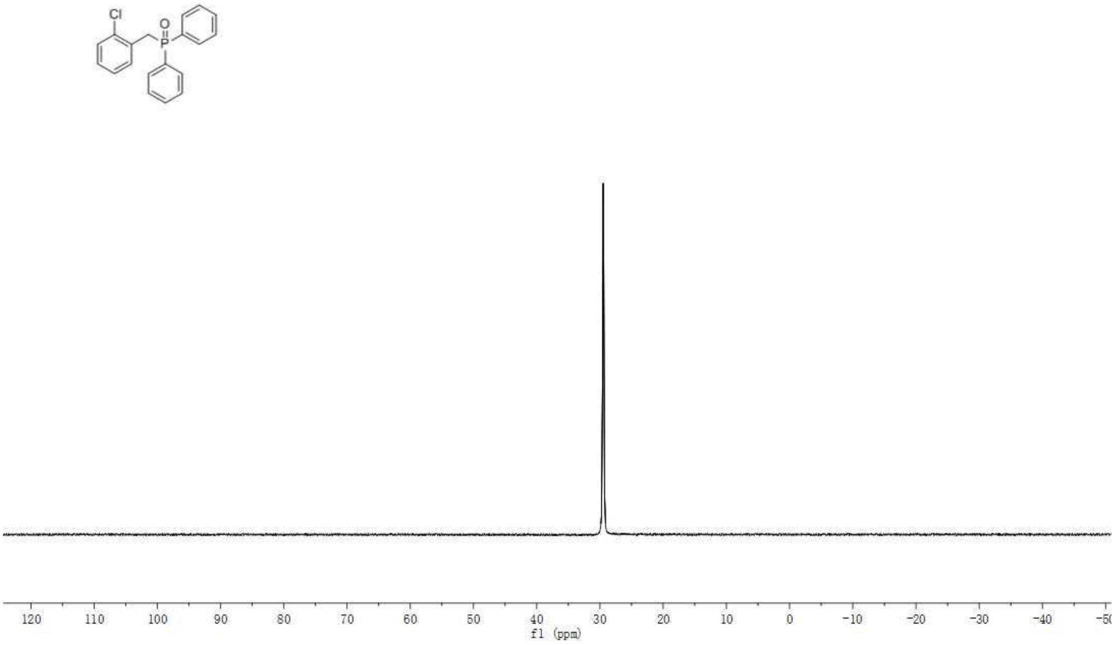

^{1}H NMR Spectrum of **194**

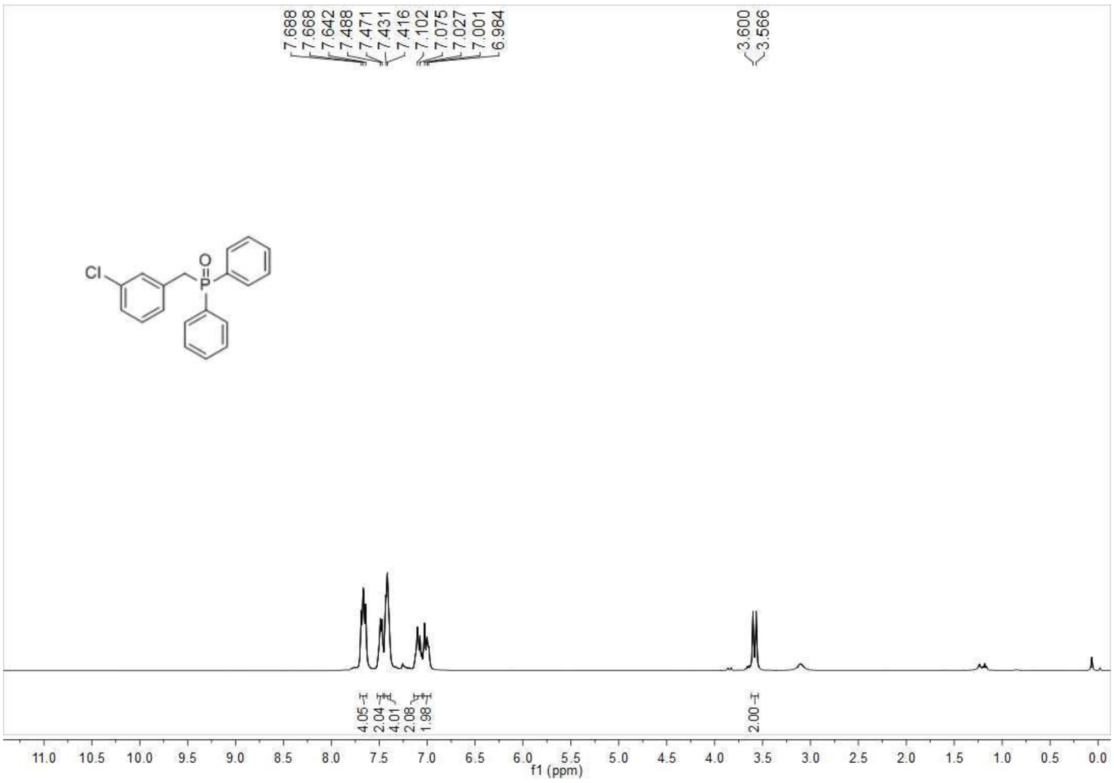

^{13}C NMR Spectrum of **194**

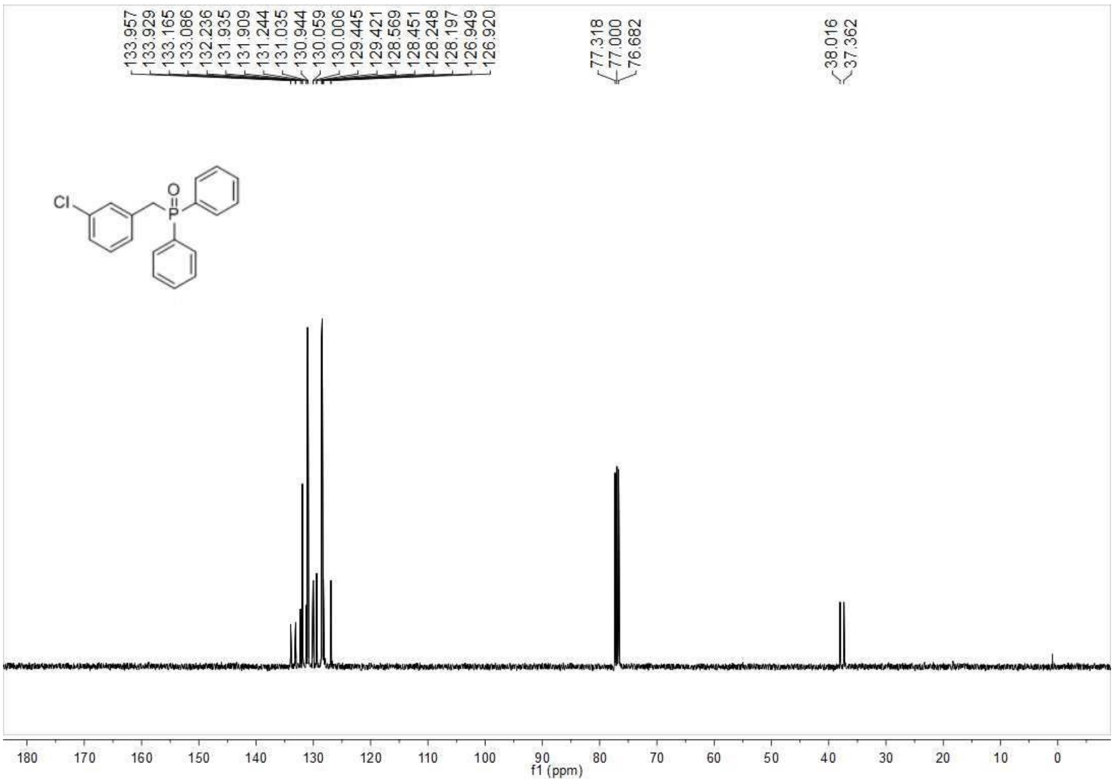

^{31}P NMR Spectrum of **194**

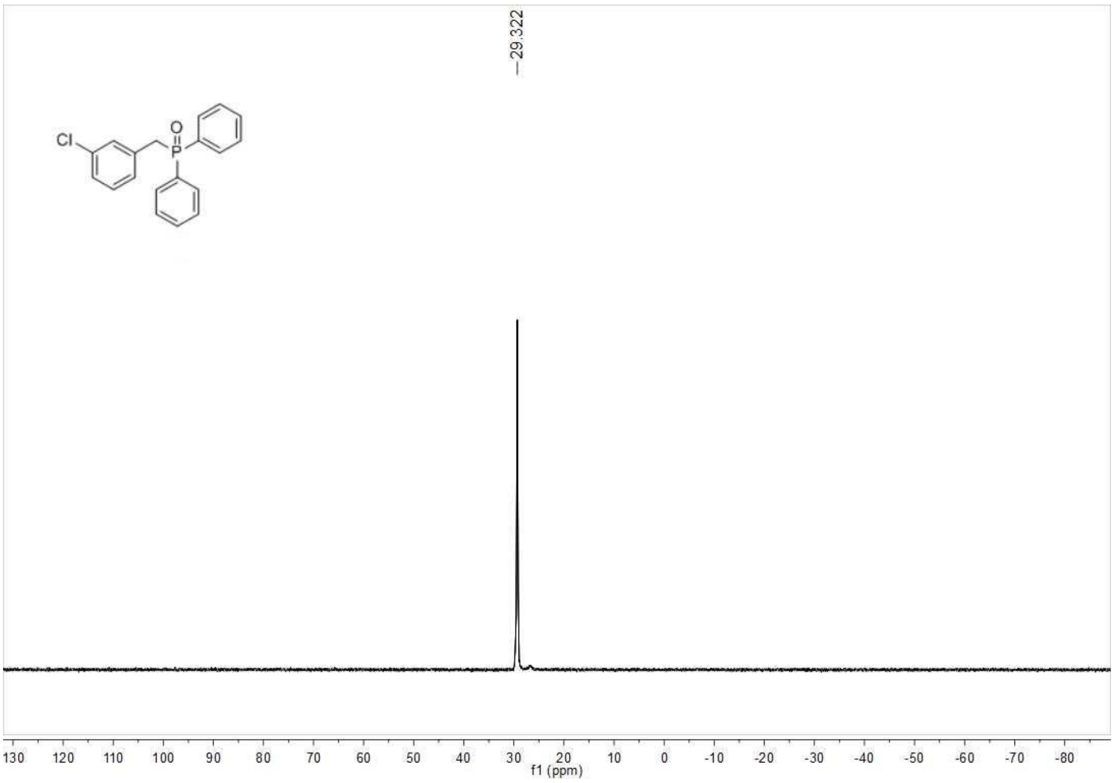

^{1}H NMR Spectrum of **195**

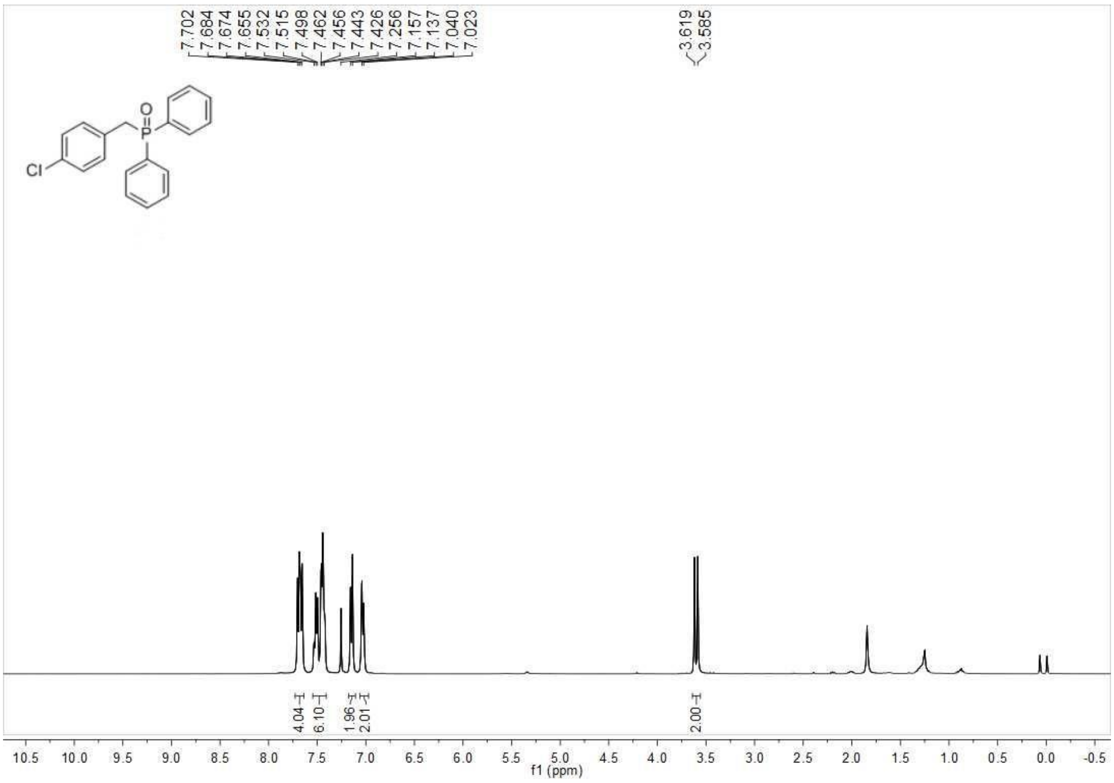

^{13}C NMR Spectrum of **195**

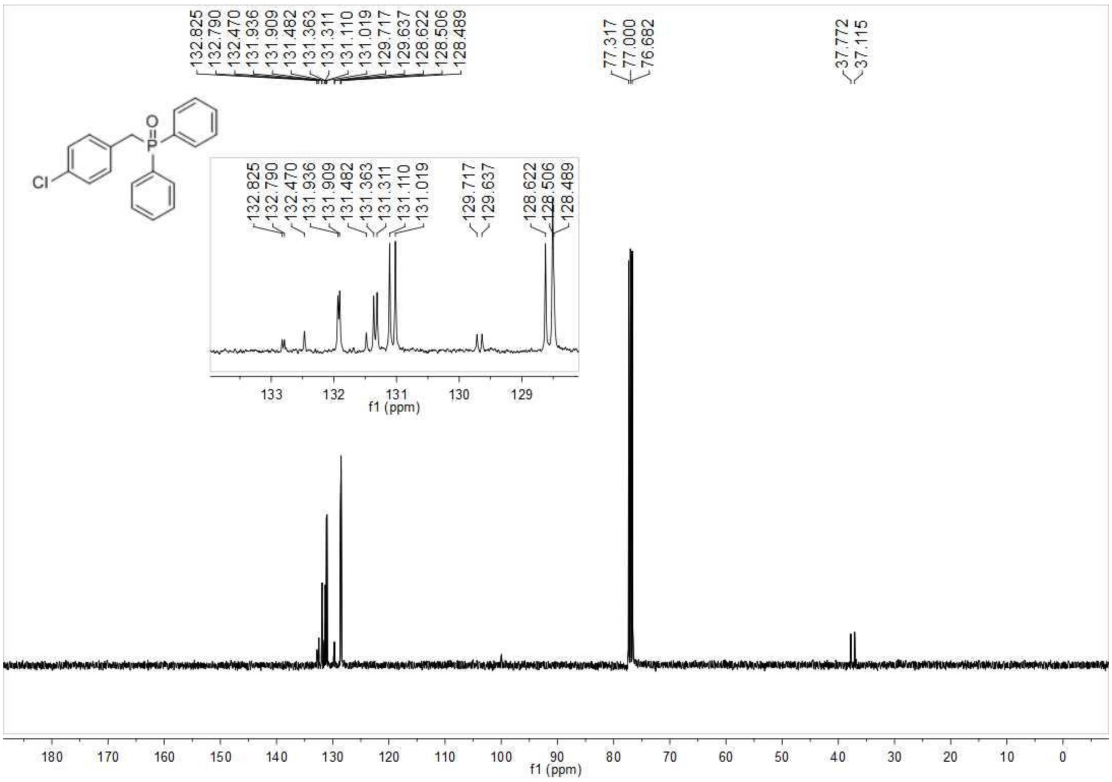

^{31}P NMR Spectrum of **195**

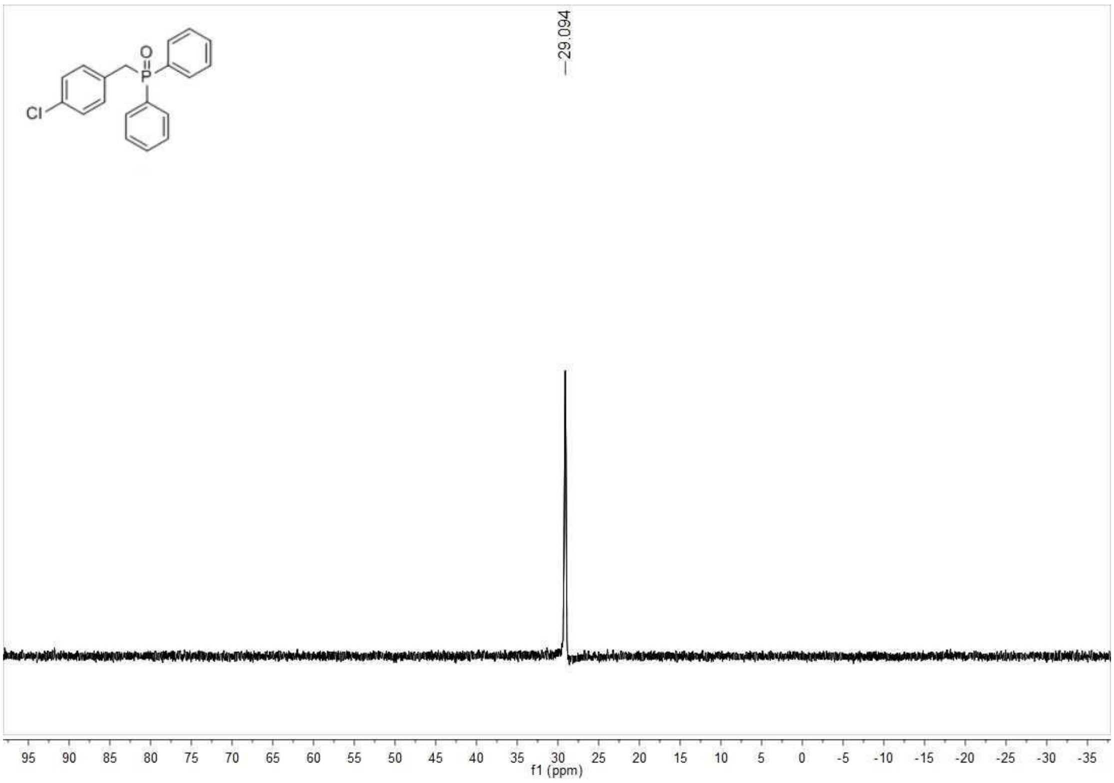

^{1}H NMR Spectrum of **196**

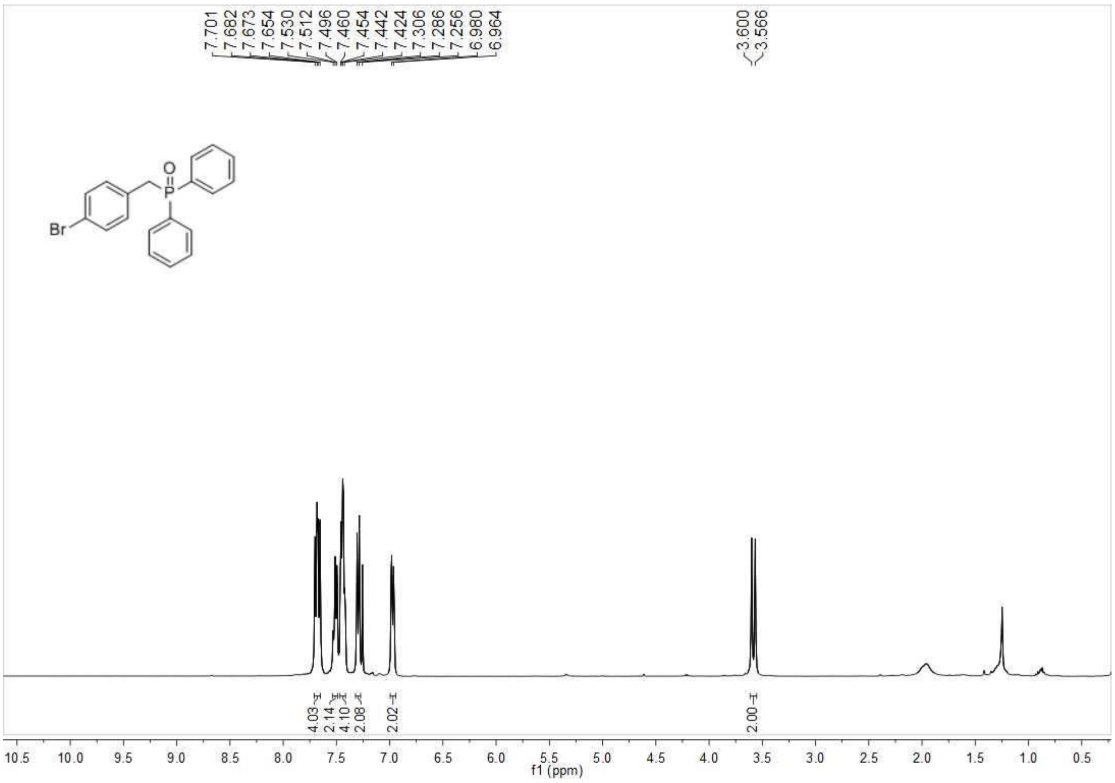

^{13}C NMR Spectrum of **196**

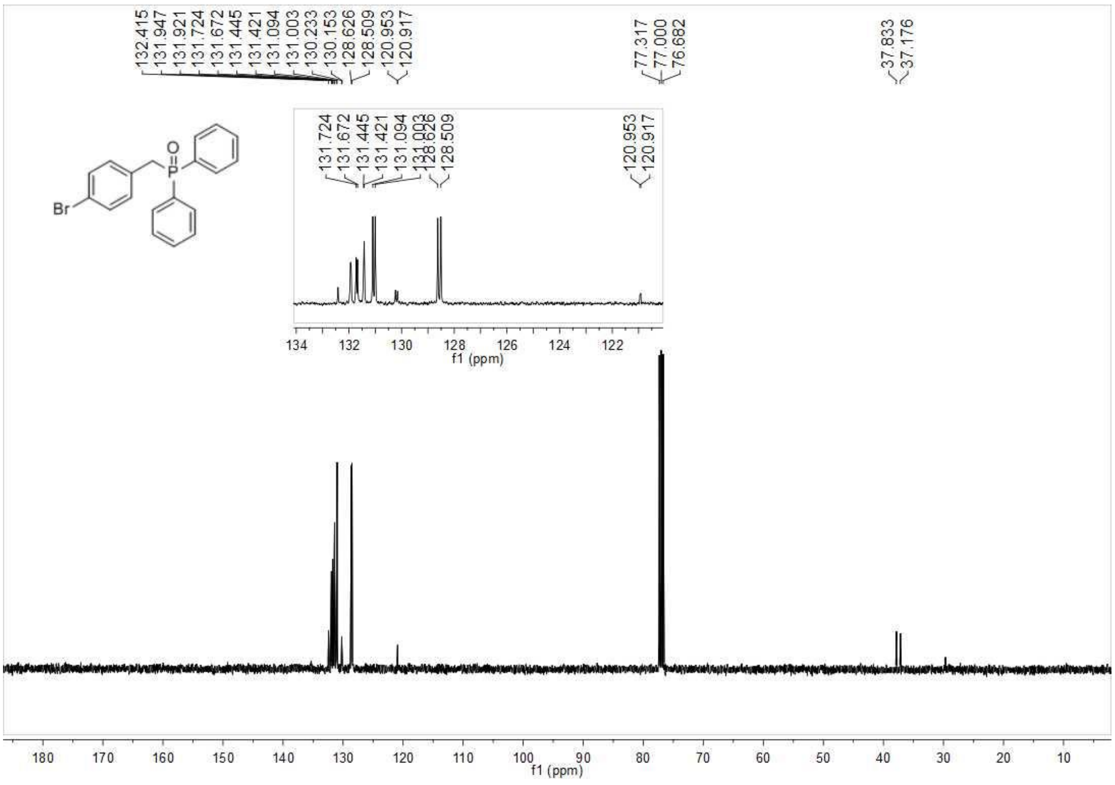

^{31}P NMR Spectrum of **196**

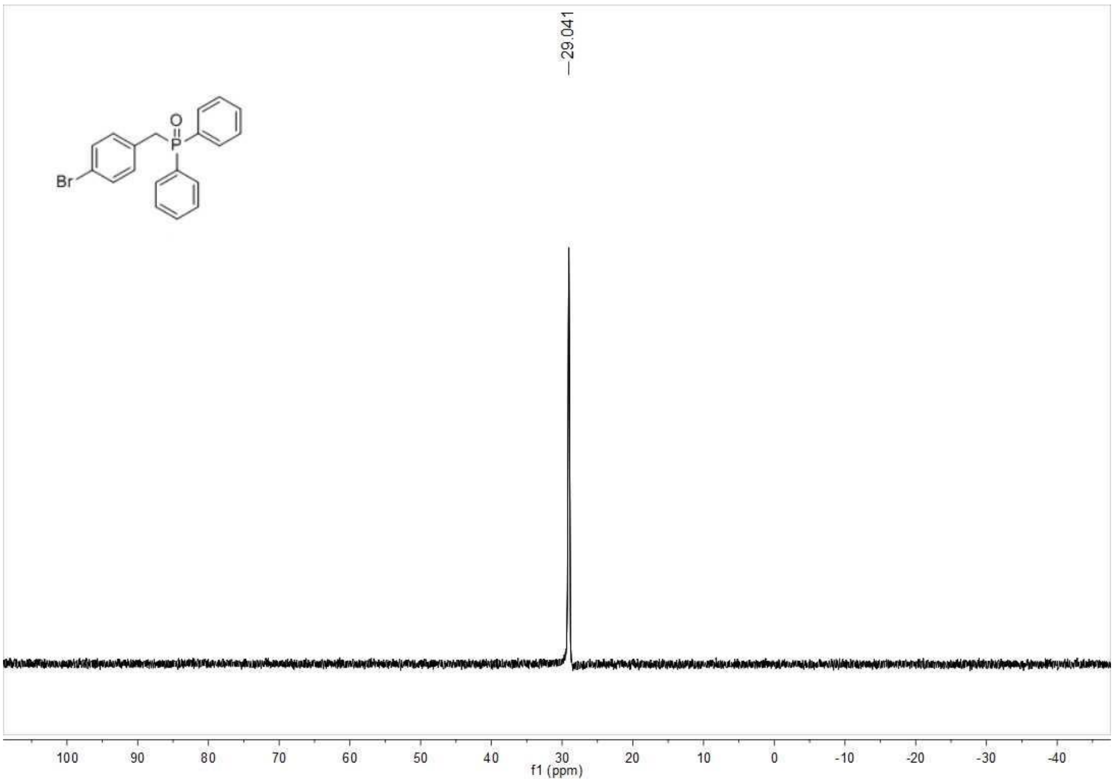

^{1}H NMR Spectrum of **197**

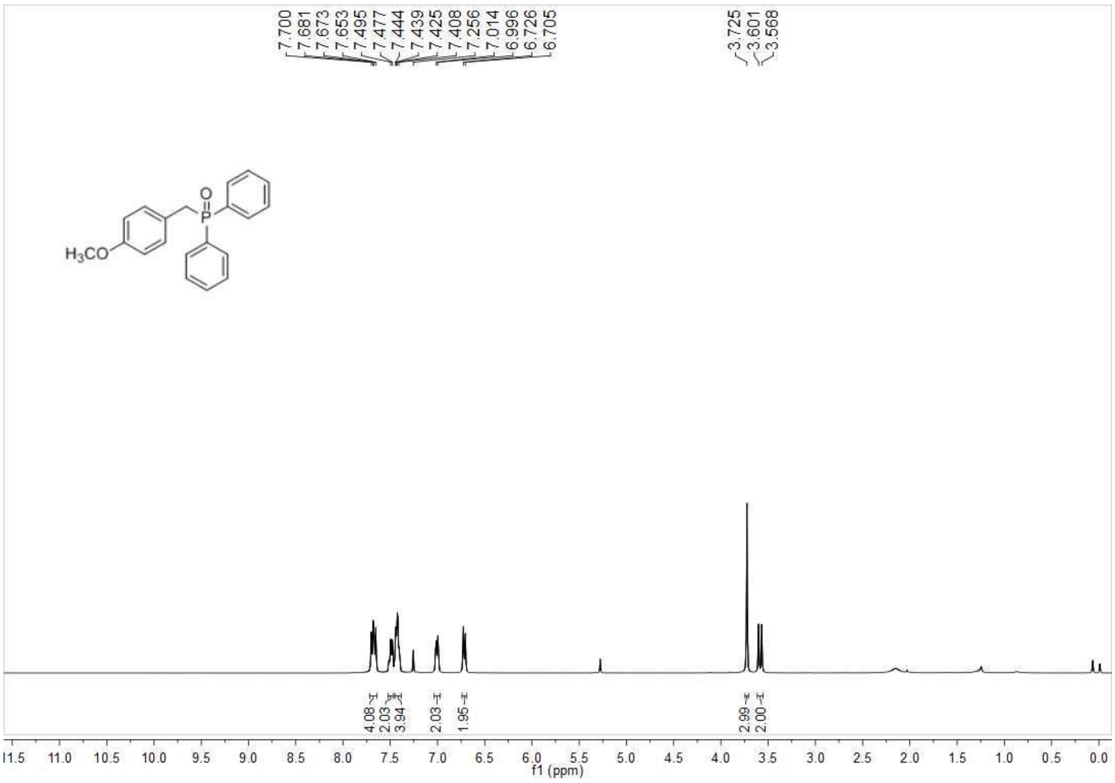

^{13}C NMR Spectrum of **197**

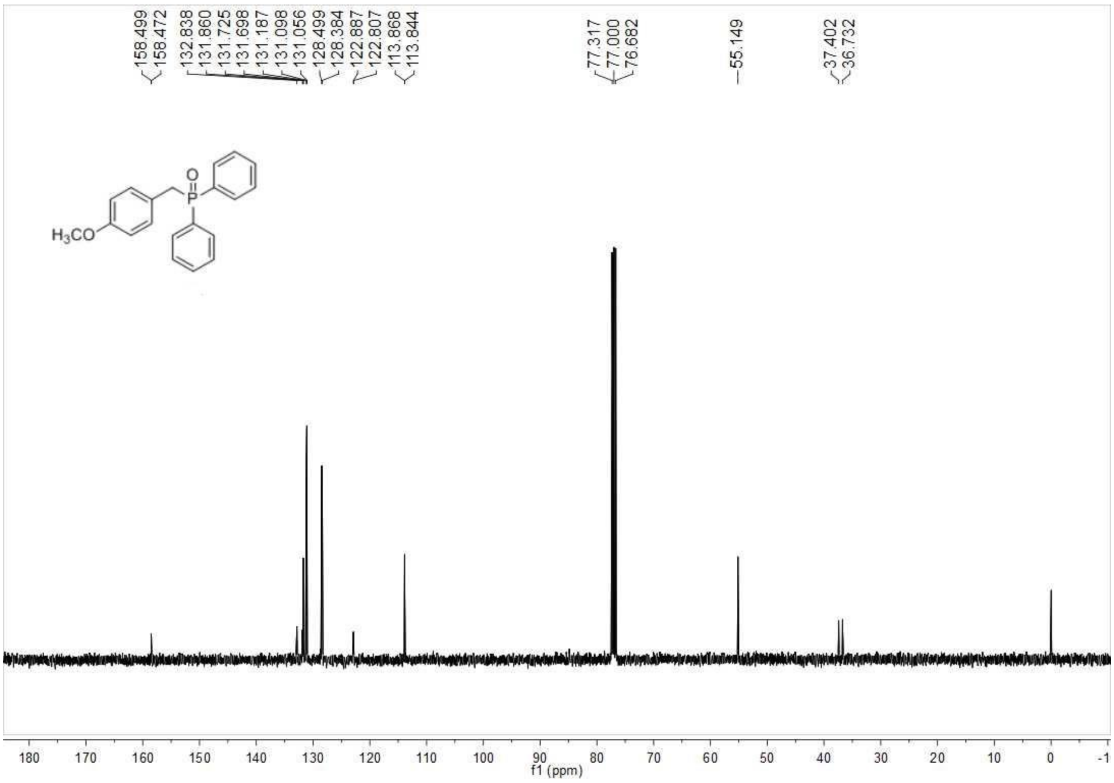

^{31}P NMR Spectrum of **197**

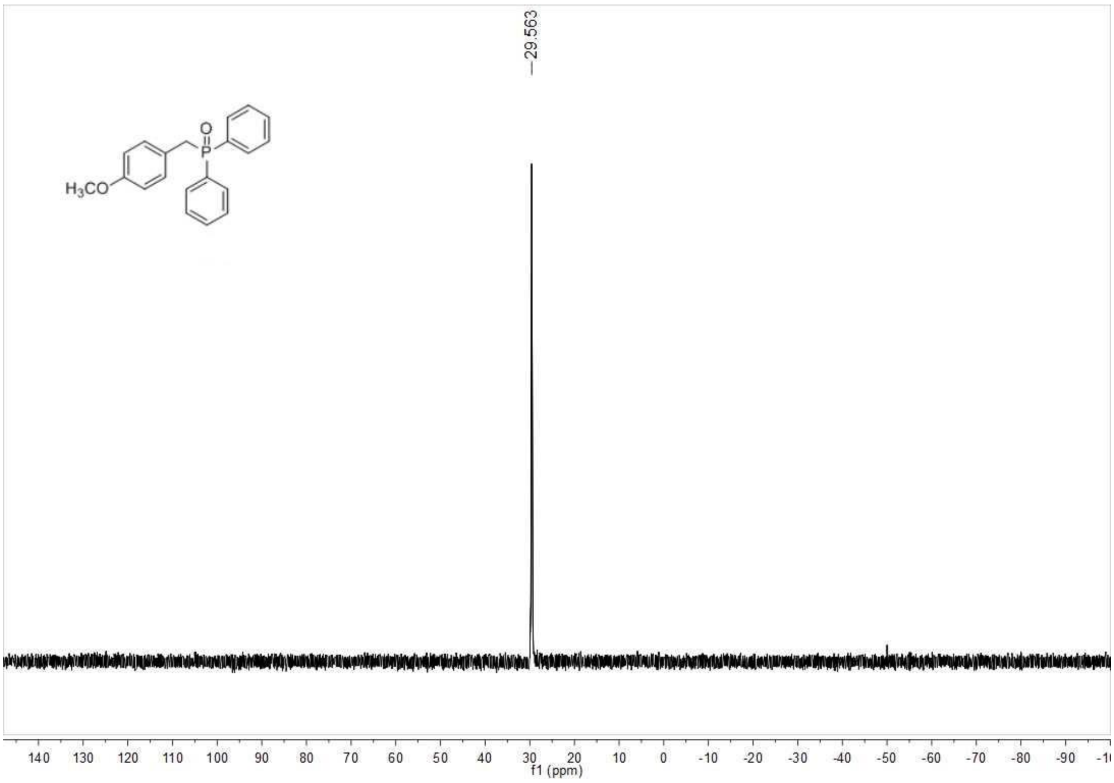

^{1}H NMR Spectrum of **198**

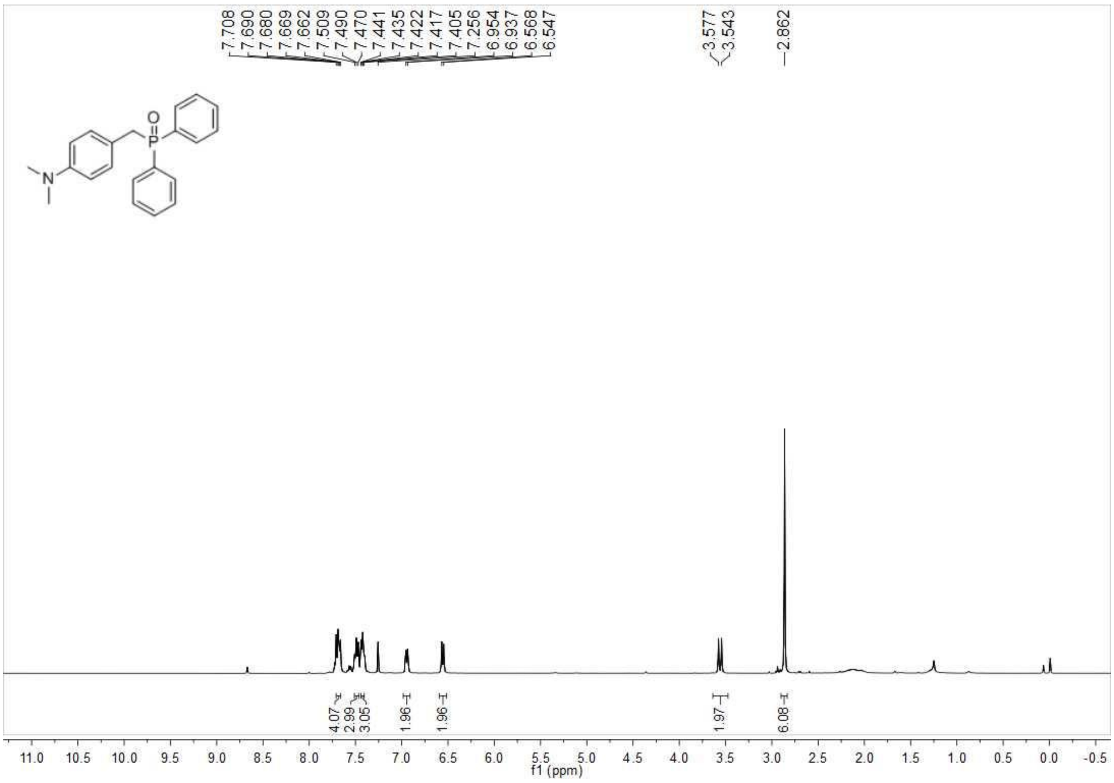

^{13}C NMR Spectrum of **198**

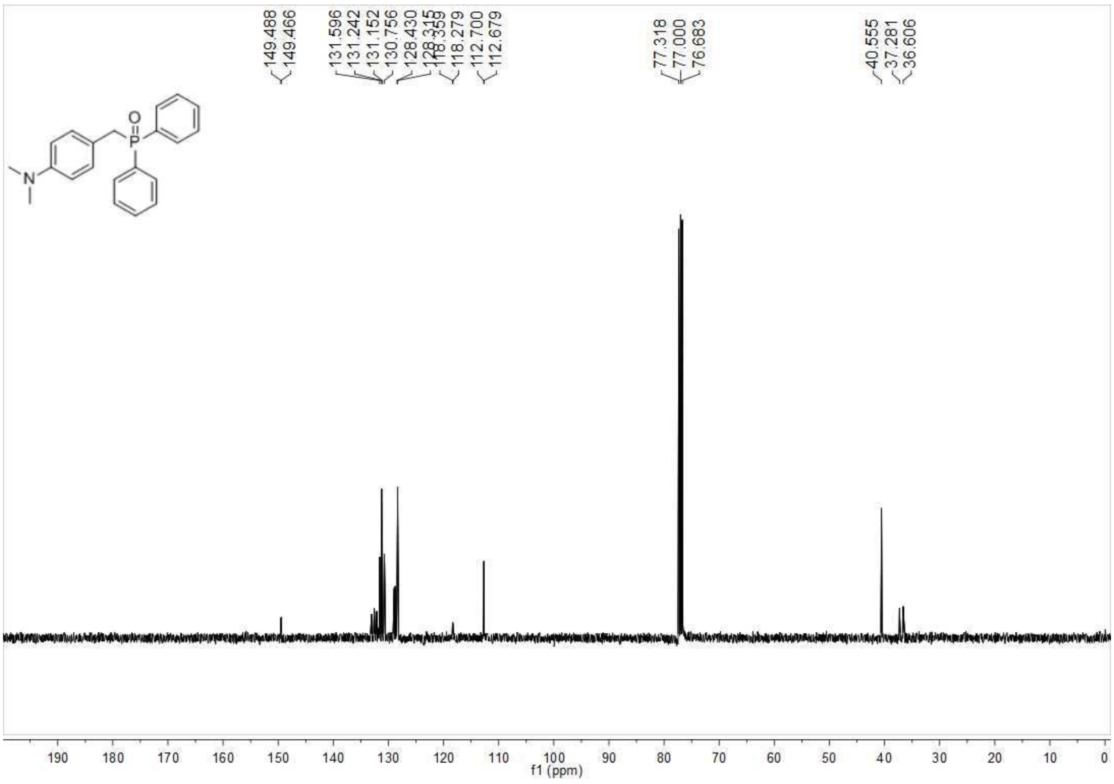

^{31}P NMR Spectrum of **198**

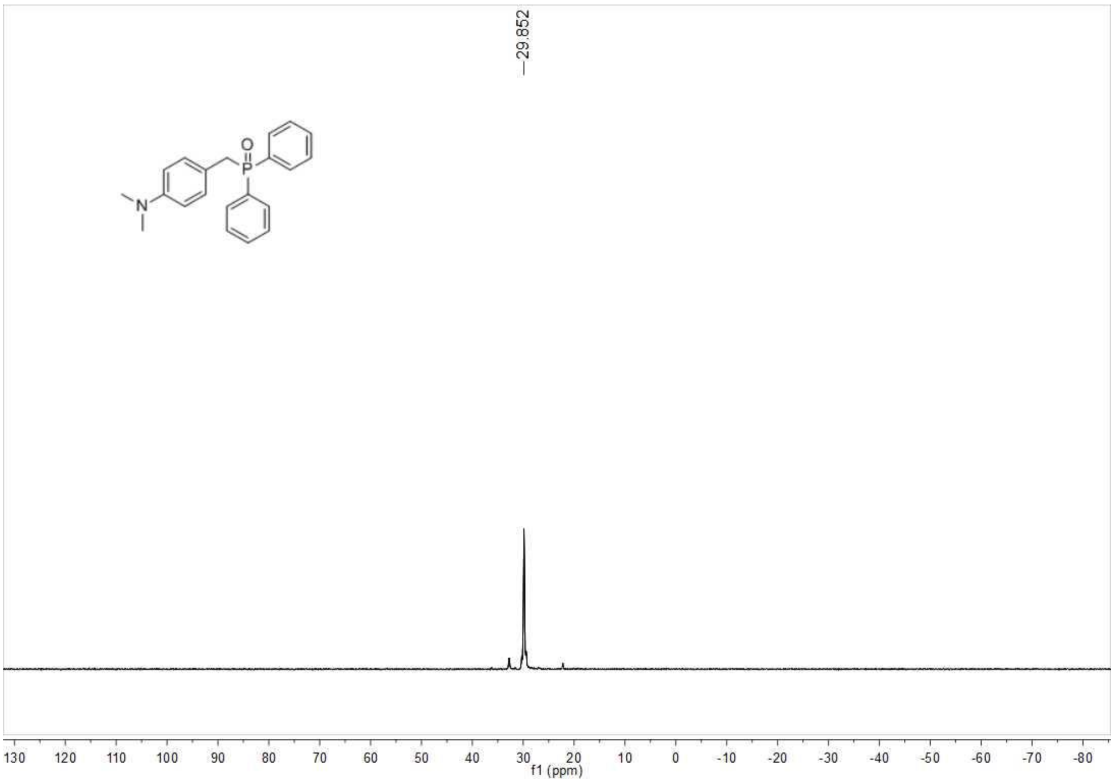

^{1}H NMR Spectrum of **199**

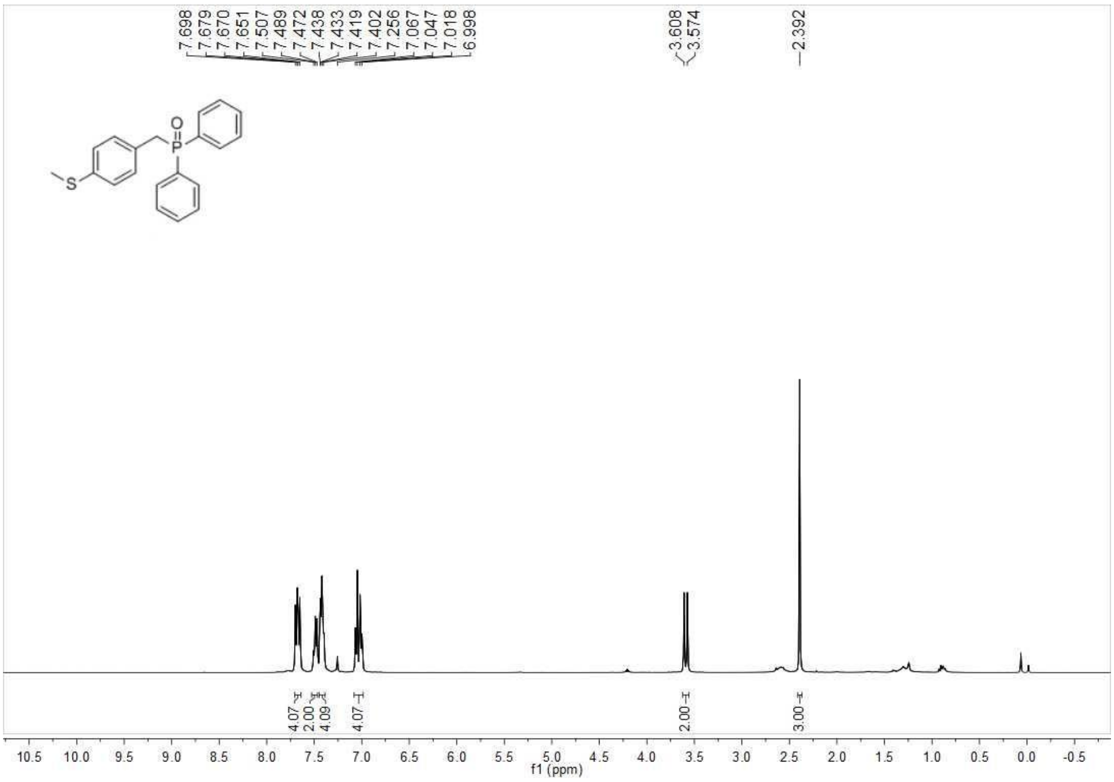

^{13}C NMR Spectrum of **199**

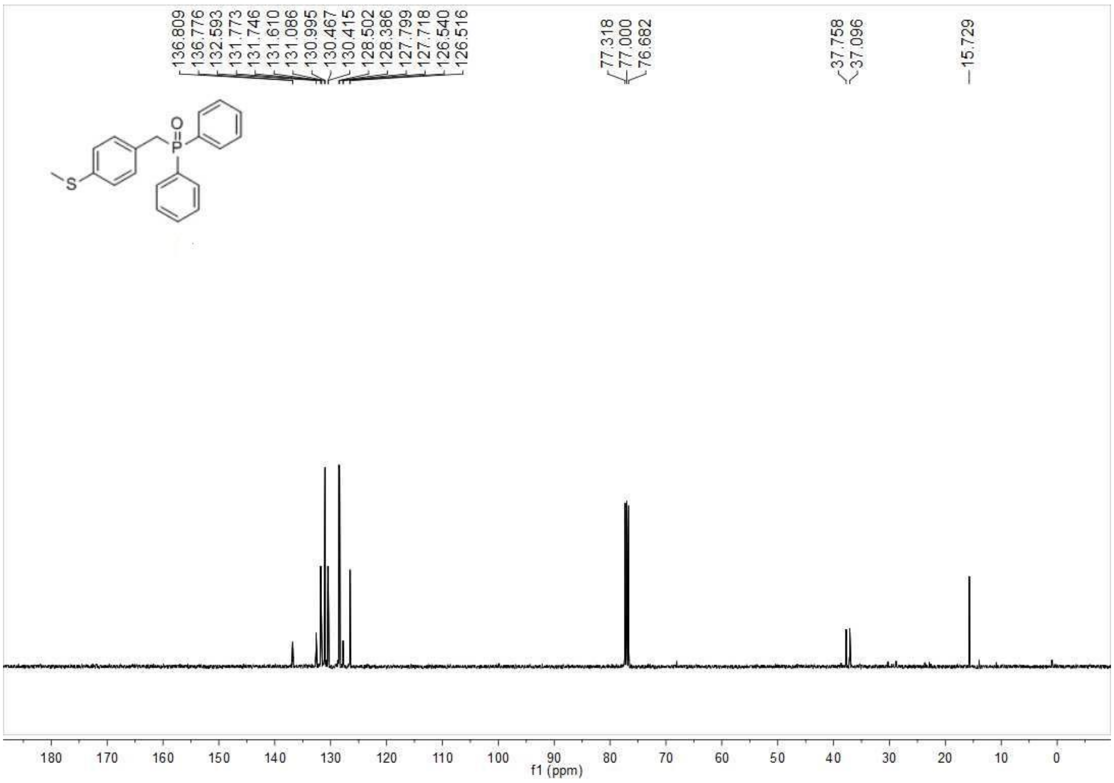

^{31}P NMR Spectrum of **199**

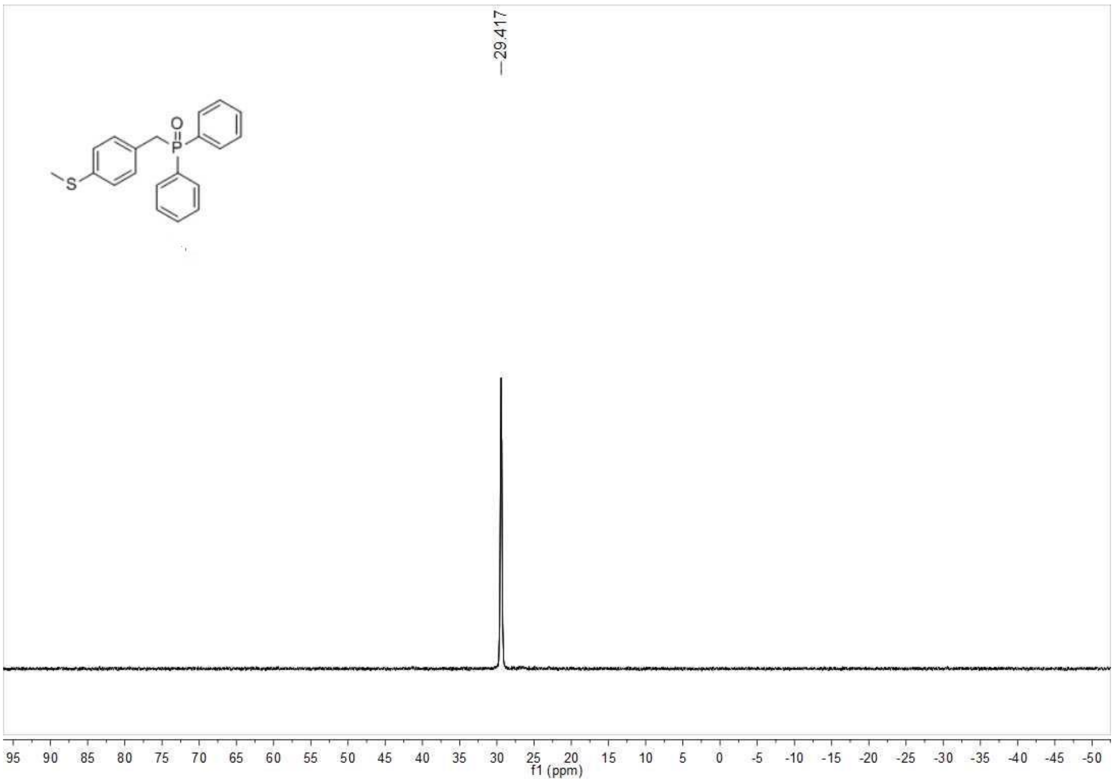

^{1}H NMR Spectrum of **200**

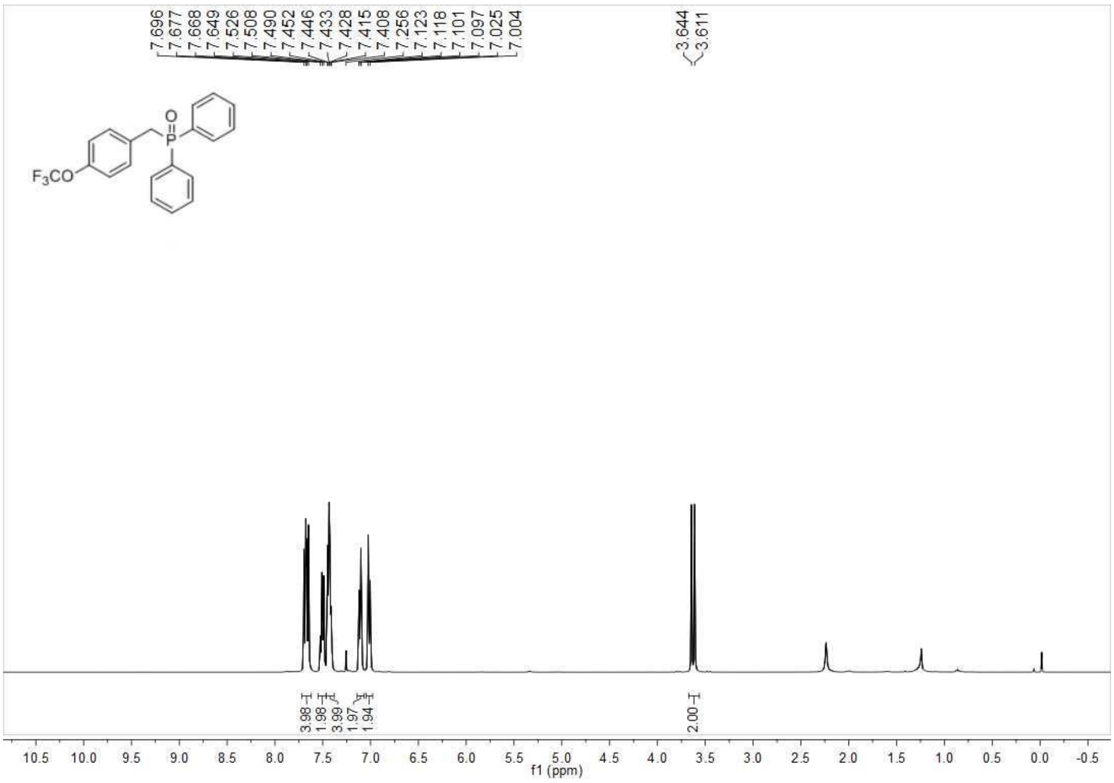

^{13}C NMR Spectrum of **200**

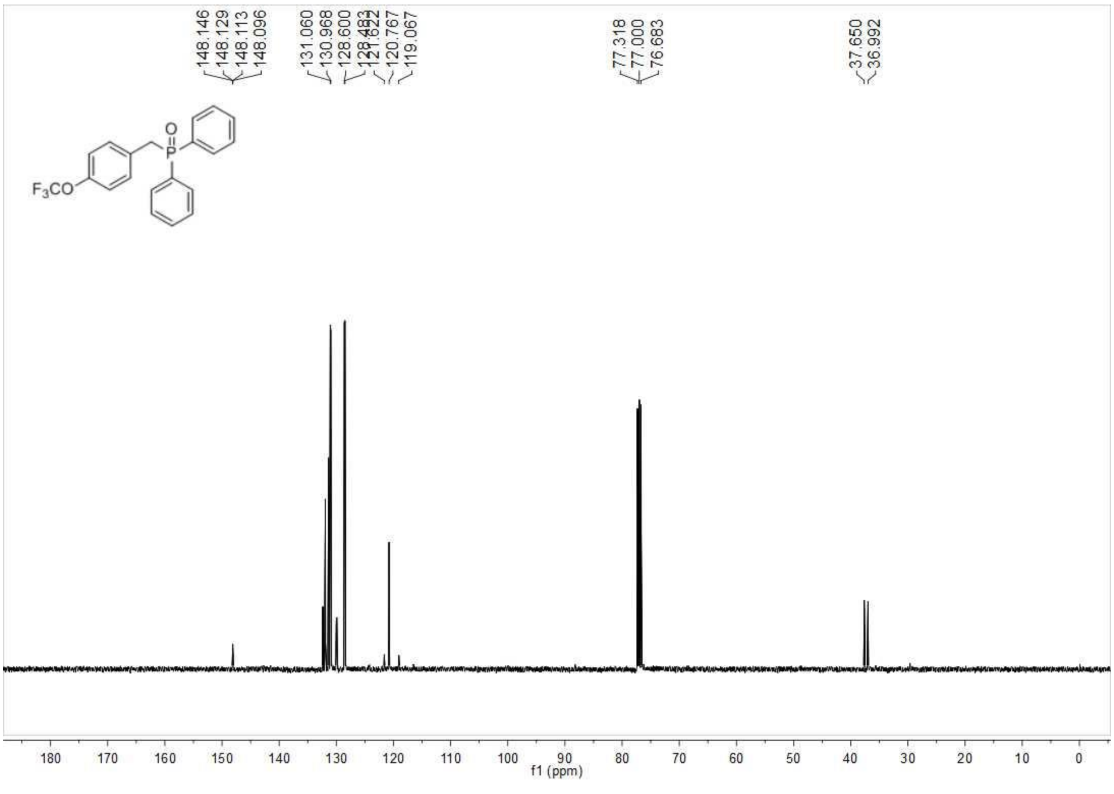

^{31}P NMR Spectrum of **200**

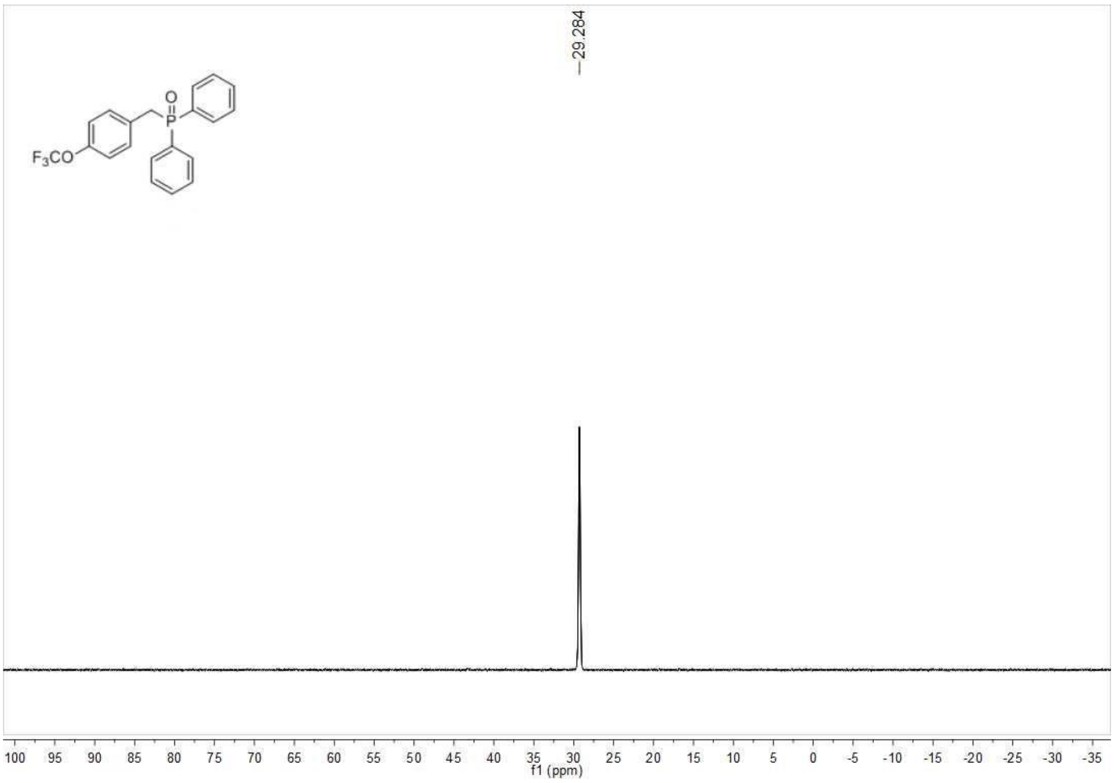

^{19}F NMR Spectrum of **200**

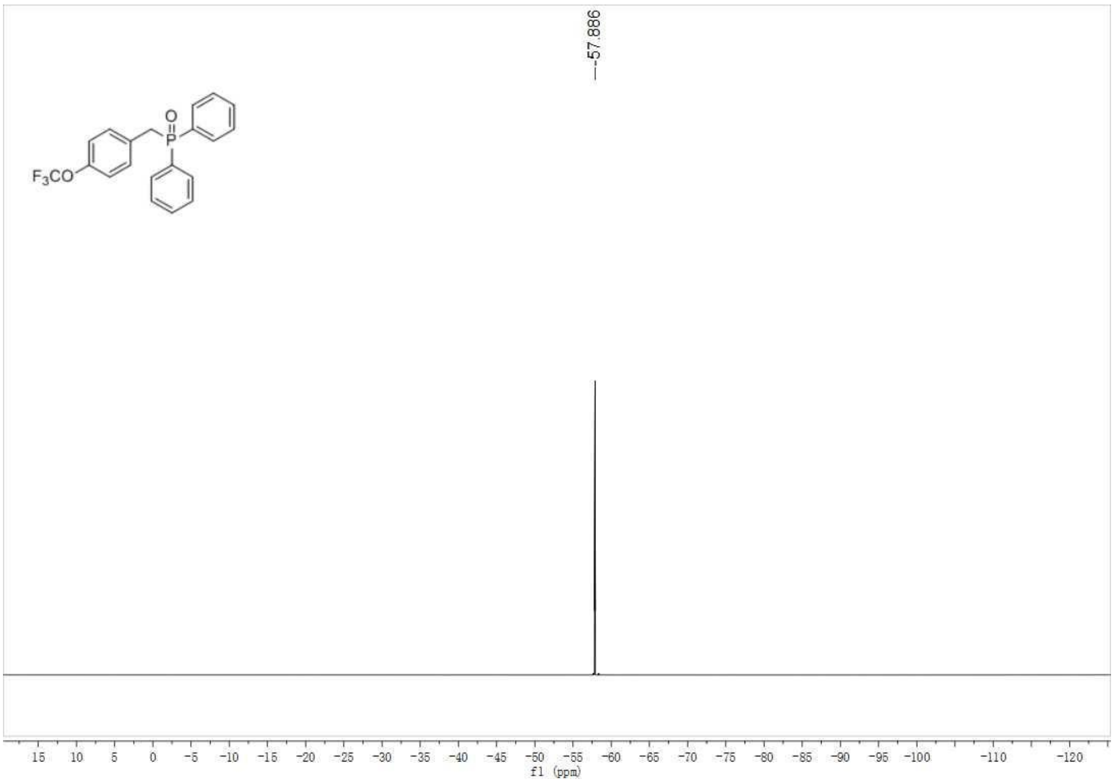

^{1}H NMR Spectrum of **201**

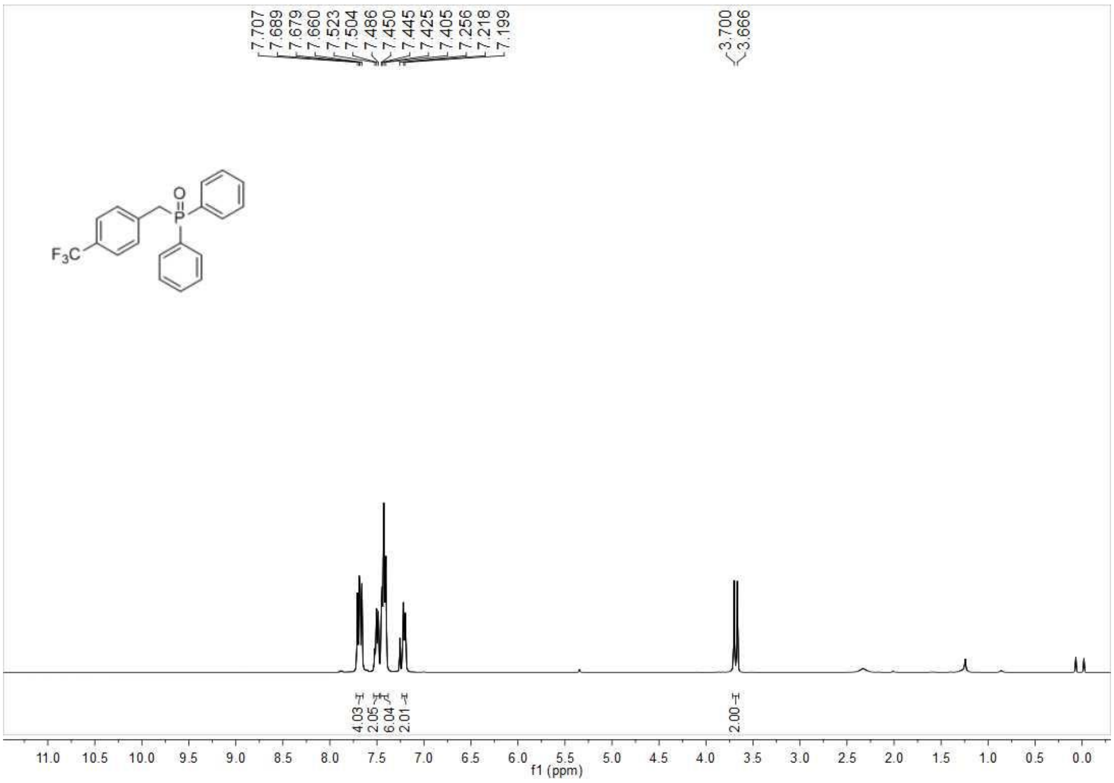

^{13}C NMR Spectrum of **201**

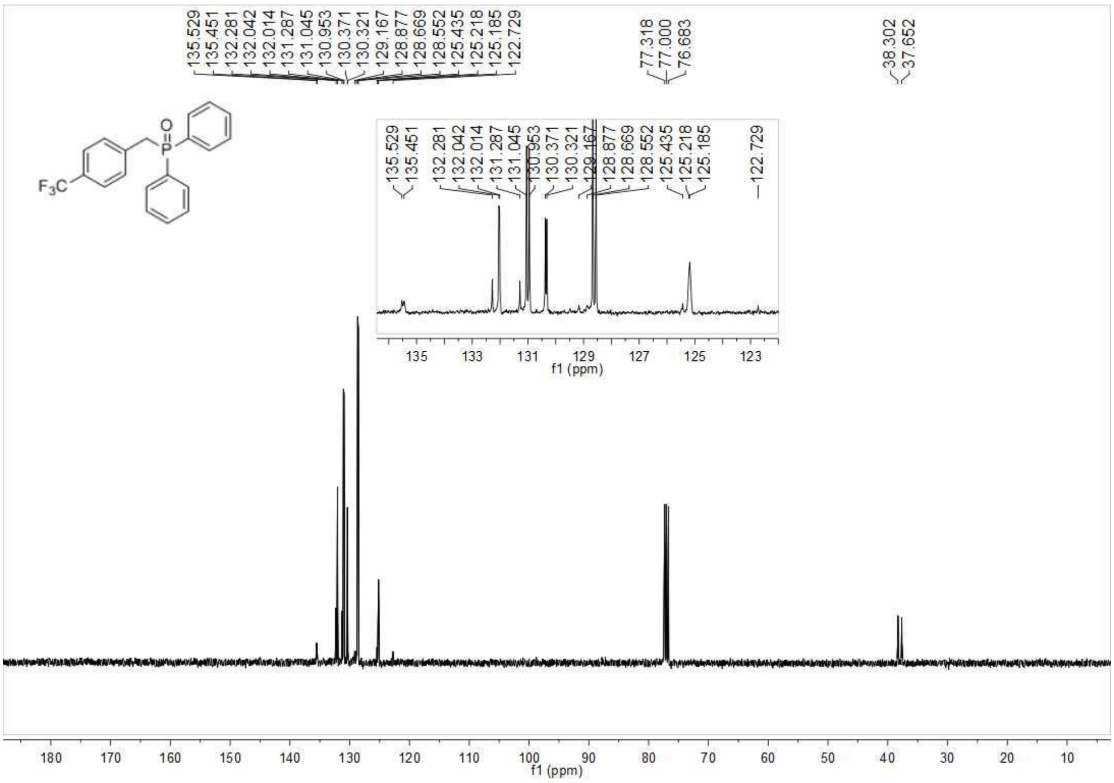

^{31}P NMR Spectrum of **201**

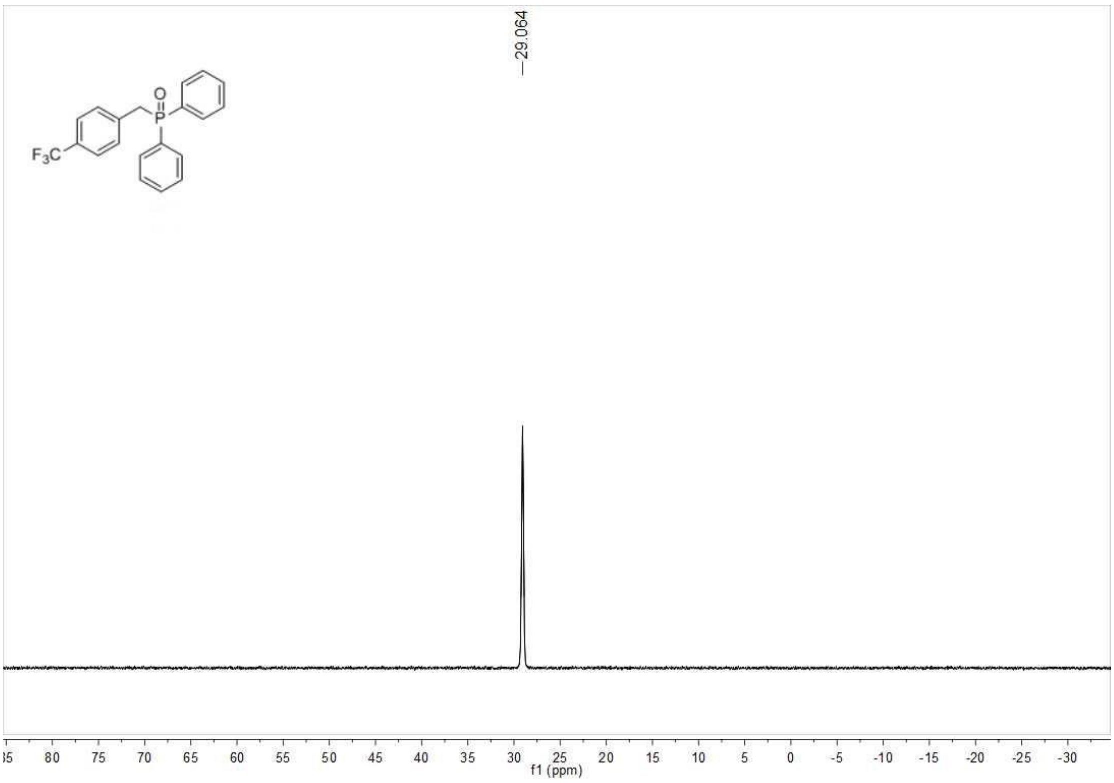

^{19}F NMR Spectrum of **201**

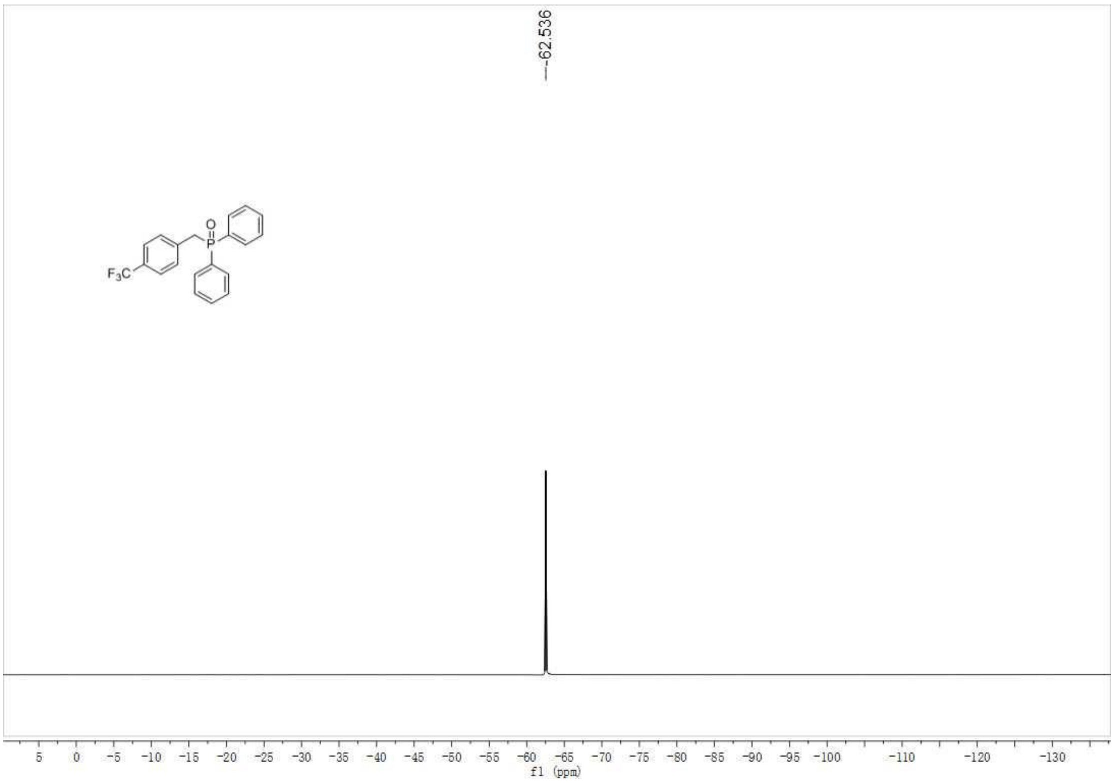

^{1}H NMR Spectrum of **202**

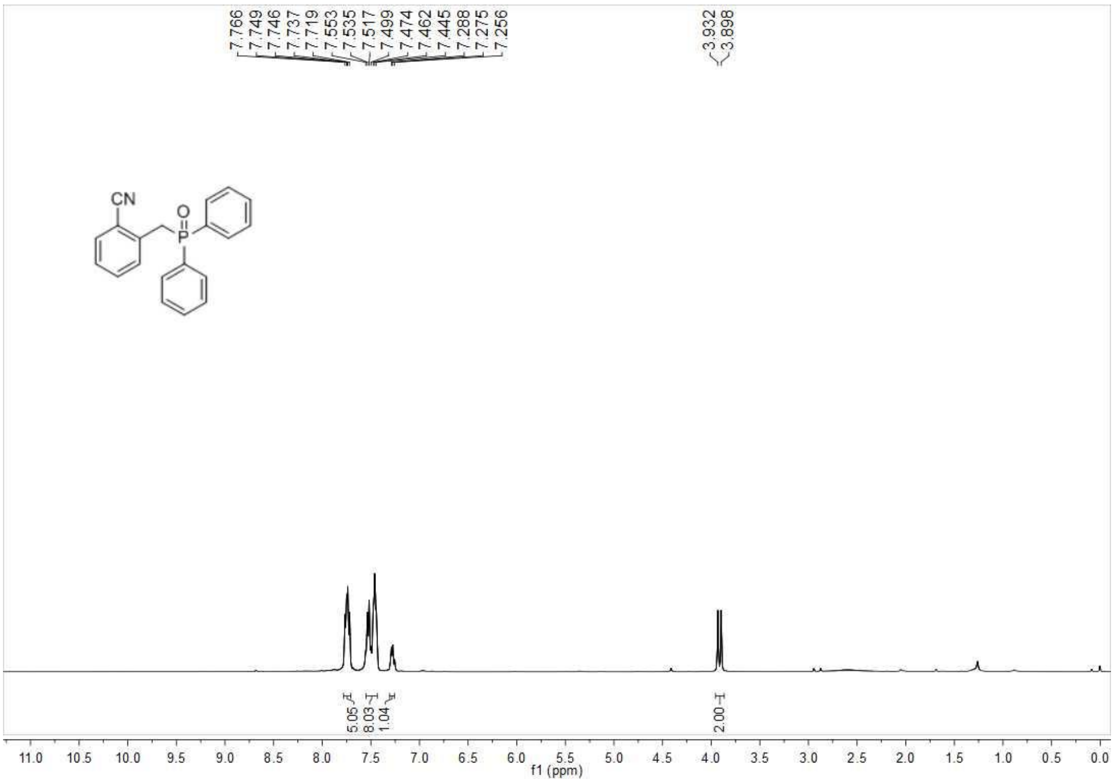

^{13}C NMR Spectrum of **202**

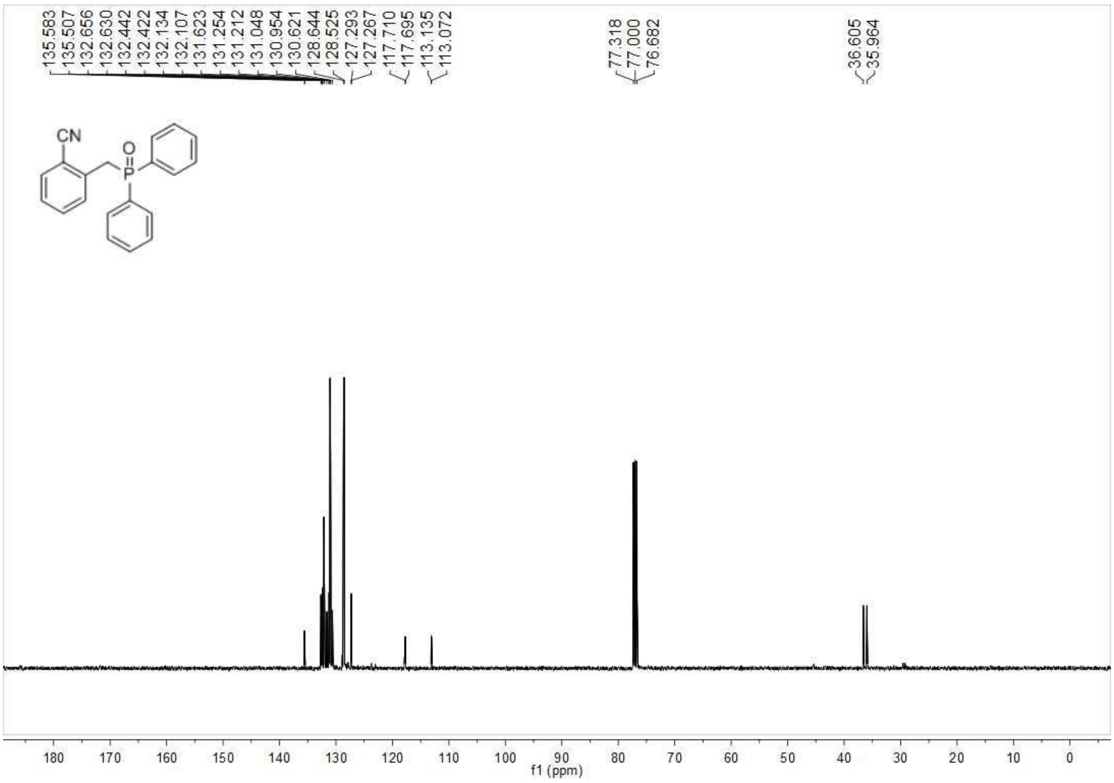

^{31}P NMR Spectrum of **202**

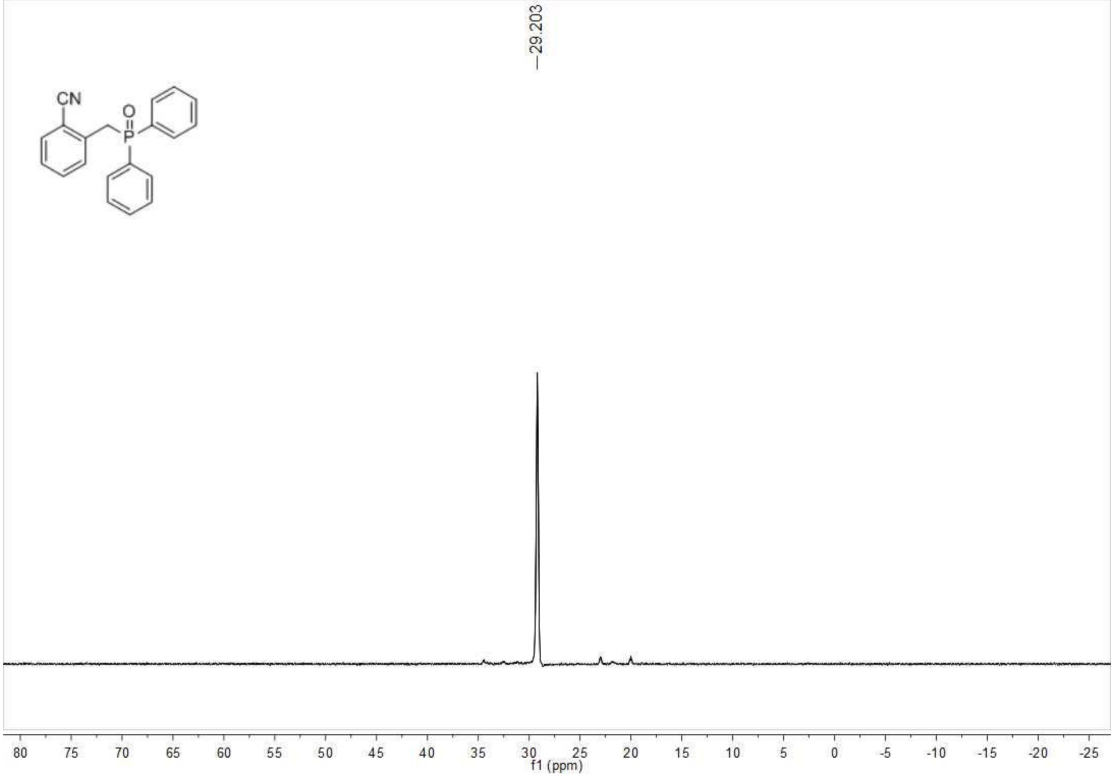

^{1}H NMR Spectrum of **203**

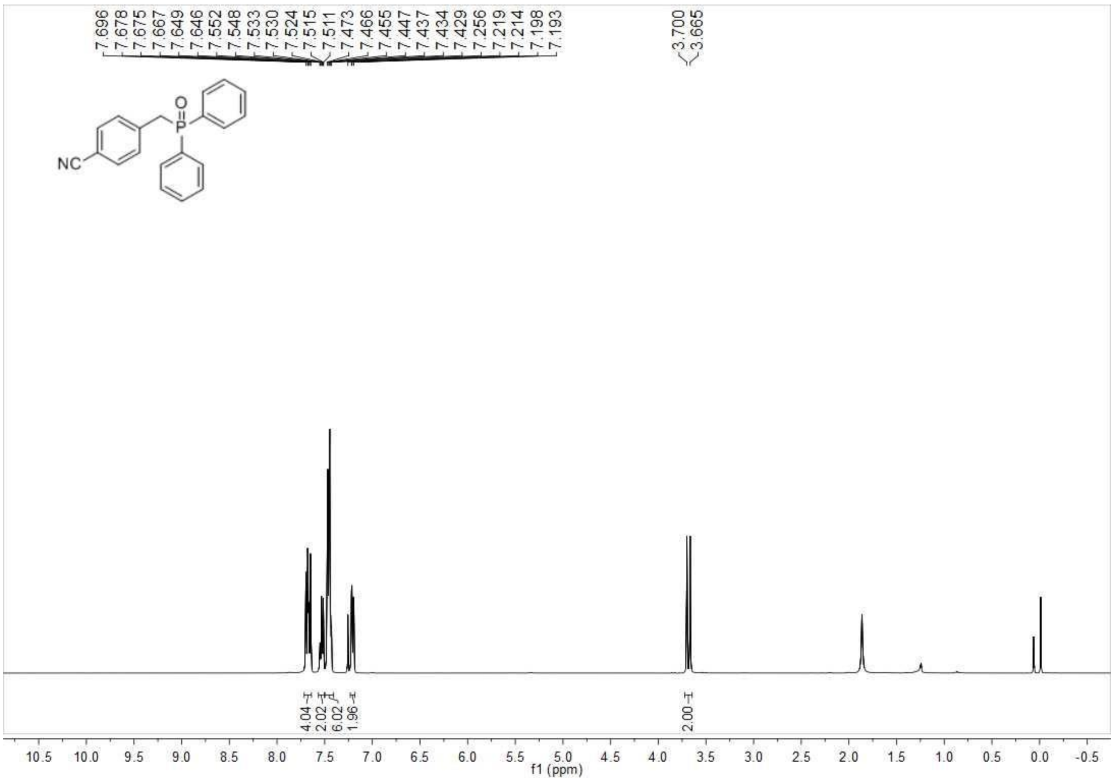

^{13}C NMR Spectrum of **203**

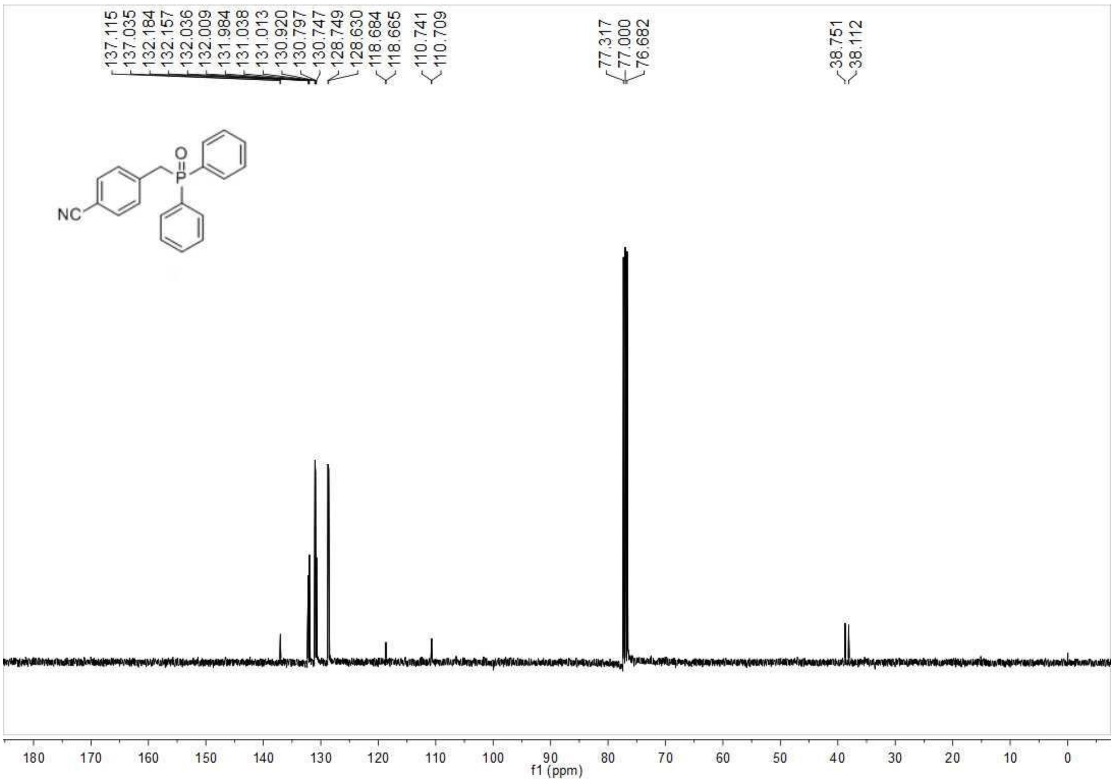

^{31}P NMR Spectrum of **203**

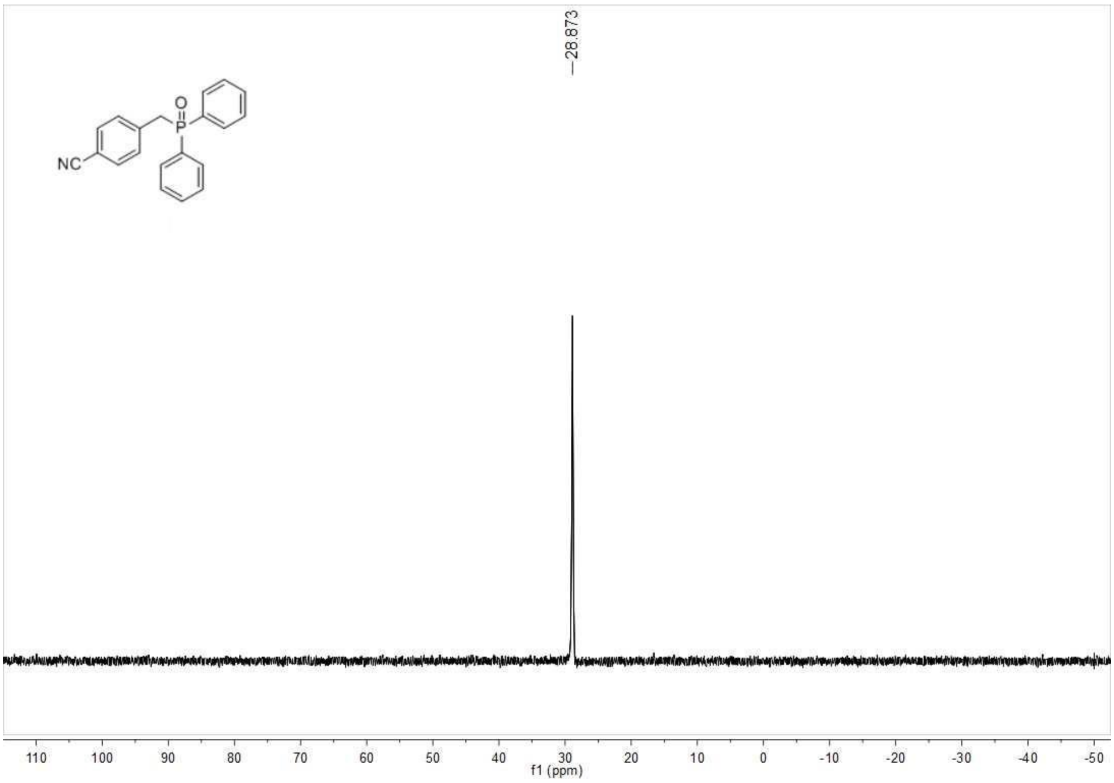

^{1}H NMR Spectrum of **204**

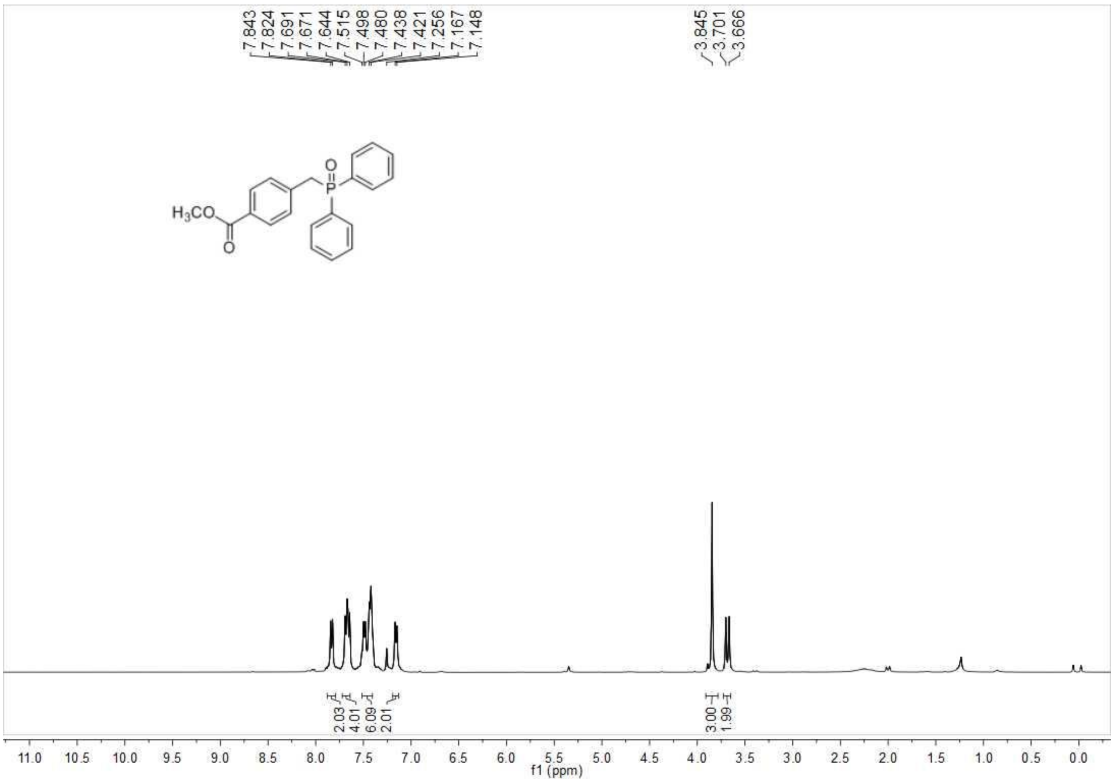

^{13}C NMR Spectrum of **204**

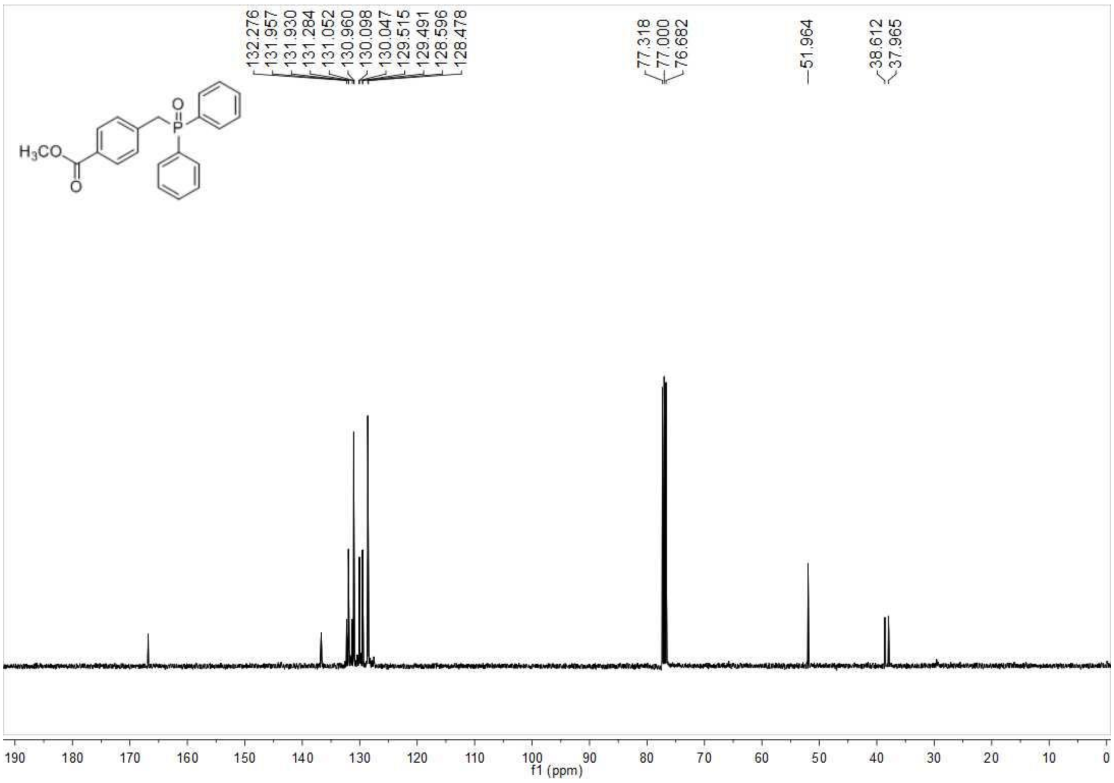

^{31}P NMR Spectrum of **204**

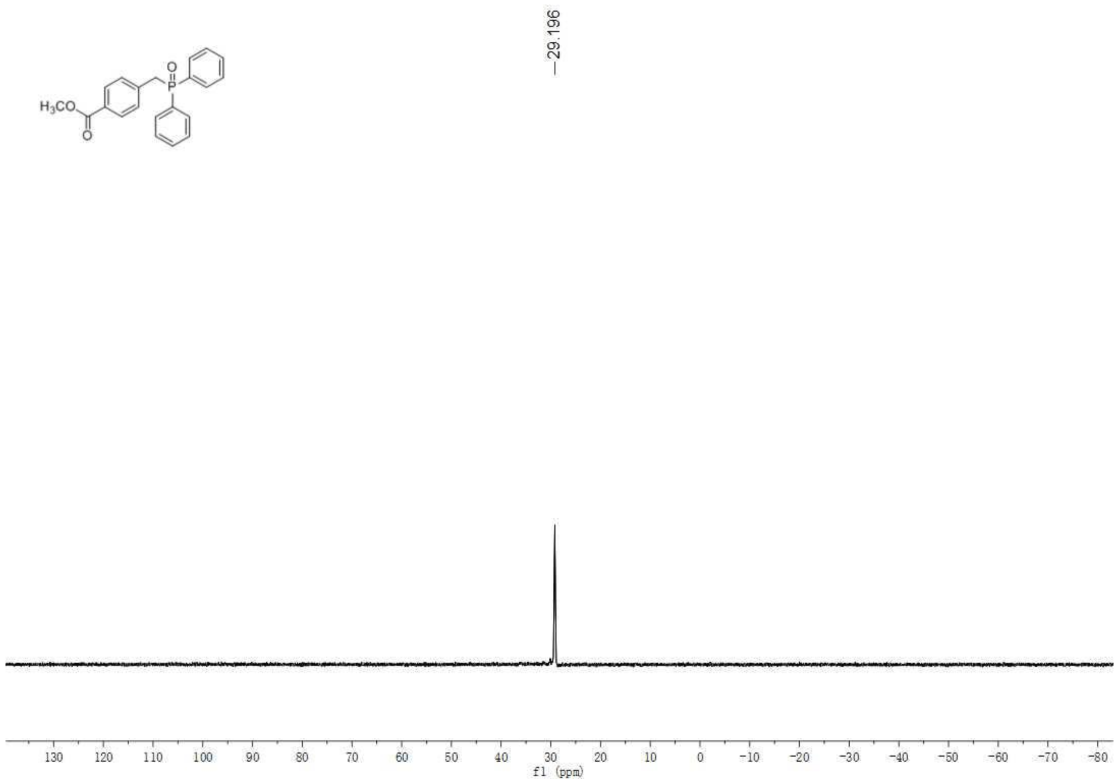

^{1}H NMR Spectrum of **205**

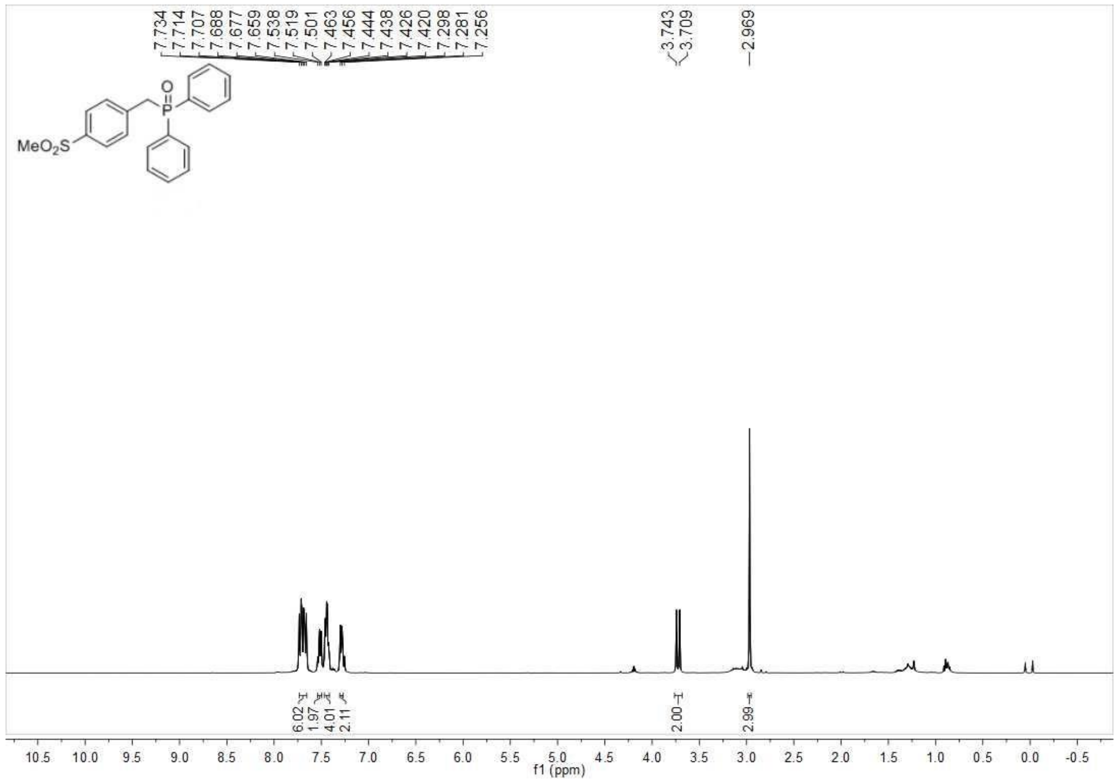

^{13}C NMR Spectrum of **205**

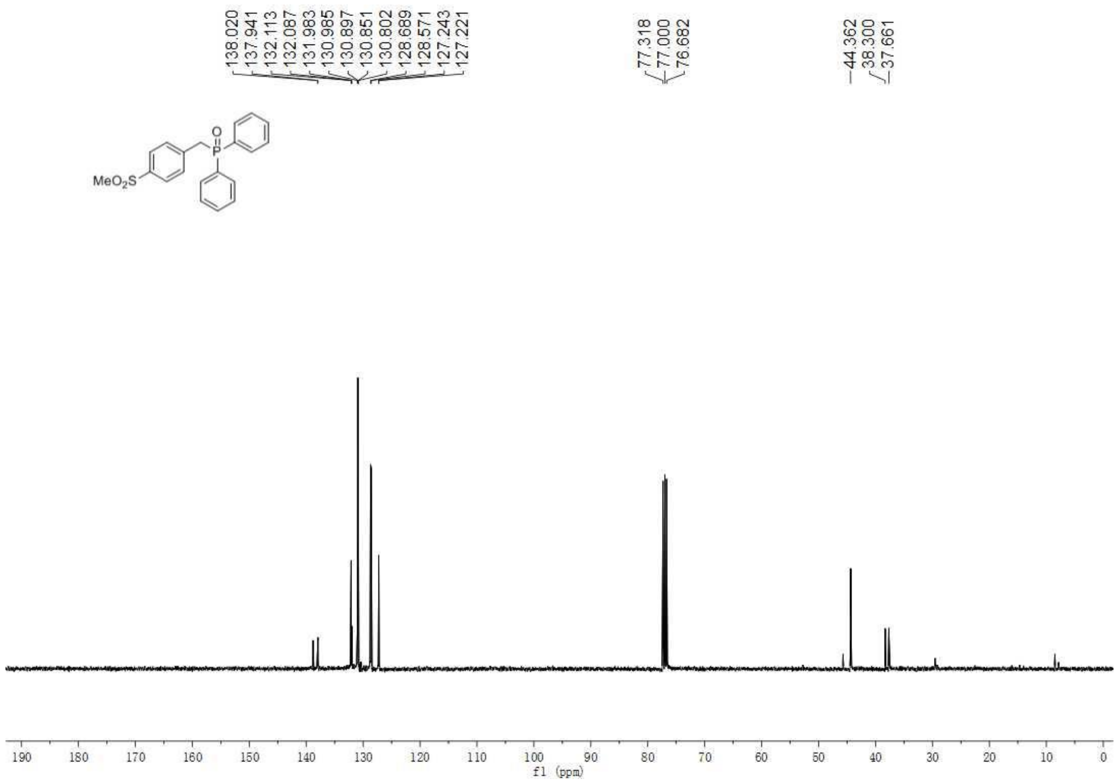

^{31}P NMR Spectrum of **205**

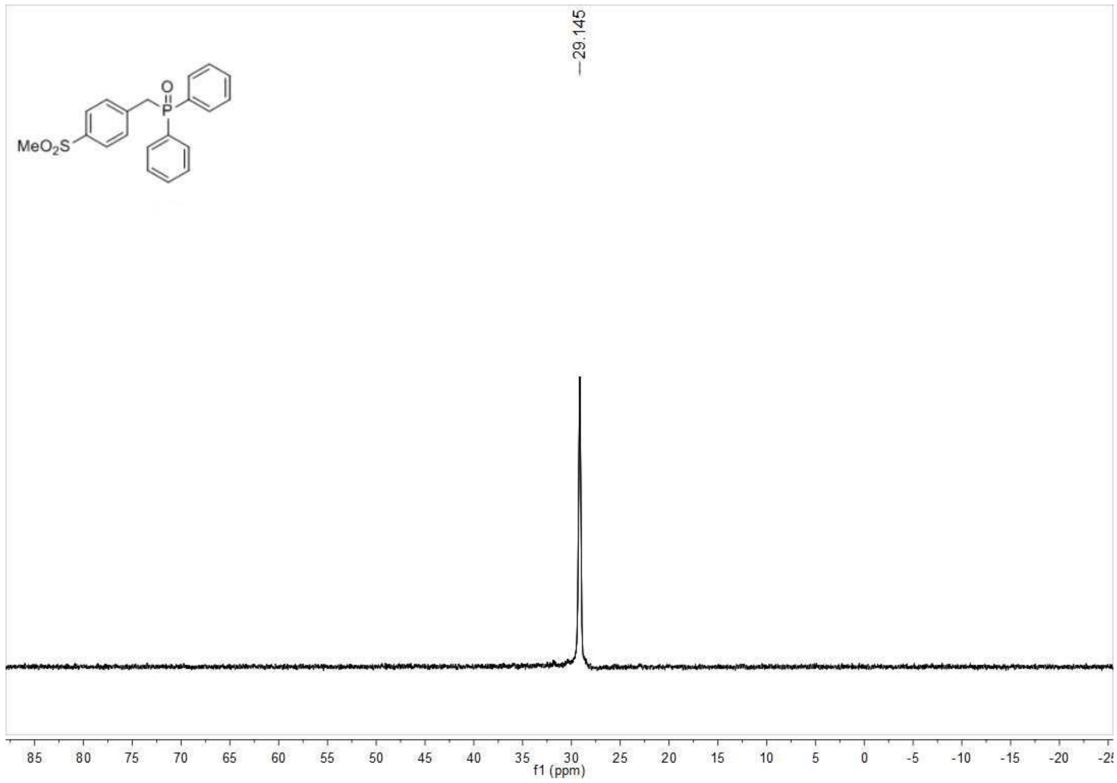

^{1}H NMR Spectrum of **206**

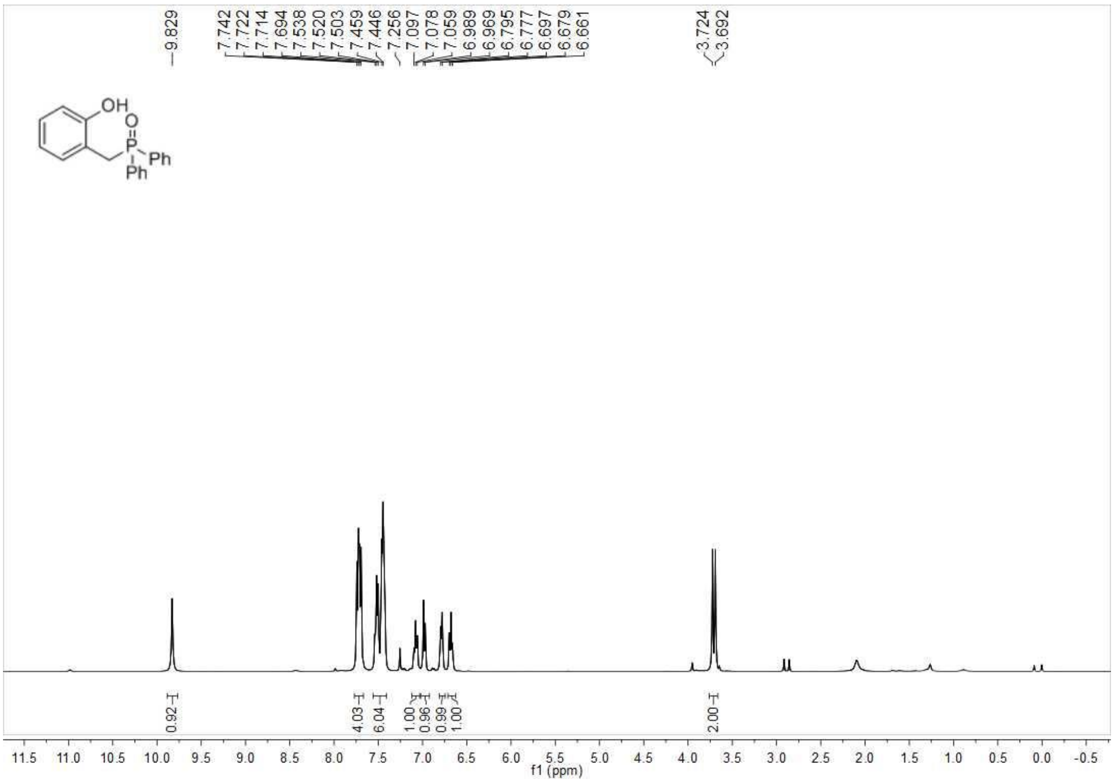

^{13}C NMR Spectrum of **206**

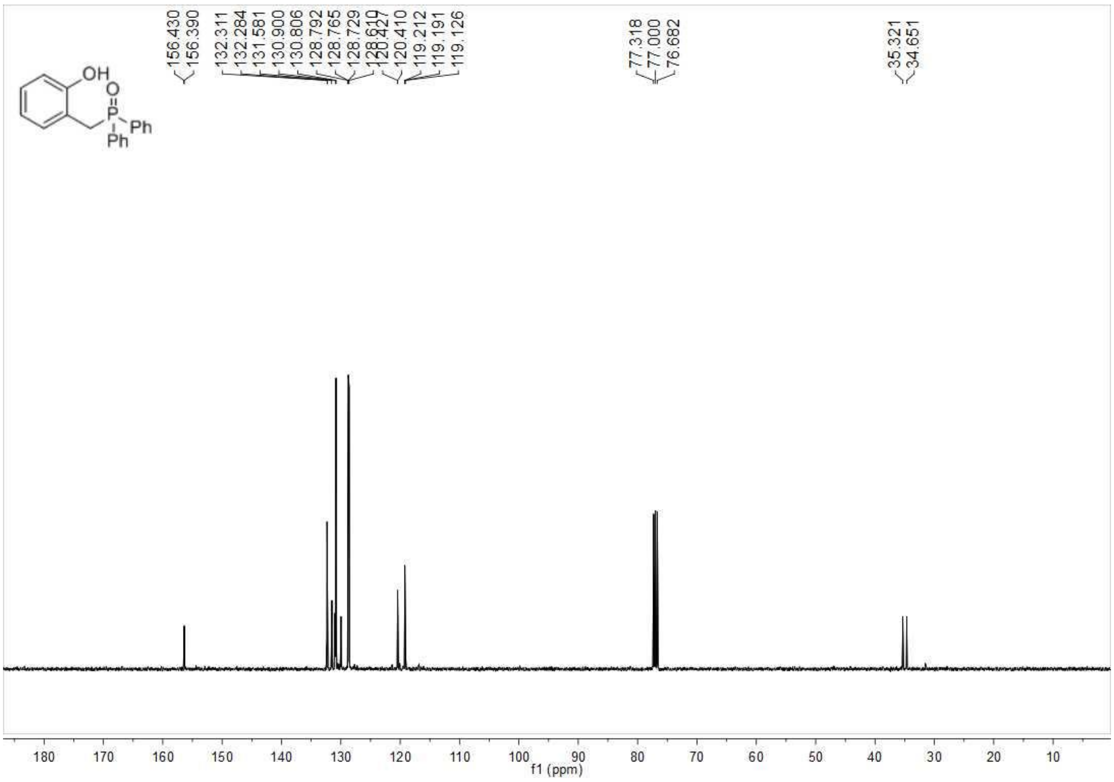

^{31}P NMR Spectrum of **206**

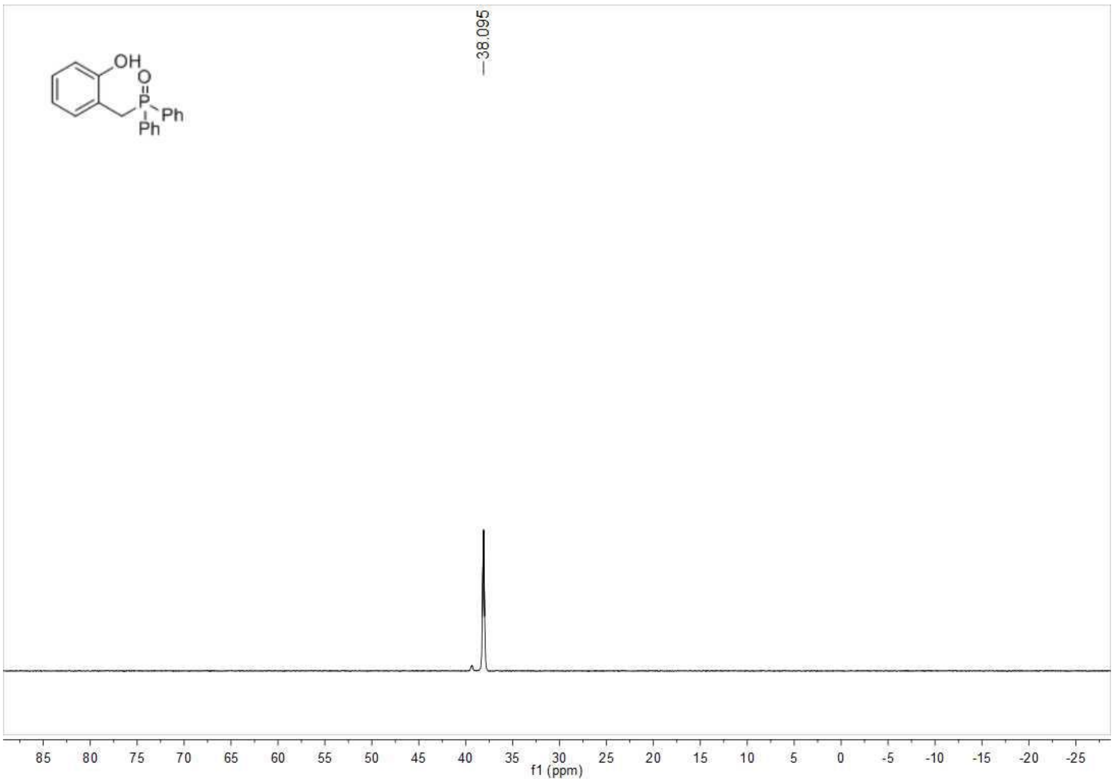

^{1}H NMR Spectrum of **207**

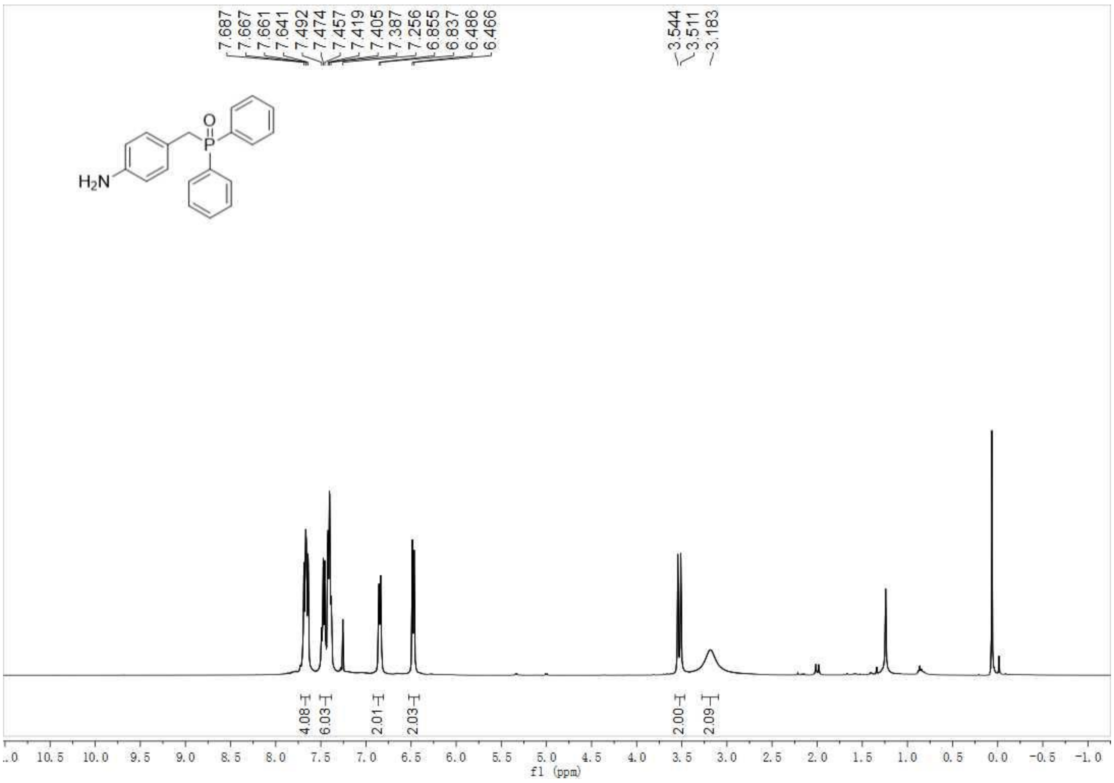

^{13}C NMR Spectrum of **207**

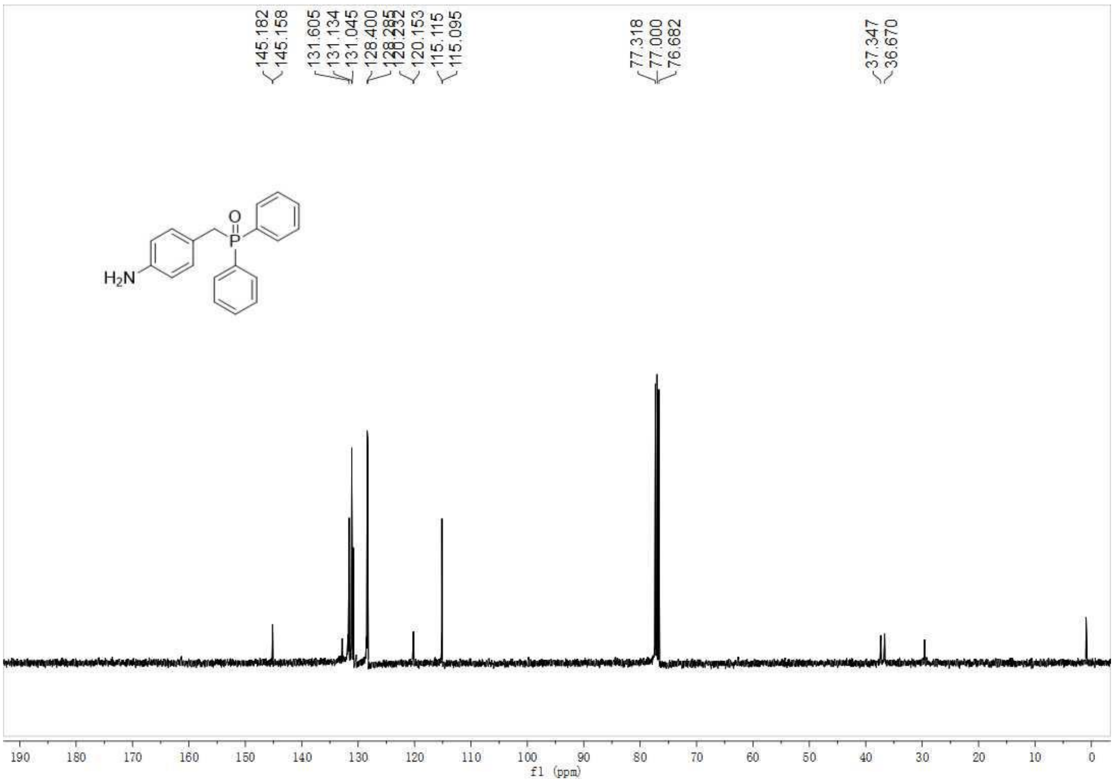

^{31}P NMR Spectrum of **207**

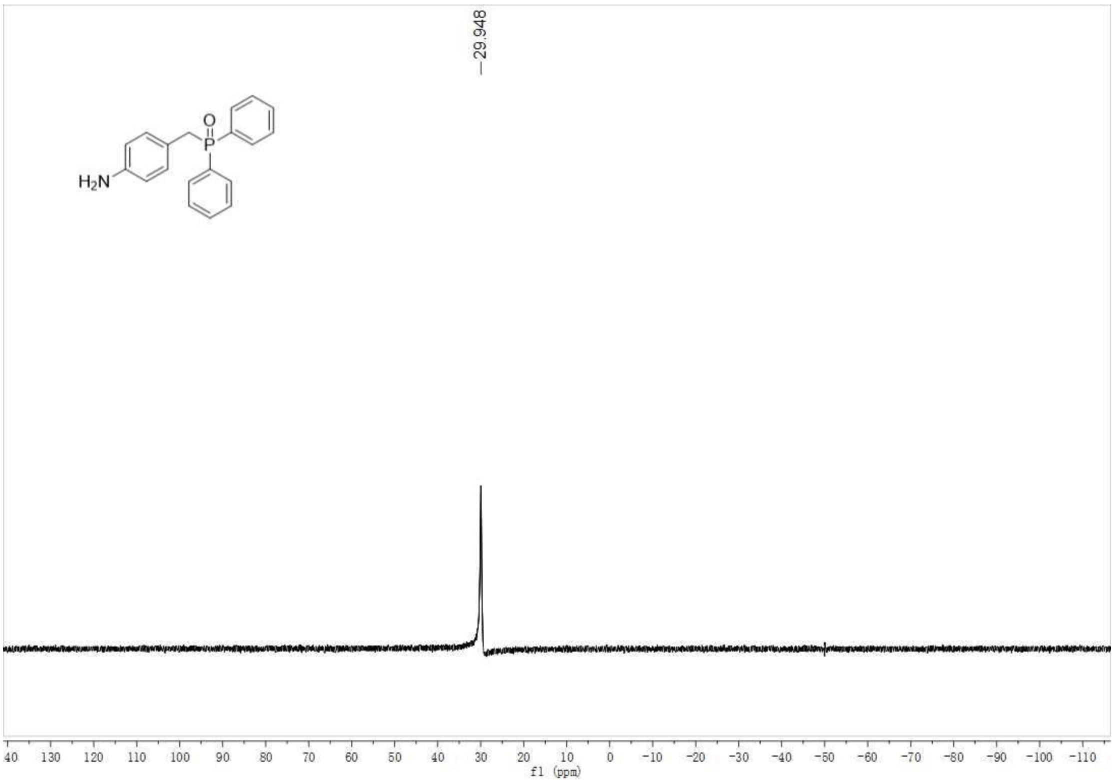

^{1}H NMR Spectrum of **208**

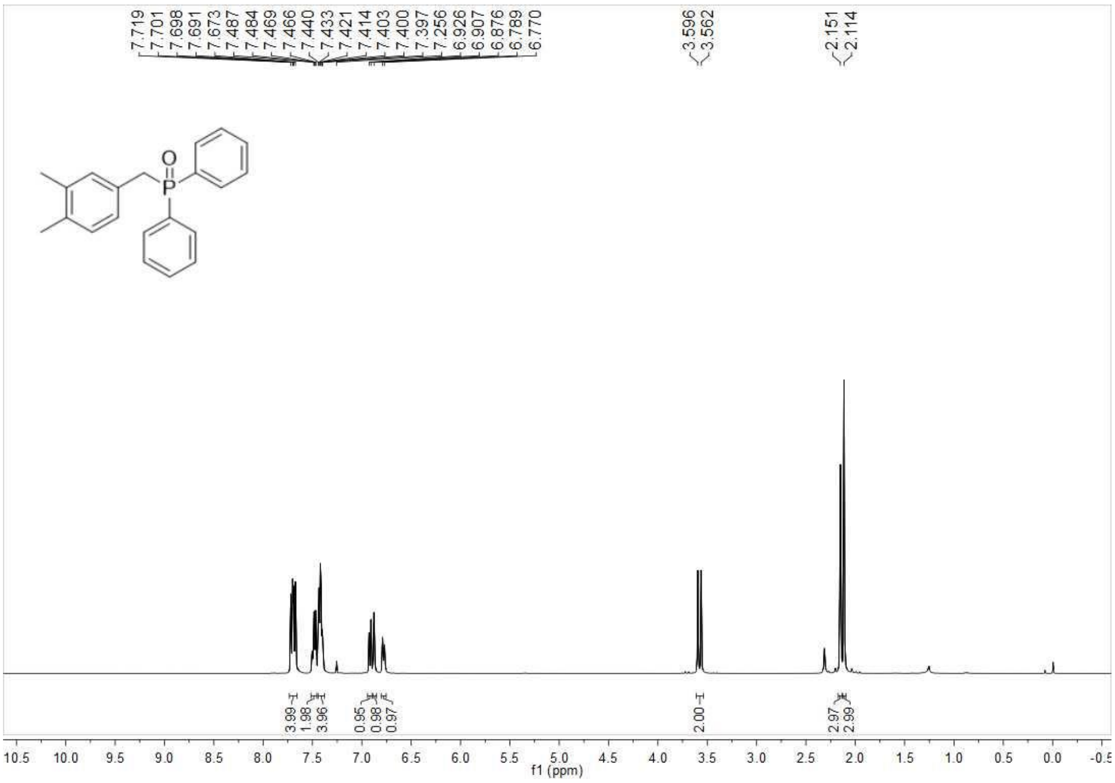

^{13}C NMR Spectrum of **208**

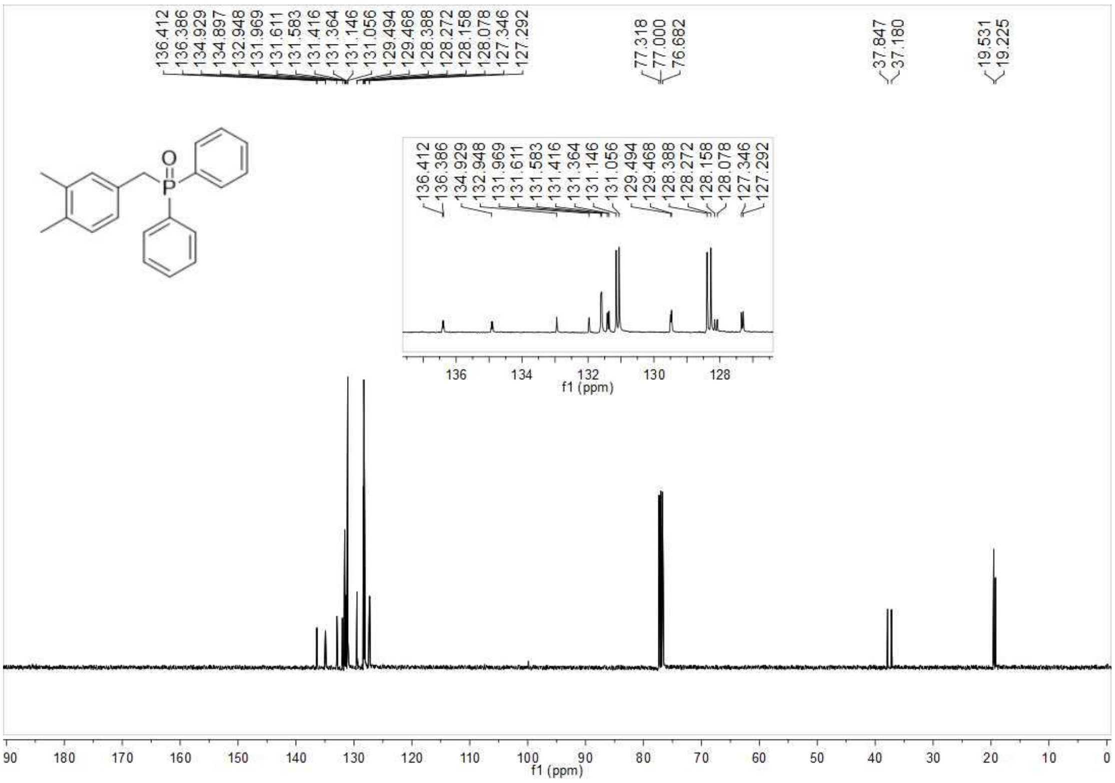

^{31}P NMR Spectrum of **208**

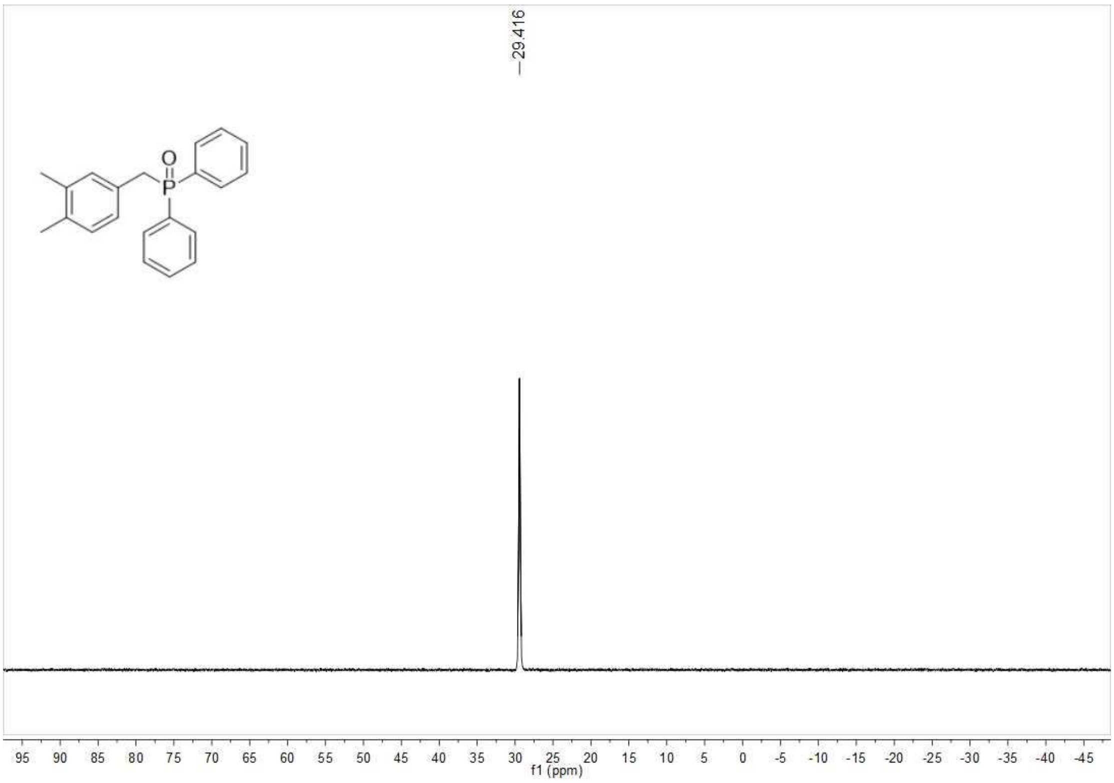

^{1}H NMR Spectrum of **209**

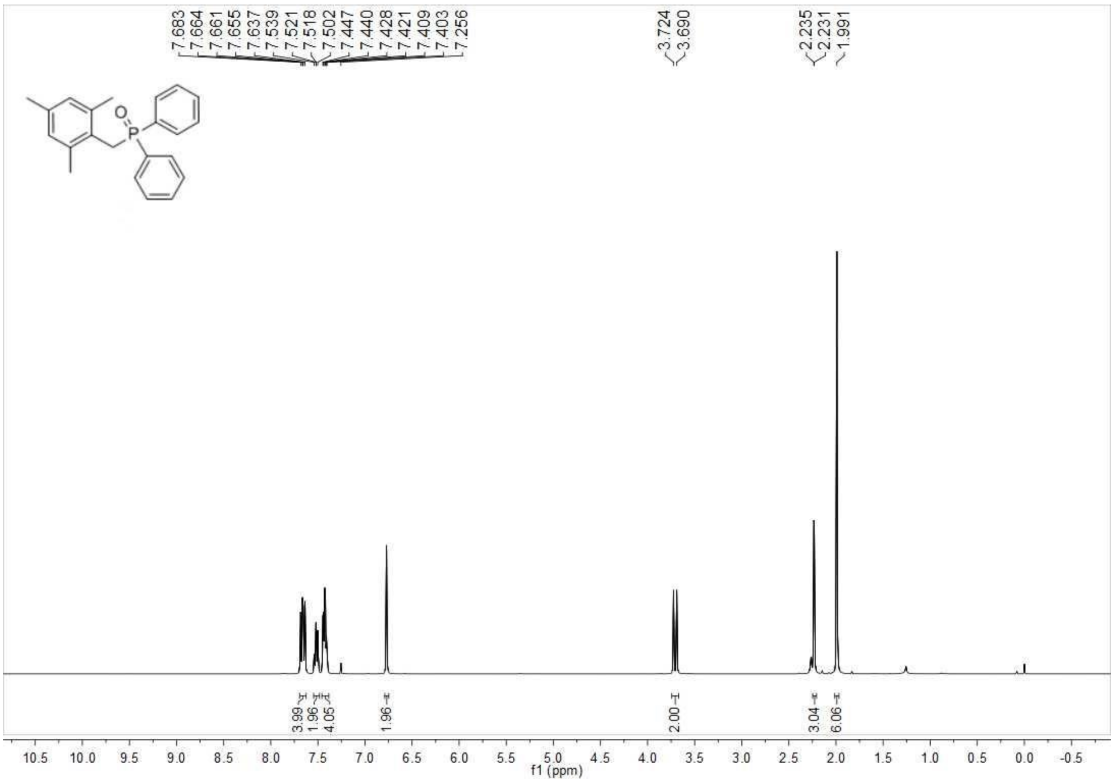

^{13}C NMR Spectrum of **209**

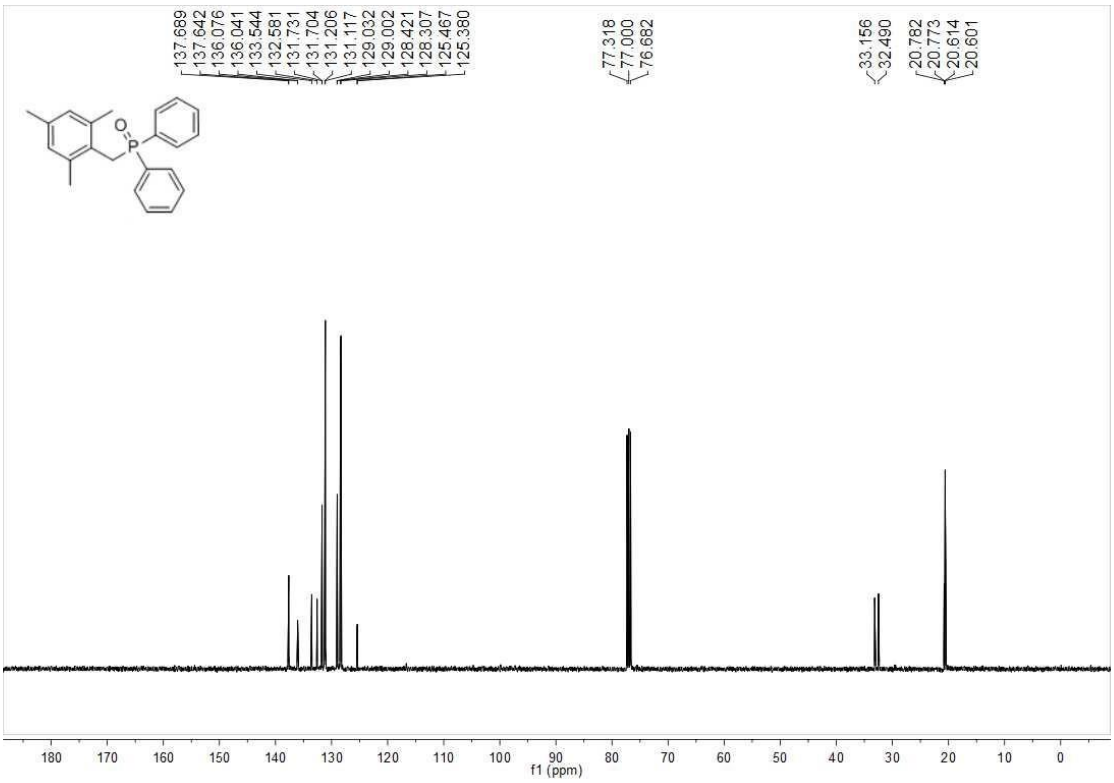

^{31}P NMR Spectrum of **209**

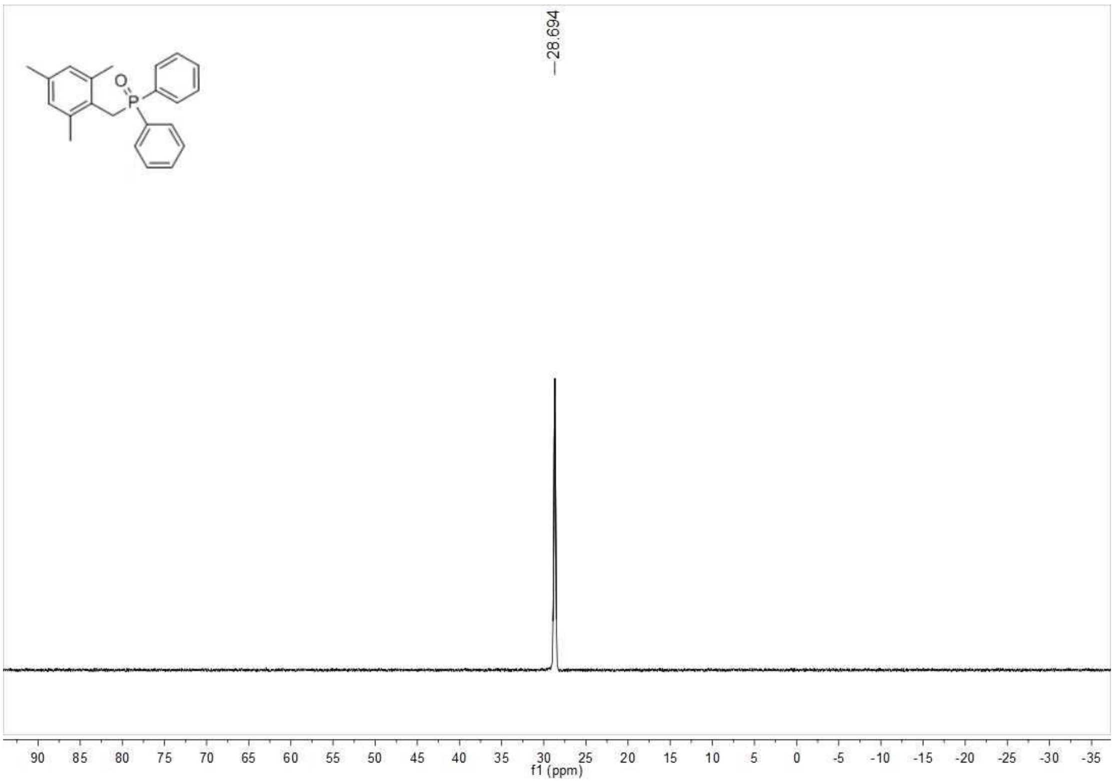

^{1}H NMR Spectrum of **210**

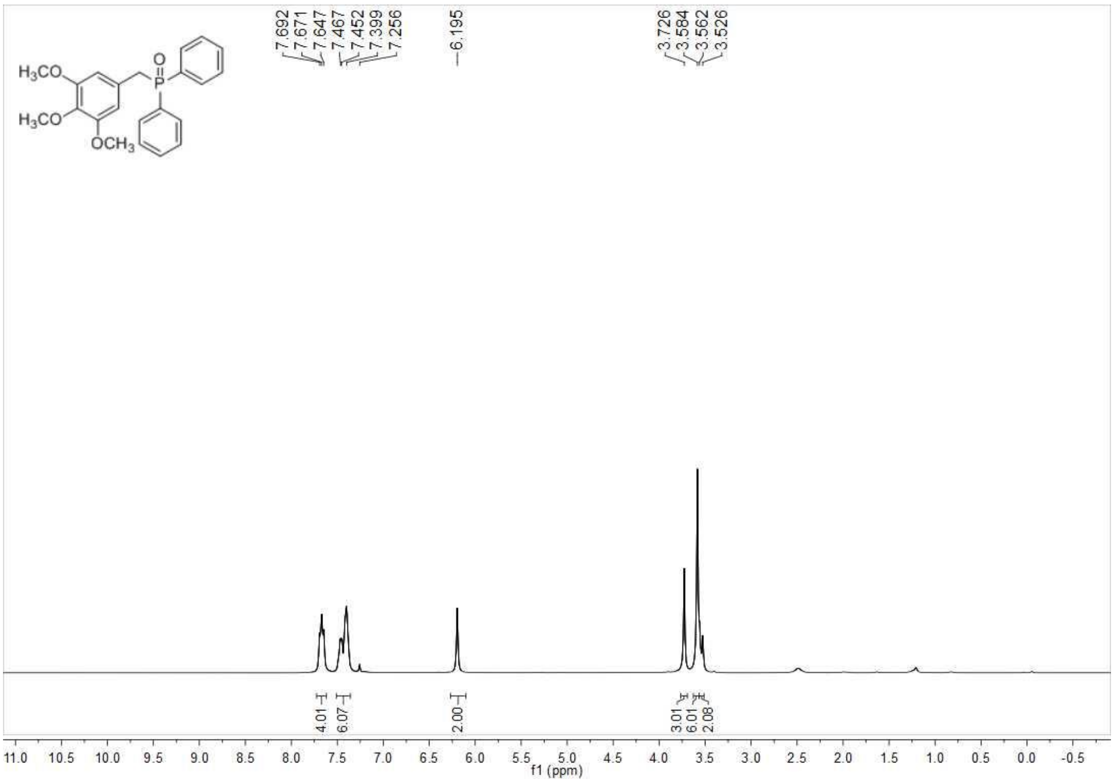

^{13}C NMR Spectrum of **210**

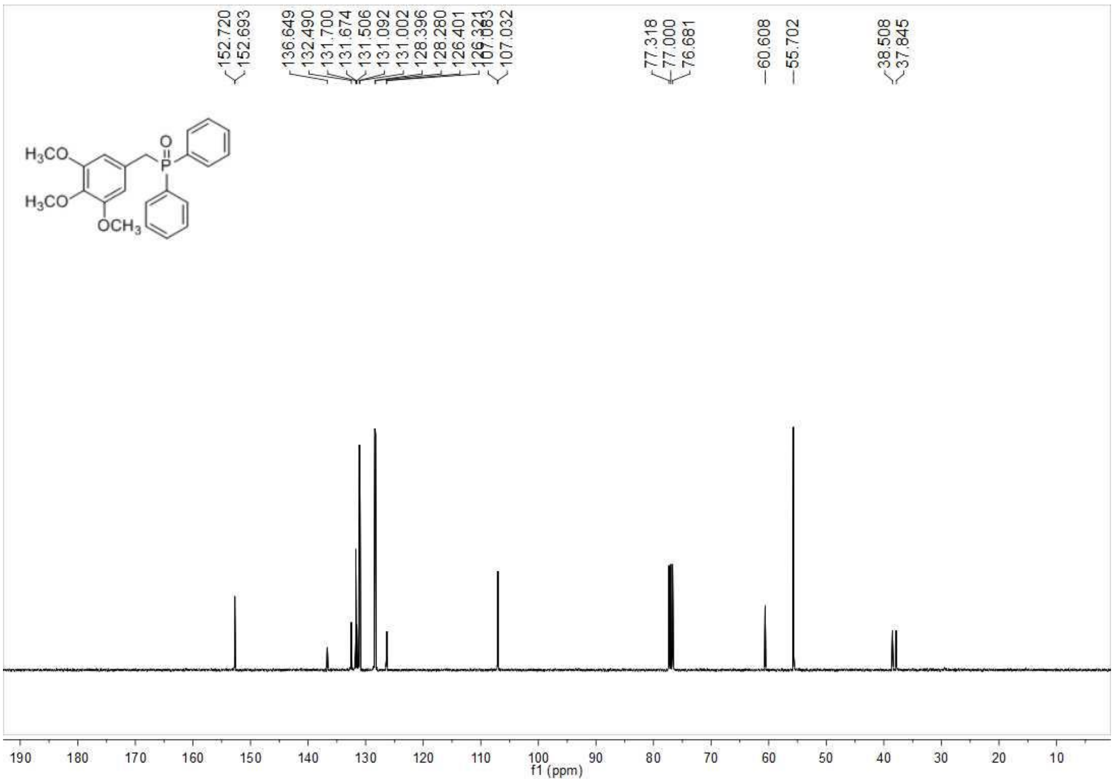

^{31}P NMR Spectrum of **210**

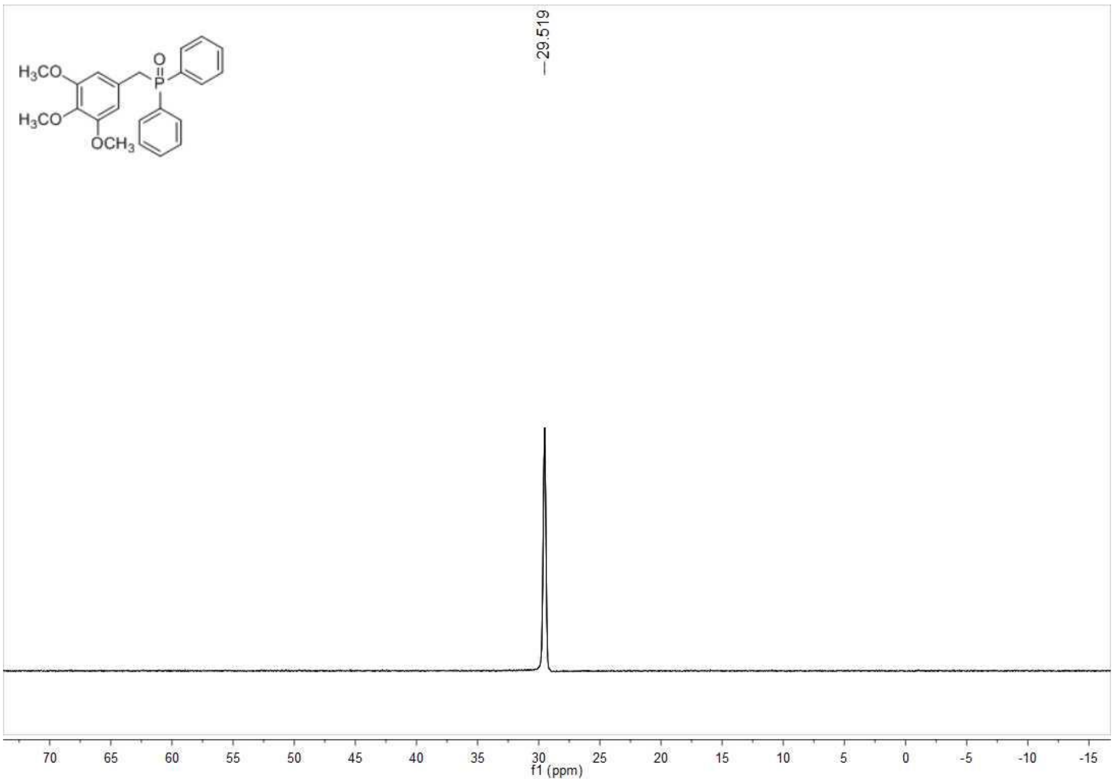

^{1}H NMR Spectrum of **211**

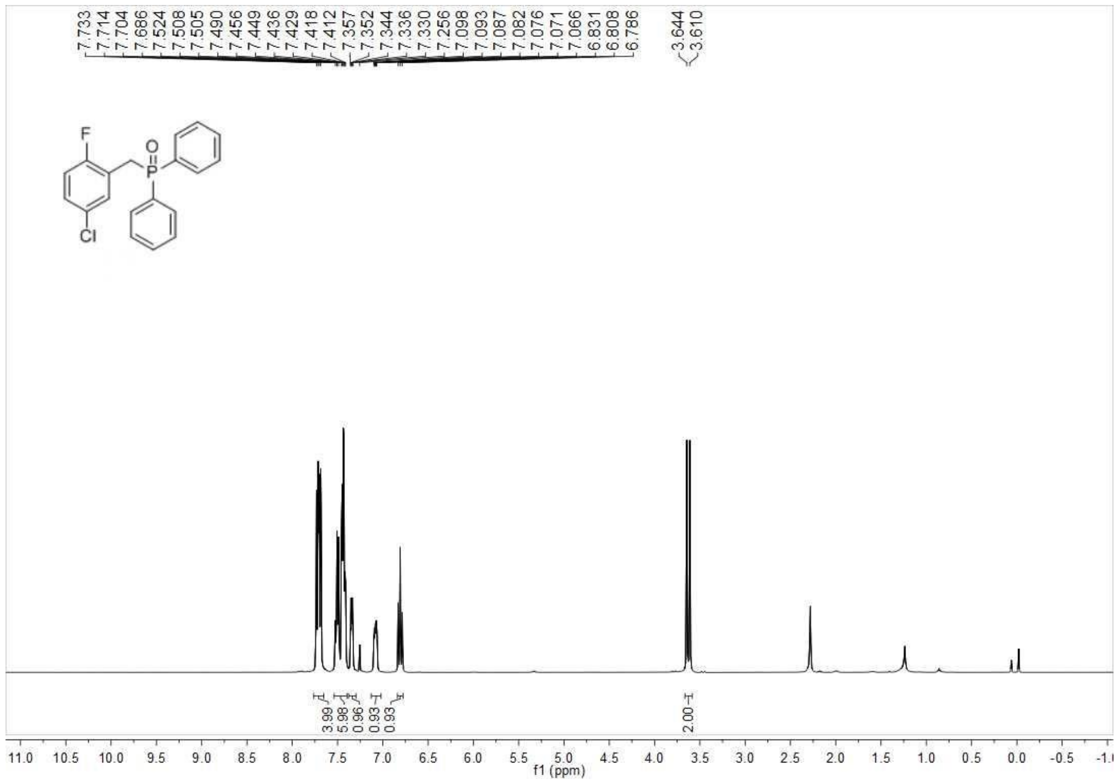

^{13}C NMR Spectrum of **211**

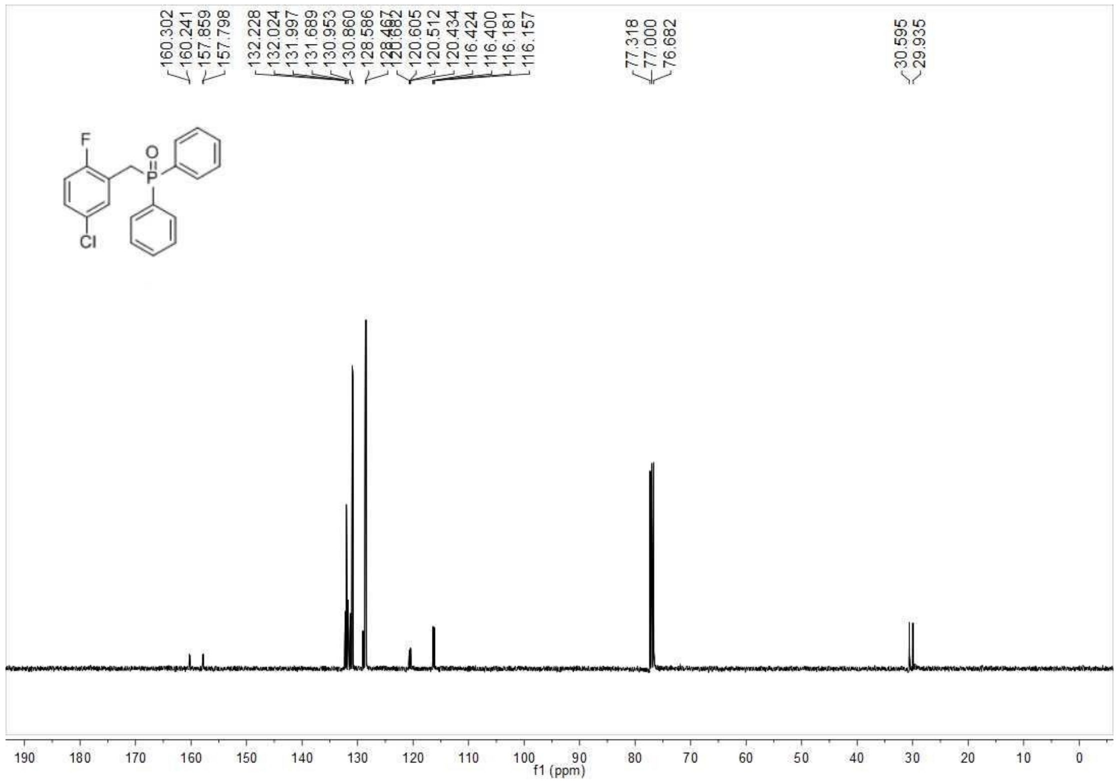

^{31}P NMR Spectrum of **211**

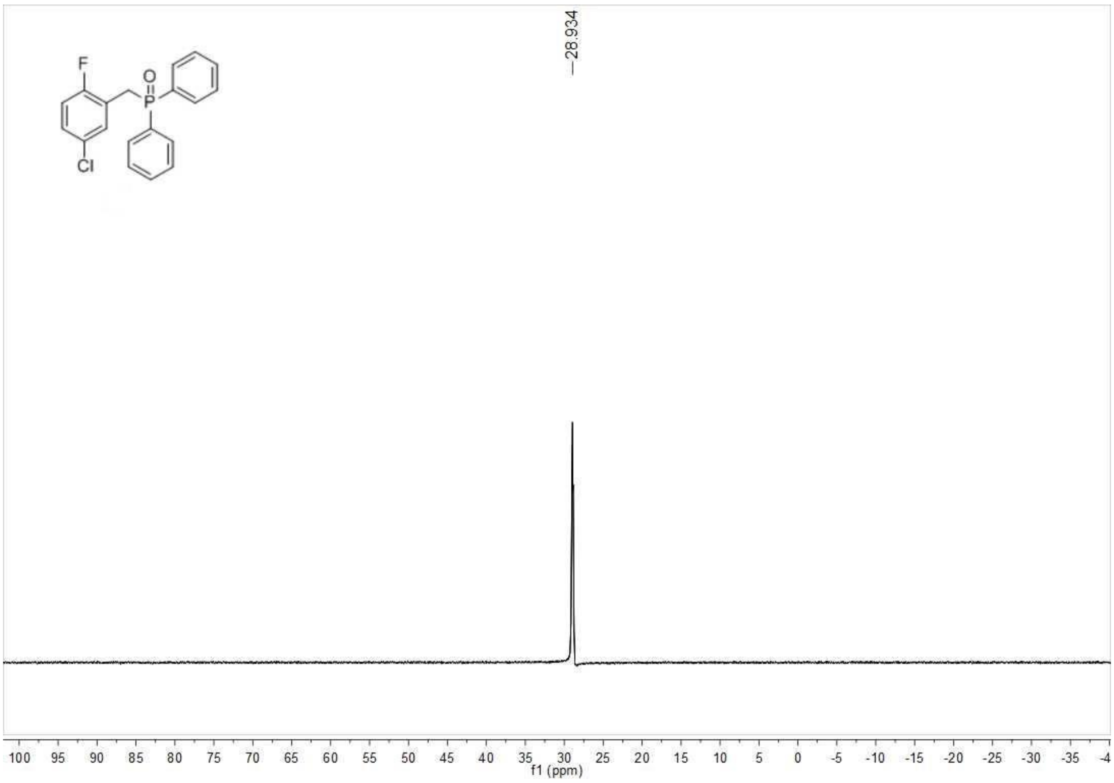

^{19}F NMR Spectrum of **212**

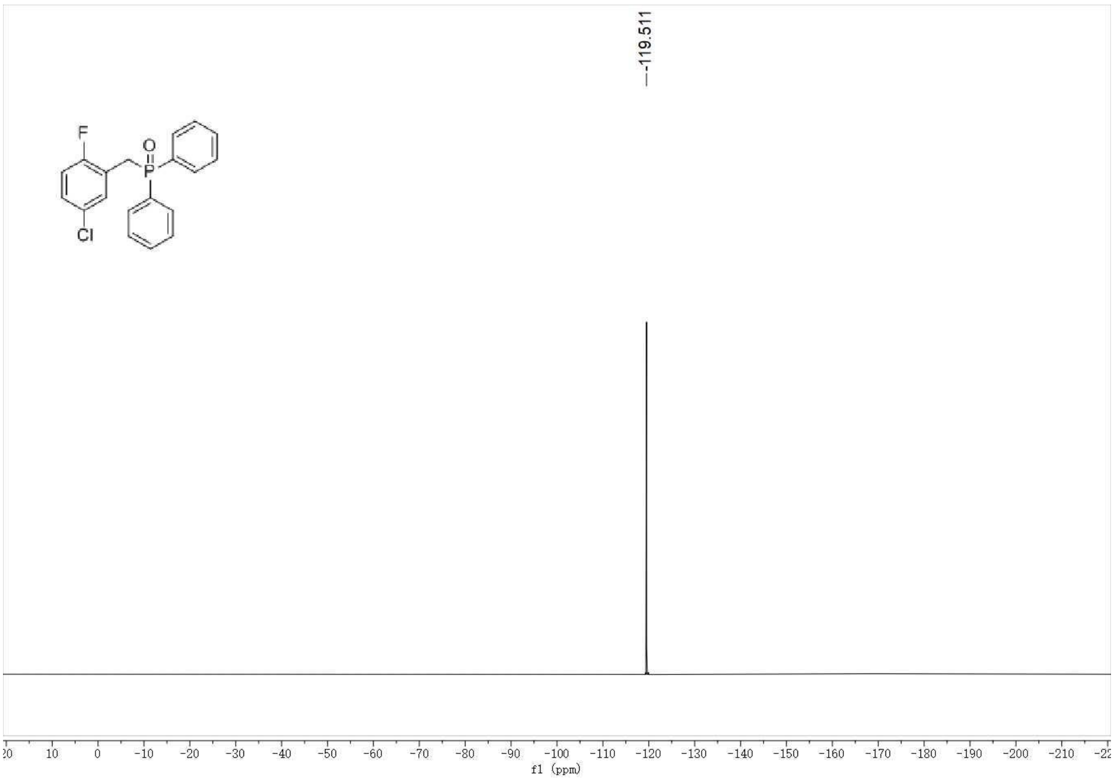

^{1}H NMR Spectrum of **212**

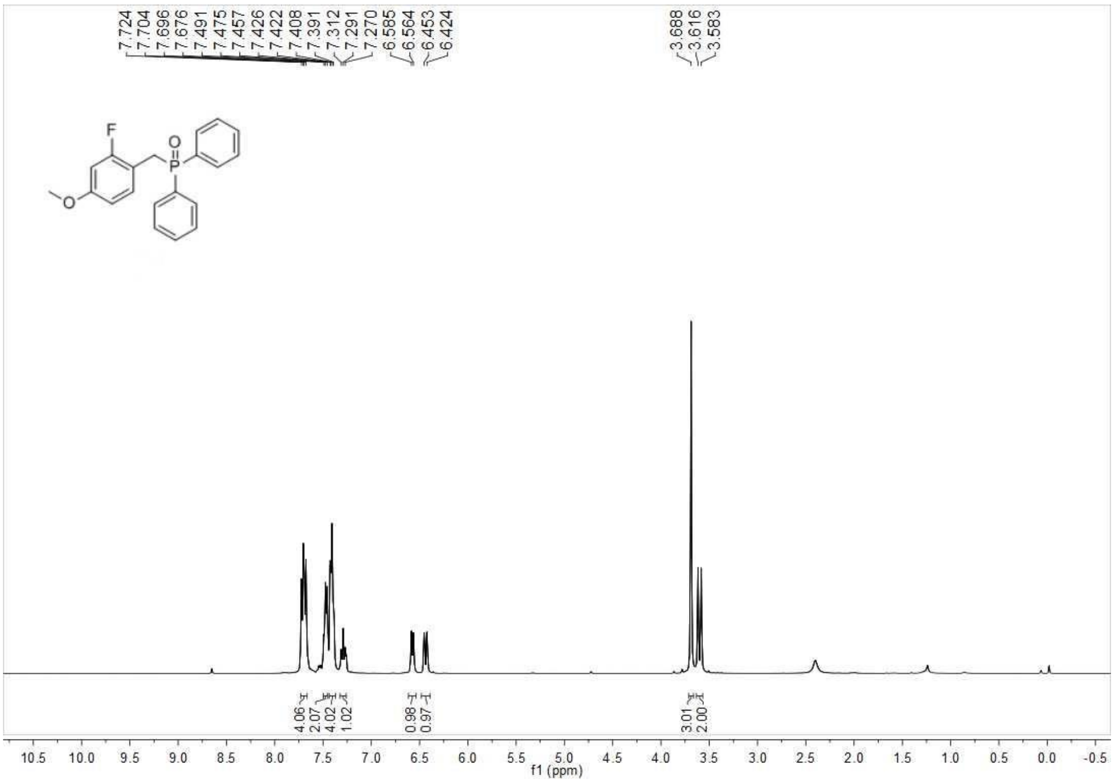

^{13}C NMR Spectrum of **212**

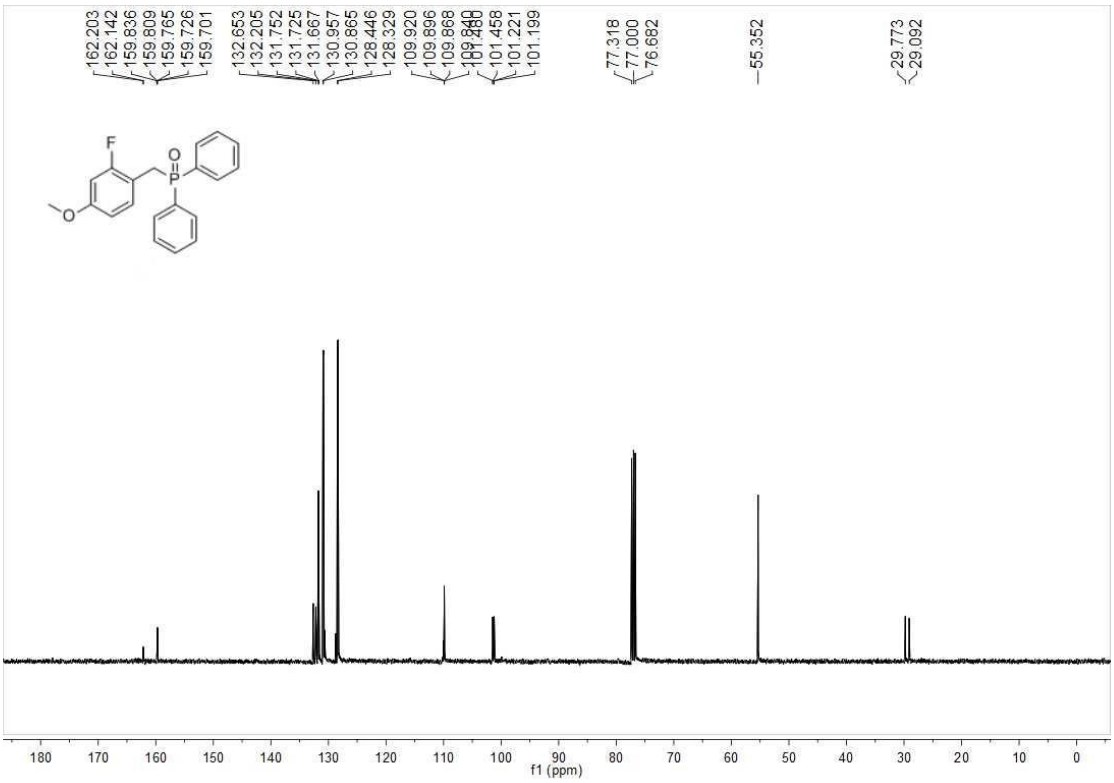

^{31}P NMR Spectrum of **212**

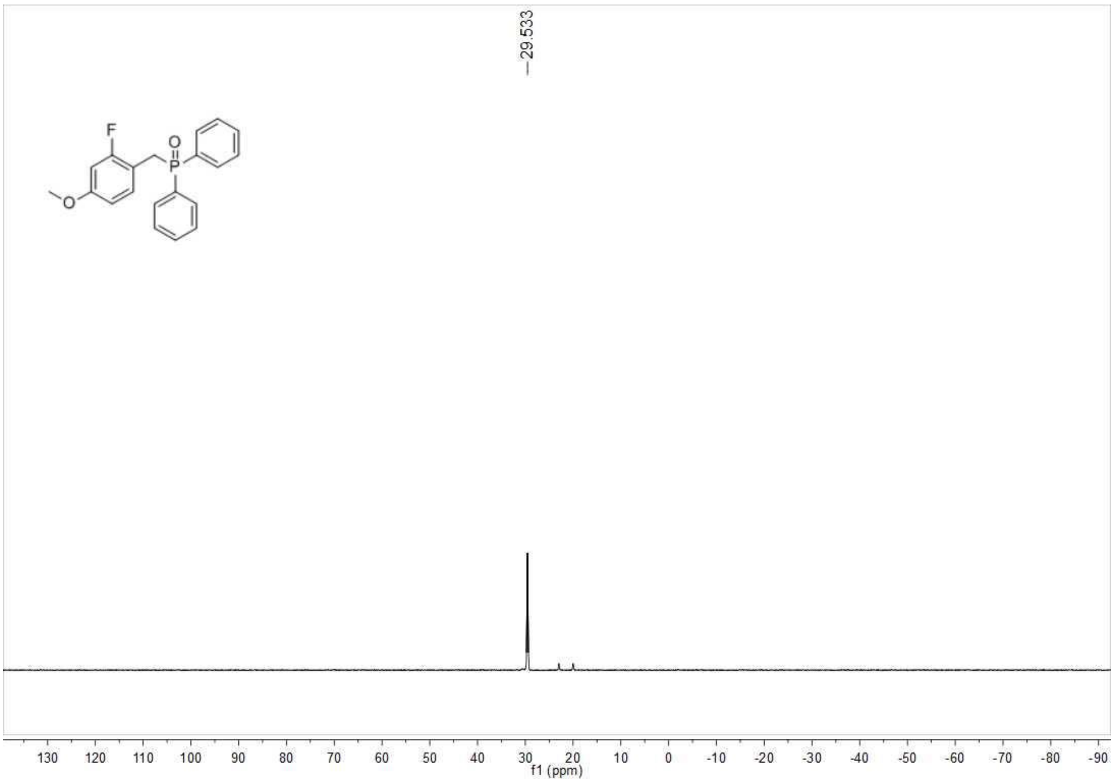

^{19}F NMR Spectrum of **212**

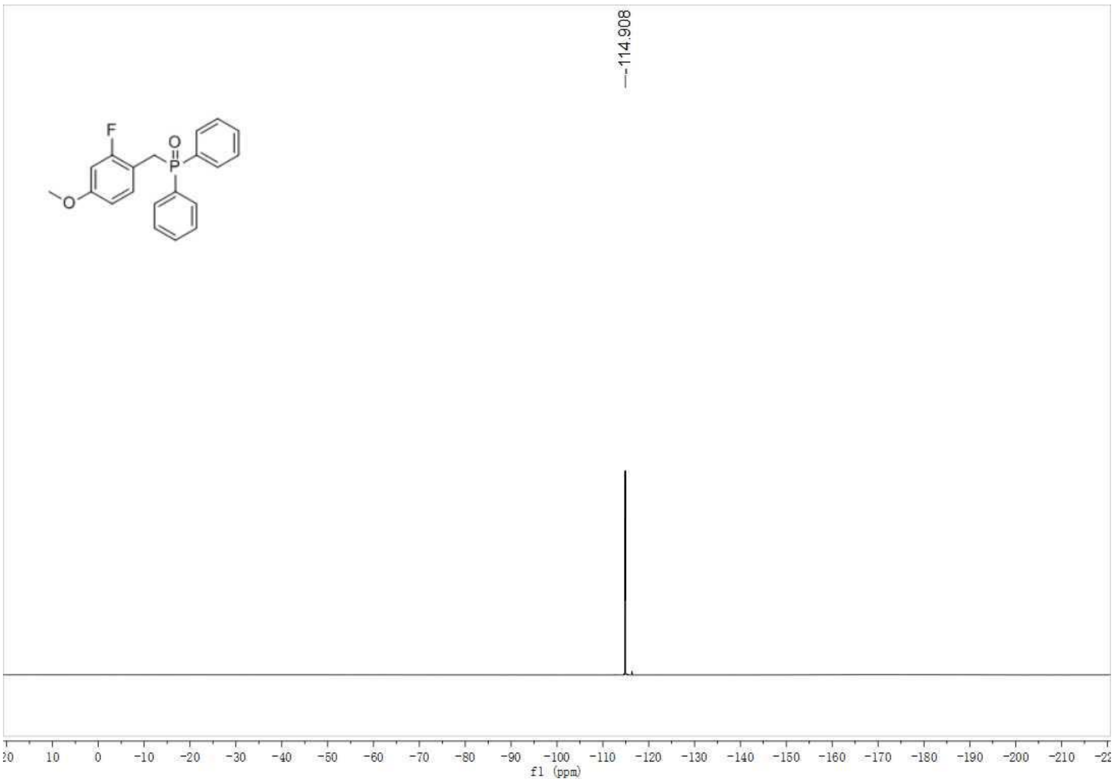

^{1}H NMR Spectrum of **213**

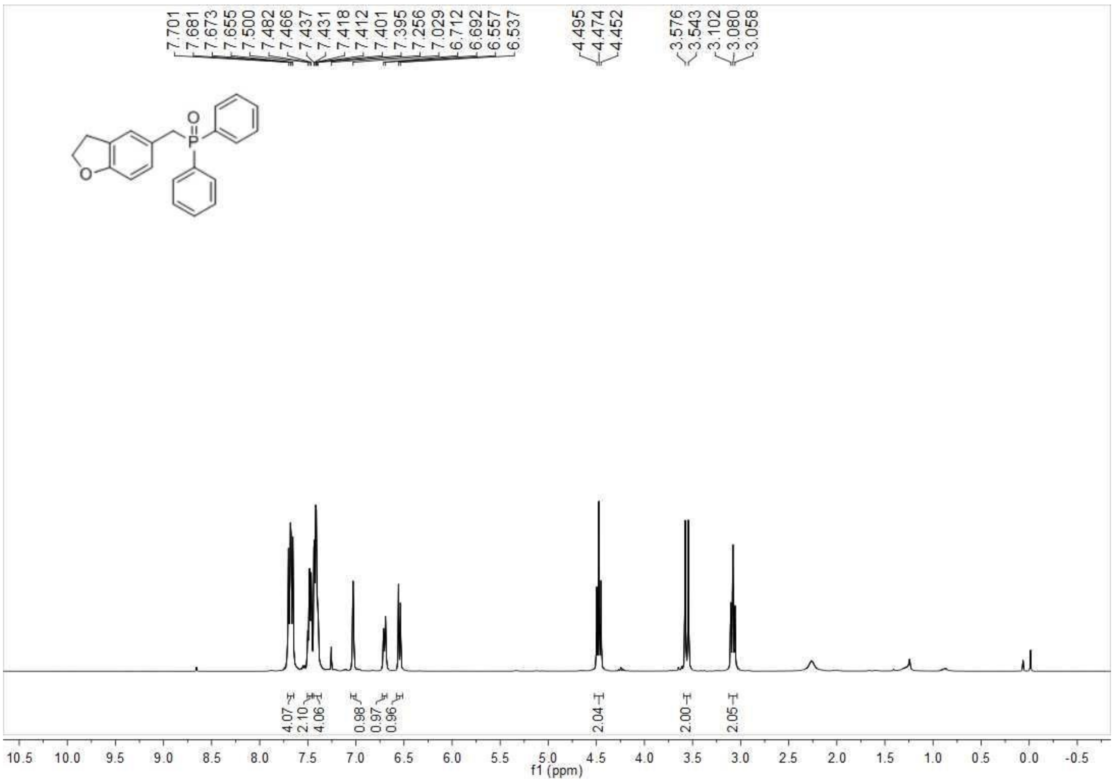

^{13}C NMR Spectrum of **213**

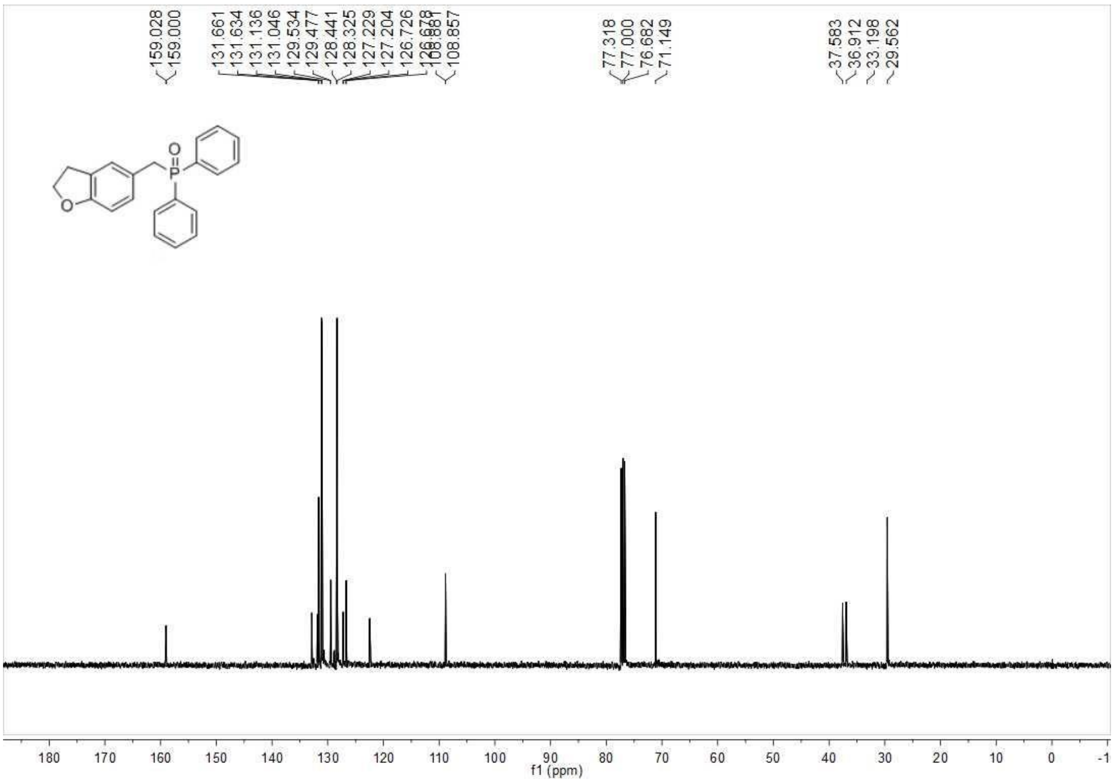

^{31}P NMR Spectrum of **213**

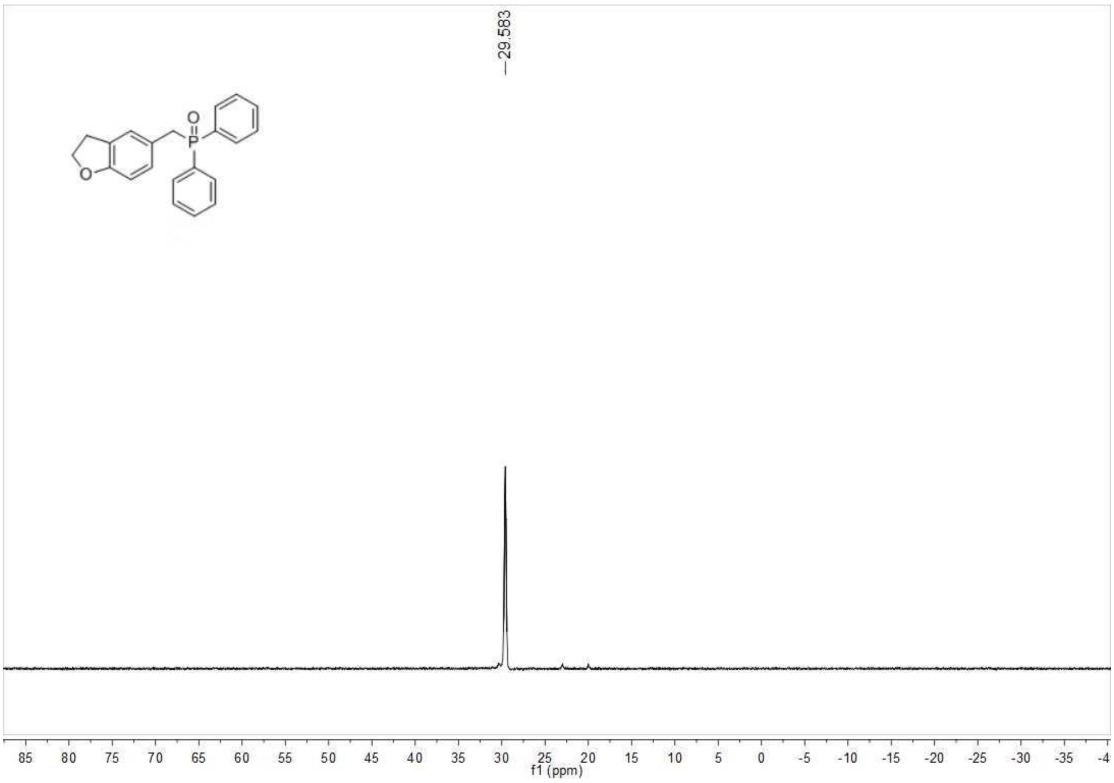

^{1}H NMR Spectrum of **214**

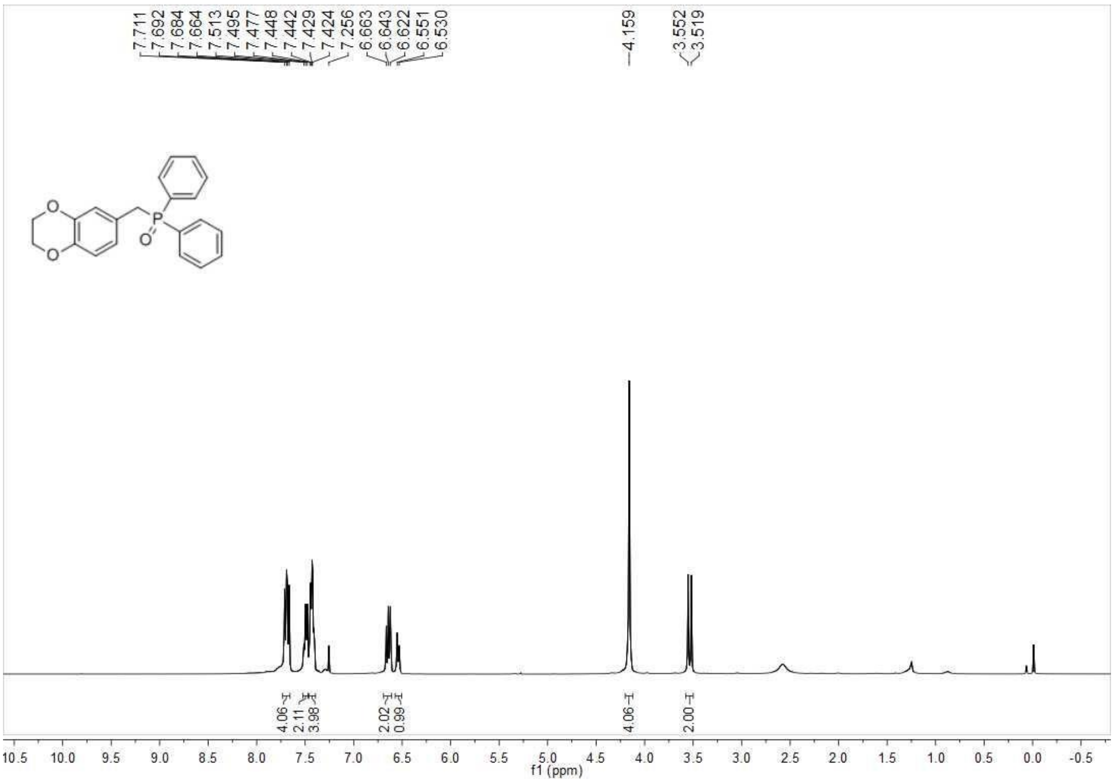

^{13}C NMR Spectrum of **214**

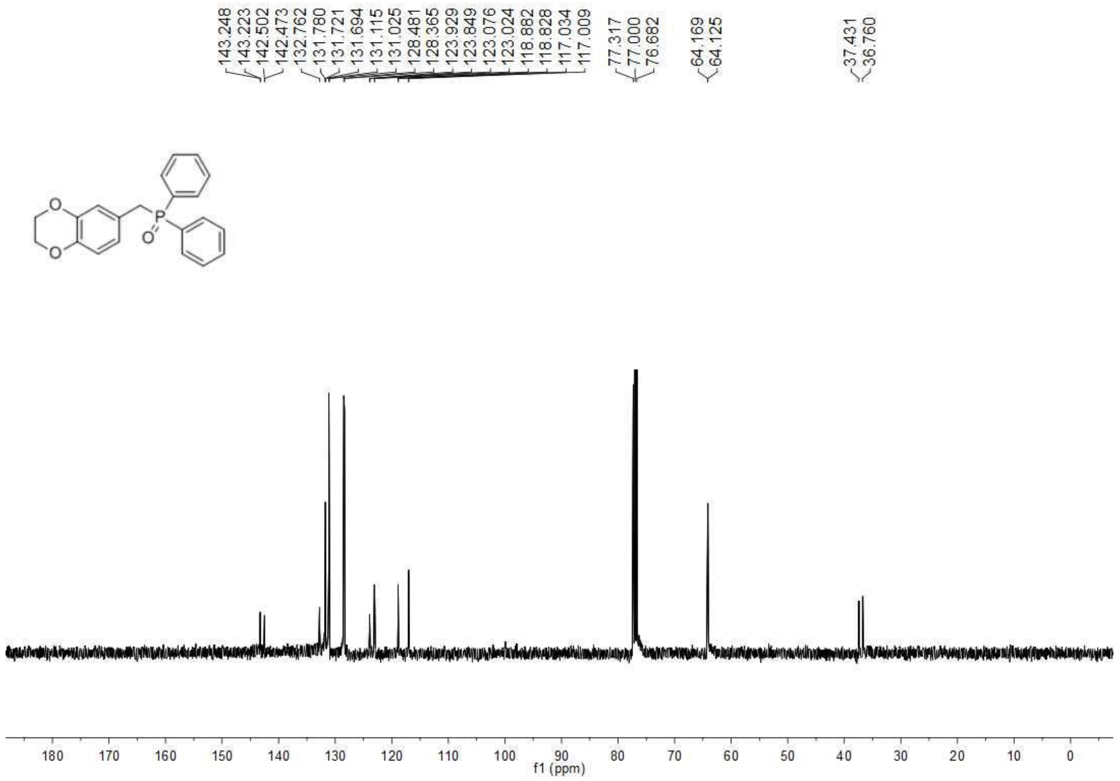

^{31}P NMR Spectrum of **214**

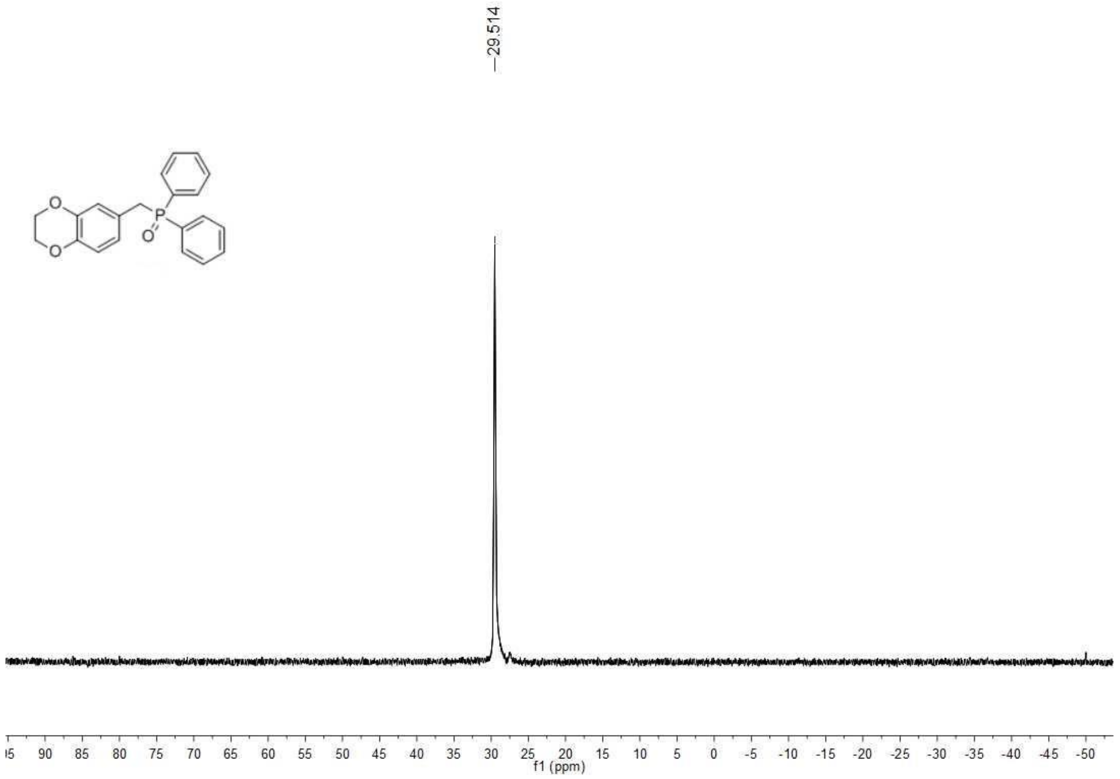

^{1}H NMR Spectrum of **215**

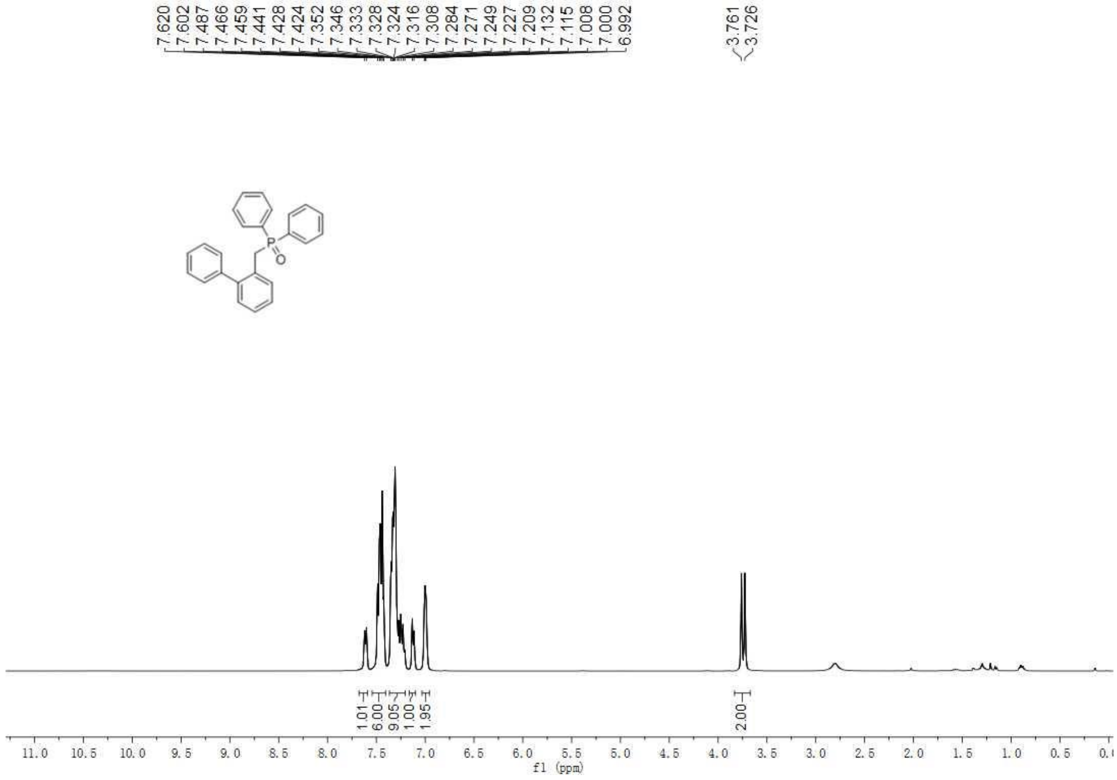

^{13}C NMR Spectrum of **215**

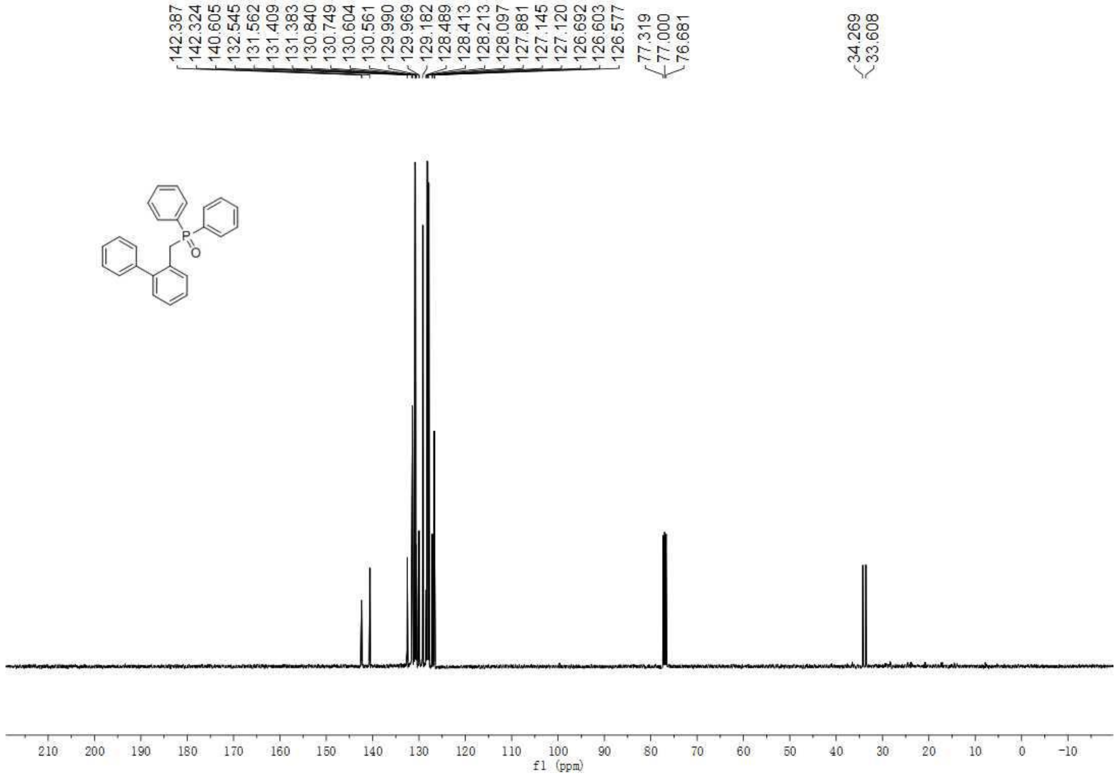

^{31}P NMR Spectrum of **215**

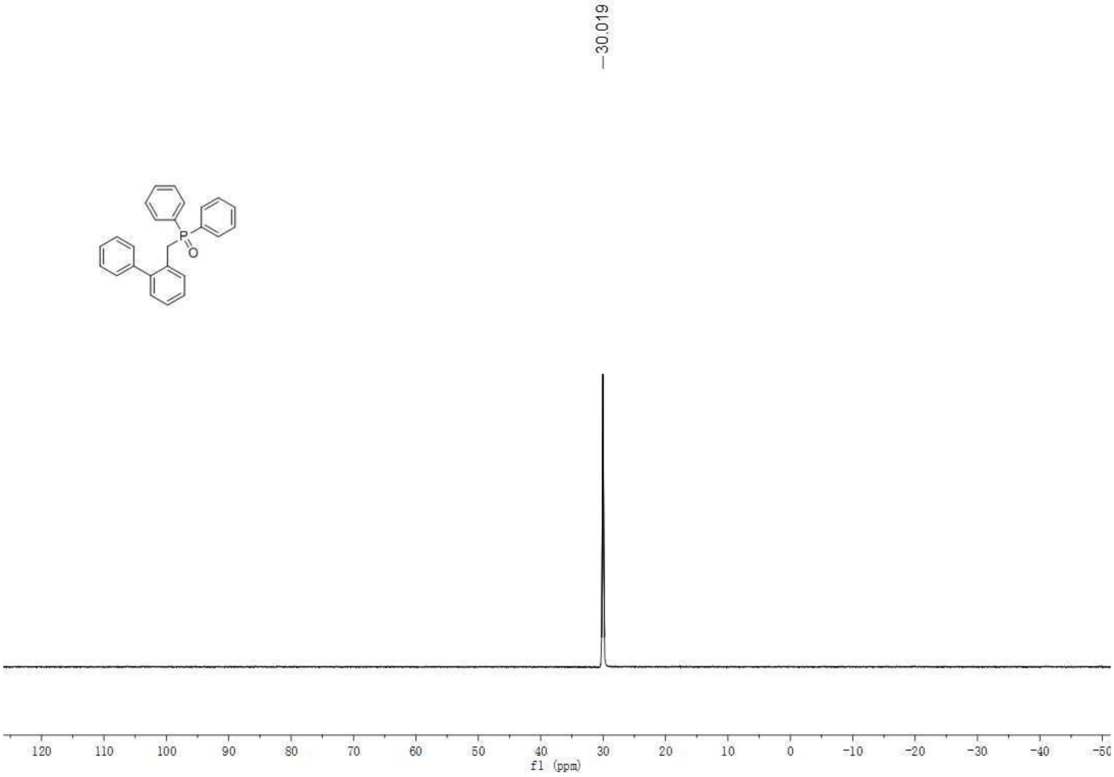

^{1}H NMR Spectrum of **216**

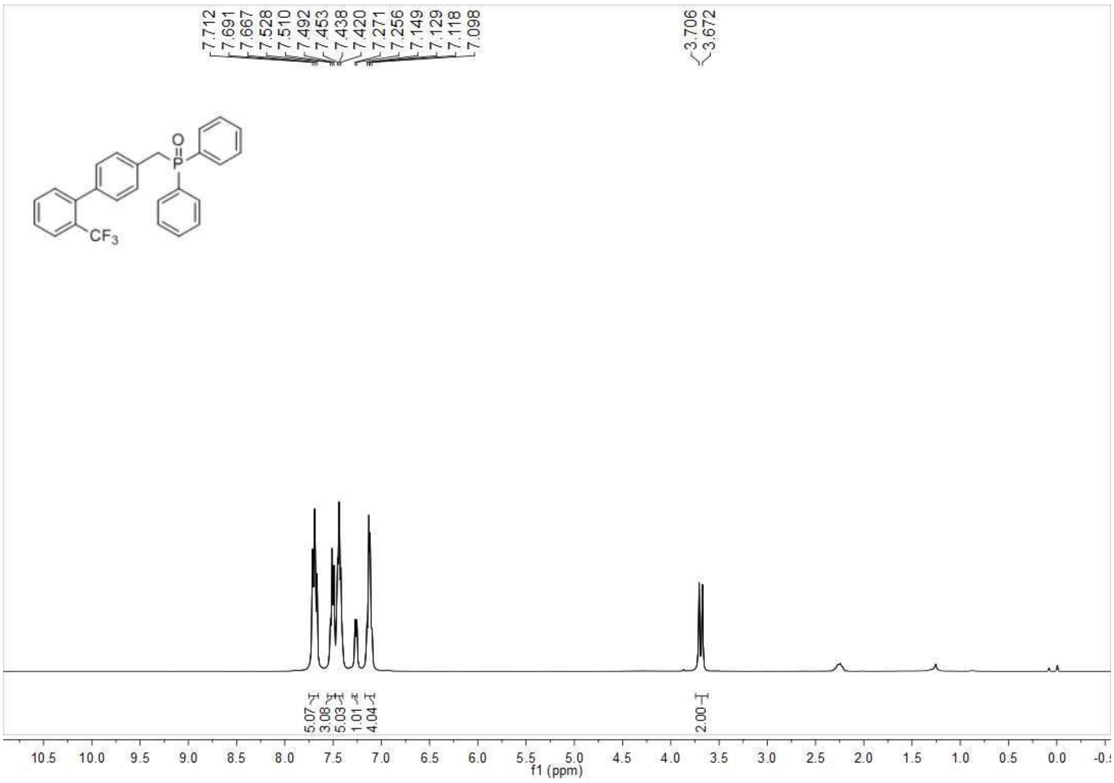

^{13}C NMR Spectrum of **216**

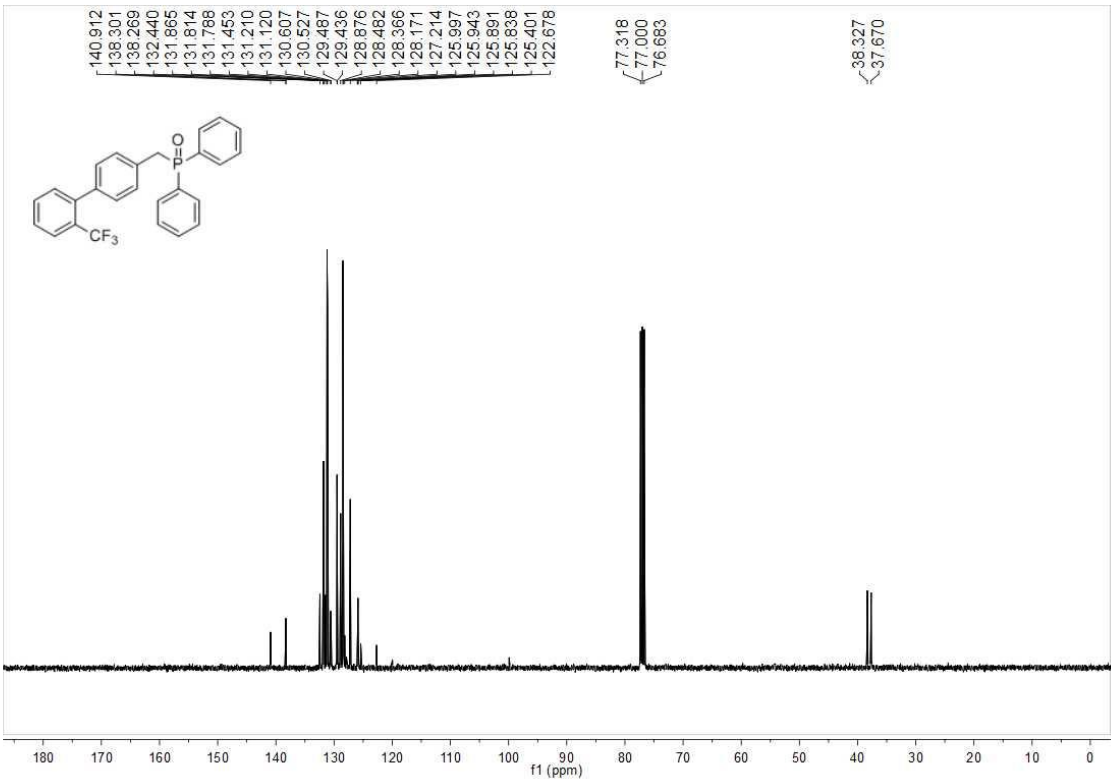

^{31}P NMR Spectrum of **216**

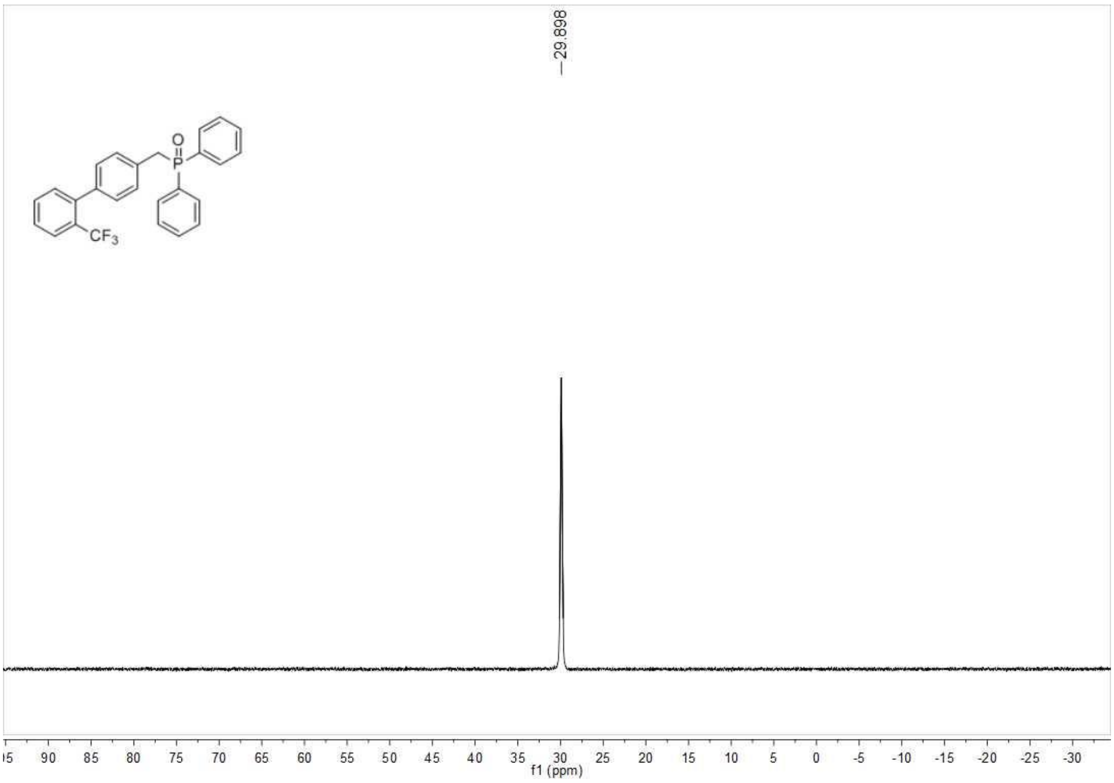

^{19}F NMR Spectrum of **216**

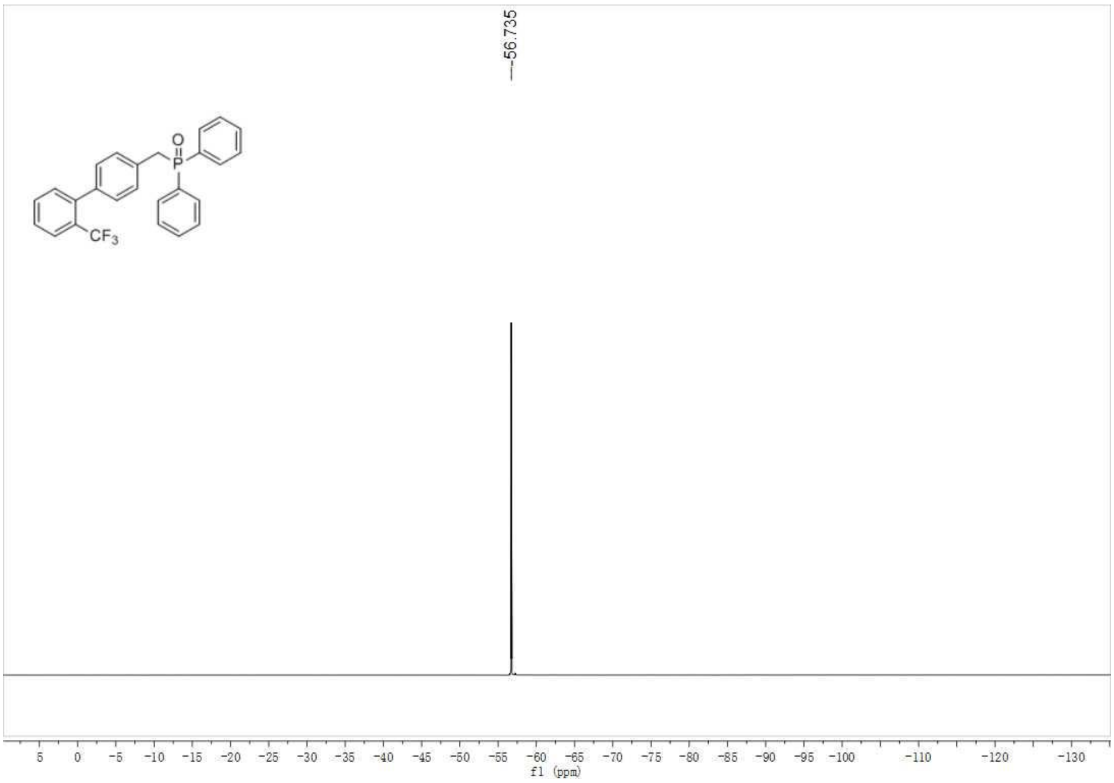

^{1}H NMR Spectrum of **217**

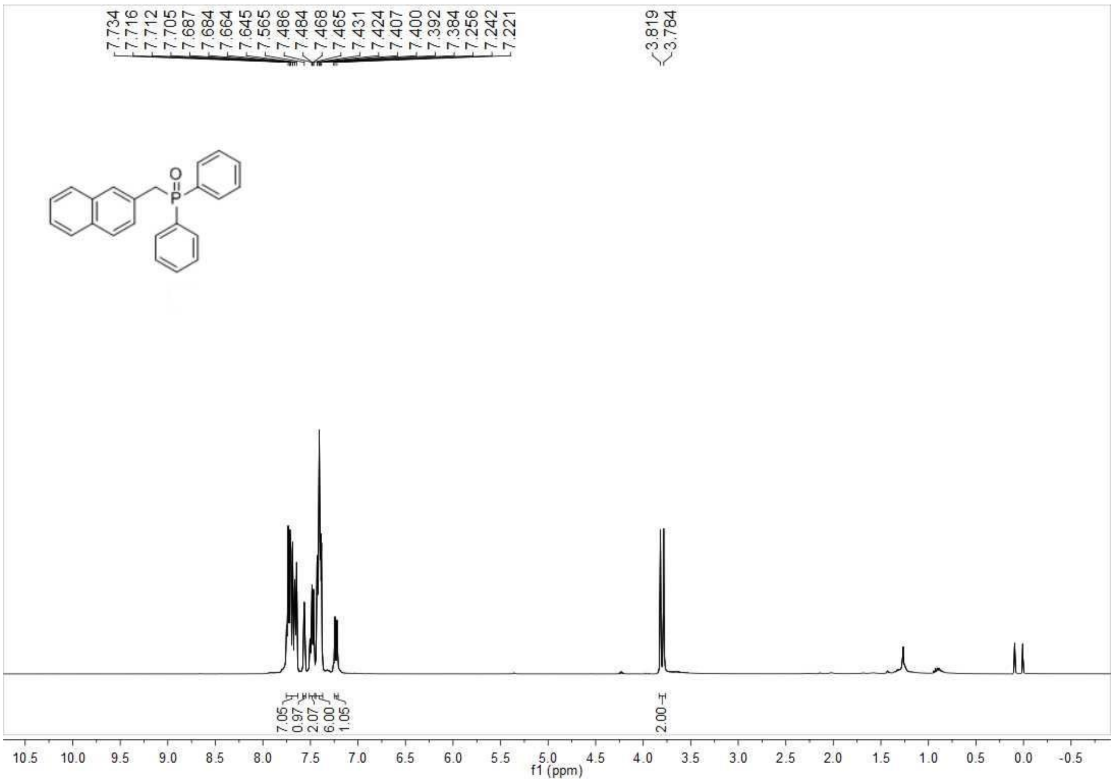

^{13}C NMR Spectrum of **217**

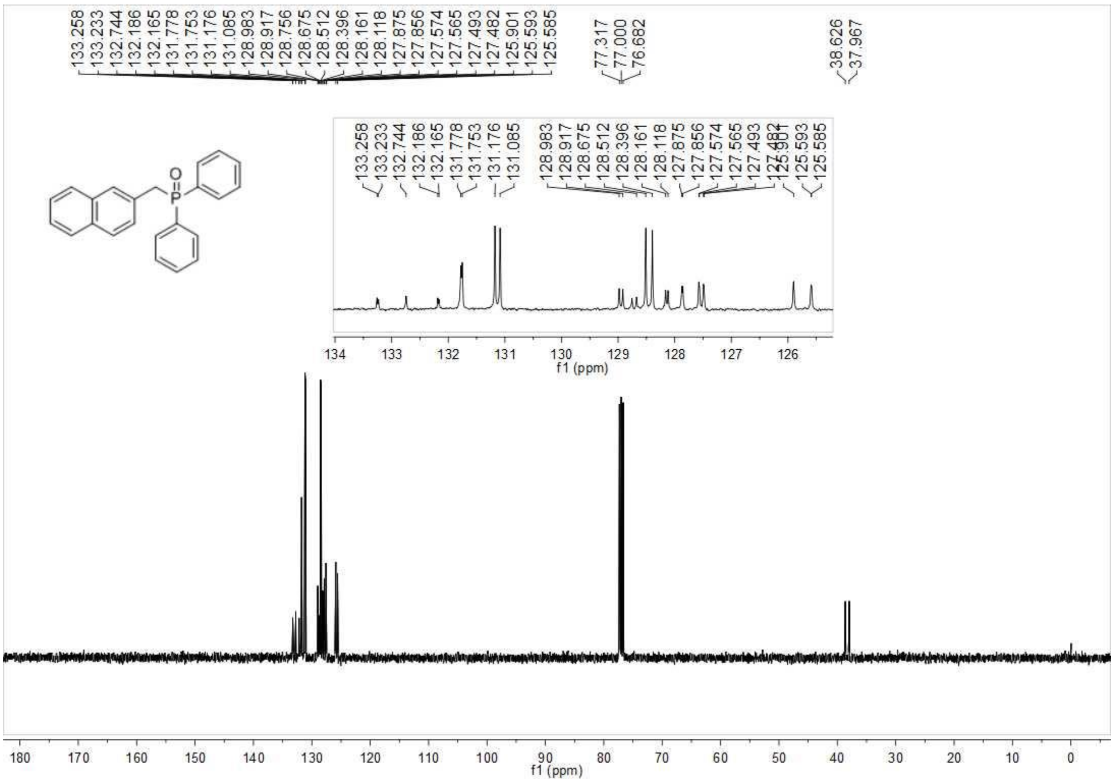

^{31}P NMR Spectrum of **217**

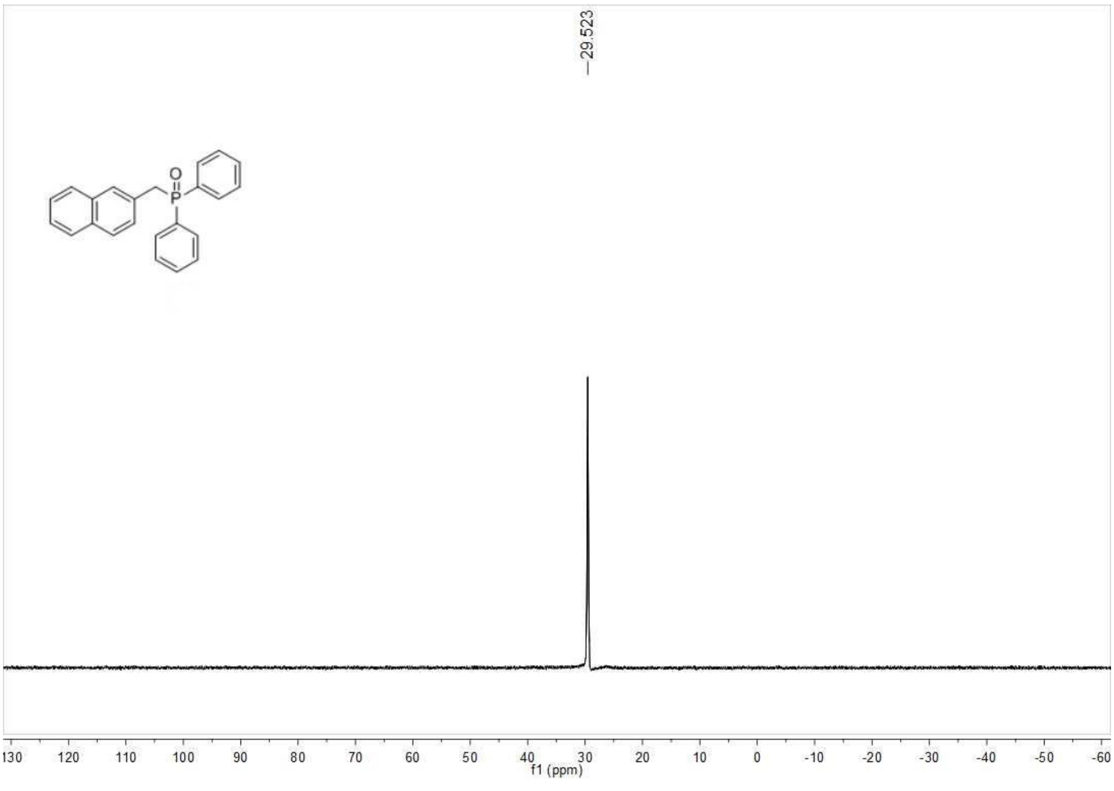

^{1}H NMR Spectrum of **218**

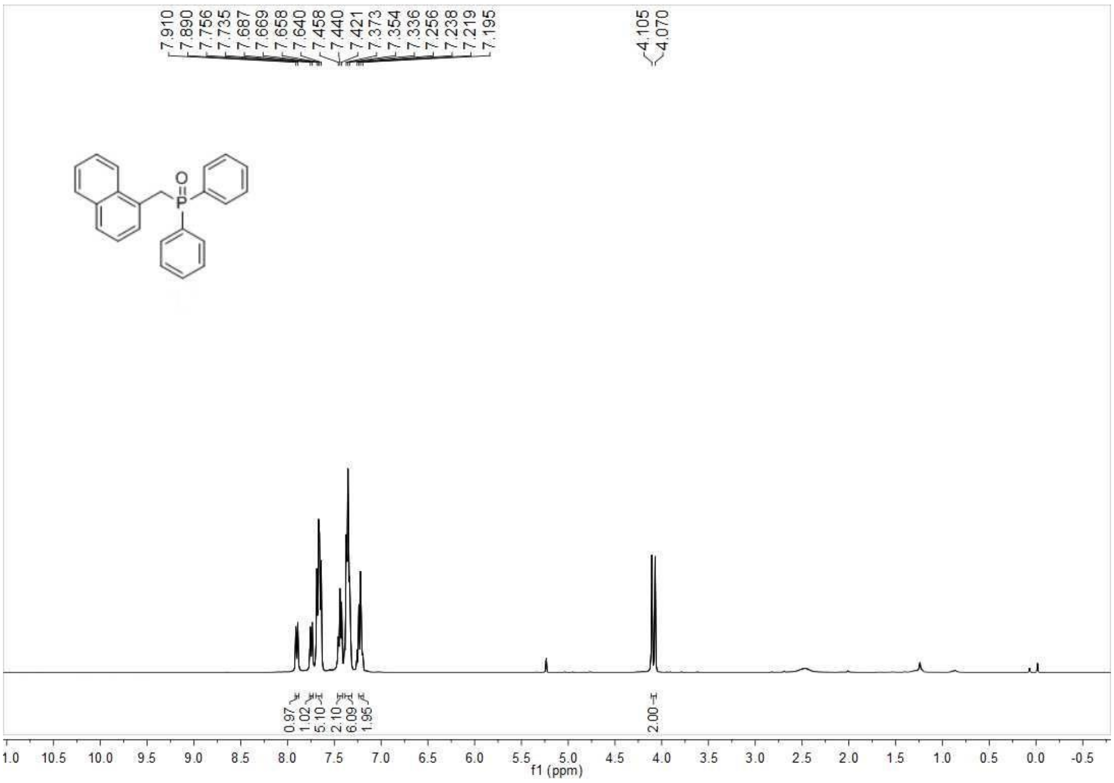

^{13}C NMR Spectrum of **218**

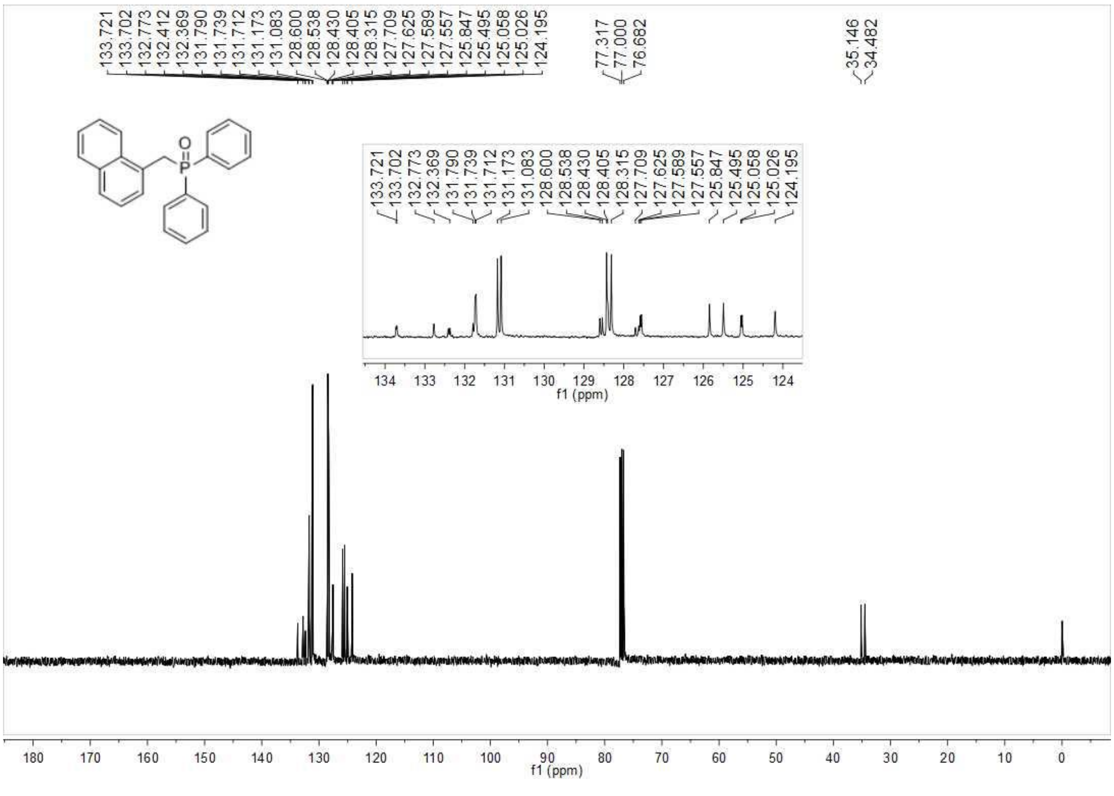

^{31}P NMR Spectrum of **218**

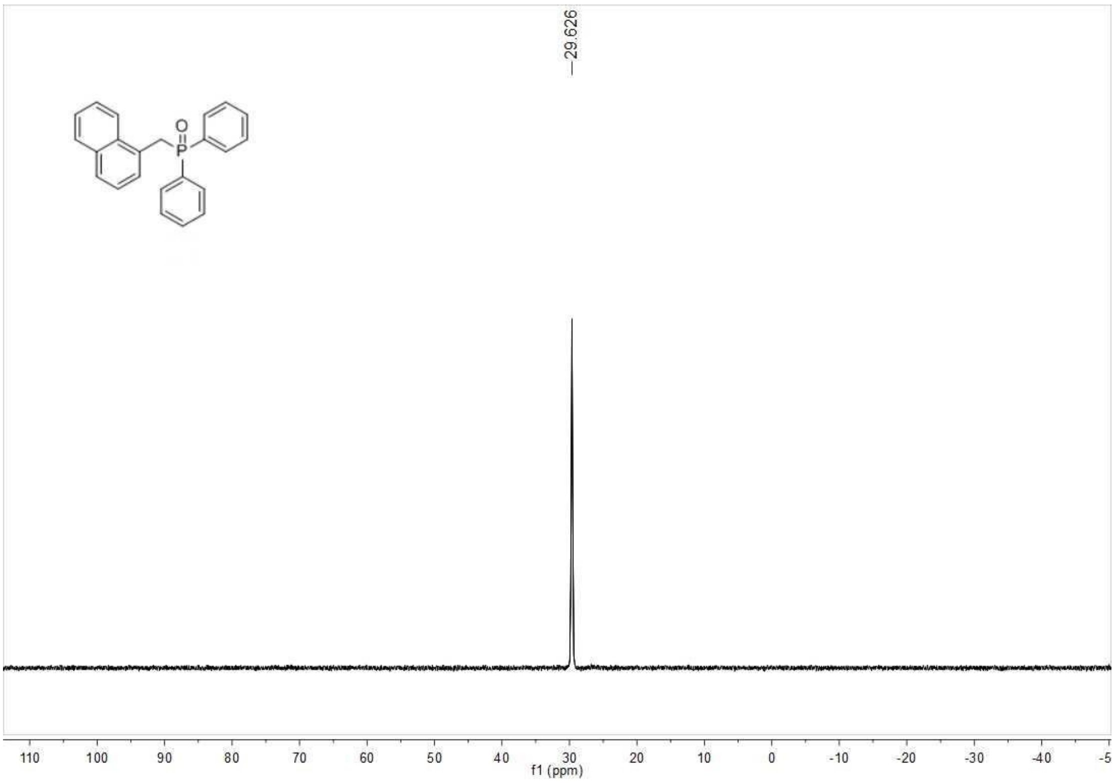

^{1}H NMR Spectrum of **219**

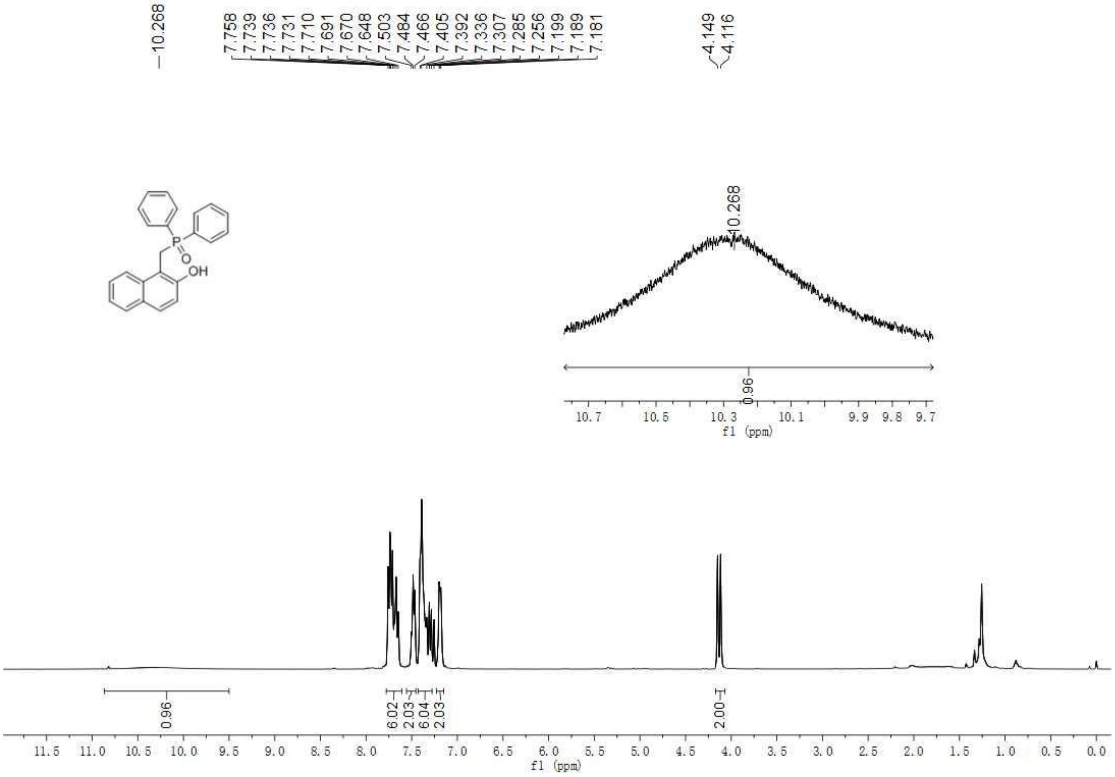

^{13}C NMR Spectrum of **219**

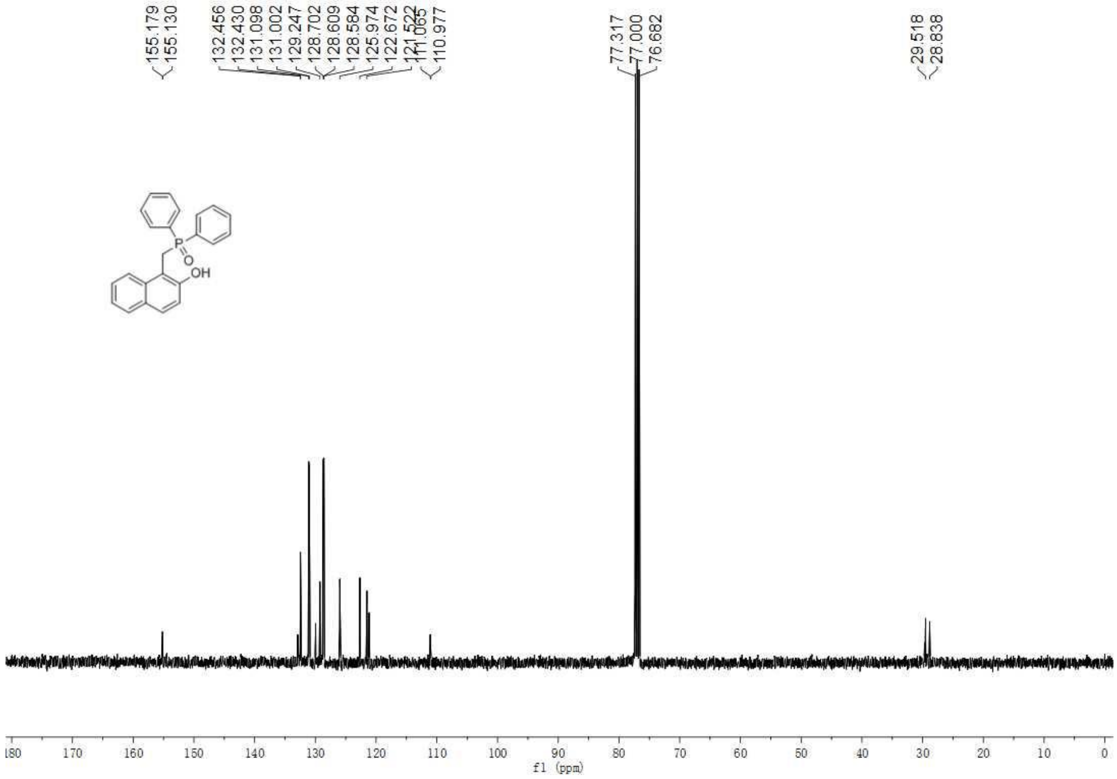

^{31}P NMR Spectrum of **219**

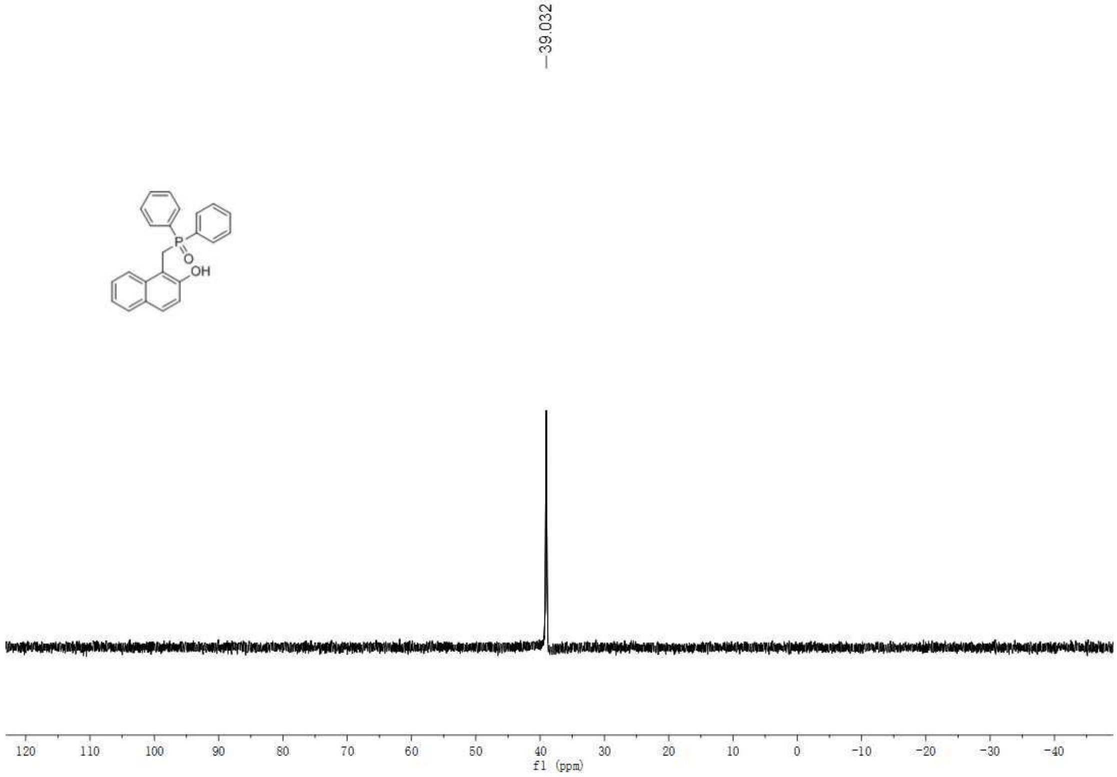

^{1}H NMR Spectrum of **220**

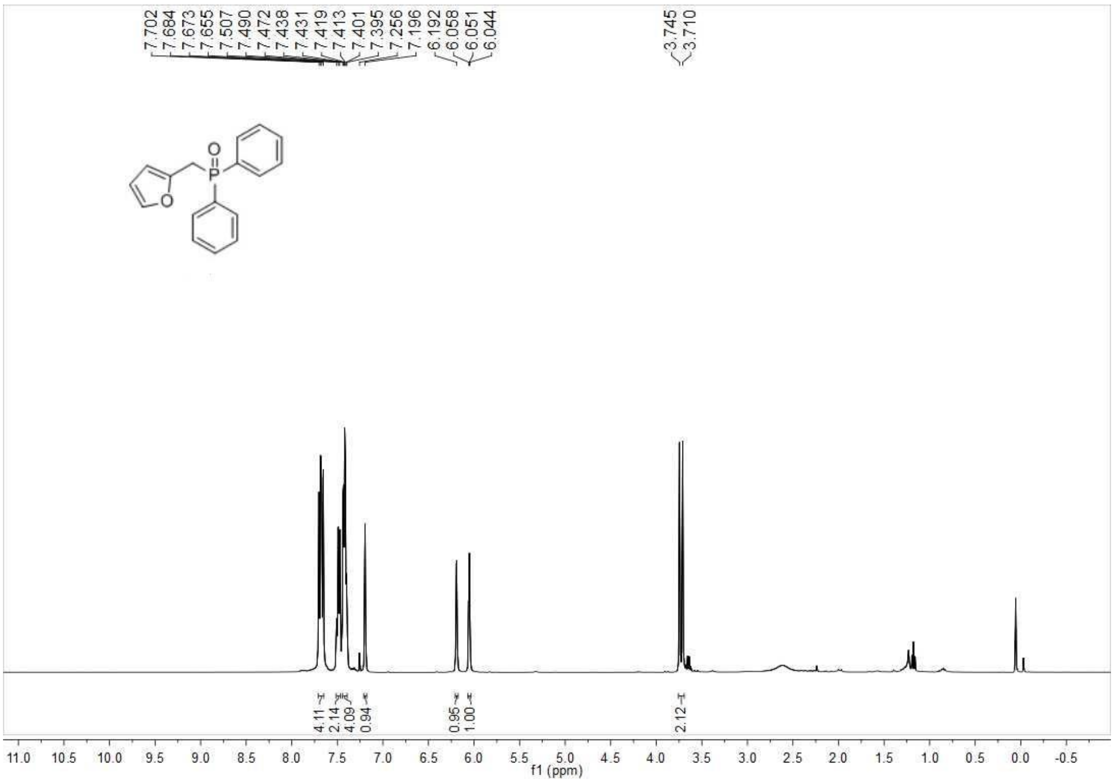

^{13}C NMR Spectrum of **220**

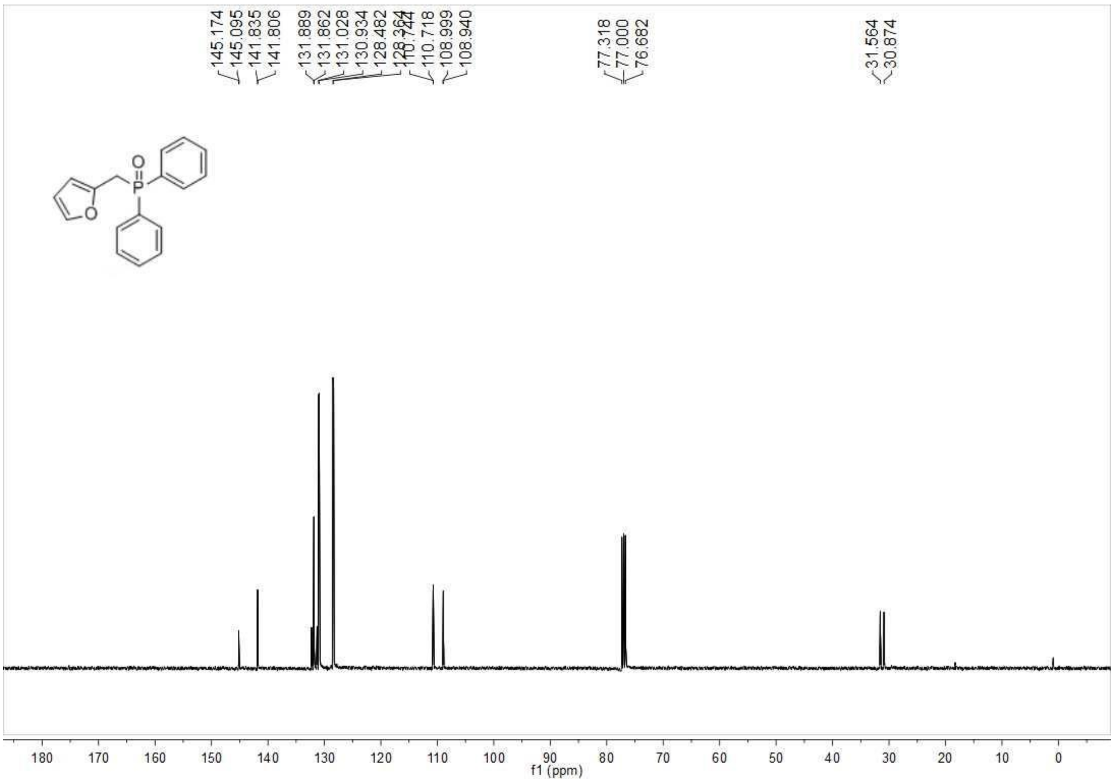

^{31}P NMR Spectrum of **220**

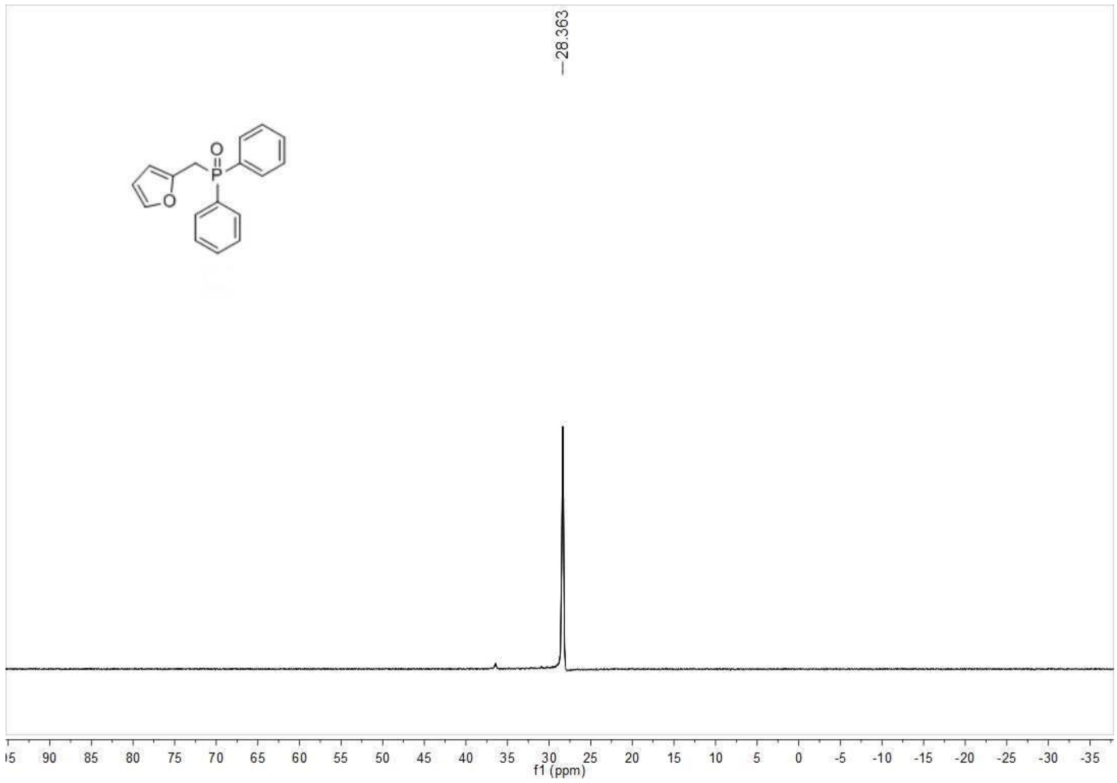

^{1}H NMR Spectrum of **221**

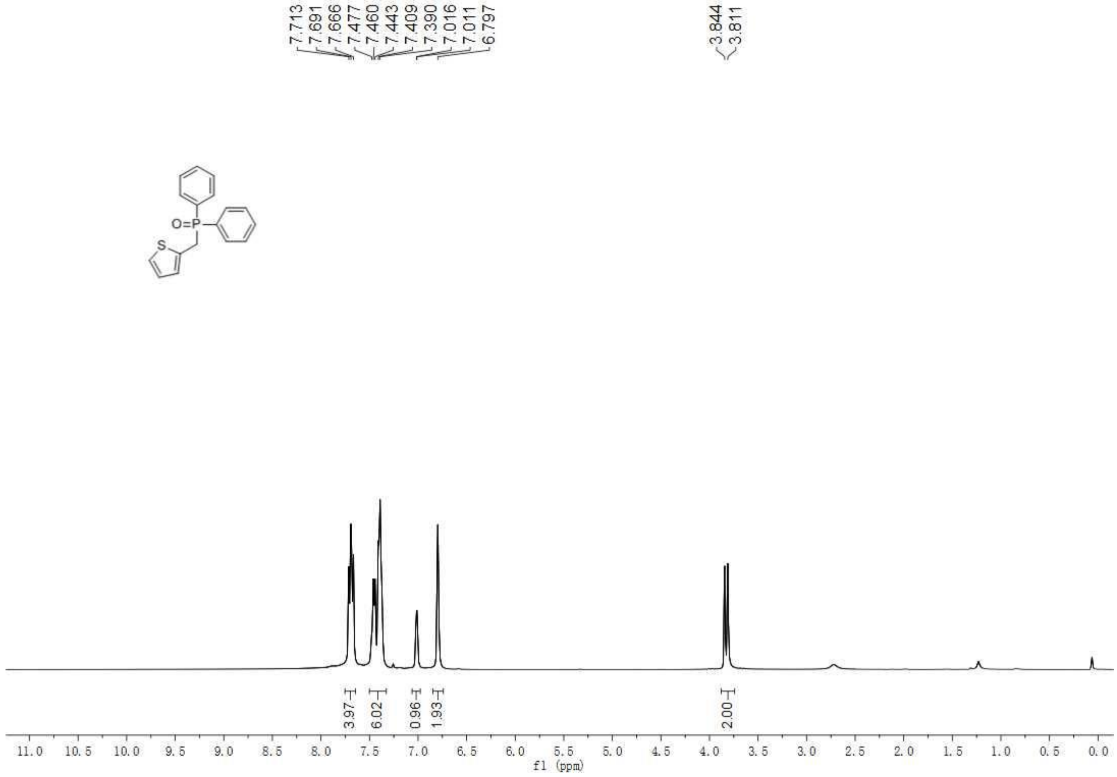

^{13}C NMR Spectrum of **221**

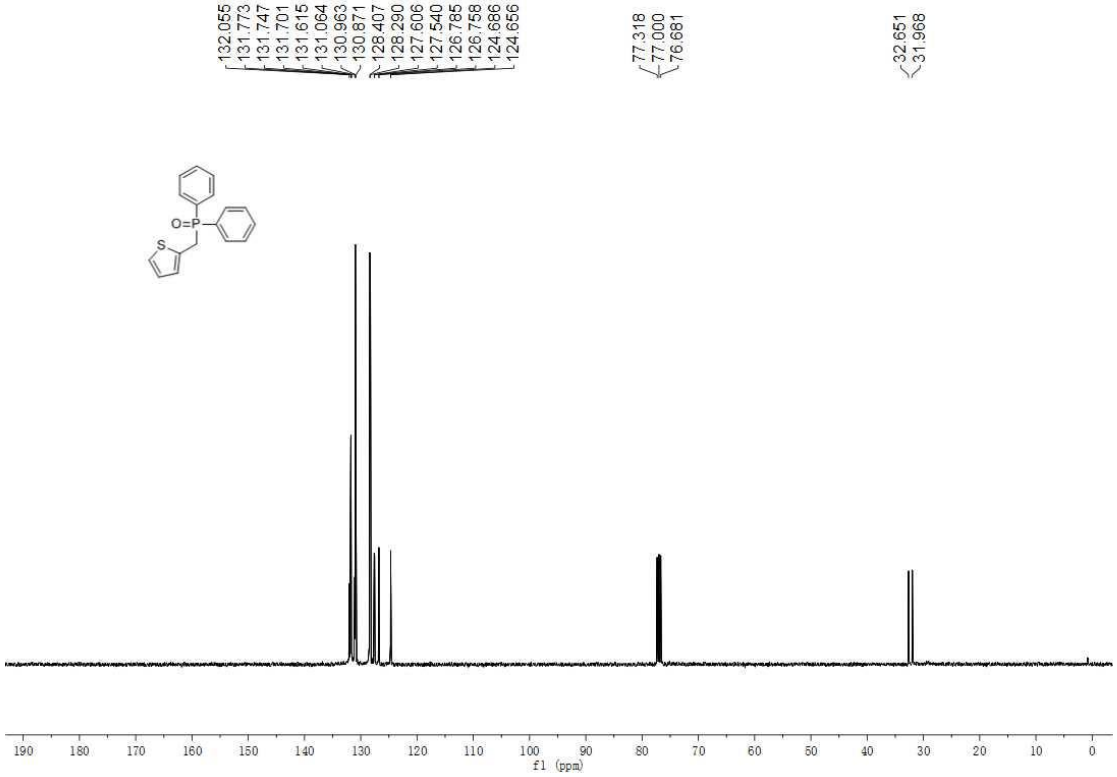

^{31}P NMR Spectrum of **221**

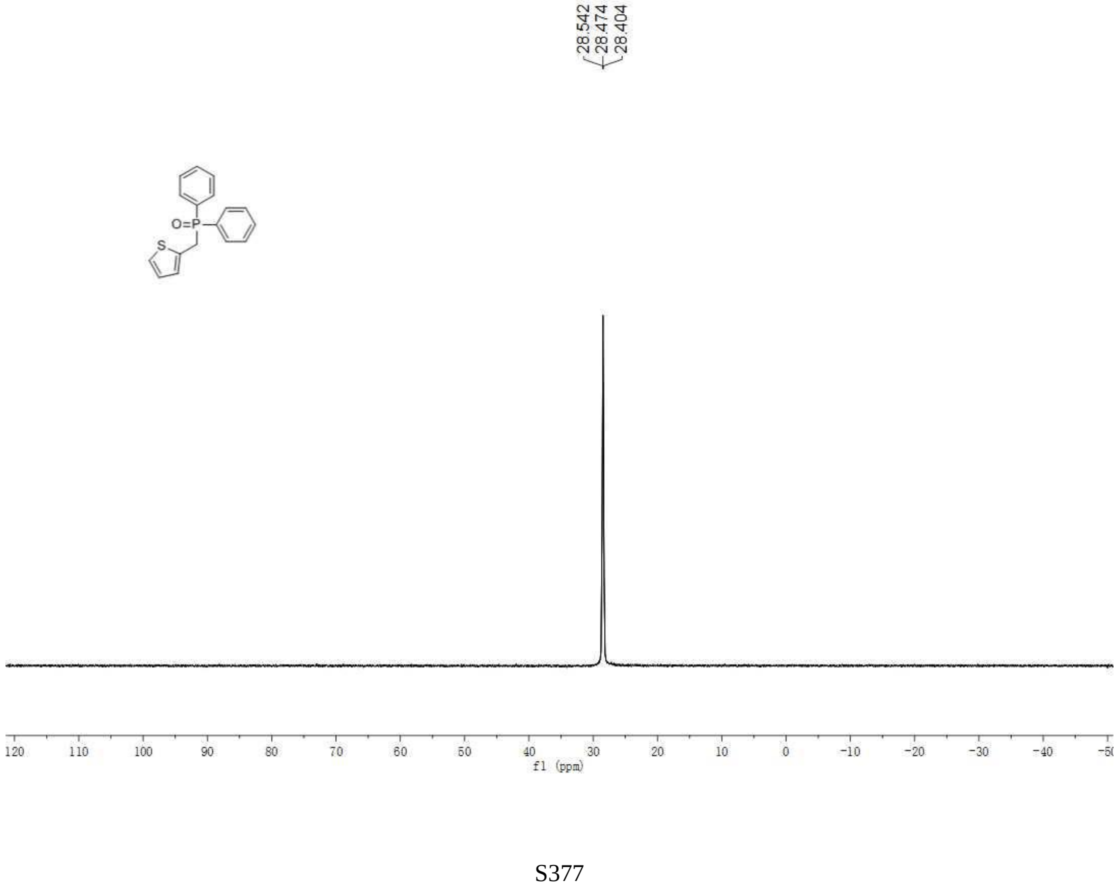

^{1}H NMR Spectrum of **222**

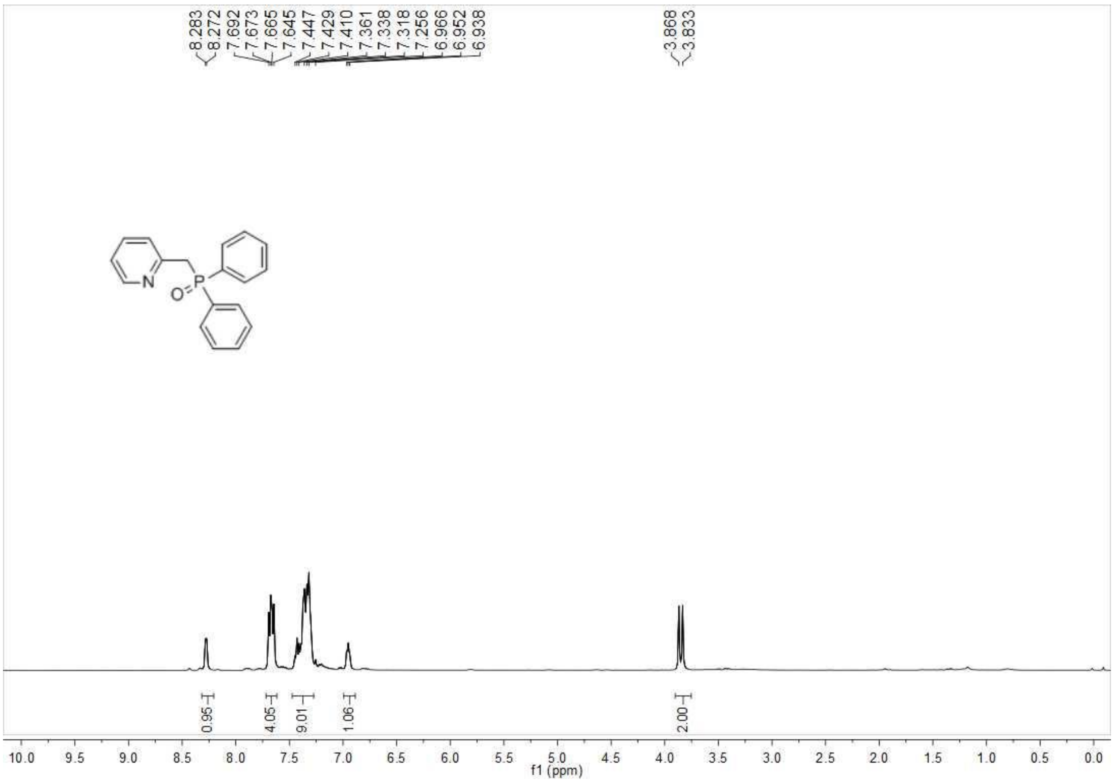

^{13}C NMR Spectrum of **222**

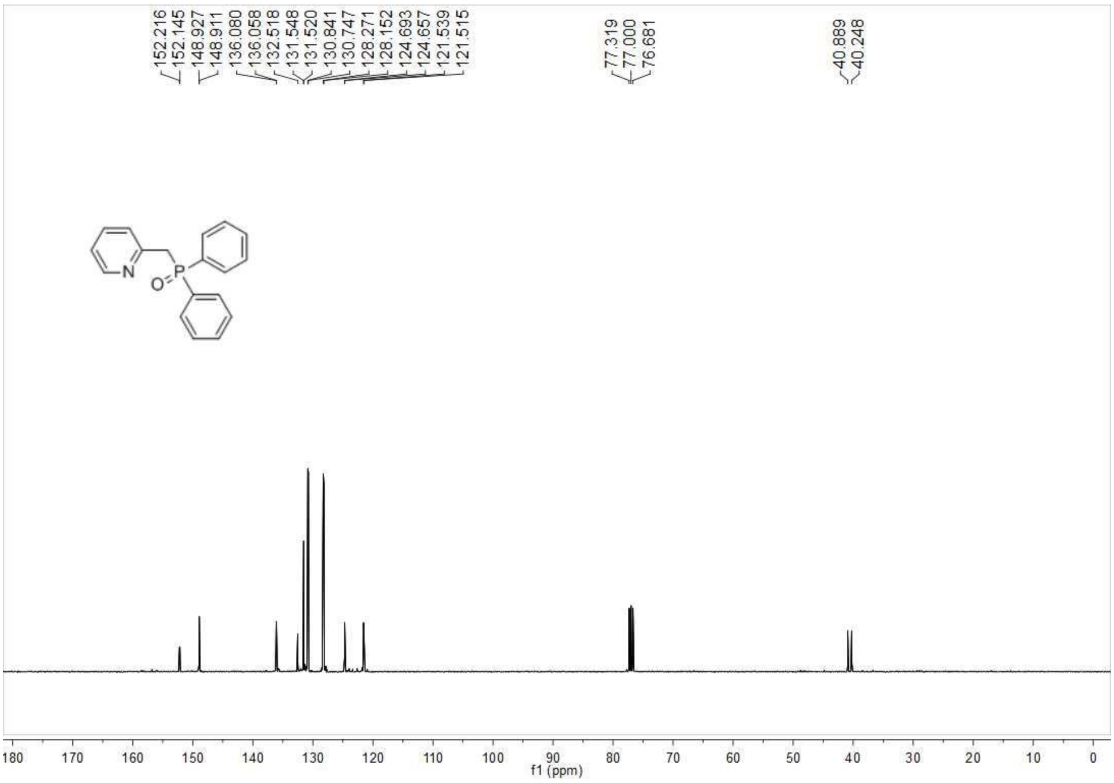

^{31}P NMR Spectrum of **222**

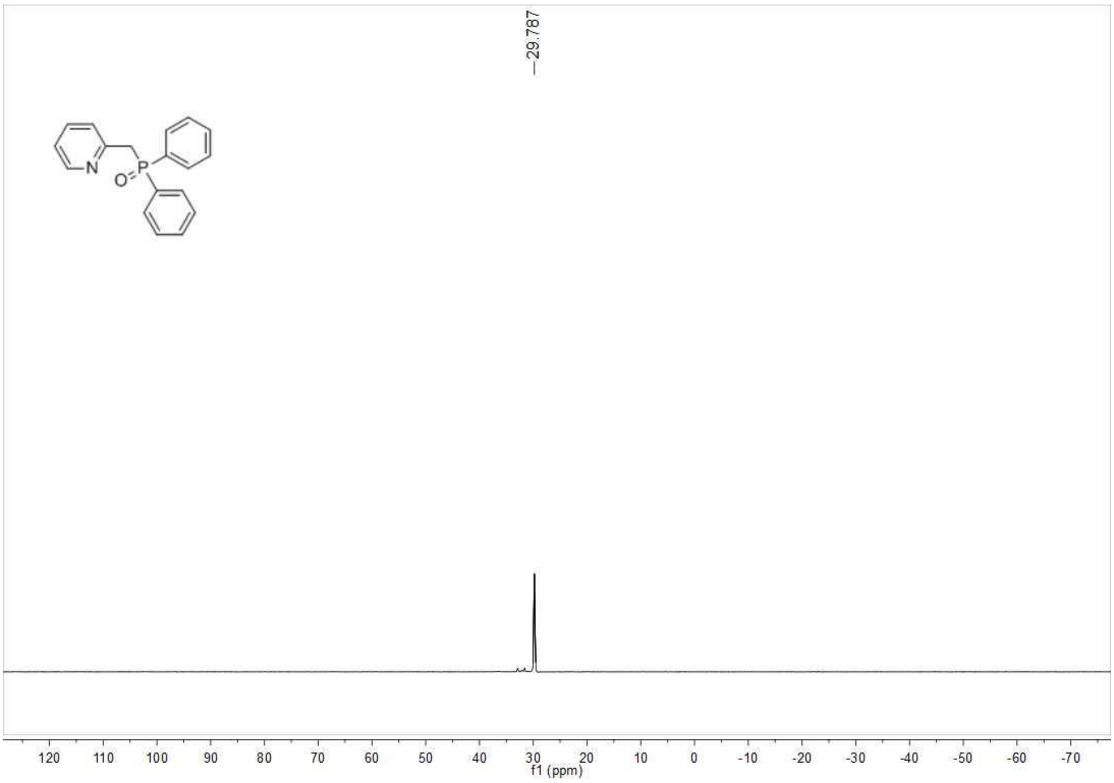

^{1}H NMR Spectrum of **223**

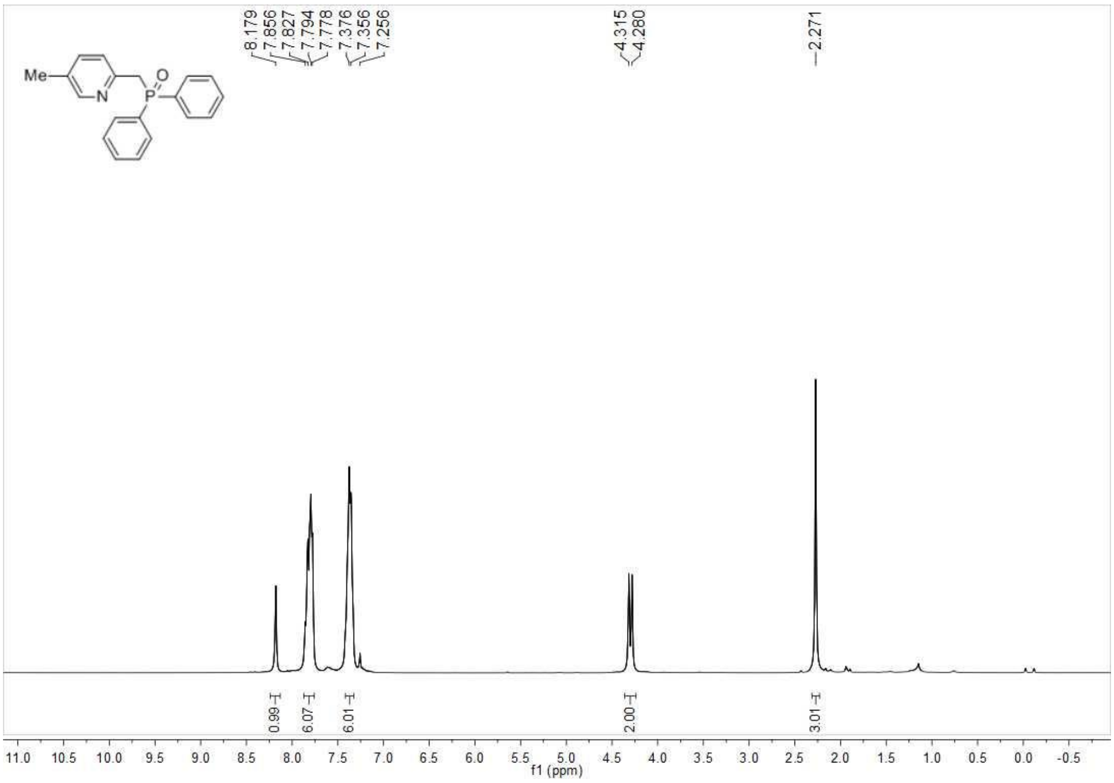

^{13}C NMR Spectrum of **223**

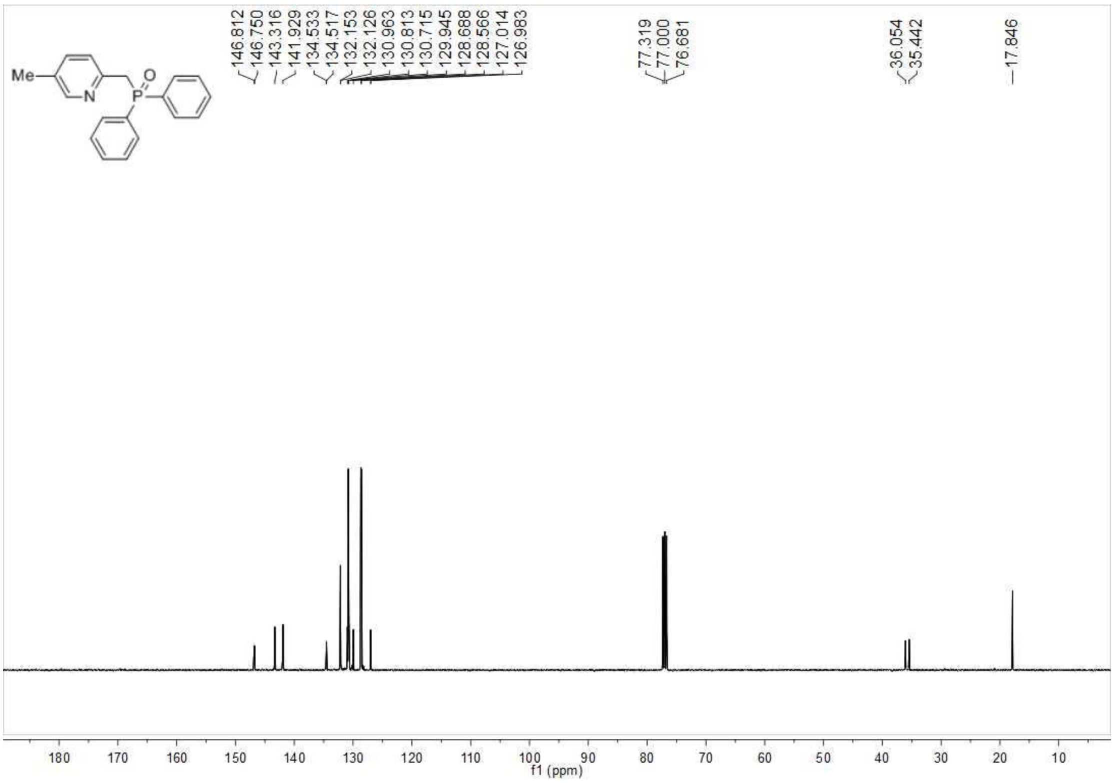

^{31}P NMR Spectrum of **223**

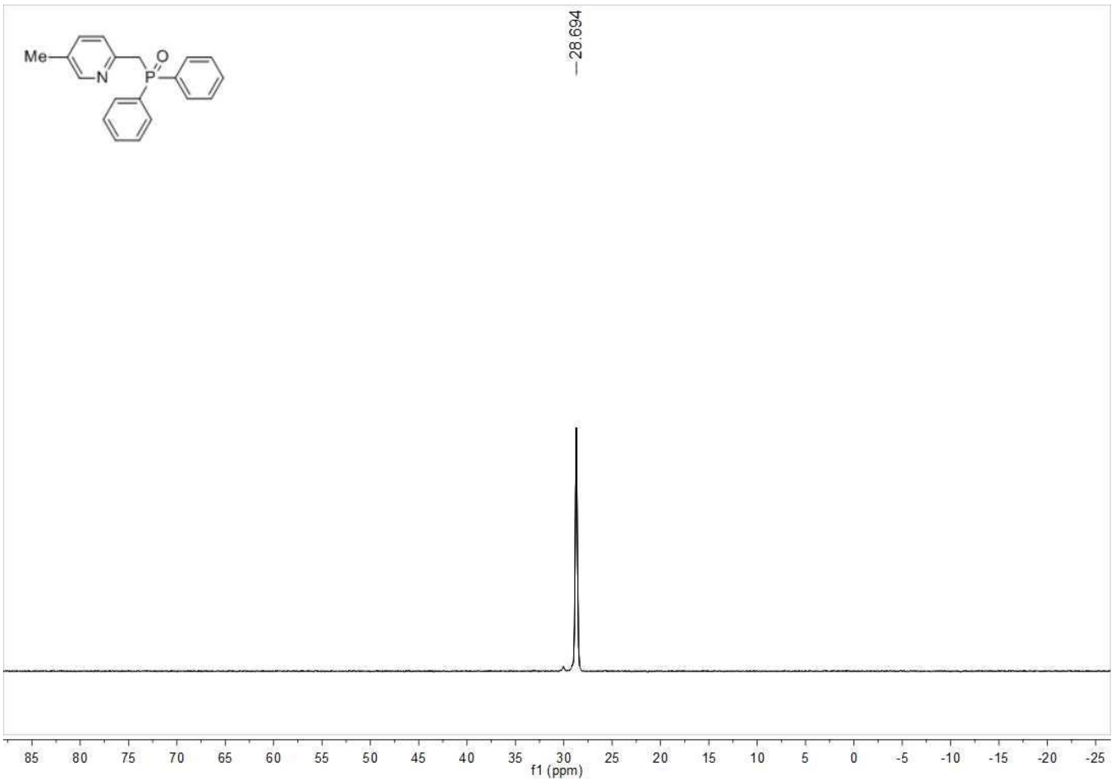

^{1}H NMR Spectrum of **224**

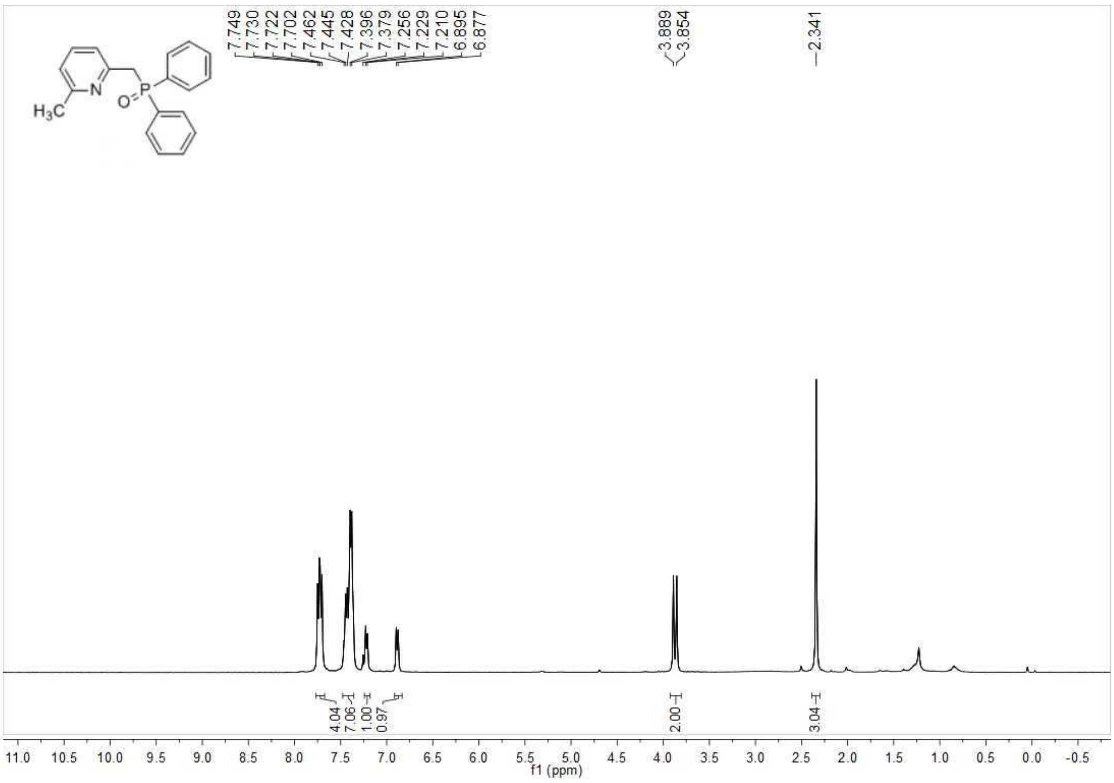

^{13}C NMR Spectrum of **224**

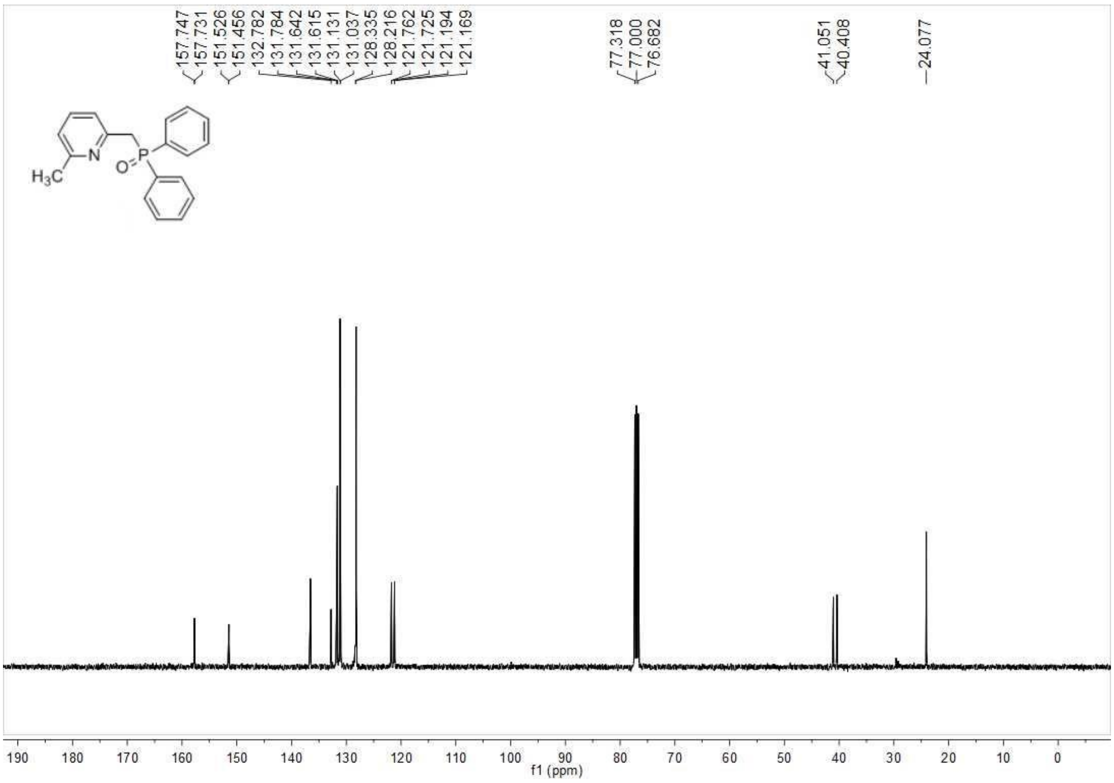

^{31}P NMR Spectrum of **224**

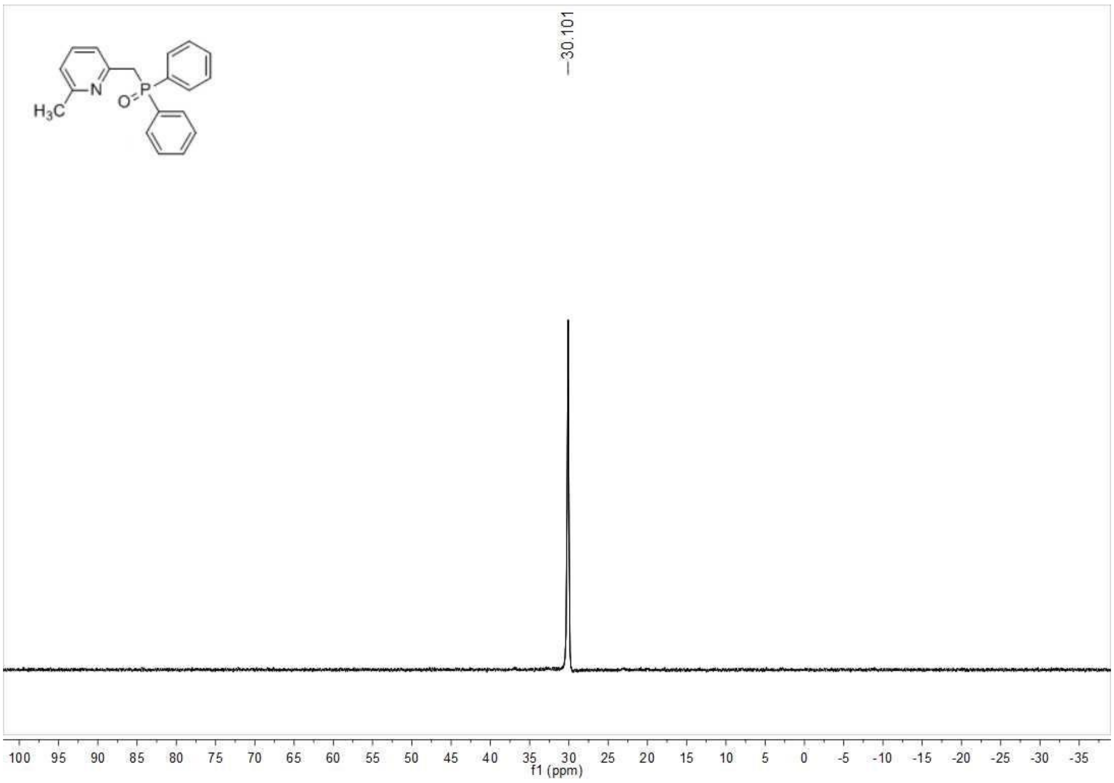

^{1}H NMR Spectrum of **225**

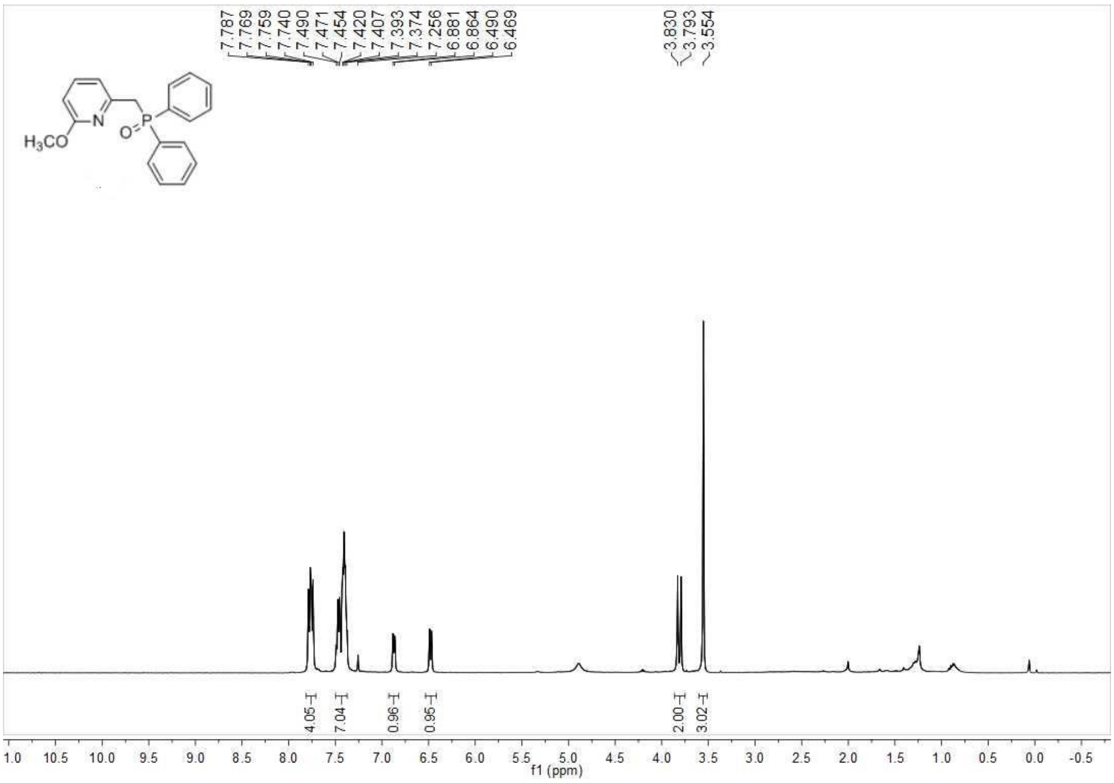

^{13}C NMR Spectrum of **225**

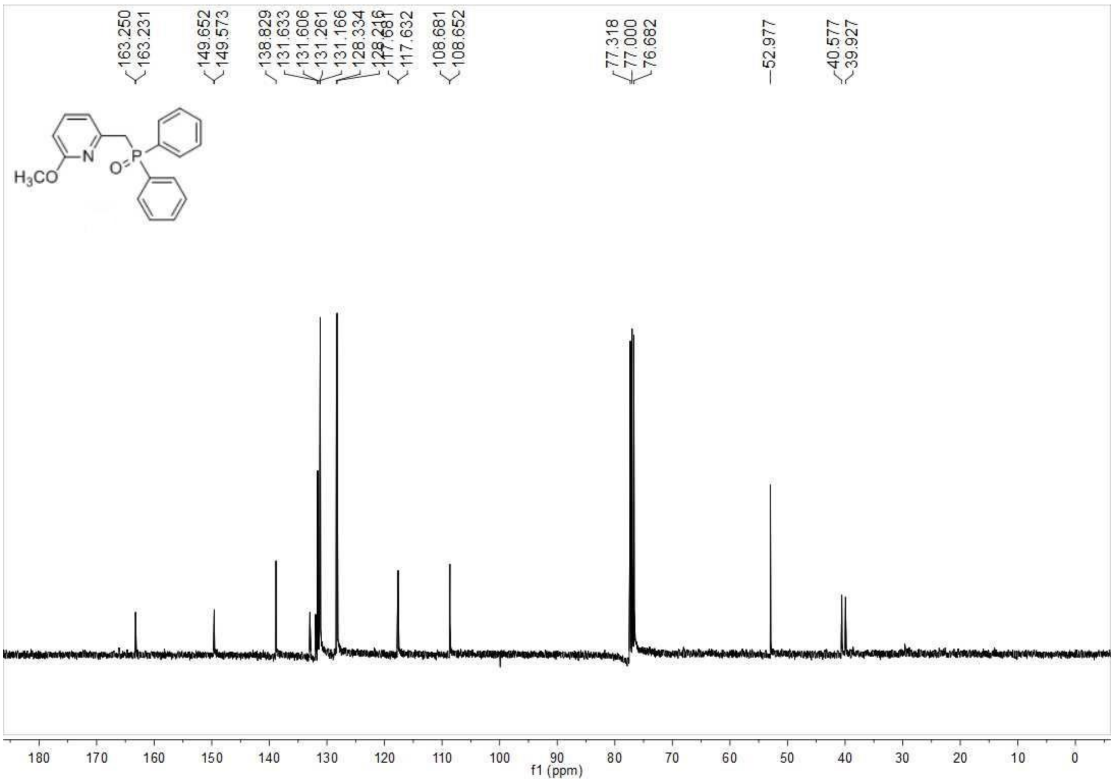

^{31}P NMR Spectrum of **225**

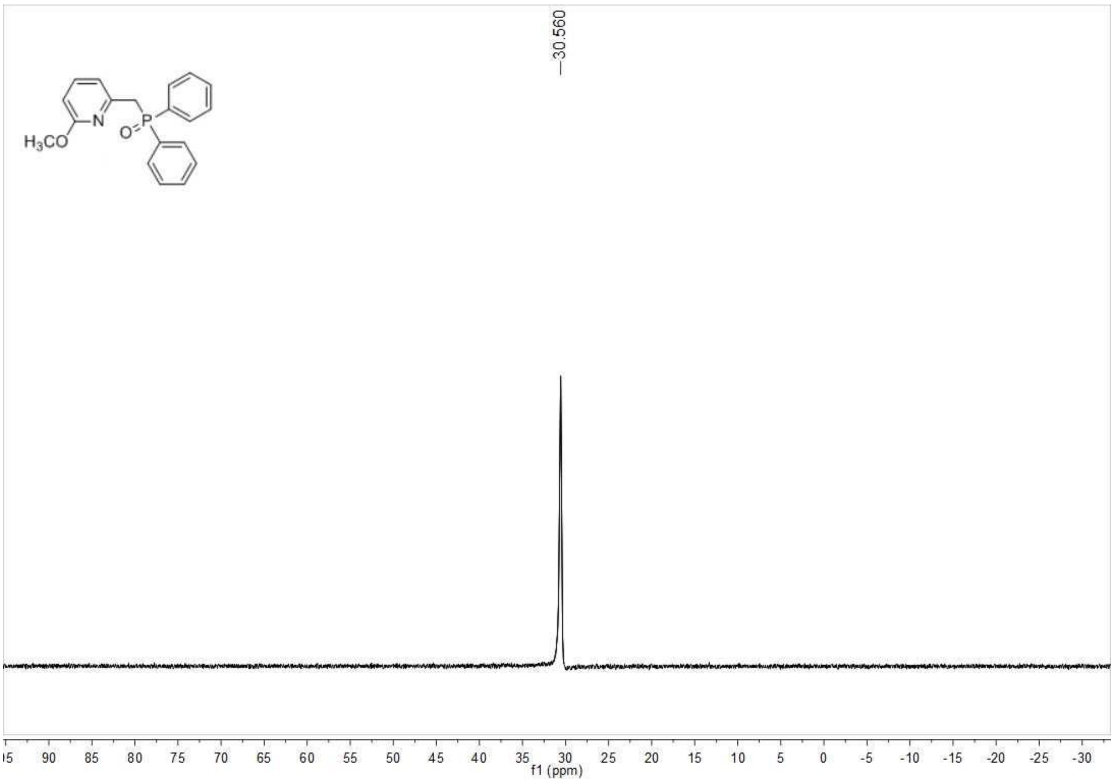

^{1}H NMR Spectrum of **226**

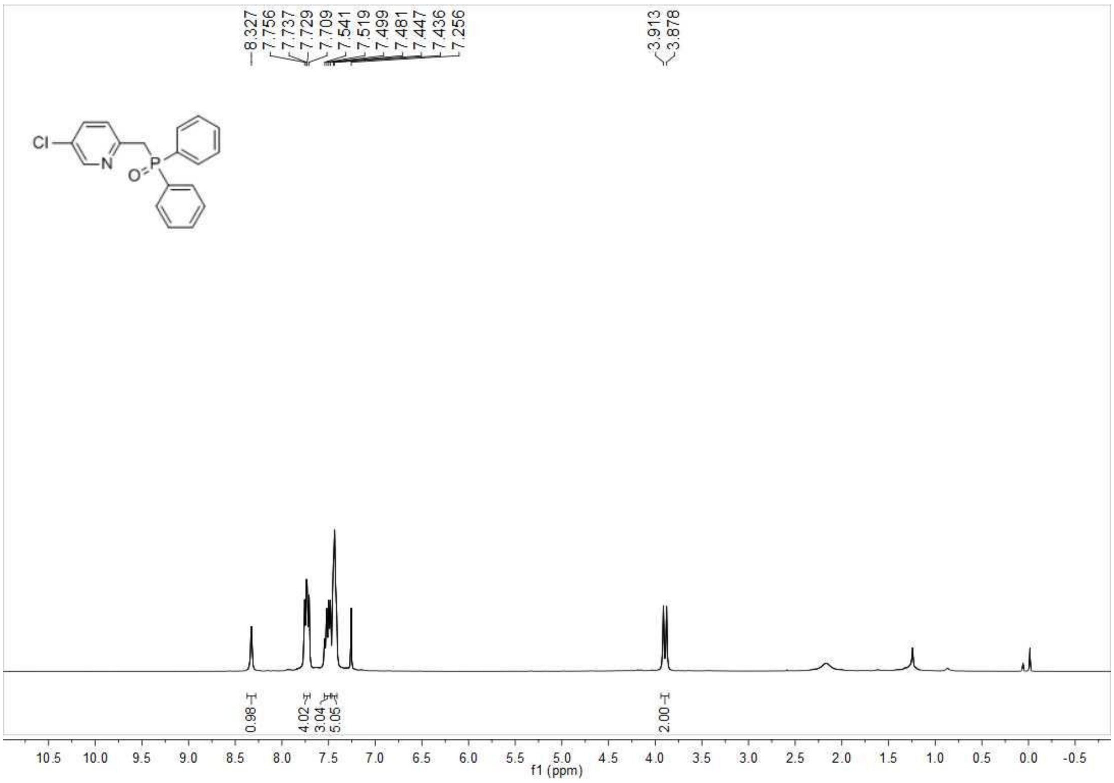

^{13}C NMR Spectrum of **226**

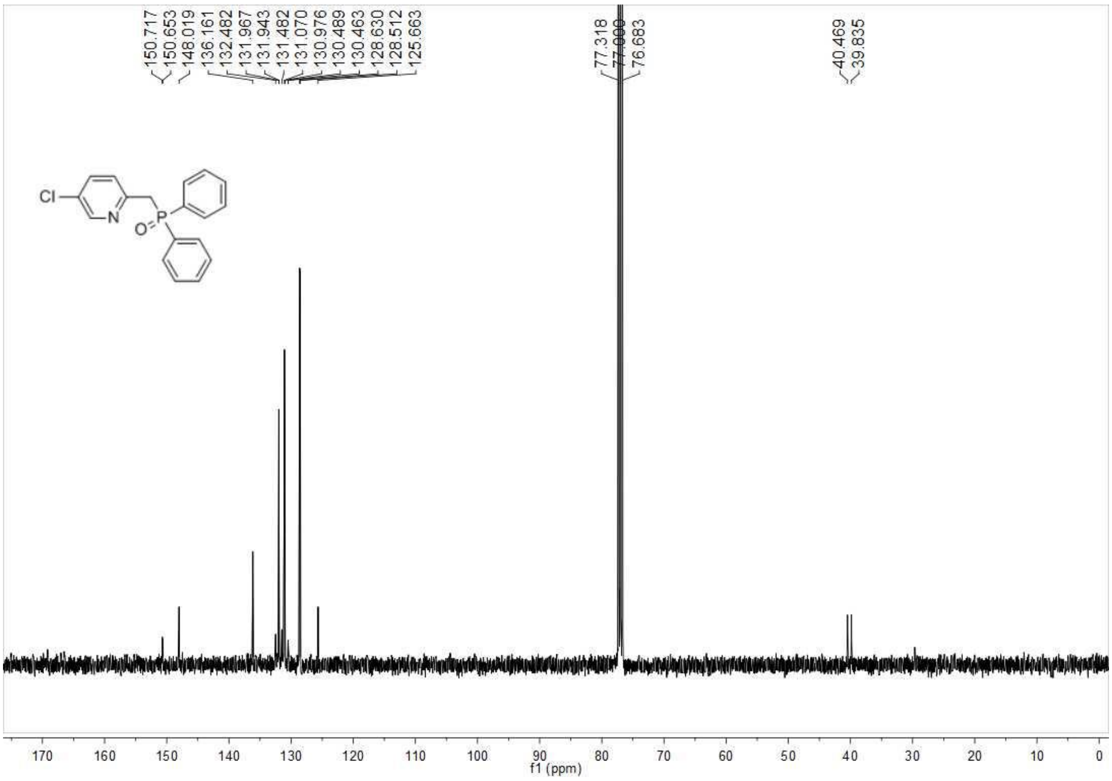

^{31}P NMR Spectrum of **226**

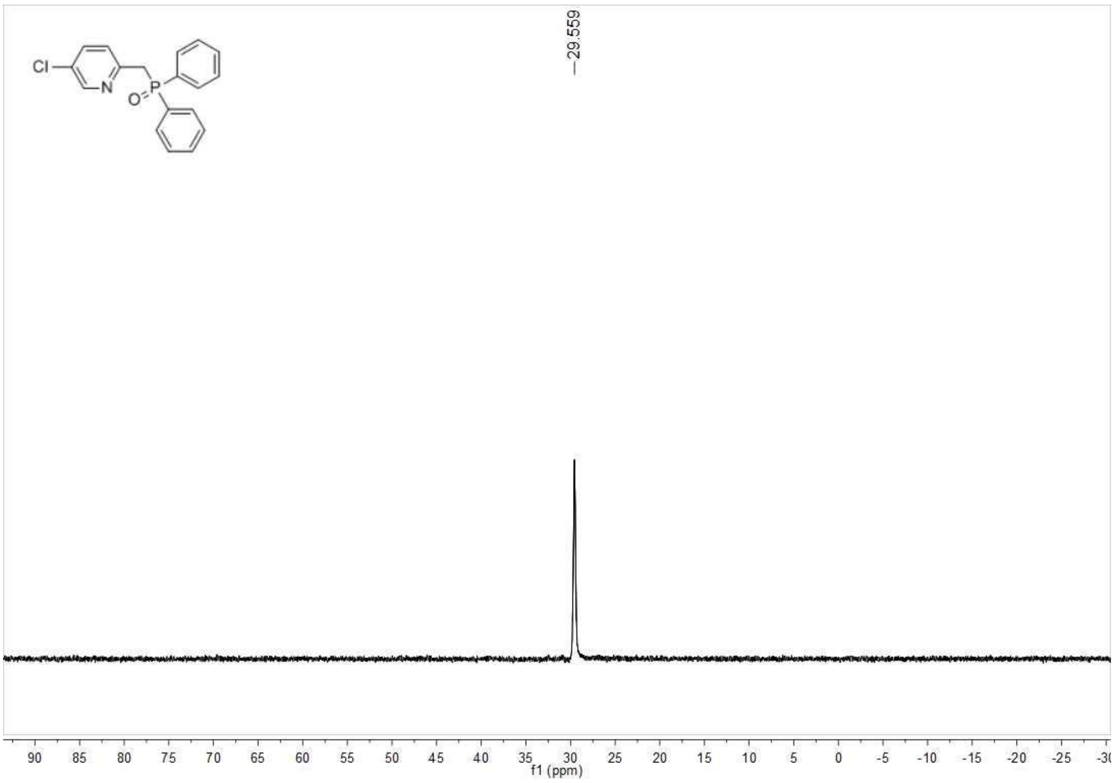

^{1}H NMR Spectrum of **227**

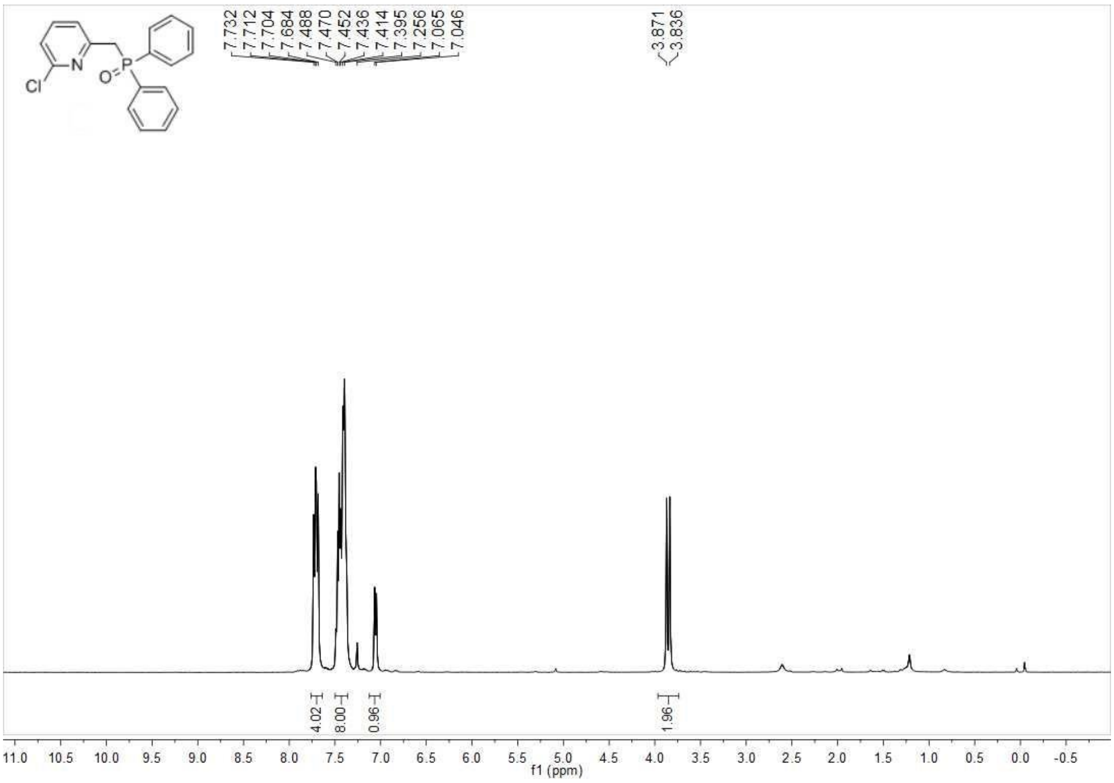

^{13}C NMR Spectrum of **227**

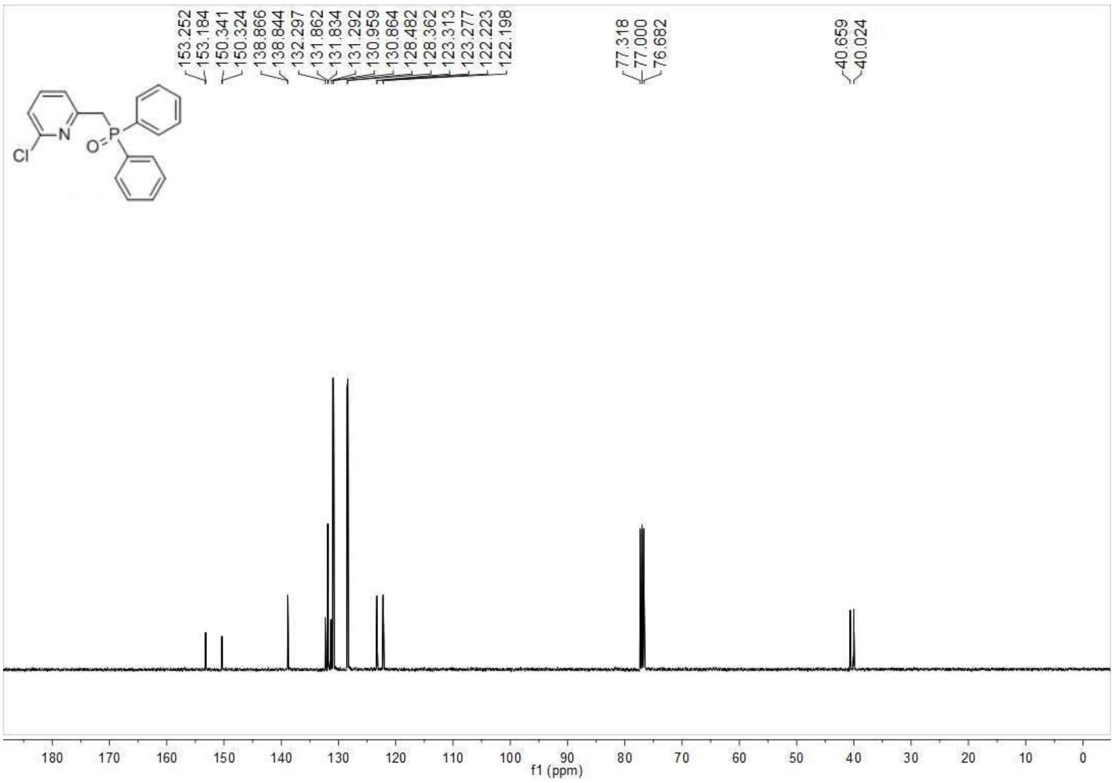

^{31}P NMR Spectrum of **227**

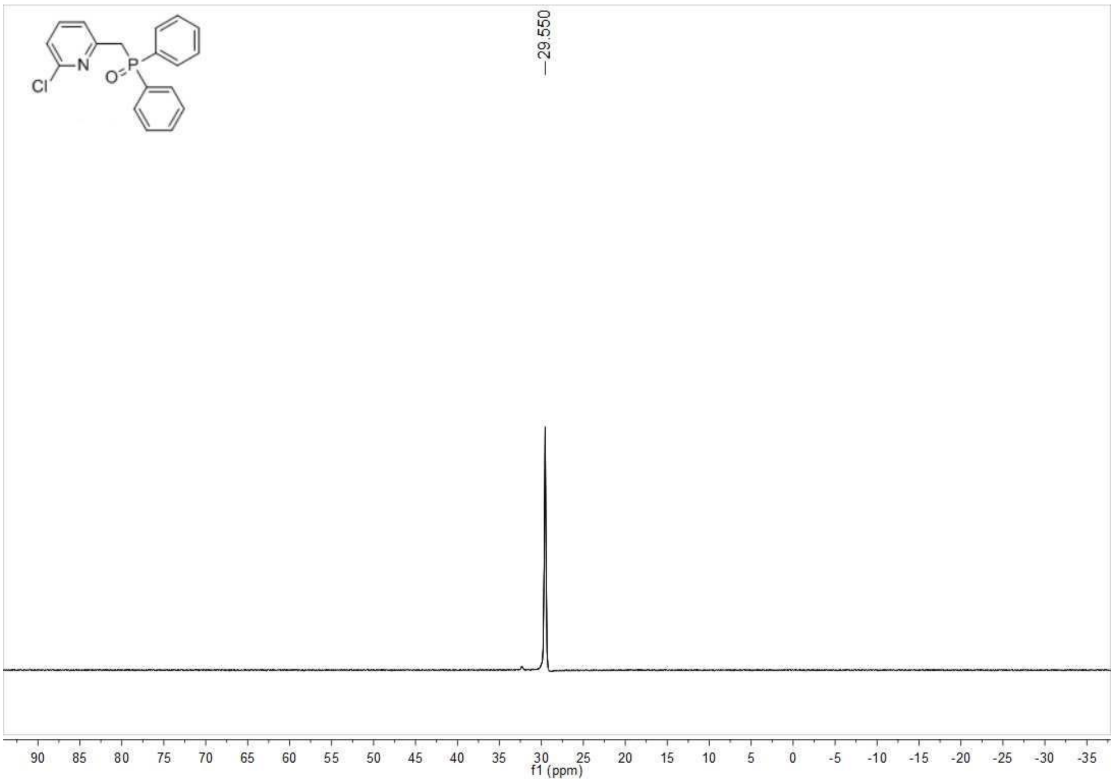

^{1}H NMR Spectrum of **228**

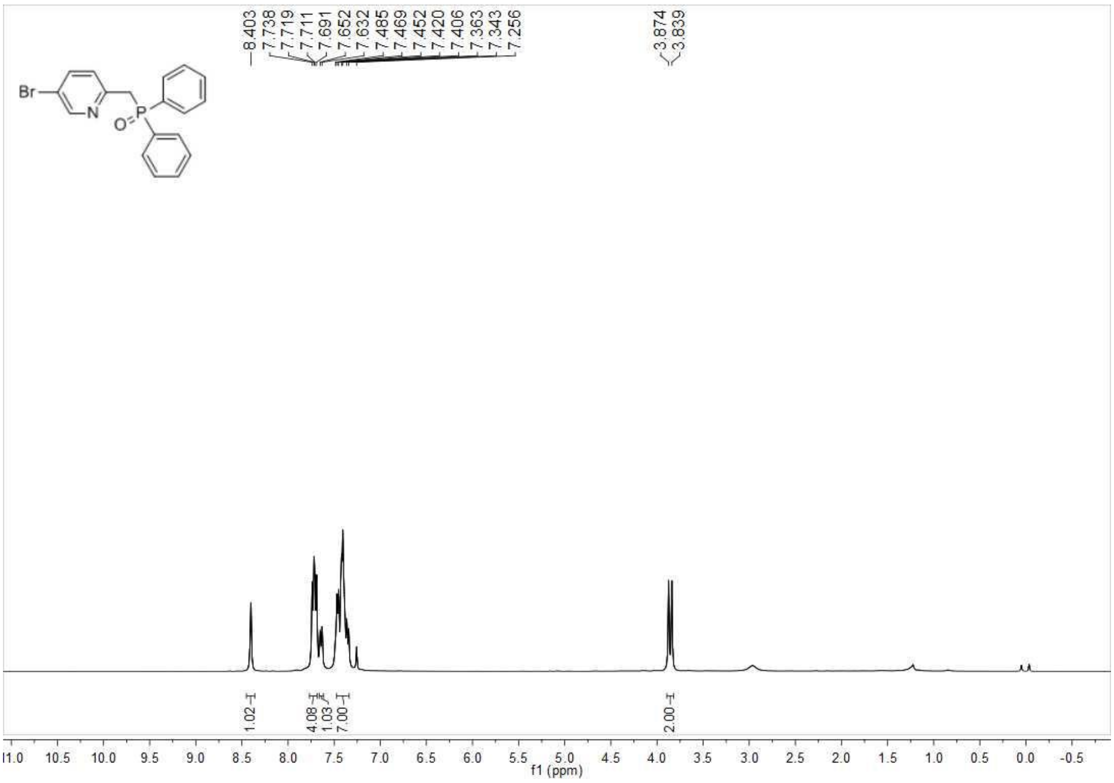

^{13}C NMR Spectrum of **228**

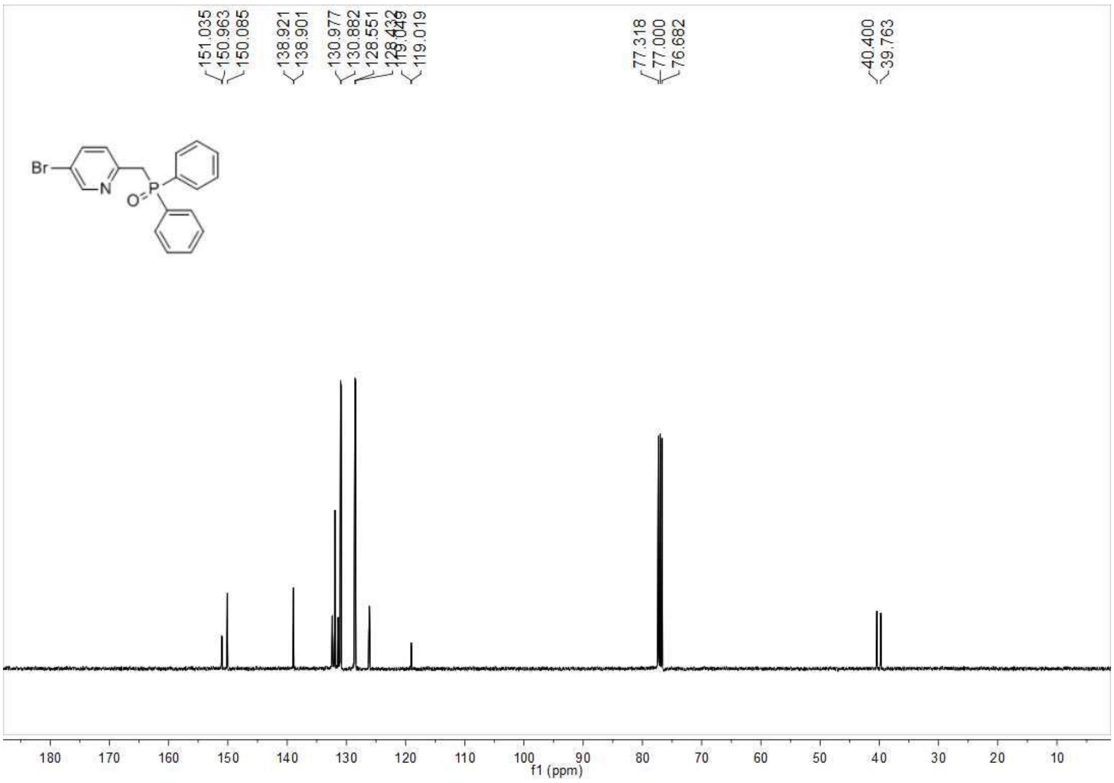

^{31}P NMR Spectrum of **228**

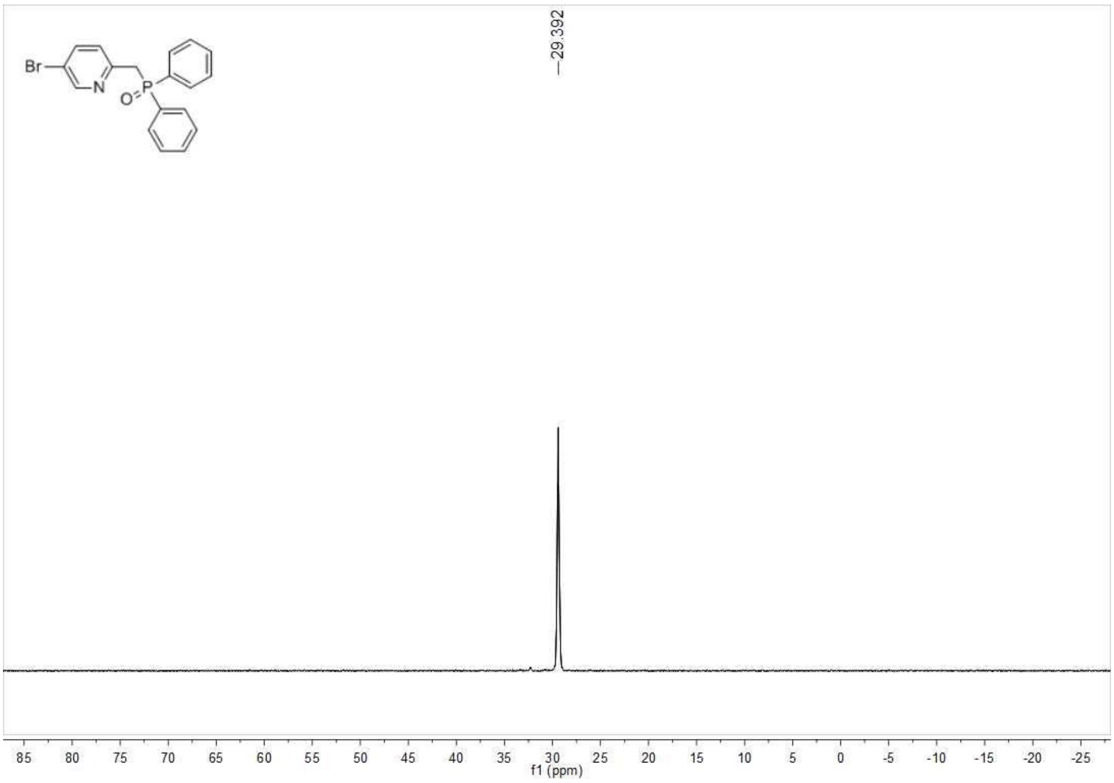

^{1}H NMR Spectrum of **229**

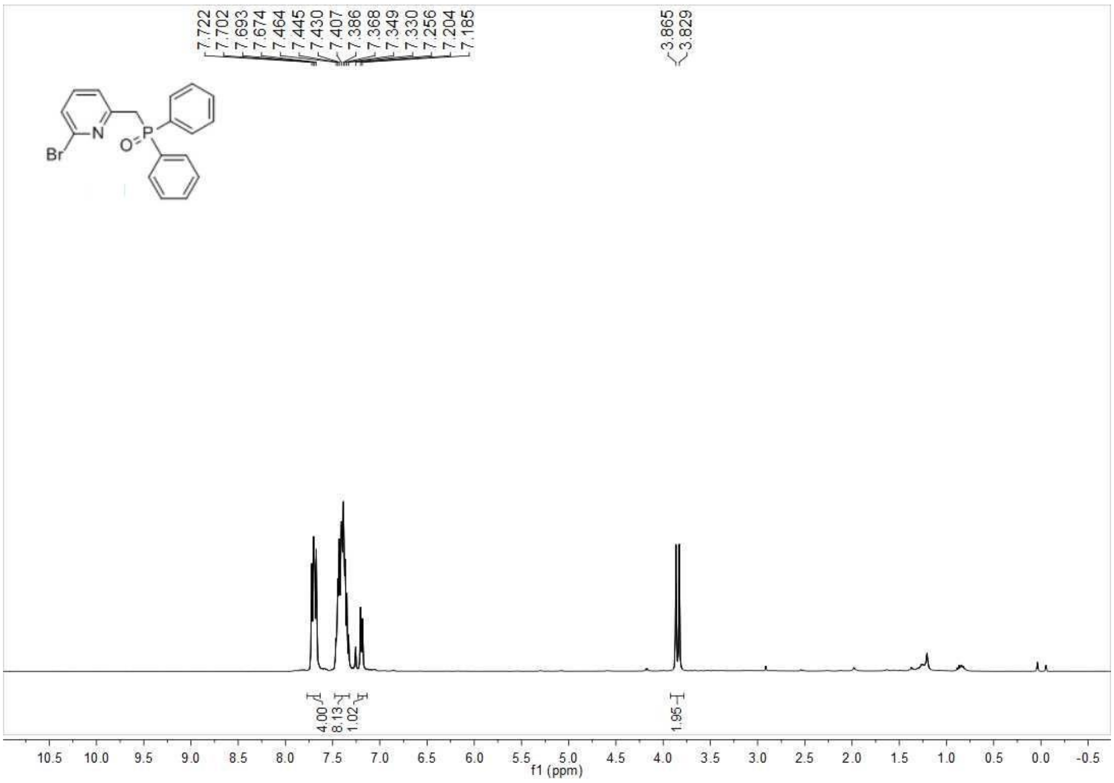

^{13}C NMR Spectrum of **229**

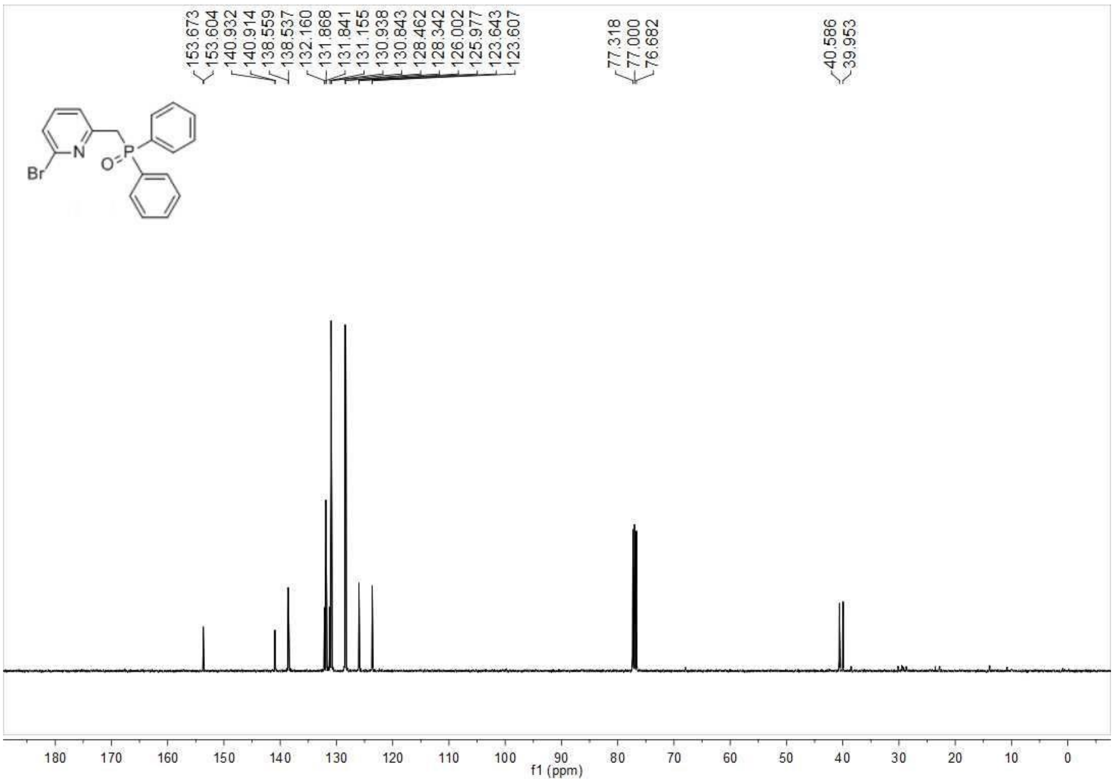

^{31}P NMR Spectrum of **229**

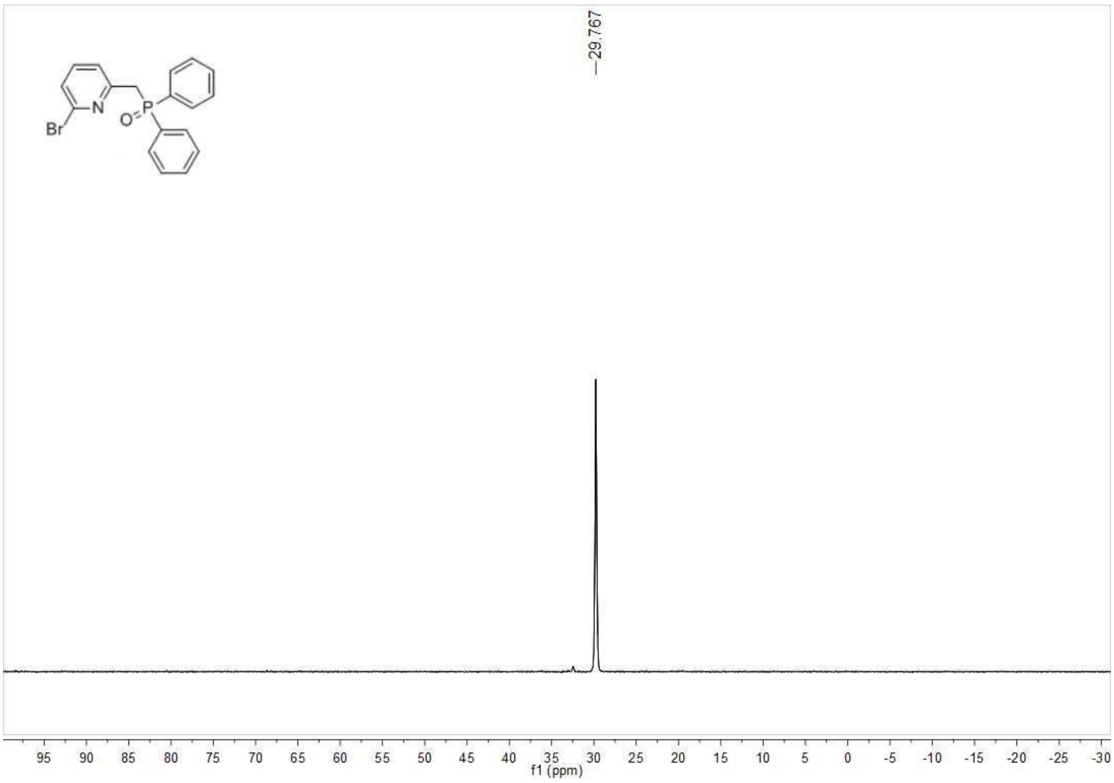

^{1}H NMR Spectrum of **230**

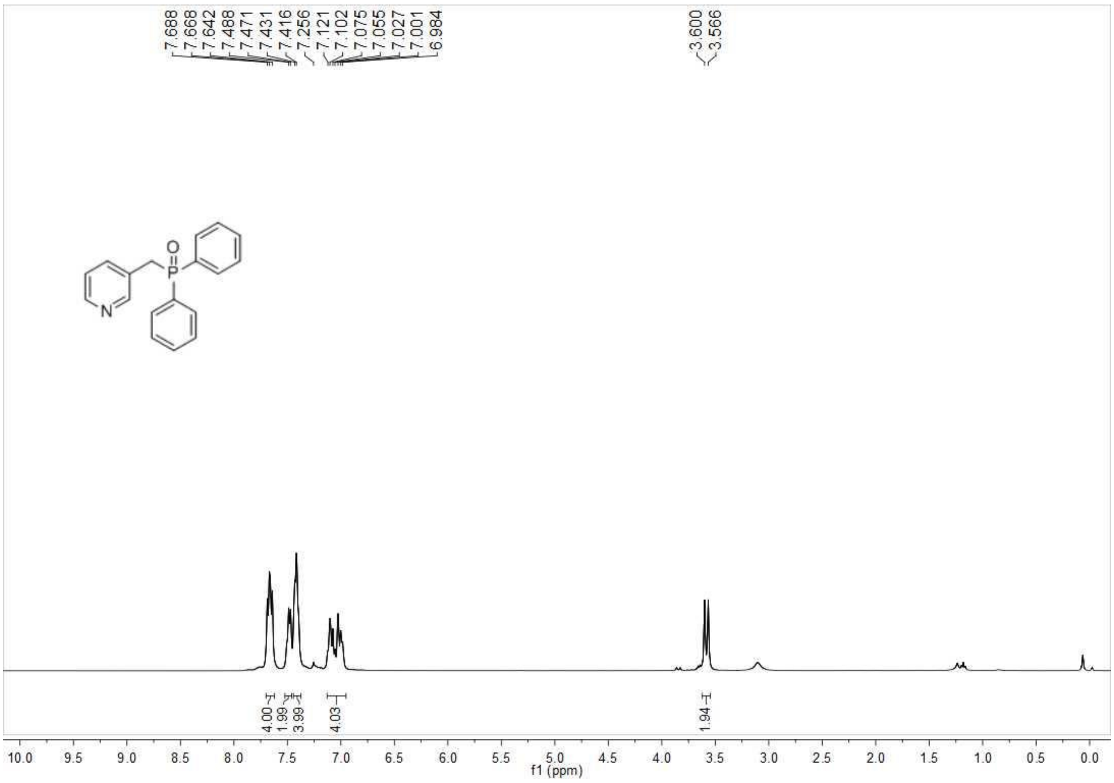

^{13}C NMR Spectrum of **230**

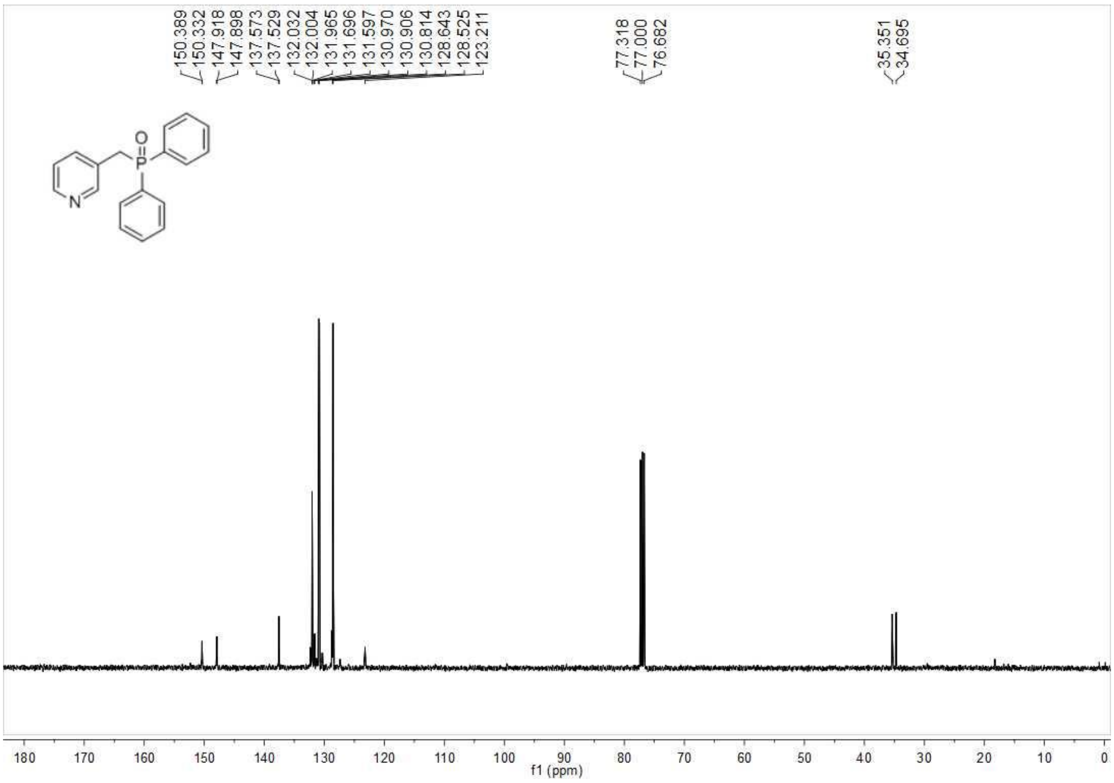

^{31}P NMR Spectrum of **230**

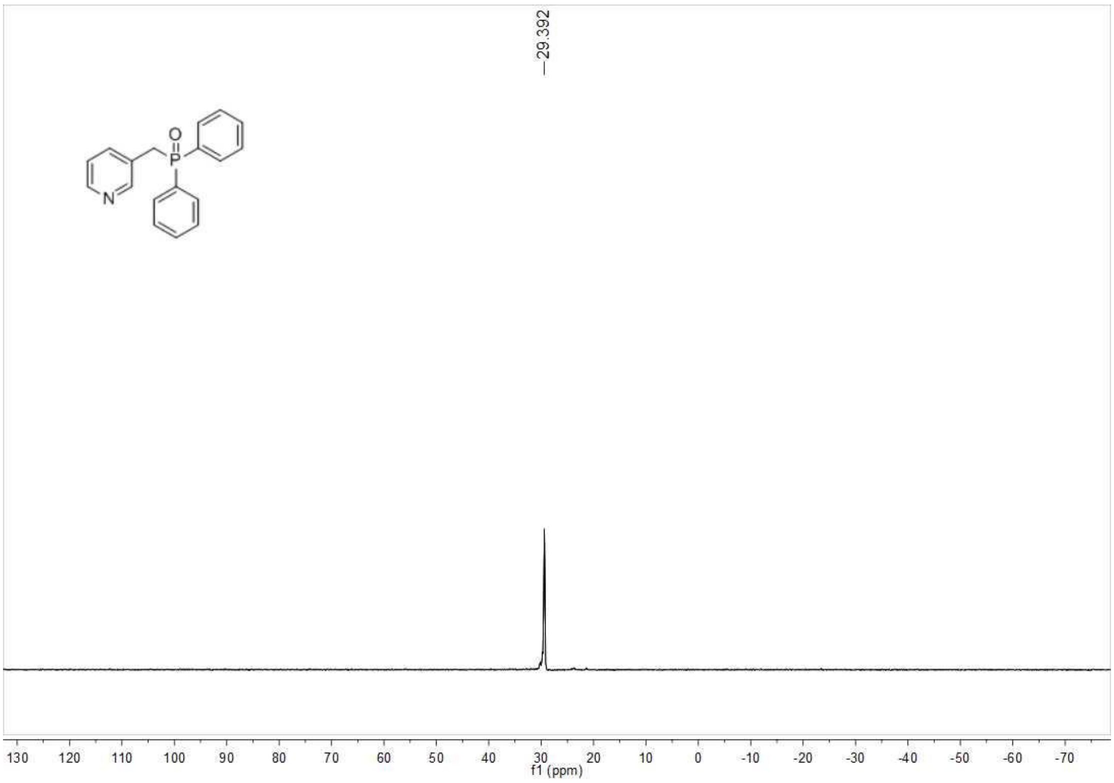

^{1}H NMR Spectrum of **231**

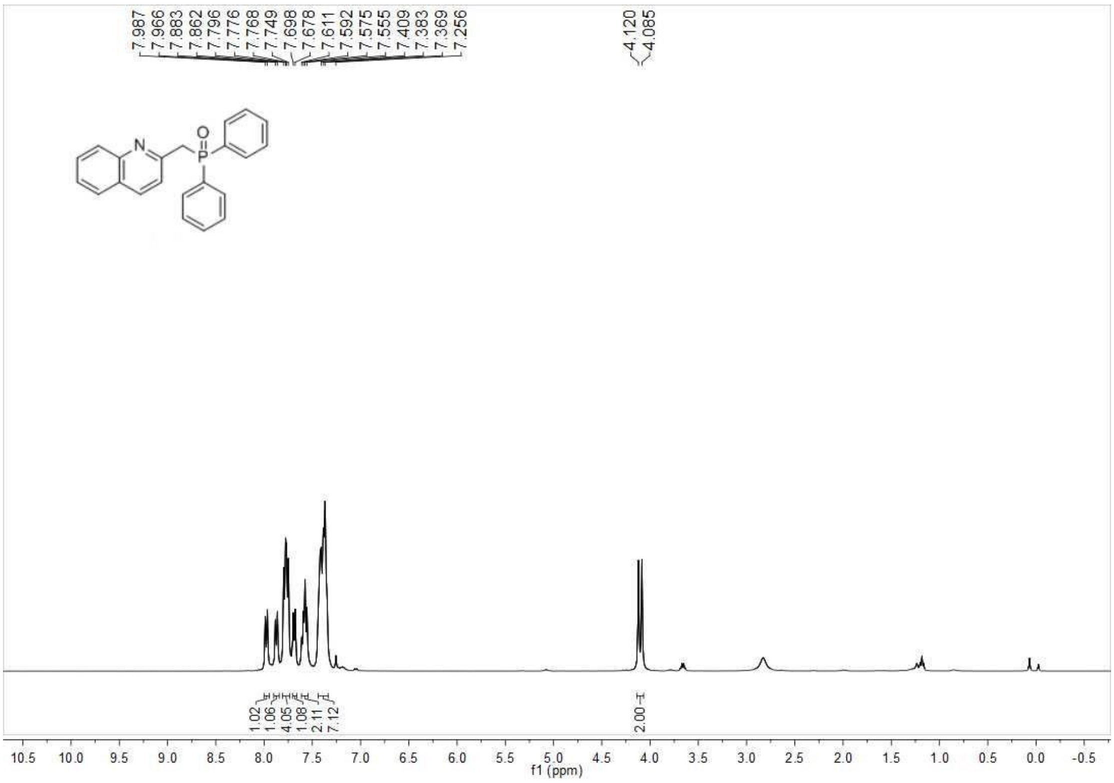

^{13}C NMR Spectrum of **231**

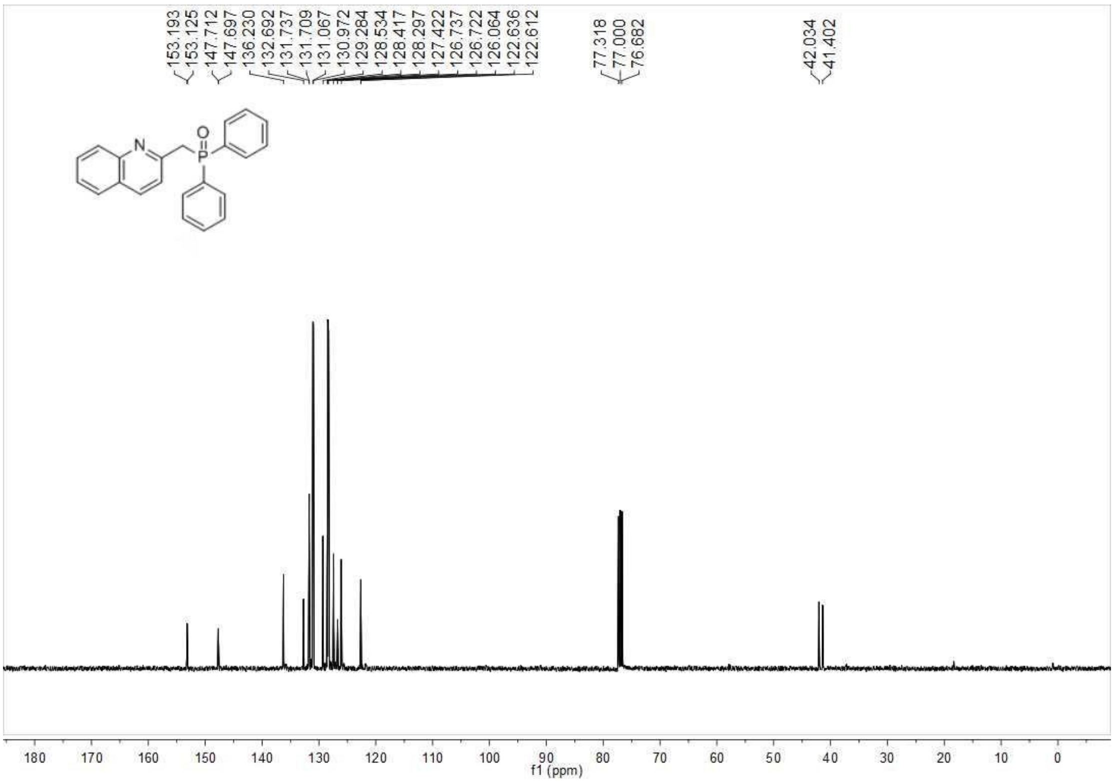

^{31}P NMR Spectrum of **231**

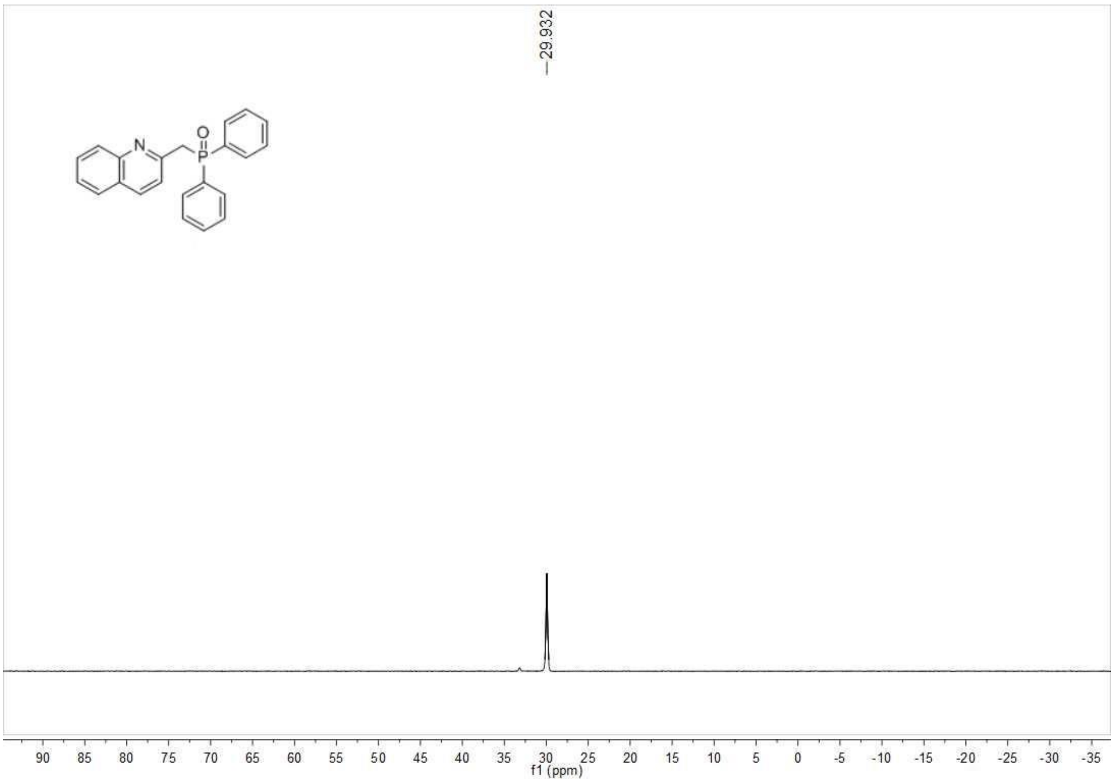

^{1}H NMR Spectrum of **232**

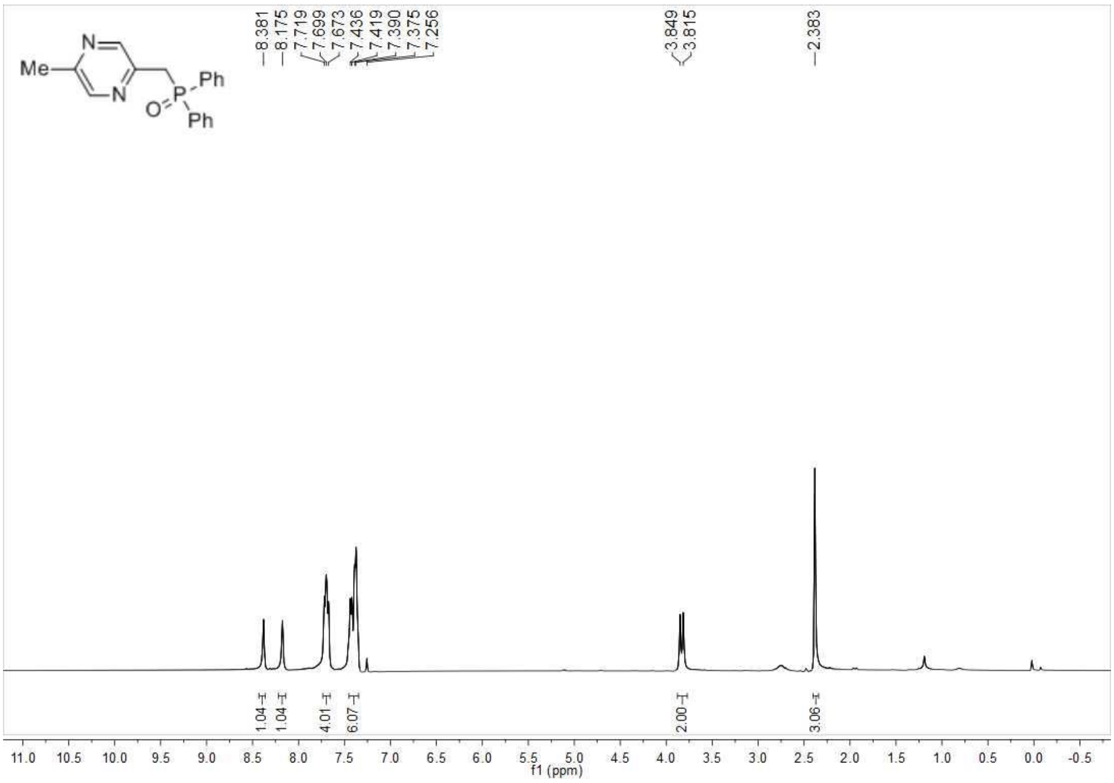

^{13}C NMR Spectrum of **232**

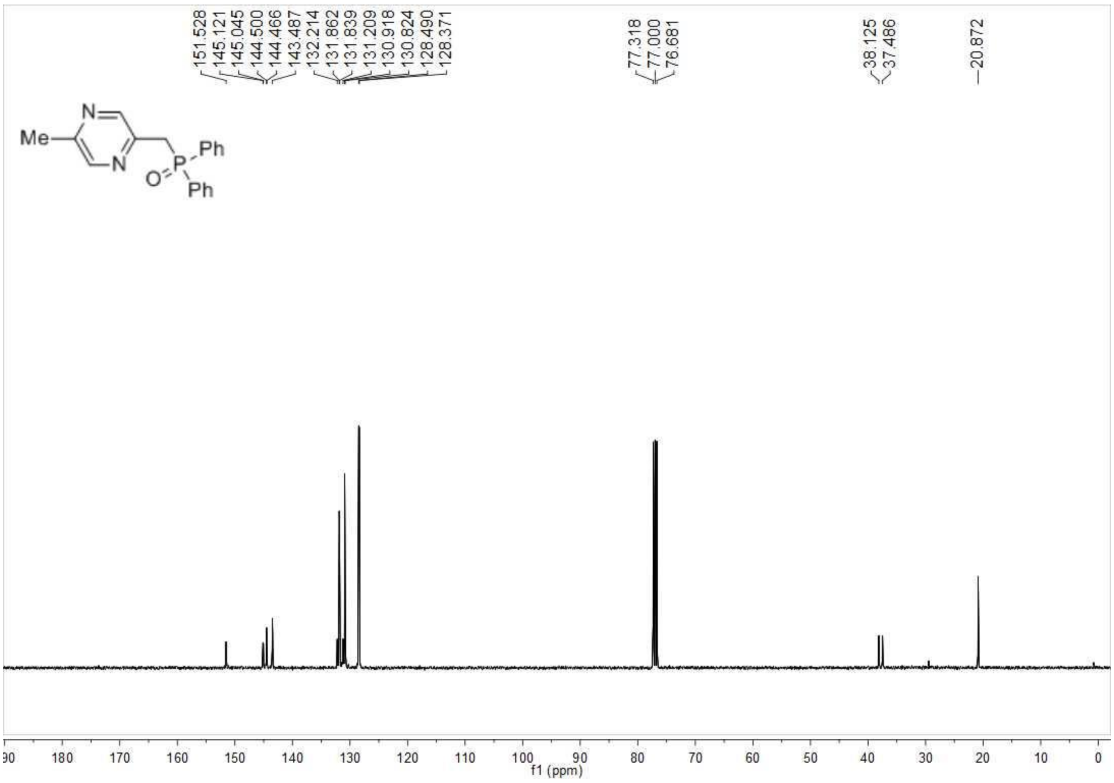

^{31}P NMR Spectrum of **232**

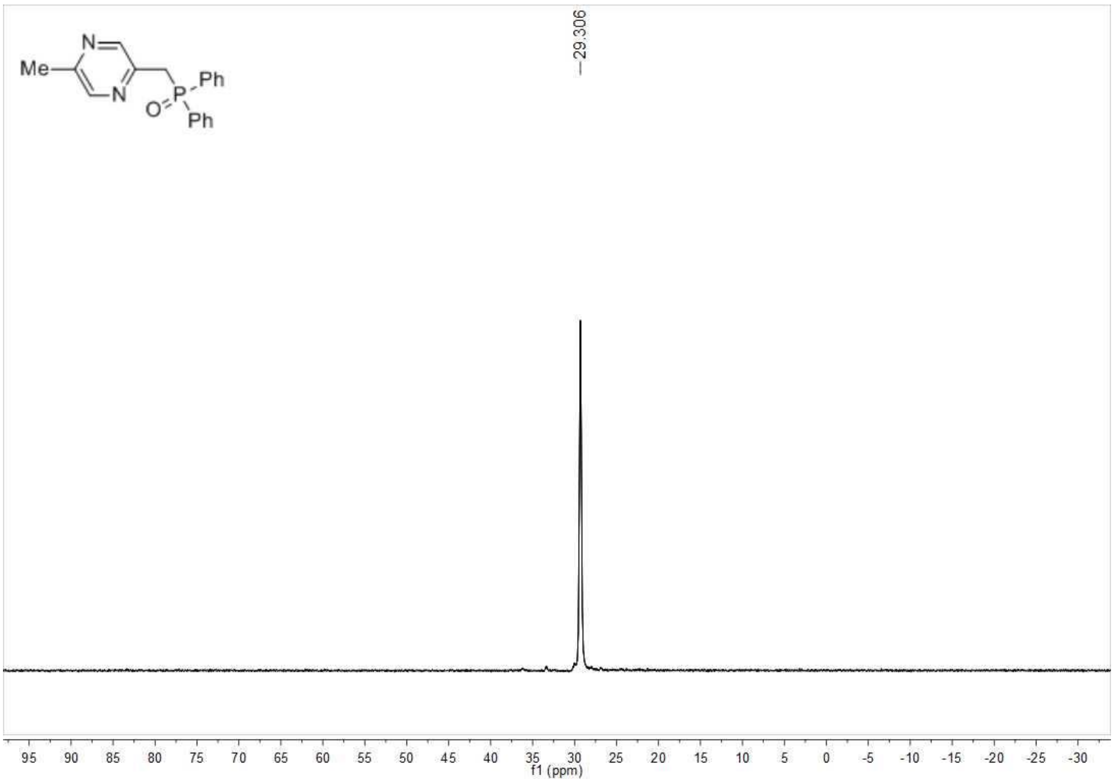

^{1}H NMR Spectrum of **233**

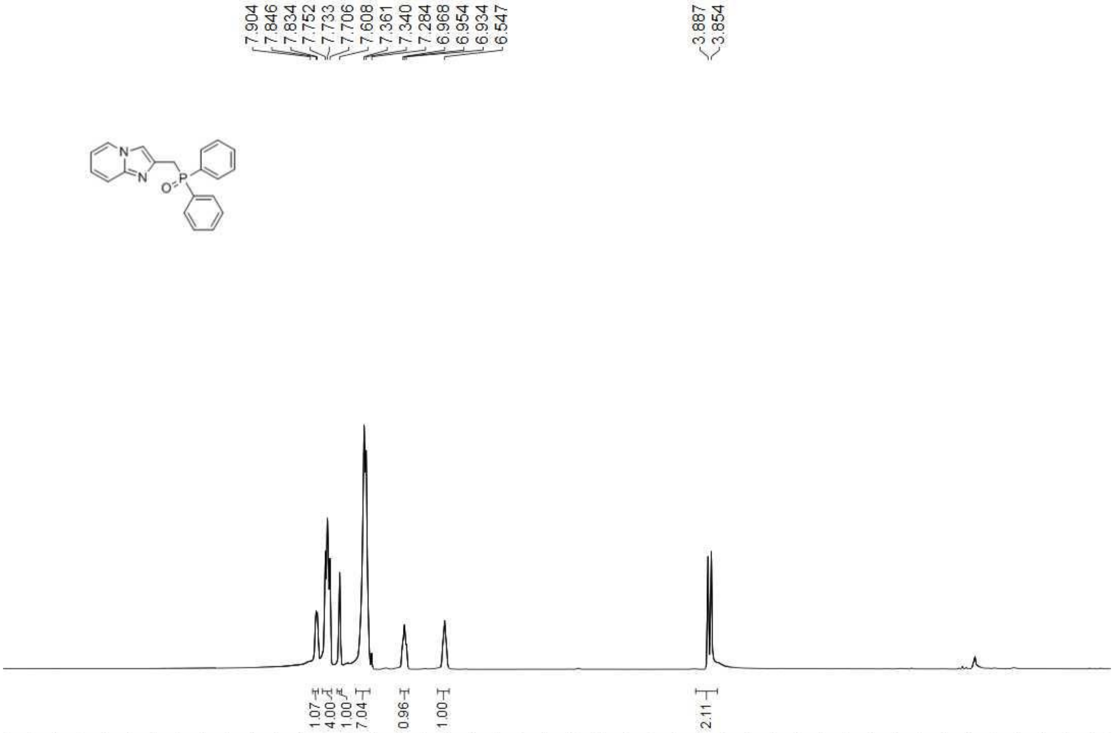

^{13}C NMR Spectrum of **233**

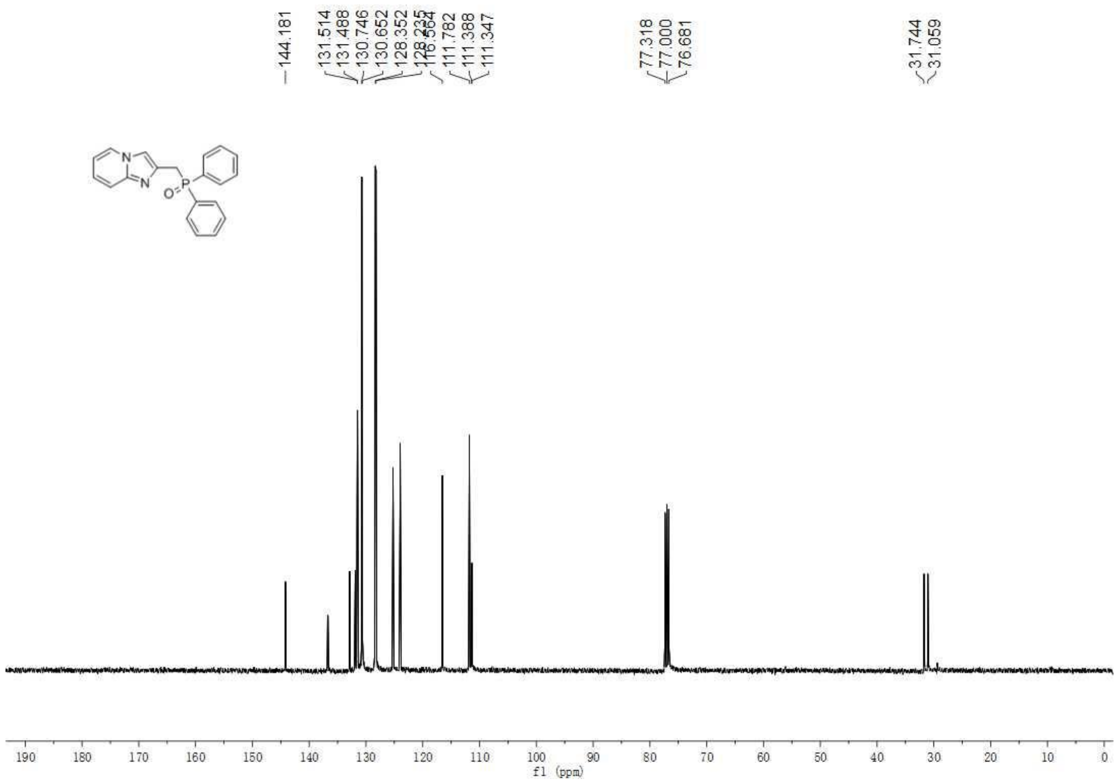

^{31}P NMR Spectrum of **233**

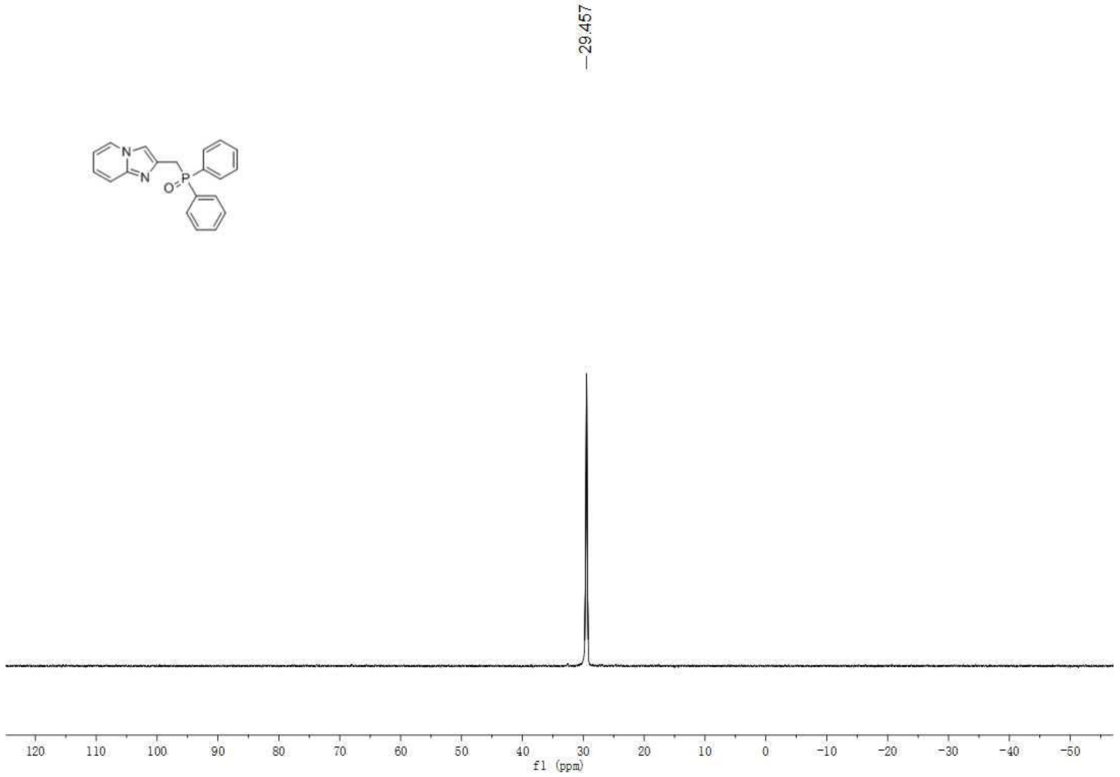

^{1}H NMR Spectrum of **234**

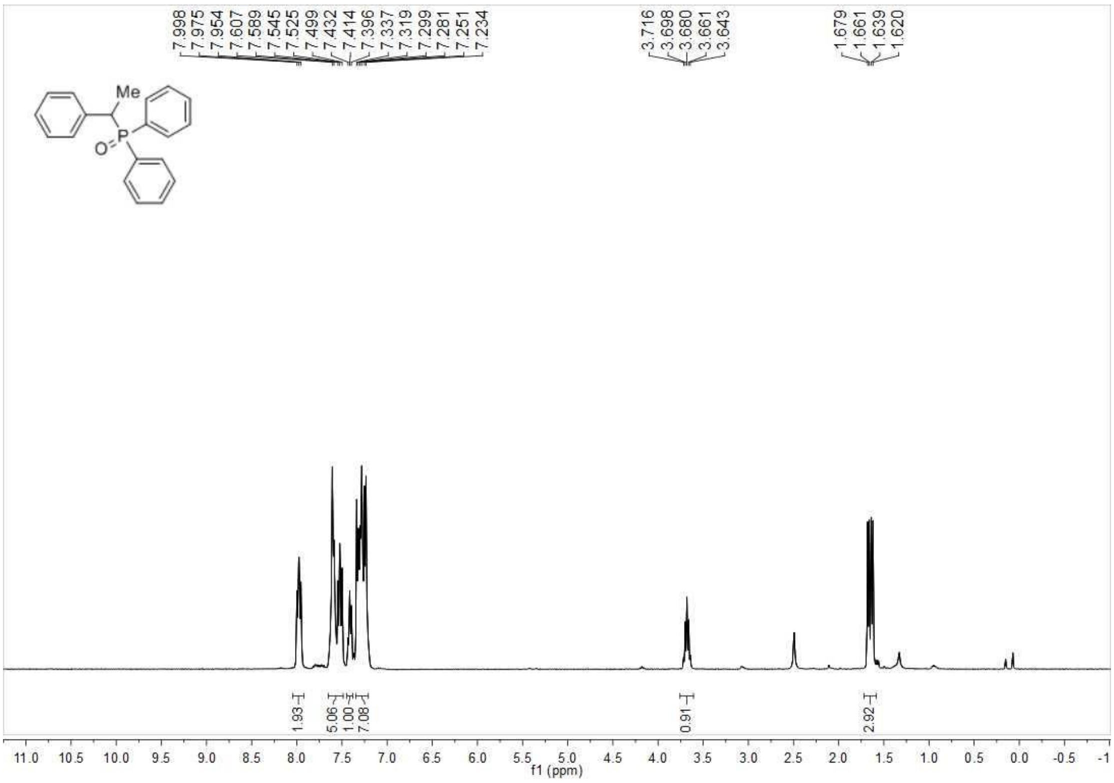

^{13}C NMR Spectrum of **234**

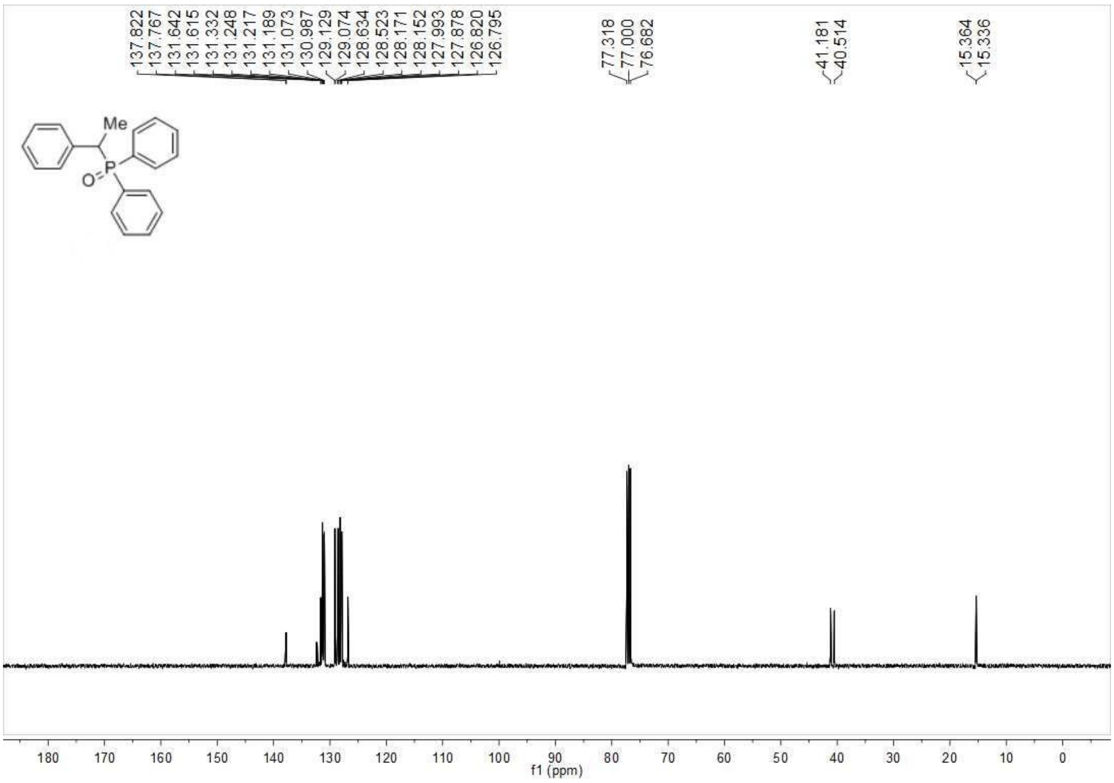

^{31}P NMR Spectrum of **234**

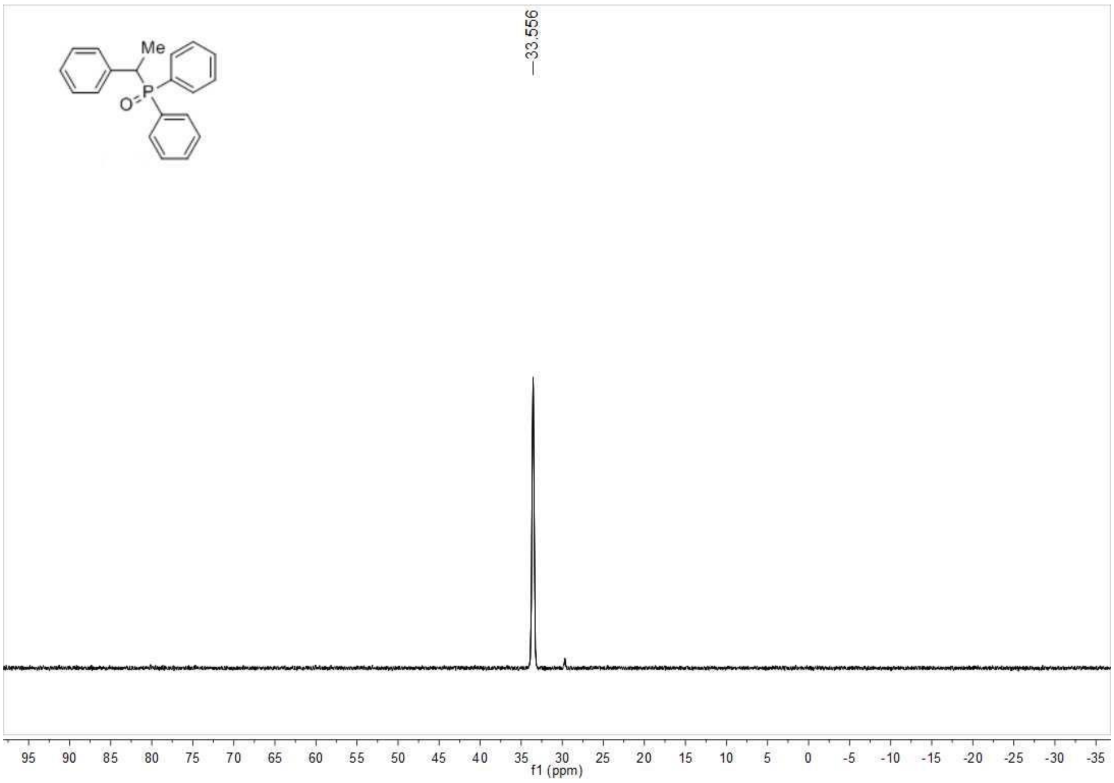

^{1}H NMR Spectrum of **235**

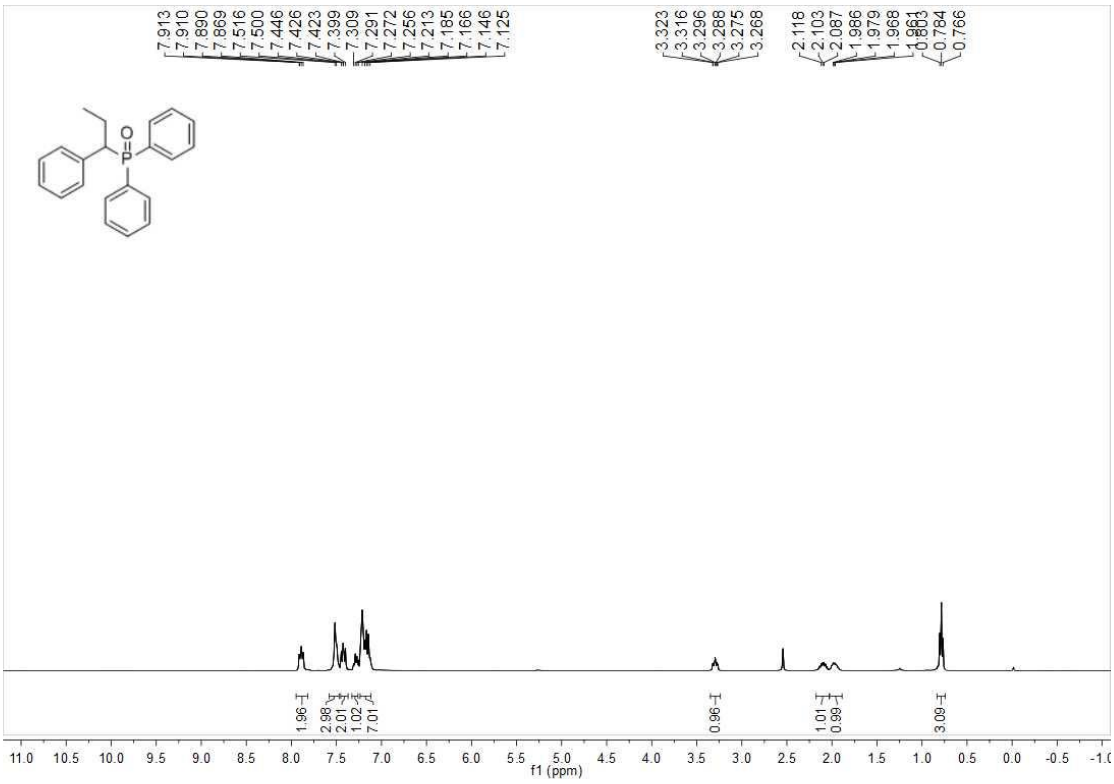

^{13}C NMR Spectrum of **235**

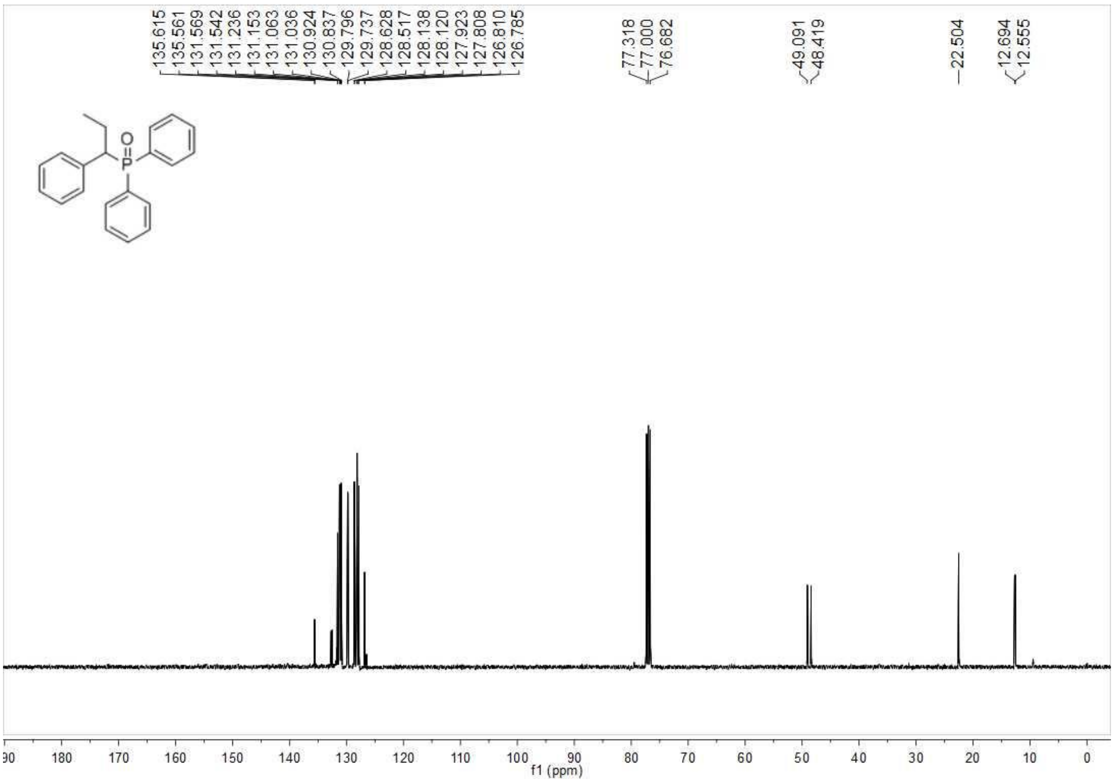

^{31}P NMR Spectrum of **235**

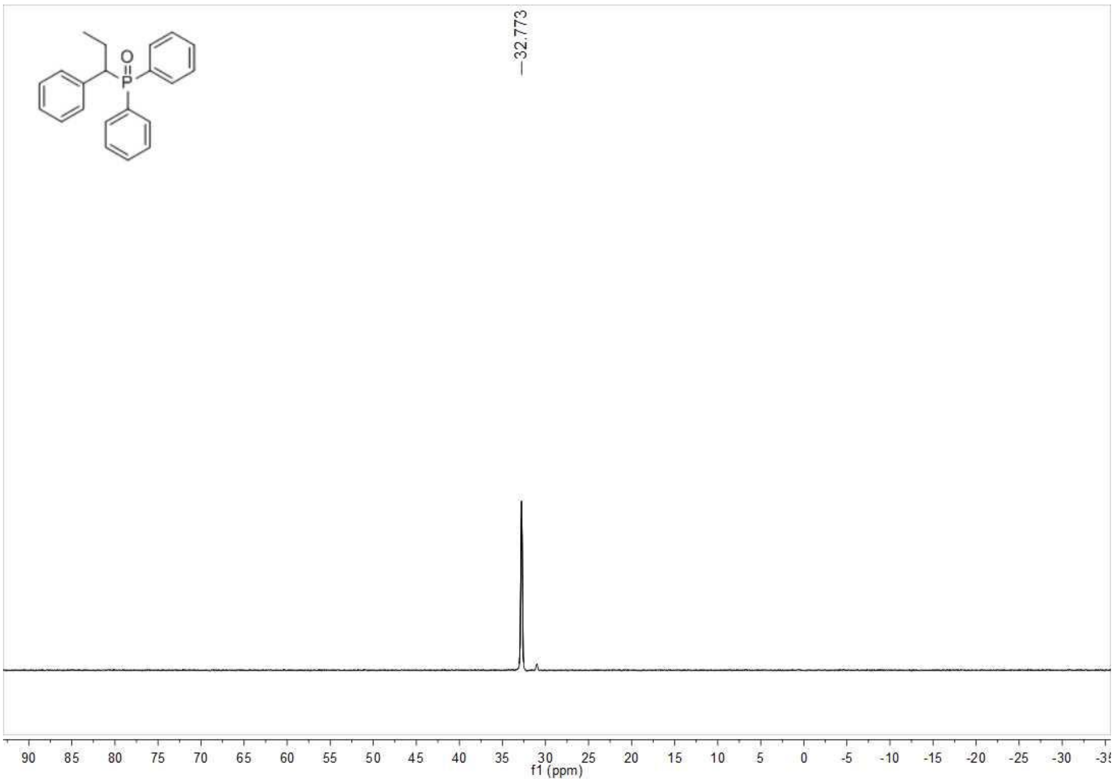

^{1}H NMR Spectrum of **236**

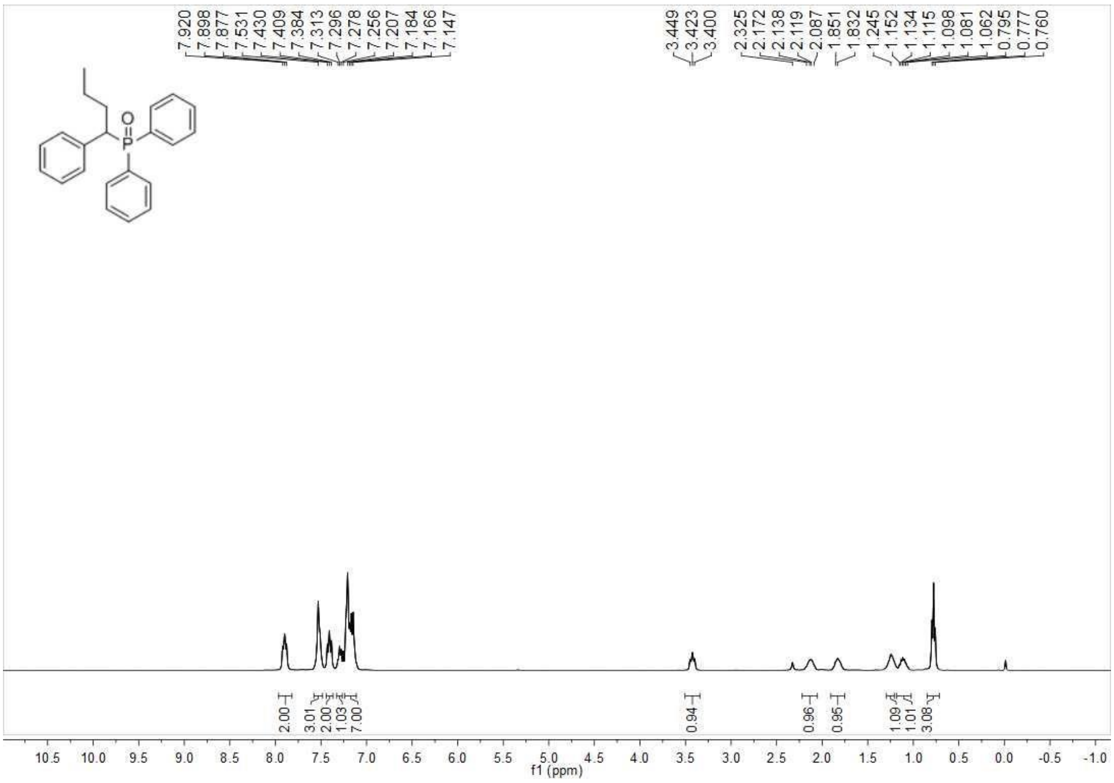

^{13}C NMR Spectrum of **236**

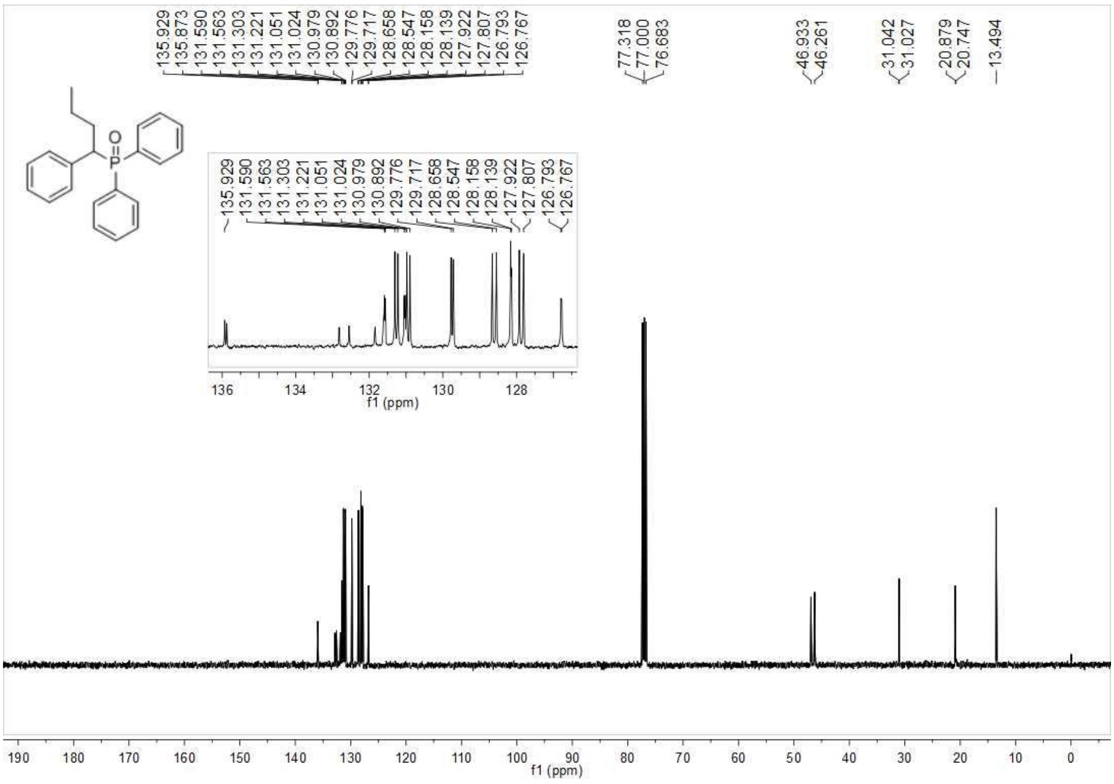

^{31}P NMR Spectrum of **236**

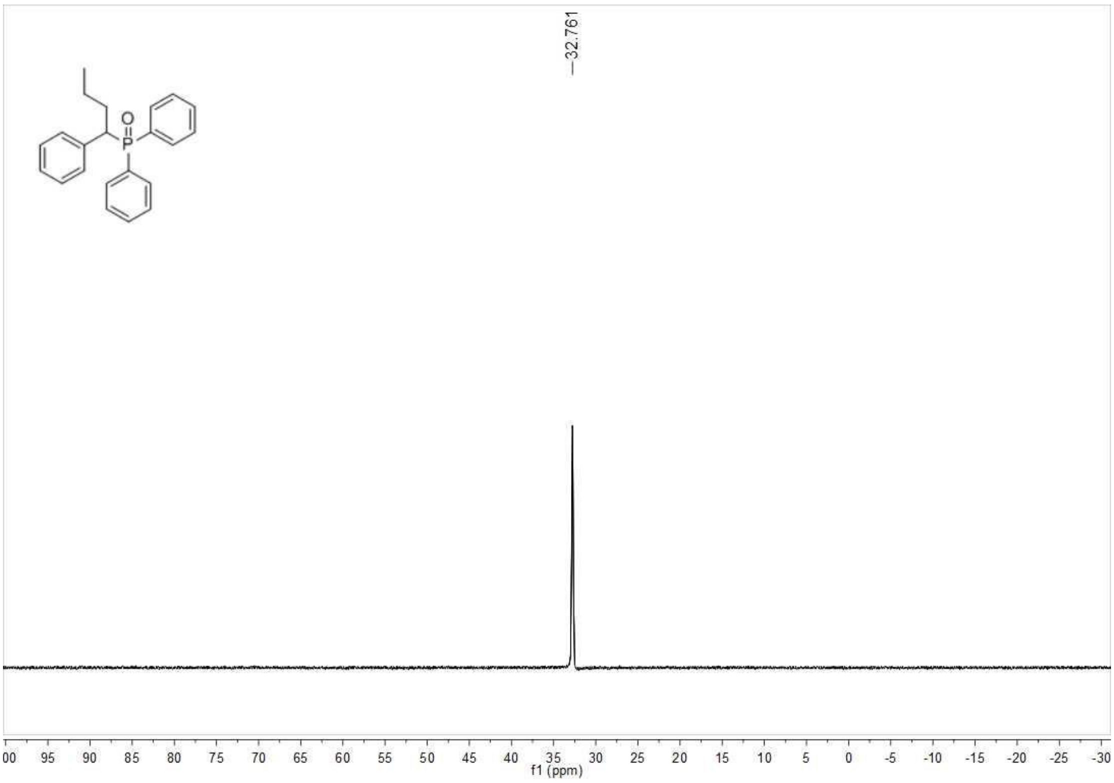

^{1}H NMR Spectrum of **237**

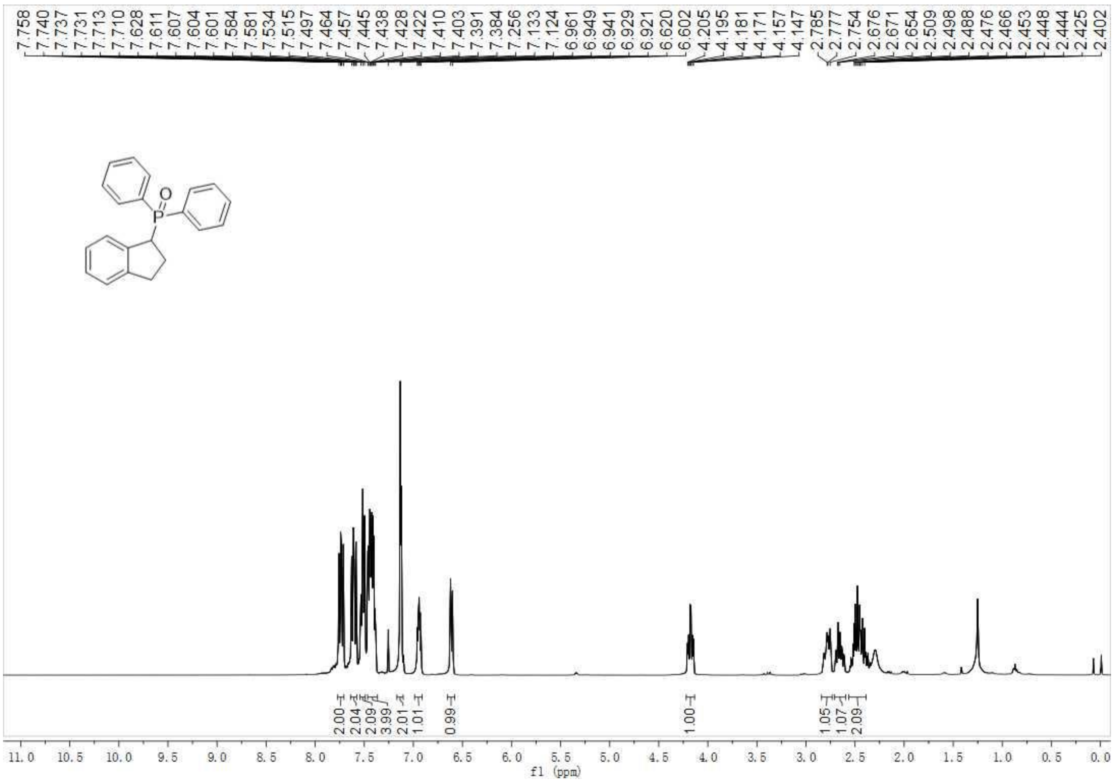

^{13}C NMR Spectrum of **237**

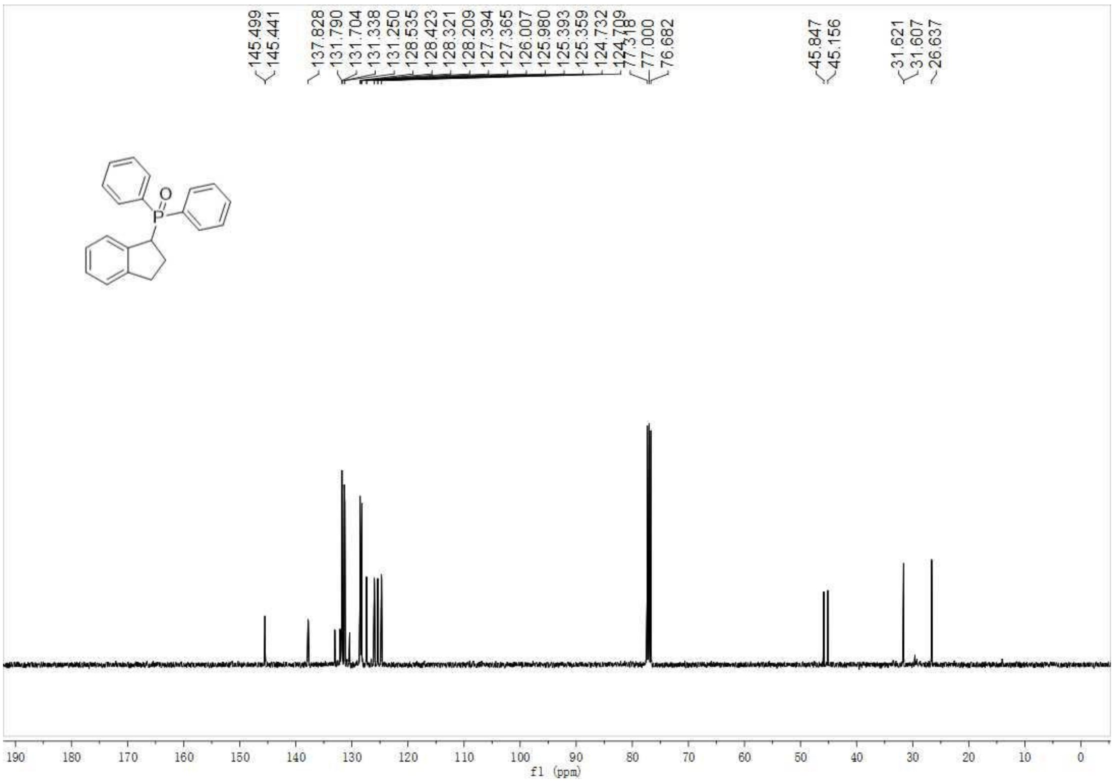

^{31}P NMR Spectrum of **237**

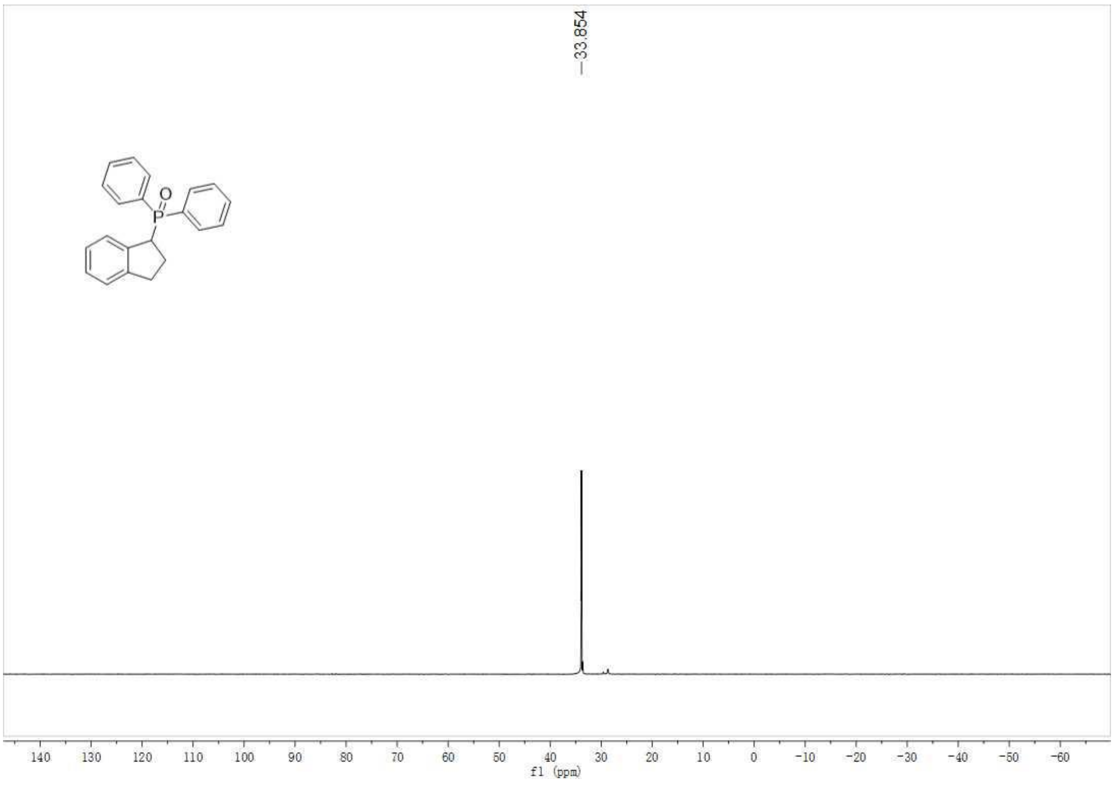

^{1}H NMR Spectrum of **238**

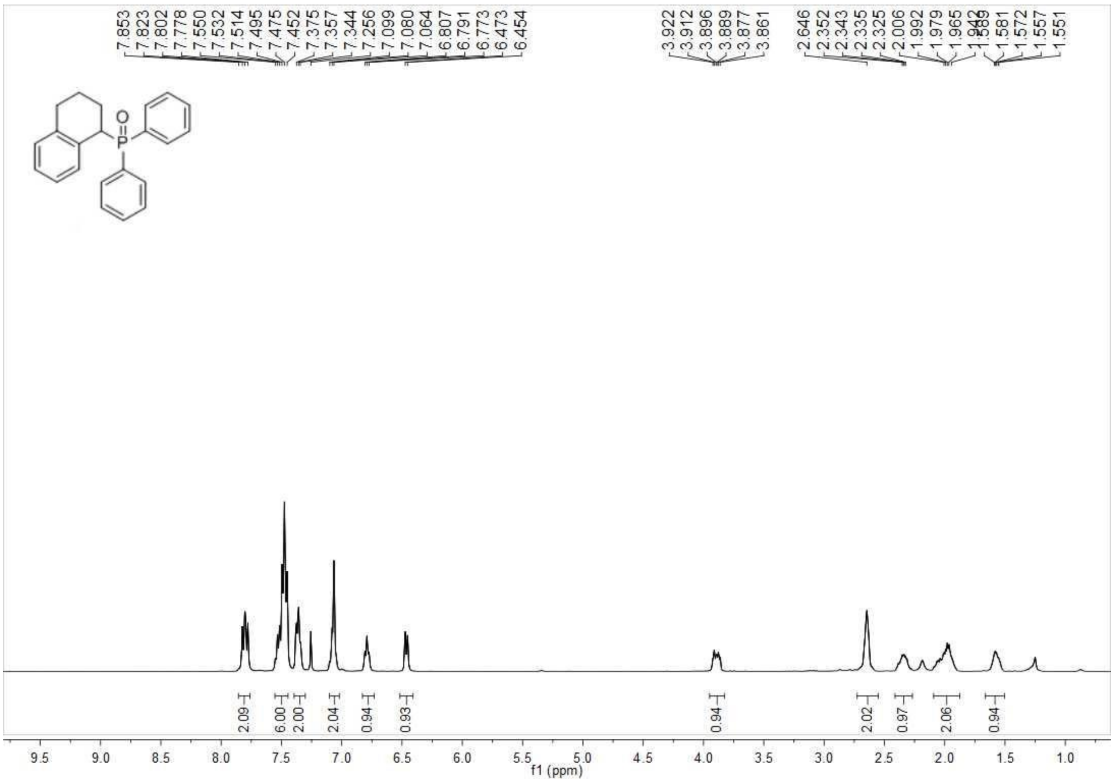

^{13}C NMR Spectrum of **238**

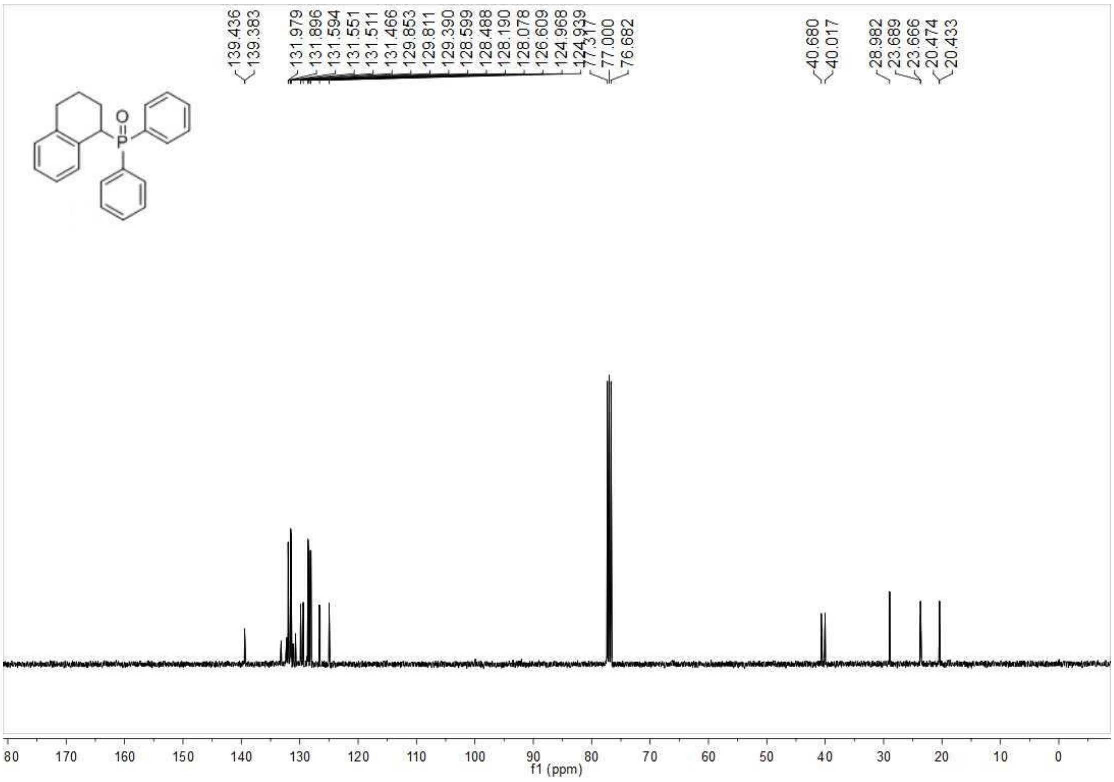

^{31}P NMR Spectrum of **238**

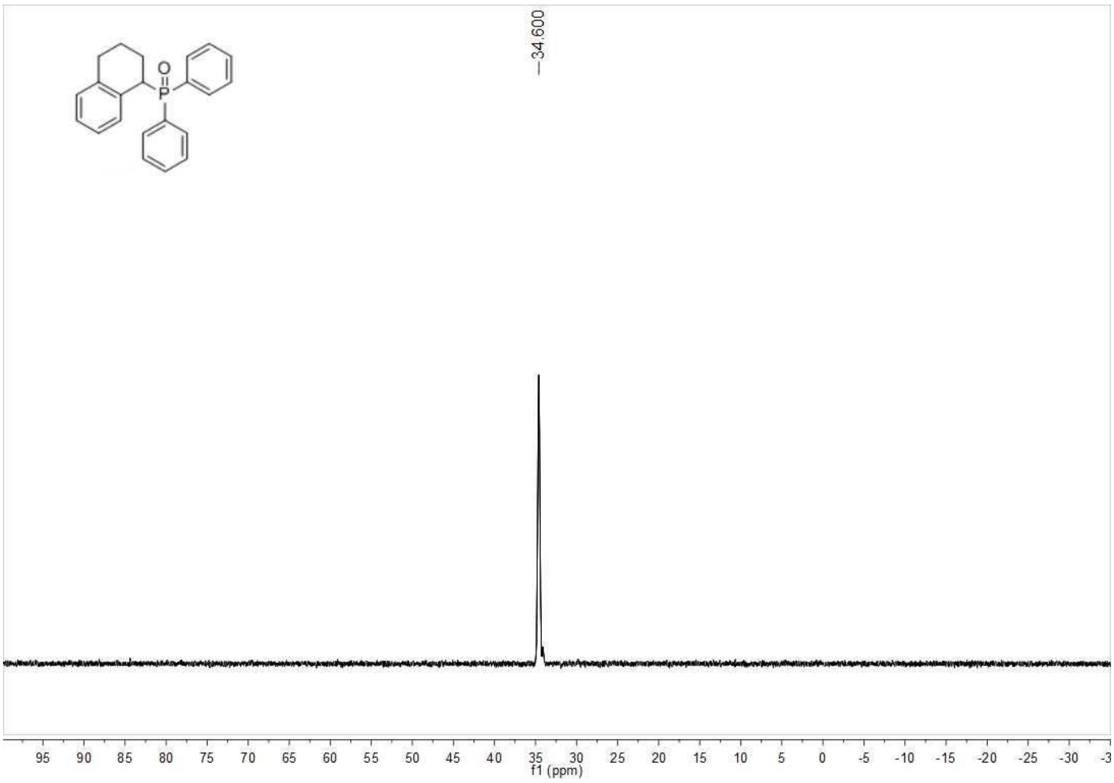

^{1}H NMR Spectrum of **239**

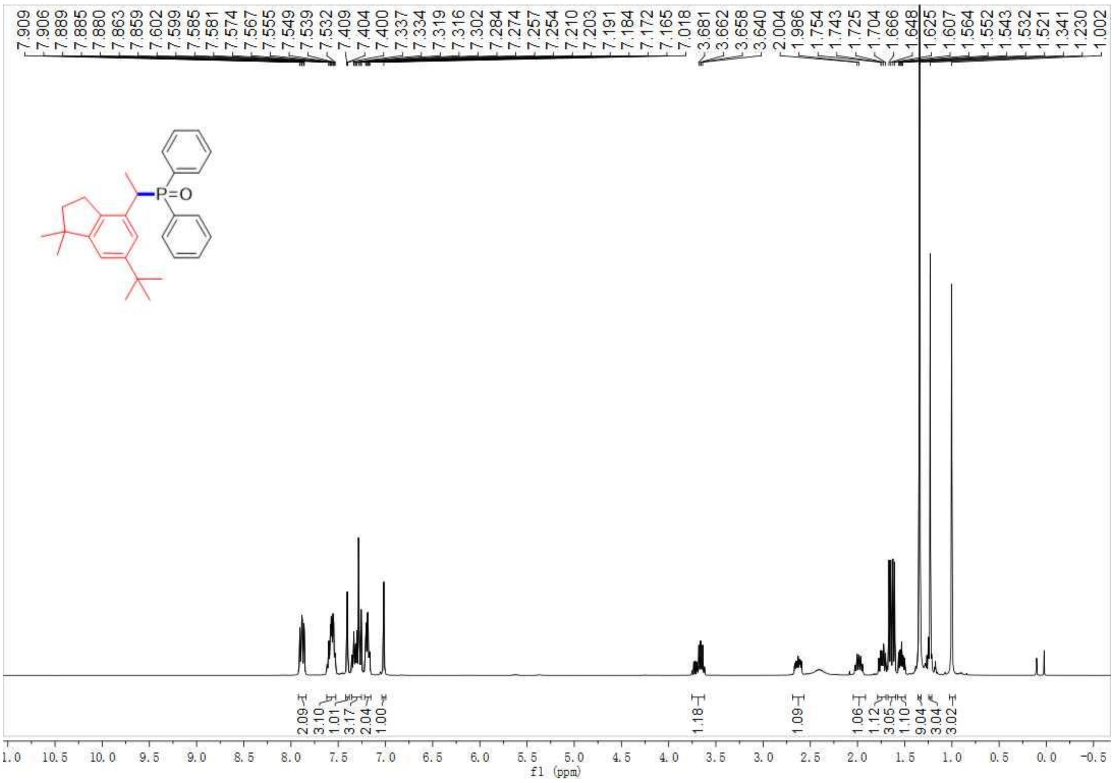

^{13}C NMR Spectrum of **239**

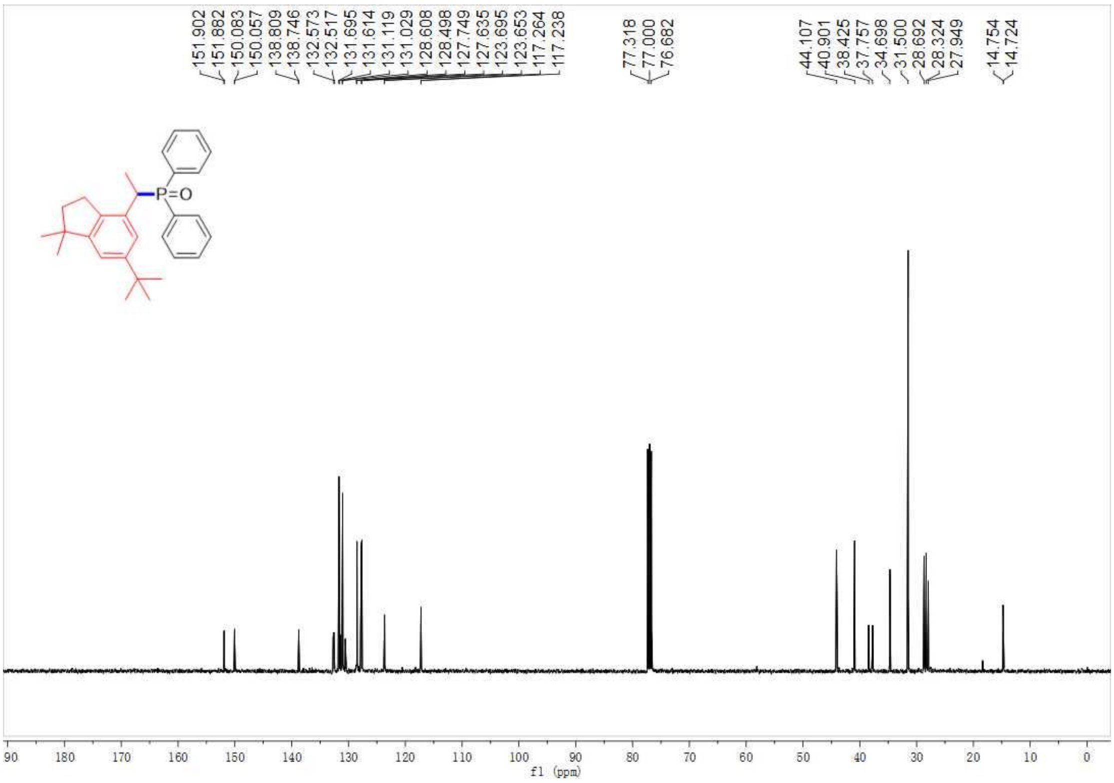

^{31}P NMR Spectrum of **239**

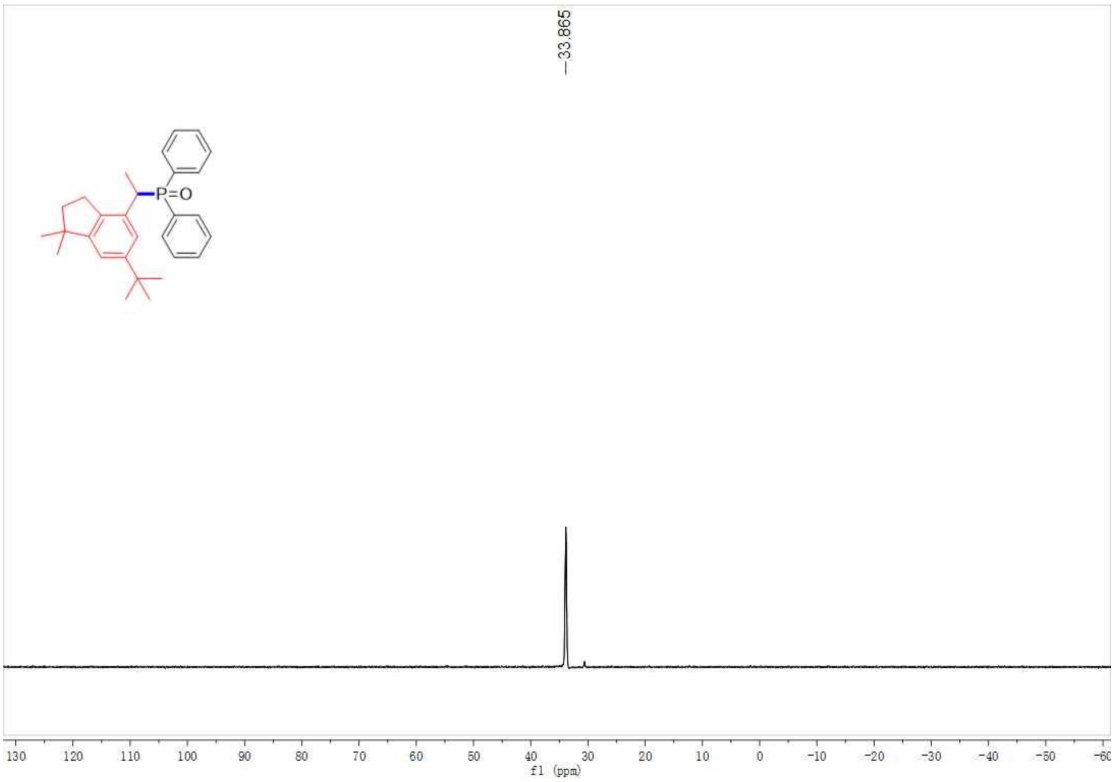

^{1}H NMR Spectrum of **240**

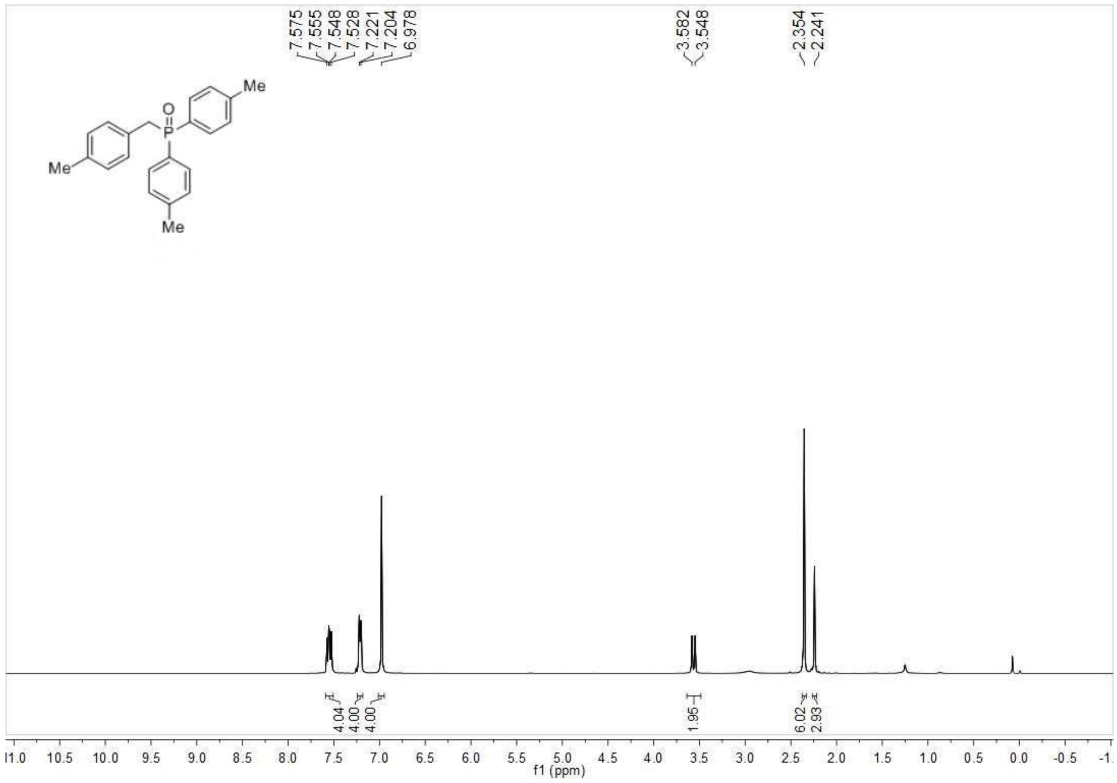

^{13}C NMR Spectrum of **240**

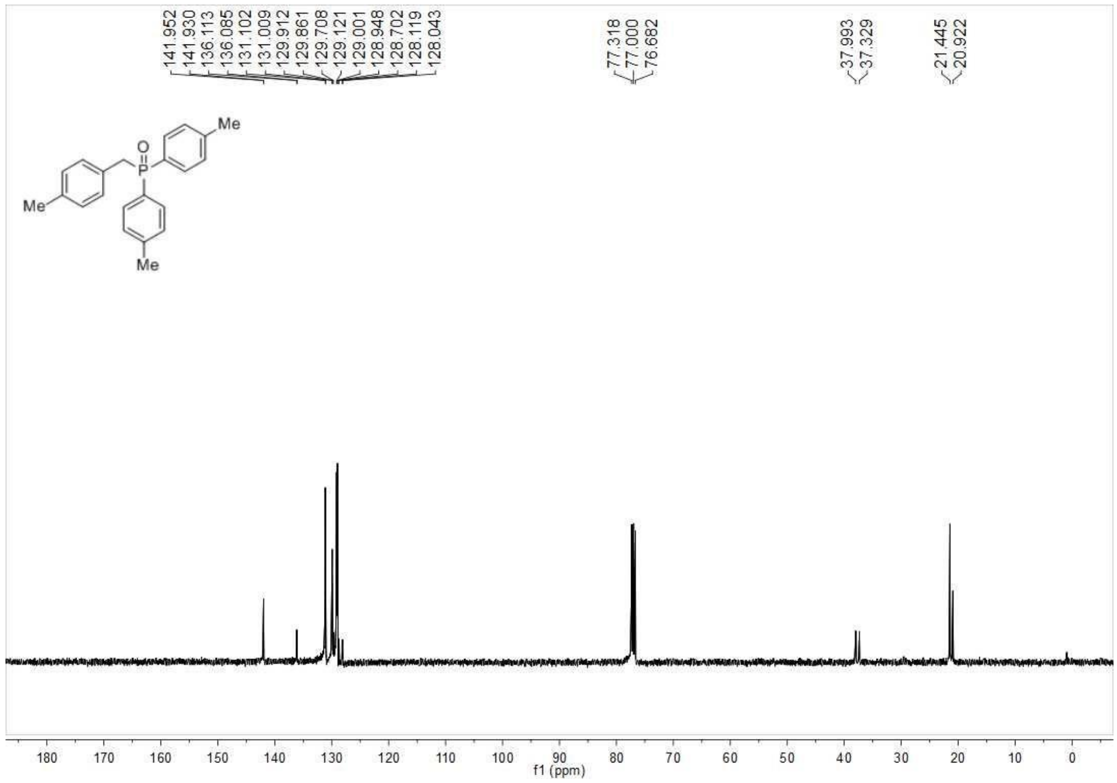

^{31}P NMR Spectrum of **240**

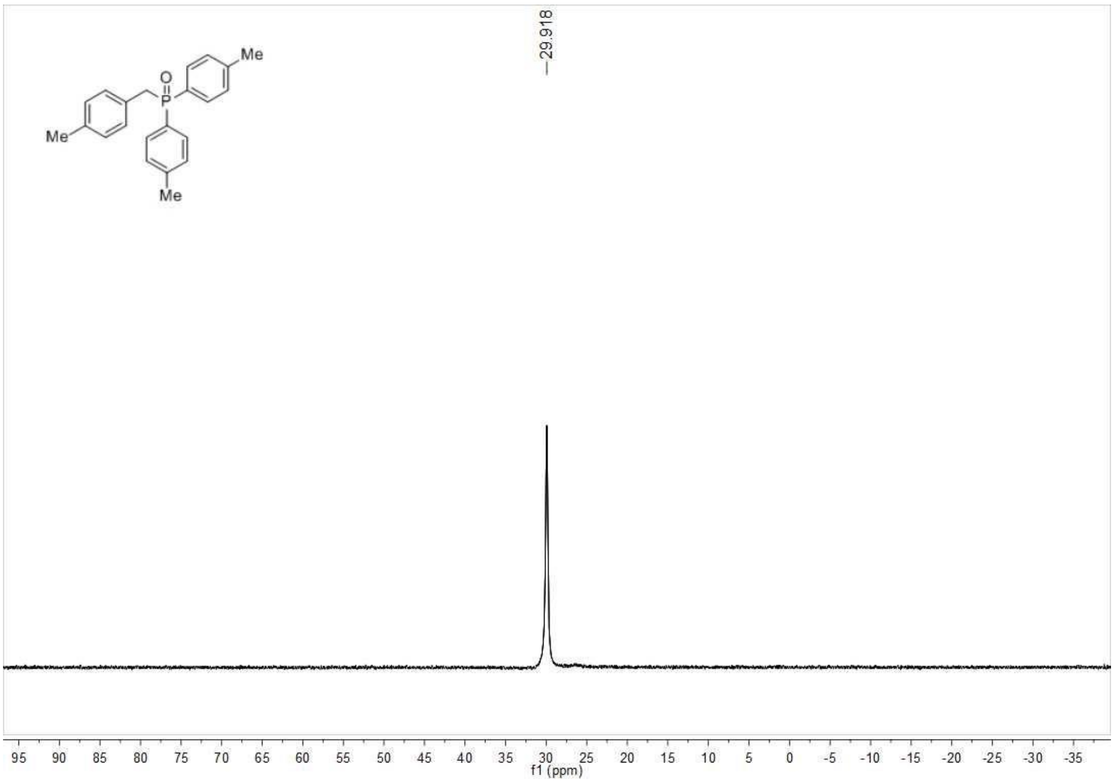

^{1}H NMR Spectrum of **241**

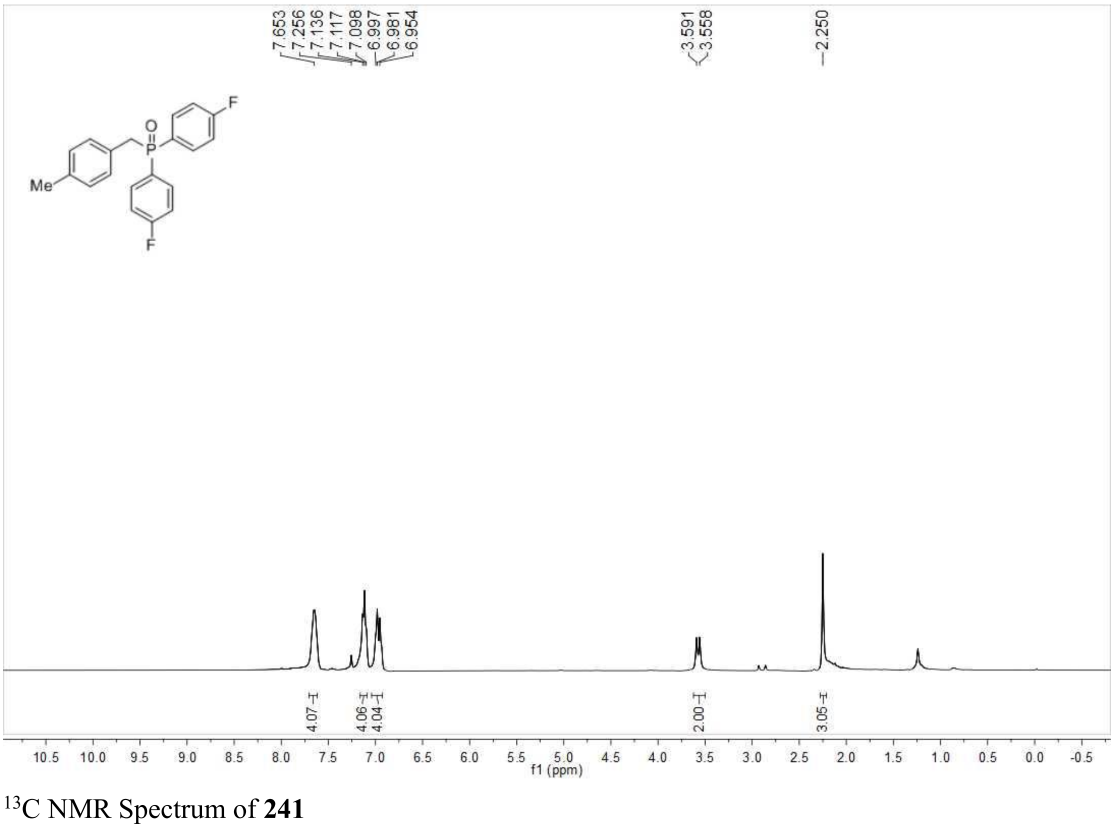

^{13}C NMR Spectrum of **241**

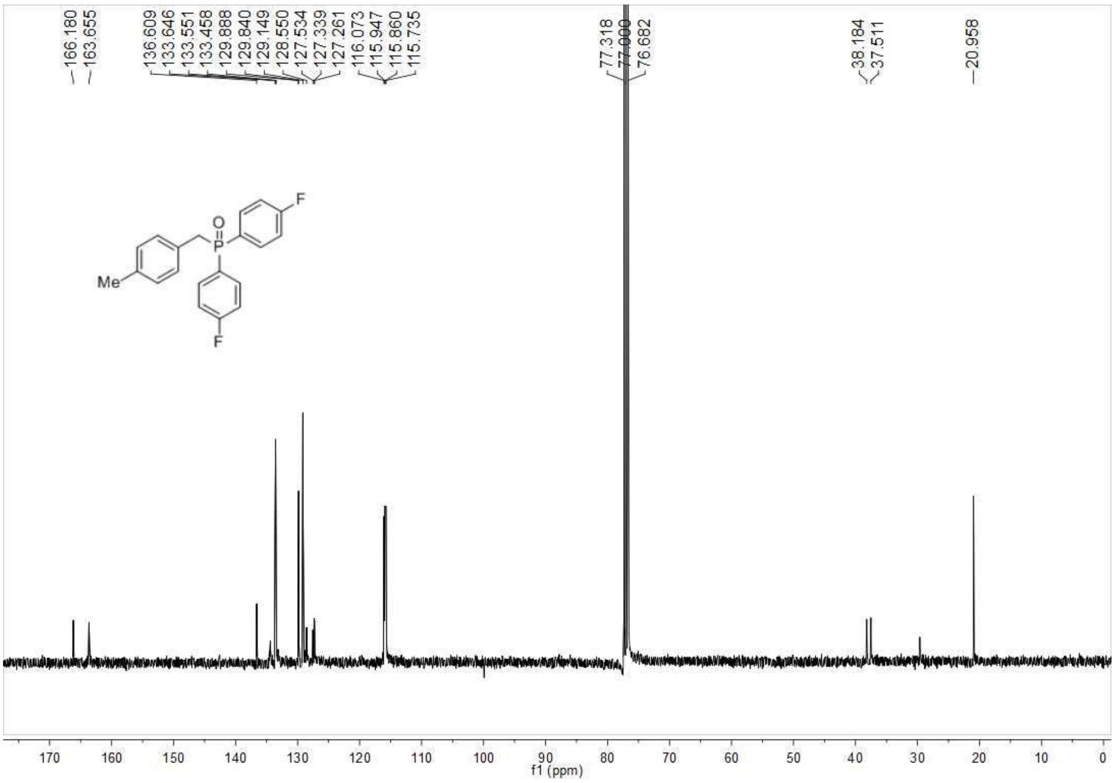

^{31}P NMR Spectrum of **241**

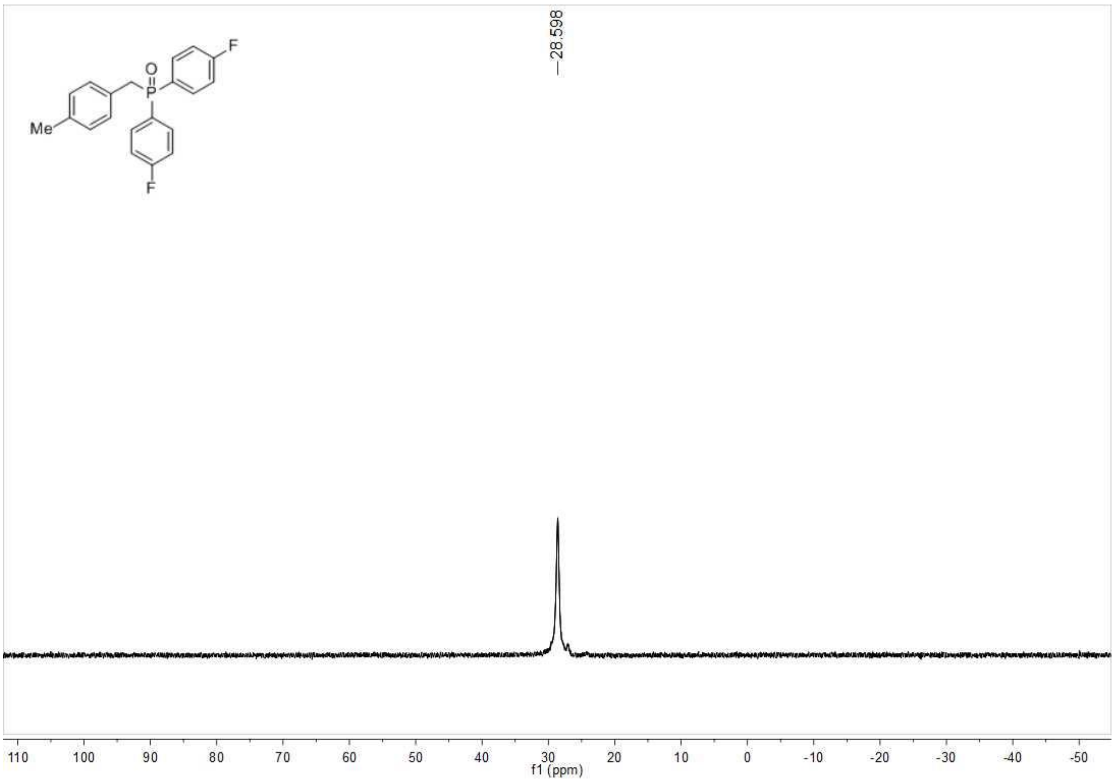

^{19}F NMR Spectrum of **242**

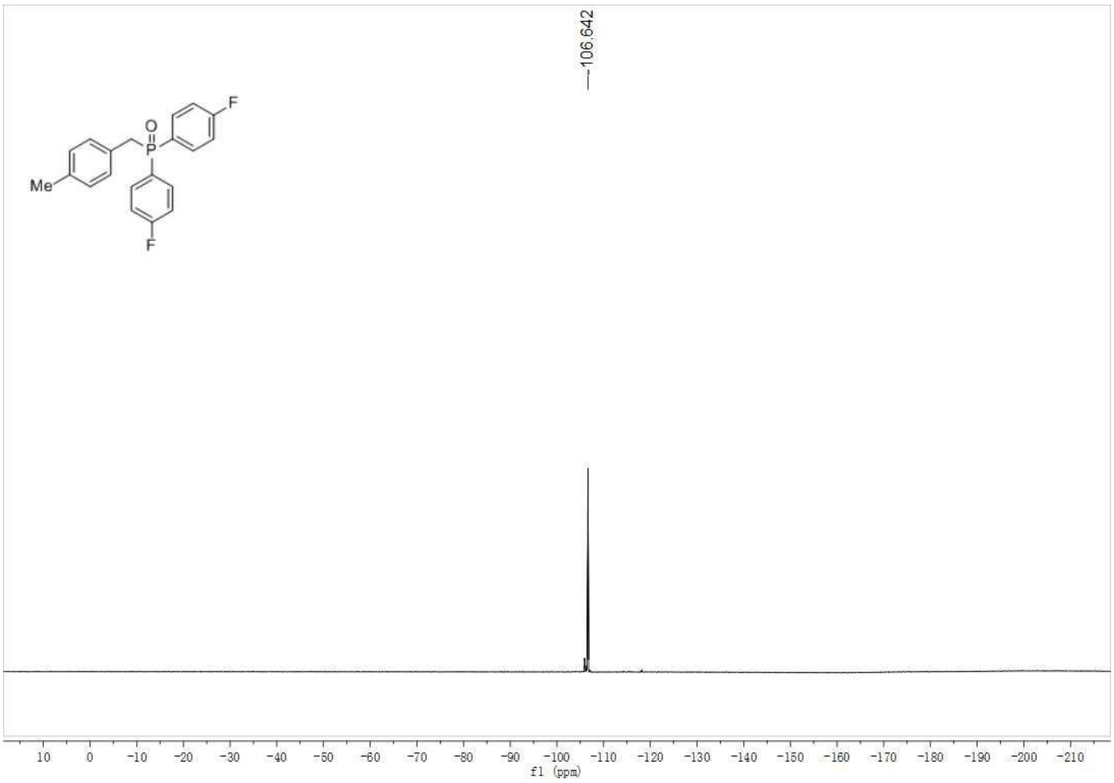

^{1}H NMR Spectrum of **242**

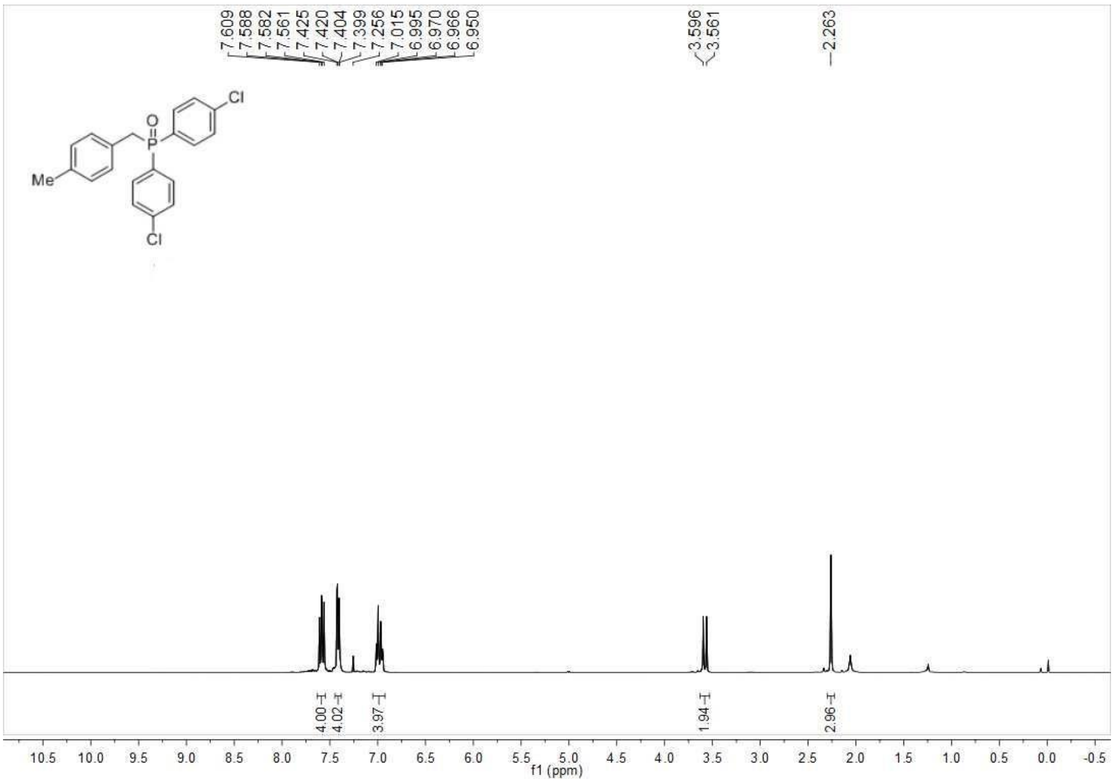

^{13}C NMR Spectrum of **242**

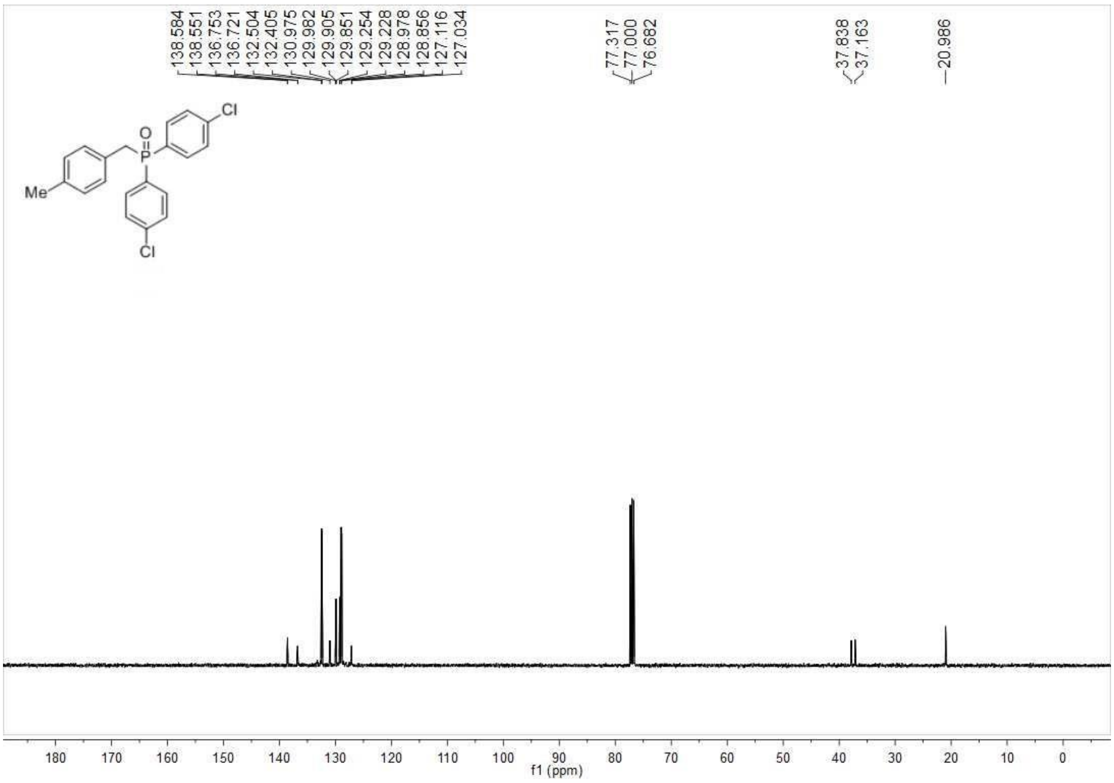

^{31}P NMR Spectrum of **242**

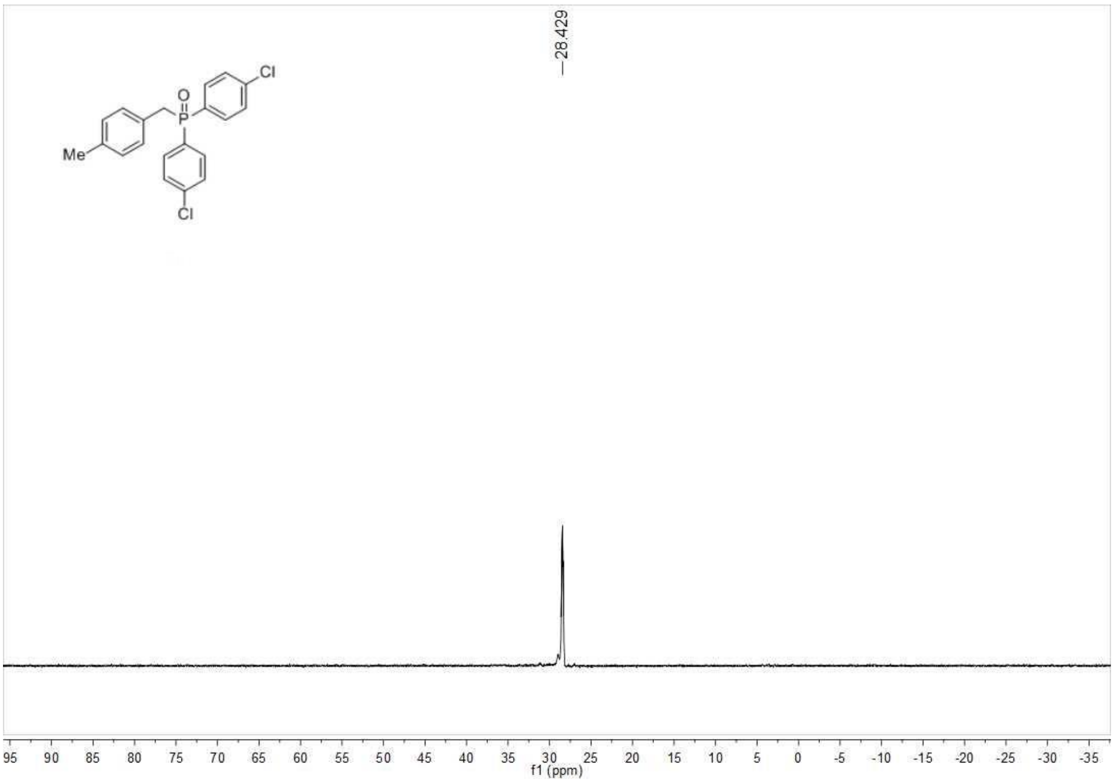

^{1}H NMR Spectrum of **243**

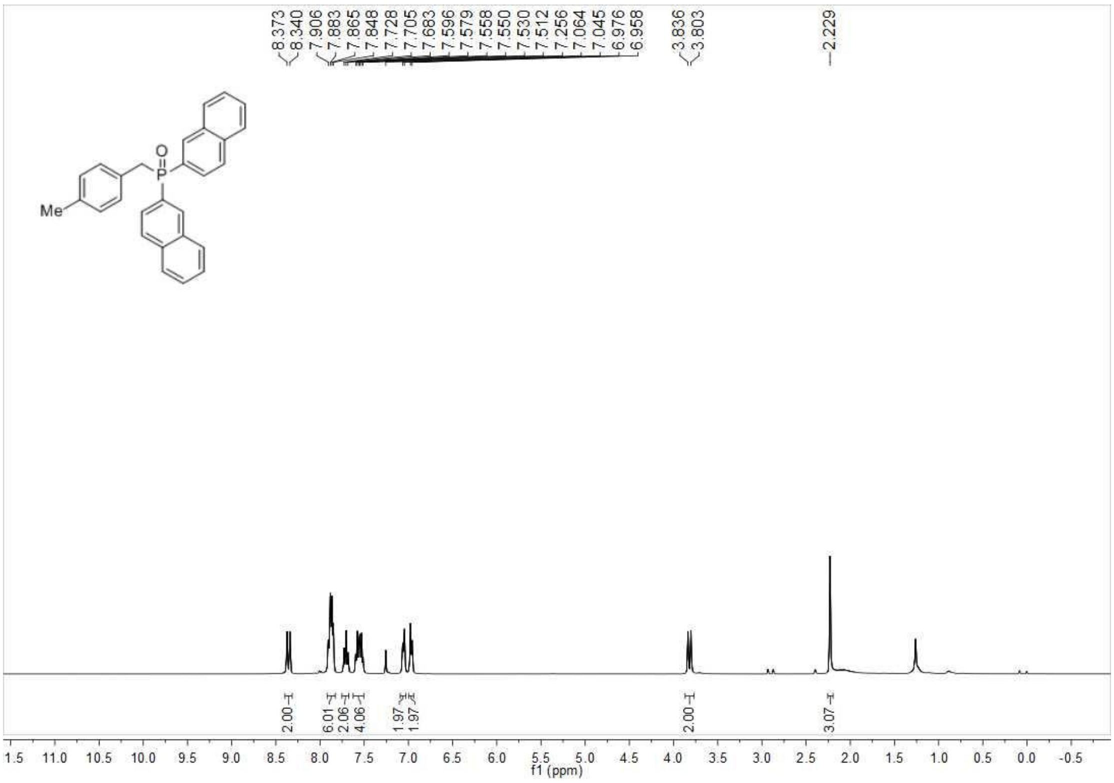

^{13}C NMR Spectrum of **243**

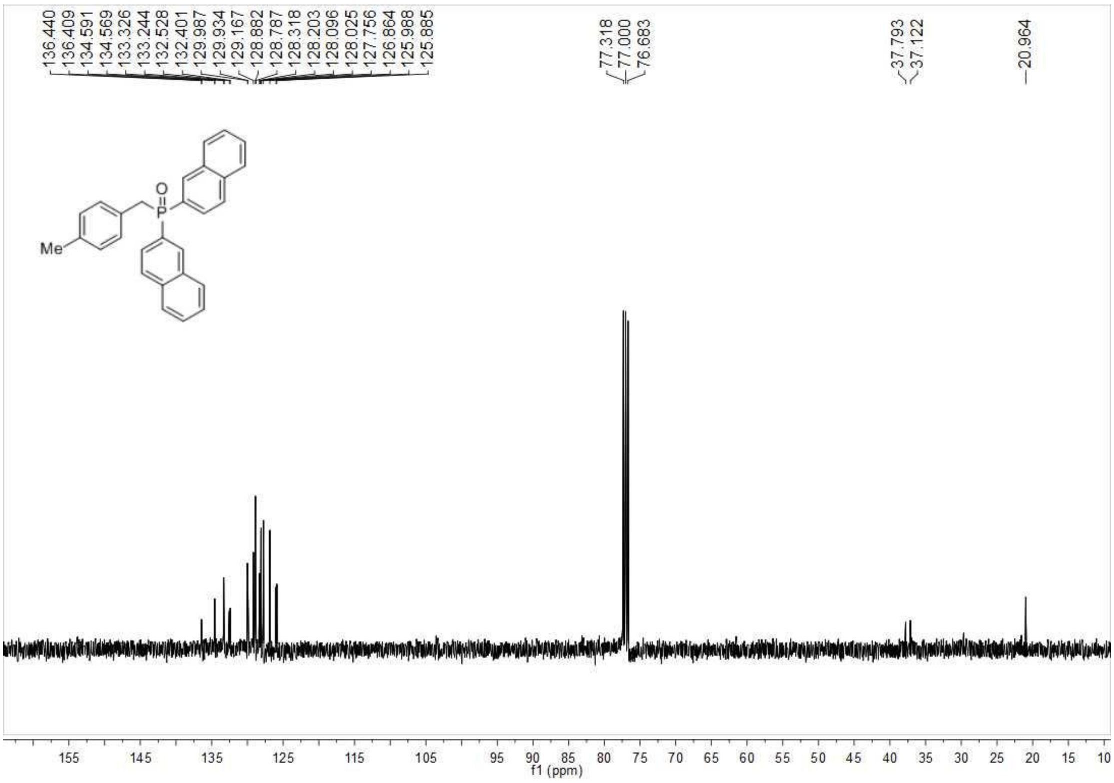

^{31}P NMR Spectrum of **243**

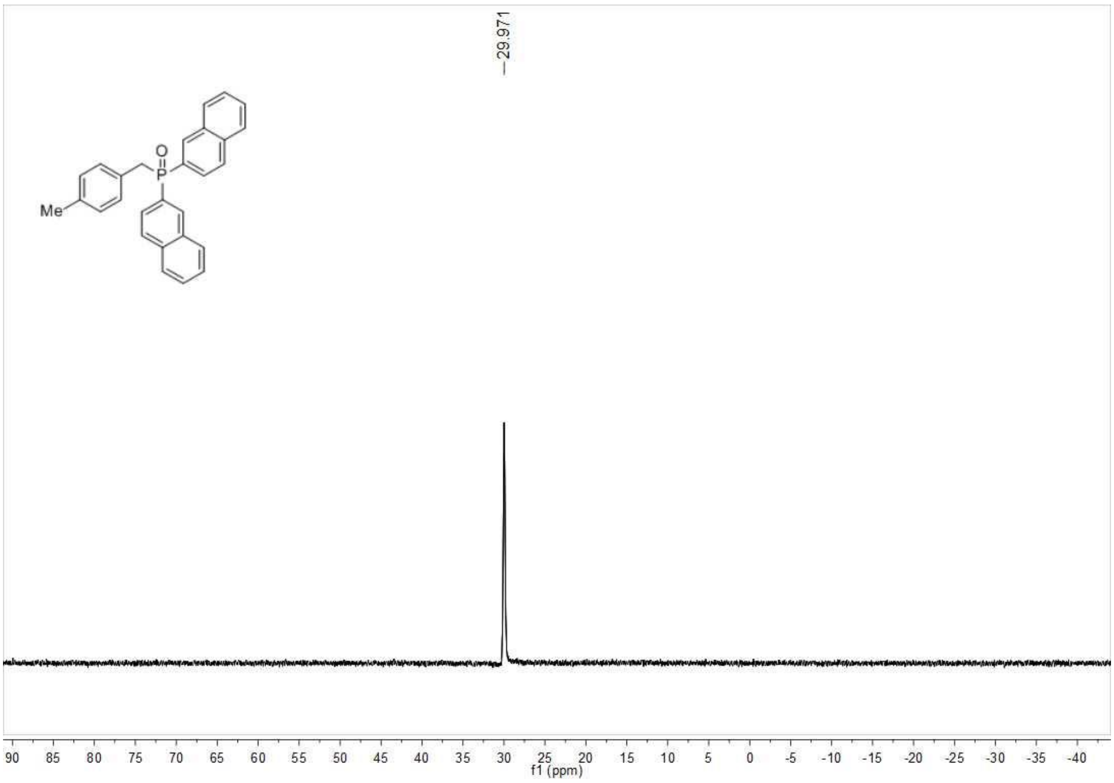

^{1}H NMR Spectrum of **244**

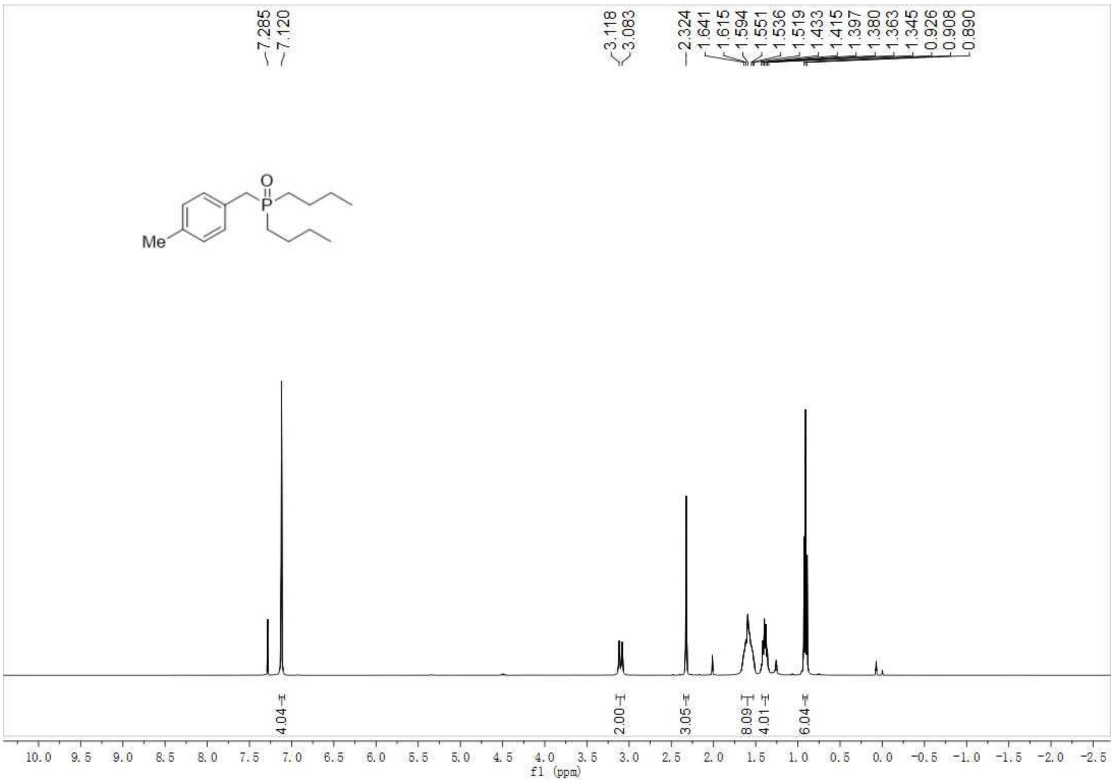

^{13}C NMR Spectrum of **244**

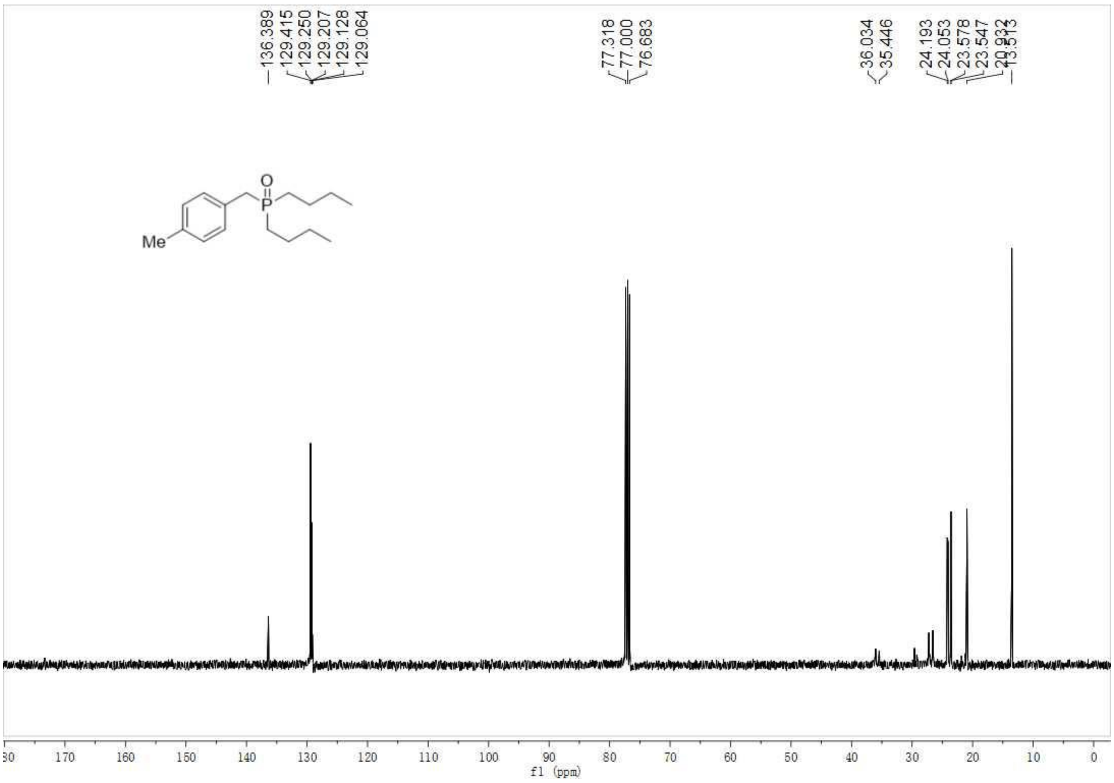

^{31}P NMR Spectrum of **244**

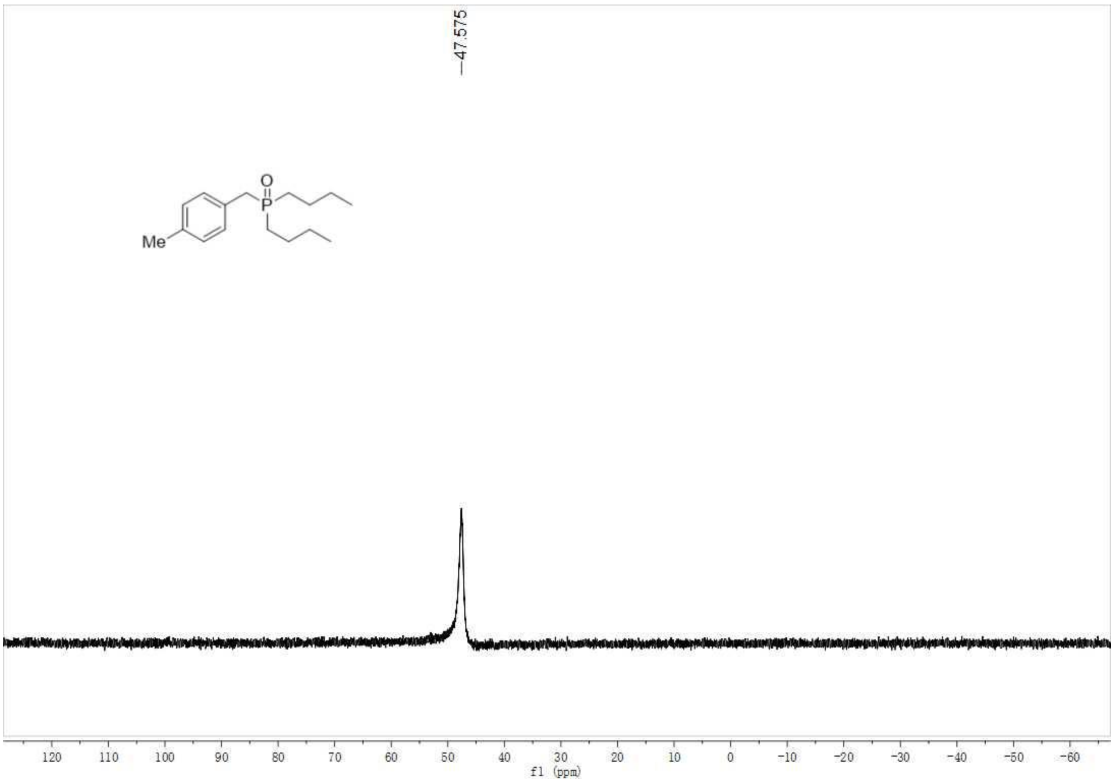

^{1}H NMR Spectrum of **245**

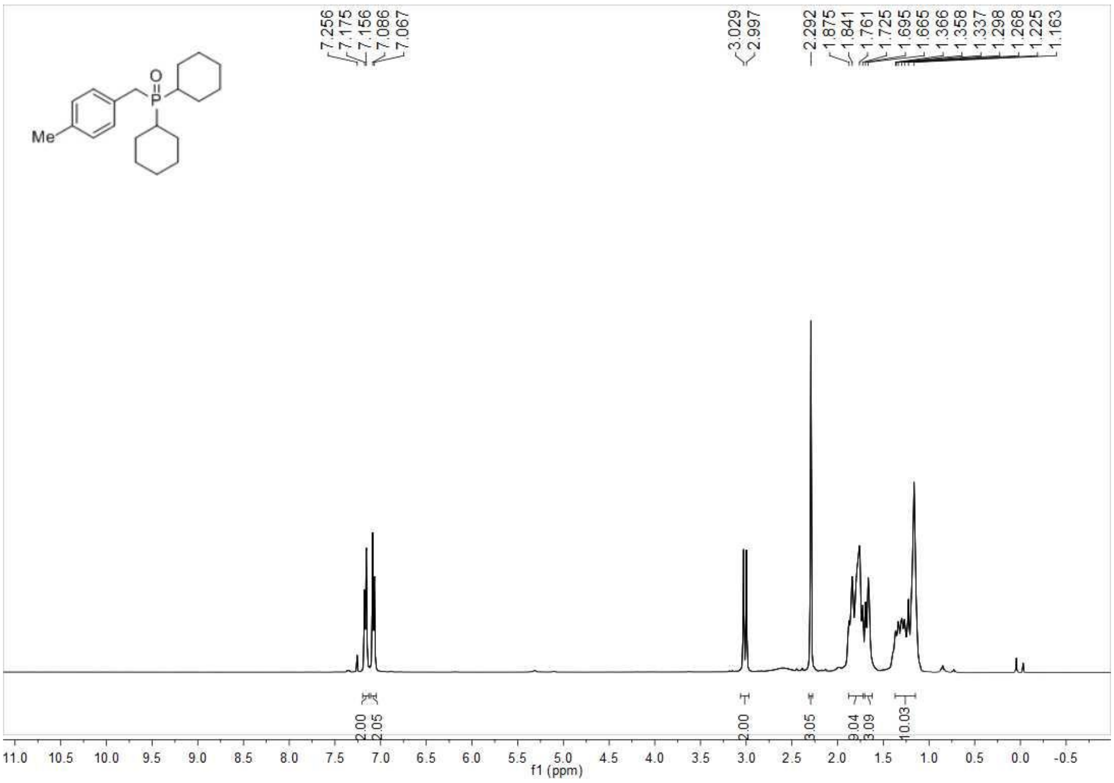

^{13}C NMR Spectrum of **245**

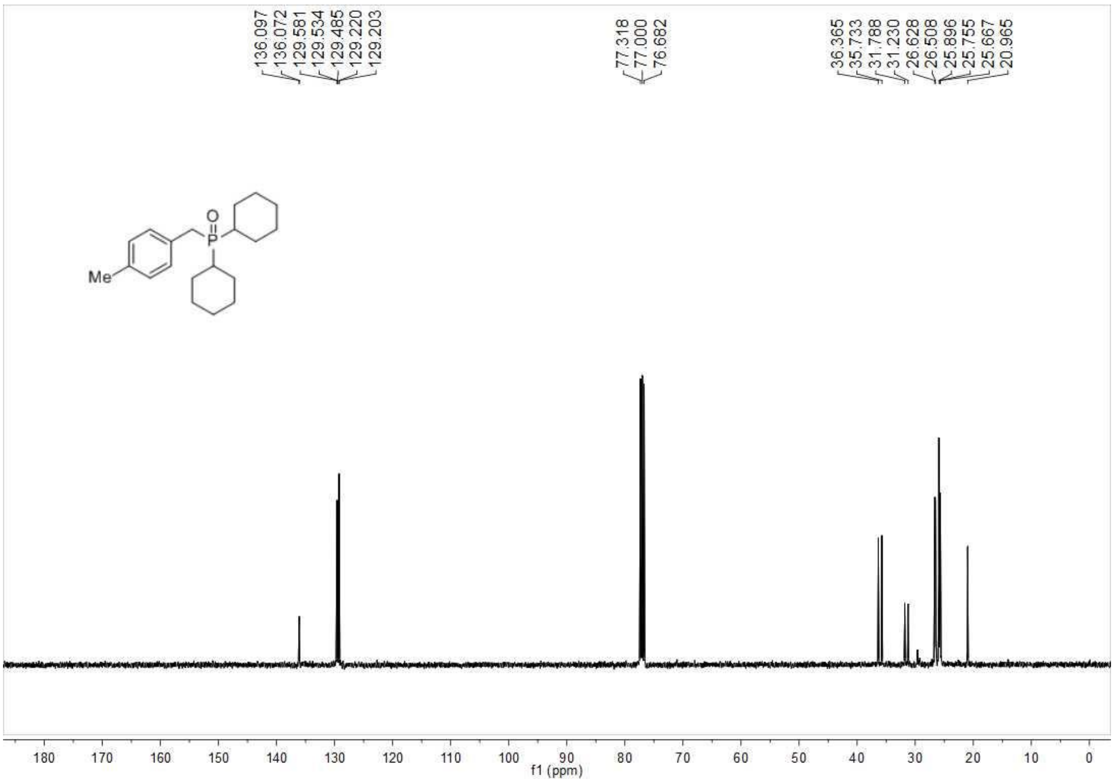

^{31}P NMR Spectrum of **245**

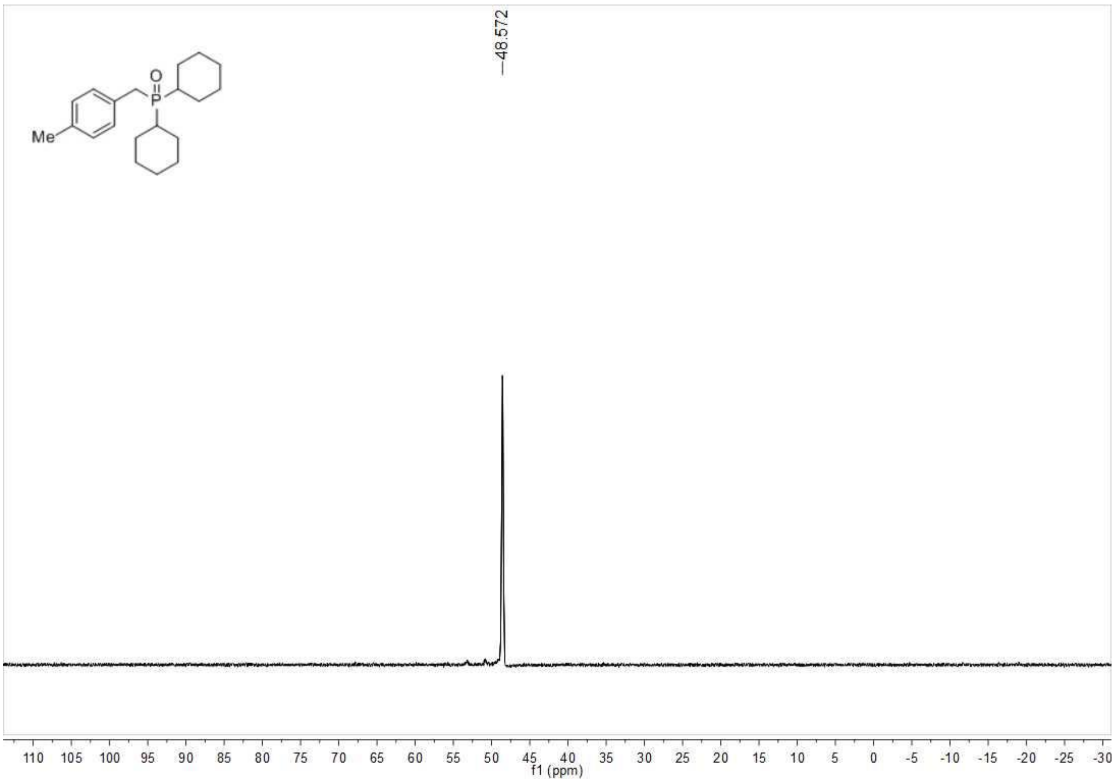

^{1}H NMR Spectrum of (*S*)-**247**

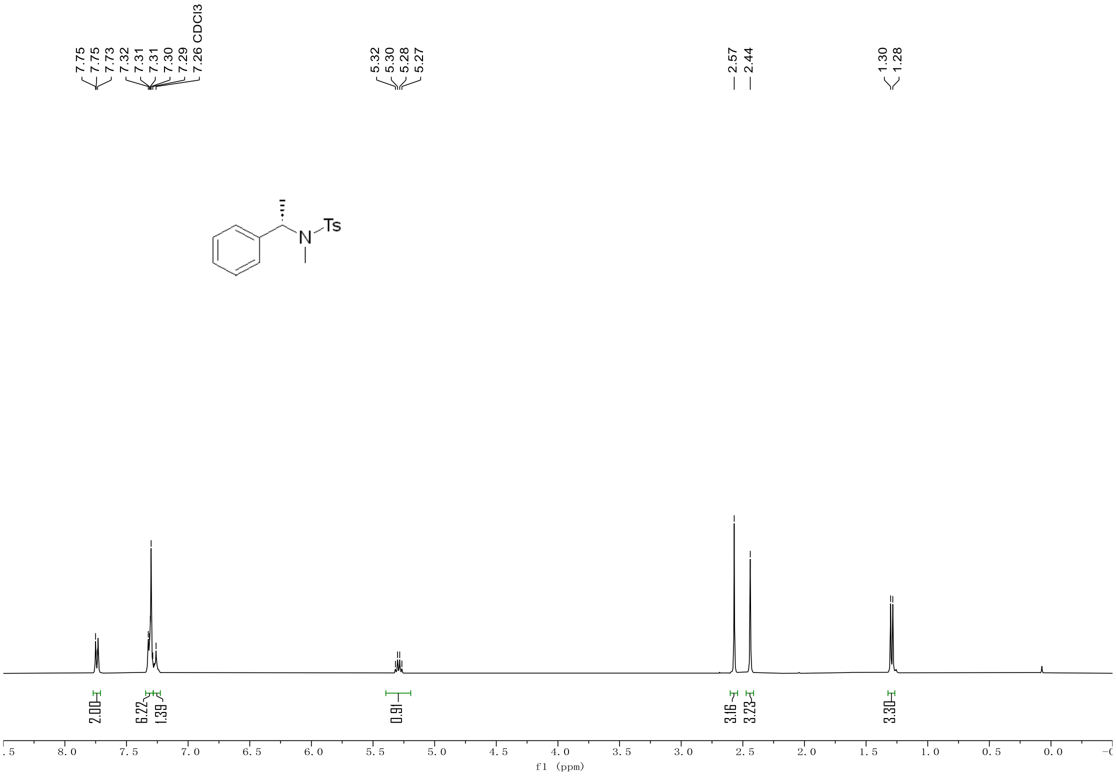

^{13}C NMR Spectrum of (*S*)-**247**

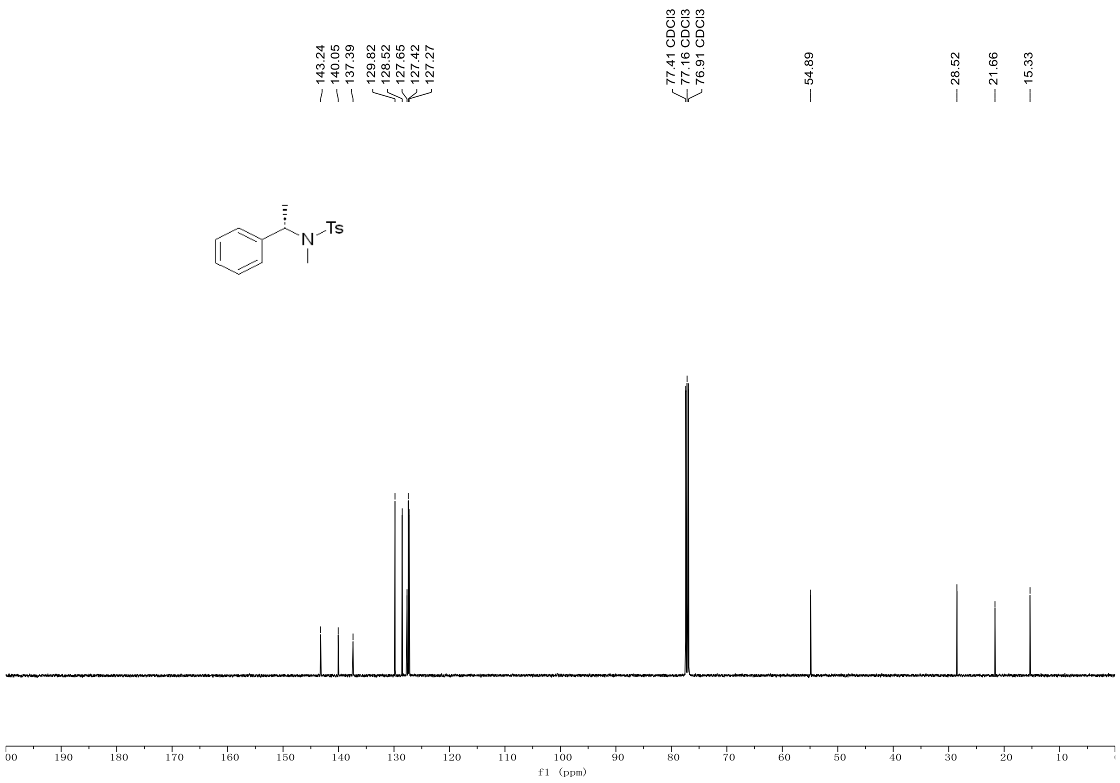

^{1}H NMR Spectrum of (*S*)-**248**

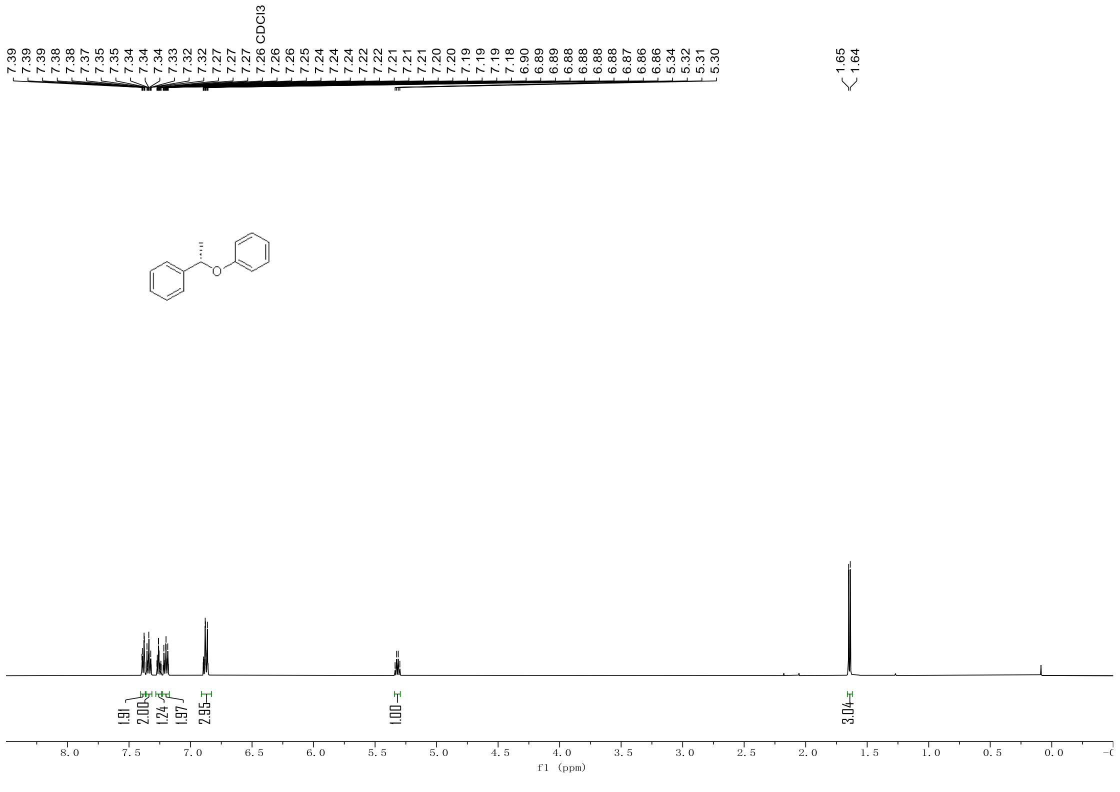

^{13}C NMR Spectrum of (*S*)-**248**

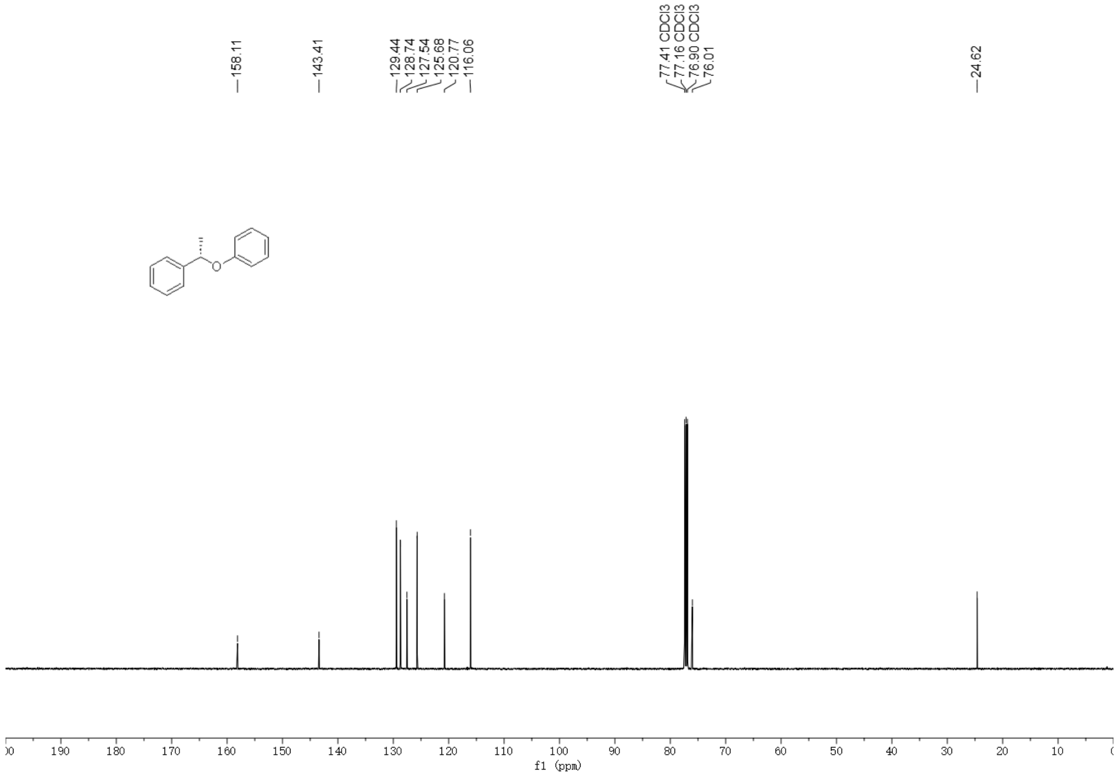

^{1}H NMR Spectrum of (*S*)-**249**

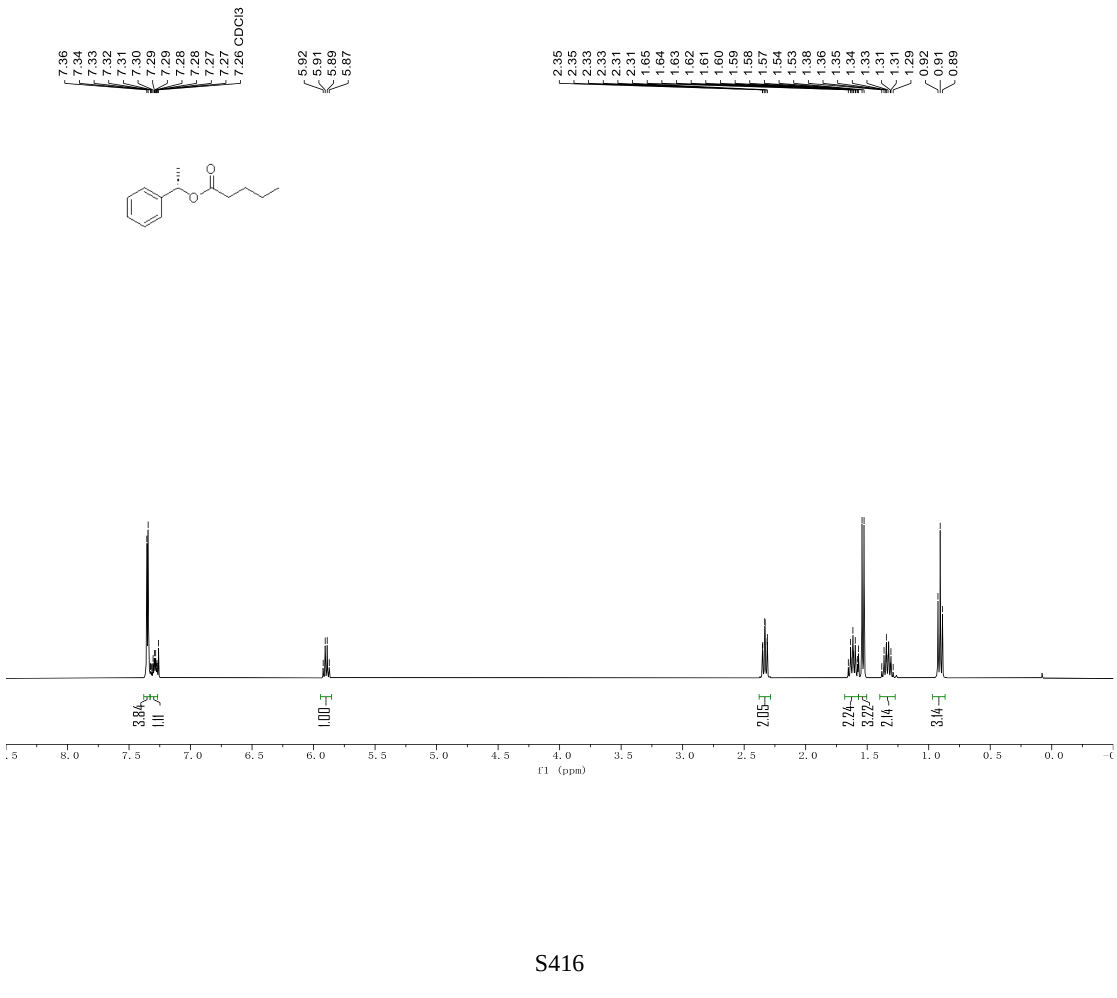

^{13}C NMR Spectrum of (*S*)-**249**

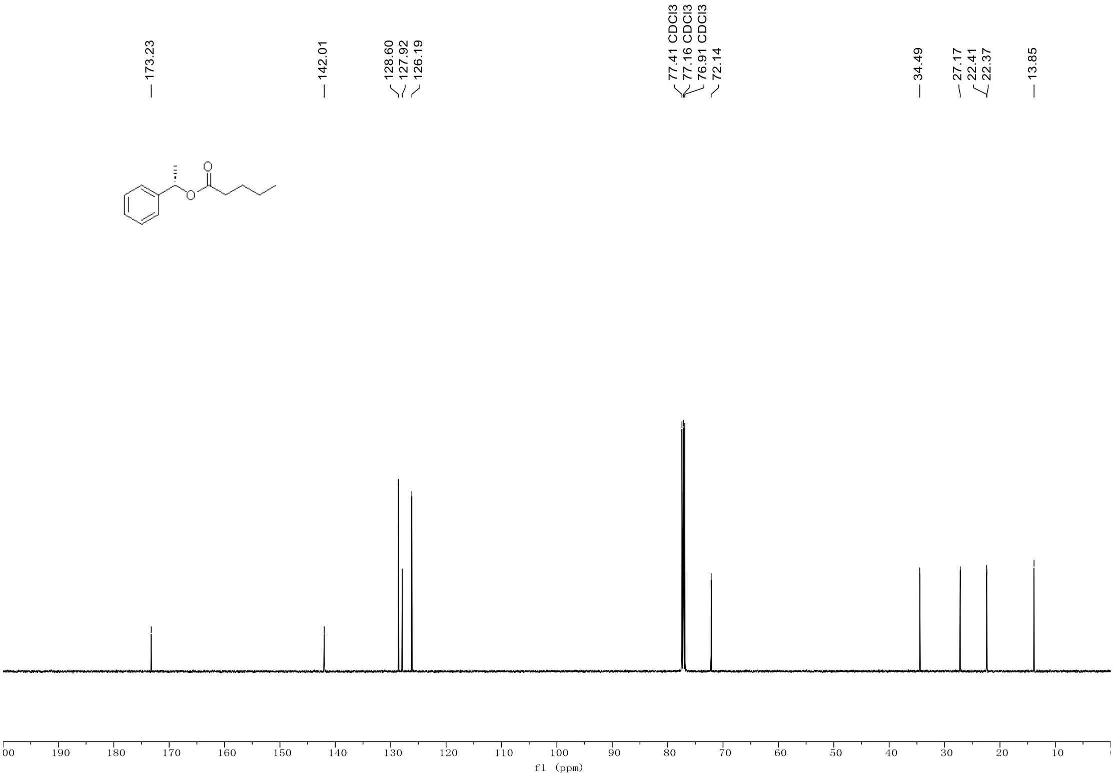

^{1}H NMR Spectrum of **250**

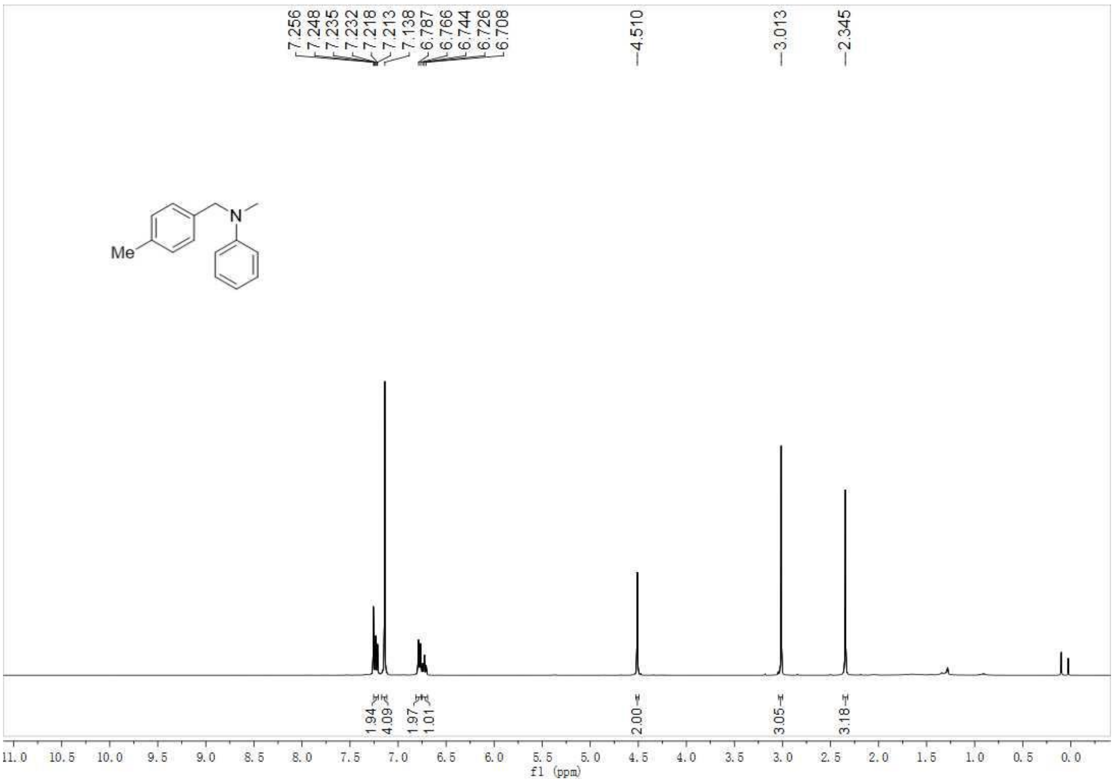

^{13}C NMR Spectrum of **250**

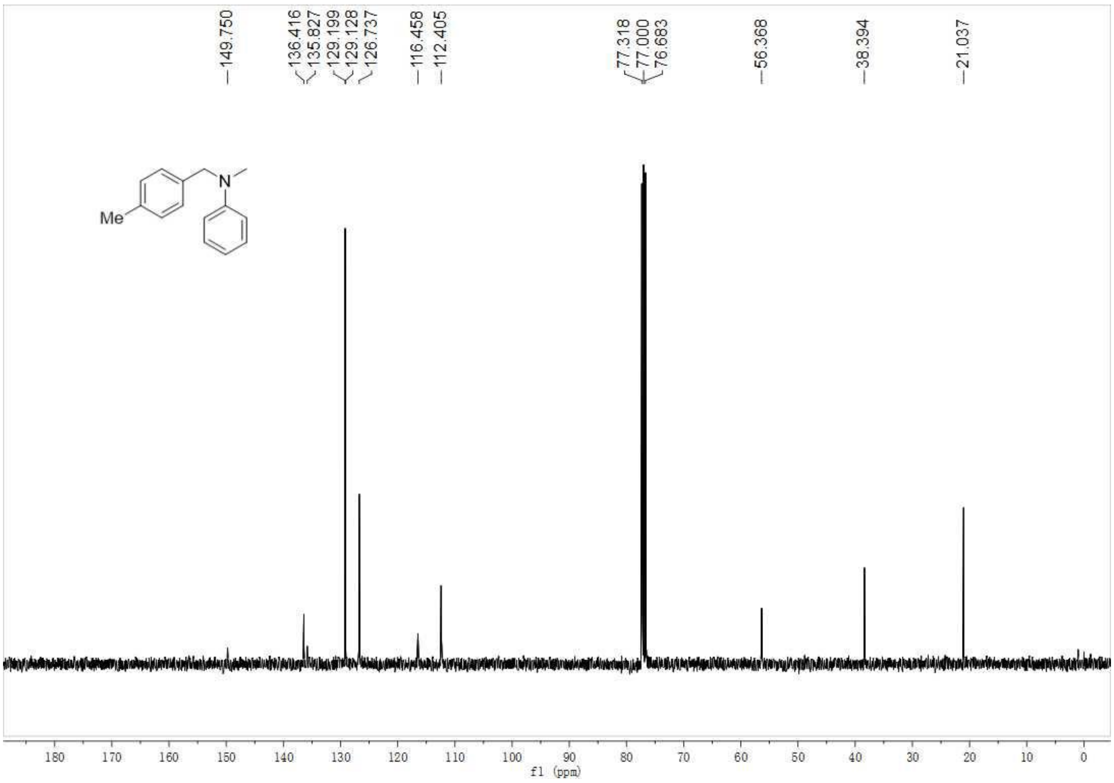

^{1}H NMR Spectrum of **251**

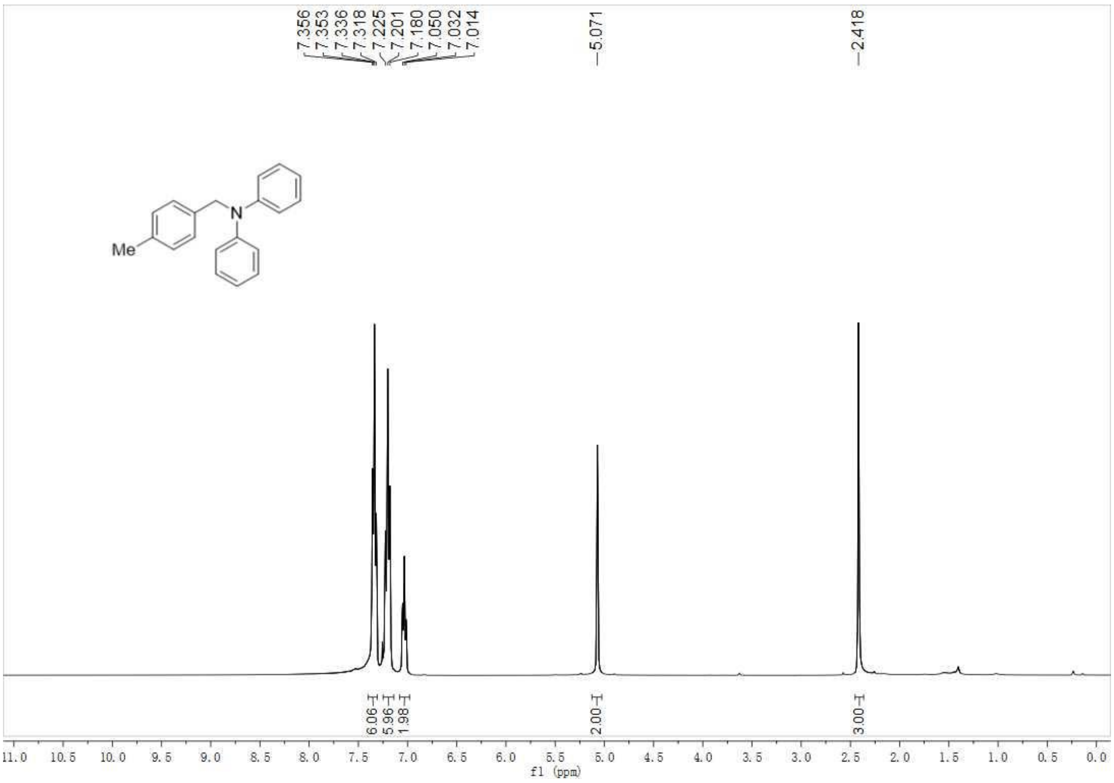

^{13}C NMR Spectrum of **251**

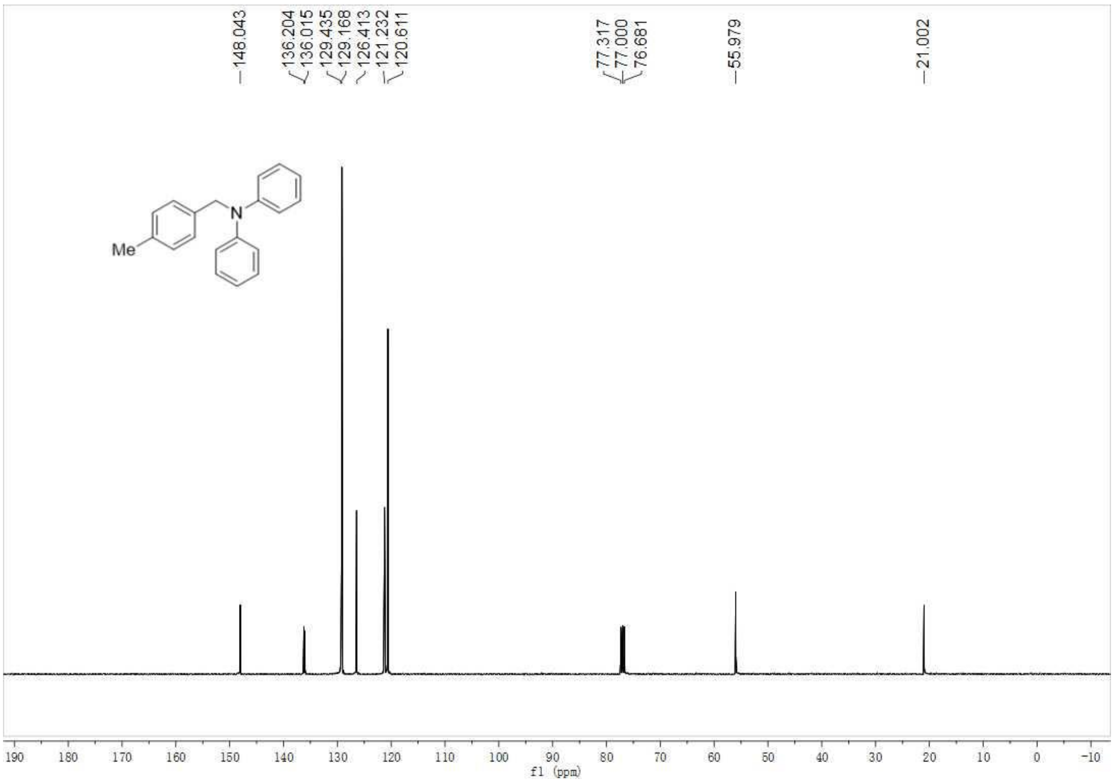

^{1}H NMR Spectrum of **252**

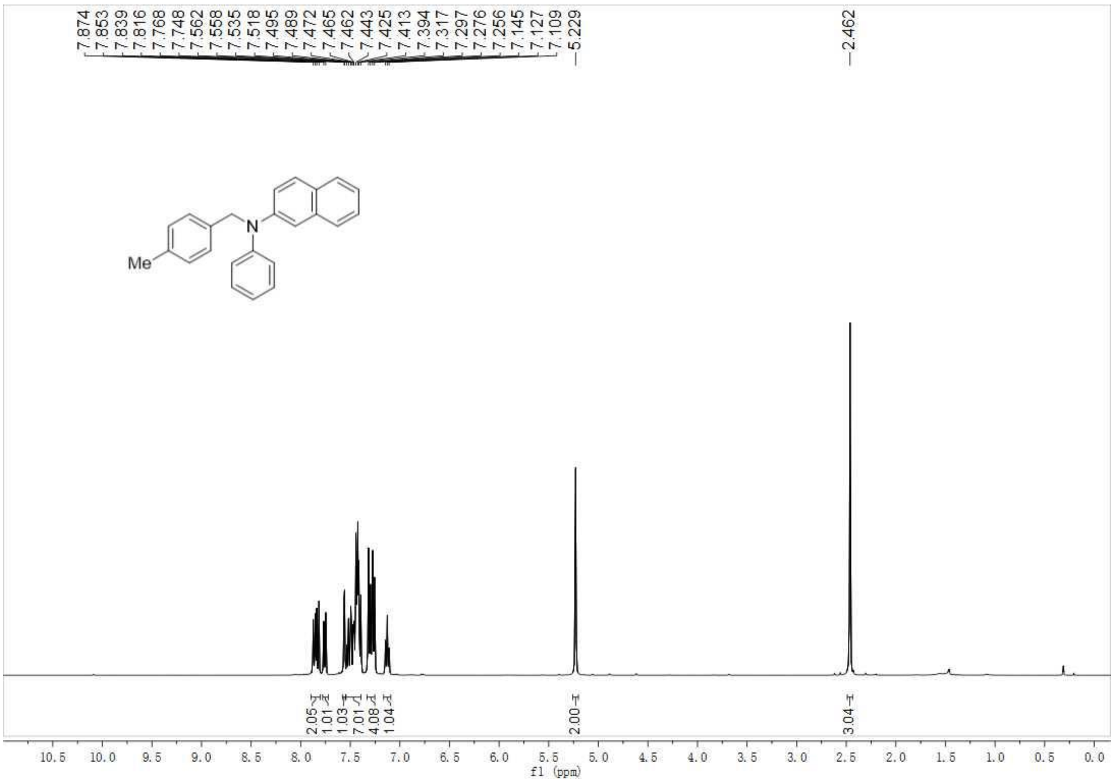

^{13}C NMR Spectrum of **252**

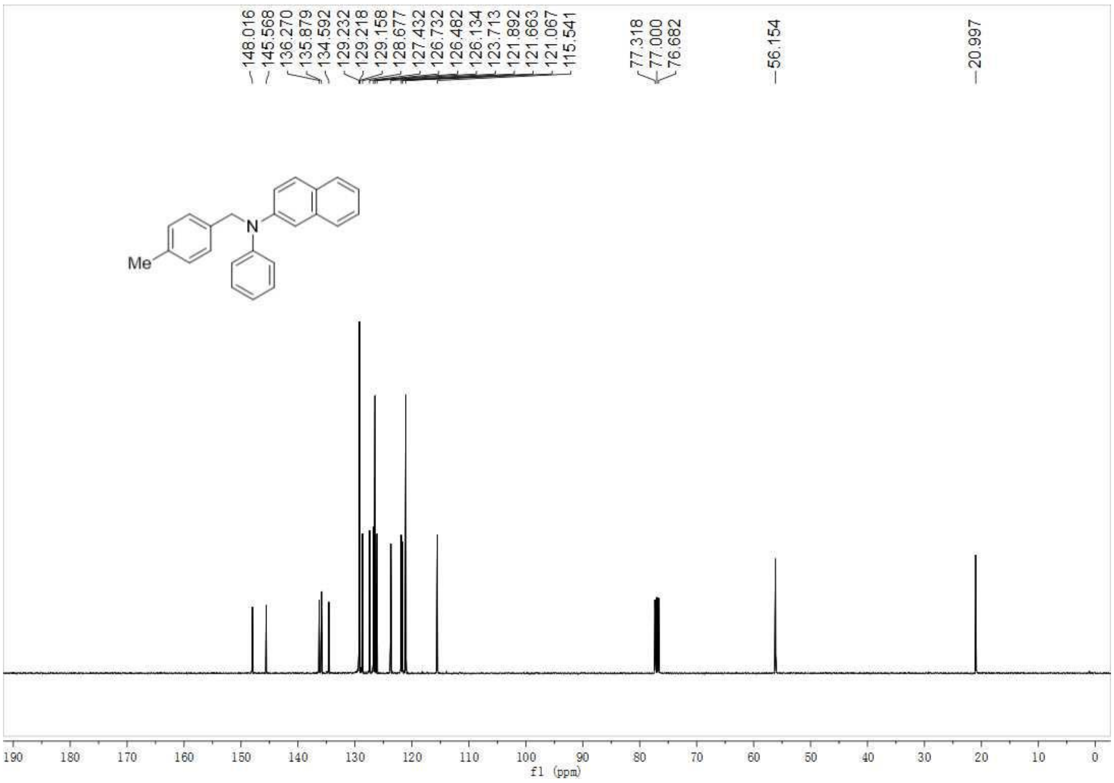

^{1}H NMR Spectrum of **253**

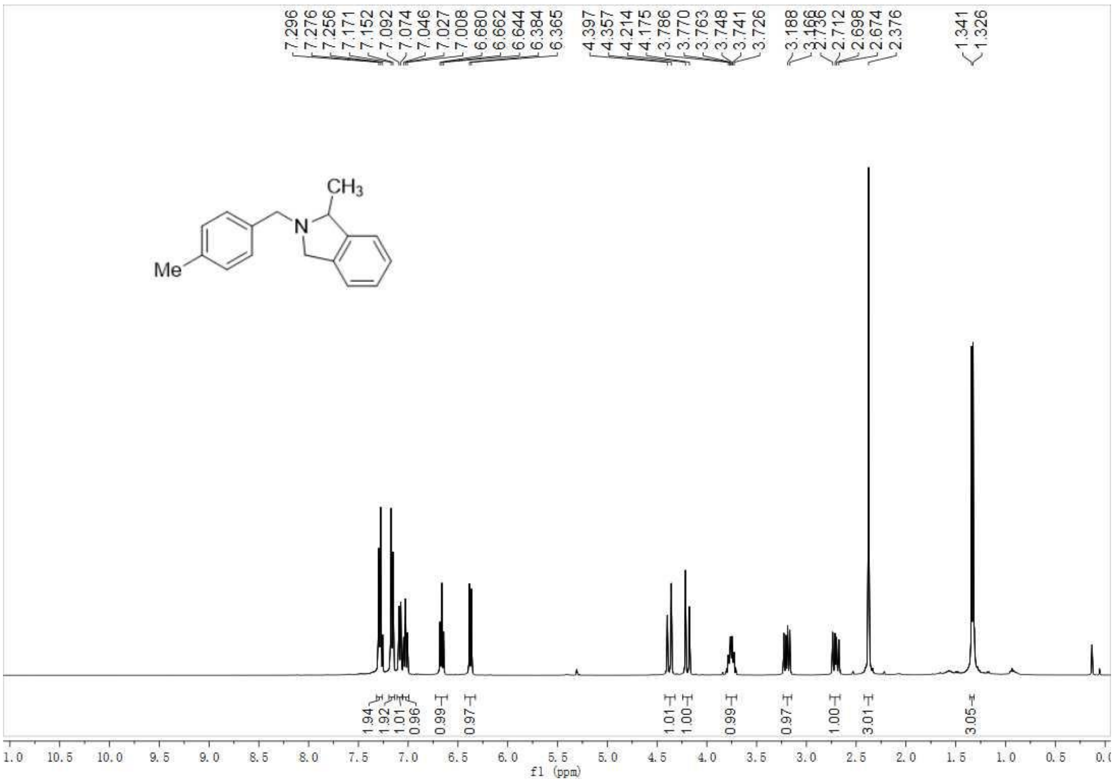

^{13}C NMR Spectrum of **253**

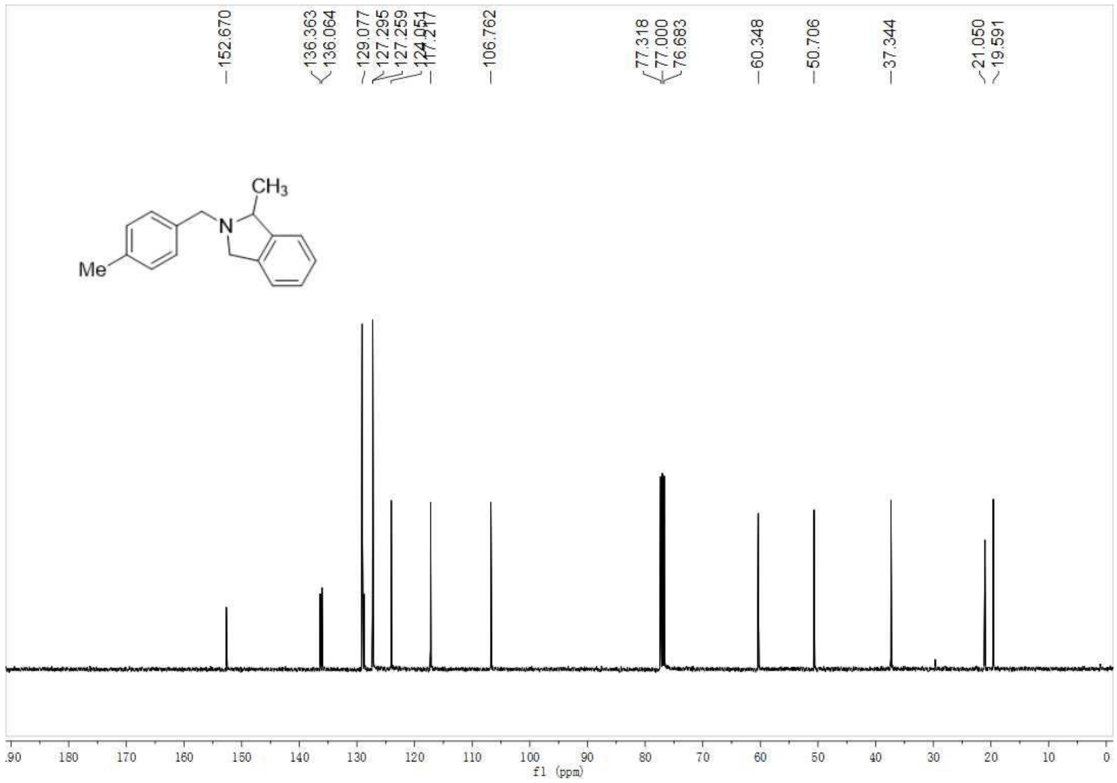

^{1}H NMR Spectrum of **254**

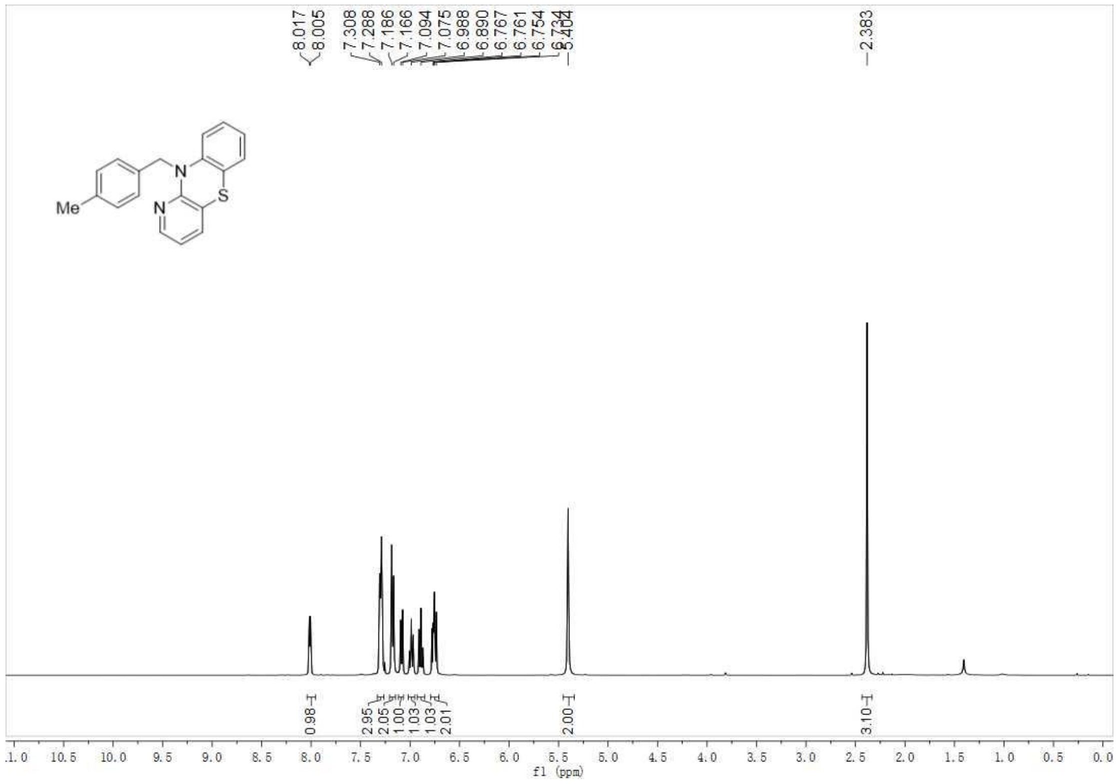

^{13}C NMR Spectrum of **254**

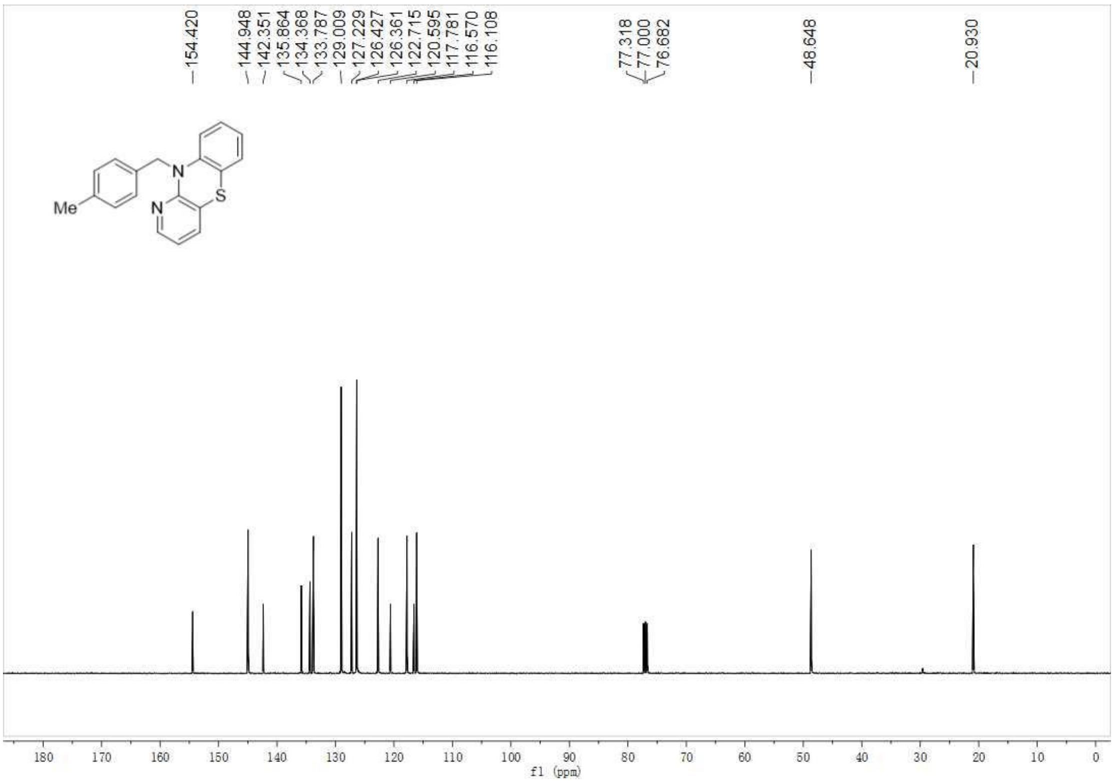

^{1}H NMR Spectrum of **255**

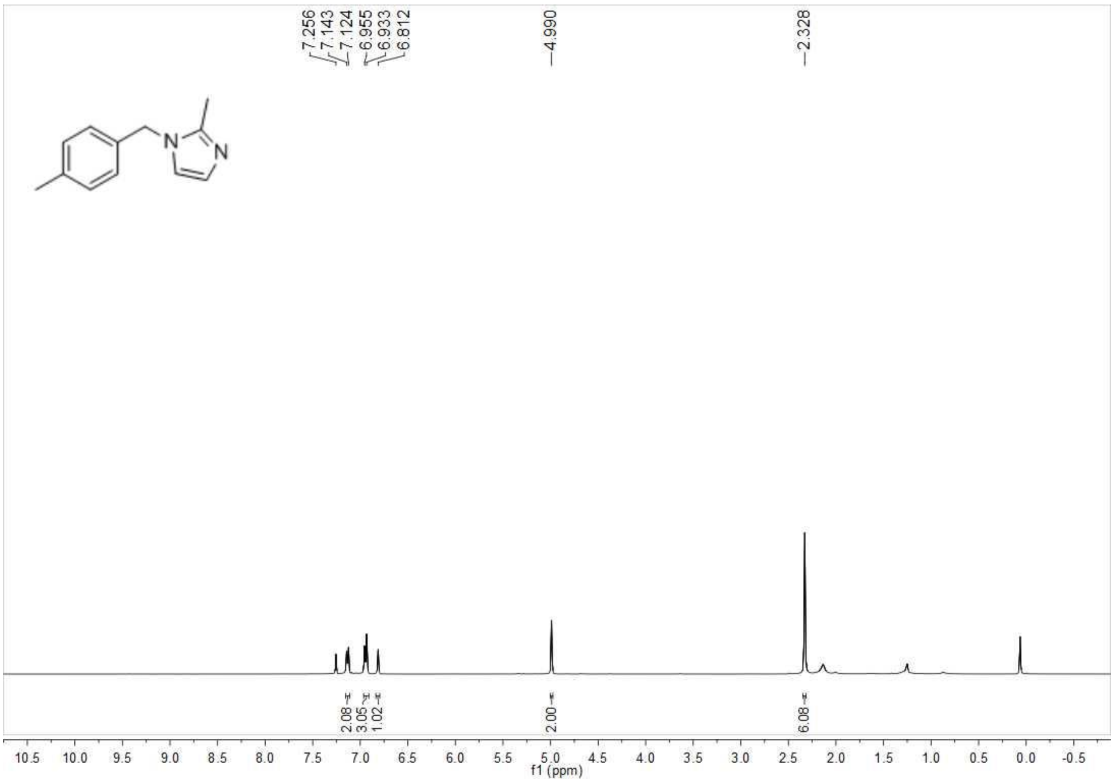

^{13}C NMR Spectrum of **255**

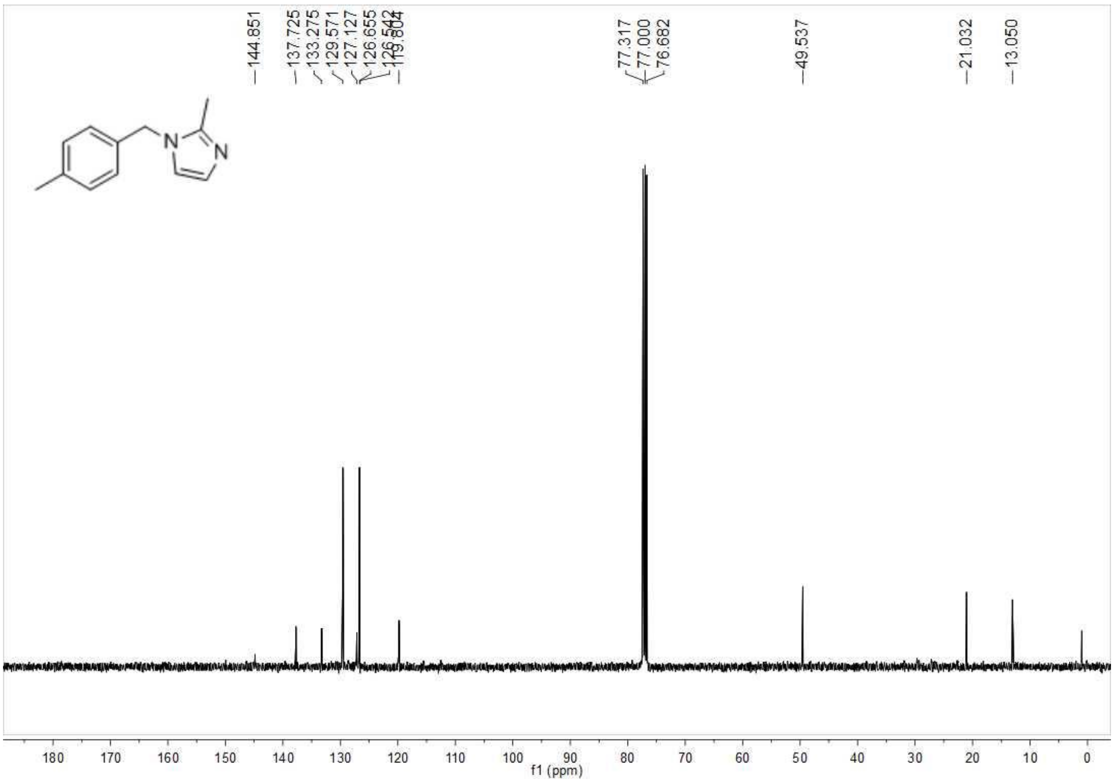

^{1}H NMR Spectrum of **256**

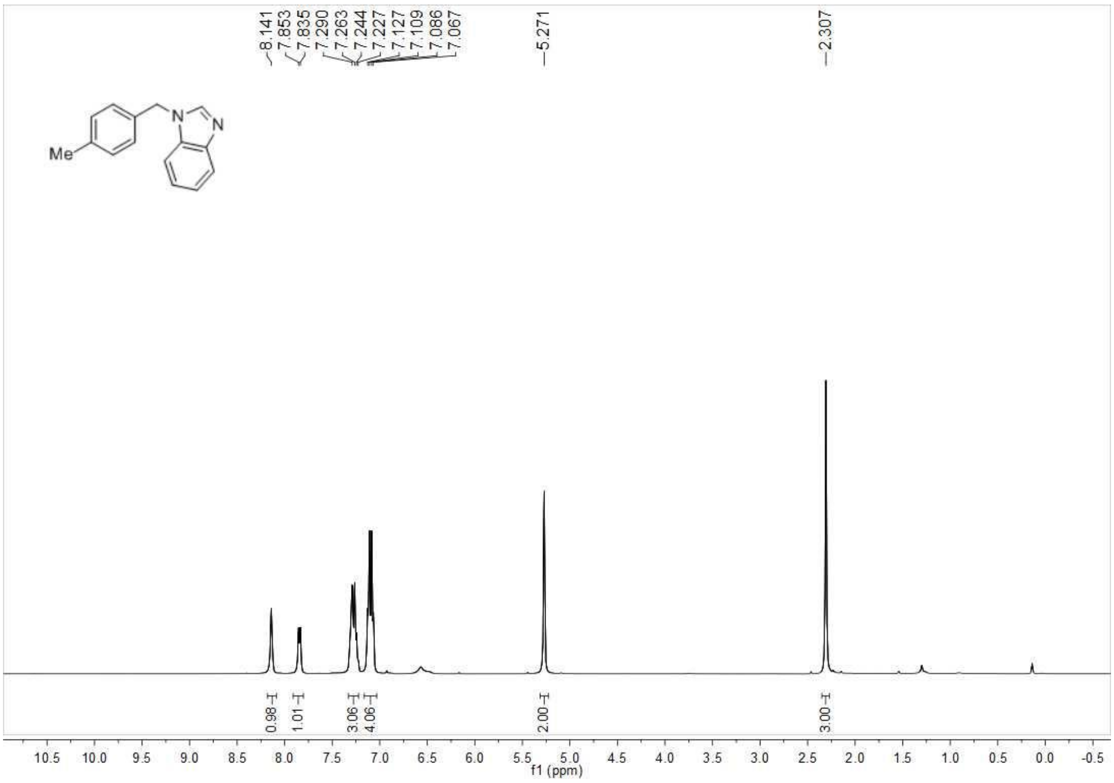

^{13}C NMR Spectrum of **256**

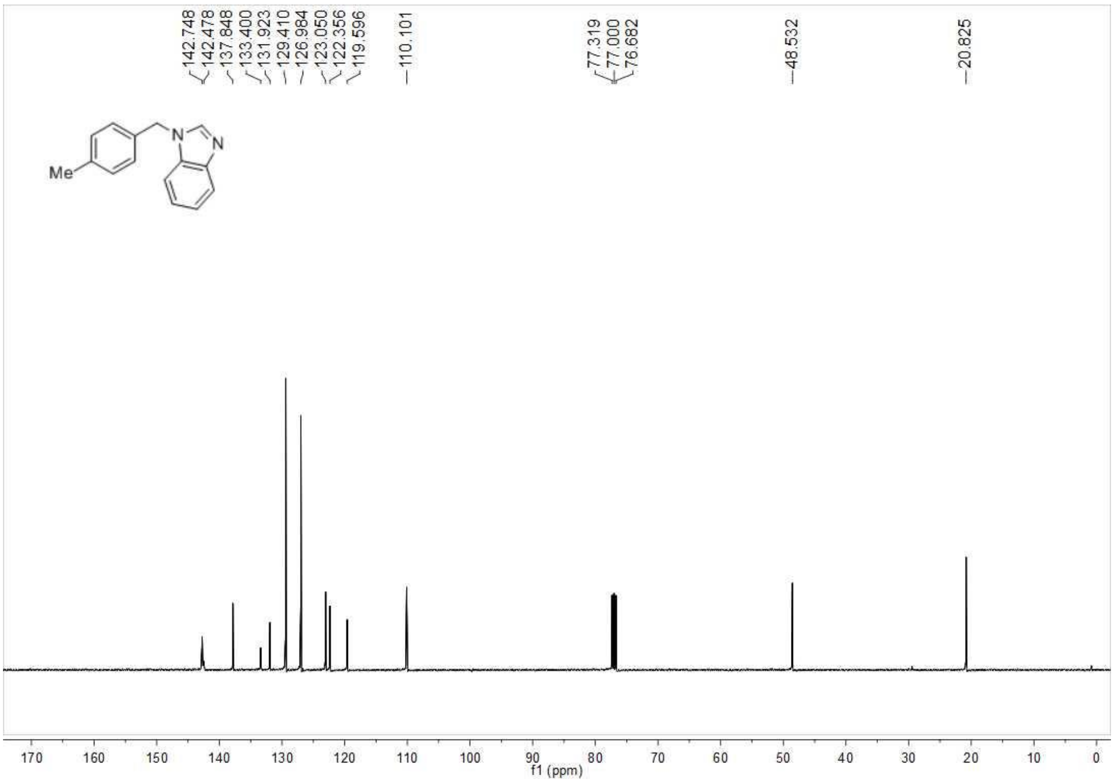

^{1}H NMR Spectrum of **257**

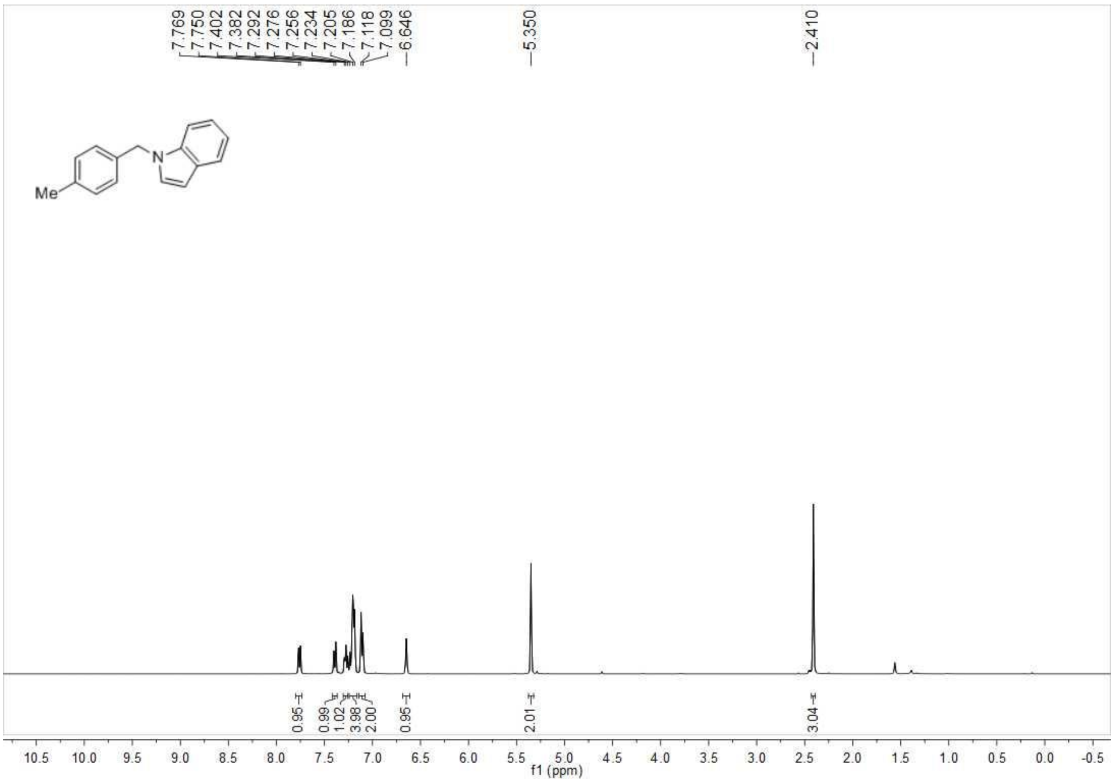

^{13}C NMR Spectrum of **257**

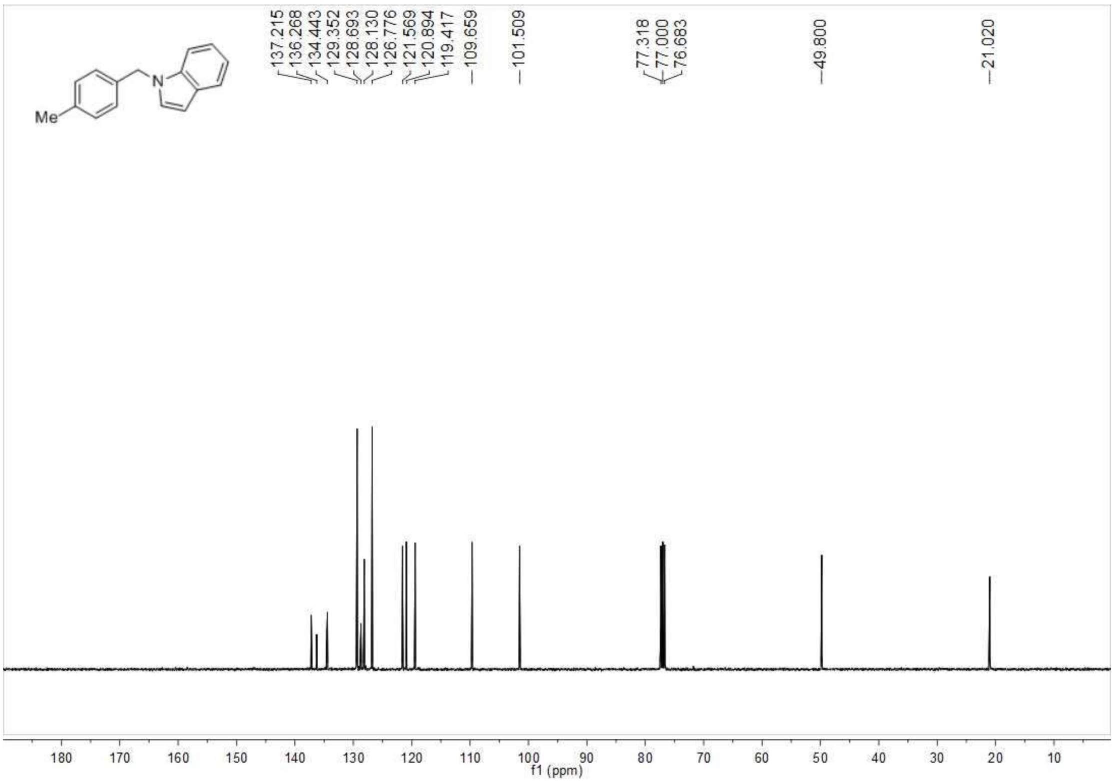

^{1}H NMR Spectrum of **258**

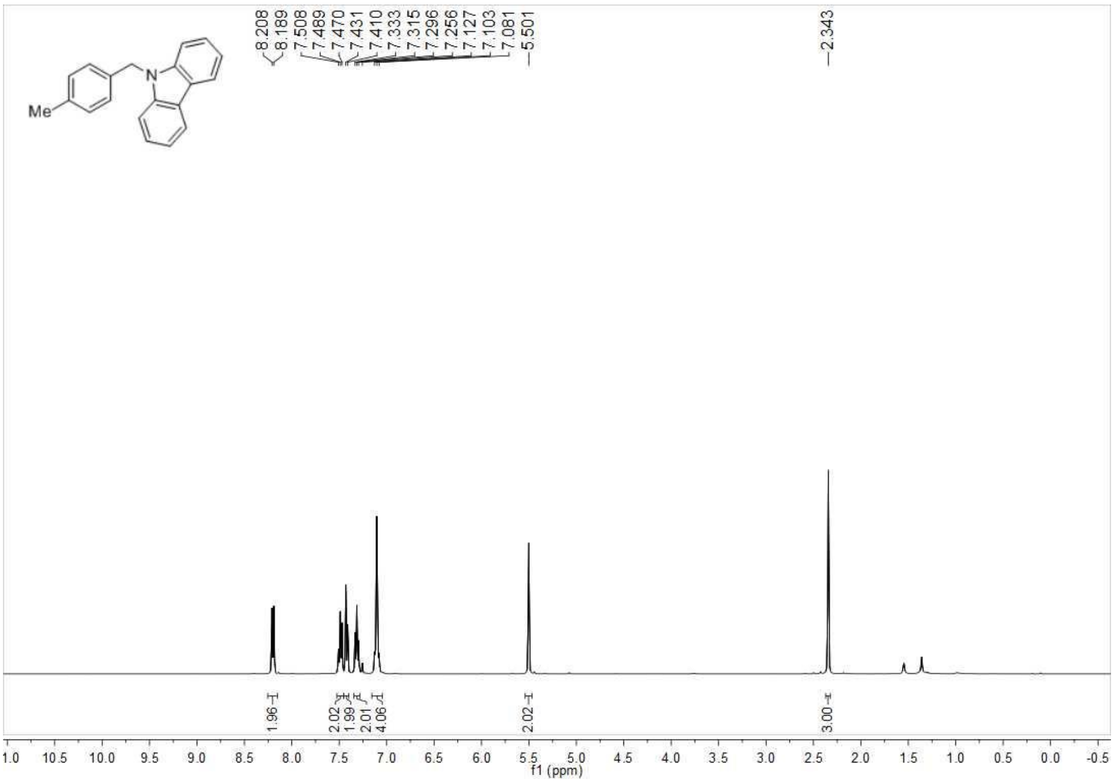

^{13}C NMR Spectrum of **258**

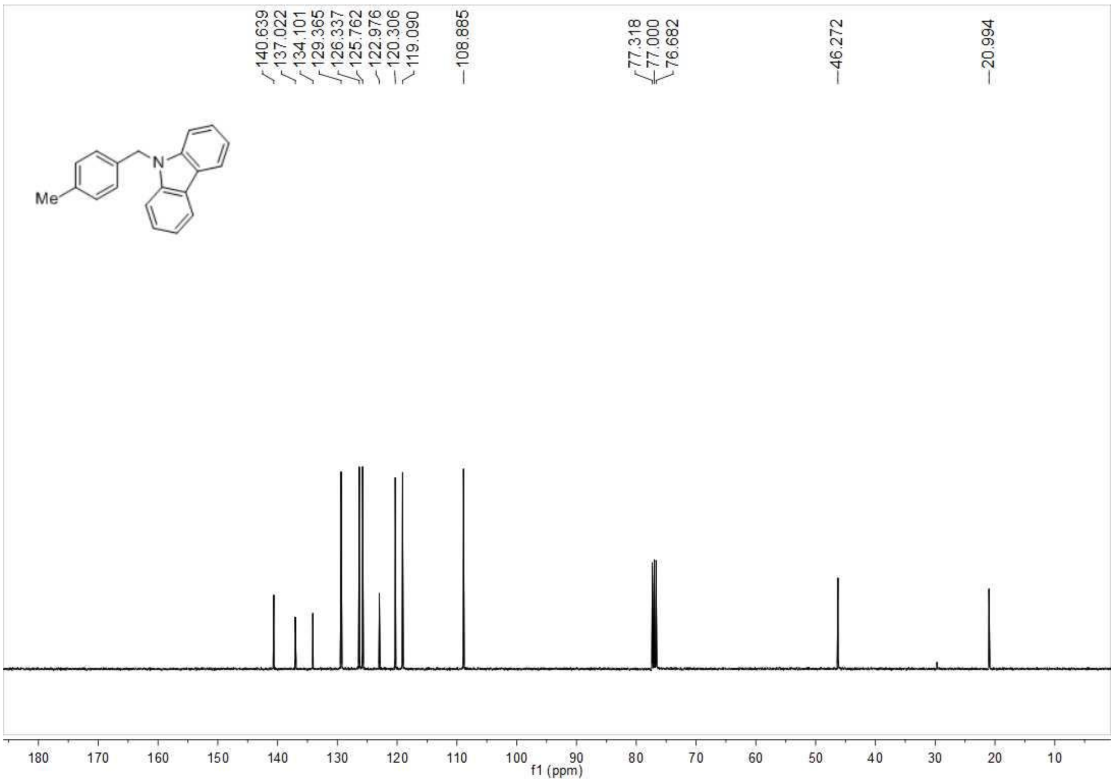

^{1}H NMR Spectrum of **259**

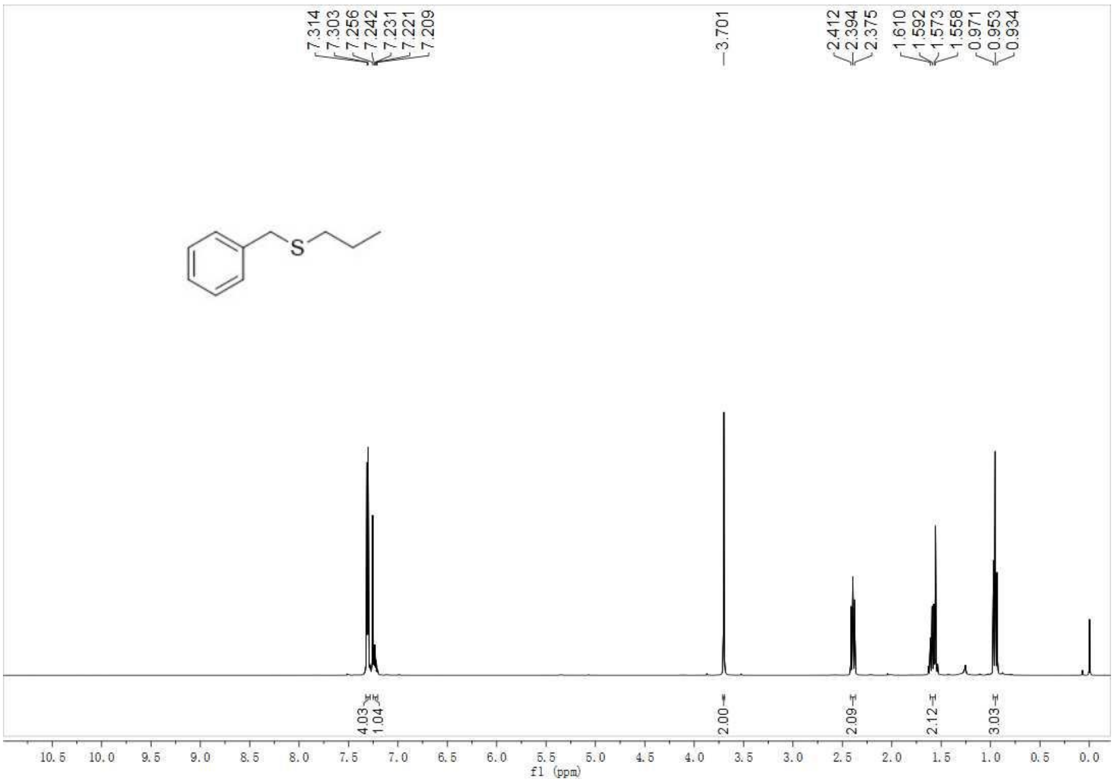

^{13}C NMR Spectrum of **259**

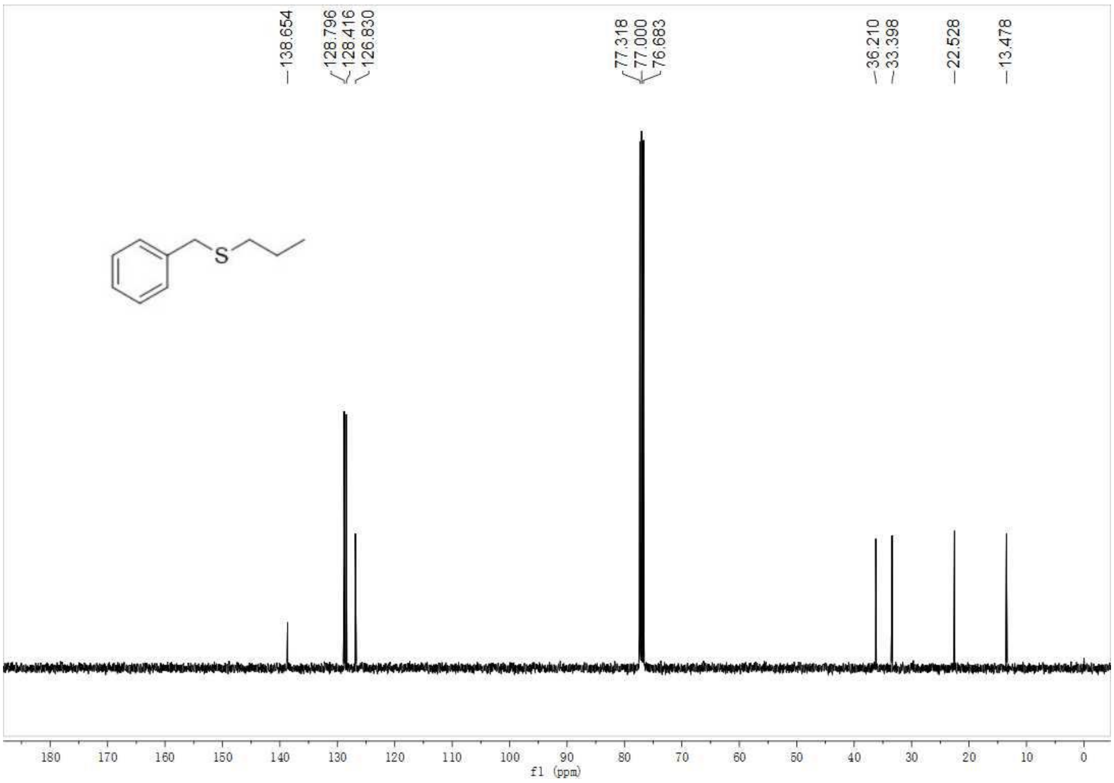

^{1}H NMR Spectrum of **260**

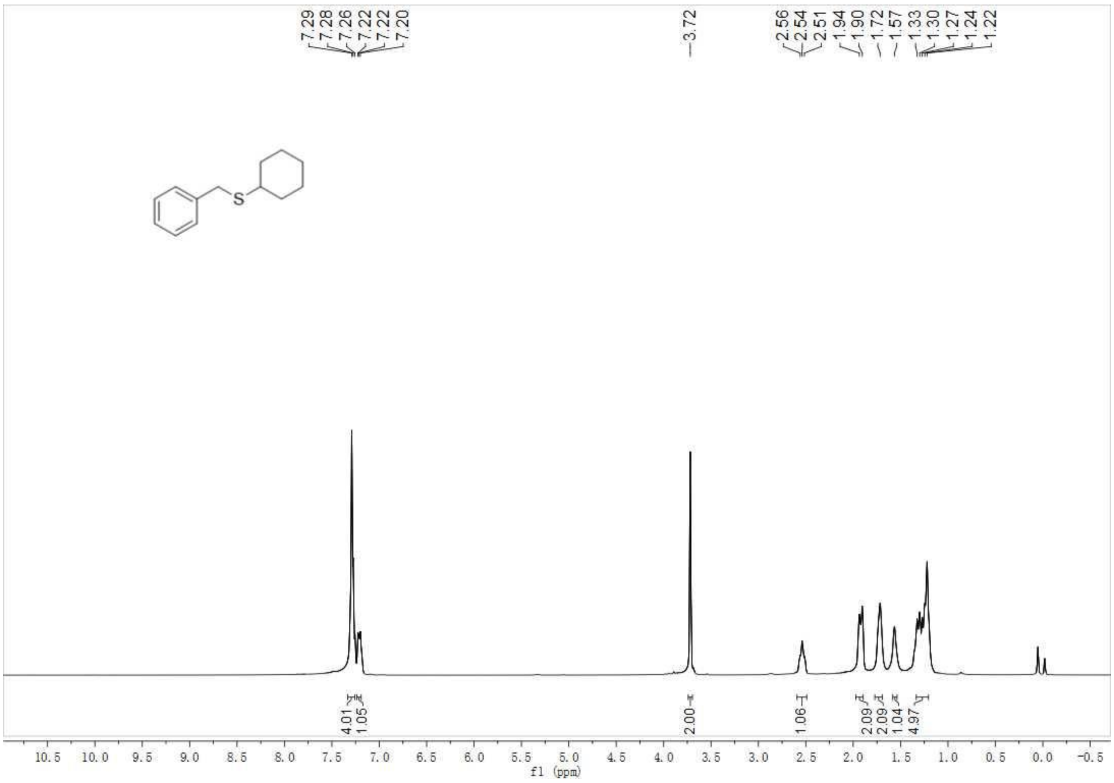

^{13}C NMR Spectrum of **260**

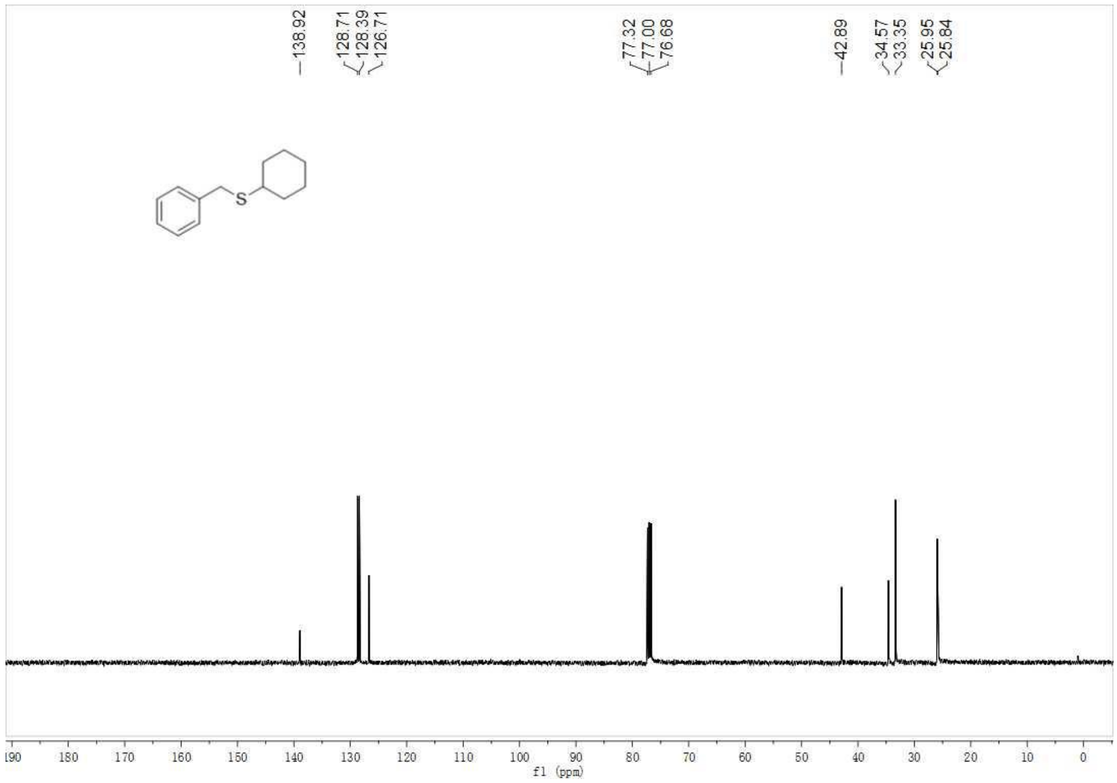

^{1}H NMR Spectrum of **261**

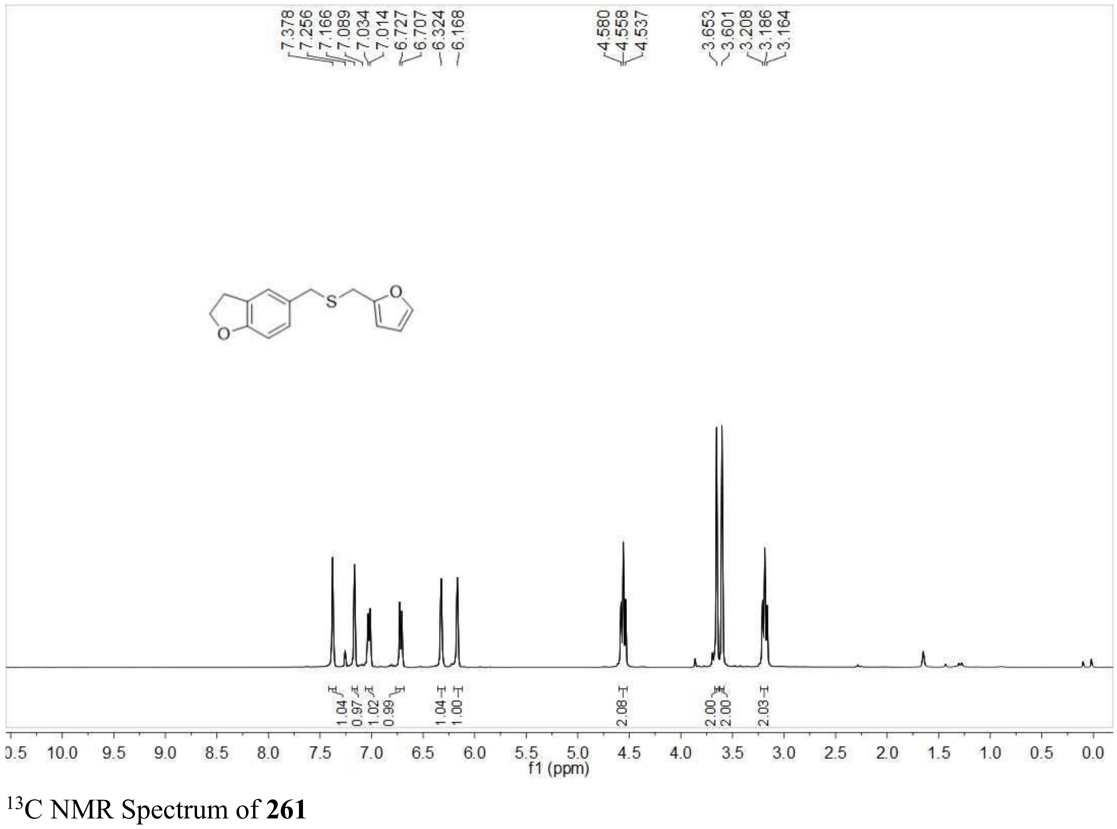

^{13}C NMR Spectrum of **261**

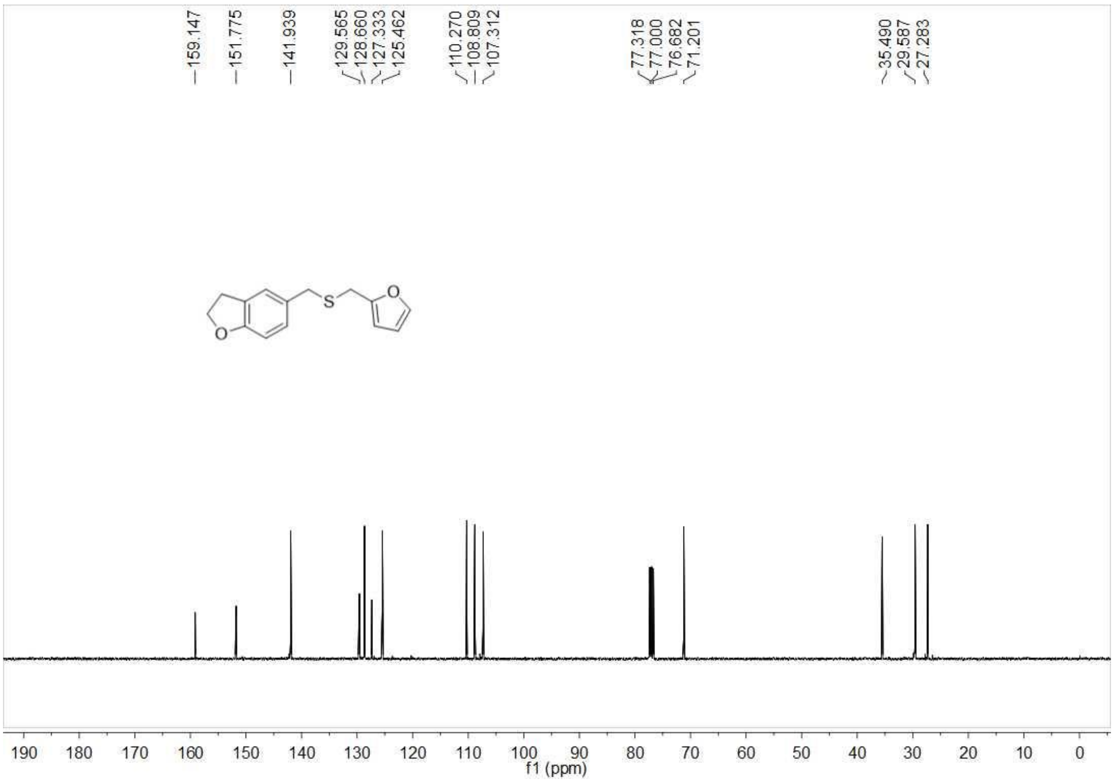

^{1}H NMR Spectrum of **262**

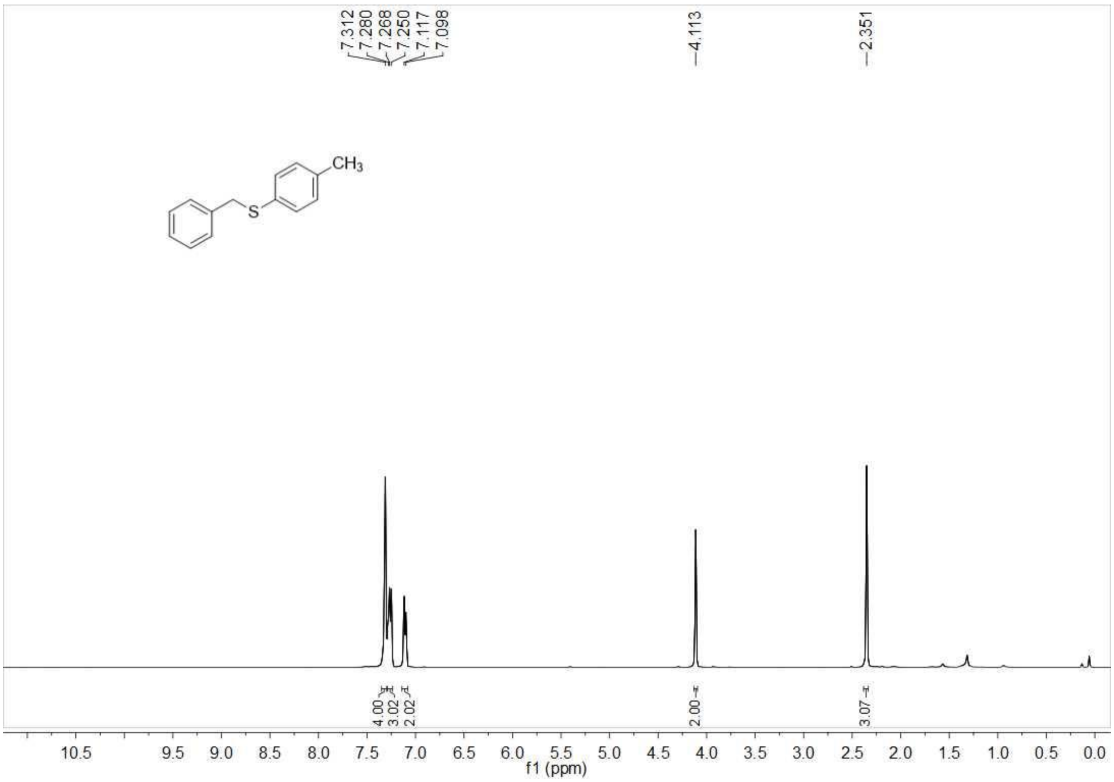

^{13}C NMR Spectrum of **262**

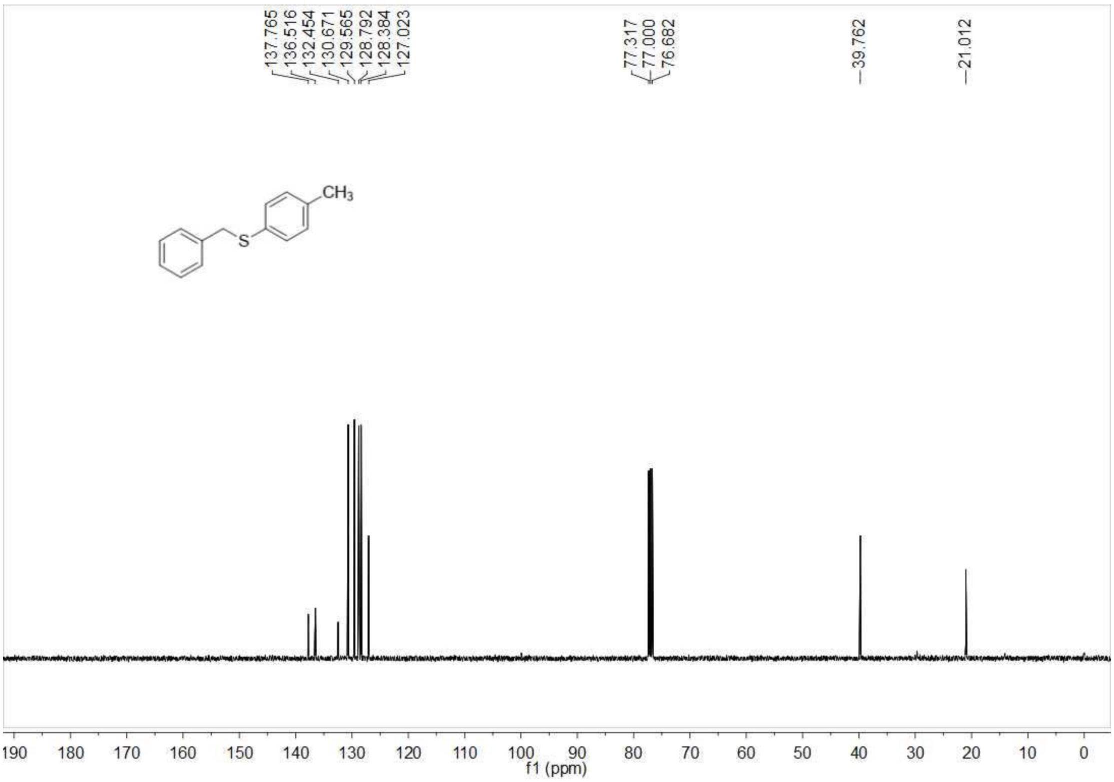

^{1}H NMR Spectrum of **263**

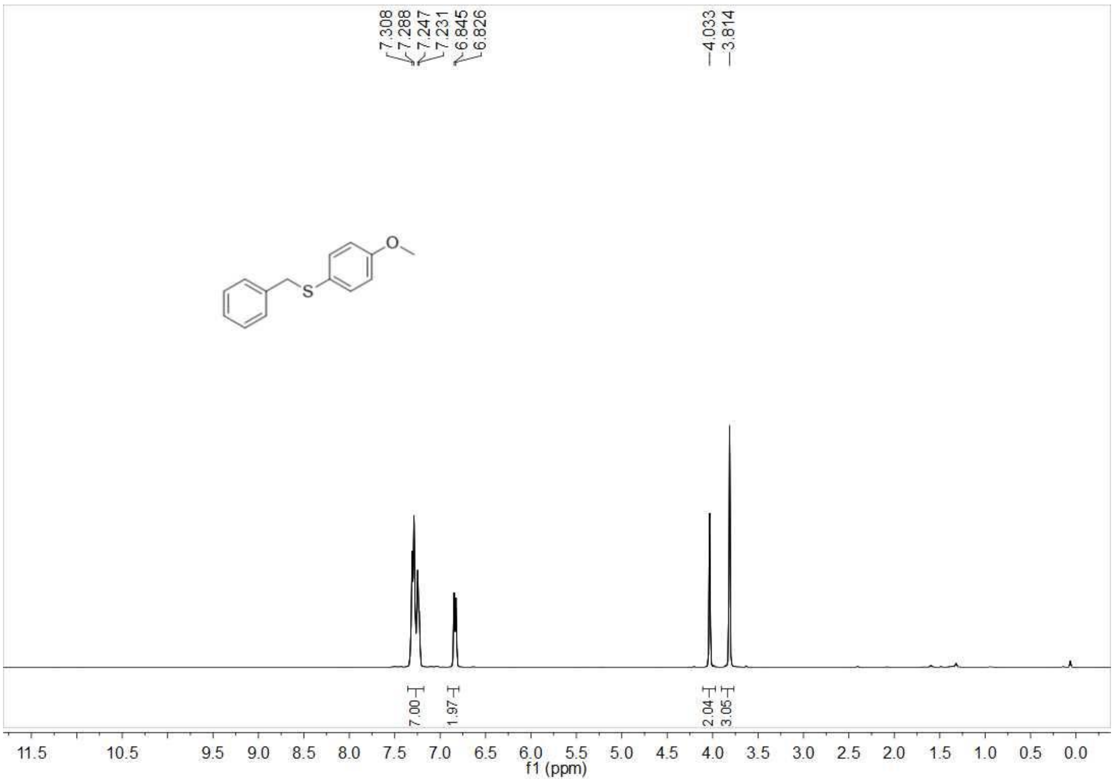

^{13}C NMR Spectrum of **263**

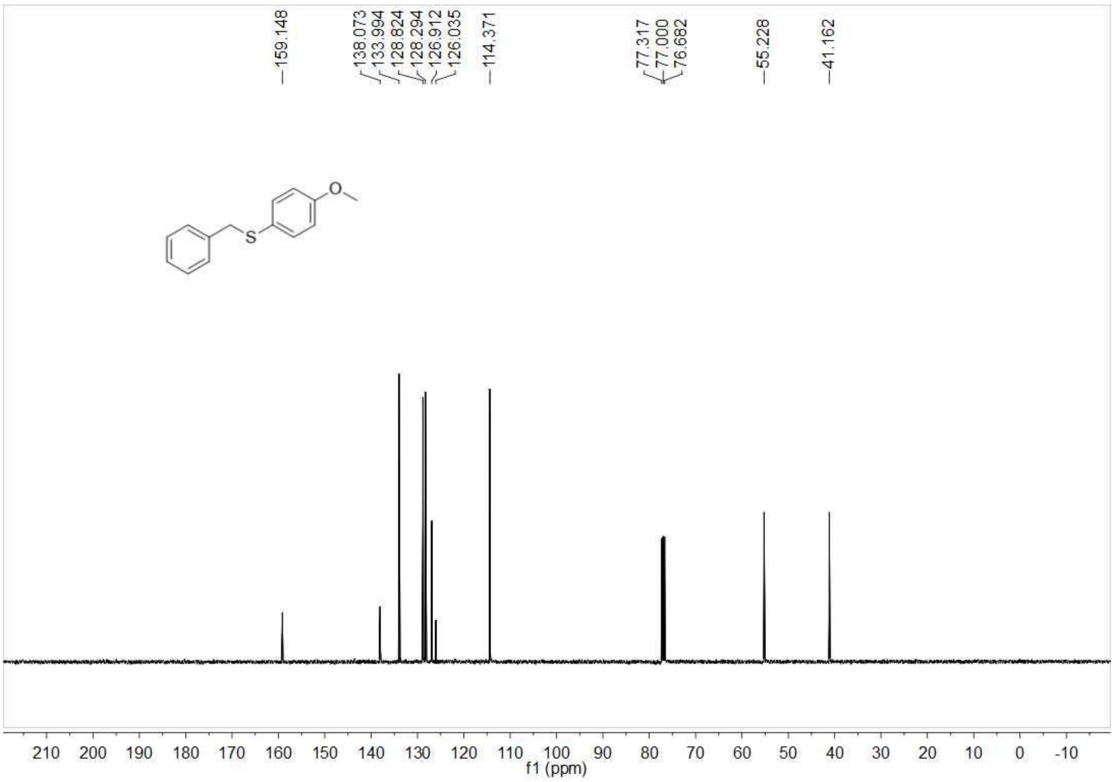

^{1}H NMR Spectrum of **264**

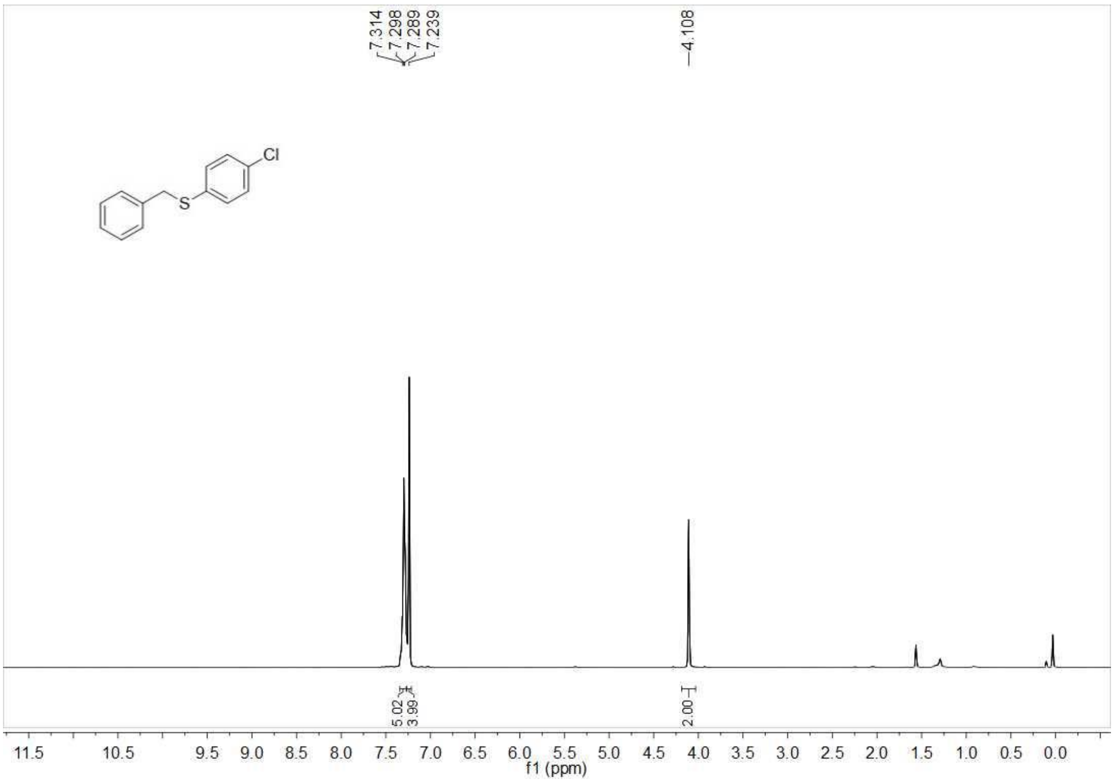

^{13}C NMR Spectrum of **264**

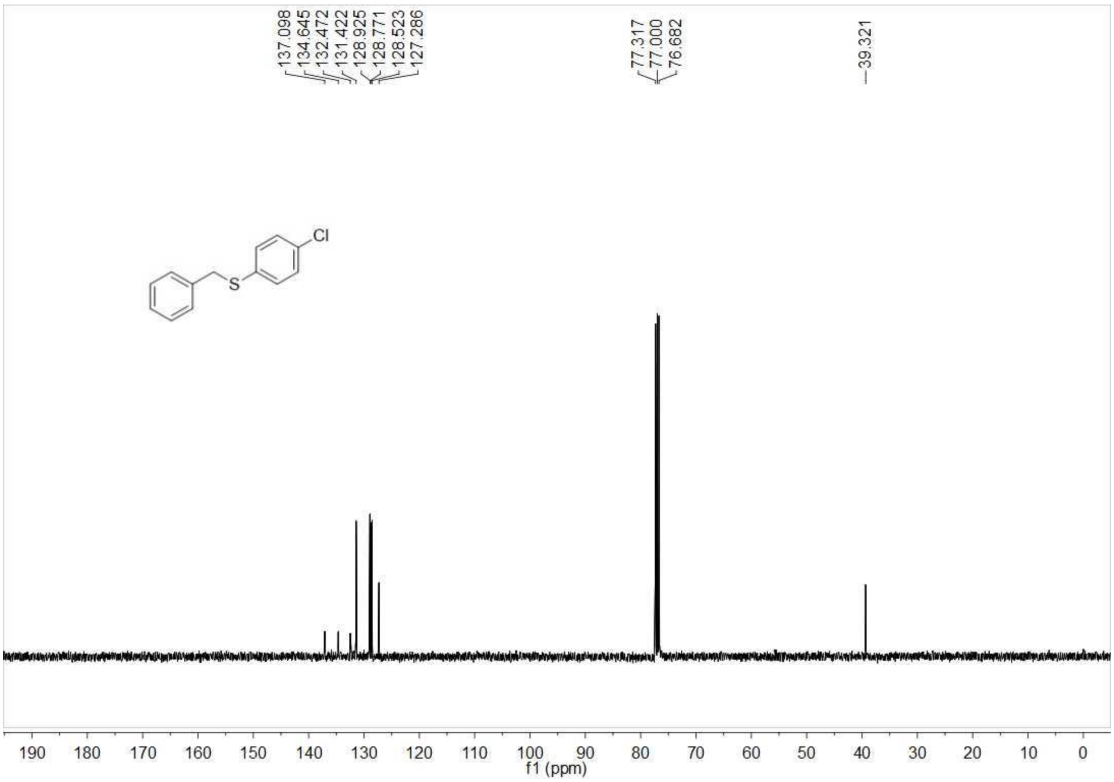

^{1}H NMR Spectrum of **265**

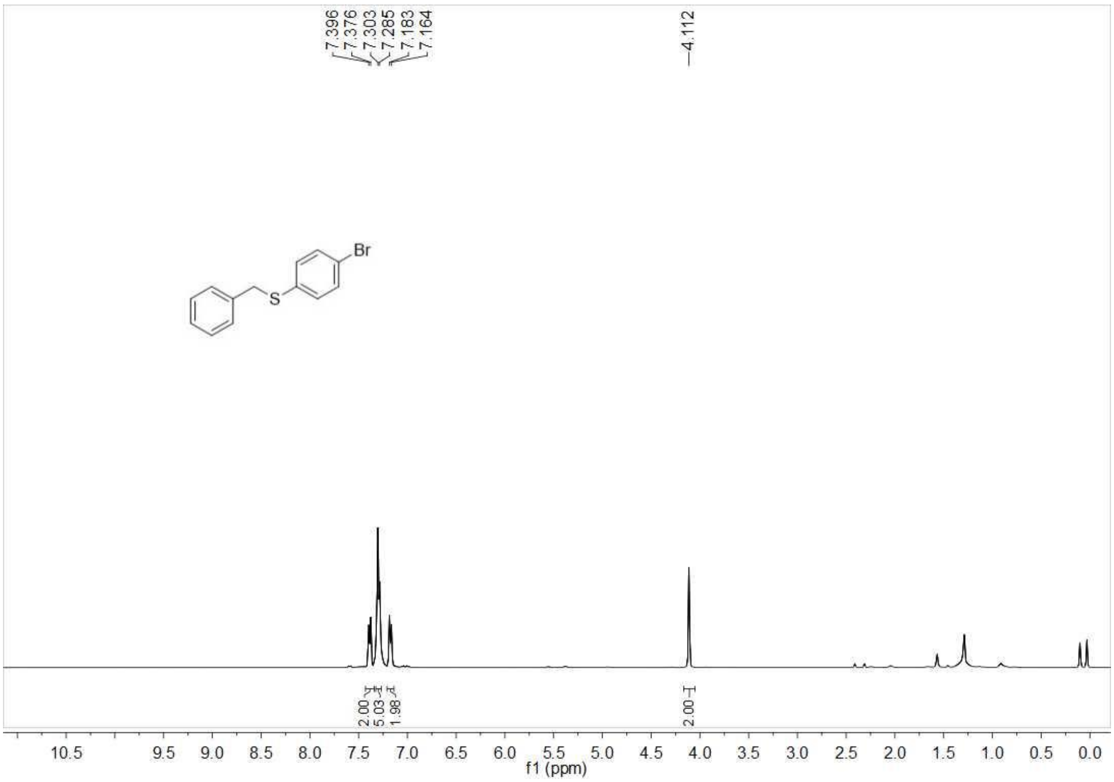

^{13}C NMR Spectrum of **265**

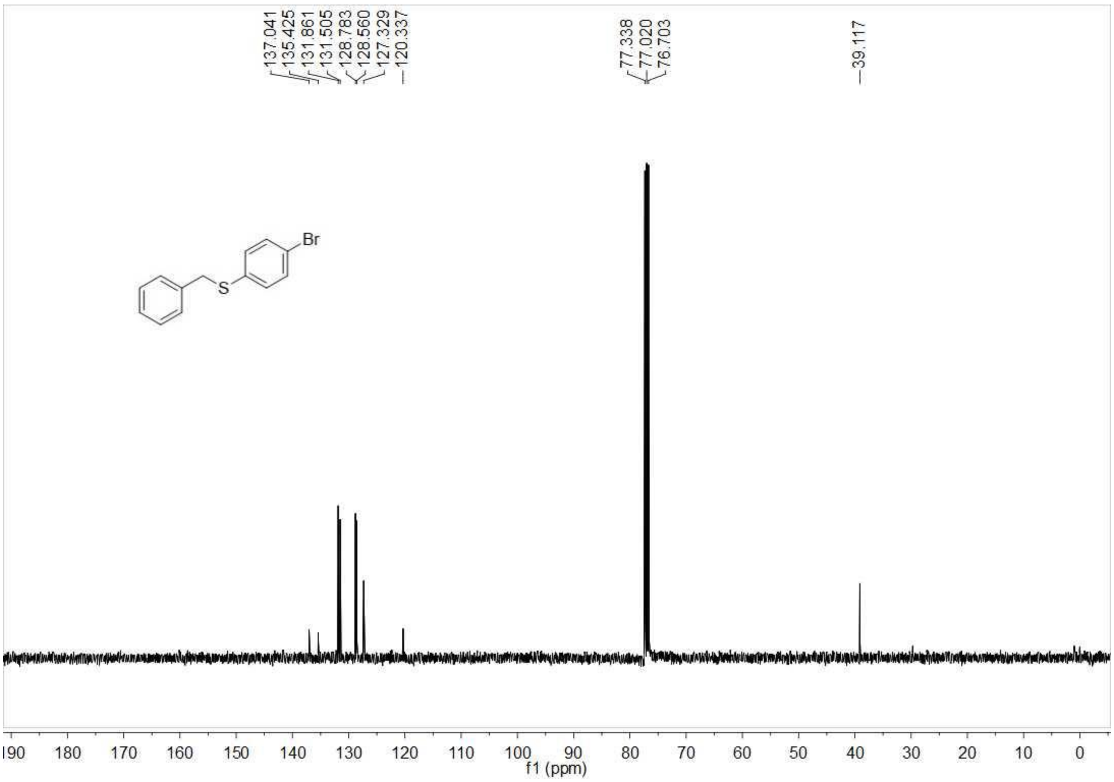

^{1}H NMR Spectrum of **266**

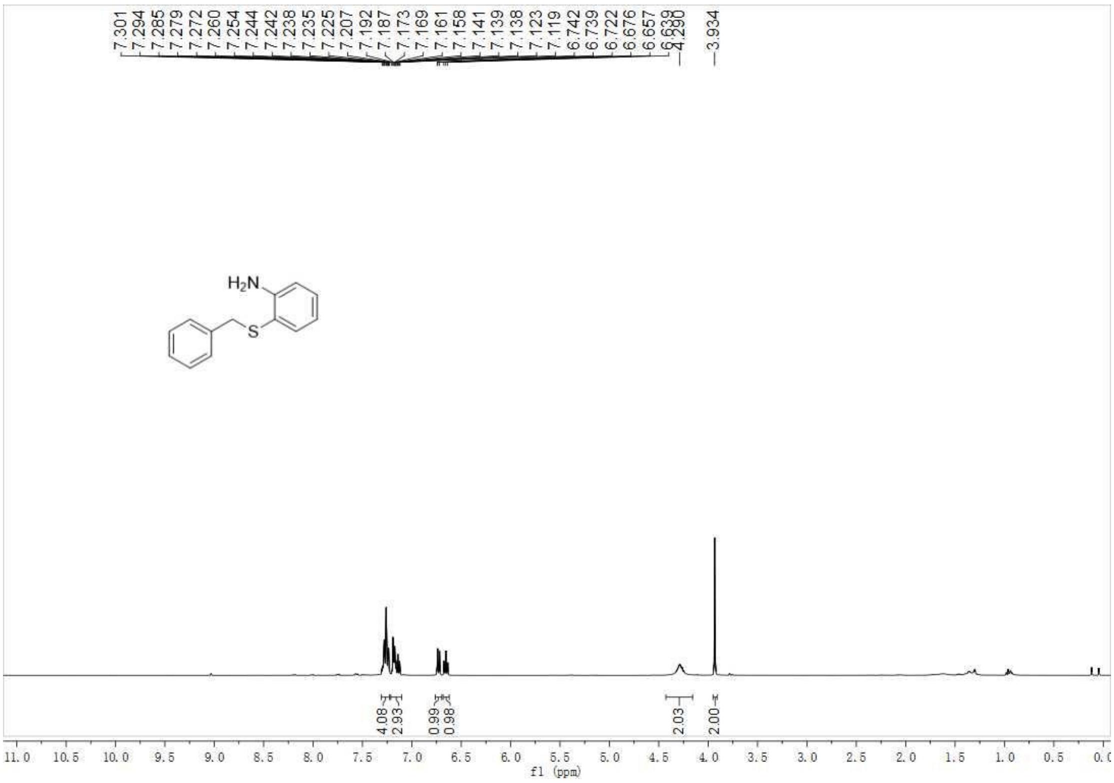

^{13}C NMR Spectrum of **266**

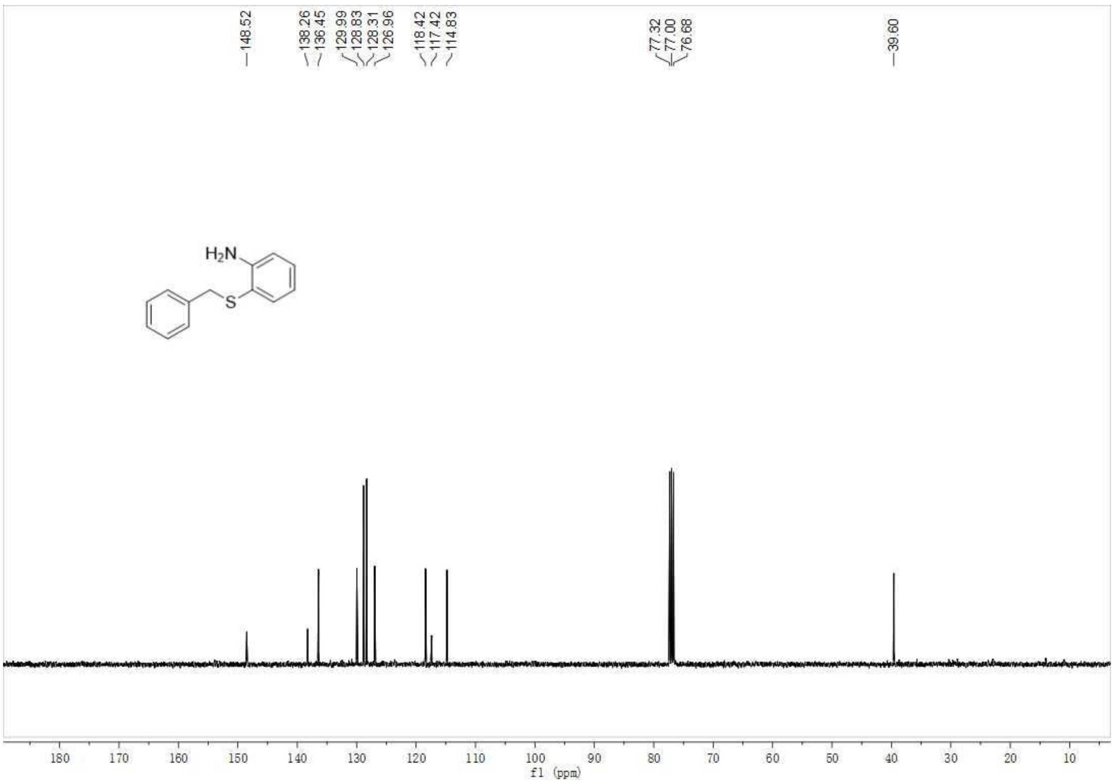

^{1}H NMR Spectrum of **267**

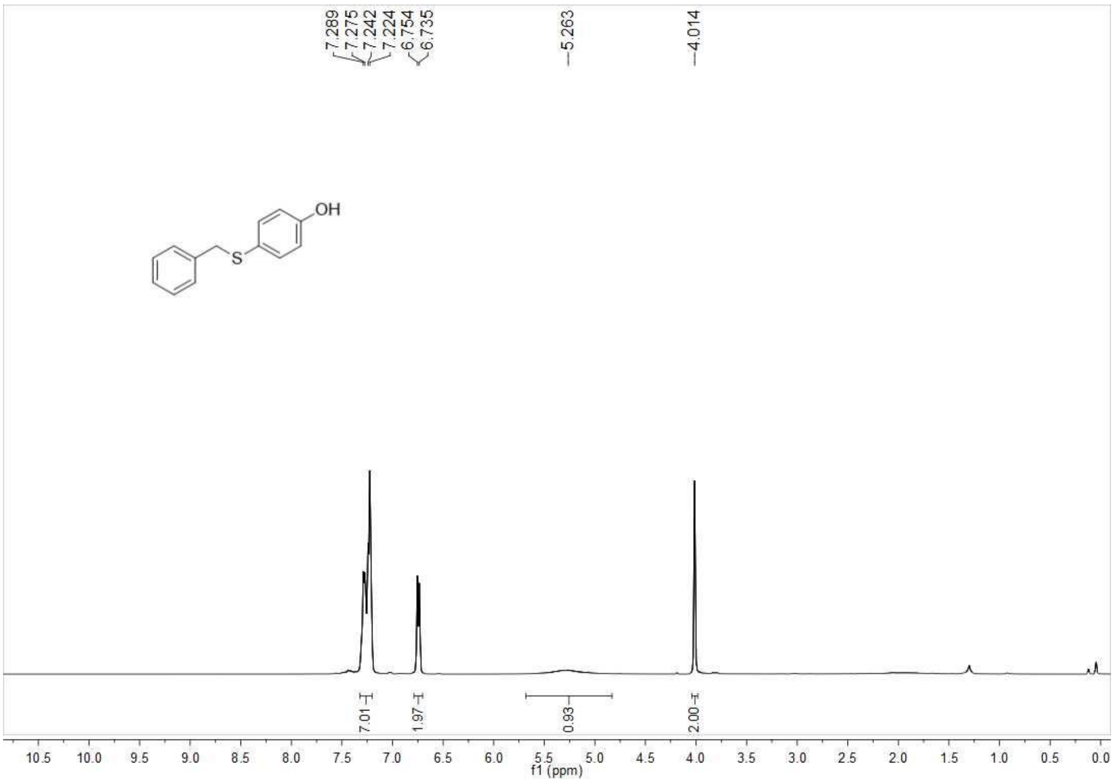

^{13}C NMR Spectrum of **267**

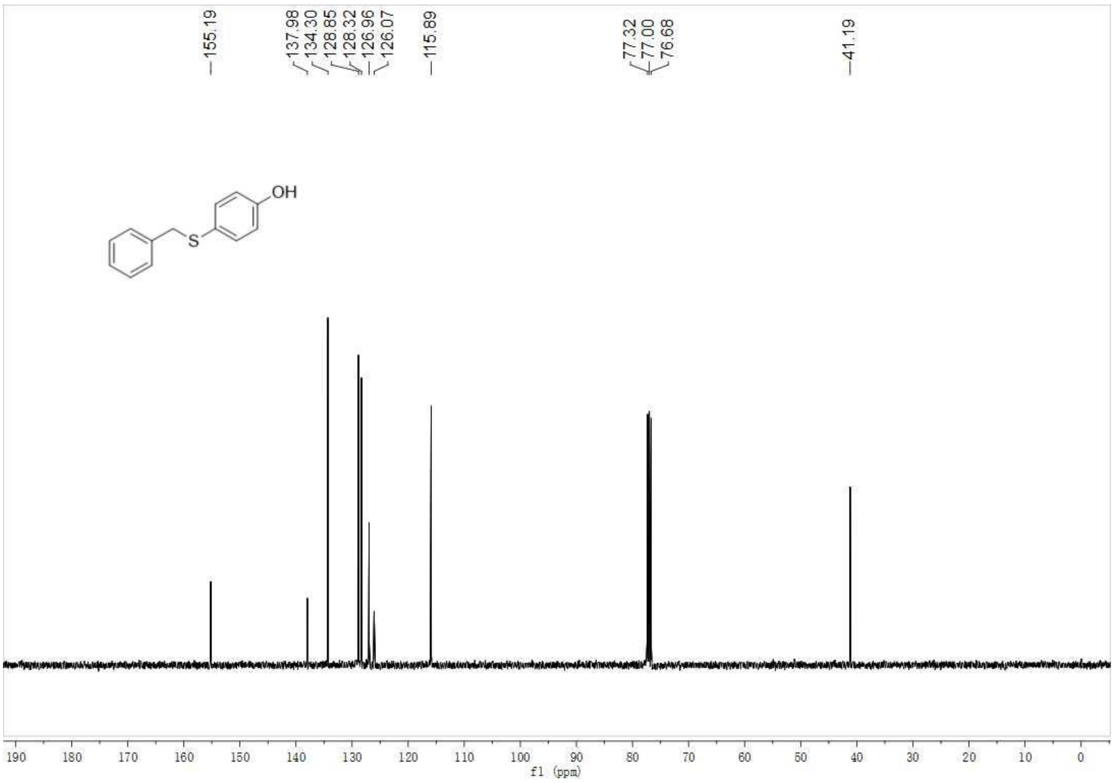

^{1}H NMR Spectrum of **268**

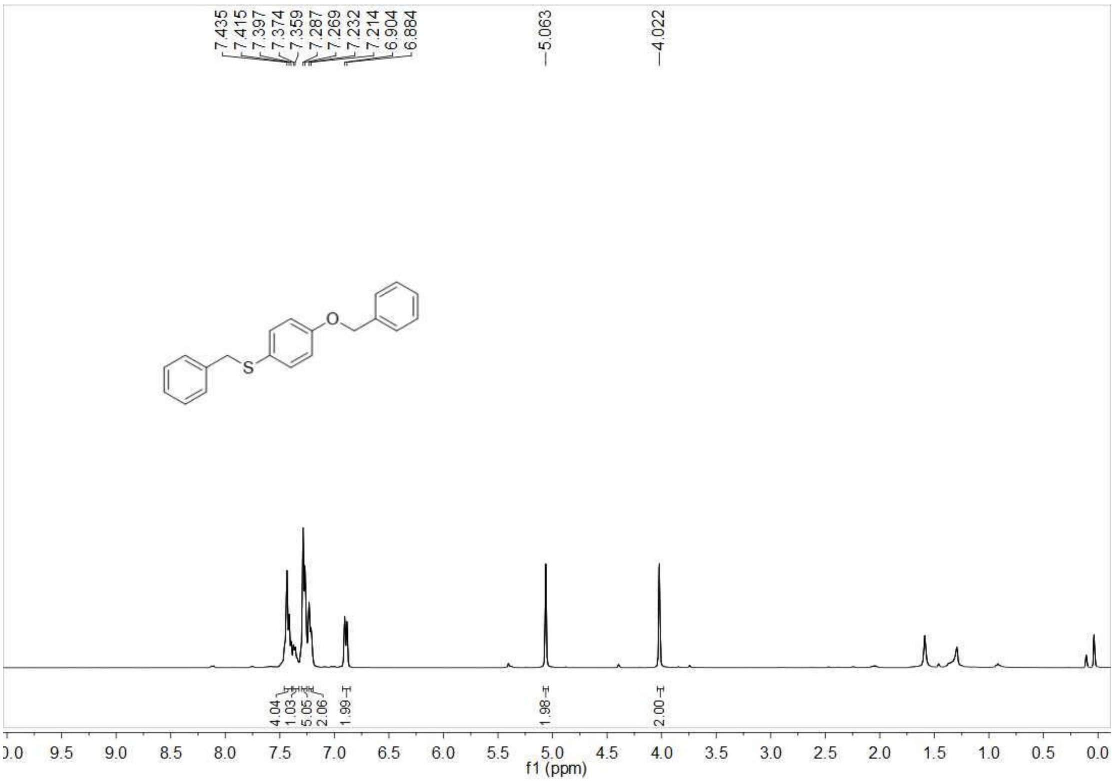

^{13}C NMR Spectrum of **268**

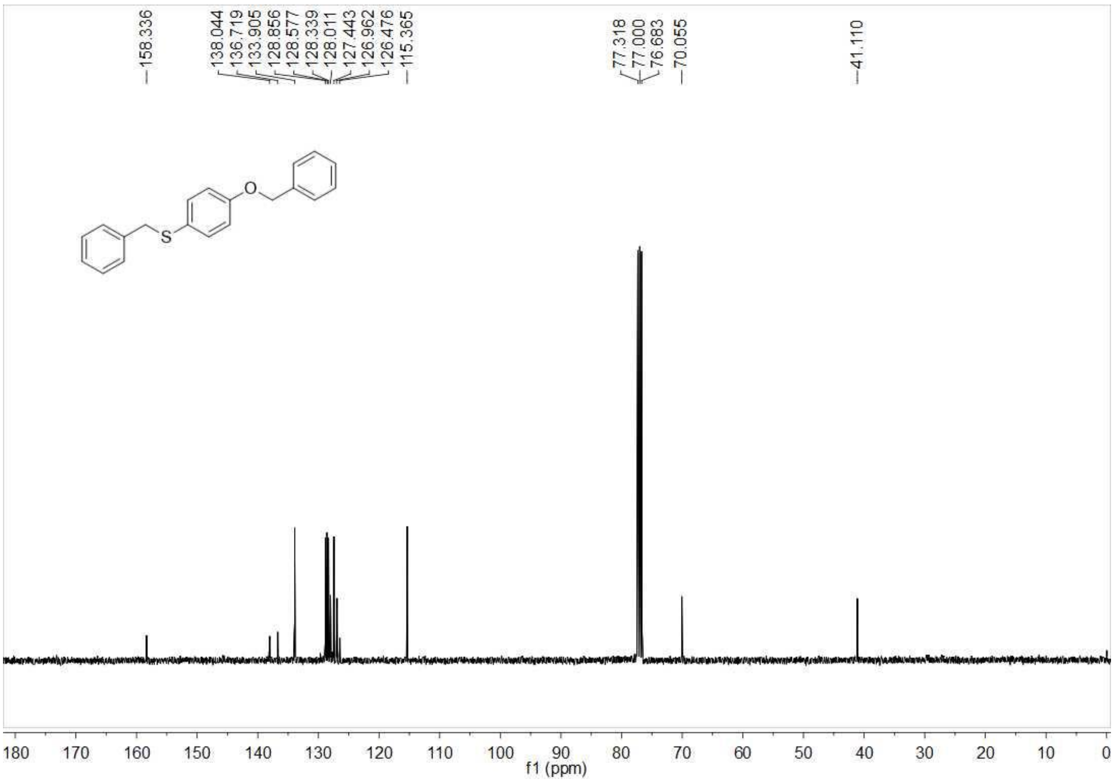

^{1}H NMR Spectrum of **269**

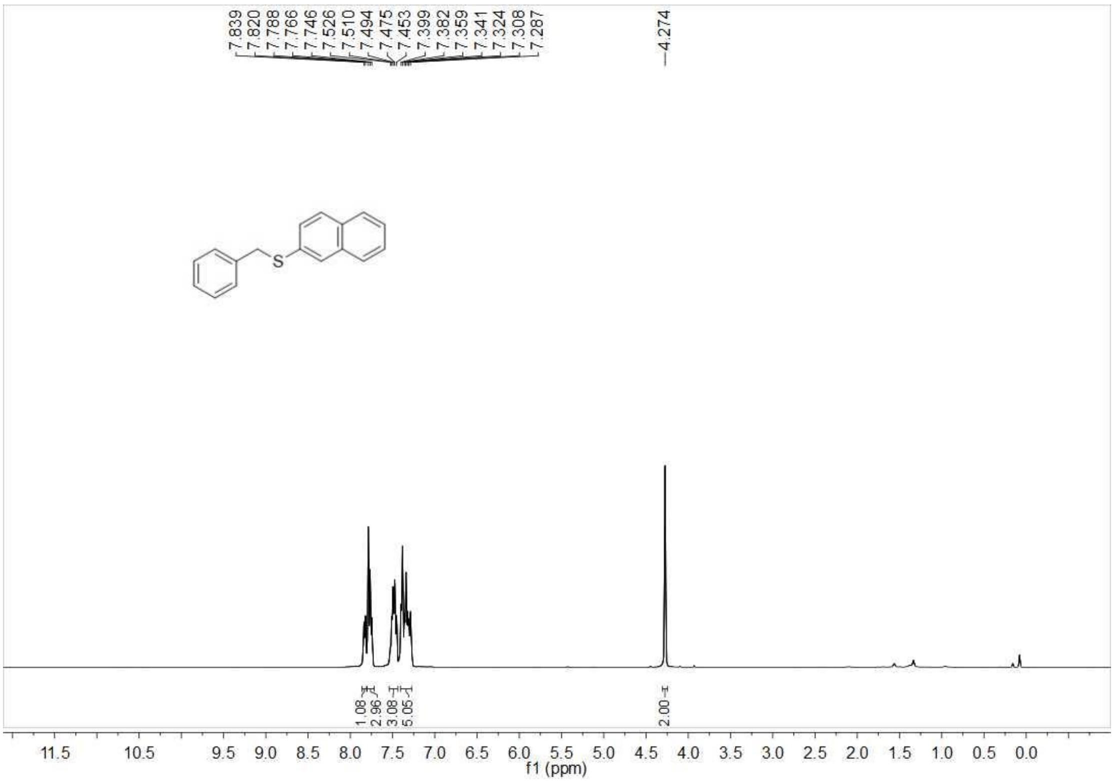

^{13}C NMR Spectrum of **269**

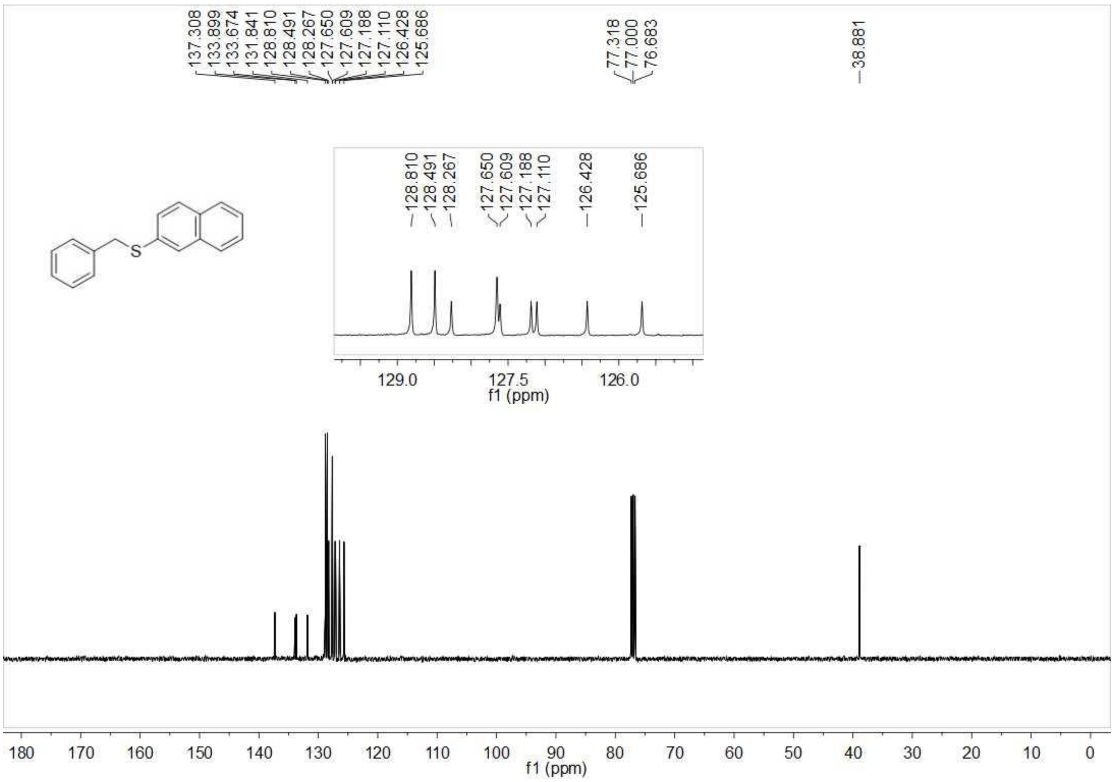

^{1}H NMR Spectrum of **270**

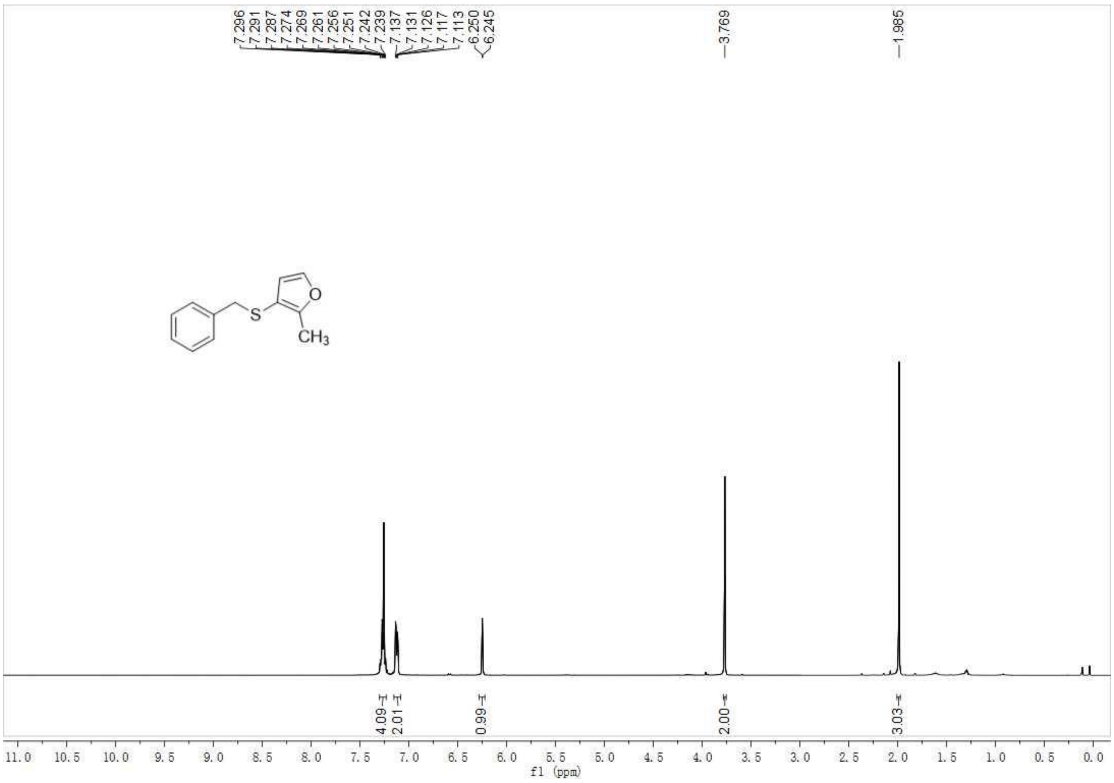

^{13}C NMR Spectrum of **270**

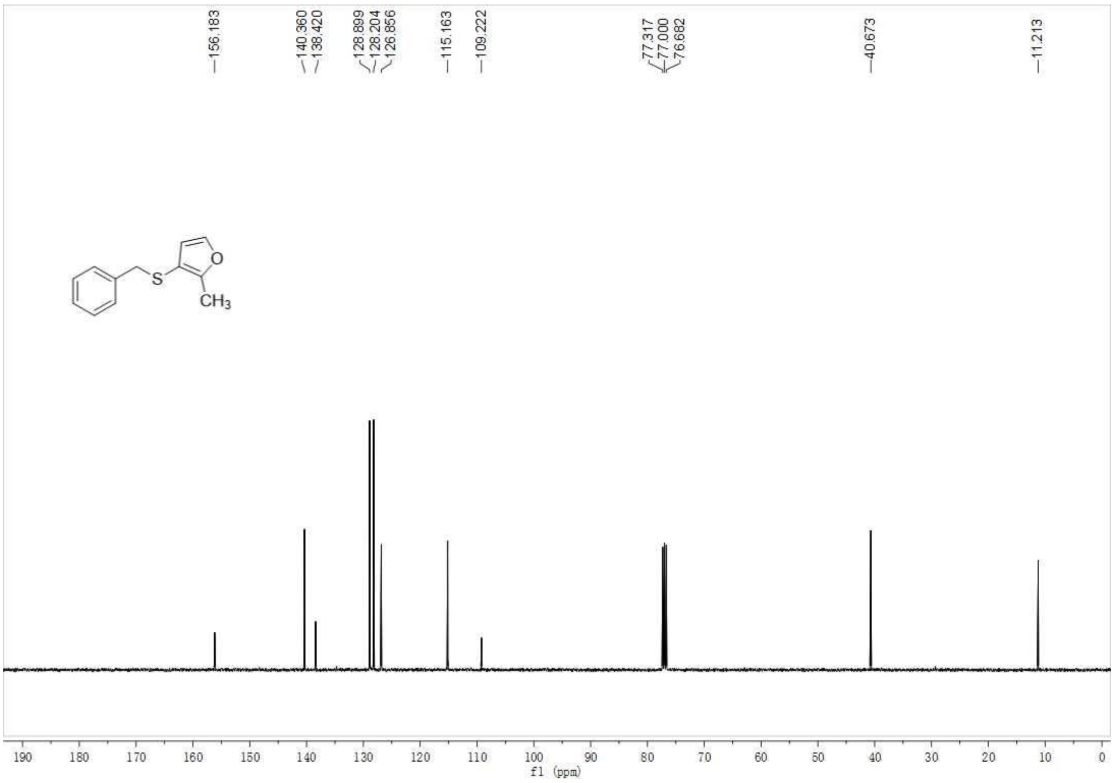

^{1}H NMR Spectrum of **271**

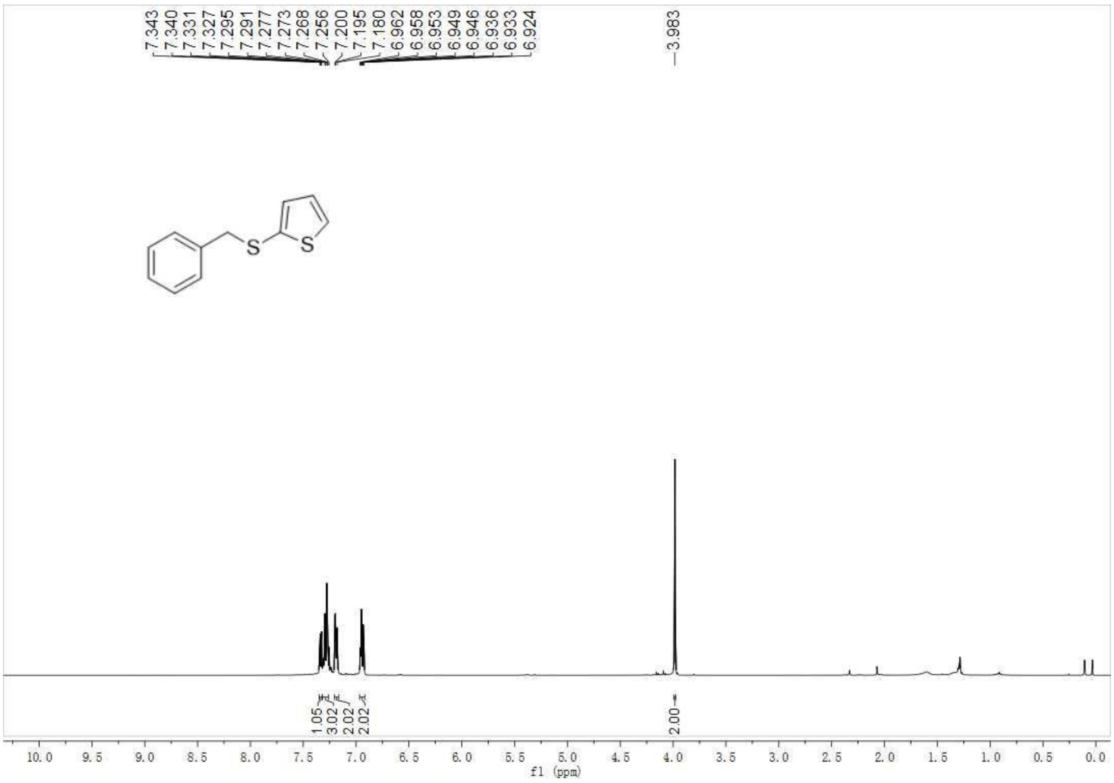

^{13}C NMR Spectrum of **271**

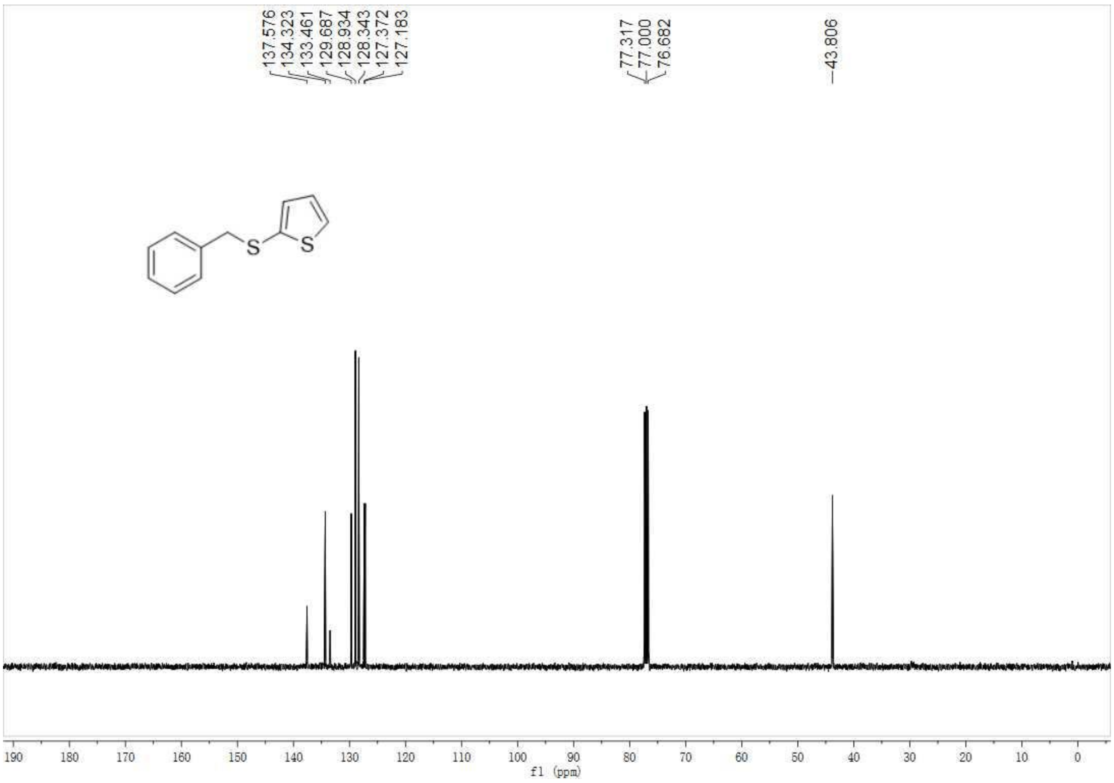

^{1}H NMR Spectrum of **272**

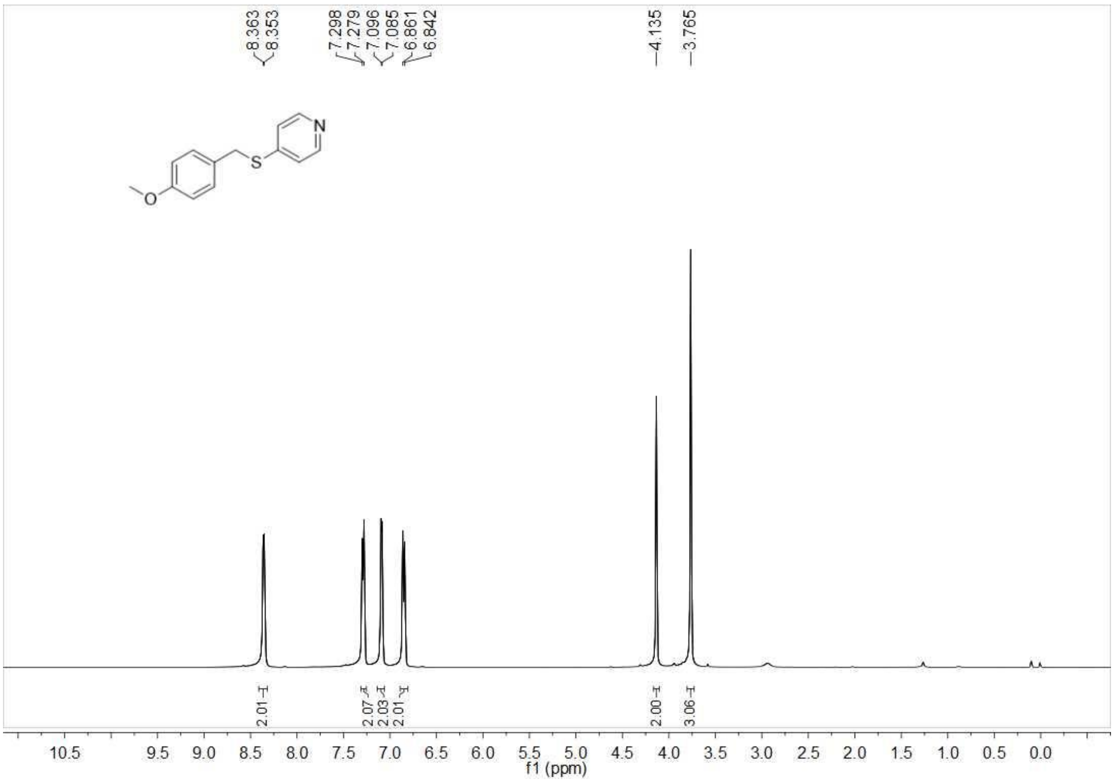

^{13}C NMR Spectrum of **272**

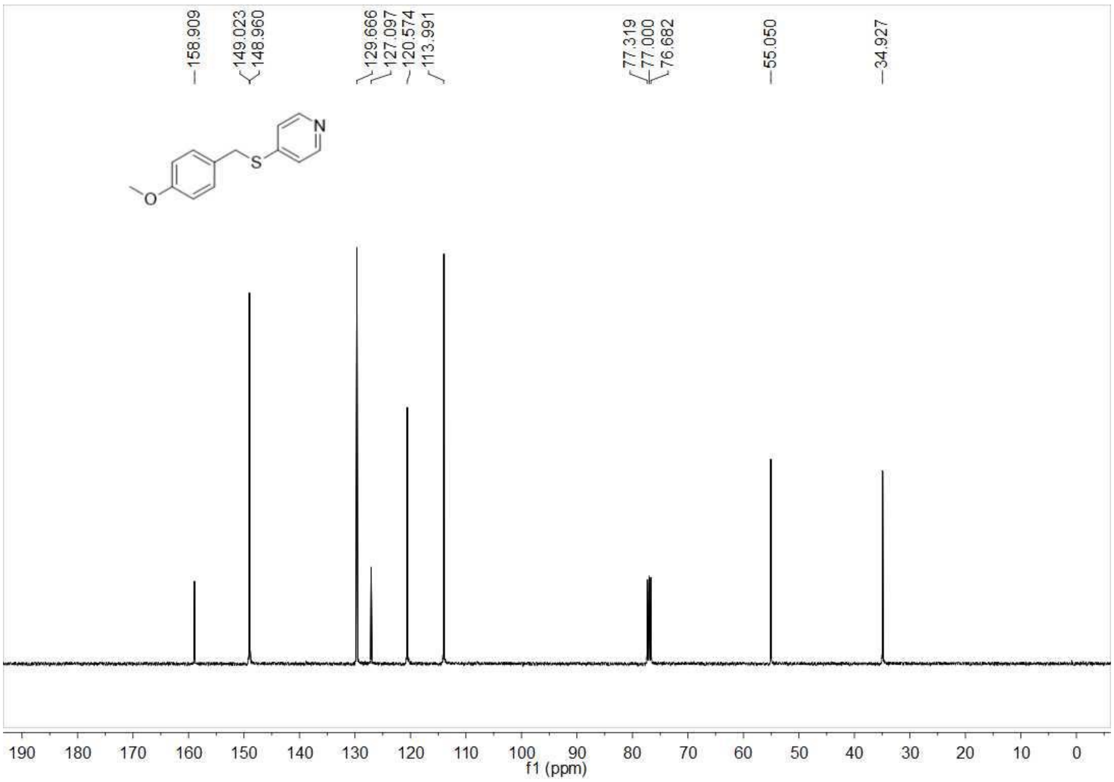

^{1}H NMR Spectrum of **273**

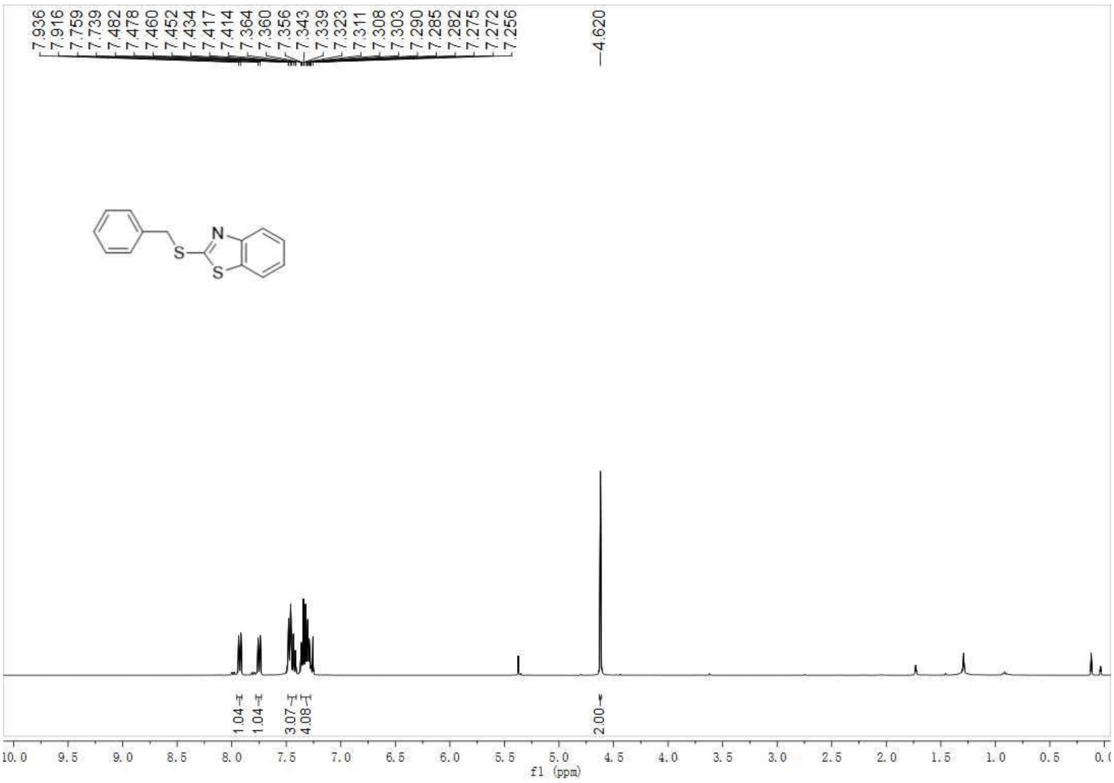

^{13}C NMR Spectrum of **273**

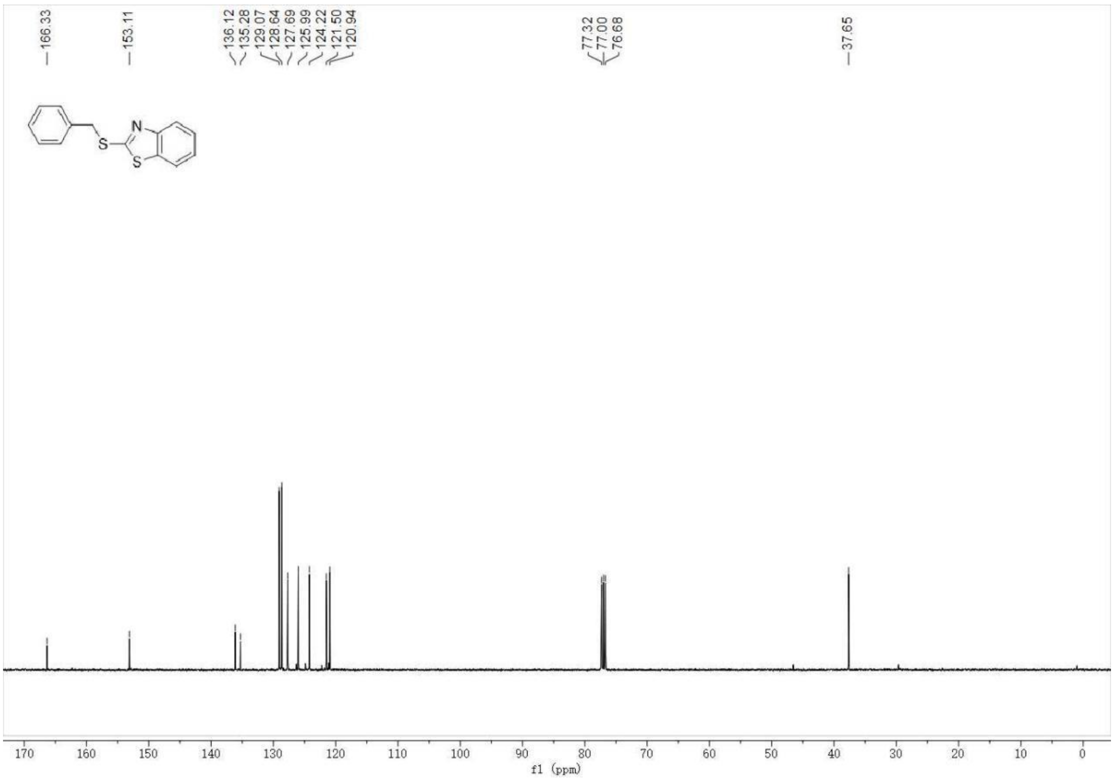

^{1}H NMR Spectrum of **274**

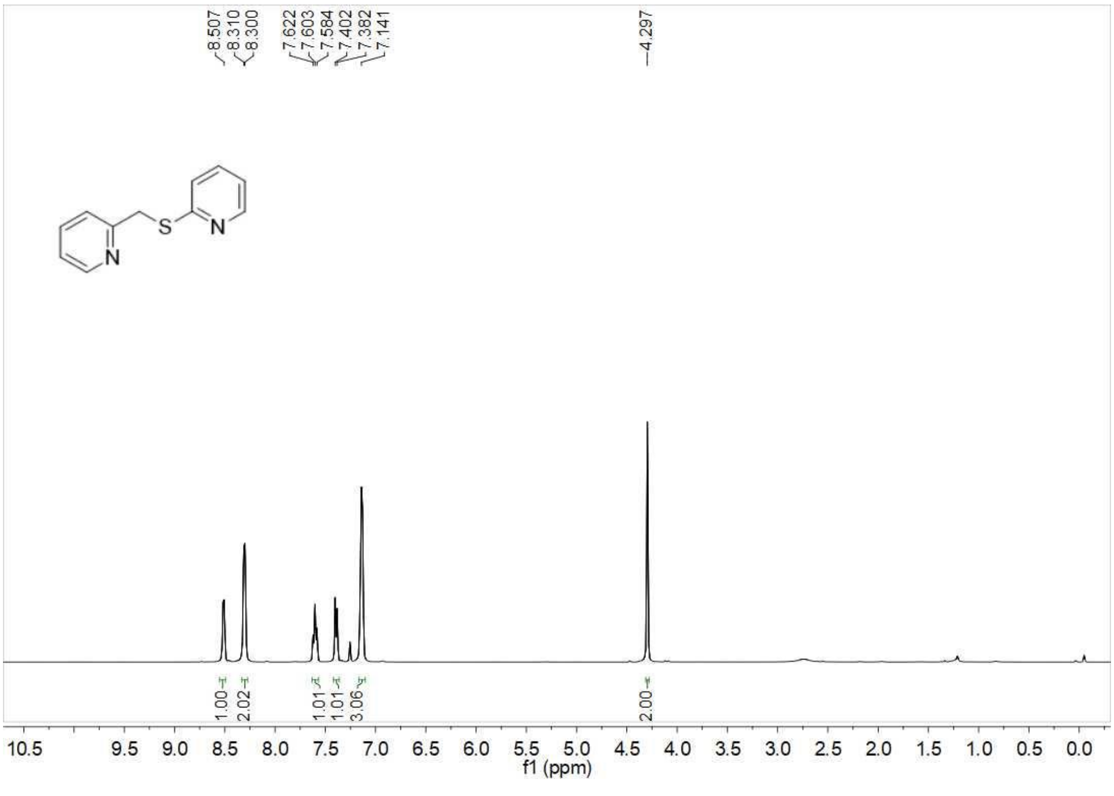

^{13}C NMR Spectrum of **274**

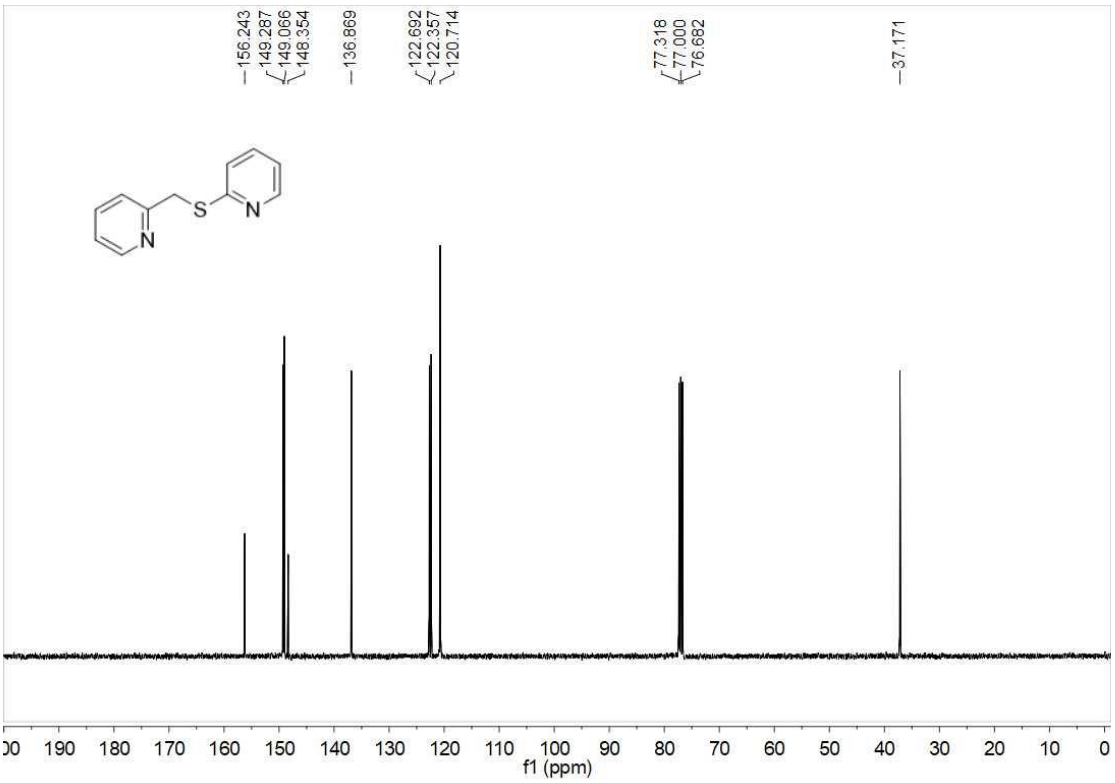

^{1}H NMR Spectrum of **275**

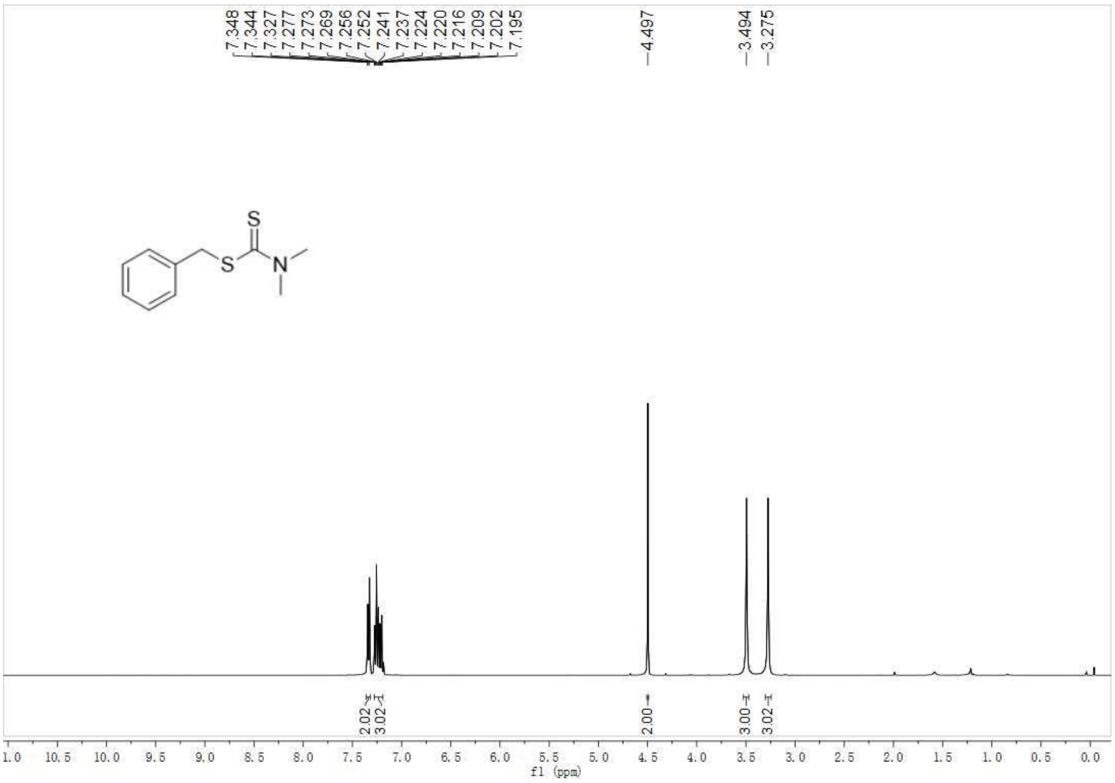

^{13}C NMR Spectrum of **275**

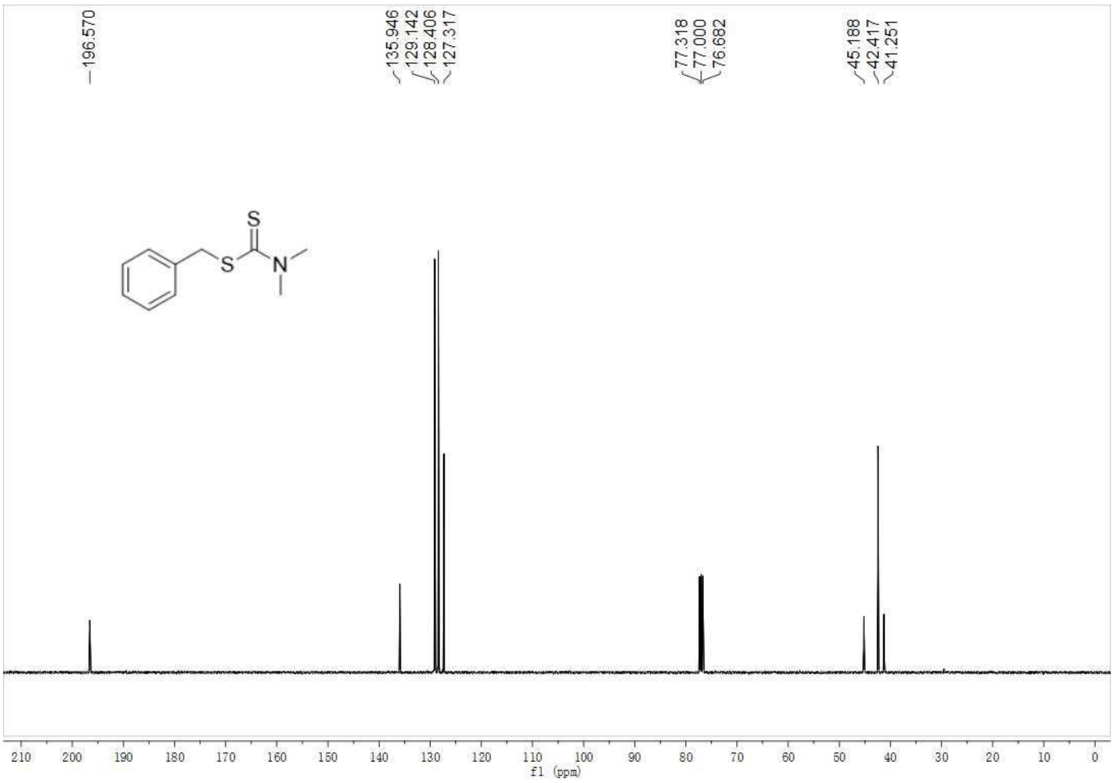

^{1}H NMR Spectrum of **276**

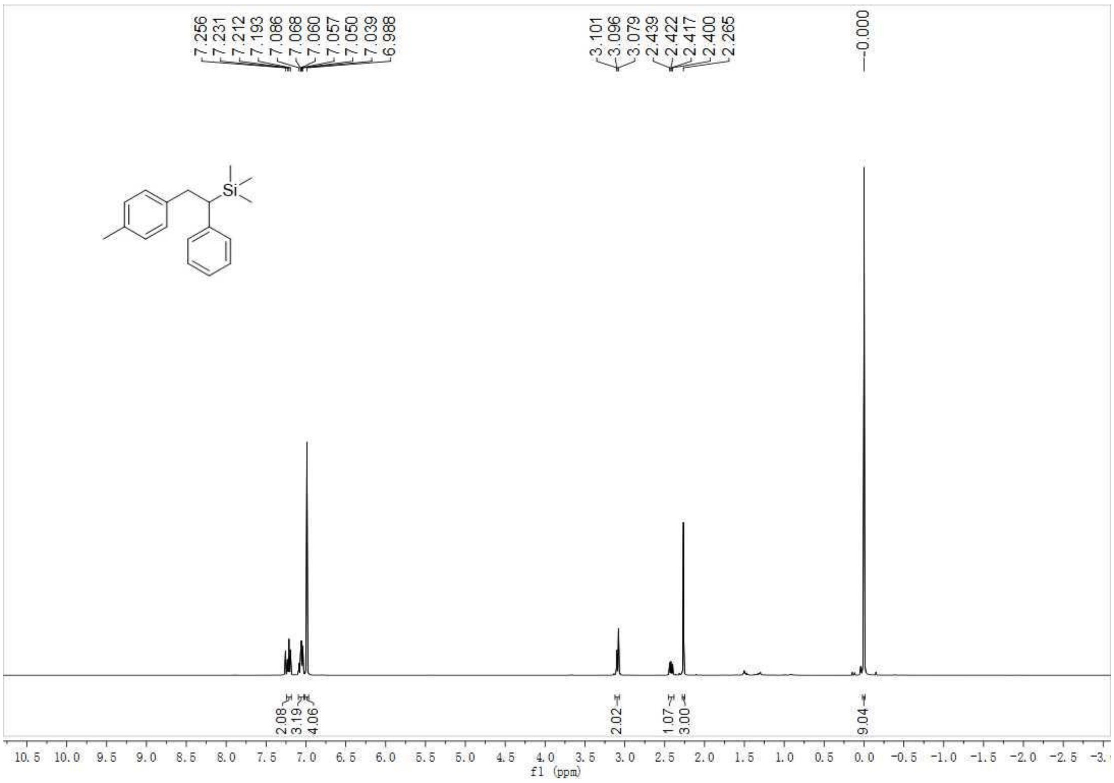

^{13}C NMR Spectrum of **276**

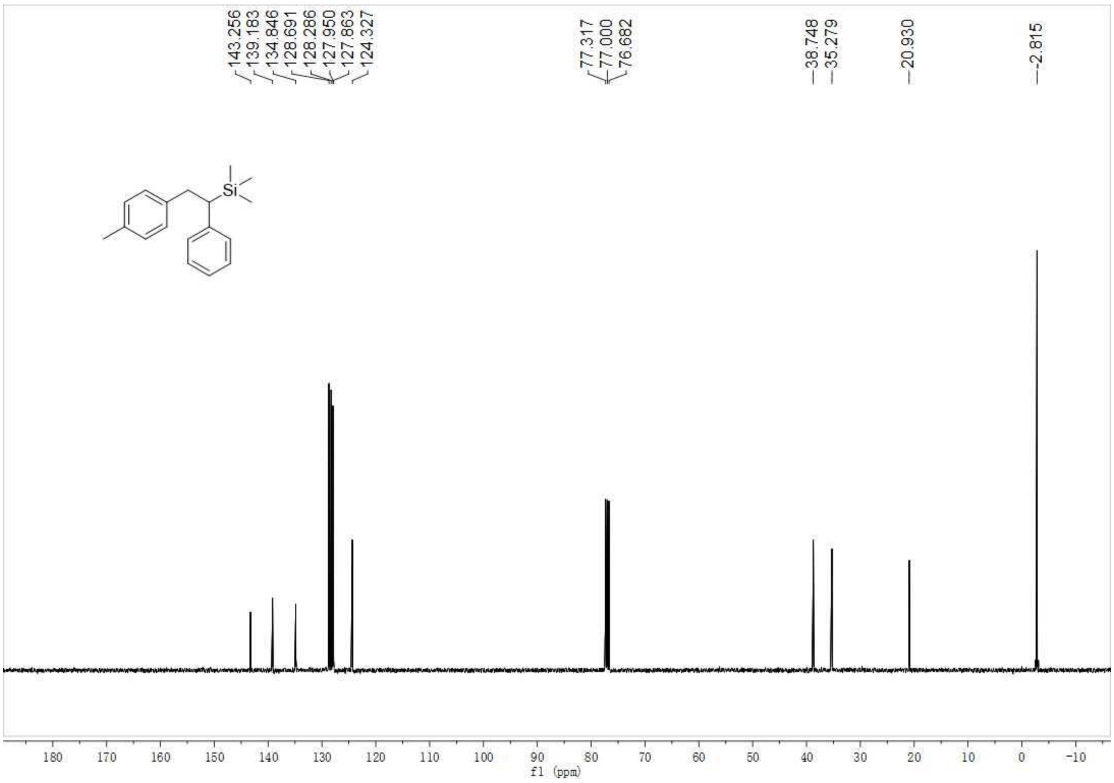

^{1}H NMR Spectrum of **277**

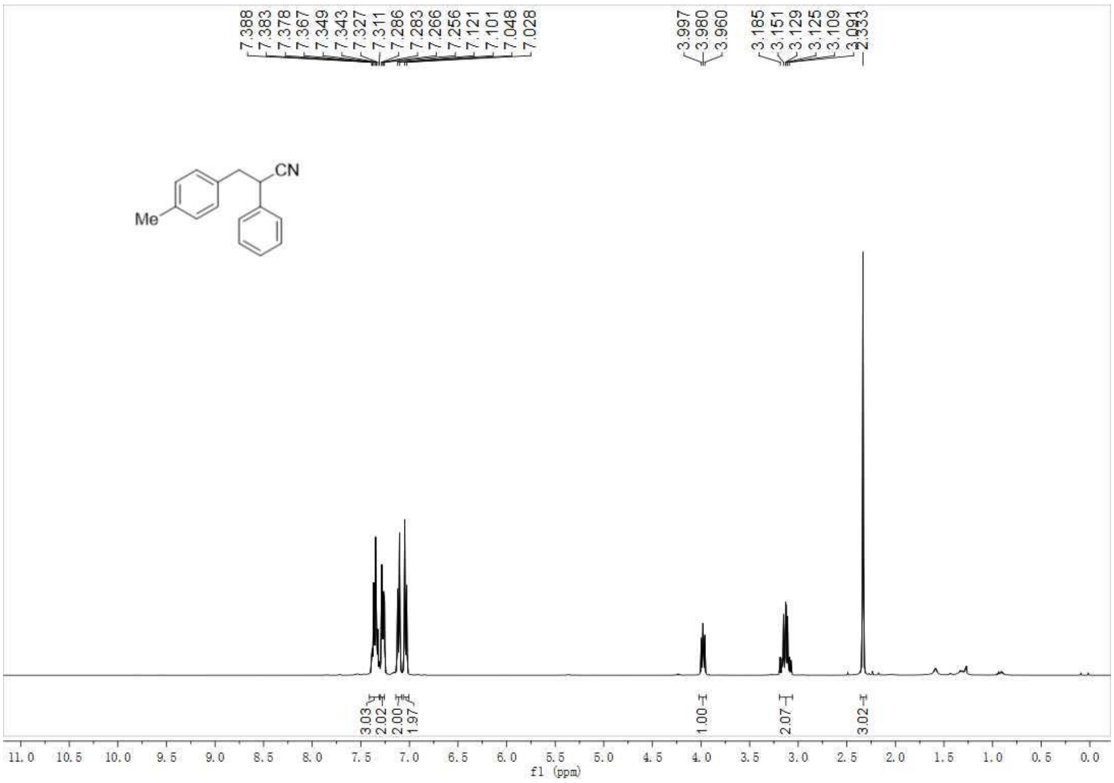

^{13}C NMR Spectrum of **277**

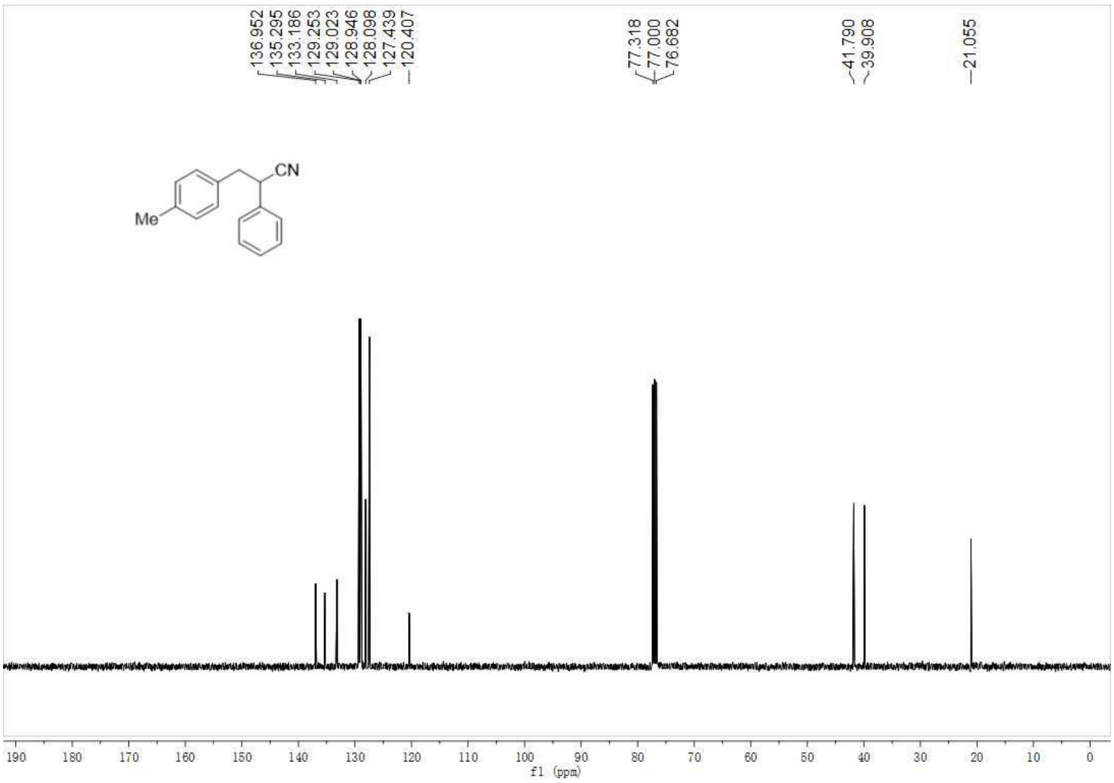

^{1}H NMR Spectrum of **278**

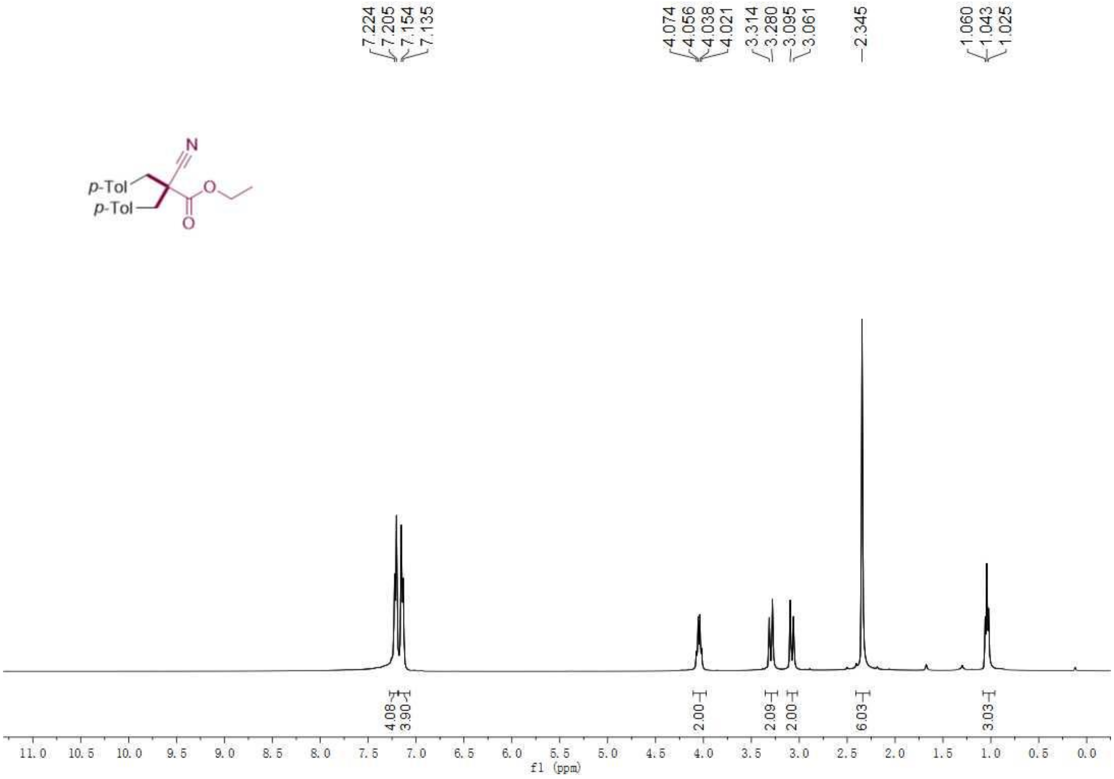

^{13}C NMR Spectrum of **278**

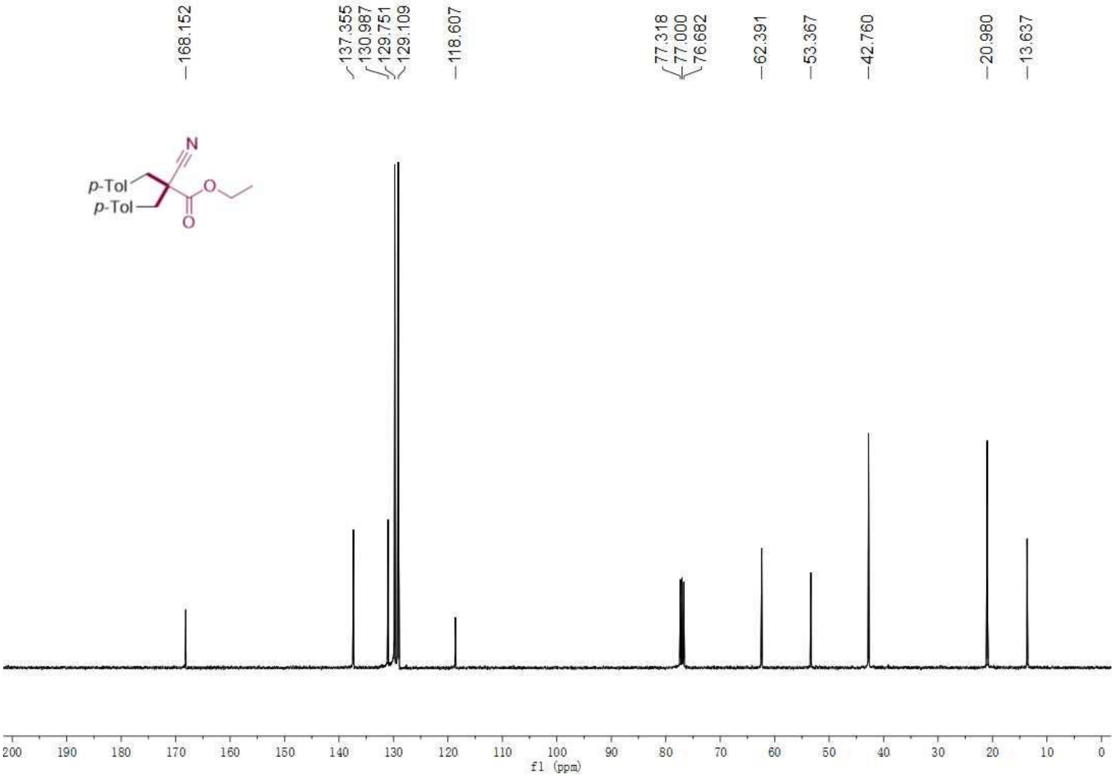

^{1}H NMR Spectrum of **279**

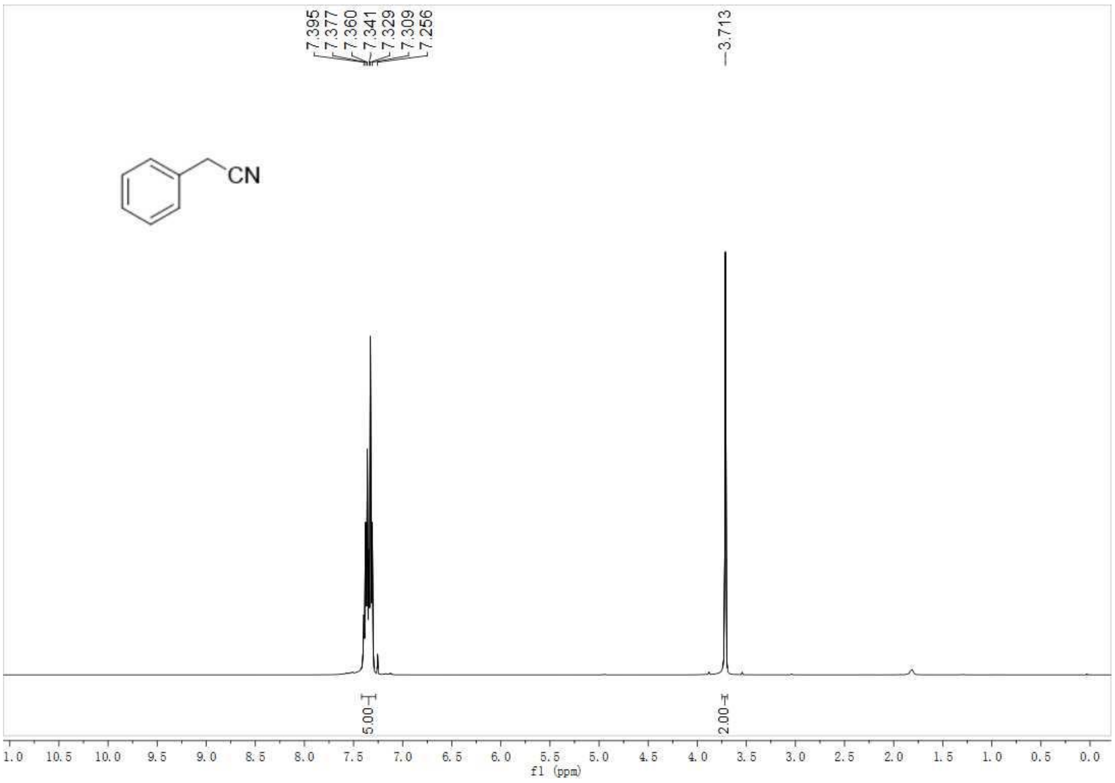

^{13}C NMR Spectrum of **279**

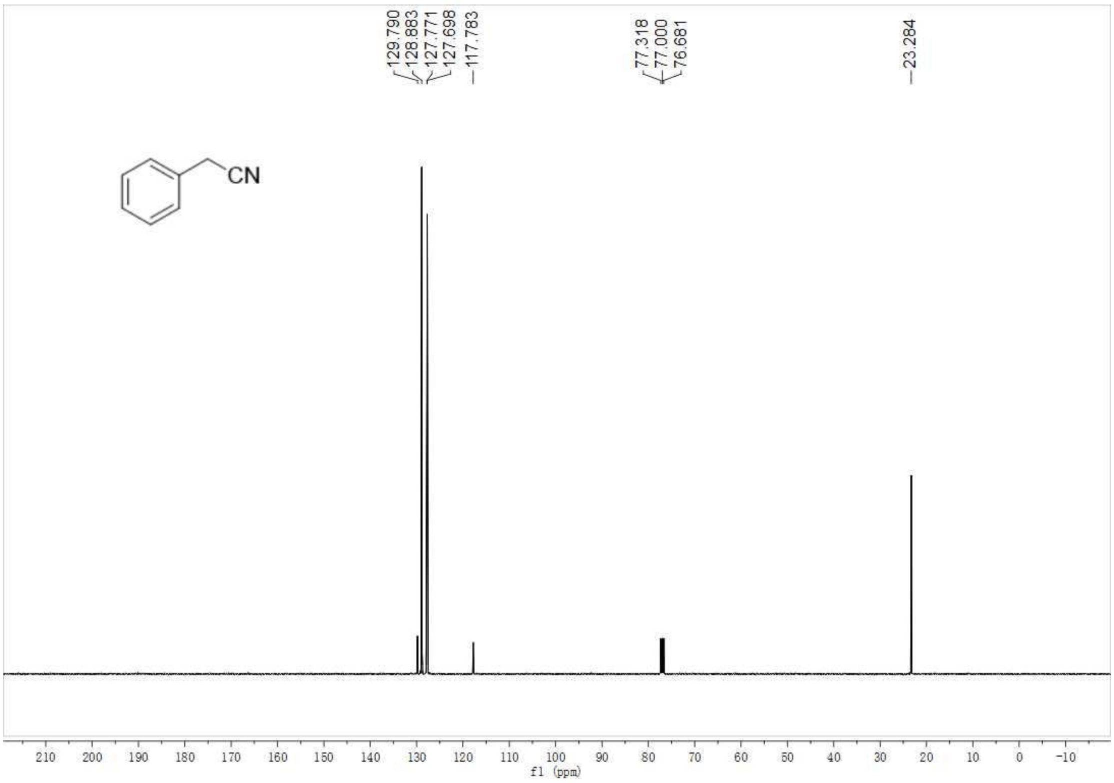

^{1}H NMR Spectrum of **280**

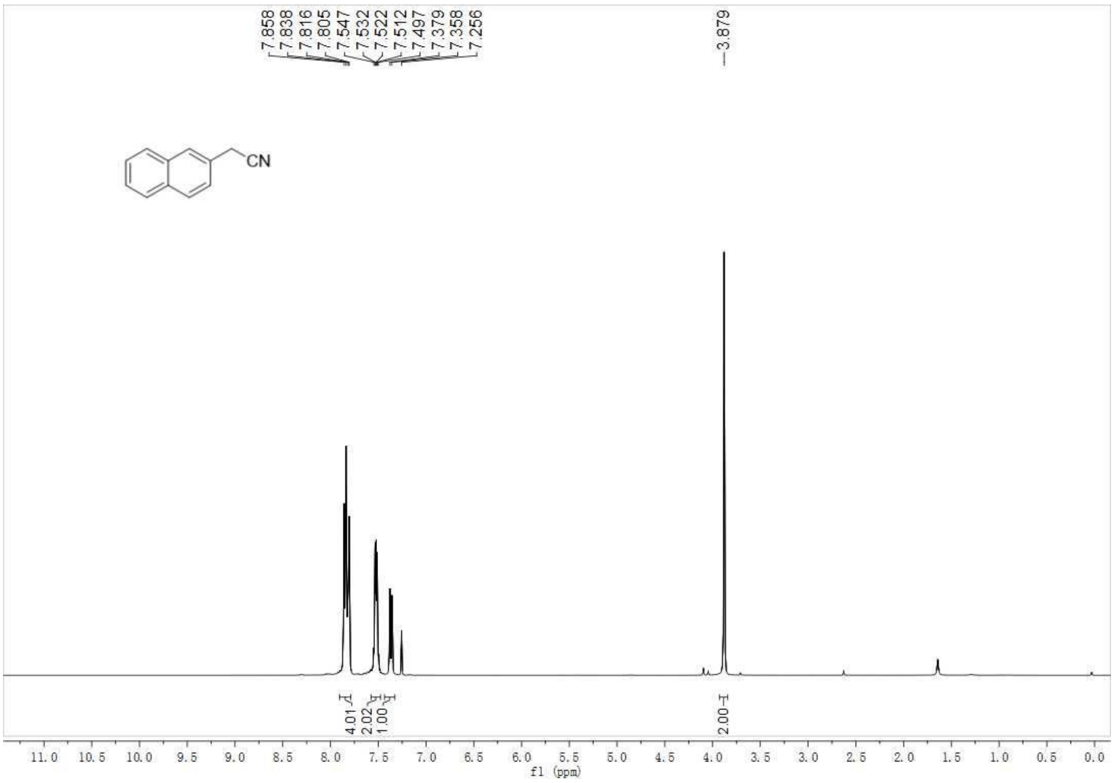

^{13}C NMR Spectrum of **280**

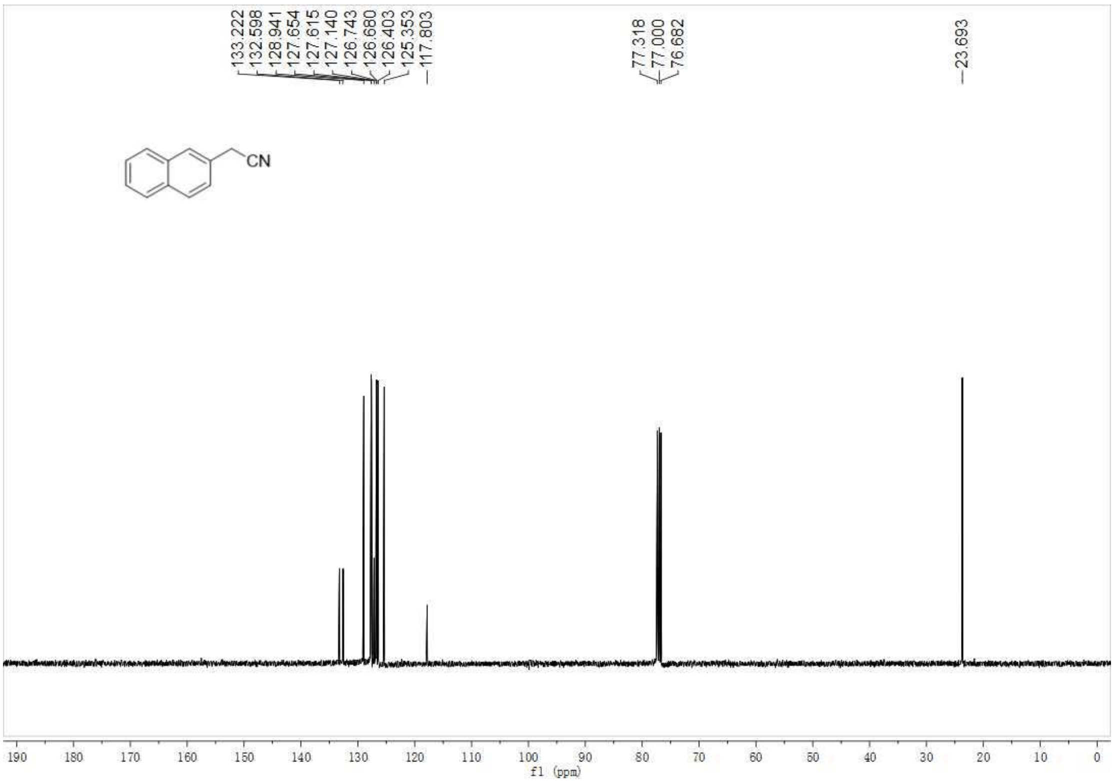

^1H NMR Spectrum of **281**

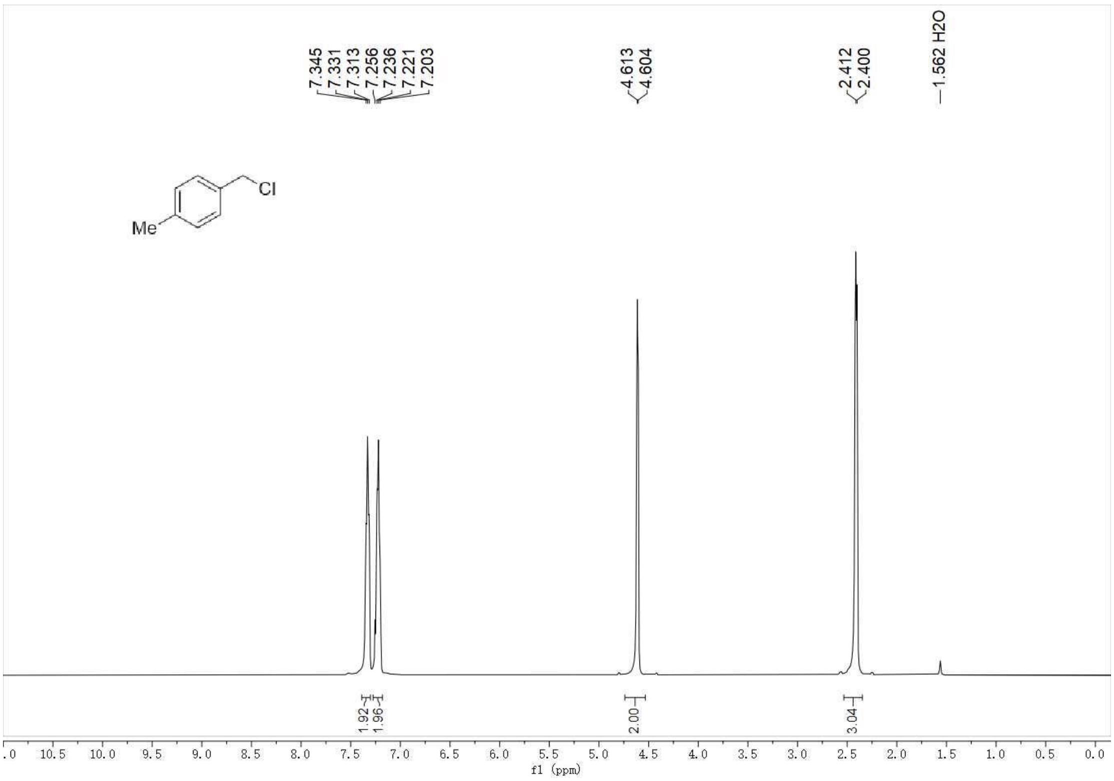

^{13}C NMR Spectrum of **281**

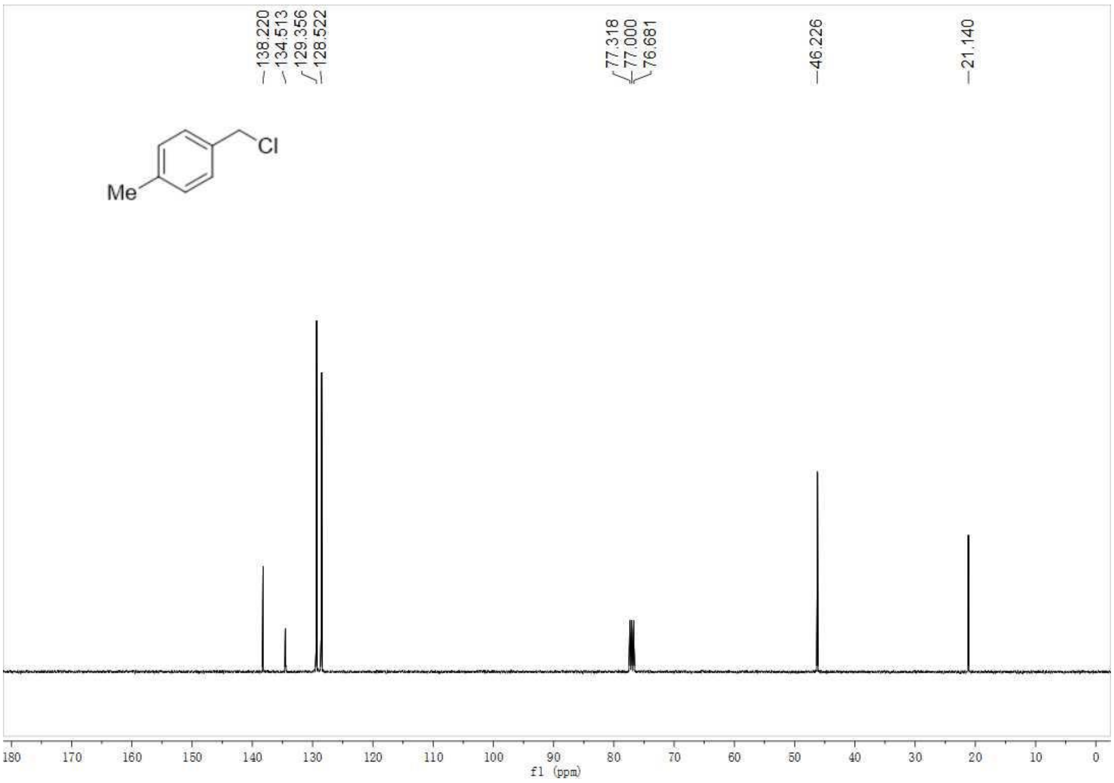

^{1}H NMR Spectrum of **282**

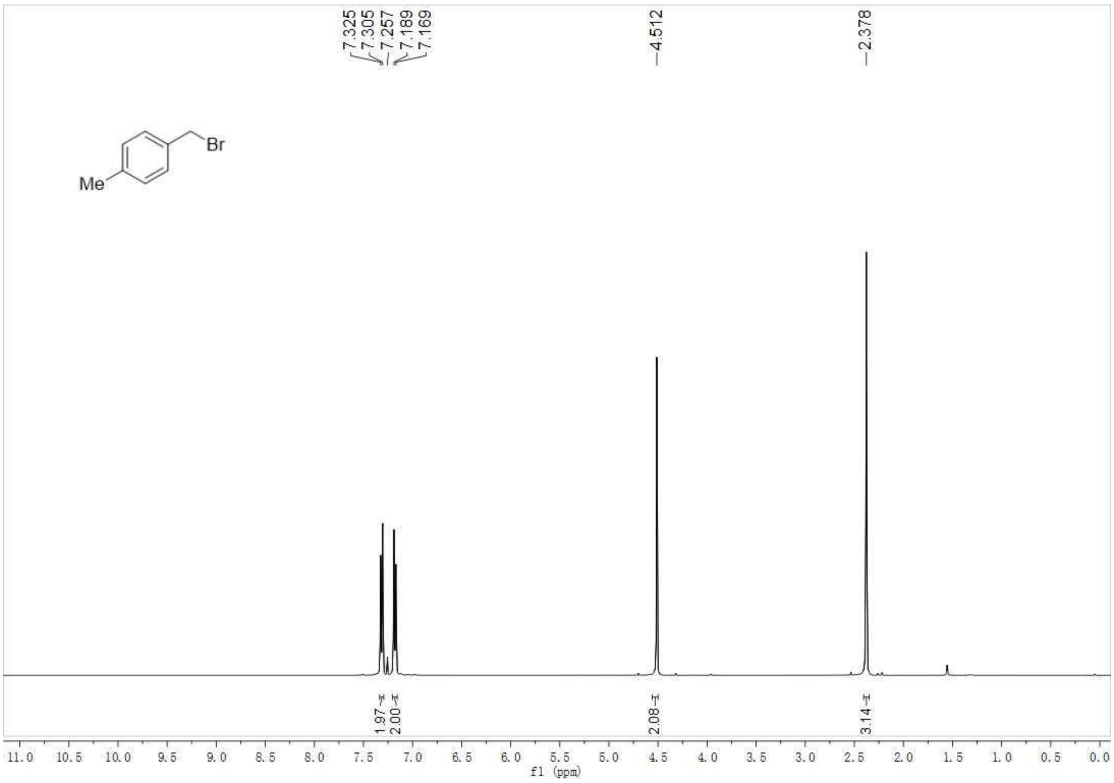

^{13}C NMR Spectrum of **282**

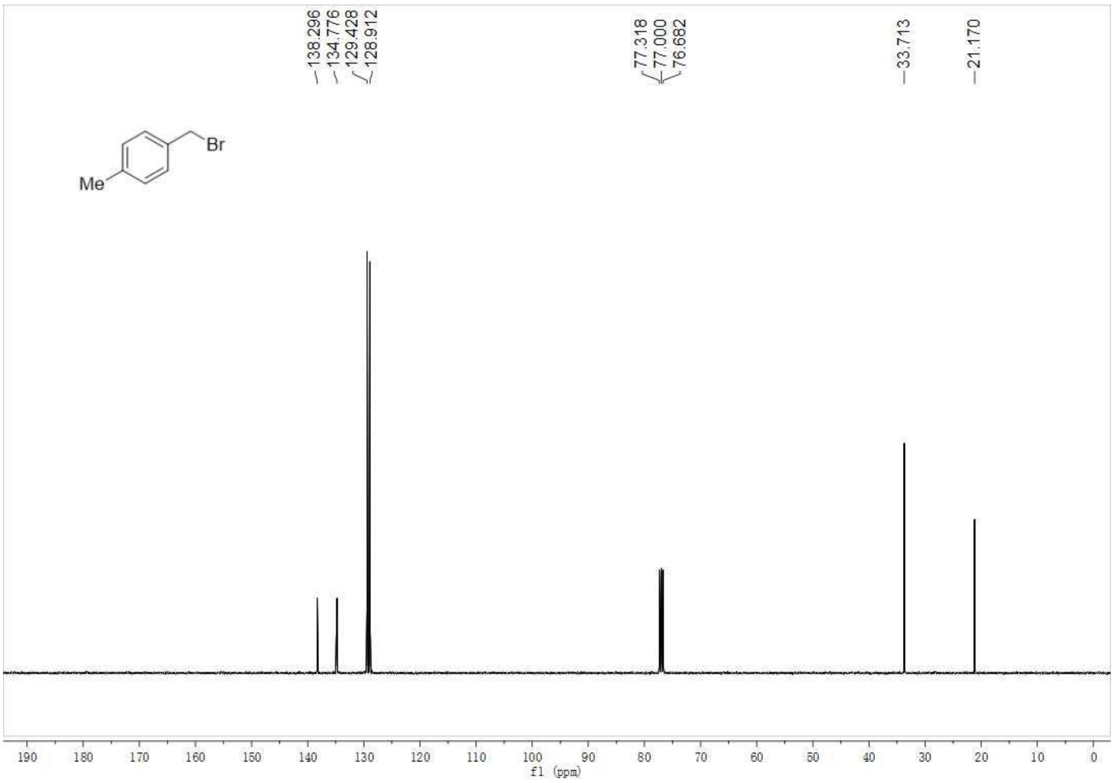

^{1}H NMR Spectrum of **283**

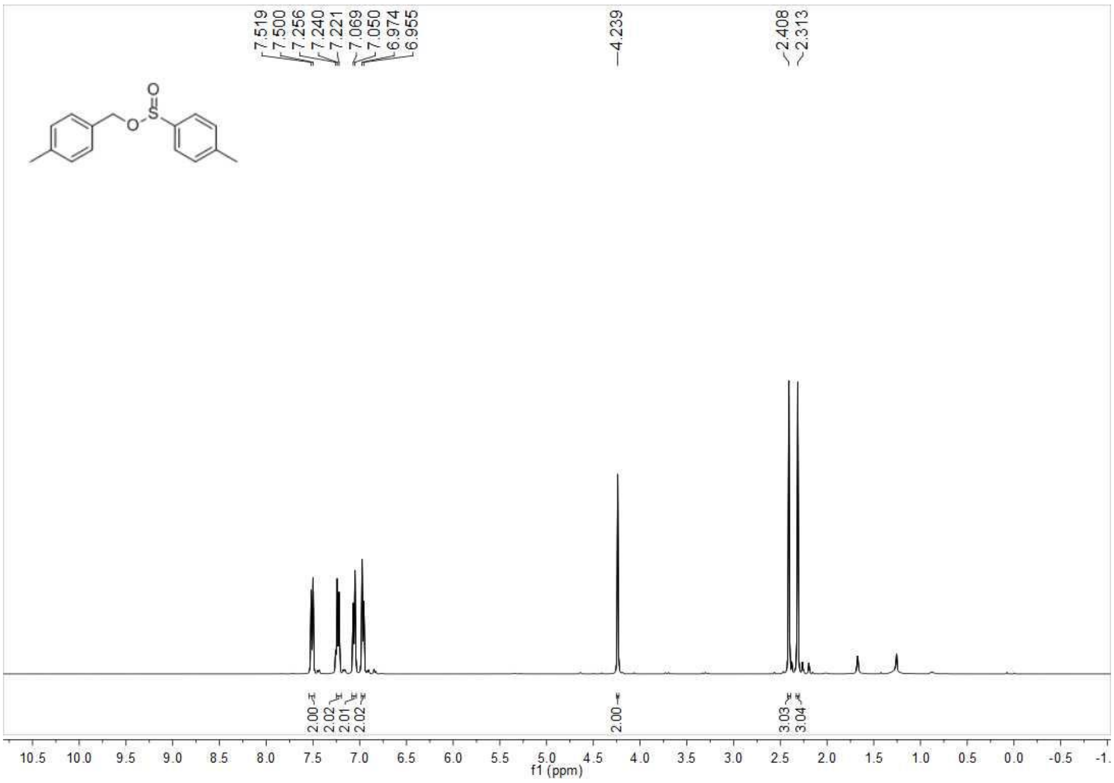

^{13}C NMR Spectrum of **283**

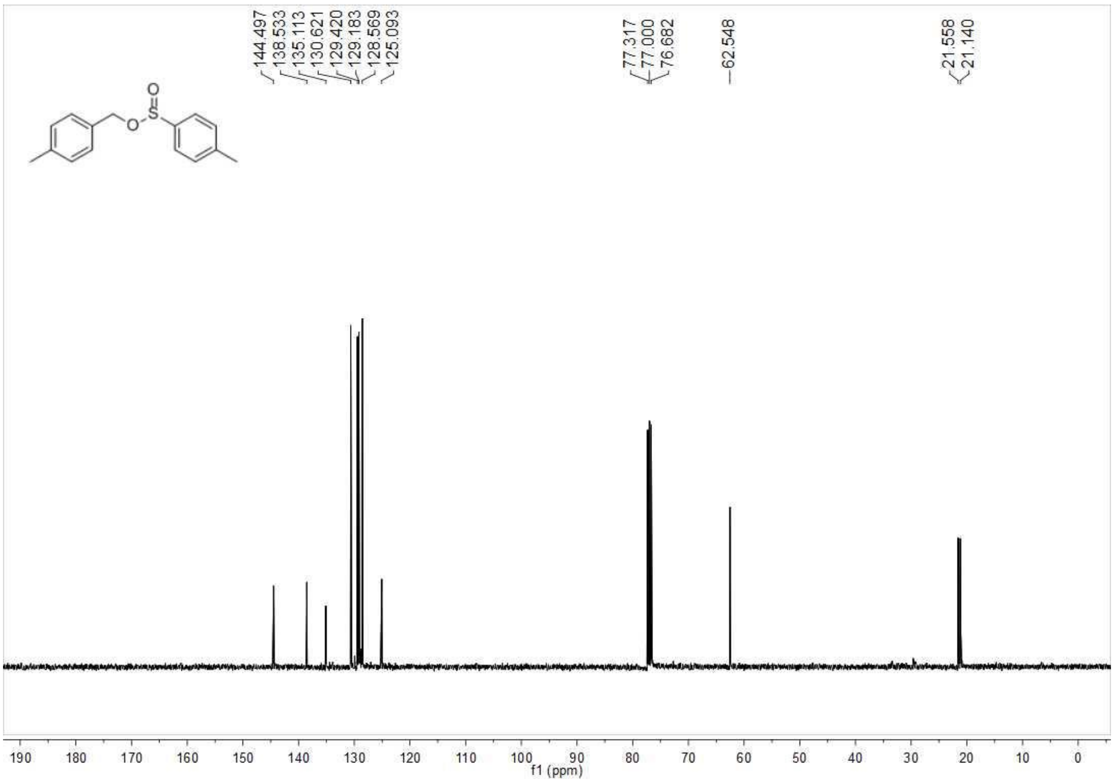

^{1}H NMR Spectrum of **284**

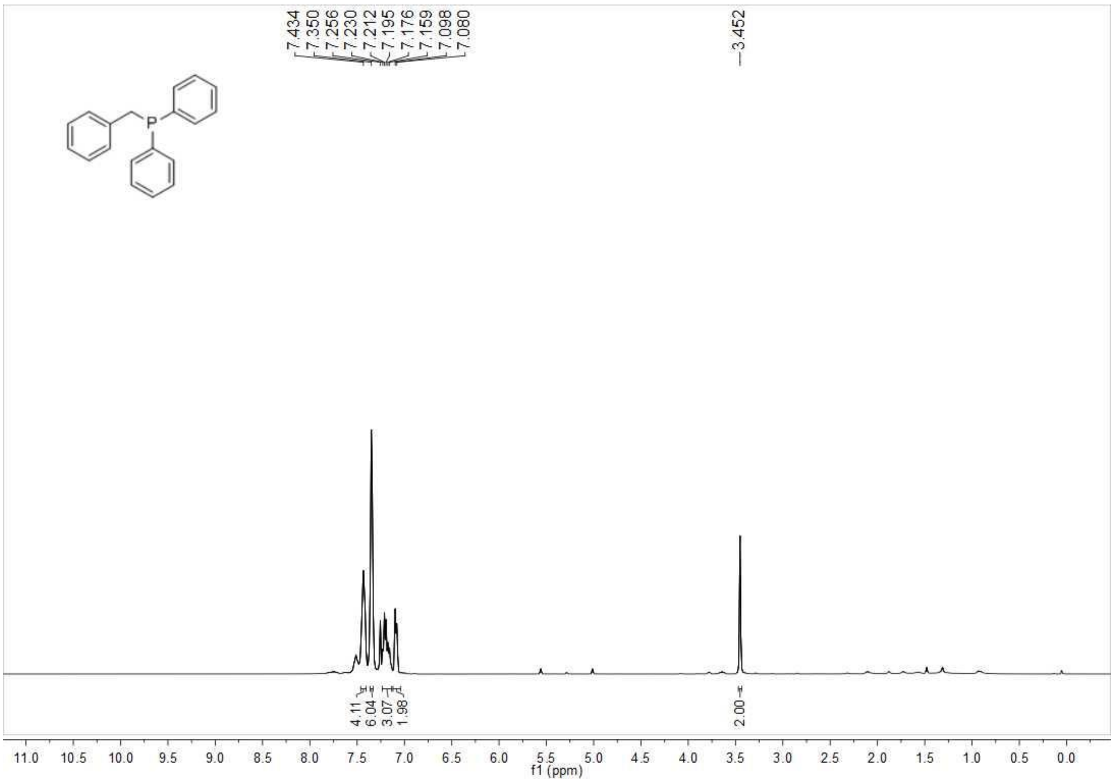

^{13}C NMR Spectrum of **284**

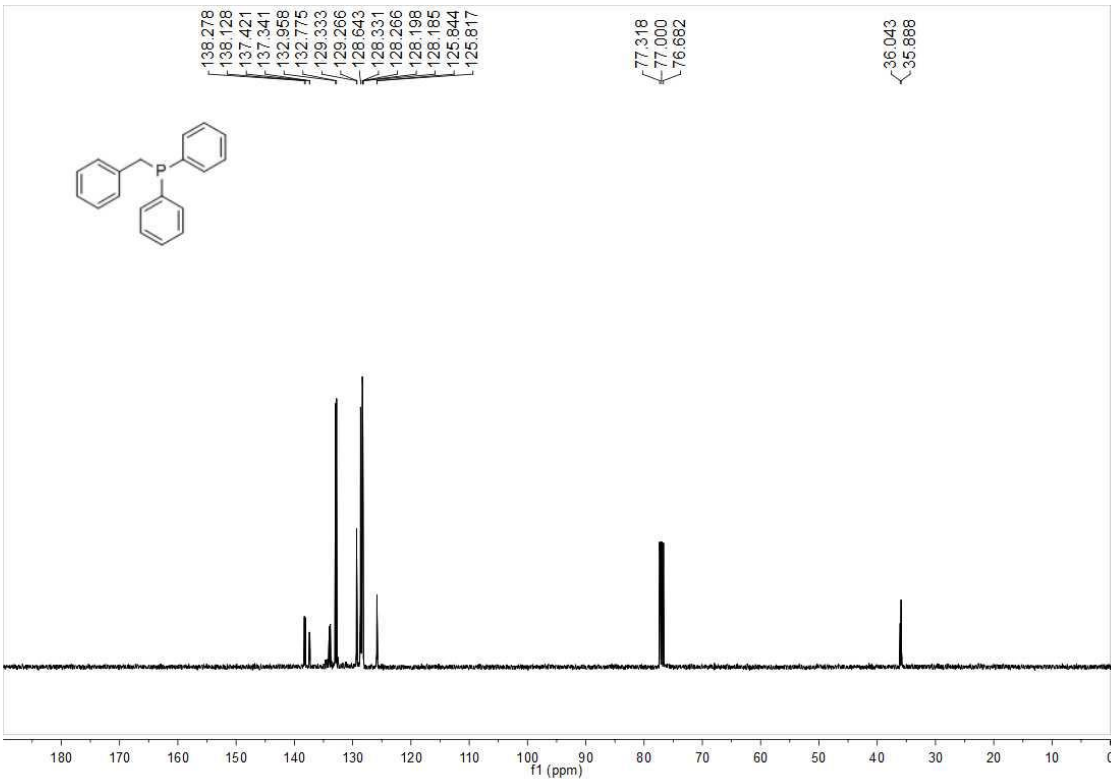

^{31}P NMR Spectrum of **284**

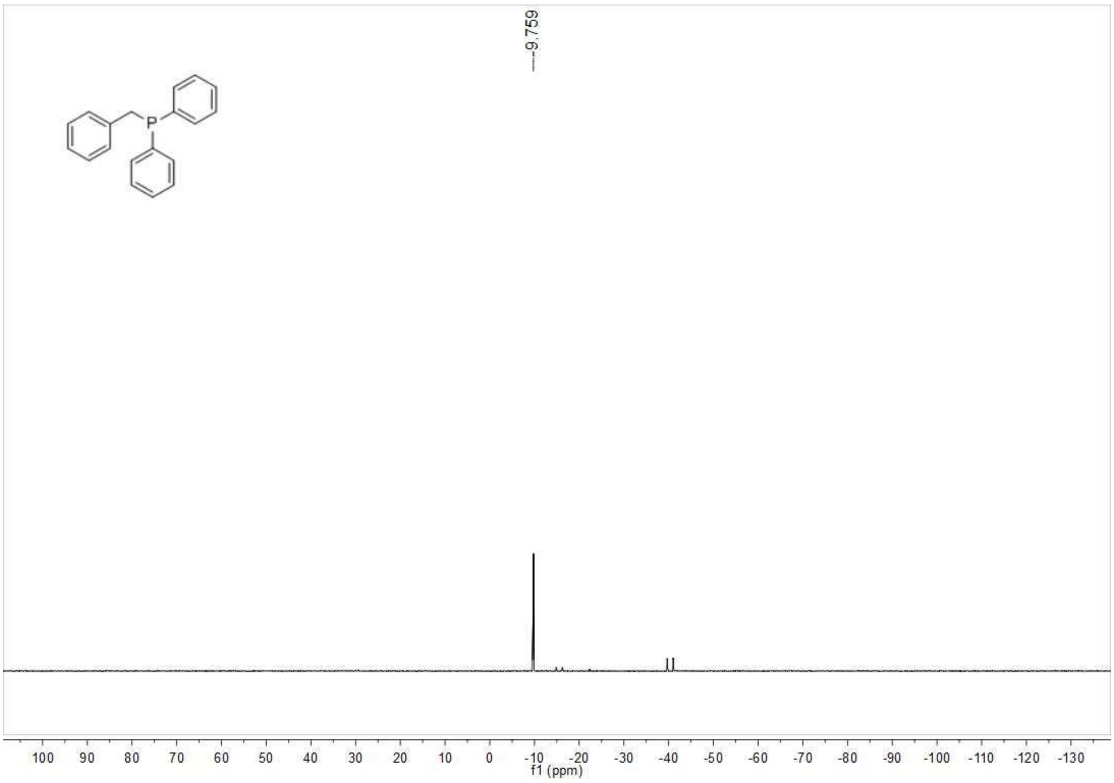

^{1}H NMR Spectrum of **285**

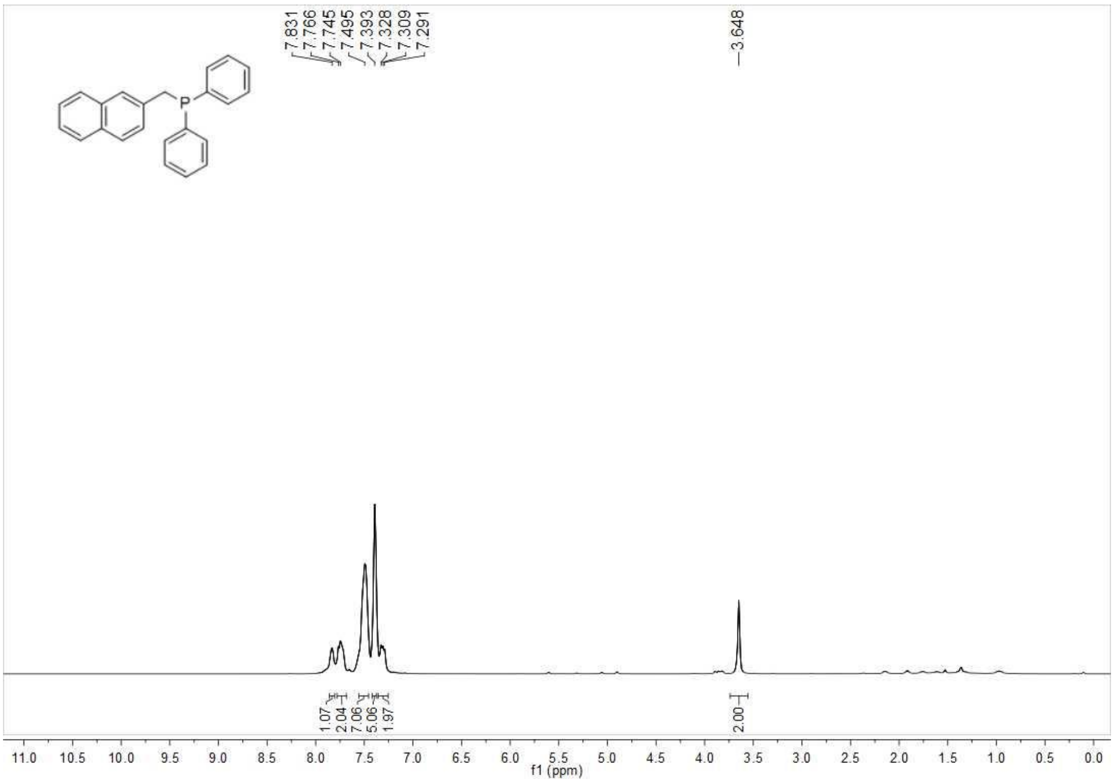

^{13}C NMR Spectrum of **285**

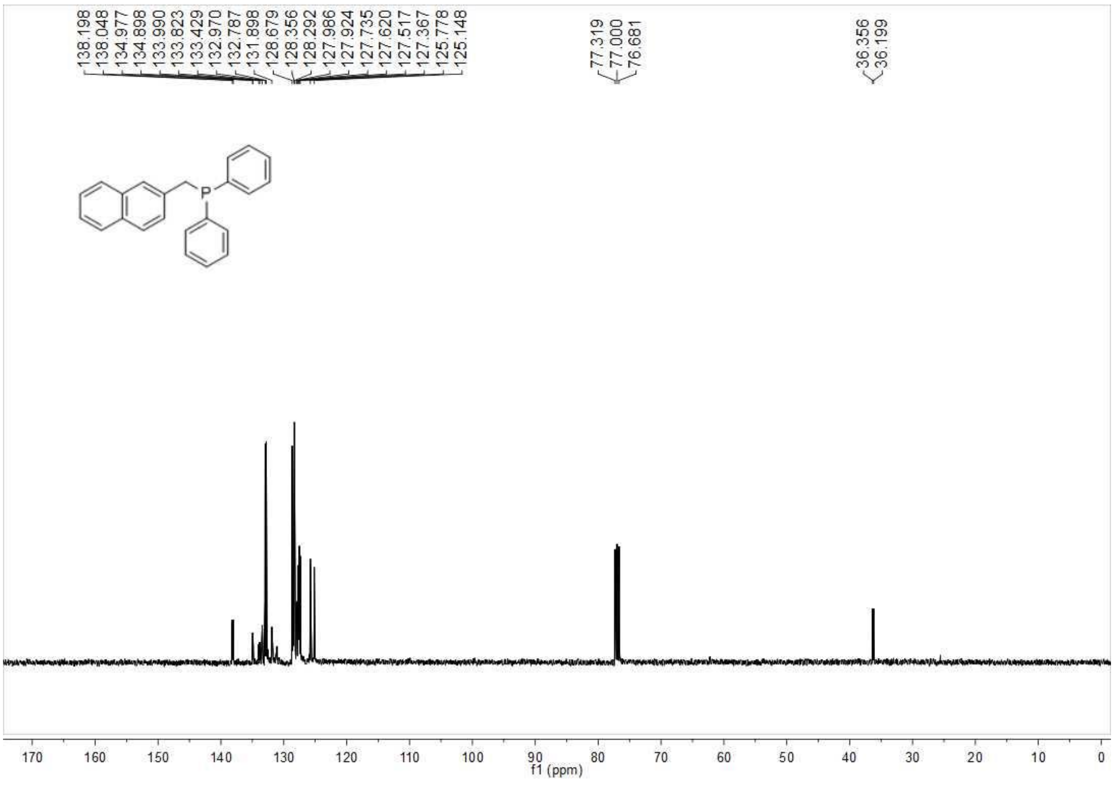

^{31}P NMR Spectrum of **285**

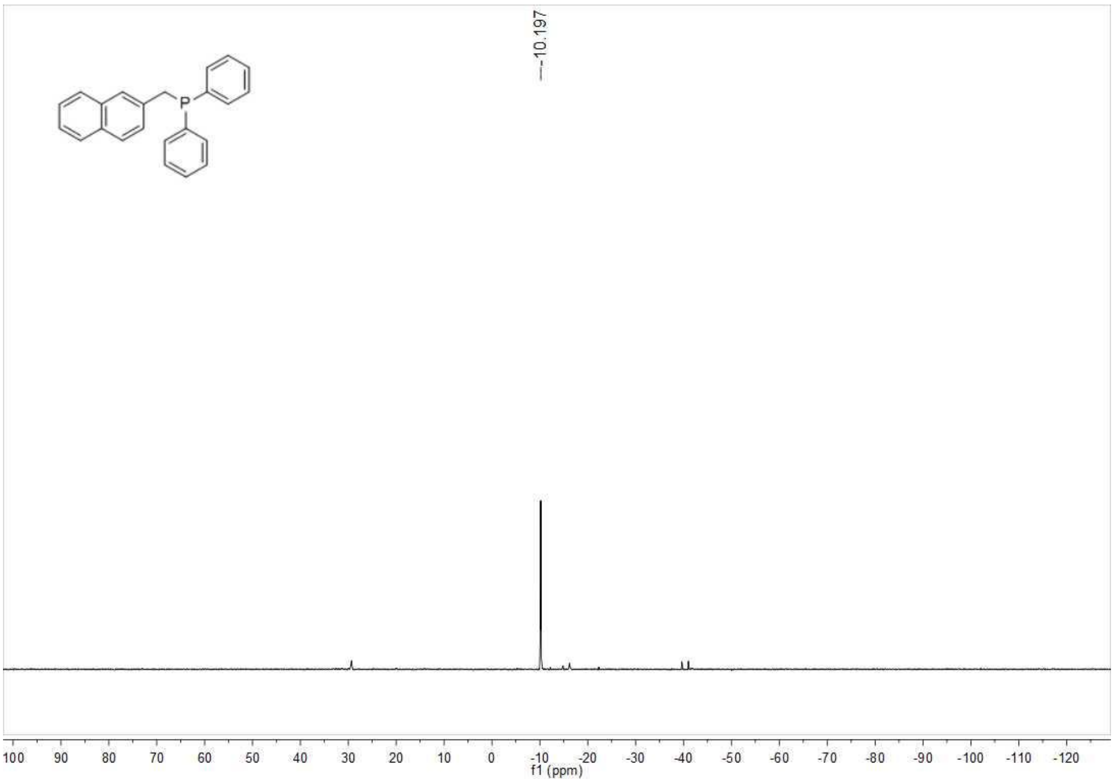

6. Chiral HPLC Traces

Chiral HPLC analysis of (*rac*)-**2B**

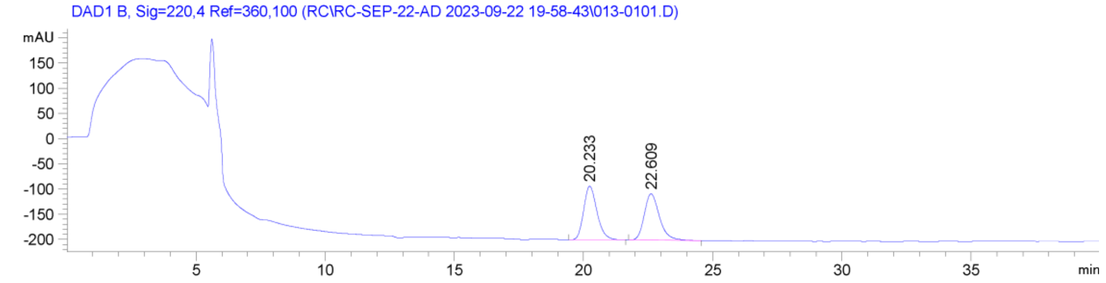

Signal 2: DAD1 B, Sig=220,4 Ref=360,100

Peak #	RetTime [min]	Type	Width [min]	Area [mAU*s]	Height [mAU]	Area %
1	20.233	BB	0.5513	3831.60303	106.60142	50.6051
2	22.609	BB	0.6224	3739.97900	91.97871	49.3949

Chiral HPLC analysis of (*R*)-**2B**

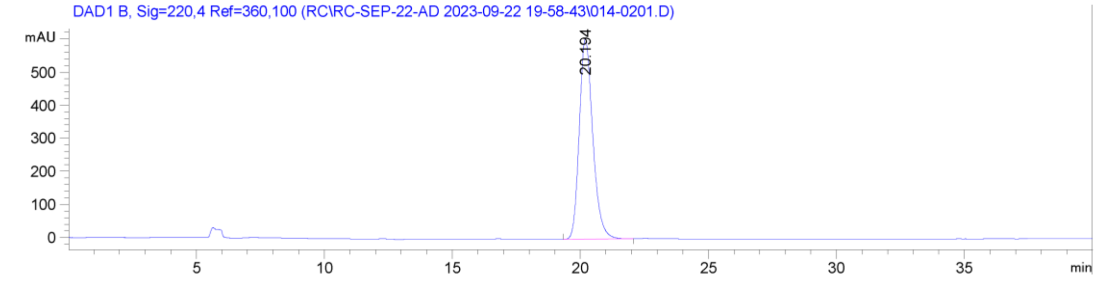

Signal 2: DAD1 B, Sig=220,4 Ref=360,100

Peak #	RetTime [min]	Type	Width [min]	Area [mAU*s]	Height [mAU]	Area %
1	20.194	BB	0.5588	2.21572e4	605.65100	100.0000

Chiral HPLC analysis of (*rac*)-**247**.

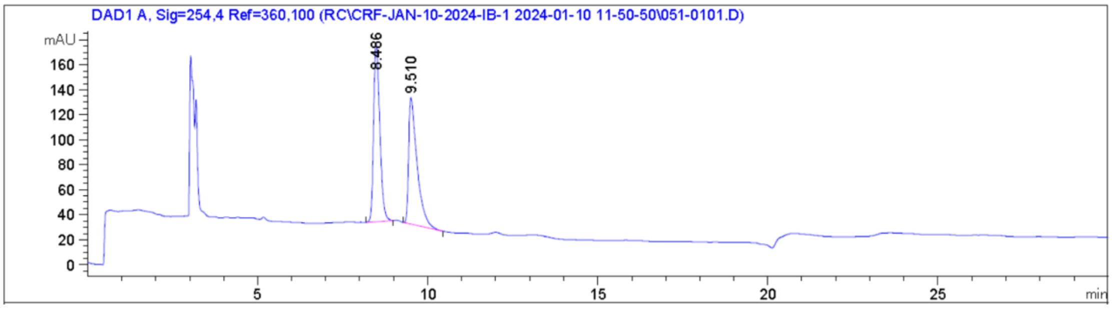

Signal 1: DAD1 A, Sig=254,4 Ref=360,100

Peak #	RetTime [min]	Type	Width [min]	Area [mAU*s]	Height [mAU]	Area %
1	8.486	BB	0.1915	1786.34204	143.21095	50.5968
2	9.510	BB	0.2491	1744.20007	101.20724	49.4032

Chiral HPLC analysis of (*S*)-**247**.

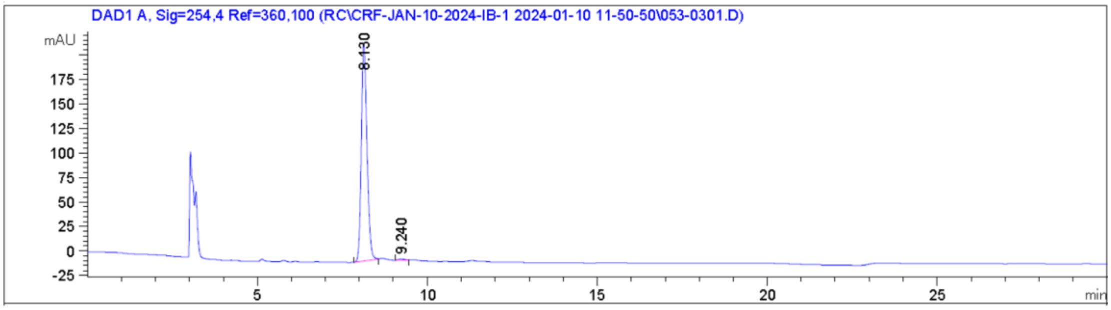

Signal 1: DAD1 A, Sig=254,4 Ref=360,100

Peak #	RetTime [min]	Type	Width [min]	Area [mAU*s]	Height [mAU]	Area %
1	8.130	BB	0.1771	2573.04565	222.27498	99.4530
2	9.240	BB	0.1815	14.15213	1.23670	0.5470

Chiral HPLC analysis of (*rac*)-**248**.

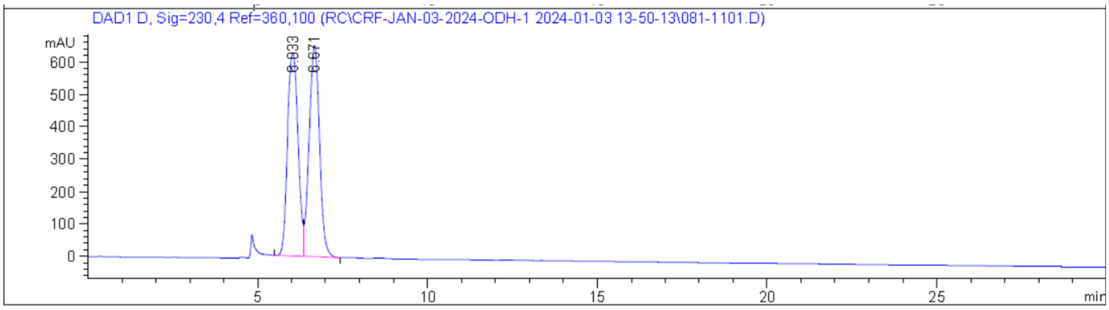

Signal 2: DAD1 D, Sig=230,4 Ref=360,100

Peak #	RetTime [min]	Type	Width [min]	Area [mAU*s]	Height [mAU]	Area %
1	6.033	BV	0.3486	1.34704e4	627.14905	49.2275
2	6.671	VB	0.3370	1.38932e4	650.91791	50.7725

Chiral HPLC analysis of (*S*)-**248**.

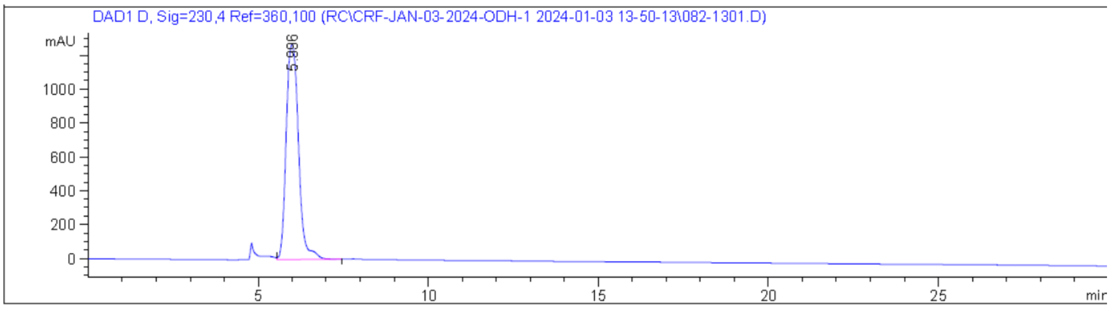

Signal 2: DAD1 D, Sig=230,4 Ref=360,100

Peak #	RetTime [min]	Type	Width [min]	Area [mAU*s]	Height [mAU]	Area %
1	5.996	VB	0.4083	3.20419e4	1270.24475	100.0000

Chiral HPLC analysis of (*rac*)-**249**.

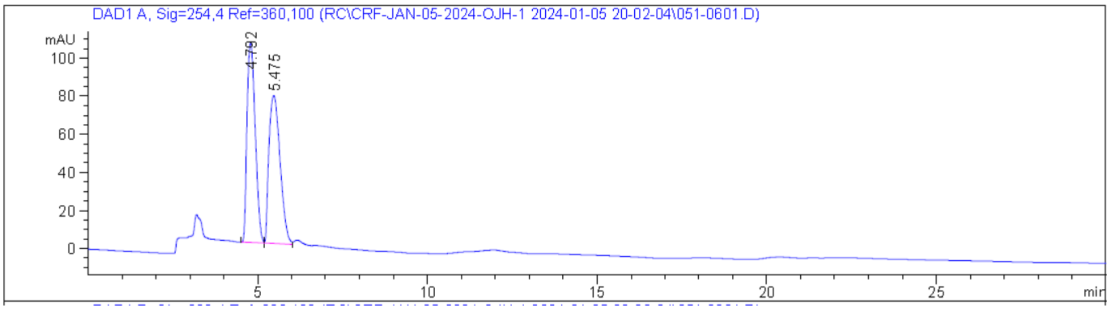

Signal 1: DAD1 A, Sig=254,4 Ref=360,100

Peak #	RetTime [min]	Type	Width [min]	Area [mAU*s]	Height [mAU]	Area %
1	4.792	BV	0.2720	1767.38940	105.18216	49.7698
2	5.475	VV	0.3675	1783.73657	77.79827	50.2302

Chiral HPLC analysis of (*S*)-**249**.

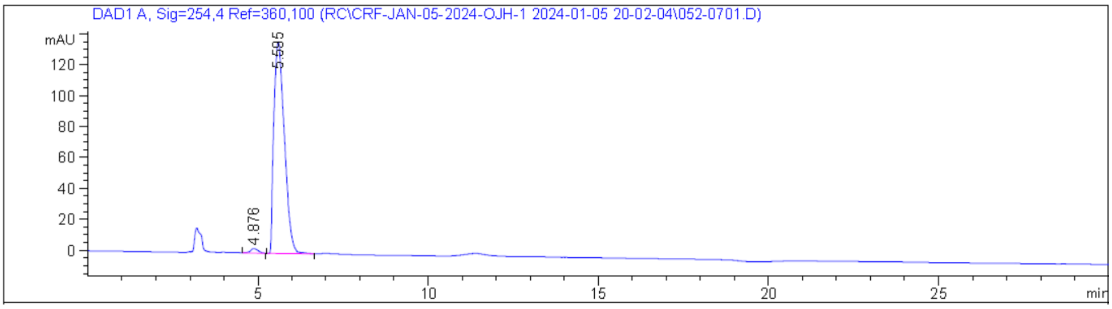

Signal 1: DAD1 A, Sig=254,4 Ref=360,100

Peak #	RetTime [min]	Type	Width [min]	Area [mAU*s]	Height [mAU]	Area %
1	4.876	BB	0.2472	45.45240	2.86382	1.5253
2	5.595	BB	0.3409	2934.50317	136.44452	98.4747

REFERENCES AND NOTES

1. F. A. Carey, R. J. Sundberg, *Advanced Organic Chemistry: Part B: Reactions and Synthesis* (Springer, 2007).

2. J. Otera, *Modern Carbonyl Chemistry* (Wiley, 2008).

3. J. Li, C.-Y. Huang, C.-J. Li, Deoxygenative functionalizations of aldehydes, ketones and carboxylic acids. *Angew. Chem. Int. Ed.* **61**, e202112770 (2022).

4. J. Barluenga, M. Tomás-Gamasa, F. Aznar, C. Valdés, Metal-free carbon–carbon bond-forming reductive coupling between boronic acids and tosylhydrazones. *Nat. Chem.* **1**, 494–499 (2009).

5. X.-J. Dai, C.-C. Li, C.-J. Li, Carbonyl Umpolung as an organometallic reagent surrogate. *Chem. Soc. Rev.* **50**, 10733–10742 (2021).

6. R. V. Jagadeesh, K. Murugesan, A. S. Alshammari, H. Neumann, M.-M. Pohl, J. Radnik, M. Beller, MOF-derived cobalt nanoparticles catalyze a general synthesis of amines. *Science* **358**, 326–332 (2017).

7. G. A. Aleku, S. P. France, H. Man, J. Mangas-Sanchez, S. L. Montgomery, M. Sharma, F. Leipold, S. Hussain, G. Grogan, N. J. Turner, A reductive aminase from *Aspergillus oryzae*. *Nat. Chem.* **9**, 961–969 (2017).

8. A. Trowbridge, D. Reich, M. J. Gaunt, Multicomponent synthesis of tertiary alkylamines by photocatalytic olefin-hydroaminoalkylation. *Nature* **561**, 522–527 (2018).

9. G. Hahn, P. Kunnas, N. de Jonge, R. Kempe, General synthesis of primary amines via reductive amination employing a reusable nickel catalyst. *Nat. Catal.* **2**, 71–77 (2019).

10. R. Kumar, N. J. Flodén, W. G. Whitehurst, M. J. Gaunt, A general carbonyl alkylative amination for tertiary amine synthesis. *Nature* **581**, 415–420 (2020).

11. S. U. Dighe, F. Juliá, A. Luridiana, J. J. Douglas, D. Leonori, A photochemical dehydrogenative strategy for aniline synthesis. *Nature* **584**, 75–81 (2020).

12. J. R. Marshall, P. Yao, S. L. Montgomery, J. D. Finnigan, T. W. Thorpe, R. B. Palmer, J. Mangas-Sanchez, R. A. M. Duncan, R. S. Heath, K. M. Graham, D. J. Cook, S. J. Charnock, N. J. Turner, Screening and characterization of a diverse panel of metagenomic imine reductases for biocatalytic reductive amination. *Nat. Chem.* **13**, 140–148 (2021).

13. T. W. Thorpe, J. R. Marshall, V. Harawa, R. E. Ruscoe, A. Cuetos, J. D. Finnigan, A. Angelastro, R. S. Heath, F. Parmeggiani, S. J. Charnock, R. M. Howard, R. Kumar, D. S. B. Daniels, G. Grogan, N. J. Turner, Multifunctional biocatalyst for conjugate reduction and reductive amination. *Nature* **604**, 86–91 (2022).

14. O. I. Afanasyev, E. Kuchuk, D. L. Usanov, D. Chusov, Reductive amination in the synthesis of pharmaceuticals. *Chem. Rev.* **119**, 11857–11911 (2019).

15. A. Trowbridge, S. M. Walton, M. J. Gaunt, New strategies for the transition-metal catalyzed synthesis of aliphatic amines. *Chem. Rev.* **120**, 2613–2692 (2020).

16. O. S. Nayal, M. S. Thakur, V. Bhatt, M. Kumar, N. Kumar, B. Singh, U. Sharma, Synthesis of tertiary arylamines: Lewis acid-catalyzed direct reductive *N*-alkylation of secondary amines with ketones through an alternative pathway. *Chem. Commun.* **52**, 9648–9651 (2016).

17. M. S. Thakur, O. S. Nayal, R. Upadhyay, N. Kumar, S. K. Maurya, 2-aminoquinazolin-4(*3H*)-one as an organocatalyst for the synthesis of tertiary amines. *Org. Lett.* **20**, 1359–1362 (2018).

18. B. Liu, Y. Li, Q. Liu, Cobalt/Lewis acid cooperative catalysis for reductive etherification of ketones and aldehydes with alcohols. *Chem. Catal.* **2**, 883–897 (2022).

19. C. Zhao, C. A. Sojdak, W. Myint, D. Seidel, Reductive etherification via anion-binding catalysis. *J. Am. Chem. Soc.* **139**, 10224–10227 (2017).

20. C. Lluna-Galán, L. Izquierdo-Aranda, R. Adam, J. R. Cabrero-Antonino, Catalytic reductive alcohol etherifications with carbonyl-based compounds or CO_2 and related transformations for the synthesis of ether derivatives. *ChemSusChem* **14**, 3744–3784 (2021).

21. L-D. Quin, *A Guide to Organophosphorus Chemistry* (Wiley-Interscience, 2000).

22. P. J. Murphy, *Organophosphorus Reagents* (Oxford Univ. Press, 2004).

23. P. A. Byrne, D. G. Gilheany, The modern interpretation of the Wittig reaction mechanism. *Chem. Soc. Rev.* **42**, 6670–6696 (2013).

24. K. C. K. Swamy, N. N. B. Kumar, E. Balaraman, K. V. P. P. Kumar, Mitsunobu and related reactions: Advances and applications. *Chem. Rev.* **109**, 2551–2651 (2009).

25. H. Guo, Y. C. Fan, Z. Sun, Y. Wu, O. Kwon, Phosphine organocatalysis. *Chem. Rev.* **118**, 10049–10293 (2018).

26. M. C. Hilton, X. Zhang, B. T. Boyle, J. V. Alegre-Requena, R. S. Paton, A. McNally, Heterobiaryl synthesis by contractive C–C coupling via P(V) intermediates. *Science* **362**, 799–804 (2018).

27. X. Zhang, K. G. Nottingham, C. Patel, J. V. Alegre-Requena, J. N. Levy, R. S. Paton, A. McNally, Phosphorus-mediated sp^2–sp^3 couplings for C–H fluoroalkylation of azines. *Nature* **594**, 217–222 (2021).

28. E. J. Miller, W. Zhao, J. D. Herr, A. T. Radosevich, A nonmetal approach to α-heterofunctionalized carbonyl derivatives by formal reductive X-H insertion. *Angew. Chem. Int. Ed.* **51**, 10605–10609 (2012).

29. V. Liepins, A. S. E. Karlström, J.-E. Bäckvall, Allylic phosphates and allylic phosphinates as electrophiles in efficient silylcupration reactions of acetylenes. *J. Org. Chem.* **67**, 2136–2143 (2002).

30. J.-B. Langlois, A. Alexakis, Dynamic kinetic asymmetric transformation in copper catalyzed allylic alkylation. *Chem. Commun.* , 3868–3870 (2009).

31. M. B. Kurosawa, K. Kato, K. Muto, J. Yamaguchi, Unified synthesis of multiply arylated alkanes by catalytic deoxygenative transformation of diarylketones. *Chem. Sci.* **13**, 10743–10751 (2022).

32. Y. Qian, Q. Dai, Z. Li, Y. Liu, J. Zhang, *O*-Phosphination of aldehydes/ketones toward phosphoric esters: Experimental and mechanistic studies. *Org. Lett.* **22**, 4742–4748 (2020).

33. R. Vardanyan, *Piperidine-Based Drug Discovery* (Elsevier, 2017).

34. E. Peris, R. H. Crabtree, Key factors in pincer ligand design. *Chem. Soc. Rev.* **47**, 1959–1968 (2018).

35. DrugBank. Drugs with Arylmethyl and Heteroarylmethyl Tertiary Amine Units. https://go.drugbank.com (accessed 15 January, 2025).

36. J. T. Edwards, R. R. Merchant, K. S. McClymont, K. W. Knouse, T. Qin, L. R. Malins, B. Vokits, S. A. Shaw, D.-H. Bao, F.-L. Wei, T. Zhou, M. D. Eastgate, P. S. Baran, Decarboxylative alkenylation. *Nature* **545**, 213–218 (2017).

37. V. G. Chandrashekhar, W. Baumann, M. Beller, R. V. Jagadeesh, Nickel-catalyzed hydrogenative coupling of nitriles and amines for general amine synthesis. *Science* **376**, 1433–1441 (2022).

38. S. Z. Ali, B. G. Budaitis, D. F. A. Fontaine, A. L. Pace, J. A. Garwin, M. C. White, Allylic C–H amination cross-coupling furnishes tertiary amines by electrophilic metal catalysis. *Science* **376**, 276–283 (2022).

39. U. S. Larsen, M. Begtrup, L. Martiny, Tritium labelling of bromhexine via amide reduction with LiB_3H_4. *J. Label. Compd. Radiopharm.* **48**, 429–434 (2005).

40. D. C. Blakemore, L. Castro, I. Churcher, D. C. Rees, A. W. Thomas, D. M. Wilson, A. Wood, Organic synthesis provides opportunities to transform drug discovery. *Nat. Chem.* **10**, 383–394 (2018).

41. K. R. Campos, P. J. Coleman, J. C. Alvarez, S. D. Dreher, R. M. Garbaccio, N. K. Terrett, R. D. Tillyer, M. D. Truppo, E. R. Parmee, The importance of synthetic chemistry in the pharmaceutical industry. *Science* **363**, eaat0805 (2019).

42. R. C. Larock, *Comprehensive Organic Transformations* (Wiley, 1999).

43. J. Otera, J. Nishikido, *Esterification: Methods, Reactions, and Applications* (Wiley, 2009).

44. K. Ishihara, S. Ohara, H. Yamamoto, Direct condensation of carboxylic acids with alcohols catalyzed by hafnium(IV) salts. *Science* **290**, 1140–1142 (2000).

45. S. Enthaler, A. Company, Palladium-catalysed hydroxylation and alkoxylation. *Chem. Soc. Rev.* **40**, 4912–4924 (2011).

46. H. Wang, K. Liang, W. Xiong, S. Samanta, W. Li, A. Lei, Electrochemical oxidation-induced etherification via C(sp^3)–H/O–H cross-coupling. *Sci. Adv.* **6**, eaaz0590 (2020).

47. R. H. Beddoe, K. G. Andrews, V. Magné, J. D. Cuthbertson, J. Saska, A. L. Shannon-Little, S. E. Shanahan, H. F. Sneddon, R. M. Denton, Redox-neutral organocatalytic Mitsunobu reactions. *Science* **365**, 910–914 (2019).

48. M. B. Kurosawa, R. Isshiki, K. Muto, J. Yamaguchi, Catalytic deoxygenative coupling of aromatic esters with organophosphorus compounds. *J. Am. Chem. Soc.* **142**, 7386–7392 (2020).

49. M. Hayashi, S. Nakamura, Catalytic enantioselective protonation of α-oxygenated ester enolates prepared through phospha-brook rearrangement. *Angew. Chem. Int. Ed.* **50**, 2249–2252 (2011).

50. P. Cheruku, S. Gohil, P. G. Andersson, Asymmetric hydrogenation of enol phosphinates by iridium catalysts having N,P ligands. *Org. Lett.* **9**, 1659–1661 (2007).

51. I. Borthakur, S. Srivastava, S. Kumari, S. Kundu, Tandem synthesis of *N*-methylated tertiary amines via three-component coupling of carbonyl compounds, amines, and methanol. *Chem. Commun.* **58**, 9822–9825 (2022).

52. S. Zhu, H. Feng, S. Hu, T. Guo, M. Li, S. Tan, L. Huang, J. Huang, Modular synthesis of unsymmetrical 1,4-diamino-2-butynes by Cu–catalyzed sequential decarboxylative A^3-coupling/petasis reaction/A^3-coupling. *Asian J. Org. Chem.* **10**, 816–819 (2021).

53. A. R. Jeon, M. E. Kim, J. K. Park, W. K. Shin, D. K. An, Mild and direct conversion of esters to morpholine amides using diisobutyl(morpholino)aluminum: Application to efficient one-pot synthesis of ketones and aldehydes from esters. *Tetrahedron* **70**, 4420–4424 (2014).

54. M. M. Faul, M. E. Kobierski, M. E. Kopach, Green chemistry approach to the synthesis of *N*-substituted piperidones. *J. Org. Chem.* **68**, 5739–5741 (2003).

55. D. R. Wallach, J. D. Chisholm, Alkylation of sulfonamides with trichloroacetimidates under thermal conditions. *J. Org. Chem.* **81**, 8035–8042 (2016).

56. H.-J. Zhang, L. Chen, M. S. Oderinde, J. T. Edwards, Y. Kawamata, P. S. Baran, Chemoselective, scalable nickel-electrocatalytic O-arylation of alcohols. *Angew. Chem. Int. Ed.* **60**, 20700–20705 (2021).

57. F. Ahad, N. Ghouri, K. M. Khan, S. Perveen, M. I. Choudhary, Synthesis of 4-substituted ethers of benzophenone and their antileishmanial activities. *R. Soc. Open Sci.* **5**, 171771 (2018).

58. X. Wen, S.-B. Wang, D.-C. Liu, G.-H. Gong, Z.-S. Quan, Synthesis and evaluation of the anti-inflammatory activity of quinoline derivatives. *Med. Chem. Res.* **24**, 2591–2603 (2015).

59. Q. Kong, W. Zhuang, G. Li, Y. Xu, Q. Jiang, Y. Wang, High contrast stimuli-responsive luminescence switching of pyrene-1-carboxylic esters triggered by a crystal-to-crystal transition. *New J. Chem.* **41**, 13784–13791 (2017).

60. S. Chun, Y. K. Chung, Transition-metal-free poly(thiazolium) iodide/1,8-diazabicyclo[5.4.0]undec-7-ene/phenazine-catalyzed esterification of aldehydes with alcohols. *Org. Lett.* **19**, 3787–3790 (2017).

61. J.-S. Zhang, T. Chen, Y. Zhou, S.-F. Yin, L.-B. Han, Catalytic sp^3C–CN bond cleavage: Ni-mediated phosphorylation of alkylnitriles. *Org. Lett.* **20**, 6746–6749 (2018).

62. B. Yang, Z.-X. Wang, Ni-catalyzed C–P coupling of aryl, benzyl, or allyl ammonium salts with P(O)H compounds. *J. Org. Chem.* **84**, 1500–1509 (2019).

63. F. Wang, M. Qu, F. Chen, Q. Xu, M. Shi, Ph_2PI as a reduction/phosphination reagent: Providing easy access to phosphine oxides. *Chem. Commun.* **48**, 8580–8582 (2012).

64. X. Ma, Q. Xu, H. Li, C. Su, L. Yu, X. Zhang, H. Cao, L.-B. Han, Alcohol-based Michaelis–Arbuzov reaction: An efficient and environmentally-benign method for C–P(O) bond formation. *Green Chem.* **20**, 3408–3413 (2018).

65. R. S. Davidson, R. A. Sheldon, S. Trippett, The reaction of tetraphenyldiphosphine with aromatic carboxylic acids. *J. Chem. Soc. C* , 1547–1552 (1967).

66. R. Shen, C. Dong, J. Yang, L.-B. Han, Copper(II) acetate-catalyzed synthesis of phosphorylated pyridines via denitrogenative C−P coupling between pyridotriazoles and P(O)H compounds. *Adv. Synth. Catal.* **360**, 4252–4258 (2018).

67. Z.-S. Chen, Z.-Z. Zhou, H.-L. Hua, X.-H. Duan, J.-Y. Luo, J. Wang, P.-X. Zhou, Y.-M. Liang, Reductive coupling reactions: A new strategy for C(sp^3)–P bond formation. *Tetrahedron* **69**, 1065–1068 (2013).

68. T. Nakajima, K. Takano, H. Maeda, Y. Ogiwara, N. Sakai, Production of alkyl aryl sulfides from aromatic disulfides and alkyl carboxylates via a disilathiane–disulfide interchange reaction. *Chem. Asian J.* **16**, 4103–4107 (2021).

69. G.-p. Lu, C. Cai, An odorless, one-pot synthesis of nitroaryl thioethers via SNAr reactions through the in situ generation of S-alkylisothiouronium salts. *RSC Adv.* **4**, 59990–59996 (2014).

70. B. Hu, H. Hu, L. Sun, R. Tang, Iodine-mediated thioetherification of alcohols with disulfides or NaSH under microwave irradiation. *Chin. J. Chem.* **30**, 2556–2562 (2012).

71. J. Yuan, X. Ma, H. Yi, C. Liu, A. Lei, I_2-catalyzed oxidative C(sp3)–H/S–H coupling: Utilizing alkanes and mercaptans as the nucleophiles. *Chem. Commun.* **50**, 14386–14389 (2014).

72. K. Ishitobi, R. Isshiki, K. K. Asahara, C. Lim, K. Muto, J. Yamaguchi, Decarbonylative aryl thioether synthesis by Ni catalysis. *Chem. Lett.* **47**, 756–759 (2018).

73. D. Wang, Z. Liu, Z. Wang, X. Ma, P. Yu, Metal- and base-free regioselective thiolation of the methyl C(sp^3)–H bond in 2-picoline N-oxides. *Green Chem.* **21**, 157–163 (2019).

74. Z. Wu, M. Lai, S. Zhang, X. Zhong, H. Song, M. Zhao, An efficient synthesis of benzyl dithiocarbamates by base-promoted cross-coupling reactions of benzyl chlorides with tetraalkylthiuram disulfides at room temperature. *Eur. J. Org. Chem.* **2018**, 7033–7036 (2018).

75. S. Bera, A. Bera, D. Banerjee, Nickel-catalyzed hydrogen-borrowing strategy: Chemo-selective alkylation of nitriles with alcohols. *Chem. Commun.* **56**, 6850–6853 (2020).

76. J. Yang, H. Dong, K. Yan, X. Song, J. Yu, J. Wen, Isocyanide-induced esterification of sulfinic acids to access sulfinates. *Adv. Synth. Catal.* **363**, 5417–5421 (2021).

77. P. B. Arockiam, U. Lennert, C. Graf, R. Rothfelder, D. J. Scott, T. G. Fischer, K. Zeitler, R. Wolf, Versatile visible-light-driven synthesis of asymmetrical phosphines and phosphonium salts. *Chem. A Eur. J.* **26**, 16374–16382 (2020).

78. J. Yang, T. Chen, L.-B. Han, C–P bond-forming reactions via C–O/P–H cross-coupling catalyzed by nickel. *J. Am. Chem. Soc.* **137**, 1782–1785 (2015).
